# Homeotic shift at the dawn of the turtle evolution

**DOI:** 10.1098/rsos.160933

**Published:** 2017-04-05

**Authors:** Tomasz Szczygielski

**Affiliations:** Institute of Paleobiology, Polish Academy of Sciences, Twarda 51/55, 00-818 Warsaw, Poland

**Keywords:** homeosis, Carnian, Norian, Pan-Testudinata, patterning, palaeontology

## Abstract

All derived turtles are characterized by one of the strongest reductions of the dorsal elements among Amniota, and have only 10 dorsal and eight cervical vertebrae. I demonstrate that the Late Triassic turtles, which represent successive stages of the shell evolution, indicate that the shift of the boundary between the cervical and dorsal sections of the vertebral column occurred over the course of several million years after the formation of complete carapace. The more generalized reptilian formula of at most seven cervicals and at least 11 dorsals is thus plesiomorphic for Testudinata. The morphological modifications associated with an anterior homeotic change of the first dorsal vertebra towards the last cervical vertebra in the Triassic turtles are partially recapitulated by the reduction of the first dorsal vertebra in crown-group Testudines, and they resemble the morphologies observed under laboratory conditions resulting from the experimental changes of *Hox* gene expression patterns. This homeotic shift hypothesis is supported by the, unique to turtles, restriction of *Hox-5* expression domains, somitic precursors of scapula, and brachial plexus branches to the cervical region, by the number of the marginal scute-forming placodes, which was larger in the Triassic than in modern turtles, and by phylogenetic analyses.

## 1. Introduction

All crown-group turtles have eight cervical vertebrae (CVs) and 10 dorsal vertebrae (DVs), although in Pleurodira the last two DV may become sacralized [1]. Such a number of presacral elements is one of the smallest among amniotes and is only approximated by agamidids, pareiasaurids, and some placodonts, ornithischians, birds and pterosaurs, and exceeded by *Eunotosaurus africanus* Seeley [2], *Euoplocephalus tutus* Lambe [3] and chamaeleonidids [4,5]. Although it must have resulted from the reduction of the ancestral amniotic presacral formulae of approximately six CVs and 20 DVs [4], the exact time and succession of the meristic changes towards the turtle-specific bauplan is unclear, especially that the earliest stem turtles are believed to have their presacral count reduced even further. The Carnian (early Late Triassic, approx. 237–227 Myr ago) *Odontochelys semitestacea* Li *et al*. [6] was described as having eight CVs and only nine DVs, the Ladinian (late Middle Triassic, approx. 242–237 Myr ago) *Pappochelys rosinae* Schoch & Sues [7] is said to have no more than nine DVs, and the Permian diapsid *Eunotosaurus africanus* had only six CV and nine DVs [5]. All of these counts are lower than expected from the turtle ancestor [4]. Müller *et al*. [4] considered the low vertebral number of *O. semitestacea* to be its autapomorphy.

**Figure 1.**
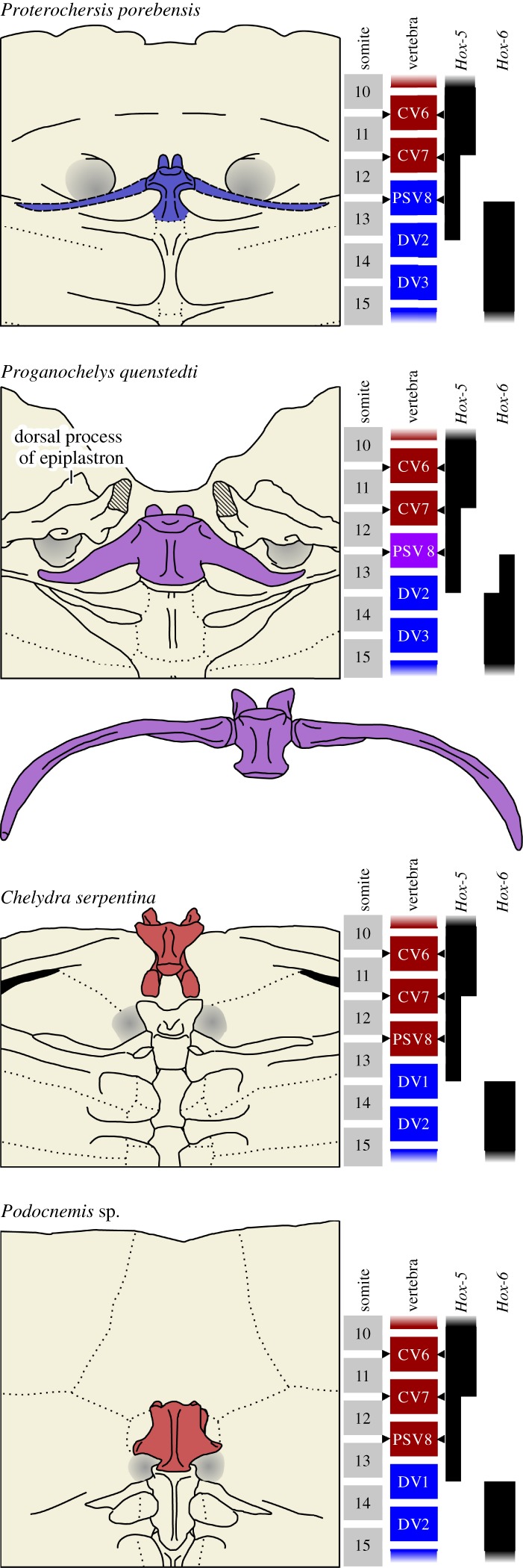
The morphology of the cervico-dorsal transition in the Late Triassic turtles *Proterochersis porebensis* and *Proganochelys quenstedti*, and recent *Chelydra serpentina* (Cryptodira) and *Podocnemis* sp. (Pleurodira) in ventral view, with somitic expression ranges of *Hox-5* and *Hox-6* genes shown. The presacral vertebra 8 (PSV8) is coloured. The ribs of PSV8 for *Prot. porebensis* were restored based on *Keuperotesta limendorsa*. For *Prog. quenstedti* both known variants of PSV8 are shown (drawings based on Gaffney [11]). PSV8 of *Ch. serpentina* is disarticulated to show its ventral aspect. The hypothetical expression ranges of *Hox* genes for the Triassic taxa are deduced from morphology. Note that PSV8 changed its character from dorsal (DV) in *Prot. porebensis* to intermediate cervico-dorsal of varied morphology in *Prog. quenstedti*, to cervical (CV) in more advanced turtles, while PSV9 (the first dorsal vertebra of modern turtles) and its ribs assumed morphology similar to the initial state of PSV8. Note that the articulation areas for scapulae (shaded grey) migrated posteriorly and medially in modern turtles towards PSV9, although the scapulae still arise from somites 8–12 (CV4–7) and the position of brachial plexus nerves (arrowheads) remains unchanged.

The oldest and most basal true turtles, Proterochersidae Nopcsa [8], and the slightly younger and more advanced *Proganochelys quenstedti* Baur [9] (all from the Late Triassic) are all currently believed to already possess the typical turtle formula of eight CVs and 10 DVs [10,11]. The exact nature of the eighth presacral in these turtles is, however, controversial. In *Proganochelys quenstedti*, this vertebra has an intermediate morphology, conspicuously large ribs, and in some adult specimens its high neural spine forms a sutural contact with the carapace (figure 1). These characteristics led Jaekel [12] to the conclusion that it should be considered the first dorsal vertebra, and that the proper presacral formula for *Prog. quenstedti* (Jaekel's *‘Triassochelys dux’*) is 7 CVs and 11 DVs. This interpretation was widely accepted by many authors, including Hoffstetter & Gasc [1] and Romer [13], and it was rejected only quite recently by Gaffney [11], based on the assumption that although the neural spine is sutured to the carapace, the vertebra itself is not. The recent description of *Proterochersis porebensis* Szczygielski & Sulej [10], however, made clear that this vertebra was indeed initially ankylosed both to the carapace and the following DVs. This observation, together with the recent progress of the homeotic gene function studies and the turtle embryology, stresses the need to reconsider the identity of the eighth presacral (PSV8) and the evolution of the turtle vertebral column once again. It also needs to be tested phylogenetically to create a hypothesis of evolutionary changes in vertebral count at the dawn of the turtle linage.

## 2. Material and methods

Most of the Polish and German Triassic turtle material was studied by the author. Part of the measurements of the axial skeleton of *O. semitestacea* were taken using ImageJ (Rasband, W.S., ImageJ, US National Institutes of Health, Bethesda, MD, USA, http://imagej.nih.gov/ij/, 1997–2016 [14,15]).

No fieldworks were performed strictly for this work. All the fossil material used here was previously published and is housed in palaeontological collections of Museum für Naturkunde (MB; Berlin, Germany), Institute of Vertebrate Paleontology and Paleoanthropology (IVPP; Beijing, China), Staatliches Museum für Naturkunde Stuttgart (SMNS; Stuttgart, Germany), and Institute of Paleobiology, Polish Academy of Sciences (ZPAL; Warsaw, Poland). Some of the ZPAL material was additionally prepared. All of it was collected during previous seasons in Poręba, Poland, from the communal area near the municipal landfill site.

It must be noted that the division of the vertebral column of fossil animals into sections is often arbitrary, because in many taxa the transitions are blurry and the morphology of segments is variable between taxa. Therefore, some set of criteria must be specified. I follow the traditional criteria used historically for turtles. The dorsal section of the vertebral column is immovable, because of flat posterior articular surfaces and sutural contact or fusion to the succeeding vertebrae and carapace. Usually, most DV participate in the carapace formation (the neural processes are broadened and form the neural bones of the carapace), but this criterion is not mandatory, because in most modern turtles the first and the last dorsal vertebrae do not form a neural, and in some taxa the number of neural bones may be partially or fully reduced [16]. The large rib size and their contact with or participation in the carapace support the classification of vertebra as a dorsal segment, but these criteria are also not mandatory, because in modern turtles the first and last pair of dorsal ribs are reduced, do not form their own set of costal bones and in some taxa do not contact the carapace [16]. The cervical region is movable, does not participate in the carapace, and generally has reduced ribs, although in some of the Triassic taxa vestigial cervical ribs were present [1,6,11]. The Triassic turtles did not possess formed cervical articulations, which were developed after the Triassic as a mechanism allowing the neck retraction [1,11,13,16], so this criterion cannot be used. Generally, most morphological characters used to classify the vertebrae into sections in modern turtles may also be used for the Triassic taxa. The criteria of vertebrae classification used by me here are essentially the same as those used previously by Jaekel [12], Hoffstetter and Gasc [1], Romer [13] and Gaffney [11].

A total of 92 phylogenetic analyses using a matrix from Bever *et al*. [17] with additions by Schoch & Sues [7], eight new characters, and one new taxon (*Candelaria barbouri* Cisneros *et al*. [18]), were performed with TNT (Willi Hennig Society [19]) in three sets. Their main objective was to check whether the reduced number of vertebral segments in *E. africanus* and Pan-Testudinata is homologous and what number of DVs is plesiomorphic for turtles. The secondary aim was to study the apparent peculiar conflict of phylogenetic signal between *Pa. rosinae* and *E. africanus* that was observed while performing the analyses encompassing both of these taxa, which was not attempted before. Each of the analyses was performed as Traditional Tree Bisection-Reconnection search (1000 replications) with Seymouriidae as the outgroup. Bootstrap values (frequency differences) were found for 1000 replications. Characters 33, 61, 66, 84, 117, 154, 192, 219, 246, 249, 253 and 272 were considered additive (ordered). The first set of analyses (1–32) was designed to test the impact of *E. africanus*, *Pa. rosinae*, *O. semitestacea*, *Prog. quenstedti* and *C. barbouri* on tree topology and support. Every possible combination of these five taxa (individually, in pairs, triplets, quaternions and all at once) was tested under the same conditions (see the electronic supplementary material for tables with settings of each of the analyses). The second set (analyses 33–70) was performed under implied weighting to reduce the impact of homoplasy. First, I attempted to find the most optimal *K*-value in terms of support for the turtle-*Eunotosaurus* and turtle-*Pappochelys* clades (analyses 33–64; the topology in these analyses was *E. africanus* (*Pa. rosinae* (*O. semitestacea* + *Prog. quenstedti*))). In these analyses, I used all the taxa and varied *K*-values. This proved to have moderate impact on the supports, and no large variance was observed for *K*-value range of 1–10. Then, I tried to find the highest possible individual support for the turtle-*Eunotosaurus* and turtle-*Pappochelys* clades separately (analyses 65–68). In order to do that, I took the most beneficial (giving the highest support for each of these clades) taxon combinations from analyses 1–32 and set the most optimal (from the support and resolution point of view) *K*-value found in analyses 33–64. Finally, I tested the topology and support for analyses without *C. barbouri* (which I found to decrease the tree support in previous analyses) and without *C. barbouri*, *O. semitestacea* and *Prog. quenstedti*. Next, I have examined the lists of synapomorphies for all the analyses thus far and found that the turtle-*Eunotosaurus* clade appears to be supported by no more than five postcranial characters (253, 247, 251, 174 and 204). To test that observation, I performed the third set of analyses (71–92). In that set, I successively removed each of these five characters with and without implied weighting and with *C. barbouri* included (analyses 71–80) and excluded (81–90). The resolution of most of the trees obtained from this set (especially analyses including *C. barbouri*) proved to be rather poor, with a large polytomy comprising basal parareptiles and eureptiles, and in some cases a second polytomy within Diapsida. To check whether this unsatisfying tree resolution results from the sole removal of characters or from the interaction of *E. africanus* with other taxa, I removed that taxon and performed the final two analyses, once again with and without the implied weighting. See the electronic supplementary material for the obtained data.

## 3. Morphology of the eight presacral in *Proterochersis porebensis* and *Proganochelys quenstedti*

**Figure 2.**
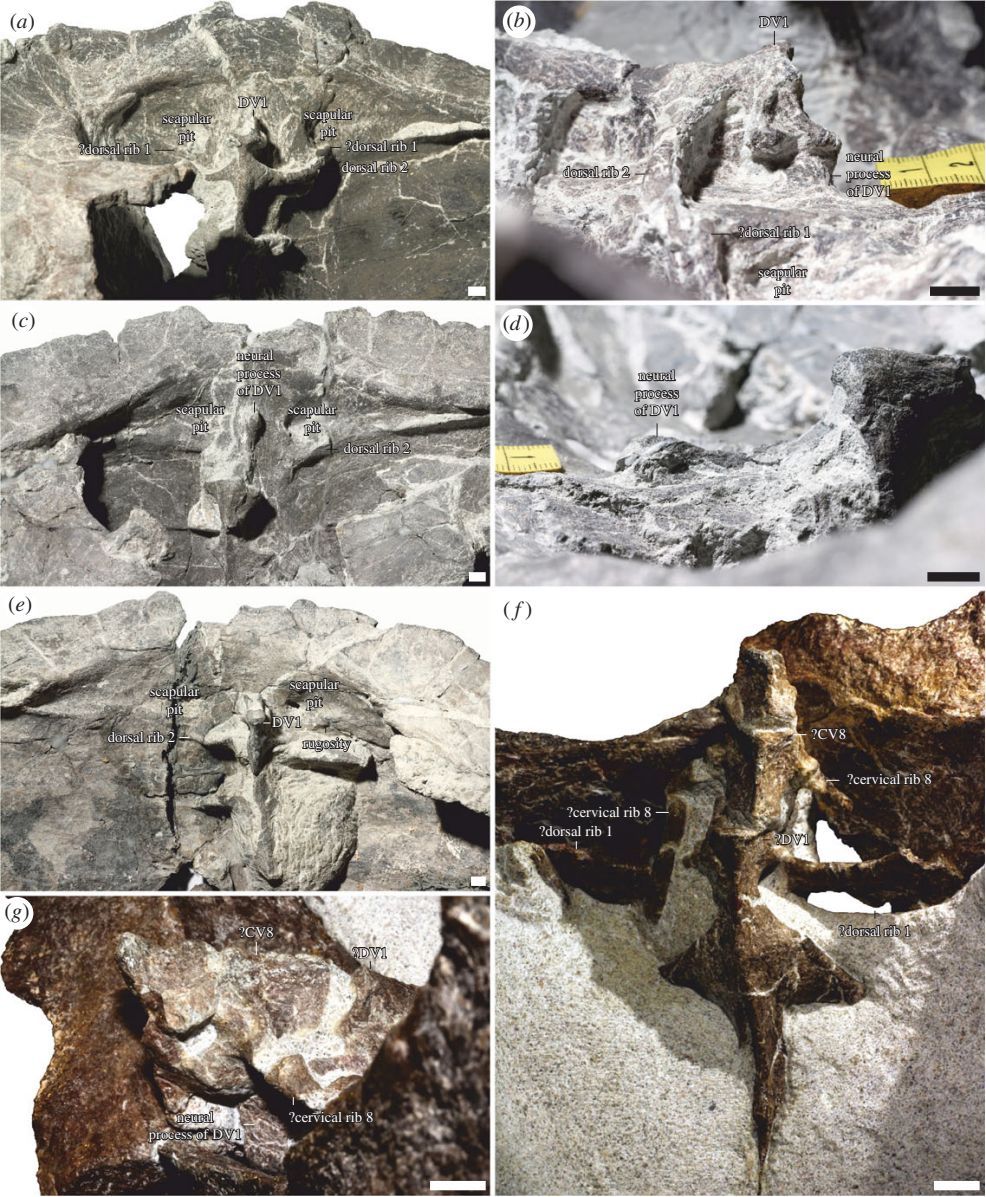
Cervico-dorsal transition in Proterochersidae. (*a,b*) *Proterochersis porebensis* ZPAL V.39/48 (holotype), (*a*) visceral and (*b*) lateral (right side, visceral surface towards top) view. (*a*) Dorsal vertebra (DV) 1 (presacral vertebra 8) is fused to the rest of the dorsal vertebral column. Splinters of bone, possibly the distal parts of the first pair of dorsal ribs, are present laterocaudally to the scapular pits, cranially to the ribs of DV2. (*b*) The neural process of DV1 can be seen fused to the carapace. The rib is missing, but the point of articulation is visible. (*c,d*) *Proterochersis porebensis* ZPAL V.39/49, (*c*) visceral and (*d*) lateral (left side, visceral surface towards top) view. (*c*) DV1 is missing, but its broken neural process is still fused to the carapace. (*d*) The neural process of DV1 sticks out from the visceral surface of the carapace. (*e*) *Proterochersis porebensis* ZPAL V.39/72, visceral view. DV1 is fused to the rest of the dorsal vertebral column. Rugosities laterocaudal to the scapular pits (best visible on the left side of the specimen, right side of the photograph) may be remnants of the articulations of the first pair of dorsal ribs with the carapace. (*f,g*) *Keuperotesta limendorsa* SMNS 17757 (holotype), (*f*) visceral and (*g*) lateral (left side, visceral surface towards top) view. Two anterior vertebrae, interpreted previously as the seventh and eighth (?CV8) cervical are free and partially disarticulated. The third (the first dorsal) is fused to the following dorsal series and contacts the carapace. The second and third vertebrae bear elongated ribs, which are at least proximally free from the carapace. The scale bars measure 1 cm. See the text for discussion.

In *Proterochersis porebensis*, the presacral vertebra (PSV) 8 is indistinguishable from the following DVs. In all specimens with this region preserved (ZPAL V.39/48, ZPAL V.39/49, and ZPAL V.39/72), it is fused both to the carapace and the vertebral column (figure 2). Notably, the nuchal bone in *Prot. porebensis* is short and it did not reach the neural spine of PSV8 (T. Sz. 2016, personal observation). Therefore, PSV8 either contacted the neural bone of DV1, or formed its own neural. The ribs of PSV8 are currently unknown for *Prot. porebensis*, but they certainly were present, as evidenced by the points of their attachment on the vertebra. Long, slender ribs are present on PSV8 of another proterochersid, *Keuperotesta limnedorsa* Szczygielski & Sulej [10]. All in all, PSV8 of *Prot. porebensis* does not differ in most aspects of morphology from the PSV9 of modern turtles, which is a true dorsal vertebra, as evidenced by *Hox* expression pattern [20], and in some aspects (not reduced ribs) is even more dorsal-like. For that reason, I advocate that PSV8 in *Proterochersis* spp. still belonged to the dorsal section of the vertebral column.

In *Proganochelys quenstedti*, two distinct morphologies of PSV8 are known: one with long, thin, posteriorly and ventrally concave, separate ribs (SMNS 16980; see the electronic supplementary material for institutional abbreviations) and the second (MB 1910.45.2) with proportionally shorter, stouter and fused, nearly horizontal ribs that were concave anteriorly and tucked behind the dorsal scapular processes [11]. The neural spine of PSV8 is free in SMNS 16980 but sutured to the carapace in MB 1910.45.2 [11]. Some of these differences (the thickness and fusion to the vertebral centrum) may potentially be attributed to the age difference between these two specimens (SMNS 16980 is subadult, while MB 1910.45.2 is adult), but some other explanation is required for the others. Merging the views first expressed by Jaekel [12] and Gaffney [11], I interpret this morphology as intermediate between the typical dorsal and cervical. In more derived turtles, PSV8 lost its contact with the carapace and ribs, and attained a large mobility, thus fully achieving cervical characteristics.

## 4. Phylogenetic results

### 4.1. The first set of analyses (1–32)

In the first round of phylogenetic analyses (1–32; electronic supplementary material, table S2), I initially tried to verify whether the low vertebral count of *Eunotosaurus africanus* is homologous with that of turtles. Secondarily, I attempted to determine the impact of *E. africanus*, *Pappochelys rosinae*, *Odontochelys semitestacea*, *Proganochelys quenstedti* and *Candelaria barbouri* on the tree topology and support. All the resulting trees differ mainly in the degree of resolution (low to moderate) and the position of *E. africanus* and stem Testudinata. Although in 12 analyses these taxa tend to form a clade, an apparent conflict between *Pa. rosinae* and *E. africanus* is notable. In analyses in which both of them and at least one more derived stem turtle is present, the support for clades (*E. africanus* (*Pa. rosinae* + more derived stem turtles)) and (*Pa. rosinae* + more derived stem turtles) is drastically weakened. Their clades with more derived stem turtles get much higher support when either *Pa. rosinae* or *E. africanus* are removed. In analyses with both *O. semitestacea* and *Prog. quenstedti* excluded, *Pa. rosinae* and *E. africanus* do not form a clade: *Pa. rosinae* nests in or near crown Diapsida, and *E. africanus* ends up in a large polytomy outside of Diapsida. When tested individually, *Pa. rosinae* is recovered in Diapsida, and *E. africanus* in large polytomy outside of Diapsida. Generally, turtles without *E. africanus* invariably are placed within Diapsida, while *E. africanus* without derived turtles invariably falls outside of Diapsida. Interestingly, the turtle-*Eunotosaurus* clade, whenever located inside of Diapsida, is nested more stemward than the turtle-only clade in most cases when *E. africanus* is removed or located elsewhere. The presence of *E. africanus* also appears to decrease the tree resolution, especially of the eureptilian branch. Overall, the bootstrap supports for both turtle-*Eunotosaurus* and turtle-*Pappochleys* clades are very low in most analyses. Peculiarly, the inclusion of *C. barbouri* results in their further significant decrease. The grouping of either *E. africanus* or *P. rosinae* with more derived testudinates attains decent support (bootstrap value of 70 or higher) only when both the other taxon and *C. barbouri* are excluded from the analysis.

The number of synapomorphies seemingly supporting the exclusive turtle-*Eunotosaurus* clade in some analyses is quite robust, reaching up to 42 characters in analysis 14 and 37 characters in analysis 1 (electronic supplementary material, table S6). That number, however, is highly dependent on the tree topology and varied, for example in analyses 5 and 8 it is only 2. The recurrence of the particular characters in different analyses is also varied. Out of the 60 characters appearing in that set of analyses as synapomorphies of the exclusive turtle-*Eunotosaurus* clade, only four are present 10 times out of 12 (characters 174—presacral vertebral count; 247—T-shaped ribs; 251—trunk length; and 253—distinctly broadened ribs) and only four more are present for more than a half of the recovered trees (characters 0—skull proportions; 33—lacrimal morphology; 84—quadratojugal, vertical process; 147—parasphenoid, cultriform process; and 148—parasphenoid, teeth). Two of these characters are problematic: character 0 is scored as 0 for *Pa. rosinae* (not as 2 like in *E. africanus*, *O. semitestacea* and *Prog. quenstedti*), and in all the analyses including the former taxon the algorithm treats this as a reversion. Character 253, on the other hand, is scored as 2 (nine or fewer ribs distinctly broadened) for *E. africanus*, *O. semitestacea* and *Prog. quenstedti*, but the correct scoring for the oldest fully shelled turtles, Proterochersidae would be 1 (10 or more ribs distinctly broadened). Unfortunately, the incompleteness of the latter (total lack of the cranial material and only partial limb remains) held me from including that taxon in the matrix. The state for *Pa. rosinae* is currently unsure. Further discoveries and, possibly, review of *O. semitestacea* material are needed to ascertain which scoring is indeed plesiomorphic for Testudinata. Furthermore, 13 out of the 60 characters appear only in analyses in which *Pa. rosinae* is absent. In nine cases the reason is that the character states for that taxon are different than in *E. africanus* and more derived pan-testudinates, and in four cases the states for both *Pa. rosinae* and *O. semitestacea* are different than in *E. africanus* and *Prog. quenstedti*. In total, 14 out of the 60 potential synapomorphies are only present in the analyses in which the turtle-*Eunotosaurus* clade is nested in a large polytomy composed of mixed parareptiles and eureptiles. These are general eureptilian or diapsid characters, and in those cases, are forced to be shown as synapomorphies of the turtle-*Eunotosaurus* clade by the unresolved tree topology. Another notable fact is that only three out of the 60 character states are shared only by pan-testudinates and *E. africanus*. In total, 31 of the other characters have at least 10 other occurrences in the matrix and 14 of them appear at least in 16 other taxa (which constitutes a third or more of all the taxa in the matrix). States for 22 out of the 60 characters are unknown for at least a third of all the taxa in the matrix and 28 are unknown for *Pa. rosinae*.

Although some decrease in support is expected when more taxa are used due to the increase of homoplasy, such drastic drops in support values caused by addition of one taxon or vastly different positions of the particular taxa depending on the presence or absence of more derived forms are troubling. I interpret them as a sign of homoplasy, which tends to pull either one of these two taxa out of the clade with Pan-Testudinata or into that clade. This seems to be supported by the high incidence of the given character states or their poor sampling. It is apparent that the position of *E. africanus* is more dependent on the position of turtles, than the other way around, and that many putative synapomorphies are in fact derived features shared by *E. africanus* and *Prog. quenstedti* (and, in some cases, *O. semitestacea*) but missing in *Pa. rosinae* (and, in some other cases, *O. semitestacea*), and possibly turtle ancestors. To reduce the impact of the homoplastic characters, I performed another set of analyses utilizing the implied weighting method.

### 4.2. The second set of analyses (33–70)

In the second round of phylogenetic analyses (33–70; electronic supplementary material, tables S3 and S4), I tried to determine the impact of homoplasy on the tree topology and support. To find the optimal *K*-value, I have performed a series of tests using full matrix. In the range of *K* = 2.5 to *K* = 5.5, I observed a uniform low bootstrap support for turtle-*Eunotosaurus* clade and good support for turtle-*Pappochelys* clade with insignificant variation that never exceeded 5%. In all cases with the exception of analysis 45 (*K* = 3.625) the turtle-*Eunotosaurus* clade was located in Diapsida (usually including *C. barbouri*). Higher and lower *K*-values result in loss of tree resolution and gradual loss of the support for both the turtle-*Eunotosaurus* and turtle-*Pappochelys* clade. Despite my efforts to locate the optimal *K*-value with the accuracy of 0.125 within the optimal range, the support for the turtle-*Eunotosaurus* clade never exceeded 17 (analyses 36, *K* = 2.5, and 58, *K* = 5.25). The turtle-*Pappochelys* clade, on the other hand, maintained support of 68–71 even in spite of the presence of *E. africanus*. The last six analyses were performed to check the different configurations of taxa in the setting of implied weighting. Analyses 65 and 66 were based on the two most optimal scenarios in terms of the turtle-*Pappochelys* clade support, that were identified among the analyses 1–32 (no *E. africanus*, *C. barbouri*, and—in analysis 66—*Prog. quenstedti*) with *K*-value of 2.75 (which gave one of the best-resolved and best-supported trees, and the best support for the turtle-*Pappochelys* clade in analyses 33–64). Both settings resulted in trees with Pan-Testudinata as sister-group to Lepidosauromorpha (including Kuehneosauridae), and great support of the turtle-*Pappochelys* clade of 90 (analysis 65) and 93 (analysis 66). Analyses 67 and 68 tested the maximal support for the turtle-*Eunotosaurus* clade. Once again, the settings were selected based on the optimal scenario taken from analyses 1–32 (no *Pa. rosinae*, *C. barbouri*, and, in analysis 68, *O. semitestacea*) and optimal *K*-value (2.5). As expected, the support for the turtle-*Eunotosaurus* clade is higher than in analyses considering all taxa, but it never reaches 70 and is higher than 60 only in the analysis excluding the most basal turtles. Analysis 69 was performed with *Pa. rosinae* and *E. africanus* but without more derived testudinates and *C. barbouri*, and just like in the analogous test not utilizing implied weighting, *Pa. rosinae* ended up nested high in Diapsida, and *E. africanus* fell out of the Diapsida to a large polytomy. Finally, analysis 70 included all the taxa with the exception of *C. barbouri*, resulting in the best tree resolution. Just like in previous analyses, the support for both the turtle-*Eunotosaurus* and turtle-*Pappochelys* clade is much higher, but still the turtle-*Eunotosaurus* clade support is only 53, while the grouping of *P. rosinae* with turtles attained 92% bootstrap support.

The list of the potential synapomorphies supporting the exclusive turtle-*Eunotosaurus* clade in analyses 33–70 is slightly shorter than in the previous series (47 versus 60 for analyses 1–32; electronic supplementary material, table S7). Excluded are most of the characters incongruent with *Pa. rosinae*. In total, 23 of the 47 characters are present in at least half of the analyses, the most common is character 84—quadratojugal, vertical process (33 analyses). Five of the 47 characters are present only in the analyses excluding *Pa. rosinae* and 17 in the analyses in which the turtle-*Eunotosaurus* clade is located in a large polytomy. Like in the previous set of analyses, only three character states are unique to the turtle-*Eunotosaurus* clade, 21 are present in more than 10 other taxa and nine in at least a third of all the taxa. Twenty-two out of 47 characters are scored as unknown in at least a third of all the taxa and 28 are unknown for *Pa. rosinae*. Interestingly, in analysis 70, which yielded the best-supported and best-resolved tree (and thus is least likely to list plesiomorphic characters as synapomorphies of the higher clades), only four characters support the turtle-*Eunotosaurus* clade: 174 (presacral vertebral count), 204 (ectepicondylar groove), 247 (T-shaped ribs), 251 (trunk length) and 253 (distinctly broadened ribs; see the discussion of that character for analyses 1–32 above). All of these characters are postcranial. To check whether these five postcranial characters indeed are the only features supporting the exclusive turtle-*Eunotosaurus* clade and make sure that this is not only an artefact of implied weighting and/or taxon sampling, I performed the third, final set of analyses.

### 4.3. The third set of analyses (71–92)

**Figure 3.**
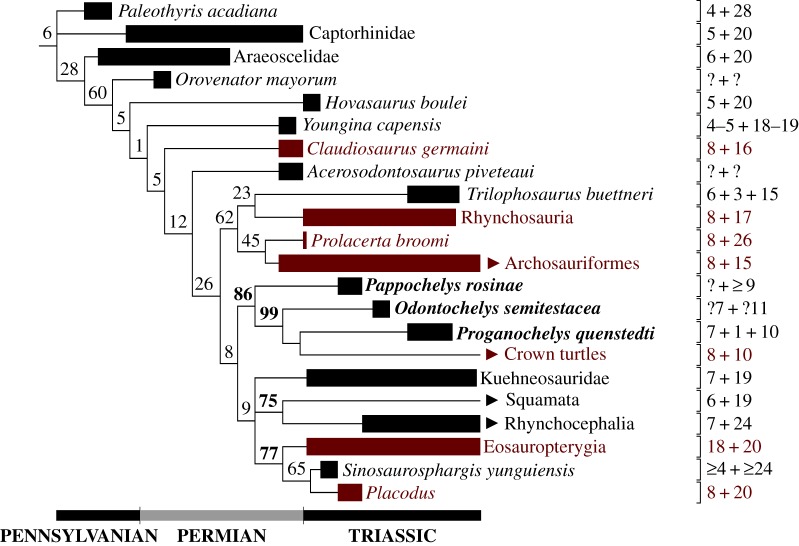
Phylogeny of Eureptilia based on the best-resolved and best-supported eureptilian tree including *Pappochelys rosinae*, *Odontochelys semitestacea* and *Proganochelys quenstedti* (analysis 92). On the right, the numbers of cervical, intermediate (if present) and dorsal vertebrae are given. The plesiomorphic vertebral formulae for suprageneric taxa are taken mostly from Müller *et al*. [4]. The taxa having more than seven cervicals are indicated in red. Arrowheads indicate taxa still alive today. The values above the nodes represent bootstrap support. See the electronic supplementary material for the settings of the phylogenetic analyses and all the other topologies.

In the third round of phylogenetic analyses (71–90; electronic supplementary material, table S5), I tried to determine the impact of characters 174, 204, 247, 251 and 253 on the tree topology and support. These five characters were found to be the only synapomorphies of the exclusive turtle-*Eunotosaurus* clade in the best-resolved tree obtained so far (analysis 70). In order to check whether these five characters are indeed the key characters supporting this clade, I have performed a set of analyses with the full dataset, successively removing each of these characters with and without implied weighting (analyses 71–80). I have also repeated the same procedure with *C. barbouri* removed, due to its tendency to lower the clade supports and tree resolution (81–90). Character 253 was removed first, due to its ambiguous nature (see discussion of that character for analyses 1–32). Character 247 was next, because both characters 247 and 253 are the only characters with states unique to *E. africanus* and Pan-Testudines, and thus are the most likely candidates for pivotal synapomorphies of that grouping. Character 204 was removed last, as it is the only potential synapomorphy located outside of the dorsal axial skeleton. As in previous analyses, both the implied weighting and the removal of *C. barbouri* resulted in better-resolved and better-supported trees. With *C. barbouri* present and implied weighting enabled it is enough to remove characters 247 and 253 to obliterate the support for grouping of *E. africanus* and turtles. With implied weighting disabled the bootstrap support for turtle-*Eunotosaurus* clade is 2, and the support is nullified with the removal of another character. Most of these analyses resulted in poorly resolved trees with turtles in a large polytomy. This was caused by *C. barbouri*, because removal of that taxon in analyses 81–90 allowed turtles and *E. africanus* to be present exclusively in Diapsida. With *C. barbouri* removed, only four characters (174, 247, 251 and 253) support the exclusive turtle-*Eunotosaurus* grouping, regardless of implied weighting. With supporting characters removed, *Eunotosaurus* tends to form a clade with *Acerosodontosaurus piveteaui* (data not shown), but this clade lacks any bootstrap support and collapses during bootstrapping into polytomy at the base of Pan-Testudines. To check whether the lacking tree resolution results from the removal of mentioned characters or from interaction of *E. africanus* with other taxa, I performed two analyses excluding *C. barbouri*, *E. africanus*, and characters 174, 247, 251 and 253 with and without implied weighting (*K* = 2.75). Without *E. africanus* the tree resolution improved, and analysis 92 (implied weighting on) yielded the tree with the best-resolved and best-supported Eureptilia (figure 3). Typically for the matrix used, the support for most clades is very poor, but the clade of *Orovenator mayorum* and more derived diapsids (including turtles as the sister-group to Lepidosauromorpha) attained the bootstrap value of 60—the highest score for the turtle-containing diapsid clade obtained in all the 92 analyses.

## 5. Discussion

### 5.1. The homeotic shift in early turtles

The gradual change of turtle PSV8 morphology in geological time strongly suggests a homeotic background, and the observed intermediate morphology resembles that occurring in some gain-of-function, loss-of-function or overexpression experiments on the *Hox* genes [21]. Several *Hox* genes belonging to the *Hox-5*, *Hox-7*, *Hox-8* and *Hox-9* families were shown to influence the morphology of the cervico-dorsal transition, and the *Hox-6* family determines the anterior limit of the dorsal section[21–23]. The common defects among the loss-of-function mutants for these genes usually involve the anterior homeosis of the last CV and the first DV, with notable reduction or loss of the first dorsal rib [21]. In single-gene mutants, the changes are usually unilateral and a large variability in penetrance and effect strength exists among individuals due to functional redundancy of other paralogues, but in paralogous mutants the penetrance may reach 100%, the effect is stronger and often bilateral [21]. Although the expression ranges for only several *Hox* genes are known for turtles (*Hoxa-5*, *Hoxb-5*, *Hoxc-6*, *Hoxa-7*, *Hoxb-7*, *Hoxc-8*) [20], the expression pattern for some of them is unusual and may indeed suggest a shift of expression. Most notably the somitic expression domain of *Hoxb-5* in turtles is restricted to the cervical region (from CV1 to CV7), while in crocodilians, birds and mammals it spans from CV2 to the caudal region of the embryo [20,24]. Similarly, the somitic expression domain of *Hoxa-5* spans from CV2 to the anterior half of the first dorsal prevertebra, while in crocodilians and mammals it enters the anterior dorsal region (in birds, however, the expression domain of *Hoxa-5* is restricted to the neck, and the expression pattern of this gene shows high flexibility among vertebrates) [20,25]. The spreading apart of the expression ranges of *Hox-5* and *Hox-6* genes in turtles (figure 1) may potentially be explained either by a cranial shift of the posterior limit of *Hox-5* expression or by a caudal shift of the anterior limit of *Hox-6* expression, and several lines of evidence support the latter hypothesis.

Firstly, the loss or reduction of the first dorsal rib in some *Hox-5* mutants occurs within the expression domain of *Hox-6* [21]*.* In modern turtles *Hox-6* range does not reach PSV8 [20], so the loss of the dorsal-like morphology of that vertebra cannot be explained by its sole exclusion from the *Hox-5* products' influence. *Hoxa-5* loss-of-function mutant mice were reported to often have large ribs on CV7, but the *Hoxa-5^-/-^* homozygotes exhibit increased perinatal lethality [26] and in modern turtles *Hoxa-5* is expressed at the level of PSV8 [20], so reversion would be needed. Therefore, I consider the anterior shift of *Hox-5* expression in turtles without the change of *Hox-6* expression domain to be unlikely.

Secondly, turtles are unique among other vertebrates in their restriction of the brachial plexus and scapula-building somites to the cervical region of the body [20,22,26,27]. The scapula in turtles derives from somites 8–12 that give rise to CV4–7 [26], but in modern turtles it chondrifies and ossifies at the level of CV8 [28], which indicates that prochondrocytes migrate one segment caudally before the chondrification starts. Although the anteroposterior position of the scapula is partially dependent on *Hoxb-5*, the position of the brachial plexus is not, as shown by homozygotic *Hoxb-5* mutant mice, which tend to have the pectoral girdle shifted rostrally, but the brachial plexus retaining its original position [29], which is not the case in turtles.

Thirdly, the presence of an additional anterior marginal scute in the Late Triassic turtles indicates that an additional somite was included in the carapace. The scutes on the turtle shell are derived from placodes, whose positions are strongly linked to the body segmentation [30,31]. Particularly, the marginal scute-forming placodes appear at every mioseptum and in modern turtles correspond to somites 12–24, from which the last two CVs, all DVs and two sacral vertebrae are derived [22,31,32]. In most Jurassic turtles (stem turtles and the most basal representatives of Pleurodira and Cryptodira; see the electronic supplementary material) the first pleural scute contacts marginals 2–5, the second pleural—marginals 5–7, the third—7–9, and the fourth—9–11. This layout thus appears to be plesiomorphic for Testudines. In *Proganochelys quenstedti* and *Proterochersis* spp., on the other hand, it is different. For *Proterochersis* spp. the correspondence is: first pleural—marginals 3–6, second—6–8, third—8–10, and fourth—10–12 or 13; and for most *Prog. quenstedti* specimens (excluding MB 1910.45.2) it is the same, with the exception of the last pleural, which corresponds to marginals 10–14 (the supramarginals are ignored here, as their presence and layout varies among the basal turtles and their impact on the layout of marginals in relation to pleurals is negligible). The marginal count in these two Late Triassic turtles is therefore shifted in such a way that the number of each marginal corresponding to the anterior limit of each pleural is one greater than in the Jurassic turtles. This is best explained by addition of a surplus scute in front of the marginal row (electronic supplementary material, table S1; see the electronic supplementary material for discussion on MB 1910.45.2 and scute layout). Interestingly, in *Keuperotesta limendorsa* PSV8 is free both from carapace and from the dorsal vertebral column, and this taxon lacks the additional anterior marginal, despite its great overall similarity to *Proterocheris* spp. [10,33]. This may support the notion that both the loss of the first marginal and exclusion of the PSV8 is indeed correlated and occurred as a result of co-option of the anteriormost dorsal segment to the cervical region (but see discussion of *K. limendorsa* below). A larger inter- and intra-specific variability is evident for the posterior marginals of the Late Triassic turtles, but the posterior section of the carapace generally exhibits more variation in turtles [34], and the most posterior marginals were derived from sacral and anterior caudal somites—this region thus may slightly differ in terms of regulation and lacks a strict correspondence with endoskeleton, typical for the presacral part of the carapace.

The varied morphology of PSV8 in *Prog. quenstedti* may indicate that the caudal shift of expression did not occur abruptly, but individually for all *Hox-6* paralogues. Only the expression range for *Hoxc-6* is known for turtles [20], but at least in mouse and chicken all of *Hox-6* paralogues have the same ranges, with anterior limit at DV1 [24]. Loss-of-function of individual *Hox-6* paralogues (or the posterior shift of their expression, which in both cases excludes DV1 from their normal influence) results in partial anterior homeosis of DV1, including partially penetrant reduction of its ribs, which tend to become shorter and often articulate with the second pair of dorsal ribs [21]—morphology very reminiscent of MB 1910.45.2 [11]. Notably, DV1 of modern turtles is also reduced. Both its ribs and the neural spine are no longer contributing to the carapace, and the ribs are short, minute, and usually articulate with the second pair of dorsal ribs, very similarly to PSV8 in *Prog. quenstedti*. This apparent recapitulation morphology, however, occurs from other reasons: in modern turtles DV1 develops under the normal influence of *Hox-6*, but is entirely excluded from the influence of *Hoxb-5*, and *Hoxa-5* is expressed only in the anterior half of the prevertebra [20]. DV1 was already excluded from the carapace in *Prog. quenstedti* [11] (but not in *Proterochersis* spp. [10]), probably as an effect of partial decoupling of *Hox-6* and *Hox-5* expression (figure 1).

### 5.2. The turtle relationships

The interpretation presented above cannot be considered in separation from the phylogeny. Three stem-testudinates, *Eunotosaurus africanus*, *Pappochelys rosinae* and *Odontochelys semitestacea*, were described as having only nine DVs [5–7]. The holotype of *O. semitestacea* was not available for revision, but its dorsal spine is ankylosed [35], making it difficult, if at all possible, to properly discern the limits of particular DVs, and my study of the other two described specimens strongly suggests that *O. semitestacea* possessed at least 18 presacrals (electronic supplementary material, figure S1; see the electronic supplementary material for discussion). All the known *Pa. rosinae* specimens are disarticulated and incomplete [7] so the exact number of DVs is currently unknown.

The simple phylogenetic test performed herein shows several previously undiscussed aspects of the current state of knowledge about the turtle origin and relationships. The recently resurrected proposal of the turtle-*Eunotosaurus* affinity [5,17], first proposed by Watson [36], is seemingly supported by numerous synapomorphies. My analysis, however, indicates that no more than four characters (which comprise 1.44% of all the characters present in the matrix) or, in a less favourable setting (all taxa and implied weighting), only two characters (0.72% of all characters, with notion about an uncertain nature of one of them—see discussion for analyses 1–32) support the exclusive turtle-*Eunotosaurus* clade. That means that all the other shared characters listed for the less-resolved trees yielded by other analyses are in fact not real, meaningful synapomorphies of that clade, but plesiomorphies inherited after the possible diapsid ancestor(s) of turtles and *Eunotosaurus*, and thus have only lesser phylogenetic value, allowing for only general ideas about the vague relationship of turtles and *Eunotosaurus*, and not a close affinity. That would automatically explain their wide distribution among the taxa. Notably, all of the characters forcing the turtle-*Eunotosaurus* grouping apply to the dorsal axial skeleton (presacral vertebral count, presence of T-shaped ribs, trunk length, number of broadened ribs) and are closely related to each other. There are no unique synapomorphies in the skull. Such a regionalization may be interpreted as a sign of a common origin of all four characters due to the same selection factor, thereby increasing the possibility of homoplasy. In fact, such interpretation was first proposed by Gow & de Klerk [37] and recently developed by Lyson *et al*. [38] as an effect of a fossorial ecology of *E. africanus*.

Lyson *et al*. [38] showed several characters, mostly located in forelimbs, to be shared by turtles and *E. africanus*. The scorings of these characters were not included in my matrix, because my analyses were finished before the publication of Lyson's *et al*. [38] paper. My observations indicate, however, that all of these proposed common characters share two main problems of many other putative synapomorphies, shown herein to lack phylogenetic meaning. Firstly, most of them are pretty common (for example, the acromion process, large unguals and well-developed deltopectoral crest and olecranon are present also in pareiasaurs, some dinosaurs, the last two characters also in captorhinids, etc.). The ecologically induced adaptations linked to movement are usually poor phylogenetic characters, because they tend to be acquired by numerous unrelated taxa as convergences. Secondly, all of these characters are either absent or weakly expressed in *Pa. rosinae* and *O. semitestacea*, and become more conspicuous in more derived testudinates. This indicates that they most likely are induced in turtles by different factors following the gradual development of the shell, and not precede the shell altogether. I propose that they are rather connected to the increased body mass and restrictions of limb movement caused by the appearance of the plastron and (later) the carapace. This, however, must be verified. The large unguals of *O. semitestacea*, proposed by Lyson *et al*. [38] to be a proof of initially fossorial ecology of turtles, are also developed (if not better) on the hind legs of that animal, thus declining the forelimb-driven digging as the explanation of their development. Additionally, large unguals are common among the terrestrial turtles, being present in the Late Triassic turtles, many testudinids [39], and meiolaniids [40], and even in some aquatic Pelomedusoides [41], in many cases not necessarily related to the digging behaviours, but rather aiding the locomotion on the land.

In no analysis the reduced dorsal vertebral count was treated as a synapomorphy of the turtle-*Eunotosaurus* clade. In analyses including *Prog. quenstedti*, the lowered number of DV was considered an autapomorphy of *E. africanus*, and in analyses excluding *Prog. quenstedti* the plesiomorphic state for the turtle-*Eunotosaurus* clade was unknown. This is congruent with my observations suggesting that the homeotic change occurred after the acquisition of the shell.

Despite the low support values in normal analyses including all taxa, the turtle-*Pappochelys* clade is well supported in most analyses utilizing implied weighting and in normal analyses excluding *E. africanus*, and both *Pa. rosinae* and more derived pan-testudinates are individually nested in similar topological environments, reaffirming the status of *Pa. rosinae* as a stem turtle. The situation of *E. africanus*, on the other hand, is more complicated. The turtle-*Eunotosaurus* clade attained decent support only in analyses excluding *Pa. rosinae* and, when tested individually or with *Pa. rosinae* only, *E. africanus* is nested in different spots on the tree. While small support or lack of thereof does not necessarily mean that the clade is invalid (and, actually, most of the resolved clades obtained from that matrix have very low support), such large shifts of position are troubling. While some changes of position are expected depending on the character and taxon sampling (for example, sometimes the more derived taxa may help to properly identify autapomorphies and plesiomorphies of a more basal taxon), it is evident that the position of *E. africanus* is unstable and more dependent on the position of turtles, than the other way around. Although some settings may produce a robust list of turtle-*Eunotosaurus* synapomorphies, depending on the current tree topology, most of these shared characters are common among the other taxa, poorly sampled or are just typical diapsid traits. Only 2–4 characters actually support such a grouping, and all of these are grouped in one body region, related to each other, and most likely they originate from the same ecology-related selection factor, and thus have limited phylogenetic weight. Besides that, nearly all the features in common are also shared with numerous other taxa. The possibly homoplastic nature of the characters that link *Eunotosaurus* to turtles was also acknowledged recently by Hirayama & Nakajima [42].

While *E. africanus* remains currently the most likely relative of the turtle ancestors, even if only because of four characters of dubious phylogenetic weight, more data are needed to unambiguously confirm or refute such grouping. First of all, many characters need to be better sampled. For example, the rib osteology of numerous taxa should be checked for possible resemblances to the turtle ossification model. Particularly, saurosphargids and archosaurs seem to be worthy subjects of such research. This is also especially true for character 257 (Sharpey's fibres on ventral portion of dorsal ribs)—due to nearly non-existent sampling (sampled only for *E. africanus*, *Pa. rosinae*, *Prog. quenstedti* and Squamata) this character never appears as synapomorphy or autapomorphy. Secondly, more data are needed about the most basal testudinates and *C. barbouri*, which proved to interact with *E. africanus* and stem turtles, and thus to have conspicuously large impact on turtle support, even though these taxa never formed a well-supported clade. Thirdly, most likely some new discoveries are needed. The debate about the turtle relationships is far from over. Possibly only corroboration of future fossils with the molecular data will bring a satisfying conclusion.

### 5.3. Morphology of *Proterochersis* spp.: plesiomorphic versus autapomorphic

The possibility that the fused PSV8 is an autapomorphy of *Proterochersis* spp. must be discussed. Although it cannot be currently ruled out with full confidence, it seems to be unlikely for several reasons. Firstly, the restriction of the brachial plexus, scapula-building somites and expression range of *Hoxb-5* and *Hoxa-5* to cervical region in modern turtles all are best explained as a result of a more anterior position of the cervico-dorsal transition in their ancestor. There is no adequate morphology seen in any post-Triassic taxa [1,13,16] but the morphology seen in *Proterochersis* spp. seem to represent such an ancestral state well. Secondly, the morphologies seen in *Proterochersis* spp., *Prog. quenstedti* and more derived turtles fit into a consistent morphocline spanning well beyond the Triassic. The gradual exclusion of PSV8 from the carapace and then the reduction of its ribs are well expressed and congruent both with the stratigraphic age of discussed taxa and their phylogenetic position [10]. The posterior shift of the cervico-dorsal transition also correlates well with the gradual exclusion of the ribs of the ninth presacral from the carapace and their subsequent reduction, which is very well documented in turtles [10,11,16]. If the dorsal-like morphology of PSV8 in *Proterochersis* spp. is an autapomorphy, then the (imperfect) dorsal-like characteristics of the same vertebra present in *Prog. quenstedti* (large ribs and sutural contact to the carapace) would also have to be acquired independently and rapidly against this trend, which is unparsimonious. Thirdly, the shortening of the neck and elongation of the fully stiffened trunk seems to have little to no adaptive sense. The stiffening of the tetrapod trunk is in virtually all known cases related to its shortening [4], which is understandable from the point of biomechanics (lowering of the number of articulations between the segments is a way to optimize the stresses and enhances its stiffness, and proportionally shorter trunk is lighter, which is especially important when it is heavily armoured). A longer neck is in such a case beneficial, increasing the head reach and range of movements (resulting in larger coverage during feeding and potentially in slightly increased capabilities of active defence). The trend towards reduction of the trunk elements and elongation of the neck is well known and obvious in turtles [1,10,13,16]. On the other hand, there seems to be no adaptive explanation for the turning of a cervical vertebra into a dorsal vertebra in turtles after the shell acquisition, especially independently twice, in *Proterochersis* spp. and *Prog. quenstedti*. Finally, the longer trunk is plesiomorphic for turtles according to most of the phylogenetic scenarios presented above (in the other, the plesiomorphic state is unknown). A short neck (below eight CVs) and a long trunk (above 10 DV) are plesiomorphic for Eureptilia, and elongation of the neck occurred as a specialization within several exclusive clades (figure 3). Turtle position in all well-resolved topologies obtained here supports the hypothesis that these plesiomorphic features were inherited by the turtle ancestor, and only later followed by the changes to the vertebral formula.

Somewhat troubling in that regard is *Keuperotesta limendorsa*, at first identified as *Proterochersis robusta* by Joyce *et al*. [33] and later named as a new proterochersid genus and species by Szczygielski & Sulej [10]. This turtle was described as having an unfused PSV8 with long cervical ribs [10,33]. If the condition seen in *Proterochersis* spp. and *Prog. quenstedti* is indeed plesiomorphic, then the free PSV8 of *K. limendorsa* must be a result of one of the four possible scenarios: either (i) *K. limendorsa* is not a proterochersid, but some more derived, unrelated turtle, which branched off after the anterior homeosis of PSV8; alternatively, *K. limendorsa* and *Proterochersis* spp. are related, but Proterochersidae as defined in Szczygielski & Sulej [10] is paraphyletic in relation to crown Testudines (this would be incongruent with phylogeny from Szczygielski & Sulej [10], most probably due to the incompleteness of that turtle); (ii) the exclusion of PSV8 from the dorsal section of the vertebral column happened independently in *K. limendorsa* and other turtles; (iii) the fusion of PSV8 to carapace and following vertebrae is ontogenetic; (iv) the vertebra considered as PSV8 in *K. limendorsa* was wrongly identified by Joyce *et al*. [33] and Szczygielski & Sulej [10], in fact it is PSV7, and PSV8 is fused in *K. limendorsa* like in *Proterochersis* spp. Scenario (i) does not pose a problem for the discussed conception, because it does not refute the plesiomorphic state in *Proterochersis* spp., but may only be verified when more material of *K. limendorsa* is available. Scenario (ii) may at first seem to be equally probable as the independent acquisition of the dorsal-like characteristics by PSV8 in *Proterochersis* spp. and *Prog. quenstedti*, but taking into account the arguments presented above (the independent developmental and morphological clues encompassing the cervical restriction of the brachial plexus, scapula-building somites and *Hox-5* expression ranges, the trend towards reduction of PSV8 visible not only in the Triassic taxa, but also in more derived turtles, the adaptiveness of the changes, and the polarity of evolutionary changes supported by phylogenetic analyses) it seems to be more parsimonious. Scenario (iii) is incongruent with observations. The holotype of *K. limendorsa* (SMNS 17757, approximately 38.5 cm long, but missing a few centimetres of the posterior part of the carapace) is approximately the same size as the holotype of *Prot. porebensis* (ZPAL V.39/48, approximately 42.5 cm long). Both are larger than most specimens of *Prot. robusta*. The last cervical appears to be fused even in much smaller specimens of the latter (e.g. SMNS 16603, approximately 32 cm long; see also discussion in Szczygielski and Sulej [10]). Scenario (iv) is currently impossible to verify due to incomplete preparation of *K. limendorsa* holotype (SMNS 17757). An inspection of the relative positions of the discussed vertebra and the pits for the dorsal scapular processes visible on the visceral surface of the carapace may in fact support such a possibility—PSV9 *sensu* Joyce *et al.* [33] and Szczygielski & Sulej [10] of *K. limendorsa* appear to indeed better correspond to the position of PSV8 of *Prot. porebensis*. Until the specimen is better prepared, it remains unknown whether this results from the interspecific variation, different shell geometry or improper initial identification. If the latter is true, the morphology of proterochersids would be even more unique than previously thought. The ribs on CV7 would be long and slender, unlike cervical ribs of any other turtle, including the Late Triassic forms, with the exception of PSV8 in *Prog. quenstedti* [6,11], and the ribs of DV1 (PSV8) would be broadened dorso-caudally and possibly articulate with the carapace similarly to the ribs of PSV9 in *Prog. quenstedti* (this is ambiguous in SMNS 17757, because the ribs of that vertebra are broken; careful observation of the visceral surface of the carapace in *Prot. porebensis* reveals small rugosities or splinters of bone behind the scapular pits, which may potentially be remnants of this articulation; figure 2). Out of the scenarios discussed above, I consider scenario (iv) to be most probable at the moment, but more data will be gathered in the future. Even if PSV8 o *K. limendorsa* is fused, the other characters differentiating this taxon from *Proterochersis* spp. are in place, so it remains taxonomically separate.

## 6. Conclusion

The exclusion of the anteriormost DV into the neck section conforms to the general trend of shell simplification evident in turtle evolution [1] and is understandable from the evolutionary point of view—the selection towards the elongation and increase of movability of the neck is crucial for animals with fully stiffened trunk.

According to Böhmer *et al*. [43] *Hox* code may be deduced from fossils based on morphology. Testudinata is the first taxon with such a great fossil record of homeosis in action. This may be impactful in several ways.

Firstly, the observation of the morphological changes over the course of millions of years enables new insight into the homeotic patterning in vertebrates, in a way fundamentally different from laboratory-based research. While lacking the controllability of the experimental methods, it allows for a more true-to-life study of the interconnections between the homeotic patterning, the morphology of the animal and its environment. A natural pace of changes may be evaluated based on the stratigraphic age of the specimens.

Secondly, the loss-/gain-of-function experiments on modern animals may be used to emulate the intermediate and plesiomorphic phenotypes, and a step-by-step succession of molecular and morphological states that occurred during phylogeny may be recreated. The expression ranges of *Hox* genes in fossil forms may be assessed using the geometric morphometrics [43].

Thirdly, the homeotic shift hypothesis explains several turtle-exclusive peculiarities, including the restriction of scapula-building somites and brachial plexus to the neck. These changes were acquired after the shell formation and were not prerequisites to the enclosure of the pectoral girdle under the carapace.

Finally, these observations may improve character polarization for phylogenetic analyses, potentially influencing our understanding of turtle evolution and ancestry.

Future palaeontological discoveries will supplement our knowledge about the morphology of stem turtles and early testudinates, and improve the temporal resolution of their phenotypic evolution. Additionally, the expression ranges of the remaining homeotic genes should be studied in modern turtles.

## Supplementary Material

supplementary information

## Data accessibility

All the data produced during this research are included in the electronic supplementary material published alongside this work. Data available from the Dryad Digital Repository: http://dx.doi.org/10.5061/dryad.g5v64 [44].
