# Supplementary material for: Homeotic shift at the dawn of the turtle evolution
Source: R Soc Open Sci. 2017 Apr 5;4(4):160933. doi: 10.1098/rsos.160933 (PMC5414250; doi:10.1098/rsos.160933)
Supplement: supplementary information [file rsos160933supp1.pdf]

# HOMEOTIC SHIFT AT THE DAWN OF THE TURTLE EVOLUTION

## SUPPLEMENTARY INFORMATION:

**EXTENDED DISCUSSION,**

**CHARACTER LIST,**

**CHARACTER MATRIX,**

**PHYLOGENETIC TREES,**

**AND**

**LISTS OF SYNAPOMORPHIES**

**TOMASZ SZCZYGIELSKI[1,*]**

[1]Institute of Paleobiology, Polish Academy of Sciences, Twarda 51/55, 00-818 Warsaw, Poland

*E-mail: t.szczygielski@twarda.pan.pl

**CONTENTS**


Institutional abbreviations ........ 4

Scute pattern of early turtles ........ 5

Vertebral column of *Odontochelys semitestacea* ........ 8

Character list ........ 13

Character matrix ........ 24

Changes to the matrix ........ 41

Phylogenetic trees and lists of synapomorphies ........ 44

Analysis 1 (all taxa) ........ 44
Analysis 2 (no *Proganochelys quenstedti*) ........ 49
Analysis 3 (no *Candelaria barbouri*) ........ 54
Analysis 4 (no *Pappochelys rosinae*) ........ 58
Analysis 5 (no *Odontochelys semitestacea*) ........ 63
Analysis 6 (no *Eunotosaurus africanus*) ........ 68
Analysis 7 (no *Proganochelys quenstedti* and *Candelaria barbouri*) ........ 72
Analysis 8 (no *Proganochelys quenstedti* and *Pappochelys rosinae*) ........ 76
Analysis 9 (no *Proganochelys quenstedti* and *Odontochelys semitestacea*) ........ 80
Analysis 10 (no *Proganochelys quenstedti* and *Eunotosaurus africanus*) ........ 85
Analysis 11 (no *Candelaria barbouri* and *Pappochelys rosinae*) ........ 90
Analysis 12 (no *Candelaria barbouri* and *Odontochelys semitestacea*) ........ 94
Analysis 13 (no *Candelaria barbouri* and *Eunotosaurus africanus*) ........ 98
Analysis 14 (no *Pappochelys rosinae* and *Odontochelys semitestacea*) ........ 102
Analysis 15 (no *Pappochelys rosinae* and *Eunotosaurus africanus*) ........ 107
Analysis 16 (no *Odontochelys semitestacea* and *Eunotosaurus africanus*) ........ 111
Analysis 17 (no *Proganochelys quenstedti*, *Candelaria barbouri*, and *Pappochelys rosinae*) ........ 116
Analysis 18 (no *Proganochelys quenstedti*, *Pappochelys rosinae*, and *Odontochelys semitestacea*) ........ 120
Analysis 19 (no *Proganochelys quenstedti*, *Odontochelys semitestacea*, and *Eunotosaurus africanus*) ........ 125
Analysis 20 (no *Proganochelys quenstedti*, *Candelaria barbouri*, and *Odontochelys semitestacea*) ........ 129
Analysis 21 (no *Proganochelys quenstedti*, *Candelaria barbouri*, and *Eunotosaurus africanus*) ........ 134
Analysis 22 (no *Proganochelys quenstedti*, *Pappochelys rosinae*, and *Eunotosaurus africanus*) ........ 138
Analysis 23 (no *Proganochelys quenstedti*, *Candelaria barbouri*, *Pappochelys rosinae*, and *Odontochelys semitestacea*) ........ 143
Analysis 24 (no *Proganochelys quenstedti*, *Candelaria barbouri*, *Pappochelys rosinae*, and *Eunotosaurus africanus*) ........ 148
Analysis 25 (no *Proganochelys quenstedti*, *Candelaria barbouri*, *Odontochelys semitestacea*, and *Eunotosaurus africanus*) ........ 153
Analysis 26 (no *Proganochelys quenstedti*, *Pappochelys rosinae*, *Odontochelys semitestacea*, and *Eunotosaurus africanus*) ........ 157
Analysis 27 (no *Candelaria barbouri*, *Pappochelys rosinae*, *Odontochelys semitestacea*, and *Eunotosaurus africanus*) ........ 161
Analysis 28 (no *Pappochelys rosinae*, *Odontochelys semitestacea*, and *Eunotosaurus africanus*) ........ 165
Analysis 29 (no *Candelaria barbouri*, *Odontochelys semitestacea*, and *Eunotosaurus africanus*) ........ 169
Analysis 30 (no *Candelaria barbouri*, *Pappochelys rosinae*, and *Eunotosaurus africanus*) ........ 173
Analysis 31 (no *Candelaria barbouri*, *Pappochelys rosinae*, and *Odontochelys semitestacea*) ........ 177
Analysis 32 (no *Proganochelys quenstedti*, *Candelaria barbouri*, *Pappochelys rosinae*, *Odontochelys semitestacea*, and *Eunotosaurus africanus*) ........ 182
Analysis 33 (all taxa, implied weighting, K = 1) ........ 186
Analysis 34 (all taxa, implied weighting, K = 2) ........ 191
Analysis 35 (all taxa, implied weighting, K = 2.375) ........ 196
Analysis 36 (all taxa, implied weighting, K = 2.5) ........ 201
Analysis 37 (all taxa, implied weighting, K = 2.625) ........ 205
Analysis 38 (all taxa, implied weighting, K = 2.75) ........ 210
Analysis 39 (all taxa, implied weighting, K = 2.875) ........ 214
Analysis 40 (all taxa, implied weighting, K = 3) ........ 218
Analysis 41 (all taxa, implied weighting, K = 3.125) ........ 222

Analysis 42 (all taxa, implied weighting, K = 3.25) ....227
Analysis 43 (all taxa, implied weighting, K = 3.375) ....232
Analysis 44 (all taxa, implied weighting, K = 3.5) ....237
Analysis 45 (all taxa, implied weighting, K = 3.625) ....242
Analysis 46 (all taxa, implied weighting, K = 3.75) ....247
Analysis 47 (all taxa, implied weighting, K = 3.875) ....252
Analysis 48 (all taxa, implied weighting, K = 4) ....257
Analysis 49 (all taxa, implied weighting, K = 4.125) ....262
Analysis 50 (all taxa, implied weighting, K = 4.25) ....267
Analysis 51 (all taxa, implied weighting, K = 4.375) ....272
Analysis 52 (all taxa, implied weighting, K = 4.5) ....277
Analysis 53 (all taxa, implied weighting, K = 4.625) ....282
Analysis 54 (all taxa, implied weighting, K = 4.75) ....287
Analysis 55 (all taxa, implied weighting, K = 4.875) ....292
Analysis 56 (all taxa, implied weighting, K = 5) ....297
Analysis 57 (all taxa, implied weighting, K = 5.125) ....302
Analysis 58 (all taxa, implied weighting, K = 5.25) ....307
Analysis 59 (all taxa, implied weighting, K = 5.375) ....312
Analysis 60 (all taxa, implied weighting, K = 5.5) ....317
Analysis 61 (all taxa, implied weighting, K = 6) ....322
Analysis 62 (all taxa, implied weighting, K = 10) ....327
Analysis 63 (all taxa, implied weighting, K = 100) ....332
Analysis 64 (all taxa, implied weighting, K = 1000) ....337
Analysis 65 (no *Candelaria barbouri* and *Eunotosaurus africanus*, implied weighting, K = 2.75) ....342
Analysis 66 (no *Proganochelys quenstedti*, *Candelaria barbouri*, and *Eunotosaurus africanus*, implied weighting, K = 2.75) ....346
Analysis 67 (no *Candelaria barbouri* and *Pappochelys rosinae*, implied weighting, K = 2.5) ....350
Analysis 68 (no *Candelaria barbouri*, *Pappochelys rosinae*, and *Odontochelys semitestacea*, implied weighting, K = 2.5)....354
Analysis 69 (no *Proganochelys quenstedti*, *Candelaria barbouri*, and *Odontochelys semitestacea*, implied weighting, K = 2.75) ....358
Analysis 70 (no *Candelaria barbouri*, implied weighting, K = 2.75)....363
Analysis 71 (all taxa, no character 253) ....367
Analysis 72 (all taxa, implied weighting, K = 2.75, no character 253) ....372
Analysis 73 (all taxa, no characters 247 and 253) ....376
Analysis 74 (all taxa, implied weighting, K = 2.75, no characters 247 and 253)....381
Analysis 75 (all taxa, no characters 247, 251, and 253) ....386
Analysis 76 (all taxa, implied weighting, K = 2.75, no characters 247, 251, and 253)....391
Analysis 77 (all taxa, no characters 174, 247, 251, and 253) ....396
Analysis 78 (all taxa, implied weighting, K = 2.75, no characters 174, 247, 251, and 253)....401
Analysis 79 (all taxa, no characters 174, 204, 247, 251, and 253) ....406
Analysis 80 (all taxa, implied weighting, K = 2.75, no characters 174, 204, 247, 251, and 253) ....411
Analysis 81 (no *Candelaria barbouri*, no character 253)....416
Analysis 82 (no *Candelaria barbouri*, implied weighting, K = 2.75, no character 253)....420
Analysis 83 (no *Candelaria barbouri*, no characters 247 and 253) ....424
Analysis 84 (no *Candelaria barbouri*, implied weighting, K = 2.75, no characters 247 and 253) ....428
Analysis 85 (no *Candelaria barbouri*, no characters 247, 251, and 253)....432
Analysis 86 (no *Candelaria barbouri*, implied weighting, K = 2.75, no characters 247, 251, and 253) ....436
Analysis 87 (no *Candelaria barbouri*, no characters 174, 247, 251, and 253)....440
Analysis 88 (no *Candelaria barbouri*, implied weighting, K = 2.75, no characters 174, 247, 251, and 253) ....445
Analysis 89 (no *Candelaria barbouri*, no characters 174, 204, 247, 251, and 253)....450
Analysis 90 (no *Candelaria barbouri*, implied weighting, K = 2.75, no characters 174, 204, 247, 251, and 253)....454
Analysis 91 (no *Candelaria barbouri* and *Eunotosaurus africanus*, no characters 174, 247, 251, and 253) ....459
Tables ....467

## INSTITUTIONAL ABBREVIATIONS

**MB** – Museum für Naturkunde, Berlin, Germany.

**IVPP** – Institute of Vertebrate Paleontology and Paleoanthropology, Beijing, China.

**SMNS** – Staatliches Museum für Naturkunde Stuttgart, Stuttgart, Germany.

**ZPAL** – Institute of Paleobiology, Polish Academy of Sciences, Warsaw, Poland.

## Scute pattern of early turtles

The number and position of placodes giving rise to the marginal scutes of turtles are shown to result from the body segmentation (Cherepanov 2015; Moustakas-Verho & Cherepanov 2015). Therefore, they may be used to estimate the number of segments building the shell, at least in the anterior (less variable) region of the carapace. In the Triassic taxa the number of scutes (and thus, the placodes) was greater than in more derived turtles, and the largest difference can be seen in the number of marginal scutes: *Proterochersis* spp. has 14-15 marginals (Szczygielski & Sulej 2016), and *Proganochelys quenstedti* typically 16 (Gaffney 1990). This number is higher than in more derived turtles, that usually have 12 marginals. When compared to the derived turtles, the layout of the marginals (1-n) relative to pleurals (I-IV) indicates, that an additional scute is present at the cranial end of marginal series both in *Proterochersis* spp. and in *Prog. quenstedti*.

*Proterochersis* spp. (from Szczygielski & Sulej 2016) and *Proganochelys quenstedti* (from Gaffney 1990):
I – 3-6, II – 6-8, III – 8-10, IV – 10-12 or 10-13.

*Kayentachelys aprix* (from Gaffney 1990 and Gaffney et al. 1987), *Eileanchelys waldmani* (from Anquetin 2010), *Heckerochelys romani* (from Sukhanov 2006), *Pleurosternon bullocki* (from Owen 1849-1884), *Notoemys laticentralis* (from Cadena et al. 2013), and *Platychelys oberndorferi* (from Bräm 1965):
I – 2-5, II – 5-7, III – 7-9, IV – 9-11.

The latter arrangement is exhibited by at least most of the stem turtles and appears to be plesiomorphic for the crown group, although it is averted e.g. by plesiochelyids (their first pleural contacts marginals 1-4 or 2-4, most likely due to their increased breadth – see Anquetin et al. 2014) and more or less by baenids (Gaffney 1972). This change appears to be correlated with the shift of PSV8 identity from dorsal to cervical.

In *Prog. quenstedti* specimen MB 1910.45.2 there is yet another marginal scute at the cranial end of the marginal row, and interestingly this is the only specimen of that taxon that exhibits the sutural contact between PSV8 and the carapace (Gaffney 1990). These two characters are not necessarily connected, however, because the sutural contact of PSV8 with the carapace appears to be related to age (in ontogenetically younger specimens the vertebra is not sutured but still articulates with the shell, and the suture seems to be the secondary modification of that articulation – see Gaffney 1990), and in turtles there exists some low variability in the number of marginals and peripherals, resulting in appearance of rudimentary additional elements in some specimens. The presence of an extra pair of marginals is most likely not related to age, because all the scutes form *in ovo*, all the peripherals start to ossify near to or soon after clutching, and there are no documented cases of their multiplication in adults.

One additional anterior somite contributing to the shell of proterochersids does not result in an increase of number of pleurals or vertebrals. This can be explained by spatial constraints. In modern turtles the first marginal placode develops at the level of the mioseptum 12 and the last marginal placode at the level of mioseptum 23. The first pleural placode, however, develops at the level of mioseptum 14, and the last at the level of mioseptum 20. This means, that there is a margin of two segments at the cranial end, and of three segments at the caudal end of the pleural series. It may be interpreted that at least two “empty” segments must be present before the first and after the last pleural placode, so the marginal series and cervical scute may encircle the carapace as an uninterrupted row. Such a margin appears to explain why in kinosterids, that have only 11 marginals, the number of pelurals and vertebrals does not change (even after reduction still there is a 2-somine margin), but the increase of marginal count to 13 in some chelonioids is correlated with addition of one pleural pair. With one additional anterior segment this prerequisite would not be met, and another segment would be needed to provide a two-segment margin in front of an additional pleural placode over mioseptum 12. Thus, such placode most likely did not develop or, if it did, the lack of space resulted in its early fusion with neighboring placodes, making it impossible to detect post-hatching. Such a potential fusion may be suggested by the unusual shape of the first vertebral scute of these turtles, but such interpretation remains speculative. In *Prog. quenstedti* the scute layout is more complex and especially the supramarginal series makes the interpretation difficult. It is possible, that the first supramarginal scute belongs to the pleural series, but again, this remains speculative. *Prog. quenstedti* has only four vertebral scutes, but the position of the posterior sulcus

**Supplementary Table 1.** Correspondence of somites, vertebrae, ribs (initially and after the anterior shift) and carapace placodes in turtles. Placodes in *Proterochersis* spp. and *Proganochelys quenstedti* inferred from morphology (see text for discussion). CV – cervical verterbra, PSV – presacral vertebra; DV – dorsal vertebra; CaV – caudal vertebra; CR – cervical rib; PSR – presacral rib; DR – dorsal rib; SR – sacral rib; CaR – caudal rib; M – marginal placode; P – pleural placode; C – cervical placode; V – vertebral placode. The presence of elements in brackets is varied between specimens and traditional names of elements of *Prog. quenstedti* are given in quotes.

| Somite | Vertebra | | Rib | | Placode | | | | | | | | |
|---|---|---|---|---|---|---|---|---|---|---|---|---|---|
| | | | Initial position | After shift | Modern turtles | | | *Proterochersis* spp. | | | *Proganochelys quenstedti* | | |
| | | | | | M | P | V | M | P | V | M | P | V |
| 1-4 | Occiput | | - | | - | | | - | | | - | | |
| 5 | 1 | CV1 | | | | | | | | | | | |
| 6 | 2 | CV2 | CR2 | CR2 | | | | | | | | | |
| 7 | 3 | CV3 | CR3 | CR3 | | | | | | | | | |
| 8 | 4 | CV4 | CR4 | CR4 | | | | | | | | | |
| 9 | 5 | CV5 | CR5 | CR5 | | | | | | | | | |
| 10 | 6 | CV6 | CR6 | CR6 | | | | | | | (M1) | | |
| 11 | 7 | CV7 | CR7 | CR7 | | | C | M1 | | C | M2 | | C |
| 12 | 8 | PSV8 | PSR8 | PSR8 | M1 | | | M2 | | | M3 | | |
| 13 | 9 | DV1 | DR1 | DR1 | M2 | | V1 | M3 | | V1 | M4 | | V1 "1/2 V1" |
| 14 | 10 | DV2 | DR2 | DR2 | M3 | P1 | | M4 | P1 | | M5 | P1 | |
| 15 | 11 | DV3 | DR3 | DR3 | M4 | | V2 | M5 | | V2 | M6 | | V2 "1/2 V1" |
| 16 | 12 | DV4 | DR4 | DR4 | M5 | P2 | | M6 | P2 | | M7 | P2 | |
| 17 | 13 | DV5 | DR5 | DR5 | M6 | | V3 | M7 | | V3 | M8 | | V3 "V2" |
| 18 | 14 | DV6 | DR6 | DR6 | M7 | P3 | | M8 | P3 | | M9 | P3 | |
| 19 | 15 | DV7 | DR7 | DR7 | M8 | | V4 | M9 | | V4 | M10 | | V4 „V3” |
| 20 | 16 | DV8 | DR8 | DR8 | M9 | P4 | | M10 | P4 | | M11 | P4 | |
| 21 | 17 | DV9 | DR9 | DR9 | M10 | | V5 | M11 | | V5 | M12 | | V5 "V4" |
| 22 | 18 | DV10 | DR10 | DR10 | M11 | | | M12 | ? | | M13 | P5 "SM12" | |
| 23 | 19 | SV1 | SR1 | SR1 | M12 | | | M13 | | ? | M14 | | V6 "SC" |
| 24 | 20 | SV2 | SR2 | SR2 | - | | | M14 | | | M15 | ? ?"M17" | |
| 25 | 21 | CaV1 | CaR1 | CaR1 | | | | (M15) | | | M16 | | |
| 26, 27 | 22 | CaV2 | CaR2 | CaR2 | | | | - | | | ?M17 | | |

of the first vertebral (Gaffney 1990) as well as the relationship of that scute to the first pair of pleurals indicate, that it is in fact composed of fused two first vertebrals.

The situation of the posterior carapacial segments is less clear. First of all, the posterior region of the carapace is generally more variable when it comes to the scute layout (Zangerl & Johnson 1957), so it may adhere less strictly to the somitic segmentation (especially, that it is composed mostly of membrane bones, and not axial skeleton derivatives). Secondly, the posterior part of the carapace is derived from sacral somites, and in the Triassic taxa apparently caudal somites, so the regulation of the scute layout might have been different. Thirdly, the Late Triassic turtles have a caudal notch (Szczygielski & Sulej 20106), which may impact the scute arrangement. The presence of an additional, fourteenth marginal scute in *Proterochersis* spp. indicates that one additional posterior segment was present in that turtle. That would fulfill the hypothetical two-somite margin requirement needed for development of another pair of pleurals, but there is no trace of them. Instead, the pleural series is terminated by the lateral wings of the last vertebral. It is currently unknown, whether the extra pleural placodes did not form (for example due to sacral rather than dorsal character of that body region, lack of contact with axial skeleton derivatives or spatial reasons linked to the presence of the caudal notch) or fused early with the last pair of vertebral placodes. In *Prog. quenstedti* the supracaudal scute seems to be obvious extra element belonging to the vertebral series, and the last (twelfth) supramarginal may potentially be treated as a part of the pleural series. If such interpretation (i.e., one extra vertebral on mioseptum 23, one extra pleural on mioseptum 22, and seventeen marginals reaching to mioseptum 26) is true, then another pleural would still fit on mioseptum 24. Its absence may be due to overcrowding of that region with

other scutes. Alternatively, the last marginal may in fact belong to the pleural series. This would violate the two somite margin rule, but with no true marginals present on the posterior edge of the carapace, the spatial constraint does not apply. In that case the posterior configuration of *Prog. quenstedti* carapace can be considered a true caudal notch.

## References


Anquetin J. 2010. The anatomy of the basal turtle *Eileanchelys waldmani* from the Middle Jurassic of the Isle of Skye, Scotland. Earth and Environmental Science Transactions of the Royal Society of Edinburgh 101:67–96.

Anquetin J., Püntener C., Billon-Bruyat J.-P. 2014. A taxonomic review of the Late Jurassic eucryptodiran turtles from the Jura Mountains (Switzerland and France). PeerJ 2:e369.

Bräm H. 1965. Die Schildkröten aus dem oberen Jura (Malm) der Gegend von Solothurn. Schweizerische Paläontologische Abhandlungen 83:1–190.

Cadena E.A., Jaramillo C.A., Bloch J.I. 2013. New material of the platychelyid turtle *Notoemys zapatocaensis* from the Early Cretaceous of Colombia; implications for understanding Pleurodira evolution. In Brinkman D.B. et al. (eds.) Morphology and Evolution of Turtles, 105–119. Springer Science+Business Media Dordrecht.

Cherepanov G.O. 2015. Scute's polymorphism as a source of evolutionary development of the turtle shell. Paleontological Journal 49:1–10.

Gaffney E.S. 1972. The systematics of the North American family Baenidae (Reptilia, Cryptodira). Bulletin of the American Museum of Natural History 147:241–320.

Gaffney E.S. 1990. The comparative osteology of the Triassic turtle *Proganochelys*. Bulletin of the American Museum of Natural History 194:1–263.

Gaffney E.S., Hutchison J.H., Jenkins F.A., Meeker L.J. 1987. Modern turtle origins: the oldest known cryptodire. Science 237:289–291.

Moustakas-Verho J.E., Cherepanov G.O. 2015. The integumental appendages of the turtle shell: an evo-devo perspective. Journal of Experimental Zoology 324B:221–229.

Owen R. 1849-1884. A History of British Fossil Reptiles, vol. IV. Cassell & Company Limited.

Sukhanov V.B. 2006. An archaic turtle, *Heckerochelys romani* gen. et sp. nov. from the Middle Jurassic of Moscow Region, Russia. Fossil Turtle Research 1:112–118.

Szczygielski T., Sulej T. 2016. Revision of the Triassic European turtles *Proterochersis* and *Murrhardtia* (Reptilia, Testudinata, Proterochersidae), with the description of new taxa from Poland and Germany. Zoological Journal of the Linnean Society 177:395–427.

Zangerl R., Johnson R.G. 1957. The nature of shield abnormalities in the turtle shell. Fieldiana Geology 29:341–362.

## VERTEBRAL COLUMN OF *ODONTOCHELYS SEMITESTACEA*

### CERVICAL VERTEBRAE

*Odontochelys semitestacea* is stated to have eight cervical vertebrae (Li et al. 2008), but the cervical column of that reptile was never illustrated well. The description by Li et al. 2008 includes the photographs of the holotype (IVPP V 15639) and paratype (IVPP V 13240) with complete neck, but the detailed morphology is very difficult to discern from these figures and the interpretative drawings skip the cervical region. IVPP V 13240 is exposed only in ventral view and only six vertebrae are visible. The third, fourth and fifth vertebrae all have uniform length of 0,8 cm. Only the anteriormost part of the sixth cervical vertebra can be seen. The cervical vertebral column of the holotype is proportionally longer. Neck (excluding atlas, axis and PSV8) to plastron + hypoischium midlength ratio indicates, that in holotype one additional cervical vertebra sticks out from above the plastron:

$$\frac{\text{Length of visible cervical vertebrae (excluding CV1, CV2, and PSV8) in IVPP V 13240}}{\text{Plastron + hypoischium length in IVPP V 13240}} = \frac{2.4\text{ cm}}{22.2\text{ cm}} = 0.11$$

$$\frac{\text{Length of visible cervical vertebrae (excluding CV1, CV2, and PSV8) in IVPP V 15639}}{\text{Plastron + hypoischium length in IVPP V 15639}} = \frac{3.3\text{ cm}}{19.2\text{ cm}} = 0.17$$

If 11% of the midlength of the plastron + hypoischium corresponds to 3 visible cervical vertebrae, then 17% corresponds to 4.6 visible vertebrae (assuming uniform average length of cervical vertebrae). Factoring in the atlas and axis and the remaining part of the last cervical vertebra that overlaps with anterior plastral lobe, this adds up to the total of 7 cervical vertebrae.

### CERVICAL-DORSAL FRONTIER

The identity of PSV8 in *O. semitestacea* remains uncertain. That vertebra was considered the eighth cervical vertebra by Li et al. 2008, but it is unsure, if the reason was its sequence of morphology. All of the known specimens of *O. semitestacea* lack complete neural series, and it is not even sure whether each of the dorsal vertebrae formed its own neural. No ribs were described or pictured for that vertebra, but if they did not form costal plates, they may easily be obscured by dorsal epiplastra processes or the following ribs, or lost (note, that the first pair of dorsal rib in turtles is always tucked mediocaudally behind the scapulae and dorsal epiplastral processes, if present). The best criterion in that case is the nature of contact between PSV8 and the following dorsal vertebra. PSV8 in the holotype of *O. semitestacea* is not displaced and forms a continuous row with the following DVs, even though the cervical, sacral and caudal vertebrae and ribs exhibit at least slight disturbance (Supp. Fig. 1a). Even the disarticulated or broken segments of the dorsal vertebral column are slightly relocated in relation to each other. This suggests that PSV8 is sutured or fused to DV1 and thus should be treated as the first dorsal rather than the last cervical vertebra.

### DORSAL VERTEBRAE

*O. semitestacea* was described and illustrated as having nine dorsal vertebrae and ten pairs of dorsal ribs (Li et al. 2008). In all of the described specimens the ribs are mostly disarticulated and some of them are often missing, even despite the overall completeness and articulation of the other skeletal elements. The vertebral column of the holotype presents rather unusual morphology (Supp. Fig. 1a). The five anteriormost and the two posteriormost elements interpreted by Li et al. 2008 as dorsal vertebrae are relatively short. The sixth and seventh vertebrae, on the other hand, are about two times longer than the rest. This is unexpected and, at least to my knowledge, no turtle exhibits such rapid and substantial anteroposterior variation in the dorsal vertebrae morphology. Such conspicuous length of the sixth and seventh dorsal vertebrae might be interpreted as the result of the compensation for the loss of one of the vertebrae (possibly the one lying between them), because the vertebral column itself does not appear to be proportionally shorter than in other Late Triassic turtles, and if all of the dorsal vertebrae had a uniform length similar to that of vertebrae 1-5 and 8-9, there would be enough space to fit at least one additional vertebral element. A closer inspection of the vertebrae (Hirayama 2009; Li et al. 2008, fig. 1a; Zhou 2010, fig. 2a), however, reveals that the limits of the vertebrae are not readily visible due at least partial co-ossification. The slight isthmuses on the dorsolateral surfaces of the purported vertebrae sixth and seventh may indicate that actually at least one or even two additional

elements are present and that the boundaries between the vertebrae presented by Li et al. 2008 may potentially represent breaks in the co-ossified vertebral column. This observation is reinforced by both IVPP V 13240 (paratype) and IVPP V 15653.

IVPP V 15653 (Supp. Fig. 1c-f) consists of a pectoral and partial pubic girdle, several caudal vertebrae, partial plastron and partially articulated dorsal vertebral column with disarticulated ribs. Lyson et al. 2013 pictured part of the specimen in their supplementary material (fig. S4 therein). There are five articulated vertebrae present and in front of them another one, disarticulated and lying on its side. There are also at least eleven disarticulated or rotated ribs present, and the posteriormost left rib, although rotated, still maintains a contact with the socket on the corresponding vertebra. On the same slab, just posterior to the articulated segment (not visible in the fig. S4 of Lyson et al. 2013) there is another, partially prepared element that resembles a vertebra (Supp. Fig. 1c-d). Approximately at the same level, but more laterally, there is yet another unambiguous dorsal vertebra and three disarticulated ribs. One of them is large, broad, and presents morphology typical for the dorsal ribs of *O. semitestacea*. Two remaining ribs are shorter (but still more than 1,5 times the length of the vertebra) and only slightly broadened. Their morphology is closer to the dorsal ribs than to the caudal transverse processes because of their length and width slightly increasing distally. This most likely is the last, slender pair of dorsal ribs mentioned by Li et al. 2008 (p. 497) and apparently ignored or overlooked by Lyson et al. 2013. Therefore, the rib interpreted by Lyson et al. 2013 as the ninth is not the last one, but the second to last or (if the ambiguous element lying just posterior to it is indeed a vertebra) third to last. All the vertebrae are of approximately equal length: 1.9 cm, 2 cm, 1.9 cm, 1.9 cm, 1.8 cm, 1.6 cm, 1.8 cm (ambiguous element), and 1.9 cm. This adds up to 14.8 cm (including the ambiguous element due to its uncertain identity) or 13 cm (excluding it) with average of 1.86 cm (excluding the ambiguous element), or 1,85 cm (including that element), proving that the abnormally elongated vertebrae VI and VII are in fact misinterpreted. Assuming uniform average length of dorsal vertebrae, nine DVs (excluding PSV8) in that specimen would be about 16.7 cm long and 10 would be 18.5 cm or 18.6 cm long. The bridge region length (measured as the shortest distance between the axillary and the inguinal notch) of the associated plastron is 7.8 cm, so the length ration of the dorsal vertebral column (excluding PSV8) to the bridge would be 2.1 for nine DVs and 2.37 or 2.38 for ten DVs. In the holotype, the dorsal vertebral column measures approximately 12.2 cm and the bridge is 5 cm long, so the resulting ratio is 2.4, which indicates that ten (or eleven, counting PSV8) dorsal vertebrae were present. If anteriormost dorsal vertebrae of *O. semitestacea* were indeed noticeably shorter than posterior ones, as it is shown by Li et al. 2008, then probably one extra vertebrae would be needed to meet this ratio.

The paratype specimen of *O. semitestacea* (IVPP V 13240) corroborates the above observations. On the left side of that specimen there are three costals visible just posterior to the bridge (Supp. Fig. 1b). They are evenly spaced and the position of the anteriormost costal suggests a point of articulation more posterior than it would appear from the fig. 1d of Li et al. 2008. This may indicate that an additional vertebra was present between the vertebrae sixth and seventh of Li et al. 2008, allowing the more posterior articulation of the first of the three visible ribs (thus solving the problem of the abnormal length of the neighboring vertebrae), or that the whole dorsal vertebral column of the paratype is pushed more caudally than in holotype (which may be suggested by the shortening of the visible segment of the neck). In any case, behind the tree visible ribs of the paratype yet another dorsal vertebra was present, that bore the shorter and slender ribs visible on the last vertebra of IVPP V 15653 (here obscured by the xiphiplastra) and covered the distance to the sacral region.

The presence of more than nine dorsal vertebrae in *Odontochelys semitestacea* seems plausible, given not only the above observations of uniform length of the posterior dorsal vertebra in that animal, the position of the posterior ribs in the paratype and the vertebral column to bridge length ratio. An indirect evidence may be found in the recently published reconstruction of *O. semitestacea* based on all the available material (Lyson et al. 2013). Its authors, who had the opportunity to study the holotype, identified nine (broadened) rib pairs even though they never mention the slender pair. The artificial shortening of the vertebral column by accidental exclusion of vertebrae led them to the erroneous restoration, in which the pelvis is located more anteriorly than previously shown, and only the hypoischium sticks out from above the plastron. That makes the silhouette of the animal more similar to that of *Eunotosaurus africanus*, but does not agree with the fossil data. Both of the articulated specimens of *O. semitestacea* published to

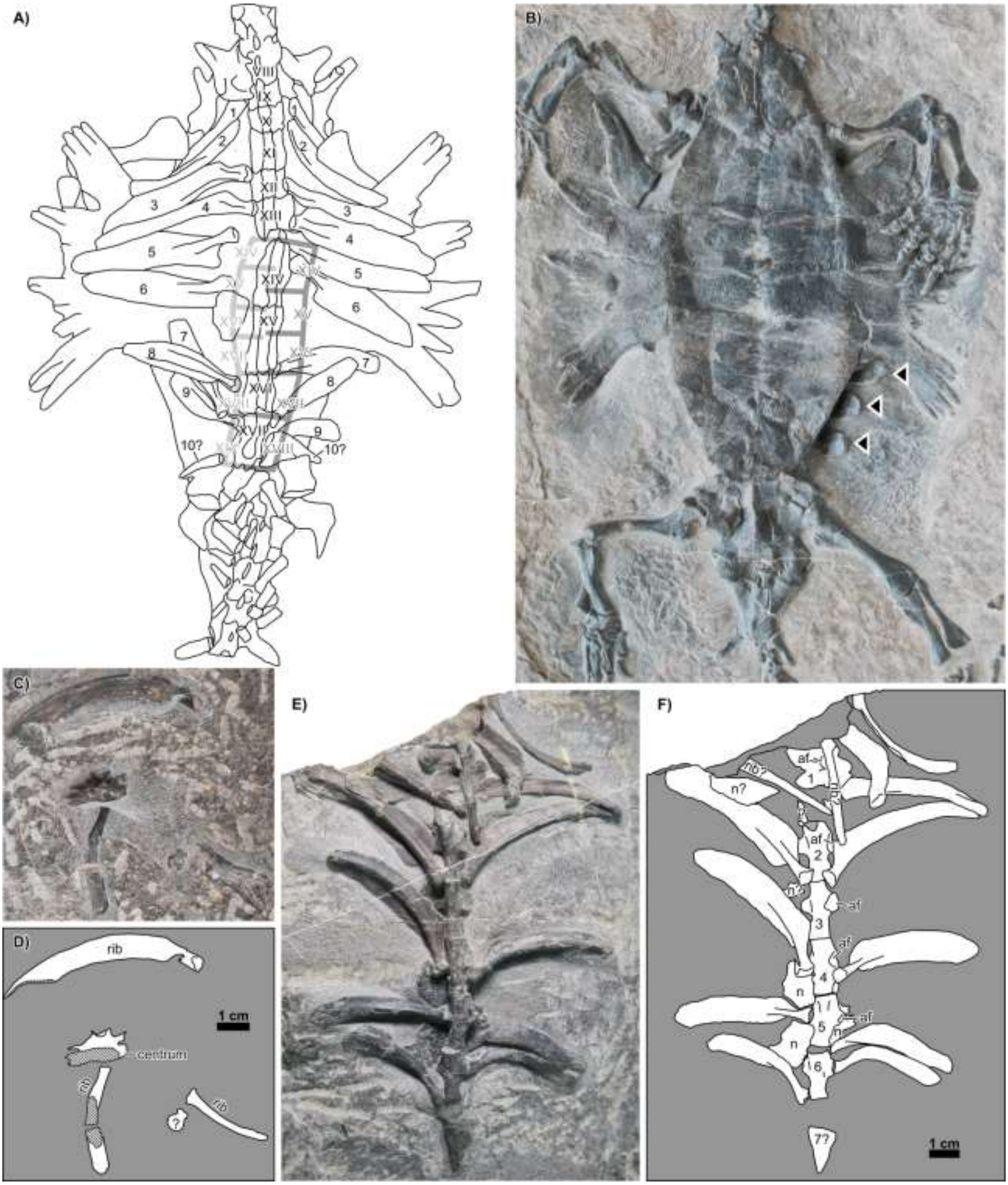


**Supplementary Figure 1.** Vertebrae and ribs of *Odontochelys semitestacea*. **A:** The holotype with abnormally elongated sixth and seventh dorsal vertebra. Dark grey are putative vertebrae limits assuming their roughly uniform length, light grey are putative vertebrae limits suggested by the isthmuses on the vertebral column. Roman numerals indicate the number of vertebra. The visible ribs are counted using arabic numerals, note that it does not necessarily correspond to their number in sequence. **B:** IVPP V 13240 with three left ribs visible in ventral view. **C, D:** IVPP V 15653, disarticulated dorsal vertebra with associated slender ribs. **E, F:** IVPP V 15653, the partially articulated set of six vertebrae and an ambiguous element, possibly the seventh vertebra. Abbreviations: af, articular facet; n, neural bone.

date have their pelves mostly posterior to the caudal end of the plastron (Li et al. 2008), and similar position of pelvis in relation to plastron is also visible in *Proganochelys quenstedti* (see Gaffney 1990). In *Palaeochersis talampayensis*

the posterior lobe of plastron is longer and mostly covers the pelvis ventrally, but the position of pelvis in relation to the bridge region and the carapace is the same (Serli et al. 2007), and such is the case also in proterochersids (Szczygielski & Sulej 2016).

### VERTEBRAL REDUCTION IN TURTLE EVOLUTION

Turtles exhibit an ongoing trend towards simplification of the shell structure and lowering the number of its elements (Zangerl 1969), and the ribs are a clear subject to that. First of all, a great reduction of the initial for amniotes, high rib count (Romer 1976; Müller et al 2010) was needed to reach the number present in earliest turtles. This, most likely, was a long, consistent process, as the rapid changes in the length and segmentation of the trunk would most likely lead to severe internal complications and, as a result, would be fatal or highly detrimental. Secondly, there is a good fossil record of the size reduction of the first and the tenth dorsal rib pair after the shell acquisition.

A high vertebral count, which is ancestral to amniotes and still very common in reptiles (Romer 1976; Müller et al 2010), is beneficial during locomotion. The high number of intervertebral joints increases the lateral flexibility of the body, lengthening the stride and boosting the walking/running effectiveness as a result. Similar movements were also utilized by numerous aquatic reptiles during swimming (Carroll 1985). The flexibility of the body, however, may be reduced in some evolutionary scenarios. Examples include the appearance of a well-developed dermal armor or broadening of the ribs as means of buoyancy control or as a defense. These adaptations reduce the flexibility of the trunk as a result of the mechanical constraints or weakening of the dorsal muscles, when the space available to them is decreased. This in some situations increases the survivability of the animal by allowing it to avoid the attacks of the carnivores or by helping it to dive in search of prey or while running away. At the same time, it disadvantages the movement of the animal, requiring changes to the mode of locomotion, and increasing the energetic cost of keeping the body raised due to its higher mass, as more muscle work is needed to keep the vertebral column, encumbered by heavier ribs or dermal ossifications, stiff. One of the possible solutions is to reduce the number of the vertebrae – the shorter the trunk and the lower the number of joints, the easier it is to keep it in position, and the resulting loss of the axial flexibility is negligible, because it is already constrained. This opens a way for a further development of dorsal armor or broadening of the ribs, because the stiffer axial skeleton may carry more, thus creating a loop of a correlated progression. Such a process might have taken place in turtle ancestors and pareiasaurs (Lee 1996). The broadening of the ribs may be linked to the consecutive lengthening of the vertebrae and reduction of the intercostal muscles, just due to shortage of available space. This loop of flexibility reduction may occur both in the terrestrial and aquatic animals, but in the aquatic setting it may be facilitated by the propulsion problems. As a rule, amniotes during locomotion move their limbs asymmetrically (Carroll 1985). This is also true for aquatic turtles with the exception of the most advanced marine forms belonging to Chelonioidea (Zangerl 1953). It poses no special problem for most swimmers, that use their tails or whole body for propulsion or to compensate for the undesirable effects (Carroll 1985). The earliest aquatic turtles, however, such as *Pa. rosinae* or *O. semitestacea*, were devoid of strong tails and had very limited body flexibility, but still not completely stiffened trunks. Even though in *O. semitestacea* there was a complete plastron that limited the possible flexure of the body, it still had no rigid connection with the axial skeleton, and the scapulocoracoids were supported only ventrally. Thus, the asymmetric movements of limbs probably created undesired stress and tensions on the vertebral column, which lowered the effectiveness of propulsion and required additional muscle work, increasing the energetic costs, to keep the axial skeleton stiff. It is very unlikely then that in such setting the vertebral number would increase from the low count of nine present in *E. africanus*, had it been inherited by turtle ancestors.

Although the dorsal vertebral centra of *Odontochelys semitestacea* are very slender, at the points of rib attachment they are much wider, therefore, similarly to *Pappochelys* rosinae (Schoch & Sues 2015), in *O. semitestacea* the vertebrae as a whole are nowhere near as profoundly elongated, as it is the case in *Eunotosaurus* africanus (Seeley 1892; Watson 1914; Lyson et al. 2013) or derived turtles. In that aspect, *E. africanus* is just more advanced than the oldest unambiguous stem turtles, which is confirmed by the phylogenetic analysis (see below). I suspect that the controversy around the dorsal count of fossil stem-testudinates may result from the assumption that all of their dorsal ribs should be broadened, but this is not the case for modern nor fossil turtles.

## References


Carroll R.T. 1985. Evolutionary constraints in aquatic diapsid reptiles. Palaeontology 33:145–155.

Gaffney E.S. 1990. The comparative osteology of the Triassic turtle *Proganochelys*. Bulletin of the American Museum of Natural History 194:1–263.

Hirayama R. 2009. Oldest known turtle *Odontochelys* [in Japanese]. O Kenshō Suru, Iden 9:2–4.

Lee M.S.Y. 1996. Correlated progression and the origin of turtles. Nature 379:812–815.

Li C., Wu X.-C., Rieppel O., Wang L.-T., Zhao L.-J. 2008. An ancestral turtle from the Late Triassic of southwestern China. Nature 456:497–501.

Lyson T.R., Bever G.S., Scheyer T.M., Hsiang A.Y., Gauthier J.A. 2013. Evolutionary origin of the turtle shell. Current Biology 23:1113–1119.

Müller J., Scheyer T.M., Head J.J., Barrett P.M., Werneburg I., Ericson P.G.P., Pol D., Sánchez-Villagra M.R. 2010. Homeotic effects, somitogenesis and the evolution of vertebral numbers in recent and fossil amniotes. PNAS 107:2118–2123.

Romer A.S. 1976. Osteology of the Reptiles. The University Chicago Press.

Schoch R., Sues H.-D. 2015. A Middle Triassic stem-turtle and the evolution of the turtle body plan. Nature doi:10.1038/nature14472.

Seeley H.G. 1892. On a new reptile from Welte Vreden (Beaufort West), *Eunotosaurus africanus* (Seeley). Quaternary Journal of the Geological Society of London 48:583–585

Sterli J., de la Fuente M.S., Rougier G.W. 2007. Anatomy and relationships of *Palaeochersis talampayensis*, a Late Triassic turtle from Argentina. Palaeontographica A 281:1–61.

Szczygielski T., Sulej T. 2016. Revision of the Triassic European turtles *Proterochersis* and *Murrhardtia* (Reptilia, Testudinata, Proterochersidae), with the description of new taxa from Poland and Germany. Zoological Journal of the Linnean Society 177:395–427.

Watson D.M.S. 1914. *Eunotosaurus africanus* Seeley, and the ancestry of the Chelonia. Proceedings of the Zoological Society of London 84:1011–1020.

Zangerl R. 1953. The vertebrate fauna of the Selma Formation of Alabama. Part IV. The turtles of the family Toxochelyidae. Fieldiana 3:135–277.

Zangerl R. 1969. The turtle shell. In Gans C. (ed.) Biology of the Reptilia. Academic Press.

Zhou Z. 2010. On some examples of missing links – perspectives from fossil evidence in China. In Long M., Gu H., Zhou Z. (eds.) Darwin's Heritage Today: Proceedings of the Darwin 200 Beijing International Conference, 326–336. Higher Education Press.

## CHARACTER LIST

New characters and states are indicated in bold.

0. Skull proportions
0: preorbital skull length equal to postorbital length
1: preorbital length exceeds postorbital skull length
2: postorbital length exceeds preorbital skull length

1. Snout, shape
0: unconstricted
1: constricted

2. Premaxilla, size
0: small
1: large, forming most of snout in front of external nares

3. Premaxilla, shape
0: horizontal ventral margin
1: down-turned ventral margin

4. Premaxilla, postnarial process
0: absent
1: present, restricting contact of the maxilla to the external nares or even excluding maxilla

5. External nares, location I
0: marginal
1: close to midline

6. External nares, location II
0: positioned anteriorly
1: situated in central or posterior area of the antorbital skull portion

7. External nares, internarial bar
0: separated by internarial bar of premaxilla
1: confluent

8. External nares, shape
0: rounded
1: elongate

9. Premaxilla, contribution to internal naris
0: present
1: absent, excluded

10. Premaxilla-maxilla suture
0: simple vertical or diagonal
1: notch present in maxilla

11. Premaxilla, dentition
0: present
1: absent

12. Premaxillary/maxillary teeth, separated by diastema
0: absent
1: present

13. Anterior (premaxillary/maxillary) teeth, orientation
0: upright
1: strongly procumbent

14. Premaxilla-frontal contact
0: absent, nasals meet in dorsomedial suture
1: present, nasals separated

15. Septomaxilla
0: present
1: absent

16. Nasals
0: present
1: absent

17. Nasals, fusion
0: paired
1: fused

18. Narial shelf
0: absent
1: present

19. Nasal/frontal ratio
0: nasal equal to or shorter than frontal
1: nasal at least one-third longer, or better

20. Maxilla, ascending process
0: absent
1: present between orbit and external nares

21. Maxilla-prefrontal contact, anterior to lacrimal
0: absent due to nasal-lacrimal contact
1: present, separating nasal and lacrimal

22. Maxilla-frontal contact
0: absent
1: present, separates prefrontal and nasal

23. Maxilla length
0: extends to posterior orbital margin
1: does not reach posterior margin of orbit

24. Maxilla, orbital exposure
0: absent
1: present

25. Maxilla-quadratojugal contact
0: absent
1: present

26. Maxilla-vomer contact
0: absent
1: present

27. Maxilla-ectopterygoid contact
0: present
1: absent

28. Maxilla-pterygoid contact
0: absent
1: present

29. Maxilla, anterolateral foramen
0: absent or if present equal in size to all other foramina
1: present at least twice the diameter of all other foramina

30. Maxilla, tooth row
0: restricted to level in front of the posterior margin of the orbit
1: extends behind posterior margin of orbit

31. Teeth, button-like durophagous
0: absent
1: present

32. Antorbital fenestra
0: absent
1: present

33. Lacrimal, morphology
0: present and contributing to external nares
1: present at least as long as tall, but excluded from external nares
2: if present small, restricted to orbital margin, or absent entirely

34. Lacrimal, orbital margin
0: lacrimal enters orbital margin
1: excluded from margin due to external contact between posteroventral part of prefrontal and posterodorsal margin of maxilla

35. Lacrimal duct
0: enclosed by lacrimal only
1: lateral border formed by maxilla

36. Lacrimal, suture with jugal
0: present
1: absent

37. Prefrontal, bulbous medial process
0: absent
1: present

38. Prefrontal, contact with palate
0: absent
1: present

39. Prefrontal, medial flange
0: narrow
1: wide

40. Foramen orbitonasale
0: absent
1: represented by a medial indentation on the lacrimal and a dorsal indentation on the palatine
2: enclosed between prefrontal, lacrimal and palatine

41. Frontal, overall shape
0: parallelogram
1: hour-glass

42. Frontal, proportions
0: length exceeds width by at least four times
1: length no greater than twice width

43. Frontal-parietal length ratio
0: >1
1: < or =1

44. Frontal-nasal suture
0: transverse
1: oblique, forming an angle of at least 30° with long axis of the skull

45. Frontals, fusion
0: absent, paired
1: present, fused

46. Frontal, contribution to orbit
0: absent, due to prefrontal-postfrontal contact along orbital margin
1: present

47. Frontal, lateral lappet
0: absent
1: present

48. Frontal, posterolateral processes
0: absent
1: present

49. Orbit, shape
0: generally circular
1: anteroposteriorly elongate so that the length exceeds the height by at least 30%

50. Dermal sculpturing
0: absent
1: present

51. Dermal sculpturing, morphology
0: tuberosities
1: tuberosities and pits
2: honeycomb pattern of ridges and pits

52. Circumorbital bumps
0: absent
1: present

53. Postfrontal
0: present
1: absent, probably through fusion with postorbital

54. Intertemporal
0: present
1: absent

55. Postorbital-parietal contact
0: absent
1: present

56. Postorbital, posterior extent
0: terminates prior to reaching posterior limit of parietal
1: reaches nearly the posterior extent of the skull

57. Postorbital, shape of posterior process
0: slender, half as wide as itis long
1: increased width, parallelogram outline in lateral aspect

58. Supratemporal
0: present
1: absent

59. Supratemporal, size
0: large
1: small

60. Postorbital-supratemporal contact
0: present
1: absent

61. Parietal, skull table proportions
0: broad, width not less than half of the length measured along the element's midline
1: weakly constricted
2: strongly constricted with the length exceeding the width by at least three times
3: forming sagittal crest

62. Parietal, shelf for adductor musculature
0: absent
1: present as shallow excavations on the lateral margins of the parietal

63. Parietal-prootic contact
0: absent
1: present

64. Parietal-opisthotic contact
0: absent
1: present

65. Pineal foramen
0: present
1: absent

66. Pineal foramen, position
0: present, displaced anteriorly
1: present, located near the middle of the parietal body
2: present, displaced posteriorly

67. Parietal-squamosal contact
0: absent
1: present

68. Parietal, fusion
0: absent
1: fully or partially fused in adult

69. Postparietal
0: present
1: absent

70. Postparietal, fusion
0: paired
1: fused

71. Postparietal, position
0: dorsally exposed, integrated into skull table
1: restricted to occipital plate

72. Posterolateral skull roof, composition
0: formed by tabular
1: formed mostly by supratemporal
2: formed by parietal and small supratemporal or parietal along

73. Tabular
0: present
1: absent

74. Tabular, position
0: part of skull table
1: restricted to occiput

75. Jugal, anterior process
0: does not extend to anterior orbital rim but reaches posterior margin of orbit
1: extends at least to level of anterior orbital rim
2: fails to reach posterior margin of orbit

76. Jugal, subtemporal process
0: present
1: absent

77. Jugal, subtemporal process extent
0: extends posteriorly no further than the middle of the cheek
1: reaches nearly the posterior limit of the skull

78. Jugal, subtemporal process morphology
0: robust, with height >50% of length
1: slender, height <50% of length

79. Squamosal, ventral extent
0: descends to ventral margin of skull
1: reaches only the approximate midlevel of the lower

temporal fossa
2: remains distinctly restricted to the dorsal region of the cheek

80. Squamosal, contribution to post-temporal fenestra
0: absent, elements separated
1: present

81. Squamosal, occipital flange
0: absent or poorly developed forming only a thin ridge
1: flange developed forming a broadly exposed lappet

82. Quadratojugal
0: present
1: absent

83. Quadratojugal, anterior process
0: present
1: absent

84. Quadratojugal, vertical process
0: absent, restricted to ventral margin of cheek
1: present, distinct dorsal extension that does not extend above the dorsoventral midline of the orbit
2: present, process extends to a point above the dorsoventral midline of the orbit

85. Quadratojugal, anterior extent
0: reaches level of posterior border of orbit
1: does not reach level of posterior border of orbit

86. Quadratojugal, ornamentation
0: absent
1: present

87. Postorbital region, ventrolateral expansion
0: rectilinear or emarginated
1: expanded below level of maxilla

88. Jugal-squamosal contact
0: present
1: absent

89. Upper temporal fenestra
0: absent
1: present

90. Upper temporal fenestra, size
0: comparable to orbit size or smaller
1: distinctly larger than orbit

91. Upper temporal fenestra, contribution from frontal
0: frontal widely separated from upper temporal fenestra
1: narrowly approaches or enters the anteromedial margin of the upper temporal fossa

92. Upper temporal fenestra, contribution from postfrontal
0: absent due to postorbital-parietal contact
1: present

93. Lower temporal fenestra
0: absent
1: present

94. Lower temporal fenestra, ventral margin
0: bounded ventrally by bone
1: open ventrally, subtemporal processes of jugal and quadratojugal not in contact

95. Lower temporal fenestra, postorbital contribution
0: present
1: absent

96. Lower temporal fenestra, quadratojugal contribution
0: absent
1: present

97. Post-temporal fenestra
0: absent
1: present

98. Post-temporal fenestra
0: small, diameter less than half of the diameter of the foramen magnum
1: large, diameter at least equal to that of the foramen magnum

99. Interpterygoid vacuity, presence
0: vacuity absent
1: present

100. Interpterygoid vacuity, anterior extent
0: extends beyond posterior margin of palatine
1: does not reach posterior margin of palatine

101. Interpterygoid vacuity, shape
0: anterior end tapers sharply
1: anterior border crescent-shaped

102. Pterygoid, palatal process
0: extends anterior to the anterior limit of the palatine
1: forms oblique suture with palatine but process ends before reaching anterior limit of palatine
2: forms transverse suture with palatine

103. Pterygoid, anterior extent
0: reaches level of posterior end of choana
1: remains posterior to choana

104. Pterygoid, teeth on palatine ramus
0: present
1: absent

105. Pterygoid, transverse flange development
0: well-developed
1: strongly reduced

106. Pterygoid, orientation of transverse flange
0: directed predominantly laterally
1: oriented in an anterolateral direction

107. Pterygoid, dentition on transverse flange
0: present
1: edentulous

108. Pterygoid, tooth morphology on the transverse flange
0: present as a shagreen of teeth
1: present but with one large distinct row of teeth along the posterior edge of the transverse flange

109. Pterygoid, ventral extent of transverse flange
0: extends below maxillary tooth row
1: terminates at or above maxillary tooth row

110. Pterygoid, quadrate ramus
0: merges smoothly into transverse flange without distinctive excavation
1: deep excavation on posterolateral surface

111. Ptergyoid, aruate flange
0: present
1: absent

112. Basipterygoid processes, orientation
0: lateral
1: anterolateral

113. Palate, basicranial articulation
0: kinetic
1: akinetic

114. Basicranial articulation, length
0: restricted to lateral margin of the parasphenoid
1: extends over much of the length of the main body of the parasphenoid

115. Ectopterygoid
0: present
1: absent

116. Ectopterygoid, teeth
0: present
1: absent

117. Ectopterygoid-pterygoid suture
0: transverse process extends along posterior margin of ectopterygoid and reaches its posterolateral corner
1: extends along posterior margin but does not reach its posterolateral corner
2: contact restricted to medial surface of ectopterygoid

118. Choana, palatal exposure
0: parallel medial border of maxilla, palatine forms posterior margin only
1: deflected posteromedially, palatine forms posterior and part of its lateral margin

119. Suborbital foramen
0: absent
1: present

120. Suborbital foramen, size
0: small
1: enlarged as fenestra

121. Suborbital foramen, lateral border
0: formed by dermal cheek—maxilla and/or jugal
1: dermal cheek excluded by palatine contact with transverse process of pterygoid or ectopterygoid

122. Palatine, teeth
0: present
1: absent

123. Vomer, teeth
0: edentulous
1: present

124. Vomer, distribution of teeth
0: extend along a majority of the vomer’s rostrocaudal length, not restricted to anterior end
1: restricted to the rostral end of the vomer

125. Vomer, alar flange
0: absent
1: present, mediolateral expansion positioned just behind contact with premaxilla

126. Quadrate, posterior excavation
0: straight posterior edge
1: posterior edge deeply excavated forming a concave region

127. Quadrate, lateral exposure
0: absent, covered by squamosal and quadratojugal
1: present

128. Quadrate, lateral conch
0: absent
1: present

129. Quadrate, anterior process
0: long, extending forward along its sutural contact with the quadrate process of the pterygoid to nearly reach the level of the transverse flange
1: short, not extending anteriorly beyond half the length of the quadrate process of the pterygoid

130. Quadrate-prootic contact
0: absent
1: present

131. Quadrate, articular surface of condyle
0: strongly convex, anteroposteriorly long
1: nearly flat, anteroposteriorly shorter than they are wide

132. Temporal notch
0: absent
1: present

133. Temporal depression
0: absent
1: present

134. Stapes, shape
0: robust, greatest depth exceeding one-third total length
1: slender, length at least four times depth
2: slender but short

135. Stapes, dorsal process
0: ossified
1: unossified or absent

136. Epipterygoid, dorsal wing
0: broad
1: narrow

137. Cranioquadrate space
0: small, quadrate ramus of pterygoid and paroccipital process of opisthotic converge posterolaterally
1: large, quadrate ramus of pterygoid and paroccipital process of opisthotic are parallel to each other

138. Supraoccipital, occipital crest
0: plate-like, no sagittal crest
1: body constricted at midline forming sagittal crest

139. Medial wall of inner ear as formed by the prootic
0: unossified
1: ossified with acoustic nerve foramina

140. Paroccipital process, orientation
0: directed primarily laterally, approximately perpendicular to the parasagittal plane
1: paroccipital process deflected posterolaterally at an angle of about 20° from the transverse width of the skull
2: paroccipital process deflected dorsolaterally at an angle of nearly 45°

141. Paroccipital process, distal end
0: ends freely or contacts skull table
1: contacts suspensorium, including quadrate, squamosal, and/or supratemporal on the occiput

142. Exoccipitals, contact each other below foramen magnum
0: present
1: absent

143. Exoccipital, lateral process
0: absent
1: present, lateral flange extending deep to paroccipital process

144. Occipital condyle, shape
0: transversely broad
1: reniform to circular

145. Basioccipital/basisphenoid relationship
0: elements separated by unossified gap
1: elements with ossified suture

146. Parasphenoid, pocket for cervical musculature
0: present
1: absent

147. Parasphenoid, cultriform process
0: long, exceeding length of parasphenoid body and reaching forward to the level of the posterior limit of the internal nares
1: short, not reaching the level of the internal nares
2: absent

148. Parasphenoid, teeth
0: present
1: absent

149. Parasphenoid wings
0: present, parasphenoid broad posteriorly
1: absent, parasphenoid narrow posteriorly

150. Ventral braincase tubera
0: absent
1: present

151. Sphenethmoid
0: present
1: absent

152. “Laterosphenoid”
0: absent
1: present

153. “Laterosphenoid”, extent
0: not in contact with parietal
1: forms osseous bridge between basisphenoid and dermal roof

154. Mandibular joint, position relative to occipital condyle
0: posterior to
1: even with
2: anterior to

155. Coronoid process
0: absent or small and formed by multiple elements
1: tall, formed by coronoid
2: tall, formed by dentary

156. Coronoid, number
0: more than one
1: only one coronoid

157. Meckelian fossa, orientation
0: faces mediodorsally, prearticular narrow
1: faces dorsally, prearticular broad

158. Meckelian fossa, length
0: long, occupies at least 25% of lower jaw length
1: short, occupies less than 25% of lower jaw length

159. Surangular, length
0: extends anterior to coronoid eminence
1: does not extend anterior to coronoid eminence

160. Surangular, lateral shelf
0: absent
1: present

161. Surangular, contribution to articular facet
0: absent
1: present

162. Splenial
0: present
1: absent

163. Splenial, mandibular symphysis
0: contributes to mandibular symphysis
1: reduced, excluded from mandibular symphysis

164. Mandibular symphysis, length
0: short
1: elongated

165. Angular, lateral exposure
0: exposed along 1/3 the lateral face of the mandible
1: exposed only as a small sliver along the lateral face
2: absent from lateral aspect

166. Prearticular, extent
0: extends anterior to coronoid eminence
1: terminates prior to reaching coronoid eminence

167. Retroarticular process, morphology
0: absent, small or narrow
1: transversely broad, dorsally concave

168. Retroarticular process, upturned
0: absent
1: present

169. Caniniform region
0: present
1: absent

170. Single caniniform tooth
0: absent
1: present

171. Tooth implantation
0: subthecodont
1: thecodont
2: ankylothecodont
3: superfically attached to bone

172. Marginal dentition, morphology
0: single cusp
1: multiple cusps
2: leaf-shaped with denticles

173. Labryrinthodont infolding
0: present
1: absent

174. Presacral vertebral count
0: more than 20
1: 20 or fewer

175. Caudal vertebral count
0: **23 or more**
**1:** 20 or fewer

176. Vertebral centra
0: notochordal
1: non-notochordal

177. Vertebral centra
0: amphicoelous
1: platycoelous
2: other

178. Accessory vertebral articulations
0: absent
1: present

179. Atlantal ribs
0: ossified
1: not ossified

180. Cervical centra
0: rounded and smooth ventrally
1: keeled ventrally

181. Cervical intercentra
0: present
1: absent

182. Cervical ribs
0: without anterior process
1: with anterior process

183. Trunk neural arches
0: swollen with heavy zygapophyseal buttress
1: narrow, strongly excavated without heavy buttress
2: swollen with narrow, tall zygapophyseal buttress

184. Dorsal intercentra
0: present
1: absent

185. Dorsal transverse processes
0: short
1: long

186. Number of sacrals
0: two
1: three or more

187. Caudal lateral projections (transverse processes)
0: absent beyond fifth caudal
1: present beyond fifth caudal

188. Caudal ribs (transverse processes)
0: L-shaped and curved
1: straight

189. Chevron position
0: intercentral
1: located on anterior pedicel

190. Cleithrum
0: present
1: absent

191. Clavicles
0: broad
1: slender

192. Interclavicle
0: rhomboidal
1: T-shaped with broad transverse bar
2: T-shaped with gracile transverse bar

193. Mineralized sternum
0: absent
1: present

194. Scapula
0: short and broad with height not exceeding width (measured at level of glenoid) by more than three times
1: tall and blade-like with height exceeding width by at least factor of four
2: tall and slender, nearly cylindrical in cross-section

195. Acromion
0: absent
1: present and blade-like
2: present and pointed cylindrical

196. Supraglenoid buttress
0: present
1: absent

197. Coracoid ossifications
0: one
1: two

198. Coracoid foramen
0: enclosed by coracoid only
1: between coracoid and scapula

199. Humerus
0: with large epicondyles
1: with reduced epicondyles

200. Humeral torsion:

0. proximal and distal ends at 45° twist

1. opposing ends at less than 20° twist

201. Humeral shaft-distal end ratio
0: shaft length less than one third maximum width of distal end of humerus
1: shaft long at least four times width of distal end

202. Humeral distal articulation
0: with distinct trochlea and capitellum
1: low double condyle

203. Supinator process
0: large and angled from shaft
1: large and confluent with shaft
2: small or absent

204. Ectepicondylar groove
0: open, notched anteriorly
1: open without notch
2: completely enclosed foramen without groove

205. Entepicondylar foramen
0: present
1: absent

206. Radius length
0: shorter than ulna
1: longer than ulna
2: radius and ulna equal in length

207. Olecranon process
0: large, well developed
1: small or absent

208. Perforating foramen in manus
0: present
1: absent

209. Ratio of metacarpals IV/III
0: metacarpal IV larger than metacarpal III
1: metacarpal IV equal or smaller than metacarpal III

210. Thyroid fenestra
0: absent
1: present

211. Iliac blade, posterior process
0: present
1: fan-shaped distally

212. Iliac blade
0: not expanded anteriorly
1: expanded anteriorly

213. Pubic tubercle
0: small and anteroventrally directed
1: large and downturned in lateral view

214. Acetabulum
0: oval
1: circular

215. Acetabular buttress
0: weak
1: triangular process

216. Femoral shaft
0: stout and straight
1: sigmoidally curved, slender

217. Fourth trochanter of femur
0: present
1: absent

218. Trochanter major of femur
0: absent
1: present, deflected distally from proximal head of femur
2: pyramidal process high on femoral shaft
3: pyramidal process at mid-length on femoral shaft

219. Intertrochanteric fossa
0: well-defined
1: reduced
2: absent

220. Distal femoral condyles
0: prominent, projecting from distal end of shaft
1: not projecting beyond distal end of femur

221. Anterior femoral condyle size
0: larger than posterior condyle
1: smaller than or equal to posterior condyle

222. Fibula
0: bowed away from tibia
1: straight and not bowed away

223. Perforating artery in pes
0: located between astragalus and calcaneum
1: located between distal ends of tibia and fibula

224. Tibia-astragalus articulation
0: loosely defined
1: tightly fitting with well-developed articulation

225. Discrete astragalus
0: absent
1: present

226. Astragalus and calcaneum
0: not fused in adult
1: fused in adult
2: specialized hinge

227. Articulation between distal tarsal 4 and astragalus
0: poorly defined
1: well defined
2: absent

228. Calcaneal tuber
0: absent
1: present

229. Distal tarsal 1
0: present
1: absent

230. Distal tarsal 5
0: present
1: absent

231. Metatarsal V
0: long and slender
1: short with broad base

232. Metatarsal V
0: straight
1: hooked

233. Metatarsal V plantar tubercle
0: absent
1: present

234. Metatarsal I/IV ratio
0: length of metatarsal I more than 50% that of metatarsal IV
1: length of metatarsal I less than 50% that of metatarsal IV

235. Number of pedal Centralia
0: lateral and medial present
1: medial centrale absent
2: both centralia absent

236. Pedal digit V
0: longer than pedal digit I
1: slender, shorter than pedal digit I

237. Metapodials
0: not overlapping proximally
1: overlapping

238. Pedal phalangeal formula
0: 2-3-4-5(4)-4

1: 2-3-4-4-3
2: 2-3-3-4-3 (or fewer)

239. Limbs
0: short and stout
1: long and slender

240. Manus and pes
0: short and broad
1: long and slender

241. Unguals
0: shorter than phalanges
1: at least 50% longer than penultimate phalanges

242. Body osteoderms
0: absent
1: present but few restricted to mid-line
2: present and spread all over back

243. Osteodermal ridges
0: absent
1: fine, regularly spaced ridges
2: heavy, irregularly spaced ridges

244. Appendicular osteoderms
0: absent
1: present as conical studs

245. Gastralia
0: present
1: absent

246. Plastron
0: absent
1: **partial fusion of gastralia resulting in forked elements**
**2: fully developed plastron present**

247. T-shaped ribs
0: absent, ribs circular or oval in cross-section
1: **present, rib distinctly T-shaped in cross-section**

248. Ribs
0: not meeting or lying on top of each other
1: touching one another and lying on top of each other

249. Number of dorsal vertebrae
0: more than 10
1: **10**
**2: 9 or fewer**

250. Length of dorsal vertebrae
0: not greatly exceeding width
1: length more than twice centrum width

251. Trunk length
0: much greater than trunk width
1: trunk length and trunk width approximating each other

252. Sacral rib distal overlap
0: broad with narrow gaps between ribs
1: small or absent with wide gaps between ribs

253. Distinctly broadened ribs
0: absent
1: 10 or more ribs distinctly broadened
2: 9 or fewer ribs distinctly broadened

254. Outgrowth of dermal bone from perichondral collar of dorsal ribs
0: absent, dorsal ribs have endochondral ossification
1: present, dermal bone growing out of perichondral collar

255. Dorsal ribs
0: distinctly curved
1: nearly straight

256. Dorsal ribs
0: ending bluntly, indicating presence of ventral ribs or sternum
1: ending smoothly and tapered, indicating loss of ventral ribs or sternum

257. Sharpey's fibres on ventral portion of dorsal ribs
0: Sharpey's fibres present on lateral edges of ribs, but do not extend onto the ventral portion of rib
1: Sharpey's fibres present on ventral portion of ribs, often asymmetrically

258. Fan-shaped (flabellate) arrangement of dorsal ribs
0: absent, with dorsal ribs roughly parallel to each other on their entire length
1: present, with distal ends of dorsal ribs farther away from each other than proximal ends

259. Dorsal neural spines distinctly broadened to form neurals
0: absent
1: present

260. Gastralia
0: without lateral and medial segments
1: with lateral and medial segments
2: paired gastralia lacking lateral and medial portions

261. Dorsal osteoderms overlying ribcage
0: not sutured together
1: sutured together

262. Peripheral bones (dermal ossifications offset from overlying scales)
0: absent
1: osteoderms lateral to ribcage, offset from overlying scales

263. Number of ventral dermal ossifications (not including pectoral girdle elements)

0: more than 10 ventral ossifications
1: 10 or fewer dermal ventral ossifications

264. Osteoderms ventral to gastralia
0: absent
1: present

265. Squamosal
0: plate-like
1: with four distinct processes

266. Deep groove on the posterior face of the distal end of femur
0: absent
1: present

267. Dorsal process of maxilla
0: absent or if present rounded
1: tall with pointed apex

268. Median part of gastralia
0: throughout facing anteriorly
1: anterior gastralia with posteriorly facing median parts, intermediate ones straight medially, and posterior ones with anteriorly facing median parts

269. Pubis, lateral process
0: absent
1: present

270. Clavicle and interclavicle incorporated into plastron
0: not incorporated
1: incorporated into immobile bony plastron

**271. Dorsal rib expansion**
**0: all ribs**
**1: all but the most anterior**
**2: all but the most posterior**

**272. Cervical vertebral count**
**0: <7**
**1: 7 or 8**
**2: >8**

**273. Snout shape in lateral view**
**0: pointed, the anterior end of the skull noticeably shallower than the posterior part**
**1: rounded or rectangular, the snout is approximately as deep as the posterior part of the skull**

**274. External nares size**
**0: small, less than half the size of the orbit**
**1: large, half the size of the orbit or larger**

**275. Position of supratemporal in relation to parietal**
**0: posterior**
**1: lateral**

**276. Ventral temporal foramen/embayment adult shape**
**0: rounded, with distinct dorsal bar**
**1: notch-like, with angular dorsal margin**

**277. Quatratojugal exposition in lateral view**
**0: only or mainly ventral to squamosal**
**1: ventral and anterior along the most of the squamosal margin**

**278. Maximum height of the skull**
**0: caudally to orbit**
**1: around the orbit level**
**2: rostrally to orbit**
**3: the height is nearly uniform behind the rostrum**

Characters 33, 61, 66, 84, 117, 154, 192, 219, 246, 249, 253, and 272 are considered additive (ordered).

## CHARACTER MATRIX

15 characters per line.

***Proganochelys quenstedti***

| | | | | | | | | | | | | | | |
|---|---|---|---|---|---|---|---|---|---|---|---|---|---|---|
| 2 | 0 | 0 | 0 | 0 | 1 | 0 | 0 | 1 | 1 | 0 | 1 | ? | ? | 0 |
| 1 | 0 | 0 | 0 | 0 | 1 | 1 | 0 | 0 | 1 | 0 | 1 | ? | 1 | 0 |
| ? | ? | 0 | 2 | 0 | 0 | 0 | 0 | 2 | 1 | ? | 0 | 1 | 0 | 0 |
| 0 | 0 | 0 | 0 | 0 | 1 | 0 | 0 | 1 | 1 | 1 | 0 | 1 | 0 | 1 |
| 1 | 0 | 0 | 1 | 1 | 1 | ? | 1 | 0 | 1 | ? | ? | 2 | 1 | ? |
| 0 | 0 | 1 | 0 | 2 | 1 | 0 | 0 | 0 | 2 | 1 | 0 | 0 | 0 | 0 |
| ? | ? | ? | 0 | ? | ? | ? | 1 | 1 | 1 | 0 | 0 | 2 | 0 | 0 |
| 0 | 1 | 0 | 0 | 1 | 1 | 1 | 1 | 0 | 0 | 1 | ? | ? | 1 | 1 |
| 1 | 1 | 0 | 1 | 0 | 0 | 1 | 1 | 1 | 0 | 1 | 1 | 1 | ? | 2 |
| 1 | 1 | 0 | 1 | 1 | 0 | 1 | 0 | 0 | 1 | 1 | 1 | 1 | 1 | 1 |
| 0 | 1 | 1 | 1 | 1 | 1 | 1 | 0 | 1 | 1 | 1 | 1 | 0 | 1 | 0 |
| 0 | 1 | 0 | 0 | ? | ? | ? | ? | ? | 1 | 1 | 1 | 0 | 0 | 1 |
| 1 | 1 | 0 | 1 | 1 | 0 | 0 | 1 | 1 | 1 | 0 | 1 | 1 | 0 | 2 |
| 2 | 1 | 0 | 1 | 0 | 1 | 0 | 0 | 2 | 2 | 1 | 0 | 0 | 1 | 0 |
| 1 | 0 | 0 | 0 | 0 | 1 | 1 | 1 | 1 | 0 | 1 | 1 | 1 | 1 | 1 |
| 1 | 1 | 1 | 0 | 0 | 1 | 1 | 1 | 0 | 1 | 0 | 0 | 1 | 2 | 0 |
| 0 | 1 | 0 | 0 | 1 | 0 | 2 | 1 | 1 | 0 | 1 | 1 | 0 | 2 | 1 |
| 1 | 1 | 1 | 1 | 1 | 2 | ? | 1 | 0 | 0 | 0 | 1 | 1 | 1 | 1 |
| 1 | 1 | 1 | 0 | 0 | 0 | ? | 0 | 0 | | | | | | |

**Seymouriidae**

| | | | | | | | | | | | | | | |
|---|---|---|---|---|---|---|---|---|---|---|---|---|---|---|
| 0 | 0 | 0 | 0 | 0 | 0 | 0 | 0 | 0 | 0 | 0 | 0 | 0 | 0 | 0 |
| 0 | 0 | 0 | 0 | 0 | 0 | 0 | 0 | 1 | 0 | 1 | 0 | 0 | 0 | 0 |
| 0 | 0 | 0 | 0 | 0 | 0 | 0 | 0 | 1 | 0 | 0 | 0 | 0 | 0 | 0 |
| 0 | 1 | 0 | 0 | 0 | 1 | 2 | 0 | 0 | 0 | 0 | 0 | 1 | 0 | 0 |
| 0 | 0 | 0 | 0 | 0 | 0 | 0 | 0 | 0 | 0 | 0 | 0 | 0 | 0 | 0 |
| [0 1] | 0 | 0 | 0 | 1 | 0 | 0 | 0 | 1 | 0 | 0 | 0 | 0 | 0 | 0 |
| ? | ? | ? | 0 | 0 | ? | ? | 0 | ? | 0 | ? | ? | 0 | 0 | 1 |
| 0 | 0 | 1 | ? | 0 | 0 | 1 | 0 | 0 | 0 | 0 | 0 | 0 | 0 | 0 |
| ? | ? | 0 | 1 | 0 | 0 | 1 | 0 | 0 | 0 | 0 | 0 | 0 | 0 | 0 |
| 1 | 0 | 0 | ? | 0 | 2 | 0 | 1 | 0 | 0 | 0 | 1 | 0 | 1 | 0 |
| 0 | 0 | 0 | ? | 2 | 0 | 0 | 0 | 0 | 0 | 0 | 0 | 0 | 0 | 0 |
| 0 | 0 | 0 | ? | 0 | 0 | 0 | 0 | 0 | 0 | 0 | 0 | 0 | 0 | 0 |
| 0 | 0 | 0 | 0 | 0 | 0 | 0 | 0 | 0 | 0 | 0 | 0 | 0 | 0 | 0 |
| 0 | 0 | 0 | 0 | 0 | 0 | 0 | 0 | 0 | 0 | 0 | 0 | 1 | 0 | 0 |
| 0 | 0 | 0 | 0 | 0 | 0 | 0 | 0 | 0 | 0 | 0 | 0 | 0 | 0 | 0 |
| 0 | 0 | 0 | 0 | 0 | 0 | 0 | 0 | 0 | 0 | 0 | 0 | 0 | 0 | 0 |
| 0 | 0 | 0 | ? | 0 | 0 | 0 | 0 | 0 | 0 | 0 | 0 | 0 | 0 | 0 |
| 0 | 0 | ? | 0 | 0 | 0 | ? | 0 | 0 | 0 | 0 | 0 | 0 | 0 | 0 |
| 0 | ? | 2 | [0 1] | 0 | 1 | ? | 0 | 3 | | | | | | |

***Pappochelys rosinae***

| | | | | | | | | | | | | | | |
|---|---|---|---|---|---|---|---|---|---|---|---|---|---|---|
| 0 | 1 | 0 | 0 | ? | 0 | 0 | 0 | ? | ? | 0 | 0 | 1 | 0 | 0 |
| ? | 0 | 0 | 0 | 0 | 1 | ? | 0 | 1 | 0 | 0 | ? | ? | ? | 0 |
| 0 | 0 | 0 | ? | ? | ? | ? | 0 | ? | ? | ? | 1 | 0 | 0 | 0 |
| 0 | 1 | 0 | 1 | 1 | 0 | ? | 0 | 0 | 1 | 0 | 0 | 0 | ? | ? |
| ? | 0 | 0 | ? | ? | 1 | ? | 0 | 0 | ? | ? | ? | 2 | 1 | ? |
| 1 | 0 | 0 | 0 | 0 | 1 | 1 | 0 | ? | ? | 1 | ? | 0 | 1 | 1 |
| 0 | 1 | 1 | 1 | 1 | 0 | ? | ? | ? | ? | ? | ? | ? | ? | ? |
| ? | ? | ? | ? | ? | ? | ? | ? | ? | ? | ? | ? | ? | ? | ? |
| ? | ? | ? | ? | ? | ? | 1 | ? | 0 | 1 | ? | 1 | ? | ? | ? |
| ? | ? | ? | ? | ? | ? | ? | ? | ? | ? | ? | ? | ? | ? | ? |
| ? | ? | ? | ? | ? | ? | ? | ? | ? | ? | ? | ? | ? | ? | ? |
| ? | ? | ? | ? | 0 | 0 | 0 | 0 | 1 | ? | 0 | 0 | 0 | 0 | ? |
| ? | ? | ? | 1 | 1 | 0 | ? | 1 | ? | ? | ? | ? | 1 | 0 | 2 |
| 0 | ? | 0 | 0 | 0 | ? | 0 | 1 | ? | ? | 1 | 2 | ? | ? | ? |
| 1 | 0 | 0 | ? | 0 | ? | 1 | 1 | ? | 0 | 1 | 1 | 1 | ? | ? |
| ? | ? | ? | ? | ? | ? | ? | ? | ? | ? | ? | ? | ? | ? | 0 |
| 1 | 1 | 0 | ? | ? | 0 | 1 | 1 | 0 | ? | 0 | ? | 1 | ? | *1* |
| *1* | *1* | *1* | *?* | *0* | *0* | *?* | *0* | *0* | *?* | *1* | *1* | *1* | *1* | 1 |
| 0 | ? | ? | 0 | 0 | ? | 0 | ? | 0 | | | | | | |

***Odontochelys semitestacea***

| | | | | | | | | | | | | | | |
|---|---|---|---|---|---|---|---|---|---|---|---|---|---|---|
| 2 | 0 | 0 | 0 | 0 | 1 | 0 | 0 | 0 | ? | ? | 0 | 0 | 0 | 0 |
| ? | 0 | 0 | 0 | 0 | ? | ? | 0 | ? | ? | ? | ? | ? | ? | ? |
| ? | 0 | 0 | 2 | 0 | ? | ? | 0 | ? | ? | ? | 0 | ? | 1 | ? |
| 0 | 0 | 0 | ? | 0 | 1 | 0 | 0 | ? | 1 | ? | ? | ? | 0 | ? |
| ? | 0 | 0 | ? | ? | 1 | ? | ? | ? | ? | ? | ? | ? | ? | ? |
| 0 | ? | ? | ? | ? | ? | ? | ? | ? | ? | ? | 0 | 0 | 0 | 0 |
| ? | ? | ? | 0 | ? | ? | ? | ? | ? | 1 | 0 | 0 | ? | ? | 0 |
| 0 | 0 | 0 | 1 | 0 | ? | 1 | 0 | 0 | 0 | ? | ? | ? | ? | ? |
| ? | ? | ? | 1 | 1 | ? | 0 | 1 | 0 | ? | ? | ? | 1 | ? | ? |
| ? | ? | ? | ? | ? | ? | ? | ? | ? | 1 | 1 | 1 | 1 | 1 | 1 |
| 0 | ? | ? | ? | 1 | ? | ? | ? | ? | ? | ? | ? | ? | ? | 0 |
| ? | ? | 0 | 0 | 1 | 0 | 0 | 0 | 1 | 1 | 0 | 1 | 0 | 0 | ? |
| 1 | ? | 0 | ? | 1 | 0 | 0 | 1 | 1 | 1 | ? | 1 | 1 | 0 | 2 |
| 2 | ? | ? | 1 | 0 | ? | 0 | 1 | ? | ? | 1 | 2 | 1 | 1 | 1 |
| 1 | ? | 0 | ? | ? | ? | 1 | ? | ? | 0 | 1 | 1 | 1 | 1 | 1 |
| 1 | 1 | 1 | 0 | 0 | ? | 1 | 1 | 0 | ? | 2 | ? | 1 | 1 | 0 |
| 0 | 1 | 0 | ? | 0 | 0 | 2 | 1 | 0 | ? | 0 | 1 | 1 | 2 | 1 |
| 1 | 1 | ? | 1 | 1 | 2 | ? | 0 | 0 | 0 | 0 | ? | 1 | 1 | 1 |
| 1 | ? | 1 | ? | ? | ? | ? | ? | ? | | | | | | |

***Eunotosaurus africanus***

| | | | | | | | | | | | | | | |
|---|---|---|---|---|---|---|---|---|---|---|---|---|---|---|
| 2 | 0 | 0 | 0 | 0 | 1 | 0 | 0 | 0 | 0 | 0 | 0 | 0 | 0 | 0 |
| 1 | 0 | 0 | 0 | 1 | 1 | 1 | 0 | 0 | 1 | 0 | 0 | 0 | 0 | 0 |
| 0 | 0 | 0 | 2 | 0 | 0 | [0 1] | 0 | 1 | 0 | 0 | 0 | 1 | 1 | 0 |
| 0 | 1 | 0 | 0 | 0 | 1 | 0 | 0 | 0 | 1 | 0 | 0 | 1 | 0 | 0 |
| 0 | 0 | 0 | 0 | 1 | 0 | 0 | 0 | 0 | 0 | 0 | 1 | 1 | 1 | ? |
| 0 | 1 | ? | ? | 0 | ? | 0 | 0 | 0 | 2 | 1 | 0 | 0 | 1 | 1 |
| 0 | 0 | 1 | 1 | 1 | 0 | 1 | 0 | ? | 1 | 0 | 0 | 1 | 1 | 0 |
| 0 | 0 | 0 | 1 | 0 | 0 | 1 | 0 | 0 | 0 | 0 | 1 | 0 | 0 | 1 |
| 1 | 0 | 0 | 1 | 1 | 0 | 0 | 1 | 0 | 0 | 1 | 0 | 1 | ? | 2 |
| 1 | 1 | 0 | 0 | ? | 0 | 1 | 1 | 0 | ? | 1 | 1 | 1 | 1 | 1 |
| 0 | 1 | 1 | 0 | 1 | 1 | 1 | 0 | 1 | 1 | 0 | 1 | 0 | 1 | 0 |
| 0 | 1 | 0 | 0 | 1 | 0 | 0 | 0 | 1 | 1 | 0 | 0 | 0 | 0 | ? |
| 1 | 1 | 0 | 1 | 0 | 0 | 0 | ? | 1 | ? | 0 | 0 | 0 | ? | 1 |
| 0 | 1 | 0 | 0 | 0 | 0 | 0 | 0 | 2 | 2 | 0 | ? | ? | ? | ? |
| 0 | 1 | 0 | ? | 0 | 0 | 1 | 1 | 0 | 1 | 1 | 1 | 0 | 0 | 0 |
| 1 | [0 1] | ? | 0 | 0 | 0 | 1 | 0 | ? | 1 | 1 | 0 | 0 | 0 | 0 |
| 0 | 0 | 0 | ? | ? | 0 | 0 | 1 | 1 | 2 | 1 | 1 | 1 | 2 | 0 |
| 0 | 0 | 1 | 0 | 0 | 2 | ? | 0 | 1 | 0 | ? | ? | 0 | 0 | 0 |
| 0 | 0 | 2 | 1 | 1 | 1 | 1 | 1 | 3 | | | | | | |

***Acerosodontosaurus piveteaui***

| | | | | | | | | | | | | | | |
|---|---|---|---|---|---|---|---|---|---|---|---|---|---|---|
| ? | ? | ? | ? | ? | ? | ? | ? | ? | ? | 0 | ? | ? | ? | ? |
| ? | ? | ? | 0 | ? | ? | ? | ? | 1 | 0 | ? | ? | ? | ? | ? |
| 0 | 0 | 0 | ? | 0 | 0 | 0 | 0 | ? | ? | ? | 0 | 0 | ? | ? |
| ? | 1 | ? | ? | 0 | 0 | ? | 0 | 0 | ? | ? | ? | 0 | ? | 1 |
| ? | ? | ? | ? | ? | ? | ? | ? | ? | ? | ? | ? | ? | ? | ? |
| 0 | 0 | 0 | 0 | 0 | ? | 0 | 0 | ? | ? | ? | ? | 0 | ? | 1 |
| 0 | 0 | 1 | 1 | 1 | 0 | ? | ? | ? | ? | ? | ? | ? | ? | ? |
| ? | ? | ? | ? | ? | ? | ? | ? | ? | ? | 0 | 1 | ? | ? | 1 |
| 1 | ? | ? | ? | ? | ? | 0 | 0 | 1 | 1 | ? | ? | 1 | ? | ? |
| ? | ? | ? | ? | ? | ? | ? | ? | ? | ? | ? | ? | ? | ? | ? |
| ? | ? | ? | ? | ? | 1 | 1 | ? | ? | ? | ? | ? | ? | ? | ? |
| ? | 1 | ? | ? | 1 | 0 | ? | 0 | 1 | 0 | ? | 0 | 0 | 0 | ? |
| ? | ? | ? | ? | 0 | 0 | 0 | ? | ? | ? | 0 | ? | ? | ? | ? |
| ? | ? | ? | ? | 0 | ? | ? | 1 | ? | 0 | 0 | 2 | 1 | 1 | 1 |
| ? | 0 | 0 | ? | ? | ? | ? | ? | ? | ? | ? | ? | ? | ? | 0 |
| ? | ? | ? | ? | ? | ? | ? | ? | ? | ? | ? | ? | ? | ? | 0 |
| 1 | ? | 0 | ? | ? | 0 | 0 | 0 | 0 | 0 | 0 | 0 | ? | 0 | 0 |
| 0 | 0 | ? | 0 | ? | ? | ? | ? | ? | 0 | 1 | 1 | 0 | ? | 0 |
| ? | ? | ? | 0 | 0 | ? | 0 | 0 | 1 | | | | | | |

***Acleistorhinus pteroticus***

| | | | | | | | | | | | | | | |
|---|---|---|---|---|---|---|---|---|---|---|---|---|---|---|
| 0 | 0 | 0 | 0 | 0 | 1 | 0 | 0 | 0 | 0 | 0 | 0 | 0 | 0 | 0 |
| 0 | 0 | 0 | 0 | 0 | 1 | 1 | 0 | 0 | 0 | 1 | 0 | 0 | 0 | 1 |
| 1 | 0 | 0 | 1 | 0 | 0 | 0 | 0 | [1 2] | ? | ? | 0 | 0 | 0 | 1 |
| 0 | 1 | 1 | 1 | 0 | 1 | 0 | 0 | 0 | 1 | 0 | 1 | 1 | 0 | 0 |
| 0 | 0 | 0 | 0 | 1 | 0 | 0 | 0 | 0 | 0 | 1 | 1 | 1 | 0 | 1 |
| 0 | 0 | 0 | 1 | 1 | 1 | 1 | 0 | 0 | 1 | 1 | 0 | 0 | 1 | 0 |
| ? | ? | ? | 1 | 0 | 1 | 1 | 1 | 1 | 1 | 0 | 0 | 0 | 0 | 0 |
| 0 | 0 | 0 | 1 | 0 | 1 | 0 | 0 | 1 | 1 | 0 | 1 | 0 | 0 | 1 |
| 0 | 0 | 0 | 1 | 0 | 0 | 0 | 0 | 0 | 0 | 0 | 1 | 1 | ? | 0 |
| 0 | ? | 1 | 0 | ? | 2 | 0 | 1 | 0 | 1 | 0 | 0 | 1 | 0 | 1 |
| 0 | ? | ? | ? | 1 | ? | 1 | ? | ? | 1 | 0 | ? | 0 | 1 | 0 |
| 0 | 1 | 0 | ? | 0 | 1 | 0 | 0 | 1 | ? | ? | ? | ? | ? | ? |
| ? | ? | ? | ? | ? | ? | ? | ? | ? | ? | ? | ? | ? | ? | ? |
| ? | ? | ? | ? | ? | ? | ? | ? | ? | ? | ? | ? | ? | ? | ? |
| ? | ? | ? | ? | ? | ? | ? | ? | ? | ? | ? | ? | ? | ? | ? |
| ? | ? | ? | ? | ? | ? | ? | ? | ? | ? | ? | ? | ? | ? | ? |
| ? | ? | ? | ? | ? | ? | ? | ? | ? | ? | ? | ? | ? | ? | ? |
| ? | ? | ? | ? | ? | ? | ? | ? | ? | ? | 0 | 0 | 0 | 0 | 0 |
| ? | ? | ? | 0 | 0 | 1 | 0 | 0 | 1 | | | | | | |

**Araeoscelidae**

| | | | | | | | | | | | | | | |
|---|---|---|---|---|---|---|---|---|---|---|---|---|---|---|
| 1 | 0 | 0 | 0 | 0 | 0 | 0 | 0 | 0 | 0 | 0 | 0 | 0 | 0 | 0 |
| 0 | 0 | 0 | 0 | 0 | 0 | 0 | 0 | 1 | 0 | 1 | 0 | 1 | 1 | 0 |
| 0 | 0 | 0 | 0 | 0 | ? | 0 | 0 | 0 | 0 | 0 | 0 | 0 | 1 | 1 |
| 0 | 1 | 0 | 1 | 0 | 0 | ? | 0 | 0 | 1 | 1 | 0 | 0 | 0 | 1 |
| 1 | 0 | 0 | ? | 0 | 0 | 0 | 1 | 0 | 0 | 0 | 1 | 2 | 0 | 1 |
| [0 1] | 0 | 0 | 1 | [0 1] | [0 1] | 1 | 0 | 0 | 0 | 1 | 0 | 0 | 1 | 1 |
| 0 | 0 | 0 | 1 | 0 | 0 | 1 | 1 | 1 | 1 | 0 | 0 | 0 | 0 | 0 |
| 0 | 1 | [0 1] | 1 | 0 | 0 | 1 | 1 | 0 | 0 | 0 | 0 | 2 | 0 | 1 |
| 1 | 0 | 0 | 1 | 0 | ? | 0 | 0 | 0 | 0 | 0 | 0 | 1 | ? | 0 |
| 0 | ? | 0 | 0 | ? | 0 | 0 | 1 | 0 | 1 | ? | 1 | 0 | [0 1] | 1 |
| 0 | 0 | ? | ? | 0 | 0 | 1 | 0 | 0 | 0 | 0 | 0 | 0 | 1 | 0 |
| 0 | 0 | 0 | 0 | 0 | 1 | 0 | 0 | 1 | 0 | 0 | 0 | 0 | 0 | 0 |
| 1 | 0 | 0 | 1 | 0 | 0 | 0 | 0 | 0 | 0 | 0 | 0 | 1 | 1 | 0 |
| 0 | 0 | 1 | 0 | 0 | 0 | 1 | 0 | [1 2] | 0 | 0 | 0 | 0 | 0 | 0 |
| 0 | 0 | 0 | 0 | 0 | 0 | 1 | 0 | 0 | 0 | 0 | 0 | 1 | 0 | 1 |
| 1 | 0 | 0 | 0 | 0 | 0 | 0 | 0 | 0 | 1 | 1 | 0 | 1 | 0 | 1 |
| 1 | 0 | 0 | ? | 0 | 0 | 0 | 0 | 0 | 0 | 0 | 0 | 0 | 0 | 0 |
| 0 | 0 | ? | 0 | 0 | ? | ? | 0 | ? | 0 | 0 | 1 | 0 | 0 | 0 |
| 0 | ? | [0 1 2] | 0 | 0 | 1 | 0 | 0 | 1 | | | | | | |

**Archosauriformes**

| | | | | | | | | | | | | | | |
|---|---|---|---|---|---|---|---|---|---|---|---|---|---|---|
| 1 | 0 | 0 | [0 1] | 1 | 1 | 0 | 0 | [0 1] | 0 | 1 | 0 | 0 | 0 | 0 |
| 1 | 0 | 0 | 0 | 1 | 1 | 0 | 0 | [0 1] | [0 1] | 0 | 0 | 0 | 0 | 1 |
| 0 | 0 | 1 | [1 2] | 0 | 0 | 0 | 0 | ? | 0 | ? | 1 | 0 | [0 1] | [0 1] |
| 0 | 1 | 0 | [0 1] | 0 | 0 | ? | 0 | 0 | 1 | 1 | 0 | 0 | 1 | 1 |
| 1 | 1 | 1 | [0 1] | 0 | 1 | 1 | 1 | 0 | 1 | ? | ? | 2 | 1 | ? |
| 1 | 0 | 1 | 1 | 1 | 1 | 0 | 0 | 0 | 1 | 1 | 0 | 0 | 1 | 1 |
| 0 | [0 1] | 0 | 1 | 0 | 0 | 1 | 1 | [0 1] | 1 | [0 1] | 0 | 0 | 0 | 0 |
| 0 | 0 | 1 | ? | 1 | 0 | 1 | 0 | 0 | 0 | 0 | 1 | [0 1] | 0 | 1 |
| 1 | 0 | 0 | 0 | ? | 0 | 1 | 1 | 0 | [0 1] | 0 | 1 | 1 | ? | ? |
| 1 | ? | 0 | [0 1] | 1 | [0 1] | 1 | 1 | ? | 1 | 1 | [0 1] | 1 | 1 | 1 |
| [0 1] | 1 | 1 | 1 | 2 | 0 | 1 | [0 1] | ? | 1 | 0 | 0 | 0 | 1 | 0 |
| 0 | 0 | 1 | 1 | 1 | 0 | 1 | 0 | 1 | [0 1] | 0 | 1 | [0 1 2] | 0 | ? |
| 1 | [0 1] | 1 | 1 | [0 1] | 1 | 0 | 1 | 1 | 0 | 1 | 1 | 1 | 1 | [0 1] |
| 0 | 1 | 0 | [0 1] | [0 1] | 0 | 1 | [0 1] | 1 | 1 | 1 | 0 | 1 | 1 | 1 |
| 0 | 0 | 0 | 1 | 1 | 0 | 1 | 0 | 3 | [1 2] | [0 1] | 1 | 1 | 0 | 1 |
| 1 | 2 | 0 | 1 | [0 1] | 1 | 1 | 1 | 0 | 0 | 1 | 0 | 1 | 0 | 1 |
| 1 | 0 | 1 | 0 | 0 | 0 | 0 | 0 | 0 | 0 | 0 | 0 | 0 | [0 1] | [0 1] |
| 0 | 0 | ? | 0 | 0 | 1 | 0 | 0 | 0 | 0 | 1 | 1 | 1 | 0 | 0 |
| 0 | ? | [0 1 2] | 0 | [0 1] | 1 | 0 | 0 | [0 3] | | | | | | |

***Australothyris smithi***

| | | | | | | | | | | | | | | |
|---|---|---|---|---|---|---|---|---|---|---|---|---|---|---|
| 0 | 0 | 0 | 0 | 0 | ? | 0 | 0 | 0 | ? | 0 | 0 | 0 | 0 | 0 |
| ? | 0 | ? | ? | ? | ? | ? | ? | 1 | 1 | 1 | ? | ? | ? | 1 |
| 0 | 0 | 0 | ? | 1 | 0 | 0 | 0 | [1 2] | 0 | 2 | 0 | ? | 0 | ? |
| 0 | 1 | ? | ? | 0 | 1 | 0 | 0 | 0 | 1 | 0 | 0 | 0 | 0 | 0 |
| 0 | 0 | 0 | ? | ? | 0 | 0 | 0 | 0 | 0 | 0 | 0 | 1 | 0 | 1 |
| 0 | ? | 0 | ? | 1 | ? | 1 | 0 | 1 | 1 | 0 | 0 | 0 | 1 | 0 |
| ? | ? | ? | 1 | 0 | 0 | 1 | 1 | 0 | 1 | 1 | 0 | ? | 1 | 0 |
| 0 | 0 | 0 | 1 | ? | 1 | 0 | 1 | 0 | 0 | 0 | 1 | 0 | 0 | ? |
| ? | ? | 0 | 0 | 0 | 0 | 0 | 0 | 0 | 1 | ? | 1 | 0 | 0 | 0 |
| 0 | ? | 0 | 0 | ? | 0 | 0 | 1 | 0 | 0 | ? | 1 | 1 | 0 | 0 |
| 1 | ? | 0 | ? | 1 | ? | 1 | ? | 0 | 0 | 0 | ? | 0 | 0 | 0 |
| 0 | 1 | 0 | ? | 1 | 0 | 0 | 0 | 1 | ? | ? | ? | ? | ? | ? |
| ? | ? | ? | ? | ? | ? | ? | ? | ? | ? | ? | ? | 0 | ? | ? |
| ? | ? | ? | ? | ? | ? | ? | ? | ? | ? | ? | ? | ? | ? | ? |
| ? | ? | ? | ? | ? | ? | ? | ? | ? | ? | ? | ? | ? | ? | ? |
| ? | ? | ? | ? | ? | ? | ? | ? | ? | ? | ? | ? | ? | ? | ? |
| ? | ? | ? | ? | ? | ? | ? | 0 | ? | ? | ? | ? | ? | 0 | ? |
| ? | ? | ? | ? | ? | ? | ? | ? | ? | ? | 0 | ? | ? | ? | ? |
| 0 | ? | ? | ? | ? | 1 | 0 | 0 | ? | | | | | | |

***Barasaurus besairiei***

| | | | | | | | | | | | | | | |
|---|---|---|---|---|---|---|---|---|---|---|---|---|---|---|
| 0 | 0 | 0 | ? | 0 | 1 | 0 | 0 | 0 | ? | 0 | 0 | ? | ? | 0 |
| ? | 0 | 0 | 1 | 0 | 1 | 0 | 0 | ? | 0 | 0 | ? | ? | ? | 1 |
| ? | 0 | 0 | 0 | ? | ? | ? | 1 | ? | 1 | ? | 0 | 0 | 0 | 0 |
| 0 | 1 | 0 | ? | 1 | 1 | ? | 0 | 0 | 1 | ? | 0 | 1 | 0 | 0 |
| 0 | 0 | 0 | ? | ? | 0 | 1 | 0 | 0 | 0 | 0 | 0 | 1 | 1 | ? |
| 0 | 1 | ? | ? | ? | 0 | ? | 0 | 0 | 1 | 0 | 0 | 0 | ? | 0 |
| ? | ? | ? | 1 | 2 | 1 | 1 | 1 | 1 | 1 | 0 | 0 | ? | 1 | 0 |
| 0 | 1 | 1 | ? | ? | 1 | 0 | 1 | 0 | 0 | 0 | 1 | ? | 0 | 1 |
| 0 | ? | 0 | ? | ? | 0 | 1 | 0 | ? | 0 | ? | 0 | 0 | 0 | ? |
| ? | ? | 1 | ? | ? | 0 | ? | 1 | 0 | 1 | ? | 1 | 1 | 1 | 1 |
| 1 | ? | ? | ? | 1 | ? | ? | ? | ? | ? | ? | ? | ? | ? | ? |
| ? | ? | ? | ? | 1 | 0 | ? | 0 | 1 | 0 | ? | ? | ? | ? | ? |
| ? | ? | ? | 2 | ? | ? | 1 | ? | ? | ? | ? | ? | 1 | 0 | 1 |
| 0 | ? | ? | ? | ? | ? | 0 | ? | 1 | ? | 1 | ? | 1 | ? | ? |
| ? | 1 | ? | ? | ? | ? | 1 | ? | ? | ? | ? | ? | 0 | 0 | 0 |
| 1 | 1 | 1 | ? | 0 | 0 | 0 | 0 | ? | 0 | 1 | 0 | 0 | 0 | 1 |
| 0 | ? | 0 | ? | ? | ? | 0 | 0 | 0 | ? | ? | 0 | 1 | 0 | ? |
| 0 | 0 | ? | 0 | ? | ? | ? | ? | ? | ? | 0 | ? | 0 | ? | 0 |
| 0 | ? | 2 | 1 | 0 | 1 | 1 | 0 | 3 | | | | | | |

***Bashkyroleter bashkyricus***

| | | | | | | | | | | | | | | |
|---|---|---|---|---|---|---|---|---|---|---|---|---|---|---|
| ? | ? | ? | ? | ? | ? | ? | 0 | ? | ? | ? | ? | ? | ? | ? |
| ? | ? | ? | 0 | ? | 1 | ? | ? | ? | ? | ? | ? | ? | ? | ? |
| ? | ? | ? | 2 | ? | ? | ? | 0 | 2 | 1 | 2 | ? | ? | ? | ? |
| ? | 1 | 0 | ? | 1 | 1 | 1 | 0 | ? | ? | ? | ? | 1 | 0 | 0 |
| 0 | ? | ? | ? | ? | 0 | 1 | 0 | ? | 0 | ? | 1 | 1 | 0 | 1 |
| 0 | 0 | 0 | ? | ? | 0 | ? | ? | 0 | ? | ? | 1 | 1 | ? | 0 |
| ? | ? | ? | 0 | ? | ? | ? | ? | ? | 1 | 1 | ? | ? | ? | ? |
| 0 | 0 | 0 | ? | ? | 1 | 0 | ? | 1 | 0 | ? | ? | ? | ? | 1 |
| 0 | ? | ? | ? | ? | 1 | ? | 0 | ? | ? | ? | 1 | 0 | ? | ? |
| ? | ? | 1 | ? | ? | 0 | ? | ? | ? | ? | ? | ? | 2 | 1 | 1 |
| ? | ? | ? | ? | ? | ? | 1 | 1 | 1 | 1 | ? | ? | ? | ? | ? |
| ? | 1 | 0 | ? | ? | 0 | ? | 0 | 1 | ? | ? | ? | ? | ? | ? |
| ? | ? | ? | ? | ? | ? | ? | ? | ? | ? | ? | ? | ? | ? | ? |
| ? | ? | ? | ? | ? | ? | ? | ? | ? | ? | ? | ? | ? | ? | ? |
| ? | ? | ? | ? | ? | ? | ? | ? | ? | ? | ? | ? | ? | ? | ? |
| ? | ? | ? | ? | ? | ? | ? | ? | ? | ? | ? | ? | ? | ? | ? |
| ? | ? | ? | ? | ? | ? | ? | ? | ? | ? | ? | ? | ? | 0 | ? |
| ? | ? | ? | ? | ? | ? | ? | ? | ? | ? | 0 | ? | 0 | ? | ? |
| ? | ? | ? | 0 | 0 | 0 | ? | 0 | 0 | | | | | | |

***Bashkyroleter mesensis***

| | | | | | | | | | | | | | | |
|---|---|---|---|---|---|---|---|---|---|---|---|---|---|---|
| 0 | 0 | 0 | 0 | 0 | 1 | 0 | 0 | 0 | ? | 0 | 0 | 1 | 0 | 0 |
| 0 | 0 | 0 | 0 | ? | 1 | 0 | 0 | 0 | 0 | 0 | ? | ? | ? | 1 |
| 0 | 0 | 0 | 1 | 0 | ? | 0 | 0 | 2 | 1 | 2 | ? | ? | ? | ? |
| ? | 1 | 0 | ? | 1 | 1 | 1 | 0 | 0 | 1 | ? | 0 | 1 | 0 | 0 |
| 0 | ? | 0 | ? | ? | 0 | 0 | 0 | ? | 0 | 1 | 1 | 1 | 0 | 1 |
| [0 1] | 0 | 0 | 0 | 1 | 0 | ? | 0 | 0 | 1 | 1 | 1 | 0 | 0 | 0 |
| ? | ? | ? | 0 | ? | ? | ? | 1 | 1 | 1 | 1 | ? | ? | 0 | ? |
| 0 | 0 | 0 | ? | ? | 0 | 0 | ? | 1 | 0 | 0 | 1 | ? | 1 | ? |
| ? | ? | ? | ? | ? | 1 | ? | 0 | ? | 0 | ? | 1 | 0 | 1 | ? |
| ? | ? | 1 | ? | ? | 0 | ? | ? | ? | ? | 1 | 1 | 2 | 1 | 1 |
| 0 | ? | ? | ? | 0 | ? | 1 | 1 | 1 | 1 | ? | ? | ? | ? | ? |
| ? | 1 | 0 | ? | 0 | ? | ? | 0 | 1 | ? | ? | ? | ? | ? | ? |
| ? | ? | ? | ? | ? | ? | ? | ? | ? | ? | ? | ? | ? | ? | ? |
| ? | ? | ? | ? | ? | ? | ? | ? | ? | ? | ? | ? | ? | ? | ? |
| ? | ? | ? | ? | ? | ? | ? | ? | ? | ? | ? | ? | ? | ? | ? |
| ? | ? | ? | ? | ? | ? | ? | ? | ? | ? | ? | ? | ? | ? | ? |
| ? | ? | ? | ? | ? | ? | ? | 0 | ? | ? | ? | ? | ? | 0 | ? |
| ? | ? | ? | ? | ? | ? | ? | ? | ? | ? | 0 | ? | 0 | ? | ? |
| ? | ? | ? | 0 | 0 | ? | ? | 0 | 0 | | | | | | |

***Belebey vegrandis***

| | | | | | | | | | | | | | | |
|---|---|---|---|---|---|---|---|---|---|---|---|---|---|---|
| 1 | 0 | 0 | 0 | 0 | 1 | 0 | 0 | 0 | 0 | 0 | 0 | 0 | 0 | 0 |
| ? | 0 | 0 | 0 | 0 | 0 | 0 | 0 | 1 | 0 | 1 | 1 | 0 | 0 | 1 |
| 0 | 0 | 0 | 0 | 0 | 0 | 0 | 0 | 2 | 1 | 2 | 0 | 0 | 0 | 1 |
| 0 | 1 | 0 | 0 | 1 | 0 | ? | 0 | 0 | 1 | 1 | 0 | 1 | 1 | 1 |
| 1 | 0 | 0 | ? | ? | 0 | 2 | 1 | 0 | 0 | 0 | 1 | 2 | 0 | 0 |
| 0 | 1 | ? | ? | 0 | 1 | 1 | 0 | 1 | 1 | 0 | 0 | 0 | 0 | 0 |
| ? | ? | ? | 1 | 0 | 1 | 1 | ? | ? | 1 | 1 | 0 | 0 | 1 | 1 |
| 1 | 2 | 1 | ? | 1 | 1 | 0 | 1 | 0 | 0 | 0 | 1 | 0 | 0 | 0 |
| ? | ? | 1 | 0 | ? | 0 | ? | 0 | 0 | 0 | 0 | 0 | 1 | ? | ? |
| ? | 0 | ? | ? | ? | 0 | ? | ? | 0 | ? | 0 | 0 | 0 | 1 | 1 |
| ? | 0 | 0 | ? | 0 | 2 | 1 | 0 | 1 | 1 | 0 | 0 | 0 | 1 | 0 |
| 0 | 1 | 0 | ? | 1 | 0 | 0 | 0 | 1 | ? | ? | ? | ? | ? | ? |
| ? | ? | ? | 2 | ? | ? | ? | ? | ? | ? | ? | ? | ? | ? | 0 |
| ? | ? | ? | ? | ? | ? | ? | ? | 1 | ? | ? | ? | ? | ? | ? |
| ? | 0 | ? | ? | ? | ? | 1 | ? | ? | ? | ? | ? | ? | ? | ? |
| ? | ? | ? | ? | ? | ? | ? | ? | ? | ? | ? | ? | ? | ? | ? |
| ? | ? | 0 | ? | ? | ? | ? | ? | ? | ? | ? | ? | ? | ? | ? |
| ? | ? | ? | ? | ? | ? | ? | ? | ? | ? | 0 | ? | 0 | ? | ? |
| ? | ? | ? | 0 | 0 | 1 | 0 | 0 | 1 | | | | | | |

***Bradysaurus* spp.**

| | | | | | | | | | | | | | | |
|---|---|---|---|---|---|---|---|---|---|---|---|---|---|---|
| 2 | 0 | 0 | 0 | 0 | 1 | 0 | 0 | 0 | 0 | ? | 0 | 0 | 0 | 0 |
| 0 | 0 | 0 | 0 | 1 | 1 | 0 | 0 | 1 | 0 | 0 | 0 | 0 | 0 | 1 |
| 0 | 0 | 0 | 0 | 0 | ? | 0 | 0 | 1 | 0 | ? | 0 | 1 | 1 | 0 |
| 0 | 0 | 0 | 0 | 0 | 1 | 1 | 1 | 0 | 1 | 1 | 0 | 1 | 0 | 0 |
| 0 | 0 | 0 | 0 | 0 | 0 | 1 | 0 | 0 | 0 | 1 | 0 | 1 | 1 | ? |
| 1 | 1 | ? | ? | 1 | 0 | 1 | 0 | 2 | 0 | 0 | 1 | 1 | 0 | 0 |
| ? | ? | ? | 0 | ? | ? | ? | 1 | 1 | 1 | 1 | 1 | 1 | 1 | 0 |
| 0 | 1 | 0 | 1 | 0 | 1 | 0 | 1 | 1 | 0 | 0 | 1 | 1 | 1 | 1 |
| 0 | 1 | 0 | 1 | 0 | 1 | 0 | 0 | 0 | 0 | ? | 1 | 1 | 0 | ? |
| 0 | ? | 1 | 1 | ? | 0 | 1 | 0 | 1 | 1 | 0 | 1 | 1 | 1 | 1 |
| 0 | 0 | 0 | ? | 1 | 1 | 1 | 1 | 1 | 1 | 0 | 1 | 0 | 0 | 0 |
| 0 | 1 | 1 | 0 | 1 | 0 | 0 | 2 | 1 | 1 | 0 | 0 | 0 | 0 | ? |
| 0 | 0 | 0 | 2 | 0 | 0 | 1 | 1 | 1 | 1 | 0 | 0 | 1 | 0 | 1 |
| 1 | ? | 1 | 0 | 0 | 0 | 0 | 0 | 2 | 2 | 0 | 0 | 1 | 0 | 0 |
| 0 | 1 | 1 | 0 | 1 | 1 | 0 | 0 | 0 | 0 | 0 | 0 | 0 | 0 | 0 |
| 1 | 1 | 0 | 0 | 0 | 1 | 0 | 0 | 0 | 0 | 2 | 1 | 0 | 2 | 0 |
| 0 | 1 | 1 | 0 | 0 | 1 | 0 | 0 | 0 | 1 | 0 | 0 | 1 | 0 | 0 |
| 0 | 0 | ? | 0 | 0 | ? | 0 | 0 | ? | ? | 0 | 0 | 0 | 0 | 0 |
| 0 | ? | 2 | 1 | 1 | 0 | ? | 0 | 3 | | | | | | |

***Candelaria barbouri***

| | | | | | | | | | | | | | | |
|---|---|---|---|---|---|---|---|---|---|---|---|---|---|---|
| 0 | 1 | 0 | 0 | 0 | 0 | 0 | 0 | 1 | ? | 0 | 0 | 0 | 0 | 0 |
| 1 | 0 | 0 | 0 | 0 | 1 | 0 | 0 | 1 | 0 | 0 | ? | ? | ? | 0 |
| 0 | 0 | 0 | 2 | 0 | ? | 0 | 0 | ? | ? | ? | 0 | 0 | 0 | ? |
| 0 | 1 | 0 | 0 | 1 | 0 | ? | 0 | 0 | ? | 0 | 0 | 0 | 0 | 0 |
| 0 | 0 | 0 | ? | ? | 0 | 0 | 1 | 0 | ? | ? | ? | 1 | ? | ? |
| 0 | 1 | ? | ? | 2 | ? | 1 | 0 | 1 | 1 | 1 | 0 | 0 | 0 | 1 |
| 0 | 0 | 0 | 1 | 1 | 1 | 1 | ? | ? | ? | ? | ? | ? | ? | ? |
| ? | ? | ? | ? | ? | ? | ? | ? | ? | ? | ? | ? | ? | ? | ? |
| ? | ? | ? | ? | ? | ? | 1 | 1 | ? | ? | ? | ? | 0 | ? | ? |
| ? | ? | ? | ? | ? | ? | ? | ? | ? | ? | ? | ? | ? | ? | ? |
| ? | ? | ? | ? | 2 | ? | ? | ? | ? | 0 | ? | ? | ? | ? | ? |
| 0 | ? | ? | ? | 0 | 0 | ? | 0 | 1 | ? | ? | ? | ? | ? | ? |
| ? | ? | ? | ? | ? | ? | ? | ? | ? | ? | ? | ? | ? | ? | ? |
| ? | ? | ? | ? | ? | ? | ? | ? | ? | ? | ? | ? | ? | ? | ? |
| ? | ? | ? | ? | ? | ? | ? | ? | ? | ? | ? | ? | ? | ? | ? |
| ? | ? | ? | ? | ? | ? | ? | ? | ? | ? | ? | ? | ? | ? | ? |
| ? | ? | ? | ? | ? | ? | ? | ? | ? | ? | ? | ? | ? | ? | ? |
| ? | ? | ? | ? | ? | ? | ? | ? | ? | ? | 0 | ? | 0 | ? | ? |
| ? | ? | ? | 0 | 0 | 1 | 1 | 1 | 3 | | | | | | |

**Captorhinidae**

| | | | | | | | | | | | | | | |
|---|---|---|---|---|---|---|---|---|---|---|---|---|---|---|
| 0 | 0 | 0 | 1 | 0 | 0 | 0 | 0 | 0 | 0 | 0 | 0 | 0 | 0 | 0 |
| ? | 0 | 0 | 0 | 0 | 0 | 0 | 0 | 1 | 0 | 0 | 1 | ? | 0 | 0 |
| 0 | 0 | 0 | 0 | 0 | 0 | 0 | 0 | 1 | 0 | 1 | 0 | 0 | 0 | 0 |
| 0 | 1 | 0 | 0 | 0 | 1 | 2 | 0 | 0 | 1 | 1 | 0 | 1 | 0 | 1 |
| 1 | 0 | 0 | 0 | 0 | 0 | [0 1] | 1 | 0 | 0 | 0 | 1 | 2 | 1 | ? |
| 1 | 0 | 0 | 0 | 0 | 1 | 1 | 0 | 1 | 0 | 1 | 0 | 0 | 0 | 0 |
| ? | ? | ? | 0 | ? | ? | ? | 1 | 1 | 1 | 0 | 0 | 0 | 0 | 0 |
| 0 | 0 | 0 | 0 | 0 | 0 | 1 | 0 | 0 | 0 | 1 | ? | ? | 0 | 1 |
| 0 | 1 | 0 | 0 | ? | 0 | 0 | 0 | 0 | 1 | 0 | 0 | 1 | ? | 0 |
| 0 | 0 | 0 | 0 | 0 | 0 | 0 | 1 | 0 | 1 | 0 | 1 | 0 | [0 1] | 1 |
| 0 | 0 | 0 | ? | 0 | 0 | 1 | 0 | 0 | 0 | 0 | 0 | 0 | 0 | 0 |
| 0 | 0 | 0 | 0 | 0 | 1 | 0 | 0 | 1 | 0 | 0 | 0 | 0 | 0 | 0 |
| 0 | 0 | 0 | 0 | 0 | 0 | 0 | 0 | 0 | 0 | [0 1] | 0 | 0 | 0 | 0 |
| 0 | 0 | 1 | 0 | 0 | 0 | 0 | 0 | 2 | 0 | 0 | 0 | 0 | 0 | 0 |
| 0 | 0 | 0 | 0 | 0 | 0 | 0 | 0 | 0 | 0 | 0 | 0 | 0 | 0 | 0 |
| 1 | 0 | 0 | 0 | 0 | 0 | 0 | 0 | 0 | 0 | 1 | 0 | 0 | 0 | 0 |
| 0 | 0 | 0 | ? | 0 | 0 | 0 | 0 | 0 | 0 | 0 | 0 | 0 | 0 | 0 |
| 0 | 0 | ? | 0 | 0 | ? | ? | 0 | 0 | 0 | 0 | 0 | 0 | 0 | 0 |
| 0 | ? | 2 | 0 | 0 | [0 1] | ? | 0 | [0 3] | | | | | | |

**Caseidae**

| | | | | | | | | | | | | | | |
|---|---|---|---|---|---|---|---|---|---|---|---|---|---|---|
| 0 | 0 | 0 | 0 | 0 | 0 | 0 | 0 | 0 | 0 | 0 | 0 | 0 | 0 | 0 |
| 0 | 0 | 0 | [0 1] | 0 | 0 | 0 | 0 | 0 | 1 | 0 | 0 | 0 | ? | 0 |
| 0 | 0 | 0 | 0 | 0 | 0 | 1 | 0 | 0 | 0 | 0 | 0 | 0 | 0 | 0 |
| 0 | 0 | 0 | 0 | 0 | 0 | ? | 0 | 0 | 1 | 1 | 1 | 1 | 0 | 0 |
| 0 | 0 | 0 | 0 | 0 | 0 | 0 | 0 | 0 | 0 | 0 | 1 | 1 | 0 | 0 |
| 0 | 0 | 0 | 1 | 0 | 0 | 1 | 0 | 0 | 0 | 0 | 0 | 0 | 0 | 0 |
| ? | ? | ? | 1 | 0 | 0 | 0 | 1 | 0 | 1 | 0 | ? | 0 | 0 | 0 |
| 0 | 0 | 0 | 1 | 0 | 0 | 0 | 0 | 0 | 0 | ? | 0 | 0 | 0 | 0 |
| ? | ? | ? | ? | ? | 0 | 0 | 0 | 0 | 0 | 0 | 0 | 0 | ? | 0 |
| 0 | 0 | 0 | 0 | 0 | 0 | 0 | 1 | 0 | 1 | 0 | [0 1] | 0 | [0 1] | 0 |
| 0 | 0 | 0 | ? | 0 | 0 | 0 | 0 | 0 | [0 1] | 0 | 0 | 0 | 0 | 0 |
| 0 | 0 | 0 | 0 | 0 | 1 | 0 | 0 | 1 | 0 | 0 | 0 | 0 | 0 | 0 |
| 0 | 0 | 0 | 1 | 0 | 0 | 0 | 0 | 0 | 0 | 0 | 0 | 0 | 0 | 1 |
| 0 | 0 | 0 | 0 | 0 | 0 | 0 | 0 | 0 | 0 | 0 | 0 | 0 | 0 | 0 |
| 0 | 0 | 0 | 0 | 0 | 0 | [0 1] | 0 | 0 | 0 | 0 | 0 | 0 | 0 | 0 |
| 1 | 0 | 0 | 0 | 0 | 0 | 0 | 0 | 0 | 0 | 0 | 0 | 0 | 0 | 0 |
| [0 1] | 0 | 0 | ? | 0 | 0 | 0 | 0 | 0 | 0 | 0 | 0 | 0 | 0 | 0 |
| 0 | 0 | ? | 0 | 0 | ? | ? | 0 | ? | 0 | 0 | 0 | 0 | 0 | 0 |
| 0 | ? | 2 | 1 | 1 | 1 | 1 | 0 | 2 | | | | | | |

***Claudiosaurus germaini***

| | | | | | | | | | | | | | | |
|---|---|---|---|---|---|---|---|---|---|---|---|---|---|---|
| 1 | 0 | 0 | 0 | 0 | 1 | 0 | 0 | 0 | 0 | 0 | 0 | 0 | 0 | 0 |
| ? | 0 | 0 | ? | 0 | 1 | 0 | 0 | 1 | 1 | ? | 0 | 1 | 0 | 0 |
| 0 | 0 | 0 | 1 | 1 | ? | 1 | 0 | ? | ? | ? | 0 | 0 | 0 | ? |
| 0 | 1 | 0 | 1 | 0 | 0 | ? | 0 | 0 | 1 | 1 | 1 | 0 | 0 | 1 |
| 1 | 0 | 1 | ? | 1 | 0 | 0 | 1 | 0 | 0 | 1 | 1 | 2 | 1 | ? |
| 0 | 0 | 0 | 1 | 0 | 1 | 1 | 0 | 0 | 0 | 1 | 0 | 0 | 1 | 1 |
| 0 | 0 | 1 | 1 | 1 | 0 | 1 | 1 | 1 | 1 | 0 | 0 | 0 | 0 | 0 |
| 1 | 1 | 0 | 1 | 1 | 0 | 1 | 1 | 0 | 0 | 0 | 1 | 2 | 0 | 1 |
| 1 | 0 | 0 | 1 | 0 | 0 | 1 | 0 | 0 | 1 | 1 | 1 | 1 | ? | ? |
| ? | ? | 0 | 0 | ? | 0 | 0 | 1 | ? | 0 | ? | 1 | 0 | 1 | 1 |
| 0 | ? | ? | ? | 0 | 0 | 1 | 0 | 0 | 1 | 0 | 0 | 0 | 1 | 0 |
| 0 | 0 | 0 | 0 | 1 | 0 | 0 | 0 | 1 | 0 | 0 | ? | 0 | 0 | 1 |
| 1 | 0 | 1 | 1 | 0 | 0 | 0 | 0 | 1 | 0 | 1 | 1 | 1 | ? | 0 |
| 0 | 1 | 0 | 0 | 1 | 0 | 1 | 1 | 2 | 1 | 0 | 0 | 1 | 0 | 1 |
| 0 | 0 | 0 | 0 | 1 | 0 | 1 | ? | 0 | 1 | 1 | 1 | 0 | 0 | 0 |
| 1 | 0 | 0 | 0 | 0 | 0 | 0 | 0 | 0 | 0 | 1 | 0 | 1 | 0 | 0 |
| 1 | 0 | 0 | ? | 0 | 0 | 0 | 0 | 0 | 0 | 0 | 0 | 0 | 0 | 0 |
| 0 | 0 | ? | 0 | 0 | ? | ? | 0 | ? | 0 | 1 | ? | 1 | 0 | 0 |
| 0 | ? | 1 | 0 | 0 | 0 | 0 | 0 | 0 | | | | | | |

***Colobomycter pholeter***

| | | | | | | | | | | | | | | |
|---|---|---|---|---|---|---|---|---|---|---|---|---|---|---|
| 0 | ? | ? | ? | ? | 1 | ? | ? | ? | ? | ? | ? | ? | ? | ? |
| ? | ? | ? | ? | ? | 1 | 1 | ? | ? | ? | 0 | ? | ? | ? | 1 |
| ? | ? | ? | 0 | ? | ? | ? | 0 | 1 | 0 | ? | ? | ? | ? | ? |
| ? | 1 | 1 | ? | 0 | 1 | 0 | ? | ? | ? | ? | ? | 1 | ? | ? |
| ? | ? | ? | ? | ? | 0 | 0 | ? | ? | 0 | 0 | 1 | ? | ? | ? |
| 0 | ? | ? | ? | ? | 1 | ? | 0 | 1 | 0 | 0 | 0 | 0 | ? | 0 |
| ? | ? | ? | 1 | 0 | ? | 1 | ? | ? | ? | ? | ? | ? | 0 | ? |
| 0 | 0 | 1 | [0 1] | ? | 1 | 0 | ? | ? | ? | 0 | 1 | ? | 0 | ? |
| ? | ? | ? | ? | ? | ? | ? | 0 | ? | ? | ? | ? | 1 | ? | ? |
| ? | ? | ? | ? | ? | ? | ? | ? | ? | ? | ? | ? | ? | 0 | ? |
| ? | ? | ? | ? | 0 | ? | ? | 0 | 0 | ? | 0 | ? | 0 | 1 | ? |
| ? | ? | 1 | ? | 0 | 1 | ? | 0 | 1 | ? | ? | ? | ? | ? | ? |
| ? | ? | ? | ? | ? | ? | ? | ? | ? | ? | ? | ? | ? | ? | ? |
| ? | ? | ? | ? | ? | ? | ? | ? | ? | ? | ? | ? | ? | ? | ? |
| ? | ? | ? | ? | ? | ? | ? | ? | ? | ? | ? | ? | ? | ? | ? |
| ? | ? | ? | ? | ? | ? | ? | ? | ? | ? | ? | ? | ? | ? | ? |
| ? | ? | ? | ? | ? | ? | ? | ? | ? | ? | ? | ? | ? | ? | ? |
| ? | ? | ? | ? | ? | ? | ? | ? | ? | ? | ? | ? | 1 | ? | ? |
| ? | ? | ? | 0 | 0 | ? | ? | ? | ? | | | | | | |

***Delorhynchus cifelli***

| | | | | | | | | | | | | | | |
|---|---|---|---|---|---|---|---|---|---|---|---|---|---|---|
| 0 | 0 | 0 | 0 | 0 | 1 | 0 | 0 | 0 | ? | ? | 0 | 0 | 0 | 0 |
| 0 | 0 | 0 | 1 | 0 | 1 | 1 | 0 | 0 | 1 | 1 | 1 | 0 | 1 | 1 |
| 0 | 0 | 0 | 2 | 0 | 0 | 0 | 0 | 1 | 1 | 2 | 0 | 0 | 0 | 1 |
| 0 | 1 | 0 | 1 | 0 | 1 | 0 | 1 | 0 | 1 | 1 | 0 | 1 | 0 | 0 |
| 0 | 0 | 0 | ? | ? | 0 | 0 | 0 | 0 | 0 | 0 | 1 | 1 | 0 | 1 |
| 0 | 0 | 0 | 1 | ? | 1 | ? | 0 | 0 | 1 | 1 | 0 | 0 | 1 | 0 |
| ? | ? | ? | 1 | 0 | 0 | 1 | 1 | ? | 1 | 1 | ? | ? | 0 | 0 |
| 0 | 0 | 0 | 1 | 0 | 0 | 1 | ? | 0 | ? | 0 | 0 | 1 | 0 | 0 |
| ? | ? | 0 | 1 | 0 | 0 | ? | 0 | ? | 0 | ? | 1 | 1 | ? | 0 |
| 0 | ? | 0 | 0 | ? | ? | ? | ? | ? | ? | ? | 1 | 1 | 0 | 1 |
| ? | ? | ? | ? | 1 | 0 | 0 | 0 | 0 | 1 | 0 | ? | 0 | 1 | 0 |
| 0 | 1 | 1 | 0 | 1 | 0 | ? | 0 | 1 | ? | ? | 0 | 0 | ? | ? |
| 1 | 0 | ? | 1 | 0 | ? | ? | ? | ? | 1 | ? | 1 | 1 | 0 | 0 |
| 0 | 0 | ? | ? | 0 | 0 | 1 | 0 | 1 | 2 | 0 | ? | ? | ? | ? |
| ? | ? | ? | ? | ? | ? | ? | ? | ? | ? | ? | ? | ? | ? | ? |
| ? | ? | ? | ? | ? | ? | ? | ? | ? | ? | ? | ? | ? | ? | ? |
| ? | ? | ? | ? | ? | ? | 0 | 0 | 0 | 0 | 0 | 0 | ? | ? | 0 |
| 0 | 0 | ? | 0 | 0 | ? | ? | ? | ? | ? | ? | ? | 1 | ? | ? |
| 0 | ? | 2 | 0 | 0 | 1 | ? | 0 | 3 | | | | | | |

**Diadectomorpha**

| | | | | | | | | | | | | | | |
|---|---|---|---|---|---|---|---|---|---|---|---|---|---|---|
| 1 | 0 | 0 | 0 | 0 | 0 | 0 | 0 | 0 | 0 | 0 | 0 | 0 | 0 | 0 |
| 0 | 0 | 0 | 0 | 0 | 0 | 0 | 0 | 0 | 0 | 1 | 0 | 0 | 0 | 0 |
| 0 | 0 | 0 | 0 | 0 | 0 | 0 | 0 | 1 | 0 | 0 | 0 | 0 | 0 | 1 |
| 0 | 1 | 0 | 0 | 0 | [0 1] | 0 | 0 | 0 | 1 | 1 | 0 | 1 | 0 | 0 |
| 0 | 0 | 0 | 0 | 1 | 0 | 0 | 0 | 0 | 0 | 1 | 1 | 0 | 0 | 0 |
| 1 | 0 | 0 | 1 | 1 | [0 1] | 0 | 0 | 0 | [0 1] | 1 | 0 | 0 | 0 | 0 |
| ? | ? | ? | 0 | 0 | ? | ? | 0 | ? | 1 | 0 | 0 | 0 | 0 | 1 |
| 0 | [0 1] | 0 | [0 1] | 0 | 0 | 0 | 0 | 0 | 0 | 0 | 0 | 0 | 0 | 0 |
| ? | ? | 1 | 0 | ? | 0 | 0 | [0 1] | 0 | [0 1] | 0 | 0 | [0 1] | 0 | 0 |
| 0 | 0 | 0 | 0 | 0 | 0 | 0 | 1 | 0 | 0 | 0 | 0 | 0 | [0 1] | 0 |
| 0 | 0 | 0 | ? | 0 | 0 | [0 1] | [0 1] | 0 | 0 | 0 | 0 | 0 | 0 | 0 |
| 0 | 0 | 0 | 0 | 0 | 0 | 0 | 0 | 0 | 0 | 0 | 0 | 0 | 0 | 0 |
| 0 | 0 | 0 | 0 | 0 | 0 | 0 | 0 | 0 | 0 | 0 | 0 | 0 | 0 | 0 |
| 0 | 0 | 0 | 0 | 0 | 0 | 0 | 0 | 0 | 0 | 0 | 0 | 0 | 0 | 0 |
| 0 | [0 1] | 0 | 0 | 0 | 0 | 0 | 0 | 0 | 0 | 0 | 0 | 0 | 0 | 0 |
| 1 | [0 1] | 0 | 0 | 0 | 0 | 0 | 0 | 0 | 0 | 0 | 0 | 0 | 0 | 0 |
| 0 | 0 | 0 | ? | 0 | 0 | 0 | 0 | 0 | 0 | 0 | 0 | 0 | 0 | 0 |
| 0 | 0 | ? | 0 | 0 | ? | ? | 0 | ? | 0 | 0 | 0 | 0 | 0 | 0 |
| 0 | ? | 2 | 0 | 0 | 0 | ? | 0 | 0 | | | | | | |

***Emeroleter levis***

| | | | | | | | | | | | | | | |
|---|---|---|---|---|---|---|---|---|---|---|---|---|---|---|
| 2 | 0 | 0 | 0 | 0 | 1 | 0 | 0 | 0 | ? | 0 | 0 | 0 | 0 | 0 |
| ? | 0 | 0 | 0 | 0 | 1 | 0 | 0 | 0 | 0 | 1 | ? | ? | ? | 1 |
| 0 | 0 | 0 | 2 | 0 | 0 | 0 | 0 | 2 | 1 | 2 | 0 | 0 | 0 | 1 |
| 0 | 1 | 0 | 1 | 1 | 1 | 0 | 0 | 0 | 1 | 1 | 0 | 1 | 0 | 0 |
| 0 | 0 | 0 | ? | ? | 0 | 1 | 0 | 0 | 0 | ? | 1 | 1 | 0 | 1 |
| 0 | 1 | ? | ? | 1 | ? | ? | 0 | 0 | 1 | 1 | 1 | 0 | 0 | 0 |
| ? | ? | ? | 0 | ? | ? | ? | ? | ? | 1 | 1 | ? | ? | ? | 0 |
| 0 | 0 | 0 | 1 | ? | 0 | 0 | ? | 1 | 0 | 0 | ? | ? | ? | 1 |
| 0 | 0 | ? | 1 | 0 | ? | ? | 0 | ? | 0 | ? | 1 | 0 | 1 | ? |
| ? | ? | ? | ? | ? | ? | ? | ? | ? | 1 | ? | 1 | 2 | 1 | 1 |
| 0 | ? | ? | ? | 0 | ? | ? | ? | ? | 1 | ? | ? | 0 | ? | 0 |
| 1 | ? | 0 | ? | 1 | 0 | ? | 0 | 1 | 0 | 0 | ? | ? | 0 | ? |
| 1 | 0 | 0 | 2 | 0 | 0 | 1 | ? | ? | 0 | 0 | 1 | ? | 0 | 1 |
| 1 | ? | ? | ? | 0 | 0 | 1 | 1 | ? | 2 | 0 | 0 | 1 | ? | 0 |
| ? | 1 | ? | ? | ? | ? | 1 | ? | 0 | ? | ? | ? | 1 | ? | 0 |
| 1 | 1 | ? | ? | 0 | 0 | 1 | 0 | ? | 0 | 0 | 0 | 1 | 0 | 1 |
| 1 | 0 | 0 | ? | ? | ? | 0 | 0 | 0 | 0 | 0 | 0 | 0 | 0 | 0 |
| 0 | 0 | ? | ? | 0 | ? | ? | ? | ? | ? | 0 | ? | 0 | ? | ? |
| ? | ? | 2 | 0 | 0 | 1 | ? | 0 | 0 | | | | | | |

**Eosauropterygia**

| | | | | | | | | | | | | | | |
|---|---|---|---|---|---|---|---|---|---|---|---|---|---|---|
| [1 2] | [0 1] | 1 | 0 | 0 | 1 | 1 | 0 | 0 | [0 1] | 0 | 0 | 0 | [0 1] | [0 1] |
| ? | [0 1] | 0 | 0 | 0 | 1 | 1 | [0 1] | 0 | [0 1] | 0 | [0 1] | 0 | 0 | 0 |
| [0 1] | 0 | 0 | 2 | ? | 1 | ? | 0 | ? | 0 | ? | 0 | 0 | [0 1] | [0 1] |
| [0 1] | 1 | [0 1] | [0 1] | 0 | 0 | ? | 0 | 0 | 1 | [0 1] | [0 1] | 0 | 1 | 1 |
| 1 | [0 1 2 3] | 1 | 1 | ? | 0 | [0 1 2] | 1 | [0 1] | 1 | ? | ? | 2 | 1 | ? |
| [0 1] | 0 | 0 | [0 1] | 0 | 1 | 1 | [0 1] | 0 | [0 1] | 1 | 0 | 0 | [0 1] | 1 |
| [0 1] | [0 1] | 1 | 1 | 1 | 0 | 1 | 1 | 0 | [0 1] | 0 | 1 | [0 1] | [0 1] | 1 |
| [0 1] | 1 | 1 | ? | 1 | 0 | 1 | ? | 1 | 0 | [0 1] | 1 | 2 | 0 | 0 |
| ? | ? | 1 | [0 1] | 0 | 0 | [0 1] | 1 | 0 | 1 | 0 | 1 | 1 | ? | ? |
| ? | 1 | 0 | [0 1] | [0 1] | 1 | 1 | 1 | 0 | 1 | [0 1] | 1 | 1 | 1 | 1 |
| 1 | 1 | 0 | ? | [0 2] | 0 | 1 | 0 | 0 | 0 | [0 1] | 1 | 0 | [0 1] | [0 1] |
| [0 1] | 0 | 1 | 1 | [0 1] | 0 | 1 | 0 | 1 | 1 | 0 | 1 | [0 1] | 1 | ? |
| 1 | 1 | 1 | 1 | 1 | 0 | 1 | 0 | 1 | [0 1] | 1 | [0 1] | [0 1] | 0 | 2 |
| 0 | 1 | 0 | 1 | [0 1] | 1 | 1 | 1 | 1 | [0 1] | [0 1] | [1 2] | 1 | 1 | ? |
| 1 | ? | 0 | 0 | 1 | 0 | 1 | 1 | 0 | [1 2] | 1 | 1 | [0 1] | 1 | 0 |
| 1 | 0 | 0 | 0 | 1 | 1 | 0 | 0 | 0 | 0 | 2 | 0 | 1 | 0 | 0 |
| 0 | 0 | 0 | ? | ? | 0 | 0 | 0 | 0 | 0 | 0 | 0 | 0 | 0 | 0 |
| 0 | 0 | ? | 0 | 0 | 1 | ? | 0 | 0 | 0 | 1 | 0 | 0 | 0 | 0 |
| 0 | ? | 0 | 0 | 0 | ? | 0 | 0 | 0 | | | | | | |

***Eudibamus cursoris***

| | | | | | | | | | | | | | | |
|---|---|---|---|---|---|---|---|---|---|---|---|---|---|---|
| ? | ? | ? | ? | ? | 1 | ? | 0 | ? | ? | ? | ? | ? | ? | ? |
| ? | ? | ? | 0 | ? | 0 | ? | ? | ? | ? | ? | ? | ? | ? | ? |
| ? | ? | ? | ? | ? | ? | ? | 0 | 2 | 1 | ? | ? | ? | ? | ? |
| ? | 1 | 0 | ? | ? | 0 | ? | 0 | 0 | ? | ? | ? | 1 | 1 | 1 |
| 1 | ? | ? | ? | ? | 0 | ? | ? | ? | ? | ? | ? | 2 | ? | ? |
| 0 | ? | ? | ? | ? | 1 | ? | ? | 1 | ? | 0 | 0 | ? | 0 | 0 |
| ? | ? | ? | 1 | ? | 1 | 1 | ? | ? | ? | ? | ? | ? | ? | 1 |
| 1 | 2 | 1 | ? | 1 | 1 | ? | ? | 0 | 0 | 0 | 1 | ? | ? | ? |
| ? | ? | ? | ? | ? | ? | ? | 0 | ? | 0 | ? | ? | ? | ? | ? |
| ? | ? | ? | ? | ? | 0 | ? | ? | ? | 0 | ? | 0 | ? | 1 | 1 |
| ? | ? | ? | ? | 2 | 2 | ? | ? | ? | ? | ? | ? | 0 | 0 | ? |
| ? | 1 | 0 | ? | 1 | 0 | 0 | 0 | 1 | 0 | 0 | ? | ? | ? | ? |
| ? | ? | ? | 2 | ? | 0 | 0 | 0 | 0 | ? | ? | ? | 1 | 0 | 0 |
| 0 | ? | ? | ? | 1 | ? | 1 | ? | 1 | ? | 1 | ? | ? | ? | ? |
| ? | 0 | ? | ? | ? | 1 | 1 | ? | 1 | ? | ? | ? | 1 | ? | 0 |
| 1 | 0 | ? | ? | 0 | ? | 0 | 0 | ? | 1 | ? | 0 | 0 | 0 | 1 |
| 1 | ? | 0 | ? | ? | 1 | 0 | 0 | 0 | 0 | 0 | 0 | ? | 0 | 0 |
| 0 | 0 | ? | 0 | 0 | ? | ? | ? | ? | ? | ? | ? | ? | ? | ? |
| ? | ? | 2 | ? | ? | ? | 0 | 0 | ? | | | | | | |

***Feeserpeton oklahomensis***

| | | | | | | | | | | | | | | |
|---|---|---|---|---|---|---|---|---|---|---|---|---|---|---|
| 0 | 0 | 0 | ? | ? | 1 | 0 | ? | 0 | ? | 0 | ? | ? | ? | 0 |
| ? | 0 | 0 | ? | 0 | 1 | 0 | 0 | 0 | ? | 1 | ? | ? | ? | 1 |
| 0 | 0 | 0 | 0 | 0 | 0 | ? | 0 | 1 | 0 | 2 | 0 | 0 | 0 | 1 |
| 0 | 1 | 1 | 1 | 0 | 1 | 1 | ? | 0 | 1 | 1 | 0 | 1 | 0 | 0 |
| 0 | 0 | 0 | ? | 0 | 0 | 0 | 0 | 0 | 0 | 1 | 1 | 1 | 1 | ? |
| 0 | ? | ? | ? | 1 | 1 | 1 | 0 | 1 | 1 | 0 | 0 | 0 | ? | 0 |
| ? | ? | ? | 1 | 0 | 0 | 1 | 1 | 1 | 1 | ? | ? | ? | ? | 0 |
| 0 | 0 | 1 | 1 | ? | 1 | 0 | 0 | 0 | 0 | 0 | 1 | 0 | 0 | 0 |
| ? | ? | 0 | 1 | 0 | 0 | ? | 0 | ? | 0 | ? | 1 | 1 | 0 | 0 |
| 0 | ? | 1 | 0 | ? | 2 | 0 | ? | ? | 1 | ? | 1 | 1 | 0 | 1 |
| 0 | ? | ? | ? | 1 | ? | ? | 1 | 1 | 1 | 0 | ? | 0 | 1 | 0 |
| 0 | 1 | 0 | ? | 0 | 1 | ? | 0 | 1 | ? | ? | ? | ? | ? | ? |
| ? | ? | ? | ? | ? | ? | ? | ? | ? | ? | ? | ? | ? | ? | ? |
| ? | ? | ? | ? | ? | ? | ? | ? | ? | ? | ? | ? | ? | ? | ? |
| ? | ? | ? | ? | ? | ? | ? | ? | ? | ? | ? | ? | ? | ? | ? |
| ? | ? | ? | ? | ? | ? | ? | ? | ? | ? | ? | ? | ? | ? | ? |
| ? | ? | ? | ? | ? | ? | ? | ? | ? | ? | ? | ? | ? | ? | ? |
| ? | ? | ? | ? | ? | ? | ? | ? | ? | ? | 0 | ? | 0 | ? | ? |
| ? | ? | ? | 0 | 0 | 1 | 0 | 0 | 3 | | | | | | |

***Hovasaurus boulei***

| | | | | | | | | | | | | | | |
|---|---|---|---|---|---|---|---|---|---|---|---|---|---|---|
| ? | ? | ? | ? | ? | ? | ? | ? | ? | ? | ? | ? | ? | ? | ? |
| ? | ? | ? | ? | ? | ? | ? | ? | ? | ? | ? | ? | ? | ? | ? |
| ? | 0 | ? | ? | ? | ? | ? | ? | ? | ? | ? | 1 | 0 | 1 | 1 |
| 0 | 1 | 0 | 1 | ? | 0 | ? | 0 | 0 | 1 | 0 | 0 | 0 | 0 | 1 |
| 0 | ? | ? | ? | ? | 0 | 0 | 1 | 0 | ? | ? | ? | 1 | 0 | ? |
| 0 | 0 | 2 | 0 | 1 | ? | 1 | ? | ? | ? | ? | ? | 0 | 1 | 1 |
| 0 | 0 | 1 | 0 | ? | 0 | ? | 1 | ? | 1 | ? | ? | ? | ? | 0 |
| ? | ? | ? | ? | ? | 0 | 1 | 1 | 1 | 0 | ? | ? | ? | ? | 1 |
| ? | ? | 0 | ? | ? | ? | 0 | 1 | ? | ? | 0 | 0 | 1 | ? | 0 |
| 1 | 1 | ? | 1 | ? | ? | 0 | 1 | 0 | 1 | ? | 0 | 0 | 0 | 1 |
| 0 | ? | ? | ? | 1 | ? | 1 | ? | ? | ? | ? | ? | ? | ? | ? |
| ? | ? | ? | ? | ? | ? | ? | 0 | 1 | 0 | 0 | 0 | 0 | 0 | ? |
| 1 | 0 | 0 | 1 | 0 | 0 | 0 | 1 | 1 | 0 | 0 | 1 | 1 | 1 | 0 |
| ? | 1 | 0 | 0 | 0 | 0 | 0 | 1 | ? | 2 | 0 | 2 | 1 | 0 | 1 |
| 0 | 0 | 0 | 0 | 0 | 1 | 1 | 0 | ? | 0 | 1 | ? | 1 | 0 | 1 |
| 1 | 0 | 0 | 0 | 0 | [0 1] | 0 | 0 | 0 | 1 | 1 | 0 | 1 | 0 | 0 |
| 1 | ? | 0 | ? | ? | 0 | 0 | 0 | 0 | 0 | 0 | 0 | ? | 0 | 0 |
| 0 | 0 | ? | 0 | 0 | ? | ? | ? | ? | 0 | 1 | ? | ? | 0 | 0 |
| 0 | ? | 2 | ? | ? | 0 | 0 | ? | 3 | | | | | | |

**Kuehneosauridae**

| | | | | | | | | | | | | | | |
|---|---|---|---|---|---|---|---|---|---|---|---|---|---|---|
| 1 | 0 | 0 | 0 | 0 | 1 | 0 | 1 | 0 | ? | 0 | 0 | 0 | 0 | 0 |
| ? | 0 | 0 | 0 | 0 | 1 | 0 | 0 | 1 | 1 | ? | 1 | 1 | ? | 0 |
| 0 | 0 | 0 | 1 | 1 | 1 | 1 | 0 | ? | 0 | ? | [0 1] | 0 | 0 | 0 |
| 0 | 1 | 0 | 1 | 0 | 0 | ? | 0 | 0 | 1 | 1 | 1 | 0 | 1 | 1 |
| 1 | 1 | 1 | ? | 0 | 0 | 1 | 1 | 0 | 1 | ? | ? | 2 | 1 | ? |
| 0 | 0 | 0 | 1 | 2 | 1 | 0 | 1 | ? | ? | ? | ? | 0 | 1 | 1 |
| 0 | 0 | 1 | 1 | 1 | 0 | ? | 1 | 0 | ? | ? | ? | ? | ? | [0 1] |
| ? | ? | 0 | 0 | ? | ? | 1 | 1 | 1 | 0 | ? | ? | ? | 0 | 1 |
| 1 | ? | [0 1] | ? | ? | ? | 1 | 1 | 1 | 1 | 0 | 1 | 1 | ? | ? |
| ? | ? | ? | 0 | ? | 0 | 1 | 1 | ? | 1 | 1 | 1 | 0 | 0 | 1 |
| 1 | ? | 0 | ? | 0 | 0 | 1 | 0 | ? | 0 | ? | ? | ? | ? | 0 |
| 1 | ? | 1 | 1 | 1 | 0 | 0 | 0 | 1 | 0 | 0 | 1 | 0 | 0 | ? |
| 1 | 1 | ? | 1 | 1 | 1 | 0 | ? | 1 | 0 | ? | ? | ? | ? | 0 |
| 0 | 1 | 0 | 0 | 0 | 0 | 1 | 1 | 1 | 0 | 1 | 2 | ? | 1 | 1 |
| 1 | ? | 0 | 0 | 1 | 0 | 1 | ? | 0 | 1 | 0 | 1 | ? | ? | ? |
| 1 | 0 | ? | ? | ? | ? | ? | ? | 0 | ? | 1 | 0 | 1 | 0 | 1 |
| ? | 0 | 0 | ? | 0 | 1 | 0 | 0 | 0 | 0 | 0 | 0 | 0 | 0 | 0 |
| 0 | 0 | ? | 0 | 0 | ? | ? | 0 | ? | 0 | 1 | ? | 1 | ? | 0 |
| 0 | ? | 2 | 0 | 0 | ? | [0 1] | ? | 3 | | | | | | |

***Lanthanosuchus watsoni***

| | | | | | | | | | | | | | | |
|---|---|---|---|---|---|---|---|---|---|---|---|---|---|---|
| ? | ? | ? | ? | ? | 1 | ? | ? | ? | ? | ? | ? | ? | ? | ? |
| ? | ? | ? | 0 | ? | ? | 0 | ? | ? | ? | 0 | ? | ? | ? | 1 |
| ? | ? | ? | ? | ? | ? | ? | ? | 1 | 0 | ? | ? | ? | ? | ? |
| ? | ? | ? | ? | 0 | 1 | 1 | 0 | ? | ? | ? | ? | ? | ? | 0 |
| ? | ? | ? | ? | ? | ? | ? | ? | ? | ? | ? | ? | 1 | ? | ? |
| ? | 1 | ? | ? | ? | 1 | ? | ? | 0 | ? | 1 | 1 | 0 | ? | 0 |
| ? | ? | ? | 1 | 0 | 1 | 1 | 1 | 0 | 1 | 0 | ? | ? | 0 | ? |
| 0 | 0 | 0 | ? | ? | 1 | 0 | ? | 1 | 1 | 0 | 1 | ? | 0 | 0 |
| ? | ? | ? | ? | ? | 0 | ? | 0 | ? | 0 | ? | 1 | 1 | ? | ? |
| ? | ? | 1 | 1 | ? | 2 | ? | ? | ? | 0 | ? | 1 | 1 | 0 | 1 |
| ? | ? | ? | ? | 2 | ? | ? | ? | ? | ? | ? | ? | ? | ? | ? |
| ? | ? | ? | ? | 1 | 0 | ? | 0 | 1 | ? | ? | ? | ? | ? | ? |
| ? | ? | ? | ? | ? | ? | ? | ? | ? | ? | ? | ? | 1 | ? | 0 |
| ? | ? | ? | ? | ? | ? | ? | ? | ? | ? | ? | ? | ? | ? | ? |
| ? | ? | ? | ? | ? | ? | ? | ? | ? | ? | ? | ? | ? | ? | ? |
| ? | ? | ? | ? | ? | ? | ? | ? | ? | ? | ? | ? | ? | ? | ? |
| ? | ? | ? | ? | ? | ? | ? | ? | ? | ? | ? | ? | ? | ? | ? |
| ? | ? | ? | ? | ? | ? | ? | ? | ? | ? | 0 | 0 | 0 | 0 | 0 |
| ? | ? | ? | ? | 0 | 1 | 0 | 0 | ? | | | | | | |

***Macroleter poezicus***

| | | | | | | | | | | | | | | |
|---|---|---|---|---|---|---|---|---|---|---|---|---|---|---|
| 1 | 0 | 0 | 0 | 0 | 1 | 0 | 0 | 0 | 1 | 0 | 0 | 0 | 0 | 0 |
| 0 | 0 | 0 | 0 | 0 | 1 | 0 | 0 | 0 | 0 | 1 | 1 | 0 | 0 | 1 |
| 0 | 0 | 0 | 1 | 0 | ? | 0 | 0 | 2 | 1 | 2 | 0 | 0 | 0 | 0 |
| 0 | 1 | 0 | 1 | 1 | 1 | 1 | 1 | 0 | 1 | 1 | 0 | 1 | 0 | 0 |
| 0 | 0 | 0 | ? | 0 | 0 | 2 | 0 | 0 | 0 | 1 | 1 | 1 | 0 | 1 |
| 1 | 1 | ? | ? | 0 | 0 | 1 | 0 | 0 | 0 | 1 | 0 | 1 | 0 | 0 |
| ? | ? | ? | 1 | 0 | 1 | 1 | 1 | 1 | 1 | 1 | 0 | 0 | 0 | 0 |
| 0 | 0 | 0 | 1 | 0 | 1 | 0 | 1 | 1 | 0 | 0 | 1 | 1 | 1 | 1 |
| 0 | 1 | 0 | 1 | 0 | 1 | 1 | 0 | 0 | 0 | 0 | ? | 0 | 0 | 2 |
| 1 | 0 | 1 | 1 | 1 | 1 | 1 | 0 | 1 | 1 | 0 | 0 | 2 | 1 | 1 |
| 0 | ? | 0 | ? | 0 | ? | 1 | 1 | 1 | 1 | 0 | 0 | 0 | 1 | 0 |
| 0 | 1 | 0 | ? | 0 | 0 | 0 | 0 | 1 | 0 | 0 | 0 | 0 | 0 | 0 |
| 0 | 0 | 0 | 2 | 0 | 0 | 1 | ? | 0 | ? | 0 | 0 | 1 | 0 | 1 |
| 0 | ? | 1 | 0 | 0 | 0 | 0 | 0 | 1 | 0 | 0 | 0 | 1 | 0 | 0 |
| 0 | 1 | 0 | 0 | 0 | 1 | 1 | 0 | 0 | 0 | 0 | 1 | ? | 0 | 0 |
| 1 | 1 | 0 | 0 | 0 | 1 | 0 | 0 | 0 | 0 | 1 | 0 | 0 | 0 | 1 |
| 0 | 0 | 0 | ? | 0 | ? | 0 | 0 | 0 | 0 | 0 | 0 | 1 | ? | ? |
| ? | ? | ? | ? | ? | ? | ? | ? | 0 | ? | 0 | 0 | 0 | 0 | 0 |
| 0 | ? | 2 | 0 | 0 | 1 | 0 | 0 | 0 | | | | | | |

***Mesosaurus* spp.**

| | | | | | | | | | | | | | | |
|---|---|---|---|---|---|---|---|---|---|---|---|---|---|---|
| 1 | 0 | 1 | 0 | 0 | 0 | 1 | 0 | 1 | 1 | 0 | 0 | 0 | 1 | 0 |
| 0 | 0 | 0 | 0 | 1 | 0 | 0 | 0 | 1 | 0 | 1 | 1 | ? | 0 | 0 |
| 0 | 0 | 0 | 1 | 0 | 0 | 0 | 0 | 0 | 0 | ? | 1 | 0 | 0 | 1 |
| 0 | 1 | 0 | 1 | 0 | 0 | ? | 0 | 0 | 1 | 1 | 0 | ? | 0 | 0 |
| 0 | 0 | 0 | ? | 0 | 0 | 0 | 1 | 0 | 0 | 0 | 1 | 1 | 0 | 1 |
| 0 | 1 | ? | ? | 0 | 1 | 1 | 0 | 1 | 0 | 0 | 0 | 0 | 1 | 0 |
| ? | ? | ? | [0 1] | 1 | 0 | 1 | 1 | 1 | 1 | 0 | 0 | 0 | 0 | 0 |
| 0 | 0 | 1 | ? | 1 | 0 | 1 | 0 | 0 | 0 | 1 | ? | ? | 0 | ? |
| ? | ? | 0 | 1 | 0 | 0 | ? | 0 | 0 | 0 | ? | 0 | 1 | ? | 0 |
| 0 | 0 | ? | 0 | ? | 0 | 0 | ? | 0 | 1 | 0 | 0 | 0 | 1 | 0 |
| 0 | ? | 0 | ? | 1 | 0 | 1 | 0 | 0 | 0 | 0 | 0 | 0 | 0 | 1 |
| 0 | 0 | 1 | 0 | 1 | 0 | 0 | 0 | 1 | 0 | 0 | 1 | 0 | 0 | ? |
| [0 1] | 0 | 0 | 0 | 1 | 0 | 0 | 0 | 0 | 0 | 0 | 0 | 0 | 0 | 0 |
| 0 | 0 | 0 | 0 | 1 | 0 | 1 | 1 | 1 | 2 | 0 | 1 | 1 | 0 | 1 |
| 0 | 0 | 0 | 0 | 0 | 0 | 1 | 1 | 0 | 1 | 1 | 1 | 0 | 0 | 0 |
| 1 | 0 | 0 | 0 | 0 | 0 | 1 | 0 | 0 | 0 | 1 | 0 | 0 | 0 | 0 |
| 0 | 0 | 0 | ? | 0 | 0 | 0 | 0 | 0 | 0 | 0 | 0 | 0 | 0 | 0 |
| 0 | 0 | ? | 0 | 0 | 0 | ? | 0 | ? | 0 | 0 | 0 | 0 | ? | 0 |
| 0 | ? | 0 | 0 | 0 | [0 1] | ? | 0 | 0 | | | | | | |

***Microleter mckinzieorum***

| | | | | | | | | | | | | | | |
|---|---|---|---|---|---|---|---|---|---|---|---|---|---|---|
| 1 | 0 | 0 | ? | ? | ? | 0 | 0 | 0 | ? | 0 | ? | 0 | ? | 0 |
| 0 | 0 | 0 | 1 | 0 | 0 | 0 | 0 | 0 | 1 | 0 | ? | ? | ? | 1 |
| 0 | 0 | 0 | 0 | 0 | ? | 1 | 0 | 1 | 1 | ? | 0 | 0 | 0 | 1 |
| 0 | 1 | 0 | 0 | 0 | 1 | 2 | 0 | 0 | 1 | 1 | 1 | 0 | 0 | 0 |
| 0 | 0 | 0 | ? | ? | 0 | 0 | 0 | 0 | 0 | 1 | 1 | 1 | ? | ? |
| 0 | 1 | ? | ? | 1 | ? | 1 | 0 | 1 | 1 | 1 | 0 | 0 | 1 | 0 |
| ? | ? | ? | 1 | 1 | 0 | 1 | ? | ? | ? | ? | ? | ? | ? | ? |
| 0 | 1 | ? | ? | ? | 1 | 0 | 0 | 0 | 0 | ? | ? | ? | 0 | ? |
| ? | ? | ? | ? | ? | ? | ? | 0 | 0 | 0 | ? | 0 | 0 | 0 | ? |
| ? | ? | ? | ? | ? | 0 | ? | ? | ? | ? | ? | ? | ? | ? | ? |
| ? | ? | ? | ? | 1 | 0 | ? | 0 | 0 | 1 | 0 | ? | 0 | ? | ? |
| 0 | 0 | 0 | ? | 1 | 0 | 0 | 0 | 1 | ? | ? | ? | ? | ? | ? |
| ? | ? | ? | ? | ? | ? | ? | ? | ? | ? | ? | ? | ? | ? | ? |
| ? | ? | ? | ? | ? | ? | ? | ? | ? | ? | ? | ? | ? | ? | ? |
| ? | ? | ? | ? | ? | ? | ? | ? | ? | ? | ? | ? | ? | ? | ? |
| ? | ? | ? | ? | ? | ? | ? | ? | ? | ? | ? | ? | ? | ? | ? |
| ? | ? | ? | ? | ? | ? | ? | ? | ? | ? | ? | ? | ? | ? | ? |
| ? | ? | ? | ? | ? | ? | ? | ? | ? | ? | 0 | ? | 0 | ? | ? |
| ? | ? | ? | 0 | 0 | 1 | 1 | 0 | 1 | | | | | | |

**Millerettidae**

| | | | | | | | | | | | | | | |
|---|---|---|---|---|---|---|---|---|---|---|---|---|---|---|
| 0 | 0 | 0 | 0 | 0 | 0 | 0 | 0 | 0 | 0 | 0 | 0 | 0 | 0 | 0 |
| 0 | 0 | 0 | 0 | 0 | 0 | 0 | 0 | 0 | 1 | 0 | 0 | 0 | 0 | 1 |
| 0 | 0 | 0 | 0 | 0 | 0 | 0 | 0 | 1 | 0 | 2 | 0 | 0 | 0 | 0 |
| 0 | 1 | 0 | 0 | 0 | 1 | 0 | 0 | 0 | 1 | 1 | 1 | 0 | 0 | 0 |
| 0 | 0 | 0 | 0 | 0 | 0 | 2 | 0 | 0 | 0 | 0 | 1 | 1 | 0 | 1 |
| 0 | 0 | 0 | 0 | 0 | 0 | 1 | 0 | 0 | 2 | 1 | 0 | 0 | 0 | 0 |
| ? | ? | ? | [0 1] | 0 | 0 | 0 | 1 | 1 | 1 | 0 | 0 | 0 | 0 | 0 |
| 0 | 0 | 0 | 1 | 0 | 0 | 0 | 0 | 0 | 0 | 0 | 1 | 1 | 0 | 1 |
| 0 | 1 | 0 | 1 | 1 | 0 | 0 | 1 | 0 | 0 | 0 | 0 | 1 | ? | 0 |
| 1 | 0 | 0 | 0 | 0 | 0 | 0 | 1 | 0 | 1 | 1 | 1 | 0 | 0 | 1 |
| 0 | 0 | 0 | ? | 1 | 0 | 1 | 0 | 0 | 0 | 0 | 0 | 0 | 0 | 0 |
| 0 | 0 | 0 | 0 | [0 1] | 0 | 0 | 0 | 1 | 0 | 0 | 0 | 0 | 0 | ? |
| 0 | 0 | 0 | 1 | 0 | 0 | 0 | ? | 0 | ? | 0 | 0 | 0 | 0 | 0 |
| 0 | ? | 0 | 0 | ? | 0 | 1 | 1 | 1 | ? | 0 | 0 | ? | 0 | ? |
| 0 | 1 | 0 | 0 | 0 | 0 | 1 | 0 | 0 | 0 | ? | ? | 0 | 0 | 0 |
| 1 | 0 | 0 | 0 | 0 | 1 | 0 | 0 | 0 | 1 | 1 | 0 | ? | 0 | 0 |
| 1 | 0 | 0 | ? | 0 | 0 | 0 | 0 | 1 | 0 | 0 | 0 | 1 | 1 | ? |
| 0 | 0 | ? | 0 | 0 | 2 | ? | 0 | 0 | 0 | 0 | 0 | 0 | 0 | 0 |
| 0 | 0 | 2 | 0 | 0 | [0 1] | 0 | 0 | [0 3] | | | | | | |

***Nycteroleter ineptus***

| | | | | | | | | | | | | | | |
|---|---|---|---|---|---|---|---|---|---|---|---|---|---|---|
| ? | ? | ? | ? | ? | 1 | ? | 0 | ? | ? | ? | ? | ? | ? | ? |
| ? | ? | ? | 0 | ? | 1 | 0 | ? | ? | ? | 0 | ? | ? | ? | 1 |
| ? | ? | ? | 2 | ? | ? | ? | 0 | 2 | 1 | 2 | ? | ? | ? | ? |
| ? | 1 | 0 | ? | 1 | 1 | 1 | 0 | ? | ? | ? | ? | 1 | 0 | 0 |
| 0 | ? | ? | ? | ? | 0 | 0 | 0 | ? | 0 | 0 | 1 | 1 | 0 | 1 |
| 0 | 0 | ? | ? | ? | 0 | ? | ? | 0 | ? | 1 | ? | 1 | ? | 0 |
| ? | ? | ? | 0 | ? | ? | ? | ? | ? | 1 | 1 | ? | ? | 0 | ? |
| 0 | 0 | 0 | ? | ? | 1 | 0 | ? | 1 | 0 | ? | ? | ? | ? | 1 |
| 0 | ? | ? | ? | ? | ? | ? | 0 | ? | 0 | ? | 1 | 0 | 1 | ? |
| ? | ? | ? | ? | ? | ? | ? | ? | ? | ? | ? | 1 | 2 | 1 | 1 |
| 0 | ? | ? | ? | 0 | ? | ? | ? | 1 | 1 | ? | ? | 0 | 1 | ? |
| ? | ? | 0 | ? | 1 | 0 | ? | 0 | 1 | ? | ? | ? | ? | ? | ? |
| ? | ? | ? | ? | ? | ? | ? | ? | ? | ? | ? | ? | ? | ? | ? |
| ? | ? | ? | ? | ? | ? | ? | ? | ? | ? | ? | ? | ? | ? | ? |
| ? | ? | ? | ? | ? | ? | ? | ? | ? | ? | ? | ? | ? | ? | ? |
| ? | ? | ? | ? | ? | ? | ? | ? | ? | ? | ? | ? | ? | ? | ? |
| ? | ? | ? | ? | ? | ? | ? | ? | ? | ? | ? | ? | ? | ? | ? |
| ? | ? | ? | ? | ? | ? | ? | ? | ? | ? | 0 | ? | 0 | ? | ? |
| ? | ? | ? | 0 | 0 | 1 | ? | 0 | 3 | | | | | | |

***Nyctiphruretus acudens***

| | | | | | | | | | | | | | | |
|---|---|---|---|---|---|---|---|---|---|---|---|---|---|---|
| 1 | 0 | 0 | 0 | 0 | 1 | 0 | 0 | 0 | 0 | 0 | 0 | 0 | 0 | 0 |
| 0 | 0 | 0 | 0 | 0 | 1 | 1 | 0 | 0 | 0 | 1 | 0 | 0 | 0 | 1 |
| 0 | 0 | 0 | 2 | 0 | ? | 0 | 0 | 2 | 1 | 2 | 1 | 0 | 0 | 0 |
| 0 | 1 | 0 | 0 | 1 | 1 | 1 | 0 | 0 | 1 | 1 | 0 | 1 | 0 | 0 |
| 0 | 0 | 0 | ? | ? | 0 | 2 | 0 | 0 | 0 | 1 | 1 | 1 | 0 | 1 |
| 0 | 1 | ? | ? | 0 | 0 | 0 | 0 | 1 | 2 | 0 | 0 | 0 | 0 | 0 |
| ? | ? | ? | 1 | 1 | 1 | 1 | 1 | 1 | 1 | 0 | 0 | ? | 0 | 0 |
| 0 | 0 | 0 | 1 | 0 | 0 | 0 | 1 | 0 | 0 | 0 | 1 | 1 | 1 | 1 |
| 0 | 0 | 0 | 1 | 0 | 1 | 0 | 0 | ? | 0 | ? | 1 | 1 | ? | ? |
| ? | ? | 1 | 0 | ? | 0 | 0 | ? | 0 | 1 | ? | 1 | 1 | 1 | 1 |
| 0 | ? | 0 | ? | 1 | 0 | 1 | ? | 1 | 1 | 0 | ? | 0 | 1 | 0 |
| 0 | 0 | 1 | 0 | 1 | 0 | 0 | 0 | 1 | 0 | ? | ? | ? | ? | ? |
| ? | ? | ? | 2 | ? | ? | 1 | ? | ? | ? | ? | ? | 1 | 0 | 1 |
| 0 | ? | ? | ? | ? | ? | 0 | ? | 1 | ? | 0 | ? | 0 | ? | ? |
| ? | 1 | ? | ? | ? | 0 | 1 | 0 | 0 | ? | ? | ? | ? | ? | 1 |
| 1 | 0 | ? | ? | ? | ? | ? | ? | ? | 0 | 0 | ? | 0 | ? | ? |
| 0 | ? | 0 | ? | ? | ? | ? | 0 | ? | ? | ? | ? | 1 | 0 | ? |
| ? | ? | ? | ? | ? | ? | ? | ? | ? | ? | 0 | 1 | 0 | ? | 0 |
| 0 | ? | 1 | 0 | 0 | 1 | 1 | 0 | 3 | | | | | | |

***Orovenator mayorum***

| | | | | | | | | | | | | | | |
|---|---|---|---|---|---|---|---|---|---|---|---|---|---|---|
| 1 | 0 | 0 | 0 | 0 | 1 | 0 | 0 | 1 | ? | 0 | 0 | 0 | 0 | 0 |
| 0 | 0 | 0 | 0 | 0 | 1 | 0 | 0 | 0 | 1 | 0 | ? | ? | ? | 0 |
| 0 | 0 | 0 | 0 | 0 | 0 | 1 | 0 | ? | ? | ? | 0 | 0 | 0 | 1 |
| 0 | 1 | 0 | 0 | 0 | 1 | 0 | 0 | 0 | ? | ? | ? | ? | ? | ? |
| ? | 0 | 1 | ? | ? | ? | ? | ? | 0 | ? | ? | ? | 2 | ? | ? |
| 0 | 0 | 0 | 1 | ? | ? | ? | ? | ? | ? | ? | ? | 0 | ? | 1 |
| 0 | 0 | 0 | 1 | 0 | 0 | ? | ? | ? | 1 | 0 | ? | ? | ? | 0 |
| 0 | 0 | 0 | 1 | ? | ? | 1 | 1 | 0 | ? | 0 | 1 | ? | 0 | 1 |
| 1 | 0 | 0 | ? | ? | 0 | 0 | ? | 0 | ? | ? | ? | ? | ? | 0 |
| 0 | 1 | ? | ? | ? | ? | 1 | ? | ? | ? | ? | 1 | 0 | 0 | ? |
| ? | ? | ? | ? | ? | 0 | 1 | ? | ? | 0 | 1 | ? | 0 | ? | ? |
| 1 | ? | 0 | ? | 1 | 0 | ? | 0 | 1 | ? | ? | 0 | 0 | ? | ? |
| 1 | ? | ? | ? | ? | ? | ? | ? | ? | ? | ? | ? | ? | ? | ? |
| ? | ? | ? | ? | ? | ? | ? | ? | ? | ? | ? | ? | ? | ? | ? |
| ? | ? | ? | ? | ? | ? | ? | ? | ? | ? | ? | ? | ? | ? | ? |
| ? | ? | ? | ? | ? | ? | ? | ? | ? | ? | ? | ? | ? | ? | ? |
| ? | ? | ? | ? | ? | ? | ? | ? | ? | ? | ? | ? | ? | ? | ? |
| ? | ? | ? | ? | ? | ? | ? | ? | ? | ? | ? | ? | 0 | ? | ? |
| ? | ? | ? | 0 | 0 | ? | 0 | ? | 1 | | | | | | |

***Owenetta* spp.**

| | | | | | | | | | | | | | | |
|---|---|---|---|---|---|---|---|---|---|---|---|---|---|---|
| 0 | 0 | 0 | 0 | 0 | 1 | 0 | 0 | 0 | 0 | 0 | 0 | 0 | 0 | 0 |
| 0 | 0 | 0 | 1 | 0 | 1 | 0 | 0 | 1 | 0 | 0 | 0 | ? | 0 | 1 |
| 0 | 0 | 0 | 1 | 0 | 0 | 0 | 1 | 2 | 1 | ? | 0 | 0 | 0 | 0 |
| 0 | 1 | 0 | 1 | 1 | 0 | ? | 0 | 0 | 1 | 0 | 0 | 1 | 0 | 0 |
| 0 | 0 | 0 | 0 | 0 | 0 | 1 | 0 | 0 | 0 | 0 | 0 | 1 | 1 | ? |
| 1 | 1 | ? | ? | 0 | 0 | 1 | 0 | 0 | 1 | 0 | 0 | 0 | 0 | 0 |
| ? | ? | ? | 1 | 1 | 1 | 1 | 1 | 1 | 1 | 0 | 0 | 1 | 1 | 0 |
| 0 | 1 | 1 | ? | 0 | 1 | 0 | 1 | 0 | 0 | 0 | 1 | 1 | 0 | 1 |
| 0 | 1 | 0 | 1 | 0 | 0 | 1 | 0 | 0 | 0 | 0 | 0 | 0 | 0 | ? |
| ? | ? | 1 | 1 | ? | 0 | 0 | 0 | 0 | 1 | ? | 1 | 1 | 1 | 1 |
| 1 | ? | 0 | ? | 1 | 1 | 1 | 1 | 1 | 1 | 0 | 0 | 0 | 1 | 0 |
| 0 | 1 | 1 | 0 | 0 | 0 | 0 | 0 | 1 | 0 | ? | 0 | 0 | 0 | ? |
| 0 | 0 | 0 | 2 | 0 | 0 | 1 | ? | 0 | ? | ? | 0 | 1 | 0 | ? |
| 0 | ? | 1 | 0 | 0 | 0 | 0 | ? | 1 | 0 | 1 | 0 | 1 | ? | ? |
| 0 | 1 | 0 | 0 | ? | 0 | 0 | 0 | 0 | ? | ? | ? | 0 | ? | 0 |
| 1 | ? | 0 | ? | ? | ? | ? | ? | 0 | ? | ? | ? | ? | ? | 1 |
| 0 | ? | 0 | ? | 0 | 0 | 0 | 0 | 0 | 0 | 0 | 0 | 1 | 0 | 0 |
| 0 | 0 | ? | 0 | 0 | ? | ? | 0 | ? | 0 | 0 | 0 | 0 | 0 | 0 |
| 0 | ? | 2 | 1 | 0 | 1 | 1 | 0 | 3 | | | | | | |

***Paleothyris acadiana***

| | | | | | | | | | | | | | | |
|---|---|---|---|---|---|---|---|---|---|---|---|---|---|---|
| 0 | 0 | 0 | 0 | 0 | 0 | 0 | 0 | 0 | 0 | 0 | 0 | 0 | 0 | 0 |
| ? | 0 | 0 | 0 | 0 | 0 | 0 | 0 | 0 | 0 | 1 | 0 | ? | 0 | 0 |
| 0 | 0 | 0 | 0 | 0 | ? | 0 | 0 | 0 | 0 | ? | 0 | 0 | 0 | 0 |
| 0 | 1 | 0 | 0 | 0 | 0 | ? | 0 | 0 | 1 | 1 | 0 | ? | 0 | 1 |
| 1 | 0 | 0 | ? | 0 | 0 | 2 | 1 | 0 | 0 | 0 | 1 | 2 | 0 | 1 |
| 0 | 0 | 0 | 0 | 0 | 1 | 1 | 0 | 0 | 0 | 1 | 0 | 0 | 0 | 0 |
| ? | ? | ? | 0 | ? | ? | ? | 1 | 1 | 1 | 0 | 0 | 1 | 0 | 0 |
| 0 | 0 | 0 | 1 | 0 | 0 | 1 | 0 | 0 | 0 | ? | 0 | ? | 0 | ? |
| ? | ? | 0 | 0 | ? | ? | ? | 0 | 0 | 1 | ? | 0 | 1 | ? | 0 |
| 0 | ? | 0 | 0 | ? | 0 | 0 | 1 | ? | 1 | ? | 0 | 0 | 0 | 1 |
| 0 | 0 | 0 | ? | 0 | 0 | 1 | ? | 0 | 0 | 0 | ? | 0 | 0 | 0 |
| 0 | ? | 0 | 0 | 0 | 1 | 0 | 0 | 1 | 0 | 0 | 0 | 0 | 0 | 0 |
| 1 | 0 | 0 | 1 | 0 | 0 | 0 | 0 | 0 | 0 | 0 | 0 | 0 | 0 | 0 |
| 0 | 0 | 1 | 0 | 0 | 0 | 1 | 0 | 1 | 0 | 0 | 0 | 0 | 0 | 0 |
| 0 | 0 | 0 | 0 | 0 | 0 | 1 | 0 | 0 | 0 | 0 | 0 | 0 | 0 | 0 |
| 1 | 0 | 0 | 0 | 0 | 0 | 0 | 0 | 0 | 0 | 1 | 0 | 1 | 0 | 1 |
| 1 | 0 | 0 | ? | 0 | 0 | 0 | 0 | 0 | 0 | 0 | 0 | 0 | 0 | 0 |
| 0 | 0 | ? | 0 | 0 | ? | ? | 0 | 0 | 0 | 0 | 0 | 0 | 0 | 0 |
| 0 | ? | 2 | 0 | 0 | 0 | ? | 0 | 3 | | | | | | |

***Placodus* spp.**

| | | | | | | | | | | | | | | |
|---|---|---|---|---|---|---|---|---|---|---|---|---|---|---|
| 2 | 1 | 1 | 0 | 0 | 1 | 1 | 0 | 0 | 1 | 0 | 0 | 1 | 1 | 0 |
| ? | 0 | 1 | 0 | 1 | 1 | 1 | 0 | 0 | 0 | ? | 1 | 0 | 0 | 1 |
| 0 | 1 | 0 | 2 | ? | 1 | ? | 0 | ? | 0 | ? | 0 | 1 | 1 | 0 |
| 0 | 0 | 0 | 1 | 0 | 0 | ? | 0 | 0 | 1 | 0 | 0 | 1 | 1 | 1 |
| 1 | 1 | 1 | 1 | ? | 0 | 1 | 1 | [0 1] | 1 | ? | ? | 2 | 1 | ? |
| 1 | 0 | 0 | 0 | 0 | 1 | 1 | 0 | 0 | 1 | 1 | 0 | 0 | 0 | 1 |
| 1 | 0 | 1 | 0 | ? | ? | ? | 1 | 0 | 1 | ? | 1 | 2 | 1 | 1 |
| 0 | 1 | 1 | ? | 0 | 0 | 1 | 1 | 1 | 0 | 0 | 1 | 2 | 1 | 1 |
| 0 | [0 1] | 0 | 0 | ? | 0 | 1 | 1 | 0 | 1 | 0 | 1 | 1 | ? | ? |
| ? | 1 | 0 | 0 | 1 | 0 | 1 | 1 | ? | 1 | ? | 1 | 1 | 1 | 1 |
| 1 | 1 | 0 | ? | 0 | 1 | 1 | 1 | 1 | 1 | 0 | ? | 0 | 0 | 1 |
| 0 | 1 | 1 | 0 | 1 | 0 | 1 | ? | 1 | 0 | 0 | 0 | 0 | 0 | ? |
| 1 | 1 | 1 | 1 | 1 | 1 | 1 | ? | 1 | 1 | 1 | 1 | 1 | 0 | 0 |
| 0 | 1 | 0 | 1 | 1 | 1 | 1 | 1 | 1 | 0 | 1 | 2 | 1 | ? | ? |
| 1 | ? | 0 | 0 | 1 | 0 | 1 | 1 | 0 | 1 | 1 | 1 | 1 | ? | 0 |
| 1 | 0 | 0 | 0 | 1 | 1 | 0 | 0 | 0 | 0 | ? | 0 | 1 | 0 | 0 |
| 0 | 0 | 1 | 0 | ? | 0 | 0 | 0 | 0 | 0 | 0 | 1 | 0 | 0 | 0 |
| 0 | 0 | ? | 0 | 0 | 1 | 1 | 0 | 0 | 1 | 1 | 0 | 0 | 0 | 0 |
| 0 | ? | 1 | 0 | 0 | ? | 0 | 0 | [0 1] | | | | | | |

***Procolophon* spp.**

| | | | | | | | | | | | | | | |
|---|---|---|---|---|---|---|---|---|---|---|---|---|---|---|
| 0 | 0 | 0 | 0 | 0 | 1 | 0 | 0 | 0 | 0 | 0 | 0 | 0 | 0 | 0 |
| 0 | 0 | 0 | 1 | 0 | 1 | 0 | 0 | 1 | 0 | 0 | 0 | 1 | 0 | 1 |
| 0 | 0 | 0 | 1 | 0 | 0 | 0 | 1 | 2 | 1 | ? | 1 | 0 | 0 | 0 |
| 0 | 1 | 0 | 1 | 1 | 0 | ? | 0 | 0 | 1 | 1 | 0 | 1 | 0 | 0 |
| 0 | 0 | 0 | 0 | 0 | 0 | 1 | 0 | 0 | 1 | ? | ? | 1 | 0 | 1 |
| 1 | 1 | ? | ? | 1 | 0 | 1 | 0 | 1 | 1 | 0 | 1 | 0 | 1 | 0 |
| ? | ? | ? | 1 | 0 | 1 | 1 | 1 | 1 | 1 | 0 | 1 | 1 | 1 | 0 |
| 0 | 1 | 1 | ? | 0 | 1 | 0 | 1 | 0 | 0 | 0 | 1 | 0 | 0 | 1 |
| 0 | 0 | 0 | 1 | 0 | 0 | 1 | 0 | 0 | 0 | 0 | 1 | 0 | 0 | 1 |
| 1 | 0 | 1 | 1 | 0 | 0 | 1 | 1 | 0 | 1 | 1 | 1 | 1 | 1 | 0 |
| 1 | 0 | 0 | ? | 1 | 1 | 1 | 1 | 1 | 1 | 0 | 0 | 0 | 1 | 0 |
| 0 | 1 | 1 | 0 | 1 | 0 | 0 | 0 | 1 | 0 | [0 1] | 0 | 0 | 0 | 0 |
| 1 | 0 | 0 | 2 | 0 | 0 | 1 | 1 | 0 | 0 | 1 | 0 | 1 | 0 | 1 |
| 0 | ? | 1 | 0 | 0 | [0 1] | 0 | 0 | 2 | 1 | 0 | 0 | 1 | 0 | 0 |
| 0 | 1 | 0 | 0 | 1 | 1 | 0 | 0 | 0 | 0 | 0 | 1 | 0 | 0 | 0 |
| 1 | 1 | 1 | 0 | 0 | 1 | 0 | 0 | 0 | 0 | 2 | 0 | 1 | 1 | 0 |
| 0 | 1 | [0 1] | 0 | 0 | 0 | 0 | 0 | 0 | 0 | 0 | 0 | 1 | 0 | 0 |
| 0 | 0 | ? | 0 | 0 | 2 | ? | 0 | 0 | 0 | 0 | 0 | 0 | 0 | 0 |
| 0 | ? | 1 | 0 | 0 | 1 | 0 | 0 | 0 | | | | | | |

***Prolacerta broomi***

| | | | | | | | | | | | | | | |
|---|---|---|---|---|---|---|---|---|---|---|---|---|---|---|
| 1 | 0 | 0 | 1 | [0 1] | [0 1] | 0 | 0 | [0 1] | [0 1] | [0 1] | 0 | 0 | 0 | 0 |
| 1 | 0 | 0 | 0 | 1 | [0 1] | 0 | 0 | 1 | [0 1] | 0 | [0 1] | 0 | 0 | 1 |
| 0 | 0 | 0 | [1 2] | 0 | 0 | 0 | 0 | ? | 0 | ? | [0 1] | 0 | 1 | [0 1] |
| 0 | 1 | 0 | 0 | 0 | 0 | ? | 0 | 0 | 1 | 1 | 0 | 0 | 0 | 1 |
| 1 | [1 2] | 1 | [0 1] | 0 | 1 | 0 | 0 | 0 | [0 1] | 1 | ? | 2 | 1 | ? |
| [0 1] | [0 1] | [0 1] | 1 | 0 | 0 | 0 | 0 | 0 | [0 1] | 1 | 0 | 0 | 1 | 1 |
| 0 | 0 | 0 | 1 | 1 | 0 | 1 | 1 | 1 | 1 | 0 | 0 | 0 | [0 1] | 0 |
| 0 | 0 | [0 1] | 1 | 1 | 0 | 1 | 1 | 0 | 0 | 0 | 1 | 0 | 0 | 1 |
| 1 | 0 | 0 | 1 | 0 | [0 1] | 1 | 1 | [0 1] | 1 | 0 | 1 | 1 | ? | 1 |
| 1 | ? | 0 | 0 | 0 | [1 2] | 1 | 1 | 0 | 1 | [0 1] | 1 | 0 | 1 | 1 |
| 1 | 1 | 0 | ? | 0 | 0 | 1 | 0 | ? | 1 | 0 | 0 | 0 | 1 | 0 |
| 0 | 1 | [0 1] | 1 | 1 | 0 | 0 | 0 | 1 | 0 | 0 | 1 | [0 2] | 0 | ? |
| 1 | [0 1] | 1 | 1 | [0 1] | 0 | 0 | 1 | 1 | 0 | 1 | 1 | 0 | 1 | 0 |
| 0 | 1 | 0 | 0 | 0 | 0 | 0 | 1 | 2 | 0 | 1 | [1 2] | 1 | 1 | 1 |
| [0 1] | 0 | 0 | 1 | 1 | 0 | 1 | ? | 0 | 1 | 0 | 1 | 1 | 0 | 0 |
| 1 | 2 | 0 | [0 1] | [0 1] | 1 | 1 | [0 1] | 0 | [0 1] | 1 | 0 | 1 | 0 | 1 |
| 1 | 0 | 0 | ? | 0 | 0 | 0 | 0 | 0 | 0 | 0 | 0 | 0 | 0 | 0 |
| 0 | 0 | ? | 0 | 0 | ? | ? | 0 | ? | 0 | 1 | 1 | ? | 0 | 0 |
| 0 | ? | ? | 0 | 0 | 1 | 0 | 0 | 0 | | | | | | |

***Rhipaeosaurus* spp.**

| | | | | | | | | | | | | | | |
|---|---|---|---|---|---|---|---|---|---|---|---|---|---|---|
| ? | ? | ? | ? | ? | ? | ? | ? | ? | ? | ? | ? | ? | ? | ? |
| ? | ? | ? | ? | ? | ? | ? | ? | ? | ? | ? | ? | ? | ? | ? |
| ? | ? | ? | ? | ? | ? | ? | ? | ? | ? | ? | ? | ? | ? | ? |
| ? | ? | ? | ? | ? | ? | ? | ? | ? | ? | ? | ? | ? | ? | ? |
| ? | ? | ? | ? | ? | ? | ? | ? | ? | ? | ? | ? | ? | ? | ? |
| ? | ? | ? | ? | ? | ? | ? | ? | ? | ? | ? | ? | ? | ? | 0 |
| ? | ? | ? | ? | ? | ? | ? | ? | ? | ? | ? | ? | ? | ? | ? |
| 0 | 0 | 0 | ? | ? | 0 | ? | ? | ? | 0 | ? | ? | ? | ? | 1 |
| 0 | ? | ? | ? | ? | ? | ? | ? | ? | ? | ? | ? | ? | ? | ? |
| ? | ? | ? | ? | ? | ? | ? | ? | ? | ? | ? | 1 | ? | 1 | 1 |
| 0 | ? | ? | ? | ? | ? | ? | ? | ? | 1 | ? | ? | ? | ? | ? |
| ? | ? | 0 | ? | ? | ? | ? | 1 | 1 | 0 | ? | ? | ? | ? | ? |
| ? | ? | ? | 2 | ? | ? | 0 | ? | ? | ? | ? | ? | ? | ? | 1 |
| 0 | ? | ? | ? | ? | ? | 0 | ? | 1 | ? | 0 | ? | 0 | ? | ? |
| ? | 1 | ? | ? | ? | 1 | 1 | ? | 0 | ? | ? | ? | ? | ? | 0 |
| 1 | 1 | ? | ? | ? | ? | ? | ? | ? | 0 | 0 | ? | 0 | 0 | ? |
| 0 | ? | ? | ? | ? | ? | ? | 0 | ? | 0 | 0 | 0 | 1 | 0 | 0 |
| 0 | ? | ? | 0 | 0 | ? | ? | ? | ? | ? | 0 | ? | ? | ? | 0 |
| ? | ? | ? | ? | ? | 1 | ? | 1 | ? | | | | | | |

**Rhynchocephalia**

| | | | | | | | | | | | | | | |
|---|---|---|---|---|---|---|---|---|---|---|---|---|---|---|
| 2 | 0 | 0 | 0 | 0 | 0 | 0 | 0 | 0 | 0 | 0 | 0 | 0 | 0 | 0 |
| 0 | 0 | 0 | 0 | 0 | 1 | 1 | 0 | 0 | 1 | 0 | 0 | 0 | 0 | 0 |
| 0 | 0 | 0 | 2 | ? | 1 | ? | 0 | ? | 0 | ? | 1 | 0 | 1 | 1 |
| 0 | 1 | 0 | 0 | 0 | 0 | ? | 0 | 0 | 1 | 1 | 1 | 0 | 1 | 1 |
| 1 | 2 | 1 | 1 | 0 | 0 | 1 | 1 | 1 | 1 | ? | ? | 2 | 1 | ? |
| 0 | 0 | 1 | 1 | 0 | 1 | 1 | 0 | 0 | 1 | 1 | 0 | 0 | 0 | 1 |
| 0 | 0 | 1 | 1 | 0 | 0 | 1 | 1 | 1 | 1 | 0 | 0 | 1 | 1 | 1 |
| 0 | 0 | 1 | ? | 1 | 0 | 1 | 0 | 0 | 0 | 0 | 1 | 0 | 0 | 1 |
| 1 | 0 | 0 | [0 1] | 1 | 0 | 1 | 1 | 1 | 1 | 0 | 1 | 1 | ? | 1 |
| 1 | 1 | 0 | 1 | 0 | 1 | 1 | 1 | 0 | 1 | 1 | 0 | 1 | 1 | 1 |
| 1 | 1 | 0 | ? | 0 | 1 | 1 | 0 | 1 | 1 | 0 | 0 | 0 | 1 | 0 |
| 1 | 1 | 0 | 0 | 1 | 0 | 3 | 0 | 1 | 0 | 0 | [0 1] | 0 | 1 | ? |
| 1 | 0 | 0 | 1 | [0 1] | 0 | 0 | 1 | 1 | 0 | 1 | 1 | 2 | 1 | 0 |
| 0 | 1 | 0 | 0 | 0 | 0 | 1 | [0 1] | 1 | 2 | 0 | 0 | 0 | 1 | 1 |
| 1 | 0 | 0 | 0 | 1 | 0 | 1 | [0 1] | 0 | 1 | 0 | 1 | 0 | 1 | 1 |
| 1 | 1 | 1 | 0 | [0 1] | 1 | 1 | 1 | 1 | 1 | 2 | 0 | 1 | 0 | 1 |
| 1 | 0 | 0 | ? | 0 | 0 | 0 | 0 | 0 | 0 | 0 | 0 | 0 | 0 | 0 |
| 0 | 0 | ? | 0 | 0 | 1 | ? | 0 | 0 | 0 | 1 | 1 | 2 | 0 | 0 |
| 0 | ? | [1 2] | 0 | 0 | ? | 0 | 0 | 0 | | | | | | |

**Rhynchosauria**

| | | | | | | | | | | | | | | |
|---|---|---|---|---|---|---|---|---|---|---|---|---|---|---|
| 0 | 0 | 0 | 1 | 1 | 1 | 0 | 1 | 1 | 1 | 1 | 0 | 1 | 0 | 0 |
| 1 | 0 | 0 | 0 | 0 | 1 | 1 | 0 | 1 | 0 | 0 | 1 | 0 | 0 | 1 |
| 0 | 0 | 0 | 1 | 0 | 0 | 0 | 0 | ? | 0 | ? | 0 | 0 | 1 | 0 |
| 0 | 1 | 0 | 0 | 0 | 0 | ? | 0 | 0 | 1 | 1 | 0 | 0 | 1 | 1 |
| 1 | 3 | 1 | 0 | 0 | 0 | 1 | 1 | 1 | 1 | ? | ? | 2 | 1 | ? |
| 1 | 0 | 1 | 1 | 1 | 1 | 0 | 0 | 0 | 1 | 1 | 0 | 0 | 1 | 1 |
| 1 | 1 | 1 | 1 | 1 | 0 | 1 | 1 | 1 | 0 | ? | ? | 0 | 0 | 0 |
| 0 | 0 | 1 | ? | 0 | 0 | 1 | 1 | 0 | 0 | 0 | 1 | 1 | 0 | 1 |
| 1 | 0 | 0 | 1 | 0 | 0 | 1 | 1 | 0 | 1 | 0 | 1 | 1 | ? | ? |
| 1 | 1 | 0 | 0 | 1 | 1 | 1 | 0 | 0 | 1 | 1 | 1 | 1 | 1 | 1 |
| 0 | 1 | 0 | ? | 0 | 1 | 1 | 0 | 0 | 1 | 1 | 1 | 0 | 1 | 0 |
| 1 | 1 | 1 | 1 | 1 | 0 | 2 | ? | 1 | 0 | 0 | 1 | 0 | 0 | 1 |
| 1 | 0 | 0 | 1 | 0 | 0 | 0 | 1 | 1 | 0 | 1 | 1 | 1 | ? | 0 |
| 0 | 1 | 0 | [0 1] | 0 | 0 | 0 | 1 | 1 | 0 | 1 | 0 | 1 | 1 | 0 |
| 0 | 0 | 0 | 1 | 1 | 0 | 1 | 1 | 0 | 1 | 0 | 1 | 1 | 1 | 1 |
| 1 | 2 | 0 | 1 | 0 | 1 | 1 | 1 | 0 | 1 | 1 | 0 | 1 | 0 | 1 |
| 1 | 1 | 0 | ? | 0 | 0 | 0 | 0 | 0 | 0 | 0 | 0 | 0 | 0 | 0 |
| 0 | 0 | ? | 0 | 0 | 1 | ? | 0 | 0 | 0 | 1 | 1 | 1 | 0 | 0 |
| 0 | ? | 1 | [0 1] | 0 | 1 | 0 | 0 | 0 | | | | | | |

***Scutosaurus* spp.**

| | | | | | | | | | | | | | | |
|---|---|---|---|---|---|---|---|---|---|---|---|---|---|---|
| 2 | 0 | 0 | 0 | 0 | 1 | 0 | 0 | 0 | 0 | 0 | 0 | 0 | 0 | 0 |
| 0 | 0 | 0 | 0 | 0 | 1 | 0 | 0 | 1 | 0 | 1 | 0 | 0 | 0 | 1 |
| 0 | 0 | 0 | 0 | 0 | ? | 0 | 0 | 1 | 0 | ? | 0 | 1 | 1 | 0 |
| 0 | 0 | 0 | 0 | 0 | 1 | 1 | 1 | 0 | 1 | 1 | 0 | 1 | 0 | 0 |
| 0 | 0 | 0 | 0 | 0 | 0 | 1 | 0 | 0 | 0 | 1 | 0 | 1 | 0 | 1 |
| 1 | 1 | ? | ? | 0 | 0 | 1 | 0 | 2 | 0 | 0 | 1 | 1 | 0 | 0 |
| ? | ? | ? | 0 | ? | ? | ? | 1 | 1 | 1 | 1 | 1 | 1 | 1 | 0 |
| 0 | 1 | 0 | 1 | 0 | 1 | 0 | 1 | 1 | 0 | 0 | 1 | 1 | 1 | 1 |
| 0 | 1 | 0 | 1 | 0 | 1 | 0 | 0 | 0 | 0 | ? | 1 | 1 | 0 | 0 |
| 1 | ? | 1 | 1 | ? | 0 | 1 | 0 | 1 | 1 | 0 | 1 | 1 | 1 | 1 |
| 0 | 0 | 0 | ? | 1 | 1 | 1 | 1 | 1 | 1 | 0 | 1 | 0 | 0 | ? |
| 0 | 1 | 1 | 0 | 1 | 0 | 0 | 2 | 1 | 1 | 1 | 0 | 0 | 0 | ? |
| 0 | 0 | 0 | 2 | 0 | 0 | 1 | 1 | 1 | 1 | 1 | 0 | 1 | 0 | 1 |
| 1 | ? | 1 | 0 | 0 | 0 | 0 | 0 | 2 | 2 | 0 | 0 | 1 | 0 | 0 |
| 0 | 1 | 1 | 0 | 1 | 1 | 0 | 0 | 1 | 0 | 0 | 0 | 0 | 0 | 0 |
| 1 | 1 | 0 | 0 | 0 | 1 | 0 | 0 | 0 | 0 | 2 | 1 | 0 | 2 | 0 |
| 0 | 1 | 1 | 2 | 1 | 1 | 0 | 0 | 0 | 0 | 0 | 1 | 1 | 0 | 0 |
| 0 | 0 | ? | 0 | 0 | ? | 0 | 0 | ? | ? | 0 | 0 | 0 | 0 | 0 |
| 0 | ? | 2 | 1 | 1 | 0 | ? | 0 | 3 | | | | | | |

***Sinosaurosphargis yunguiensis***

| | | | | | | | | | | | | | | |
|---|---|---|---|---|---|---|---|---|---|---|---|---|---|---|
| 1 | 1 | 1 | 0 | 0 | 1 | 1 | 0 | 1 | 0 | ? | 0 | 0 | 0 | 0 |
| ? | 0 | 1 | 0 | 0 | 1 | 1 | 0 | 1 | 0 | ? | 0 | 0 | 0 | ? |
| 1 | 0 | 0 | 2 | ? | ? | ? | 0 | ? | ? | ? | 0 | 1 | 1 | 1 |
| 0 | 1 | 0 | 1 | 0 | ? | ? | 0 | 1 | 1 | ? | 0 | 0 | 1 | 1 |
| 1 | 1 | 1 | ? | ? | 0 | 1 | 1 | 1 | 1 | ? | ? | 2 | 1 | ? |
| 1 | 0 | 0 | 1 | 0 | ? | ? | 1 | ? | ? | ? | ? | ? | 0 | 0 |
| ? | ? | ? | 1 | 1 | ? | ? | ? | ? | 1 | 0 | 1 | 1 | 1 | 1 |
| 0 | 1 | 1 | ? | 1 | ? | ? | 1 | 1 | ? | 0 | 1 | 2 | ? | 0 |
| ? | ? | 1 | ? | ? | ? | ? | 0 | ? | 1 | ? | 1 | 1 | ? | ? |
| ? | ? | ? | ? | ? | 1 | ? | ? | ? | 1 | 1 | 1 | 1 | 1 | ? |
| 0 | ? | ? | ? | 2 | 0 | 1 | ? | ? | ? | ? | ? | 0 | 1 | 0 |
| ? | ? | 0 | ? | 1 | 0 | 0 | 0 | ? | ? | ? | ? | ? | ? | ? |
| ? | ? | ? | ? | ? | ? | ? | ? | ? | ? | ? | ? | ? | ? | ? |
| ? | ? | ? | ? | ? | ? | ? | ? | ? | ? | ? | ? | ? | ? | ? |
| ? | ? | ? | ? | ? | ? | ? | ? | ? | ? | ? | ? | ? | ? | ? |
| ? | ? | ? | ? | ? | ? | ? | ? | ? | ? | ? | ? | ? | ? | ? |
| ? | ? | ? | ? | ? | ? | ? | 0 | ? | 0 | ? | 1 | ? | 1 | ? |
| 1 | ? | ? | ? | ? | ? | ? | ? | ? | 1 | 1 | 0 | 0 | 0 | 0 |
| 0 | ? | ? | 0 | 0 | ? | 0 | ? | ? | | | | | | |

**Squamata**

| | | | | | | | | | | | | | | |
|---|---|---|---|---|---|---|---|---|---|---|---|---|---|---|
| [0 1] | 0 | [0 1] | 0 | 0 | [0 1] | 0 | 0 | [0 1] | [0 1] | 0 | 0 | 0 | 0 | 0 |
| 0 | 0 | 0 | 0 | 0 | 1 | 1 | 0 | 1 | 0 | 0 | 1 | 0 | 0 | 0 |
| 0 | 0 | 0 | 2 | 0 | 1 | 0 | 0 | ? | 0 | ? | 1 | 0 | [0 1] | 1 |
| 1 | 1 | 0 | 0 | [0 1] | 0 | ? | 0 | 0 | 1 | 1 | [0 1] | 0 | [0 1] | 1 |
| 1 | 2 | 1 | 1 | 0 | 0 | 1 | 1 | 1 | 1 | ? | ? | 2 | 1 | ? |
| 1 | [0 1] | 0 | 1 | 2 | 0 | 0 | 1 | ? | ? | ? | ? | 0 | 1 | 1 |
| 0 | 0 | 0 | 1 | 1 | 0 | ? | 1 | 1 | 1 | 0 | 0 | 1 | 1 | 0 |
| 0 | 1 | 1 | ? | 0 | 0 | 1 | 0 | 0 | 0 | 0 | 1 | 2 | 0 | 1 |
| 1 | 0 | 1 | 0 | ? | 0 | 1 | 1 | 1 | 1 | 0 | 1 | 1 | ? | 1 |
| 1 | 1 | 0 | 1 | 1 | 1 | 1 | 1 | 0 | 1 | 1 | 0 | 1 | 1 | 1 |
| 1 | 1 | 0 | ? | 0 | 1 | 1 | 0 | 1 | 1 | 1 | [0 1] | 0 | 1 | 0 |
| 1 | 1 | 1 | 0 | 1 | 0 | [0 3] | [0 1] | 1 | [0 1] | 0 | 1 | [0 2] | [0 1] | 1 |
| 1 | 0 | 0 | 1 | 1 | 0 | 0 | 1 | 1 | [0 1] | 1 | [0 1] | 2 | 1 | 0 |
| 0 | 1 | 0 | 0 | 0 | 1 | 1 | 1 | 1 | [0 2] | 1 | 0 | [0 1] | 1 | 1 |
| 1 | 0 | 0 | 0 | 1 | 0 | 1 | [0 1] | 0 | 1 | 0 | 1 | [0 1] | 1 | 1 |
| 1 | 1 | 1 | 0 | 1 | 1 | 1 | 1 | 1 | [0 1] | 2 | 0 | 1 | 0 | 1 |
| 1 | 0 | 0 | ? | 0 | 1 | 0 | 0 | 0 | 0 | 0 | 0 | 0 | 0 | 0 |
| 0 | 0 | 0 | 0 | 0 | ? | ? | 0 | ? | 0 | 1 | 1 | 2 | 0 | 0 |
| 0 | ? | [0 1 2] | 0 | 0 | 0 | 0 | ? | 0 | | | | | | |

***Trilophosaurus buettneri***

| | | | | | | | | | | | | | | |
|---|---|---|---|---|---|---|---|---|---|---|---|---|---|---|
| 1 | 0 | 0 | 0 | 1 | 0 | 0 | 0 | 1 | 0 | 0 | 1 | ? | ? | 0 |
| 1 | 0 | 0 | 0 | 0 | ? | ? | 0 | ? | ? | ? | 0 | ? | ? | 1 |
| 0 | 0 | 0 | ? | ? | ? | ? | ? | ? | ? | ? | 0 | 0 | 1 | 1 |
| 0 | 1 | 0 | 0 | 0 | 0 | ? | 0 | ? | ? | 0 | ? | ? | 1 | 1 |
| 1 | 3 | 1 | 1 | ? | 1 | ? | ? | 0 | 1 | ? | ? | 2 | 1 | ? |
| ? | 0 | 0 | 1 | ? | ? | ? | ? | ? | ? | ? | ? | 0 | ? | 1 |
| 1 | 1 | 1 | 0 | ? | ? | ? | 1 | 1 | 1 | ? | 0 | 0 | ? | 1 |
| 0 | 1 | 1 | 1 | 0 | 0 | 1 | 1 | 1 | 0 | 0 | 1 | ? | ? | 1 |
| 1 | 0 | 1 | 0 | ? | ? | 1 | 1 | 0 | ? | ? | 1 | 1 | ? | ? |
| 1 | 0 | ? | ? | 1 | 1 | 1 | ? | ? | 0 | 1 | ? | 1 | 1 | 1 |
| 1 | 1 | 0 | ? | 1 | 1 | 1 | 1 | 0 | 0 | 0 | 0 | 0 | 1 | 0 |
| ? | 1 | 1 | 0 | 1 | 0 | 0 | 0 | 1 | 0 | ? | 1 | [1 2] | 0 | 1 |
| 1 | 0 | 1 | 1 | 0 | 0 | 0 | ? | 1 | 0 | 1 | 1 | 1 | ? | 1 |
| 0 | 1 | 0 | 0 | 0 | 0 | 0 | 1 | 2 | 0 | 1 | 0 | 0 | 0 | 0 |
| 0 | 0 | 0 | 1 | 1 | 0 | 1 | 1 | 0 | 1 | 0 | 1 | 1 | 0 | 0 |
| 1 | 2 | 0 | 1 | 0 | 1 | 1 | 1 | 0 | 1 | 1 | 0 | 1 | 0 | 1 |
| 1 | 0 | 0 | ? | 0 | 0 | 0 | 0 | 0 | 0 | 0 | 0 | 0 | 0 | 0 |
| 0 | 0 | ? | 0 | 0 | ? | ? | 0 | ? | 0 | 1 | 1 | 1 | 0 | 0 |
| 0 | ? | 0 | 0 | 0 | ? | ? | 0 | 0 | | | | | | |

***Youngina capensis***

| | | | | | | | | | | | | | | |
|---|---|---|---|---|---|---|---|---|---|---|---|---|---|---|
| 1 | 0 | 0 | 0 | 0 | 0 | 0 | 0 | 0 | 0 | 0 | 0 | 0 | 0 | 0 |
| 0 | 0 | 0 | 0 | 0 | 1 | 1 | 0 | 0 | 0 | 1 | 0 | 1 | 0 | 0 |
| 0 | 0 | 0 | 1 | 0 | 0 | 0 | 0 | 0 | 0 | 0 | 0 | 0 | 1 | 0 |
| 0 | 1 | 0 | 1 | 0 | 0 | ? | 0 | 0 | 1 | 1 | 1 | 0 | 0 | 1 |
| 1 | 0 | 1 | 0 | 0 | 0 | 0 | 1 | 0 | 0 | 0 | 1 | 2 | 0 | 1 |
| 1 | 0 | 0 | 1 | 0 | 1 | 1 | 0 | 0 | 0 | 1 | 0 | 0 | 1 | 1 |
| 0 | 0 | 0 | 1 | 0 | 0 | 1 | 1 | 1 | 1 | 0 | 0 | 0 | 0 | 0 |
| 0 | 0 | 0 | 1 | 1 | 0 | 1 | 1 | 0 | 0 | 0 | 1 | 0 | 0 | 1 |
| 1 | 0 | 0 | 1 | 0 | 0 | 0 | 1 | 0 | 1 | 0 | 0 | 1 | ? | 1 |
| 1 | ? | 0 | 0 | 1 | 0 | 1 | 1 | 0 | 1 | 0 | 1 | 0 | 0 | 1 |
| 0 | 0 | 0 | ? | 0 | 0 | 1 | 0 | 0 | 1 | 0 | 0 | 0 | 0 | 0 |
| 0 | ? | 0 | 0 | 1 | 1 | 0 | 0 | 1 | 0 | 0 | 0 | 0 | 0 | 1 |
| 1 | 0 | 0 | 1 | 0 | 0 | 0 | 1 | ? | 0 | [0 1] | 1 | [0 1] | 1 | ? |
| 0 | 1 | 0 | 0 | 0 | 0 | 0 | 1 | 1 | [0 2] | 0 | [0 1] | 1 | 0 | 1 |
| 0 | 1 | 0 | 0 | 1 | 1 | 1 | ? | 0 | 1 | 0 | 1 | 1 | 0 | 1 |
| 1 | 0 | 0 | 0 | 0 | 0 | 1 | 0 | 0 | 1 | 1 | 0 | 1 | 0 | 1 |
| 1 | 0 | 0 | ? | 0 | 0 | 0 | 0 | 0 | 0 | 0 | 0 | 0 | 0 | 0 |
| 0 | 0 | ? | 0 | 0 | ? | ? | 0 | ? | 0 | 1 | ? | 1 | 0 | 0 |
| 0 | ? | 2 | 0 | 0 | 0 | 0 | 0 | 0 | | | | | | |

## CHANGES TO THE MATRIX

The matrix used herein is based on the matrix of Bever et al. 2015 with additions made by Schoch & Sues 2015 and eight new characters added. I corrected some errors found in the matrix of Bever et al. 2015 and redefined several characters. The corrections include:

- Rescoring of characters 7, 8, 55, 77, 102, 103, and 110 according to Gaffney 1990 and personal observations, and, in some cases, following fig. 4 of Bever et al. 2015.
- Rescoring of character 174 for Archosauriformes and Squamata to [01] following Müller et al. 2010.
- Redefining the state 0 of character 175 (Caudal vertebral count) to “22 or more” (*Odontochelys semitestacea* was described by Li et al. 2008 as having at least 22 caudal vertebrae, thus it could fit neither of the original states).
- Rescoring the character 190 for *Odontochelys semitestacea* and *Eunotosaurus africanus* following Li et al. 2008 and Lyson et al. 2013b.
- Rescoring of character 191 for *Proganochelys quenstedti* and *Odontochelys semitestacea* following Gaffney 1990, Li et al. 2008, Gilbert et al. 2001 and other turtle morphogenetic literature, and personal observations. Both the dorsal epiplastral processes in early Pan-Testudinata and the embryonic primordia of epiplastra in modern Testudines are slender and gracile, and these elements are interpreted as true homologues of clavicles. They should not be mistaken with plate-like epiplastra as a whole, which are secondary ossifications forming around the gracile primordia.
- Rescoring of character 192 for *Proganochelys quenstedti* and *Odontochelys semitestacea* following Gaffney 1990, Li et al. 2008, Gilbert et al. 2001 and other turtle morphogenetic literature, and personal observations. Although the entoplastron of the crown-group turtles is usually rhomboidal, the embryonic primordium homologous to interclavicle is in many cases T-shaped, and in the Late Triassic turtles the long posterior process of the entoplastron (exposed only viscerally) indicates, that this shape is plesiomorphic for Pan-Testudines. Similarly, to clavicles, the derived adult morphology of entoplastron should not be considered primary.
- Rescoring of character 193 for *Odontochelys semitestacea* following Li et al. 2008 and personal observations. There is no trace of and no room for the mineralized sternum in that animal.
- Rescoring of character 195 for *Odontochelys semitestacea* following Li et al. 2008 and personal observations.
- Rescoring of character 242 for *Proganochelys quenstedti* and *Odontochelys semitestacea* following Gaffney 1990, Li et al. 2008, and personal observations. There are no trunk osteoderms present in these animals, and I refrain from scoring that character positively for *P. quenstedti* because of its neck and tail spines. According to current knowledge they are obviously not homologous to the trunk osteoderms of Archosauriformes, pareiasaurs, placodonts etc., and scoring that character positively for *P. quenstedti* would only result in increased homoplasy in the matrix.
- Rescoring of character 243 for *Odontochelys semitestacea* following Li et al. 2008 and personal observations (see above).
- Rescoring of character 245 for *Odontochelys semitestacea* following Li et al. 2008 and personal observations. The plastron is widely accepted as the derivative of gastralia, so the oldest plastron-bearing animal must be scored positively for presence of gastralia.
- Redefinition of character 246 states to “0: absent; 1: partial fusion of gastralia resulting in forked elements; 2: fully developed plastron present” to score the intermediate state present in *Pappochelys rosinae* according to Schoch & Sues 2015.
- Redefinition of character 247 state 1 to “present, rib distinctly T-shaped in cross-section”, excluding the mention of metaplastic ossification. Firstly, the presence of dermal ossification around the ribs is already scored elsewhere (character 254), secondly, according to Lyson et al. 2013a in *Eunotosaurus africanus* “There is no evidence of metaplastic ossification at any stage of rib formation, unlike in undisputed turtles in which the costals incorporate interwoven structural fibers”. Therefore, with the original definition only the uncontroversial stem-turtles would be scored positively for that character, making it useless.
- Rescoring of character 248 for *Odontochelys semitestacea* following Li et al. 2008, personal observations, and in line with all the new reconstructions of *O. semitestacea*.
- Redefinition of character 249 states to “0: more than 10; 1: 10; 2: 9 or fewer”. The original definitions artificially divided taxa to two groups and a priori strongly implied homology of rib number between Pan-Testudinata and *E.*

*africanus*, even though the number is not exactly the same. I think that three gradual states treated as ordered are better fit for that character. I rescored that character accordingly for *Proganochelys quenstedti*, *Eunotosaurus africanus*, and *Bradysaurus*. Although I consider the morphology of the eighth presacral vertebra of *P. quenstedti* as transitional, so it can potentially be scored either with "0", "1", or even as an additional state between states 0 and 1, I decided to score it as "0". That way it represents the plesiomorphic state present in *Proterochersis* spp., which was excluded from the matrix due to its incompleteness and lack of cranial material. *Odontochelys semitestacea* is scored "?", because the identity of the eighth presacral needs to be confirmed, although I presume "0" will be the correct state. Prior to the analyses 1-70 documented below I performed a series of analyses with this character scored for *O. semitestacea* as "?", "0", or "1", and the effect on the support was minimal.

- Rescoring of character 250 for *O. semitestacea* following Li et al. 2008 and personal observations. The dorsal vertebrae in *Odontochelys* are not more than two times longer than wide. This is even true for most vertebrae of *Proterochersis porebensis*.
- Rescoring of character 251 for *Scutosaurus* and *Sinosaurosphargis yunguiensis* based on Hirasawa et al. 2013 and personal observations.
- Rescoring of character 253 for Archosauriformes. Several taxa, including aetosaurs, have more or less broadened ribs.
- Removal of the duplicated scorings for character 248 which were present in the matrix provided by Bever et al. 2015 instead of character 253 and restoration of character 253.
- Rescoring of character 254 for Archosauriformes following Hirasawa et al. 2013. At least in birds the dermal outgrowths may form on the ribs.
- Rescoring of character 256 for *Odontochelys semitestacea* following Li et al. 2008 and personal observations.
- Rescoring of character 263 for *Odontochelys semitestacea* and *Proganochelys quenstedti* based on Schoch & Sues 2015. *Pappochelys rosinae* shows that the plastral bones (except ento- and epiplastra) are composed from serially fused gastralia (Schoch & Sues 2015), therefore each plastral bone is homologous to more than one ventral ossification.
- Rescoring of character 264 for *Sinosaurosphargis yunguiensis* following Hirasawa et al. 2013.
- Rescoring of several characters for *Rhipaeosaurus* following Chudinov 1957.
- Removal of character "Number of dorsal ribs". In all the taxa present in the matrix the number of the dorsal ribs and vertebrae is strictly correlated, and because of that this character was effectively a duplicate of character 249.

I also supplemented the matrix of Bever et al. 2015 with characters from Schoch and Sues 2015 and with eight new characters (271-278). All taxa were scored based on the references provided by Bever et al. 2015. Finally, I included *Pappochelys rosinae* (scored following Schoch & Sues 2015) and an enigmatic Middle Triassic parareptile with fenestrated temporal region, *Candelaria barbouri* (scored following Cisneros et al. 2004).

Note that the character numbers are taken from my modified matrix and thus does not correspond to the original numbers from Bever et al. 2015 and Schoch & Sues 2015.

## References


Bever G.S., Lyson T.R., Field D.J., Bhullar B.-A.S. 2015. Evolutionary origin of the turtle skull. Nature 525:239–242.

Chudinov P.K. 1957. Cotylosaurs from the Upper Permian red Ural deposits [in Russian]. Akademiya Nauk SSSR, Trudy Paleontologicheskogo Instituta 68:2–87.

Cisneros J.C., Damiani R., Schultz C., da Rosa Á., Schwanke C, Neto L.W., Aurélio P.L.P. 2004. A procolophonoid reptile with temporal fenestration from the Middle Triassic of Brazil. Proceedings of the Royal Society B 271:1541–1546.

Gaffney E.S. 1990. The comparative osteology of the Triassic turtle *Proganochelys*. Bulletin of the American Museum of Natural History 194:1–263.

Gilbert S.F., Loredo G.A., Burke A.C. 2001. Morphogenesis of the turtle shell: the development of a novel structure in tetrapod evolution. Evolution & Development 3:47–58.

Hirasawa T., Nagashima H., Kuratani S. 2013. The endoskeletal origin of the turtle carapace. Nature Communications 4:1–7.

Li C., Wu X.-C., Rieppel O., Wang L.-T., Zhao L.-J. 2008. An ancestral turtle from the Late Triassic of southwestern China. Nature 456:497–501.

Lyson T.R., Bever G.S., Scheyer T.M., Hsiang A.Y., Gauthier J.A. 2013a. Evolutionary origin of the turtle shell. Current Biology 23:1–7.

Lyson T.R., Bhullar B.-A.S., Bever G.S., Joyce W.G., de Queiroz K., Abzhanov A., Gauthier J.A. 2013b. Homology of the enigmatic nuchal bone reveals novel reorganization of the shoulder girdle in the evolution of the turtle shell. Evolution & Development 15:317–325.

Müller J., Scheyer T.M., Head J.J., Barrett P.M., Werneburg I., Ericson P.G.P., Pol D., Sánchez-Villagra M.R. 2010. Homeotic effects, somitogenesis and the evolution of vertebral numbers in recent and fossil amniotes. PNAS 107:2118–2123.

Schoch R.R., Sues H.-D. 2015. A Middle-Triassic stem-turtle and the evolution of the turtle body plan. Nature 523:584–587.

## PHYLOGENETIC TREES AND LISTS OF SYNAPOMORPHIES

Nodes and taxa lacking apomorphies are not listed. Bootstrap values above and node numbers below branches.

### ANALYSIS 1
### (ALL TAXA)

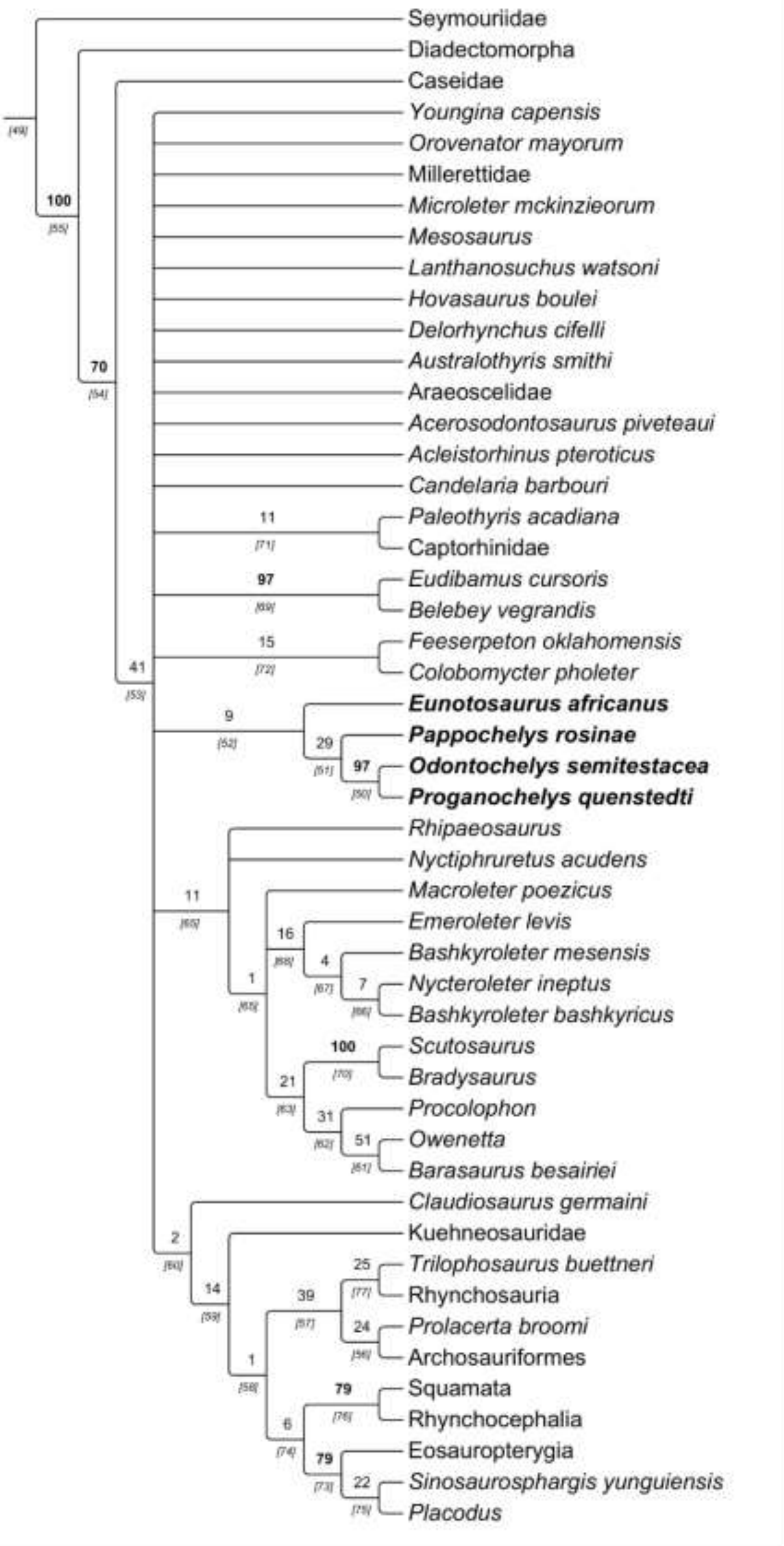

***Proganochelys quenstedti*:**
Char. 8: 0 → 1
Char. 11: 0 → 1
Char. 106: 0 → 1
Char. 108: 1 → 0
Char. 109: 0 → 1
Char. 128: 0 → 1
Char. 175: 0 → 1
Char. 202: 1 → 0
Char. 207: 1 → 0
Char. 209: 1 → 0
Char. 244: 0 → 1
Char. 248: 0 → 1
Char. 250: 0 → 1
Char. 252: 1 → 0
Char. 262: 0 → 1

***Pappochelys rosinae*:**
Char. 1: 0 → 1
Char. 5: 1 → 0
Char. 12: 0 → 1
Char. 23: 0 → 1
Char. 41: 0 → 1
Char. 49: 0 → 1
Char. 50: 1 → 0
Char. 75: 0 → 1
Char. 129: 0 → 1
Char. 169: 1 → 0
Char. 260: 2 → 0
Char. 265: 0 → 1

***Odontochelys semitestacea*:**
Char. 43: 0 → 1

***Eunotosaurus africanus*:**
Char. 19: 0 → 1
Char. 43: 0 → 1
Char. 76: 0 → 1
Char. 97: 1 → 0
Char. 103: 0 → 1
Char. 153: 1 → 0
Char. 192: 1 → 0
Char. 202: 1 → 0
Char. 211: 0 → 1
Char. 219: 0 → 1
Char. 248: 0 → 1
Char. 249: 0 → 2
Char. 250: 0 → 1
Char. 263: 0 → 1
Char. 273: 0 → 1
Char. 274: 0 → 1
Char. 276: 0 → 1
Char. 277: 0 → 1

***Acerosodontosaurus piveteaui*:**
Char. 23: 0 → 1
Char. 50: 1 → 0
Char. 59: 0 → 1
Char. 78: 1 → 0
Char. 81: 1 → 0
Char. 89: 0 → 1
Char. 94: 0 → 1
Char. 128: 0 → 1
Char. 129: 0 → 1
Char. 155: 0 → 1
Char. 206: 0 → 2
Char. 208: 0 → 1
Char. 265: 0 → 1
Char. 266: 0 → 1
Char. 278: 3 → 1

***Acleistorhinus pteroticus*:**
Char. 21: 0 → 1
Char. 30: 0 → 1
Char. 47: 0 → 1
Char. 55: 1 → 0
Char. 56: 0 → 1
Char. 57: 0 → 1
Char. 64: 0 → 1
Char. 70: 0 → 1
Char. 79: 0 → 1
Char. 95: 0 → 1
Char. 110: 0 → 1
Char. 113: 0 → 1
Char. 114: 0 → 1
Char. 120: 1 → 0
Char. 131: 0 → 1
Char. 137: 0 → 1
Char. 140: 0 → 2
Char. 146: 1 → 0
Char. 147: 0 → 1
Char. 169: 1 → 0
Char. 170: 0 → 1
Char. 278: 3 → 1

**Araeoscelidae:**
Char. 0: 0 → 1
Char. 5: 1 → 0
Char. 20: 1 → 0
Char. 23: 0 → 1
Char. 27: 0 → 1
Char. 28: 0 → 1
Char. 38: 1 → 0
Char. 40: 2 → 0
Char. 43: 0 → 1
Char. 50: 1 → 0
Char. 59: 0 → 1
Char. 60: 0 → 1
Char. 67: 0 → 1
Char. 72: 1 → 2
Char. 84: 1 → 0
Char. 89: 0 → 1
Char. 106: 0 → 1
Char. 116: 1 → 0
Char. 117: 0 → 2
Char. 154: 1 → 0
Char. 159: 1 → 0
Char. 166: 1 → 0
Char. 169: 1 → 0
Char. 170: 0 → 1
Char. 193: 0 → 1
Char. 197: 0 → 1
Char. 202: 1 → 0
Char. 207: 1 → 0
Char. 209: 1 → 0
Char. 221: 1 → 0
Char. 224: 0 → 1
Char. 239: 0 → 1
Char. 266: 0 → 1
Char. 278: 3 → 1

**Archosauriformes:**
Char. 32: 0 → 1
Char. 94: 1 → 0
Char. 112: 1 → 0
Char. 152: 0 → 1
Char. 154: 0 → 2
Char. 166: 1 → 0
Char. 171: 0 → 1
Char. 185: 0 → 1
Char. 204: 0 → 1
Char. 218: 0 → 3
Char. 224: 0 → 1
Char. 242: 0 → 1

***Australothyris smithi*:**
Char. 23: 0 → 1
Char. 24: 0 → 1
Char. 34: 0 → 1
Char. 55: 1 → 0
Char. 71: 1 → 0
Char. 79: 0 → 1
Char. 83: 0 → 1
Char. 85: 1 → 0
Char. 98: 1 → 0
Char. 100: 0 → 1
Char. 103: 0 → 1
Char. 110: 0 → 1
Char. 123: 1 → 0
Char. 129: 0 → 1
Char. 131: 0 → 1
Char. 132: 1 → 0
Char. 144: 1 → 0
Char. 147: 0 → 1
Char. 149: 1 → 0
Char. 150: 0 → 1
Char. 159: 1 → 0
Char. 163: 1 → 0
Char. 192: 1 → 0

***Barasaurus besairiei*:**
Char. 33: 1 → 0
Char. 75: 1 → 0
Char. 216: 0 → 1

***Bashkyroleter bashkyricus*:**
Char. 275: 1 → 0

***Bashkyroleter mesensis*:**
Char. 169: 1 → 0

***Belebey vegrandis*:**
Char. 154: 1 → 0

***Bradysaurus* spp.:**
Char. 19: 0 → 1
Char. 73: 0 → 1
Char. 79: 0 → 1
Char. 135: 1 → 0
Char. 249: 0 → 1

***Candelaria barbouri*:**
Char. 1: 0 → 1
Char. 5: 1 → 0
Char. 8: 0 → 1
Char. 15: 0 → 1
Char. 23: 0 → 1
Char. 25: 1 → 0
Char. 33: 01 → 2
Char. 49: 0 → 1
Char. 50: 1 → 0
Char. 55: 1 → 0
Char. 67: 0 → 1
Char. 76: 0 → 1
Char. 79: 0 → 2
Char. 83: 0 → 1
Char. 88: 1 → 0
Char. 89: 0 → 1
Char. 94: 0 → 1
Char. 95: 0 → 1
Char. 126: 0 → 1
Char. 127: 0 → 1
Char. 132: 1 → 0
Char. 154: 1 → 2
Char. 159: 1 → 0
Char. 169: 1 → 0
Char. 276: 0 → 1
Char. 277: 0 → 1

**Captorhinidae:**
Char. 3: 0 → 1
Char. 23: 0 → 1
Char. 25: 1 → 0
Char. 26: 0 → 1
Char. 73: 0 → 1
Char. 75: 0 → 1
Char. 83: 0 → 1
Char. 108: 1 → 0
Char. 180: 1 → 0
Char. 183: 1 → 0
Char. 201: 1 → 0
Char. 203: 1 → 2
Char. 216: 1 → 0
Char. 240: 1 → 0

**Caseidae:**
Char. 24: 0 → 1
Char. 25: 1 → 0
Char. 36: 0 → 1
Char. 38: 1 → 0
Char. 46: 1 → 0
Char. 50: 1 → 0
Char. 56: 0 → 1
Char. 170: 0 → 1
Char. 194: 0 → 1
Char. 273: 0 → 1
Char. 274: 0 → 1
Char. 278: 3 → 2

***Claudiosaurus germaini*:**
Char. 64: 0 → 1
Char. 84: 1 → 0
Char. 105: 0 → 1
Char. 130: 0 → 1
Char. 144: 1 → 0
Char. 166: 1 → 0
Char. 199: 0 → 1
Char. 203: 1 → 2
Char. 204: 0 → 1
Char. 220: 0 → 1

***Colobomycter pholeter*:**
Char. 21: 0 → 1
Char. 25: 1 → 0
Char. 84: 1 → 0
Char. 154: 1 → 0
Char. 167: 0 → 1
Char. 267: 0 → 1

***Delorhynchus cifelli*:**
Char. 18: 0 → 1
Char. 21: 0 → 1
Char. 24: 0 → 1
Char. 26: 0 → 1
Char. 28: 0 → 1
Char. 33: 01 → 2
Char. 39: 0 → 1
Char. 52: 0 → 1
Char. 57: 0 → 1
Char. 100: 0 → 1
Char. 116: 1 → 0
Char. 117: 0 → 1
Char. 119: 1 → 0
Char. 131: 0 → 1
Char. 147: 0 → 1
Char. 156: 1 → 0
Char. 167: 0 → 1
Char. 189: 0 → 1
Char. 191: 0 → 1
Char. 202: 1 → 0
Char. 204: 0 → 2
Char. 267: 0 → 1

**Diadectomorpha:**
Char. 0: 0 → 1
Char. 64: 0 → 1
Char. 70: 0 → 1
Char. 122: 0 → 1
Char. 123: 1 → 0
Char. 146: 1 → 0
Char. 275: 1 → 0
Char. 278: 3 → 0

***Emeroleter levis*:**
Char. 0: 0 → 2
Char. 51: 1 → 0

**Eosauropterygia:**
Char. 159: 1 → 0
Char. 166: 1 → 0
Char. 174: 0 → 1
Char. 194: 0 → 2
Char. 272: 1 → 0

***Eudibamus cursoris*:**
Char. 154: 1 → 2
Char. 163: 1 → 0

***Feeserpeton oklahomensis*:**
Char. 51: 0 → 1

Char. 70: 0 → 1
Char. 157: 0 → 1
Char. 158: 0 → 1

***Hovasaurus boulei*:**
Char. 41: 0 → 1
Char. 43: 0 → 1
Char. 50: 1 → 0
Char. 55: 1 → 0
Char. 59: 0 → 1
Char. 67: 0 → 1
Char. 77: 0 → 2
Char. 78: 1 → 0
Char. 79: 0 → 1
Char. 89: 0 → 1
Char. 93: 1 → 0
Char. 113: 0 → 1
Char. 127: 0 → 1
Char. 135: 0 → 1
Char. 136: 0 → 1
Char. 138: 0 → 1
Char. 146: 1 → 0
Char. 187: 0 → 1
Char. 188: 0 → 1
Char. 191: 0 → 1
Char. 193: 0 → 1
Char. 196: 0 → 1
Char. 201: 1 → 0
Char. 204: 0 → 2
Char. 206: 0 → 2
Char. 215: 0 → 1
Char. 220: 0 → 1
Char. 224: 0 → 1
Char. 265: 0 → 1
Char. 275: 1 → 0

**Kuehneosauridae:**
Char. 7: 0 → 1
Char. 26: 0 → 1
Char. 44: 1 → 0
Char. 79: 0 → 2
Char. 82: 0 → 1
Char. 98: 1 → 0
Char. 108: 1 → 0
Char. 113: 0 → 1
Char. 128: 0 → 1
Char. 159: 1 → 0
Char. 181: 0 → 1
Char. 185: 0 → 1
Char. 206: 0 → 2
Char. 245: 0 → 1

***Lanthanosuchus watsoni*:**
Char. 25: 1 → 0
Char. 51: 0 → 1
Char. 76: 0 → 1
Char. 86: 0 → 1
Char. 95: 0 → 1
Char. 98: 1 → 0
Char. 110: 0 → 1
Char. 113: 0 → 1
Char. 114: 0 → 1
Char. 119: 1 → 0
Char. 131: 0 → 1
Char. 137: 0 → 1
Char. 138: 0 → 1
Char. 140: 0 → 2
Char. 144: 1 → 0
Char. 147: 0 → 1
Char. 154: 1 → 2

***Macroleter poezicus*:**
Char. 0: 0 → 1
Char. 9: 0 → 1
Char. 26: 0 → 1
Char. 52: 0 → 1
Char. 66: 1 → 2
Char. 84: 1 → 0
Char. 87: 0 → 1
Char. 134: 0 → 2
Char. 140: 0 → 1
Char. 146: 1 → 0
Char. 169: 1 → 0
Char. 235: 0 → 1

***Mesosaurus* spp.:**
Char. 0: 0 → 1
Char. 2: 0 → 1
Char. 5: 1 → 0
Char. 6: 0 → 1
Char. 8: 0 → 1
Char. 9: 0 → 1
Char. 13: 0 → 1
Char. 19: 0 → 1
Char. 20: 1 → 0
Char. 23: 0 → 1
Char. 26: 0 → 1
Char. 38: 1 → 0
Char. 41: 0 → 1
Char. 50: 1 → 0
Char. 67: 0 → 1
Char. 76: 0 → 1
Char. 83: 0 → 1
Char. 84: 1 → 0
Char. 85: 1 → 0
Char. 94: 0 → 1
Char. 107: 0 → 1
Char. 109: 0 → 1
Char. 115: 0 → 1
Char. 146: 1 → 0
Char. 148: 0 → 1
Char. 149: 1 → 0
Char. 159: 1 → 0
Char. 163: 1 → 0
Char. 164: 0 → 1
Char. 166: 1 → 0
Char. 167: 0 → 1
Char. 176: 0 → 1
Char. 183: 1 → 0
Char. 184: 0 → 1
Char. 192: 1 → 0
Char. 199: 0 → 1
Char. 204: 0 → 2
Char. 206: 0 → 1
Char. 217: 0 → 1
Char. 219: 0 → 1
Char. 220: 0 → 1
Char. 231: 0 → 1
Char. 234: 1 → 0
Char. 240: 1 → 0
Char. 260: 2 → 0
Char. 272: 2 → 0
Char. 278: 3 → 0

***Microleter mckinzieorum*:**
Char. 0: 0 → 1
Char. 18: 0 → 1
Char. 20: 1 → 0
Char. 24: 0 → 1
Char. 25: 1 → 0
Char. 36: 0 → 1
Char. 39: 0 → 1
Char. 51: 0 → 2
Char. 56: 0 → 1
Char. 70: 0 → 1
Char. 76: 0 → 1
Char. 79: 0 → 1
Char. 83: 0 → 1
Char. 94: 0 → 1
Char. 106: 0 → 1
Char. 110: 0 → 1
Char. 132: 1 → 0
Char. 166: 1 → 0
Char. 276: 0 → 1
Char. 278: 3 → 1

**Millerettidae:**
Char. 5: 1 → 0
Char. 20: 1 → 0
Char. 24: 0 → 1
Char. 25: 1 → 0
Char. 44: 1 → 0
Char. 56: 0 → 1
Char. 66: 0 → 2
Char. 78: 1 → 0
Char. 80: 1 → 0
Char. 84: 1 → 2
Char. 88: 1 → 0
Char. 96: 1 → 0
Char. 117: 0 → 1
Char. 120: 1 → 0
Char. 121: 0 → 1
Char. 124: 0 → 1
Char. 127: 0 → 1
Char. 135: 0 → 1
Char. 145: 0 → 1
Char. 159: 1 → 0
Char. 163: 1 → 0
Char. 166: 1 → 0
Char. 180: 1 → 0
Char. 192: 1 → 0
Char. 211: 0 → 1
Char. 230: 0 → 1
Char. 248: 0 → 1
Char. 252: 0 → 1
Char. 253: 0 → 1

***Nycteroleter ineptus*:**
Char. 278: 0 → 3

***Nyctiphruretus acudens*:**
Char. 0: 0 → 1
Char. 21: 0 → 1
Char. 33: 1 → 2
Char. 41: 0 → 1
Char. 66: 1 → 2
Char. 81: 1 → 0
Char. 83: 0 → 1
Char. 84: 1 → 2
Char. 85: 1 → 0
Char. 94: 0 → 1
Char. 166: 1 → 0
Char. 167: 0 → 1
Char. 224: 0 → 1
Char. 266: 0 → 1
Char. 272: 2 → 1
Char. 276: 0 → 1

***Orovenator mayorum*:**
Char. 0: 0 → 1
Char. 8: 0 → 1
Char. 24: 0 → 1
Char. 25: 1 → 0
Char. 36: 0 → 1
Char. 62: 0 → 1
Char. 72: 1 → 2
Char. 89: 0 → 1
Char. 136: 0 → 1
Char. 141: 0 → 1
Char. 159: 1 → 0
Char. 160: 0 → 1
Char. 165: 0 → 1
Char. 278: 3 → 1

***Owenetta* spp.:**
Char. 169: 1 → 0

***Paleothyris acadiana*:**
Char. 38: 1 → 0
Char. 50: 1 → 0
Char. 66: 1 → 2
Char. 102: 0 → 1
Char. 146: 1 → 0
Char. 239: 0 → 1

***Placodus* spp.:**
Char. 0: 1 → 2
Char. 9: 0 → 1
Char. 12: 0 → 1
Char. 13: 0 → 1
Char. 19: 0 → 1
Char. 26: 0 → 1
Char. 31: 0 → 1
Char. 44: 1 → 0
Char. 46: 1 → 0
Char. 57: 0 → 1
Char. 78: 1 → 0
Char. 93: 1 → 0
Char. 102: 1 → 2
Char. 109: 1 → 0
Char. 140: 1 → 0
Char. 155: 0 → 1
Char. 163: 1 → 0
Char. 164: 0 → 1

***Procolophon* spp.:**
Char. 41: 0 → 1
Char. 69: 0 → 1
Char. 79: 0 → 1
Char. 83: 0 → 1
Char. 88: 0 → 1
Char. 117: 1 → 0
Char. 149: 1 → 0
Char. 204: 0 → 1
Char. 237: 0 → 1
Char. 238: 0 → 1
Char. 272: 2 → 1

***Prolacerta broomi*:**
Char. 58: 1 → 0
Char. 66: 1 → 0
Char. 67: 1 → 0
Char. 80: 1 → 0
Char. 139: 1 → 0
Char. 147: 1 → 0
Char. 192: 1 → 0
Char. 203: 1 → 2
Char. 206: 0 → 12

***Rhipaeosaurus* spp.:**
Char. 172: 0 → 1
Char. 277: 0 → 1

**Rhynchocephalia:**
Char. 0: 1 → 2
Char. 24: 0 → 1
Char. 75: 1 → 0
Char. 77: 0 → 1
Char. 94: 1 → 0
Char. 117: 12 → 0
Char. 139: 1 → 0
Char. 167: 1 → 0
Char. 205: 1 → 0

**Rhynchosauria:**
Char. 0: 1 → 0
Char. 7: 0 → 1
Char. 9: 0 → 1
Char. 26: 0 → 1
Char. 44: 1 → 0
Char. 68: 0 → 1
Char. 99: 1 → 0
Char. 150: 1 → 0
Char. 160: 0 → 1
Char. 161: 0 → 1
Char. 171: 0 → 2
Char. 182: 1 → 0
Char. 223: 0 → 1
Char. 224: 0 → 1
Char. 241: 0 → 1

***Scutosaurus* spp.:**
Char. 175: 0 → 1
Char. 218: 0 → 1
Char. 243: 0 → 2
Char. 244: 0 → 1
Char. 251: 0 → 1

***Sinosaurosphargis yunguiensis*:**
Char. 8: 0 → 1
Char. 30: 0 → 1

Char. 53: 0 → 1
Char. 82: 0 → 1
Char. 89: 1 → 0
Char. 127: 1 → 0
Char. 150: 1 → 0
Char. 154: 0 → 2
Char. 167: 1 → 0
Char. 253: 0 → 1
Char. 255: 0 → 1

**Squamata:**
Char. 26: 0 → 1
Char. 45: 0 → 1
Char. 79: 0 → 2
Char. 80: 1 → 0
Char. 82: 0 → 1
Char. 92: 1 → 0
Char. 109: 1 → 0
Char. 160: 0 → 1
Char. 245: 0 → 1

***Trilophosaurus buettneri*:**
Char. 5: 1 → 0
Char. 11: 0 → 1
Char. 55: 1 → 0
Char. 93: 1 → 0
Char. 104: 0 → 1
Char. 113: 0 → 1
Char. 122: 0 → 1
Char. 144: 1 → 0
Char. 154: 0 → 1
Char. 157: 0 → 1
Char. 159: 1 → 0
Char. 177: 0 → 12
Char. 194: 0 → 1
Char. 203: 1 → 2
Char. 207: 1 → 0
Char. 208: 1 → 0
Char. 272: 1 → 0

***Youngina capensis*:**
Char. 0: 0 → 1
Char. 5: 1 → 0
Char. 21: 0 → 1
Char. 27: 0 → 1
Char. 38: 1 → 0
Char. 40: 2 → 0
Char. 43: 0 → 1
Char. 44: 1 → 0
Char. 50: 1 → 0
Char. 56: 0 → 1
Char. 59: 0 → 1
Char. 60: 0 → 1
Char. 62: 0 → 1
Char. 67: 0 → 1
Char. 72: 1 → 2
Char. 75: 0 → 1
Char. 84: 1 → 0
Char. 89: 0 → 1
Char. 109: 0 → 1
Char. 127: 0 → 1
Char. 129: 0 → 1
Char. 134: 0 → 1
Char. 135: 0 → 1

Char. 141: 0 → 1
Char. 154: 1 → 0
Char. 163: 1 → 0
Char. 170: 0 → 1
Char. 179: 0 → 1
Char. 187: 0 → 1
Char. 191: 0 → 1
Char. 193: 0 → 1
Char. 196: 0 → 1
Char. 201: 1 → 0
Char. 211: 0 → 1
Char. 214: 0 → 1
Char. 215: 0 → 1
Char. 219: 0 → 1
Char. 224: 0 → 1
Char. 231: 0 → 1
Char. 239: 0 → 1
Char. 265: 0 → 1
Char. 267: 0 → 1
Char. 275: 1 → 0
Char. 278: 3 → 0

**Node 50:**
Char. 46: 1 → 0
Char. 88: 1 → 0
Char. 93: 1 → 0
Char. 176: 0 → 1
Char. 195: 0 → 2
Char. 198: 0 → 1
Char. 246: 1 → 2
Char. 259: 0 → 1
Char. 270: 0 → 1

**Node 51:**
Char. 65: 0 → 1
Char. 72: 1 → 2
Char. 131: 0 → 1
Char. 184: 0 → 1
Char. 205: 0 → 1
Char. 210: 0 → 1
Char. 241: 0 → 1
Char. 246: 0 → 1
Char. 254: 0 → 1
Char. 255: 0 → 1
Char. 256: 0 → 1
Char. 267: 0 → 1
Char. 268: 0 → 1
Char. 269: 0 → 1
Char. 278: 3 → 0

**Node 52:**
Char. 15: 0 → 1
Char. 21: 0 → 1
Char. 25: 1 → 0
Char. 33: 01 → 2
Char. 44: 1 → 0
Char. 64: 0 → 1
Char. 73: 0 → 1
Char. 84: 1 → 2
Char. 94: 0 → 1
Char. 127: 0 → 1
Char. 130: 0 → 1
Char. 134: 0 → 2
Char. 135: 0 → 1

Char. 136: 0 → 1
Char. 141: 0 → 1
Char. 145: 0 → 1
Char. 147: 0 → 1
Char. 148: 0 → 1
Char. 151: 0 → 1
Char. 152: 0 → 1
Char. 155: 0 → 1
Char. 158: 0 → 1
Char. 161: 0 → 1
Char. 174: 0 → 1
Char. 181: 0 → 1
Char. 188: 0 → 1
Char. 196: 0 → 1
Char. 201: 1 → 0
Char. 203: 1 → 2
Char. 204: 0 → 2
Char. 217: 0 → 1
Char. 220: 0 → 1
Char. 231: 0 → 1
Char. 247: 0 → 1
Char. 251: 0 → 1
Char. 252: 0 → 1
Char. 253: 0 → 2

**Node 53:**
Char. 5: 0 → 1
Char. 20: 0 → 1
Char. 40: 0 → 2
Char. 57: 1 → 0
Char. 74: 0 → 1
Char. 80: 0 → 1
Char. 84: 0 → 1
Char. 88: 0 → 1
Char. 116: 0 → 1
Char. 119: 0 → 1
Char. 132: 0 → 1
Char. 149: 0 → 1
Char. 156: 0 → 1
Char. 163: 0 → 1
Char. 166: 0 → 1
Char. 169: 0 → 1
Char. 180: 0 → 1
Char. 192: 0 → 1
Char. 201: 0 → 1
Char. 202: 0 → 1
Char. 203: 0 → 1
Char. 209: 0 → 1
Char. 221: 0 → 1
Char. 234: 0 → 1
Char. 235: 0 → 1

**Node 54:**
Char. 72: 0 → 1
Char. 79: 1 → 0
Char. 81: 0 → 1
Char. 93: 0 → 1
Char. 97: 0 → 1
Char. 104: 1 → 0
Char. 144: 0 → 1
Char. 173: 0 → 1
Char. 183: 0 → 1

**Node 56:**
Char. 19: 0 → 1
Char. 92: 1 → 0

**Node 57:**
Char. 4: 0 → 1
Char. 15: 0 → 1
Char. 29: 0 → 1
Char. 213: 0 → 1
Char. 226: 0 → 2
Char. 228: 0 → 1

**Node 58:**
Char. 43: 0 → 1
Char. 75: 0 → 1
Char. 107: 0 → 1
Char. 140: 0 → 1
Char. 147: 0 → 1

**Node 59:**
Char. 58: 0 → 1
Char. 61: 0 → 1
Char. 66: 0 → 1
Char. 69: 0 → 1
Char. 127: 0 → 1
Char. 141: 0 → 1
Char. 150: 0 → 1
Char. 167: 0 → 1
Char. 205: 0 → 1
Char. 208: 0 → 1
Char. 239: 0 → 1

**Node 60:**
Char. 0: 0 → 1
Char. 23: 0 → 1
Char. 50: 1 → 0
Char. 59: 0 → 1
Char. 60: 0 → 1
Char. 62: 0 → 1
Char. 67: 0 → 1
Char. 70: 0 → 1
Char. 72: 1 → 2
Char. 73: 0 → 1
Char. 89: 0 → 1
Char. 94: 0 → 1
Char. 109: 0 → 1
Char. 117: 0 → 12
Char. 126: 0 → 1
Char. 129: 0 → 1
Char. 131: 0 → 1
Char. 154: 1 → 0
Char. 179: 0 → 1
Char. 182: 0 → 1
Char. 188: 0 → 1
Char. 190: 0 → 1
Char. 191: 0 → 1
Char. 196: 0 → 1
Char. 214: 0 → 1
Char. 219: 0 → 1
Char. 265: 0 → 1
Char. 267: 0 → 1

**Node 61:**
Char. 73: 0 → 1
Char. 131: 1 → 0

Char. 205: 0 → 1
Char. 276: 0 → 1

**Node 62:**
Char. 18: 0 → 1
Char. 37: 0 → 1
Char. 100: 1 → 0
Char. 107: 0 → 1
Char. 113: 1 → 0
Char. 118: 1 → 0
Char. 125: 1 → 0
Char. 150: 0 → 1

**Node 63:**
Char. 23: 0 → 1
Char. 71: 1 → 0
Char. 85: 1 → 0
Char. 102: 0 → 1
Char. 103: 0 → 1
Char. 106: 0 → 1
Char. 167: 0 → 1
Char. 214: 0 → 1
Char. 216: 1 → 0
Char. 235: 0 → 2
Char. 241: 0 → 1

**Node 64:**
Char. 100: 0 → 1
Char. 113: 0 → 1
Char. 132: 1 → 0
Char. 138: 0 → 1
Char. 141: 0 → 1

**Node 65:**
Char. 38: 1 → 2
Char. 39: 0 → 1
Char. 44: 1 → 0
Char. 49: 0 → 1
Char. 51: 0 → 1
Char. 57: 0 → 1
Char. 66: 0 → 1
Char. 70: 0 → 1
Char. 76: 0 → 1
Char. 80: 1 → 0
Char. 88: 1 → 0
Char. 95: 0 → 1
Char. 117: 0 → 1
Char. 118: 0 → 1
Char. 120: 1 → 0
Char. 125: 0 → 1
Char. 131: 0 → 1
Char. 137: 0 → 1
Char. 147: 0 → 1
Char. 148: 0 → 1
Char. 158: 0 → 1
Char. 183: 1 → 2
Char. 194: 0 → 1
Char. 201: 1 → 0
Char. 211: 0 → 1
Char. 234: 1 → 0
Char. 235: 1 → 0
Char. 240: 1 → 0
Char. 252: 0 → 1

**Node 66:**
Char. 87: 0 → 1

**Node 67:**
Char. 25: 1 → 0
Char. 76: 1 → 0

**Node 68:**
Char. 79: 0 → 1
Char. 93: 1 → 0
Char. 133: 0 → 1

**Node 69:**
Char. 20: 1 → 0
Char. 38: 1 → 2
Char. 39: 0 → 1
Char. 50: 1 → 0
Char. 57: 0 → 1
Char. 58: 0 → 1
Char. 59: 0 → 1
Char. 60: 0 → 1
Char. 72: 1 → 2
Char. 83: 0 → 1
Char. 85: 1 → 0
Char. 88: 1 → 0
Char. 95: 0 → 1
Char. 104: 0 → 1
Char. 105: 0 → 1
Char. 106: 0 → 2
Char. 107: 0 → 1
Char. 109: 0 → 1
Char. 110: 0 → 1
Char. 146: 1 → 0
Char. 148: 0 → 1
Char. 155: 0 → 2
Char. 183: 1 → 2

**Node 70:**
Char. 0: 0 → 2
Char. 33: 1 → 0
Char. 38: 2 → 1
Char. 39: 1 → 0
Char. 42: 0 → 1
Char. 43: 0 → 1
Char. 46: 1 → 0
Char. 48: 1 → 0
Char. 49: 1 → 0
Char. 52: 0 → 1
Char. 83: 0 → 2
Char. 84: 1 → 0
Char. 87: 0 → 1
Char. 93: 1 → 0
Char. 132: 0 → 1
Char. 161: 0 → 1
Char. 163: 1 → 0
Char. 172: 0 → 2
Char. 174: 0 → 1
Char. 188: 0 → 1
Char. 189: 0 → 1
Char. 195: 0 → 1
Char. 204: 0 → 2
Char. 212: 0 → 1
Char. 221: 1 → 0
Char. 236: 0 → 1
Char. 238: 0 → 2
Char. 242: 0 → 1
Char. 245: 0 → 1
Char. 274: 0 → 1
Char. 275: 1 → 0

**Node 71:**
Char. 5: 1 → 0
Char. 20: 1 → 0
Char. 44: 1 → 0
Char. 59: 0 → 1
Char. 60: 0 → 1
Char. 66: 0 → 1
Char. 67: 0 → 1
Char. 72: 1 → 2
Char. 78: 1 → 0
Char. 84: 1 → 0
Char. 88: 1 → 0
Char. 93: 1 → 0
Char. 123: 1 → 0
Char. 129: 0 → 1
Char. 154: 1 → 0
Char. 159: 1 → 0
Char. 163: 1 → 0
Char. 169: 1 → 0
Char. 170: 0 → 1
Char. 192: 1 → 0
Char. 197: 0 → 1
Char. 202: 1 → 0
Char. 207: 1 → 0
Char. 209: 1 → 0
Char. 221: 1 → 0
Char. 234: 1 → 0

**Node 72:**
Char. 47: 0 → 1
Char. 57: 0 → 1
Char. 83: 0 → 1
Char. 85: 1 → 0
Char. 107: 0 → 1
Char. 110: 0 → 1
Char. 169: 1 → 0
Char. 170: 0 → 1

**Node 73:**
Char. 2: 0 → 1
Char. 6: 0 → 1
Char. 98: 1 → 0
Char. 101: 0 → 1
Char. 113: 0 → 1
Char. 181: 0 → 1
Char. 186: 0 → 1
Char. 198: 0 → 1
Char. 206: 0 → 2
Char. 220: 0 → 1
Char. 239: 1 → 0
Char. 240: 1 → 0

**Node 74:**
Char. 33: 1 → 2
Char. 68: 0 → 1
Char. 102: 0 → 1
Char. 103: 0 → 1
Char. 223: 0 → 1
Char. 229: 0 → 1
Char. 235: 1 → 2

**Node 75:**
Char. 17: 0 → 1
Char. 42: 0 → 1
Char. 251: 0 → 1
Char. 264: 0 → 1

**Node 76:**
Char. 41: 0 → 1
Char. 61: 1 → 2
Char. 112: 1 → 0
Char. 128: 0 → 1
Char. 138: 0 → 1
Char. 146: 1 → 0
Char. 155: 0 → 1
Char. 182: 1 → 0
Char. 192: 1 → 2
Char. 224: 0 → 1
Char. 226: 0 → 1
Char. 227: 0 → 1
Char. 233: 0 → 1

**Node 77:**
Char. 61: 1 → 3
Char. 90: 0 → 1
Char. 91: 0 → 1
Char. 109: 1 → 0
Char. 155: 0 → 1
Char. 209: 1 → 0

**ANALYSIS 2**
**(NO *PROGANOCHELYS QUENSTEDTI*)**

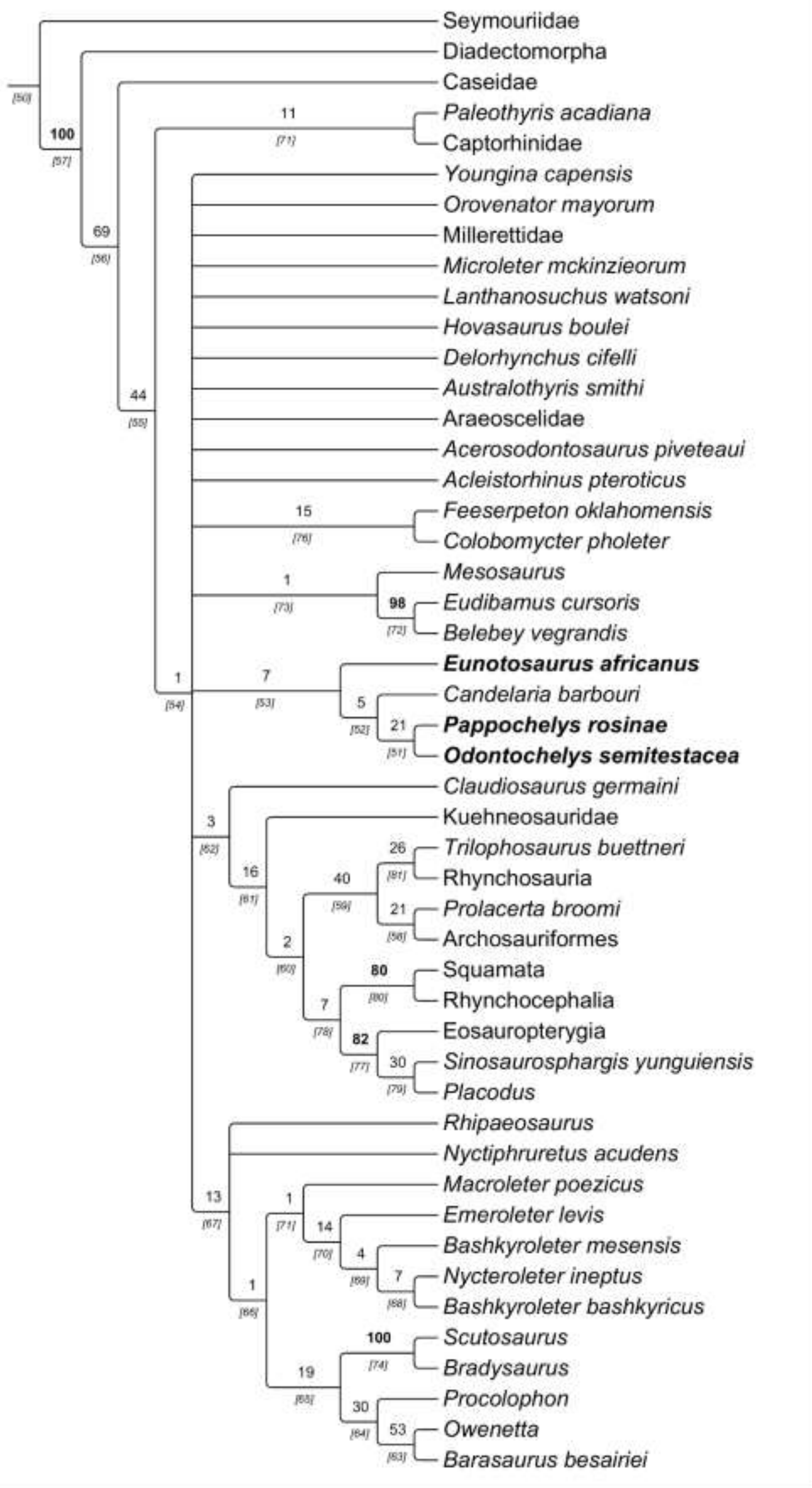

**Seymouriidae:**
Char. 23: 0 → 1
Char. 51: 0 → 2
Char. 54: 1 → 0
Char. 55: 1 → 0
Char. 83: 0 → 1
Char. 85: 1 → 0
Char. 99: 1 → 0
Char. 107: 0 → 1
Char. 140: 0 → 2
Char. 154: 1 → 2
Char. 207: 0 → 1
Char. 225: 1 → 0
Char. 260: 2 → 0

***Pappochelys rosinae*:**
Char. 12: 0 → 1
Char. 41: 0 → 1
Char. 75: 0 → 1
Char. 260: 2 → 0
Char. 265: 0 → 1

***Odontochelys semitestacea*:**
Char. 0: 0 → 2
Char. 43: 0 → 1
Char. 46: 1 → 0
Char. 89: 1 → 0
Char. 93: 1 → 0
Char. 176: 0 → 1
Char. 195: 0 → 2
Char. 198: 0 → 1
Char. 246: 1 → 2
Char. 259: 0 → 1
Char. 270: 0 → 1

***Eunotosaurus africanus*:**
Char. 0: 0 → 2
Char. 19: 0 → 1
Char. 21: 0 → 1
Char. 24: 0 → 1
Char. 42: 0 → 1
Char. 43: 0 → 1
Char. 57: 0 → 1
Char. 81: 1 → 0
Char. 84: 1 → 2
Char. 192: 1 → 0
Char. 202: 1 → 0
Char. 211: 0 → 1
Char. 219: 0 → 1
Char. 222: 1 → 0
Char. 237: 1 → 0
Char. 248: 0 → 1
Char. 250: 0 → 1
Char. 263: 0 → 1
Char. 273: 0 → 1
Char. 274: 0 → 1

***Acerosodontosaurus piveteaui*:**
Char. 23: 0 → 1
Char. 50: 1 → 0
Char. 59: 0 → 1
Char. 78: 1 → 0
Char. 81: 1 → 0
Char. 89: 0 → 1
Char. 94: 0 → 1
Char. 128: 0 → 1
Char. 129: 0 → 1
Char. 155: 0 → 1
Char. 206: 0 → 2
Char. 208: 0 → 1
Char. 265: 0 → 1
Char. 266: 0 → 1
Char. 278: 3 → 1

***Acleistorhinus pteroticus*:**
Char. 21: 0 → 1
Char. 30: 0 → 1
Char. 47: 0 → 1
Char. 55: 1 → 0
Char. 56: 0 → 1
Char. 57: 0 → 1
Char. 64: 0 → 1
Char. 70: 0 → 1
Char. 79: 0 → 1
Char. 95: 0 → 1
Char. 110: 0 → 1
Char. 112: 1 → 0
Char. 113: 0 → 1
Char. 114: 0 → 1
Char. 120: 1 → 0
Char. 137: 0 → 1
Char. 140: 0 → 2
Char. 146: 1 → 0
Char. 147: 0 → 1
Char. 169: 1 → 0
Char. 170: 0 → 1
Char. 278: 3 → 1

**Araeoscelidae:**
Char. 0: 0 → 1
Char. 5: 1 → 0
Char. 20: 1 → 0
Char. 23: 0 → 1
Char. 27: 0 → 1
Char. 28: 0 → 1
Char. 29: 1 → 0
Char. 38: 1 → 0
Char. 40: 2 → 0
Char. 43: 0 → 1
Char. 50: 1 → 0
Char. 59: 0 → 1
Char. 60: 0 → 1
Char. 67: 0 → 1
Char. 72: 1 → 2
Char. 84: 1 → 0
Char. 89: 0 → 1
Char. 92: 1 → 0
Char. 106: 0 → 1
Char. 116: 1 → 0
Char. 117: 0 → 2
Char. 154: 1 → 0
Char. 159: 1 → 0
Char. 166: 1 → 0
Char. 169: 1 → 0
Char. 170: 0 → 1
Char. 187: 1 → 0
Char. 193: 0 → 1
Char. 197: 0 → 1
Char. 202: 1 → 0
Char. 207: 1 → 0
Char. 209: 1 → 0
Char. 221: 1 → 0
Char. 224: 0 → 1
Char. 239: 0 → 1
Char. 266: 0 → 1
Char. 278: 3 → 1

**Archosauriformes:**
Char. 32: 0 → 1
Char. 94: 1 → 0
Char. 112: 1 → 0
Char. 152: 0 → 1
Char. 154: 0 → 2
Char. 166: 1 → 0
Char. 171: 0 → 1
Char. 185: 0 → 1
Char. 204: 0 → 1
Char. 218: 0 → 3
Char. 224: 0 → 1
Char. 242: 0 → 1

***Australothyris smithi*:**
Char. 23: 0 → 1
Char. 24: 0 → 1
Char. 34: 0 → 1
Char. 55: 1 → 0
Char. 71: 1 → 0
Char. 79: 0 → 1
Char. 83: 0 → 1
Char. 85: 1 → 0
Char. 98: 1 → 0
Char. 100: 0 → 1
Char. 103: 0 → 1
Char. 110: 0 → 1
Char. 123: 1 → 0
Char. 129: 0 → 1
Char. 132: 1 → 0
Char. 144: 1 → 0
Char. 147: 0 → 1
Char. 149: 1 → 0
Char. 150: 0 → 1
Char. 159: 1 → 0
Char. 163: 1 → 0
Char. 192: 1 → 0

***Barasaurus besairiei*:**
Char. 33: 1 → 0
Char. 75: 1 → 0
Char. 216: 0 → 1

***Bashkyroleter bashkyricus*:**
Char. 275: 1 → 0

***Bashkyroleter mesensis*:**
Char. 169: 1 → 0

***Belebey vegrandis*:**
Char. 154: 1 → 0

***Bradysaurus* spp.:**
Char. 19: 0 → 1
Char. 73: 0 → 1
Char. 79: 0 → 1
Char. 135: 1 → 0
Char. 249: 0 → 1

***Candelaria barbouri*:**
Char. 8: 0 → 1
Char. 67: 0 → 1
Char. 79: 0 → 2
Char. 92: 1 → 0
Char. 95: 0 → 1
Char. 132: 1 → 0
Char. 154: 1 → 2

**Captorhinidae:**
Char. 3: 0 → 1
Char. 23: 0 → 1
Char. 25: 1 → 0
Char. 26: 0 → 1
Char. 73: 0 → 1
Char. 75: 0 → 1
Char. 83: 0 → 1
Char. 108: 1 → 0
Char. 183: 1 → 0
Char. 203: 1 → 2

**Caseidae:**
Char. 24: 0 → 1
Char. 25: 1 → 0
Char. 36: 0 → 1
Char. 38: 1 → 0
Char. 46: 1 → 0
Char. 50: 1 → 0
Char. 56: 0 → 1
Char. 85: 1 → 0
Char. 98: 1 → 0
Char. 194: 0 → 1
Char. 273: 0 → 1
Char. 274: 0 → 1

***Claudiosaurus germaini*:**
Char. 64: 0 → 1
Char. 84: 1 → 0
Char. 105: 0 → 1
Char. 130: 0 → 1
Char. 144: 1 → 0
Char. 166: 1 → 0
Char. 187: 1 → 0
Char. 199: 0 → 1
Char. 203: 1 → 2
Char. 220: 0 → 1
Char. 222: 1 → 0

***Colobomycter pholeter*:**
Char. 21: 0 → 1
Char. 25: 1 → 0
Char. 84: 1 → 0
Char. 154: 1 → 0
Char. 167: 0 → 1
Char. 267: 0 → 1

***Delorhynchus cifelli*:**
Char. 18: 0 → 1
Char. 21: 0 → 1
Char. 24: 0 → 1
Char. 26: 0 → 1
Char. 28: 0 → 1
Char. 33: 01 → 2
Char. 39: 0 → 1
Char. 52: 0 → 1
Char. 57: 0 → 1
Char. 100: 0 → 1
Char. 116: 1 → 0
Char. 117: 0 → 1
Char. 119: 1 → 0
Char. 147: 0 → 1
Char. 156: 1 → 0
Char. 167: 0 → 1
Char. 189: 0 → 1
Char. 191: 0 → 1
Char. 202: 1 → 0
Char. 267: 0 → 1

**Diadectomorpha:**
Char. 0: 0 → 1
Char. 44: 0 → 1
Char. 70: 0 → 1
Char. 75: 0 → 1
Char. 122: 0 → 1
Char. 146: 1 → 0

***Emeroleter levis*:**
Char. 0: 0 → 2
Char. 51: 1 → 0

**Eosauropterygia:**
Char. 159: 1 → 0
Char. 166: 1 → 0
Char. 174: 0 → 1
Char. 194: 0 → 2
Char. 272: 1 → 0

***Eudibamus cursoris*:**
Char. 154: 1 → 2

***Feeserpeton oklahomensis*:**
Char. 51: 0 → 1
Char. 70: 0 → 1
Char. 157: 0 → 1
Char. 158: 0 → 1

***Hovasaurus boulei*:**
Char. 41: 0 → 1
Char. 43: 0 → 1
Char. 50: 1 → 0
Char. 55: 1 → 0
Char. 59: 0 → 1
Char. 67: 0 → 1
Char. 77: 0 → 2
Char. 78: 1 → 0
Char. 79: 0 → 1
Char. 89: 0 → 1
Char. 93: 1 → 0
Char. 113: 0 → 1
Char. 127: 0 → 1
Char. 135: 0 → 1
Char. 138: 0 → 1
Char. 146: 1 → 0
Char. 188: 0 → 1
Char. 191: 0 → 1

Char. 193: 0 → 1
Char. 206: 0 → 2
Char. 215: 0 → 1
Char. 220: 0 → 1
Char. 224: 0 → 1
Char. 265: 0 → 1
Char. 275: 1 → 0

**Kuehneosauridae:**
Char. 7: 0 → 1
Char. 26: 0 → 1
Char. 44: 1 → 0
Char. 79: 0 → 2
Char. 82: 0 → 1
Char. 98: 1 → 0
Char. 108: 1 → 0
Char. 113: 0 → 1
Char. 128: 0 → 1
Char. 159: 1 → 0
Char. 181: 0 → 1
Char. 185: 0 → 1
Char. 206: 0 → 2
Char. 245: 0 → 1

***Lanthanosuchus watsoni*:**
Char. 25: 1 → 0
Char. 51: 0 → 1
Char. 76: 0 → 1
Char. 86: 0 → 1
Char. 95: 0 → 1
Char. 98: 1 → 0
Char. 110: 0 → 1
Char. 113: 0 → 1
Char. 114: 0 → 1
Char. 119: 1 → 0
Char. 137: 0 → 1
Char. 138: 0 → 1
Char. 140: 0 → 2
Char. 144: 1 → 0
Char. 147: 0 → 1
Char. 154: 1 → 2

***Macroleter poezicus*:**
Char. 0: 0 → 1
Char. 52: 0 → 1
Char. 66: 1 → 2
Char. 84: 1 → 0
Char. 87: 0 → 1
Char. 140: 0 → 1
Char. 146: 1 → 0
Char. 169: 1 → 0
Char. 235: 0 → 1

***Mesosaurus* spp.:**
Char. 2: 0 → 1
Char. 5: 1 → 0
Char. 6: 0 → 1
Char. 8: 0 → 1
Char. 9: 0 → 1
Char. 13: 0 → 1
Char. 19: 0 → 1
Char. 29: 1 → 0
Char. 41: 0 → 1
Char. 84: 1 → 0
Char. 94: 0 → 1
Char. 112: 1 → 0
Char. 115: 0 → 1
Char. 149: 1 → 0
Char. 159: 1 → 0
Char. 164: 0 → 1
Char. 166: 1 → 0
Char. 167: 0 → 1
Char. 192: 1 → 0
Char. 222: 1 → 0
Char. 231: 0 → 1
Char. 234: 1 → 0
Char. 240: 1 → 0
Char. 272: 2 → 0

***Microleter mckinzieorum*:**
Char. 0: 0 → 1
Char. 18: 0 → 1
Char. 20: 1 → 0
Char. 24: 0 → 1
Char. 25: 1 → 0
Char. 36: 0 → 1
Char. 39: 0 → 1
Char. 48: 1 → 0
Char. 51: 0 → 2
Char. 56: 0 → 1
Char. 70: 0 → 1
Char. 76: 0 → 1
Char. 79: 0 → 1
Char. 83: 0 → 1
Char. 94: 0 → 1
Char. 106: 0 → 1
Char. 110: 0 → 1
Char. 112: 1 → 0
Char. 132: 1 → 0
Char. 166: 1 → 0
Char. 276: 0 → 1
Char. 278: 3 → 1

**Millerettidae:**
Char. 5: 1 → 0
Char. 20: 1 → 0
Char. 24: 0 → 1
Char. 25: 1 → 0
Char. 44: 1 → 0
Char. 48: 1 → 0
Char. 56: 0 → 1
Char. 66: 0 → 2
Char. 78: 1 → 0
Char. 80: 1 → 0
Char. 84: 1 → 2
Char. 88: 1 → 0
Char. 96: 1 → 0
Char. 112: 1 → 0
Char. 117: 0 → 1
Char. 120: 1 → 0
Char. 121: 0 → 1
Char. 124: 0 → 1
Char. 127: 0 → 1
Char. 135: 0 → 1
Char. 145: 0 → 1
Char. 159: 1 → 0
Char. 163: 1 → 0
Char. 166: 1 → 0
Char. 180: 1 → 0
Char. 192: 1 → 0
Char. 211: 0 → 1
Char. 222: 1 → 0
Char. 230: 0 → 1
Char. 248: 0 → 1
Char. 252: 0 → 1
Char. 253: 0 → 1

***Nycteroleter ineptus*:**
Char. 278: 0 → 3

***Nyctiphruretus acudens*:**
Char. 0: 0 → 1
Char. 21: 0 → 1
Char. 33: 1 → 2
Char. 41: 0 → 1
Char. 48: 1 → 0
Char. 66: 1 → 2
Char. 81: 1 → 0
Char. 83: 0 → 1
Char. 84: 1 → 2
Char. 94: 0 → 1
Char. 166: 1 → 0
Char. 167: 0 → 1
Char. 224: 0 → 1
Char. 266: 0 → 1
Char. 272: 2 → 1
Char. 276: 0 → 1

***Orovenator mayorum*:**
Char. 0: 0 → 1
Char. 8: 0 → 1
Char. 24: 0 → 1
Char. 25: 1 → 0
Char. 29: 1 → 0
Char. 36: 0 → 1
Char. 48: 1 → 0
Char. 62: 0 → 1
Char. 72: 1 → 2
Char. 89: 0 → 1
Char. 92: 1 → 0
Char. 141: 0 → 1
Char. 159: 1 → 0
Char. 160: 0 → 1
Char. 165: 0 → 1
Char. 278: 3 → 1

***Owenetta* spp.:**
Char. 169: 1 → 0

***Paleothyris acadiana*:**
Char. 38: 1 → 0
Char. 50: 1 → 0
Char. 66: 1 → 2
Char. 102: 0 → 1
Char. 146: 1 → 0
Char. 239: 0 → 1

***Placodus* spp.:**
Char. 0: 1 → 2
Char. 9: 0 → 1
Char. 12: 0 → 1
Char. 13: 0 → 1
Char. 19: 0 → 1
Char. 26: 0 → 1
Char. 31: 0 → 1
Char. 44: 1 → 0
Char. 46: 1 → 0
Char. 57: 0 → 1
Char. 78: 1 → 0
Char. 93: 1 → 0
Char. 102: 1 → 2
Char. 109: 1 → 0
Char. 140: 1 → 0
Char. 155: 0 → 1
Char. 163: 1 → 0
Char. 164: 0 → 1

***Procolophon* spp.:**
Char. 41: 0 → 1
Char. 69: 0 → 1
Char. 79: 0 → 1
Char. 83: 0 → 1
Char. 88: 0 → 1
Char. 117: 1 → 0
Char. 149: 1 → 0
Char. 237: 0 → 1
Char. 238: 0 → 1
Char. 272: 2 → 1
Char. 278: 3 → 0

***Prolacerta broomi*:**
Char. 58: 1 → 0
Char. 66: 1 → 0
Char. 67: 1 → 0
Char. 80: 1 → 0
Char. 139: 1 → 0
Char. 147: 1 → 0
Char. 192: 1 → 0
Char. 203: 1 → 2
Char. 206: 0 → 12

***Rhipaeosaurus* spp.:**
Char. 172: 0 → 1
Char. 277: 0 → 1

**Rhynchocephalia:**
Char. 0: 1 → 2
Char. 24: 0 → 1
Char. 75: 1 → 0
Char. 77: 0 → 1
Char. 94: 1 → 0
Char. 117: 12 → 0
Char. 139: 1 → 0
Char. 167: 1 → 0
Char. 205: 1 → 0

**Rhynchosauria:**
Char. 0: 1 → 0
Char. 7: 0 → 1
Char. 9: 0 → 1
Char. 26: 0 → 1
Char. 44: 1 → 0
Char. 68: 0 → 1
Char. 99: 1 → 0
Char. 150: 1 → 0
Char. 160: 0 → 1
Char. 161: 0 → 1
Char. 171: 0 → 2
Char. 182: 1 → 0
Char. 223: 0 → 1
Char. 224: 0 → 1
Char. 241: 0 → 1

***Scutosaurus* spp.:**
Char. 175: 0 → 1
Char. 218: 0 → 1
Char. 243: 0 → 2
Char. 244: 0 → 1
Char. 251: 0 → 1

***Sinosaurosphargis yunguiensis*:**
Char. 8: 0 → 1
Char. 30: 0 → 1
Char. 53: 0 → 1
Char. 82: 0 → 1
Char. 89: 1 → 0
Char. 127: 1 → 0
Char. 150: 1 → 0
Char. 154: 0 → 2
Char. 167: 1 → 0
Char. 253: 0 → 1
Char. 255: 0 → 1

**Squamata:**
Char. 26: 0 → 1
Char. 45: 0 → 1
Char. 79: 0 → 2
Char. 80: 1 → 0
Char. 82: 0 → 1
Char. 92: 1 → 0
Char. 109: 1 → 0
Char. 160: 0 → 1
Char. 245: 0 → 1

***Trilophosaurus buettneri*:**
Char. 5: 1 → 0
Char. 11: 0 → 1
Char. 55: 1 → 0
Char. 93: 1 → 0
Char. 104: 0 → 1
Char. 113: 0 → 1
Char. 122: 0 → 1
Char. 144: 1 → 0
Char. 154: 0 → 1
Char. 157: 0 → 1
Char. 159: 1 → 0
Char. 177: 0 → 12
Char. 194: 0 → 1
Char. 203: 1 → 2
Char. 207: 1 → 0
Char. 208: 1 → 0
Char. 272: 1 → 0

***Youngina capensis*:**
Char. 0: 0 → 1
Char. 5: 1 → 0
Char. 21: 0 → 1
Char. 27: 0 → 1
Char. 29: 1 → 0
Char. 38: 1 → 0
Char. 40: 2 → 0
Char. 43: 0 → 1

Char. 44: 1 → 0
Char. 50: 1 → 0
Char. 56: 0 → 1
Char. 59: 0 → 1
Char. 60: 0 → 1
Char. 62: 0 → 1
Char. 67: 0 → 1
Char. 72: 1 → 2
Char. 75: 0 → 1
Char. 84: 1 → 0
Char. 89: 0 → 1
Char. 92: 1 → 0
Char. 109: 0 → 1
Char. 127: 0 → 1
Char. 129: 0 → 1
Char. 134: 0 → 1
Char. 135: 0 → 1
Char. 141: 0 → 1
Char. 154: 1 → 0
Char. 163: 1 → 0
Char. 170: 0 → 1
Char. 179: 0 → 1
Char. 191: 0 → 1
Char. 193: 0 → 1
Char. 211: 0 → 1
Char. 214: 0 → 1
Char. 215: 0 → 1
Char. 219: 0 → 1
Char. 224: 0 → 1
Char. 231: 0 → 1
Char. 239: 0 → 1
Char. 265: 0 → 1
Char. 267: 0 → 1
Char. 275: 1 → 0
Char. 278: 3 → 0

**Node 51:**
Char. 65: 0 → 1
Char. 267: 0 → 1

**Node 52:**
Char. 23: 0 → 1

**Node 53:**
Char. 15: 0 → 1
Char. 25: 1 → 0
Char. 29: 1 → 0
Char. 33: 01 → 2
Char. 44: 1 → 0
Char. 55: 1 → 0
Char. 73: 0 → 1
Char. 89: 0 → 1
Char. 94: 0 → 1
Char. 112: 1 → 0
Char. 124: 0 → 1
Char. 127: 0 → 1
Char. 145: 0 → 1
Char. 147: 0 → 1
Char. 148: 0 → 1
Char. 174: 0 → 1
Char. 188: 0 → 1
Char. 217: 0 → 1
Char. 220: 0 → 1
Char. 231: 0 → 1
Char. 247: 0 → 1
Char. 251: 0 → 1
Char. 252: 0 → 1
Char. 253: 0 → 2
Char. 277: 0 → 1

**Node 54:**
Char. 5: 0 → 1
Char. 20: 0 → 1
Char. 29: 0 → 1
Char. 44: 0 → 1
Char. 48: 0 → 1
Char. 57: 1 → 0
Char. 84: 0 → 1
Char. 88: 0 → 1
Char. 112: 0 → 1
Char. 116: 0 → 1
Char. 121: 1 → 0
Char. 154: 0 → 1
Char. 159: 0 → 1
Char. 163: 0 → 1
Char. 166: 0 → 1
Char. 169: 0 → 1
Char. 187: 0 → 1
Char. 192: 0 → 1
Char. 202: 0 → 1
Char. 207: 0 → 1
Char. 209: 0 → 1
Char. 221: 0 → 1
Char. 222: 0 → 1
Char. 234: 0 → 1

**Node 55:**
Char. 74: 0 → 1
Char. 119: 0 → 1
Char. 149: 0 → 1
Char. 203: 0 → 1
Char. 235: 0 → 1

**Node 56:**
Char. 72: 0 → 1
Char. 79: 1 → 0
Char. 81: 0 → 1
Char. 173: 0 → 1

**Node 57:**
Char. 78: 0 → 1
Char. 126: 1 → 0
Char. 135: 1 → 0
Char. 154: 1 → 0

**Node 58:**
Char. 19: 0 → 1
Char. 92: 1 → 0

**Node 59:**
Char. 4: 0 → 1
Char. 15: 0 → 1
Char. 29: 0 → 1
Char. 213: 0 → 1
Char. 226: 0 → 2
Char. 228: 0 → 1

**Node 60:**
Char. 43: 0 → 1
Char. 75: 0 → 1
Char. 107: 0 → 1
Char. 140: 0 → 1
Char. 147: 0 → 1

**Node 61:**
Char. 58: 0 → 1
Char. 61: 0 → 1
Char. 66: 0 → 1
Char. 69: 0 → 1
Char. 127: 0 → 1
Char. 141: 0 → 1
Char. 150: 0 → 1
Char. 167: 0 → 1
Char. 205: 0 → 1
Char. 208: 0 → 1
Char. 239: 0 → 1

**Node 62:**
Char. 0: 0 → 1
Char. 23: 0 → 1
Char. 29: 1 → 0
Char. 50: 1 → 0
Char. 59: 0 → 1
Char. 60: 0 → 1
Char. 62: 0 → 1
Char. 67: 0 → 1
Char. 70: 0 → 1
Char. 72: 1 → 2
Char. 73: 0 → 1
Char. 89: 0 → 1
Char. 94: 0 → 1
Char. 109: 0 → 1
Char. 117: 0 → 12
Char. 126: 0 → 1
Char. 129: 0 → 1
Char. 154: 1 → 0
Char. 179: 0 → 1
Char. 182: 0 → 1
Char. 188: 0 → 1
Char. 190: 0 → 1
Char. 191: 0 → 1
Char. 214: 0 → 1
Char. 219: 0 → 1
Char. 265: 0 → 1
Char. 267: 0 → 1

**Node 63:**
Char. 73: 0 → 1
Char. 131: 1 → 0
Char. 205: 0 → 1
Char. 239: 0 → 1
Char. 276: 0 → 1

**Node 64:**
Char. 18: 0 → 1
Char. 37: 0 → 1
Char. 107: 0 → 1
Char. 118: 1 → 0
Char. 125: 1 → 0
Char. 150: 0 → 1

**Node 65:**
Char. 23: 0 → 1
Char. 71: 1 → 0
Char. 102: 0 → 1
Char. 103: 0 → 1
Char. 106: 0 → 1
Char. 167: 0 → 1
Char. 214: 0 → 1
Char. 216: 1 → 0
Char. 222: 1 → 0
Char. 235: 0 → 2
Char. 241: 0 → 1

**Node 66:**
Char. 138: 0 → 1
Char. 141: 0 → 1

**Node 67:**
Char. 38: 1 → 2
Char. 39: 0 → 1
Char. 44: 1 → 0
Char. 49: 0 → 1
Char. 51: 0 → 1
Char. 57: 0 → 1
Char. 66: 0 → 1
Char. 70: 0 → 1
Char. 76: 0 → 1
Char. 80: 1 → 0
Char. 88: 1 → 0
Char. 95: 0 → 1
Char. 117: 0 → 1
Char. 118: 0 → 1
Char. 120: 1 → 0
Char. 125: 0 → 1
Char. 137: 0 → 1
Char. 147: 0 → 1
Char. 148: 0 → 1
Char. 158: 0 → 1
Char. 183: 1 → 2
Char. 194: 0 → 1
Char. 211: 0 → 1
Char. 234: 1 → 0
Char. 235: 1 → 0
Char. 237: 1 → 0
Char. 240: 1 → 0
Char. 252: 0 → 1

**Node 68:**
Char. 87: 0 → 1

**Node 69:**
Char. 25: 1 → 0
Char. 76: 1 → 0

**Node 70:**
Char. 79: 0 → 1
Char. 93: 1 → 0
Char. 133: 0 → 1

**Node 71:**
Char. 147: 1 → 2
Char. 154: 1 → 0
Char. 239: 0 → 1
Char. 278: 3 → 0

**Node 72:**
Char. 39: 0 → 1
Char. 58: 0 → 1
Char. 59: 0 → 1
Char. 60: 0 → 1
Char. 72: 1 → 2
Char. 88: 1 → 0
Char. 95: 0 → 1
Char. 104: 0 → 1
Char. 105: 0 → 1
Char. 106: 0 → 2
Char. 110: 0 → 1
Char. 155: 0 → 2

**Node 73:**
Char. 0: 0 → 1
Char. 20: 1 → 0
Char. 23: 0 → 1
Char. 26: 0 → 1
Char. 50: 1 → 0
Char. 67: 0 → 1
Char. 76: 0 → 1
Char. 83: 0 → 1
Char. 85: 1 → 0
Char. 107: 0 → 1
Char. 109: 0 → 1
Char. 146: 1 → 0
Char. 148: 0 → 1
Char. 187: 1 → 0
Char. 199: 0 → 1
Char. 237: 1 → 0

**Node 74:**
Char. 0: 0 → 2
Char. 33: 1 → 0
Char. 38: 2 → 1
Char. 39: 1 → 0
Char. 42: 0 → 1
Char. 43: 0 → 1
Char. 46: 1 → 0
Char. 48: 1 → 0
Char. 49: 1 → 0
Char. 52: 0 → 1
Char. 83: 0 → 2
Char. 84: 1 → 0
Char. 87: 0 → 1
Char. 93: 1 → 0
Char. 161: 0 → 1
Char. 163: 1 → 0
Char. 172: 0 → 2
Char. 174: 0 → 1
Char. 188: 0 → 1
Char. 189: 0 → 1
Char. 195: 0 → 1
Char. 212: 0 → 1
Char. 221: 1 → 0
Char. 236: 0 → 1
Char. 238: 0 → 2
Char. 242: 0 → 1
Char. 245: 0 → 1
Char. 274: 0 → 1
Char. 275: 1 → 0

**Node 75:**
Char. 59: 0 → 1
Char. 60: 0 → 1
Char. 66: 0 → 1

Char. 67: 0 → 1
Char. 72: 1 → 2
Char. 78: 1 → 0
Char. 129: 0 → 1
Char. 197: 0 → 1

**Node 76:**
Char. 47: 0 → 1
Char. 57: 0 → 1
Char. 83: 0 → 1
Char. 85: 1 → 0
Char. 107: 0 → 1
Char. 110: 0 → 1

Char. 169: 1 → 0
Char. 170: 0 → 1

**Node 77:**
Char. 2: 0 → 1
Char. 6: 0 → 1
Char. 98: 1 → 0
Char. 101: 0 → 1
Char. 113: 0 → 1
Char. 181: 0 → 1
Char. 186: 0 → 1
Char. 198: 0 → 1
Char. 206: 0 → 2
Char. 220: 0 → 1

Char. 239: 1 → 0
Char. 240: 1 → 0

**Node 78:**
Char. 33: 1 → 2
Char. 68: 0 → 1
Char. 102: 0 → 1
Char. 103: 0 → 1
Char. 223: 0 → 1
Char. 229: 0 → 1
Char. 235: 1 → 2

**Node 79:**
Char. 17: 0 → 1

Char. 42: 0 → 1
Char. 251: 0 → 1
Char. 264: 0 → 1

**Node 80:**
Char. 41: 0 → 1
Char. 61: 1 → 2
Char. 112: 1 → 0
Char. 128: 0 → 1
Char. 138: 0 → 1
Char. 146: 1 → 0
Char. 155: 0 → 1
Char. 182: 1 → 0
Char. 192: 1 → 2

Char. 224: 0 → 1
Char. 226: 0 → 1
Char. 227: 0 → 1
Char. 233: 0 → 1

**Node 81:**
Char. 61: 1 → 3
Char. 90: 0 → 1
Char. 91: 0 → 1
Char. 109: 1 → 0
Char. 155: 0 → 1
Char. 209: 1 → 0

**ANALYSIS 3**
**(NO *CANDELARIA BARBOURI*)**

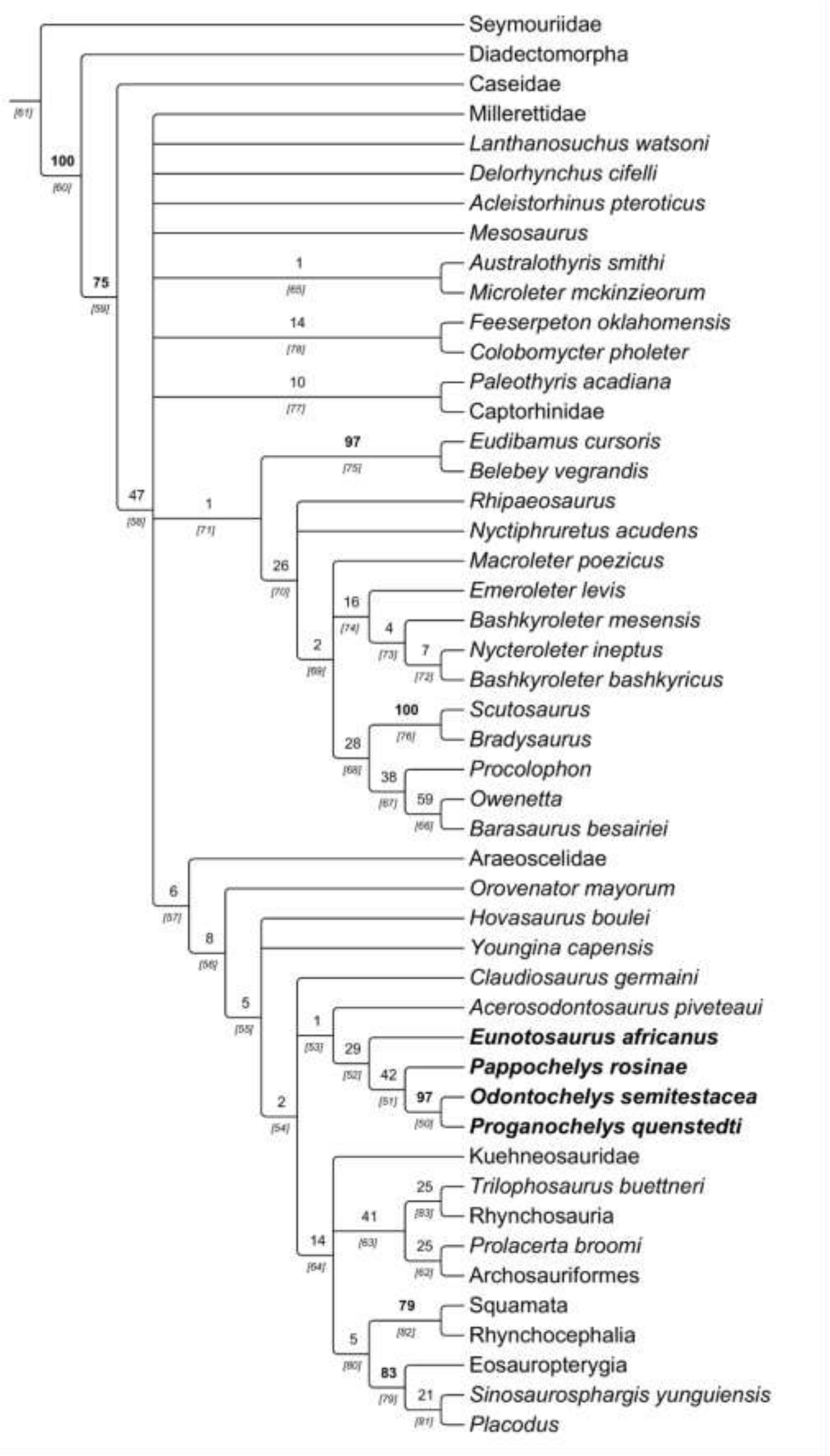

***Proganochelys quenstedti*:**
Char. 8: 0 → 1
Char. 11: 0 → 1
Char. 106: 0 → 1
Char. 108: 1 → 0
Char. 128: 0 → 1
Char. 175: 0 → 1
Char. 202: 1 → 0
Char. 206: 2 → 0
Char. 207: 1 → 0
Char. 209: 1 → 0
Char. 244: 0 → 1
Char. 248: 0 → 1
Char. 250: 0 → 1
Char. 252: 1 → 0
Char. 262: 0 → 1

**Seymouriidae:**
Char. 85: 1 → 0

***Pappochelys rosinae*:**
Char. 0: 2 → 0
Char. 1: 0 → 1
Char. 5: 1 → 0
Char. 12: 0 → 1
Char. 41: 0 → 1
Char. 49: 0 → 1
Char. 75: 0 → 1
Char. 81: 0 → 1
Char. 169: 1 → 0
Char. 260: 2 → 0

***Eunotosaurus africanus*:**
Char. 19: 0 → 1
Char. 59: 1 → 0
Char. 60: 1 → 0
Char. 72: 2 → 1
Char. 76: 0 → 1
Char. 97: 1 → 0
Char. 103: 0 → 1
Char. 131: 1 → 0
Char. 153: 1 → 0
Char. 191: 1 → 0
Char. 192: 1 → 0
Char. 202: 1 → 0
Char. 211: 0 → 1
Char. 222: 1 → 0
Char. 237: 1 → 0
Char. 248: 0 → 1
Char. 249: 0 → 2
Char. 250: 0 → 1
Char. 263: 0 → 1
Char. 272: 1 → 2
Char. 273: 0 → 1
Char. 274: 0 → 1
Char. 275: 0 → 1
Char. 276: 0 → 1
Char. 277: 0 → 1
Char. 278: 0 → 3

***Acerosodontosaurus piveteaui*:**
Char. 127: 1 → 0
Char. 128: 0 → 1
Char. 278: 0 → 1

***Acleistorhinus pteroticus*:**
Char. 20: 0 → 1
Char. 21: 0 → 1
Char. 30: 0 → 1
Char. 33: 0 → 1
Char. 47: 0 → 1
Char. 55: 1 → 0
Char. 56: 0 → 1
Char. 64: 0 → 1
Char. 70: 0 → 1
Char. 79: 0 → 1
Char. 95: 0 → 1
Char. 110: 0 → 1
Char. 113: 0 → 1
Char. 114: 0 → 1
Char. 131: 0 → 1
Char. 137: 0 → 1
Char. 140: 0 → 2
Char. 146: 1 → 0
Char. 159: 0 → 1
Char. 169: 1 → 0
Char. 170: 0 → 1
Char. 278: 3 → 1

**Araeoscelidae:**
Char. 5: 1 → 0
Char. 23: 0 → 1
Char. 28: 0 → 1
Char. 106: 0 → 1
Char. 116: 1 → 0
Char. 117: 01 → 2
Char. 169: 1 → 0
Char. 170: 0 → 1
Char. 197: 0 → 1
Char. 239: 0 → 1

**Archosauriformes:**
Char. 32: 0 → 1
Char. 94: 1 → 0
Char. 112: 1 → 0
Char. 152: 0 → 1
Char. 154: 0 → 2
Char. 166: 1 → 0
Char. 171: 0 → 1
Char. 185: 0 → 1
Char. 204: 0 → 1
Char. 218: 0 → 3
Char. 224: 0 → 1
Char. 242: 0 → 1

***Australothyris smithi*:**
Char. 23: 0 → 1
Char. 34: 0 → 1
Char. 55: 1 → 0
Char. 71: 1 → 0
Char. 85: 1 → 0
Char. 112: 0 → 1
Char. 129: 0 → 1
Char. 131: 0 → 1

***Barasaurus besairiei*:**
Char. 33: 1 → 0
Char. 75: 1 → 0
Char. 216: 0 → 1

***Bashkyroleter bashkyricus*:**
Char. 275: 1 → 0

***Bashkyroleter mesensis*:**
Char. 169: 1 → 0

***Belebey vegrandis*:**
Char. 154: 1 → 0

***Bradysaurus* spp.:**
Char. 19: 0 → 1
Char. 73: 0 → 1
Char. 79: 0 → 1
Char. 135: 1 → 0
Char. 249: 0 → 1

**Captorhinidae:**
Char. 3: 0 → 1
Char. 23: 0 → 1
Char. 25: 1 → 0
Char. 26: 0 → 1
Char. 73: 0 → 1
Char. 75: 0 → 1
Char. 83: 0 → 1
Char. 108: 1 → 0
Char. 183: 1 → 0
Char. 201: 1 → 0
Char. 203: 1 → 2
Char. 216: 1 → 0

**Caseidae:**
Char. 24: 0 → 1
Char. 25: 1 → 0
Char. 36: 0 → 1
Char. 38: 1 → 0
Char. 46: 1 → 0
Char. 56: 0 → 1
Char. 85: 1 → 0
Char. 96: 1 → 0
Char. 170: 0 → 1
Char. 194: 0 → 1
Char. 273: 0 → 1
Char. 274: 0 → 1
Char. 278: 3 → 2

***Claudiosaurus germaini*:**
Char. 24: 0 → 1
Char. 34: 0 → 1
Char. 36: 0 → 1
Char. 43: 1 → 0
Char. 56: 0 → 1
Char. 105: 0 → 1
Char. 106: 0 → 1
Char. 117: 01 → 2
Char. 127: 1 → 0
Char. 141: 1 → 0
Char. 144: 1 → 0
Char. 187: 1 → 0
Char. 199: 0 → 1
Char. 204: 0 → 1
Char. 222: 1 → 0
Char. 234: 1 → 0

***Colobomycter pholeter*:**
Char. 21: 0 → 1
Char. 25: 1 → 0
Char. 84: 1 → 0
Char. 154: 1 → 0
Char. 167: 0 → 1
Char. 267: 0 → 1

***Delorhynchus cifelli*:**
Char. 18: 0 → 1
Char. 20: 0 → 1
Char. 21: 0 → 1
Char. 24: 0 → 1
Char. 26: 0 → 1
Char. 28: 0 → 1
Char. 33: 0 → 2
Char. 39: 0 → 1
Char. 52: 0 → 1
Char. 100: 0 → 1
Char. 111: 0 → 1
Char. 116: 1 → 0
Char. 117: 0 → 1
Char. 131: 0 → 1
Char. 156: 1 → 0
Char. 159: 0 → 1
Char. 167: 0 → 1
Char. 180: 0 → 1
Char. 189: 0 → 1
Char. 191: 0 → 1
Char. 192: 0 → 1
Char. 204: 0 → 2
Char. 267: 0 → 1

**Diadectomorpha:**
Char. 0: 0 → 1
Char. 64: 0 → 1
Char. 70: 0 → 1
Char. 75: 0 → 1
Char. 122: 0 → 1
Char. 123: 1 → 0
Char. 146: 1 → 0
Char. 275: 1 → 0
Char. 278: 3 → 0

***Emeroleter levis*:**
Char. 51: 1 → 0

**Eosauropterygia:**
Char. 159: 1 → 0
Char. 166: 1 → 0
Char. 174: 0 → 1
Char. 194: 0 → 2
Char. 272: 1 → 0

***Eudibamus cursoris*:**
Char. 154: 1 → 2

***Feeserpeton oklahomensis*:**
Char. 51: 0 → 1
Char. 70: 0 → 1
Char. 157: 0 → 1
Char. 158: 0 → 1

***Hovasaurus boulei*:**
Char. 41: 0 → 1
Char. 55: 1 → 0
Char. 60: 1 → 0
Char. 72: 2 → 1
Char. 77: 0 → 2
Char. 78: 1 → 0
Char. 79: 0 → 1
Char. 93: 1 → 0
Char. 113: 0 → 1
Char. 138: 0 → 1
Char. 141: 1 → 0
Char. 146: 1 → 0
Char. 154: 0 → 1
Char. 204: 0 → 2
Char. 206: 0 → 2
Char. 278: 0 → 3

**Kuehneosauridae:**
Char. 7: 0 → 1
Char. 24: 0 → 1
Char. 26: 0 → 1
Char. 34: 0 → 1
Char. 36: 0 → 1
Char. 43: 1 → 0
Char. 56: 0 → 1
Char. 79: 0 → 2
Char. 82: 0 → 1
Char. 98: 1 → 0
Char. 108: 1 → 0
Char. 113: 0 → 1
Char. 128: 0 → 1
Char. 148: 1 → 0
Char. 159: 1 → 0
Char. 181: 0 → 1
Char. 185: 0 → 1
Char. 206: 0 → 2
Char. 245: 0 → 1
Char. 272: 1 → 2
Char. 278: 0 → 3

***Lanthanosuchus watsoni*:**
Char. 25: 1 → 0
Char. 51: 0 → 1
Char. 76: 0 → 1
Char. 86: 0 → 1
Char. 95: 0 → 1
Char. 98: 1 → 0
Char. 110: 0 → 1
Char. 113: 0 → 1
Char. 114: 0 → 1
Char. 131: 0 → 1
Char. 137: 0 → 1
Char. 138: 0 → 1
Char. 140: 0 → 2
Char. 144: 1 → 0
Char. 154: 1 → 2
Char. 192: 0 → 1

***Macroleter poezicus*:**
Char. 9: 0 → 1
Char. 26: 0 → 1
Char. 52: 0 → 1
Char. 84: 1 → 0
Char. 87: 0 → 1
Char. 134: 0 → 2

Char. 139: 0 → 1
Char. 140: 0 → 1
Char. 146: 1 → 0
Char. 169: 1 → 0
Char. 235: 0 → 1

***Mesosaurus* spp.:**
Char. 0: 0 → 1
Char. 2: 0 → 1
Char. 5: 1 → 0
Char. 6: 0 → 1
Char. 8: 0 → 1
Char. 9: 0 → 1
Char. 13: 0 → 1
Char. 19: 0 → 1
Char. 23: 0 → 1
Char. 26: 0 → 1
Char. 29: 1 → 0
Char. 33: 0 → 1
Char. 38: 1 → 0
Char. 41: 0 → 1
Char. 50: 1 → 0
Char. 67: 0 → 1
Char. 76: 0 → 1
Char. 83: 0 → 1
Char. 84: 1 → 0
Char. 85: 1 → 0
Char. 94: 0 → 1
Char. 107: 0 → 1
Char. 109: 0 → 1
Char. 111: 0 → 1
Char. 115: 0 → 1
Char. 146: 1 → 0
Char. 148: 0 → 1
Char. 149: 1 → 0
Char. 164: 0 → 1
Char. 167: 0 → 1
Char. 176: 0 → 1
Char. 183: 1 → 0
Char. 184: 0 → 1
Char. 199: 0 → 1
Char. 202: 0 → 1
Char. 204: 0 → 2
Char. 206: 0 → 1
Char. 207: 0 → 1
Char. 209: 0 → 1
Char. 217: 0 → 1
Char. 219: 0 → 1
Char. 220: 0 → 1
Char. 231: 0 → 1
Char. 260: 2 → 0
Char. 272: 2 → 0
Char. 278: 3 → 0

***Microleter mckinzieorum*:**
Char. 0: 0 → 1
Char. 25: 1 → 0
Char. 36: 0 → 1
Char. 39: 0 → 1
Char. 51: 0 → 2
Char. 56: 0 → 1
Char. 70: 0 → 1
Char. 94: 0 → 1
Char. 106: 0 → 1
Char. 159: 0 → 1
Char. 276: 0 → 1

**Millerettidae:**
Char. 5: 1 → 0
Char. 24: 0 → 1
Char. 25: 1 → 0
Char. 44: 1 → 0
Char. 56: 0 → 1
Char. 57: 1 → 0
Char. 66: 0 → 2
Char. 78: 1 → 0
Char. 80: 1 → 0
Char. 84: 1 → 2
Char. 88: 1 → 0
Char. 96: 1 → 0
Char. 117: 0 → 1
Char. 124: 0 → 1
Char. 127: 0 → 1
Char. 135: 0 → 1
Char. 145: 0 → 1
Char. 202: 0 → 1
Char. 211: 0 → 1
Char. 230: 0 → 1
Char. 234: 0 → 1
Char. 248: 0 → 1
Char. 252: 0 → 1
Char. 253: 0 → 1

***Nycteroleter ineptus*:**
Char. 278: 0 → 3

***Nyctiphruretus acudens*:**
Char. 21: 0 → 1
Char. 33: 1 → 2
Char. 41: 0 → 1
Char. 81: 1 → 0
Char. 84: 1 → 2
Char. 94: 0 → 1
Char. 167: 0 → 1
Char. 215: 1 → 0
Char. 224: 0 → 1
Char. 266: 0 → 1
Char. 272: 2 → 1
Char. 276: 0 → 1

***Orovenator mayorum*:**
Char. 8: 0 → 1
Char. 24: 0 → 1
Char. 36: 0 → 1
Char. 160: 0 → 1
Char. 165: 0 → 1

***Owenetta* spp.:**
Char. 169: 1 → 0

***Paleothyris acadiana*:**
Char. 38: 1 → 0
Char. 50: 1 → 0
Char. 66: 1 → 2
Char. 102: 0 → 1
Char. 146: 1 → 0
Char. 180: 0 → 1
Char. 237: 0 → 1
Char. 239: 0 → 1

***Placodus* spp.:**
Char. 0: 1 → 2
Char. 9: 0 → 1
Char. 12: 0 → 1
Char. 13: 0 → 1
Char. 19: 0 → 1
Char. 26: 0 → 1
Char. 31: 0 → 1
Char. 46: 1 → 0
Char. 57: 0 → 1
Char. 78: 1 → 0
Char. 93: 1 → 0
Char. 102: 1 → 2
Char. 109: 1 → 0
Char. 140: 1 → 0
Char. 155: 0 → 1
Char. 163: 1 → 0
Char. 164: 0 → 1

***Procolophon* spp.:**
Char. 41: 0 → 1
Char. 69: 0 → 1
Char. 79: 0 → 1
Char. 83: 0 → 1
Char. 88: 0 → 1
Char. 117: 1 → 0
Char. 149: 1 → 0
Char. 180: 0 → 1
Char. 204: 0 → 1
Char. 237: 0 → 1
Char. 238: 0 → 1
Char. 272: 2 → 1

***Prolacerta broomi*:**
Char. 58: 1 → 0
Char. 66: 1 → 0
Char. 67: 1 → 0
Char. 80: 1 → 0
Char. 139: 1 → 0
Char. 192: 1 → 0
Char. 203: 1 → 2
Char. 206: 0 → 12

***Rhipaeosaurus* spp.:**
Char. 172: 0 → 1
Char. 277: 0 → 1

**Rhynchocephalia:**
Char. 0: 1 → 2
Char. 24: 0 → 1
Char. 77: 0 → 1
Char. 94: 1 → 0
Char. 139: 1 → 0
Char. 167: 1 → 0
Char. 205: 1 → 0

**Rhynchosauria:**
Char. 0: 1 → 0
Char. 7: 0 → 1
Char. 9: 0 → 1
Char. 26: 0 → 1
Char. 68: 0 → 1
Char. 99: 1 → 0
Char. 150: 1 → 0
Char. 160: 0 → 1
Char. 161: 0 → 1
Char. 171: 0 → 2
Char. 223: 0 → 1
Char. 224: 0 → 1
Char. 241: 0 → 1

***Scutosaurus* spp.:**
Char. 175: 0 → 1
Char. 218: 0 → 1
Char. 243: 0 → 2
Char. 244: 0 → 1
Char. 251: 0 → 1

***Sinosaurosphargis yunguiensis*:**
Char. 8: 0 → 1
Char. 30: 0 → 1
Char. 53: 0 → 1
Char. 82: 0 → 1
Char. 89: 1 → 0
Char. 127: 1 → 0
Char. 150: 1 → 0
Char. 154: 0 → 2
Char. 167: 1 → 0
Char. 253: 0 → 1
Char. 255: 0 → 1

**Squamata:**
Char. 26: 0 → 1
Char. 45: 0 → 1
Char. 79: 0 → 2
Char. 80: 1 → 0
Char. 82: 0 → 1
Char. 92: 1 → 0
Char. 109: 1 → 0
Char. 160: 0 → 1
Char. 245: 0 → 1

***Trilophosaurus buettneri*:**
Char. 5: 1 → 0
Char. 11: 0 → 1
Char. 55: 1 → 0
Char. 93: 1 → 0
Char. 104: 0 → 1
Char. 106: 0 → 1
Char. 113: 0 → 1
Char. 122: 0 → 1
Char. 136: 1 → 0
Char. 144: 1 → 0
Char. 154: 0 → 1
Char. 157: 0 → 1
Char. 159: 1 → 0
Char. 177: 0 → 12
Char. 194: 0 → 1
Char. 203: 1 → 2
Char. 207: 1 → 0
Char. 208: 1 → 0
Char. 272: 1 → 0

***Youngina capensis*:**
Char. 5: 1 → 0
Char. 56: 0 → 1
Char. 75: 0 → 1
Char. 170: 0 → 1
Char. 211: 0 → 1
Char. 239: 0 → 1

**Node 50:**
Char. 46: 1 → 0
Char. 88: 1 → 0
Char. 89: 1 → 0
Char. 93: 1 → 0
Char. 176: 0 → 1
Char. 195: 0 → 2
Char. 198: 0 → 1
Char. 246: 1 → 2
Char. 259: 0 → 1
Char. 265: 1 → 0
Char. 270: 0 → 1

**Node 51:**
Char. 65: 0 → 1
Char. 184: 0 → 1
Char. 205: 0 → 1
Char. 210: 0 → 1
Char. 219: 1 → 0
Char. 241: 0 → 1
Char. 246: 0 → 1
Char. 254: 0 → 1
Char. 255: 0 → 1
Char. 256: 0 → 1
Char. 268: 0 → 1
Char. 269: 0 → 1

**Node 52:**
Char. 174: 0 → 1
Char. 204: 0 → 2
Char. 247: 0 → 1
Char. 251: 0 → 1
Char. 253: 0 → 2

**Node 53:**
Char. 78: 1 → 0
Char. 155: 0 → 1
Char. 206: 0 → 2

**Node 54:**
Char. 23: 0 → 1
Char. 73: 0 → 1
Char. 94: 0 → 1
Char. 131: 0 → 1
Char. 145: 0 → 1
Char. 148: 0 → 1
Char. 151: 0 → 1
Char. 217: 0 → 1
Char. 224: 1 → 0
Char. 272: 2 → 1

**Node 55:**
Char. 33: 0 → 1
Char. 135: 0 → 1
Char. 159: 0 → 1
Char. 267: 0 → 1
Char. 278: 1 → 0

**Node 56:**
Char. 20: 0 → 1
Char. 62: 0 → 1
Char. 141: 0 → 1

**Node 57:**
Char. 0: 0 → 1
Char. 29: 1 → 0
Char. 40: 2 → 0
Char. 57: 1 → 0
Char. 59: 0 → 1
Char. 60: 0 → 1
Char. 67: 0 → 1
Char. 72: 1 → 2
Char. 89: 0 → 1
Char. 111: 0 → 1
Char. 112: 0 → 1
Char. 120: 0 → 1
Char. 154: 1 → 0
Char. 180: 0 → 1
Char. 192: 0 → 1
Char. 193: 0 → 1
Char. 222: 0 → 1
Char. 224: 0 → 1
Char. 234: 0 → 1
Char. 237: 0 → 1
Char. 266: 0 → 1
Char. 278: 3 → 1

**Node 58:**
Char. 5: 0 → 1
Char. 29: 0 → 1
Char. 40: 0 → 2
Char. 74: 0 → 1
Char. 80: 0 → 1
Char. 88: 0 → 1
Char. 116: 0 → 1
Char. 132: 0 → 1
Char. 149: 0 → 1
Char. 156: 0 → 1
Char. 169: 0 → 1
Char. 201: 0 → 1
Char. 203: 0 → 1
Char. 235: 0 → 1
Char. 276: 1 → 0

**Node 59:**
Char. 79: 1 → 0
Char. 97: 0 → 1
Char. 104: 1 → 0
Char. 144: 0 → 1
Char. 183: 0 → 1

**Node 60:**
Char. 23: 1 → 0
Char. 55: 0 → 1
Char. 83: 1 → 0
Char. 126: 1 → 0
Char. 154: 2 → 01

**Node 62:**
Char. 19: 0 → 1
Char. 92: 1 → 0

**Node 63:**
Char. 4: 0 → 1
Char. 29: 0 → 1
Char. 48: 1 → 0
Char. 213: 0 → 1
Char. 226: 0 → 2
Char. 228: 0 → 1
Char. 275: 0 → 1

**Node 64:**
Char. 58: 0 → 1
Char. 61: 0 → 1
Char. 66: 0 → 1
Char. 69: 0 → 1
Char. 150: 0 → 1
Char. 167: 0 → 1
Char. 176: 0 → 1
Char. 205: 0 → 1
Char. 230: 0 → 1
Char. 239: 0 → 1
Char. 260: 2 → 1

**Node 65:**
Char. 24: 0 → 1
Char. 57: 1 → 0
Char. 79: 0 → 1
Char. 83: 0 → 1
Char. 110: 0 → 1
Char. 132: 1 → 0

**Node 66:**
Char. 73: 0 → 1
Char. 131: 1 → 0
Char. 205: 0 → 1
Char. 276: 0 → 1

**Node 67:**
Char. 18: 0 → 1
Char. 37: 0 → 1
Char. 100: 1 → 0
Char. 107: 0 → 1
Char. 113: 1 → 0
Char. 118: 1 → 0
Char. 125: 1 → 0
Char. 150: 0 → 1

**Node 68:**
Char. 23: 0 → 1
Char. 71: 1 → 0
Char. 102: 0 → 1
Char. 103: 0 → 1
Char. 106: 0 → 1
Char. 167: 0 → 1
Char. 214: 0 → 1
Char. 216: 1 → 0
Char. 235: 0 → 2
Char. 241: 0 → 1

**Node 69:**
Char. 113: 0 → 1
Char. 132: 1 → 0
Char. 138: 0 → 1
Char. 141: 0 → 1
Char. 207: 0 → 1

**Node 70:**
Char. 20: 0 → 1
Char. 33: 0 → 1
Char. 44: 1 → 0
Char. 70: 0 → 1
Char. 80: 1 → 0
Char. 117: 0 → 1
Char. 118: 0 → 1
Char. 125: 0 → 1
Char. 131: 0 → 1
Char. 194: 0 → 1
Char. 201: 1 → 0
Char. 211: 0 → 1

**Node 71:**
Char. 38: 1 → 2
Char. 39: 0 → 1
Char. 49: 0 → 1
Char. 66: 0 → 12
Char. 76: 0 → 1
Char. 88: 1 → 0
Char. 95: 0 → 1
Char. 112: 0 → 1
Char. 148: 0 → 1
Char. 158: 0 → 1
Char. 159: 0 → 1
Char. 183: 1 → 2
Char. 192: 0 → 1
Char. 215: 0 → 1
Char. 239: 0 → 1

**Node 72:**
Char. 87: 0 → 1

**Node 73:**
Char. 25: 1 → 0
Char. 76: 1 → 0

**Node 74:**
Char. 79: 0 → 1
Char. 93: 1 → 0
Char. 133: 0 → 1

**Node 75:**
Char. 50: 1 → 0
Char. 58: 0 → 1
Char. 59: 0 → 1
Char. 60: 0 → 1
Char. 72: 1 → 2
Char. 104: 0 → 1
Char. 105: 0 → 1
Char. 106: 0 → 2
Char. 107: 0 → 1
Char. 109: 0 → 1
Char. 110: 0 → 1
Char. 146: 1 → 0
Char. 155: 0 → 2

**Node 76:**
Char. 33: 1 → 0
Char. 38: 2 → 1
Char. 39: 1 → 0
Char. 42: 0 → 1
Char. 43: 0 → 1
Char. 46: 1 → 0
Char. 48: 1 → 0
Char. 49: 1 → 0
Char. 52: 0 → 1
Char. 83: 0 → 2
Char. 84: 1 → 0
Char. 87: 0 → 1
Char. 93: 1 → 0
Char. 132: 0 → 1
Char. 161: 0 → 1
Char. 163: 1 → 0
Char. 172: 0 → 2
Char. 174: 0 → 1
Char. 188: 0 → 1
Char. 189: 0 → 1
Char. 195: 0 → 1
Char. 204: 0 → 2
Char. 212: 0 → 1
Char. 236: 0 → 1
Char. 238: 0 → 2
Char. 242: 0 → 1
Char. 274: 0 → 1
Char. 275: 1 → 0

**Node 77:**
Char. 5: 1 → 0
Char. 29: 1 → 0
Char. 44: 1 → 0
Char. 59: 0 → 1
Char. 60: 0 → 1
Char. 66: 0 → 1
Char. 67: 0 → 1
Char. 72: 1 → 2
Char. 78: 1 → 0
Char. 84: 1 → 0
Char. 88: 1 → 0
Char. 93: 1 → 0
Char. 111: 0 → 1
Char. 123: 1 → 0
Char. 129: 0 → 1
Char. 154: 1 → 0
Char. 169: 1 → 0
Char. 170: 0 → 1
Char. 197: 0 → 1

**Node 78:**
Char. 20: 0 → 1
Char. 47: 0 → 1
Char. 83: 0 → 1
Char. 85: 1 → 0
Char. 107: 0 → 1
Char. 110: 0 → 1
Char. 169: 1 → 0
Char. 170: 0 → 1

**Node 79:**
Char. 2: 0 → 1
Char. 6: 0 → 1
Char. 98: 1 → 0
Char. 101: 0 → 1
Char. 113: 0 → 1
Char. 181: 0 → 1
Char. 186: 0 → 1
Char. 193: 1 → 0
Char. 198: 0 → 1
Char. 206: 0 → 2
Char. 220: 0 → 1
Char. 234: 1 → 0
Char. 239: 1 → 0
Char. 240: 1 → 0
Char. 266: 1 → 0

**Node 80:**
Char. 33: 1 → 2
Char. 68: 0 → 1
Char. 102: 0 → 1
Char. 103: 0 → 1
Char. 223: 0 → 1
Char. 229: 0 → 1
Char. 235: 1 → 2

**Node 81:**
Char. 17: 0 → 1
Char. 42: 0 → 1
Char. 251: 0 → 1
Char. 264: 0 → 1

**Node 82:**
Char. 41: 0 → 1
Char. 48: 1 → 0
Char. 61: 1 → 2
Char. 112: 1 → 0
Char. 128: 0 → 1
Char. 138: 0 → 1
Char. 146: 1 → 0
Char. 155: 0 → 1
Char. 192: 1 → 2
Char. 224: 0 → 1
Char. 226: 0 → 1
Char. 227: 0 → 1
Char. 233: 0 → 1

**Node 83:**
Char. 61: 1 → 3
Char. 90: 0 → 1
Char. 91: 0 → 1
Char. 109: 1 → 0
Char. 155: 0 → 1
Char. 209: 1 → 0

**ANALYSIS 4**
**(NO *PAPPOCHELYS ROSINAE*)**

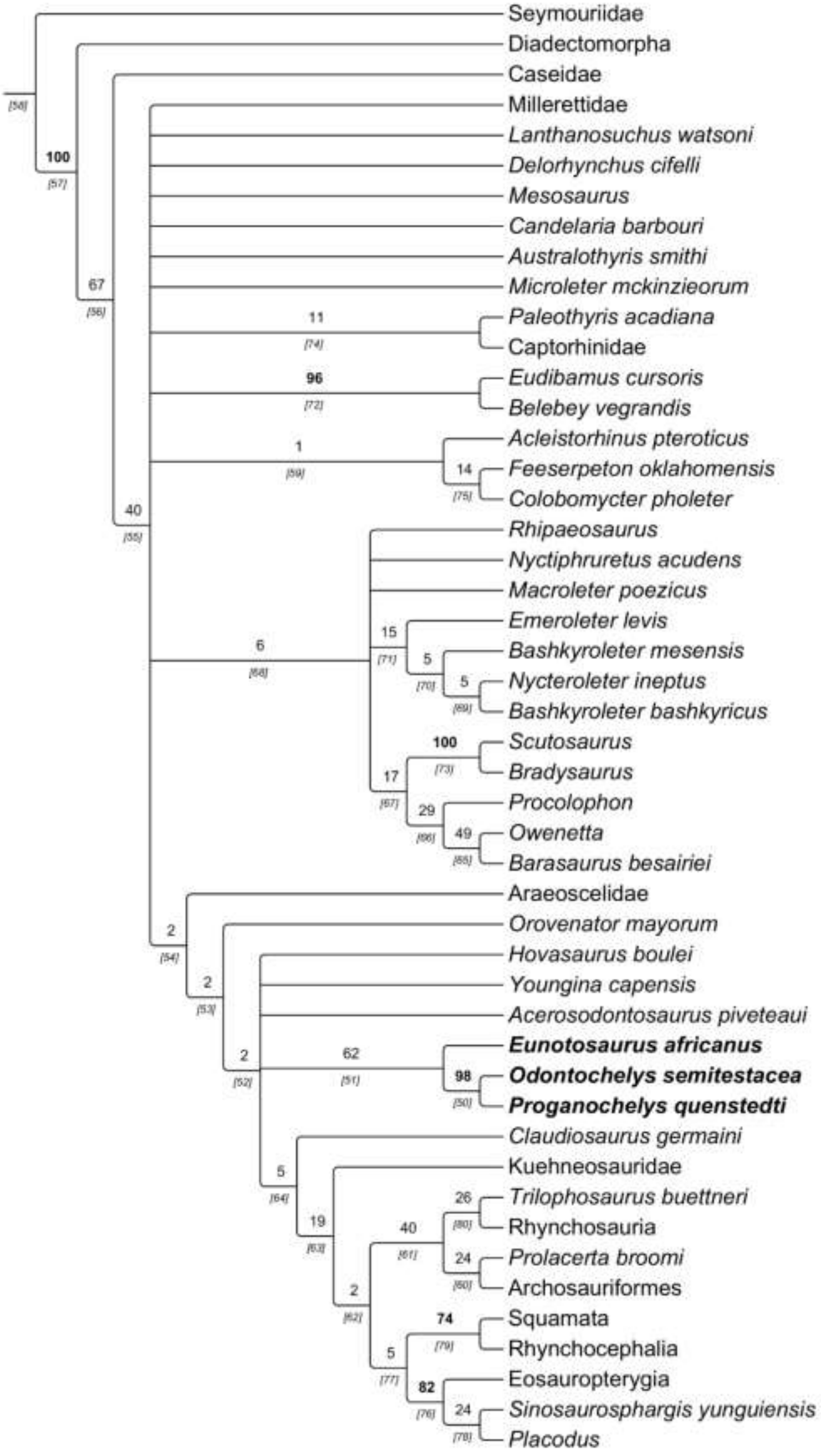

***Proganochelys quenstedti*:**
Char. 8: 0 → 1
Char. 11: 0 → 1
Char. 43: 1 → 0
Char. 106: 0 → 1
Char. 108: 1 → 0
Char. 126: 0 → 1
Char. 128: 0 → 1
Char. 175: 0 → 1
Char. 207: 1 → 0
Char. 209: 1 → 0
Char. 244: 0 → 1
Char. 262: 0 → 1

**Seymouriidae:**
Char. 54: 1 → 0
Char. 85: 1 → 0

***Odontochelys semitestacea*:**
Char. 206: 0 → 2

***Eunotosaurus africanus*:**
Char. 19: 0 → 1
Char. 55: 1 → 0
Char. 59: 1 → 0
Char. 60: 1 → 0
Char. 67: 1 → 0
Char. 72: 2 → 1
Char. 76: 0 → 1
Char. 97: 1 → 0
Char. 103: 0 → 1
Char. 153: 1 → 0
Char. 191: 1 → 0
Char. 192: 1 → 0
Char. 211: 0 → 1
Char. 222: 1 → 0
Char. 237: 1 → 0
Char. 249: 0 → 2
Char. 263: 0 → 1
Char. 273: 0 → 1
Char. 274: 0 → 1
Char. 275: 0 → 1
Char. 277: 0 → 1
Char. 278: 0 → 3

***Acerosodontosaurus piveteaui*:**
Char. 23: 0 → 1
Char. 81: 1 → 0
Char. 127: 1 → 0
Char. 128: 0 → 1
Char. 155: 0 → 1
Char. 206: 0 → 2
Char. 208: 0 → 1
Char. 278: 0 → 1

***Acleistorhinus pteroticus*:**
Char. 30: 0 → 1
Char. 33: 0 → 1
Char. 55: 1 → 0
Char. 56: 0 → 1
Char. 64: 0 → 1
Char. 95: 0 → 1
Char. 113: 0 → 1
Char. 114: 0 → 1
Char. 146: 1 → 0
Char. 278: 3 → 1

**Araeoscelidae:**
Char. 23: 0 → 1
Char. 28: 0 → 1
Char. 106: 0 → 1
Char. 116: 1 → 0
Char. 117: 0 → 2
Char. 169: 1 → 0
Char. 170: 0 → 1
Char. 197: 0 → 1
Char. 239: 0 → 1

**Archosauriformes:**
Char. 32: 0 → 1
Char. 94: 1 → 0
Char. 112: 1 → 0
Char. 152: 0 → 1
Char. 154: 0 → 2
Char. 166: 1 → 0
Char. 171: 0 → 1
Char. 185: 0 → 1
Char. 204: 0 → 1
Char. 218: 0 → 3
Char. 242: 0 → 1

***Australothyris smithi*:**
Char. 23: 0 → 1
Char. 24: 0 → 1
Char. 34: 0 → 1
Char. 55: 1 → 0
Char. 57: 1 → 0
Char. 71: 1 → 0
Char. 79: 0 → 1
Char. 83: 0 → 1
Char. 85: 1 → 0
Char. 98: 1 → 0
Char. 100: 0 → 1
Char. 103: 0 → 1
Char. 110: 0 → 1
Char. 112: 0 → 1
Char. 123: 1 → 0
Char. 129: 0 → 1
Char. 131: 0 → 1
Char. 132: 1 → 0
Char. 144: 1 → 0
Char. 147: 0 → 1
Char. 149: 1 → 0
Char. 150: 0 → 1

***Barasaurus besairiei*:**
Char. 33: 1 → 0
Char. 75: 1 → 0
Char. 216: 0 → 1

***Bashkyroleter bashkyricus*:**
Char. 275: 1 → 0

***Bashkyroleter mesensis*:**
Char. 169: 1 → 0

***Belebey vegrandis*:**
Char. 154: 1 → 0
Char. 163: 0 → 1

***Bradysaurus* spp.:**
Char. 19: 0 → 1
Char. 73: 0 → 1
Char. 79: 0 → 1
Char. 135: 1 → 0
Char. 249: 0 → 1

***Candelaria barbouri*:**
Char. 1: 0 → 1
Char. 8: 0 → 1
Char. 15: 0 → 1
Char. 20: 0 → 1
Char. 23: 0 → 1
Char. 25: 1 → 0
Char. 29: 1 → 0
Char. 33: 0 → 2
Char. 49: 0 → 1
Char. 50: 1 → 0
Char. 55: 1 → 0
Char. 57: 1 → 0
Char. 67: 0 → 1
Char. 79: 0 → 2
Char. 83: 0 → 1
Char. 89: 0 → 1
Char. 94: 0 → 1
Char. 95: 0 → 1
Char. 126: 0 → 1
Char. 127: 0 → 1
Char. 132: 1 → 0
Char. 154: 1 → 2
Char. 169: 1 → 0
Char. 276: 0 → 1
Char. 277: 0 → 1

**Captorhinidae:**
Char. 3: 0 → 1
Char. 23: 0 → 1
Char. 25: 1 → 0
Char. 26: 0 → 1
Char. 73: 0 → 1
Char. 75: 0 → 1
Char. 83: 0 → 1
Char. 108: 1 → 0
Char. 183: 1 → 0
Char. 201: 1 → 0
Char. 203: 1 → 2
Char. 216: 1 → 0

**Caseidae:**
Char. 24: 0 → 1
Char. 25: 1 → 0
Char. 36: 0 → 1
Char. 38: 1 → 0
Char. 46: 1 → 0
Char. 56: 0 → 1
Char. 85: 1 → 0
Char. 170: 0 → 1
Char. 194: 0 → 1
Char. 273: 0 → 1
Char. 274: 0 → 1
Char. 276: 0 → 1

***Claudiosaurus germaini*:**
Char. 64: 0 → 1
Char. 105: 0 → 1
Char. 127: 1 → 0
Char. 130: 0 → 1
Char. 141: 1 → 0
Char. 144: 1 → 0
Char. 166: 1 → 0
Char. 187: 1 → 0
Char. 199: 0 → 1
Char. 203: 1 → 2
Char. 204: 0 → 1
Char. 222: 1 → 0

***Colobomycter pholeter*:**
Char. 25: 1 → 0
Char. 84: 1 → 0
Char. 154: 1 → 0
Char. 167: 0 → 1
Char. 267: 0 → 1

***Delorhynchus cifelli*:**
Char. 18: 0 → 1
Char. 20: 0 → 1
Char. 21: 0 → 1
Char. 24: 0 → 1
Char. 26: 0 → 1
Char. 28: 0 → 1
Char. 33: 0 → 2
Char. 39: 0 → 1
Char. 48: 0 → 1
Char. 52: 0 → 1
Char. 100: 0 → 1
Char. 111: 0 → 1
Char. 116: 1 → 0
Char. 117: 0 → 1
Char. 131: 0 → 1
Char. 147: 0 → 1
Char. 156: 1 → 0
Char. 159: 0 → 1
Char. 163: 0 → 1
Char. 167: 0 → 1
Char. 180: 0 → 1
Char. 189: 0 → 1
Char. 191: 0 → 1
Char. 204: 0 → 2
Char. 267: 0 → 1

**Diadectomorpha:**
Char. 0: 0 → 1
Char. 64: 0 → 1
Char. 70: 0 → 1
Char. 122: 0 → 1
Char. 123: 1 → 0
Char. 146: 1 → 0
Char. 275: 1 → 0

***Emeroleter levis*:**
Char. 0: 0 → 2
Char. 51: 1 → 0

**Eosauropterygia:**
Char. 159: 1 → 0
Char. 166: 1 → 0
Char. 174: 0 → 1
Char. 194: 0 → 2
Char. 272: 1 → 0

***Eudibamus cursoris*:**
Char. 154: 1 → 2

***Feeserpeton oklahomensis*:**
Char. 51: 0 → 1
Char. 157: 0 → 1
Char. 158: 0 → 1

***Hovasaurus boulei*:**
Char. 41: 0 → 1
Char. 55: 1 → 0
Char. 60: 1 → 0
Char. 72: 2 → 1
Char. 77: 0 → 2
Char. 79: 0 → 1
Char. 93: 1 → 0
Char. 113: 0 → 1
Char. 138: 0 → 1
Char. 141: 1 → 0
Char. 146: 1 → 0
Char. 204: 0 → 2
Char. 206: 0 → 2
Char. 278: 0 → 3

**Kuehneosauridae:**
Char. 7: 0 → 1
Char. 26: 0 → 1
Char. 79: 0 → 2
Char. 82: 0 → 1
Char. 98: 1 → 0
Char. 108: 1 → 0
Char. 113: 0 → 1
Char. 128: 0 → 1
Char. 159: 1 → 0
Char. 181: 0 → 1
Char. 185: 0 → 1
Char. 206: 0 → 2
Char. 245: 0 → 1
Char. 278: 0 → 3

***Lanthanosuchus watsoni*:**
Char. 25: 1 → 0
Char. 51: 0 → 1
Char. 86: 0 → 1
Char. 95: 0 → 1
Char. 98: 1 → 0
Char. 110: 0 → 1
Char. 113: 0 → 1
Char. 114: 0 → 1
Char. 131: 0 → 1
Char. 137: 0 → 1
Char. 138: 0 → 1
Char. 140: 0 → 2
Char. 144: 1 → 0
Char. 147: 0 → 1
Char. 154: 1 → 2

***Macroleter poezicus*:**
Char. 0: 0 → 1
Char. 9: 0 → 1
Char. 26: 0 → 1
Char. 52: 0 → 1
Char. 66: 1 → 2
Char. 75: 0 → 1
Char. 84: 1 → 0
Char. 87: 0 → 1
Char. 110: 0 → 1
Char. 126: 0 → 1
Char. 134: 0 → 2
Char. 139: 0 → 1
Char. 140: 0 → 1
Char. 143: 0 → 1
Char. 146: 1 → 0
Char. 147: 1 → 2
Char. 154: 1 → 0
Char. 169: 1 → 0
Char. 235: 0 → 1
Char. 278: 3 → 0

***Mesosaurus* spp.:**
Char. 0: 0 → 1
Char. 2: 0 → 1
Char. 6: 0 → 1
Char. 8: 0 → 1
Char. 9: 0 → 1
Char. 13: 0 → 1
Char. 19: 0 → 1
Char. 23: 0 → 1
Char. 26: 0 → 1
Char. 29: 1 → 0
Char. 33: 0 → 1
Char. 38: 1 → 0
Char. 41: 0 → 1
Char. 48: 0 → 1
Char. 50: 1 → 0
Char. 67: 0 → 1
Char. 83: 0 → 1
Char. 84: 1 → 0
Char. 85: 1 → 0
Char. 94: 0 → 1
Char. 107: 0 → 1
Char. 109: 0 → 1
Char. 111: 0 → 1
Char. 115: 0 → 1
Char. 146: 1 → 0
Char. 148: 0 → 1
Char. 149: 1 → 0
Char. 164: 0 → 1
Char. 167: 0 → 1
Char. 176: 0 → 1
Char. 183: 1 → 0
Char. 184: 0 → 1
Char. 199: 0 → 1
Char. 202: 0 → 1
Char. 204: 0 → 2
Char. 206: 0 → 1
Char. 209: 0 → 1
Char. 217: 0 → 1
Char. 219: 0 → 1
Char. 220: 0 → 1
Char. 231: 0 → 1
Char. 260: 2 → 0
Char. 272: 2 → 0
Char. 278: 3 → 0

***Microleter mckinzieorum*:**
Char. 0: 0 → 1
Char. 18: 0 → 1
Char. 24: 0 → 1
Char. 25: 1 → 0
Char. 36: 0 → 1
Char. 39: 0 → 1
Char. 51: 0 → 2
Char. 56: 0 → 1
Char. 57: 1 → 0
Char. 70: 0 → 1
Char. 79: 0 → 1
Char. 83: 0 → 1
Char. 94: 0 → 1
Char. 106: 0 → 1
Char. 110: 0 → 1
Char. 132: 1 → 0
Char. 159: 0 → 1
Char. 276: 0 → 1
Char. 278: 3 → 1

**Millerettidae:**
Char. 24: 0 → 1
Char. 25: 1 → 0
Char. 44: 1 → 0
Char. 56: 0 → 1
Char. 57: 1 → 0
Char. 66: 0 → 2
Char. 78: 1 → 0
Char. 80: 1 → 0
Char. 84: 1 → 2
Char. 96: 1 → 0
Char. 117: 0 → 1
Char. 124: 0 → 1
Char. 127: 0 → 1
Char. 135: 0 → 1
Char. 145: 0 → 1
Char. 202: 0 → 1
Char. 211: 0 → 1
Char. 230: 0 → 1
Char. 234: 0 → 1
Char. 248: 0 → 1
Char. 252: 0 → 1
Char. 253: 0 → 1

***Nycteroleter ineptus*:**
Char. 278: 0 → 3

***Nyctiphruretus acudens*:**
Char. 0: 0 → 1
Char. 21: 0 → 1
Char. 33: 1 → 2
Char. 41: 0 → 1
Char. 66: 1 → 2
Char. 81: 1 → 0
Char. 83: 0 → 1
Char. 84: 1 → 2
Char. 85: 1 → 0
Char. 94: 0 → 1
Char. 166: 1 → 0
Char. 167: 0 → 1
Char. 215: 1 → 0
Char. 224: 0 → 1
Char. 226: 1 → 0
Char. 266: 0 → 1
Char. 272: 2 → 1
Char. 276: 0 → 1

***Orovenator mayorum*:**
Char. 8: 0 → 1
Char. 36: 0 → 1
Char. 160: 0 → 1
Char. 165: 0 → 1

***Owenetta* spp.:**
Char. 169: 1 → 0

***Paleothyris acadiana*:**
Char. 38: 1 → 0
Char. 50: 1 → 0
Char. 66: 1 → 2
Char. 102: 0 → 1
Char. 146: 1 → 0
Char. 180: 0 → 1
Char. 237: 0 → 1
Char. 239: 0 → 1

***Placodus* spp.:**
Char. 0: 1 → 2
Char. 9: 0 → 1
Char. 12: 0 → 1
Char. 13: 0 → 1
Char. 19: 0 → 1
Char. 26: 0 → 1
Char. 31: 0 → 1
Char. 46: 1 → 0
Char. 57: 0 → 1
Char. 78: 1 → 0
Char. 93: 1 → 0
Char. 102: 1 → 2
Char. 109: 1 → 0
Char. 140: 1 → 0
Char. 155: 0 → 1
Char. 163: 1 → 0
Char. 164: 0 → 1

***Procolophon* spp.:**
Char. 41: 0 → 1
Char. 69: 0 → 1
Char. 79: 0 → 1
Char. 83: 0 → 1
Char. 88: 0 → 1
Char. 117: 1 → 0
Char. 149: 1 → 0
Char. 180: 0 → 1
Char. 204: 0 → 1
Char. 237: 0 → 1
Char. 238: 0 → 1
Char. 272: 2 → 1
Char. 278: 3 → 0

***Prolacerta broomi*:**
Char. 58: 1 → 0
Char. 66: 1 → 0
Char. 67: 1 → 0
Char. 80: 1 → 0
Char. 139: 1 → 0
Char. 147: 1 → 0
Char. 192: 1 → 0
Char. 203: 1 → 2
Char. 206: 0 → 12

***Rhipaeosaurus* spp.:**
Char. 172: 0 → 1
Char. 186: 1 → 0
Char. 277: 0 → 1

**Rhynchocephalia:**
Char. 0: 1 → 2
Char. 24: 0 → 1
Char. 75: 1 → 0
Char. 77: 0 → 1
Char. 94: 1 → 0
Char. 117: 12 → 0
Char. 139: 1 → 0
Char. 167: 1 → 0
Char. 205: 1 → 0

**Rhynchosauria:**
Char. 0: 1 → 0
Char. 7: 0 → 1
Char. 9: 0 → 1
Char. 26: 0 → 1
Char. 68: 0 → 1
Char. 99: 1 → 0
Char. 150: 1 → 0
Char. 160: 0 → 1
Char. 161: 0 → 1
Char. 171: 0 → 2
Char. 182: 1 → 0
Char. 223: 0 → 1
Char. 241: 0 → 1

***Scutosaurus* spp.:**
Char. 175: 0 → 1
Char. 218: 0 → 1
Char. 243: 0 → 2
Char. 244: 0 → 1
Char. 251: 0 → 1

***Sinosaurosphargis yunguiensis*:**
Char. 8: 0 → 1
Char. 30: 0 → 1
Char. 53: 0 → 1
Char. 82: 0 → 1
Char. 89: 1 → 0
Char. 127: 1 → 0
Char. 150: 1 → 0
Char. 154: 0 → 2
Char. 167: 1 → 0
Char. 253: 0 → 1
Char. 255: 0 → 1

**Squamata:**
Char. 26: 0 → 1
Char. 45: 0 → 1
Char. 79: 0 → 2
Char. 80: 1 → 0
Char. 82: 0 → 1
Char. 92: 1 → 0
Char. 109: 1 → 0
Char. 160: 0 → 1
Char. 245: 0 → 1

***Trilophosaurus buettneri*:**
Char. 5: 1 → 0
Char. 11: 0 → 1
Char. 55: 1 → 0
Char. 93: 1 → 0
Char. 104: 0 → 1
Char. 113: 0 → 1
Char. 122: 0 → 1
Char. 136: 1 → 0
Char. 144: 1 → 0
Char. 154: 0 → 1
Char. 157: 0 → 1
Char. 159: 1 → 0
Char. 177: 0 → 12
Char. 194: 0 → 1
Char. 203: 1 → 2
Char. 207: 1 → 0
Char. 208: 1 → 0
Char. 272: 1 → 0

***Youngina capensis*:**
Char. 5: 1 → 0
Char. 25: 0 → 1
Char. 56: 0 → 1
Char. 75: 0 → 1
Char. 92: 1 → 0
Char. 94: 1 → 0
Char. 163: 1 → 0
Char. 170: 0 → 1
Char. 211: 0 → 1
Char. 214: 0 → 1
Char. 239: 0 → 1

**Node 50:**
Char. 46: 1 → 0
Char. 65: 0 → 1
Char. 88: 1 → 0
Char. 89: 1 → 0
Char. 93: 1 → 0
Char. 176: 0 → 1
Char. 184: 0 → 1
Char. 195: 0 → 2
Char. 198: 0 → 1
Char. 205: 0 → 1
Char. 210: 0 → 1
Char. 223: 0 → 1
Char. 232: 0 → 1
Char. 241: 0 → 1
Char. 246: 0 → 2
Char. 254: 0 → 1
Char. 255: 0 → 1
Char. 256: 0 → 1
Char. 258: 0 → 1
Char. 259: 0 → 1
Char. 268: 0 → 1
Char. 269: 0 → 1
Char. 270: 0 → 1
Char. 272: 2 → 1

**Node 51:**
Char. 0: 1 → 2
Char. 15: 0 → 1
Char. 33: 1 → 2
Char. 42: 0 → 1
Char. 48: 1 → 0
Char. 50: 0 → 1
Char. 57: 0 → 1
Char. 62: 1 → 0
Char. 64: 0 → 1
Char. 73: 0 → 1
Char. 81: 1 → 0
Char. 84: 01 → 2
Char. 129: 1 → 0
Char. 130: 0 → 1
Char. 134: 01 → 2
Char. 147: 0 → 1
Char. 148: 0 → 1
Char. 152: 0 → 1
Char. 155: 0 → 1
Char. 158: 0 → 1
Char. 161: 0 → 1
Char. 174: 0 → 1
Char. 181: 0 → 1
Char. 203: 1 → 2
Char. 204: 0 → 2
Char. 240: 1 → 0
Char. 247: 0 → 1
Char. 251: 0 → 1
Char. 253: 0 → 2

**Node 52:**
Char. 33: 0 → 1
Char. 92: 0 → 1
Char. 94: 0 → 1
Char. 135: 0 → 1
Char. 159: 0 → 1
Char. 278: 1 → 0

**Node 53:**
Char. 20: 0 → 1
Char. 25: 1 → 0
Char. 62: 0 → 1
Char. 141: 0 → 1

**Node 54:**
Char. 0: 0 → 1
Char. 27: 0 → 1
Char. 29: 1 → 0
Char. 40: 2 → 0
Char. 57: 1 → 0
Char. 59: 0 → 1
Char. 60: 0 → 1
Char. 67: 0 → 1
Char. 72: 1 → 2
Char. 89: 0 → 1
Char. 111: 0 → 1
Char. 112: 0 → 1
Char. 120: 0 → 1
Char. 163: 0 → 1
Char. 180: 0 → 1
Char. 193: 0 → 1
Char. 222: 0 → 1
Char. 234: 0 → 1
Char. 237: 0 → 1
Char. 266: 0 → 1
Char. 278: 3 → 1

**Node 55:**
Char. 29: 0 → 1
Char. 40: 0 → 2
Char. 74: 0 → 1
Char. 84: 0 → 1
Char. 116: 0 → 1
Char. 132: 0 → 1
Char. 149: 0 → 1
Char. 156: 0 → 1
Char. 169: 0 → 1
Char. 201: 0 → 1
Char. 203: 0 → 1
Char. 235: 0 → 1

**Node 56:**
Char. 72: 0 → 1
Char. 75: 1 → 0
Char. 97: 0 → 1
Char. 104: 1 → 0
Char. 144: 0 → 1

**Node 57:**
Char. 23: 1 → 0
Char. 55: 0 → 1
Char. 78: 0 → 1
Char. 126: 1 → 0

**Node 59:**
Char. 20: 0 → 1
Char. 47: 0 → 1
Char. 48: 0 → 1
Char. 79: 0 → 1
Char. 110: 0 → 1
Char. 131: 0 → 1
Char. 137: 0 → 1
Char. 140: 0 → 2
Char. 147: 0 → 1
Char. 159: 0 → 1
Char. 163: 0 → 1
Char. 169: 1 → 0
Char. 170: 0 → 1

**Node 60:**
Char. 19: 0 → 1
Char. 92: 1 → 0

**Node 61:**
Char. 4: 0 → 1
Char. 15: 0 → 1
Char. 29: 0 → 1
Char. 213: 0 → 1
Char. 226: 0 → 2
Char. 228: 0 → 1
Char. 275: 0 → 1

**Node 62:**
Char. 27: 1 → 0
Char. 75: 0 → 1
Char. 107: 0 → 1
Char. 140: 0 → 1
Char. 147: 0 → 1

**Node 63:**
Char. 58: 0 → 1
Char. 61: 0 → 1
Char. 66: 0 → 1
Char. 69: 0 → 1
Char. 150: 0 → 1
Char. 167: 0 → 1
Char. 205: 0 → 1
Char. 208: 0 → 1
Char. 239: 0 → 1

**Node 64:**
Char. 23: 0 → 1
Char. 70: 0 → 1
Char. 73: 0 → 1
Char. 117: 0 → 12
Char. 126: 0 → 1
Char. 131: 0 → 1
Char. 182: 0 → 1
Char. 190: 0 → 1
Char. 201: 0 → 1
Char. 214: 0 → 1

**Node 65:**
Char. 73: 0 → 1
Char. 131: 1 → 0
Char. 205: 0 → 1
Char. 276: 0 → 1

**Node 66:**
Char. 18: 0 → 1
Char. 37: 0 → 1
Char. 107: 0 → 1
Char. 118: 1 → 0
Char. 125: 1 → 0
Char. 126: 0 → 1
Char. 150: 0 → 1

**Node 67:**
Char. 23: 0 → 1
Char. 71: 1 → 0
Char. 75: 0 → 1
Char. 85: 1 → 0
Char. 102: 0 → 1
Char. 103: 0 → 1
Char. 106: 0 → 1
Char. 110: 0 → 1
Char. 155: 0 → 1
Char. 167: 0 → 1
Char. 214: 0 → 1
Char. 216: 1 → 0
Char. 235: 0 → 2
Char. 241: 0 → 1

**Node 68:**
Char. 20: 0 → 1
Char. 33: 0 → 1
Char. 38: 1 → 2
Char. 39: 0 → 1
Char. 44: 1 → 0
Char. 49: 0 → 1
Char. 51: 0 → 1
Char. 66: 0 → 1
Char. 70: 0 → 1
Char. 80: 1 → 0
Char. 95: 0 → 1
Char. 112: 0 → 1
Char. 117: 0 → 1
Char. 118: 0 → 1
Char. 125: 0 → 1
Char. 131: 0 → 1
Char. 135: 0 → 1
Char. 137: 0 → 1
Char. 147: 0 → 1
Char. 148: 0 → 1
Char. 157: 0 → 1
Char. 158: 0 → 1
Char. 159: 0 → 1
Char. 163: 0 → 1
Char. 183: 1 → 2
Char. 186: 0 → 1
Char. 194: 0 → 1
Char. 197: 0 → 1
Char. 201: 1 → 0
Char. 211: 0 → 1
Char. 215: 0 → 1
Char. 226: 0 → 1
Char. 235: 1 → 0
Char. 252: 0 → 1

**Node 69:**
Char. 87: 0 → 1
Char. 110: 0 → 1

**Node 70:**
Char. 25: 1 → 0
Char. 76: 1 → 0

**Node 71:**
Char. 79: 0 → 1
Char. 86: 0 → 1
Char. 93: 1 → 0
Char. 133: 0 → 1
Char. 147: 1 → 2
Char. 154: 1 → 0
Char. 278: 3 → 0

**Node 72:**
Char. 38: 1 → 2
Char. 39: 0 → 1
Char. 50: 1 → 0
Char. 58: 0 → 1
Char. 59: 0 → 1
Char. 60: 0 → 1
Char. 72: 1 → 2
Char. 83: 0 → 1
Char. 85: 1 → 0
Char. 95: 0 → 1
Char. 104: 0 → 1
Char. 105: 0 → 1
Char. 106: 0 → 2
Char. 107: 0 → 1
Char. 109: 0 → 1
Char. 110: 0 → 1
Char. 146: 1 → 0
Char. 148: 0 → 1
Char. 155: 0 → 2
Char. 183: 1 → 2

**Node 73:**
Char. 0: 0 → 2
Char. 33: 1 → 0
Char. 38: 2 → 1
Char. 39: 1 → 0
Char. 42: 0 → 1
Char. 43: 0 → 1
Char. 46: 1 → 0
Char. 49: 1 → 0
Char. 52: 0 → 1
Char. 83: 0 → 2
Char. 84: 1 → 0
Char. 87: 0 → 1
Char. 93: 1 → 0
Char. 143: 0 → 1
Char. 161: 0 → 1
Char. 163: 1 → 0
Char. 172: 0 → 2
Char. 174: 0 → 1
Char. 188: 0 → 1
Char. 189: 0 → 1
Char. 195: 0 → 1
Char. 204: 0 → 2
Char. 212: 0 → 1
Char. 236: 0 → 1
Char. 238: 0 → 2
Char. 242: 0 → 1
Char. 245: 0 → 1
Char. 274: 0 → 1
Char. 275: 1 → 0

**Node 74:**
Char. 29: 1 → 0
Char. 44: 1 → 0
Char. 59: 0 → 1
Char. 60: 0 → 1
Char. 66: 0 → 1
Char. 67: 0 → 1
Char. 72: 1 → 2
Char. 78: 1 → 0
Char. 84: 1 → 0
Char. 93: 1 → 0
Char. 111: 0 → 1
Char. 123: 1 → 0
Char. 129: 0 → 1
Char. 154: 1 → 0
Char. 169: 1 → 0
Char. 170: 0 → 1
Char. 197: 0 → 1

**Node 75:**
Char. 83: 0 → 1
Char. 85: 1 → 0
Char. 107: 0 → 1

**Node 76:**
Char. 2: 0 → 1
Char. 6: 0 → 1
Char. 98: 1 → 0
Char. 101: 0 → 1
Char. 113: 0 → 1
Char. 181: 0 → 1
Char. 186: 0 → 1
Char. 193: 1 → 0

Char. 198: 0 → 1
Char. 206: 0 → 2
Char. 220: 0 → 1
Char. 239: 1 → 0
Char. 240: 1 → 0
Char. 266: 1 → 0

**Node 77:**
Char. 33: 1 → 2
Char. 68: 0 → 1
Char. 102: 0 → 1
Char. 103: 0 → 1
Char. 223: 0 → 1
Char. 229: 0 → 1
Char. 235: 1 → 2

**Node 78:**
Char. 17: 0 → 1
Char. 42: 0 → 1
Char. 251: 0 → 1
Char. 264: 0 → 1

**Node 79:**
Char. 41: 0 → 1
Char. 61: 1 → 2
Char. 112: 1 → 0
Char. 128: 0 → 1
Char. 138: 0 → 1
Char. 146: 1 → 0
Char. 155: 0 → 1
Char. 182: 1 → 0
Char. 192: 1 → 2
Char. 226: 0 → 1
Char. 227: 0 → 1
Char. 233: 0 → 1

**Node 80:**
Char. 61: 1 → 3
Char. 90: 0 → 1
Char. 91: 0 → 1
Char. 109: 1 → 0
Char. 155: 0 → 1
Char. 209: 1 → 0

**ANALYSIS 5**
**(NO *ODONTOCHELYS SEMITESTACEA*)**

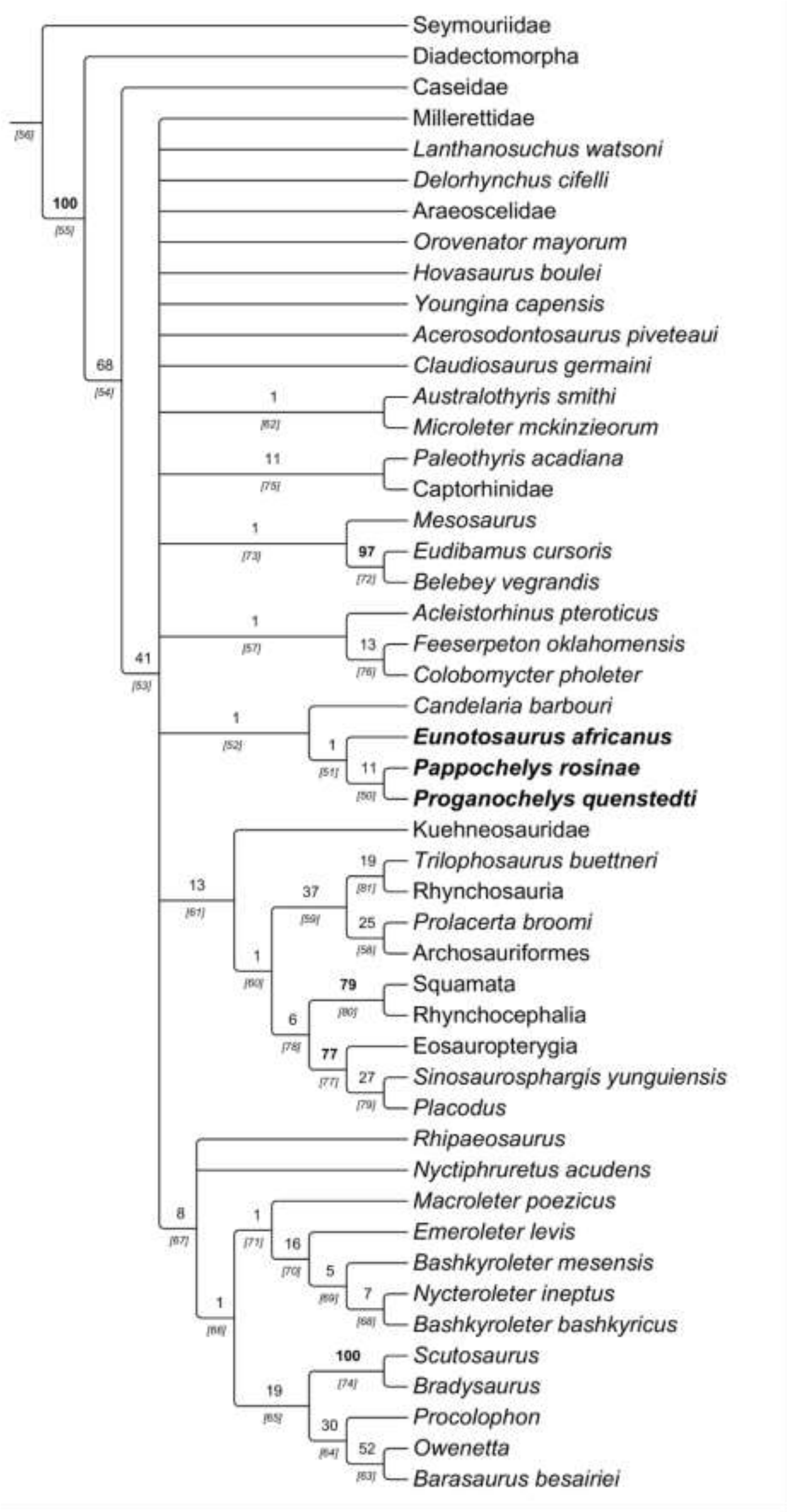

***Proganochelys quenstedti*:**
Char. 11: 0 → 1
Char. 46: 1 → 0
Char. 53: 0 → 1
Char. 55: 0 → 1
Char. 77: 0 → 1
Char. 79: 0 → 2
Char. 88: 1 → 0
Char. 89: 1 → 0
Char. 93: 1 → 0
Char. 128: 0 → 1
Char. 175: 0 → 1
Char. 176: 0 → 1
Char. 195: 0 → 2
Char. 198: 0 → 1
Char. 246: 1 → 2
Char. 259: 0 → 1
Char. 262: 0 → 1
Char. 270: 0 → 1

**Seymouriidae:**
Char. 51: 0 → 2
Char. 54: 1 → 0
Char. 99: 1 → 0
Char. 107: 0 → 1
Char. 126: 0 → 1
Char. 154: 1 → 2
Char. 225: 1 → 0
Char. 260: 2 → 0

***Pappochelys rosinae*:**
Char. 1: 0 → 1
Char. 5: 1 → 0
Char. 23: 0 → 1
Char. 41: 0 → 1
Char. 48: 0 → 1
Char. 49: 0 → 1
Char. 75: 0 → 1
Char. 129: 0 → 1
Char. 206: 0 → 2
Char. 260: 2 → 0
Char. 265: 0 → 1

***Eunotosaurus africanus*:**
Char. 19: 0 → 1
Char. 43: 0 → 1
Char. 97: 1 → 0
Char. 103: 0 → 1
Char. 112: 1 → 0
Char. 124: 0 → 1
Char. 153: 1 → 0
Char. 192: 1 → 0
Char. 211: 0 → 1
Char. 219: 0 → 1
Char. 237: 1 → 0
Char. 249: 0 → 2
Char. 263: 0 → 1
Char. 273: 0 → 1
Char. 274: 0 → 1

***Acerosodontosaurus piveteaui*:**
Char. 23: 0 → 1
Char. 59: 0 → 1
Char. 78: 1 → 0
Char. 81: 1 → 0
Char. 89: 0 → 1
Char. 94: 0 → 1
Char. 128: 0 → 1
Char. 129: 0 → 1
Char. 155: 0 → 1
Char. 206: 0 → 2
Char. 208: 0 → 1
Char. 265: 0 → 1
Char. 266: 0 → 1
Char. 278: 3 → 1

***Acleistorhinus pteroticus*:**
Char. 30: 0 → 1
Char. 55: 1 → 0
Char. 56: 0 → 1
Char. 64: 0 → 1
Char. 95: 0 → 1
Char. 113: 0 → 1
Char. 114: 0 → 1
Char. 146: 1 → 0
Char. 278: 3 → 1

**Araeoscelidae:**
Char. 5: 1 → 0
Char. 20: 1 → 0
Char. 23: 0 → 1
Char. 27: 0 → 1
Char. 28: 0 → 1
Char. 38: 1 → 0
Char. 40: 2 → 0
Char. 43: 0 → 1
Char. 59: 0 → 1
Char. 60: 0 → 1
Char. 72: 1 → 2
Char. 84: 1 → 0
Char. 89: 0 → 1
Char. 92: 1 → 0
Char. 106: 0 → 1
Char. 116: 1 → 0
Char. 117: 01 → 2
Char. 154: 1 → 0
Char. 169: 1 → 0
Char. 170: 0 → 1
Char. 193: 0 → 1
Char. 197: 0 → 1
Char. 202: 1 → 0
Char. 207: 1 → 0
Char. 209: 1 → 0
Char. 221: 1 → 0
Char. 224: 0 → 1
Char. 239: 0 → 1
Char. 266: 0 → 1
Char. 278: 3 → 1

**Archosauriformes:**
Char. 32: 0 → 1
Char. 94: 1 → 0
Char. 112: 1 → 0
Char. 152: 0 → 1
Char. 154: 0 → 2
Char. 166: 1 → 0
Char. 171: 0 → 1
Char. 185: 0 → 1
Char. 204: 0 → 1
Char. 218: 0 → 3
Char. 224: 0 → 1
Char. 242: 0 → 1

***Australothyris smithi*:**
Char. 23: 0 → 1
Char. 34: 0 → 1
Char. 55: 1 → 0
Char. 71: 1 → 0
Char. 85: 1 → 0
Char. 129: 0 → 1
Char. 131: 0 → 1

***Barasaurus besairiei*:**
Char. 33: 1 → 0
Char. 75: 1 → 0
Char. 216: 0 → 1

***Bashkyroleter bashkyricus*:**
Char. 275: 1 → 0

***Bashkyroleter mesensis*:**
Char. 169: 1 → 0

***Belebey vegrandis*:**
Char. 154: 1 → 0

***Bradysaurus* spp.:**
Char. 19: 0 → 1
Char. 73: 0 → 1
Char. 79: 0 → 1
Char. 135: 1 → 0
Char. 249: 0 → 1

***Candelaria barbouri*:**
Char. 1: 0 → 1
Char. 5: 1 → 0
Char. 23: 0 → 1
Char. 49: 0 → 1
Char. 79: 0 → 2
Char. 83: 0 → 1
Char. 88: 1 → 0
Char. 92: 1 → 0
Char. 95: 0 → 1
Char. 132: 1 → 0
Char. 154: 1 → 2

**Captorhinidae:**
Char. 3: 0 → 1
Char. 23: 0 → 1
Char. 25: 1 → 0
Char. 26: 0 → 1
Char. 73: 0 → 1
Char. 75: 0 → 1
Char. 83: 0 → 1
Char. 108: 1 → 0
Char. 180: 1 → 0
Char. 183: 1 → 0
Char. 201: 1 → 0
Char. 203: 1 → 2
Char. 216: 1 → 0
Char. 237: 1 → 0
Char. 240: 1 → 0

**Caseidae:**
Char. 24: 0 → 1
Char. 25: 1 → 0
Char. 36: 0 → 1
Char. 38: 1 → 0
Char. 46: 1 → 0
Char. 56: 0 → 1
Char. 170: 0 → 1
Char. 194: 0 → 1
Char. 273: 0 → 1
Char. 274: 0 → 1
Char. 278: 3 → 2

***Claudiosaurus germaini*:**
Char. 23: 0 → 1
Char. 24: 0 → 1
Char. 27: 0 → 1
Char. 34: 0 → 1
Char. 36: 0 → 1
Char. 56: 0 → 1
Char. 59: 0 → 1
Char. 60: 0 → 1
Char. 62: 0 → 1
Char. 64: 0 → 1
Char. 70: 0 → 1
Char. 72: 1 → 2
Char. 73: 0 → 1
Char. 84: 1 → 0
Char. 89: 0 → 1
Char. 94: 0 → 1
Char. 105: 0 → 1
Char. 106: 0 → 1
Char. 109: 0 → 1
Char. 117: 01 → 2
Char. 126: 0 → 1
Char. 129: 0 → 1
Char. 130: 0 → 1
Char. 131: 0 → 1
Char. 144: 1 → 0
Char. 148: 0 → 1
Char. 154: 1 → 0
Char. 182: 0 → 1
Char. 188: 0 → 1
Char. 190: 0 → 1
Char. 191: 0 → 1
Char. 199: 0 → 1
Char. 203: 1 → 2
Char. 204: 0 → 1
Char. 214: 0 → 1
Char. 219: 0 → 1
Char. 220: 0 → 1
Char. 234: 1 → 0
Char. 265: 0 → 1
Char. 267: 0 → 1
Char. 272: 2 → 1
Char. 275: 1 → 0
Char. 278: 3 → 0

***Colobomycter pholeter*:**
Char. 25: 1 → 0
Char. 84: 1 → 0
Char. 154: 1 → 0
Char. 167: 0 → 1
Char. 267: 0 → 1

***Delorhynchus cifelli*:**
Char. 18: 0 → 1
Char. 21: 0 → 1
Char. 24: 0 → 1
Char. 26: 0 → 1
Char. 28: 0 → 1
Char. 29: 0 → 1
Char. 33: 01 → 2
Char. 39: 0 → 1
Char. 52: 0 → 1
Char. 57: 0 → 1
Char. 67: 1 → 0
Char. 100: 0 → 1
Char. 116: 1 → 0
Char. 119: 1 → 0
Char. 131: 0 → 1
Char. 147: 0 → 1
Char. 156: 1 → 0
Char. 167: 0 → 1
Char. 189: 0 → 1
Char. 191: 0 → 1
Char. 202: 1 → 0
Char. 204: 0 → 2
Char. 267: 0 → 1

**Diadectomorpha:**
Char. 64: 0 → 1
Char. 70: 0 → 1
Char. 75: 0 → 1
Char. 122: 0 → 1
Char. 123: 1 → 0
Char. 146: 1 → 0
Char. 275: 1 → 0
Char. 278: 3 → 0

***Emeroleter levis*:**
Char. 51: 1 → 0

**Eosauropterygia:**
Char. 159: 1 → 0
Char. 166: 1 → 0
Char. 174: 0 → 1
Char. 194: 0 → 2
Char. 272: 1 → 0

***Eudibamus cursoris*:**
Char. 154: 1 → 2

***Feeserpeton oklahomensis*:**
Char. 51: 0 → 1
Char. 157: 0 → 1
Char. 158: 0 → 1

***Hovasaurus boulei*:**
Char. 41: 0 → 1
Char. 43: 0 → 1
Char. 55: 1 → 0
Char. 59: 0 → 1
Char. 77: 0 → 2
Char. 78: 1 → 0
Char. 79: 0 → 1
Char. 89: 0 → 1
Char. 93: 1 → 0
Char. 113: 0 → 1
Char. 127: 0 → 1

Char. 135: 0 → 1
Char. 136: 0 → 1
Char. 138: 0 → 1
Char. 146: 1 → 0
Char. 188: 0 → 1
Char. 191: 0 → 1
Char. 193: 0 → 1
Char. 201: 1 → 0
Char. 204: 0 → 2
Char. 206: 0 → 2
Char. 215: 0 → 1
Char. 220: 0 → 1
Char. 224: 0 → 1
Char. 265: 0 → 1
Char. 275: 1 → 0

**Kuehneosauridae:**
Char. 7: 0 → 1
Char. 24: 0 → 1
Char. 26: 0 → 1
Char. 27: 0 → 1
Char. 34: 0 → 1
Char. 36: 0 → 1
Char. 44: 1 → 0
Char. 56: 0 → 1
Char. 79: 0 → 2
Char. 82: 0 → 1
Char. 98: 1 → 0
Char. 108: 1 → 0
Char. 113: 0 → 1
Char. 128: 0 → 1
Char. 181: 0 → 1
Char. 185: 0 → 1
Char. 206: 0 → 2
Char. 245: 0 → 1

***Lanthanosuchus watsoni*:**
Char. 25: 1 → 0
Char. 29: 0 → 1
Char. 51: 0 → 1
Char. 76: 0 → 1
Char. 86: 0 → 1
Char. 95: 0 → 1
Char. 98: 1 → 0
Char. 110: 0 → 1
Char. 111: 1 → 0
Char. 113: 0 → 1
Char. 114: 0 → 1
Char. 119: 1 → 0
Char. 131: 0 → 1
Char. 137: 0 → 1
Char. 138: 0 → 1
Char. 140: 0 → 2
Char. 144: 1 → 0
Char. 147: 0 → 1
Char. 154: 1 → 2

***Macroleter poezicus*:**
Char. 52: 0 → 1
Char. 66: 1 → 2
Char. 84: 1 → 0
Char. 87: 0 → 1
Char. 140: 0 → 1
Char. 146: 1 → 0

Char. 169: 1 → 0
Char. 235: 0 → 1

***Mesosaurus* spp.:**
Char. 2: 0 → 1
Char. 5: 1 → 0
Char. 6: 0 → 1
Char. 8: 0 → 1
Char. 9: 0 → 1
Char. 13: 0 → 1
Char. 19: 0 → 1
Char. 41: 0 → 1
Char. 84: 1 → 0
Char. 94: 0 → 1
Char. 112: 1 → 0
Char. 115: 0 → 1
Char. 149: 1 → 0
Char. 164: 0 → 1
Char. 167: 0 → 1
Char. 192: 1 → 0
Char. 231: 0 → 1
Char. 234: 1 → 0
Char. 240: 1 → 0
Char. 272: 2 → 0

***Microleter mckinzieorum*:**
Char. 25: 1 → 0
Char. 36: 0 → 1
Char. 39: 0 → 1
Char. 51: 0 → 2
Char. 56: 0 → 1
Char. 70: 0 → 1
Char. 94: 0 → 1
Char. 106: 0 → 1
Char. 112: 1 → 0
Char. 276: 0 → 1

**Millerettidae:**
Char. 5: 1 → 0
Char. 20: 1 → 0
Char. 24: 0 → 1
Char. 25: 1 → 0
Char. 29: 0 → 1
Char. 44: 1 → 0
Char. 56: 0 → 1
Char. 66: 0 → 2
Char. 67: 1 → 0
Char. 78: 1 → 0
Char. 80: 1 → 0
Char. 84: 1 → 2
Char. 88: 1 → 0
Char. 96: 1 → 0
Char. 111: 1 → 0
Char. 112: 1 → 0
Char. 120: 1 → 0
Char. 121: 0 → 1
Char. 124: 0 → 1
Char. 127: 0 → 1
Char. 135: 0 → 1
Char. 145: 0 → 1
Char. 163: 1 → 0
Char. 180: 1 → 0
Char. 192: 1 → 0
Char. 211: 0 → 1

Char. 230: 0 → 1
Char. 248: 0 → 1
Char. 252: 0 → 1
Char. 253: 0 → 1

***Nycteroleter ineptus*:**
Char. 278: 0 → 3

***Nyctiphruretus acudens*:**
Char. 21: 0 → 1
Char. 33: 1 → 2
Char. 41: 0 → 1
Char. 66: 1 → 2
Char. 81: 1 → 0
Char. 83: 0 → 1
Char. 84: 1 → 2
Char. 94: 0 → 1
Char. 167: 0 → 1
Char. 224: 0 → 1
Char. 266: 0 → 1
Char. 272: 2 → 1
Char. 276: 0 → 1

***Orovenator mayorum*:**
Char. 8: 0 → 1
Char. 24: 0 → 1
Char. 25: 1 → 0
Char. 36: 0 → 1
Char. 62: 0 → 1
Char. 72: 1 → 2
Char. 89: 0 → 1
Char. 92: 1 → 0
Char. 136: 0 → 1
Char. 141: 0 → 1
Char. 160: 0 → 1
Char. 165: 0 → 1
Char. 278: 3 → 1

***Owenetta* spp.:**
Char. 169: 1 → 0

***Paleothyris acadiana*:**
Char. 38: 1 → 0
Char. 66: 1 → 2
Char. 102: 0 → 1
Char. 146: 1 → 0
Char. 239: 0 → 1

***Placodus* spp.:**
Char. 0: 1 → 2
Char. 9: 0 → 1
Char. 12: 0 → 1
Char. 13: 0 → 1
Char. 19: 0 → 1
Char. 26: 0 → 1
Char. 31: 0 → 1
Char. 44: 1 → 0
Char. 46: 1 → 0
Char. 57: 0 → 1
Char. 78: 1 → 0
Char. 93: 1 → 0
Char. 102: 1 → 2
Char. 140: 1 → 0
Char. 155: 0 → 1

Char. 163: 1 → 0
Char. 164: 0 → 1

***Procolophon* spp.:**
Char. 41: 0 → 1
Char. 69: 0 → 1
Char. 79: 0 → 1
Char. 83: 0 → 1
Char. 88: 0 → 1
Char. 117: 1 → 0
Char. 149: 1 → 0
Char. 204: 0 → 1
Char. 237: 0 → 1
Char. 238: 0 → 1
Char. 272: 2 → 1
Char. 278: 3 → 0

***Prolacerta broomi*:**
Char. 58: 1 → 0
Char. 66: 1 → 0
Char. 67: 1 → 0
Char. 80: 1 → 0
Char. 139: 1 → 0
Char. 147: 1 → 0
Char. 192: 1 → 0
Char. 203: 1 → 2
Char. 206: 0 → 12

***Rhipaeosaurus* spp.:**
Char. 172: 0 → 1
Char. 277: 0 → 1

**Rhynchocephalia:**
Char. 0: 1 → 2
Char. 24: 0 → 1
Char. 75: 1 → 0
Char. 77: 0 → 1
Char. 94: 1 → 0
Char. 139: 1 → 0
Char. 167: 1 → 0
Char. 205: 1 → 0

**Rhynchosauria:**
Char. 0: 1 → 0
Char. 7: 0 → 1
Char. 9: 0 → 1
Char. 26: 0 → 1
Char. 44: 1 → 0
Char. 68: 0 → 1
Char. 99: 1 → 0
Char. 150: 1 → 0
Char. 160: 0 → 1
Char. 161: 0 → 1
Char. 171: 0 → 2
Char. 223: 0 → 1
Char. 224: 0 → 1
Char. 241: 0 → 1

***Scutosaurus* spp.:**
Char. 175: 0 → 1
Char. 218: 0 → 1
Char. 243: 0 → 2
Char. 244: 0 → 1
Char. 251: 0 → 1

***Sinosaurosphargis yunguiensis*:**
Char. 8: 0 → 1
Char. 30: 0 → 1
Char. 53: 0 → 1
Char. 82: 0 → 1
Char. 89: 1 → 0
Char. 127: 1 → 0
Char. 150: 1 → 0
Char. 154: 0 → 2
Char. 167: 1 → 0
Char. 253: 0 → 1
Char. 255: 0 → 1

**Squamata:**
Char. 26: 0 → 1
Char. 45: 0 → 1
Char. 79: 0 → 2
Char. 80: 1 → 0
Char. 82: 0 → 1
Char. 92: 1 → 0
Char. 160: 0 → 1
Char. 245: 0 → 1

***Trilophosaurus buettneri*:**
Char. 5: 1 → 0
Char. 11: 0 → 1
Char. 55: 1 → 0
Char. 93: 1 → 0
Char. 104: 0 → 1
Char. 106: 0 → 1
Char. 113: 0 → 1
Char. 122: 0 → 1
Char. 144: 1 → 0
Char. 154: 0 → 1
Char. 157: 0 → 1
Char. 159: 1 → 0
Char. 177: 0 → 12
Char. 194: 0 → 1
Char. 203: 1 → 2
Char. 207: 1 → 0
Char. 208: 1 → 0
Char. 272: 1 → 0

***Youngina capensis*:**
Char. 5: 1 → 0
Char. 21: 0 → 1
Char. 27: 0 → 1
Char. 38: 1 → 0
Char. 40: 2 → 0
Char. 43: 0 → 1
Char. 44: 1 → 0
Char. 56: 0 → 1
Char. 59: 0 → 1
Char. 60: 0 → 1
Char. 62: 0 → 1
Char. 72: 1 → 2
Char. 75: 0 → 1
Char. 84: 1 → 0
Char. 89: 0 → 1
Char. 92: 1 → 0
Char. 109: 0 → 1
Char. 127: 0 → 1
Char. 129: 0 → 1

Char. 134: 0 → 1
Char. 135: 0 → 1
Char. 141: 0 → 1
Char. 154: 1 → 0
Char. 163: 1 → 0
Char. 170: 0 → 1
Char. 191: 0 → 1
Char. 193: 0 → 1
Char. 201: 1 → 0
Char. 211: 0 → 1
Char. 214: 0 → 1
Char. 215: 0 → 1
Char. 219: 0 → 1
Char. 224: 0 → 1
Char. 231: 0 → 1
Char. 239: 0 → 1
Char. 265: 0 → 1
Char. 267: 0 → 1
Char. 275: 1 → 0
Char. 278: 3 → 0

**Node 50:**
Char. 65: 0 → 1
Char. 72: 1 → 2
Char. 131: 0 → 1
Char. 184: 0 → 1
Char. 205: 0 → 1
Char. 210: 0 → 1
Char. 241: 0 → 1
Char. 246: 0 → 1
Char. 254: 0 → 1
Char. 255: 0 → 1
Char. 256: 0 → 1
Char. 267: 0 → 1
Char. 268: 0 → 1
Char. 269: 0 → 1
Char. 278: 3 → 0

**Node 51:**
Char. 21: 0 → 1
Char. 84: 1 → 2

**Node 52:**
Char. 15: 0 → 1
Char. 25: 1 → 0
Char. 33: 01 → 2
Char. 55: 1 → 0
Char. 89: 0 → 1
Char. 94: 0 → 1
Char. 127: 0 → 1

**Node 53:**
Char. 5: 0 → 1
Char. 20: 0 → 1
Char. 40: 0 → 2
Char. 57: 1 → 0
Char. 67: 0 → 1
Char. 74: 0 → 1
Char. 80: 0 → 1
Char. 84: 0 → 1
Char. 88: 0 → 1
Char. 112: 0 → 1
Char. 116: 0 → 1
Char. 119: 0 → 1
Char. 149: 0 → 1
Char. 156: 0 → 1
Char. 163: 0 → 1
Char. 169: 0 → 1
Char. 180: 0 → 1
Char. 192: 0 → 1
Char. 201: 0 → 1
Char. 202: 0 → 1
Char. 203: 0 → 1
Char. 209: 0 → 1
Char. 221: 0 → 1
Char. 234: 0 → 1
Char. 235: 0 → 1
Char. 237: 0 → 1

**Node 54:**
Char. 72: 0 → 1
Char. 79: 1 → 0
Char. 81: 0 → 1
Char. 93: 0 → 1
Char. 97: 0 → 1
Char. 183: 0 → 1

**Node 57:**
Char. 29: 0 → 1
Char. 47: 0 → 1
Char. 57: 0 → 1
Char. 67: 1 → 0
Char. 79: 0 → 1
Char. 110: 0 → 1
Char. 111: 1 → 0
Char. 112: 1 → 0
Char. 131: 0 → 1
Char. 137: 0 → 1
Char. 140: 0 → 2
Char. 147: 0 → 1
Char. 169: 1 → 0
Char. 170: 0 → 1

**Node 58:**
Char. 19: 0 → 1
Char. 92: 1 → 0

**Node 59:**
Char. 4: 0 → 1
Char. 15: 0 → 1
Char. 29: 0 → 1
Char. 213: 0 → 1
Char. 226: 0 → 2
Char. 228: 0 → 1

**Node 60:**
Char. 43: 0 → 1
Char. 75: 0 → 1
Char. 107: 0 → 1
Char. 140: 0 → 1
Char. 147: 0 → 1
Char. 148: 0 → 1
Char. 272: 2 → 1
Char. 278: 3 → 0

**Node 61:**
Char. 58: 0 → 1
Char. 59: 0 → 1
Char. 60: 0 → 1
Char. 61: 0 → 1
Char. 62: 0 → 1
Char. 66: 0 → 1
Char. 69: 0 → 1
Char. 72: 1 → 2
Char. 73: 0 → 1
Char. 89: 0 → 1
Char. 94: 0 → 1
Char. 126: 0 → 1
Char. 127: 0 → 1
Char. 129: 0 → 1
Char. 131: 0 → 1
Char. 141: 0 → 1
Char. 145: 0 → 1
Char. 150: 0 → 1
Char. 154: 1 → 0
Char. 167: 0 → 1
Char. 176: 0 → 1
Char. 188: 0 → 1
Char. 205: 0 → 1
Char. 208: 0 → 1
Char. 214: 0 → 1
Char. 219: 0 → 1
Char. 239: 0 → 1
Char. 265: 0 → 1

**Node 62:**
Char. 24: 0 → 1
Char. 29: 0 → 1
Char. 67: 1 → 0
Char. 79: 0 → 1
Char. 83: 0 → 1
Char. 110: 0 → 1
Char. 111: 1 → 0
Char. 132: 1 → 0

**Node 63:**
Char. 73: 0 → 1
Char. 131: 1 → 0
Char. 205: 0 → 1
Char. 239: 0 → 1
Char. 276: 0 → 1

**Node 64:**
Char. 18: 0 → 1
Char. 37: 0 → 1
Char. 107: 0 → 1
Char. 118: 1 → 0
Char. 125: 1 → 0
Char. 150: 0 → 1

**Node 65:**
Char. 23: 0 → 1
Char. 71: 1 → 0
Char. 102: 0 → 1
Char. 103: 0 → 1
Char. 106: 0 → 1
Char. 167: 0 → 1
Char. 214: 0 → 1
Char. 216: 1 → 0
Char. 235: 0 → 2
Char. 241: 0 → 1

**Node 66:**
Char. 138: 0 → 1
Char. 141: 0 → 1

**Node 67:**
Char. 29: 0 → 1
Char. 38: 1 → 2
Char. 39: 0 → 1
Char. 44: 1 → 0
Char. 49: 0 → 1
Char. 51: 0 → 1
Char. 57: 0 → 1
Char. 66: 0 → 1
Char. 67: 1 → 0
Char. 70: 0 → 1
Char. 76: 0 → 1
Char. 80: 1 → 0
Char. 88: 1 → 0
Char. 95: 0 → 1
Char. 111: 1 → 0
Char. 118: 0 → 1
Char. 120: 1 → 0
Char. 125: 0 → 1
Char. 131: 0 → 1
Char. 137: 0 → 1
Char. 147: 0 → 1
Char. 148: 0 → 1
Char. 158: 0 → 1
Char. 183: 1 → 2
Char. 194: 0 → 1
Char. 201: 1 → 0
Char. 211: 0 → 1
Char. 234: 1 → 0
Char. 235: 1 → 0
Char. 237: 1 → 0
Char. 240: 1 → 0
Char. 252: 0 → 1

**Node 68:**
Char. 87: 0 → 1

**Node 69:**
Char. 25: 1 → 0
Char. 76: 1 → 0

**Node 70:**
Char. 79: 0 → 1
Char. 93: 1 → 0
Char. 133: 0 → 1

**Node 71:**
Char. 147: 1 → 2
Char. 154: 1 → 0
Char. 239: 0 → 1
Char. 278: 3 → 0

**Node 72:**
Char. 39: 0 → 1
Char. 58: 0 → 1
Char. 59: 0 → 1
Char. 60: 0 → 1
Char. 72: 1 → 2
Char. 88: 1 → 0
Char. 95: 0 → 1
Char. 104: 0 → 1
Char. 105: 0 → 1
Char. 106: 0 → 2
Char. 110: 0 → 1
Char. 155: 0 → 2

**Node 73:**
Char. 20: 1 → 0
Char. 23: 0 → 1
Char. 26: 0 → 1
Char. 76: 0 → 1
Char. 83: 0 → 1
Char. 85: 1 → 0
Char. 107: 0 → 1
Char. 109: 0 → 1
Char. 146: 1 → 0
Char. 148: 0 → 1
Char. 199: 0 → 1
Char. 237: 1 → 0

**Node 74:**
Char. 33: 1 → 0
Char. 38: 2 → 1
Char. 39: 1 → 0
Char. 42: 0 → 1
Char. 43: 0 → 1
Char. 46: 1 → 0
Char. 49: 1 → 0
Char. 52: 0 → 1
Char. 83: 0 → 2
Char. 84: 1 → 0
Char. 87: 0 → 1
Char. 93: 1 → 0
Char. 161: 0 → 1
Char. 163: 1 → 0
Char. 172: 0 → 2
Char. 174: 0 → 1
Char. 188: 0 → 1
Char. 189: 0 → 1
Char. 195: 0 → 1
Char. 204: 0 → 2
Char. 212: 0 → 1
Char. 221: 1 → 0
Char. 236: 0 → 1
Char. 238: 0 → 2
Char. 242: 0 → 1
Char. 245: 0 → 1
Char. 274: 0 → 1
Char. 275: 1 → 0

**Node 75:**
Char. 5: 1 → 0
Char. 20: 1 → 0
Char. 44: 1 → 0
Char. 59: 0 → 1
Char. 60: 0 → 1
Char. 66: 0 → 1
Char. 72: 1 → 2
Char. 78: 1 → 0
Char. 84: 1 → 0
Char. 88: 1 → 0
Char. 93: 1 → 0
Char. 112: 1 → 0
Char. 123: 1 → 0
Char. 129: 0 → 1

Char. 154: 1 → 0
Char. 163: 1 → 0
Char. 169: 1 → 0
Char. 170: 0 → 1
Char. 192: 1 → 0
Char. 197: 0 → 1
Char. 202: 1 → 0
Char. 207: 1 → 0
Char. 209: 1 → 0
Char. 221: 1 → 0
Char. 234: 1 → 0

**Node 76:**
Char. 83: 0 → 1
Char. 85: 1 → 0
Char. 107: 0 → 1

**Node 77:**
Char. 2: 0 → 1
Char. 6: 0 → 1
Char. 98: 1 → 0
Char. 101: 0 → 1
Char. 113: 0 → 1
Char. 181: 0 → 1
Char. 186: 0 → 1
Char. 198: 0 → 1
Char. 206: 0 → 2
Char. 220: 0 → 1
Char. 234: 1 → 0
Char. 239: 1 → 0
Char. 240: 1 → 0

**Node 78:**
Char. 33: 1 → 2
Char. 68: 0 → 1
Char. 102: 0 → 1
Char. 103: 0 → 1
Char. 223: 0 → 1
Char. 229: 0 → 1
Char. 235: 1 → 2

**Node 79:**
Char. 17: 0 → 1
Char. 42: 0 → 1
Char. 251: 0 → 1
Char. 264: 0 → 1

**Node 80:**
Char. 41: 0 → 1
Char. 61: 1 → 2
Char. 112: 1 → 0
Char. 128: 0 → 1
Char. 138: 0 → 1
Char. 146: 1 → 0
Char. 155: 0 → 1
Char. 192: 1 → 2
Char. 224: 0 → 1
Char. 226: 0 → 1
Char. 227: 0 → 1
Char. 233: 0 → 1

**Node 81:**
Char. 61: 1 → 3
Char. 90: 0 → 1
Char. 91: 0 → 1
Char. 155: 0 → 1
Char. 209: 1 → 0

**ANALYSIS 6**
**(NO *EUNOTOSAURUS AFRICANUS*)**

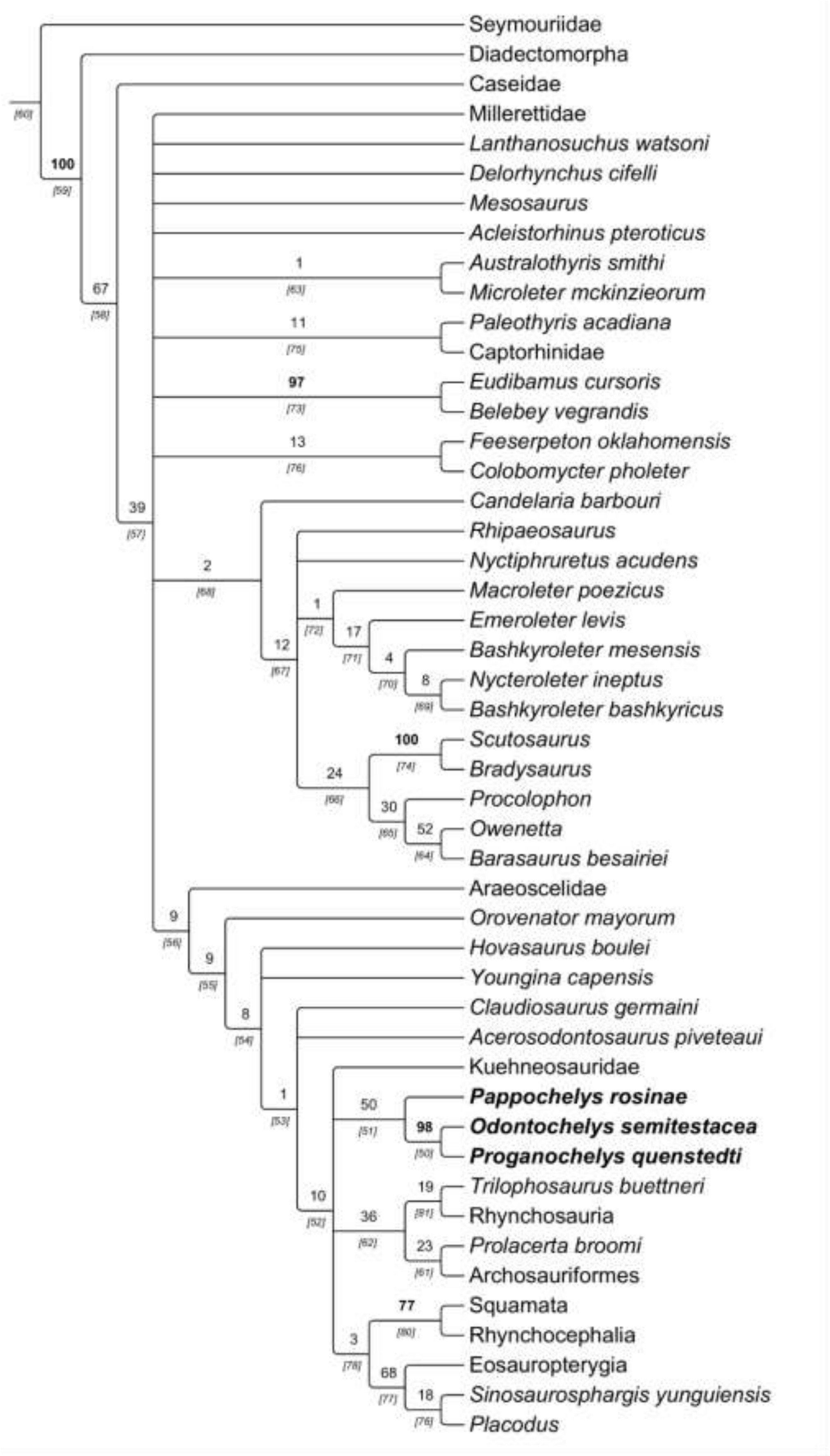

***Proganochelys quenstedti*:**
Char. 8: 0 → 1
Char. 11: 0 → 1
Char. 108: 1 → 0
Char. 128: 0 → 1
Char. 175: 0 → 1
Char. 202: 1 → 0
Char. 207: 1 → 0
Char. 209: 1 → 0
Char. 244: 0 → 1
Char. 248: 0 → 1
Char. 250: 0 → 1
Char. 262: 0 → 1

**Seymouriidae:**
Char. 23: 0 → 1
Char. 51: 0 → 2
Char. 54: 1 → 0
Char. 71: 1 → 0
Char. 83: 0 → 1
Char. 85: 1 → 0
Char. 99: 1 → 0
Char. 107: 0 → 1
Char. 126: 0 → 1
Char. 140: 0 → 2
Char. 154: 1 → 2
Char. 225: 1 → 0
Char. 260: 2 → 0

***Pappochelys rosinae*:**
Char. 1: 0 → 1
Char. 5: 1 → 0
Char. 12: 0 → 1
Char. 41: 0 → 1
Char. 49: 0 → 1
Char. 55: 1 → 0
Char. 67: 1 → 0
Char. 169: 1 → 0
Char. 176: 1 → 0

***Odontochelys semitestacea*:**
Char. 109: 1 → 0
Char. 112: 1 → 0
Char. 124: 0 → 1
Char. 126: 1 → 0

***Acerosodontosaurus piveteaui*:**
Char. 78: 1 → 0
Char. 128: 0 → 1
Char. 155: 0 → 1
Char. 267: 1 → 0
Char. 278: 0 → 1

***Acleistorhinus pteroticus*:**
Char. 20: 0 → 1
Char. 21: 0 → 1
Char. 30: 0 → 1
Char. 33: 0 → 1
Char. 47: 0 → 1
Char. 48: 0 → 1
Char. 55: 1 → 0
Char. 56: 0 → 1
Char. 64: 0 → 1
Char. 70: 0 → 1
Char. 79: 0 → 1
Char. 95: 0 → 1
Char. 110: 0 → 1
Char. 113: 0 → 1
Char. 114: 0 → 1
Char. 131: 0 → 1
Char. 137: 0 → 1
Char. 140: 0 → 2
Char. 146: 1 → 0
Char. 159: 0 → 1
Char. 169: 1 → 0
Char. 170: 0 → 1
Char. 278: 3 → 1

**Araeoscelidae:**
Char. 5: 1 → 0
Char. 23: 0 → 1
Char. 28: 0 → 1
Char. 106: 0 → 1
Char. 116: 1 → 0
Char. 166: 1 → 0
Char. 169: 1 → 0
Char. 170: 0 → 1
Char. 197: 0 → 1
Char. 239: 0 → 1

**Archosauriformes:**
Char. 32: 0 → 1
Char. 94: 1 → 0
Char. 112: 1 → 0
Char. 123: 1 → 0
Char. 152: 0 → 1
Char. 154: 0 → 2
Char. 166: 1 → 0
Char. 171: 0 → 1
Char. 185: 0 → 1
Char. 204: 0 → 1
Char. 218: 0 → 3
Char. 242: 0 → 1

***Australothyris smithi*:**
Char. 23: 0 → 1
Char. 34: 0 → 1
Char. 55: 1 → 0
Char. 71: 1 → 0
Char. 85: 1 → 0
Char. 112: 0 → 1
Char. 129: 0 → 1
Char. 131: 0 → 1

***Barasaurus besairiei*:**
Char. 33: 1 → 0
Char. 75: 1 → 0
Char. 216: 0 → 1

***Bashkyroleter bashkyricus*:**
Char. 275: 1 → 0

***Bashkyroleter mesensis*:**
Char. 169: 1 → 0

***Belebey vegrandis*:**
Char. 154: 1 → 0

***Bradysaurus* spp.:**
Char. 19: 0 → 1
Char. 73: 0 → 1
Char. 79: 0 → 1
Char. 135: 1 → 0
Char. 249: 0 → 1

***Candelaria barbouri*:**
Char. 1: 0 → 1
Char. 5: 1 → 0
Char. 8: 0 → 1
Char. 15: 0 → 1
Char. 23: 0 → 1
Char. 25: 1 → 0
Char. 29: 1 → 0
Char. 50: 1 → 0
Char. 55: 1 → 0
Char. 57: 1 → 0
Char. 67: 0 → 1
Char. 79: 0 → 2
Char. 89: 0 → 1
Char. 127: 0 → 1
Char. 154: 1 → 2
Char. 169: 1 → 0
Char. 277: 0 → 1

**Captorhinidae:**
Char. 3: 0 → 1
Char. 23: 0 → 1
Char. 25: 1 → 0
Char. 26: 0 → 1
Char. 73: 0 → 1
Char. 75: 0 → 1
Char. 83: 0 → 1
Char. 108: 1 → 0
Char. 183: 1 → 0
Char. 201: 1 → 0
Char. 203: 1 → 2
Char. 216: 1 → 0

**Caseidae:**
Char. 24: 0 → 1
Char. 25: 1 → 0
Char. 36: 0 → 1
Char. 38: 1 → 0
Char. 46: 1 → 0
Char. 50: 1 → 0
Char. 56: 0 → 1
Char. 85: 1 → 0
Char. 96: 1 → 0
Char. 170: 0 → 1
Char. 194: 0 → 1
Char. 274: 0 → 1
Char. 278: 3 → 2

***Claudiosaurus germaini*:**
Char. 24: 0 → 1
Char. 34: 0 → 1
Char. 36: 0 → 1
Char. 56: 0 → 1
Char. 64: 0 → 1
Char. 105: 0 → 1
Char. 130: 0 → 1
Char. 144: 1 → 0
Char. 166: 1 → 0
Char. 187: 1 → 0
Char. 199: 0 → 1
Char. 203: 1 → 2
Char. 204: 0 → 1
Char. 220: 0 → 1
Char. 222: 1 → 0
Char. 234: 1 → 0

***Colobomycter pholeter*:**
Char. 21: 0 → 1
Char. 25: 1 → 0
Char. 84: 1 → 0
Char. 154: 1 → 0
Char. 167: 0 → 1
Char. 267: 0 → 1

***Delorhynchus cifelli*:**
Char. 18: 0 → 1
Char. 20: 0 → 1
Char. 21: 0 → 1
Char. 24: 0 → 1
Char. 26: 0 → 1
Char. 28: 0 → 1
Char. 33: 0 → 2
Char. 39: 0 → 1
Char. 48: 0 → 1
Char. 52: 0 → 1
Char. 100: 0 → 1
Char. 111: 0 → 1
Char. 116: 1 → 0
Char. 117: 0 → 1
Char. 131: 0 → 1
Char. 156: 1 → 0
Char. 159: 0 → 1
Char. 167: 0 → 1
Char. 180: 0 → 1
Char. 189: 0 → 1
Char. 191: 0 → 1
Char. 204: 0 → 2
Char. 267: 0 → 1

**Diadectomorpha:**
Char. 0: 0 → 1
Char. 70: 0 → 1
Char. 75: 0 → 1
Char. 122: 0 → 1
Char. 123: 1 → 0
Char. 146: 1 → 0
Char. 275: 1 → 0
Char. 278: 3 → 0

***Emeroleter levis*:**
Char. 0: 0 → 2
Char. 51: 1 → 0

**Eosauropterygia:**
Char. 159: 1 → 0
Char. 166: 1 → 0
Char. 168: 0 → 1
Char. 174: 0 → 1
Char. 194: 0 → 2
Char. 272: 1 → 0

***Eudibamus cursoris*:**
Char. 154: 1 → 2

***Feeserpeton oklahomensis*:**
Char. 51: 0 → 1
Char. 70: 0 → 1
Char. 157: 0 → 1
Char. 158: 0 → 1

***Hovasaurus boulei*:**
Char. 41: 0 → 1
Char. 55: 1 → 0
Char. 60: 1 → 0
Char. 72: 2 → 1
Char. 77: 0 → 2
Char. 78: 1 → 0
Char. 79: 0 → 1
Char. 93: 1 → 0
Char. 113: 0 → 1
Char. 138: 0 → 1
Char. 146: 1 → 0
Char. 154: 0 → 1
Char. 204: 0 → 2
Char. 220: 0 → 1
Char. 278: 0 → 3

**Kuehneosauridae:**
Char. 7: 0 → 1
Char. 24: 0 → 1
Char. 34: 0 → 1
Char. 35: 0 → 1
Char. 36: 0 → 1
Char. 56: 0 → 1
Char. 79: 0 → 2
Char. 82: 0 → 1
Char. 98: 1 → 0
Char. 108: 1 → 0
Char. 113: 0 → 1
Char. 128: 0 → 1
Char. 147: 1 → 0
Char. 148: 1 → 0
Char. 159: 1 → 0
Char. 165: 0 → 1
Char. 168: 0 → 1
Char. 185: 0 → 1
Char. 245: 0 → 1
Char. 272: 1 → 2
Char. 278: 0 → 3

***Lanthanosuchus watsoni*:**
Char. 25: 1 → 0
Char. 51: 0 → 1
Char. 76: 0 → 1
Char. 86: 0 → 1
Char. 95: 0 → 1
Char. 98: 1 → 0
Char. 110: 0 → 1
Char. 113: 0 → 1
Char. 114: 0 → 1
Char. 131: 0 → 1
Char. 137: 0 → 1
Char. 138: 0 → 1
Char. 140: 0 → 2

Char. 144: 1 → 0
Char. 154: 1 → 2

***Macroleter poezicus*:**
Char. 0: 0 → 1
Char. 52: 0 → 1
Char. 66: 1 → 2
Char. 75: 0 → 1
Char. 84: 1 → 0
Char. 87: 0 → 1
Char. 110: 0 → 1
Char. 121: 0 → 1
Char. 140: 0 → 1
Char. 146: 1 → 0
Char. 169: 1 → 0
Char. 235: 0 → 1

***Mesosaurus* spp.:**
Char. 0: 0 → 1
Char. 2: 0 → 1
Char. 5: 1 → 0
Char. 6: 0 → 1
Char. 8: 0 → 1
Char. 9: 0 → 1
Char. 13: 0 → 1
Char. 19: 0 → 1
Char. 23: 0 → 1
Char. 26: 0 → 1
Char. 29: 1 → 0
Char. 33: 0 → 1
Char. 38: 1 → 0
Char. 41: 0 → 1
Char. 48: 0 → 1
Char. 50: 1 → 0
Char. 67: 0 → 1
Char. 76: 0 → 1
Char. 83: 0 → 1
Char. 84: 1 → 0
Char. 85: 1 → 0
Char. 94: 0 → 1
Char. 107: 0 → 1
Char. 109: 0 → 1
Char. 111: 0 → 1
Char. 115: 0 → 1
Char. 146: 1 → 0
Char. 148: 0 → 1
Char. 149: 1 → 0
Char. 164: 0 → 1
Char. 166: 1 → 0
Char. 167: 0 → 1
Char. 176: 0 → 1
Char. 183: 1 → 0
Char. 184: 0 → 1
Char. 199: 0 → 1
Char. 202: 0 → 1
Char. 204: 0 → 2
Char. 206: 0 → 1
Char. 207: 0 → 1
Char. 209: 0 → 1
Char. 217: 0 → 1
Char. 219: 0 → 1
Char. 220: 0 → 1
Char. 231: 0 → 1
Char. 260: 2 → 0

Char. 272: 2 → 0
Char. 278: 3 → 0

***Microleter mckinzieorum*:**
Char. 0: 0 → 1
Char. 25: 1 → 0
Char. 36: 0 → 1
Char. 39: 0 → 1
Char. 51: 0 → 2
Char. 56: 0 → 1
Char. 70: 0 → 1
Char. 94: 0 → 1
Char. 106: 0 → 1
Char. 159: 0 → 1
Char. 166: 1 → 0
Char. 276: 0 → 1

**Millerettidae:**
Char. 5: 1 → 0
Char. 24: 0 → 1
Char. 25: 1 → 0
Char. 44: 1 → 0
Char. 56: 0 → 1
Char. 57: 1 → 0
Char. 66: 0 → 2
Char. 78: 1 → 0
Char. 80: 1 → 0
Char. 84: 1 → 2
Char. 96: 1 → 0
Char. 117: 0 → 1
Char. 121: 0 → 1
Char. 124: 0 → 1
Char. 127: 0 → 1
Char. 135: 0 → 1
Char. 145: 0 → 1
Char. 166: 1 → 0
Char. 202: 0 → 1
Char. 211: 0 → 1
Char. 230: 0 → 1
Char. 234: 0 → 1
Char. 248: 0 → 1
Char. 252: 0 → 1
Char. 253: 0 → 1

***Nycteroleter ineptus*:**
Char. 278: 0 → 3

***Nyctiphruretus acudens*:**
Char. 0: 0 → 1
Char. 21: 0 → 1
Char. 41: 0 → 1
Char. 66: 1 → 2
Char. 81: 1 → 0
Char. 84: 1 → 2
Char. 166: 1 → 0
Char. 167: 0 → 1
Char. 215: 1 → 0
Char. 224: 0 → 1
Char. 226: 1 → 0
Char. 266: 0 → 1
Char. 272: 2 → 1

***Orovenator mayorum*:**
Char. 8: 0 → 1
Char. 24: 0 → 1

Char. 36: 0 → 1
Char. 160: 0 → 1
Char. 165: 0 → 1

***Owenetta* spp.:**
Char. 169: 1 → 0

***Paleothyris acadiana*:**
Char. 38: 1 → 0
Char. 50: 1 → 0
Char. 66: 1 → 2
Char. 102: 0 → 1
Char. 146: 1 → 0
Char. 180: 0 → 1
Char. 237: 0 → 1
Char. 239: 0 → 1

***Placodus* spp.:**
Char. 0: 1 → 2
Char. 9: 0 → 1
Char. 12: 0 → 1
Char. 13: 0 → 1
Char. 19: 0 → 1
Char. 31: 0 → 1
Char. 46: 1 → 0
Char. 57: 0 → 1
Char. 78: 1 → 0
Char. 93: 1 → 0
Char. 102: 1 → 2
Char. 109: 1 → 0
Char. 140: 1 → 0
Char. 155: 0 → 1
Char. 163: 1 → 0
Char. 164: 0 → 1

***Procolophon* spp.:**
Char. 41: 0 → 1
Char. 69: 0 → 1
Char. 79: 0 → 1
Char. 88: 0 → 1
Char. 117: 1 → 0
Char. 149: 1 → 0
Char. 180: 0 → 1
Char. 204: 0 → 1
Char. 237: 0 → 1
Char. 238: 0 → 1
Char. 272: 2 → 1
Char. 278: 3 → 0

***Prolacerta broomi*:**
Char. 58: 1 → 0
Char. 66: 1 → 0
Char. 67: 1 → 0
Char. 80: 1 → 0
Char. 139: 1 → 0
Char. 147: 1 → 0
Char. 192: 1 → 0
Char. 203: 1 → 2

***Rhipaeosaurus* spp.:**
Char. 172: 0 → 1
Char. 186: 1 → 0
Char. 277: 0 → 1

**Rhynchocephalia:**
Char. 0: 1 → 2
Char. 24: 0 → 1
Char. 77: 0 → 1
Char. 94: 1 → 0
Char. 117: 12 → 0
Char. 139: 1 → 0
Char. 167: 1 → 0
Char. 205: 1 → 0

**Rhynchosauria:**
Char. 0: 1 → 0
Char. 7: 0 → 1
Char. 9: 0 → 1
Char. 63: 1 → 0
Char. 68: 0 → 1
Char. 99: 1 → 0
Char. 150: 1 → 0
Char. 160: 0 → 1
Char. 161: 0 → 1
Char. 171: 0 → 2
Char. 241: 0 → 1

***Scutosaurus* spp.:**
Char. 175: 0 → 1
Char. 218: 0 → 1
Char. 243: 0 → 2
Char. 244: 0 → 1
Char. 251: 0 → 1

***Sinosaurosphargis yunguiensis*:**
Char. 8: 0 → 1
Char. 30: 0 → 1
Char. 53: 0 → 1
Char. 82: 0 → 1
Char. 89: 1 → 0
Char. 127: 1 → 0
Char. 150: 1 → 0
Char. 154: 0 → 2
Char. 167: 1 → 0
Char. 253: 0 → 1
Char. 255: 0 → 1

**Squamata:**
Char. 45: 0 → 1
Char. 79: 0 → 2
Char. 80: 1 → 0
Char. 82: 0 → 1
Char. 92: 1 → 0
Char. 109: 1 → 0
Char. 160: 0 → 1
Char. 245: 0 → 1

***Trilophosaurus buettneri*:**
Char. 5: 1 → 0
Char. 11: 0 → 1
Char. 55: 1 → 0
Char. 93: 1 → 0
Char. 104: 0 → 1
Char. 113: 0 → 1
Char. 122: 0 → 1
Char. 123: 1 → 0
Char. 136: 1 → 0
Char. 144: 1 → 0

Char. 154: 0 → 1
Char. 157: 0 → 1
Char. 159: 1 → 0
Char. 177: 0 → 12
Char. 194: 0 → 1
Char. 203: 1 → 2
Char. 207: 1 → 0
Char. 208: 1 → 0
Char. 272: 1 → 0

***Youngina capensis*:**
Char. 5: 1 → 0
Char. 56: 0 → 1
Char. 75: 0 → 1
Char. 170: 0 → 1
Char. 211: 0 → 1
Char. 231: 0 → 1
Char. 239: 0 → 1

**Node 50:**
Char. 46: 1 → 0
Char. 50: 0 → 1
Char. 88: 1 → 0
Char. 89: 1 → 0
Char. 93: 1 → 0
Char. 195: 0 → 2
Char. 198: 0 → 1
Char. 240: 1 → 0
Char. 246: 1 → 2
Char. 259: 0 → 1
Char. 265: 1 → 0
Char. 270: 0 → 1

**Node 51:**
Char. 61: 1 → 0
Char. 62: 1 → 0
Char. 65: 0 → 1
Char. 78: 1 → 0
Char. 193: 1 → 0
Char. 194: 0 → 2
Char. 214: 1 → 0
Char. 219: 1 → 0
Char. 220: 0 → 1
Char. 241: 0 → 1
Char. 246: 0 → 1
Char. 247: 0 → 1
Char. 254: 0 → 1
Char. 255: 0 → 1
Char. 256: 0 → 1
Char. 268: 0 → 1
Char. 269: 0 → 1

**Node 52:**
Char. 58: 0 → 1
Char. 61: 0 → 1
Char. 66: 0 → 1
Char. 69: 0 → 1
Char. 84: 0 → 1
Char. 147: 0 → 1
Char. 150: 0 → 1
Char. 167: 0 → 1
Char. 176: 0 → 1
Char. 184: 0 → 1
Char. 205: 0 → 1

Char. 210: 0 → 1
Char. 230: 0 → 1
Char. 231: 0 → 1
Char. 232: 0 → 1

**Node 53:**
Char. 23: 0 → 1
Char. 70: 0 → 1
Char. 73: 0 → 1
Char. 94: 0 → 1
Char. 131: 0 → 1
Char. 148: 0 → 1
Char. 272: 2 → 1

**Node 54:**
Char. 33: 0 → 1
Char. 135: 0 → 1
Char. 159: 0 → 1
Char. 267: 0 → 1
Char. 278: 1 → 0

**Node 55:**
Char. 20: 0 → 1
Char. 62: 0 → 1

**Node 56:**
Char. 0: 0 → 1
Char. 27: 0 → 1
Char. 29: 1 → 0
Char. 38: 1 → 0
Char. 40: 2 → 0
Char. 57: 1 → 0
Char. 59: 0 → 1
Char. 60: 0 → 1
Char. 67: 0 → 1
Char. 72: 1 → 2
Char. 84: 1 → 0
Char. 89: 0 → 1
Char. 111: 0 → 1
Char. 112: 0 → 1
Char. 120: 0 → 1
Char. 154: 1 → 0
Char. 180: 0 → 1
Char. 193: 0 → 1
Char. 222: 0 → 1
Char. 224: 0 → 1
Char. 234: 0 → 1
Char. 237: 0 → 1
Char. 266: 0 → 1
Char. 278: 3 → 1

**Node 57:**
Char. 5: 0 → 1
Char. 29: 0 → 1
Char. 40: 0 → 2
Char. 74: 0 → 1
Char. 80: 0 → 1
Char. 116: 0 → 1
Char. 149: 0 → 1
Char. 166: 0 → 1
Char. 169: 0 → 1
Char. 201: 0 → 1
Char. 203: 0 → 1
Char. 235: 0 → 1
Char. 276: 1 → 0

**Node 58:**
Char. 81: 0 → 1
Char. 97: 0 → 1
Char. 144: 0 → 1

**Node 59:**
Char. 55: 0 → 1
Char. 111: 1 → 0
Char. 135: 1 → 0

**Node 61:**
Char. 19: 0 → 1
Char. 92: 1 → 0

**Node 62:**
Char. 4: 0 → 1
Char. 29: 0 → 1
Char. 48: 1 → 0
Char. 107: 0 → 1
Char. 140: 0 → 1
Char. 210: 1 → 0
Char. 213: 0 → 1
Char. 226: 0 → 2
Char. 228: 0 → 1
Char. 275: 0 → 1

**Node 63:**
Char. 24: 0 → 1
Char. 57: 1 → 0
Char. 79: 0 → 1
Char. 83: 0 → 1
Char. 110: 0 → 1
Char. 132: 1 → 0

**Node 64:**
Char. 73: 0 → 1
Char. 131: 1 → 0
Char. 205: 0 → 1
Char. 239: 0 → 1

**Node 65:**
Char. 18: 0 → 1
Char. 37: 0 → 1
Char. 48: 0 → 1
Char. 107: 0 → 1
Char. 118: 1 → 0
Char. 125: 1 → 0
Char. 150: 0 → 1

**Node 66:**
Char. 23: 0 → 1
Char. 71: 1 → 0
Char. 75: 0 → 1
Char. 102: 0 → 1
Char. 103: 0 → 1
Char. 106: 0 → 1
Char. 110: 0 → 1
Char. 155: 0 → 1
Char. 167: 0 → 1
Char. 207: 0 → 1
Char. 214: 0 → 1
Char. 216: 1 → 0
Char. 235: 0 → 2
Char. 241: 0 → 1

**Node 67:**
Char. 66: 0 → 1
Char. 159: 0 → 1

**Node 68:**
Char. 20: 0 → 1
Char. 33: 0 → 12
Char. 49: 0 → 1
Char. 76: 0 → 1
Char. 95: 0 → 1

**Node 69:**
Char. 87: 0 → 1
Char. 110: 0 → 1

**Node 70:**
Char. 25: 1 → 0
Char. 76: 1 → 0

**Node 71:**
Char. 79: 0 → 1
Char. 86: 0 → 1
Char. 93: 1 → 0
Char. 133: 0 → 1

**Node 72:**
Char. 48: 0 → 1
Char. 100: 0 → 1
Char. 113: 0 → 1
Char. 147: 1 → 2
Char. 154: 1 → 0
Char. 207: 0 → 1
Char. 239: 0 → 1
Char. 278: 3 → 0

**Node 73:**
Char. 38: 1 → 2
Char. 39: 0 → 1
Char. 50: 1 → 0
Char. 58: 0 → 1
Char. 59: 0 → 1
Char. 60: 0 → 1
Char. 72: 1 → 2
Char. 83: 0 → 1
Char. 85: 1 → 0
Char. 95: 0 → 1
Char. 104: 0 → 1
Char. 105: 0 → 1
Char. 106: 0 → 2
Char. 107: 0 → 1
Char. 109: 0 → 1
Char. 110: 0 → 1
Char. 146: 1 → 0
Char. 148: 0 → 1
Char. 155: 0 → 2
Char. 183: 1 → 2

**Node 74:**
Char. 0: 0 → 2
Char. 33: 1 → 0
Char. 38: 2 → 1
Char. 39: 1 → 0
Char. 42: 0 → 1
Char. 43: 0 → 1
Char. 46: 1 → 0
Char. 49: 1 → 0
Char. 52: 0 → 1
Char. 83: 01 → 2
Char. 84: 1 → 0
Char. 87: 0 → 1
Char. 93: 1 → 0
Char. 100: 0 → 1
Char. 113: 0 → 1
Char. 143: 0 → 1
Char. 161: 0 → 1
Char. 163: 1 → 0
Char. 172: 0 → 2
Char. 174: 0 → 1
Char. 188: 0 → 1
Char. 189: 0 → 1
Char. 195: 0 → 1
Char. 204: 0 → 2
Char. 212: 0 → 1
Char. 236: 0 → 1
Char. 238: 0 → 2
Char. 242: 0 → 1
Char. 245: 0 → 1
Char. 274: 0 → 1
Char. 275: 1 → 0

**Node 75:**
Char. 5: 1 → 0
Char. 29: 1 → 0
Char. 44: 1 → 0
Char. 59: 0 → 1
Char. 60: 0 → 1
Char. 66: 0 → 1
Char. 67: 0 → 1
Char. 72: 1 → 2
Char. 78: 1 → 0
Char. 84: 1 → 0
Char. 93: 1 → 0
Char. 111: 0 → 1
Char. 123: 1 → 0
Char. 129: 0 → 1
Char. 154: 1 → 0
Char. 169: 1 → 0
Char. 170: 0 → 1
Char. 197: 0 → 1

**Node 76:**
Char. 20: 0 → 1
Char. 47: 0 → 1
Char. 83: 0 → 1
Char. 85: 1 → 0
Char. 107: 0 → 1
Char. 110: 0 → 1
Char. 169: 1 → 0
Char. 170: 0 → 1

**Node 77:**
Char. 2: 0 → 1
Char. 6: 0 → 1
Char. 98: 1 → 0
Char. 101: 0 → 1
Char. 113: 0 → 1
Char. 186: 0 → 1
Char. 193: 1 → 0
Char. 198: 0 → 1
Char. 220: 0 → 1
Char. 231: 1 → 0
Char. 232: 1 → 0
Char. 234: 1 → 0
Char. 240: 1 → 0
Char. 266: 1 → 0

**Node 78:**
Char. 33: 1 → 2
Char. 35: 0 → 1
Char. 68: 0 → 1
Char. 102: 0 → 1
Char. 103: 0 → 1
Char. 107: 0 → 1
Char. 123: 1 → 0
Char. 140: 0 → 1
Char. 229: 0 → 1
Char. 235: 1 → 2

**Node 79:**
Char. 17: 0 → 1
Char. 42: 0 → 1
Char. 251: 0 → 1
Char. 264: 0 → 1

**Node 80:**
Char. 41: 0 → 1
Char. 48: 1 → 0
Char. 61: 1 → 2
Char. 112: 1 → 0
Char. 128: 0 → 1
Char. 138: 0 → 1
Char. 146: 1 → 0
Char. 155: 0 → 1
Char. 165: 0 → 1
Char. 192: 1 → 2
Char. 226: 0 → 1
Char. 227: 0 → 1
Char. 233: 0 → 1

**Node 81:**
Char. 61: 1 → 3
Char. 90: 0 → 1
Char. 91: 0 → 1
Char. 109: 1 → 0
Char. 155: 0 → 1
Char. 209: 1 → 0

**ANALYSIS 7**
**(NO *PROGANOCHELYS QUENSTEDTI* AND *CANDELARIA BARBOURI*)**

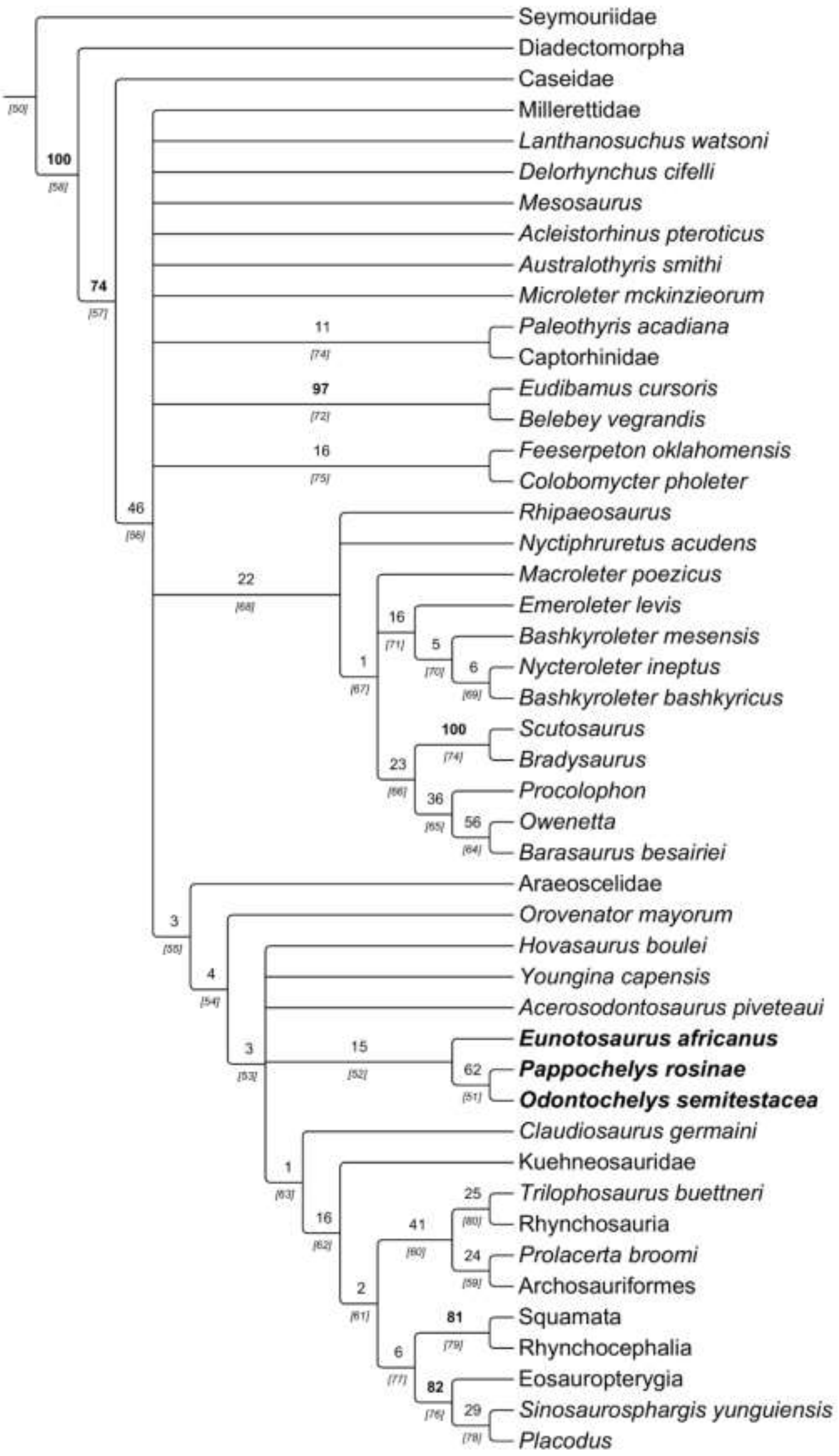

**Seymouriidae:**
Char. 51: 0 → 2
Char. 54: 1 → 0
Char. 85: 1 → 0
Char. 99: 1 → 0
Char. 107: 0 → 1
Char. 140: 0 → 2
Char. 207: 0 → 1
Char. 225: 1 → 0
Char. 260: 2 → 0

***Pappochelys rosinae*:**
Char. 0: 2 → 0
Char. 1: 0 → 1
Char. 5: 1 → 0
Char. 12: 0 → 1
Char. 41: 0 → 1
Char. 43: 1 → 0
Char. 49: 0 → 1
Char. 75: 0 → 1
Char. 126: 0 → 1
Char. 169: 1 → 0
Char. 260: 2 → 0

***Odontochelys semitestacea*:**
Char. 46: 1 → 0
Char. 88: 1 → 0
Char. 89: 1 → 0
Char. 93: 1 → 0
Char. 176: 0 → 1
Char. 195: 0 → 2
Char. 198: 0 → 1
Char. 246: 1 → 2
Char. 259: 0 → 1
Char. 265: 1 → 0
Char. 270: 0 → 1

***Eunotosaurus africanus*:**
Char. 19: 0 → 1
Char. 24: 0 → 1
Char. 42: 0 → 1
Char. 48: 1 → 0
Char. 57: 0 → 1
Char. 72: 2 → 1
Char. 76: 0 → 1
Char. 81: 1 → 0
Char. 129: 1 → 0
Char. 191: 1 → 0
Char. 192: 1 → 0
Char. 202: 1 → 0
Char. 211: 0 → 1
Char. 222: 1 → 0
Char. 237: 1 → 0
Char. 248: 0 → 1
Char. 250: 0 → 1
Char. 263: 0 → 1
Char. 273: 0 → 1
Char. 274: 0 → 1
Char. 276: 0 → 1
Char. 278: 0 → 3

***Acerosodontosaurus piveteaui*:**
Char. 81: 1 → 0
Char. 127: 1 → 0
Char. 128: 0 → 1
Char. 155: 0 → 1
Char. 208: 0 → 1
Char. 278: 0 → 1

***Acleistorhinus pteroticus*:**
Char. 20: 0 → 1
Char. 21: 0 → 1
Char. 30: 0 → 1
Char. 33: 0 → 1
Char. 47: 0 → 1
Char. 55: 1 → 0
Char. 56: 0 → 1
Char. 64: 0 → 1
Char. 70: 0 → 1
Char. 79: 0 → 1
Char. 95: 0 → 1
Char. 110: 0 → 1
Char. 113: 0 → 1
Char. 114: 0 → 1
Char. 121: 1 → 0
Char. 131: 0 → 1
Char. 137: 0 → 1
Char. 140: 0 → 2
Char. 146: 1 → 0
Char. 169: 1 → 0
Char. 170: 0 → 1
Char. 278: 3 → 1

**Araeoscelidae:**
Char. 5: 1 → 0
Char. 28: 0 → 1
Char. 106: 0 → 1
Char. 116: 1 → 0
Char. 117: 0 → 2
Char. 169: 1 → 0
Char. 170: 0 → 1
Char. 197: 0 → 1
Char. 239: 0 → 1

**Archosauriformes:**
Char. 32: 0 → 1
Char. 94: 1 → 0
Char. 112: 1 → 0
Char. 152: 0 → 1
Char. 154: 0 → 2
Char. 166: 1 → 0
Char. 171: 0 → 1
Char. 185: 0 → 1
Char. 204: 0 → 1
Char. 218: 0 → 3
Char. 242: 0 → 1

***Australothyris smithi*:**
Char. 23: 0 → 1
Char. 24: 0 → 1
Char. 34: 0 → 1
Char. 55: 1 → 0
Char. 57: 1 → 0
Char. 71: 1 → 0
Char. 79: 0 → 1
Char. 83: 0 → 1
Char. 85: 1 → 0
Char. 98: 1 → 0
Char. 100: 0 → 1
Char. 103: 0 → 1
Char. 110: 0 → 1
Char. 112: 0 → 1
Char. 123: 1 → 0
Char. 129: 0 → 1
Char. 131: 0 → 1
Char. 132: 1 → 0
Char. 144: 1 → 0
Char. 149: 1 → 0
Char. 150: 0 → 1

***Barasaurus besairiei*:**
Char. 33: 1 → 0
Char. 75: 1 → 0
Char. 216: 0 → 1

***Bashkyroleter bashkyricus*:**
Char. 275: 1 → 0

***Bashkyroleter mesensis*:**
Char. 169: 1 → 0

***Belebey vegrandis*:**
Char. 154: 1 → 0

***Bradysaurus* spp.:**
Char. 19: 0 → 1
Char. 73: 0 → 1
Char. 79: 0 → 1
Char. 135: 1 → 0
Char. 249: 0 → 1

**Captorhinidae:**
Char. 3: 0 → 1
Char. 23: 0 → 1
Char. 25: 1 → 0
Char. 26: 0 → 1
Char. 73: 0 → 1
Char. 75: 0 → 1
Char. 83: 0 → 1
Char. 108: 1 → 0
Char. 183: 1 → 0
Char. 201: 1 → 0
Char. 203: 1 → 2
Char. 216: 1 → 0

**Caseidae:**
Char. 24: 0 → 1
Char. 25: 1 → 0
Char. 36: 0 → 1
Char. 38: 1 → 0
Char. 46: 1 → 0
Char. 50: 1 → 0
Char. 56: 0 → 1
Char. 85: 1 → 0
Char. 96: 1 → 0
Char. 98: 1 → 0
Char. 170: 0 → 1
Char. 194: 0 → 1
Char. 273: 0 → 1
Char. 274: 0 → 1
Char. 278: 3 → 2

***Claudiosaurus germaini*:**
Char. 64: 0 → 1
Char. 105: 0 → 1
Char. 127: 1 → 0
Char. 130: 0 → 1
Char. 141: 1 → 0
Char. 144: 1 → 0
Char. 166: 1 → 0
Char. 187: 1 → 0
Char. 199: 0 → 1
Char. 203: 1 → 2
Char. 204: 0 → 1
Char. 222: 1 → 0

***Colobomycter pholeter*:**
Char. 21: 0 → 1
Char. 25: 1 → 0
Char. 84: 1 → 0
Char. 154: 1 → 0
Char. 167: 0 → 1
Char. 267: 0 → 1

***Delorhynchus cifelli*:**
Char. 18: 0 → 1
Char. 20: 0 → 1
Char. 21: 0 → 1
Char. 24: 0 → 1
Char. 26: 0 → 1
Char. 28: 0 → 1
Char. 33: 0 → 2
Char. 39: 0 → 1
Char. 52: 0 → 1
Char. 100: 0 → 1
Char. 111: 0 → 1
Char. 116: 1 → 0
Char. 117: 0 → 1
Char. 131: 0 → 1
Char. 156: 1 → 0
Char. 167: 0 → 1
Char. 180: 0 → 1
Char. 189: 0 → 1
Char. 191: 0 → 1
Char. 204: 0 → 2
Char. 267: 0 → 1

**Diadectomorpha:**
Char. 0: 0 → 1
Char. 70: 0 → 1
Char. 75: 0 → 1
Char. 122: 0 → 1
Char. 123: 1 → 0
Char. 146: 1 → 0
Char. 275: 1 → 0
Char. 278: 3 → 0

***Emeroleter levis*:**
Char. 0: 0 → 2
Char. 51: 1 → 0

**Eosauropterygia:**
Char. 159: 1 → 0
Char. 166: 1 → 0
Char. 174: 0 → 1
Char. 194: 0 → 2
Char. 272: 1 → 0

***Eudibamus cursoris*:**
Char. 154: 1 → 2

***Feeserpeton oklahomensis*:**
Char. 51: 0 → 1
Char. 70: 0 → 1
Char. 157: 0 → 1
Char. 158: 0 → 1

***Hovasaurus boulei*:**
Char. 41: 0 → 1
Char. 55: 1 → 0
Char. 72: 2 → 1
Char. 77: 0 → 2
Char. 79: 0 → 1
Char. 93: 1 → 0
Char. 113: 0 → 1
Char. 138: 0 → 1
Char. 141: 1 → 0
Char. 146: 1 → 0
Char. 204: 0 → 2
Char. 215: 0 → 1
Char. 278: 0 → 3

**Kuehneosauridae:**
Char. 7: 0 → 1
Char. 26: 0 → 1
Char. 79: 0 → 2
Char. 82: 0 → 1
Char. 98: 1 → 0
Char. 108: 1 → 0
Char. 113: 0 → 1
Char. 128: 0 → 1
Char. 159: 1 → 0
Char. 181: 0 → 1
Char. 185: 0 → 1
Char. 245: 0 → 1
Char. 278: 0 → 3

***Lanthanosuchus watsoni*:**
Char. 25: 1 → 0
Char. 51: 0 → 1
Char. 76: 0 → 1
Char. 86: 0 → 1
Char. 95: 0 → 1
Char. 98: 1 → 0
Char. 110: 0 → 1
Char. 113: 0 → 1
Char. 114: 0 → 1
Char. 131: 0 → 1
Char. 137: 0 → 1
Char. 138: 0 → 1
Char. 140: 0 → 2
Char. 144: 1 → 0
Char. 154: 1 → 2

***Macroleter poezicus*:**
Char. 0: 0 → 1
Char. 9: 0 → 1
Char. 26: 0 → 1
Char. 52: 0 → 1
Char. 66: 1 → 2
Char. 84: 1 → 0
Char. 87: 0 → 1
Char. 134: 0 → 2

Char. 139: 0 → 1
Char. 140: 0 → 1
Char. 146: 1 → 0
Char. 169: 1 → 0
Char. 235: 0 → 1

***Mesosaurus* spp.:**
Char. 0: 0 → 1
Char. 2: 0 → 1
Char. 5: 1 → 0
Char. 6: 0 → 1
Char. 8: 0 → 1
Char. 9: 0 → 1
Char. 13: 0 → 1
Char. 19: 0 → 1
Char. 23: 0 → 1
Char. 26: 0 → 1
Char. 29: 1 → 0
Char. 33: 0 → 1
Char. 38: 1 → 0
Char. 41: 0 → 1
Char. 50: 1 → 0
Char. 67: 0 → 1
Char. 76: 0 → 1
Char. 83: 0 → 1
Char. 84: 1 → 0
Char. 85: 1 → 0
Char. 94: 0 → 1
Char. 107: 0 → 1
Char. 109: 0 → 1
Char. 111: 0 → 1
Char. 115: 0 → 1
Char. 146: 1 → 0
Char. 148: 0 → 1
Char. 149: 1 → 0
Char. 164: 0 → 1
Char. 167: 0 → 1
Char. 176: 0 → 1
Char. 183: 1 → 0
Char. 184: 0 → 1
Char. 199: 0 → 1
Char. 202: 0 → 1
Char. 204: 0 → 2
Char. 206: 0 → 1
Char. 207: 0 → 1
Char. 209: 0 → 1
Char. 217: 0 → 1
Char. 219: 0 → 1
Char. 220: 0 → 1
Char. 231: 0 → 1
Char. 260: 2 → 0
Char. 272: 2 → 0
Char. 278: 3 → 0

***Microleter mckinzieorum*:**
Char. 0: 0 → 1
Char. 18: 0 → 1
Char. 24: 0 → 1
Char. 25: 1 → 0
Char. 36: 0 → 1
Char. 39: 0 → 1
Char. 51: 0 → 2
Char. 56: 0 → 1
Char. 57: 1 → 0
Char. 70: 0 → 1
Char. 76: 0 → 1
Char. 79: 0 → 1
Char. 83: 0 → 1
Char. 94: 0 → 1
Char. 106: 0 → 1
Char. 110: 0 → 1
Char. 132: 1 → 0
Char. 276: 0 → 1
Char. 278: 3 → 1

**Millerettidae:**
Char. 5: 1 → 0
Char. 24: 0 → 1
Char. 25: 1 → 0
Char. 44: 1 → 0
Char. 56: 0 → 1
Char. 57: 1 → 0
Char. 66: 0 → 2
Char. 78: 1 → 0
Char. 80: 1 → 0
Char. 84: 1 → 2
Char. 88: 1 → 0
Char. 96: 1 → 0
Char. 117: 0 → 1
Char. 124: 0 → 1
Char. 127: 0 → 1
Char. 135: 0 → 1
Char. 145: 0 → 1
Char. 202: 0 → 1
Char. 211: 0 → 1
Char. 230: 0 → 1
Char. 234: 0 → 1
Char. 248: 0 → 1
Char. 252: 0 → 1
Char. 253: 0 → 1

***Nycteroleter ineptus*:**
Char. 278: 0 → 3

***Nyctiphruretus acudens*:**
Char. 0: 0 → 1
Char. 21: 0 → 1
Char. 33: 1 → 2
Char. 41: 0 → 1
Char. 66: 1 → 2
Char. 81: 1 → 0
Char. 83: 0 → 1
Char. 84: 1 → 2
Char. 85: 1 → 0
Char. 94: 0 → 1
Char. 121: 1 → 0
Char. 167: 0 → 1
Char. 224: 0 → 1
Char. 266: 0 → 1
Char. 272: 2 → 1
Char. 276: 0 → 1

***Orovenator mayorum*:**
Char. 8: 0 → 1
Char. 24: 0 → 1
Char. 36: 0 → 1
Char. 160: 0 → 1
Char. 165: 0 → 1

***Owenetta* spp.:**
Char. 169: 1 → 0

***Paleothyris acadiana*:**
Char. 38: 1 → 0
Char. 50: 1 → 0
Char. 66: 1 → 2
Char. 102: 0 → 1
Char. 146: 1 → 0
Char. 180: 0 → 1
Char. 237: 0 → 1
Char. 239: 0 → 1

***Placodus* spp.:**
Char. 0: 1 → 2
Char. 9: 0 → 1
Char. 12: 0 → 1
Char. 13: 0 → 1
Char. 19: 0 → 1
Char. 26: 0 → 1
Char. 31: 0 → 1
Char. 46: 1 → 0
Char. 57: 0 → 1
Char. 78: 1 → 0
Char. 93: 1 → 0
Char. 102: 1 → 2
Char. 109: 1 → 0
Char. 140: 1 → 0
Char. 155: 0 → 1
Char. 163: 1 → 0
Char. 164: 0 → 1

***Procolophon* spp.:**
Char. 41: 0 → 1
Char. 69: 0 → 1
Char. 79: 0 → 1
Char. 83: 0 → 1
Char. 88: 0 → 1
Char. 117: 1 → 0
Char. 121: 1 → 0
Char. 149: 1 → 0
Char. 180: 0 → 1
Char. 204: 0 → 1
Char. 237: 0 → 1
Char. 238: 0 → 1
Char. 272: 2 → 1

***Prolacerta broomi*:**
Char. 58: 1 → 0
Char. 66: 1 → 0
Char. 67: 1 → 0
Char. 80: 1 → 0
Char. 139: 1 → 0
Char. 147: 1 → 0
Char. 192: 1 → 0
Char. 203: 1 → 2

***Rhipaeosaurus* spp.:**
Char. 172: 0 → 1
Char. 277: 0 → 1

**Rhynchocephalia:**
Char. 0: 1 → 2
Char. 24: 0 → 1
Char. 75: 1 → 0
Char. 77: 0 → 1
Char. 94: 1 → 0
Char. 117: 12 → 0
Char. 139: 1 → 0
Char. 167: 1 → 0
Char. 205: 1 → 0

**Rhynchosauria:**
Char. 0: 1 → 0
Char. 7: 0 → 1
Char. 9: 0 → 1
Char. 26: 0 → 1
Char. 68: 0 → 1
Char. 99: 1 → 0
Char. 150: 1 → 0
Char. 160: 0 → 1
Char. 161: 0 → 1
Char. 171: 0 → 2
Char. 182: 1 → 0
Char. 223: 0 → 1
Char. 241: 0 → 1

***Scutosaurus* spp.:**
Char. 175: 0 → 1
Char. 218: 0 → 1
Char. 243: 0 → 2
Char. 244: 0 → 1
Char. 251: 0 → 1

***Sinosaurosphargis yunguiensis*:**
Char. 8: 0 → 1
Char. 30: 0 → 1
Char. 53: 0 → 1
Char. 82: 0 → 1
Char. 89: 1 → 0
Char. 127: 1 → 0
Char. 150: 1 → 0
Char. 154: 0 → 2
Char. 167: 1 → 0
Char. 253: 0 → 1
Char. 255: 0 → 1

**Squamata:**
Char. 26: 0 → 1
Char. 45: 0 → 1
Char. 79: 0 → 2
Char. 80: 1 → 0
Char. 82: 0 → 1
Char. 92: 1 → 0
Char. 109: 1 → 0
Char. 160: 0 → 1
Char. 245: 0 → 1

***Trilophosaurus buettneri*:**
Char. 5: 1 → 0
Char. 11: 0 → 1
Char. 55: 1 → 0
Char. 93: 1 → 0
Char. 104: 0 → 1
Char. 113: 0 → 1
Char. 122: 0 → 1
Char. 136: 1 → 0
Char. 144: 1 → 0
Char. 154: 0 → 1
Char. 157: 0 → 1
Char. 159: 1 → 0
Char. 177: 0 → 12
Char. 194: 0 → 1
Char. 203: 1 → 2
Char. 207: 1 → 0
Char. 208: 1 → 0
Char. 272: 1 → 0

***Youngina capensis*:**
Char. 5: 1 → 0
Char. 25: 0 → 1
Char. 56: 0 → 1
Char. 75: 0 → 1
Char. 92: 1 → 0
Char. 94: 1 → 0
Char. 163: 1 → 0
Char. 170: 0 → 1
Char. 211: 0 → 1
Char. 214: 0 → 1
Char. 215: 0 → 1
Char. 239: 0 → 1

**Node 50:**
Char. 8: 1 → 0
Char. 15: 1 → 0
Char. 20: 1 → 0
Char. 25: 0 → 1
Char. 33: 2 → 0
Char. 67: 1 → 0
Char. 79: 2 → 1
Char. 127: 1 → 0

**Node 51:**
Char. 65: 0 → 1
Char. 184: 0 → 1
Char. 205: 0 → 1
Char. 210: 0 → 1
Char. 241: 0 → 1
Char. 246: 0 → 1
Char. 254: 0 → 1
Char. 255: 0 → 1
Char. 256: 0 → 1
Char. 268: 0 → 1
Char. 269: 0 → 1

**Node 52:**
Char. 0: 1 → 2
Char. 33: 1 → 2
Char. 55: 1 → 0
Char. 62: 1 → 0
Char. 67: 1 → 0
Char. 73: 0 → 1
Char. 112: 1 → 0
Char. 124: 0 → 1
Char. 147: 0 → 1
Char. 148: 0 → 1
Char. 174: 0 → 1
Char. 247: 0 → 1
Char. 251: 0 → 1
Char. 252: 0 → 1
Char. 253: 0 → 2

**Node 53:**
Char. 33: 0 → 1

Char. 92: 0 → 1
Char. 94: 0 → 1
Char. 135: 0 → 1
Char. 278: 1 → 0

**Node 54:**
Char. 20: 0 → 1
Char. 25: 1 → 0
Char. 62: 0 → 1
Char. 141: 0 → 1

**Node 55:**
Char. 0: 0 → 1
Char. 27: 0 → 1
Char. 29: 1 → 0
Char. 40: 2 → 0
Char. 57: 1 → 0
Char. 59: 0 → 1
Char. 67: 0 → 1
Char. 72: 1 → 2
Char. 89: 0 → 1
Char. 111: 0 → 1
Char. 112: 0 → 1
Char. 120: 0 → 1
Char. 121: 1 → 0
Char. 180: 0 → 1
Char. 193: 0 → 1
Char. 222: 0 → 1
Char. 234: 0 → 1
Char. 237: 0 → 1
Char. 266: 0 → 1
Char. 278: 3 → 1

**Node 56:**
Char. 5: 0 → 1
Char. 29: 0 → 1
Char. 40: 0 → 2
Char. 74: 0 → 1
Char. 88: 0 → 1
Char. 116: 0 → 1
Char. 132: 0 → 1
Char. 149: 0 → 1
Char. 169: 0 → 1
Char. 201: 0 → 1
Char. 203: 0 → 1
Char. 235: 0 → 1
Char. 276: 1 → 0

**Node 57:**
Char. 79: 1 → 0
Char. 81: 0 → 1
Char. 93: 0 → 1

**Node 58:**
Char. 78: 0 → 1
Char. 111: 1 → 0
Char. 126: 1 → 0
Char. 135: 1 → 0

**Node 59:**
Char. 19: 0 → 1
Char. 92: 1 → 0

**Node 60:**
Char. 4: 0 → 1
Char. 15: 0 → 1
Char. 29: 0 → 1
Char. 213: 0 → 1
Char. 226: 0 → 2
Char. 228: 0 → 1
Char. 275: 0 → 1

**Node 61:**
Char. 27: 1 → 0
Char. 75: 0 → 1
Char. 107: 0 → 1
Char. 140: 0 → 1
Char. 147: 0 → 1

**Node 62:**
Char. 58: 0 → 1
Char. 61: 0 → 1
Char. 66: 0 → 1
Char. 69: 0 → 1
Char. 150: 0 → 1
Char. 167: 0 → 1
Char. 205: 0 → 1
Char. 208: 0 → 1
Char. 239: 0 → 1

**Node 63:**
Char. 70: 0 → 1
Char. 73: 0 → 1
Char. 117: 0 → 12
Char. 126: 0 → 1
Char. 131: 0 → 1
Char. 182: 0 → 1
Char. 190: 0 → 1
Char. 201: 0 → 1
Char. 214: 0 → 1

**Node 64:**
Char. 73: 0 → 1
Char. 131: 1 → 0
Char. 205: 0 → 1
Char. 276: 0 → 1

**Node 65:**
Char. 18: 0 → 1
Char. 37: 0 → 1
Char. 100: 1 → 0
Char. 107: 0 → 1
Char. 113: 1 → 0
Char. 118: 1 → 0
Char. 125: 1 → 0
Char. 150: 0 → 1

**Node 66:**
Char. 23: 0 → 1
Char. 71: 1 → 0
Char. 85: 1 → 0
Char. 102: 0 → 1
Char. 103: 0 → 1
Char. 106: 0 → 1
Char. 167: 0 → 1
Char. 214: 0 → 1
Char. 216: 1 → 0
Char. 235: 0 → 2
Char. 241: 0 → 1

**Node 67:**
Char. 100: 0 → 1
Char. 113: 0 → 1
Char. 132: 1 → 0
Char. 138: 0 → 1
Char. 141: 0 → 1
Char. 207: 0 → 1

**Node 68:**
Char. 20: 0 → 1
Char. 33: 0 → 1
Char. 38: 1 → 2
Char. 39: 0 → 1
Char. 44: 1 → 0
Char. 49: 0 → 1
Char. 51: 0 → 1
Char. 66: 0 → 1
Char. 70: 0 → 1
Char. 76: 0 → 1
Char. 80: 1 → 0
Char. 88: 1 → 0
Char. 95: 0 → 1
Char. 112: 0 → 1
Char. 117: 0 → 1
Char. 118: 0 → 1
Char. 125: 0 → 1
Char. 131: 0 → 1
Char. 137: 0 → 1
Char. 148: 0 → 1
Char. 158: 0 → 1
Char. 183: 1 → 2
Char. 194: 0 → 1
Char. 201: 1 → 0
Char. 211: 0 → 1
Char. 235: 1 → 0
Char. 252: 0 → 1

**Node 69:**
Char. 87: 0 → 1

**Node 70:**
Char. 25: 1 → 0
Char. 76: 1 → 0

**Node 71:**
Char. 79: 0 → 1
Char. 93: 1 → 0
Char. 133: 0 → 1

**Node 72:**
Char. 38: 1 → 2
Char. 39: 0 → 1
Char. 50: 1 → 0
Char. 58: 0 → 1
Char. 59: 0 → 1
Char. 60: 0 → 1
Char. 72: 1 → 2
Char. 83: 0 → 1
Char. 85: 1 → 0
Char. 88: 1 → 0
Char. 95: 0 → 1
Char. 104: 0 → 1
Char. 105: 0 → 1
Char. 106: 0 → 2
Char. 107: 0 → 1
Char. 109: 0 → 1
Char. 110: 0 → 1
Char. 146: 1 → 0
Char. 148: 0 → 1
Char. 155: 0 → 2
Char. 183: 1 → 2

**Node 73:**
Char. 0: 0 → 2
Char. 33: 1 → 0
Char. 38: 2 → 1
Char. 39: 1 → 0
Char. 42: 0 → 1
Char. 43: 0 → 1
Char. 46: 1 → 0
Char. 48: 1 → 0
Char. 49: 1 → 0
Char. 52: 0 → 1
Char. 83: 0 → 2
Char. 84: 1 → 0
Char. 87: 0 → 1
Char. 93: 1 → 0
Char. 132: 0 → 1
Char. 161: 0 → 1
Char. 163: 1 → 0
Char. 172: 0 → 2
Char. 174: 0 → 1
Char. 188: 0 → 1
Char. 189: 0 → 1
Char. 195: 0 → 1
Char. 204: 0 → 2
Char. 212: 0 → 1
Char. 236: 0 → 1
Char. 238: 0 → 2
Char. 242: 0 → 1
Char. 245: 0 → 1
Char. 274: 0 → 1
Char. 275: 1 → 0

**Node 74:**
Char. 5: 1 → 0
Char. 29: 1 → 0
Char. 44: 1 → 0
Char. 59: 0 → 1
Char. 60: 0 → 1
Char. 66: 0 → 1
Char. 67: 0 → 1
Char. 72: 1 → 2
Char. 78: 1 → 0
Char. 84: 1 → 0
Char. 88: 1 → 0
Char. 93: 1 → 0
Char. 111: 0 → 1
Char. 123: 1 → 0
Char. 129: 0 → 1
Char. 154: 1 → 0
Char. 169: 1 → 0
Char. 170: 0 → 1
Char. 197: 0 → 1

**Node 75:**
Char. 20: 0 → 1
Char. 47: 0 → 1
Char. 83: 0 → 1
Char. 85: 1 → 0
Char. 107: 0 → 1
Char. 110: 0 → 1
Char. 169: 1 → 0
Char. 170: 0 → 1

**Node 76:**
Char. 2: 0 → 1
Char. 6: 0 → 1
Char. 98: 1 → 0
Char. 101: 0 → 1
Char. 113: 0 → 1
Char. 181: 0 → 1
Char. 186: 0 → 1
Char. 193: 1 → 0
Char. 198: 0 → 1
Char. 220: 0 → 1
Char. 239: 1 → 0
Char. 240: 1 → 0
Char. 266: 1 → 0

**Node 77:**
Char. 33: 1 → 2
Char. 68: 0 → 1
Char. 102: 0 → 1
Char. 103: 0 → 1
Char. 223: 0 → 1
Char. 229: 0 → 1
Char. 235: 1 → 2

**Node 78:**
Char. 17: 0 → 1
Char. 42: 0 → 1
Char. 251: 0 → 1
Char. 264: 0 → 1

**Node 79:**
Char. 41: 0 → 1
Char. 61: 1 → 2
Char. 112: 1 → 0
Char. 128: 0 → 1
Char. 138: 0 → 1
Char. 146: 1 → 0
Char. 155: 0 → 1
Char. 182: 1 → 0
Char. 192: 1 → 2
Char. 226: 0 → 1
Char. 227: 0 → 1
Char. 233: 0 → 1

**Node 80:**
Char. 61: 1 → 3
Char. 90: 0 → 1
Char. 91: 0 → 1
Char. 109: 1 → 0
Char. 155: 0 → 1
Char. 209: 1 → 0

**ANALYSIS 8**
**(NO *PROGANOCHELYS QUENSTEDTI* AND *PAPPOCHELYS ROSINAE*)**

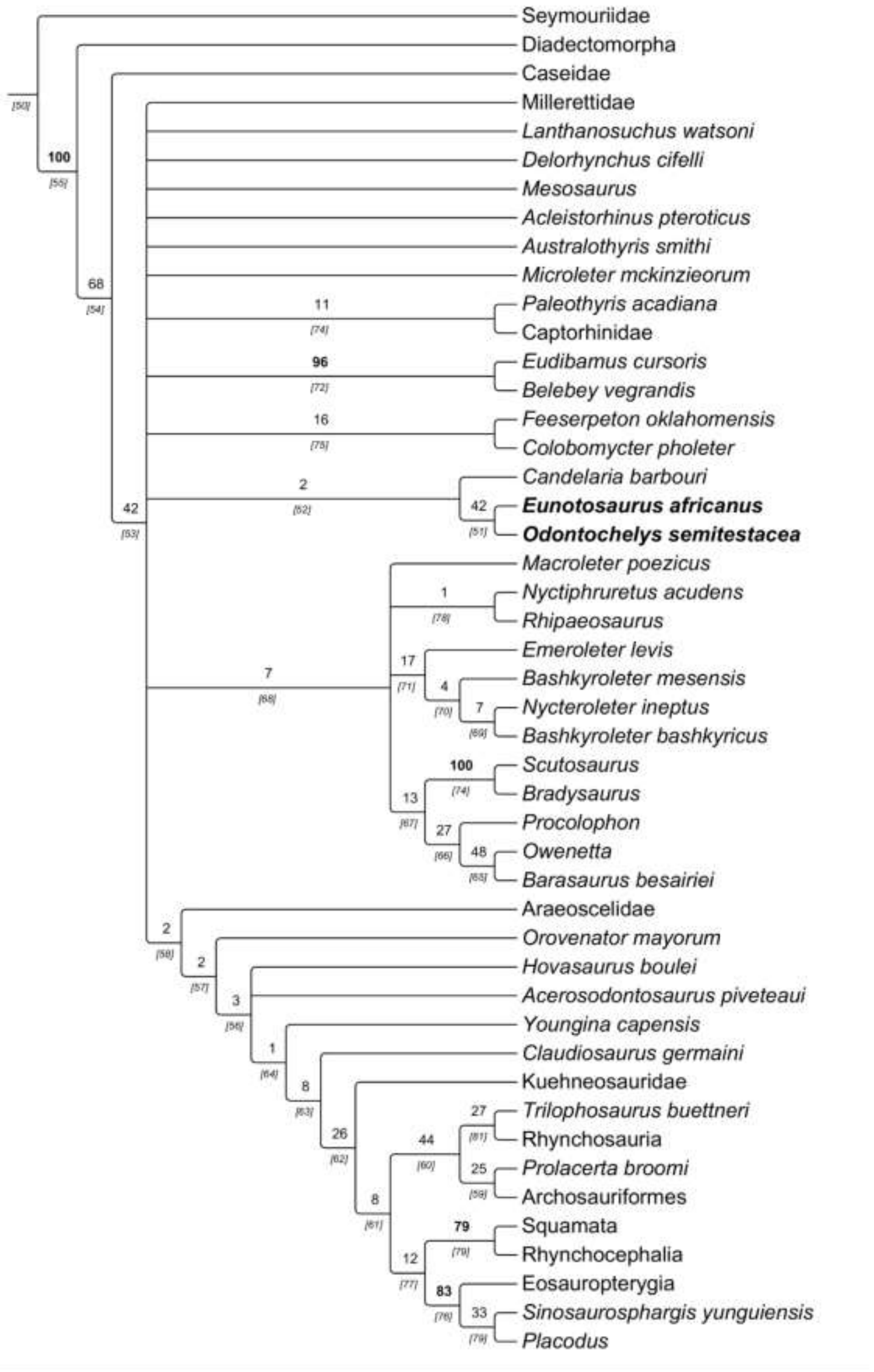

**Seymouriidae:**
Char. 51: 0 → 2
Char. 54: 1 → 0
Char. 83: 0 → 1
Char. 85: 1 → 0
Char. 99: 1 → 0
Char. 107: 0 → 1
Char. 140: 0 → 2
Char. 154: 1 → 2
Char. 207: 0 → 1
Char. 225: 1 → 0

***Odontochelys semitestacea*:**
Char. 46: 1 → 0
Char. 65: 0 → 1
Char. 93: 1 → 0
Char. 176: 0 → 1
Char. 184: 0 → 1
Char. 191: 0 → 1
Char. 195: 0 → 2
Char. 198: 0 → 1
Char. 202: 0 → 1
Char. 205: 0 → 1
Char. 210: 0 → 1
Char. 222: 0 → 1
Char. 223: 0 → 1
Char. 224: 0 → 1
Char. 232: 0 → 1
Char. 235: 1 → 2
Char. 237: 0 → 1
Char. 238: 0 → 1
Char. 241: 0 → 1
Char. 246: 0 → 2
Char. 254: 0 → 1
Char. 255: 0 → 1
Char. 256: 0 → 1
Char. 258: 0 → 1
Char. 259: 0 → 1
Char. 267: 0 → 1
Char. 268: 0 → 1
Char. 269: 0 → 1
Char. 270: 0 → 1
Char. 272: 2 → 1

***Eunotosaurus africanus*:**
Char. 19: 0 → 1
Char. 219: 0 → 1
Char. 248: 0 → 1
Char. 250: 0 → 1
Char. 263: 0 → 1

***Acerosodontosaurus piveteaui*:**
Char. 81: 1 → 0
Char. 128: 0 → 1
Char. 155: 0 → 1
Char. 208: 0 → 1

***Acleistorhinus pteroticus*:**
Char. 20: 0 → 1
Char. 21: 0 → 1
Char. 30: 0 → 1
Char. 33: 0 → 1
Char. 47: 0 → 1
Char. 55: 1 → 0
Char. 56: 0 → 1
Char. 64: 0 → 1
Char. 70: 0 → 1
Char. 79: 0 → 1
Char. 95: 0 → 1
Char. 110: 0 → 1
Char. 113: 0 → 1
Char. 114: 0 → 1
Char. 131: 0 → 1
Char. 137: 0 → 1
Char. 140: 0 → 2
Char. 146: 1 → 0
Char. 169: 1 → 0
Char. 170: 0 → 1
Char. 278: 3 → 1

**Araeoscelidae:**
Char. 5: 1 → 0
Char. 28: 0 → 1
Char. 106: 0 → 1
Char. 116: 1 → 0
Char. 166: 1 → 0
Char. 169: 1 → 0
Char. 170: 0 → 1
Char. 197: 0 → 1
Char. 207: 1 → 0
Char. 221: 1 → 0
Char. 239: 0 → 1

**Archosauriformes:**
Char. 32: 0 → 1
Char. 94: 1 → 0
Char. 112: 1 → 0
Char. 152: 0 → 1
Char. 154: 0 → 2
Char. 166: 1 → 0
Char. 171: 0 → 1
Char. 185: 0 → 1
Char. 204: 0 → 1
Char. 218: 0 → 3
Char. 242: 0 → 1

***Australothyris smithi*:**
Char. 23: 0 → 1
Char. 24: 0 → 1
Char. 34: 0 → 1
Char. 55: 1 → 0
Char. 57: 1 → 0
Char. 71: 1 → 0
Char. 79: 0 → 1
Char. 83: 0 → 1
Char. 85: 1 → 0
Char. 98: 1 → 0
Char. 100: 0 → 1
Char. 103: 0 → 1
Char. 110: 0 → 1
Char. 112: 0 → 1
Char. 123: 1 → 0
Char. 129: 0 → 1
Char. 131: 0 → 1
Char. 132: 1 → 0
Char. 144: 1 → 0
Char. 149: 1 → 0
Char. 150: 0 → 1
Char. 163: 1 → 0

***Barasaurus besairiei*:**
Char. 33: 1 → 0
Char. 75: 1 → 0
Char. 216: 0 → 1

***Bashkyroleter bashkyricus*:**
Char. 275: 1 → 0

***Bashkyroleter mesensis*:**
Char. 169: 1 → 0

***Belebey vegrandis*:**
Char. 154: 1 → 0

***Bradysaurus* spp.:**
Char. 19: 0 → 1
Char. 73: 0 → 1
Char. 79: 0 → 1
Char. 135: 1 → 0
Char. 249: 0 → 1

***Candelaria barbouri*:**
Char. 1: 0 → 1
Char. 5: 1 → 0
Char. 8: 0 → 1
Char. 23: 0 → 1
Char. 49: 0 → 1
Char. 50: 1 → 0
Char. 57: 1 → 0
Char. 67: 0 → 1
Char. 79: 0 → 2
Char. 83: 0 → 1
Char. 95: 0 → 1
Char. 126: 0 → 1
Char. 132: 1 → 0
Char. 154: 1 → 2
Char. 169: 1 → 0

**Captorhinidae:**
Char. 3: 0 → 1
Char. 23: 0 → 1
Char. 25: 1 → 0
Char. 26: 0 → 1
Char. 73: 0 → 1
Char. 75: 0 → 1
Char. 83: 0 → 1
Char. 108: 1 → 0
Char. 183: 1 → 0
Char. 201: 1 → 0
Char. 203: 1 → 2
Char. 216: 1 → 0

**Caseidae:**
Char. 24: 0 → 1
Char. 25: 1 → 0
Char. 36: 0 → 1
Char. 38: 1 → 0
Char. 46: 1 → 0
Char. 50: 1 → 0
Char. 56: 0 → 1
Char. 85: 1 → 0
Char. 98: 1 → 0
Char. 170: 0 → 1
Char. 194: 0 → 1
Char. 273: 0 → 1
Char. 274: 0 → 1
Char. 276: 0 → 1

***Claudiosaurus germaini*:**
Char. 64: 0 → 1
Char. 105: 0 → 1
Char. 130: 0 → 1
Char. 144: 1 → 0
Char. 166: 1 → 0
Char. 187: 1 → 0
Char. 199: 0 → 1
Char. 203: 1 → 2
Char. 204: 0 → 1
Char. 220: 0 → 1
Char. 222: 1 → 0

***Colobomycter pholeter*:**
Char. 21: 0 → 1
Char. 25: 1 → 0
Char. 84: 1 → 0
Char. 154: 1 → 0
Char. 167: 0 → 1
Char. 267: 0 → 1

***Delorhynchus cifelli*:**
Char. 18: 0 → 1
Char. 20: 0 → 1
Char. 21: 0 → 1
Char. 24: 0 → 1
Char. 26: 0 → 1
Char. 28: 0 → 1
Char. 33: 0 → 2
Char. 39: 0 → 1
Char. 52: 0 → 1
Char. 100: 0 → 1
Char. 111: 0 → 1
Char. 116: 1 → 0
Char. 117: 0 → 1
Char. 119: 1 → 0
Char. 131: 0 → 1
Char. 156: 1 → 0
Char. 167: 0 → 1
Char. 189: 0 → 1
Char. 191: 0 → 1
Char. 267: 0 → 1

**Diadectomorpha:**
Char. 0: 0 → 1
Char. 70: 0 → 1
Char. 122: 0 → 1
Char. 123: 1 → 0
Char. 146: 1 → 0

***Emeroleter levis*:**
Char. 0: 0 → 2
Char. 51: 1 → 0

**Eosauropterygia:**
Char. 159: 1 → 0
Char. 166: 1 → 0
Char. 174: 0 → 1
Char. 194: 0 → 2
Char. 272: 1 → 0

***Eudibamus cursoris*:**
Char. 154: 1 → 2
Char. 163: 1 → 0

***Feeserpeton oklahomensis*:**
Char. 51: 0 → 1
Char. 70: 0 → 1
Char. 157: 0 → 1
Char. 158: 0 → 1

***Hovasaurus boulei*:**
Char. 41: 0 → 1
Char. 55: 1 → 0
Char. 72: 2 → 1
Char. 77: 0 → 2
Char. 79: 0 → 1
Char. 93: 1 → 0
Char. 113: 0 → 1
Char. 138: 0 → 1
Char. 146: 1 → 0
Char. 204: 0 → 2
Char. 220: 0 → 1
Char. 278: 1 → 3

**Kuehneosauridae:**
Char. 7: 0 → 1
Char. 26: 0 → 1
Char. 79: 0 → 2
Char. 82: 0 → 1
Char. 98: 1 → 0
Char. 108: 1 → 0
Char. 113: 0 → 1
Char. 128: 0 → 1
Char. 159: 1 → 0
Char. 181: 0 → 1
Char. 185: 0 → 1
Char. 206: 0 → 2
Char. 245: 0 → 1
Char. 278: 0 → 3

***Lanthanosuchus watsoni*:**
Char. 25: 1 → 0
Char. 51: 0 → 1
Char. 86: 0 → 1
Char. 95: 0 → 1
Char. 98: 1 → 0
Char. 110: 0 → 1
Char. 113: 0 → 1
Char. 114: 0 → 1
Char. 119: 1 → 0
Char. 131: 0 → 1
Char. 137: 0 → 1
Char. 138: 0 → 1
Char. 140: 0 → 2
Char. 144: 1 → 0
Char. 154: 1 → 2

***Macroleter poezicus*:**
Char. 0: 0 → 1
Char. 9: 0 → 1
Char. 26: 0 → 1
Char. 52: 0 → 1

Char. 66: 1 → 2
Char. 75: 0 → 1
Char. 84: 1 → 0
Char. 87: 0 → 1
Char. 110: 0 → 1
Char. 126: 0 → 1
Char. 134: 0 → 2
Char. 139: 0 → 1
Char. 140: 0 → 1
Char. 143: 0 → 1
Char. 146: 1 → 0
Char. 147: 1 → 2
Char. 154: 1 → 0
Char. 169: 1 → 0
Char. 278: 3 → 0

***Mesosaurus* spp.:**
Char. 0: 0 → 1
Char. 2: 0 → 1
Char. 5: 1 → 0
Char. 6: 0 → 1
Char. 8: 0 → 1
Char. 9: 0 → 1
Char. 13: 0 → 1
Char. 19: 0 → 1
Char. 23: 0 → 1
Char. 26: 0 → 1
Char. 29: 1 → 0
Char. 33: 0 → 1
Char. 38: 1 → 0
Char. 41: 0 → 1
Char. 50: 1 → 0
Char. 67: 0 → 1
Char. 83: 0 → 1
Char. 84: 1 → 0
Char. 85: 1 → 0
Char. 94: 0 → 1
Char. 107: 0 → 1
Char. 109: 0 → 1
Char. 111: 0 → 1
Char. 115: 0 → 1
Char. 146: 1 → 0
Char. 147: 1 → 0
Char. 148: 0 → 1
Char. 149: 1 → 0
Char. 163: 1 → 0
Char. 164: 0 → 1
Char. 166: 1 → 0
Char. 167: 0 → 1
Char. 176: 0 → 1
Char. 183: 1 → 0
Char. 184: 0 → 1
Char. 199: 0 → 1
Char. 202: 0 → 1
Char. 206: 0 → 1
Char. 209: 0 → 1
Char. 217: 0 → 1
Char. 219: 0 → 1
Char. 220: 0 → 1
Char. 231: 0 → 1
Char. 260: 2 → 0
Char. 272: 2 → 0
Char. 278: 3 → 0

***Microleter mckinzieorum*:**
Char. 0: 0 → 1
Char. 18: 0 → 1
Char. 24: 0 → 1
Char. 25: 1 → 0
Char. 36: 0 → 1
Char. 39: 0 → 1
Char. 51: 0 → 2
Char. 56: 0 → 1
Char. 57: 1 → 0
Char. 70: 0 → 1
Char. 79: 0 → 1
Char. 83: 0 → 1
Char. 94: 0 → 1
Char. 106: 0 → 1
Char. 110: 0 → 1
Char. 132: 1 → 0
Char. 166: 1 → 0
Char. 276: 0 → 1
Char. 278: 3 → 1

**Millerettidae:**
Char. 5: 1 → 0
Char. 24: 0 → 1
Char. 25: 1 → 0
Char. 44: 1 → 0
Char. 56: 0 → 1
Char. 57: 1 → 0
Char. 66: 0 → 2
Char. 78: 1 → 0
Char. 80: 1 → 0
Char. 84: 1 → 2
Char. 96: 1 → 0
Char. 117: 0 → 1
Char. 124: 0 → 1
Char. 127: 0 → 1
Char. 135: 0 → 1
Char. 145: 0 → 1
Char. 147: 1 → 0
Char. 163: 1 → 0
Char. 166: 1 → 0
Char. 202: 0 → 1
Char. 211: 0 → 1
Char. 230: 0 → 1
Char. 240: 0 → 1
Char. 248: 0 → 1
Char. 252: 0 → 1
Char. 253: 0 → 1

***Nycteroleter ineptus*:**
Char. 278: 0 → 3

***Nyctiphruretus acudens*:**
Char. 167: 0 → 1
Char. 215: 1 → 0
Char. 224: 0 → 1
Char. 226: 1 → 0

***Orovenator mayorum*:**
Char. 8: 0 → 1
Char. 24: 0 → 1
Char. 36: 0 → 1
Char. 160: 0 → 1
Char. 165: 0 → 1

***Owenetta* spp.:**
Char. 169: 1 → 0

***Paleothyris acadiana*:**
Char. 38: 1 → 0
Char. 50: 1 → 0
Char. 66: 1 → 2
Char. 102: 0 → 1
Char. 146: 1 → 0
Char. 237: 0 → 1
Char. 239: 0 → 1
Char. 240: 0 → 1

***Placodus* spp.:**
Char. 0: 1 → 2
Char. 9: 0 → 1
Char. 12: 0 → 1
Char. 13: 0 → 1
Char. 19: 0 → 1
Char. 26: 0 → 1
Char. 31: 0 → 1
Char. 46: 1 → 0
Char. 57: 0 → 1
Char. 78: 1 → 0
Char. 93: 1 → 0
Char. 102: 1 → 2
Char. 109: 1 → 0
Char. 140: 1 → 0
Char. 155: 0 → 1
Char. 163: 1 → 0
Char. 164: 0 → 1

***Procolophon* spp.:**
Char. 41: 0 → 1
Char. 69: 0 → 1
Char. 79: 0 → 1
Char. 83: 0 → 1
Char. 88: 0 → 1
Char. 117: 1 → 0
Char. 149: 1 → 0
Char. 237: 0 → 1
Char. 238: 0 → 1
Char. 272: 2 → 1
Char. 278: 3 → 0

***Prolacerta broomi*:**
Char. 58: 1 → 0
Char. 66: 1 → 0
Char. 67: 1 → 0
Char. 80: 1 → 0
Char. 139: 1 → 0
Char. 147: 1 → 0
Char. 192: 1 → 0
Char. 203: 1 → 2
Char. 206: 0 → 12

***Rhipaeosaurus* spp.:**
Char. 172: 0 → 1
Char. 186: 1 → 0
Char. 277: 0 → 1

**Rhynchocephalia:**
Char. 0: 1 → 2
Char. 24: 0 → 1
Char. 75: 1 → 0
Char. 77: 0 → 1
Char. 94: 1 → 0
Char. 117: 12 → 0
Char. 139: 1 → 0
Char. 167: 1 → 0
Char. 205: 1 → 0

**Rhynchosauria:**
Char. 0: 1 → 0
Char. 7: 0 → 1
Char. 9: 0 → 1
Char. 26: 0 → 1
Char. 68: 0 → 1
Char. 99: 1 → 0
Char. 150: 1 → 0
Char. 160: 0 → 1
Char. 161: 0 → 1
Char. 171: 0 → 2
Char. 182: 1 → 0
Char. 223: 0 → 1
Char. 241: 0 → 1

***Scutosaurus* spp.:**
Char. 175: 0 → 1
Char. 218: 0 → 1
Char. 243: 0 → 2
Char. 244: 0 → 1
Char. 251: 0 → 1

***Sinosaurosphargis yunguiensis*:**
Char. 8: 0 → 1
Char. 30: 0 → 1
Char. 53: 0 → 1
Char. 82: 0 → 1
Char. 89: 1 → 0
Char. 127: 1 → 0
Char. 150: 1 → 0
Char. 154: 0 → 2
Char. 167: 1 → 0
Char. 253: 0 → 1
Char. 255: 0 → 1

**Squamata:**
Char. 26: 0 → 1
Char. 45: 0 → 1
Char. 79: 0 → 2
Char. 80: 1 → 0
Char. 82: 0 → 1
Char. 92: 1 → 0
Char. 109: 1 → 0
Char. 160: 0 → 1
Char. 245: 0 → 1

***Trilophosaurus buettneri*:**
Char. 5: 1 → 0
Char. 11: 0 → 1
Char. 55: 1 → 0
Char. 93: 1 → 0
Char. 104: 0 → 1
Char. 113: 0 → 1
Char. 122: 0 → 1
Char. 136: 1 → 0
Char. 144: 1 → 0
Char. 154: 0 → 1
Char. 157: 0 → 1
Char. 159: 1 → 0
Char. 177: 0 → 12
Char. 194: 0 → 1
Char. 203: 1 → 2
Char. 207: 1 → 0
Char. 208: 1 → 0
Char. 272: 1 → 0

***Youngina capensis*:**
Char. 5: 1 → 0
Char. 21: 0 → 1
Char. 75: 0 → 1
Char. 92: 1 → 0
Char. 163: 1 → 0
Char. 170: 0 → 1
Char. 211: 0 → 1

**Node 50:**
Char. 20: 1 → 0
Char. 25: 0 → 1
Char. 65: 1 → 0
Char. 72: 2 → 0
Char. 73: 1 → 0
Char. 131: 1 → 0
Char. 184: 1 → 0
Char. 187: 1 → 0
Char. 192: 1 → 0
Char. 194: 2 → 0
Char. 205: 1 → 0
Char. 210: 1 → 0
Char. 217: 1 → 0
Char. 220: 1 → 0
Char. 221: 1 → 0
Char. 222: 1 → 0
Char. 241: 1 → 0
Char. 246: 1 → 0
Char. 247: 1 → 0
Char. 254: 1 → 0
Char. 255: 1 → 0
Char. 256: 1 → 0
Char. 266: 1 → 0
Char. 267: 1 → 0
Char. 268: 1 → 0
Char. 269: 1 → 0

**Node 51:**
Char. 0: 0 → 2
Char. 43: 0 → 1

**Node 52:**
Char. 15: 0 → 1
Char. 20: 0 → 1
Char. 25: 1 → 0
Char. 29: 1 → 0
Char. 33: 0 → 2
Char. 55: 1 → 0
Char. 94: 0 → 1
Char. 127: 0 → 1
Char. 276: 0 → 1
Char. 277: 0 → 1

**Node 53:**
Char. 5: 0 → 1
Char. 29: 0 → 1

Char. 40: 0 → 2
Char. 74: 0 → 1
Char. 116: 0 → 1
Char. 119: 0 → 1
Char. 147: 0 → 1
Char. 149: 0 → 1
Char. 163: 0 → 1
Char. 166: 0 → 1
Char. 169: 0 → 1
Char. 201: 0 → 1
Char. 203: 0 → 1
Char. 207: 0 → 1
Char. 221: 0 → 1
Char. 235: 0 → 1

**Node 54:**
Char. 72: 0 → 1
Char. 81: 0 → 1
Char. 93: 0 → 1

**Node 55:**
Char. 78: 0 → 1
Char. 111: 1 → 0
Char. 126: 1 → 0
Char. 135: 1 → 0

**Node 56:**
Char. 135: 0 → 1

**Node 57:**
Char. 20: 0 → 1
Char. 62: 0 → 1

**Node 58:**
Char. 0: 0 → 1
Char. 27: 0 → 1
Char. 29: 1 → 0
Char. 38: 1 → 0
Char. 40: 2 → 0
Char. 57: 1 → 0
Char. 59: 0 → 1
Char. 67: 0 → 1
Char. 72: 1 → 2
Char. 84: 1 → 0
Char. 89: 0 → 1
Char. 111: 0 → 1
Char. 112: 0 → 1
Char. 120: 0 → 1
Char. 147: 1 → 0
Char. 193: 0 → 1
Char. 222: 0 → 1
Char. 237: 0 → 1
Char. 240: 0 → 1
Char. 266: 0 → 1
Char. 278: 3 → 1

**Node 59:**
Char. 19: 0 → 1
Char. 92: 1 → 0

**Node 60:**
Char. 4: 0 → 1
Char. 15: 0 → 1
Char. 29: 0 → 1
Char. 213: 0 → 1
Char. 226: 0 → 2
Char. 228: 0 → 1
Char. 275: 0 → 1

**Node 61:**
Char. 27: 1 → 0
Char. 75: 0 → 1
Char. 107: 0 → 1
Char. 140: 0 → 1
Char. 147: 0 → 1

**Node 62:**
Char. 58: 0 → 1
Char. 61: 0 → 1
Char. 66: 0 → 1
Char. 69: 0 → 1
Char. 150: 0 → 1
Char. 167: 0 → 1
Char. 205: 0 → 1
Char. 208: 0 → 1

**Node 63:**
Char. 70: 0 → 1
Char. 73: 0 → 1
Char. 126: 0 → 1
Char. 131: 0 → 1
Char. 182: 0 → 1

**Node 64:**
Char. 56: 0 → 1
Char. 134: 0 → 1
Char. 214: 0 → 1
Char. 219: 0 → 1
Char. 267: 0 → 1
Char. 278: 1 → 0

**Node 65:**
Char. 73: 0 → 1
Char. 131: 1 → 0
Char. 205: 0 → 1
Char. 276: 0 → 1

**Node 66:**
Char. 18: 0 → 1
Char. 37: 0 → 1
Char. 107: 0 → 1
Char. 118: 1 → 0
Char. 125: 1 → 0
Char. 126: 0 → 1
Char. 150: 0 → 1

**Node 67:**
Char. 23: 0 → 1
Char. 71: 1 → 0
Char. 75: 0 → 1
Char. 85: 1 → 0
Char. 102: 0 → 1
Char. 103: 0 → 1
Char. 106: 0 → 1
Char. 110: 0 → 1
Char. 155: 0 → 1
Char. 167: 0 → 1
Char. 214: 0 → 1
Char. 216: 1 → 0
Char. 241: 0 → 1

**Node 68:**
Char. 20: 0 → 1
Char. 33: 0 → 1
Char. 38: 1 → 2
Char. 39: 0 → 1
Char. 44: 1 → 0
Char. 49: 0 → 1
Char. 51: 0 → 1
Char. 66: 0 → 1
Char. 70: 0 → 1
Char. 80: 1 → 0
Char. 95: 0 → 1
Char. 112: 0 → 1
Char. 117: 0 → 1
Char. 118: 0 → 1
Char. 125: 0 → 1
Char. 131: 0 → 1
Char. 135: 0 → 1
Char. 137: 0 → 1
Char. 148: 0 → 1
Char. 157: 0 → 1
Char. 158: 0 → 1
Char. 183: 1 → 2
Char. 186: 0 → 1
Char. 194: 0 → 1
Char. 197: 0 → 1
Char. 201: 1 → 0
Char. 211: 0 → 1
Char. 215: 0 → 1
Char. 226: 0 → 1
Char. 252: 0 → 1

**Node 69:**
Char. 87: 0 → 1
Char. 110: 0 → 1

**Node 70:**
Char. 25: 1 → 0
Char. 76: 1 → 0

**Node 71:**
Char. 79: 0 → 1
Char. 86: 0 → 1
Char. 93: 1 → 0
Char. 133: 0 → 1
Char. 147: 1 → 2
Char. 154: 1 → 0
Char. 278: 3 → 0

**Node 72:**
Char. 38: 1 → 2
Char. 39: 0 → 1
Char. 50: 1 → 0
Char. 58: 0 → 1
Char. 59: 0 → 1
Char. 60: 0 → 1
Char. 72: 1 → 2
Char. 83: 0 → 1
Char. 85: 1 → 0
Char. 95: 0 → 1
Char. 104: 0 → 1
Char. 105: 0 → 1
Char. 106: 0 → 2
Char. 107: 0 → 1
Char. 109: 0 → 1
Char. 110: 0 → 1
Char. 146: 1 → 0
Char. 148: 0 → 1
Char. 155: 0 → 2
Char. 183: 1 → 2

**Node 73:**
Char. 0: 0 → 2
Char. 33: 1 → 0
Char. 38: 2 → 1
Char. 39: 1 → 0
Char. 42: 0 → 1
Char. 43: 0 → 1
Char. 46: 1 → 0
Char. 49: 1 → 0
Char. 52: 0 → 1
Char. 83: 0 → 2
Char. 84: 1 → 0
Char. 87: 0 → 1
Char. 93: 1 → 0
Char. 143: 0 → 1
Char. 161: 0 → 1
Char. 163: 1 → 0
Char. 172: 0 → 2
Char. 174: 0 → 1
Char. 188: 0 → 1
Char. 189: 0 → 1
Char. 195: 0 → 1
Char. 212: 0 → 1
Char. 221: 1 → 0
Char. 236: 0 → 1
Char. 238: 0 → 2
Char. 242: 0 → 1
Char. 245: 0 → 1
Char. 274: 0 → 1
Char. 275: 1 → 0

**Node 74:**
Char. 5: 1 → 0
Char. 29: 1 → 0
Char. 44: 1 → 0
Char. 59: 0 → 1
Char. 60: 0 → 1
Char. 66: 0 → 1
Char. 67: 0 → 1
Char. 72: 1 → 2
Char. 78: 1 → 0
Char. 84: 1 → 0
Char. 93: 1 → 0
Char. 111: 0 → 1
Char. 123: 1 → 0
Char. 129: 0 → 1
Char. 147: 1 → 0
Char. 154: 1 → 0
Char. 163: 1 → 0
Char. 169: 1 → 0
Char. 170: 0 → 1
Char. 197: 0 → 1
Char. 207: 1 → 0
Char. 221: 1 → 0

**Node 75:**
Char. 20: 0 → 1
Char. 47: 0 → 1
Char. 83: 0 → 1
Char. 85: 1 → 0
Char. 107: 0 → 1
Char. 110: 0 → 1
Char. 169: 1 → 0
Char. 170: 0 → 1

**Node 76:**
Char. 2: 0 → 1
Char. 6: 0 → 1
Char. 98: 1 → 0
Char. 101: 0 → 1
Char. 113: 0 → 1
Char. 181: 0 → 1
Char. 186: 0 → 1
Char. 193: 1 → 0
Char. 198: 0 → 1
Char. 206: 0 → 2
Char. 220: 0 → 1
Char. 239: 1 → 0
Char. 240: 1 → 0
Char. 266: 1 → 0

**Node 77:**
Char. 33: 1 → 2
Char. 68: 0 → 1
Char. 102: 0 → 1
Char. 103: 0 → 1
Char. 223: 0 → 1
Char. 229: 0 → 1
Char. 235: 1 → 2

**Node 78:**
Char. 207: 1 → 0

**Node 79:**
Char. 17: 0 → 1
Char. 42: 0 → 1
Char. 251: 0 → 1
Char. 264: 0 → 1

**Node 80:**
Char. 41: 0 → 1
Char. 61: 1 → 2
Char. 112: 1 → 0
Char. 128: 0 → 1
Char. 138: 0 → 1
Char. 146: 1 → 0
Char. 155: 0 → 1
Char. 182: 1 → 0
Char. 192: 1 → 2
Char. 226: 0 → 1
Char. 227: 0 → 1
Char. 233: 0 → 1

**Node 81:**
Char. 61: 1 → 3
Char. 90: 0 → 1
Char. 91: 0 → 1
Char. 109: 1 → 0
Char. 155: 0 → 1
Char. 209: 1 → 0

**ANALYSIS 9**
**(NO *PROGANOCHELYS QUENSTEDTI* AND *ODONTOCHELYS SEMITESTACEA*)**

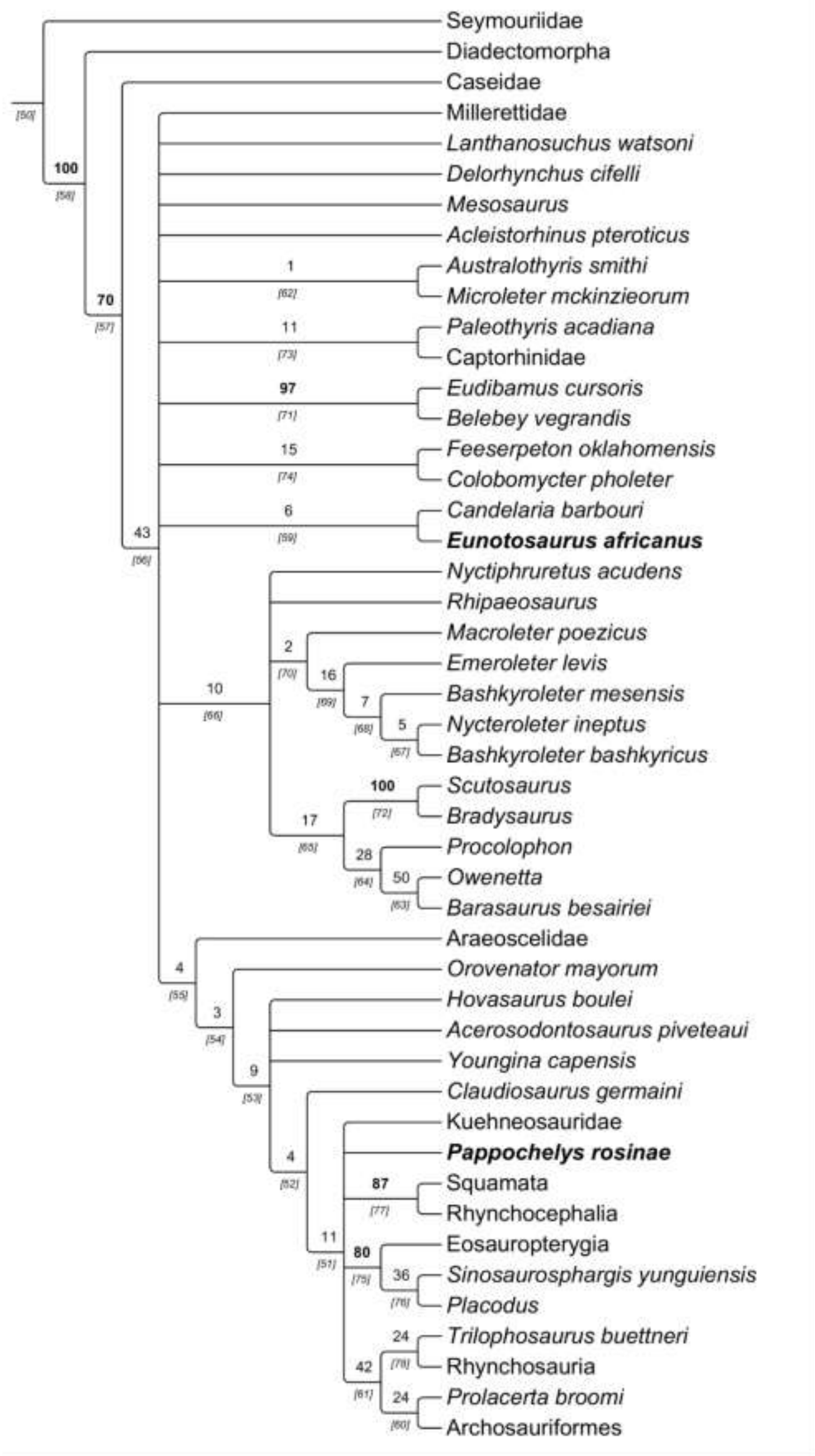

**Seymouriidae:**
Char. 23: 0 → 1
Char. 51: 0 → 2
Char. 54: 1 → 0
Char. 55: 1 → 0
Char. 83: 0 → 1
Char. 85: 1 → 0
Char. 99: 1 → 0
Char. 107: 0 → 1
Char. 140: 0 → 2
Char. 154: 1 → 2
Char. 225: 1 → 0
Char. 260: 2 → 0

***Pappochelys rosinae*:**
Char. 0: 1 → 0
Char. 1: 0 → 1
Char. 5: 1 → 0
Char. 12: 0 → 1
Char. 43: 1 → 0
Char. 49: 0 → 1
Char. 55: 1 → 0
Char. 61: 1 → 0
Char. 62: 1 → 0
Char. 65: 0 → 1
Char. 67: 1 → 0
Char. 78: 1 → 0
Char. 91: 0 → 1
Char. 169: 1 → 0
Char. 176: 1 → 0
Char. 193: 1 → 0
Char. 194: 0 → 2
Char. 201: 1 → 0
Char. 214: 1 → 0
Char. 219: 1 → 0
Char. 220: 0 → 1
Char. 241: 0 → 1
Char. 246: 0 → 1
Char. 247: 0 → 1
Char. 252: 0 → 1
Char. 254: 0 → 1
Char. 255: 0 → 1
Char. 256: 0 → 1
Char. 260: 1 → 0
Char. 268: 0 → 1
Char. 269: 0 → 1

***Eunotosaurus africanus*:**
Char. 0: 0 → 2
Char. 19: 0 → 1
Char. 21: 0 → 1
Char. 24: 0 → 1
Char. 42: 0 → 1
Char. 43: 0 → 1
Char. 81: 1 → 0
Char. 84: 1 → 2
Char. 92: 0 → 1
Char. 273: 0 → 1
Char. 274: 0 → 1

***Acerosodontosaurus piveteaui*:**
Char. 78: 1 → 0
Char. 81: 1 → 0
Char. 128: 0 → 1
Char. 155: 0 → 1
Char. 208: 0 → 1

***Acleistorhinus pteroticus*:**
Char. 20: 0 → 1
Char. 21: 0 → 1
Char. 30: 0 → 1
Char. 33: 0 → 1
Char. 47: 0 → 1
Char. 48: 0 → 1
Char. 55: 1 → 0
Char. 56: 0 → 1
Char. 64: 0 → 1
Char. 70: 0 → 1
Char. 79: 0 → 1
Char. 95: 0 → 1
Char. 110: 0 → 1
Char. 113: 0 → 1
Char. 114: 0 → 1
Char. 131: 0 → 1
Char. 137: 0 → 1
Char. 140: 0 → 2
Char. 146: 1 → 0
Char. 169: 1 → 0
Char. 170: 0 → 1
Char. 278: 3 → 1

**Araeoscelidae:**
Char. 5: 1 → 0
Char. 28: 0 → 1
Char. 106: 0 → 1
Char. 116: 1 → 0
Char. 166: 1 → 0
Char. 169: 1 → 0
Char. 170: 0 → 1
Char. 197: 0 → 1
Char. 221: 1 → 0
Char. 239: 0 → 1

**Archosauriformes:**
Char. 32: 0 → 1
Char. 94: 1 → 0
Char. 112: 1 → 0
Char. 152: 0 → 1
Char. 154: 0 → 2
Char. 166: 1 → 0
Char. 171: 0 → 1
Char. 185: 0 → 1
Char. 204: 0 → 1
Char. 218: 0 → 3
Char. 242: 0 → 1

***Australothyris smithi*:**
Char. 23: 0 → 1
Char. 34: 0 → 1
Char. 55: 1 → 0
Char. 71: 1 → 0
Char. 85: 1 → 0
Char. 112: 0 → 1
Char. 129: 0 → 1
Char. 131: 0 → 1

***Barasaurus besairiei*:**
Char. 33: 1 → 0
Char. 75: 1 → 0
Char. 216: 0 → 1

***Bashkyroleter bashkyricus*:**
Char. 275: 1 → 0

***Bashkyroleter mesensis*:**
Char. 169: 1 → 0

***Belebey vegrandis*:**
Char. 154: 1 → 0

***Bradysaurus* spp.:**
Char. 19: 0 → 1
Char. 73: 0 → 1
Char. 79: 0 → 1
Char. 135: 1 → 0
Char. 249: 0 → 1

***Candelaria barbouri*:**
Char. 1: 0 → 1
Char. 5: 1 → 0
Char. 8: 0 → 1
Char. 23: 0 → 1
Char. 49: 0 → 1
Char. 50: 1 → 0
Char. 57: 1 → 0
Char. 67: 0 → 1
Char. 79: 0 → 2
Char. 83: 0 → 1
Char. 95: 0 → 1
Char. 126: 0 → 1
Char. 132: 1 → 0
Char. 154: 1 → 2
Char. 169: 1 → 0

**Captorhinidae:**
Char. 3: 0 → 1
Char. 23: 0 → 1
Char. 25: 1 → 0
Char. 26: 0 → 1
Char. 73: 0 → 1
Char. 75: 0 → 1
Char. 83: 0 → 1
Char. 108: 1 → 0
Char. 183: 1 → 0
Char. 201: 1 → 0
Char. 203: 1 → 2
Char. 216: 1 → 0

**Caseidae:**
Char. 24: 0 → 1
Char. 25: 1 → 0
Char. 36: 0 → 1
Char. 38: 1 → 0
Char. 46: 1 → 0
Char. 50: 1 → 0
Char. 56: 0 → 1
Char. 85: 1 → 0
Char. 98: 1 → 0
Char. 170: 0 → 1
Char. 194: 0 → 1
Char. 273: 0 → 1
Char. 274: 0 → 1

***Claudiosaurus germaini*:**
Char. 24: 0 → 1
Char. 34: 0 → 1
Char. 36: 0 → 1
Char. 43: 1 → 0
Char. 56: 0 → 1
Char. 64: 0 → 1
Char. 105: 0 → 1
Char. 130: 0 → 1
Char. 144: 1 → 0
Char. 166: 1 → 0
Char. 187: 1 → 0
Char. 199: 0 → 1
Char. 203: 1 → 2
Char. 204: 0 → 1
Char. 220: 0 → 1
Char. 222: 1 → 0

***Colobomycter pholeter*:**
Char. 21: 0 → 1
Char. 25: 1 → 0
Char. 84: 1 → 0
Char. 154: 1 → 0
Char. 167: 0 → 1
Char. 267: 0 → 1

***Delorhynchus cifelli*:**
Char. 18: 0 → 1
Char. 20: 0 → 1
Char. 21: 0 → 1
Char. 24: 0 → 1
Char. 26: 0 → 1
Char. 28: 0 → 1
Char. 33: 0 → 2
Char. 39: 0 → 1
Char. 48: 0 → 1
Char. 52: 0 → 1
Char. 100: 0 → 1
Char. 111: 0 → 1
Char. 116: 1 → 0
Char. 117: 0 → 1
Char. 119: 1 → 0
Char. 131: 0 → 1
Char. 156: 1 → 0
Char. 167: 0 → 1
Char. 189: 0 → 1
Char. 191: 0 → 1
Char. 192: 0 → 1
Char. 267: 0 → 1

**Diadectomorpha:**
Char. 0: 0 → 1
Char. 70: 0 → 1
Char. 75: 0 → 1
Char. 122: 0 → 1
Char. 123: 1 → 0
Char. 146: 1 → 0

***Emeroleter levis*:**
Char. 0: 0 → 2
Char. 51: 1 → 0

**Eosauropterygia:**
Char. 159: 1 → 0
Char. 166: 1 → 0
Char. 174: 0 → 1
Char. 178: 0 → 1
Char. 194: 0 → 2
Char. 272: 1 → 0

***Eudibamus cursoris*:**
Char. 154: 1 → 2
Char. 163: 1 → 0

***Feeserpeton oklahomensis*:**
Char. 51: 0 → 1
Char. 70: 0 → 1
Char. 157: 0 → 1
Char. 158: 0 → 1

***Hovasaurus boulei*:**
Char. 41: 0 → 1
Char. 55: 1 → 0
Char. 60: 1 → 0
Char. 72: 2 → 1
Char. 77: 0 → 2
Char. 78: 1 → 0
Char. 79: 0 → 1
Char. 93: 1 → 0
Char. 113: 0 → 1
Char. 138: 0 → 1
Char. 146: 1 → 0
Char. 154: 0 → 1
Char. 204: 0 → 2
Char. 220: 0 → 1
Char. 278: 01 → 3

**Kuehneosauridae:**
Char. 7: 0 → 1
Char. 24: 0 → 1
Char. 26: 0 → 1
Char. 27: 0 → 1
Char. 34: 0 → 1
Char. 36: 0 → 1
Char. 43: 1 → 0
Char. 56: 0 → 1
Char. 75: 1 → 0
Char. 79: 0 → 2
Char. 81: 1 → 0
Char. 82: 0 → 1
Char. 98: 1 → 0
Char. 107: 1 → 0
Char. 108: 1 → 0
Char. 113: 0 → 1
Char. 128: 0 → 1
Char. 140: 1 → 0
Char. 147: 1 → 0
Char. 148: 1 → 0
Char. 159: 1 → 0
Char. 181: 0 → 1
Char. 185: 0 → 1
Char. 245: 0 → 1
Char. 272: 1 → 2
Char. 278: 0 → 3

***Lanthanosuchus watsoni*:**
Char. 25: 1 → 0
Char. 51: 0 → 1
Char. 86: 0 → 1
Char. 95: 0 → 1

Char. 98: 1 → 0
Char. 110: 0 → 1
Char. 113: 0 → 1
Char. 114: 0 → 1
Char. 119: 1 → 0
Char. 131: 0 → 1
Char. 137: 0 → 1
Char. 138: 0 → 1
Char. 140: 0 → 2
Char. 144: 1 → 0
Char. 154: 1 → 2
Char. 192: 0 → 1

***Macroleter poezicus*:**
Char. 0: 0 → 1
Char. 52: 0 → 1
Char. 66: 1 → 2
Char. 75: 0 → 1
Char. 84: 1 → 0
Char. 87: 0 → 1
Char. 110: 0 → 1
Char. 140: 0 → 1
Char. 146: 1 → 0
Char. 169: 1 → 0
Char. 235: 0 → 1

***Mesosaurus* spp.:**
Char. 0: 0 → 1
Char. 2: 0 → 1
Char. 5: 1 → 0
Char. 6: 0 → 1
Char. 8: 0 → 1
Char. 9: 0 → 1
Char. 13: 0 → 1
Char. 19: 0 → 1
Char. 23: 0 → 1
Char. 26: 0 → 1
Char. 29: 1 → 0
Char. 33: 0 → 1
Char. 38: 1 → 0
Char. 41: 0 → 1
Char. 48: 0 → 1
Char. 50: 1 → 0
Char. 67: 0 → 1
Char. 83: 0 → 1
Char. 84: 1 → 0
Char. 85: 1 → 0
Char. 94: 0 → 1
Char. 107: 0 → 1
Char. 109: 0 → 1
Char. 111: 0 → 1
Char. 115: 0 → 1
Char. 146: 1 → 0
Char. 147: 1 → 0
Char. 148: 0 → 1
Char. 149: 1 → 0
Char. 163: 1 → 0
Char. 164: 0 → 1
Char. 166: 1 → 0
Char. 167: 0 → 1
Char. 176: 0 → 1
Char. 183: 1 → 0
Char. 184: 0 → 1
Char. 199: 0 → 1

Char. 202: 0 → 1
Char. 206: 0 → 1
Char. 207: 0 → 1
Char. 209: 0 → 1
Char. 217: 0 → 1
Char. 219: 0 → 1
Char. 220: 0 → 1
Char. 231: 0 → 1
Char. 260: 2 → 0
Char. 272: 2 → 0
Char. 278: 3 → 0

***Microleter mckinzieorum*:**
Char. 0: 0 → 1
Char. 25: 1 → 0
Char. 36: 0 → 1
Char. 39: 0 → 1
Char. 51: 0 → 2
Char. 56: 0 → 1
Char. 70: 0 → 1
Char. 94: 0 → 1
Char. 106: 0 → 1
Char. 166: 1 → 0
Char. 276: 0 → 1

**Millerettidae:**
Char. 5: 1 → 0
Char. 24: 0 → 1
Char. 25: 1 → 0
Char. 44: 1 → 0
Char. 56: 0 → 1
Char. 57: 1 → 0
Char. 66: 0 → 2
Char. 78: 1 → 0
Char. 80: 1 → 0
Char. 84: 1 → 2
Char. 96: 1 → 0
Char. 117: 0 → 1
Char. 124: 0 → 1
Char. 127: 0 → 1
Char. 135: 0 → 1
Char. 145: 0 → 1
Char. 147: 1 → 0
Char. 163: 1 → 0
Char. 166: 1 → 0
Char. 202: 0 → 1
Char. 211: 0 → 1
Char. 230: 0 → 1
Char. 240: 0 → 1
Char. 248: 0 → 1
Char. 252: 0 → 1
Char. 253: 0 → 1

***Nycteroleter ineptus*:**
Char. 278: 0 → 3

***Nyctiphruretus acudens*:**
Char. 0: 0 → 1
Char. 21: 0 → 1
Char. 33: 1 → 2
Char. 41: 0 → 1
Char. 66: 1 → 2
Char. 81: 1 → 0
Char. 83: 0 → 1

Char. 84: 1 → 2
Char. 94: 0 → 1
Char. 166: 1 → 0
Char. 167: 0 → 1
Char. 215: 1 → 0
Char. 224: 0 → 1
Char. 226: 1 → 0
Char. 266: 0 → 1
Char. 272: 2 → 1
Char. 276: 0 → 1

***Orovenator mayorum*:**
Char. 8: 0 → 1
Char. 24: 0 → 1
Char. 36: 0 → 1
Char. 160: 0 → 1
Char. 165: 0 → 1

***Owenetta* spp.:**
Char. 169: 1 → 0

***Paleothyris acadiana*:**
Char. 38: 1 → 0
Char. 50: 1 → 0
Char. 66: 1 → 2
Char. 102: 0 → 1
Char. 146: 1 → 0
Char. 237: 0 → 1
Char. 239: 0 → 1
Char. 240: 0 → 1

***Placodus* spp.:**
Char. 0: 1 → 2
Char. 9: 0 → 1
Char. 12: 0 → 1
Char. 13: 0 → 1
Char. 19: 0 → 1
Char. 26: 0 → 1
Char. 31: 0 → 1
Char. 46: 1 → 0
Char. 57: 0 → 1
Char. 78: 1 → 0
Char. 93: 1 → 0
Char. 109: 1 → 0
Char. 140: 1 → 0
Char. 155: 0 → 1
Char. 163: 1 → 0
Char. 164: 0 → 1

***Procolophon* spp.:**
Char. 41: 0 → 1
Char. 69: 0 → 1
Char. 79: 0 → 1
Char. 83: 0 → 1
Char. 88: 0 → 1
Char. 117: 1 → 0
Char. 149: 1 → 0
Char. 237: 0 → 1
Char. 238: 0 → 1
Char. 272: 2 → 1
Char. 278: 3 → 0

***Prolacerta broomi*:**
Char. 58: 1 → 0
Char. 66: 1 → 0

Char. 67: 1 → 0
Char. 80: 1 → 0
Char. 123: 0 → 1
Char. 139: 1 → 0
Char. 147: 1 → 0
Char. 192: 1 → 0
Char. 203: 1 → 2

***Rhipaeosaurus* spp.:**
Char. 172: 0 → 1
Char. 186: 1 → 0
Char. 277: 0 → 1

**Rhynchocephalia:**
Char. 0: 1 → 2
Char. 23: 1 → 0
Char. 24: 0 → 1
Char. 75: 1 → 0
Char. 77: 0 → 1
Char. 88: 1 → 0
Char. 94: 1 → 0
Char. 104: 0 → 1
Char. 117: 12 → 0
Char. 139: 1 → 0
Char. 167: 1 → 0
Char. 205: 1 → 0

**Rhynchosauria:**
Char. 0: 1 → 0
Char. 7: 0 → 1
Char. 9: 0 → 1
Char. 26: 0 → 1
Char. 63: 1 → 0
Char. 68: 0 → 1
Char. 99: 1 → 0
Char. 123: 0 → 1
Char. 150: 1 → 0
Char. 160: 0 → 1
Char. 161: 0 → 1
Char. 171: 0 → 2
Char. 182: 1 → 0
Char. 241: 0 → 1

***Scutosaurus* spp.:**
Char. 175: 0 → 1
Char. 218: 0 → 1
Char. 243: 0 → 2
Char. 244: 0 → 1
Char. 251: 0 → 1

***Sinosaurosphargis yunguiensis*:**
Char. 8: 0 → 1
Char. 30: 0 → 1
Char. 53: 0 → 1
Char. 82: 0 → 1
Char. 89: 1 → 0
Char. 127: 1 → 0
Char. 150: 1 → 0
Char. 154: 0 → 2
Char. 167: 1 → 0
Char. 253: 0 → 1
Char. 255: 0 → 1

**Squamata:**
Char. 26: 0 → 1
Char. 45: 0 → 1
Char. 79: 0 → 2
Char. 80: 1 → 0
Char. 81: 1 → 0
Char. 82: 0 → 1
Char. 92: 1 → 0
Char. 109: 1 → 0
Char. 122: 0 → 1
Char. 160: 0 → 1
Char. 200: 0 → 1
Char. 245: 0 → 1

***Trilophosaurus buettneri*:**
Char. 5: 1 → 0
Char. 11: 0 → 1
Char. 55: 1 → 0
Char. 93: 1 → 0
Char. 104: 0 → 1
Char. 113: 0 → 1
Char. 122: 0 → 1
Char. 136: 1 → 0
Char. 144: 1 → 0
Char. 154: 0 → 1
Char. 157: 0 → 1
Char. 159: 1 → 0
Char. 177: 0 → 12
Char. 194: 0 → 1
Char. 203: 1 → 2
Char. 207: 1 → 0
Char. 208: 1 → 0
Char. 272: 1 → 0

***Youngina capensis*:**
Char. 5: 1 → 0
Char. 56: 0 → 1
Char. 75: 0 → 1
Char. 92: 1 → 0
Char. 163: 1 → 0
Char. 170: 0 → 1
Char. 211: 0 → 1
Char. 231: 0 → 1
Char. 239: 0 → 1

**Node 50:**
Char. 0: 2 → 0
Char. 5: 1 → 0
Char. 33: 2 → 0
Char. 46: 0 → 1
Char. 65: 1 → 0
Char. 127: 1 → 0
Char. 145: 1 → 0
Char. 147: 1 → 0
Char. 149: 1 → 0
Char. 174: 1 → 0
Char. 176: 1 → 0
Char. 180: 1 → 0
Char. 184: 1 → 0
Char. 187: 1 → 0
Char. 188: 1 → 0
Char. 189: 1 → 0
Char. 191: 1 → 0
Char. 192: 1 → 0

Char. 194: 2 → 0
Char. 195: 2 → 0
Char. 198: 1 → 0
Char. 205: 1 → 0
Char. 208: 1 → 0
Char. 210: 1 → 0
Char. 220: 1 → 0
Char. 221: 1 → 0
Char. 222: 1 → 0
Char. 223: 1 → 0
Char. 224: 1 → 0
Char. 226: 1 → 0
Char. 227: 1 → 0
Char. 231: 1 → 0
Char. 232: 1 → 0
Char. 237: 1 → 0
Char. 241: 1 → 0
Char. 246: 2 → 0
Char. 247: 1 → 0
Char. 251: 1 → 0
Char. 253: 2 → 0
Char. 254: 1 → 0
Char. 255: 1 → 0
Char. 256: 1 → 0
Char. 258: 1 → 0
Char. 259: 1 → 0
Char. 267: 1 → 0
Char. 268: 1 → 0
Char. 269: 1 → 0
Char. 270: 1 → 0
Char. 272: 1 → 2

**Node 51:**
Char. 27: 1 → 0
Char. 58: 0 → 1
Char. 61: 0 → 1
Char. 66: 0 → 1
Char. 69: 0 → 1
Char. 75: 0 → 1
Char. 84: 0 → 1
Char. 107: 0 → 1
Char. 123: 1 → 0
Char. 140: 0 → 1
Char. 147: 0 → 1
Char. 150: 0 → 1
Char. 167: 0 → 1
Char. 184: 0 → 1
Char. 205: 0 → 1
Char. 208: 0 → 1
Char. 210: 0 → 1
Char. 230: 0 → 1

**Node 52:**
Char. 70: 0 → 1
Char. 73: 0 → 1
Char. 126: 0 → 1
Char. 131: 0 → 1
Char. 148: 0 → 1
Char. 182: 0 → 1
Char. 190: 0 → 1
Char. 272: 2 → 1

**Node 53:**
Char. 33: 0 → 1
Char. 92: 0 → 1
Char. 135: 0 → 1

**Node 54:**
Char. 20: 0 → 1
Char. 62: 0 → 1

**Node 55:**
Char. 0: 0 → 1
Char. 27: 0 → 1
Char. 29: 1 → 0
Char. 38: 1 → 0
Char. 40: 2 → 0
Char. 57: 1 → 0
Char. 59: 0 → 1
Char. 60: 0 → 1
Char. 67: 0 → 1
Char. 72: 1 → 2
Char. 84: 1 → 0
Char. 89: 0 → 1
Char. 111: 0 → 1
Char. 112: 0 → 1
Char. 120: 0 → 1
Char. 147: 1 → 0
Char. 154: 1 → 0
Char. 192: 0 → 1
Char. 193: 0 → 1
Char. 222: 0 → 1
Char. 237: 0 → 1
Char. 240: 0 → 1
Char. 266: 0 → 1
Char. 278: 3 → 1

**Node 56:**
Char. 5: 0 → 1
Char. 29: 0 → 1
Char. 40: 0 → 2
Char. 74: 0 → 1
Char. 116: 0 → 1
Char. 119: 0 → 1
Char. 147: 0 → 1
Char. 149: 0 → 1
Char. 163: 0 → 1
Char. 166: 0 → 1
Char. 169: 0 → 1
Char. 201: 0 → 1
Char. 203: 0 → 1
Char. 221: 0 → 1
Char. 235: 0 → 1

**Node 57:**
Char. 72: 0 → 1
Char. 79: 1 → 0
Char. 81: 0 → 1
Char. 93: 0 → 1

**Node 58:**
Char. 78: 0 → 1
Char. 111: 1 → 0
Char. 135: 1 → 0

**Node 59:**
Char. 15: 0 → 1
Char. 20: 0 → 1
Char. 25: 1 → 0
Char. 29: 1 → 0
Char. 33: 0 → 2
Char. 55: 1 → 0
Char. 89: 0 → 1
Char. 94: 0 → 1
Char. 127: 0 → 1
Char. 276: 0 → 1
Char. 277: 0 → 1

**Node 60:**
Char. 19: 0 → 1
Char. 92: 1 → 0

**Node 61:**
Char. 4: 0 → 1
Char. 15: 0 → 1
Char. 29: 0 → 1
Char. 35: 1 → 0
Char. 48: 1 → 0
Char. 81: 1 → 0
Char. 210: 1 → 0
Char. 213: 0 → 1
Char. 226: 0 → 2
Char. 228: 0 → 1
Char. 275: 0 → 1

**Node 62:**
Char. 24: 0 → 1
Char. 57: 1 → 0
Char. 79: 0 → 1
Char. 83: 0 → 1
Char. 110: 0 → 1
Char. 132: 1 → 0

**Node 63:**
Char. 73: 0 → 1
Char. 131: 1 → 0
Char. 205: 0 → 1
Char. 239: 0 → 1
Char. 276: 0 → 1

**Node 64:**
Char. 18: 0 → 1
Char. 37: 0 → 1
Char. 48: 0 → 1
Char. 107: 0 → 1
Char. 118: 1 → 0
Char. 125: 1 → 0
Char. 126: 0 → 1
Char. 132: 1 → 0
Char. 150: 0 → 1

**Node 65:**
Char. 23: 0 → 1
Char. 71: 1 → 0
Char. 75: 0 → 1
Char. 102: 0 → 1
Char. 103: 0 → 1
Char. 106: 0 → 1
Char. 110: 0 → 1
Char. 155: 0 → 1
Char. 167: 0 → 1
Char. 207: 0 → 1
Char. 214: 0 → 1
Char. 216: 1 → 0
Char. 235: 0 → 2
Char. 241: 0 → 1

**Node 66:**
Char. 20: 0 → 1
Char. 33: 0 → 1
Char. 38: 1 → 2
Char. 39: 0 → 1
Char. 44: 1 → 0
Char. 49: 0 → 1
Char. 51: 0 → 1
Char. 66: 0 → 1
Char. 70: 0 → 1
Char. 80: 1 → 0
Char. 95: 0 → 1
Char. 112: 0 → 1
Char. 117: 0 → 1
Char. 118: 0 → 1
Char. 125: 0 → 1
Char. 131: 0 → 1
Char. 135: 0 → 1
Char. 137: 0 → 1
Char. 148: 0 → 1
Char. 157: 0 → 1
Char. 158: 0 → 1
Char. 183: 1 → 2
Char. 186: 0 → 1
Char. 192: 0 → 1
Char. 194: 0 → 1
Char. 197: 0 → 1
Char. 201: 1 → 0
Char. 211: 0 → 1
Char. 215: 0 → 1
Char. 226: 0 → 1
Char. 235: 1 → 0
Char. 252: 0 → 1

**Node 67:**
Char. 87: 0 → 1
Char. 110: 0 → 1

**Node 68:**
Char. 25: 1 → 0
Char. 76: 1 → 0

**Node 69:**
Char. 79: 0 → 1
Char. 86: 0 → 1
Char. 93: 1 → 0
Char. 133: 0 → 1

**Node 70:**
Char. 48: 0 → 1
Char. 100: 0 → 1
Char. 113: 0 → 1
Char. 132: 1 → 0
Char. 147: 1 → 2
Char. 154: 1 → 0
Char. 207: 0 → 1
Char. 239: 0 → 1
Char. 278: 3 → 0

**Node 71:**
Char. 38: 1 → 2
Char. 39: 0 → 1
Char. 50: 1 → 0
Char. 58: 0 → 1
Char. 59: 0 → 1
Char. 60: 0 → 1
Char. 72: 1 → 2
Char. 83: 0 → 1
Char. 85: 1 → 0
Char. 95: 0 → 1
Char. 104: 0 → 1
Char. 105: 0 → 1
Char. 106: 0 → 2
Char. 107: 0 → 1
Char. 109: 0 → 1
Char. 110: 0 → 1
Char. 146: 1 → 0
Char. 148: 0 → 1
Char. 155: 0 → 2
Char. 183: 1 → 2

**Node 72:**
Char. 0: 0 → 2
Char. 33: 1 → 0
Char. 38: 2 → 1
Char. 39: 1 → 0
Char. 42: 0 → 1
Char. 43: 0 → 1
Char. 46: 1 → 0
Char. 49: 1 → 0
Char. 52: 0 → 1
Char. 83: 0 → 2
Char. 84: 1 → 0
Char. 87: 0 → 1
Char. 93: 1 → 0
Char. 100: 0 → 1
Char. 113: 0 → 1
Char. 143: 0 → 1
Char. 161: 0 → 1
Char. 163: 1 → 0
Char. 172: 0 → 2
Char. 174: 0 → 1
Char. 188: 0 → 1
Char. 189: 0 → 1
Char. 195: 0 → 1
Char. 212: 0 → 1
Char. 221: 1 → 0
Char. 236: 0 → 1
Char. 238: 0 → 2
Char. 242: 0 → 1
Char. 245: 0 → 1
Char. 274: 0 → 1
Char. 275: 1 → 0

**Node 73:**
Char. 5: 1 → 0
Char. 29: 1 → 0
Char. 44: 1 → 0
Char. 59: 0 → 1
Char. 60: 0 → 1
Char. 66: 0 → 1
Char. 67: 0 → 1
Char. 72: 1 → 2
Char. 78: 1 → 0
Char. 84: 1 → 0
Char. 93: 1 → 0

Char. 111: 0 → 1
Char. 123: 1 → 0
Char. 129: 0 → 1
Char. 147: 1 → 0
Char. 154: 1 → 0
Char. 163: 1 → 0
Char. 169: 1 → 0
Char. 170: 0 → 1
Char. 197: 0 → 1
Char. 221: 1 → 0

**Node 74:**
Char. 20: 0 → 1
Char. 47: 0 → 1
Char. 83: 0 → 1
Char. 85: 1 → 0
Char. 107: 0 → 1
Char. 110: 0 → 1
Char. 169: 1 → 0
Char. 170: 0 → 1

**Node 75:**
Char. 2: 0 → 1
Char. 6: 0 → 1
Char. 33: 1 → 2
Char. 98: 1 → 0
Char. 101: 0 → 1
Char. 104: 0 → 1
Char. 113: 0 → 1
Char. 181: 0 → 1
Char. 186: 0 → 1
Char. 193: 1 → 0
Char. 198: 0 → 1
Char. 200: 0 → 1
Char. 220: 0 → 1
Char. 240: 1 → 0
Char. 266: 1 → 0
Char. 267: 1 → 0

**Node 76:**
Char. 17: 0 → 1
Char. 42: 0 → 1
Char. 251: 0 → 1
Char. 264: 0 → 1

**Node 77:**
Char. 33: 1 → 2
Char. 48: 1 → 0
Char. 61: 1 → 2
Char. 68: 0 → 1
Char. 112: 1 → 0
Char. 128: 0 → 1
Char. 138: 0 → 1
Char. 146: 1 → 0
Char. 155: 0 → 1
Char. 158: 0 → 1
Char. 182: 1 → 0
Char. 192: 1 → 2
Char. 226: 0 → 1
Char. 227: 0 → 1
Char. 233: 0 → 1
Char. 235: 1 → 2
Char. 267: 1 → 2

**Node 78:**
Char. 61: 1 → 3
Char. 90: 0 → 1
Char. 91: 0 → 1
Char. 109: 1 → 0
Char. 155: 0 → 1
Char. 209: 1 → 0

## ANALYSIS 10
## (NO *PROGANOCHELYS QUENSTEDTI* AND *EUNOTOSAURUS AFRICANUS*)

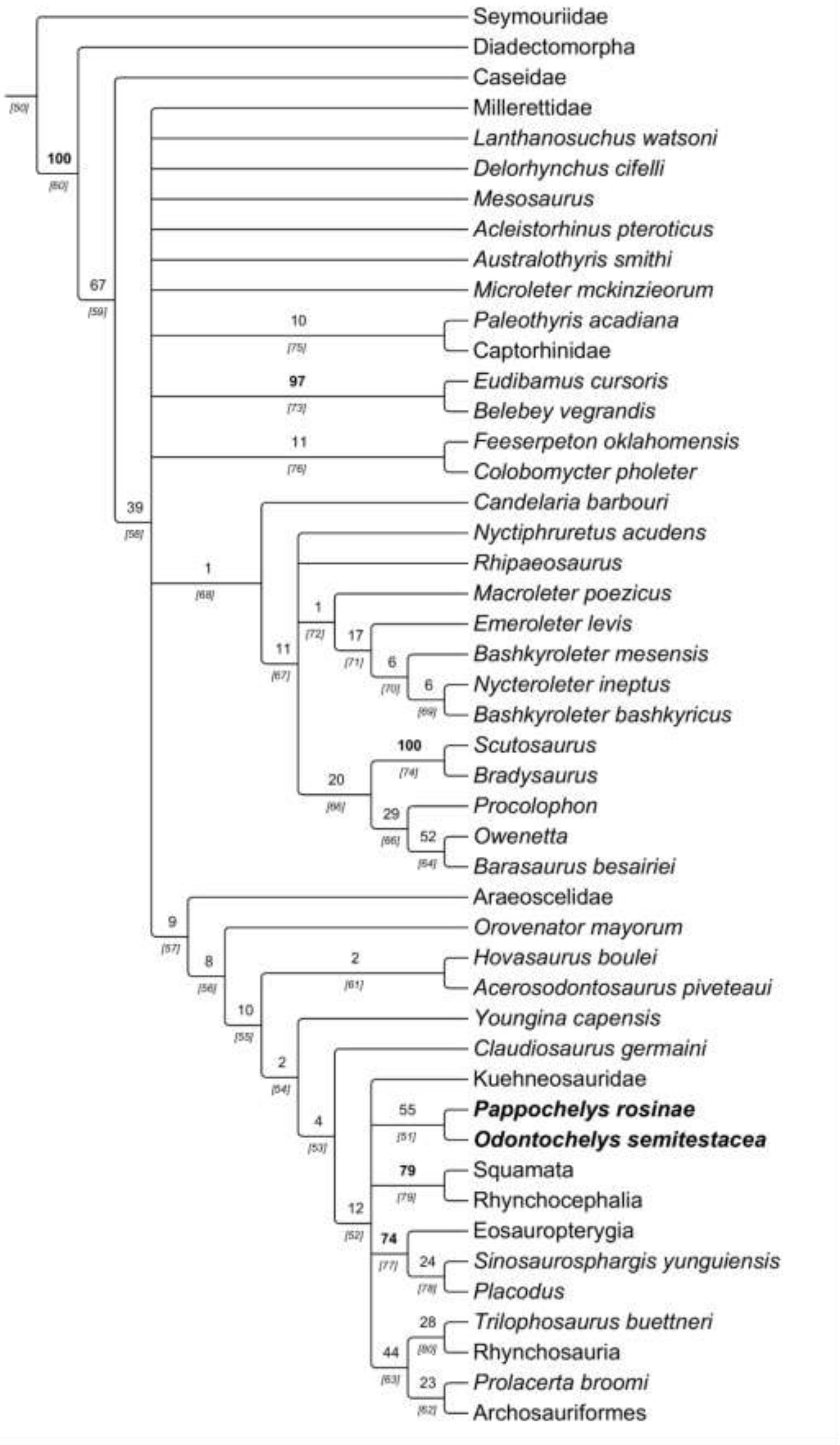

**Seymouriidae:**
Char. 23: 0 → 1
Char. 51: 0 → 2
Char. 54: 1 → 0
Char. 71: 1 → 0
Char. 83: 0 → 1
Char. 85: 1 → 0
Char. 99: 1 → 0
Char. 107: 0 → 1
Char. 140: 0 → 2
Char. 154: 1 → 2
Char. 207: 0 → 1
Char. 225: 1 → 0
Char. 260: 2 → 0

***Pappochelys rosinae*:**
Char. 1: 0 → 1
Char. 5: 1 → 0
Char. 12: 0 → 1
Char. 41: 0 → 1
Char. 43: 1 → 0
Char. 49: 0 → 1
Char. 169: 1 → 0
Char. 176: 1 → 0

***Odontochelys semitestacea*:**
Char. 46: 1 → 0
Char. 50: 0 → 1
Char. 88: 1 → 0
Char. 89: 1 → 0
Char. 93: 1 → 0
Char. 126: 1 → 0
Char. 195: 0 → 2
Char. 198: 0 → 1
Char. 240: 1 → 0
Char. 246: 1 → 2
Char. 259: 0 → 1
Char. 265: 1 → 0
Char. 270: 0 → 1

***Acerosodontosaurus piveteaui*:**
Char. 81: 1 → 0
Char. 208: 0 → 1
Char. 224: 1 → 0

***Acleistorhinus pteroticus*:**
Char. 20: 0 → 1
Char. 21: 0 → 1
Char. 30: 0 → 1
Char. 33: 0 → 1
Char. 47: 0 → 1
Char. 48: 0 → 1
Char. 55: 1 → 0
Char. 56: 0 → 1
Char. 64: 0 → 1
Char. 70: 0 → 1
Char. 79: 0 → 1
Char. 95: 0 → 1
Char. 110: 0 → 1
Char. 113: 0 → 1
Char. 114: 0 → 1
Char. 131: 0 → 1
Char. 137: 0 → 1
Char. 140: 0 → 2
Char. 146: 1 → 0
Char. 159: 0 → 1
Char. 169: 1 → 0
Char. 170: 0 → 1
Char. 278: 3 → 1

**Araeoscelidae:**
Char. 5: 1 → 0
Char. 28: 0 → 1
Char. 106: 0 → 1
Char. 116: 1 → 0
Char. 166: 1 → 0
Char. 169: 1 → 0
Char. 170: 0 → 1
Char. 197: 0 → 1

**Archosauriformes:**
Char. 32: 0 → 1
Char. 94: 1 → 0
Char. 112: 1 → 0
Char. 152: 0 → 1
Char. 154: 0 → 2
Char. 166: 1 → 0
Char. 171: 0 → 1
Char. 185: 0 → 1
Char. 204: 0 → 1
Char. 218: 0 → 3
Char. 242: 0 → 1

***Australothyris smithi*:**
Char. 23: 0 → 1
Char. 24: 0 → 1
Char. 34: 0 → 1
Char. 55: 1 → 0
Char. 57: 1 → 0
Char. 71: 1 → 0
Char. 79: 0 → 1
Char. 85: 1 → 0
Char. 98: 1 → 0
Char. 100: 0 → 1
Char. 103: 0 → 1
Char. 110: 0 → 1
Char. 112: 0 → 1
Char. 123: 1 → 0
Char. 129: 0 → 1
Char. 131: 0 → 1
Char. 132: 1 → 0
Char. 144: 1 → 0
Char. 149: 1 → 0
Char. 150: 0 → 1

***Barasaurus besairiei*:**
Char. 33: 1 → 0
Char. 75: 1 → 0
Char. 216: 0 → 1

***Bashkyroleter bashkyricus*:**
Char. 275: 1 → 0

***Bashkyroleter mesensis*:**
Char. 169: 1 → 0

***Belebey vegrandis*:**
Char. 154: 1 → 0

***Bradysaurus* spp.:**
Char. 19: 0 → 1
Char. 73: 0 → 1
Char. 79: 0 → 1
Char. 135: 1 → 0
Char. 249: 0 → 1

***Candelaria barbouri*:**
Char. 1: 0 → 1
Char. 5: 1 → 0
Char. 8: 0 → 1
Char. 15: 0 → 1
Char. 23: 0 → 1
Char. 25: 1 → 0
Char. 29: 1 → 0
Char. 50: 1 → 0
Char. 55: 1 → 0
Char. 57: 1 → 0
Char. 67: 0 → 1
Char. 79: 0 → 2
Char. 89: 0 → 1
Char. 127: 0 → 1
Char. 154: 1 → 2
Char. 169: 1 → 0
Char. 277: 0 → 1

**Captorhinidae:**
Char. 3: 0 → 1
Char. 23: 0 → 1
Char. 25: 1 → 0
Char. 26: 0 → 1
Char. 73: 0 → 1
Char. 75: 0 → 1
Char. 108: 1 → 0
Char. 183: 1 → 0
Char. 201: 1 → 0
Char. 203: 1 → 2
Char. 216: 1 → 0

**Caseidae:**
Char. 24: 0 → 1
Char. 25: 1 → 0
Char. 36: 0 → 1
Char. 38: 1 → 0
Char. 46: 1 → 0
Char. 50: 1 → 0
Char. 56: 0 → 1
Char. 85: 1 → 0
Char. 96: 1 → 0
Char. 98: 1 → 0
Char. 170: 0 → 1
Char. 194: 0 → 1
Char. 273: 0 → 1
Char. 274: 0 → 1
Char. 278: 3 → 2

***Claudiosaurus germaini*:**
Char. 24: 0 → 1
Char. 34: 0 → 1
Char. 36: 0 → 1
Char. 43: 1 → 0
Char. 64: 0 → 1
Char. 105: 0 → 1
Char. 106: 0 → 1
Char. 130: 0 → 1
Char. 144: 1 → 0
Char. 166: 1 → 0
Char. 187: 1 → 0
Char. 199: 0 → 1
Char. 203: 1 → 2
Char. 204: 0 → 1
Char. 220: 0 → 1
Char. 222: 1 → 0
Char. 224: 1 → 0

***Colobomycter pholeter*:**
Char. 21: 0 → 1
Char. 25: 1 → 0
Char. 84: 1 → 0
Char. 154: 1 → 0
Char. 167: 0 → 1
Char. 267: 0 → 1

***Delorhynchus cifelli*:**
Char. 18: 0 → 1
Char. 20: 0 → 1
Char. 21: 0 → 1
Char. 24: 0 → 1
Char. 26: 0 → 1
Char. 28: 0 → 1
Char. 33: 0 → 2
Char. 39: 0 → 1
Char. 48: 0 → 1
Char. 52: 0 → 1
Char. 100: 0 → 1
Char. 111: 0 → 1
Char. 116: 1 → 0
Char. 117: 0 → 1
Char. 131: 0 → 1
Char. 156: 1 → 0
Char. 159: 0 → 1
Char. 167: 0 → 1
Char. 180: 0 → 1
Char. 189: 0 → 1
Char. 191: 0 → 1
Char. 204: 0 → 2
Char. 267: 0 → 1

**Diadectomorpha:**
Char. 0: 0 → 1
Char. 70: 0 → 1
Char. 75: 0 → 1
Char. 122: 0 → 1
Char. 123: 1 → 0
Char. 146: 1 → 0
Char. 275: 1 → 0
Char. 278: 3 → 0

***Emeroleter levis*:**
Char. 0: 0 → 2
Char. 51: 1 → 0

**Eosauropterygia:**
Char. 159: 1 → 0
Char. 166: 1 → 0
Char. 168: 0 → 1
Char. 174: 0 → 1
Char. 178: 0 → 1
Char. 194: 0 → 2
Char. 272: 1 → 0

***Eudibamus cursoris*:**
Char. 154: 1 → 2

***Feeserpeton oklahomensis*:**
Char. 51: 0 → 1
Char. 70: 0 → 1
Char. 157: 0 → 1
Char. 158: 0 → 1

***Hovasaurus boulei*:**
Char. 41: 0 → 1
Char. 77: 0 → 2
Char. 79: 0 → 1
Char. 93: 1 → 0
Char. 204: 0 → 2
Char. 278: 1 → 3

**Kuehneosauridae:**
Char. 7: 0 → 1
Char. 24: 0 → 1
Char. 26: 0 → 1
Char. 27: 0 → 1
Char. 34: 0 → 1
Char. 36: 0 → 1
Char. 43: 1 → 0
Char. 79: 0 → 2
Char. 81: 1 → 0
Char. 82: 0 → 1
Char. 98: 1 → 0
Char. 108: 1 → 0
Char. 113: 0 → 1
Char. 128: 0 → 1
Char. 140: 1 → 0
Char. 147: 1 → 0
Char. 148: 1 → 0
Char. 159: 1 → 0
Char. 168: 0 → 1
Char. 181: 0 → 1
Char. 185: 0 → 1
Char. 245: 0 → 1
Char. 272: 1 → 2
Char. 278: 0 → 3

***Lanthanosuchus watsoni*:**
Char. 25: 1 → 0
Char. 51: 0 → 1
Char. 76: 0 → 1
Char. 86: 0 → 1
Char. 95: 0 → 1
Char. 98: 1 → 0
Char. 110: 0 → 1
Char. 113: 0 → 1
Char. 114: 0 → 1
Char. 131: 0 → 1
Char. 137: 0 → 1
Char. 138: 0 → 1
Char. 140: 0 → 2
Char. 144: 1 → 0
Char. 154: 1 → 2

***Macroleter poezicus*:**
Char. 0: 0 → 1
Char. 52: 0 → 1
Char. 66: 1 → 2
Char. 75: 0 → 1
Char. 84: 1 → 0
Char. 87: 0 → 1
Char. 110: 0 → 1
Char. 140: 0 → 1
Char. 146: 1 → 0
Char. 169: 1 → 0
Char. 235: 0 → 1

***Mesosaurus* spp.:**
Char. 0: 0 → 1
Char. 2: 0 → 1
Char. 5: 1 → 0
Char. 6: 0 → 1
Char. 8: 0 → 1
Char. 9: 0 → 1
Char. 13: 0 → 1
Char. 19: 0 → 1
Char. 23: 0 → 1
Char. 26: 0 → 1
Char. 29: 1 → 0
Char. 33: 0 → 1
Char. 38: 1 → 0
Char. 41: 0 → 1
Char. 48: 0 → 1
Char. 50: 1 → 0
Char. 67: 0 → 1
Char. 76: 0 → 1
Char. 84: 1 → 0
Char. 85: 1 → 0
Char. 94: 0 → 1
Char. 107: 0 → 1
Char. 109: 0 → 1
Char. 111: 0 → 1
Char. 115: 0 → 1
Char. 146: 1 → 0
Char. 148: 0 → 1
Char. 149: 1 → 0
Char. 164: 0 → 1
Char. 166: 1 → 0
Char. 167: 0 → 1
Char. 176: 0 → 1
Char. 183: 1 → 0
Char. 184: 0 → 1
Char. 199: 0 → 1
Char. 202: 0 → 1
Char. 204: 0 → 2
Char. 206: 0 → 1
Char. 207: 0 → 1
Char. 209: 0 → 1
Char. 217: 0 → 1
Char. 219: 0 → 1
Char. 220: 0 → 1
Char. 231: 0 → 1
Char. 260: 2 → 0
Char. 272: 2 → 0
Char. 278: 3 → 0

***Microleter mckinzieorum*:**
Char. 0: 0 → 1
Char. 18: 0 → 1
Char. 24: 0 → 1
Char. 25: 1 → 0
Char. 36: 0 → 1
Char. 39: 0 → 1
Char. 51: 0 → 2
Char. 56: 0 → 1
Char. 57: 1 → 0
Char. 70: 0 → 1
Char. 76: 0 → 1
Char. 79: 0 → 1
Char. 94: 0 → 1
Char. 106: 0 → 1
Char. 110: 0 → 1
Char. 132: 1 → 0
Char. 159: 0 → 1
Char. 166: 1 → 0
Char. 276: 0 → 1
Char. 278: 3 → 1

**Millerettidae:**
Char. 5: 1 → 0
Char. 24: 0 → 1
Char. 25: 1 → 0
Char. 44: 1 → 0
Char. 56: 0 → 1
Char. 57: 1 → 0
Char. 66: 0 → 2
Char. 78: 1 → 0
Char. 80: 1 → 0
Char. 84: 1 → 2
Char. 88: 1 → 0
Char. 96: 1 → 0
Char. 117: 0 → 1
Char. 124: 0 → 1
Char. 127: 0 → 1
Char. 135: 0 → 1
Char. 145: 0 → 1
Char. 166: 1 → 0
Char. 202: 0 → 1
Char. 211: 0 → 1
Char. 230: 0 → 1
Char. 234: 0 → 1
Char. 248: 0 → 1
Char. 252: 0 → 1
Char. 253: 0 → 1

***Nycteroleter ineptus*:**
Char. 278: 0 → 3

***Nyctiphruretus acudens*:**
Char. 0: 0 → 1
Char. 21: 0 → 1
Char. 41: 0 → 1
Char. 66: 1 → 2
Char. 81: 1 → 0
Char. 84: 1 → 2
Char. 166: 1 → 0
Char. 167: 0 → 1
Char. 215: 1 → 0
Char. 224: 0 → 1
Char. 226: 1 → 0
Char. 266: 0 → 1
Char. 272: 2 → 1

***Orovenator mayorum*:**
Char. 8: 0 → 1
Char. 24: 0 → 1
Char. 36: 0 → 1
Char. 160: 0 → 1
Char. 165: 0 → 1

***Owenetta* spp.:**
Char. 169: 1 → 0

***Paleothyris acadiana*:**
Char. 38: 1 → 0
Char. 50: 1 → 0
Char. 66: 1 → 2
Char. 102: 0 → 1
Char. 146: 1 → 0
Char. 180: 0 → 1
Char. 237: 0 → 1
Char. 239: 0 → 1

***Placodus* spp.:**
Char. 0: 1 → 2
Char. 9: 0 → 1
Char. 12: 0 → 1
Char. 13: 0 → 1
Char. 19: 0 → 1
Char. 26: 0 → 1
Char. 31: 0 → 1
Char. 46: 1 → 0
Char. 57: 0 → 1
Char. 78: 1 → 0
Char. 93: 1 → 0
Char. 109: 1 → 0
Char. 140: 1 → 0
Char. 155: 0 → 1
Char. 163: 1 → 0
Char. 164: 0 → 1

***Procolophon* spp.:**
Char. 41: 0 → 1
Char. 69: 0 → 1
Char. 79: 0 → 1
Char. 88: 0 → 1
Char. 117: 1 → 0
Char. 149: 1 → 0
Char. 180: 0 → 1
Char. 204: 0 → 1
Char. 237: 0 → 1
Char. 238: 0 → 1
Char. 272: 2 → 1
Char. 278: 3 → 0

***Prolacerta broomi*:**
Char. 58: 1 → 0
Char. 66: 1 → 0
Char. 67: 1 → 0
Char. 80: 1 → 0
Char. 139: 1 → 0
Char. 147: 1 → 0
Char. 192: 1 → 0
Char. 203: 1 → 2
Char. 224: 1 → 0

***Rhipaeosaurus* spp.:**
Char. 172: 0 → 1
Char. 186: 1 → 0
Char. 277: 0 → 1

**Rhynchocephalia:**
Char. 0: 1 → 2
Char. 23: 1 → 0
Char. 24: 0 → 1
Char. 77: 0 → 1
Char. 88: 1 → 0
Char. 94: 1 → 0
Char. 104: 0 → 1
Char. 117: 12 → 0
Char. 139: 1 → 0
Char. 167: 1 → 0
Char. 205: 1 → 0

**Rhynchosauria:**
Char. 0: 1 → 0
Char. 7: 0 → 1
Char. 9: 0 → 1
Char. 26: 0 → 1
Char. 63: 1 → 0
Char. 68: 0 → 1
Char. 99: 1 → 0
Char. 150: 1 → 0
Char. 160: 0 → 1
Char. 161: 0 → 1
Char. 171: 0 → 2
Char. 241: 0 → 1

***Scutosaurus* spp.:**
Char. 175: 0 → 1
Char. 218: 0 → 1
Char. 243: 0 → 2
Char. 244: 0 → 1
Char. 251: 0 → 1

***Sinosaurosphargis yunguiensis*:**
Char. 8: 0 → 1
Char. 30: 0 → 1
Char. 44: 0 → 1
Char. 53: 0 → 1
Char. 82: 0 → 1
Char. 89: 1 → 0
Char. 127: 1 → 0
Char. 150: 1 → 0
Char. 154: 0 → 2
Char. 167: 1 → 0
Char. 253: 0 → 1
Char. 255: 0 → 1

**Squamata:**
Char. 26: 0 → 1
Char. 45: 0 → 1
Char. 79: 0 → 2
Char. 80: 1 → 0
Char. 81: 1 → 0
Char. 82: 0 → 1
Char. 92: 1 → 0
Char. 106: 0 → 1
Char. 109: 1 → 0
Char. 122: 0 → 1
Char. 160: 0 → 1
Char. 200: 0 → 1
Char. 245: 0 → 1

***Trilophosaurus buettneri*:**
Char. 5: 1 → 0
Char. 11: 0 → 1
Char. 44: 0 → 1
Char. 55: 1 → 0
Char. 93: 1 → 0
Char. 104: 0 → 1
Char. 106: 0 → 1
Char. 113: 0 → 1
Char. 122: 0 → 1
Char. 136: 1 → 0
Char. 144: 1 → 0
Char. 154: 0 → 1
Char. 157: 0 → 1
Char. 159: 1 → 0
Char. 177: 0 → 12
Char. 194: 0 → 1
Char. 203: 1 → 2
Char. 207: 1 → 0
Char. 208: 1 → 0
Char. 224: 1 → 0
Char. 272: 1 → 0

***Youngina capensis*:**
Char. 5: 1 → 0
Char. 170: 0 → 1
Char. 211: 0 → 1

**Node 50:**
Char. 0: 2 → 0
Char. 5: 1 → 0
Char. 15: 1 → 0
Char. 20: 1 → 0
Char. 21: 1 → 0
Char. 24: 1 → 0
Char. 25: 0 → 1
Char. 33: 2 → 0
Char. 42: 1 → 0
Char. 73: 1 → 0
Char. 84: 2 → 01
Char. 119: 1 → 0
Char. 127: 1 → 0
Char. 130: 1 → 0
Char. 134: 2 → 0
Char. 136: 1 → 0
Char. 141: 1 → 0
Char. 145: 1 → 0
Char. 147: 1 → 0
Char. 149: 1 → 0
Char. 151: 1 → 0
Char. 152: 1 → 0
Char. 155: 1 → 0
Char. 158: 1 → 0
Char. 159: 1 → 0
Char. 161: 1 → 0
Char. 163: 1 → 0
Char. 166: 1 → 0
Char. 174: 1 → 0
Char. 180: 1 → 0
Char. 181: 1 → 0
Char. 188: 1 → 0

Char. 196: 1 → 0
Char. 203: 2 → 0
Char. 204: 2 → 0
Char. 217: 1 → 0
Char. 220: 1 → 0
Char. 221: 1 → 0
Char. 231: 1 → 0
Char. 234: 1 → 0
Char. 247: 1 → 0
Char. 248: 1 → 0
Char. 250: 1 → 0
Char. 251: 1 → 0
Char. 253: 2 → 0

**Node 51:**
Char. 61: 1 → 0
Char. 62: 1 → 0
Char. 65: 0 → 1
Char. 193: 1 → 0
Char. 194: 0 → 2
Char. 201: 1 → 0
Char. 219: 1 → 0
Char. 220: 0 → 1
Char. 241: 0 → 1
Char. 246: 0 → 1
Char. 247: 0 → 1
Char. 252: 0 → 1
Char. 254: 0 → 1
Char. 255: 0 → 1
Char. 256: 0 → 1
Char. 268: 0 → 1
Char. 269: 0 → 1

**Node 52:**
Char. 27: 1 → 0
Char. 58: 0 → 1
Char. 61: 0 → 1
Char. 66: 0 → 1
Char. 69: 0 → 1
Char. 84: 0 → 1
Char. 140: 0 → 1
Char. 147: 0 → 1
Char. 150: 0 → 1
Char. 167: 0 → 1
Char. 184: 0 → 1
Char. 205: 0 → 1
Char. 208: 0 → 1
Char. 210: 0 → 1
Char. 223: 0 → 1
Char. 230: 0 → 1
Char. 232: 0 → 1

**Node 53:**
Char. 70: 0 → 1
Char. 73: 0 → 1
Char. 126: 0 → 1
Char. 131: 0 → 1
Char. 148: 0 → 1
Char. 272: 2 → 1

**Node 54:**
Char. 44: 1 → 0
Char. 134: 0 → 1
Char. 214: 0 → 1
Char. 219: 0 → 1
Char. 267: 0 → 1
Char. 278: 1 → 0

**Node 55:**
Char. 135: 0 → 1

**Node 56:**
Char. 20: 0 → 1
Char. 62: 0 → 1

**Node 57:**
Char. 0: 0 → 1
Char. 27: 0 → 1
Char. 29: 1 → 0
Char. 38: 1 → 0
Char. 40: 2 → 0
Char. 57: 1 → 0
Char. 59: 0 → 1
Char. 67: 0 → 1
Char. 72: 1 → 2
Char. 84: 1 → 0
Char. 89: 0 → 1
Char. 111: 0 → 1
Char. 112: 0 → 1
Char. 120: 0 → 1
Char. 180: 0 → 1
Char. 193: 0 → 1
Char. 222: 0 → 1
Char. 224: 0 → 1
Char. 234: 0 → 1
Char. 237: 0 → 1
Char. 266: 0 → 1
Char. 278: 3 → 1

**Node 58:**
Char. 5: 0 → 1
Char. 29: 0 → 1
Char. 40: 0 → 2
Char. 74: 0 → 1
Char. 88: 0 → 1
Char. 116: 0 → 1
Char. 149: 0 → 1
Char. 166: 0 → 1
Char. 169: 0 → 1
Char. 201: 0 → 1
Char. 203: 0 → 1
Char. 235: 0 → 1
Char. 276: 1 → 0

**Node 59:**
Char. 81: 0 → 1
Char. 93: 0 → 1
Char. 97: 0 → 1

**Node 60:**
Char. 78: 0 → 1
Char. 111: 1 → 0
Char. 135: 1 → 0

**Node 61:**
Char. 78: 1 → 0
Char. 206: 0 → 2

**Node 62:**
Char. 19: 0 → 1
Char. 92: 1 → 0

**Node 63:**
Char. 4: 0 → 1
Char. 15: 0 → 1
Char. 29: 0 → 1
Char. 35: 1 → 0
Char. 48: 1 → 0
Char. 81: 1 → 0
Char. 210: 1 → 0
Char. 213: 0 → 1
Char. 226: 0 → 2
Char. 228: 0 → 1
Char. 275: 0 → 1

**Node 64:**
Char. 73: 0 → 1
Char. 131: 1 → 0
Char. 205: 0 → 1
Char. 239: 0 → 1

**Node 65:**
Char. 18: 0 → 1
Char. 37: 0 → 1
Char. 48: 0 → 1
Char. 107: 0 → 1
Char. 118: 1 → 0
Char. 125: 1 → 0
Char. 150: 0 → 1

**Node 66:**
Char. 23: 0 → 1
Char. 71: 1 → 0
Char. 75: 0 → 1
Char. 102: 0 → 1
Char. 103: 0 → 1
Char. 106: 0 → 1
Char. 110: 0 → 1
Char. 155: 0 → 1
Char. 167: 0 → 1
Char. 207: 0 → 1
Char. 214: 0 → 1
Char. 216: 1 → 0
Char. 235: 0 → 2
Char. 241: 0 → 1

**Node 67:**
Char. 66: 0 → 1
Char. 159: 0 → 1

**Node 68:**
Char. 20: 0 → 1
Char. 33: 0 → 12
Char. 49: 0 → 1
Char. 76: 0 → 1
Char. 88: 1 → 0
Char. 95: 0 → 1

**Node 69:**
Char. 87: 0 → 1
Char. 110: 0 → 1

**Node 70:**
Char. 25: 1 → 0
Char. 76: 1 → 0

**Node 71:**
Char. 79: 0 → 1
Char. 86: 0 → 1
Char. 93: 1 → 0
Char. 133: 0 → 1

**Node 72:**
Char. 48: 0 → 1
Char. 100: 0 → 1
Char. 113: 0 → 1
Char. 147: 1 → 2
Char. 154: 1 → 0
Char. 207: 0 → 1
Char. 239: 0 → 1
Char. 278: 3 → 0

**Node 73:**
Char. 38: 1 → 2
Char. 39: 0 → 1
Char. 50: 1 → 0
Char. 58: 0 → 1
Char. 59: 0 → 1
Char. 60: 0 → 1
Char. 72: 1 → 2
Char. 85: 1 → 0
Char. 88: 1 → 0
Char. 95: 0 → 1
Char. 104: 0 → 1
Char. 105: 0 → 1
Char. 106: 0 → 2
Char. 107: 0 → 1
Char. 109: 0 → 1
Char. 110: 0 → 1
Char. 146: 1 → 0
Char. 148: 0 → 1
Char. 155: 0 → 2
Char. 183: 1 → 2

**Node 74:**
Char. 0: 0 → 2
Char. 33: 1 → 0
Char. 38: 2 → 1
Char. 39: 1 → 0
Char. 42: 0 → 1
Char. 43: 0 → 1
Char. 46: 1 → 0
Char. 49: 1 → 0
Char. 52: 0 → 1
Char. 83: 01 → 2
Char. 84: 1 → 0
Char. 87: 0 → 1
Char. 93: 1 → 0
Char. 100: 0 → 1
Char. 113: 0 → 1
Char. 143: 0 → 1
Char. 161: 0 → 1
Char. 163: 1 → 0
Char. 172: 0 → 2
Char. 174: 0 → 1
Char. 188: 0 → 1
Char. 189: 0 → 1
Char. 195: 0 → 1
Char. 204: 0 → 2
Char. 212: 0 → 1
Char. 236: 0 → 1
Char. 238: 0 → 2
Char. 242: 0 → 1
Char. 245: 0 → 1
Char. 274: 0 → 1
Char. 275: 1 → 0

**Node 75:**
Char. 5: 1 → 0
Char. 29: 1 → 0
Char. 44: 1 → 0
Char. 59: 0 → 1
Char. 60: 0 → 1
Char. 66: 0 → 1
Char. 67: 0 → 1
Char. 72: 1 → 2
Char. 78: 1 → 0
Char. 84: 1 → 0
Char. 88: 1 → 0
Char. 93: 1 → 0
Char. 111: 0 → 1
Char. 123: 1 → 0
Char. 129: 0 → 1
Char. 154: 1 → 0
Char. 169: 1 → 0
Char. 170: 0 → 1
Char. 197: 0 → 1

**Node 76:**
Char. 20: 0 → 1
Char. 47: 0 → 1
Char. 85: 1 → 0
Char. 107: 0 → 1
Char. 110: 0 → 1
Char. 169: 1 → 0
Char. 170: 0 → 1

**Node 77:**
Char. 2: 0 → 1
Char. 6: 0 → 1
Char. 98: 1 → 0
Char. 101: 0 → 1
Char. 104: 0 → 1
Char. 106: 0 → 1
Char. 113: 0 → 1
Char. 181: 0 → 1
Char. 186: 0 → 1
Char. 193: 1 → 0
Char. 198: 0 → 1
Char. 200: 0 → 1
Char. 220: 0 → 1
Char. 224: 1 → 0
Char. 229: 0 → 1
Char. 231: 1 → 0
Char. 232: 1 → 0
Char. 240: 1 → 0
Char. 266: 1 → 0
Char. 267: 1 → 0

**Node 78:**
Char. 17: 0 → 1
Char. 42: 0 → 1
Char. 251: 0 → 1
Char. 264: 0 → 1

**Node 79:**
Char. 41: 0 → 1
Char. 44: 0 → 1
Char. 48: 1 → 0
Char. 61: 1 → 2
Char. 68: 0 → 1
Char. 112: 1 → 0
Char. 128: 0 → 1
Char. 138: 0 → 1
Char. 146: 1 → 0
Char. 155: 0 → 1
Char. 158: 0 → 1
Char. 192: 1 → 2
Char. 226: 0 → 1
Char. 227: 0 → 1
Char. 233: 0 → 1
Char. 267: 1 → 2

**Node 80:**
Char. 61: 1 → 3
Char. 90: 0 → 1
Char. 91: 0 → 1
Char. 109: 1 → 0
Char. 155: 0 → 1
Char. 209: 1 → 0

**ANALYSIS 11**
**(NO *CANDELARIA BARBOURI* AND *PAPPOCHELYS ROSINAE*)**

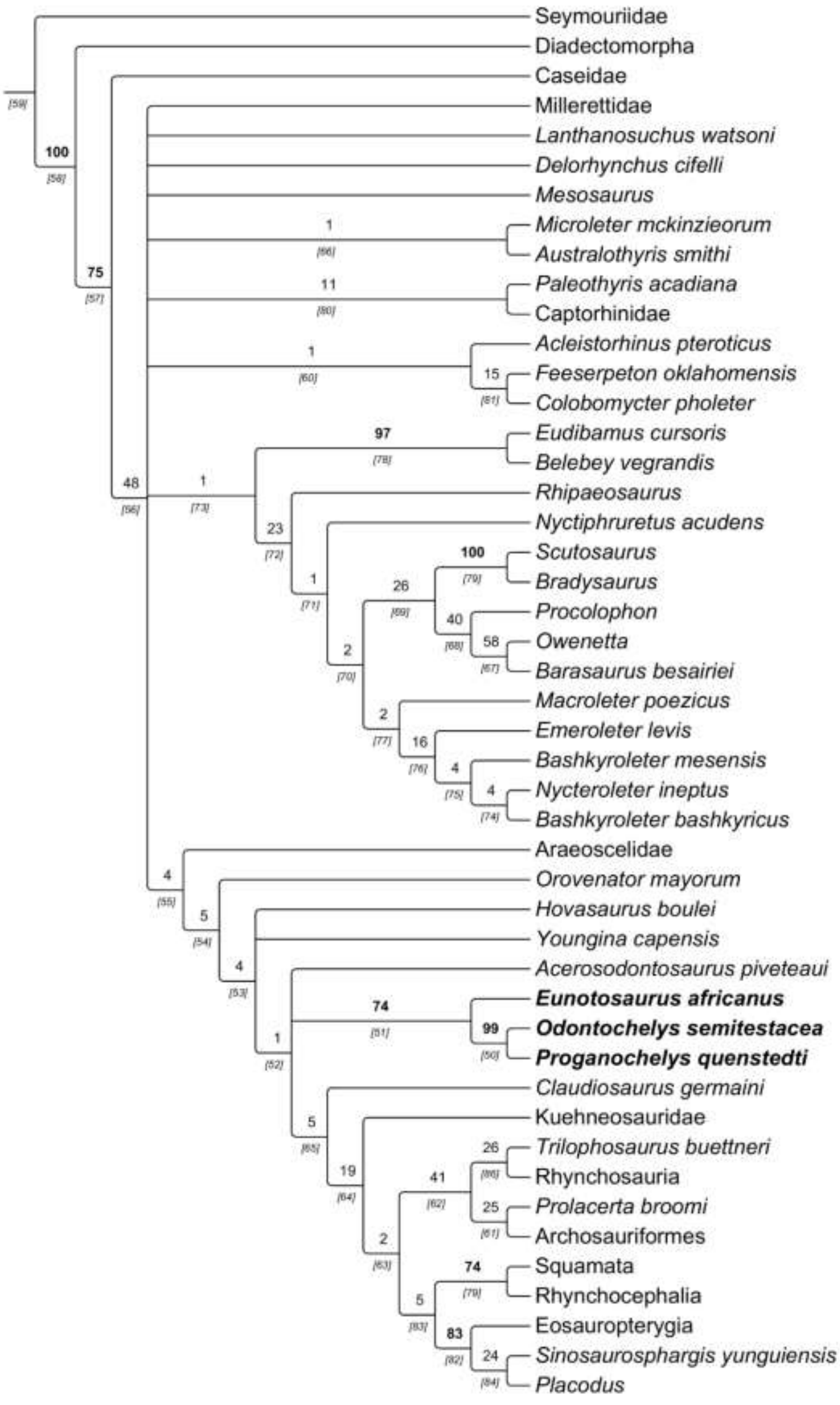

***Proganochelys quenstedti*:**
Char. 8: 0 → 1
Char. 11: 0 → 1
Char. 43: 1 → 0
Char. 106: 0 → 1
Char. 108: 1 → 0
Char. 126: 0 → 1
Char. 128: 0 → 1
Char. 175: 0 → 1
Char. 207: 1 → 0
Char. 209: 1 → 0
Char. 244: 0 → 1
Char. 262: 0 → 1

**Seymouriidae:**
Char. 54: 1 → 0
Char. 85: 1 → 0

***Odontochelys semitestacea*:**
Char. 206: 0 → 2

***Eunotosaurus africanus*:**
Char. 19: 0 → 1
Char. 55: 1 → 0
Char. 59: 1 → 0
Char. 60: 1 → 0
Char. 67: 1 → 0
Char. 72: 2 → 1
Char. 76: 0 → 1
Char. 97: 1 → 0
Char. 103: 0 → 1
Char. 153: 1 → 0
Char. 191: 1 → 0
Char. 192: 1 → 0
Char. 211: 0 → 1
Char. 222: 1 → 0
Char. 237: 1 → 0
Char. 249: 0 → 2
Char. 263: 0 → 1
Char. 273: 0 → 1
Char. 274: 0 → 1
Char. 275: 0 → 1
Char. 277: 0 → 1
Char. 278: 0 → 3

***Acerosodontosaurus piveteaui*:**
Char. 127: 1 → 0
Char. 128: 0 → 1
Char. 206: 0 → 2
Char. 278: 0 → 1

***Acleistorhinus pteroticus*:**
Char. 30: 0 → 1
Char. 33: 0 → 1
Char. 55: 1 → 0
Char. 56: 0 → 1
Char. 64: 0 → 1
Char. 95: 0 → 1
Char. 113: 0 → 1
Char. 114: 0 → 1
Char. 146: 1 → 0
Char. 278: 3 → 1

**Araeoscelidae:**
Char. 23: 0 → 1
Char. 28: 0 → 1
Char. 106: 0 → 1
Char. 116: 1 → 0
Char. 117: 0 → 2
Char. 169: 1 → 0
Char. 170: 0 → 1
Char. 197: 0 → 1
Char. 239: 0 → 1

**Archosauriformes:**
Char. 32: 0 → 1
Char. 94: 1 → 0
Char. 112: 1 → 0
Char. 152: 0 → 1
Char. 154: 0 → 2
Char. 166: 1 → 0
Char. 171: 0 → 1
Char. 185: 0 → 1
Char. 204: 0 → 1
Char. 218: 0 → 3
Char. 224: 0 → 1
Char. 242: 0 → 1

***Australothyris smithi*:**
Char. 23: 0 → 1
Char. 34: 0 → 1
Char. 55: 1 → 0
Char. 71: 1 → 0
Char. 85: 1 → 0
Char. 112: 0 → 1
Char. 129: 0 → 1
Char. 131: 0 → 1
Char. 166: 0 → 1

***Barasaurus besairiei*:**
Char. 33: 1 → 0
Char. 75: 1 → 0
Char. 216: 0 → 1

***Bashkyroleter bashkyricus*:**
Char. 275: 1 → 0

***Bashkyroleter mesensis*:**
Char. 169: 1 → 0

***Belebey vegrandis*:**
Char. 154: 1 → 0

***Bradysaurus* spp.:**
Char. 19: 0 → 1
Char. 73: 0 → 1
Char. 79: 0 → 1
Char. 135: 1 → 0
Char. 249: 0 → 1

**Captorhinidae:**
Char. 3: 0 → 1
Char. 23: 0 → 1
Char. 25: 1 → 0
Char. 26: 0 → 1
Char. 73: 0 → 1
Char. 75: 0 → 1
Char. 83: 0 → 1
Char. 108: 1 → 0
Char. 183: 1 → 0
Char. 201: 1 → 0
Char. 203: 1 → 2
Char. 216: 1 → 0

**Caseidae:**
Char. 24: 0 → 1
Char. 25: 1 → 0
Char. 36: 0 → 1
Char. 38: 1 → 0
Char. 46: 1 → 0
Char. 56: 0 → 1
Char. 85: 1 → 0
Char. 96: 1 → 0
Char. 170: 0 → 1
Char. 194: 0 → 1
Char. 273: 0 → 1
Char. 274: 0 → 1
Char. 278: 3 → 2

***Claudiosaurus germaini*:**
Char. 81: 0 → 1
Char. 105: 0 → 1
Char. 127: 1 → 0
Char. 141: 1 → 0
Char. 144: 1 → 0
Char. 166: 1 → 0
Char. 187: 1 → 0
Char. 199: 0 → 1
Char. 204: 0 → 1
Char. 208: 1 → 0
Char. 222: 1 → 0

***Colobomycter pholeter*:**
Char. 25: 1 → 0
Char. 84: 1 → 0
Char. 154: 1 → 0
Char. 167: 0 → 1
Char. 267: 0 → 1

***Delorhynchus cifelli*:**
Char. 18: 0 → 1
Char. 20: 0 → 1
Char. 21: 0 → 1
Char. 24: 0 → 1
Char. 26: 0 → 1
Char. 28: 0 → 1
Char. 33: 0 → 2
Char. 39: 0 → 1
Char. 48: 0 → 1
Char. 52: 0 → 1
Char. 100: 0 → 1
Char. 111: 0 → 1
Char. 116: 1 → 0
Char. 117: 0 → 1
Char. 131: 0 → 1
Char. 147: 0 → 1
Char. 156: 1 → 0
Char. 159: 0 → 1
Char. 163: 0 → 1
Char. 166: 0 → 1
Char. 167: 0 → 1
Char. 180: 0 → 1
Char. 189: 0 → 1
Char. 191: 0 → 1
Char. 192: 0 → 1
Char. 204: 0 → 2
Char. 267: 0 → 1

**Diadectomorpha:**
Char. 0: 0 → 1
Char. 64: 0 → 1
Char. 70: 0 → 1
Char. 122: 0 → 1
Char. 123: 1 → 0
Char. 146: 1 → 0
Char. 275: 1 → 0
Char. 278: 3 → 0

***Emeroleter levis*:**
Char. 51: 1 → 0

**Eosauropterygia:**
Char. 159: 1 → 0
Char. 166: 1 → 0
Char. 174: 0 → 1
Char. 194: 0 → 2
Char. 272: 1 → 0

***Eudibamus cursoris*:**
Char. 154: 1 → 2

***Feeserpeton oklahomensis*:**
Char. 51: 0 → 1
Char. 157: 0 → 1
Char. 158: 0 → 1

***Hovasaurus boulei*:**
Char. 41: 0 → 1
Char. 55: 1 → 0
Char. 60: 1 → 0
Char. 72: 2 → 1
Char. 77: 0 → 2
Char. 79: 0 → 1
Char. 93: 1 → 0
Char. 113: 0 → 1
Char. 138: 0 → 1
Char. 141: 1 → 0
Char. 146: 1 → 0
Char. 204: 0 → 2
Char. 206: 0 → 2
Char. 278: 0 → 3

**Kuehneosauridae:**
Char. 7: 0 → 1
Char. 26: 0 → 1
Char. 79: 0 → 2
Char. 82: 0 → 1
Char. 98: 1 → 0
Char. 108: 1 → 0
Char. 113: 0 → 1
Char. 128: 0 → 1
Char. 148: 1 → 0
Char. 159: 1 → 0
Char. 181: 0 → 1
Char. 185: 0 → 1
Char. 206: 0 → 2
Char. 245: 0 → 1
Char. 278: 0 → 3

***Lanthanosuchus watsoni*:**
Char. 25: 1 → 0
Char. 51: 0 → 1
Char. 76: 0 → 1
Char. 86: 0 → 1
Char. 95: 0 → 1
Char. 98: 1 → 0
Char. 110: 0 → 1
Char. 113: 0 → 1
Char. 114: 0 → 1
Char. 131: 0 → 1
Char. 137: 0 → 1
Char. 138: 0 → 1
Char. 140: 0 → 2
Char. 144: 1 → 0
Char. 147: 0 → 1
Char. 154: 1 → 2
Char. 192: 0 → 1

***Macroleter poezicus*:**
Char. 52: 0 → 1
Char. 84: 1 → 0
Char. 87: 0 → 1
Char. 140: 0 → 1
Char. 146: 1 → 0
Char. 169: 1 → 0
Char. 235: 0 → 1

***Mesosaurus* spp.:**
Char. 0: 0 → 1
Char. 2: 0 → 1
Char. 6: 0 → 1
Char. 8: 0 → 1
Char. 9: 0 → 1
Char. 13: 0 → 1
Char. 19: 0 → 1
Char. 23: 0 → 1
Char. 26: 0 → 1
Char. 29: 1 → 0
Char. 33: 0 → 1
Char. 38: 1 → 0
Char. 41: 0 → 1
Char. 48: 0 → 1
Char. 50: 1 → 0
Char. 67: 0 → 1
Char. 76: 0 → 1
Char. 83: 0 → 1
Char. 84: 1 → 0
Char. 85: 1 → 0
Char. 94: 0 → 1
Char. 107: 0 → 1
Char. 109: 0 → 1
Char. 111: 0 → 1
Char. 115: 0 → 1
Char. 146: 1 → 0
Char. 148: 0 → 1
Char. 149: 1 → 0
Char. 164: 0 → 1
Char. 167: 0 → 1
Char. 176: 0 → 1
Char. 183: 1 → 0

Char. 184: 0 → 1
Char. 199: 0 → 1
Char. 202: 0 → 1
Char. 204: 0 → 2
Char. 206: 0 → 1
Char. 207: 0 → 1
Char. 209: 0 → 1
Char. 217: 0 → 1
Char. 219: 0 → 1
Char. 220: 0 → 1
Char. 231: 0 → 1
Char. 260: 2 → 0
Char. 272: 2 → 0
Char. 278: 3 → 0

***Microleter mckinzieorum*:**
Char. 0: 0 → 1
Char. 25: 1 → 0
Char. 36: 0 → 1
Char. 39: 0 → 1
Char. 51: 0 → 2
Char. 56: 0 → 1
Char. 70: 0 → 1
Char. 94: 0 → 1
Char. 106: 0 → 1
Char. 159: 0 → 1
Char. 276: 0 → 1

**Millerettidae:**
Char. 24: 0 → 1
Char. 25: 1 → 0
Char. 44: 1 → 0
Char. 56: 0 → 1
Char. 57: 1 → 0
Char. 66: 0 → 2
Char. 78: 1 → 0
Char. 80: 1 → 0
Char. 84: 1 → 2
Char. 88: 1 → 0
Char. 96: 1 → 0
Char. 117: 0 → 1
Char. 124: 0 → 1
Char. 127: 0 → 1
Char. 135: 0 → 1
Char. 145: 0 → 1
Char. 202: 0 → 1
Char. 211: 0 → 1
Char. 230: 0 → 1
Char. 234: 0 → 1
Char. 248: 0 → 1
Char. 252: 0 → 1
Char. 253: 0 → 1

***Nycteroleter ineptus*:**
Char. 278: 0 → 3

***Nyctiphruretus acudens*:**
Char. 21: 0 → 1
Char. 33: 1 → 2
Char. 41: 0 → 1
Char. 81: 1 → 0
Char. 84: 1 → 2
Char. 94: 0 → 1
Char. 215: 1 → 0

Char. 224: 0 → 1
Char. 266: 0 → 1
Char. 272: 2 → 1
Char. 276: 0 → 1

***Orovenator mayorum*:**
Char. 8: 0 → 1
Char. 36: 0 → 1
Char. 160: 0 → 1
Char. 165: 0 → 1

***Owenetta* spp.:**
Char. 169: 1 → 0

***Paleothyris acadiana*:**
Char. 38: 1 → 0
Char. 50: 1 → 0
Char. 66: 1 → 2
Char. 102: 0 → 1
Char. 146: 1 → 0
Char. 180: 0 → 1
Char. 237: 0 → 1
Char. 239: 0 → 1

***Placodus* spp.:**
Char. 0: 1 → 2
Char. 9: 0 → 1
Char. 12: 0 → 1
Char. 13: 0 → 1
Char. 19: 0 → 1
Char. 26: 0 → 1
Char. 31: 0 → 1
Char. 46: 1 → 0
Char. 57: 0 → 1
Char. 78: 1 → 0
Char. 93: 1 → 0
Char. 102: 1 → 2
Char. 109: 1 → 0
Char. 140: 1 → 0
Char. 155: 0 → 1
Char. 163: 1 → 0
Char. 164: 0 → 1

***Procolophon* spp.:**
Char. 41: 0 → 1
Char. 69: 0 → 1
Char. 79: 0 → 1
Char. 88: 0 → 1
Char. 117: 1 → 0
Char. 149: 1 → 0
Char. 180: 0 → 1
Char. 204: 0 → 1
Char. 237: 0 → 1
Char. 238: 0 → 1
Char. 272: 2 → 1
Char. 278: 3 → 0

***Prolacerta broomi*:**
Char. 58: 1 → 0
Char. 66: 1 → 0
Char. 67: 1 → 0
Char. 80: 1 → 0
Char. 139: 1 → 0
Char. 147: 1 → 0
Char. 192: 1 → 0

Char. 203: 1 → 2
Char. 206: 0 → 12

***Rhipaeosaurus* spp.:**
Char. 172: 0 → 1
Char. 277: 0 → 1

**Rhynchocephalia:**
Char. 0: 1 → 2
Char. 24: 0 → 1
Char. 75: 1 → 0
Char. 77: 0 → 1
Char. 94: 1 → 0
Char. 117: 12 → 0
Char. 139: 1 → 0
Char. 167: 1 → 0
Char. 205: 1 → 0

**Rhynchosauria:**
Char. 0: 1 → 0
Char. 7: 0 → 1
Char. 9: 0 → 1
Char. 26: 0 → 1
Char. 68: 0 → 1
Char. 99: 1 → 0
Char. 150: 1 → 0
Char. 160: 0 → 1
Char. 161: 0 → 1
Char. 171: 0 → 2
Char. 182: 1 → 0
Char. 223: 0 → 1
Char. 224: 0 → 1
Char. 241: 0 → 1

***Scutosaurus* spp.:**
Char. 175: 0 → 1
Char. 218: 0 → 1
Char. 243: 0 → 2
Char. 244: 0 → 1
Char. 251: 0 → 1

***Sinosaurosphargis yunguiensis*:**
Char. 8: 0 → 1
Char. 30: 0 → 1
Char. 53: 0 → 1
Char. 82: 0 → 1
Char. 89: 1 → 0
Char. 127: 1 → 0
Char. 150: 1 → 0
Char. 154: 0 → 2
Char. 167: 1 → 0
Char. 253: 0 → 1
Char. 255: 0 → 1

**Squamata:**
Char. 26: 0 → 1
Char. 45: 0 → 1
Char. 79: 0 → 2
Char. 80: 1 → 0
Char. 82: 0 → 1
Char. 92: 1 → 0
Char. 109: 1 → 0
Char. 160: 0 → 1
Char. 245: 0 → 1

***Trilophosaurus buettneri*:**
Char. 5: 1 → 0
Char. 11: 0 → 1
Char. 55: 1 → 0
Char. 93: 1 → 0
Char. 104: 0 → 1
Char. 113: 0 → 1
Char. 122: 0 → 1
Char. 136: 1 → 0
Char. 144: 1 → 0
Char. 154: 0 → 1
Char. 157: 0 → 1
Char. 159: 1 → 0
Char. 177: 0 → 12
Char. 194: 0 → 1
Char. 203: 1 → 2
Char. 207: 1 → 0
Char. 208: 1 → 0
Char. 272: 1 → 0

***Youngina capensis*:**
Char. 56: 0 → 1
Char. 75: 0 → 1
Char. 170: 0 → 1
Char. 211: 0 → 1
Char. 214: 0 → 1
Char. 239: 0 → 1

**Node 50:**
Char. 46: 1 → 0
Char. 65: 0 → 1
Char. 88: 1 → 0
Char. 89: 1 → 0
Char. 93: 1 → 0
Char. 176: 0 → 1
Char. 184: 0 → 1
Char. 195: 0 → 2
Char. 198: 0 → 1
Char. 205: 0 → 1
Char. 210: 0 → 1
Char. 223: 0 → 1
Char. 224: 0 → 1
Char. 232: 0 → 1
Char. 241: 0 → 1
Char. 246: 0 → 2
Char. 254: 0 → 1
Char. 255: 0 → 1
Char. 256: 0 → 1
Char. 258: 0 → 1
Char. 259: 0 → 1
Char. 268: 0 → 1
Char. 269: 0 → 1
Char. 270: 0 → 1

**Node 51:**
Char. 0: 1 → 2
Char. 33: 1 → 2
Char. 42: 0 → 1
Char. 48: 1 → 0
Char. 50: 0 → 1
Char. 57: 0 → 1
Char. 62: 1 → 0
Char. 84: 01 → 2
Char. 129: 1 → 0

Char. 147: 0 → 1
Char. 152: 0 → 1
Char. 158: 0 → 1
Char. 161: 0 → 1
Char. 174: 0 → 1
Char. 181: 0 → 1
Char. 204: 0 → 2
Char. 240: 1 → 0
Char. 247: 0 → 1
Char. 251: 0 → 1
Char. 253: 0 → 2

**Node 52:**
Char. 73: 0 → 1
Char. 81: 1 → 0
Char. 94: 0 → 1
Char. 145: 0 → 1
Char. 148: 0 → 1
Char. 151: 0 → 1
Char. 208: 0 → 1
Char. 217: 0 → 1
Char. 224: 1 → 0

**Node 53:**
Char. 33: 0 → 1
Char. 135: 0 → 1
Char. 159: 0 → 1
Char. 278: 1 → 0

**Node 54:**
Char. 20: 0 → 1
Char. 62: 0 → 1
Char. 141: 0 → 1

**Node 55:**
Char. 0: 0 → 1
Char. 27: 0 → 1
Char. 29: 1 → 0
Char. 40: 2 → 0
Char. 57: 1 → 0
Char. 59: 0 → 1
Char. 60: 0 → 1
Char. 67: 0 → 1
Char. 72: 1 → 2
Char. 89: 0 → 1
Char. 111: 0 → 1
Char. 112: 0 → 1
Char. 120: 0 → 1
Char. 180: 0 → 1
Char. 192: 0 → 1
Char. 193: 0 → 1
Char. 222: 0 → 1
Char. 224: 0 → 1
Char. 234: 0 → 1
Char. 237: 0 → 1
Char. 266: 0 → 1
Char. 278: 3 → 1

**Node 56:**
Char. 29: 0 → 1
Char. 40: 0 → 2
Char. 74: 0 → 1
Char. 88: 0 → 1
Char. 116: 0 → 1
Char. 132: 0 → 1

Char. 149: 0 → 1
Char. 156: 0 → 1
Char. 169: 0 → 1
Char. 201: 0 → 1
Char. 203: 0 → 1
Char. 235: 0 → 1

**Node 57:**
Char. 97: 0 → 1
Char. 104: 1 → 0
Char. 144: 0 → 1

**Node 58:**
Char. 23: 1 → 0
Char. 55: 0 → 1
Char. 78: 0 → 1
Char. 83: 1 → 0
Char. 126: 1 → 0
Char. 154: 2 → 01

**Node 59:**
Char. 1: 1 → 0
Char. 20: 1 → 0
Char. 25: 0 → 1
Char. 49: 1 → 0
Char. 57: 0 → 1
Char. 89: 1 → 0
Char. 94: 1 → 0

**Node 60:**
Char. 20: 0 → 1
Char. 47: 0 → 1
Char. 48: 0 → 1
Char. 79: 0 → 1
Char. 110: 0 → 1
Char. 131: 0 → 1
Char. 137: 0 → 1
Char. 140: 0 → 2
Char. 147: 0 → 1
Char. 159: 0 → 1
Char. 163: 0 → 1
Char. 166: 0 → 1
Char. 169: 1 → 0
Char. 170: 0 → 1

**Node 61:**
Char. 19: 0 → 1
Char. 92: 1 → 0

**Node 62:**
Char. 4: 0 → 1
Char. 29: 0 → 1
Char. 213: 0 → 1
Char. 226: 0 → 2
Char. 228: 0 → 1
Char. 275: 0 → 1

**Node 63:**
Char. 27: 1 → 0
Char. 75: 0 → 1
Char. 107: 0 → 1
Char. 140: 0 → 1
Char. 147: 0 → 1

**Node 64:**
Char. 58: 0 → 1
Char. 61: 0 → 1
Char. 66: 0 → 1
Char. 69: 0 → 1
Char. 150: 0 → 1
Char. 167: 0 → 1
Char. 205: 0 → 1
Char. 239: 0 → 1

**Node 65:**
Char. 70: 0 → 1
Char. 117: 0 → 12
Char. 126: 0 → 1
Char. 182: 0 → 1
Char. 190: 0 → 1
Char. 201: 0 → 1
Char. 214: 0 → 1

**Node 66:**
Char. 24: 0 → 1
Char. 57: 1 → 0
Char. 79: 0 → 1
Char. 83: 0 → 1
Char. 110: 0 → 1
Char. 132: 1 → 0

**Node 67:**
Char. 73: 0 → 1
Char. 131: 1 → 0
Char. 205: 0 → 1
Char. 276: 0 → 1

**Node 68:**
Char. 18: 0 → 1
Char. 37: 0 → 1
Char. 107: 0 → 1
Char. 118: 1 → 0
Char. 125: 1 → 0
Char. 150: 0 → 1

**Node 69:**
Char. 23: 0 → 1
Char. 71: 1 → 0
Char. 102: 0 → 1
Char. 103: 0 → 1
Char. 106: 0 → 1
Char. 214: 0 → 1
Char. 216: 1 → 0
Char. 235: 0 → 2
Char. 241: 0 → 1

**Node 70:**
Char. 138: 0 → 1
Char. 141: 0 → 1
Char. 207: 0 → 1

**Node 71:**
Char. 186: 0 → 1

**Node 72:**
Char. 194: 0 → 1
Char. 201: 1 → 0
Char. 211: 0 → 1

**Node 73:**
Char. 38: 1 → 2
Char. 39: 0 → 1
Char. 49: 0 → 1
Char. 66: 0 → 12
Char. 76: 0 → 1
Char. 85: 1 → 0
Char. 88: 1 → 0
Char. 95: 0 → 1
Char. 112: 0 → 1
Char. 148: 0 → 1
Char. 158: 0 → 1
Char. 159: 0 → 1
Char. 183: 1 → 2
Char. 192: 0 → 1
Char. 215: 0 → 1

**Node 74:**
Char. 87: 0 → 1

**Node 75:**
Char. 25: 1 → 0
Char. 76: 1 → 0

**Node 76:**
Char. 79: 0 → 1
Char. 93: 1 → 0
Char. 133: 0 → 1

**Node 77:**
Char. 85: 0 → 1
Char. 147: 1 → 2
Char. 154: 1 → 0
Char. 278: 3 → 0

**Node 78:**
Char. 50: 1 → 0
Char. 58: 0 → 1
Char. 59: 0 → 1
Char. 60: 0 → 1
Char. 72: 1 → 2
Char. 104: 0 → 1
Char. 105: 0 → 1
Char. 106: 0 → 2
Char. 107: 0 → 1
Char. 109: 0 → 1
Char. 110: 0 → 1
Char. 146: 1 → 0
Char. 155: 0 → 2

**Node 79:**
Char. 33: 1 → 0
Char. 38: 2 → 1
Char. 39: 1 → 0
Char. 42: 0 → 1
Char. 43: 0 → 1
Char. 46: 1 → 0
Char. 49: 1 → 0
Char. 52: 0 → 1
Char. 83: 01 → 2
Char. 84: 1 → 0
Char. 87: 0 → 1
Char. 93: 1 → 0
Char. 161: 0 → 1
Char. 163: 1 → 0
Char. 172: 0 → 2
Char. 174: 0 → 1
Char. 188: 0 → 1
Char. 189: 0 → 1
Char. 195: 0 → 1
Char. 204: 0 → 2
Char. 212: 0 → 1
Char. 236: 0 → 1
Char. 238: 0 → 2
Char. 242: 0 → 1
Char. 274: 0 → 1
Char. 275: 1 → 0

**Node 80:**
Char. 29: 1 → 0
Char. 44: 1 → 0
Char. 59: 0 → 1
Char. 60: 0 → 1
Char. 66: 0 → 1
Char. 67: 0 → 1
Char. 72: 1 → 2
Char. 78: 1 → 0
Char. 84: 1 → 0
Char. 88: 1 → 0
Char. 93: 1 → 0
Char. 111: 0 → 1
Char. 123: 1 → 0
Char. 129: 0 → 1
Char. 154: 1 → 0
Char. 169: 1 → 0
Char. 170: 0 → 1
Char. 197: 0 → 1

**Node 81:**
Char. 83: 0 → 1
Char. 85: 1 → 0
Char. 107: 0 → 1

**Node 82:**
Char. 2: 0 → 1
Char. 6: 0 → 1
Char. 98: 1 → 0
Char. 101: 0 → 1
Char. 113: 0 → 1
Char. 181: 0 → 1
Char. 186: 0 → 1
Char. 193: 1 → 0
Char. 198: 0 → 1
Char. 206: 0 → 2
Char. 220: 0 → 1
Char. 239: 1 → 0
Char. 240: 1 → 0
Char. 266: 1 → 0

**Node 83:**
Char. 33: 1 → 2
Char. 68: 0 → 1
Char. 102: 0 → 1
Char. 103: 0 → 1
Char. 223: 0 → 1
Char. 229: 0 → 1
Char. 235: 1 → 2

**Node 84:**
Char. 17: 0 → 1
Char. 42: 0 → 1
Char. 251: 0 → 1
Char. 264: 0 → 1

**Node 85:**
Char. 41: 0 → 1
Char. 61: 1 → 2
Char. 112: 1 → 0
Char. 128: 0 → 1
Char. 138: 0 → 1
Char. 146: 1 → 0
Char. 155: 0 → 1
Char. 182: 1 → 0
Char. 192: 1 → 2
Char. 224: 0 → 1
Char. 226: 0 → 1
Char. 227: 0 → 1
Char. 233: 0 → 1

**Node 86:**
Char. 61: 1 → 3
Char. 90: 0 → 1
Char. 91: 0 → 1
Char. 109: 1 → 0
Char. 155: 0 → 1
Char. 209: 1 → 0

**ANALYSIS 12**
**(NO *CANDELARIA BARBOURI* AND *ODONTOCHELYS SEMITESTACEA*)**

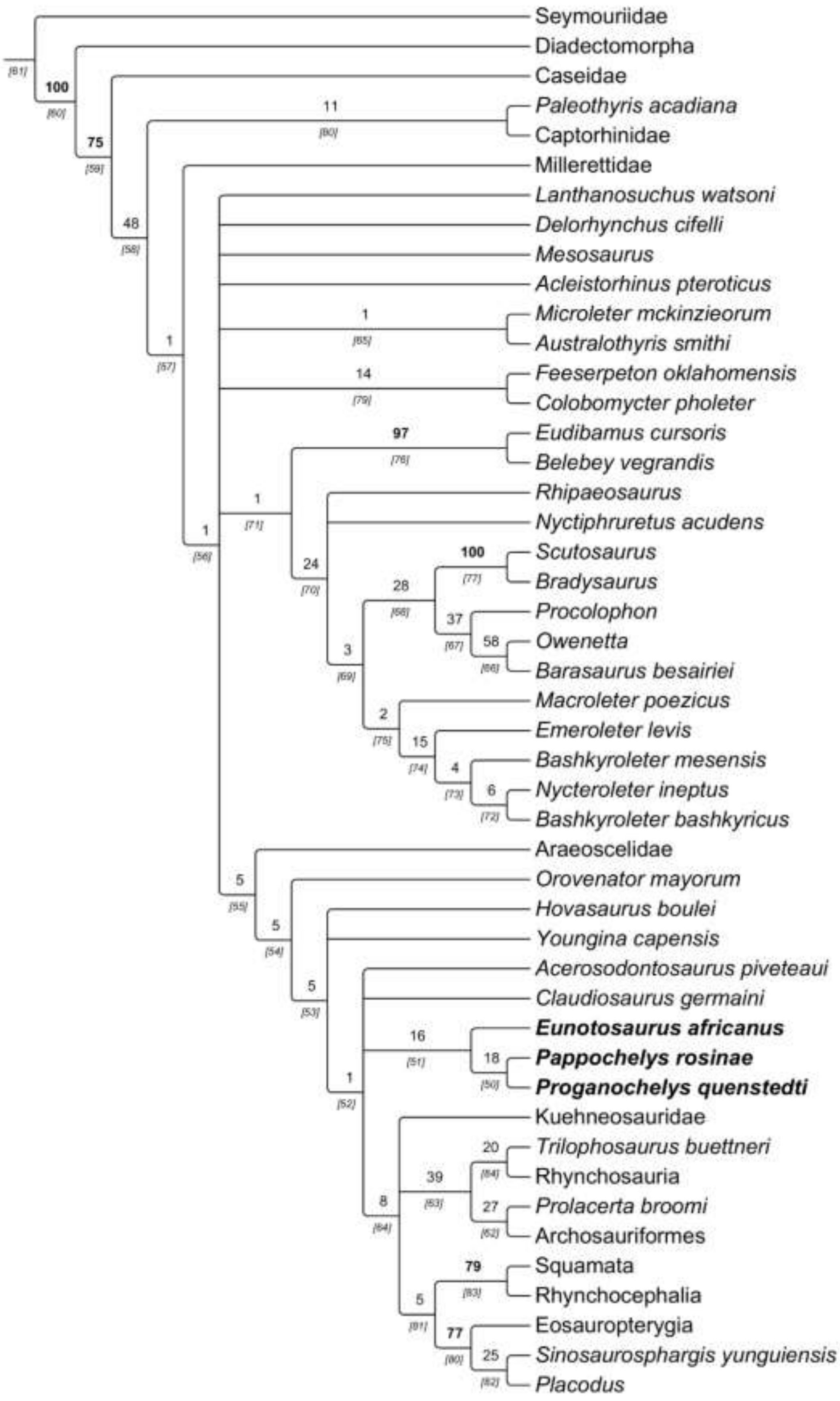

***Proganochelys quenstedti*:**
Char. 11: 0 → 1
Char. 46: 1 → 0
Char. 53: 0 → 1
Char. 77: 0 → 1
Char. 79: 0 → 2
Char. 88: 1 → 0
Char. 89: 1 → 0
Char. 93: 1 → 0
Char. 128: 0 → 1
Char. 175: 0 → 1
Char. 176: 0 → 1
Char. 195: 0 → 2
Char. 198: 0 → 1
Char. 246: 1 → 2
Char. 259: 0 → 1
Char. 262: 0 → 1
Char. 265: 1 → 0
Char. 270: 0 → 1

**Seymouriidae:**
Char. 51: 0 → 2
Char. 54: 1 → 0
Char. 85: 1 → 0
Char. 99: 1 → 0
Char. 107: 0 → 1
Char. 225: 1 → 0
Char. 260: 2 → 0

***Pappochelys rosinae*:**
Char. 0: 2 → 0
Char. 1: 0 → 1
Char. 5: 1 → 0
Char. 41: 0 → 1
Char. 49: 0 → 1
Char. 75: 0 → 1
Char. 81: 0 → 1
Char. 206: 0 → 2
Char. 260: 2 → 0

***Eunotosaurus africanus*:**
Char. 19: 0 → 1
Char. 59: 1 → 0
Char. 60: 1 → 0
Char. 72: 2 → 1
Char. 76: 0 → 1
Char. 97: 1 → 0
Char. 103: 0 → 1
Char. 109: 1 → 0
Char. 112: 1 → 0
Char. 124: 0 → 1
Char. 131: 1 → 0
Char. 153: 1 → 0
Char. 191: 1 → 0
Char. 192: 1 → 0
Char. 211: 0 → 1
Char. 222: 1 → 0
Char. 237: 1 → 0
Char. 249: 0 → 2
Char. 263: 0 → 1
Char. 267: 1 → 0
Char. 272: 1 → 2
Char. 273: 0 → 1
Char. 274: 0 → 1
Char. 275: 0 → 1
Char. 276: 0 → 1
Char. 277: 0 → 1
Char. 278: 0 → 3

***Acerosodontosaurus piveteaui*:**
Char. 78: 1 → 0
Char. 127: 1 → 0
Char. 128: 0 → 1
Char. 155: 0 → 1
Char. 206: 0 → 2
Char. 267: 1 → 0
Char. 278: 0 → 1

***Acleistorhinus pteroticus*:**
Char. 21: 0 → 1
Char. 30: 0 → 1
Char. 33: 0 → 1
Char. 47: 0 → 1
Char. 55: 1 → 0
Char. 56: 0 → 1
Char. 64: 0 → 1
Char. 70: 0 → 1
Char. 79: 0 → 1
Char. 95: 0 → 1
Char. 113: 0 → 1
Char. 114: 0 → 1
Char. 140: 0 → 2
Char. 146: 1 → 0
Char. 169: 1 → 0
Char. 170: 0 → 1

**Araeoscelidae:**
Char. 5: 1 → 0
Char. 23: 0 → 1
Char. 28: 0 → 1
Char. 106: 0 → 1
Char. 116: 1 → 0
Char. 117: 01 → 2
Char. 166: 1 → 0
Char. 169: 1 → 0
Char. 170: 0 → 1
Char. 197: 0 → 1
Char. 221: 1 → 0
Char. 239: 0 → 1

**Archosauriformes:**
Char. 32: 0 → 1
Char. 94: 1 → 0
Char. 112: 1 → 0
Char. 152: 0 → 1
Char. 154: 0 → 2
Char. 166: 1 → 0
Char. 171: 0 → 1
Char. 185: 0 → 1
Char. 204: 0 → 1
Char. 218: 0 → 3
Char. 224: 0 → 1
Char. 242: 0 → 1

***Australothyris smithi*:**
Char. 23: 0 → 1
Char. 34: 0 → 1
Char. 55: 1 → 0
Char. 71: 1 → 0
Char. 85: 1 → 0
Char. 112: 0 → 1
Char. 129: 0 → 1
Char. 159: 1 → 0

***Barasaurus besairiei*:**
Char. 33: 1 → 0
Char. 75: 1 → 0
Char. 216: 0 → 1

***Bashkyroleter bashkyricus*:**
Char. 275: 1 → 0

***Bashkyroleter mesensis*:**
Char. 169: 1 → 0

***Belebey vegrandis*:**
Char. 154: 1 → 0

***Bradysaurus* spp.:**
Char. 19: 0 → 1
Char. 73: 0 → 1
Char. 79: 0 → 1
Char. 135: 1 → 0
Char. 249: 0 → 1

**Captorhinidae:**
Char. 3: 0 → 1
Char. 23: 0 → 1
Char. 26: 0 → 1
Char. 73: 0 → 1
Char. 75: 0 → 1
Char. 83: 0 → 1
Char. 108: 1 → 0
Char. 183: 1 → 0
Char. 203: 1 → 2

**Caseidae:**
Char. 24: 0 → 1
Char. 36: 0 → 1
Char. 38: 1 → 0
Char. 46: 1 → 0
Char. 50: 1 → 0
Char. 56: 0 → 1
Char. 85: 1 → 0
Char. 194: 0 → 1
Char. 273: 0 → 1
Char. 274: 0 → 1
Char. 278: 3 → 2

***Claudiosaurus germaini*:**
Char. 24: 0 → 1
Char. 34: 0 → 1
Char. 36: 0 → 1
Char. 43: 1 → 0
Char. 56: 0 → 1
Char. 81: 0 → 1
Char. 105: 0 → 1
Char. 117: 01 → 2
Char. 127: 1 → 0
Char. 141: 1 → 0
Char. 144: 1 → 0
Char. 166: 1 → 0
Char. 187: 1 → 0
Char. 190: 0 → 1
Char. 199: 0 → 1
Char. 204: 02 → 1
Char. 208: 1 → 0
Char. 222: 1 → 0
Char. 234: 1 → 0

***Colobomycter pholeter*:**
Char. 21: 0 → 1
Char. 25: 1 → 0
Char. 84: 1 → 0
Char. 154: 1 → 0
Char. 167: 0 → 1
Char. 267: 0 → 1

***Delorhynchus cifelli*:**
Char. 18: 0 → 1
Char. 21: 0 → 1
Char. 24: 0 → 1
Char. 26: 0 → 1
Char. 28: 0 → 1
Char. 33: 0 → 2
Char. 39: 0 → 1
Char. 52: 0 → 1
Char. 100: 0 → 1
Char. 111: 0 → 1
Char. 116: 1 → 0
Char. 117: 0 → 1
Char. 156: 1 → 0
Char. 167: 0 → 1
Char. 189: 0 → 1
Char. 191: 0 → 1
Char. 267: 0 → 1

**Diadectomorpha:**
Char. 0: 0 → 1
Char. 44: 0 → 1
Char. 64: 0 → 1
Char. 70: 0 → 1
Char. 75: 0 → 1
Char. 122: 0 → 1
Char. 146: 1 → 0
Char. 275: 1 → 0
Char. 278: 3 → 0

***Emeroleter levis*:**
Char. 51: 1 → 0

**Eosauropterygia:**
Char. 159: 1 → 0
Char. 166: 1 → 0
Char. 174: 0 → 1
Char. 194: 0 → 2
Char. 272: 1 → 0

***Eudibamus cursoris*:**
Char. 154: 1 → 2
Char. 163: 1 → 0

***Feeserpeton oklahomensis*:**
Char. 51: 0 → 1
Char. 70: 0 → 1
Char. 157: 0 → 1
Char. 158: 0 → 1

***Hovasaurus boulei*:**
Char. 41: 0 → 1
Char. 55: 1 → 0
Char. 60: 1 → 0
Char. 72: 2 → 1
Char. 77: 0 → 2
Char. 78: 1 → 0
Char. 79: 0 → 1
Char. 93: 1 → 0
Char. 113: 0 → 1
Char. 138: 0 → 1
Char. 141: 1 → 0
Char. 146: 1 → 0
Char. 154: 0 → 1
Char. 206: 0 → 2
Char. 278: 0 → 3

**Kuehneosauridae:**
Char. 7: 0 → 1
Char. 24: 0 → 1
Char. 26: 0 → 1
Char. 34: 0 → 1
Char. 36: 0 → 1
Char. 43: 1 → 0
Char. 56: 0 → 1
Char. 79: 0 → 2
Char. 82: 0 → 1
Char. 98: 1 → 0
Char. 108: 1 → 0
Char. 113: 0 → 1
Char. 128: 0 → 1
Char. 148: 1 → 0
Char. 159: 1 → 0
Char. 181: 0 → 1
Char. 185: 0 → 1
Char. 206: 0 → 2
Char. 245: 0 → 1
Char. 272: 1 → 2
Char. 278: 0 → 3

***Lanthanosuchus watsoni*:**
Char. 25: 1 → 0
Char. 51: 0 → 1
Char. 86: 0 → 1
Char. 95: 0 → 1
Char. 98: 1 → 0
Char. 113: 0 → 1
Char. 114: 0 → 1
Char. 138: 0 → 1
Char. 140: 0 → 2
Char. 144: 1 → 0
Char. 154: 1 → 2

***Macroleter poezicus*:**
Char. 52: 0 → 1
Char. 84: 1 → 0
Char. 87: 0 → 1
Char. 140: 0 → 1
Char. 146: 1 → 0
Char. 169: 1 → 0
Char. 235: 0 → 1

***Mesosaurus* spp.:**
Char. 0: 0 → 1

Char. 2: 0 → 1
Char. 5: 1 → 0
Char. 6: 0 → 1
Char. 8: 0 → 1
Char. 9: 0 → 1
Char. 13: 0 → 1
Char. 19: 0 → 1
Char. 23: 0 → 1
Char. 26: 0 → 1
Char. 29: 1 → 0
Char. 33: 0 → 1
Char. 38: 1 → 0
Char. 41: 0 → 1
Char. 50: 1 → 0
Char. 67: 0 → 1
Char. 83: 0 → 1
Char. 84: 1 → 0
Char. 85: 1 → 0
Char. 94: 0 → 1
Char. 107: 0 → 1
Char. 109: 0 → 1
Char. 111: 0 → 1
Char. 115: 0 → 1
Char. 131: 1 → 0
Char. 146: 1 → 0
Char. 147: 1 → 0
Char. 148: 0 → 1
Char. 149: 1 → 0
Char. 159: 1 → 0
Char. 163: 1 → 0
Char. 164: 0 → 1
Char. 166: 1 → 0
Char. 167: 0 → 1
Char. 176: 0 → 1
Char. 183: 1 → 0
Char. 184: 0 → 1
Char. 192: 1 → 0
Char. 199: 0 → 1
Char. 202: 0 → 1
Char. 206: 0 → 1
Char. 207: 0 → 1
Char. 209: 0 → 1
Char. 217: 0 → 1
Char. 219: 0 → 1
Char. 220: 0 → 1
Char. 231: 0 → 1
Char. 260: 2 → 0
Char. 272: 2 → 0
Char. 278: 13 → 0

***Microleter mckinzieorum*:**
Char. 0: 0 → 1
Char. 25: 1 → 0
Char. 36: 0 → 1
Char. 39: 0 → 1
Char. 51: 0 → 2
Char. 56: 0 → 1
Char. 70: 0 → 1
Char. 94: 0 → 1
Char. 106: 0 → 1
Char. 131: 1 → 0
Char. 166: 1 → 0
Char. 276: 0 → 1

**Millerettidae:**
Char. 24: 0 → 1
Char. 56: 0 → 1
Char. 57: 1 → 0
Char. 66: 01 → 2
Char. 84: 1 → 2
Char. 117: 0 → 1
Char. 127: 0 → 1
Char. 135: 0 → 1
Char. 145: 0 → 1
Char. 202: 0 → 1
Char. 211: 0 → 1
Char. 230: 0 → 1
Char. 248: 0 → 1
Char. 252: 0 → 1
Char. 253: 0 → 1

***Nycteroleter ineptus*:**
Char. 278: 0 → 3

***Nyctiphruretus acudens*:**
Char. 21: 0 → 1
Char. 33: 1 → 2
Char. 41: 0 → 1
Char. 81: 1 → 0
Char. 84: 1 → 2
Char. 94: 0 → 1
Char. 166: 1 → 0
Char. 167: 0 → 1
Char. 215: 1 → 0
Char. 224: 0 → 1
Char. 266: 0 → 1
Char. 272: 2 → 1
Char. 276: 0 → 1

***Orovenator mayorum*:**
Char. 8: 0 → 1
Char. 24: 0 → 1
Char. 36: 0 → 1
Char. 48: 1 → 0
Char. 160: 0 → 1
Char. 165: 0 → 1

***Owenetta* spp.:**
Char. 169: 1 → 0

***Paleothyris acadiana*:**
Char. 38: 1 → 0
Char. 50: 1 → 0
Char. 66: 1 → 2
Char. 102: 0 → 1
Char. 146: 1 → 0
Char. 180: 0 → 1
Char. 237: 0 → 1
Char. 239: 0 → 1

***Placodus* spp.:**
Char. 0: 1 → 2
Char. 9: 0 → 1
Char. 12: 0 → 1
Char. 13: 0 → 1
Char. 19: 0 → 1
Char. 26: 0 → 1
Char. 31: 0 → 1
Char. 46: 1 → 0
Char. 57: 0 → 1
Char. 78: 1 → 0
Char. 93: 1 → 0
Char. 102: 1 → 2
Char. 109: 1 → 0
Char. 140: 1 → 0
Char. 155: 0 → 1
Char. 163: 1 → 0
Char. 164: 0 → 1

***Procolophon* spp.:**
Char. 41: 0 → 1
Char. 69: 0 → 1
Char. 79: 0 → 1
Char. 88: 0 → 1
Char. 117: 1 → 0
Char. 149: 1 → 0
Char. 237: 0 → 1
Char. 238: 0 → 1
Char. 272: 2 → 1
Char. 278: 3 → 0

***Prolacerta broomi*:**
Char. 58: 1 → 0
Char. 66: 1 → 0
Char. 67: 1 → 0
Char. 80: 1 → 0
Char. 139: 1 → 0
Char. 192: 1 → 0
Char. 203: 1 → 2
Char. 206: 0 → 12

***Rhipaeosaurus* spp.:**
Char. 172: 0 → 1
Char. 277: 0 → 1

**Rhynchocephalia:**
Char. 0: 1 → 2
Char. 24: 0 → 1
Char. 77: 0 → 1
Char. 94: 1 → 0
Char. 139: 1 → 0
Char. 167: 1 → 0
Char. 205: 1 → 0

**Rhynchosauria:**
Char. 0: 1 → 0
Char. 7: 0 → 1
Char. 9: 0 → 1
Char. 26: 0 → 1
Char. 68: 0 → 1
Char. 99: 1 → 0
Char. 150: 1 → 0
Char. 160: 0 → 1
Char. 161: 0 → 1
Char. 171: 0 → 2
Char. 223: 0 → 1
Char. 224: 0 → 1
Char. 241: 0 → 1

***Scutosaurus* spp.:**
Char. 175: 0 → 1
Char. 218: 0 → 1
Char. 243: 0 → 2
Char. 244: 0 → 1
Char. 251: 0 → 1

***Sinosaurosphargis yunguiensis*:**
Char. 8: 0 → 1
Char. 30: 0 → 1
Char. 53: 0 → 1
Char. 82: 0 → 1
Char. 89: 1 → 0
Char. 127: 1 → 0
Char. 150: 1 → 0
Char. 154: 0 → 2
Char. 167: 1 → 0
Char. 253: 0 → 1
Char. 255: 0 → 1

**Squamata:**
Char. 26: 0 → 1
Char. 45: 0 → 1
Char. 79: 0 → 2
Char. 80: 1 → 0
Char. 82: 0 → 1
Char. 92: 1 → 0
Char. 109: 1 → 0
Char. 160: 0 → 1
Char. 245: 0 → 1

***Trilophosaurus buettneri*:**
Char. 5: 1 → 0
Char. 11: 0 → 1
Char. 55: 1 → 0
Char. 93: 1 → 0
Char. 104: 0 → 1
Char. 113: 0 → 1
Char. 122: 0 → 1
Char. 136: 1 → 0
Char. 144: 1 → 0
Char. 154: 0 → 1
Char. 157: 0 → 1
Char. 159: 1 → 0
Char. 177: 0 → 12
Char. 194: 0 → 1
Char. 203: 1 → 2
Char. 207: 1 → 0
Char. 208: 1 → 0
Char. 272: 1 → 0

***Youngina capensis*:**
Char. 5: 1 → 0
Char. 56: 0 → 1
Char. 75: 0 → 1
Char. 163: 1 → 0
Char. 170: 0 → 1
Char. 211: 0 → 1
Char. 239: 0 → 1

**Node 50:**
Char. 43: 1 → 0
Char. 65: 0 → 1
Char. 184: 0 → 1
Char. 205: 0 → 1
Char. 210: 0 → 1
Char. 219: 1 → 0
Char. 241: 0 → 1
Char. 246: 0 → 1
Char. 254: 0 → 1
Char. 255: 0 → 1
Char. 256: 0 → 1
Char. 268: 0 → 1
Char. 269: 0 → 1

**Node 51:**
Char. 0: 1 → 2
Char. 33: 1 → 2
Char. 62: 1 → 0
Char. 84: 01 → 2
Char. 152: 0 → 1
Char. 154: 0 → 1
Char. 155: 0 → 1
Char. 158: 0 → 1
Char. 161: 0 → 1
Char. 174: 0 → 1
Char. 181: 0 → 1
Char. 247: 0 → 1
Char. 251: 0 → 1
Char. 253: 0 → 2

**Node 52:**
Char. 23: 0 → 1
Char. 73: 0 → 1
Char. 81: 1 → 0
Char. 94: 0 → 1
Char. 131: 0 → 1
Char. 145: 0 → 1
Char. 148: 0 → 1
Char. 151: 0 → 1
Char. 208: 0 → 1
Char. 217: 0 → 1
Char. 224: 1 → 0
Char. 272: 2 → 1

**Node 53:**
Char. 33: 0 → 1
Char. 135: 0 → 1
Char. 267: 0 → 1
Char. 278: 1 → 0

**Node 54:**
Char. 62: 0 → 1
Char. 141: 0 → 1

**Node 55:**
Char. 0: 0 → 1
Char. 29: 1 → 0
Char. 40: 2 → 0
Char. 57: 1 → 0
Char. 59: 0 → 1
Char. 60: 0 → 1
Char. 67: 0 → 1
Char. 72: 1 → 2
Char. 89: 0 → 1
Char. 111: 0 → 1
Char. 112: 0 → 1
Char. 120: 0 → 1
Char. 131: 1 → 0
Char. 147: 1 → 0
Char. 154: 1 → 0
Char. 193: 0 → 1
Char. 224: 0 → 1

Char. 237: 0 → 1
Char. 266: 0 → 1

**Node 56:**
Char. 5: 0 → 1
Char. 44: 0 → 1
Char. 48: 0 → 1
Char. 88: 0 → 1
Char. 121: 1 → 0
Char. 131: 0 → 1
Char. 147: 0 → 1
Char. 159: 0 → 1
Char. 163: 0 → 1
Char. 166: 0 → 1
Char. 192: 0 → 1

**Node 57:**
Char. 29: 0 → 1
Char. 84: 0 → 1
Char. 116: 0 → 1
Char. 154: 0 → 1

**Node 58:**
Char. 74: 0 → 1
Char. 132: 0 → 1
Char. 149: 0 → 1
Char. 156: 0 → 1
Char. 203: 0 → 1
Char. 235: 0 → 1

**Node 59:**
Char. 79: 1 → 0
Char. 97: 0 → 1
Char. 183: 0 → 1

**Node 60:**
Char. 23: 1 → 0
Char. 55: 0 → 1
Char. 83: 1 → 0
Char. 111: 1 → 0
Char. 154: 12 → 0
Char. 207: 1 → 0

**Node 61:**
Char. 33: 2 → 0
Char. 127: 1 → 0

**Node 62:**
Char. 19: 0 → 1
Char. 92: 1 → 0

**Node 63:**
Char. 4: 0 → 1
Char. 29: 0 → 1
Char. 48: 1 → 0
Char. 213: 0 → 1
Char. 226: 0 → 2
Char. 228: 0 → 1
Char. 275: 0 → 1

**Node 64:**
Char. 58: 0 → 1
Char. 61: 0 → 1
Char. 66: 0 → 1
Char. 69: 0 → 1
Char. 150: 0 → 1
Char. 167: 0 → 1
Char. 176: 0 → 1
Char. 190: 0 → 1
Char. 205: 0 → 1
Char. 230: 0 → 1
Char. 239: 0 → 1
Char. 260: 2 → 1

**Node 65:**
Char. 24: 0 → 1
Char. 57: 1 → 0
Char. 79: 0 → 1
Char. 83: 0 → 1
Char. 132: 1 → 0

**Node 66:**
Char. 73: 0 → 1
Char. 131: 1 → 0
Char. 205: 0 → 1
Char. 276: 0 → 1

**Node 67:**
Char. 18: 0 → 1
Char. 37: 0 → 1
Char. 107: 0 → 1
Char. 118: 1 → 0
Char. 125: 1 → 0
Char. 150: 0 → 1

**Node 68:**
Char. 23: 0 → 1
Char. 71: 1 → 0
Char. 102: 0 → 1
Char. 103: 0 → 1
Char. 106: 0 → 1
Char. 167: 0 → 1
Char. 214: 0 → 1
Char. 216: 1 → 0
Char. 235: 0 → 2
Char. 241: 0 → 1

**Node 69:**
Char. 138: 0 → 1
Char. 141: 0 → 1
Char. 207: 0 → 1

**Node 70:**
Char. 33: 0 → 1
Char. 44: 1 → 0
Char. 70: 0 → 1
Char. 80: 1 → 0
Char. 117: 0 → 1
Char. 118: 0 → 1
Char. 125: 0 → 1
Char. 194: 0 → 1
Char. 201: 1 → 0
Char. 211: 0 → 1

**Node 71:**
Char. 38: 1 → 2
Char. 39: 0 → 1
Char. 49: 0 → 1
Char. 66: 0 → 12
Char. 85: 1 → 0
Char. 88: 1 → 0
Char. 95: 0 → 1
Char. 112: 0 → 1
Char. 148: 0 → 1
Char. 158: 0 → 1
Char. 183: 1 → 2
Char. 215: 0 → 1

**Node 72:**
Char. 87: 0 → 1

**Node 73:**
Char. 25: 1 → 0
Char. 76: 1 → 0

**Node 74:**
Char. 79: 0 → 1
Char. 93: 1 → 0
Char. 133: 0 → 1

**Node 75:**
Char. 85: 0 → 1
Char. 147: 1 → 2
Char. 154: 1 → 0
Char. 278: 3 → 0

**Node 76:**
Char. 50: 1 → 0
Char. 58: 0 → 1
Char. 59: 0 → 1
Char. 60: 0 → 1
Char. 72: 1 → 2
Char. 104: 0 → 1
Char. 105: 0 → 1
Char. 106: 0 → 2
Char. 107: 0 → 1
Char. 109: 0 → 1
Char. 146: 1 → 0
Char. 155: 0 → 2

**Node 77:**
Char. 33: 1 → 0
Char. 38: 2 → 1
Char. 39: 1 → 0
Char. 42: 0 → 1
Char. 43: 0 → 1
Char. 46: 1 → 0
Char. 49: 1 → 0
Char. 52: 0 → 1
Char. 83: 01 → 2
Char. 84: 1 → 0
Char. 87: 0 → 1
Char. 93: 1 → 0
Char. 161: 0 → 1
Char. 163: 1 → 0
Char. 172: 0 → 2
Char. 174: 0 → 1
Char. 188: 0 → 1
Char. 189: 0 → 1
Char. 195: 0 → 1
Char. 212: 0 → 1
Char. 221: 1 → 0
Char. 236: 0 → 1
Char. 238: 0 → 2
Char. 242: 0 → 1
Char. 274: 0 → 1
Char. 275: 1 → 0

**Node 78:**
Char. 59: 0 → 1
Char. 60: 0 → 1
Char. 67: 0 → 1
Char. 72: 1 → 2
Char. 111: 0 → 1
Char. 129: 0 → 1
Char. 197: 0 → 1

**Node 79:**
Char. 47: 0 → 1
Char. 83: 0 → 1
Char. 85: 1 → 0
Char. 107: 0 → 1
Char. 169: 1 → 0
Char. 170: 0 → 1

**Node 80:**
Char. 2: 0 → 1
Char. 6: 0 → 1
Char. 98: 1 → 0
Char. 101: 0 → 1
Char. 113: 0 → 1
Char. 181: 0 → 1
Char. 186: 0 → 1
Char. 193: 1 → 0
Char. 198: 0 → 1
Char. 206: 0 → 2
Char. 220: 0 → 1
Char. 234: 1 → 0
Char. 239: 1 → 0
Char. 240: 1 → 0
Char. 266: 1 → 0

**Node 81:**
Char. 33: 1 → 2
Char. 68: 0 → 1
Char. 102: 0 → 1
Char. 103: 0 → 1
Char. 223: 0 → 1
Char. 229: 0 → 1
Char. 235: 1 → 2

**Node 82:**
Char. 17: 0 → 1
Char. 42: 0 → 1
Char. 251: 0 → 1
Char. 264: 0 → 1

**Node 83:**
Char. 41: 0 → 1
Char. 48: 1 → 0
Char. 61: 1 → 2
Char. 112: 1 → 0
Char. 128: 0 → 1
Char. 138: 0 → 1
Char. 146: 1 → 0
Char. 155: 0 → 1
Char. 192: 1 → 2
Char. 224: 0 → 1
Char. 226: 0 → 1
Char. 227: 0 → 1
Char. 233: 0 → 1

**Node 84:**
Char. 61: 1 → 3
Char. 90: 0 → 1
Char. 91: 0 → 1
Char. 109: 1 → 0
Char. 155: 0 → 1
Char. 209: 1 → 0

**ANALYSIS 13**
**(NO *CANDELARIA BARBOURI* AND *EUNOTOSAURUS AFRICANUS*)**

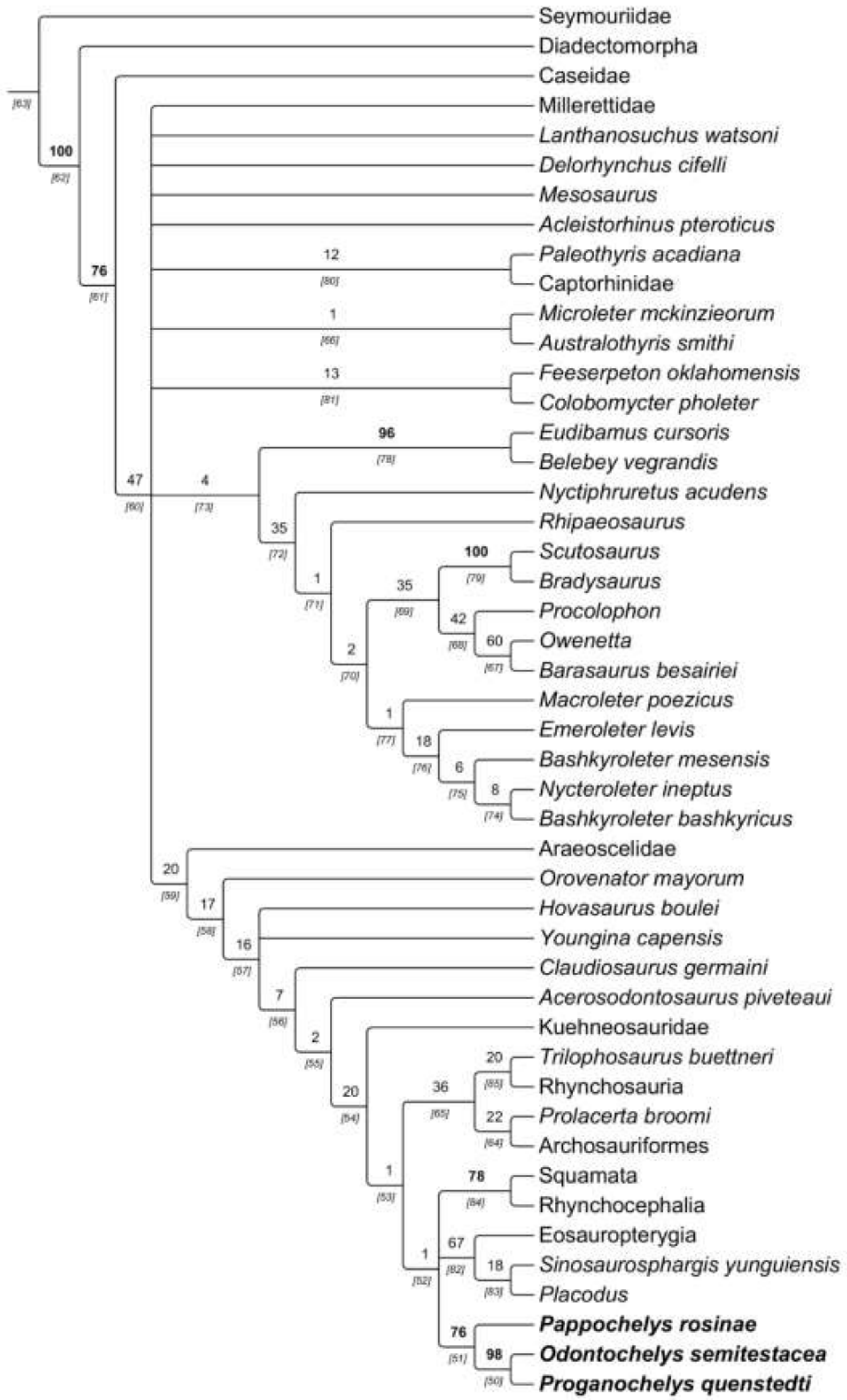

***Proganochelys quenstedti*:**
Char. 8: 0 → 1
Char. 11: 0 → 1
Char. 108: 1 → 0
Char. 128: 0 → 1
Char. 175: 0 → 1
Char. 202: 1 → 0
Char. 206: 2 → 0
Char. 207: 1 → 0
Char. 209: 1 → 0
Char. 235: 2 → 0
Char. 244: 0 → 1
Char. 248: 0 → 1
Char. 250: 0 → 1
Char. 262: 0 → 1

**Seymouriidae:**
Char. 51: 0 → 2
Char. 54: 1 → 0
Char. 71: 1 → 0
Char. 85: 1 → 0
Char. 99: 1 → 0
Char. 107: 0 → 1
Char. 140: 0 → 2
Char. 225: 1 → 0
Char. 260: 2 → 0

***Pappochelys rosinae*:**
Char. 1: 0 → 1
Char. 5: 1 → 0
Char. 12: 0 → 1
Char. 41: 0 → 1
Char. 49: 0 → 1
Char. 55: 1 → 0
Char. 67: 1 → 0
Char. 169: 1 → 0
Char. 176: 1 → 0

***Odontochelys semitestacea*:**
Char. 109: 1 → 0
Char. 112: 1 → 0
Char. 124: 0 → 1
Char. 126: 1 → 0

***Acerosodontosaurus piveteaui*:**
Char. 78: 1 → 0
Char. 267: 1 → 0
Char. 278: 0 → 1

***Acleistorhinus pteroticus*:**
Char. 20: 0 → 1
Char. 21: 0 → 1
Char. 30: 0 → 1
Char. 33: 0 → 1
Char. 47: 0 → 1
Char. 55: 1 → 0
Char. 56: 0 → 1
Char. 64: 0 → 1
Char. 70: 0 → 1
Char. 79: 0 → 1
Char. 95: 0 → 1
Char. 110: 0 → 1
Char. 113: 0 → 1
Char. 114: 0 → 1
Char. 131: 0 → 1
Char. 137: 0 → 1
Char. 140: 0 → 2
Char. 146: 1 → 0
Char. 159: 0 → 1
Char. 166: 0 → 1
Char. 169: 1 → 0
Char. 170: 0 → 1
Char. 278: 3 → 1

**Araeoscelidae:**
Char. 5: 1 → 0
Char. 23: 0 → 1
Char. 28: 0 → 1
Char. 106: 0 → 1
Char. 116: 1 → 0
Char. 169: 1 → 0
Char. 170: 0 → 1
Char. 197: 0 → 1
Char. 239: 0 → 1

**Archosauriformes:**
Char. 32: 0 → 1
Char. 94: 1 → 0
Char. 112: 1 → 0
Char. 152: 0 → 1
Char. 154: 0 → 2
Char. 166: 1 → 0
Char. 171: 0 → 1
Char. 185: 0 → 1
Char. 204: 0 → 1
Char. 218: 0 → 3
Char. 242: 0 → 1

***Australothyris smithi*:**
Char. 23: 0 → 1
Char. 34: 0 → 1
Char. 55: 1 → 0
Char. 71: 1 → 0
Char. 85: 1 → 0
Char. 112: 0 → 1
Char. 129: 0 → 1
Char. 131: 0 → 1
Char. 166: 0 → 1

***Barasaurus besairiei*:**
Char. 33: 1 → 0
Char. 75: 1 → 0
Char. 216: 0 → 1

***Bashkyroleter bashkyricus*:**
Char. 275: 1 → 0

***Bashkyroleter mesensis*:**
Char. 169: 1 → 0

***Belebey vegrandis*:**
Char. 154: 1 → 0

***Bradysaurus* spp.:**
Char. 19: 0 → 1
Char. 73: 0 → 1
Char. 79: 0 → 1
Char. 135: 1 → 0
Char. 249: 0 → 1

**Captorhinidae:**
Char. 3: 0 → 1
Char. 23: 0 → 1
Char. 25: 1 → 0
Char. 26: 0 → 1
Char. 73: 0 → 1
Char. 75: 0 → 1
Char. 83: 0 → 1
Char. 108: 1 → 0
Char. 183: 1 → 0
Char. 201: 1 → 0
Char. 203: 1 → 2
Char. 216: 1 → 0

**Caseidae:**
Char. 24: 0 → 1
Char. 25: 1 → 0
Char. 36: 0 → 1
Char. 38: 1 → 0
Char. 46: 1 → 0
Char. 50: 1 → 0
Char. 56: 0 → 1
Char. 85: 1 → 0
Char. 96: 1 → 0
Char. 170: 0 → 1
Char. 194: 0 → 1
Char. 273: 0 → 1
Char. 274: 0 → 1
Char. 278: 3 → 2

***Claudiosaurus germaini*:**
Char. 24: 0 → 1
Char. 34: 0 → 1
Char. 36: 0 → 1
Char. 64: 0 → 1
Char. 105: 0 → 1
Char. 130: 0 → 1
Char. 144: 1 → 0
Char. 187: 1 → 0
Char. 199: 0 → 1
Char. 203: 1 → 2
Char. 204: 0 → 1
Char. 220: 0 → 1
Char. 222: 1 → 0
Char. 234: 1 → 0

***Colobomycter pholeter*:**
Char. 21: 0 → 1
Char. 25: 1 → 0
Char. 84: 1 → 0
Char. 154: 1 → 0
Char. 167: 0 → 1
Char. 267: 0 → 1

***Delorhynchus cifelli*:**
Char. 18: 0 → 1
Char. 20: 0 → 1
Char. 21: 0 → 1
Char. 24: 0 → 1
Char. 26: 0 → 1
Char. 28: 0 → 1
Char. 33: 0 → 2
Char. 39: 0 → 1
Char. 52: 0 → 1
Char. 100: 0 → 1
Char. 111: 0 → 1
Char. 116: 1 → 0
Char. 117: 0 → 1
Char. 131: 0 → 1
Char. 156: 1 → 0
Char. 159: 0 → 1
Char. 166: 0 → 1
Char. 167: 0 → 1
Char. 180: 0 → 1
Char. 189: 0 → 1
Char. 191: 0 → 1
Char. 192: 0 → 1
Char. 204: 0 → 2
Char. 267: 0 → 1

**Diadectomorpha:**
Char. 0: 0 → 1
Char. 70: 0 → 1
Char. 75: 0 → 1
Char. 122: 0 → 1
Char. 123: 1 → 0
Char. 146: 1 → 0
Char. 275: 1 → 0
Char. 278: 3 → 0

***Emeroleter levis*:**
Char. 51: 1 → 0

**Eosauropterygia:**
Char. 158: 1 → 0
Char. 159: 1 → 0
Char. 166: 1 → 0
Char. 168: 0 → 1
Char. 174: 0 → 1
Char. 178: 0 → 1
Char. 194: 0 → 2
Char. 272: 1 → 0

***Eudibamus cursoris*:**
Char. 154: 1 → 2

***Feeserpeton oklahomensis*:**
Char. 51: 0 → 1
Char. 70: 0 → 1
Char. 157: 0 → 1
Char. 158: 0 → 1

***Hovasaurus boulei*:**
Char. 41: 0 → 1
Char. 55: 1 → 0
Char. 60: 1 → 0
Char. 72: 2 → 1
Char. 77: 0 → 2
Char. 78: 1 → 0
Char. 79: 0 → 1
Char. 93: 1 → 0
Char. 113: 0 → 1
Char. 138: 0 → 1
Char. 146: 1 → 0
Char. 154: 0 → 1
Char. 204: 0 → 2
Char. 206: 0 → 2
Char. 220: 0 → 1
Char. 278: 0 → 3

**Kuehneosauridae:**
Char. 7: 0 → 1
Char. 24: 0 → 1
Char. 34: 0 → 1
Char. 36: 0 → 1
Char. 79: 0 → 2
Char. 82: 0 → 1
Char. 98: 1 → 0
Char. 108: 1 → 0
Char. 113: 0 → 1
Char. 159: 1 → 0
Char. 165: 0 → 1
Char. 185: 0 → 1
Char. 245: 0 → 1
Char. 278: 0 → 3

***Lanthanosuchus watsoni*:**
Char. 25: 1 → 0
Char. 51: 0 → 1
Char. 76: 0 → 1
Char. 86: 0 → 1
Char. 95: 0 → 1
Char. 98: 1 → 0
Char. 110: 0 → 1
Char. 113: 0 → 1
Char. 114: 0 → 1
Char. 131: 0 → 1
Char. 137: 0 → 1
Char. 138: 0 → 1
Char. 140: 0 → 2
Char. 144: 1 → 0
Char. 154: 1 → 2
Char. 192: 0 → 1

***Macroleter poezicus*:**
Char. 52: 0 → 1
Char. 84: 1 → 0
Char. 87: 0 → 1
Char. 140: 0 → 1
Char. 146: 1 → 0
Char. 169: 1 → 0
Char. 235: 0 → 1

***Mesosaurus* spp.:**
Char. 0: 0 → 1
Char. 2: 0 → 1
Char. 5: 1 → 0
Char. 6: 0 → 1
Char. 8: 0 → 1
Char. 9: 0 → 1
Char. 13: 0 → 1
Char. 19: 0 → 1
Char. 23: 0 → 1
Char. 26: 0 → 1
Char. 29: 1 → 0
Char. 33: 0 → 1
Char. 38: 1 → 0
Char. 41: 0 → 1
Char. 50: 1 → 0
Char. 67: 0 → 1
Char. 76: 0 → 1

Char. 83: 0 → 1
Char. 84: 1 → 0
Char. 85: 1 → 0
Char. 94: 0 → 1
Char. 107: 0 → 1
Char. 109: 0 → 1
Char. 111: 0 → 1
Char. 115: 0 → 1
Char. 146: 1 → 0
Char. 148: 0 → 1
Char. 149: 1 → 0
Char. 164: 0 → 1
Char. 167: 0 → 1
Char. 176: 0 → 1
Char. 183: 1 → 0
Char. 184: 0 → 1
Char. 199: 0 → 1
Char. 202: 0 → 1
Char. 204: 0 → 2
Char. 206: 0 → 1
Char. 207: 0 → 1
Char. 209: 0 → 1
Char. 217: 0 → 1
Char. 219: 0 → 1
Char. 220: 0 → 1
Char. 231: 0 → 1
Char. 260: 2 → 0
Char. 272: 2 → 0
Char. 278: 3 → 0

***Microleter mckinzieorum*:**
Char. 0: 0 → 1
Char. 25: 1 → 0
Char. 36: 0 → 1
Char. 39: 0 → 1
Char. 51: 0 → 2
Char. 56: 0 → 1
Char. 70: 0 → 1
Char. 94: 0 → 1
Char. 106: 0 → 1
Char. 159: 0 → 1
Char. 276: 0 → 1

**Millerettidae:**
Char. 5: 1 → 0
Char. 24: 0 → 1
Char. 25: 1 → 0
Char. 44: 1 → 0
Char. 56: 0 → 1
Char. 57: 1 → 0
Char. 66: 0 → 2
Char. 78: 1 → 0
Char. 80: 1 → 0
Char. 84: 1 → 2
Char. 88: 1 → 0
Char. 96: 1 → 0
Char. 117: 0 → 1
Char. 121: 0 → 1
Char. 124: 0 → 1
Char. 127: 0 → 1
Char. 135: 0 → 1
Char. 145: 0 → 1
Char. 202: 0 → 1
Char. 211: 0 → 1
Char. 230: 0 → 1
Char. 234: 0 → 1
Char. 248: 0 → 1
Char. 252: 0 → 1
Char. 253: 0 → 1

***Nycteroleter ineptus*:**
Char. 278: 0 → 3

***Nyctiphruretus acudens*:**
Char. 21: 0 → 1
Char. 33: 1 → 2
Char. 41: 0 → 1
Char. 81: 1 → 0
Char. 84: 1 → 2
Char. 94: 0 → 1
Char. 167: 0 → 1
Char. 224: 0 → 1
Char. 266: 0 → 1
Char. 272: 2 → 1
Char. 276: 0 → 1

***Orovenator mayorum*:**
Char. 8: 0 → 1
Char. 24: 0 → 1
Char. 36: 0 → 1
Char. 160: 0 → 1
Char. 165: 0 → 1

***Owenetta* spp.:**
Char. 169: 1 → 0

***Paleothyris acadiana*:**
Char. 38: 1 → 0
Char. 50: 1 → 0
Char. 66: 1 → 2
Char. 102: 0 → 1
Char. 146: 1 → 0
Char. 180: 0 → 1
Char. 237: 0 → 1
Char. 239: 0 → 1

***Placodus* spp.:**
Char. 9: 0 → 1
Char. 12: 0 → 1
Char. 13: 0 → 1
Char. 19: 0 → 1
Char. 31: 0 → 1
Char. 46: 1 → 0
Char. 57: 0 → 1
Char. 78: 1 → 0
Char. 93: 1 → 0
Char. 102: 1 → 2
Char. 109: 1 → 0
Char. 140: 1 → 0
Char. 163: 1 → 0
Char. 164: 0 → 1

***Procolophon* spp.:**
Char. 41: 0 → 1
Char. 69: 0 → 1
Char. 79: 0 → 1
Char. 88: 0 → 1
Char. 117: 1 → 0
Char. 149: 1 → 0
Char. 180: 0 → 1
Char. 204: 0 → 1
Char. 237: 0 → 1
Char. 238: 0 → 1
Char. 272: 2 → 1
Char. 278: 3 → 0

***Prolacerta broomi*:**
Char. 58: 1 → 0
Char. 66: 1 → 0
Char. 67: 1 → 0
Char. 80: 1 → 0
Char. 139: 1 → 0
Char. 147: 1 → 0
Char. 192: 1 → 0
Char. 203: 1 → 2

***Rhipaeosaurus* spp.:**
Char. 172: 0 → 1
Char. 277: 0 → 1

**Rhynchocephalia:**
Char. 24: 0 → 1
Char. 75: 1 → 0
Char. 77: 0 → 1
Char. 94: 1 → 0
Char. 104: 0 → 1
Char. 117: 12 → 0
Char. 139: 1 → 0
Char. 167: 1 → 0
Char. 200: 1 → 0
Char. 205: 1 → 0

**Rhynchosauria:**
Char. 0: 1 → 0
Char. 7: 0 → 1
Char. 9: 0 → 1
Char. 68: 0 → 1
Char. 99: 1 → 0
Char. 150: 1 → 0
Char. 160: 0 → 1
Char. 161: 0 → 1
Char. 171: 0 → 2
Char. 223: 0 → 1
Char. 241: 0 → 1

***Scutosaurus* spp.:**
Char. 175: 0 → 1
Char. 218: 0 → 1
Char. 243: 0 → 2
Char. 244: 0 → 1
Char. 251: 0 → 1

***Sinosaurosphargis yunguiensis*:**
Char. 8: 0 → 1
Char. 30: 0 → 1
Char. 53: 0 → 1
Char. 82: 0 → 1
Char. 89: 1 → 0
Char. 127: 1 → 0
Char. 150: 1 → 0
Char. 154: 0 → 2
Char. 167: 1 → 0
Char. 253: 0 → 1
Char. 255: 0 → 1

**Squamata:**
Char. 45: 0 → 1
Char. 79: 0 → 2
Char. 80: 1 → 0
Char. 82: 0 → 1
Char. 92: 1 → 0
Char. 109: 1 → 0
Char. 122: 0 → 1
Char. 160: 0 → 1
Char. 245: 0 → 1

***Trilophosaurus buettneri*:**
Char. 5: 1 → 0
Char. 11: 0 → 1
Char. 55: 1 → 0
Char. 93: 1 → 0
Char. 104: 0 → 1
Char. 113: 0 → 1
Char. 122: 0 → 1
Char. 136: 1 → 0
Char. 144: 1 → 0
Char. 154: 0 → 1
Char. 157: 0 → 1
Char. 159: 1 → 0
Char. 177: 0 → 12
Char. 194: 0 → 1
Char. 203: 1 → 2
Char. 207: 1 → 0
Char. 208: 1 → 0
Char. 272: 1 → 0

***Youngina capensis*:**
Char. 5: 1 → 0
Char. 21: 0 → 1
Char. 75: 0 → 1
Char. 170: 0 → 1
Char. 211: 0 → 1
Char. 231: 0 → 1
Char. 239: 0 → 1

**Node 50:**
Char. 46: 1 → 0
Char. 50: 0 → 1
Char. 75: 1 → 0
Char. 89: 1 → 0
Char. 93: 1 → 0
Char. 195: 0 → 2
Char. 198: 0 → 1
Char. 240: 1 → 0
Char. 246: 1 → 2
Char. 259: 0 → 1
Char. 265: 1 → 0
Char. 270: 0 → 1

**Node 51:**
Char. 61: 1 → 0
Char. 62: 1 → 0
Char. 65: 0 → 1
Char. 78: 1 → 0
Char. 194: 0 → 2
Char. 201: 1 → 0
Char. 214: 1 → 0
Char. 219: 1 → 0
Char. 241: 0 → 1
Char. 246: 0 → 1
Char. 247: 0 → 1
Char. 254: 0 → 1
Char. 255: 0 → 1
Char. 256: 0 → 1
Char. 268: 0 → 1
Char. 269: 0 → 1

**Node 52:**
Char. 33: 1 → 2
Char. 102: 0 → 1
Char. 158: 0 → 1
Char. 200: 0 → 1
Char. 223: 0 → 1
Char. 235: 1 → 2

**Node 53:**
Char. 27: 1 → 0
Char. 75: 0 → 1
Char. 107: 0 → 1
Char. 140: 0 → 1
Char. 147: 0 → 1

**Node 54:**
Char. 176: 0 → 1
Char. 205: 0 → 1

**Node 55:**
Char. 81: 1 → 0
Char. 166: 0 → 1
Char. 206: 0 → 2
Char. 208: 0 → 1

**Node 56:**
Char. 23: 0 → 1
Char. 70: 0 → 1
Char. 73: 0 → 1
Char. 94: 0 → 1
Char. 131: 0 → 1

**Node 57:**
Char. 33: 0 → 1
Char. 135: 0 → 1
Char. 159: 0 → 1
Char. 267: 0 → 1
Char. 278: 1 → 0

**Node 58:**
Char. 20: 0 → 1
Char. 62: 0 → 1

**Node 59:**
Char. 0: 0 → 1
Char. 27: 0 → 1
Char. 29: 1 → 0
Char. 38: 1 → 0
Char. 40: 2 → 0
Char. 57: 1 → 0
Char. 59: 0 → 1
Char. 60: 0 → 1
Char. 67: 0 → 1
Char. 72: 1 → 2
Char. 84: 1 → 0

Char. 89: 0 → 1
Char. 111: 0 → 1
Char. 112: 0 → 1
Char. 120: 0 → 1
Char. 154: 1 → 0
Char. 180: 0 → 1
Char. 192: 0 → 1
Char. 193: 0 → 1
Char. 222: 0 → 1
Char. 224: 0 → 1
Char. 234: 0 → 1
Char. 237: 0 → 1
Char. 266: 0 → 1
Char. 278: 3 → 1

**Node 60:**
Char. 5: 0 → 1
Char. 29: 0 → 1
Char. 40: 0 → 2
Char. 74: 0 → 1
Char. 80: 0 → 1
Char. 88: 0 → 1
Char. 116: 0 → 1
Char. 132: 0 → 1
Char. 149: 0 → 1
Char. 169: 0 → 1
Char. 201: 0 → 1
Char. 203: 0 → 1
Char. 235: 0 → 1
Char. 276: 1 → 0

**Node 61:**
Char. 81: 0 → 1
Char. 97: 0 → 1
Char. 144: 0 → 1

**Node 62:**
Char. 55: 0 → 1
Char. 111: 1 → 0
Char. 135: 1 → 0

**Node 63:**
Char. 15: 1 → 0
Char. 20: 1 → 0
Char. 25: 0 → 1
Char. 33: 2 → 0
Char. 76: 1 → 0
Char. 89: 1 → 0
Char. 94: 1 → 0
Char. 127: 1 → 0
Char. 277: 1 → 0

**Node 64:**
Char. 19: 0 → 1
Char. 92: 1 → 0

**Node 65:**
Char. 4: 0 → 1
Char. 29: 0 → 1
Char. 213: 0 → 1
Char. 228: 0 → 1
Char. 275: 0 → 1

**Node 66:**
Char. 24: 0 → 1
Char. 57: 1 → 0
Char. 79: 0 → 1
Char. 83: 0 → 1
Char. 110: 0 → 1
Char. 132: 1 → 0

**Node 67:**
Char. 73: 0 → 1
Char. 131: 1 → 0
Char. 205: 0 → 1
Char. 276: 0 → 1

**Node 68:**
Char. 18: 0 → 1
Char. 37: 0 → 1
Char. 107: 0 → 1
Char. 118: 1 → 0
Char. 125: 1 → 0
Char. 150: 0 → 1

**Node 69:**
Char. 23: 0 → 1
Char. 71: 1 → 0
Char. 102: 0 → 1
Char. 103: 0 → 1
Char. 106: 0 → 1
Char. 167: 0 → 1
Char. 214: 0 → 1
Char. 216: 1 → 0
Char. 235: 0 → 2
Char. 241: 0 → 1

**Node 70:**
Char. 207: 0 → 1

**Node 71:**
Char. 226: 0 → 1

**Node 72:**
Char. 20: 0 → 1
Char. 33: 0 → 1
Char. 44: 1 → 0
Char. 70: 0 → 1
Char. 80: 1 → 0
Char. 117: 0 → 1
Char. 118: 0 → 1
Char. 125: 0 → 1
Char. 131: 0 → 1
Char. 194: 0 → 1
Char. 201: 1 → 0
Char. 211: 0 → 1

**Node 73:**
Char. 38: 1 → 2
Char. 39: 0 → 1
Char. 49: 0 → 1
Char. 66: 0 → 12
Char. 76: 0 → 1
Char. 85: 1 → 0
Char. 88: 1 → 0
Char. 95: 0 → 1
Char. 112: 0 → 1
Char. 148: 0 → 1
Char. 158: 0 → 1
Char. 159: 0 → 1
Char. 183: 1 → 2
Char. 192: 0 → 1

**Node 74:**
Char. 87: 0 → 1

**Node 75:**
Char. 25: 1 → 0
Char. 76: 1 → 0

**Node 76:**
Char. 79: 0 → 1
Char. 93: 1 → 0
Char. 133: 0 → 1

**Node 77:**
Char. 85: 0 → 1
Char. 147: 1 → 2
Char. 154: 1 → 0
Char. 278: 3 → 0

**Node 78:**
Char. 50: 1 → 0
Char. 58: 0 → 1
Char. 59: 0 → 1
Char. 60: 0 → 1
Char. 72: 1 → 2
Char. 104: 0 → 1
Char. 105: 0 → 1
Char. 106: 0 → 2
Char. 107: 0 → 1
Char. 109: 0 → 1
Char. 110: 0 → 1
Char. 146: 1 → 0
Char. 155: 0 → 2

**Node 79:**
Char. 33: 1 → 0
Char. 38: 2 → 1
Char. 39: 1 → 0
Char. 42: 0 → 1
Char. 43: 0 → 1
Char. 46: 1 → 0
Char. 49: 1 → 0
Char. 52: 0 → 1
Char. 83: 01 → 2
Char. 84: 1 → 0
Char. 87: 0 → 1
Char. 93: 1 → 0
Char. 161: 0 → 1
Char. 163: 1 → 0
Char. 172: 0 → 2
Char. 174: 0 → 1
Char. 188: 0 → 1
Char. 189: 0 → 1
Char. 195: 0 → 1
Char. 204: 0 → 2
Char. 212: 0 → 1
Char. 236: 0 → 1
Char. 238: 0 → 2
Char. 242: 0 → 1
Char. 274: 0 → 1
Char. 275: 1 → 0

**Node 80:**
Char. 5: 1 → 0
Char. 29: 1 → 0
Char. 44: 1 → 0
Char. 59: 0 → 1
Char. 60: 0 → 1
Char. 66: 0 → 1
Char. 67: 0 → 1
Char. 72: 1 → 2
Char. 78: 1 → 0
Char. 84: 1 → 0
Char. 88: 1 → 0
Char. 93: 1 → 0
Char. 111: 0 → 1
Char. 123: 1 → 0
Char. 129: 0 → 1
Char. 154: 1 → 0
Char. 169: 1 → 0
Char. 170: 0 → 1
Char. 197: 0 → 1

**Node 81:**
Char. 20: 0 → 1
Char. 47: 0 → 1
Char. 83: 0 → 1
Char. 85: 1 → 0
Char. 107: 0 → 1
Char. 110: 0 → 1
Char. 169: 1 → 0
Char. 170: 0 → 1

**Node 82:**
Char. 2: 0 → 1
Char. 6: 0 → 1
Char. 98: 1 → 0
Char. 101: 0 → 1
Char. 104: 0 → 1
Char. 113: 0 → 1
Char. 186: 0 → 1
Char. 198: 0 → 1
Char. 231: 1 → 0
Char. 232: 1 → 0
Char. 234: 1 → 0
Char. 240: 1 → 0
Char. 266: 1 → 0
Char. 267: 1 → 0

**Node 83:**
Char. 17: 0 → 1
Char. 42: 0 → 1
Char. 251: 0 → 1
Char. 264: 0 → 1

**Node 84:**
Char. 41: 0 → 1
Char. 61: 1 → 2
Char. 112: 1 → 0
Char. 128: 0 → 1
Char. 146: 1 → 0
Char. 165: 0 → 1
Char. 192: 1 → 2
Char. 206: 2 → 0
Char. 233: 0 → 1
Char. 267: 1 → 2

**Node 85:**
Char. 61: 1 → 3
Char. 90: 0 → 1
Char. 91: 0 → 1
Char. 109: 1 → 0
Char. 209: 1 → 0

**Analysis 14**
**(no *Pappochelys rosinae* and *Odontochelys semitestacea*)**

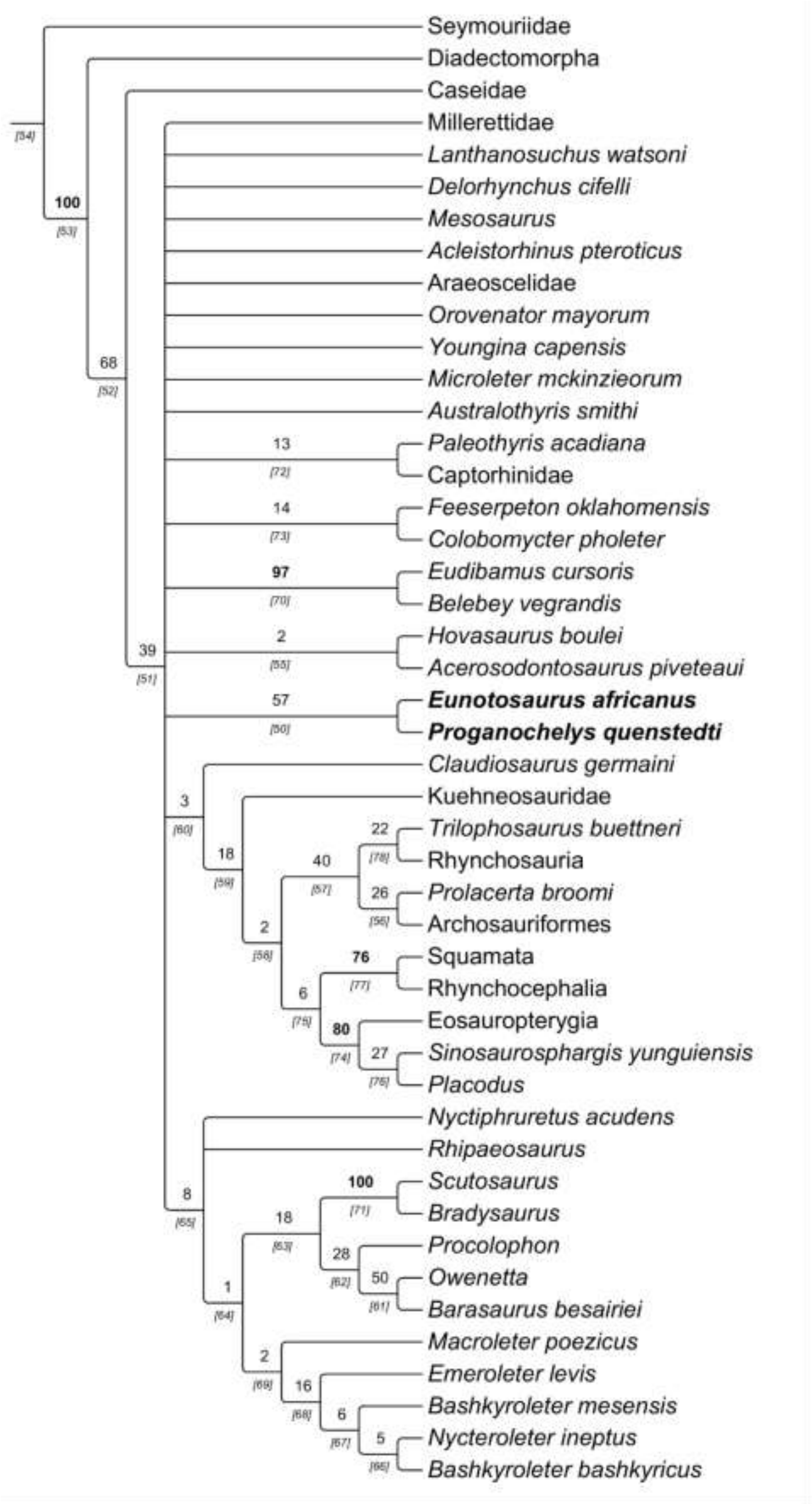

***Proganochelys quenstedti*:**
Char. 8: 0 → 1
Char. 9: 0 → 1
Char. 11: 0 → 1
Char. 26: 0 → 1
Char. 28: 0 → 1
Char. 38: 1 → 2
Char. 39: 0 → 1
Char. 46: 1 → 0
Char. 53: 0 → 1
Char. 59: 0 → 1
Char. 60: 0 → 1
Char. 63: 0 → 1
Char. 65: 0 → 1
Char. 69: 0 → 1
Char. 72: 1 → 2
Char. 79: 0 → 2
Char. 88: 1 → 0
Char. 93: 1 → 0
Char. 106: 0 → 1
Char. 108: 1 → 0
Char. 109: 0 → 1
Char. 110: 0 → 1
Char. 115: 0 → 1
Char. 118: 0 → 1
Char. 121: 0 → 1
Char. 126: 0 → 1
Char. 128: 0 → 1
Char. 131: 0 → 1
Char. 138: 0 → 1
Char. 142: 1 → 0
Char. 160: 0 → 1
Char. 175: 0 → 1
Char. 176: 0 → 1
Char. 184: 0 → 1
Char. 191: 0 → 1
Char. 195: 0 → 2
Char. 198: 0 → 1
Char. 200: 0 → 1
Char. 205: 0 → 1
Char. 210: 0 → 1
Char. 215: 0 → 1
Char. 218: 0 → 1
Char. 223: 0 → 1
Char. 224: 0 → 1
Char. 230: 0 → 1
Char. 232: 0 → 1
Char. 235: 1 → 0
Char. 238: 0 → 2
Char. 241: 0 → 1
Char. 246: 0 → 2
Char. 254: 0 → 1
Char. 255: 0 → 1
Char. 256: 0 → 1
Char. 258: 0 → 1
Char. 259: 0 → 1
Char. 262: 0 → 1
Char. 267: 0 → 1
Char. 268: 0 → 1
Char. 269: 0 → 1
Char. 270: 0 → 1
Char. 271: 0 → 1
Char. 272: 2 → 1
Char. 275: 1 → 0
Char. 278: 3 → 0

**Seymouriidae:**
Char. 51: 0 → 2
Char. 54: 1 → 0
Char. 85: 1 → 0
Char. 99: 1 → 0
Char. 107: 0 → 1
Char. 154: 1 → 2
Char. 225: 1 → 0

***Eunotosaurus africanus*:**
Char. 19: 0 → 1
Char. 43: 0 → 1
Char. 55: 1 → 0
Char. 76: 0 → 1
Char. 89: 0 → 1
Char. 97: 1 → 0
Char. 103: 0 → 1
Char. 112: 1 → 0
Char. 124: 0 → 1
Char. 153: 1 → 0
Char. 192: 1 → 0
Char. 211: 0 → 1
Char. 219: 0 → 1
Char. 249: 0 → 2
Char. 252: 0 → 1
Char. 263: 0 → 1
Char. 273: 0 → 1
Char. 274: 0 → 1
Char. 277: 0 → 1

***Acerosodontosaurus piveteaui*:**
Char. 81: 1 → 0
Char. 208: 0 → 1
Char. 278: 3 → 1

***Acleistorhinus pteroticus*:**
Char. 21: 0 → 1
Char. 30: 0 → 1
Char. 47: 0 → 1
Char. 55: 1 → 0
Char. 56: 0 → 1
Char. 64: 0 → 1
Char. 70: 0 → 1
Char. 79: 0 → 1
Char. 95: 0 → 1
Char. 110: 0 → 1
Char. 112: 1 → 0
Char. 113: 0 → 1
Char. 114: 0 → 1
Char. 120: 1 → 0
Char. 131: 0 → 1
Char. 137: 0 → 1
Char. 140: 0 → 2
Char. 146: 1 → 0
Char. 147: 0 → 1
Char. 169: 1 → 0
Char. 170: 0 → 1
Char. 278: 3 → 1

**Araeoscelidae:**
Char. 0: 0 → 1
Char. 5: 1 → 0
Char. 20: 1 → 0
Char. 23: 0 → 1
Char. 27: 0 → 1
Char. 28: 0 → 1
Char. 38: 1 → 0
Char. 40: 2 → 0
Char. 43: 0 → 1
Char. 50: 1 → 0
Char. 59: 0 → 1
Char. 60: 0 → 1
Char. 72: 1 → 2
Char. 84: 1 → 0
Char. 89: 0 → 1
Char. 106: 0 → 1
Char. 116: 1 → 0
Char. 117: 0 → 2
Char. 154: 1 → 0
Char. 159: 1 → 0
Char. 166: 1 → 0
Char. 169: 1 → 0
Char. 170: 0 → 1
Char. 193: 0 → 1
Char. 197: 0 → 1
Char. 221: 1 → 0
Char. 224: 0 → 1
Char. 239: 0 → 1
Char. 266: 0 → 1
Char. 278: 3 → 1

**Archosauriformes:**
Char. 32: 0 → 1
Char. 94: 1 → 0
Char. 112: 1 → 0
Char. 152: 0 → 1
Char. 154: 0 → 2
Char. 166: 1 → 0
Char. 171: 0 → 1
Char. 185: 0 → 1
Char. 204: 0 → 1
Char. 218: 0 → 3
Char. 224: 0 → 1
Char. 242: 0 → 1

***Australothyris smithi*:**
Char. 23: 0 → 1
Char. 24: 0 → 1
Char. 34: 0 → 1
Char. 55: 1 → 0
Char. 71: 1 → 0
Char. 79: 0 → 1
Char. 83: 0 → 1
Char. 85: 1 → 0
Char. 98: 1 → 0
Char. 100: 0 → 1
Char. 103: 0 → 1
Char. 110: 0 → 1
Char. 123: 1 → 0
Char. 129: 0 → 1
Char. 131: 0 → 1
Char. 132: 1 → 0
Char. 144: 1 → 0
Char. 147: 0 → 1
Char. 149: 1 → 0
Char. 150: 0 → 1
Char. 159: 1 → 0
Char. 163: 1 → 0
Char. 192: 1 → 0

***Barasaurus besairiei*:**
Char. 33: 1 → 0
Char. 75: 1 → 0
Char. 216: 0 → 1

***Bashkyroleter bashkyricus*:**
Char. 275: 1 → 0

***Bashkyroleter mesensis*:**
Char. 169: 1 → 0

***Belebey vegrandis*:**
Char. 154: 1 → 0

***Bradysaurus* spp.:**
Char. 19: 0 → 1
Char. 73: 0 → 1
Char. 79: 0 → 1
Char. 135: 1 → 0
Char. 249: 0 → 1

***Candelaria barbouri*:**
Char. 1: 0 → 1
Char. 5: 1 → 0
Char. 8: 0 → 1
Char. 15: 0 → 1
Char. 23: 0 → 1
Char. 25: 1 → 0
Char. 33: 01 → 2
Char. 49: 0 → 1
Char. 50: 1 → 0
Char. 55: 1 → 0
Char. 76: 0 → 1
Char. 79: 0 → 2
Char. 83: 0 → 1
Char. 88: 1 → 0
Char. 89: 0 → 1
Char. 94: 0 → 1
Char. 95: 0 → 1
Char. 126: 0 → 1
Char. 127: 0 → 1
Char. 132: 1 → 0
Char. 154: 1 → 2
Char. 159: 1 → 0
Char. 169: 1 → 0
Char. 276: 0 → 1
Char. 277: 0 → 1

**Captorhinidae:**
Char. 3: 0 → 1
Char. 23: 0 → 1
Char. 25: 1 → 0
Char. 26: 0 → 1
Char. 73: 0 → 1
Char. 75: 0 → 1
Char. 83: 0 → 1
Char. 108: 1 → 0
Char. 180: 1 → 0
Char. 183: 1 → 0
Char. 201: 1 → 0
Char. 203: 1 → 2
Char. 216: 1 → 0
Char. 240: 1 → 0

**Caseidae:**
Char. 24: 0 → 1
Char. 25: 1 → 0
Char. 36: 0 → 1
Char. 38: 1 → 0
Char. 46: 1 → 0
Char. 50: 1 → 0
Char. 56: 0 → 1
Char. 85: 1 → 0
Char. 170: 0 → 1
Char. 194: 0 → 1
Char. 273: 0 → 1
Char. 274: 0 → 1
Char. 276: 0 → 1

***Claudiosaurus germaini*:**
Char. 64: 0 → 1
Char. 84: 1 → 0
Char. 105: 0 → 1
Char. 130: 0 → 1
Char. 144: 1 → 0
Char. 166: 1 → 0
Char. 199: 0 → 1
Char. 203: 1 → 2
Char. 204: 0 → 1
Char. 220: 0 → 1

***Colobomycter pholeter*:**
Char. 21: 0 → 1
Char. 25: 1 → 0
Char. 84: 1 → 0
Char. 154: 1 → 0
Char. 167: 0 → 1
Char. 267: 0 → 1

***Delorhynchus cifelli*:**
Char. 18: 0 → 1
Char. 21: 0 → 1
Char. 24: 0 → 1
Char. 26: 0 → 1
Char. 28: 0 → 1
Char. 33: 01 → 2
Char. 39: 0 → 1
Char. 52: 0 → 1
Char. 100: 0 → 1
Char. 116: 1 → 0
Char. 117: 0 → 1
Char. 119: 1 → 0
Char. 131: 0 → 1
Char. 147: 0 → 1
Char. 156: 1 → 0
Char. 167: 0 → 1
Char. 189: 0 → 1
Char. 191: 0 → 1
Char. 204: 0 → 2
Char. 267: 0 → 1

**Diadectomorpha:**
Char. 0: 0 → 1
Char. 64: 0 → 1
Char. 70: 0 → 1

Char. 122: 0 → 1
Char. 123: 1 → 0
Char. 146: 1 → 0
Char. 275: 1 → 0

***Emeroleter levis*:**
Char. 0: 0 → 2
Char. 51: 1 → 0

**Eosauropterygia:**
Char. 159: 1 → 0
Char. 166: 1 → 0
Char. 174: 0 → 1
Char. 194: 0 → 2
Char. 272: 1 → 0

***Eudibamus cursoris*:**
Char. 154: 1 → 2
Char. 163: 1 → 0

***Feeserpeton oklahomensis*:**
Char. 51: 0 → 1
Char. 70: 0 → 1
Char. 157: 0 → 1
Char. 158: 0 → 1

***Hovasaurus boulei*:**
Char. 41: 0 → 1
Char. 77: 0 → 2
Char. 79: 0 → 1
Char. 93: 1 → 0
Char. 127: 0 → 1
Char. 204: 0 → 2
Char. 224: 0 → 1

**Kuehneosauridae:**
Char. 7: 0 → 1
Char. 26: 0 → 1
Char. 44: 1 → 0
Char. 79: 0 → 2
Char. 82: 0 → 1
Char. 98: 1 → 0
Char. 108: 1 → 0
Char. 113: 0 → 1
Char. 128: 0 → 1
Char. 159: 1 → 0
Char. 181: 0 → 1
Char. 185: 0 → 1
Char. 206: 0 → 2
Char. 245: 0 → 1

***Lanthanosuchus watsoni*:**
Char. 25: 1 → 0
Char. 51: 0 → 1
Char. 76: 0 → 1
Char. 86: 0 → 1
Char. 95: 0 → 1
Char. 98: 1 → 0
Char. 110: 0 → 1
Char. 113: 0 → 1
Char. 114: 0 → 1
Char. 119: 1 → 0
Char. 131: 0 → 1
Char. 137: 0 → 1
Char. 138: 0 → 1
Char. 140: 0 → 2
Char. 144: 1 → 0
Char. 147: 0 → 1
Char. 154: 1 → 2

***Macroleter poezicus*:**
Char. 0: 0 → 1
Char. 52: 0 → 1
Char. 66: 1 → 2
Char. 84: 1 → 0
Char. 87: 0 → 1
Char. 140: 0 → 1
Char. 146: 1 → 0
Char. 169: 1 → 0
Char. 235: 0 → 1

***Mesosaurus* spp.:**
Char. 0: 0 → 1
Char. 2: 0 → 1
Char. 5: 1 → 0
Char. 6: 0 → 1
Char. 8: 0 → 1
Char. 9: 0 → 1
Char. 13: 0 → 1
Char. 19: 0 → 1
Char. 20: 1 → 0
Char. 23: 0 → 1
Char. 26: 0 → 1
Char. 38: 1 → 0
Char. 41: 0 → 1
Char. 50: 1 → 0
Char. 76: 0 → 1
Char. 83: 0 → 1
Char. 84: 1 → 0
Char. 85: 1 → 0
Char. 94: 0 → 1
Char. 107: 0 → 1
Char. 109: 0 → 1
Char. 112: 1 → 0
Char. 115: 0 → 1
Char. 146: 1 → 0
Char. 148: 0 → 1
Char. 149: 1 → 0
Char. 159: 1 → 0
Char. 163: 1 → 0
Char. 164: 0 → 1
Char. 166: 1 → 0
Char. 167: 0 → 1
Char. 176: 0 → 1
Char. 183: 1 → 0
Char. 184: 0 → 1
Char. 192: 1 → 0
Char. 199: 0 → 1
Char. 202: 0 → 1
Char. 204: 0 → 2
Char. 206: 0 → 1
Char. 207: 0 → 1
Char. 209: 0 → 1
Char. 217: 0 → 1
Char. 219: 0 → 1
Char. 220: 0 → 1
Char. 231: 0 → 1
Char. 234: 1 → 0
Char. 240: 1 → 0
Char. 260: 2 → 0
Char. 272: 2 → 0
Char. 278: 3 → 0

***Microleter mckinzieorum*:**
Char. 0: 0 → 1
Char. 18: 0 → 1
Char. 20: 1 → 0
Char. 24: 0 → 1
Char. 25: 1 → 0
Char. 36: 0 → 1
Char. 39: 0 → 1
Char. 51: 0 → 2
Char. 56: 0 → 1
Char. 70: 0 → 1
Char. 76: 0 → 1
Char. 79: 0 → 1
Char. 83: 0 → 1
Char. 94: 0 → 1
Char. 106: 0 → 1
Char. 110: 0 → 1
Char. 112: 1 → 0
Char. 132: 1 → 0
Char. 166: 1 → 0
Char. 276: 0 → 1
Char. 278: 3 → 1

**Millerettidae:**
Char. 5: 1 → 0
Char. 20: 1 → 0
Char. 24: 0 → 1
Char. 25: 1 → 0
Char. 44: 1 → 0
Char. 56: 0 → 1
Char. 66: 0 → 2
Char. 78: 1 → 0
Char. 80: 1 → 0
Char. 84: 1 → 2
Char. 88: 1 → 0
Char. 96: 1 → 0
Char. 112: 1 → 0
Char. 117: 0 → 1
Char. 120: 1 → 0
Char. 121: 0 → 1
Char. 124: 0 → 1
Char. 127: 0 → 1
Char. 135: 0 → 1
Char. 145: 0 → 1
Char. 159: 1 → 0
Char. 163: 1 → 0
Char. 166: 1 → 0
Char. 180: 1 → 0
Char. 192: 1 → 0
Char. 202: 0 → 1
Char. 211: 0 → 1
Char. 230: 0 → 1
Char. 248: 0 → 1
Char. 252: 0 → 1
Char. 253: 0 → 1

***Nycteroleter ineptus*:**
Char. 278: 0 → 3

***Nyctiphruretus acudens*:**
Char. 0: 0 → 1
Char. 21: 0 → 1
Char. 33: 1 → 2
Char. 41: 0 → 1
Char. 66: 1 → 2
Char. 81: 1 → 0
Char. 83: 0 → 1
Char. 84: 1 → 2
Char. 94: 0 → 1
Char. 166: 1 → 0
Char. 167: 0 → 1
Char. 224: 0 → 1
Char. 266: 0 → 1
Char. 272: 2 → 1
Char. 276: 0 → 1

***Orovenator mayorum*:**
Char. 0: 0 → 1
Char. 8: 0 → 1
Char. 24: 0 → 1
Char. 25: 1 → 0
Char. 36: 0 → 1
Char. 62: 0 → 1
Char. 72: 1 → 2
Char. 89: 0 → 1
Char. 136: 0 → 1
Char. 141: 0 → 1
Char. 159: 1 → 0
Char. 160: 0 → 1
Char. 165: 0 → 1
Char. 278: 3 → 1

***Owenetta* spp.:**
Char. 169: 1 → 0

***Paleothyris acadiana*:**
Char. 38: 1 → 0
Char. 50: 1 → 0
Char. 66: 1 → 2
Char. 102: 0 → 1
Char. 146: 1 → 0
Char. 239: 0 → 1

***Placodus* spp.:**
Char. 0: 1 → 2
Char. 9: 0 → 1
Char. 12: 0 → 1
Char. 13: 0 → 1
Char. 19: 0 → 1
Char. 26: 0 → 1
Char. 31: 0 → 1
Char. 44: 1 → 0
Char. 46: 1 → 0
Char. 57: 0 → 1
Char. 78: 1 → 0
Char. 93: 1 → 0
Char. 102: 1 → 2
Char. 109: 1 → 0
Char. 140: 1 → 0
Char. 155: 0 → 1
Char. 163: 1 → 0
Char. 164: 0 → 1

***Procolophon* spp.:**
Char. 41: 0 → 1
Char. 69: 0 → 1
Char. 79: 0 → 1
Char. 83: 0 → 1
Char. 88: 0 → 1
Char. 117: 1 → 0
Char. 149: 1 → 0
Char. 204: 0 → 1
Char. 237: 0 → 1
Char. 238: 0 → 1
Char. 272: 2 → 1
Char. 278: 3 → 0

***Prolacerta broomi*:**
Char. 58: 1 → 0
Char. 66: 1 → 0
Char. 67: 1 → 0
Char. 80: 1 → 0
Char. 139: 1 → 0
Char. 147: 1 → 0
Char. 192: 1 → 0
Char. 203: 1 → 2
Char. 206: 0 → 12

***Rhipaeosaurus* spp.:**
Char. 172: 0 → 1
Char. 277: 0 → 1

**Rhynchocephalia:**
Char. 0: 1 → 2
Char. 24: 0 → 1
Char. 75: 1 → 0
Char. 77: 0 → 1
Char. 94: 1 → 0
Char. 117: 12 → 0
Char. 139: 1 → 0
Char. 167: 1 → 0
Char. 205: 1 → 0

**Rhynchosauria:**
Char. 0: 1 → 0
Char. 7: 0 → 1
Char. 9: 0 → 1
Char. 26: 0 → 1
Char. 44: 1 → 0
Char. 68: 0 → 1
Char. 99: 1 → 0
Char. 150: 1 → 0
Char. 160: 0 → 1
Char. 161: 0 → 1
Char. 171: 0 → 2
Char. 182: 1 → 0
Char. 223: 0 → 1
Char. 224: 0 → 1
Char. 241: 0 → 1

***Scutosaurus* spp.:**
Char. 175: 0 → 1
Char. 218: 0 → 1
Char. 243: 0 → 2
Char. 244: 0 → 1
Char. 251: 0 → 1

***Sinosaurosphargis yunguiensis*:**
Char. 8: 0 → 1
Char. 30: 0 → 1
Char. 53: 0 → 1
Char. 82: 0 → 1
Char. 89: 1 → 0
Char. 127: 1 → 0
Char. 150: 1 → 0
Char. 154: 0 → 2
Char. 167: 1 → 0
Char. 253: 0 → 1
Char. 255: 0 → 1

**Squamata:**
Char. 26: 0 → 1
Char. 45: 0 → 1
Char. 79: 0 → 2
Char. 80: 1 → 0
Char. 82: 0 → 1
Char. 92: 1 → 0
Char. 109: 1 → 0
Char. 160: 0 → 1
Char. 245: 0 → 1

***Trilophosaurus buettneri*:**
Char. 5: 1 → 0
Char. 11: 0 → 1
Char. 55: 1 → 0
Char. 93: 1 → 0
Char. 104: 0 → 1
Char. 113: 0 → 1
Char. 122: 0 → 1
Char. 144: 1 → 0
Char. 154: 0 → 1
Char. 157: 0 → 1
Char. 159: 1 → 0
Char. 177: 0 → 12
Char. 194: 0 → 1
Char. 203: 1 → 2
Char. 207: 1 → 0
Char. 208: 1 → 0
Char. 272: 1 → 0

***Youngina capensis*:**
Char. 0: 0 → 1
Char. 5: 1 → 0
Char. 21: 0 → 1
Char. 27: 0 → 1
Char. 38: 1 → 0
Char. 40: 2 → 0
Char. 43: 0 → 1
Char. 44: 1 → 0
Char. 50: 1 → 0
Char. 56: 0 → 1
Char. 59: 0 → 1
Char. 60: 0 → 1
Char. 62: 0 → 1
Char. 72: 1 → 2
Char. 75: 0 → 1
Char. 84: 1 → 0
Char. 89: 0 → 1
Char. 109: 0 → 1
Char. 127: 0 → 1
Char. 129: 0 → 1
Char. 134: 0 → 1
Char. 135: 0 → 1
Char. 141: 0 → 1
Char. 154: 1 → 0
Char. 163: 1 → 0
Char. 170: 0 → 1
Char. 179: 0 → 1
Char. 187: 0 → 1
Char. 191: 0 → 1
Char. 193: 0 → 1
Char. 196: 0 → 1
Char. 201: 1 → 0
Char. 202: 0 → 1
Char. 207: 0 → 1
Char. 209: 0 → 1
Char. 211: 0 → 1
Char. 214: 0 → 1
Char. 215: 0 → 1
Char. 219: 0 → 1
Char. 224: 0 → 1
Char. 231: 0 → 1
Char. 239: 0 → 1
Char. 265: 0 → 1
Char. 267: 0 → 1
Char. 275: 1 → 0
Char. 278: 3 → 0

**Node 50:**
Char. 0: 0 → 2
Char. 15: 0 → 1
Char. 21: 0 → 1
Char. 24: 0 → 1
Char. 25: 1 → 0
Char. 33: 01 → 2
Char. 42: 0 → 1
Char. 44: 1 → 0
Char. 64: 0 → 1
Char. 73: 0 → 1
Char. 81: 1 → 0
Char. 84: 1 → 2
Char. 127: 0 → 1
Char. 130: 0 → 1
Char. 134: 0 → 2
Char. 135: 0 → 1
Char. 136: 0 → 1
Char. 141: 0 → 1
Char. 145: 0 → 1
Char. 147: 0 → 1
Char. 148: 0 → 1
Char. 151: 0 → 1
Char. 152: 0 → 1
Char. 155: 0 → 1
Char. 158: 0 → 1
Char. 161: 0 → 1
Char. 174: 0 → 1
Char. 181: 0 → 1
Char. 188: 0 → 1
Char. 196: 0 → 1
Char. 201: 1 → 0
Char. 203: 1 → 2
Char. 204: 0 → 2
Char. 217: 0 → 1
Char. 220: 0 → 1
Char. 231: 0 → 1
Char. 240: 1 → 0
Char. 247: 0 → 1
Char. 248: 0 → 1
Char. 250: 0 → 1
Char. 251: 0 → 1
Char. 253: 0 → 2

**Node 51:**
Char. 5: 0 → 1
Char. 20: 0 → 1
Char. 40: 0 → 2
Char. 74: 0 → 1
Char. 84: 0 → 1
Char. 88: 0 → 1
Char. 112: 0 → 1
Char. 116: 0 → 1
Char. 119: 0 → 1
Char. 149: 0 → 1
Char. 156: 0 → 1
Char. 163: 0 → 1
Char. 166: 0 → 1
Char. 169: 0 → 1
Char. 180: 0 → 1
Char. 192: 0 → 1
Char. 201: 0 → 1
Char. 203: 0 → 1
Char. 221: 0 → 1
Char. 234: 0 → 1
Char. 235: 0 → 1

**Node 52:**
Char. 72: 0 → 1
Char. 93: 0 → 1
Char. 97: 0 → 1

**Node 53:**
Char. 23: 1 → 0
Char. 55: 0 → 1
Char. 78: 0 → 1
Char. 207: 1 → 0

**Node 54:**
Char. 65: 1 → 0
Char. 184: 1 → 0
Char. 187: 1 → 0
Char. 192: 1 → 0
Char. 194: 2 → 0
Char. 202: 1 → 0
Char. 205: 1 → 0
Char. 206: 2 → 0
Char. 210: 1 → 0
Char. 220: 1 → 0
Char. 221: 1 → 0
Char. 222: 1 → 0
Char. 241: 1 → 0
Char. 246: 1 → 0
Char. 247: 1 → 0
Char. 252: 1 → 0
Char. 254: 1 → 0
Char. 255: 1 → 0
Char. 256: 1 → 0
Char. 267: 1 → 0
Char. 268: 1 → 0
Char. 269: 1 → 0

**Node 55:**
Char. 50: 1 → 0
Char. 59: 0 → 1
Char. 78: 1 → 0
Char. 89: 0 → 1
Char. 202: 0 → 1
Char. 206: 0 → 2
Char. 207: 0 → 1
Char. 209: 0 → 1
Char. 265: 0 → 1

**Node 56:**
Char. 19: 0 → 1
Char. 92: 1 → 0

**Node 57:**
Char. 4: 0 → 1
Char. 15: 0 → 1
Char. 29: 0 → 1
Char. 213: 0 → 1
Char. 226: 0 → 2
Char. 228: 0 → 1

**Node 58:**
Char. 43: 0 → 1
Char. 75: 0 → 1
Char. 107: 0 → 1
Char. 140: 0 → 1
Char. 147: 0 → 1

**Node 59:**
Char. 58: 0 → 1
Char. 61: 0 → 1
Char. 66: 0 → 1
Char. 69: 0 → 1
Char. 127: 0 → 1
Char. 141: 0 → 1
Char. 150: 0 → 1
Char. 167: 0 → 1
Char. 205: 0 → 1
Char. 208: 0 → 1
Char. 239: 0 → 1

**Node 60:**
Char. 0: 0 → 1
Char. 23: 0 → 1
Char. 50: 1 → 0
Char. 59: 0 → 1
Char. 60: 0 → 1
Char. 62: 0 → 1
Char. 70: 0 → 1
Char. 72: 1 → 2
Char. 73: 0 → 1
Char. 89: 0 → 1
Char. 94: 0 → 1
Char. 109: 0 → 1
Char. 117: 0 → 12
Char. 126: 0 → 1
Char. 129: 0 → 1
Char. 131: 0 → 1
Char. 154: 1 → 0
Char. 179: 0 → 1
Char. 182: 0 → 1
Char. 188: 0 → 1
Char. 190: 0 → 1
Char. 191: 0 → 1
Char. 196: 0 → 1
Char. 202: 0 → 1
Char. 207: 0 → 1
Char. 209: 0 → 1
Char. 214: 0 → 1
Char. 219: 0 → 1
Char. 265: 0 → 1
Char. 267: 0 → 1

**Node 61:**
Char. 73: 0 → 1
Char. 131: 1 → 0
Char. 205: 0 → 1
Char. 239: 0 → 1
Char. 276: 0 → 1

**Node 62:**
Char. 18: 0 → 1
Char. 37: 0 → 1
Char. 107: 0 → 1
Char. 118: 1 → 0
Char. 125: 1 → 0
Char. 150: 0 → 1

**Node 63:**
Char. 23: 0 → 1
Char. 71: 1 → 0
Char. 102: 0 → 1
Char. 103: 0 → 1
Char. 106: 0 → 1
Char. 167: 0 → 1
Char. 214: 0 → 1
Char. 216: 1 → 0
Char. 235: 0 → 2
Char. 241: 0 → 1

**Node 64:**
Char. 138: 0 → 1
Char. 141: 0 → 1
Char. 207: 0 → 1

**Node 65:**
Char. 38: 1 → 2
Char. 39: 0 → 1
Char. 44: 1 → 0
Char. 49: 0 → 1
Char. 51: 0 → 1
Char. 66: 0 → 1
Char. 70: 0 → 1
Char. 76: 0 → 1
Char. 80: 1 → 0
Char. 88: 1 → 0
Char. 95: 0 → 1
Char. 117: 0 → 1
Char. 118: 0 → 1
Char. 120: 1 → 0
Char. 125: 0 → 1
Char. 131: 0 → 1
Char. 137: 0 → 1
Char. 147: 0 → 1
Char. 148: 0 → 1

Char. 158: 0 → 1
Char. 183: 1 → 2
Char. 194: 0 → 1
Char. 201: 1 → 0
Char. 211: 0 → 1
Char. 234: 1 → 0
Char. 235: 1 → 0
Char. 240: 1 → 0
Char. 252: 0 → 1

**Node 66:**
Char. 87: 0 → 1

**Node 67:**
Char. 25: 1 → 0
Char. 76: 1 → 0

**Node 68:**
Char. 79: 0 → 1
Char. 93: 1 → 0
Char. 133: 0 → 1

**Node 69:**
Char. 147: 1 → 2
Char. 154: 1 → 0
Char. 239: 0 → 1
Char. 278: 3 → 0

**Node 70:**
Char. 20: 1 → 0
Char. 38: 1 → 2
Char. 39: 0 → 1
Char. 50: 1 → 0
Char. 58: 0 → 1
Char. 59: 0 → 1
Char. 60: 0 → 1
Char. 72: 1 → 2
Char. 83: 0 → 1
Char. 85: 1 → 0
Char. 88: 1 → 0
Char. 95: 0 → 1
Char. 104: 0 → 1
Char. 105: 0 → 1
Char. 106: 0 → 2
Char. 107: 0 → 1
Char. 109: 0 → 1
Char. 110: 0 → 1
Char. 146: 1 → 0
Char. 148: 0 → 1
Char. 155: 0 → 2
Char. 183: 1 → 2

**Node 71:**
Char. 0: 0 → 2
Char. 33: 1 → 0
Char. 38: 2 → 1
Char. 39: 1 → 0
Char. 42: 0 → 1
Char. 43: 0 → 1
Char. 46: 1 → 0
Char. 49: 1 → 0
Char. 52: 0 → 1
Char. 83: 0 → 2
Char. 84: 1 → 0
Char. 87: 0 → 1
Char. 93: 1 → 0
Char. 161: 0 → 1
Char. 163: 1 → 0
Char. 172: 0 → 2
Char. 174: 0 → 1
Char. 188: 0 → 1
Char. 189: 0 → 1
Char. 195: 0 → 1
Char. 204: 0 → 2
Char. 212: 0 → 1
Char. 221: 1 → 0
Char. 236: 0 → 1
Char. 238: 0 → 2
Char. 242: 0 → 1
Char. 245: 0 → 1
Char. 274: 0 → 1
Char. 275: 1 → 0

**Node 72:**
Char. 5: 1 → 0
Char. 20: 1 → 0
Char. 44: 1 → 0
Char. 59: 0 → 1
Char. 60: 0 → 1
Char. 66: 0 → 1
Char. 72: 1 → 2
Char. 78: 1 → 0
Char. 84: 1 → 0
Char. 88: 1 → 0
Char. 93: 1 → 0
Char. 112: 1 → 0
Char. 123: 1 → 0
Char. 129: 0 → 1
Char. 154: 1 → 0
Char. 159: 1 → 0
Char. 163: 1 → 0
Char. 169: 1 → 0
Char. 170: 0 → 1
Char. 192: 1 → 0
Char. 197: 0 → 1
Char. 221: 1 → 0
Char. 234: 1 → 0

**Node 73:**
Char. 47: 0 → 1
Char. 83: 0 → 1
Char. 85: 1 → 0
Char. 107: 0 → 1
Char. 110: 0 → 1
Char. 169: 1 → 0
Char. 170: 0 → 1

**Node 74:**
Char. 2: 0 → 1
Char. 6: 0 → 1
Char. 98: 1 → 0
Char. 101: 0 → 1
Char. 113: 0 → 1
Char. 181: 0 → 1
Char. 186: 0 → 1
Char. 198: 0 → 1
Char. 206: 0 → 2
Char. 220: 0 → 1
Char. 239: 1 → 0
Char. 240: 1 → 0

**Node 75:**
Char. 33: 1 → 2
Char. 68: 0 → 1
Char. 102: 0 → 1
Char. 103: 0 → 1
Char. 223: 0 → 1
Char. 229: 0 → 1
Char. 235: 1 → 2

**Node 76:**
Char. 17: 0 → 1
Char. 42: 0 → 1
Char. 251: 0 → 1
Char. 264: 0 → 1

**Node 77:**
Char. 41: 0 → 1
Char. 61: 1 → 2
Char. 112: 1 → 0
Char. 128: 0 → 1
Char. 138: 0 → 1
Char. 146: 1 → 0
Char. 155: 0 → 1
Char. 182: 1 → 0
Char. 192: 1 → 2
Char. 224: 0 → 1
Char. 226: 0 → 1
Char. 227: 0 → 1
Char. 233: 0 → 1

**Node 78:**
Char. 61: 1 → 3
Char. 90: 0 → 1
Char. 91: 0 → 1
Char. 109: 1 → 0
Char. 155: 0 → 1
Char. 209: 1 → 0

**ANALYSIS 15**
**(NO *PAPPOCHELYS ROSINAE* AND *EUNOTOSAURUS AFRICANUS*)**

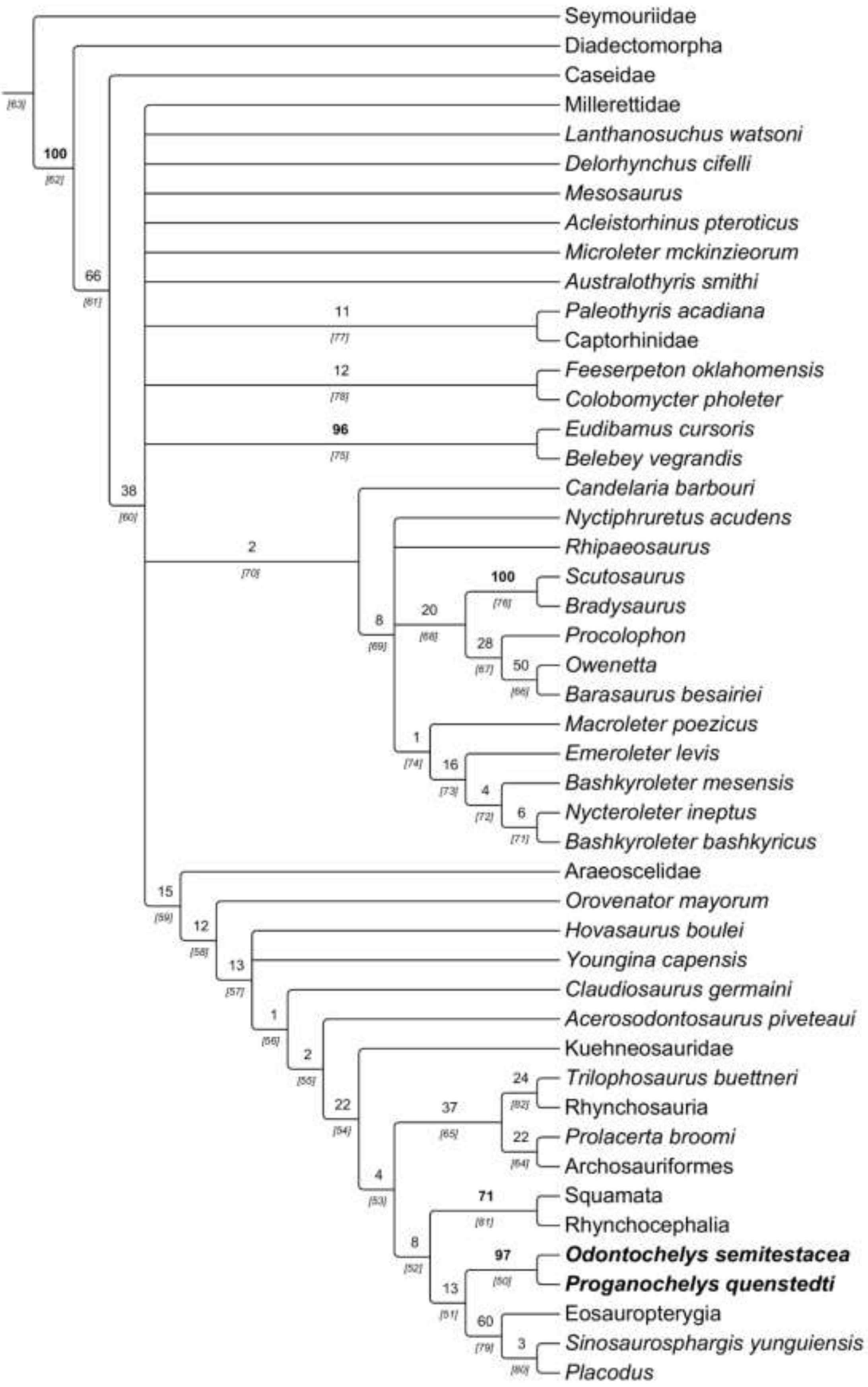

***Proganochelys quenstedti*:**
Char. 8: 0 → 1
Char. 11: 0 → 1
Char. 43: 1 → 0
Char. 108: 1 → 0
Char. 175: 0 → 1
Char. 202: 1 → 0
Char. 207: 1 → 0
Char. 209: 1 → 0
Char. 235: 2 → 0
Char. 244: 0 → 1
Char. 248: 0 → 1
Char. 250: 0 → 1
Char. 262: 0 → 1

**Seymouriidae:**
Char. 51: 0 → 2
Char. 54: 1 → 0
Char. 71: 1 → 0
Char. 83: 0 → 1
Char. 85: 1 → 0
Char. 99: 1 → 0
Char. 107: 0 → 1
Char. 140: 0 → 2
Char. 154: 1 → 2
Char. 225: 1 → 0

***Odontochelys semitestacea*:**
Char. 109: 1 → 0
Char. 112: 1 → 0
Char. 124: 0 → 1
Char. 126: 1 → 0
Char. 252: 0 → 1

***Acerosodontosaurus piveteaui*:**
Char. 78: 1 → 0
Char. 267: 1 → 0
Char. 278: 0 → 1

***Acleistorhinus pteroticus*:**
Char. 20: 0 → 1
Char. 21: 0 → 1
Char. 30: 0 → 1
Char. 33: 0 → 1
Char. 47: 0 → 1
Char. 48: 0 → 1
Char. 55: 1 → 0
Char. 56: 0 → 1
Char. 64: 0 → 1
Char. 70: 0 → 1
Char. 79: 0 → 1
Char. 95: 0 → 1
Char. 110: 0 → 1
Char. 113: 0 → 1
Char. 114: 0 → 1
Char. 131: 0 → 1
Char. 137: 0 → 1
Char. 140: 0 → 2
Char. 146: 1 → 0
Char. 159: 0 → 1
Char. 169: 1 → 0
Char. 170: 0 → 1
Char. 278: 3 → 1

**Araeoscelidae:**
Char. 5: 1 → 0
Char. 23: 0 → 1
Char. 28: 0 → 1
Char. 106: 0 → 1
Char. 116: 1 → 0
Char. 169: 1 → 0
Char. 170: 0 → 1
Char. 197: 0 → 1
Char. 239: 0 → 1

**Archosauriformes:**
Char. 32: 0 → 1
Char. 94: 1 → 0
Char. 112: 1 → 0
Char. 152: 0 → 1
Char. 154: 0 → 2
Char. 166: 1 → 0
Char. 171: 0 → 1
Char. 185: 0 → 1
Char. 204: 0 → 1
Char. 218: 0 → 3
Char. 242: 0 → 1

***Australothyris smithi*:**
Char. 23: 0 → 1
Char. 24: 0 → 1
Char. 34: 0 → 1
Char. 55: 1 → 0
Char. 57: 1 → 0
Char. 71: 1 → 0
Char. 79: 0 → 1
Char. 85: 1 → 0
Char. 98: 1 → 0
Char. 100: 0 → 1
Char. 103: 0 → 1
Char. 110: 0 → 1
Char. 112: 0 → 1
Char. 123: 1 → 0
Char. 129: 0 → 1
Char. 131: 0 → 1
Char. 132: 1 → 0
Char. 144: 1 → 0
Char. 149: 1 → 0
Char. 150: 0 → 1

***Barasaurus besairiei*:**
Char. 33: 1 → 0
Char. 75: 1 → 0
Char. 216: 0 → 1

***Bashkyroleter bashkyricus*:**
Char. 275: 1 → 0

***Bashkyroleter mesensis*:**
Char. 169: 1 → 0

***Belebey vegrandis*:**
Char. 154: 1 → 0

***Bradysaurus* spp.:**
Char. 19: 0 → 1
Char. 73: 0 → 1
Char. 79: 0 → 1
Char. 135: 1 → 0
Char. 249: 0 → 1

***Candelaria barbouri*:**
Char. 1: 0 → 1
Char. 5: 1 → 0
Char. 8: 0 → 1
Char. 15: 0 → 1
Char. 23: 0 → 1
Char. 25: 1 → 0
Char. 29: 1 → 0
Char. 50: 1 → 0
Char. 55: 1 → 0
Char. 57: 1 → 0
Char. 67: 0 → 1
Char. 79: 0 → 2
Char. 89: 0 → 1
Char. 127: 0 → 1
Char. 154: 1 → 2
Char. 169: 1 → 0
Char. 277: 0 → 1

**Captorhinidae:**
Char. 3: 0 → 1
Char. 23: 0 → 1
Char. 25: 1 → 0
Char. 26: 0 → 1
Char. 73: 0 → 1
Char. 75: 0 → 1
Char. 108: 1 → 0
Char. 183: 1 → 0
Char. 201: 1 → 0
Char. 203: 1 → 2
Char. 216: 1 → 0

**Caseidae:**
Char. 24: 0 → 1
Char. 25: 1 → 0
Char. 36: 0 → 1
Char. 38: 1 → 0
Char. 46: 1 → 0
Char. 50: 1 → 0
Char. 56: 0 → 1
Char. 85: 1 → 0
Char. 96: 1 → 0
Char. 170: 0 → 1
Char. 194: 0 → 1
Char. 273: 0 → 1
Char. 274: 0 → 1
Char. 278: 3 → 2

***Claudiosaurus germaini*:**
Char. 24: 0 → 1
Char. 34: 0 → 1
Char. 36: 0 → 1
Char. 64: 0 → 1
Char. 105: 0 → 1
Char. 130: 0 → 1
Char. 144: 1 → 0
Char. 187: 1 → 0
Char. 199: 0 → 1
Char. 203: 1 → 2
Char. 204: 0 → 1
Char. 220: 0 → 1
Char. 222: 1 → 0
Char. 234: 1 → 0

***Colobomycter pholeter*:**
Char. 21: 0 → 1
Char. 25: 1 → 0
Char. 84: 1 → 0
Char. 154: 1 → 0
Char. 167: 0 → 1
Char. 267: 0 → 1

***Delorhynchus cifelli*:**
Char. 18: 0 → 1
Char. 20: 0 → 1
Char. 21: 0 → 1
Char. 24: 0 → 1
Char. 26: 0 → 1
Char. 28: 0 → 1
Char. 33: 0 → 2
Char. 39: 0 → 1
Char. 48: 0 → 1
Char. 52: 0 → 1
Char. 100: 0 → 1
Char. 111: 0 → 1
Char. 116: 1 → 0
Char. 117: 0 → 1
Char. 131: 0 → 1
Char. 156: 1 → 0
Char. 159: 0 → 1
Char. 167: 0 → 1
Char. 180: 0 → 1
Char. 189: 0 → 1
Char. 191: 0 → 1
Char. 204: 0 → 2
Char. 267: 0 → 1

**Diadectomorpha:**
Char. 0: 0 → 1
Char. 70: 0 → 1
Char. 122: 0 → 1
Char. 123: 1 → 0
Char. 146: 1 → 0
Char. 275: 1 → 0
Char. 278: 3 → 0

***Emeroleter levis*:**
Char. 0: 0 → 2
Char. 51: 1 → 0

**Eosauropterygia:**
Char. 158: 1 → 0
Char. 159: 1 → 0
Char. 166: 1 → 0
Char. 168: 0 → 1
Char. 178: 0 → 1
Char. 272: 1 → 0

***Eudibamus cursoris*:**
Char. 154: 1 → 2

***Feeserpeton oklahomensis*:**
Char. 51: 0 → 1
Char. 70: 0 → 1
Char. 157: 0 → 1
Char. 158: 0 → 1

***Hovasaurus boulei*:**
Char. 41: 0 → 1
Char. 55: 1 → 0
Char. 60: 1 → 0
Char. 72: 2 → 1
Char. 77: 0 → 2
Char. 78: 1 → 0
Char. 79: 0 → 1
Char. 93: 1 → 0
Char. 113: 0 → 1
Char. 138: 0 → 1
Char. 146: 1 → 0
Char. 154: 0 → 1
Char. 204: 0 → 2
Char. 206: 0 → 2
Char. 220: 0 → 1
Char. 278: 0 → 3

**Kuehneosauridae:**
Char. 7: 0 → 1
Char. 24: 0 → 1
Char. 34: 0 → 1
Char. 36: 0 → 1
Char. 82: 0 → 1
Char. 98: 1 → 0
Char. 108: 1 → 0
Char. 113: 0 → 1
Char. 159: 1 → 0
Char. 181: 0 → 1
Char. 185: 0 → 1
Char. 245: 0 → 1
Char. 278: 0 → 3

***Lanthanosuchus watsoni*:**
Char. 25: 1 → 0
Char. 51: 0 → 1
Char. 76: 0 → 1
Char. 86: 0 → 1
Char. 95: 0 → 1
Char. 98: 1 → 0
Char. 110: 0 → 1
Char. 113: 0 → 1
Char. 114: 0 → 1
Char. 131: 0 → 1
Char. 137: 0 → 1
Char. 138: 0 → 1
Char. 140: 0 → 2
Char. 144: 1 → 0
Char. 154: 1 → 2

***Macroleter poezicus*:**
Char. 0: 0 → 1
Char. 52: 0 → 1
Char. 66: 1 → 2
Char. 75: 0 → 1
Char. 84: 1 → 0
Char. 87: 0 → 1
Char. 110: 0 → 1
Char. 121: 0 → 1
Char. 140: 0 → 1
Char. 146: 1 → 0

Char. 169: 1 → 0
Char. 235: 0 → 1

***Mesosaurus* spp.:**
Char. 0: 0 → 1
Char. 2: 0 → 1
Char. 5: 1 → 0
Char. 6: 0 → 1
Char. 8: 0 → 1
Char. 9: 0 → 1
Char. 13: 0 → 1
Char. 19: 0 → 1
Char. 23: 0 → 1
Char. 26: 0 → 1
Char. 29: 1 → 0
Char. 33: 0 → 1
Char. 38: 1 → 0
Char. 41: 0 → 1
Char. 48: 0 → 1
Char. 50: 1 → 0
Char. 67: 0 → 1
Char. 76: 0 → 1
Char. 84: 1 → 0
Char. 85: 1 → 0
Char. 94: 0 → 1
Char. 107: 0 → 1
Char. 109: 0 → 1
Char. 111: 0 → 1
Char. 115: 0 → 1
Char. 146: 1 → 0
Char. 148: 0 → 1
Char. 149: 1 → 0
Char. 164: 0 → 1
Char. 167: 0 → 1
Char. 176: 0 → 1
Char. 183: 1 → 0
Char. 184: 0 → 1
Char. 199: 0 → 1
Char. 202: 0 → 1
Char. 204: 0 → 2
Char. 206: 0 → 1
Char. 207: 0 → 1
Char. 209: 0 → 1
Char. 217: 0 → 1
Char. 219: 0 → 1
Char. 220: 0 → 1
Char. 231: 0 → 1
Char. 272: 2 → 0
Char. 278: 3 → 0

***Microleter mckinzieorum*:**
Char. 0: 0 → 1
Char. 18: 0 → 1
Char. 24: 0 → 1
Char. 25: 1 → 0
Char. 36: 0 → 1
Char. 39: 0 → 1
Char. 51: 0 → 2
Char. 56: 0 → 1
Char. 57: 1 → 0
Char. 70: 0 → 1
Char. 76: 0 → 1
Char. 79: 0 → 1
Char. 94: 0 → 1
Char. 106: 0 → 1
Char. 110: 0 → 1
Char. 132: 1 → 0
Char. 159: 0 → 1
Char. 276: 0 → 1
Char. 278: 3 → 1

**Millerettidae:**
Char. 5: 1 → 0
Char. 24: 0 → 1
Char. 25: 1 → 0
Char. 44: 1 → 0
Char. 56: 0 → 1
Char. 57: 1 → 0
Char. 66: 0 → 2
Char. 78: 1 → 0
Char. 80: 1 → 0
Char. 84: 1 → 2
Char. 88: 1 → 0
Char. 96: 1 → 0
Char. 117: 0 → 1
Char. 121: 0 → 1
Char. 124: 0 → 1
Char. 127: 0 → 1
Char. 135: 0 → 1
Char. 145: 0 → 1
Char. 202: 0 → 1
Char. 211: 0 → 1
Char. 230: 0 → 1
Char. 234: 0 → 1
Char. 248: 0 → 1
Char. 252: 0 → 1
Char. 253: 0 → 1

***Nycteroleter ineptus*:**
Char. 278: 0 → 3

***Nyctiphruretus acudens*:**
Char. 0: 0 → 1
Char. 21: 0 → 1
Char. 41: 0 → 1
Char. 66: 1 → 2
Char. 81: 1 → 0
Char. 84: 1 → 2
Char. 167: 0 → 1
Char. 215: 1 → 0
Char. 224: 0 → 1
Char. 226: 1 → 0
Char. 266: 0 → 1
Char. 272: 2 → 1

***Orovenator mayorum*:**
Char. 8: 0 → 1
Char. 24: 0 → 1
Char. 36: 0 → 1
Char. 160: 0 → 1
Char. 165: 0 → 1

***Owenetta* spp.:**
Char. 169: 1 → 0

***Paleothyris acadiana*:**
Char. 38: 1 → 0
Char. 50: 1 → 0
Char. 66: 1 → 2
Char. 102: 0 → 1
Char. 146: 1 → 0
Char. 180: 0 → 1
Char. 237: 0 → 1
Char. 239: 0 → 1

***Placodus* spp.:**
Char. 12: 0 → 1
Char. 13: 0 → 1
Char. 19: 0 → 1
Char. 31: 0 → 1
Char. 46: 1 → 0
Char. 57: 0 → 1
Char. 93: 1 → 0
Char. 102: 1 → 2
Char. 109: 1 → 0
Char. 140: 1 → 0
Char. 163: 1 → 0
Char. 164: 0 → 1

***Procolophon* spp.:**
Char. 41: 0 → 1
Char. 69: 0 → 1
Char. 79: 0 → 1
Char. 88: 0 → 1
Char. 117: 1 → 0
Char. 149: 1 → 0
Char. 180: 0 → 1
Char. 204: 0 → 1
Char. 237: 0 → 1
Char. 238: 0 → 1
Char. 272: 2 → 1
Char. 278: 3 → 0

***Prolacerta broomi*:**
Char. 58: 1 → 0
Char. 66: 1 → 0
Char. 67: 1 → 0
Char. 80: 1 → 0
Char. 139: 1 → 0
Char. 147: 1 → 0
Char. 192: 1 → 0
Char. 203: 1 → 2

***Rhipaeosaurus* spp.:**
Char. 172: 0 → 1
Char. 186: 1 → 0
Char. 277: 0 → 1

**Rhynchocephalia:**
Char. 24: 0 → 1
Char. 81: 0 → 1
Char. 94: 1 → 0
Char. 104: 0 → 1
Char. 117: 12 → 0
Char. 139: 1 → 0
Char. 167: 1 → 0
Char. 205: 1 → 0

**Rhynchosauria:**
Char. 0: 1 → 0
Char. 7: 0 → 1
Char. 9: 0 → 1
Char. 68: 0 → 1
Char. 99: 1 → 0
Char. 150: 1 → 0
Char. 160: 0 → 1
Char. 161: 0 → 1
Char. 171: 0 → 2
Char. 223: 0 → 1
Char. 241: 0 → 1

***Scutosaurus* spp.:**
Char. 175: 0 → 1
Char. 218: 0 → 1
Char. 243: 0 → 2
Char. 244: 0 → 1
Char. 251: 0 → 1

***Sinosaurosphargis yunguiensis*:**
Char. 8: 0 → 1
Char. 23: 0 → 1
Char. 30: 0 → 1
Char. 53: 0 → 1
Char. 82: 0 → 1
Char. 89: 1 → 0
Char. 127: 1 → 0
Char. 150: 1 → 0
Char. 154: 0 → 2
Char. 167: 1 → 0
Char. 253: 0 → 1
Char. 255: 0 → 1

**Squamata:**
Char. 45: 0 → 1
Char. 80: 1 → 0
Char. 82: 0 → 1
Char. 92: 1 → 0
Char. 109: 1 → 0
Char. 122: 0 → 1
Char. 160: 0 → 1
Char. 245: 0 → 1

***Trilophosaurus buettneri*:**
Char. 5: 1 → 0
Char. 11: 0 → 1
Char. 55: 1 → 0
Char. 93: 1 → 0
Char. 104: 0 → 1
Char. 113: 0 → 1
Char. 122: 0 → 1
Char. 136: 1 → 0
Char. 144: 1 → 0
Char. 154: 0 → 1
Char. 157: 0 → 1
Char. 159: 1 → 0
Char. 177: 0 → 12
Char. 194: 0 → 1
Char. 203: 1 → 2
Char. 207: 1 → 0
Char. 208: 1 → 0
Char. 272: 1 → 0

***Youngina capensis*:**
Char. 5: 1 → 0
Char. 21: 0 → 1
Char. 75: 0 → 1
Char. 170: 0 → 1
Char. 211: 0 → 1
Char. 231: 0 → 1
Char. 239: 0 → 1

**Node 50:**
Char. 46: 1 → 0
Char. 50: 0 → 1
Char. 58: 1 → 0
Char. 61: 1 → 0
Char. 62: 1 → 0
Char. 65: 0 → 1
Char. 89: 1 → 0
Char. 93: 1 → 0
Char. 107: 1 → 0
Char. 150: 1 → 0
Char. 154: 0 → 1
Char. 167: 1 → 0
Char. 195: 0 → 2
Char. 201: 1 → 0
Char. 219: 1 → 0
Char. 241: 0 → 1
Char. 246: 0 → 2
Char. 247: 0 → 1
Char. 253: 0 → 2
Char. 254: 0 → 1
Char. 255: 0 → 1
Char. 256: 0 → 1
Char. 258: 0 → 1
Char. 259: 0 → 1
Char. 260: 1 → 2
Char. 265: 1 → 0
Char. 268: 0 → 1
Char. 269: 0 → 1
Char. 270: 0 → 1

**Node 51:**
Char. 161: 0 → 1
Char. 181: 0 → 1
Char. 189: 0 → 1
Char. 193: 1 → 0
Char. 198: 0 → 1
Char. 220: 0 → 1
Char. 239: 1 → 0
Char. 240: 1 → 0

**Node 52:**
Char. 33: 1 → 2
Char. 102: 0 → 1
Char. 158: 0 → 1
Char. 223: 0 → 1
Char. 235: 1 → 2

**Node 53:**
Char. 27: 1 → 0
Char. 48: 1 → 0
Char. 107: 0 → 1
Char. 140: 0 → 1
Char. 147: 0 → 1

**Node 54:**
Char. 176: 0 → 1
Char. 205: 0 → 1
Char. 239: 0 → 1

**Node 55:**
Char. 81: 1 → 0
Char. 208: 0 → 1

**Node 56:**
Char. 23: 0 → 1
Char. 70: 0 → 1
Char. 73: 0 → 1
Char. 94: 0 → 1
Char. 131: 0 → 1

**Node 57:**
Char. 33: 0 → 1
Char. 135: 0 → 1
Char. 159: 0 → 1
Char. 267: 0 → 1
Char. 278: 1 → 0

**Node 58:**
Char. 20: 0 → 1
Char. 62: 0 → 1

**Node 59:**
Char. 0: 0 → 1
Char. 27: 0 → 1
Char. 29: 1 → 0
Char. 38: 1 → 0
Char. 40: 2 → 0
Char. 57: 1 → 0
Char. 59: 0 → 1
Char. 60: 0 → 1
Char. 67: 0 → 1
Char. 72: 1 → 2
Char. 84: 1 → 0
Char. 89: 0 → 1
Char. 111: 0 → 1
Char. 112: 0 → 1
Char. 120: 0 → 1
Char. 154: 1 → 0
Char. 180: 0 → 1
Char. 193: 0 → 1
Char. 222: 0 → 1
Char. 224: 0 → 1
Char. 234: 0 → 1
Char. 237: 0 → 1
Char. 266: 0 → 1
Char. 278: 3 → 1

**Node 60:**
Char. 5: 0 → 1
Char. 29: 0 → 1
Char. 40: 0 → 2
Char. 74: 0 → 1
Char. 88: 0 → 1
Char. 116: 0 → 1
Char. 149: 0 → 1
Char. 169: 0 → 1
Char. 201: 0 → 1
Char. 203: 0 → 1
Char. 235: 0 → 1

**Node 61:**
Char. 81: 0 → 1
Char. 97: 0 → 1
Char. 144: 0 → 1

**Node 62:**
Char. 55: 0 → 1
Char. 78: 0 → 1
Char. 111: 1 → 0
Char. 135: 1 → 0

**Node 63:**
Char. 20: 1 → 0
Char. 25: 0 → 1
Char. 73: 1 → 0
Char. 88: 1 → 0
Char. 89: 1 → 0
Char. 94: 1 → 0
Char. 217: 1 → 0
Char. 220: 1 → 0
Char. 221: 1 → 0
Char. 247: 1 → 0
Char. 252: 1 → 0

**Node 64:**
Char. 19: 0 → 1
Char. 92: 1 → 0

**Node 65:**
Char. 4: 0 → 1
Char. 29: 0 → 1
Char. 213: 0 → 1
Char. 228: 0 → 1
Char. 275: 0 → 1

**Node 66:**
Char. 73: 0 → 1
Char. 131: 1 → 0
Char. 205: 0 → 1
Char. 239: 0 → 1

**Node 67:**
Char. 18: 0 → 1
Char. 37: 0 → 1
Char. 48: 0 → 1
Char. 107: 0 → 1
Char. 118: 1 → 0
Char. 125: 1 → 0
Char. 150: 0 → 1

**Node 68:**
Char. 23: 0 → 1
Char. 71: 1 → 0
Char. 75: 0 → 1
Char. 102: 0 → 1
Char. 103: 0 → 1
Char. 106: 0 → 1
Char. 110: 0 → 1
Char. 155: 0 → 1
Char. 167: 0 → 1
Char. 207: 0 → 1
Char. 214: 0 → 1
Char. 216: 1 → 0
Char. 235: 0 → 2
Char. 241: 0 → 1

**Node 69:**
Char. 66: 0 → 1
Char. 159: 0 → 1

**Node 70:**
Char. 20: 0 → 1
Char. 33: 0 → 12
Char. 49: 0 → 1
Char. 76: 0 → 1
Char. 88: 1 → 0
Char. 95: 0 → 1

**Node 71:**
Char. 87: 0 → 1
Char. 110: 0 → 1

**Node 72:**
Char. 25: 1 → 0
Char. 76: 1 → 0

**Node 73:**
Char. 79: 0 → 1
Char. 86: 0 → 1
Char. 93: 1 → 0
Char. 133: 0 → 1

**Node 74:**
Char. 48: 0 → 1
Char. 100: 0 → 1
Char. 113: 0 → 1
Char. 147: 1 → 2
Char. 154: 1 → 0
Char. 207: 0 → 1
Char. 239: 0 → 1
Char. 278: 3 → 0

**Node 75:**
Char. 38: 1 → 2
Char. 39: 0 → 1
Char. 50: 1 → 0
Char. 58: 0 → 1
Char. 59: 0 → 1
Char. 60: 0 → 1
Char. 72: 1 → 2
Char. 85: 1 → 0
Char. 88: 1 → 0
Char. 95: 0 → 1
Char. 104: 0 → 1
Char. 105: 0 → 1
Char. 106: 0 → 2
Char. 107: 0 → 1
Char. 109: 0 → 1
Char. 110: 0 → 1
Char. 146: 1 → 0
Char. 148: 0 → 1
Char. 155: 0 → 2
Char. 183: 1 → 2

**Node 76:**
Char. 0: 0 → 2
Char. 33: 1 → 0
Char. 38: 2 → 1
Char. 39: 1 → 0
Char. 42: 0 → 1
Char. 43: 0 → 1
Char. 46: 1 → 0
Char. 49: 1 → 0
Char. 52: 0 → 1
Char. 83: 01 → 2
Char. 84: 1 → 0
Char. 87: 0 → 1
Char. 93: 1 → 0
Char. 100: 0 → 1
Char. 113: 0 → 1
Char. 143: 0 → 1
Char. 161: 0 → 1
Char. 163: 1 → 0
Char. 172: 0 → 2
Char. 174: 0 → 1
Char. 188: 0 → 1
Char. 189: 0 → 1
Char. 195: 0 → 1
Char. 204: 0 → 2
Char. 212: 0 → 1
Char. 236: 0 → 1
Char. 238: 0 → 2
Char. 242: 0 → 1
Char. 245: 0 → 1
Char. 274: 0 → 1
Char. 275: 1 → 0

**Node 77:**
Char. 5: 1 → 0
Char. 29: 1 → 0
Char. 44: 1 → 0
Char. 59: 0 → 1
Char. 60: 0 → 1
Char. 66: 0 → 1
Char. 67: 0 → 1
Char. 72: 1 → 2
Char. 78: 1 → 0
Char. 84: 1 → 0
Char. 88: 1 → 0
Char. 93: 1 → 0
Char. 111: 0 → 1
Char. 123: 1 → 0
Char. 129: 0 → 1
Char. 154: 1 → 0
Char. 169: 1 → 0
Char. 170: 0 → 1
Char. 197: 0 → 1

**Node 78:**
Char. 20: 0 → 1
Char. 47: 0 → 1
Char. 85: 1 → 0
Char. 107: 0 → 1
Char. 110: 0 → 1
Char. 169: 1 → 0
Char. 170: 0 → 1

**Node 79:**
Char. 2: 0 → 1
Char. 6: 0 → 1
Char. 81: 0 → 1
Char. 98: 1 → 0
Char. 101: 0 → 1
Char. 104: 0 → 1
Char. 113: 0 → 1
Char. 186: 0 → 1
Char. 231: 1 → 0
Char. 232: 1 → 0
Char. 234: 1 → 0
Char. 266: 1 → 0
Char. 267: 1 → 0

**Node 80:**
Char. 17: 0 → 1
Char. 264: 0 → 1

**Node 81:**
Char. 41: 0 → 1
Char. 61: 1 → 2
Char. 112: 1 → 0
Char. 146: 1 → 0
Char. 192: 1 → 2
Char. 233: 0 → 1
Char. 267: 1 → 2

**Node 82:**
Char. 61: 1 → 3
Char. 90: 0 → 1
Char. 91: 0 → 1
Char. 109: 1 → 0
Char. 209: 1 → 0

**ANALYSIS 16**

**(NO *ODONTOCHELYS SEMITESTACEA* AND *EUNOTOSAURUS AFRICANUS*)**

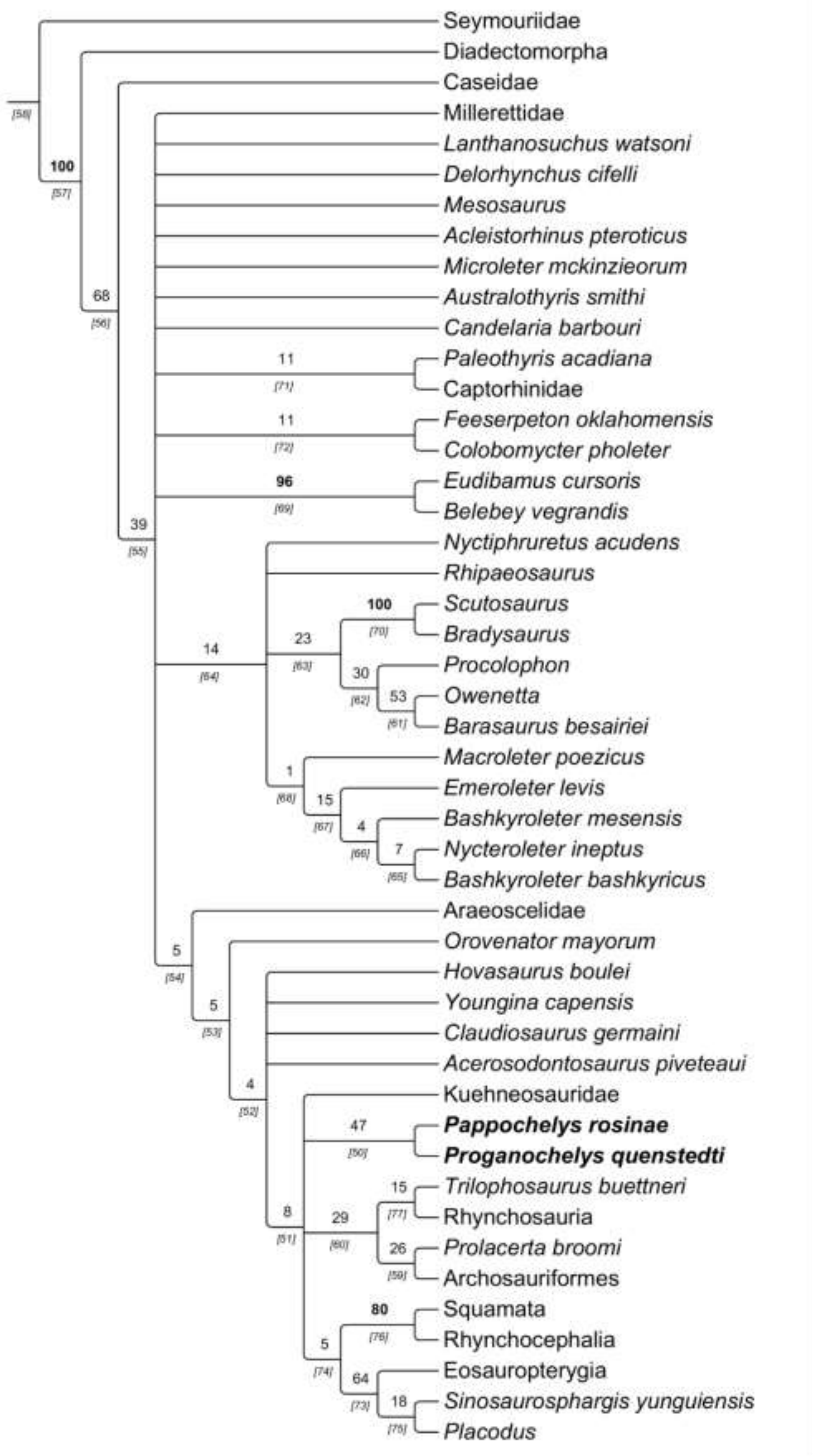

***Proganochelys quenstedti*:**
Char. 11: 0 → 1
Char. 23: 1 → 0
Char. 24: 0 → 1
Char. 42: 0 → 1
Char. 46: 1 → 0
Char. 48: 1 → 0
Char. 50: 0 → 1
Char. 53: 0 → 1
Char. 57: 0 → 1
Char. 77: 0 → 1
Char. 79: 0 → 2
Char. 88: 1 → 0
Char. 89: 1 → 0
Char. 93: 1 → 0
Char. 128: 0 → 1
Char. 129: 1 → 0
Char. 175: 0 → 1
Char. 195: 0 → 2
Char. 198: 0 → 1
Char. 202: 1 → 0
Char. 240: 1 → 0
Char. 246: 1 → 2
Char. 248: 0 → 1
Char. 250: 0 → 1
Char. 259: 0 → 1
Char. 262: 0 → 1
Char. 265: 1 → 0
Char. 270: 0 → 1

**Seymouriidae:**
Char. 23: 0 → 1
Char. 51: 0 → 2
Char. 54: 1 → 0
Char. 71: 1 → 0
Char. 83: 0 → 1
Char. 85: 1 → 0
Char. 99: 1 → 0
Char. 107: 0 → 1
Char. 126: 0 → 1
Char. 140: 0 → 2
Char. 154: 1 → 2
Char. 225: 1 → 0
Char. 260: 2 → 0

***Pappochelys rosinae*:**
Char. 1: 0 → 1
Char. 5: 1 → 0
Char. 41: 0 → 1
Char. 49: 0 → 1
Char. 55: 1 → 0
Char. 67: 1 → 0
Char. 176: 1 → 0
Char. 206: 0 → 2
Char. 252: 0 → 1

***Acerosodontosaurus piveteaui*:**
Char. 78: 1 → 0
Char. 81: 1 → 0
Char. 128: 0 → 1
Char. 155: 0 → 1
Char. 206: 0 → 2
Char. 208: 0 → 1
Char. 224: 1 → 0
Char. 267: 1 → 0
Char. 278: 0 → 1

***Acleistorhinus pteroticus*:**
Char. 20: 0 → 1
Char. 21: 0 → 1
Char. 30: 0 → 1
Char. 33: 0 → 1
Char. 47: 0 → 1
Char. 48: 0 → 1
Char. 55: 1 → 0
Char. 56: 0 → 1
Char. 64: 0 → 1
Char. 70: 0 → 1
Char. 79: 0 → 1
Char. 95: 0 → 1
Char. 110: 0 → 1
Char. 113: 0 → 1
Char. 114: 0 → 1
Char. 131: 0 → 1
Char. 137: 0 → 1
Char. 140: 0 → 2
Char. 146: 1 → 0
Char. 159: 0 → 1
Char. 169: 1 → 0
Char. 170: 0 → 1
Char. 278: 3 → 1

**Araeoscelidae:**
Char. 5: 1 → 0
Char. 28: 0 → 1
Char. 116: 1 → 0
Char. 166: 1 → 0
Char. 169: 1 → 0
Char. 170: 0 → 1
Char. 197: 0 → 1
Char. 239: 0 → 1

**Archosauriformes:**
Char. 32: 0 → 1
Char. 94: 1 → 0
Char. 112: 1 → 0
Char. 123: 1 → 0
Char. 152: 0 → 1
Char. 154: 0 → 2
Char. 166: 1 → 0
Char. 171: 0 → 1
Char. 185: 0 → 1
Char. 204: 0 → 1
Char. 218: 0 → 3
Char. 242: 0 → 1

***Australothyris smithi*:**
Char. 23: 0 → 1
Char. 24: 0 → 1
Char. 34: 0 → 1
Char. 55: 1 → 0
Char. 57: 1 → 0
Char. 71: 1 → 0
Char. 79: 0 → 1
Char. 85: 1 → 0
Char. 98: 1 → 0
Char. 100: 0 → 1
Char. 103: 0 → 1
Char. 110: 0 → 1
Char. 112: 0 → 1
Char. 123: 1 → 0
Char. 129: 0 → 1
Char. 131: 0 → 1
Char. 132: 1 → 0
Char. 144: 1 → 0
Char. 149: 1 → 0
Char. 150: 0 → 1

***Barasaurus besairiei*:**
Char. 33: 1 → 0
Char. 75: 1 → 0
Char. 216: 0 → 1

***Bashkyroleter bashkyricus*:**
Char. 275: 1 → 0

***Bashkyroleter mesensis*:**
Char. 169: 1 → 0

***Belebey vegrandis*:**
Char. 154: 1 → 0

***Bradysaurus* spp.:**
Char. 19: 0 → 1
Char. 73: 0 → 1
Char. 79: 0 → 1
Char. 135: 1 → 0
Char. 249: 0 → 1

***Candelaria barbouri*:**
Char. 1: 0 → 1
Char. 5: 1 → 0
Char. 8: 0 → 1
Char. 15: 0 → 1
Char. 20: 0 → 1
Char. 23: 0 → 1
Char. 25: 1 → 0
Char. 29: 1 → 0
Char. 33: 0 → 2
Char. 49: 0 → 1
Char. 50: 1 → 0
Char. 55: 1 → 0
Char. 57: 1 → 0
Char. 67: 0 → 1
Char. 79: 0 → 2
Char. 89: 0 → 1
Char. 94: 0 → 1
Char. 95: 0 → 1
Char. 126: 0 → 1
Char. 127: 0 → 1
Char. 132: 1 → 0
Char. 154: 1 → 2
Char. 169: 1 → 0
Char. 276: 0 → 1
Char. 277: 0 → 1

**Captorhinidae:**
Char. 3: 0 → 1
Char. 23: 0 → 1
Char. 25: 1 → 0
Char. 26: 0 → 1
Char. 73: 0 → 1
Char. 75: 0 → 1
Char. 108: 1 → 0
Char. 183: 1 → 0
Char. 201: 1 → 0
Char. 203: 1 → 2
Char. 216: 1 → 0

**Caseidae:**
Char. 24: 0 → 1
Char. 25: 1 → 0
Char. 36: 0 → 1
Char. 38: 1 → 0
Char. 46: 1 → 0
Char. 50: 1 → 0
Char. 56: 0 → 1
Char. 85: 1 → 0
Char. 96: 1 → 0
Char. 170: 0 → 1
Char. 194: 0 → 1
Char. 274: 0 → 1
Char. 278: 3 → 2

***Claudiosaurus germaini*:**
Char. 24: 0 → 1
Char. 34: 0 → 1
Char. 36: 0 → 1
Char. 56: 0 → 1
Char. 64: 0 → 1
Char. 73: 0 → 1
Char. 105: 0 → 1
Char. 126: 0 → 1
Char. 130: 0 → 1
Char. 131: 0 → 1
Char. 144: 1 → 0
Char. 148: 0 → 1
Char. 166: 1 → 0
Char. 182: 0 → 1
Char. 187: 1 → 0
Char. 190: 0 → 1
Char. 199: 0 → 1
Char. 203: 1 → 2
Char. 204: 0 → 1
Char. 220: 0 → 1
Char. 222: 1 → 0
Char. 224: 1 → 0
Char. 234: 1 → 0
Char. 272: 2 → 1

***Colobomycter pholeter*:**
Char. 21: 0 → 1
Char. 25: 1 → 0
Char. 84: 1 → 0
Char. 154: 1 → 0
Char. 167: 0 → 1
Char. 267: 0 → 1

***Delorhynchus cifelli*:**
Char. 18: 0 → 1
Char. 20: 0 → 1
Char. 21: 0 → 1
Char. 24: 0 → 1
Char. 26: 0 → 1
Char. 28: 0 → 1
Char. 33: 0 → 2
Char. 39: 0 → 1
Char. 48: 0 → 1
Char. 52: 0 → 1
Char. 100: 0 → 1
Char. 111: 0 → 1
Char. 116: 1 → 0
Char. 117: 0 → 1
Char. 131: 0 → 1
Char. 156: 1 → 0
Char. 159: 0 → 1
Char. 167: 0 → 1
Char. 180: 0 → 1
Char. 189: 0 → 1
Char. 191: 0 → 1
Char. 204: 0 → 2
Char. 267: 0 → 1

**Diadectomorpha:**
Char. 0: 0 → 1
Char. 70: 0 → 1
Char. 75: 0 → 1
Char. 122: 0 → 1
Char. 123: 1 → 0
Char. 146: 1 → 0
Char. 275: 1 → 0
Char. 278: 3 → 0

***Emeroleter levis*:**
Char. 0: 0 → 2
Char. 51: 1 → 0

**Eosauropterygia:**
Char. 159: 1 → 0
Char. 166: 1 → 0
Char. 168: 0 → 1
Char. 174: 0 → 1
Char. 194: 0 → 2
Char. 272: 1 → 0

***Eudibamus cursoris*:**
Char. 154: 1 → 2

***Feeserpeton oklahomensis*:**
Char. 51: 0 → 1
Char. 70: 0 → 1
Char. 157: 0 → 1
Char. 158: 0 → 1

***Hovasaurus boulei*:**
Char. 41: 0 → 1
Char. 55: 1 → 0
Char. 60: 1 → 0
Char. 72: 2 → 1
Char. 77: 0 → 2
Char. 78: 1 → 0
Char. 79: 0 → 1
Char. 93: 1 → 0
Char. 113: 0 → 1
Char. 138: 0 → 1
Char. 146: 1 → 0
Char. 154: 0 → 1
Char. 204: 0 → 2
Char. 206: 0 → 2
Char. 214: 1 → 0
Char. 215: 0 → 1

Char. 219: 1 → 0
Char. 220: 0 → 1
Char. 278: 0 → 3

**Kuehneosauridae:**
Char. 7: 0 → 1
Char. 24: 0 → 1
Char. 34: 0 → 1
Char. 35: 0 → 1
Char. 36: 0 → 1
Char. 56: 0 → 1
Char. 79: 0 → 2
Char. 82: 0 → 1
Char. 98: 1 → 0
Char. 113: 0 → 1
Char. 128: 0 → 1
Char. 147: 1 → 0
Char. 148: 1 → 0
Char. 159: 1 → 0
Char. 165: 0 → 1
Char. 168: 0 → 1
Char. 185: 0 → 1
Char. 206: 0 → 2
Char. 245: 0 → 1
Char. 272: 1 → 2
Char. 278: 0 → 3

***Lanthanosuchus watsoni*:**
Char. 25: 1 → 0
Char. 51: 0 → 1
Char. 86: 0 → 1
Char. 95: 0 → 1
Char. 98: 1 → 0
Char. 110: 0 → 1
Char. 113: 0 → 1
Char. 114: 0 → 1
Char. 131: 0 → 1
Char. 137: 0 → 1
Char. 138: 0 → 1
Char. 140: 0 → 2
Char. 144: 1 → 0
Char. 154: 1 → 2

***Macroleter poezicus*:**
Char. 0: 0 → 1
Char. 52: 0 → 1
Char. 66: 1 → 2
Char. 75: 0 → 1
Char. 84: 1 → 0
Char. 87: 0 → 1
Char. 110: 0 → 1
Char. 121: 0 → 1
Char. 140: 0 → 1
Char. 146: 1 → 0
Char. 169: 1 → 0
Char. 235: 0 → 1

***Mesosaurus* spp.:**
Char. 0: 0 → 1
Char. 2: 0 → 1
Char. 5: 1 → 0
Char. 6: 0 → 1
Char. 8: 0 → 1
Char. 9: 0 → 1

Char. 13: 0 → 1
Char. 19: 0 → 1
Char. 23: 0 → 1
Char. 26: 0 → 1
Char. 29: 1 → 0
Char. 33: 0 → 1
Char. 38: 1 → 0
Char. 41: 0 → 1
Char. 48: 0 → 1
Char. 50: 1 → 0
Char. 67: 0 → 1
Char. 84: 1 → 0
Char. 85: 1 → 0
Char. 94: 0 → 1
Char. 107: 0 → 1
Char. 109: 0 → 1
Char. 111: 0 → 1
Char. 115: 0 → 1
Char. 146: 1 → 0
Char. 148: 0 → 1
Char. 149: 1 → 0
Char. 164: 0 → 1
Char. 166: 1 → 0
Char. 167: 0 → 1
Char. 176: 0 → 1
Char. 183: 1 → 0
Char. 184: 0 → 1
Char. 199: 0 → 1
Char. 202: 0 → 1
Char. 204: 0 → 2
Char. 206: 0 → 1
Char. 207: 0 → 1
Char. 209: 0 → 1
Char. 217: 0 → 1
Char. 219: 0 → 1
Char. 220: 0 → 1
Char. 231: 0 → 1
Char. 260: 2 → 0
Char. 272: 2 → 0
Char. 278: 3 → 0

***Microleter mckinzieorum*:**
Char. 0: 0 → 1
Char. 18: 0 → 1
Char. 24: 0 → 1
Char. 25: 1 → 0
Char. 36: 0 → 1
Char. 39: 0 → 1
Char. 51: 0 → 2
Char. 56: 0 → 1
Char. 57: 1 → 0
Char. 70: 0 → 1
Char. 79: 0 → 1
Char. 94: 0 → 1
Char. 106: 0 → 1
Char. 110: 0 → 1
Char. 132: 1 → 0
Char. 159: 0 → 1
Char. 166: 1 → 0
Char. 276: 0 → 1
Char. 278: 3 → 1

**Millerettidae:**
Char. 5: 1 → 0

Char. 24: 0 → 1
Char. 25: 1 → 0
Char. 44: 1 → 0
Char. 56: 0 → 1
Char. 57: 1 → 0
Char. 66: 0 → 2
Char. 78: 1 → 0
Char. 80: 1 → 0
Char. 84: 1 → 2
Char. 96: 1 → 0
Char. 117: 0 → 1
Char. 121: 0 → 1
Char. 124: 0 → 1
Char. 127: 0 → 1
Char. 135: 0 → 1
Char. 145: 0 → 1
Char. 166: 1 → 0
Char. 202: 0 → 1
Char. 211: 0 → 1
Char. 230: 0 → 1
Char. 234: 0 → 1
Char. 248: 0 → 1
Char. 252: 0 → 1
Char. 253: 0 → 1

***Nycteroleter ineptus*:**
Char. 278: 0 → 3

***Nyctiphruretus acudens*:**
Char. 0: 0 → 1
Char. 21: 0 → 1
Char. 33: 1 → 2
Char. 41: 0 → 1
Char. 66: 1 → 2
Char. 81: 1 → 0
Char. 84: 1 → 2
Char. 94: 0 → 1
Char. 166: 1 → 0
Char. 167: 0 → 1
Char. 215: 1 → 0
Char. 224: 0 → 1
Char. 226: 1 → 0
Char. 266: 0 → 1
Char. 272: 2 → 1
Char. 276: 0 → 1

***Orovenator mayorum*:**
Char. 8: 0 → 1
Char. 24: 0 → 1
Char. 36: 0 → 1
Char. 160: 0 → 1
Char. 165: 0 → 1

***Owenetta* spp.:**
Char. 169: 1 → 0

***Paleothyris acadiana*:**
Char. 38: 1 → 0
Char. 50: 1 → 0
Char. 66: 1 → 2
Char. 102: 0 → 1
Char. 146: 1 → 0
Char. 180: 0 → 1
Char. 237: 0 → 1
Char. 239: 0 → 1

***Placodus* spp.:**
Char. 0: 1 → 2
Char. 9: 0 → 1
Char. 12: 0 → 1
Char. 13: 0 → 1
Char. 19: 0 → 1
Char. 31: 0 → 1
Char. 46: 1 → 0
Char. 57: 0 → 1
Char. 78: 1 → 0
Char. 93: 1 → 0
Char. 102: 1 → 2
Char. 109: 1 → 0
Char. 140: 1 → 0
Char. 155: 0 → 1
Char. 163: 1 → 0
Char. 164: 0 → 1

***Procolophon* spp.:**
Char. 41: 0 → 1
Char. 69: 0 → 1
Char. 79: 0 → 1
Char. 88: 0 → 1
Char. 117: 1 → 0
Char. 149: 1 → 0
Char. 180: 0 → 1
Char. 204: 0 → 1
Char. 237: 0 → 1
Char. 238: 0 → 1
Char. 272: 2 → 1
Char. 278: 3 → 0

***Prolacerta broomi*:**
Char. 58: 1 → 0
Char. 66: 1 → 0
Char. 67: 1 → 0
Char. 80: 1 → 0
Char. 139: 1 → 0
Char. 147: 1 → 0
Char. 192: 1 → 0
Char. 203: 1 → 2
Char. 206: 0 → 12
Char. 224: 1 → 0

***Rhipaeosaurus* spp.:**
Char. 172: 0 → 1
Char. 186: 1 → 0
Char. 277: 0 → 1

**Rhynchocephalia:**
Char. 0: 1 → 2
Char. 24: 0 → 1
Char. 77: 0 → 1
Char. 94: 1 → 0
Char. 117: 12 → 0
Char. 139: 1 → 0
Char. 167: 1 → 0
Char. 205: 1 → 0

**Rhynchosauria:**
Char. 0: 1 → 0
Char. 7: 0 → 1
Char. 9: 0 → 1
Char. 63: 1 → 0
Char. 68: 0 → 1

Char. 99: 1 → 0
Char. 150: 1 → 0
Char. 160: 0 → 1
Char. 161: 0 → 1
Char. 171: 0 → 2
Char. 241: 0 → 1

***Scutosaurus* spp.:**
Char. 175: 0 → 1
Char. 218: 0 → 1
Char. 243: 0 → 2
Char. 244: 0 → 1
Char. 251: 0 → 1

***Sinosaurosphargis yunguiensis*:**
Char. 8: 0 → 1
Char. 30: 0 → 1
Char. 53: 0 → 1
Char. 82: 0 → 1
Char. 89: 1 → 0
Char. 127: 1 → 0
Char. 150: 1 → 0
Char. 154: 0 → 2
Char. 167: 1 → 0
Char. 253: 0 → 1
Char. 255: 0 → 1

**Squamata:**
Char. 45: 0 → 1
Char. 79: 0 → 2
Char. 80: 1 → 0
Char. 82: 0 → 1
Char. 92: 1 → 0
Char. 109: 1 → 0
Char. 160: 0 → 1
Char. 245: 0 → 1

***Trilophosaurus buettneri*:**
Char. 5: 1 → 0
Char. 11: 0 → 1
Char. 55: 1 → 0
Char. 93: 1 → 0
Char. 104: 0 → 1
Char. 113: 0 → 1
Char. 122: 0 → 1
Char. 123: 1 → 0
Char. 136: 1 → 0
Char. 144: 1 → 0
Char. 154: 0 → 1
Char. 157: 0 → 1
Char. 159: 1 → 0
Char. 177: 0 → 12
Char. 194: 0 → 1
Char. 203: 1 → 2
Char. 207: 1 → 0
Char. 208: 1 → 0
Char. 224: 1 → 0
Char. 272: 1 → 0

***Youngina capensis*:**
Char. 5: 1 → 0
Char. 23: 1 → 0
Char. 56: 0 → 1
Char. 75: 0 → 1

Char. 92: 1 → 0
Char. 94: 1 → 0
Char. 117: 12 → 0
Char. 163: 1 → 0
Char. 170: 0 → 1
Char. 211: 0 → 1
Char. 215: 0 → 1
Char. 231: 0 → 1
Char. 239: 0 → 1

**Node 50:**
Char. 61: 1 → 0
Char. 62: 1 → 0
Char. 65: 0 → 1
Char. 78: 1 → 0
Char. 193: 1 → 0
Char. 194: 0 → 2
Char. 214: 1 → 0
Char. 219: 1 → 0
Char. 220: 0 → 1
Char. 241: 0 → 1
Char. 246: 0 → 1
Char. 247: 0 → 1
Char. 254: 0 → 1
Char. 255: 0 → 1
Char. 256: 0 → 1
Char. 268: 0 → 1
Char. 269: 0 → 1

**Node 51:**
Char. 58: 0 → 1
Char. 61: 0 → 1
Char. 63: 0 → 1
Char. 66: 0 → 1
Char. 69: 0 → 1
Char. 73: 0 → 1
Char. 84: 0 → 1
Char. 126: 0 → 1
Char. 131: 0 → 1
Char. 145: 0 → 1
Char. 147: 0 → 1
Char. 148: 0 → 1
Char. 150: 0 → 1
Char. 151: 0 → 1
Char. 167: 0 → 1
Char. 176: 0 → 1
Char. 184: 0 → 1
Char. 205: 0 → 1
Char. 208: 0 → 1
Char. 210: 0 → 1
Char. 217: 0 → 1
Char. 230: 0 → 1
Char. 231: 0 → 1
Char. 232: 0 → 1
Char. 272: 2 → 1

**Node 52:**
Char. 33: 0 → 1
Char. 92: 0 → 1
Char. 94: 0 → 1
Char. 135: 0 → 1
Char. 159: 0 → 1
Char. 267: 0 → 1
Char. 278: 1 → 0

**Node 53:**
Char. 20: 0 → 1
Char. 62: 0 → 1

**Node 54:**
Char. 0: 0 → 1
Char. 27: 0 → 1
Char. 29: 1 → 0
Char. 38: 1 → 0
Char. 40: 2 → 0
Char. 57: 1 → 0
Char. 59: 0 → 1
Char. 60: 0 → 1
Char. 67: 0 → 1
Char. 72: 1 → 2
Char. 84: 1 → 0
Char. 89: 0 → 1
Char. 111: 0 → 1
Char. 112: 0 → 1
Char. 117: 0 → 12
Char. 120: 0 → 1
Char. 154: 1 → 0
Char. 180: 0 → 1
Char. 193: 0 → 1
Char. 222: 0 → 1
Char. 224: 0 → 1
Char. 234: 0 → 1
Char. 237: 0 → 1
Char. 266: 0 → 1
Char. 278: 3 → 1

**Node 55:**
Char. 5: 0 → 1
Char. 29: 0 → 1
Char. 40: 0 → 2
Char. 74: 0 → 1
Char. 80: 0 → 1
Char. 116: 0 → 1
Char. 149: 0 → 1
Char. 166: 0 → 1
Char. 169: 0 → 1
Char. 201: 0 → 1
Char. 203: 0 → 1
Char. 235: 0 → 1
Char. 276: 1 → 0

**Node 56:**
Char. 81: 0 → 1
Char. 93: 0 → 1
Char. 97: 0 → 1

**Node 57:**
Char. 55: 0 → 1
Char. 111: 1 → 0
Char. 135: 1 → 0
Char. 207: 1 → 0

**Node 58:**
Char. 0: 2 → 0
Char. 5: 1 → 0
Char. 33: 2 → 0
Char. 43: 1 → 0
Char. 124: 1 → 0
Char. 127: 1 → 0
Char. 145: 1 → 0
Char. 147: 1 → 0
Char. 149: 1 → 0
Char. 169: 1 → 0
Char. 174: 1 → 0
Char. 180: 1 → 0
Char. 188: 1 → 0
Char. 220: 1 → 0
Char. 221: 1 → 0
Char. 231: 1 → 0
Char. 247: 1 → 0
Char. 251: 1 → 0
Char. 252: 1 → 0
Char. 253: 2 → 0

**Node 59:**
Char. 19: 0 → 1
Char. 92: 1 → 0

**Node 60:**
Char. 4: 0 → 1
Char. 29: 0 → 1
Char. 48: 1 → 0
Char. 107: 0 → 1
Char. 140: 0 → 1
Char. 210: 1 → 0
Char. 213: 0 → 1
Char. 226: 0 → 2
Char. 228: 0 → 1
Char. 275: 0 → 1

**Node 61:**
Char. 73: 0 → 1
Char. 131: 1 → 0
Char. 205: 0 → 1
Char. 239: 0 → 1
Char. 276: 0 → 1

**Node 62:**
Char. 18: 0 → 1
Char. 37: 0 → 1
Char. 48: 0 → 1
Char. 107: 0 → 1
Char. 118: 1 → 0
Char. 125: 1 → 0
Char. 126: 0 → 1
Char. 132: 1 → 0
Char. 150: 0 → 1

**Node 63:**
Char. 23: 0 → 1
Char. 71: 1 → 0
Char. 75: 0 → 1
Char. 102: 0 → 1
Char. 103: 0 → 1
Char. 106: 0 → 1
Char. 110: 0 → 1
Char. 155: 0 → 1
Char. 167: 0 → 1
Char. 207: 0 → 1
Char. 214: 0 → 1
Char. 216: 1 → 0
Char. 235: 0 → 2
Char. 241: 0 → 1

**Node 64:**
Char. 20: 0 → 1
Char. 33: 0 → 1
Char. 38: 1 → 2
Char. 39: 0 → 1
Char. 44: 1 → 0
Char. 49: 0 → 1
Char. 51: 0 → 1
Char. 66: 0 → 1
Char. 70: 0 → 1
Char. 80: 1 → 0
Char. 95: 0 → 1
Char. 112: 0 → 1
Char. 117: 0 → 1
Char. 118: 0 → 1
Char. 125: 0 → 1
Char. 131: 0 → 1
Char. 135: 0 → 1
Char. 137: 0 → 1
Char. 148: 0 → 1
Char. 157: 0 → 1
Char. 158: 0 → 1
Char. 159: 0 → 1
Char. 183: 1 → 2
Char. 186: 0 → 1
Char. 194: 0 → 1
Char. 197: 0 → 1
Char. 201: 1 → 0
Char. 211: 0 → 1
Char. 215: 0 → 1
Char. 226: 0 → 1
Char. 235: 1 → 0
Char. 252: 0 → 1

**Node 65:**
Char. 87: 0 → 1
Char. 110: 0 → 1

**Node 66:**
Char. 25: 1 → 0
Char. 76: 1 → 0

**Node 67:**
Char. 79: 0 → 1
Char. 86: 0 → 1
Char. 93: 1 → 0
Char. 133: 0 → 1

**Node 68:**
Char. 48: 0 → 1
Char. 100: 0 → 1
Char. 113: 0 → 1
Char. 132: 1 → 0
Char. 147: 1 → 2
Char. 154: 1 → 0
Char. 207: 0 → 1
Char. 239: 0 → 1
Char. 278: 3 → 0

**Node 69:**
Char. 38: 1 → 2
Char. 39: 0 → 1
Char. 50: 1 → 0
Char. 58: 0 → 1
Char. 59: 0 → 1
Char. 60: 0 → 1
Char. 72: 1 → 2
Char. 85: 1 → 0
Char. 95: 0 → 1
Char. 104: 0 → 1
Char. 105: 0 → 1
Char. 106: 0 → 2
Char. 107: 0 → 1
Char. 109: 0 → 1
Char. 110: 0 → 1
Char. 146: 1 → 0
Char. 148: 0 → 1
Char. 155: 0 → 2
Char. 183: 1 → 2

**Node 70:**
Char. 0: 0 → 2
Char. 33: 1 → 0
Char. 38: 2 → 1
Char. 39: 1 → 0
Char. 42: 0 → 1
Char. 43: 0 → 1
Char. 46: 1 → 0
Char. 49: 1 → 0
Char. 52: 0 → 1
Char. 83: 01 → 2
Char. 84: 1 → 0
Char. 87: 0 → 1
Char. 93: 1 → 0
Char. 100: 0 → 1
Char. 113: 0 → 1
Char. 143: 0 → 1
Char. 161: 0 → 1
Char. 163: 1 → 0
Char. 172: 0 → 2
Char. 174: 0 → 1
Char. 188: 0 → 1
Char. 189: 0 → 1
Char. 195: 0 → 1
Char. 204: 0 → 2
Char. 212: 0 → 1
Char. 236: 0 → 1
Char. 238: 0 → 2
Char. 242: 0 → 1
Char. 245: 0 → 1
Char. 274: 0 → 1
Char. 275: 1 → 0

**Node 71:**
Char. 5: 1 → 0
Char. 29: 1 → 0
Char. 44: 1 → 0
Char. 59: 0 → 1
Char. 60: 0 → 1
Char. 66: 0 → 1
Char. 67: 0 → 1
Char. 72: 1 → 2
Char. 78: 1 → 0
Char. 84: 1 → 0
Char. 93: 1 → 0
Char. 111: 0 → 1
Char. 123: 1 → 0
Char. 129: 0 → 1
Char. 154: 1 → 0

Char. 169: 1 → 0
Char. 170: 0 → 1
Char. 197: 0 → 1

**Node 72:**
Char. 20: 0 → 1
Char. 47: 0 → 1
Char. 85: 1 → 0
Char. 107: 0 → 1
Char. 110: 0 → 1
Char. 169: 1 → 0
Char. 170: 0 → 1

**Node 73:**
Char. 2: 0 → 1
Char. 6: 0 → 1
Char. 98: 1 → 0
Char. 101: 0 → 1
Char. 113: 0 → 1
Char. 182: 0 → 1
Char. 186: 0 → 1
Char. 193: 1 → 0
Char. 198: 0 → 1
Char. 206: 0 → 2
Char. 220: 0 → 1
Char. 224: 1 → 0
Char. 231: 1 → 0
Char. 232: 1 → 0
Char. 234: 1 → 0
Char. 240: 1 → 0
Char. 266: 1 → 0

**Node 74:**
Char. 33: 1 → 2
Char. 35: 0 → 1
Char. 68: 0 → 1
Char. 102: 0 → 1
Char. 103: 0 → 1
Char. 107: 0 → 1
Char. 123: 1 → 0
Char. 140: 0 → 1
Char. 229: 0 → 1
Char. 235: 1 → 2

**Node 75:**
Char. 17: 0 → 1
Char. 42: 0 → 1
Char. 251: 0 → 1
Char. 264: 0 → 1

**Node 76:**
Char. 41: 0 → 1
Char. 48: 1 → 0
Char. 61: 1 → 2
Char. 112: 1 → 0
Char. 128: 0 → 1
Char. 138: 0 → 1
Char. 146: 1 → 0
Char. 155: 0 → 1
Char. 165: 0 → 1
Char. 192: 1 → 2
Char. 226: 0 → 1
Char. 227: 0 → 1
Char. 233: 0 → 1

**Node 77:**
Char. 61: 1 → 3
Char. 90: 0 → 1
Char. 91: 0 → 1
Char. 109: 1 → 0
Char. 155: 0 → 1
Char. 209: 1 → 0

## Analysis 17
### (no *Proganochelys quenstedti*, *Candelaria barbouri*, and *Pappochelys rosinae*)

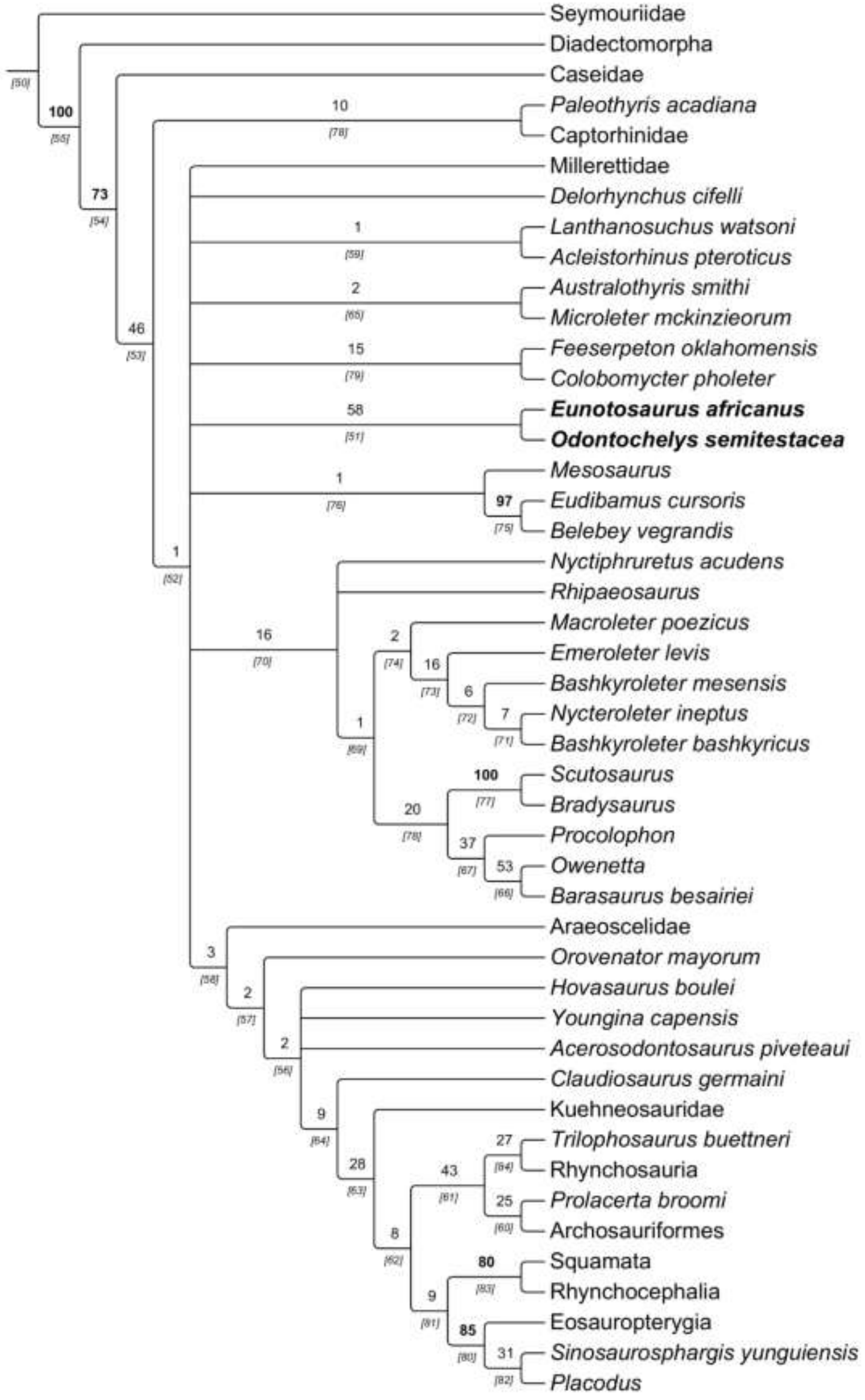

**Seymouriidae:**
Char. 51: 0 → 2
Char. 54: 1 → 0
Char. 85: 1 → 0
Char. 99: 1 → 0
Char. 107: 0 → 1
Char. 140: 0 → 2
Char. 207: 0 → 1
Char. 225: 1 → 0

***Odontochelys semitestacea*:**
Char. 46: 1 → 0
Char. 65: 0 → 1
Char. 88: 1 → 0
Char. 93: 1 → 0
Char. 176: 0 → 1
Char. 184: 0 → 1
Char. 191: 0 → 1
Char. 195: 0 → 2
Char. 198: 0 → 1
Char. 202: 0 → 1
Char. 205: 0 → 1
Char. 210: 0 → 1
Char. 222: 0 → 1
Char. 223: 0 → 1
Char. 224: 0 → 1
Char. 232: 0 → 1
Char. 235: 1 → 2
Char. 237: 0 → 1
Char. 238: 0 → 1
Char. 241: 0 → 1
Char. 246: 0 → 2
Char. 254: 0 → 1
Char. 255: 0 → 1
Char. 256: 0 → 1
Char. 258: 0 → 1
Char. 259: 0 → 1
Char. 267: 0 → 1
Char. 268: 0 → 1
Char. 269: 0 → 1
Char. 270: 0 → 1
Char. 272: 2 → 1

***Eunotosaurus africanus*:**
Char. 19: 0 → 1
Char. 89: 0 → 1
Char. 192: 1 → 0
Char. 219: 0 → 1
Char. 248: 0 → 1
Char. 250: 0 → 1
Char. 263: 0 → 1

***Acerosodontosaurus piveteaui*:**
Char. 78: 1 → 0
Char. 81: 1 → 0
Char. 128: 0 → 1
Char. 155: 0 → 1
Char. 206: 0 → 2
Char. 208: 0 → 1

***Acleistorhinus pteroticus*:**
Char. 21: 0 → 1
Char. 146: 1 → 0
Char. 169: 1 → 0
Char. 170: 0 → 1

**Araeoscelidae:**
Char. 5: 1 → 0
Char. 20: 1 → 0
Char. 28: 0 → 1
Char. 106: 0 → 1
Char. 116: 1 → 0
Char. 166: 1 → 0
Char. 169: 1 → 0
Char. 170: 0 → 1
Char. 197: 0 → 1
Char. 221: 1 → 0
Char. 239: 0 → 1

**Archosauriformes:**
Char. 32: 0 → 1
Char. 94: 1 → 0
Char. 112: 1 → 0
Char. 152: 0 → 1
Char. 154: 0 → 2
Char. 166: 1 → 0
Char. 171: 0 → 1
Char. 185: 0 → 1
Char. 204: 0 → 1
Char. 218: 0 → 3
Char. 242: 0 → 1

***Australothyris smithi*:**
Char. 23: 0 → 1
Char. 34: 0 → 1
Char. 55: 1 → 0
Char. 71: 1 → 0
Char. 85: 1 → 0
Char. 112: 0 → 1
Char. 129: 0 → 1
Char. 131: 0 → 1
Char. 159: 1 → 0

***Barasaurus besairiei*:**
Char. 33: 1 → 0
Char. 75: 1 → 0
Char. 216: 0 → 1

***Bashkyroleter bashkyricus*:**
Char. 275: 1 → 0

***Bashkyroleter mesensis*:**
Char. 169: 1 → 0

***Belebey vegrandis*:**
Char. 154: 1 → 0

***Bradysaurus* spp.:**
Char. 19: 0 → 1
Char. 73: 0 → 1
Char. 79: 0 → 1
Char. 135: 1 → 0
Char. 249: 0 → 1

**Captorhinidae:**
Char. 3: 0 → 1
Char. 23: 0 → 1
Char. 25: 1 → 0
Char. 26: 0 → 1
Char. 73: 0 → 1
Char. 75: 0 → 1
Char. 83: 0 → 1
Char. 108: 1 → 0
Char. 183: 1 → 0
Char. 203: 1 → 2

**Caseidae:**
Char. 24: 0 → 1
Char. 25: 1 → 0
Char. 36: 0 → 1
Char. 38: 1 → 0
Char. 46: 1 → 0
Char. 56: 0 → 1
Char. 85: 1 → 0
Char. 96: 1 → 0
Char. 98: 1 → 0
Char. 194: 0 → 1
Char. 273: 0 → 1
Char. 274: 0 → 1

***Claudiosaurus germaini*:**
Char. 64: 0 → 1
Char. 105: 0 → 1
Char. 130: 0 → 1
Char. 144: 1 → 0
Char. 166: 1 → 0
Char. 187: 1 → 0
Char. 199: 0 → 1
Char. 203: 1 → 2
Char. 204: 0 → 1
Char. 220: 0 → 1
Char. 222: 1 → 0

***Colobomycter pholeter*:**
Char. 21: 0 → 1
Char. 25: 1 → 0
Char. 84: 1 → 0
Char. 154: 1 → 0
Char. 167: 0 → 1
Char. 267: 0 → 1

***Delorhynchus cifelli*:**
Char. 18: 0 → 1
Char. 21: 0 → 1
Char. 24: 0 → 1
Char. 26: 0 → 1
Char. 28: 0 → 1
Char. 33: 0 → 2
Char. 39: 0 → 1
Char. 52: 0 → 1
Char. 100: 0 → 1
Char. 111: 0 → 1
Char. 116: 1 → 0
Char. 117: 0 → 1
Char. 119: 1 → 0
Char. 131: 0 → 1
Char. 156: 1 → 0
Char. 167: 0 → 1
Char. 189: 0 → 1
Char. 191: 0 → 1
Char. 267: 0 → 1

**Diadectomorpha:**
Char. 0: 0 → 1
Char. 44: 0 → 1
Char. 70: 0 → 1
Char. 75: 0 → 1
Char. 122: 0 → 1
Char. 146: 1 → 0
Char. 275: 1 → 0

***Emeroleter levis*:**
Char. 0: 0 → 2
Char. 51: 1 → 0

**Eosauropterygia:**
Char. 159: 1 → 0
Char. 166: 1 → 0
Char. 174: 0 → 1
Char. 194: 0 → 2
Char. 272: 1 → 0

***Eudibamus cursoris*:**
Char. 154: 1 → 2

***Feeserpeton oklahomensis*:**
Char. 51: 0 → 1
Char. 70: 0 → 1
Char. 157: 0 → 1
Char. 158: 0 → 1

***Hovasaurus boulei*:**
Char. 41: 0 → 1
Char. 55: 1 → 0
Char. 60: 1 → 0
Char. 72: 2 → 1
Char. 77: 0 → 2
Char. 78: 1 → 0
Char. 79: 0 → 1
Char. 93: 1 → 0
Char. 113: 0 → 1
Char. 138: 0 → 1
Char. 146: 1 → 0
Char. 154: 0 → 1
Char. 204: 0 → 2
Char. 206: 0 → 2
Char. 220: 0 → 1
Char. 278: 01 → 3

**Kuehneosauridae:**
Char. 7: 0 → 1
Char. 26: 0 → 1
Char. 44: 1 → 0
Char. 79: 0 → 2
Char. 82: 0 → 1
Char. 98: 1 → 0
Char. 108: 1 → 0
Char. 113: 0 → 1
Char. 128: 0 → 1
Char. 159: 1 → 0
Char. 181: 0 → 1
Char. 185: 0 → 1
Char. 206: 0 → 2
Char. 245: 0 → 1
Char. 278: 0 → 3

***Lanthanosuchus watsoni*:**
Char. 25: 1 → 0
Char. 51: 0 → 1
Char. 86: 0 → 1
Char. 98: 1 → 0
Char. 119: 1 → 0
Char. 138: 0 → 1
Char. 144: 1 → 0
Char. 154: 1 → 2

***Macroleter poezicus*:**
Char. 0: 0 → 1
Char. 52: 0 → 1
Char. 66: 1 → 2
Char. 84: 1 → 0
Char. 87: 0 → 1
Char. 140: 0 → 1
Char. 146: 1 → 0
Char. 169: 1 → 0
Char. 204: 2 → 0
Char. 235: 0 → 1

***Mesosaurus* spp.:**
Char. 2: 0 → 1
Char. 5: 1 → 0
Char. 6: 0 → 1
Char. 8: 0 → 1
Char. 9: 0 → 1
Char. 13: 0 → 1
Char. 19: 0 → 1
Char. 29: 1 → 0
Char. 33: 0 → 1
Char. 41: 0 → 1
Char. 84: 1 → 0
Char. 94: 0 → 1
Char. 111: 0 → 1
Char. 115: 0 → 1
Char. 149: 1 → 0
Char. 159: 1 → 0
Char. 164: 0 → 1
Char. 166: 1 → 0
Char. 167: 0 → 1
Char. 192: 1 → 0
Char. 231: 0 → 1
Char. 234: 1 → 0
Char. 272: 2 → 0

***Microleter mckinzieorum*:**
Char. 0: 0 → 1
Char. 25: 1 → 0
Char. 36: 0 → 1
Char. 39: 0 → 1
Char. 51: 0 → 2
Char. 56: 0 → 1
Char. 70: 0 → 1
Char. 94: 0 → 1
Char. 106: 0 → 1
Char. 166: 1 → 0
Char. 276: 0 → 1

**Millerettidae:**
Char. 5: 1 → 0
Char. 20: 1 → 0
Char. 24: 0 → 1

Char. 25: 1 → 0
Char. 44: 1 → 0
Char. 56: 0 → 1
Char. 57: 1 → 0
Char. 66: 0 → 2
Char. 78: 1 → 0
Char. 80: 1 → 0
Char. 84: 1 → 2
Char. 88: 1 → 0
Char. 96: 1 → 0
Char. 117: 0 → 1
Char. 121: 0 → 1
Char. 124: 0 → 1
Char. 127: 0 → 1
Char. 135: 0 → 1
Char. 145: 0 → 1
Char. 147: 1 → 0
Char. 159: 1 → 0
Char. 163: 1 → 0
Char. 166: 1 → 0
Char. 192: 1 → 0
Char. 202: 0 → 1
Char. 230: 0 → 1
Char. 240: 0 → 1
Char. 248: 0 → 1
Char. 253: 0 → 1

***Nycteroleter ineptus*:**
Char. 278: 0 → 3

***Nyctiphruretus acudens*:**
Char. 0: 0 → 1
Char. 21: 0 → 1
Char. 33: 1 → 2
Char. 41: 0 → 1
Char. 66: 1 → 2
Char. 81: 1 → 0
Char. 83: 0 → 1
Char. 84: 1 → 2
Char. 94: 0 → 1
Char. 166: 1 → 0
Char. 167: 0 → 1
Char. 224: 0 → 1
Char. 266: 0 → 1
Char. 272: 2 → 1
Char. 276: 0 → 1

***Orovenator mayorum*:**
Char. 8: 0 → 1
Char. 24: 0 → 1
Char. 36: 0 → 1
Char. 160: 0 → 1
Char. 165: 0 → 1

***Owenetta* spp.:**
Char. 169: 1 → 0

***Paleothyris acadiana*:**
Char. 38: 1 → 0
Char. 66: 1 → 2
Char. 102: 0 → 1
Char. 146: 1 → 0
Char. 237: 0 → 1
Char. 239: 0 → 1
Char. 240: 0 → 1

***Placodus* spp.:**
Char. 0: 1 → 2
Char. 9: 0 → 1
Char. 12: 0 → 1
Char. 13: 0 → 1
Char. 19: 0 → 1
Char. 26: 0 → 1
Char. 31: 0 → 1
Char. 44: 1 → 0
Char. 46: 1 → 0
Char. 57: 0 → 1
Char. 78: 1 → 0
Char. 93: 1 → 0
Char. 102: 1 → 2
Char. 109: 1 → 0
Char. 140: 1 → 0
Char. 155: 0 → 1
Char. 163: 1 → 0
Char. 164: 0 → 1

***Procolophon* spp.:**
Char. 41: 0 → 1
Char. 69: 0 → 1
Char. 79: 0 → 1
Char. 83: 0 → 1
Char. 88: 0 → 1
Char. 117: 1 → 0
Char. 149: 1 → 0
Char. 237: 0 → 1
Char. 238: 0 → 1
Char. 272: 2 → 1
Char. 278: 3 → 0

***Prolacerta broomi*:**
Char. 58: 1 → 0
Char. 66: 1 → 0
Char. 67: 1 → 0
Char. 80: 1 → 0
Char. 139: 1 → 0
Char. 147: 1 → 0
Char. 192: 1 → 0
Char. 203: 1 → 2
Char. 206: 0 → 12

***Rhipaeosaurus* spp.:**
Char. 172: 0 → 1
Char. 277: 0 → 1

**Rhynchocephalia:**
Char. 0: 1 → 2
Char. 24: 0 → 1
Char. 75: 1 → 0
Char. 77: 0 → 1
Char. 94: 1 → 0
Char. 117: 12 → 0
Char. 139: 1 → 0
Char. 167: 1 → 0
Char. 205: 1 → 0

**Rhynchosauria:**
Char. 0: 1 → 0
Char. 7: 0 → 1
Char. 9: 0 → 1
Char. 26: 0 → 1
Char. 44: 1 → 0

Char. 68: 0 → 1
Char. 99: 1 → 0
Char. 150: 1 → 0
Char. 160: 0 → 1
Char. 161: 0 → 1
Char. 171: 0 → 2
Char. 182: 1 → 0
Char. 223: 0 → 1
Char. 241: 0 → 1

***Scutosaurus* spp.:**
Char. 175: 0 → 1
Char. 218: 0 → 1
Char. 243: 0 → 2
Char. 244: 0 → 1
Char. 251: 0 → 1

***Sinosaurosphargis yunguiensis*:**
Char. 8: 0 → 1
Char. 30: 0 → 1
Char. 53: 0 → 1
Char. 82: 0 → 1
Char. 89: 1 → 0
Char. 127: 1 → 0
Char. 150: 1 → 0
Char. 154: 0 → 2
Char. 167: 1 → 0
Char. 253: 0 → 1
Char. 255: 0 → 1

**Squamata:**
Char. 26: 0 → 1
Char. 45: 0 → 1
Char. 79: 0 → 2
Char. 80: 1 → 0
Char. 82: 0 → 1
Char. 92: 1 → 0
Char. 109: 1 → 0
Char. 160: 0 → 1
Char. 245: 0 → 1

***Trilophosaurus buettneri*:**
Char. 5: 1 → 0
Char. 11: 0 → 1
Char. 55: 1 → 0
Char. 93: 1 → 0
Char. 104: 0 → 1
Char. 113: 0 → 1
Char. 122: 0 → 1
Char. 136: 1 → 0
Char. 144: 1 → 0
Char. 154: 0 → 1
Char. 157: 0 → 1
Char. 159: 1 → 0
Char. 177: 0 → 12
Char. 194: 0 → 1
Char. 203: 1 → 2
Char. 207: 1 → 0
Char. 208: 1 → 0
Char. 272: 1 → 0

***Youngina capensis*:**
Char. 5: 1 → 0
Char. 21: 0 → 1

Char. 44: 1 → 0
Char. 75: 0 → 1
Char. 92: 1 → 0
Char. 163: 1 → 0
Char. 170: 0 → 1
Char. 211: 0 → 1
Char. 231: 0 → 1
Char. 239: 0 → 1

**Node 50:**
Char. 8: 1 → 0
Char. 15: 1 → 0
Char. 20: 1 → 0
Char. 25: 0 → 1
Char. 33: 2 → 0
Char. 72: 2 → 0
Char. 73: 1 → 0
Char. 79: 2 → 1
Char. 84: 1 → 0
Char. 94: 1 → 0
Char. 127: 1 → 0
Char. 131: 1 → 0
Char. 184: 1 → 0
Char. 187: 1 → 0
Char. 192: 1 → 0
Char. 194: 2 → 0
Char. 205: 1 → 0
Char. 210: 1 → 0
Char. 217: 1 → 0
Char. 220: 1 → 0
Char. 221: 1 → 0
Char. 222: 1 → 0
Char. 241: 1 → 0
Char. 246: 1 → 0
Char. 247: 1 → 0
Char. 254: 1 → 0
Char. 255: 1 → 0
Char. 256: 1 → 0
Char. 266: 1 → 0
Char. 268: 1 → 0
Char. 269: 1 → 0

**Node 51:**
Char. 0: 0 → 2
Char. 33: 0 → 2
Char. 43: 0 → 1
Char. 111: 0 → 1
Char. 124: 0 → 1
Char. 127: 0 → 1
Char. 145: 0 → 1
Char. 148: 0 → 1
Char. 174: 0 → 1
Char. 188: 0 → 1
Char. 220: 0 → 1
Char. 231: 0 → 1
Char. 247: 0 → 1
Char. 251: 0 → 1
Char. 253: 0 → 2

**Node 52:**
Char. 5: 0 → 1
Char. 20: 0 → 1
Char. 29: 0 → 1
Char. 44: 0 → 1

Char. 84: 0 → 1
Char. 88: 0 → 1
Char. 116: 0 → 1
Char. 121: 1 → 0
Char. 147: 0 → 1
Char. 154: 0 → 1
Char. 159: 0 → 1
Char. 163: 0 → 1
Char. 166: 0 → 1
Char. 169: 0 → 1
Char. 192: 0 → 1
Char. 204: 0 → 2
Char. 221: 0 → 1
Char. 234: 0 → 1

**Node 53:**
Char. 74: 0 → 1
Char. 119: 0 → 1
Char. 132: 0 → 1
Char. 149: 0 → 1
Char. 203: 0 → 1
Char. 235: 0 → 1

**Node 54:**
Char. 72: 0 → 1
Char. 79: 1 → 0

**Node 55:**
Char. 23: 1 → 0
Char. 55: 0 → 1
Char. 78: 0 → 1
Char. 111: 1 → 0
Char. 126: 1 → 0
Char. 135: 1 → 0
Char. 154: 12 → 0

**Node 56:**
Char. 33: 0 → 1
Char. 135: 0 → 1

**Node 57:**
Char. 62: 0 → 1

**Node 58:**
Char. 0: 0 → 1
Char. 27: 0 → 1
Char. 29: 1 → 0
Char. 38: 1 → 0
Char. 40: 2 → 0
Char. 57: 1 → 0
Char. 59: 0 → 1
Char. 60: 0 → 1
Char. 67: 0 → 1
Char. 72: 1 → 2
Char. 84: 1 → 0
Char. 89: 0 → 1
Char. 111: 0 → 1
Char. 112: 0 → 1
Char. 120: 0 → 1
Char. 147: 1 → 0
Char. 154: 1 → 0
Char. 193: 0 → 1
Char. 204: 2 → 0
Char. 222: 0 → 1
Char. 237: 0 → 1

Char. 240: 0 → 1
Char. 266: 0 → 1
Char. 278: 3 → 1

**Node 59:**
Char. 95: 0 → 1
Char. 110: 0 → 1
Char. 113: 0 → 1
Char. 114: 0 → 1
Char. 131: 0 → 1
Char. 137: 0 → 1
Char. 140: 0 → 2

**Node 60:**
Char. 19: 0 → 1
Char. 92: 1 → 0

**Node 61:**
Char. 4: 0 → 1
Char. 15: 0 → 1
Char. 29: 0 → 1
Char. 213: 0 → 1
Char. 226: 0 → 2
Char. 228: 0 → 1
Char. 275: 0 → 1

**Node 62:**
Char. 27: 1 → 0
Char. 75: 0 → 1
Char. 107: 0 → 1
Char. 140: 0 → 1
Char. 147: 0 → 1

**Node 63:**
Char. 58: 0 → 1
Char. 61: 0 → 1
Char. 66: 0 → 1
Char. 69: 0 → 1
Char. 150: 0 → 1
Char. 167: 0 → 1
Char. 205: 0 → 1
Char. 208: 0 → 1
Char. 239: 0 → 1

**Node 64:**
Char. 70: 0 → 1
Char. 73: 0 → 1
Char. 126: 0 → 1
Char. 131: 0 → 1
Char. 182: 0 → 1
Char. 190: 0 → 1

**Node 65:**
Char. 24: 0 → 1
Char. 57: 1 → 0
Char. 79: 0 → 1
Char. 83: 0 → 1
Char. 110: 0 → 1
Char. 132: 1 → 0

**Node 66:**
Char. 73: 0 → 1
Char. 131: 1 → 0
Char. 205: 0 → 1
Char. 239: 0 → 1
Char. 276: 0 → 1

**Node 67:**
Char. 18: 0 → 1
Char. 37: 0 → 1
Char. 107: 0 → 1
Char. 118: 1 → 0
Char. 125: 1 → 0
Char. 150: 0 → 1

**Node 68:**
Char. 23: 0 → 1
Char. 71: 1 → 0
Char. 102: 0 → 1
Char. 103: 0 → 1
Char. 106: 0 → 1
Char. 167: 0 → 1
Char. 214: 0 → 1
Char. 216: 1 → 0
Char. 235: 0 → 2
Char. 241: 0 → 1

**Node 69:**
Char. 138: 0 → 1
Char. 141: 0 → 1

**Node 70:**
Char. 33: 0 → 1
Char. 38: 1 → 2
Char. 39: 0 → 1
Char. 44: 1 → 0
Char. 49: 0 → 1
Char. 51: 0 → 1
Char. 66: 0 → 1
Char. 70: 0 → 1
Char. 80: 1 → 0
Char. 88: 1 → 0
Char. 95: 0 → 1
Char. 112: 0 → 1
Char. 117: 0 → 1
Char. 118: 0 → 1
Char. 125: 0 → 1
Char. 131: 0 → 1
Char. 137: 0 → 1
Char. 148: 0 → 1
Char. 158: 0 → 1
Char. 183: 1 → 2
Char. 194: 0 → 1
Char. 234: 1 → 0
Char. 235: 1 → 0

**Node 71:**
Char. 87: 0 → 1

**Node 72:**
Char. 25: 1 → 0
Char. 76: 1 → 0

**Node 73:**
Char. 79: 0 → 1
Char. 93: 1 → 0
Char. 133: 0 → 1

**Node 74:**
Char. 147: 1 → 2
Char. 154: 1 → 0
Char. 239: 0 → 1
Char. 278: 3 → 0

**Node 75:**
Char. 39: 0 → 1
Char. 58: 0 → 1
Char. 59: 0 → 1
Char. 60: 0 → 1
Char. 72: 1 → 2
Char. 88: 1 → 0
Char. 95: 0 → 1
Char. 104: 0 → 1
Char. 105: 0 → 1
Char. 106: 0 → 2
Char. 110: 0 → 1
Char. 155: 0 → 2

**Node 76:**
Char. 0: 0 → 1
Char. 20: 1 → 0
Char. 23: 0 → 1
Char. 26: 0 → 1
Char. 50: 1 → 0
Char. 67: 0 → 1
Char. 83: 0 → 1
Char. 85: 1 → 0
Char. 107: 0 → 1
Char. 109: 0 → 1
Char. 146: 1 → 0
Char. 147: 1 → 0
Char. 148: 0 → 1
Char. 199: 0 → 1

**Node 77:**
Char. 0: 0 → 2
Char. 33: 1 → 0
Char. 38: 2 → 1
Char. 39: 1 → 0
Char. 42: 0 → 1
Char. 43: 0 → 1
Char. 46: 1 → 0
Char. 49: 1 → 0
Char. 52: 0 → 1
Char. 83: 0 → 2
Char. 84: 1 → 0
Char. 87: 0 → 1
Char. 93: 1 → 0
Char. 161: 0 → 1
Char. 163: 1 → 0
Char. 172: 0 → 2
Char. 174: 0 → 1
Char. 188: 0 → 1
Char. 189: 0 → 1
Char. 195: 0 → 1
Char. 212: 0 → 1
Char. 221: 1 → 0
Char. 236: 0 → 1
Char. 238: 0 → 2
Char. 242: 0 → 1
Char. 245: 0 → 1
Char. 274: 0 → 1
Char. 275: 1 → 0

**Node 78:**
Char. 59: 0 → 1
Char. 60: 0 → 1
Char. 66: 0 → 1
Char. 67: 0 → 1
Char. 72: 1 → 2
Char. 78: 1 → 0
Char. 111: 0 → 1
Char. 129: 0 → 1
Char. 197: 0 → 1

**Node 79:**
Char. 47: 0 → 1
Char. 83: 0 → 1
Char. 85: 1 → 0
Char. 107: 0 → 1
Char. 110: 0 → 1
Char. 169: 1 → 0
Char. 170: 0 → 1

**Node 80:**
Char. 2: 0 → 1
Char. 6: 0 → 1
Char. 98: 1 → 0
Char. 101: 0 → 1
Char. 113: 0 → 1
Char. 181: 0 → 1
Char. 186: 0 → 1
Char. 193: 1 → 0
Char. 198: 0 → 1
Char. 206: 0 → 2
Char. 220: 0 → 1
Char. 239: 1 → 0
Char. 240: 1 → 0
Char. 266: 1 → 0

**Node 81:**
Char. 33: 1 → 2
Char. 68: 0 → 1
Char. 102: 0 → 1
Char. 103: 0 → 1
Char. 223: 0 → 1
Char. 229: 0 → 1
Char. 235: 1 → 2

**Node 82:**
Char. 17: 0 → 1
Char. 42: 0 → 1
Char. 251: 0 → 1
Char. 264: 0 → 1

**Node 83:**
Char. 41: 0 → 1
Char. 61: 1 → 2
Char. 112: 1 → 0
Char. 128: 0 → 1
Char. 138: 0 → 1
Char. 146: 1 → 0
Char. 155: 0 → 1
Char. 182: 1 → 0
Char. 192: 1 → 2
Char. 226: 0 → 1
Char. 227: 0 → 1
Char. 233: 0 → 1

**Node 84:**
Char. 61: 1 → 3
Char. 90: 0 → 1
Char. 91: 0 → 1
Char. 109: 1 → 0
Char. 155: 0 → 1
Char. 209: 1 → 0

**ANALYSIS 18**
**(NO *PROGANOCHELYS QUENSTEDTI*, *PAPPOCHELYS ROSINAE*, AND *ODONTOCHELYS SEMITESTACEA*)**

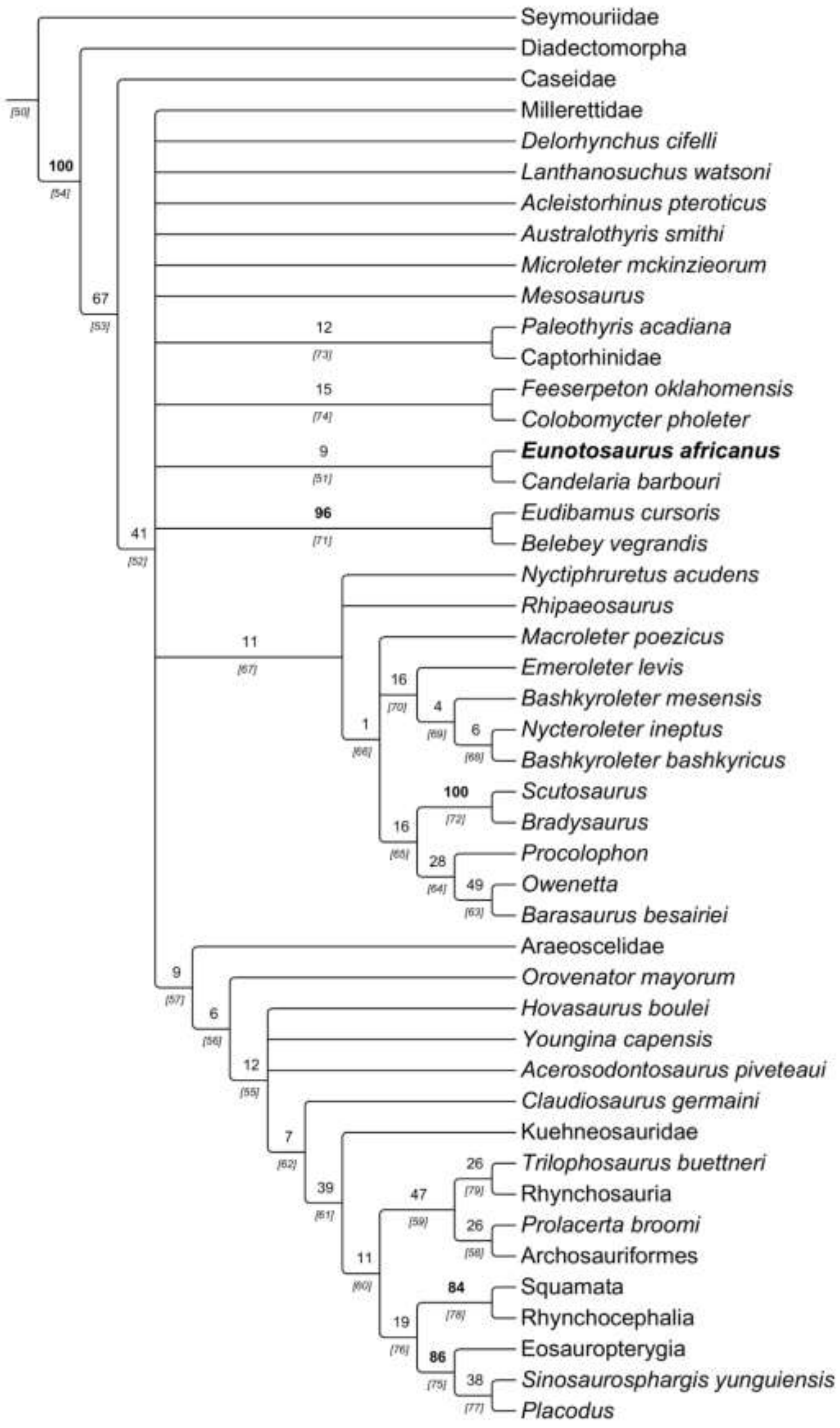

**Seymouriidae:**
Char. 51: 0 → 2
Char. 54: 1 → 0
Char. 83: 0 → 1
Char. 85: 1 → 0
Char. 99: 1 → 0
Char. 107: 0 → 1
Char. 140: 0 → 2
Char. 154: 1 → 2
Char. 225: 1 → 0
Char. 260: 2 → 0

***Eunotosaurus africanus*:**
Char. 0: 0 → 2
Char. 19: 0 → 1
Char. 21: 0 → 1
Char. 24: 0 → 1
Char. 42: 0 → 1
Char. 43: 0 → 1
Char. 81: 1 → 0
Char. 84: 1 → 2
Char. 273: 0 → 1
Char. 274: 0 → 1

***Acerosodontosaurus piveteaui*:**
Char. 78: 1 → 0
Char. 81: 1 → 0
Char. 128: 0 → 1
Char. 155: 0 → 1
Char. 206: 0 → 2
Char. 208: 0 → 1

***Acleistorhinus pteroticus*:**
Char. 20: 0 → 1
Char. 21: 0 → 1
Char. 30: 0 → 1
Char. 33: 0 → 1
Char. 47: 0 → 1
Char. 48: 0 → 1
Char. 55: 1 → 0
Char. 56: 0 → 1
Char. 64: 0 → 1
Char. 70: 0 → 1
Char. 79: 0 → 1
Char. 95: 0 → 1
Char. 110: 0 → 1
Char. 113: 0 → 1
Char. 114: 0 → 1
Char. 131: 0 → 1
Char. 137: 0 → 1
Char. 140: 0 → 2
Char. 146: 1 → 0
Char. 169: 1 → 0
Char. 170: 0 → 1
Char. 278: 3 → 1

**Araeoscelidae:**
Char. 5: 1 → 0
Char. 28: 0 → 1
Char. 106: 0 → 1
Char. 116: 1 → 0
Char. 166: 1 → 0
Char. 169: 1 → 0
Char. 170: 0 → 1
Char. 197: 0 → 1
Char. 221: 1 → 0
Char. 239: 0 → 1

**Archosauriformes:**
Char. 32: 0 → 1
Char. 94: 1 → 0
Char. 112: 1 → 0
Char. 152: 0 → 1
Char. 154: 0 → 2
Char. 166: 1 → 0
Char. 171: 0 → 1
Char. 185: 0 → 1
Char. 204: 0 → 1
Char. 218: 0 → 3
Char. 242: 0 → 1

***Australothyris smithi*:**
Char. 23: 0 → 1
Char. 24: 0 → 1
Char. 34: 0 → 1
Char. 55: 1 → 0
Char. 57: 1 → 0
Char. 71: 1 → 0
Char. 79: 0 → 1
Char. 83: 0 → 1
Char. 85: 1 → 0
Char. 98: 1 → 0
Char. 100: 0 → 1
Char. 103: 0 → 1
Char. 110: 0 → 1
Char. 112: 0 → 1
Char. 123: 1 → 0
Char. 129: 0 → 1
Char. 131: 0 → 1
Char. 132: 1 → 0
Char. 144: 1 → 0
Char. 149: 1 → 0
Char. 150: 0 → 1
Char. 163: 1 → 0

***Barasaurus besairiei*:**
Char. 33: 1 → 0
Char. 75: 1 → 0
Char. 216: 0 → 1

***Bashkyroleter bashkyricus*:**
Char. 275: 1 → 0

***Bashkyroleter mesensis*:**
Char. 169: 1 → 0

***Belebey vegrandis*:**
Char. 154: 1 → 0

***Bradysaurus* spp.:**
Char. 19: 0 → 1
Char. 73: 0 → 1
Char. 79: 0 → 1
Char. 135: 1 → 0
Char. 249: 0 → 1

***Candelaria barbouri*:**
Char. 1: 0 → 1
Char. 5: 1 → 0
Char. 8: 0 → 1
Char. 23: 0 → 1
Char. 49: 0 → 1
Char. 50: 1 → 0
Char. 57: 1 → 0
Char. 67: 0 → 1
Char. 79: 0 → 2
Char. 83: 0 → 1
Char. 88: 1 → 0
Char. 95: 0 → 1
Char. 126: 0 → 1
Char. 132: 1 → 0
Char. 154: 1 → 2
Char. 169: 1 → 0

**Captorhinidae:**
Char. 3: 0 → 1
Char. 23: 0 → 1
Char. 25: 1 → 0
Char. 26: 0 → 1
Char. 73: 0 → 1
Char. 75: 0 → 1
Char. 83: 0 → 1
Char. 108: 1 → 0
Char. 183: 1 → 0
Char. 201: 1 → 0
Char. 203: 1 → 2
Char. 216: 1 → 0

**Caseidae:**
Char. 24: 0 → 1
Char. 25: 1 → 0
Char. 36: 0 → 1
Char. 38: 1 → 0
Char. 46: 1 → 0
Char. 50: 1 → 0
Char. 56: 0 → 1
Char. 85: 1 → 0
Char. 98: 1 → 0
Char. 170: 0 → 1
Char. 194: 0 → 1
Char. 273: 0 → 1
Char. 274: 0 → 1
Char. 276: 0 → 1

***Claudiosaurus germaini*:**
Char. 64: 0 → 1
Char. 105: 0 → 1
Char. 130: 0 → 1
Char. 144: 1 → 0
Char. 166: 1 → 0
Char. 187: 1 → 0
Char. 199: 0 → 1
Char. 203: 1 → 2
Char. 204: 0 → 1
Char. 220: 0 → 1
Char. 222: 1 → 0

***Colobomycter pholeter*:**
Char. 21: 0 → 1
Char. 25: 1 → 0
Char. 84: 1 → 0
Char. 154: 1 → 0
Char. 167: 0 → 1
Char. 267: 0 → 1

***Delorhynchus cifelli*:**
Char. 18: 0 → 1
Char. 20: 0 → 1
Char. 21: 0 → 1
Char. 24: 0 → 1
Char. 26: 0 → 1
Char. 28: 0 → 1
Char. 33: 0 → 2
Char. 39: 0 → 1
Char. 48: 0 → 1
Char. 52: 0 → 1
Char. 100: 0 → 1
Char. 111: 0 → 1
Char. 116: 1 → 0
Char. 117: 0 → 1
Char. 119: 1 → 0
Char. 131: 0 → 1
Char. 156: 1 → 0
Char. 167: 0 → 1
Char. 189: 0 → 1
Char. 191: 0 → 1
Char. 192: 0 → 1
Char. 267: 0 → 1

**Diadectomorpha:**
Char. 0: 0 → 1
Char. 70: 0 → 1
Char. 75: 0 → 1
Char. 122: 0 → 1
Char. 123: 1 → 0
Char. 146: 1 → 0

***Emeroleter levis*:**
Char. 0: 0 → 2
Char. 51: 1 → 0

**Eosauropterygia:**
Char. 159: 1 → 0
Char. 166: 1 → 0
Char. 174: 0 → 1
Char. 194: 0 → 2
Char. 272: 1 → 0

***Eudibamus cursoris*:**
Char. 154: 1 → 2
Char. 163: 1 → 0

***Feeserpeton oklahomensis*:**
Char. 51: 0 → 1
Char. 70: 0 → 1
Char. 157: 0 → 1
Char. 158: 0 → 1

***Hovasaurus boulei*:**
Char. 41: 0 → 1
Char. 55: 1 → 0
Char. 60: 1 → 0
Char. 72: 2 → 1
Char. 77: 0 → 2
Char. 78: 1 → 0
Char. 79: 0 → 1
Char. 93: 1 → 0
Char. 113: 0 → 1
Char. 138: 0 → 1
Char. 146: 1 → 0
Char. 154: 0 → 1
Char. 204: 0 → 2
Char. 206: 0 → 2
Char. 220: 0 → 1
Char. 278: 01 → 3

**Kuehneosauridae:**
Char. 7: 0 → 1
Char. 26: 0 → 1
Char. 44: 1 → 0
Char. 79: 0 → 2
Char. 82: 0 → 1
Char. 98: 1 → 0
Char. 108: 1 → 0
Char. 113: 0 → 1
Char. 128: 0 → 1
Char. 159: 1 → 0
Char. 181: 0 → 1
Char. 185: 0 → 1
Char. 206: 0 → 2
Char. 245: 0 → 1
Char. 278: 0 → 3

***Lanthanosuchus watsoni*:**
Char. 25: 1 → 0
Char. 51: 0 → 1
Char. 86: 0 → 1
Char. 95: 0 → 1
Char. 98: 1 → 0
Char. 110: 0 → 1
Char. 113: 0 → 1
Char. 114: 0 → 1
Char. 119: 1 → 0
Char. 131: 0 → 1
Char. 137: 0 → 1
Char. 138: 0 → 1
Char. 140: 0 → 2
Char. 144: 1 → 0
Char. 154: 1 → 2
Char. 192: 0 → 1

***Macroleter poezicus*:**
Char. 0: 0 → 1
Char. 9: 0 → 1
Char. 26: 0 → 1
Char. 52: 0 → 1
Char. 66: 1 → 2
Char. 84: 1 → 0
Char. 87: 0 → 1
Char. 134: 0 → 2
Char. 139: 0 → 1
Char. 140: 0 → 1
Char. 146: 1 → 0
Char. 169: 1 → 0
Char. 235: 0 → 1

***Mesosaurus* spp.:**
Char. 0: 0 → 1
Char. 2: 0 → 1
Char. 5: 1 → 0
Char. 6: 0 → 1

Char. 8: 0 → 1
Char. 9: 0 → 1
Char. 13: 0 → 1
Char. 19: 0 → 1
Char. 23: 0 → 1
Char. 26: 0 → 1
Char. 29: 1 → 0
Char. 33: 0 → 1
Char. 38: 1 → 0
Char. 41: 0 → 1
Char. 48: 0 → 1
Char. 50: 1 → 0
Char. 67: 0 → 1
Char. 83: 0 → 1
Char. 84: 1 → 0
Char. 85: 1 → 0
Char. 94: 0 → 1
Char. 107: 0 → 1
Char. 109: 0 → 1
Char. 111: 0 → 1
Char. 115: 0 → 1
Char. 146: 1 → 0
Char. 147: 1 → 0
Char. 148: 0 → 1
Char. 149: 1 → 0
Char. 163: 1 → 0
Char. 164: 0 → 1
Char. 166: 1 → 0
Char. 167: 0 → 1
Char. 176: 0 → 1
Char. 183: 1 → 0
Char. 184: 0 → 1
Char. 199: 0 → 1
Char. 202: 0 → 1
Char. 206: 0 → 1
Char. 207: 0 → 1
Char. 209: 0 → 1
Char. 217: 0 → 1
Char. 219: 0 → 1
Char. 220: 0 → 1
Char. 231: 0 → 1
Char. 260: 2 → 0
Char. 272: 2 → 0
Char. 278: 3 → 0

***Microleter mckinzieorum*:**
Char. 0: 0 → 1
Char. 18: 0 → 1
Char. 24: 0 → 1
Char. 25: 1 → 0
Char. 36: 0 → 1
Char. 39: 0 → 1
Char. 51: 0 → 2
Char. 56: 0 → 1
Char. 57: 1 → 0
Char. 70: 0 → 1
Char. 79: 0 → 1
Char. 83: 0 → 1
Char. 94: 0 → 1
Char. 106: 0 → 1
Char. 110: 0 → 1
Char. 132: 1 → 0
Char. 166: 1 → 0
Char. 276: 0 → 1
Char. 278: 3 → 1

**Millerettidae:**
Char. 5: 1 → 0
Char. 24: 0 → 1
Char. 25: 1 → 0
Char. 44: 1 → 0
Char. 56: 0 → 1
Char. 57: 1 → 0
Char. 66: 0 → 2
Char. 78: 1 → 0
Char. 80: 1 → 0
Char. 84: 1 → 2
Char. 88: 1 → 0
Char. 96: 1 → 0
Char. 117: 0 → 1
Char. 124: 0 → 1
Char. 127: 0 → 1
Char. 135: 0 → 1
Char. 145: 0 → 1
Char. 147: 1 → 0
Char. 163: 1 → 0
Char. 166: 1 → 0
Char. 202: 0 → 1
Char. 211: 0 → 1
Char. 230: 0 → 1
Char. 240: 0 → 1
Char. 248: 0 → 1
Char. 252: 0 → 1
Char. 253: 0 → 1

***Nycteroleter ineptus*:**
Char. 278: 0 → 3

***Nyctiphruretus acudens*:**
Char. 0: 0 → 1
Char. 21: 0 → 1
Char. 33: 1 → 2
Char. 41: 0 → 1
Char. 66: 1 → 2
Char. 81: 1 → 0
Char. 83: 0 → 1
Char. 84: 1 → 2
Char. 85: 1 → 0
Char. 94: 0 → 1
Char. 166: 1 → 0
Char. 167: 0 → 1
Char. 224: 0 → 1
Char. 266: 0 → 1
Char. 272: 2 → 1
Char. 276: 0 → 1

***Orovenator mayorum*:**
Char. 8: 0 → 1
Char. 24: 0 → 1
Char. 36: 0 → 1
Char. 160: 0 → 1
Char. 165: 0 → 1

***Owenetta* spp.:**
Char. 169: 1 → 0

***Paleothyris acadiana*:**
Char. 38: 1 → 0
Char. 50: 1 → 0
Char. 66: 1 → 2
Char. 102: 0 → 1
Char. 146: 1 → 0
Char. 237: 0 → 1
Char. 239: 0 → 1
Char. 240: 0 → 1

***Placodus* spp.:**
Char. 0: 1 → 2
Char. 9: 0 → 1
Char. 12: 0 → 1
Char. 13: 0 → 1
Char. 19: 0 → 1
Char. 26: 0 → 1
Char. 31: 0 → 1
Char. 44: 1 → 0
Char. 46: 1 → 0
Char. 57: 0 → 1
Char. 78: 1 → 0
Char. 93: 1 → 0
Char. 102: 1 → 2
Char. 109: 1 → 0
Char. 140: 1 → 0
Char. 155: 0 → 1
Char. 163: 1 → 0
Char. 164: 0 → 1

***Procolophon* spp.:**
Char. 41: 0 → 1
Char. 69: 0 → 1
Char. 79: 0 → 1
Char. 83: 0 → 1
Char. 88: 0 → 1
Char. 117: 1 → 0
Char. 149: 1 → 0
Char. 237: 0 → 1
Char. 238: 0 → 1
Char. 272: 2 → 1

***Prolacerta broomi*:**
Char. 58: 1 → 0
Char. 66: 1 → 0
Char. 67: 1 → 0
Char. 80: 1 → 0
Char. 139: 1 → 0
Char. 147: 1 → 0
Char. 192: 1 → 0
Char. 203: 1 → 2
Char. 206: 0 → 12

***Rhipaeosaurus* spp.:**
Char. 172: 0 → 1
Char. 277: 0 → 1

**Rhynchocephalia:**
Char. 0: 1 → 2
Char. 24: 0 → 1
Char. 75: 1 → 0
Char. 77: 0 → 1
Char. 94: 1 → 0
Char. 117: 12 → 0
Char. 139: 1 → 0
Char. 167: 1 → 0
Char. 205: 1 → 0

**Rhynchosauria:**
Char. 0: 1 → 0
Char. 7: 0 → 1
Char. 9: 0 → 1
Char. 26: 0 → 1
Char. 44: 1 → 0
Char. 68: 0 → 1
Char. 99: 1 → 0
Char. 150: 1 → 0
Char. 160: 0 → 1
Char. 161: 0 → 1
Char. 171: 0 → 2
Char. 182: 1 → 0
Char. 223: 0 → 1
Char. 241: 0 → 1

***Scutosaurus* spp.:**
Char. 175: 0 → 1
Char. 218: 0 → 1
Char. 243: 0 → 2
Char. 244: 0 → 1
Char. 251: 0 → 1

***Sinosaurosphargis yunguiensis*:**
Char. 8: 0 → 1
Char. 30: 0 → 1
Char. 53: 0 → 1
Char. 82: 0 → 1
Char. 89: 1 → 0
Char. 127: 1 → 0
Char. 150: 1 → 0
Char. 154: 0 → 2
Char. 167: 1 → 0
Char. 253: 0 → 1
Char. 255: 0 → 1

**Squamata:**
Char. 26: 0 → 1
Char. 45: 0 → 1
Char. 79: 0 → 2
Char. 80: 1 → 0
Char. 82: 0 → 1
Char. 92: 1 → 0
Char. 109: 1 → 0
Char. 160: 0 → 1
Char. 245: 0 → 1

***Trilophosaurus buettneri*:**
Char. 5: 1 → 0
Char. 11: 0 → 1
Char. 55: 1 → 0
Char. 93: 1 → 0
Char. 104: 0 → 1
Char. 113: 0 → 1
Char. 122: 0 → 1
Char. 136: 1 → 0
Char. 144: 1 → 0
Char. 154: 0 → 1
Char. 157: 0 → 1
Char. 159: 1 → 0
Char. 177: 0 → 12
Char. 194: 0 → 1
Char. 203: 1 → 2
Char. 207: 1 → 0
Char. 208: 1 → 0
Char. 272: 1 → 0

***Youngina capensis*:**
Char. 5: 1 → 0
Char. 21: 0 → 1
Char. 44: 1 → 0
Char. 75: 0 → 1
Char. 92: 1 → 0
Char. 163: 1 → 0
Char. 170: 0 → 1
Char. 211: 0 → 1
Char. 231: 0 → 1
Char. 239: 0 → 1

**Node 50:**
Char. 20: 1 → 0
Char. 25: 0 → 1
Char. 33: 2 → 0
Char. 65: 1 → 0
Char. 72: 2 → 0
Char. 73: 1 → 0
Char. 127: 1 → 0
Char. 131: 1 → 0
Char. 145: 1 → 0
Char. 147: 1 → 0
Char. 149: 1 → 0
Char. 174: 1 → 0
Char. 180: 1 → 0
Char. 184: 1 → 0
Char. 187: 1 → 0
Char. 188: 1 → 0
Char. 189: 1 → 0
Char. 191: 1 → 0
Char. 192: 1 → 0
Char. 194: 2 → 0
Char. 205: 1 → 0
Char. 208: 1 → 0
Char. 210: 1 → 0
Char. 217: 1 → 0
Char. 220: 1 → 0
Char. 221: 1 → 0
Char. 222: 1 → 0
Char. 223: 1 → 0
Char. 224: 1 → 0
Char. 226: 1 → 0
Char. 227: 1 → 0
Char. 231: 1 → 0
Char. 232: 1 → 0
Char. 237: 1 → 0
Char. 241: 1 → 0
Char. 246: 12 → 0
Char. 247: 1 → 0
Char. 251: 1 → 0
Char. 253: 2 → 0
Char. 254: 1 → 0
Char. 255: 1 → 0
Char. 256: 1 → 0
Char. 258: 1 → 0
Char. 266: 1 → 0
Char. 267: 1 → 0
Char. 268: 1 → 0

Char. 269: 1 → 0
Char. 272: 1 → 2

**Node 51:**
Char. 15: 0 → 1
Char. 20: 0 → 1
Char. 25: 1 → 0
Char. 29: 1 → 0
Char. 33: 0 → 2
Char. 55: 1 → 0
Char. 89: 0 → 1
Char. 94: 0 → 1
Char. 127: 0 → 1
Char. 276: 0 → 1
Char. 277: 0 → 1

**Node 52:**
Char. 5: 0 → 1
Char. 29: 0 → 1
Char. 40: 0 → 2
Char. 74: 0 → 1
Char. 88: 0 → 1
Char. 116: 0 → 1
Char. 119: 0 → 1
Char. 147: 0 → 1
Char. 149: 0 → 1
Char. 163: 0 → 1
Char. 166: 0 → 1
Char. 169: 0 → 1
Char. 201: 0 → 1
Char. 203: 0 → 1
Char. 221: 0 → 1
Char. 235: 0 → 1

**Node 53:**
Char. 72: 0 → 1
Char. 81: 0 → 1
Char. 93: 0 → 1

**Node 54:**
Char. 78: 0 → 1
Char. 111: 1 → 0
Char. 126: 1 → 0
Char. 135: 1 → 0

**Node 55:**
Char. 33: 0 → 1
Char. 135: 0 → 1

**Node 56:**
Char. 20: 0 → 1
Char. 62: 0 → 1

**Node 57:**
Char. 0: 0 → 1
Char. 27: 0 → 1
Char. 29: 1 → 0
Char. 38: 1 → 0
Char. 40: 2 → 0
Char. 57: 1 → 0
Char. 59: 0 → 1
Char. 60: 0 → 1
Char. 67: 0 → 1
Char. 72: 1 → 2
Char. 84: 1 → 0
Char. 89: 0 → 1
Char. 111: 0 → 1
Char. 112: 0 → 1
Char. 120: 0 → 1
Char. 147: 1 → 0
Char. 154: 1 → 0
Char. 192: 0 → 1
Char. 193: 0 → 1
Char. 222: 0 → 1
Char. 237: 0 → 1
Char. 240: 0 → 1
Char. 266: 0 → 1
Char. 278: 3 → 1

**Node 58:**
Char. 19: 0 → 1
Char. 92: 1 → 0

**Node 59:**
Char. 4: 0 → 1
Char. 15: 0 → 1
Char. 29: 0 → 1
Char. 213: 0 → 1
Char. 226: 0 → 2
Char. 228: 0 → 1
Char. 275: 0 → 1

**Node 60:**
Char. 27: 1 → 0
Char. 75: 0 → 1
Char. 107: 0 → 1
Char. 140: 0 → 1
Char. 147: 0 → 1

**Node 61:**
Char. 58: 0 → 1
Char. 61: 0 → 1
Char. 66: 0 → 1
Char. 69: 0 → 1
Char. 150: 0 → 1
Char. 167: 0 → 1
Char. 205: 0 → 1
Char. 208: 0 → 1
Char. 239: 0 → 1

**Node 62:**
Char. 70: 0 → 1
Char. 73: 0 → 1
Char. 126: 0 → 1
Char. 131: 0 → 1
Char. 182: 0 → 1
Char. 190: 0 → 1

**Node 63:**
Char. 73: 0 → 1
Char. 131: 1 → 0
Char. 205: 0 → 1
Char. 276: 0 → 1

**Node 64:**
Char. 18: 0 → 1
Char. 37: 0 → 1
Char. 100: 1 → 0
Char. 107: 0 → 1
Char. 113: 1 → 0
Char. 118: 1 → 0
Char. 125: 1 → 0
Char. 150: 0 → 1

**Node 65:**
Char. 23: 0 → 1
Char. 71: 1 → 0
Char. 85: 1 → 0
Char. 102: 0 → 1
Char. 103: 0 → 1
Char. 106: 0 → 1
Char. 167: 0 → 1
Char. 214: 0 → 1
Char. 216: 1 → 0
Char. 235: 0 → 2
Char. 241: 0 → 1

**Node 66:**
Char. 48: 0 → 1
Char. 100: 0 → 1
Char. 113: 0 → 1
Char. 132: 1 → 0
Char. 138: 0 → 1
Char. 141: 0 → 1
Char. 207: 0 → 1

**Node 67:**
Char. 20: 0 → 1
Char. 33: 0 → 1
Char. 38: 1 → 2
Char. 39: 0 → 1
Char. 44: 1 → 0
Char. 49: 0 → 1
Char. 51: 0 → 1
Char. 66: 0 → 1
Char. 70: 0 → 1
Char. 80: 1 → 0
Char. 88: 1 → 0
Char. 95: 0 → 1
Char. 112: 0 → 1
Char. 117: 0 → 1
Char. 118: 0 → 1
Char. 125: 0 → 1
Char. 131: 0 → 1
Char. 137: 0 → 1
Char. 148: 0 → 1
Char. 158: 0 → 1
Char. 183: 1 → 2
Char. 192: 0 → 1
Char. 194: 0 → 1
Char. 201: 1 → 0
Char. 211: 0 → 1
Char. 235: 1 → 0
Char. 252: 0 → 1

**Node 68:**
Char. 87: 0 → 1

**Node 69:**
Char. 25: 1 → 0
Char. 76: 1 → 0

**Node 70:**
Char. 79: 0 → 1
Char. 93: 1 → 0
Char. 133: 0 → 1

**Node 71:**
Char. 38: 1 → 2
Char. 39: 0 → 1
Char. 50: 1 → 0
Char. 58: 0 → 1
Char. 59: 0 → 1
Char. 60: 0 → 1
Char. 72: 1 → 2
Char. 83: 0 → 1
Char. 85: 1 → 0
Char. 88: 1 → 0
Char. 95: 0 → 1
Char. 104: 0 → 1
Char. 105: 0 → 1
Char. 106: 0 → 2
Char. 107: 0 → 1
Char. 109: 0 → 1
Char. 110: 0 → 1
Char. 146: 1 → 0
Char. 148: 0 → 1
Char. 155: 0 → 2
Char. 183: 1 → 2

**Node 72:**
Char. 0: 0 → 2
Char. 33: 1 → 0
Char. 38: 2 → 1
Char. 39: 1 → 0
Char. 42: 0 → 1
Char. 43: 0 → 1
Char. 46: 1 → 0
Char. 48: 1 → 0
Char. 49: 1 → 0
Char. 52: 0 → 1
Char. 83: 0 → 2
Char. 84: 1 → 0
Char. 87: 0 → 1
Char. 93: 1 → 0
Char. 132: 0 → 1
Char. 161: 0 → 1
Char. 163: 1 → 0
Char. 172: 0 → 2
Char. 174: 0 → 1
Char. 188: 0 → 1
Char. 189: 0 → 1
Char. 195: 0 → 1
Char. 212: 0 → 1
Char. 221: 1 → 0
Char. 236: 0 → 1
Char. 238: 0 → 2
Char. 242: 0 → 1
Char. 245: 0 → 1
Char. 274: 0 → 1
Char. 275: 1 → 0

**Node 73:**
Char. 5: 1 → 0
Char. 29: 1 → 0
Char. 44: 1 → 0
Char. 59: 0 → 1
Char. 60: 0 → 1
Char. 66: 0 → 1
Char. 67: 0 → 1
Char. 72: 1 → 2
Char. 78: 1 → 0
Char. 84: 1 → 0
Char. 88: 1 → 0
Char. 93: 1 → 0
Char. 111: 0 → 1
Char. 123: 1 → 0
Char. 129: 0 → 1
Char. 147: 1 → 0
Char. 154: 1 → 0
Char. 163: 1 → 0
Char. 169: 1 → 0
Char. 170: 0 → 1
Char. 197: 0 → 1
Char. 221: 1 → 0

**Node 74:**
Char. 20: 0 → 1
Char. 47: 0 → 1
Char. 83: 0 → 1
Char. 85: 1 → 0
Char. 107: 0 → 1
Char. 110: 0 → 1
Char. 169: 1 → 0
Char. 170: 0 → 1

**Node 75:**
Char. 2: 0 → 1
Char. 6: 0 → 1
Char. 98: 1 → 0
Char. 101: 0 → 1
Char. 113: 0 → 1
Char. 181: 0 → 1
Char. 186: 0 → 1
Char. 193: 1 → 0
Char. 198: 0 → 1
Char. 206: 0 → 2
Char. 220: 0 → 1
Char. 239: 1 → 0
Char. 240: 1 → 0
Char. 266: 1 → 0

**Node 76:**
Char. 33: 1 → 2
Char. 68: 0 → 1
Char. 102: 0 → 1
Char. 103: 0 → 1
Char. 223: 0 → 1
Char. 229: 0 → 1
Char. 235: 1 → 2

**Node 77:**
Char. 17: 0 → 1
Char. 42: 0 → 1
Char. 251: 0 → 1
Char. 264: 0 → 1

**Node 78:**
Char. 41: 0 → 1
Char. 61: 1 → 2
Char. 112: 1 → 0
Char. 128: 0 → 1
Char. 138: 0 → 1

Char. 146: 1 → 0
Char. 155: 0 → 1
Char. 182: 1 → 0
Char. 192: 1 → 2

Char. 226: 0 → 1
Char. 227: 0 → 1
Char. 233: 0 → 1

**Node 79:**
Char. 61: 1 → 3
Char. 90: 0 → 1
Char. 91: 0 → 1

Char. 109: 1 → 0
Char. 155: 0 → 1
Char. 209: 1 → 0

**ANALYSIS 19**

**(NO *PROGANOCHELYS QUENSTEDTI*, *ODONTOCHELYS SEMITESTACEA*, AND *EUNOTOSAURUS AFRICANUS*)**

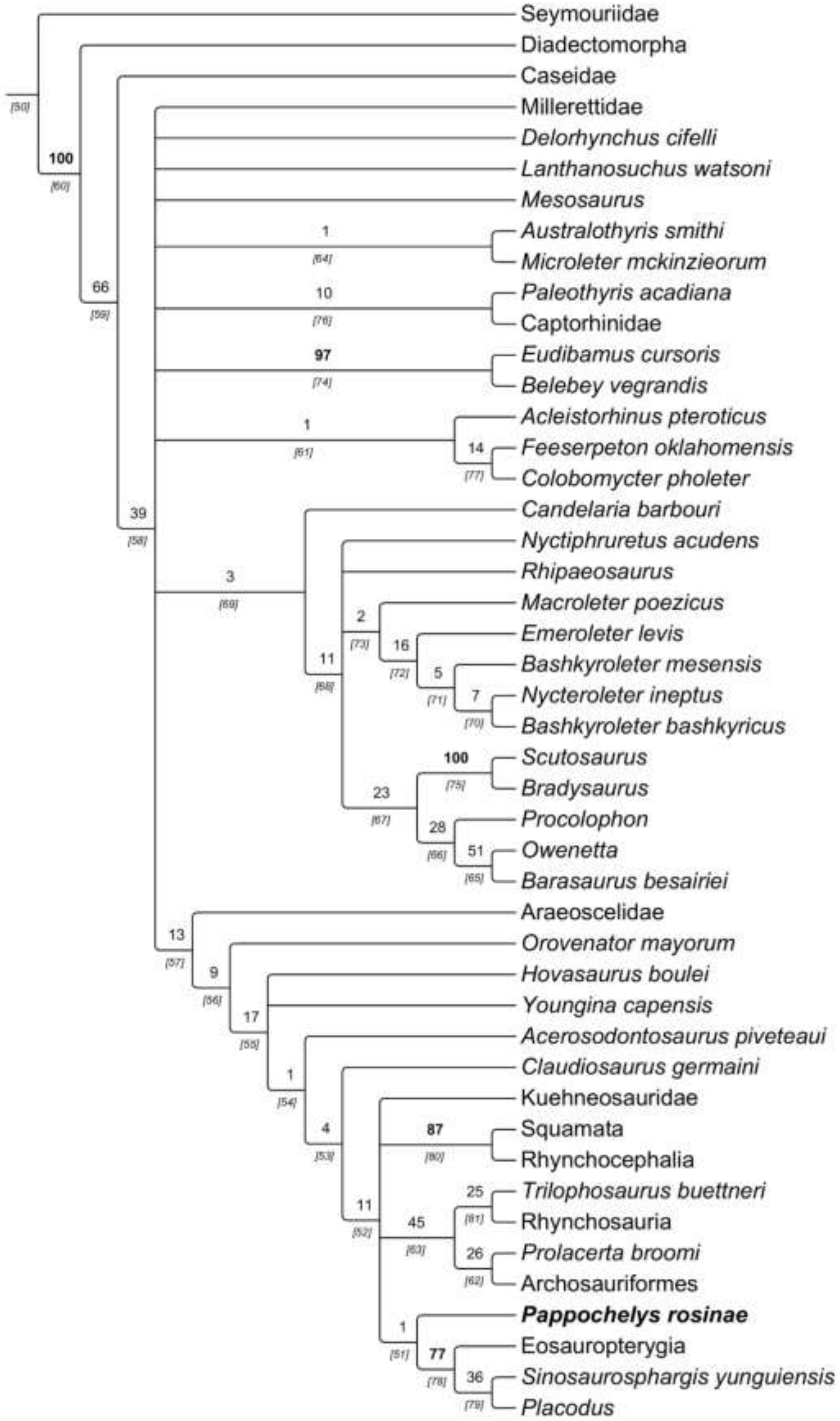

**Seymouriidae:**
Char. 23: 0 → 1
Char. 51: 0 → 2
Char. 54: 1 → 0
Char. 71: 1 → 0
Char. 83: 0 → 1
Char. 85: 1 → 0
Char. 99: 1 → 0
Char. 107: 0 → 1
Char. 126: 0 → 1
Char. 140: 0 → 2
Char. 154: 1 → 2
Char. 225: 1 → 0
Char. 260: 2 → 0

***Pappochelys rosinae*:**
Char. 0: 1 → 0
Char. 5: 1 → 0
Char. 12: 0 → 1
Char. 43: 1 → 0
Char. 49: 0 → 1
Char. 61: 1 → 0
Char. 62: 1 → 0
Char. 65: 0 → 1
Char. 67: 1 → 0
Char. 91: 0 → 1
Char. 169: 1 → 0
Char. 201: 1 → 0
Char. 214: 1 → 0
Char. 219: 1 → 0
Char. 241: 0 → 1
Char. 246: 0 → 1
Char. 247: 0 → 1
Char. 252: 0 → 1
Char. 254: 0 → 1
Char. 255: 0 → 1
Char. 256: 0 → 1
Char. 260: 1 → 0
Char. 268: 0 → 1
Char. 269: 0 → 1

***Acerosodontosaurus piveteaui*:**
Char. 78: 1 → 0
Char. 128: 0 → 1
Char. 155: 0 → 1
Char. 206: 0 → 2

***Acleistorhinus pteroticus*:**
Char. 30: 0 → 1
Char. 33: 0 → 1
Char. 55: 1 → 0
Char. 56: 0 → 1
Char. 64: 0 → 1
Char. 95: 0 → 1
Char. 113: 0 → 1
Char. 114: 0 → 1
Char. 146: 1 → 0
Char. 278: 3 → 1

**Araeoscelidae:**
Char. 23: 0 → 1
Char. 28: 0 → 1
Char. 106: 0 → 1
Char. 116: 1 → 0
Char. 169: 1 → 0
Char. 170: 0 → 1
Char. 197: 0 → 1
Char. 239: 0 → 1

**Archosauriformes:**
Char. 32: 0 → 1
Char. 94: 1 → 0
Char. 112: 1 → 0
Char. 152: 0 → 1
Char. 154: 0 → 2
Char. 166: 1 → 0
Char. 171: 0 → 1
Char. 185: 0 → 1
Char. 204: 0 → 1
Char. 218: 0 → 3
Char. 224: 0 → 1
Char. 242: 0 → 1

***Australothyris smithi*:**
Char. 23: 0 → 1
Char. 34: 0 → 1
Char. 55: 1 → 0
Char. 71: 1 → 0
Char. 85: 1 → 0
Char. 112: 0 → 1
Char. 129: 0 → 1
Char. 131: 0 → 1

***Barasaurus besairiei*:**
Char. 33: 1 → 0
Char. 75: 1 → 0
Char. 216: 0 → 1

***Bashkyroleter bashkyricus*:**
Char. 275: 1 → 0

***Bashkyroleter mesensis*:**
Char. 169: 1 → 0

***Belebey vegrandis*:**
Char. 154: 1 → 0
Char. 163: 0 → 1

***Bradysaurus* spp.:**
Char. 19: 0 → 1
Char. 73: 0 → 1
Char. 79: 0 → 1
Char. 135: 1 → 0
Char. 249: 0 → 1

***Candelaria barbouri*:**
Char. 1: 0 → 1
Char. 8: 0 → 1
Char. 15: 0 → 1
Char. 23: 0 → 1
Char. 25: 1 → 0
Char. 29: 1 → 0
Char. 50: 1 → 0
Char. 55: 1 → 0
Char. 57: 1 → 0
Char. 67: 0 → 1
Char. 79: 0 → 2
Char. 89: 0 → 1
Char. 127: 0 → 1
Char. 154: 1 → 2
Char. 169: 1 → 0
Char. 277: 0 → 1

**Captorhinidae:**
Char. 3: 0 → 1
Char. 23: 0 → 1
Char. 25: 1 → 0
Char. 26: 0 → 1
Char. 73: 0 → 1
Char. 75: 0 → 1
Char. 83: 0 → 1
Char. 108: 1 → 0
Char. 183: 1 → 0
Char. 201: 1 → 0
Char. 203: 1 → 2
Char. 216: 1 → 0

**Caseidae:**
Char. 24: 0 → 1
Char. 25: 1 → 0
Char. 36: 0 → 1
Char. 38: 1 → 0
Char. 46: 1 → 0
Char. 50: 1 → 0
Char. 56: 0 → 1
Char. 85: 1 → 0
Char. 96: 1 → 0
Char. 98: 1 → 0
Char. 170: 0 → 1
Char. 194: 0 → 1
Char. 273: 0 → 1
Char. 274: 0 → 1
Char. 278: 3 → 2

***Claudiosaurus germaini*:**
Char. 24: 0 → 1
Char. 34: 0 → 1
Char. 36: 0 → 1
Char. 43: 1 → 0
Char. 64: 0 → 1
Char. 105: 0 → 1
Char. 130: 0 → 1
Char. 144: 1 → 0
Char. 187: 1 → 0
Char. 199: 0 → 1
Char. 203: 1 → 2
Char. 204: 0 → 1
Char. 220: 0 → 1
Char. 222: 1 → 0

***Colobomycter pholeter*:**
Char. 25: 1 → 0
Char. 84: 1 → 0
Char. 154: 1 → 0
Char. 167: 0 → 1
Char. 267: 0 → 1

***Delorhynchus cifelli*:**
Char. 18: 0 → 1
Char. 20: 0 → 1
Char. 21: 0 → 1
Char. 24: 0 → 1
Char. 26: 0 → 1
Char. 28: 0 → 1
Char. 33: 0 → 2
Char. 39: 0 → 1
Char. 48: 0 → 1
Char. 52: 0 → 1
Char. 100: 0 → 1
Char. 111: 0 → 1
Char. 116: 1 → 0
Char. 131: 0 → 1
Char. 147: 0 → 1
Char. 156: 1 → 0
Char. 159: 0 → 1
Char. 163: 0 → 1
Char. 167: 0 → 1
Char. 180: 0 → 1
Char. 189: 0 → 1
Char. 191: 0 → 1
Char. 204: 0 → 2
Char. 267: 0 → 1

**Diadectomorpha:**
Char. 0: 0 → 1
Char. 70: 0 → 1
Char. 75: 0 → 1
Char. 122: 0 → 1
Char. 123: 1 → 0
Char. 146: 1 → 0
Char. 275: 1 → 0
Char. 278: 3 → 0

***Emeroleter levis*:**
Char. 0: 0 → 2
Char. 51: 1 → 0

**Eosauropterygia:**
Char. 159: 1 → 0
Char. 166: 1 → 0
Char. 174: 0 → 1
Char. 178: 0 → 1
Char. 272: 1 → 0

***Eudibamus cursoris*:**
Char. 154: 1 → 2

***Feeserpeton oklahomensis*:**
Char. 51: 0 → 1
Char. 157: 0 → 1
Char. 158: 0 → 1

***Hovasaurus boulei*:**
Char. 41: 0 → 1
Char. 55: 1 → 0
Char. 60: 1 → 0
Char. 72: 2 → 1
Char. 77: 0 → 2
Char. 78: 1 → 0
Char. 79: 0 → 1
Char. 93: 1 → 0
Char. 113: 0 → 1
Char. 138: 0 → 1
Char. 146: 1 → 0
Char. 154: 0 → 1
Char. 204: 0 → 2
Char. 206: 0 → 2
Char. 220: 0 → 1
Char. 278: 01 → 3

**Kuehneosauridae:**
Char. 7: 0 → 1
Char. 24: 0 → 1
Char. 26: 0 → 1
Char. 27: 0 → 1
Char. 34: 0 → 1
Char. 36: 0 → 1
Char. 43: 1 → 0
Char. 79: 0 → 2
Char. 82: 0 → 1
Char. 98: 1 → 0
Char. 107: 1 → 0
Char. 108: 1 → 0
Char. 113: 0 → 1
Char. 128: 0 → 1
Char. 140: 1 → 0
Char. 147: 1 → 0
Char. 148: 1 → 0
Char. 159: 1 → 0
Char. 181: 0 → 1
Char. 185: 0 → 1
Char. 206: 0 → 2
Char. 245: 0 → 1
Char. 272: 1 → 2
Char. 278: 0 → 3

***Lanthanosuchus watsoni*:**
Char. 25: 1 → 0
Char. 51: 0 → 1
Char. 76: 0 → 1
Char. 86: 0 → 1
Char. 95: 0 → 1
Char. 98: 1 → 0
Char. 110: 0 → 1
Char. 113: 0 → 1
Char. 114: 0 → 1
Char. 131: 0 → 1
Char. 137: 0 → 1
Char. 138: 0 → 1
Char. 140: 0 → 2
Char. 144: 1 → 0
Char. 147: 0 → 1
Char. 154: 1 → 2

***Macroleter poezicus*:**
Char. 0: 0 → 1
Char. 52: 0 → 1
Char. 66: 1 → 2
Char. 75: 0 → 1
Char. 84: 1 → 0
Char. 87: 0 → 1
Char. 110: 0 → 1
Char. 140: 0 → 1
Char. 146: 1 → 0
Char. 169: 1 → 0
Char. 235: 0 → 1

***Mesosaurus* spp.:**
Char. 0: 0 → 1
Char. 2: 0 → 1
Char. 6: 0 → 1

Char. 8: 0 → 1
Char. 9: 0 → 1
Char. 13: 0 → 1
Char. 19: 0 → 1
Char. 23: 0 → 1
Char. 26: 0 → 1
Char. 29: 1 → 0
Char. 33: 0 → 1
Char. 38: 1 → 0
Char. 41: 0 → 1
Char. 48: 0 → 1
Char. 50: 1 → 0
Char. 67: 0 → 1
Char. 76: 0 → 1
Char. 83: 0 → 1
Char. 84: 1 → 0
Char. 85: 1 → 0
Char. 94: 0 → 1
Char. 107: 0 → 1
Char. 109: 0 → 1
Char. 111: 0 → 1
Char. 115: 0 → 1
Char. 146: 1 → 0
Char. 148: 0 → 1
Char. 149: 1 → 0
Char. 164: 0 → 1
Char. 167: 0 → 1
Char. 176: 0 → 1
Char. 183: 1 → 0
Char. 184: 0 → 1
Char. 199: 0 → 1
Char. 202: 0 → 1
Char. 204: 0 → 2
Char. 206: 0 → 1
Char. 207: 0 → 1
Char. 209: 0 → 1
Char. 217: 0 → 1
Char. 219: 0 → 1
Char. 220: 0 → 1
Char. 231: 0 → 1
Char. 260: 2 → 0
Char. 272: 2 → 0
Char. 278: 3 → 0

***Microleter mckinzieorum*:**
Char. 0: 0 → 1
Char. 25: 1 → 0
Char. 36: 0 → 1
Char. 39: 0 → 1
Char. 51: 0 → 2
Char. 56: 0 → 1
Char. 70: 0 → 1
Char. 94: 0 → 1
Char. 106: 0 → 1
Char. 159: 0 → 1
Char. 276: 0 → 1

**Millerettidae:**
Char. 24: 0 → 1
Char. 25: 1 → 0
Char. 44: 1 → 0
Char. 56: 0 → 1
Char. 57: 1 → 0
Char. 66: 0 → 2
Char. 78: 1 → 0
Char. 80: 1 → 0
Char. 84: 1 → 2
Char. 96: 1 → 0
Char. 124: 0 → 1
Char. 127: 0 → 1
Char. 135: 0 → 1
Char. 145: 0 → 1
Char. 202: 0 → 1
Char. 211: 0 → 1
Char. 230: 0 → 1
Char. 234: 0 → 1
Char. 248: 0 → 1
Char. 252: 0 → 1
Char. 253: 0 → 1

***Nycteroleter ineptus*:**
Char. 278: 0 → 3

***Nyctiphruretus acudens*:**
Char. 0: 0 → 1
Char. 21: 0 → 1
Char. 41: 0 → 1
Char. 66: 1 → 2
Char. 81: 1 → 0
Char. 84: 1 → 2
Char. 167: 0 → 1
Char. 215: 1 → 0
Char. 224: 0 → 1
Char. 226: 1 → 0
Char. 266: 0 → 1
Char. 272: 2 → 1

***Orovenator mayorum*:**
Char. 8: 0 → 1
Char. 24: 0 → 1
Char. 36: 0 → 1
Char. 160: 0 → 1
Char. 165: 0 → 1

***Owenetta* spp.:**
Char. 169: 1 → 0

***Paleothyris acadiana*:**
Char. 38: 1 → 0
Char. 50: 1 → 0
Char. 66: 1 → 2
Char. 102: 0 → 1
Char. 146: 1 → 0
Char. 180: 0 → 1
Char. 237: 0 → 1
Char. 239: 0 → 1

***Placodus* spp.:**
Char. 0: 1 → 2
Char. 9: 0 → 1
Char. 12: 0 → 1
Char. 13: 0 → 1
Char. 19: 0 → 1
Char. 26: 0 → 1
Char. 31: 0 → 1
Char. 46: 1 → 0
Char. 57: 0 → 1
Char. 93: 1 → 0
Char. 109: 1 → 0
Char. 140: 1 → 0
Char. 155: 0 → 1
Char. 163: 1 → 0
Char. 164: 0 → 1

***Procolophon* spp.:**
Char. 41: 0 → 1
Char. 69: 0 → 1
Char. 79: 0 → 1
Char. 88: 0 → 1
Char. 117: 1 → 0
Char. 149: 1 → 0
Char. 180: 0 → 1
Char. 204: 0 → 1
Char. 237: 0 → 1
Char. 238: 0 → 1
Char. 272: 2 → 1
Char. 278: 3 → 0

***Prolacerta broomi*:**
Char. 58: 1 → 0
Char. 66: 1 → 0
Char. 67: 1 → 0
Char. 80: 1 → 0
Char. 123: 0 → 1
Char. 139: 1 → 0
Char. 147: 1 → 0
Char. 192: 1 → 0
Char. 203: 1 → 2
Char. 206: 0 → 12

***Rhipaeosaurus* spp.:**
Char. 172: 0 → 1
Char. 186: 1 → 0
Char. 277: 0 → 1

**Rhynchocephalia:**
Char. 0: 1 → 2
Char. 23: 1 → 0
Char. 24: 0 → 1
Char. 77: 0 → 1
Char. 88: 1 → 0
Char. 94: 1 → 0
Char. 104: 0 → 1
Char. 117: 12 → 0
Char. 139: 1 → 0
Char. 167: 1 → 0
Char. 205: 1 → 0

**Rhynchosauria:**
Char. 0: 1 → 0
Char. 7: 0 → 1
Char. 9: 0 → 1
Char. 26: 0 → 1
Char. 63: 1 → 0
Char. 68: 0 → 1
Char. 99: 1 → 0
Char. 123: 0 → 1
Char. 150: 1 → 0
Char. 160: 0 → 1
Char. 161: 0 → 1
Char. 171: 0 → 2
Char. 182: 1 → 0
Char. 224: 0 → 1
Char. 241: 0 → 1

***Scutosaurus* spp.:**
Char. 175: 0 → 1
Char. 218: 0 → 1
Char. 243: 0 → 2
Char. 244: 0 → 1
Char. 251: 0 → 1

***Sinosaurosphargis yunguiensis*:**
Char. 8: 0 → 1
Char. 30: 0 → 1
Char. 53: 0 → 1
Char. 82: 0 → 1
Char. 89: 1 → 0
Char. 127: 1 → 0
Char. 150: 1 → 0
Char. 154: 0 → 2
Char. 167: 1 → 0
Char. 253: 0 → 1
Char. 255: 0 → 1

**Squamata:**
Char. 26: 0 → 1
Char. 45: 0 → 1
Char. 79: 0 → 2
Char. 80: 1 → 0
Char. 82: 0 → 1
Char. 92: 1 → 0
Char. 109: 1 → 0
Char. 122: 0 → 1
Char. 160: 0 → 1
Char. 200: 0 → 1
Char. 245: 0 → 1

***Trilophosaurus buettneri*:**
Char. 5: 1 → 0
Char. 11: 0 → 1
Char. 55: 1 → 0
Char. 93: 1 → 0
Char. 104: 0 → 1
Char. 113: 0 → 1
Char. 122: 0 → 1
Char. 136: 1 → 0
Char. 144: 1 → 0
Char. 154: 0 → 1
Char. 157: 0 → 1
Char. 159: 1 → 0
Char. 177: 0 → 12
Char. 194: 0 → 1
Char. 203: 1 → 2
Char. 207: 1 → 0
Char. 208: 1 → 0
Char. 272: 1 → 0

***Youngina capensis*:**
Char. 75: 0 → 1
Char. 170: 0 → 1
Char. 211: 0 → 1
Char. 231: 0 → 1
Char. 239: 0 → 1

**Node 50:**
Char. 0: 2 → 0
Char. 5: 1 → 0
Char. 15: 1 → 0
Char. 20: 1 → 0
Char. 21: 1 → 0
Char. 24: 1 → 0
Char. 25: 0 → 1
Char. 33: 2 → 0
Char. 42: 1 → 0
Char. 73: 1 → 0
Char. 84: 2 → 01
Char. 119: 1 → 0
Char. 127: 1 → 0
Char. 130: 1 → 0
Char. 134: 2 → 0
Char. 136: 1 → 0
Char. 141: 1 → 0
Char. 145: 1 → 0
Char. 147: 1 → 0
Char. 149: 1 → 0
Char. 151: 1 → 0
Char. 152: 1 → 0
Char. 155: 1 → 0
Char. 158: 1 → 0
Char. 159: 1 → 0
Char. 161: 1 → 0
Char. 163: 1 → 0
Char. 166: 1 → 0
Char. 169: 1 → 0
Char. 174: 1 → 0
Char. 180: 1 → 0
Char. 181: 1 → 0
Char. 187: 1 → 0
Char. 188: 1 → 0
Char. 189: 1 → 0
Char. 194: 2 → 0
Char. 196: 1 → 0
Char. 203: 2 → 0
Char. 204: 2 → 0
Char. 208: 1 → 0
Char. 217: 1 → 0
Char. 220: 1 → 0
Char. 221: 1 → 0
Char. 226: 1 → 0
Char. 227: 1 → 0
Char. 231: 1 → 0
Char. 234: 1 → 0
Char. 247: 1 → 0
Char. 251: 1 → 0
Char. 253: 2 → 0

**Node 51:**
Char. 1: 0 → 1
Char. 55: 1 → 0
Char. 193: 1 → 0
Char. 206: 0 → 2
Char. 220: 0 → 1
Char. 239: 1 → 0

**Node 52:**
Char. 27: 1 → 0
Char. 58: 0 → 1
Char. 61: 0 → 1
Char. 66: 0 → 1
Char. 69: 0 → 1
Char. 84: 0 → 1
Char. 107: 0 → 1

Char. 123: 1 → 0
Char. 140: 0 → 1
Char. 147: 0 → 1
Char. 150: 0 → 1
Char. 167: 0 → 1
Char. 184: 0 → 1
Char. 205: 0 → 1
Char. 210: 0 → 1
Char. 230: 0 → 1
Char. 239: 0 → 1

**Node 53:**
Char. 126: 0 → 1
Char. 190: 0 → 1

**Node 54:**
Char. 23: 0 → 1
Char. 94: 0 → 1
Char. 224: 1 → 0

**Node 55:**
Char. 33: 0 → 1
Char. 135: 0 → 1
Char. 159: 0 → 1

**Node 56:**
Char. 20: 0 → 1
Char. 62: 0 → 1

**Node 57:**
Char. 0: 0 → 1
Char. 27: 0 → 1
Char. 29: 1 → 0
Char. 38: 1 → 0
Char. 40: 2 → 0
Char. 57: 1 → 0
Char. 59: 0 → 1
Char. 60: 0 → 1
Char. 67: 0 → 1
Char. 72: 1 → 2
Char. 84: 1 → 0
Char. 89: 0 → 1
Char. 111: 0 → 1
Char. 112: 0 → 1
Char. 120: 0 → 1
Char. 154: 1 → 0
Char. 180: 0 → 1
Char. 193: 0 → 1
Char. 222: 0 → 1
Char. 224: 0 → 1
Char. 234: 0 → 1
Char. 237: 0 → 1
Char. 266: 0 → 1
Char. 278: 3 → 1

**Node 58:**
Char. 29: 0 → 1
Char. 40: 0 → 2
Char. 74: 0 → 1
Char. 116: 0 → 1
Char. 149: 0 → 1
Char. 169: 0 → 1
Char. 201: 0 → 1
Char. 203: 0 → 1
Char. 235: 0 → 1
Char. 276: 1 → 0

**Node 59:**
Char. 81: 0 → 1
Char. 93: 0 → 1
Char. 97: 0 → 1

**Node 60:**
Char. 78: 0 → 1
Char. 111: 1 → 0
Char. 135: 1 → 0

**Node 61:**
Char. 20: 0 → 1
Char. 47: 0 → 1
Char. 48: 0 → 1
Char. 79: 0 → 1
Char. 110: 0 → 1
Char. 131: 0 → 1
Char. 137: 0 → 1
Char. 140: 0 → 2
Char. 147: 0 → 1
Char. 159: 0 → 1
Char. 163: 0 → 1
Char. 169: 1 → 0
Char. 170: 0 → 1

**Node 62:**
Char. 19: 0 → 1
Char. 92: 1 → 0

**Node 63:**
Char. 4: 0 → 1
Char. 15: 0 → 1
Char. 29: 0 → 1
Char. 35: 1 → 0
Char. 48: 1 → 0
Char. 210: 1 → 0
Char. 213: 0 → 1
Char. 226: 0 → 2
Char. 228: 0 → 1
Char. 275: 0 → 1

**Node 64:**
Char. 24: 0 → 1
Char. 57: 1 → 0
Char. 79: 0 → 1
Char. 83: 0 → 1
Char. 110: 0 → 1
Char. 132: 1 → 0

**Node 65:**
Char. 73: 0 → 1
Char. 131: 1 → 0
Char. 205: 0 → 1
Char. 239: 0 → 1

**Node 66:**
Char. 18: 0 → 1
Char. 37: 0 → 1
Char. 48: 0 → 1
Char. 107: 0 → 1
Char. 118: 1 → 0
Char. 125: 1 → 0
Char. 150: 0 → 1

**Node 67:**
Char. 23: 0 → 1
Char. 71: 1 → 0
Char. 75: 0 → 1
Char. 102: 0 → 1
Char. 103: 0 → 1
Char. 106: 0 → 1
Char. 110: 0 → 1
Char. 155: 0 → 1
Char. 167: 0 → 1
Char. 207: 0 → 1
Char. 214: 0 → 1
Char. 216: 1 → 0
Char. 235: 0 → 2
Char. 241: 0 → 1

**Node 68:**
Char. 66: 0 → 1
Char. 159: 0 → 1

**Node 69:**
Char. 20: 0 → 1
Char. 33: 0 → 12
Char. 49: 0 → 1
Char. 76: 0 → 1
Char. 95: 0 → 1

**Node 70:**
Char. 87: 0 → 1
Char. 110: 0 → 1

**Node 71:**
Char. 25: 1 → 0
Char. 76: 1 → 0

**Node 72:**
Char. 79: 0 → 1
Char. 86: 0 → 1
Char. 93: 1 → 0
Char. 133: 0 → 1

**Node 73:**
Char. 48: 0 → 1
Char. 100: 0 → 1
Char. 113: 0 → 1
Char. 147: 1 → 2
Char. 154: 1 → 0
Char. 207: 0 → 1
Char. 239: 0 → 1
Char. 278: 3 → 0

**Node 74:**
Char. 38: 1 → 2
Char. 39: 0 → 1
Char. 50: 1 → 0
Char. 58: 0 → 1
Char. 59: 0 → 1
Char. 60: 0 → 1
Char. 72: 1 → 2
Char. 83: 0 → 1
Char. 85: 1 → 0
Char. 95: 0 → 1
Char. 104: 0 → 1
Char. 105: 0 → 1
Char. 106: 0 → 2
Char. 107: 0 → 1
Char. 109: 0 → 1
Char. 110: 0 → 1
Char. 146: 1 → 0
Char. 148: 0 → 1
Char. 155: 0 → 2
Char. 183: 1 → 2

**Node 75:**
Char. 0: 0 → 2
Char. 33: 1 → 0
Char. 38: 2 → 1
Char. 39: 1 → 0
Char. 42: 0 → 1
Char. 43: 0 → 1
Char. 46: 1 → 0
Char. 49: 1 → 0
Char. 52: 0 → 1
Char. 83: 01 → 2
Char. 84: 1 → 0
Char. 87: 0 → 1
Char. 93: 1 → 0
Char. 100: 0 → 1
Char. 113: 0 → 1
Char. 143: 0 → 1
Char. 161: 0 → 1
Char. 163: 1 → 0
Char. 172: 0 → 2
Char. 174: 0 → 1
Char. 188: 0 → 1
Char. 189: 0 → 1
Char. 195: 0 → 1
Char. 204: 0 → 2
Char. 212: 0 → 1
Char. 236: 0 → 1
Char. 238: 0 → 2
Char. 242: 0 → 1
Char. 245: 0 → 1
Char. 274: 0 → 1
Char. 275: 1 → 0

**Node 76:**
Char. 29: 1 → 0
Char. 44: 1 → 0
Char. 59: 0 → 1
Char. 60: 0 → 1
Char. 66: 0 → 1
Char. 67: 0 → 1
Char. 72: 1 → 2
Char. 78: 1 → 0
Char. 84: 1 → 0
Char. 93: 1 → 0
Char. 111: 0 → 1
Char. 123: 1 → 0
Char. 129: 0 → 1
Char. 154: 1 → 0
Char. 169: 1 → 0
Char. 170: 0 → 1
Char. 197: 0 → 1

**Node 77:**
Char. 83: 0 → 1
Char. 85: 1 → 0
Char. 107: 0 → 1

**Node 78:**
Char. 2: 0 → 1
Char. 6: 0 → 1
Char. 198: 0 → 1
Char. 240: 1 → 0
Char. 266: 1 → 0
Char. 267: 1 → 0

**Node 79:**
Char. 17: 0 → 1
Char. 42: 0 → 1
Char. 251: 0 → 1
Char. 264: 0 → 1

**Node 80:**
Char. 33: 1 → 2
Char. 48: 1 → 0
Char. 61: 1 → 2
Char. 68: 0 → 1
Char. 112: 1 → 0
Char. 128: 0 → 1
Char. 138: 0 → 1
Char. 146: 1 → 0
Char. 155: 0 → 1
Char. 158: 0 → 1
Char. 182: 1 → 0
Char. 192: 1 → 2
Char. 224: 0 → 1
Char. 226: 0 → 1
Char. 227: 0 → 1
Char. 233: 0 → 1
Char. 235: 1 → 2
Char. 267: 1 → 2

**Node 81:**
Char. 61: 1 → 3
Char. 90: 0 → 1
Char. 91: 0 → 1
Char. 109: 1 → 0
Char. 155: 0 → 1
Char. 209: 1 → 0

**ANALYSIS 20**
**(NO *PROGANOCHELYS QUENSTEDTI*, *CANDELARIA BARBOURI*, AND *ODONTOCHELYS SEMITESTACEA*)**

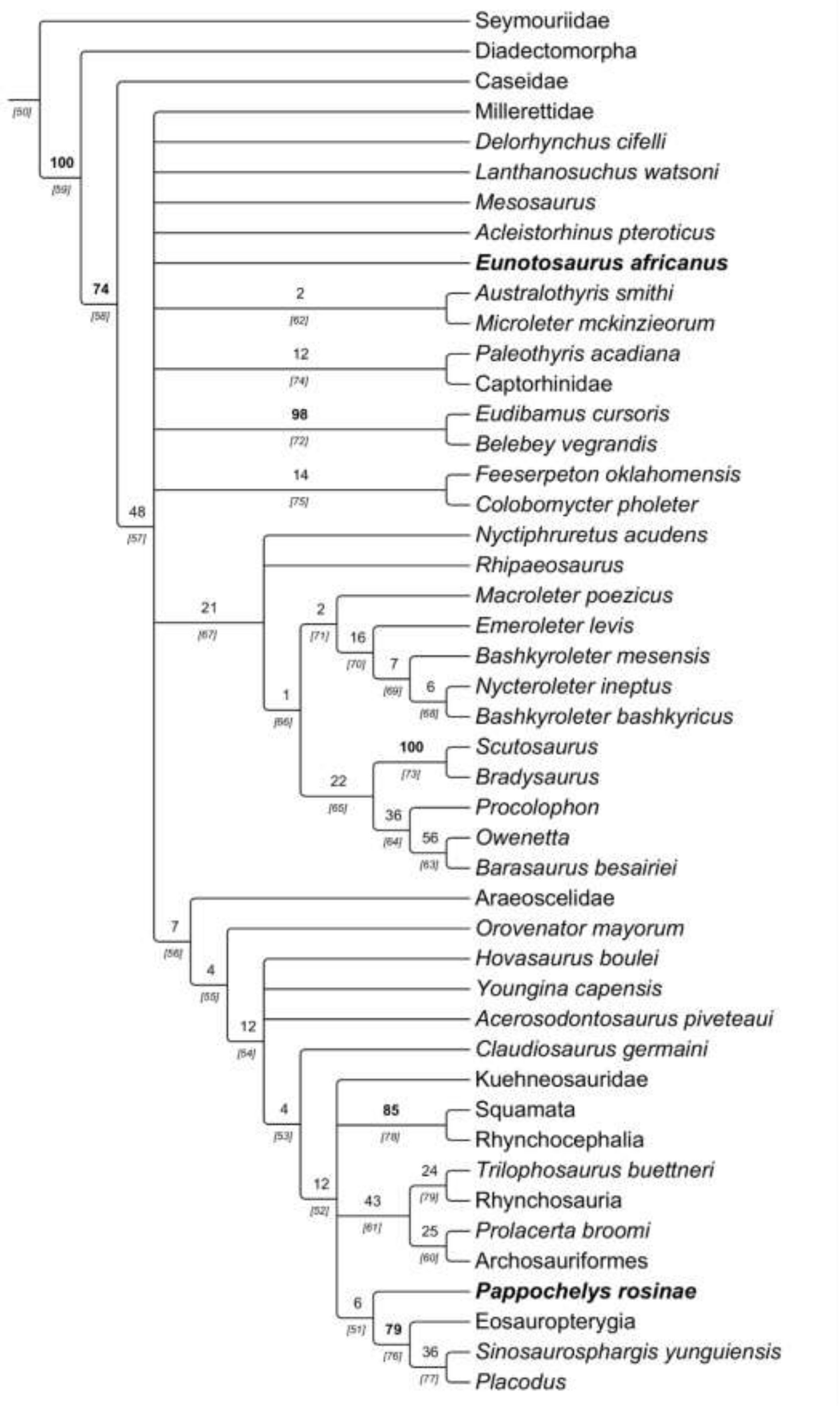

**Seymouriidae:**
Char. 51: 0 → 2
Char. 54: 1 → 0
Char. 85: 1 → 0
Char. 99: 1 → 0
Char. 107: 0 → 1
Char. 140: 0 → 2
Char. 154: 1 → 2
Char. 225: 1 → 0
Char. 260: 2 → 0

***Pappochelys rosinae*:**
Char. 0: 1 → 0
Char. 5: 1 → 0
Char. 12: 0 → 1
Char. 43: 1 → 0
Char. 49: 0 → 1
Char. 61: 1 → 0
Char. 62: 1 → 0
Char. 65: 0 → 1
Char. 67: 1 → 0
Char. 91: 0 → 1
Char. 169: 1 → 0
Char. 201: 1 → 0
Char. 214: 1 → 0
Char. 219: 1 → 0
Char. 241: 0 → 1
Char. 246: 0 → 1
Char. 247: 0 → 1
Char. 252: 0 → 1
Char. 254: 0 → 1
Char. 255: 0 → 1
Char. 256: 0 → 1
Char. 260: 1 → 0
Char. 268: 0 → 1
Char. 269: 0 → 1

***Eunotosaurus africanus*:**
Char. 0: 0 → 2
Char. 15: 0 → 1
Char. 19: 0 → 1
Char. 20: 0 → 1
Char. 21: 0 → 1
Char. 24: 0 → 1
Char. 25: 1 → 0
Char. 29: 1 → 0
Char. 33: 0 → 2
Char. 40: 2 → 0
Char. 42: 0 → 1
Char. 43: 0 → 1
Char. 44: 1 → 0
Char. 55: 1 → 0
Char. 64: 0 → 1
Char. 73: 0 → 1
Char. 81: 1 → 0
Char. 84: 1 → 2
Char. 89: 0 → 1
Char. 92: 0 → 1
Char. 94: 0 → 1
Char. 97: 1 → 0
Char. 102: 0 → 1
Char. 103: 0 → 1
Char. 111: 0 → 1
Char. 120: 0 → 1
Char. 124: 0 → 1
Char. 127: 0 → 1
Char. 130: 0 → 1
Char. 134: 0 → 2
Char. 135: 0 → 1
Char. 136: 0 → 1
Char. 141: 0 → 1
Char. 145: 0 → 1
Char. 148: 0 → 1
Char. 151: 0 → 1
Char. 152: 0 → 1
Char. 153: 1 → 0
Char. 155: 0 → 1
Char. 158: 0 → 1
Char. 161: 0 → 1
Char. 174: 0 → 1
Char. 181: 0 → 1
Char. 188: 0 → 1
Char. 194: 0 → 1
Char. 196: 0 → 1
Char. 201: 1 → 0
Char. 203: 1 → 2
Char. 211: 0 → 1
Char. 217: 0 → 1
Char. 219: 0 → 1
Char. 220: 0 → 1
Char. 231: 0 → 1
Char. 247: 0 → 1
Char. 248: 0 → 1
Char. 249: 0 → 2
Char. 250: 0 → 1
Char. 251: 0 → 1
Char. 252: 0 → 1
Char. 253: 0 → 2
Char. 263: 0 → 1
Char. 273: 0 → 1
Char. 274: 0 → 1
Char. 276: 0 → 1
Char. 277: 0 → 1

***Acerosodontosaurus piveteaui*:**
Char. 78: 1 → 0
Char. 81: 1 → 0
Char. 128: 0 → 1
Char. 155: 0 → 1
Char. 206: 0 → 2
Char. 208: 0 → 1

***Acleistorhinus pteroticus*:**
Char. 20: 0 → 1
Char. 21: 0 → 1
Char. 30: 0 → 1
Char. 33: 0 → 1
Char. 47: 0 → 1
Char. 48: 0 → 1
Char. 55: 1 → 0
Char. 56: 0 → 1
Char. 64: 0 → 1
Char. 70: 0 → 1
Char. 79: 0 → 1
Char. 95: 0 → 1
Char. 110: 0 → 1
Char. 113: 0 → 1
Char. 114: 0 → 1
Char. 131: 0 → 1
Char. 137: 0 → 1
Char. 140: 0 → 2
Char. 146: 1 → 0
Char. 169: 1 → 0
Char. 170: 0 → 1
Char. 278: 3 → 1

**Araeoscelidae:**
Char. 5: 1 → 0
Char. 28: 0 → 1
Char. 106: 0 → 1
Char. 116: 1 → 0
Char. 166: 1 → 0
Char. 169: 1 → 0
Char. 170: 0 → 1
Char. 197: 0 → 1
Char. 221: 1 → 0
Char. 239: 0 → 1

**Archosauriformes:**
Char. 32: 0 → 1
Char. 94: 1 → 0
Char. 112: 1 → 0
Char. 152: 0 → 1
Char. 154: 0 → 2
Char. 166: 1 → 0
Char. 171: 0 → 1
Char. 185: 0 → 1
Char. 204: 0 → 1
Char. 218: 0 → 3
Char. 242: 0 → 1

***Australothyris smithi*:**
Char. 23: 0 → 1
Char. 34: 0 → 1
Char. 55: 1 → 0
Char. 71: 1 → 0
Char. 85: 1 → 0
Char. 112: 0 → 1
Char. 129: 0 → 1
Char. 131: 0 → 1
Char. 159: 1 → 0

***Barasaurus besairiei*:**
Char. 33: 1 → 0
Char. 75: 1 → 0
Char. 216: 0 → 1

***Bashkyroleter bashkyricus*:**
Char. 275: 1 → 0

***Bashkyroleter mesensis*:**
Char. 169: 1 → 0

***Belebey vegrandis*:**
Char. 154: 1 → 0

***Bradysaurus* spp.:**
Char. 19: 0 → 1
Char. 73: 0 → 1
Char. 79: 0 → 1
Char. 135: 1 → 0
Char. 249: 0 → 1

**Captorhinidae:**
Char. 3: 0 → 1
Char. 23: 0 → 1
Char. 25: 1 → 0
Char. 26: 0 → 1
Char. 73: 0 → 1
Char. 75: 0 → 1
Char. 83: 0 → 1
Char. 108: 1 → 0
Char. 183: 1 → 0
Char. 201: 1 → 0
Char. 203: 1 → 2
Char. 216: 1 → 0

**Caseidae:**
Char. 24: 0 → 1
Char. 25: 1 → 0
Char. 36: 0 → 1
Char. 38: 1 → 0
Char. 46: 1 → 0
Char. 50: 1 → 0
Char. 56: 0 → 1
Char. 85: 1 → 0
Char. 96: 1 → 0
Char. 98: 1 → 0
Char. 170: 0 → 1
Char. 194: 0 → 1
Char. 273: 0 → 1
Char. 274: 0 → 1
Char. 278: 3 → 2

***Claudiosaurus germaini*:**
Char. 24: 0 → 1
Char. 34: 0 → 1
Char. 36: 0 → 1
Char. 43: 1 → 0
Char. 64: 0 → 1
Char. 105: 0 → 1
Char. 130: 0 → 1
Char. 144: 1 → 0
Char. 166: 1 → 0
Char. 187: 1 → 0
Char. 199: 0 → 1
Char. 203: 1 → 2
Char. 204: 0 → 1
Char. 220: 0 → 1
Char. 222: 1 → 0

***Colobomycter pholeter*:**
Char. 21: 0 → 1
Char. 25: 1 → 0
Char. 84: 1 → 0
Char. 154: 1 → 0
Char. 167: 0 → 1
Char. 267: 0 → 1

***Delorhynchus cifelli*:**
Char. 18: 0 → 1
Char. 20: 0 → 1
Char. 21: 0 → 1
Char. 24: 0 → 1
Char. 26: 0 → 1
Char. 28: 0 → 1
Char. 33: 0 → 2
Char. 39: 0 → 1
Char. 48: 0 → 1
Char. 52: 0 → 1
Char. 100: 0 → 1
Char. 111: 0 → 1
Char. 116: 1 → 0
Char. 117: 0 → 1
Char. 119: 1 → 0
Char. 131: 0 → 1
Char. 156: 1 → 0
Char. 167: 0 → 1
Char. 189: 0 → 1
Char. 191: 0 → 1
Char. 192: 0 → 1
Char. 267: 0 → 1

**Diadectomorpha:**
Char. 0: 0 → 1
Char. 70: 0 → 1
Char. 75: 0 → 1
Char. 122: 0 → 1
Char. 123: 1 → 0
Char. 146: 1 → 0
Char. 275: 1 → 0
Char. 278: 3 → 0

***Emeroleter levis*:**
Char. 0: 0 → 2
Char. 51: 1 → 0

**Eosauropterygia:**
Char. 159: 1 → 0
Char. 166: 1 → 0
Char. 174: 0 → 1
Char. 178: 0 → 1
Char. 272: 1 → 0

***Eudibamus cursoris*:**
Char. 154: 1 → 2
Char. 163: 1 → 0

***Feeserpeton oklahomensis*:**
Char. 51: 0 → 1
Char. 70: 0 → 1
Char. 157: 0 → 1
Char. 158: 0 → 1

***Hovasaurus boulei*:**
Char. 41: 0 → 1
Char. 55: 1 → 0
Char. 60: 1 → 0
Char. 72: 2 → 1
Char. 77: 0 → 2
Char. 78: 1 → 0
Char. 79: 0 → 1
Char. 93: 1 → 0
Char. 113: 0 → 1
Char. 138: 0 → 1
Char. 146: 1 → 0
Char. 154: 0 → 1
Char. 204: 0 → 2
Char. 206: 0 → 2
Char. 220: 0 → 1
Char. 278: 01 → 3

**Kuehneosauridae:**
Char. 7: 0 → 1
Char. 24: 0 → 1
Char. 26: 0 → 1
Char. 27: 0 → 1
Char. 34: 0 → 1
Char. 36: 0 → 1
Char. 43: 1 → 0
Char. 79: 0 → 2
Char. 82: 0 → 1
Char. 98: 1 → 0
Char. 107: 1 → 0
Char. 108: 1 → 0
Char. 113: 0 → 1
Char. 128: 0 → 1
Char. 140: 1 → 0
Char. 147: 1 → 0
Char. 148: 1 → 0
Char. 159: 1 → 0
Char. 181: 0 → 1
Char. 185: 0 → 1
Char. 206: 0 → 2
Char. 245: 0 → 1
Char. 272: 1 → 2
Char. 278: 0 → 3

***Lanthanosuchus watsoni*:**
Char. 25: 1 → 0
Char. 51: 0 → 1
Char. 86: 0 → 1
Char. 95: 0 → 1
Char. 98: 1 → 0
Char. 110: 0 → 1
Char. 113: 0 → 1
Char. 114: 0 → 1
Char. 119: 1 → 0
Char. 131: 0 → 1
Char. 137: 0 → 1
Char. 138: 0 → 1
Char. 140: 0 → 2
Char. 144: 1 → 0
Char. 154: 1 → 2
Char. 192: 0 → 1

***Macroleter poezicus*:**
Char. 0: 0 → 1
Char. 52: 0 → 1
Char. 66: 1 → 2
Char. 84: 1 → 0
Char. 87: 0 → 1
Char. 140: 0 → 1
Char. 146: 1 → 0
Char. 169: 1 → 0
Char. 235: 0 → 1

***Mesosaurus* spp.:**
Char. 0: 0 → 1
Char. 2: 0 → 1
Char. 5: 1 → 0
Char. 6: 0 → 1
Char. 8: 0 → 1
Char. 9: 0 → 1
Char. 13: 0 → 1
Char. 19: 0 → 1
Char. 23: 0 → 1
Char. 26: 0 → 1
Char. 29: 1 → 0
Char. 33: 0 → 1
Char. 38: 1 → 0
Char. 41: 0 → 1
Char. 48: 0 → 1
Char. 50: 1 → 0
Char. 67: 0 → 1
Char. 83: 0 → 1
Char. 84: 1 → 0
Char. 85: 1 → 0
Char. 94: 0 → 1
Char. 107: 0 → 1
Char. 109: 0 → 1
Char. 111: 0 → 1
Char. 115: 0 → 1
Char. 146: 1 → 0
Char. 147: 1 → 0
Char. 148: 0 → 1
Char. 149: 1 → 0
Char. 159: 1 → 0
Char. 163: 1 → 0
Char. 164: 0 → 1
Char. 166: 1 → 0
Char. 167: 0 → 1
Char. 176: 0 → 1
Char. 183: 1 → 0
Char. 184: 0 → 1
Char. 199: 0 → 1
Char. 202: 0 → 1
Char. 206: 0 → 1
Char. 207: 0 → 1
Char. 209: 0 → 1
Char. 217: 0 → 1
Char. 219: 0 → 1
Char. 220: 0 → 1
Char. 231: 0 → 1
Char. 260: 2 → 0
Char. 272: 2 → 0
Char. 278: 3 → 0

***Microleter mckinzieorum*:**
Char. 0: 0 → 1
Char. 25: 1 → 0
Char. 36: 0 → 1
Char. 39: 0 → 1
Char. 51: 0 → 2
Char. 56: 0 → 1
Char. 70: 0 → 1
Char. 94: 0 → 1
Char. 106: 0 → 1
Char. 166: 1 → 0
Char. 276: 0 → 1

**Millerettidae:**
Char. 5: 1 → 0
Char. 24: 0 → 1
Char. 25: 1 → 0
Char. 44: 1 → 0
Char. 56: 0 → 1
Char. 57: 1 → 0
Char. 66: 0 → 2
Char. 78: 1 → 0
Char. 80: 1 → 0
Char. 84: 1 → 2
Char. 88: 1 → 0
Char. 96: 1 → 0
Char. 117: 0 → 1
Char. 124: 0 → 1
Char. 127: 0 → 1
Char. 135: 0 → 1
Char. 145: 0 → 1
Char. 147: 1 → 0
Char. 159: 1 → 0
Char. 163: 1 → 0
Char. 166: 1 → 0
Char. 202: 0 → 1
Char. 211: 0 → 1
Char. 230: 0 → 1
Char. 240: 0 → 1
Char. 248: 0 → 1
Char. 252: 0 → 1
Char. 253: 0 → 1

***Nycteroleter ineptus*:**
Char. 278: 0 → 3

***Nyctiphruretus acudens*:**
Char. 0: 0 → 1
Char. 21: 0 → 1
Char. 33: 1 → 2
Char. 41: 0 → 1
Char. 66: 1 → 2
Char. 81: 1 → 0
Char. 83: 0 → 1
Char. 84: 1 → 2
Char. 94: 0 → 1
Char. 166: 1 → 0
Char. 167: 0 → 1
Char. 224: 0 → 1
Char. 266: 0 → 1
Char. 272: 2 → 1
Char. 276: 0 → 1

***Orovenator mayorum*:**
Char. 8: 0 → 1
Char. 24: 0 → 1
Char. 36: 0 → 1
Char. 160: 0 → 1
Char. 165: 0 → 1

***Owenetta* spp.:**
Char. 169: 1 → 0

***Paleothyris acadiana*:**
Char. 38: 1 → 0
Char. 50: 1 → 0
Char. 66: 1 → 2
Char. 102: 0 → 1
Char. 146: 1 → 0
Char. 237: 0 → 1
Char. 239: 0 → 1
Char. 240: 0 → 1

***Placodus* spp.:**
Char. 0: 1 → 2
Char. 9: 0 → 1
Char. 12: 0 → 1
Char. 13: 0 → 1
Char. 19: 0 → 1
Char. 26: 0 → 1
Char. 31: 0 → 1
Char. 46: 1 → 0
Char. 57: 0 → 1
Char. 93: 1 → 0
Char. 109: 1 → 0
Char. 140: 1 → 0
Char. 155: 0 → 1
Char. 163: 1 → 0
Char. 164: 0 → 1

***Procolophon* spp.:**
Char. 41: 0 → 1
Char. 69: 0 → 1
Char. 79: 0 → 1
Char. 83: 0 → 1
Char. 88: 0 → 1
Char. 117: 1 → 0
Char. 149: 1 → 0
Char. 237: 0 → 1
Char. 238: 0 → 1
Char. 272: 2 → 1
Char. 278: 3 → 0

***Prolacerta broomi*:**
Char. 58: 1 → 0
Char. 66: 1 → 0
Char. 67: 1 → 0
Char. 80: 1 → 0
Char. 123: 0 → 1
Char. 139: 1 → 0
Char. 147: 1 → 0
Char. 192: 1 → 0
Char. 203: 1 → 2
Char. 206: 0 → 12

***Rhipaeosaurus* spp.:**
Char. 172: 0 → 1
Char. 277: 0 → 1

**Rhynchocephalia:**
Char. 0: 1 → 2
Char. 23: 1 → 0
Char. 24: 0 → 1
Char. 77: 0 → 1
Char. 88: 1 → 0
Char. 94: 1 → 0
Char. 104: 0 → 1
Char. 117: 12 → 0
Char. 139: 1 → 0
Char. 167: 1 → 0
Char. 205: 1 → 0

**Rhynchosauria:**
Char. 0: 1 → 0
Char. 7: 0 → 1
Char. 9: 0 → 1
Char. 26: 0 → 1
Char. 63: 1 → 0
Char. 68: 0 → 1
Char. 99: 1 → 0
Char. 123: 0 → 1
Char. 150: 1 → 0
Char. 160: 0 → 1
Char. 161: 0 → 1
Char. 171: 0 → 2
Char. 182: 1 → 0
Char. 241: 0 → 1

***Scutosaurus* spp.:**
Char. 175: 0 → 1
Char. 218: 0 → 1
Char. 243: 0 → 2
Char. 244: 0 → 1
Char. 251: 0 → 1

***Sinosaurosphargis yunguiensis*:**
Char. 8: 0 → 1
Char. 30: 0 → 1
Char. 53: 0 → 1
Char. 82: 0 → 1
Char. 89: 1 → 0
Char. 127: 1 → 0
Char. 150: 1 → 0
Char. 154: 0 → 2
Char. 167: 1 → 0
Char. 253: 0 → 1
Char. 255: 0 → 1

**Squamata:**
Char. 26: 0 → 1
Char. 45: 0 → 1
Char. 79: 0 → 2
Char. 80: 1 → 0
Char. 82: 0 → 1
Char. 92: 1 → 0
Char. 109: 1 → 0
Char. 122: 0 → 1
Char. 160: 0 → 1
Char. 200: 0 → 1
Char. 245: 0 → 1

***Trilophosaurus buettneri*:**
Char. 5: 1 → 0
Char. 11: 0 → 1
Char. 55: 1 → 0
Char. 93: 1 → 0
Char. 104: 0 → 1
Char. 113: 0 → 1
Char. 122: 0 → 1
Char. 136: 1 → 0
Char. 144: 1 → 0
Char. 154: 0 → 1
Char. 157: 0 → 1
Char. 159: 1 → 0
Char. 177: 0 → 12
Char. 194: 0 → 1
Char. 203: 1 → 2
Char. 207: 1 → 0
Char. 208: 1 → 0
Char. 272: 1 → 0

***Youngina capensis*:**
Char. 5: 1 → 0
Char. 75: 0 → 1
Char. 92: 1 → 0
Char. 163: 1 → 0

Char. 170: 0 → 1
Char. 211: 0 → 1
Char. 231: 0 → 1
Char. 239: 0 → 1

**Node 50:**
Char. 15: 1 → 0
Char. 20: 1 → 0
Char. 25: 0 → 1
Char. 33: 2 → 0
Char. 67: 1 → 0
Char. 79: 2 → 1
Char. 127: 1 → 0
Char. 145: 1 → 0
Char. 147: 1 → 0
Char. 149: 1 → 0
Char. 174: 1 → 0
Char. 176: 1 → 0
Char. 180: 1 → 0
Char. 184: 1 → 0
Char. 187: 1 → 0
Char. 188: 1 → 0
Char. 189: 1 → 0
Char. 191: 1 → 0
Char. 192: 1 → 0
Char. 194: 2 → 0
Char. 195: 2 → 0
Char. 198: 1 → 0
Char. 205: 1 → 0
Char. 208: 1 → 0
Char. 210: 1 → 0
Char. 220: 1 → 0
Char. 221: 1 → 0
Char. 222: 1 → 0
Char. 223: 1 → 0
Char. 224: 1 → 0
Char. 226: 1 → 0
Char. 227: 1 → 0
Char. 231: 1 → 0
Char. 232: 1 → 0
Char. 237: 1 → 0
Char. 241: 1 → 0
Char. 246: 2 → 0
Char. 247: 1 → 0
Char. 251: 1 → 0
Char. 253: 2 → 0
Char. 254: 1 → 0
Char. 255: 1 → 0
Char. 256: 1 → 0
Char. 258: 1 → 0
Char. 259: 1 → 0
Char. 268: 1 → 0
Char. 269: 1 → 0
Char. 270: 1 → 0
Char. 272: 1 → 2

**Node 51:**
Char. 1: 0 → 1
Char. 55: 1 → 0
Char. 193: 1 → 0
Char. 206: 0 → 2
Char. 220: 0 → 1
Char. 239: 1 → 0

**Node 52:**
Char. 27: 1 → 0
Char. 58: 0 → 1
Char. 61: 0 → 1
Char. 66: 0 → 1
Char. 69: 0 → 1
Char. 84: 0 → 1
Char. 107: 0 → 1
Char. 123: 1 → 0
Char. 140: 0 → 1
Char. 147: 0 → 1
Char. 150: 0 → 1
Char. 167: 0 → 1
Char. 184: 0 → 1
Char. 205: 0 → 1
Char. 208: 0 → 1
Char. 210: 0 → 1
Char. 230: 0 → 1
Char. 239: 0 → 1

**Node 53:**
Char. 70: 0 → 1
Char. 73: 0 → 1
Char. 126: 0 → 1
Char. 131: 0 → 1
Char. 148: 0 → 1
Char. 182: 0 → 1
Char. 190: 0 → 1
Char. 272: 2 → 1

**Node 54:**
Char. 33: 0 → 1
Char. 92: 0 → 1
Char. 135: 0 → 1

**Node 55:**
Char. 20: 0 → 1
Char. 62: 0 → 1

**Node 56:**
Char. 0: 0 → 1
Char. 27: 0 → 1
Char. 29: 1 → 0
Char. 38: 1 → 0
Char. 40: 2 → 0
Char. 57: 1 → 0
Char. 59: 0 → 1
Char. 60: 0 → 1
Char. 67: 0 → 1
Char. 72: 1 → 2
Char. 84: 1 → 0
Char. 89: 0 → 1
Char. 111: 0 → 1
Char. 112: 0 → 1
Char. 120: 0 → 1
Char. 147: 1 → 0
Char. 154: 1 → 0
Char. 192: 0 → 1
Char. 193: 0 → 1
Char. 222: 0 → 1
Char. 237: 0 → 1
Char. 240: 0 → 1
Char. 266: 0 → 1
Char. 278: 3 → 1

**Node 57:**
Char. 5: 0 → 1
Char. 29: 0 → 1
Char. 40: 0 → 2
Char. 74: 0 → 1
Char. 88: 0 → 1
Char. 116: 0 → 1
Char. 119: 0 → 1
Char. 147: 0 → 1
Char. 149: 0 → 1
Char. 163: 0 → 1
Char. 166: 0 → 1
Char. 169: 0 → 1
Char. 201: 0 → 1
Char. 203: 0 → 1
Char. 221: 0 → 1
Char. 235: 0 → 1
Char. 276: 1 → 0

**Node 58:**
Char. 79: 1 → 0
Char. 81: 0 → 1
Char. 93: 0 → 1

**Node 59:**
Char. 78: 0 → 1
Char. 111: 1 → 0
Char. 126: 1 → 0
Char. 135: 1 → 0

**Node 60:**
Char. 19: 0 → 1
Char. 92: 1 → 0

**Node 61:**
Char. 4: 0 → 1
Char. 15: 0 → 1
Char. 29: 0 → 1
Char. 35: 1 → 0
Char. 48: 1 → 0
Char. 210: 1 → 0
Char. 213: 0 → 1
Char. 226: 0 → 2
Char. 228: 0 → 1
Char. 275: 0 → 1

**Node 62:**
Char. 24: 0 → 1
Char. 57: 1 → 0
Char. 79: 0 → 1
Char. 83: 0 → 1
Char. 110: 0 → 1
Char. 132: 1 → 0

**Node 63:**
Char. 73: 0 → 1
Char. 131: 1 → 0
Char. 205: 0 → 1
Char. 239: 0 → 1
Char. 276: 0 → 1

**Node 64:**
Char. 18: 0 → 1
Char. 37: 0 → 1
Char. 107: 0 → 1
Char. 118: 1 → 0
Char. 125: 1 → 0
Char. 150: 0 → 1

**Node 65:**
Char. 23: 0 → 1
Char. 71: 1 → 0
Char. 102: 0 → 1
Char. 103: 0 → 1
Char. 106: 0 → 1
Char. 167: 0 → 1
Char. 214: 0 → 1
Char. 216: 1 → 0
Char. 235: 0 → 2
Char. 241: 0 → 1

**Node 66:**
Char. 138: 0 → 1
Char. 141: 0 → 1
Char. 207: 0 → 1

**Node 67:**
Char. 20: 0 → 1
Char. 33: 0 → 1
Char. 38: 1 → 2
Char. 39: 0 → 1
Char. 44: 1 → 0
Char. 49: 0 → 1
Char. 51: 0 → 1
Char. 66: 0 → 1
Char. 70: 0 → 1
Char. 80: 1 → 0
Char. 88: 1 → 0
Char. 95: 0 → 1
Char. 112: 0 → 1
Char. 117: 0 → 1
Char. 118: 0 → 1
Char. 125: 0 → 1
Char. 131: 0 → 1
Char. 137: 0 → 1
Char. 148: 0 → 1
Char. 158: 0 → 1
Char. 183: 1 → 2
Char. 192: 0 → 1
Char. 194: 0 → 1
Char. 201: 1 → 0
Char. 211: 0 → 1
Char. 235: 1 → 0
Char. 252: 0 → 1

**Node 68:**
Char. 87: 0 → 1

**Node 69:**
Char. 25: 1 → 0
Char. 76: 1 → 0

**Node 70:**
Char. 79: 0 → 1
Char. 93: 1 → 0
Char. 133: 0 → 1

**Node 71:**
Char. 147: 1 → 2
Char. 154: 1 → 0
Char. 239: 0 → 1
Char. 278: 3 → 0

**Node 72:**
Char. 38: 1 → 2
Char. 39: 0 → 1
Char. 50: 1 → 0
Char. 58: 0 → 1
Char. 59: 0 → 1
Char. 60: 0 → 1
Char. 72: 1 → 2
Char. 83: 0 → 1
Char. 85: 1 → 0
Char. 88: 1 → 0
Char. 95: 0 → 1
Char. 104: 0 → 1
Char. 105: 0 → 1
Char. 106: 0 → 2
Char. 107: 0 → 1
Char. 109: 0 → 1
Char. 110: 0 → 1
Char. 146: 1 → 0
Char. 148: 0 → 1
Char. 155: 0 → 2
Char. 183: 1 → 2

**Node 73:**
Char. 0: 0 → 2
Char. 33: 1 → 0
Char. 38: 2 → 1
Char. 39: 1 → 0
Char. 42: 0 → 1
Char. 43: 0 → 1
Char. 46: 1 → 0
Char. 49: 1 → 0
Char. 52: 0 → 1
Char. 83: 0 → 2
Char. 84: 1 → 0
Char. 87: 0 → 1
Char. 93: 1 → 0
Char. 161: 0 → 1
Char. 163: 1 → 0
Char. 172: 0 → 2
Char. 174: 0 → 1
Char. 188: 0 → 1
Char. 189: 0 → 1
Char. 195: 0 → 1
Char. 212: 0 → 1
Char. 221: 1 → 0
Char. 236: 0 → 1
Char. 238: 0 → 2
Char. 242: 0 → 1
Char. 245: 0 → 1
Char. 274: 0 → 1
Char. 275: 1 → 0

**Node 74:**
Char. 5: 1 → 0
Char. 29: 1 → 0
Char. 44: 1 → 0
Char. 59: 0 → 1
Char. 60: 0 → 1
Char. 66: 0 → 1
Char. 67: 0 → 1

Char. 72: 1 → 2
Char. 78: 1 → 0
Char. 84: 1 → 0
Char. 88: 1 → 0
Char. 93: 1 → 0
Char. 111: 0 → 1
Char. 123: 1 → 0
Char. 129: 0 → 1
Char. 147: 1 → 0
Char. 154: 1 → 0
Char. 159: 1 → 0
Char. 163: 1 → 0
Char. 169: 1 → 0
Char. 170: 0 → 1
Char. 197: 0 → 1
Char. 221: 1 → 0

**Node 75:**
Char. 20: 0 → 1
Char. 47: 0 → 1
Char. 83: 0 → 1
Char. 85: 1 → 0
Char. 107: 0 → 1
Char. 110: 0 → 1
Char. 169: 1 → 0
Char. 170: 0 → 1

**Node 76:**
Char. 2: 0 → 1
Char. 6: 0 → 1
Char. 198: 0 → 1
Char. 240: 1 → 0
Char. 266: 1 → 0
Char. 267: 1 → 0

**Node 77:**
Char. 17: 0 → 1
Char. 42: 0 → 1
Char. 251: 0 → 1
Char. 264: 0 → 1

**Node 78:**
Char. 33: 1 → 2
Char. 48: 1 → 0
Char. 61: 1 → 2
Char. 68: 0 → 1
Char. 112: 1 → 0
Char. 128: 0 → 1
Char. 138: 0 → 1
Char. 146: 1 → 0
Char. 155: 0 → 1
Char. 158: 0 → 1
Char. 182: 1 → 0
Char. 192: 1 → 2
Char. 226: 0 → 1
Char. 227: 0 → 1
Char. 233: 0 → 1
Char. 235: 1 → 2
Char. 267: 1 → 2

**Node 79:**
Char. 61: 1 → 3
Char. 90: 0 → 1
Char. 91: 0 → 1
Char. 109: 1 → 0
Char. 155: 0 → 1
Char. 209: 1 → 0

**ANALYSIS 21**

**(NO *PROGANOCHELYS QUENSTEDTI*, *CANDELARIA BARBOURI*, AND *EUNOTOSAURUS AFRICANUS*)**

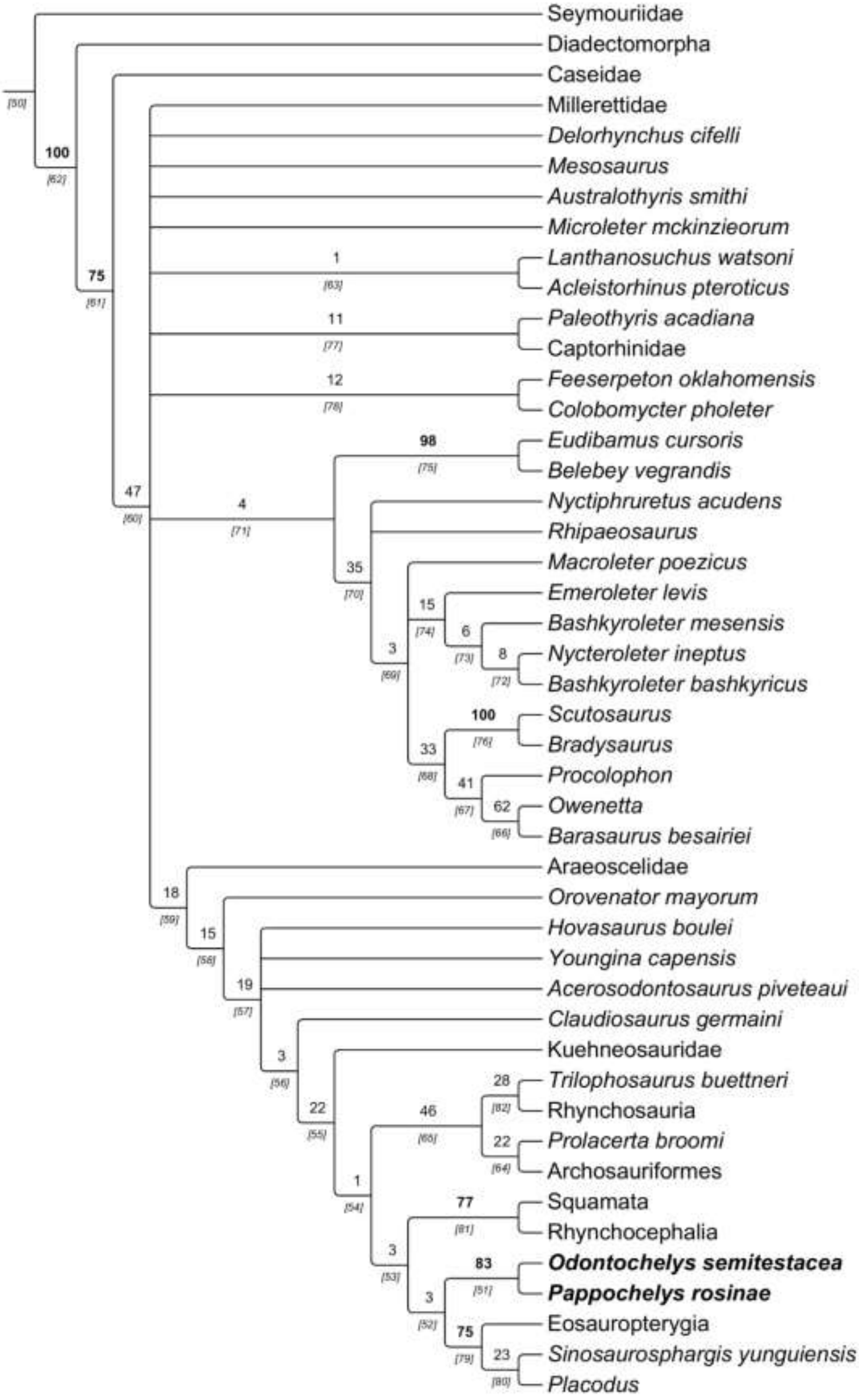

**Seymouriidae:**
Char. 23: 0 → 1
Char. 51: 0 → 2
Char. 54: 1 → 0
Char. 71: 1 → 0
Char. 83: 0 → 1
Char. 85: 1 → 0
Char. 99: 1 → 0
Char. 107: 0 → 1
Char. 140: 0 → 2
Char. 154: 1 → 2
Char. 207: 0 → 1
Char. 225: 1 → 0
Char. 260: 2 → 0

***Pappochelys rosinae*:**
Char. 5: 1 → 0
Char. 12: 0 → 1
Char. 41: 0 → 1
Char. 43: 1 → 0
Char. 49: 0 → 1
Char. 169: 1 → 0
Char. 176: 1 → 0

***Odontochelys semitestacea*:**
Char. 46: 1 → 0
Char. 50: 0 → 1
Char. 75: 1 → 0
Char. 89: 1 → 0
Char. 93: 1 → 0
Char. 126: 1 → 0
Char. 195: 0 → 2
Char. 246: 1 → 2
Char. 259: 0 → 1
Char. 265: 1 → 0
Char. 270: 0 → 1

***Acerosodontosaurus piveteaui*:**
Char. 78: 1 → 0
Char. 81: 1 → 0
Char. 128: 0 → 1
Char. 155: 0 → 1
Char. 206: 0 → 2
Char. 208: 0 → 1
Char. 224: 1 → 0

***Acleistorhinus pteroticus*:**
Char. 21: 0 → 1
Char. 146: 1 → 0
Char. 169: 1 → 0
Char. 170: 0 → 1

**Araeoscelidae:**
Char. 28: 0 → 1
Char. 106: 0 → 1
Char. 116: 1 → 0
Char. 169: 1 → 0
Char. 170: 0 → 1
Char. 197: 0 → 1
Char. 239: 0 → 1

**Archosauriformes:**
Char. 32: 0 → 1
Char. 94: 1 → 0
Char. 112: 1 → 0
Char. 152: 0 → 1
Char. 154: 0 → 2
Char. 166: 1 → 0
Char. 171: 0 → 1
Char. 185: 0 → 1
Char. 204: 0 → 1
Char. 218: 0 → 3
Char. 242: 0 → 1

***Australothyris smithi*:**
Char. 23: 0 → 1
Char. 24: 0 → 1
Char. 34: 0 → 1
Char. 55: 1 → 0
Char. 57: 1 → 0
Char. 71: 1 → 0
Char. 79: 0 → 1
Char. 85: 1 → 0
Char. 98: 1 → 0
Char. 100: 0 → 1
Char. 103: 0 → 1
Char. 110: 0 → 1
Char. 112: 0 → 1
Char. 123: 1 → 0
Char. 129: 0 → 1
Char. 131: 0 → 1
Char. 132: 1 → 0
Char. 144: 1 → 0
Char. 147: 0 → 1
Char. 149: 1 → 0
Char. 150: 0 → 1

***Barasaurus besairiei*:**
Char. 33: 1 → 0
Char. 75: 1 → 0
Char. 216: 0 → 1

***Bashkyroleter bashkyricus*:**
Char. 275: 1 → 0

***Bashkyroleter mesensis*:**
Char. 169: 1 → 0

***Belebey vegrandis*:**
Char. 154: 1 → 0

***Bradysaurus* spp.:**
Char. 19: 0 → 1
Char. 73: 0 → 1
Char. 79: 0 → 1
Char. 135: 1 → 0
Char. 249: 0 → 1

**Captorhinidae:**
Char. 3: 0 → 1
Char. 23: 0 → 1
Char. 25: 1 → 0
Char. 26: 0 → 1
Char. 73: 0 → 1
Char. 75: 0 → 1
Char. 108: 1 → 0
Char. 183: 1 → 0
Char. 201: 1 → 0
Char. 203: 1 → 2
Char. 216: 1 → 0

**Caseidae:**
Char. 24: 0 → 1
Char. 25: 1 → 0
Char. 36: 0 → 1
Char. 38: 1 → 0
Char. 46: 1 → 0
Char. 50: 1 → 0
Char. 56: 0 → 1
Char. 85: 1 → 0
Char. 96: 1 → 0
Char. 98: 1 → 0
Char. 170: 0 → 1
Char. 194: 0 → 1
Char. 273: 0 → 1
Char. 274: 0 → 1
Char. 278: 3 → 2

***Claudiosaurus germaini*:**
Char. 64: 0 → 1
Char. 105: 0 → 1
Char. 106: 0 → 1
Char. 130: 0 → 1
Char. 144: 1 → 0
Char. 187: 1 → 0
Char. 199: 0 → 1
Char. 203: 1 → 2
Char. 204: 0 → 1
Char. 220: 0 → 1
Char. 222: 1 → 0
Char. 224: 1 → 0

***Colobomycter pholeter*:**
Char. 21: 0 → 1
Char. 25: 1 → 0
Char. 84: 1 → 0
Char. 154: 1 → 0
Char. 167: 0 → 1
Char. 267: 0 → 1

***Delorhynchus cifelli*:**
Char. 18: 0 → 1
Char. 20: 0 → 1
Char. 21: 0 → 1
Char. 24: 0 → 1
Char. 26: 0 → 1
Char. 28: 0 → 1
Char. 33: 0 → 2
Char. 39: 0 → 1
Char. 52: 0 → 1
Char. 100: 0 → 1
Char. 111: 0 → 1
Char. 116: 1 → 0
Char. 117: 0 → 1
Char. 131: 0 → 1
Char. 147: 0 → 1
Char. 156: 1 → 0
Char. 159: 0 → 1
Char. 167: 0 → 1
Char. 180: 0 → 1
Char. 189: 0 → 1
Char. 191: 0 → 1
Char. 192: 0 → 1
Char. 204: 0 → 2
Char. 267: 0 → 1

**Diadectomorpha:**
Char. 0: 0 → 1
Char. 70: 0 → 1
Char. 75: 0 → 1
Char. 122: 0 → 1
Char. 123: 1 → 0
Char. 146: 1 → 0
Char. 275: 1 → 0
Char. 278: 3 → 0

***Emeroleter levis*:**
Char. 51: 1 → 0

**Eosauropterygia:**
Char. 159: 1 → 0
Char. 166: 1 → 0
Char. 168: 0 → 1
Char. 178: 0 → 1
Char. 272: 1 → 0

***Eudibamus cursoris*:**
Char. 154: 1 → 2

***Feeserpeton oklahomensis*:**
Char. 51: 0 → 1
Char. 70: 0 → 1
Char. 157: 0 → 1
Char. 158: 0 → 1

***Hovasaurus boulei*:**
Char. 41: 0 → 1
Char. 55: 1 → 0
Char. 60: 1 → 0
Char. 72: 2 → 1
Char. 77: 0 → 2
Char. 78: 1 → 0
Char. 79: 0 → 1
Char. 93: 1 → 0
Char. 113: 0 → 1
Char. 138: 0 → 1
Char. 146: 1 → 0
Char. 154: 0 → 1
Char. 204: 0 → 2
Char. 206: 0 → 2
Char. 220: 0 → 1
Char. 278: 01 → 3

**Kuehneosauridae:**
Char. 7: 0 → 1
Char. 26: 0 → 1
Char. 79: 0 → 2
Char. 82: 0 → 1
Char. 98: 1 → 0
Char. 108: 1 → 0
Char. 113: 0 → 1
Char. 128: 0 → 1
Char. 159: 1 → 0
Char. 181: 0 → 1
Char. 185: 0 → 1
Char. 206: 0 → 2
Char. 245: 0 → 1
Char. 278: 0 → 3

***Lanthanosuchus watsoni*:**
Char. 25: 1 → 0
Char. 51: 0 → 1
Char. 76: 0 → 1
Char. 86: 0 → 1
Char. 98: 1 → 0
Char. 138: 0 → 1
Char. 144: 1 → 0
Char. 154: 1 → 2

***Macroleter poezicus*:**
Char. 9: 0 → 1
Char. 26: 0 → 1
Char. 52: 0 → 1
Char. 84: 1 → 0
Char. 87: 0 → 1
Char. 134: 0 → 2
Char. 139: 0 → 1
Char. 140: 0 → 1
Char. 146: 1 → 0
Char. 169: 1 → 0
Char. 235: 0 → 1

***Mesosaurus* spp.:**
Char. 0: 0 → 1
Char. 2: 0 → 1
Char. 6: 0 → 1
Char. 8: 0 → 1
Char. 9: 0 → 1
Char. 13: 0 → 1
Char. 19: 0 → 1
Char. 23: 0 → 1
Char. 26: 0 → 1
Char. 29: 1 → 0
Char. 33: 0 → 1
Char. 38: 1 → 0
Char. 41: 0 → 1
Char. 50: 1 → 0
Char. 67: 0 → 1
Char. 76: 0 → 1
Char. 84: 1 → 0
Char. 85: 1 → 0
Char. 94: 0 → 1
Char. 107: 0 → 1
Char. 109: 0 → 1
Char. 111: 0 → 1
Char. 115: 0 → 1
Char. 146: 1 → 0
Char. 148: 0 → 1
Char. 149: 1 → 0
Char. 164: 0 → 1
Char. 167: 0 → 1
Char. 176: 0 → 1
Char. 183: 1 → 0
Char. 184: 0 → 1
Char. 199: 0 → 1
Char. 202: 0 → 1
Char. 204: 0 → 2
Char. 206: 0 → 1
Char. 207: 0 → 1
Char. 209: 0 → 1

Char. 217: 0 → 1
Char. 219: 0 → 1
Char. 220: 0 → 1
Char. 231: 0 → 1
Char. 260: 2 → 0
Char. 272: 2 → 0
Char. 278: 3 → 0

***Microleter mckinzieorum*:**
Char. 0: 0 → 1
Char. 18: 0 → 1
Char. 24: 0 → 1
Char. 25: 1 → 0
Char. 36: 0 → 1
Char. 39: 0 → 1
Char. 51: 0 → 2
Char. 56: 0 → 1
Char. 57: 1 → 0
Char. 70: 0 → 1
Char. 76: 0 → 1
Char. 79: 0 → 1
Char. 94: 0 → 1
Char. 106: 0 → 1
Char. 110: 0 → 1
Char. 132: 1 → 0
Char. 159: 0 → 1
Char. 276: 0 → 1
Char. 278: 3 → 1

**Millerettidae:**
Char. 24: 0 → 1
Char. 25: 1 → 0
Char. 44: 1 → 0
Char. 56: 0 → 1
Char. 57: 1 → 0
Char. 66: 0 → 2
Char. 78: 1 → 0
Char. 80: 1 → 0
Char. 84: 1 → 2
Char. 88: 1 → 0
Char. 96: 1 → 0
Char. 117: 0 → 1
Char. 124: 0 → 1
Char. 127: 0 → 1
Char. 135: 0 → 1
Char. 145: 0 → 1
Char. 202: 0 → 1
Char. 211: 0 → 1
Char. 230: 0 → 1
Char. 234: 0 → 1
Char. 248: 0 → 1
Char. 252: 0 → 1
Char. 253: 0 → 1

***Nycteroleter ineptus*:**
Char. 278: 0 → 3

***Nyctiphruretus acudens*:**
Char. 21: 0 → 1
Char. 33: 1 → 2
Char. 41: 0 → 1
Char. 81: 1 → 0
Char. 84: 1 → 2
Char. 94: 0 → 1
Char. 167: 0 → 1
Char. 215: 1 → 0
Char. 224: 0 → 1
Char. 266: 0 → 1
Char. 272: 2 → 1
Char. 276: 0 → 1

***Orovenator mayorum*:**
Char. 8: 0 → 1
Char. 24: 0 → 1
Char. 36: 0 → 1
Char. 160: 0 → 1
Char. 165: 0 → 1

***Owenetta* spp.:**
Char. 169: 1 → 0

***Paleothyris acadiana*:**
Char. 38: 1 → 0
Char. 50: 1 → 0
Char. 66: 1 → 2
Char. 102: 0 → 1
Char. 146: 1 → 0
Char. 180: 0 → 1
Char. 237: 0 → 1
Char. 239: 0 → 1

***Placodus* spp.:**
Char. 9: 0 → 1
Char. 12: 0 → 1
Char. 13: 0 → 1
Char. 19: 0 → 1
Char. 26: 0 → 1
Char. 31: 0 → 1
Char. 46: 1 → 0
Char. 57: 0 → 1
Char. 93: 1 → 0
Char. 102: 1 → 2
Char. 109: 1 → 0
Char. 140: 1 → 0
Char. 155: 0 → 1
Char. 163: 1 → 0
Char. 164: 0 → 1

***Procolophon* spp.:**
Char. 41: 0 → 1
Char. 69: 0 → 1
Char. 79: 0 → 1
Char. 83: 0 → 1
Char. 88: 0 → 1
Char. 117: 1 → 0
Char. 149: 1 → 0
Char. 180: 0 → 1
Char. 204: 0 → 1
Char. 237: 0 → 1
Char. 238: 0 → 1
Char. 272: 2 → 1

***Prolacerta broomi*:**
Char. 58: 1 → 0
Char. 66: 1 → 0
Char. 67: 1 → 0
Char. 80: 1 → 0
Char. 139: 1 → 0
Char. 147: 1 → 0
Char. 192: 1 → 0
Char. 203: 1 → 2
Char. 206: 0 → 12
Char. 224: 1 → 0

***Rhipaeosaurus* spp.:**
Char. 172: 0 → 1
Char. 277: 0 → 1

**Rhynchocephalia:**
Char. 23: 1 → 0
Char. 24: 0 → 1
Char. 75: 1 → 0
Char. 77: 0 → 1
Char. 94: 1 → 0
Char. 104: 0 → 1
Char. 117: 12 → 0
Char. 139: 1 → 0
Char. 167: 1 → 0
Char. 205: 1 → 0

**Rhynchosauria:**
Char. 0: 1 → 0
Char. 7: 0 → 1
Char. 9: 0 → 1
Char. 26: 0 → 1
Char. 68: 0 → 1
Char. 99: 1 → 0
Char. 150: 1 → 0
Char. 160: 0 → 1
Char. 161: 0 → 1
Char. 171: 0 → 2
Char. 223: 0 → 1
Char. 241: 0 → 1

***Scutosaurus* spp.:**
Char. 175: 0 → 1
Char. 218: 0 → 1
Char. 243: 0 → 2
Char. 244: 0 → 1
Char. 251: 0 → 1

***Sinosaurosphargis yunguiensis*:**
Char. 8: 0 → 1
Char. 30: 0 → 1
Char. 53: 0 → 1
Char. 82: 0 → 1
Char. 89: 1 → 0
Char. 127: 1 → 0
Char. 150: 1 → 0
Char. 154: 0 → 2
Char. 167: 1 → 0
Char. 253: 0 → 1
Char. 255: 0 → 1

**Squamata:**
Char. 26: 0 → 1
Char. 45: 0 → 1
Char. 79: 0 → 2
Char. 80: 1 → 0
Char. 82: 0 → 1
Char. 92: 1 → 0
Char. 106: 0 → 1
Char. 109: 1 → 0
Char. 160: 0 → 1
Char. 245: 0 → 1

***Trilophosaurus buettneri*:**
Char. 5: 1 → 0
Char. 11: 0 → 1
Char. 55: 1 → 0
Char. 93: 1 → 0
Char. 104: 0 → 1
Char. 106: 0 → 1
Char. 113: 0 → 1
Char. 122: 0 → 1
Char. 136: 1 → 0
Char. 144: 1 → 0
Char. 154: 0 → 1
Char. 157: 0 → 1
Char. 159: 1 → 0
Char. 177: 0 → 12
Char. 194: 0 → 1
Char. 203: 1 → 2
Char. 207: 1 → 0
Char. 208: 1 → 0
Char. 224: 1 → 0
Char. 272: 1 → 0

***Youngina capensis*:**
Char. 21: 0 → 1
Char. 75: 0 → 1
Char. 92: 1 → 0
Char. 170: 0 → 1
Char. 211: 0 → 1
Char. 231: 0 → 1
Char. 239: 0 → 1

**Node 50:**
Char. 15: 1 → 0
Char. 20: 1 → 0
Char. 25: 0 → 1
Char. 33: 2 → 0
Char. 73: 1 → 0
Char. 79: 2 → 1
Char. 94: 1 → 0
Char. 119: 1 → 0
Char. 127: 1 → 0
Char. 130: 1 → 0
Char. 134: 2 → 0
Char. 136: 1 → 0
Char. 141: 1 → 0
Char. 145: 1 → 0
Char. 147: 1 → 0
Char. 149: 1 → 0
Char. 151: 1 → 0
Char. 152: 1 → 0
Char. 155: 1 → 0
Char. 158: 1 → 0
Char. 161: 1 → 0
Char. 163: 1 → 0
Char. 166: 1 → 0
Char. 174: 1 → 0
Char. 180: 1 → 0
Char. 181: 1 → 0
Char. 188: 1 → 0
Char. 196: 1 → 0
Char. 203: 2 → 0
Char. 204: 2 → 0
Char. 217: 1 → 0
Char. 220: 1 → 0
Char. 221: 1 → 0
Char. 231: 1 → 0
Char. 234: 1 → 0
Char. 247: 1 → 0
Char. 248: 1 → 0
Char. 250: 1 → 0
Char. 251: 1 → 0
Char. 253: 2 → 0

**Node 51:**
Char. 61: 1 → 0
Char. 62: 1 → 0
Char. 65: 0 → 1
Char. 201: 1 → 0
Char. 219: 1 → 0
Char. 241: 0 → 1
Char. 246: 0 → 1
Char. 247: 0 → 1
Char. 252: 0 → 1
Char. 254: 0 → 1
Char. 255: 0 → 1
Char. 256: 0 → 1
Char. 268: 0 → 1
Char. 269: 0 → 1

**Node 52:**
Char. 55: 1 → 0
Char. 189: 0 → 1
Char. 193: 1 → 0
Char. 206: 0 → 2
Char. 220: 0 → 1
Char. 239: 1 → 0

**Node 53:**
Char. 33: 1 → 2
Char. 102: 0 → 1
Char. 103: 0 → 1
Char. 223: 0 → 1
Char. 235: 1 → 2

**Node 54:**
Char. 27: 1 → 0
Char. 75: 0 → 1
Char. 107: 0 → 1
Char. 140: 0 → 1
Char. 147: 0 → 1

**Node 55:**
Char. 58: 0 → 1
Char. 61: 0 → 1
Char. 66: 0 → 1
Char. 69: 0 → 1
Char. 150: 0 → 1
Char. 167: 0 → 1
Char. 205: 0 → 1
Char. 208: 0 → 1
Char. 239: 0 → 1

**Node 56:**
Char. 70: 0 → 1
Char. 73: 0 → 1
Char. 126: 0 → 1

Char. 131: 0 → 1
Char. 190: 0 → 1

**Node 57:**
Char. 33: 0 → 1
Char. 92: 0 → 1
Char. 135: 0 → 1
Char. 159: 0 → 1

**Node 58:**
Char. 20: 0 → 1
Char. 62: 0 → 1

**Node 59:**
Char. 0: 0 → 1
Char. 27: 0 → 1
Char. 29: 1 → 0
Char. 38: 1 → 0
Char. 40: 2 → 0
Char. 57: 1 → 0
Char. 59: 0 → 1
Char. 60: 0 → 1
Char. 67: 0 → 1
Char. 72: 1 → 2
Char. 84: 1 → 0
Char. 89: 0 → 1
Char. 111: 0 → 1
Char. 112: 0 → 1
Char. 120: 0 → 1
Char. 154: 1 → 0
Char. 180: 0 → 1
Char. 192: 0 → 1
Char. 193: 0 → 1
Char. 222: 0 → 1
Char. 224: 0 → 1
Char. 234: 0 → 1
Char. 237: 0 → 1
Char. 266: 0 → 1
Char. 278: 3 → 1

**Node 60:**
Char. 29: 0 → 1
Char. 40: 0 → 2
Char. 74: 0 → 1
Char. 88: 0 → 1
Char. 116: 0 → 1
Char. 149: 0 → 1
Char. 169: 0 → 1
Char. 201: 0 → 1
Char. 203: 0 → 1
Char. 235: 0 → 1
Char. 276: 1 → 0

**Node 61:**
Char. 79: 1 → 0
Char. 81: 0 → 1
Char. 97: 0 → 1

**Node 62:**
Char. 55: 0 → 1
Char. 78: 0 → 1
Char. 111: 1 → 0
Char. 126: 1 → 0
Char. 135: 1 → 0

**Node 63:**
Char. 95: 0 → 1
Char. 110: 0 → 1
Char. 113: 0 → 1
Char. 114: 0 → 1
Char. 131: 0 → 1
Char. 137: 0 → 1
Char. 140: 0 → 2
Char. 147: 0 → 1

**Node 64:**
Char. 19: 0 → 1
Char. 92: 1 → 0

**Node 65:**
Char. 4: 0 → 1
Char. 15: 0 → 1
Char. 29: 0 → 1
Char. 213: 0 → 1
Char. 228: 0 → 1
Char. 275: 0 → 1

**Node 66:**
Char. 73: 0 → 1
Char. 131: 1 → 0
Char. 205: 0 → 1
Char. 276: 0 → 1

**Node 67:**
Char. 18: 0 → 1
Char. 37: 0 → 1
Char. 100: 1 → 0
Char. 107: 0 → 1
Char. 113: 1 → 0
Char. 118: 1 → 0
Char. 125: 1 → 0
Char. 150: 0 → 1

**Node 68:**
Char. 23: 0 → 1
Char. 71: 1 → 0
Char. 102: 0 → 1
Char. 103: 0 → 1
Char. 106: 0 → 1
Char. 167: 0 → 1
Char. 214: 0 → 1
Char. 216: 1 → 0
Char. 235: 0 → 2
Char. 241: 0 → 1

**Node 69:**
Char. 113: 0 → 1
Char. 132: 1 → 0
Char. 138: 0 → 1
Char. 141: 0 → 1
Char. 207: 0 → 1

**Node 70:**
Char. 20: 0 → 1
Char. 33: 0 → 1
Char. 44: 1 → 0
Char. 70: 0 → 1
Char. 80: 1 → 0
Char. 117: 0 → 1
Char. 118: 0 → 1
Char. 125: 0 → 1
Char. 131: 0 → 1
Char. 147: 0 → 1
Char. 194: 0 → 1
Char. 201: 1 → 0
Char. 211: 0 → 1

**Node 71:**
Char. 38: 1 → 2
Char. 39: 0 → 1
Char. 49: 0 → 1
Char. 66: 0 → 12
Char. 76: 0 → 1
Char. 88: 1 → 0
Char. 95: 0 → 1
Char. 112: 0 → 1
Char. 148: 0 → 1
Char. 158: 0 → 1
Char. 159: 0 → 1
Char. 183: 1 → 2
Char. 192: 0 → 1
Char. 215: 0 → 1
Char. 239: 0 → 1

**Node 72:**
Char. 87: 0 → 1

**Node 73:**
Char. 25: 1 → 0
Char. 76: 1 → 0

**Node 74:**
Char. 79: 0 → 1
Char. 93: 1 → 0
Char. 133: 0 → 1

**Node 75:**
Char. 50: 1 → 0
Char. 58: 0 → 1
Char. 59: 0 → 1
Char. 60: 0 → 1
Char. 72: 1 → 2
Char. 104: 0 → 1
Char. 105: 0 → 1
Char. 106: 0 → 2
Char. 107: 0 → 1
Char. 109: 0 → 1
Char. 110: 0 → 1
Char. 146: 1 → 0
Char. 155: 0 → 2

**Node 76:**
Char. 33: 1 → 0
Char. 38: 2 → 1
Char. 39: 1 → 0
Char. 42: 0 → 1
Char. 43: 0 → 1
Char. 46: 1 → 0
Char. 48: 1 → 0
Char. 49: 1 → 0
Char. 52: 0 → 1
Char. 83: 0 → 2
Char. 84: 1 → 0
Char. 87: 0 → 1
Char. 93: 1 → 0
Char. 132: 0 → 1
Char. 161: 0 → 1
Char. 163: 1 → 0
Char. 172: 0 → 2
Char. 174: 0 → 1
Char. 188: 0 → 1
Char. 189: 0 → 1
Char. 195: 0 → 1
Char. 204: 0 → 2
Char. 212: 0 → 1
Char. 236: 0 → 1
Char. 238: 0 → 2
Char. 242: 0 → 1
Char. 274: 0 → 1
Char. 275: 1 → 0

**Node 77:**
Char. 29: 1 → 0
Char. 44: 1 → 0
Char. 59: 0 → 1
Char. 60: 0 → 1
Char. 66: 0 → 1
Char. 67: 0 → 1
Char. 72: 1 → 2
Char. 78: 1 → 0
Char. 84: 1 → 0
Char. 88: 1 → 0
Char. 93: 1 → 0
Char. 111: 0 → 1
Char. 123: 1 → 0
Char. 129: 0 → 1
Char. 154: 1 → 0
Char. 169: 1 → 0
Char. 170: 0 → 1
Char. 197: 0 → 1

**Node 78:**
Char. 20: 0 → 1
Char. 47: 0 → 1
Char. 85: 1 → 0
Char. 107: 0 → 1
Char. 110: 0 → 1
Char. 169: 1 → 0
Char. 170: 0 → 1

**Node 79:**
Char. 2: 0 → 1
Char. 6: 0 → 1
Char. 101: 0 → 1
Char. 104: 0 → 1
Char. 106: 0 → 1
Char. 113: 0 → 1
Char. 186: 0 → 1
Char. 224: 1 → 0
Char. 231: 1 → 0
Char. 232: 1 → 0
Char. 266: 1 → 0
Char. 267: 1 → 0

**Node 80:**
Char. 17: 0 → 1
Char. 42: 0 → 1
Char. 264: 0 → 1

**Node 81:**
Char. 41: 0 → 1
Char. 61: 1 → 2
Char. 128: 0 → 1
Char. 138: 0 → 1
Char. 146: 1 → 0
Char. 155: 0 → 1
Char. 192: 1 → 2
Char. 233: 0 → 1
Char. 267: 1 → 2

**Node 82:**
Char. 61: 1 → 3
Char. 90: 0 → 1
Char. 91: 0 → 1
Char. 109: 1 → 0
Char. 155: 0 → 1
Char. 209: 1 → 0

**ANALYSIS 22**
**(NO *PROGANOCHELYS QUENSTEDTI*, *PAPPOCHELYS ROSINAE*, AND *EUNOTOSAURUS AFRICANUS*)**

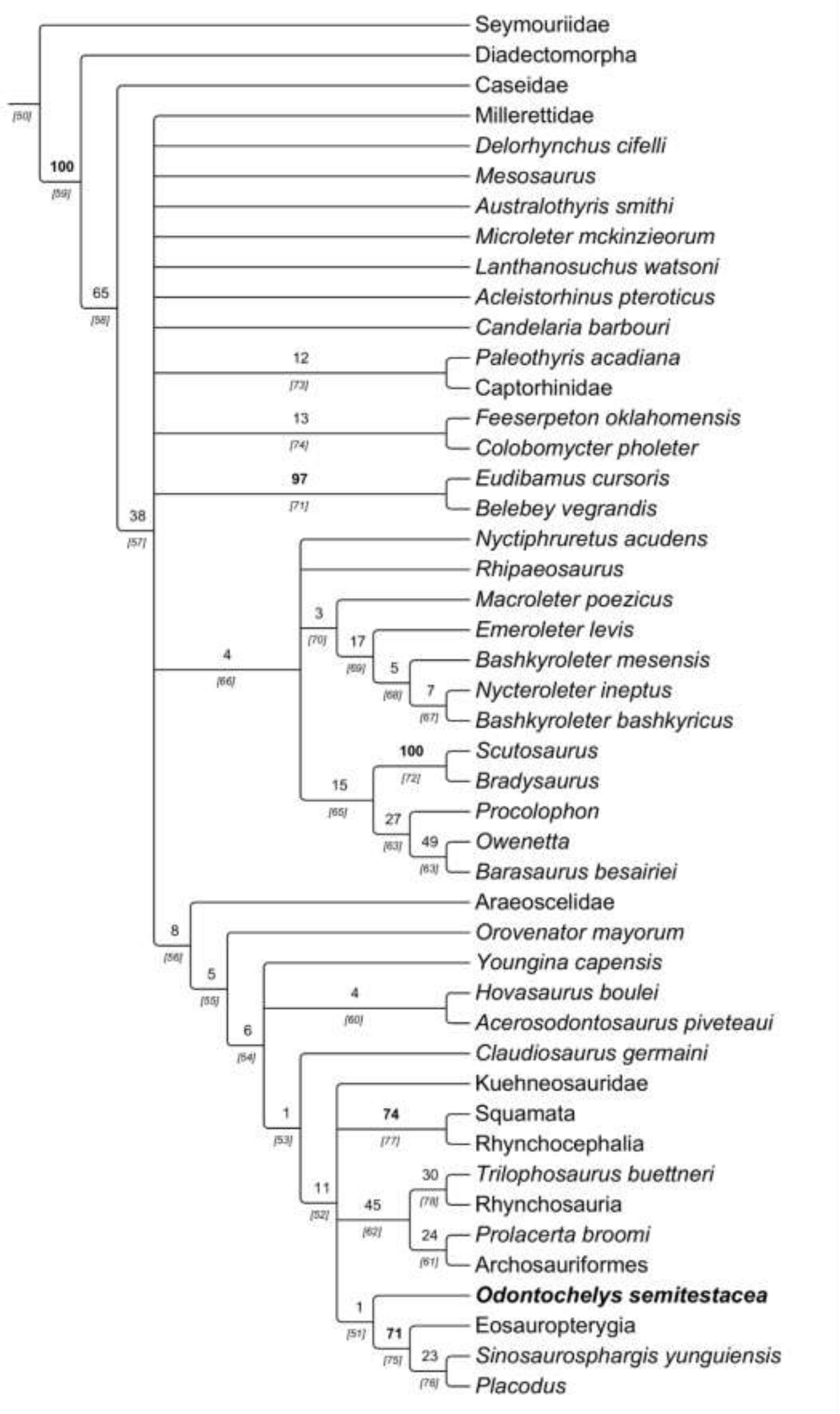

**Seymouriidae:**
Char. 23: 0 → 1
Char. 51: 0 → 2
Char. 54: 1 → 0
Char. 71: 1 → 0
Char. 83: 0 → 1
Char. 85: 1 → 0
Char. 99: 1 → 0
Char. 107: 0 → 1
Char. 140: 0 → 2
Char. 154: 1 → 2
Char. 207: 0 → 1
Char. 225: 1 → 0
Char. 260: 2 → 0

***Odontochelys semitestacea*:**
Char. 46: 1 → 0
Char. 50: 0 → 1
Char. 58: 1 → 0
Char. 61: 1 → 0
Char. 62: 1 → 0
Char. 65: 0 → 1
Char. 89: 1 → 0
Char. 93: 1 → 0
Char. 109: 1 → 0
Char. 112: 1 → 0
Char. 124: 0 → 1
Char. 126: 1 → 0
Char. 150: 1 → 0
Char. 154: 0 → 1
Char. 167: 1 → 0
Char. 195: 0 → 2
Char. 201: 1 → 0
Char. 219: 1 → 0
Char. 226: 0 → 1
Char. 227: 0 → 1
Char. 238: 0 → 1
Char. 241: 0 → 1
Char. 246: 0 → 2
Char. 247: 0 → 1
Char. 252: 0 → 1
Char. 253: 0 → 2
Char. 254: 0 → 1
Char. 255: 0 → 1
Char. 256: 0 → 1
Char. 258: 0 → 1
Char. 259: 0 → 1
Char. 260: 1 → 2
Char. 265: 1 → 0
Char. 268: 0 → 1
Char. 269: 0 → 1
Char. 270: 0 → 1

***Acerosodontosaurus piveteaui*:**
Char. 81: 1 → 0
Char. 208: 0 → 1
Char. 224: 1 → 0

***Acleistorhinus pteroticus*:**
Char. 20: 0 → 1
Char. 21: 0 → 1
Char. 30: 0 → 1
Char. 33: 0 → 1
Char. 47: 0 → 1
Char. 48: 0 → 1
Char. 55: 1 → 0
Char. 56: 0 → 1
Char. 64: 0 → 1
Char. 70: 0 → 1
Char. 79: 0 → 1
Char. 95: 0 → 1
Char. 110: 0 → 1
Char. 113: 0 → 1
Char. 114: 0 → 1
Char. 131: 0 → 1
Char. 137: 0 → 1
Char. 140: 0 → 2
Char. 146: 1 → 0
Char. 159: 0 → 1
Char. 169: 1 → 0
Char. 170: 0 → 1
Char. 278: 3 → 1

**Araeoscelidae:**
Char. 5: 1 → 0
Char. 28: 0 → 1
Char. 106: 0 → 1
Char. 116: 1 → 0
Char. 166: 1 → 0
Char. 169: 1 → 0
Char. 170: 0 → 1
Char. 197: 0 → 1

**Archosauriformes:**
Char. 32: 0 → 1
Char. 94: 1 → 0
Char. 112: 1 → 0
Char. 152: 0 → 1
Char. 154: 0 → 2
Char. 166: 1 → 0
Char. 171: 0 → 1
Char. 185: 0 → 1
Char. 204: 0 → 1
Char. 218: 0 → 3
Char. 242: 0 → 1

***Australothyris smithi*:**
Char. 23: 0 → 1
Char. 24: 0 → 1
Char. 34: 0 → 1
Char. 55: 1 → 0
Char. 57: 1 → 0
Char. 71: 1 → 0
Char. 79: 0 → 1
Char. 85: 1 → 0
Char. 98: 1 → 0
Char. 100: 0 → 1
Char. 103: 0 → 1
Char. 110: 0 → 1
Char. 112: 0 → 1
Char. 123: 1 → 0
Char. 129: 0 → 1
Char. 131: 0 → 1
Char. 132: 1 → 0
Char. 144: 1 → 0
Char. 149: 1 → 0
Char. 150: 0 → 1

***Barasaurus besairiei*:**
Char. 33: 1 → 0
Char. 75: 1 → 0
Char. 216: 0 → 1

***Bashkyroleter bashkyricus*:**
Char. 275: 1 → 0

***Bashkyroleter mesensis*:**
Char. 169: 1 → 0

***Belebey vegrandis*:**
Char. 154: 1 → 0

***Bradysaurus* spp.:**
Char. 19: 0 → 1
Char. 73: 0 → 1
Char. 79: 0 → 1
Char. 135: 1 → 0
Char. 249: 0 → 1

***Candelaria barbouri*:**
Char. 1: 0 → 1
Char. 5: 1 → 0
Char. 8: 0 → 1
Char. 15: 0 → 1
Char. 20: 0 → 1
Char. 23: 0 → 1
Char. 25: 1 → 0
Char. 29: 1 → 0
Char. 33: 0 → 2
Char. 49: 0 → 1
Char. 50: 1 → 0
Char. 55: 1 → 0
Char. 57: 1 → 0
Char. 67: 0 → 1
Char. 79: 0 → 2
Char. 89: 0 → 1
Char. 94: 0 → 1
Char. 95: 0 → 1
Char. 126: 0 → 1
Char. 127: 0 → 1
Char. 132: 1 → 0
Char. 154: 1 → 2
Char. 169: 1 → 0
Char. 276: 0 → 1
Char. 277: 0 → 1

**Captorhinidae:**
Char. 3: 0 → 1
Char. 23: 0 → 1
Char. 25: 1 → 0
Char. 26: 0 → 1
Char. 73: 0 → 1
Char. 75: 0 → 1
Char. 108: 1 → 0
Char. 183: 1 → 0
Char. 201: 1 → 0
Char. 203: 1 → 2
Char. 216: 1 → 0

**Caseidae:**
Char. 24: 0 → 1
Char. 25: 1 → 0
Char. 36: 0 → 1
Char. 38: 1 → 0
Char. 46: 1 → 0
Char. 50: 1 → 0
Char. 56: 0 → 1
Char. 85: 1 → 0
Char. 96: 1 → 0
Char. 98: 1 → 0
Char. 170: 0 → 1
Char. 194: 0 → 1
Char. 273: 0 → 1
Char. 274: 0 → 1

***Claudiosaurus germaini*:**
Char. 24: 0 → 1
Char. 34: 0 → 1
Char. 36: 0 → 1
Char. 43: 1 → 0
Char. 64: 0 → 1
Char. 105: 0 → 1
Char. 106: 0 → 1
Char. 130: 0 → 1
Char. 144: 1 → 0
Char. 166: 1 → 0
Char. 187: 1 → 0
Char. 199: 0 → 1
Char. 203: 1 → 2
Char. 204: 0 → 1
Char. 220: 0 → 1
Char. 222: 1 → 0
Char. 224: 1 → 0

***Colobomycter pholeter*:**
Char. 21: 0 → 1
Char. 25: 1 → 0
Char. 84: 1 → 0
Char. 154: 1 → 0
Char. 167: 0 → 1
Char. 267: 0 → 1

***Delorhynchus cifelli*:**
Char. 18: 0 → 1
Char. 20: 0 → 1
Char. 21: 0 → 1
Char. 24: 0 → 1
Char. 26: 0 → 1
Char. 28: 0 → 1
Char. 33: 0 → 2
Char. 39: 0 → 1
Char. 48: 0 → 1
Char. 52: 0 → 1
Char. 100: 0 → 1
Char. 111: 0 → 1
Char. 116: 1 → 0
Char. 117: 0 → 1
Char. 131: 0 → 1
Char. 156: 1 → 0
Char. 159: 0 → 1
Char. 167: 0 → 1
Char. 180: 0 → 1
Char. 189: 0 → 1
Char. 191: 0 → 1
Char. 204: 0 → 2
Char. 267: 0 → 1

**Diadectomorpha:**
Char. 0: 0 → 1
Char. 70: 0 → 1
Char. 75: 0 → 1
Char. 122: 0 → 1
Char. 123: 1 → 0
Char. 146: 1 → 0
Char. 275: 1 → 0

***Emeroleter levis*:**
Char. 0: 0 → 2
Char. 51: 1 → 0

**Eosauropterygia:**
Char. 159: 1 → 0
Char. 166: 1 → 0
Char. 168: 0 → 1
Char. 178: 0 → 1
Char. 272: 1 → 0

***Eudibamus cursoris*:**
Char. 154: 1 → 2

***Feeserpeton oklahomensis*:**
Char. 51: 0 → 1
Char. 70: 0 → 1
Char. 157: 0 → 1
Char. 158: 0 → 1

***Hovasaurus boulei*:**
Char. 41: 0 → 1
Char. 77: 0 → 2
Char. 79: 0 → 1
Char. 93: 1 → 0
Char. 204: 0 → 2

**Kuehneosauridae:**
Char. 7: 0 → 1
Char. 24: 0 → 1
Char. 26: 0 → 1
Char. 27: 0 → 1
Char. 34: 0 → 1
Char. 36: 0 → 1
Char. 43: 1 → 0
Char. 44: 1 → 0
Char. 79: 0 → 2
Char. 82: 0 → 1
Char. 98: 1 → 0
Char. 108: 1 → 0
Char. 113: 0 → 1
Char. 128: 0 → 1
Char. 140: 1 → 0
Char. 147: 1 → 0
Char. 148: 1 → 0
Char. 159: 1 → 0
Char. 168: 0 → 1
Char. 181: 0 → 1
Char. 185: 0 → 1
Char. 206: 0 → 2
Char. 245: 0 → 1

Char. 272: 1 → 2
Char. 278: 0 → 3

***Lanthanosuchus watsoni*:**
Char. 25: 1 → 0
Char. 51: 0 → 1
Char. 86: 0 → 1
Char. 95: 0 → 1
Char. 98: 1 → 0
Char. 110: 0 → 1
Char. 113: 0 → 1
Char. 114: 0 → 1
Char. 131: 0 → 1
Char. 137: 0 → 1
Char. 138: 0 → 1
Char. 140: 0 → 2
Char. 144: 1 → 0
Char. 154: 1 → 2

***Macroleter poezicus*:**
Char. 0: 0 → 1
Char. 52: 0 → 1
Char. 66: 1 → 2
Char. 75: 0 → 1
Char. 84: 1 → 0
Char. 87: 0 → 1
Char. 110: 0 → 1
Char. 140: 0 → 1
Char. 146: 1 → 0
Char. 169: 1 → 0
Char. 235: 0 → 1

***Mesosaurus* spp.:**
Char. 0: 0 → 1
Char. 2: 0 → 1
Char. 5: 1 → 0
Char. 6: 0 → 1
Char. 8: 0 → 1
Char. 9: 0 → 1
Char. 13: 0 → 1
Char. 19: 0 → 1
Char. 23: 0 → 1
Char. 26: 0 → 1
Char. 29: 1 → 0
Char. 33: 0 → 1
Char. 38: 1 → 0
Char. 41: 0 → 1
Char. 48: 0 → 1
Char. 50: 1 → 0
Char. 67: 0 → 1
Char. 84: 1 → 0
Char. 85: 1 → 0
Char. 94: 0 → 1
Char. 107: 0 → 1
Char. 109: 0 → 1
Char. 111: 0 → 1
Char. 115: 0 → 1
Char. 146: 1 → 0
Char. 148: 0 → 1
Char. 149: 1 → 0
Char. 164: 0 → 1
Char. 166: 1 → 0
Char. 167: 0 → 1
Char. 176: 0 → 1
Char. 183: 1 → 0
Char. 184: 0 → 1
Char. 199: 0 → 1
Char. 202: 0 → 1
Char. 204: 0 → 2
Char. 206: 0 → 1
Char. 207: 0 → 1
Char. 209: 0 → 1
Char. 217: 0 → 1
Char. 219: 0 → 1
Char. 220: 0 → 1
Char. 231: 0 → 1
Char. 260: 2 → 0
Char. 272: 2 → 0
Char. 278: 3 → 0

***Microleter mckinzieorum*:**
Char. 0: 0 → 1
Char. 18: 0 → 1
Char. 24: 0 → 1
Char. 25: 1 → 0
Char. 36: 0 → 1
Char. 39: 0 → 1
Char. 51: 0 → 2
Char. 56: 0 → 1
Char. 57: 1 → 0
Char. 70: 0 → 1
Char. 79: 0 → 1
Char. 94: 0 → 1
Char. 106: 0 → 1
Char. 110: 0 → 1
Char. 132: 1 → 0
Char. 159: 0 → 1
Char. 166: 1 → 0
Char. 276: 0 → 1
Char. 278: 3 → 1

**Millerettidae:**
Char. 5: 1 → 0
Char. 24: 0 → 1
Char. 25: 1 → 0
Char. 44: 1 → 0
Char. 56: 0 → 1
Char. 57: 1 → 0
Char. 66: 0 → 2
Char. 78: 1 → 0
Char. 80: 1 → 0
Char. 84: 1 → 2
Char. 96: 1 → 0
Char. 117: 0 → 1
Char. 124: 0 → 1
Char. 127: 0 → 1
Char. 135: 0 → 1
Char. 145: 0 → 1
Char. 166: 1 → 0
Char. 202: 0 → 1
Char. 211: 0 → 1
Char. 230: 0 → 1
Char. 234: 0 → 1
Char. 248: 0 → 1
Char. 252: 0 → 1
Char. 253: 0 → 1

***Nycteroleter ineptus*:**
Char. 278: 0 → 3

***Nyctiphruretus acudens*:**
Char. 0: 0 → 1
Char. 21: 0 → 1
Char. 33: 1 → 2
Char. 41: 0 → 1
Char. 66: 1 → 2
Char. 81: 1 → 0
Char. 84: 1 → 2
Char. 94: 0 → 1
Char. 166: 1 → 0
Char. 167: 0 → 1
Char. 215: 1 → 0
Char. 224: 0 → 1
Char. 226: 1 → 0
Char. 266: 0 → 1
Char. 272: 2 → 1
Char. 276: 0 → 1

***Orovenator mayorum*:**
Char. 8: 0 → 1
Char. 24: 0 → 1
Char. 36: 0 → 1
Char. 160: 0 → 1
Char. 165: 0 → 1

***Owenetta* spp.:**
Char. 169: 1 → 0

***Paleothyris acadiana*:**
Char. 38: 1 → 0
Char. 50: 1 → 0
Char. 66: 1 → 2
Char. 102: 0 → 1
Char. 146: 1 → 0
Char. 180: 0 → 1
Char. 237: 0 → 1
Char. 239: 0 → 1

***Placodus* spp.:**
Char. 9: 0 → 1
Char. 12: 0 → 1
Char. 13: 0 → 1
Char. 19: 0 → 1
Char. 26: 0 → 1
Char. 31: 0 → 1
Char. 44: 1 → 0
Char. 46: 1 → 0
Char. 57: 0 → 1
Char. 78: 1 → 0
Char. 93: 1 → 0
Char. 109: 1 → 0
Char. 140: 1 → 0
Char. 155: 0 → 1
Char. 163: 1 → 0
Char. 164: 0 → 1

***Procolophon* spp.:**
Char. 41: 0 → 1
Char. 69: 0 → 1
Char. 79: 0 → 1
Char. 88: 0 → 1
Char. 117: 1 → 0
Char. 149: 1 → 0
Char. 180: 0 → 1
Char. 204: 0 → 1
Char. 237: 0 → 1
Char. 238: 0 → 1
Char. 272: 2 → 1
Char. 278: 3 → 0

***Prolacerta broomi*:**
Char. 58: 1 → 0
Char. 66: 1 → 0
Char. 67: 1 → 0
Char. 80: 1 → 0
Char. 139: 1 → 0
Char. 147: 1 → 0
Char. 192: 1 → 0
Char. 203: 1 → 2
Char. 206: 0 → 12
Char. 224: 1 → 0

***Rhipaeosaurus* spp.:**
Char. 172: 0 → 1
Char. 186: 1 → 0
Char. 277: 0 → 1

**Rhynchocephalia:**
Char. 0: 1 → 2
Char. 23: 1 → 0
Char. 24: 0 → 1
Char. 77: 0 → 1
Char. 88: 1 → 0
Char. 94: 1 → 0
Char. 104: 0 → 1
Char. 117: 12 → 0
Char. 139: 1 → 0
Char. 167: 1 → 0
Char. 205: 1 → 0

**Rhynchosauria:**
Char. 0: 1 → 0
Char. 7: 0 → 1
Char. 9: 0 → 1
Char. 26: 0 → 1
Char. 44: 1 → 0
Char. 63: 1 → 0
Char. 68: 0 → 1
Char. 99: 1 → 0
Char. 150: 1 → 0
Char. 160: 0 → 1
Char. 161: 0 → 1
Char. 171: 0 → 2
Char. 241: 0 → 1

***Scutosaurus* spp.:**
Char. 175: 0 → 1
Char. 218: 0 → 1
Char. 243: 0 → 2
Char. 244: 0 → 1
Char. 251: 0 → 1

***Sinosaurosphargis yunguiensis*:**
Char. 8: 0 → 1
Char. 30: 0 → 1
Char. 53: 0 → 1
Char. 82: 0 → 1
Char. 89: 1 → 0
Char. 127: 1 → 0
Char. 150: 1 → 0
Char. 154: 0 → 2
Char. 167: 1 → 0
Char. 253: 0 → 1
Char. 255: 0 → 1

**Squamata:**
Char. 26: 0 → 1
Char. 45: 0 → 1
Char. 75: 0 → 1
Char. 79: 0 → 2
Char. 80: 1 → 0
Char. 82: 0 → 1
Char. 92: 1 → 0
Char. 106: 0 → 1
Char. 109: 1 → 0
Char. 122: 0 → 1
Char. 160: 0 → 1
Char. 200: 0 → 1
Char. 245: 0 → 1

***Trilophosaurus buettneri*:**
Char. 5: 1 → 0
Char. 11: 0 → 1
Char. 55: 1 → 0
Char. 93: 1 → 0
Char. 104: 0 → 1
Char. 106: 0 → 1
Char. 113: 0 → 1
Char. 122: 0 → 1
Char. 136: 1 → 0
Char. 144: 1 → 0
Char. 154: 0 → 1
Char. 157: 0 → 1
Char. 159: 1 → 0
Char. 177: 0 → 12
Char. 194: 0 → 1
Char. 203: 1 → 2
Char. 207: 1 → 0
Char. 208: 1 → 0
Char. 224: 1 → 0
Char. 272: 1 → 0

***Youngina capensis*:**
Char. 5: 1 → 0
Char. 44: 1 → 0
Char. 75: 0 → 1
Char. 170: 0 → 1
Char. 211: 0 → 1
Char. 231: 0 → 1

**Node 50:**
Char. 15: 1 → 0
Char. 20: 1 → 0
Char. 21: 1 → 0
Char. 25: 0 → 1
Char. 33: 2 → 0
Char. 72: 2 → 0
Char. 73: 1 → 0
Char. 84: 2 → 01
Char. 94: 1 → 0

Char. 119: 1 → 0
Char. 127: 1 → 0
Char. 130: 1 → 0
Char. 134: 2 → 0
Char. 136: 1 → 0
Char. 141: 1 → 0
Char. 145: 1 → 0
Char. 147: 1 → 0
Char. 149: 1 → 0
Char. 151: 1 → 0
Char. 152: 1 → 0
Char. 155: 1 → 0
Char. 158: 1 → 0
Char. 159: 1 → 0
Char. 161: 1 → 0
Char. 163: 1 → 0
Char. 166: 1 → 0
Char. 174: 1 → 0
Char. 180: 1 → 0
Char. 181: 1 → 0
Char. 187: 1 → 0
Char. 188: 1 → 0
Char. 194: 2 → 0
Char. 196: 1 → 0
Char. 203: 2 → 0
Char. 204: 2 → 0
Char. 217: 1 → 0
Char. 220: 1 → 0
Char. 221: 1 → 0
Char. 231: 1 → 0
Char. 234: 1 → 0
Char. 247: 1 → 0
Char. 251: 1 → 0
Char. 253: 2 → 0
Char. 266: 1 → 0

**Node 51:**
Char. 33: 1 → 2
Char. 88: 1 → 0
Char. 189: 0 → 1
Char. 193: 1 → 0
Char. 198: 0 → 1
Char. 206: 0 → 2
Char. 220: 0 → 1
Char. 235: 1 → 2
Char. 239: 1 → 0
Char. 240: 1 → 0

**Node 52:**
Char. 27: 1 → 0
Char. 58: 0 → 1
Char. 61: 0 → 1
Char. 66: 0 → 1
Char. 69: 0 → 1
Char. 84: 0 → 1
Char. 140: 0 → 1
Char. 147: 0 → 1
Char. 150: 0 → 1
Char. 167: 0 → 1
Char. 184: 0 → 1
Char. 205: 0 → 1
Char. 208: 0 → 1
Char. 210: 0 → 1
Char. 230: 0 → 1
Char. 231: 0 → 1
Char. 232: 0 → 1

**Node 53:**
Char. 70: 0 → 1
Char. 73: 0 → 1
Char. 126: 0 → 1
Char. 131: 0 → 1
Char. 148: 0 → 1
Char. 190: 0 → 1
Char. 272: 2 → 1

**Node 54:**
Char. 33: 0 → 1
Char. 135: 0 → 1
Char. 159: 0 → 1

**Node 55:**
Char. 20: 0 → 1
Char. 62: 0 → 1

**Node 56:**
Char. 0: 0 → 1
Char. 27: 0 → 1
Char. 29: 1 → 0
Char. 38: 1 → 0
Char. 40: 2 → 0
Char. 57: 1 → 0
Char. 59: 0 → 1
Char. 60: 0 → 1
Char. 67: 0 → 1
Char. 72: 1 → 2
Char. 84: 1 → 0
Char. 89: 0 → 1
Char. 111: 0 → 1
Char. 112: 0 → 1
Char. 120: 0 → 1
Char. 154: 1 → 0
Char. 180: 0 → 1
Char. 193: 0 → 1
Char. 222: 0 → 1
Char. 224: 0 → 1
Char. 234: 0 → 1
Char. 237: 0 → 1
Char. 266: 0 → 1
Char. 278: 3 → 1

**Node 57:**
Char. 5: 0 → 1
Char. 29: 0 → 1
Char. 40: 0 → 2
Char. 74: 0 → 1
Char. 116: 0 → 1
Char. 149: 0 → 1
Char. 166: 0 → 1
Char. 169: 0 → 1
Char. 201: 0 → 1
Char. 203: 0 → 1
Char. 235: 0 → 1

**Node 58:**
Char. 72: 0 → 1
Char. 81: 0 → 1
Char. 97: 0 → 1

**Node 59:**
Char. 55: 0 → 1
Char. 78: 0 → 1
Char. 111: 1 → 0
Char. 126: 1 → 0
Char. 135: 1 → 0

**Node 60:**
Char. 78: 1 → 0
Char. 206: 0 → 2

**Node 61:**
Char. 19: 0 → 1
Char. 92: 1 → 0

**Node 62:**
Char. 4: 0 → 1
Char. 15: 0 → 1
Char. 29: 0 → 1
Char. 35: 1 → 0
Char. 48: 1 → 0
Char. 75: 0 → 1
Char. 210: 1 → 0
Char. 213: 0 → 1
Char. 226: 0 → 2
Char. 228: 0 → 1
Char. 275: 0 → 1

**Node 63:**
Char. 73: 0 → 1
Char. 131: 1 → 0
Char. 205: 0 → 1
Char. 239: 0 → 1
Char. 276: 0 → 1

**Node 64:**
Char. 18: 0 → 1
Char. 37: 0 → 1
Char. 48: 0 → 1
Char. 107: 0 → 1
Char. 118: 1 → 0
Char. 125: 1 → 0
Char. 126: 0 → 1
Char. 132: 1 → 0
Char. 150: 0 → 1

**Node 65:**
Char. 23: 0 → 1
Char. 71: 1 → 0
Char. 75: 0 → 1
Char. 102: 0 → 1
Char. 103: 0 → 1
Char. 106: 0 → 1
Char. 110: 0 → 1
Char. 155: 0 → 1
Char. 167: 0 → 1
Char. 207: 0 → 1
Char. 214: 0 → 1
Char. 216: 1 → 0
Char. 235: 0 → 2
Char. 241: 0 → 1

**Node 66:**
Char. 20: 0 → 1
Char. 33: 0 → 1
Char. 38: 1 → 2
Char. 39: 0 → 1
Char. 44: 1 → 0
Char. 49: 0 → 1
Char. 51: 0 → 1
Char. 66: 0 → 1
Char. 70: 0 → 1
Char. 80: 1 → 0
Char. 95: 0 → 1
Char. 112: 0 → 1
Char. 117: 0 → 1
Char. 118: 0 → 1
Char. 125: 0 → 1
Char. 131: 0 → 1
Char. 135: 0 → 1
Char. 137: 0 → 1
Char. 148: 0 → 1
Char. 157: 0 → 1
Char. 158: 0 → 1
Char. 159: 0 → 1
Char. 183: 1 → 2
Char. 186: 0 → 1
Char. 194: 0 → 1
Char. 197: 0 → 1
Char. 201: 1 → 0
Char. 211: 0 → 1
Char. 215: 0 → 1
Char. 226: 0 → 1
Char. 235: 1 → 0
Char. 252: 0 → 1

**Node 67:**
Char. 87: 0 → 1
Char. 110: 0 → 1

**Node 68:**
Char. 25: 1 → 0
Char. 76: 1 → 0

**Node 69:**
Char. 79: 0 → 1
Char. 86: 0 → 1
Char. 93: 1 → 0
Char. 133: 0 → 1

**Node 70:**
Char. 48: 0 → 1
Char. 100: 0 → 1
Char. 113: 0 → 1
Char. 132: 1 → 0
Char. 147: 1 → 2
Char. 154: 1 → 0
Char. 207: 0 → 1
Char. 239: 0 → 1
Char. 278: 3 → 0

**Node 71:**
Char. 38: 1 → 2
Char. 39: 0 → 1
Char. 50: 1 → 0
Char. 58: 0 → 1
Char. 59: 0 → 1
Char. 60: 0 → 1
Char. 72: 1 → 2
Char. 85: 1 → 0
Char. 95: 0 → 1
Char. 104: 0 → 1
Char. 105: 0 → 1
Char. 106: 0 → 2
Char. 107: 0 → 1
Char. 109: 0 → 1
Char. 110: 0 → 1
Char. 146: 1 → 0
Char. 148: 0 → 1
Char. 155: 0 → 2
Char. 183: 1 → 2

**Node 72:**
Char. 0: 0 → 2
Char. 33: 1 → 0
Char. 38: 2 → 1
Char. 39: 1 → 0
Char. 42: 0 → 1
Char. 43: 0 → 1
Char. 46: 1 → 0
Char. 49: 1 → 0
Char. 52: 0 → 1
Char. 83: 01 → 2
Char. 84: 1 → 0
Char. 87: 0 → 1
Char. 93: 1 → 0
Char. 100: 0 → 1
Char. 113: 0 → 1
Char. 143: 0 → 1
Char. 161: 0 → 1
Char. 163: 1 → 0
Char. 172: 0 → 2
Char. 174: 0 → 1
Char. 188: 0 → 1
Char. 189: 0 → 1
Char. 195: 0 → 1
Char. 204: 0 → 2
Char. 212: 0 → 1
Char. 236: 0 → 1
Char. 238: 0 → 2
Char. 242: 0 → 1
Char. 245: 0 → 1
Char. 274: 0 → 1
Char. 275: 1 → 0

**Node 73:**
Char. 5: 1 → 0
Char. 29: 1 → 0
Char. 44: 1 → 0
Char. 59: 0 → 1
Char. 60: 0 → 1
Char. 66: 0 → 1
Char. 67: 0 → 1
Char. 72: 1 → 2
Char. 78: 1 → 0
Char. 84: 1 → 0
Char. 93: 1 → 0
Char. 111: 0 → 1
Char. 123: 1 → 0
Char. 129: 0 → 1
Char. 154: 1 → 0
Char. 169: 1 → 0
Char. 170: 0 → 1
Char. 197: 0 → 1

**Node 74:**
Char. 20: 0 → 1
Char. 47: 0 → 1
Char. 85: 1 → 0
Char. 107: 0 → 1
Char. 110: 0 → 1
Char. 169: 1 → 0
Char. 170: 0 → 1

**Node 75:**
Char. 2: 0 → 1
Char. 6: 0 → 1
Char. 101: 0 → 1
Char. 104: 0 → 1
Char. 106: 0 → 1
Char. 113: 0 → 1
Char. 186: 0 → 1
Char. 224: 1 → 0
Char. 229: 0 → 1
Char. 231: 1 → 0
Char. 232: 1 → 0
Char. 267: 1 → 0

**Node 76:**
Char. 17: 0 → 1
Char. 42: 0 → 1
Char. 264: 0 → 1

**Node 77:**
Char. 33: 1 → 2
Char. 41: 0 → 1
Char. 48: 1 → 0
Char. 61: 1 → 2
Char. 68: 0 → 1
Char. 112: 1 → 0
Char. 128: 0 → 1
Char. 138: 0 → 1
Char. 146: 1 → 0
Char. 155: 0 → 1
Char. 158: 0 → 1
Char. 192: 1 → 2
Char. 226: 0 → 1
Char. 227: 0 → 1
Char. 233: 0 → 1
Char. 235: 1 → 2
Char. 267: 1 → 2

**Node 78:**
Char. 61: 1 → 3
Char. 90: 0 → 1
Char. 91: 0 → 1
Char. 109: 1 → 0
Char. 155: 0 → 1
Char. 209: 1 → 0

**ANALYSIS 23**
**(NO *PROGANOCHELYS QUENSTEDTI*, *CANDELARIA BARBOURI*, *PAPPOCHELYS ROSINAE*, AND *ODONTOCHELYS SEMITESTACEA*)**

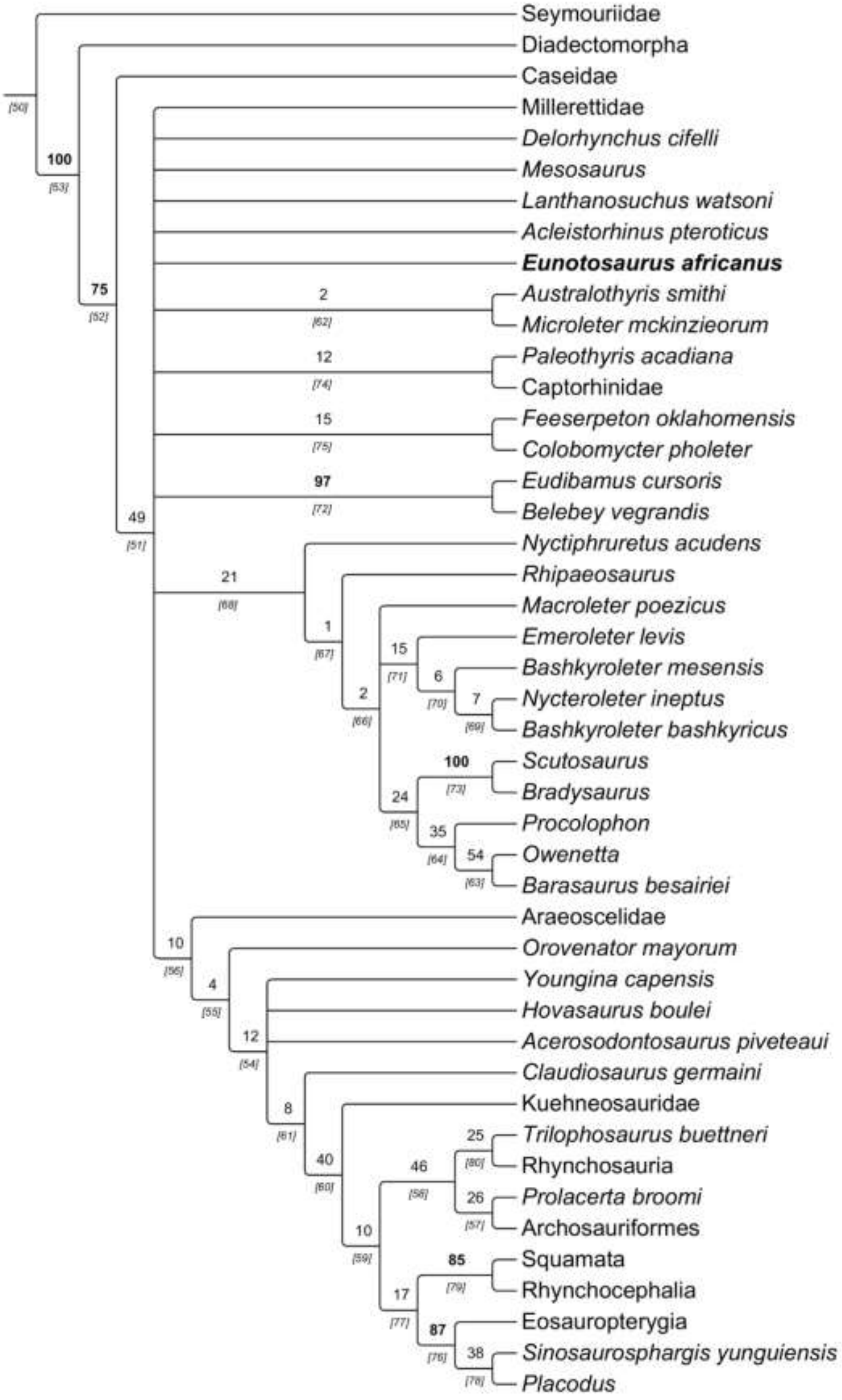

**Seymouriidae:**
Char. 51: 0 → 2
Char. 54: 1 → 0
Char. 85: 1 → 0
Char. 99: 1 → 0
Char. 107: 0 → 1
Char. 140: 0 → 2
Char. 154: 1 → 2
Char. 225: 1 → 0
Char. 260: 2 → 0

***Eunotosaurus africanus*:**
Char. 0: 0 → 2
Char. 15: 0 → 1
Char. 19: 0 → 1
Char. 20: 0 → 1
Char. 21: 0 → 1
Char. 24: 0 → 1
Char. 25: 1 → 0
Char. 29: 1 → 0
Char. 33: 0 → 2
Char. 40: 2 → 0
Char. 42: 0 → 1
Char. 43: 0 → 1
Char. 44: 1 → 0
Char. 55: 1 → 0
Char. 64: 0 → 1
Char. 73: 0 → 1
Char. 81: 1 → 0
Char. 84: 1 → 2
Char. 89: 0 → 1
Char. 94: 0 → 1
Char. 97: 1 → 0
Char. 102: 0 → 1
Char. 103: 0 → 1
Char. 111: 0 → 1
Char. 120: 0 → 1
Char. 124: 0 → 1
Char. 127: 0 → 1
Char. 130: 0 → 1
Char. 134: 0 → 2
Char. 135: 0 → 1
Char. 136: 0 → 1
Char. 141: 0 → 1
Char. 145: 0 → 1
Char. 148: 0 → 1
Char. 151: 0 → 1
Char. 152: 0 → 1
Char. 153: 1 → 0
Char. 155: 0 → 1
Char. 158: 0 → 1
Char. 161: 0 → 1
Char. 174: 0 → 1
Char. 181: 0 → 1
Char. 188: 0 → 1
Char. 194: 0 → 1
Char. 196: 0 → 1
Char. 201: 1 → 0
Char. 203: 1 → 2
Char. 211: 0 → 1
Char. 217: 0 → 1
Char. 219: 0 → 1
Char. 220: 0 → 1
Char. 231: 0 → 1
Char. 247: 0 → 1
Char. 248: 0 → 1
Char. 249: 0 → 2
Char. 250: 0 → 1
Char. 251: 0 → 1
Char. 252: 0 → 1
Char. 253: 0 → 2
Char. 263: 0 → 1
Char. 273: 0 → 1
Char. 274: 0 → 1
Char. 276: 0 → 1
Char. 277: 0 → 1

***Acerosodontosaurus piveteaui*:**
Char. 78: 1 → 0
Char. 81: 1 → 0
Char. 128: 0 → 1
Char. 155: 0 → 1
Char. 206: 0 → 2
Char. 208: 0 → 1

***Acleistorhinus pteroticus*:**
Char. 20: 0 → 1
Char. 21: 0 → 1
Char. 30: 0 → 1
Char. 33: 0 → 1
Char. 47: 0 → 1
Char. 48: 0 → 1
Char. 55: 1 → 0
Char. 56: 0 → 1
Char. 64: 0 → 1
Char. 70: 0 → 1
Char. 79: 0 → 1
Char. 95: 0 → 1
Char. 110: 0 → 1
Char. 113: 0 → 1
Char. 114: 0 → 1
Char. 131: 0 → 1
Char. 137: 0 → 1
Char. 140: 0 → 2
Char. 146: 1 → 0
Char. 169: 1 → 0
Char. 170: 0 → 1
Char. 278: 3 → 1

**Araeoscelidae:**
Char. 5: 1 → 0
Char. 28: 0 → 1
Char. 106: 0 → 1
Char. 116: 1 → 0
Char. 166: 1 → 0
Char. 169: 1 → 0
Char. 170: 0 → 1
Char. 197: 0 → 1
Char. 221: 1 → 0
Char. 239: 0 → 1

**Archosauriformes:**
Char. 32: 0 → 1
Char. 94: 1 → 0
Char. 112: 1 → 0
Char. 152: 0 → 1
Char. 154: 0 → 2
Char. 166: 1 → 0
Char. 171: 0 → 1
Char. 185: 0 → 1
Char. 204: 0 → 1
Char. 218: 0 → 3
Char. 242: 0 → 1

***Australothyris smithi*:**
Char. 23: 0 → 1
Char. 34: 0 → 1
Char. 55: 1 → 0
Char. 71: 1 → 0
Char. 85: 1 → 0
Char. 112: 0 → 1
Char. 129: 0 → 1
Char. 131: 0 → 1
Char. 159: 1 → 0

***Barasaurus besairiei*:**
Char. 33: 1 → 0
Char. 75: 1 → 0
Char. 216: 0 → 1

***Bashkyroleter bashkyricus*:**
Char. 275: 1 → 0

***Bashkyroleter mesensis*:**
Char. 169: 1 → 0

***Belebey vegrandis*:**
Char. 154: 1 → 0

***Bradysaurus* spp.:**
Char. 19: 0 → 1
Char. 73: 0 → 1
Char. 79: 0 → 1
Char. 135: 1 → 0
Char. 249: 0 → 1

**Captorhinidae:**
Char. 3: 0 → 1
Char. 23: 0 → 1
Char. 25: 1 → 0
Char. 26: 0 → 1
Char. 73: 0 → 1
Char. 75: 0 → 1
Char. 83: 0 → 1
Char. 108: 1 → 0
Char. 183: 1 → 0
Char. 201: 1 → 0
Char. 203: 1 → 2
Char. 216: 1 → 0

**Caseidae:**
Char. 24: 0 → 1
Char. 25: 1 → 0
Char. 36: 0 → 1
Char. 38: 1 → 0
Char. 46: 1 → 0
Char. 50: 1 → 0
Char. 56: 0 → 1
Char. 85: 1 → 0
Char. 96: 1 → 0
Char. 98: 1 → 0
Char. 170: 0 → 1
Char. 194: 0 → 1
Char. 273: 0 → 1
Char. 274: 0 → 1

***Claudiosaurus germaini*:**
Char. 64: 0 → 1
Char. 105: 0 → 1
Char. 130: 0 → 1
Char. 144: 1 → 0
Char. 166: 1 → 0
Char. 187: 1 → 0
Char. 199: 0 → 1
Char. 203: 1 → 2
Char. 204: 0 → 1
Char. 220: 0 → 1
Char. 222: 1 → 0

***Colobomycter pholeter*:**
Char. 21: 0 → 1
Char. 25: 1 → 0
Char. 84: 1 → 0
Char. 154: 1 → 0
Char. 167: 0 → 1
Char. 267: 0 → 1

***Delorhynchus cifelli*:**
Char. 18: 0 → 1
Char. 20: 0 → 1
Char. 21: 0 → 1
Char. 24: 0 → 1
Char. 26: 0 → 1
Char. 28: 0 → 1
Char. 33: 0 → 2
Char. 39: 0 → 1
Char. 48: 0 → 1
Char. 52: 0 → 1
Char. 100: 0 → 1
Char. 111: 0 → 1
Char. 116: 1 → 0
Char. 117: 0 → 1
Char. 119: 1 → 0
Char. 131: 0 → 1
Char. 156: 1 → 0
Char. 167: 0 → 1
Char. 189: 0 → 1
Char. 191: 0 → 1
Char. 192: 0 → 1
Char. 267: 0 → 1

**Diadectomorpha:**
Char. 0: 0 → 1
Char. 70: 0 → 1
Char. 75: 0 → 1
Char. 122: 0 → 1
Char. 123: 1 → 0
Char. 146: 1 → 0
Char. 275: 1 → 0

***Emeroleter levis*:**
Char. 0: 0 → 2
Char. 51: 1 → 0

**Eosauropterygia:**
Char. 159: 1 → 0
Char. 166: 1 → 0
Char. 174: 0 → 1
Char. 194: 0 → 2
Char. 272: 1 → 0

***Eudibamus cursoris*:**
Char. 154: 1 → 2
Char. 163: 1 → 0

***Feeserpeton oklahomensis*:**
Char. 51: 0 → 1
Char. 70: 0 → 1
Char. 157: 0 → 1
Char. 158: 0 → 1

***Hovasaurus boulei*:**
Char. 41: 0 → 1
Char. 55: 1 → 0
Char. 60: 1 → 0
Char. 72: 2 → 1
Char. 77: 0 → 2
Char. 78: 1 → 0
Char. 79: 0 → 1
Char. 93: 1 → 0
Char. 113: 0 → 1
Char. 138: 0 → 1
Char. 146: 1 → 0
Char. 154: 0 → 1
Char. 204: 0 → 2
Char. 206: 0 → 2
Char. 220: 0 → 1
Char. 278: 01 → 3

**Kuehneosauridae:**
Char. 7: 0 → 1
Char. 26: 0 → 1
Char. 44: 1 → 0
Char. 79: 0 → 2
Char. 82: 0 → 1
Char. 98: 1 → 0
Char. 108: 1 → 0
Char. 113: 0 → 1
Char. 128: 0 → 1
Char. 159: 1 → 0
Char. 181: 0 → 1
Char. 185: 0 → 1
Char. 206: 0 → 2
Char. 245: 0 → 1
Char. 278: 0 → 3

***Lanthanosuchus watsoni*:**
Char. 25: 1 → 0
Char. 51: 0 → 1
Char. 86: 0 → 1
Char. 95: 0 → 1
Char. 98: 1 → 0
Char. 110: 0 → 1
Char. 113: 0 → 1
Char. 114: 0 → 1
Char. 119: 1 → 0
Char. 131: 0 → 1
Char. 137: 0 → 1
Char. 138: 0 → 1
Char. 140: 0 → 2
Char. 144: 1 → 0

Char. 154: 1 → 2
Char. 192: 0 → 1

***Macroleter poezicus*:**
Char. 0: 0 → 1
Char. 9: 0 → 1
Char. 26: 0 → 1
Char. 52: 0 → 1
Char. 66: 1 → 2
Char. 84: 1 → 0
Char. 87: 0 → 1
Char. 134: 0 → 2
Char. 139: 0 → 1
Char. 140: 0 → 1
Char. 146: 1 → 0
Char. 169: 1 → 0
Char. 235: 0 → 1

***Mesosaurus* spp.:**
Char. 0: 0 → 1
Char. 2: 0 → 1
Char. 5: 1 → 0
Char. 6: 0 → 1
Char. 8: 0 → 1
Char. 9: 0 → 1
Char. 13: 0 → 1
Char. 19: 0 → 1
Char. 23: 0 → 1
Char. 26: 0 → 1
Char. 29: 1 → 0
Char. 33: 0 → 1
Char. 38: 1 → 0
Char. 41: 0 → 1
Char. 48: 0 → 1
Char. 50: 1 → 0
Char. 67: 0 → 1
Char. 83: 0 → 1
Char. 84: 1 → 0
Char. 85: 1 → 0
Char. 94: 0 → 1
Char. 107: 0 → 1
Char. 109: 0 → 1
Char. 111: 0 → 1
Char. 115: 0 → 1
Char. 146: 1 → 0
Char. 147: 1 → 0
Char. 148: 0 → 1
Char. 149: 1 → 0
Char. 159: 1 → 0
Char. 163: 1 → 0
Char. 164: 0 → 1
Char. 166: 1 → 0
Char. 167: 0 → 1
Char. 176: 0 → 1
Char. 183: 1 → 0
Char. 184: 0 → 1
Char. 199: 0 → 1
Char. 202: 0 → 1
Char. 206: 0 → 1
Char. 207: 0 → 1
Char. 209: 0 → 1
Char. 217: 0 → 1
Char. 219: 0 → 1
Char. 220: 0 → 1
Char. 231: 0 → 1
Char. 260: 2 → 0
Char. 272: 2 → 0
Char. 278: 3 → 0

***Microleter mckinzieorum*:**
Char. 0: 0 → 1
Char. 25: 1 → 0
Char. 36: 0 → 1
Char. 39: 0 → 1
Char. 51: 0 → 2
Char. 56: 0 → 1
Char. 70: 0 → 1
Char. 94: 0 → 1
Char. 106: 0 → 1
Char. 166: 1 → 0
Char. 276: 0 → 1

**Millerettidae:**
Char. 5: 1 → 0
Char. 24: 0 → 1
Char. 25: 1 → 0
Char. 44: 1 → 0
Char. 56: 0 → 1
Char. 57: 1 → 0
Char. 66: 0 → 2
Char. 78: 1 → 0
Char. 80: 1 → 0
Char. 84: 1 → 2
Char. 88: 1 → 0
Char. 96: 1 → 0
Char. 117: 0 → 1
Char. 124: 0 → 1
Char. 127: 0 → 1
Char. 135: 0 → 1
Char. 145: 0 → 1
Char. 147: 1 → 0
Char. 159: 1 → 0
Char. 163: 1 → 0
Char. 166: 1 → 0
Char. 202: 0 → 1
Char. 211: 0 → 1
Char. 230: 0 → 1
Char. 240: 0 → 1
Char. 248: 0 → 1
Char. 252: 0 → 1
Char. 253: 0 → 1

***Nycteroleter ineptus*:**
Char. 278: 0 → 3

***Nyctiphruretus acudens*:**
Char. 0: 0 → 1
Char. 21: 0 → 1
Char. 33: 1 → 2
Char. 41: 0 → 1
Char. 66: 1 → 2
Char. 81: 1 → 0
Char. 83: 0 → 1
Char. 84: 1 → 2
Char. 85: 1 → 0
Char. 94: 0 → 1
Char. 166: 1 → 0
Char. 167: 0 → 1
Char. 224: 0 → 1
Char. 266: 0 → 1
Char. 272: 2 → 1
Char. 276: 0 → 1

***Orovenator mayorum*:**
Char. 8: 0 → 1
Char. 24: 0 → 1
Char. 36: 0 → 1
Char. 160: 0 → 1
Char. 165: 0 → 1

***Owenetta* spp.:**
Char. 169: 1 → 0

***Paleothyris acadiana*:**
Char. 38: 1 → 0
Char. 50: 1 → 0
Char. 66: 1 → 2
Char. 102: 0 → 1
Char. 146: 1 → 0
Char. 237: 0 → 1
Char. 239: 0 → 1
Char. 240: 0 → 1

***Placodus* spp.:**
Char. 0: 1 → 2
Char. 9: 0 → 1
Char. 12: 0 → 1
Char. 13: 0 → 1
Char. 19: 0 → 1
Char. 26: 0 → 1
Char. 31: 0 → 1
Char. 44: 1 → 0
Char. 46: 1 → 0
Char. 57: 0 → 1
Char. 78: 1 → 0
Char. 93: 1 → 0
Char. 102: 1 → 2
Char. 109: 1 → 0
Char. 140: 1 → 0
Char. 155: 0 → 1
Char. 163: 1 → 0
Char. 164: 0 → 1

***Procolophon* spp.:**
Char. 41: 0 → 1
Char. 69: 0 → 1
Char. 79: 0 → 1
Char. 83: 0 → 1
Char. 88: 0 → 1
Char. 117: 1 → 0
Char. 149: 1 → 0
Char. 237: 0 → 1
Char. 238: 0 → 1
Char. 272: 2 → 1

***Prolacerta broomi*:**
Char. 58: 1 → 0
Char. 66: 1 → 0
Char. 67: 1 → 0
Char. 80: 1 → 0
Char. 139: 1 → 0
Char. 147: 1 → 0
Char. 192: 1 → 0
Char. 203: 1 → 2
Char. 206: 0 → 12

***Rhipaeosaurus* spp.:**
Char. 172: 0 → 1
Char. 277: 0 → 1

**Rhynchocephalia:**
Char. 0: 1 → 2
Char. 24: 0 → 1
Char. 75: 1 → 0
Char. 77: 0 → 1
Char. 94: 1 → 0
Char. 117: 12 → 0
Char. 139: 1 → 0
Char. 167: 1 → 0
Char. 205: 1 → 0

**Rhynchosauria:**
Char. 0: 1 → 0
Char. 7: 0 → 1
Char. 9: 0 → 1
Char. 26: 0 → 1
Char. 44: 1 → 0
Char. 68: 0 → 1
Char. 99: 1 → 0
Char. 150: 1 → 0
Char. 160: 0 → 1
Char. 161: 0 → 1
Char. 171: 0 → 2
Char. 182: 1 → 0
Char. 223: 0 → 1
Char. 241: 0 → 1

***Scutosaurus* spp.:**
Char. 175: 0 → 1
Char. 218: 0 → 1
Char. 243: 0 → 2
Char. 244: 0 → 1
Char. 251: 0 → 1

***Sinosaurosphargis yunguiensis*:**
Char. 8: 0 → 1
Char. 30: 0 → 1
Char. 53: 0 → 1
Char. 82: 0 → 1
Char. 89: 1 → 0
Char. 127: 1 → 0
Char. 150: 1 → 0
Char. 154: 0 → 2
Char. 167: 1 → 0
Char. 253: 0 → 1
Char. 255: 0 → 1

**Squamata:**
Char. 26: 0 → 1
Char. 45: 0 → 1
Char. 79: 0 → 2
Char. 80: 1 → 0
Char. 82: 0 → 1
Char. 92: 1 → 0
Char. 109: 1 → 0
Char. 160: 0 → 1
Char. 245: 0 → 1

***Trilophosaurus buettneri*:**
Char. 5: 1 → 0
Char. 11: 0 → 1
Char. 55: 1 → 0
Char. 93: 1 → 0
Char. 104: 0 → 1
Char. 113: 0 → 1
Char. 122: 0 → 1
Char. 136: 1 → 0
Char. 144: 1 → 0
Char. 154: 0 → 1
Char. 157: 0 → 1
Char. 159: 1 → 0
Char. 177: 0 → 12
Char. 194: 0 → 1
Char. 203: 1 → 2
Char. 207: 1 → 0
Char. 208: 1 → 0
Char. 272: 1 → 0

***Youngina capensis*:**
Char. 5: 1 → 0
Char. 21: 0 → 1
Char. 44: 1 → 0
Char. 75: 0 → 1
Char. 92: 1 → 0
Char. 163: 1 → 0
Char. 170: 0 → 1
Char. 211: 0 → 1
Char. 231: 0 → 1
Char. 239: 0 → 1

**Node 50:**
Char. 15: 1 → 0
Char. 20: 1 → 0
Char. 25: 0 → 1
Char. 33: 2 → 0
Char. 65: 1 → 0
Char. 72: 2 → 0
Char. 73: 1 → 0
Char. 79: 2 → 1
Char. 94: 1 → 0
Char. 127: 1 → 0
Char. 131: 1 → 0
Char. 145: 1 → 0
Char. 147: 1 → 0
Char. 149: 1 → 0
Char. 174: 1 → 0
Char. 180: 1 → 0
Char. 184: 1 → 0
Char. 187: 1 → 0
Char. 188: 1 → 0
Char. 189: 1 → 0
Char. 191: 1 → 0
Char. 192: 1 → 0
Char. 194: 2 → 0
Char. 205: 1 → 0
Char. 208: 1 → 0
Char. 210: 1 → 0
Char. 217: 1 → 0
Char. 220: 1 → 0
Char. 221: 1 → 0
Char. 222: 1 → 0
Char. 223: 1 → 0

Char. 224: 1 → 0
Char. 226: 1 → 0
Char. 227: 1 → 0
Char. 231: 1 → 0
Char. 232: 1 → 0
Char. 237: 1 → 0
Char. 241: 1 → 0
Char. 246: 12 → 0
Char. 247: 1 → 0
Char. 251: 1 → 0
Char. 253: 2 → 0
Char. 254: 1 → 0
Char. 255: 1 → 0
Char. 256: 1 → 0
Char. 258: 1 → 0
Char. 266: 1 → 0
Char. 267: 1 → 0
Char. 268: 1 → 0
Char. 269: 1 → 0
Char. 272: 1 → 2

**Node 51:**
Char. 5: 0 → 1
Char. 29: 0 → 1
Char. 40: 0 → 2
Char. 74: 0 → 1
Char. 88: 0 → 1
Char. 116: 0 → 1
Char. 119: 0 → 1
Char. 147: 0 → 1
Char. 149: 0 → 1
Char. 163: 0 → 1
Char. 166: 0 → 1
Char. 169: 0 → 1
Char. 201: 0 → 1
Char. 203: 0 → 1
Char. 221: 0 → 1
Char. 235: 0 → 1

**Node 52:**
Char. 72: 0 → 1
Char. 79: 1 → 0
Char. 93: 0 → 1

**Node 53:**
Char. 23: 1 → 0
Char. 55: 0 → 1
Char. 78: 0 → 1
Char. 111: 1 → 0
Char. 126: 1 → 0
Char. 135: 1 → 0

**Node 54:**
Char. 33: 0 → 1
Char. 135: 0 → 1

**Node 55:**
Char. 20: 0 → 1
Char. 62: 0 → 1

**Node 56:**
Char. 0: 0 → 1
Char. 27: 0 → 1
Char. 29: 1 → 0
Char. 38: 1 → 0

Char. 40: 2 → 0
Char. 57: 1 → 0
Char. 59: 0 → 1
Char. 60: 0 → 1
Char. 67: 0 → 1
Char. 72: 1 → 2
Char. 84: 1 → 0
Char. 89: 0 → 1
Char. 111: 0 → 1
Char. 112: 0 → 1
Char. 120: 0 → 1
Char. 147: 1 → 0
Char. 154: 1 → 0
Char. 192: 0 → 1
Char. 193: 0 → 1
Char. 222: 0 → 1
Char. 237: 0 → 1
Char. 240: 0 → 1
Char. 266: 0 → 1
Char. 278: 3 → 1

**Node 57:**
Char. 19: 0 → 1
Char. 92: 1 → 0

**Node 58:**
Char. 4: 0 → 1
Char. 15: 0 → 1
Char. 29: 0 → 1
Char. 213: 0 → 1
Char. 226: 0 → 2
Char. 228: 0 → 1
Char. 275: 0 → 1

**Node 59:**
Char. 27: 1 → 0
Char. 75: 0 → 1
Char. 107: 0 → 1
Char. 140: 0 → 1
Char. 147: 0 → 1

**Node 60:**
Char. 58: 0 → 1
Char. 61: 0 → 1
Char. 66: 0 → 1
Char. 69: 0 → 1
Char. 150: 0 → 1
Char. 167: 0 → 1
Char. 205: 0 → 1
Char. 208: 0 → 1
Char. 239: 0 → 1

**Node 61:**
Char. 70: 0 → 1
Char. 73: 0 → 1
Char. 126: 0 → 1
Char. 131: 0 → 1
Char. 182: 0 → 1
Char. 190: 0 → 1

**Node 62:**
Char. 24: 0 → 1
Char. 57: 1 → 0
Char. 79: 0 → 1
Char. 83: 0 → 1

Char. 110: 0 → 1
Char. 132: 1 → 0

**Node 63:**
Char. 73: 0 → 1
Char. 131: 1 → 0
Char. 205: 0 → 1
Char. 276: 0 → 1

**Node 64:**
Char. 18: 0 → 1
Char. 37: 0 → 1
Char. 100: 1 → 0
Char. 107: 0 → 1
Char. 113: 1 → 0
Char. 118: 1 → 0
Char. 125: 1 → 0
Char. 150: 0 → 1

**Node 65:**
Char. 23: 0 → 1
Char. 71: 1 → 0
Char. 85: 1 → 0
Char. 102: 0 → 1
Char. 103: 0 → 1
Char. 106: 0 → 1
Char. 167: 0 → 1
Char. 214: 0 → 1
Char. 216: 1 → 0
Char. 235: 0 → 2
Char. 241: 0 → 1

**Node 66:**
Char. 207: 0 → 1

**Node 67:**
Char. 215: 0 → 1
Char. 226: 0 → 1

**Node 68:**
Char. 20: 0 → 1
Char. 33: 0 → 1
Char. 38: 1 → 2
Char. 39: 0 → 1
Char. 44: 1 → 0
Char. 49: 0 → 1
Char. 51: 0 → 1
Char. 66: 0 → 1
Char. 70: 0 → 1
Char. 80: 1 → 0
Char. 88: 1 → 0
Char. 95: 0 → 1
Char. 112: 0 → 1
Char. 117: 0 → 1
Char. 118: 0 → 1
Char. 125: 0 → 1
Char. 131: 0 → 1
Char. 137: 0 → 1
Char. 148: 0 → 1
Char. 158: 0 → 1
Char. 183: 1 → 2
Char. 192: 0 → 1
Char. 194: 0 → 1
Char. 201: 1 → 0
Char. 211: 0 → 1

Char. 235: 1 → 0
Char. 252: 0 → 1

**Node 69:**
Char. 87: 0 → 1

**Node 70:**
Char. 25: 1 → 0
Char. 76: 1 → 0

**Node 71:**
Char. 79: 0 → 1
Char. 93: 1 → 0
Char. 133: 0 → 1

**Node 72:**
Char. 38: 1 → 2
Char. 39: 0 → 1
Char. 50: 1 → 0
Char. 58: 0 → 1
Char. 59: 0 → 1
Char. 60: 0 → 1
Char. 72: 1 → 2
Char. 83: 0 → 1
Char. 85: 1 → 0
Char. 88: 1 → 0
Char. 95: 0 → 1
Char. 104: 0 → 1
Char. 105: 0 → 1
Char. 106: 0 → 2
Char. 107: 0 → 1
Char. 109: 0 → 1
Char. 110: 0 → 1
Char. 146: 1 → 0
Char. 148: 0 → 1
Char. 155: 0 → 2
Char. 183: 1 → 2

**Node 73:**
Char. 0: 0 → 2
Char. 33: 1 → 0
Char. 38: 2 → 1
Char. 39: 1 → 0
Char. 42: 0 → 1
Char. 43: 0 → 1
Char. 46: 1 → 0
Char. 48: 1 → 0
Char. 49: 1 → 0
Char. 52: 0 → 1
Char. 83: 0 → 2
Char. 84: 1 → 0
Char. 87: 0 → 1
Char. 93: 1 → 0
Char. 132: 0 → 1
Char. 161: 0 → 1
Char. 163: 1 → 0
Char. 172: 0 → 2
Char. 174: 0 → 1
Char. 188: 0 → 1
Char. 189: 0 → 1
Char. 195: 0 → 1
Char. 212: 0 → 1
Char. 221: 1 → 0
Char. 236: 0 → 1
Char. 238: 0 → 2

Char. 242: 0 → 1
Char. 245: 0 → 1
Char. 274: 0 → 1
Char. 275: 1 → 0

**Node 74:**
Char. 5: 1 → 0
Char. 29: 1 → 0
Char. 44: 1 → 0
Char. 59: 0 → 1
Char. 60: 0 → 1
Char. 66: 0 → 1
Char. 67: 0 → 1
Char. 72: 1 → 2
Char. 78: 1 → 0
Char. 84: 1 → 0
Char. 88: 1 → 0
Char. 93: 1 → 0
Char. 111: 0 → 1
Char. 123: 1 → 0
Char. 129: 0 → 1
Char. 147: 1 → 0
Char. 154: 1 → 0
Char. 159: 1 → 0
Char. 163: 1 → 0
Char. 169: 1 → 0
Char. 170: 0 → 1
Char. 197: 0 → 1
Char. 221: 1 → 0

**Node 75:**
Char. 20: 0 → 1
Char. 47: 0 → 1
Char. 83: 0 → 1
Char. 85: 1 → 0
Char. 107: 0 → 1
Char. 110: 0 → 1
Char. 169: 1 → 0
Char. 170: 0 → 1

**Node 76:**
Char. 2: 0 → 1
Char. 6: 0 → 1
Char. 98: 1 → 0
Char. 101: 0 → 1
Char. 113: 0 → 1
Char. 181: 0 → 1
Char. 186: 0 → 1
Char. 193: 1 → 0
Char. 198: 0 → 1
Char. 206: 0 → 2
Char. 220: 0 → 1
Char. 239: 1 → 0
Char. 240: 1 → 0
Char. 266: 1 → 0

**Node 77:**
Char. 33: 1 → 2
Char. 68: 0 → 1
Char. 102: 0 → 1
Char. 103: 0 → 1
Char. 223: 0 → 1
Char. 229: 0 → 1
Char. 235: 1 → 2

**Node 78:**
Char. 17: 0 → 1
Char. 42: 0 → 1
Char. 251: 0 → 1
Char. 264: 0 → 1

**Node 79:**
Char. 41: 0 → 1
Char. 61: 1 → 2
Char. 112: 1 → 0
Char. 128: 0 → 1
Char. 138: 0 → 1
Char. 146: 1 → 0
Char. 155: 0 → 1
Char. 182: 1 → 0
Char. 192: 1 → 2
Char. 226: 0 → 1
Char. 227: 0 → 1
Char. 233: 0 → 1

**Node 80:**
Char. 61: 1 → 3
Char. 90: 0 → 1
Char. 91: 0 → 1
Char. 109: 1 → 0
Char. 155: 0 → 1
Char. 209: 1 → 0

**ANALYSIS 24**
**(NO *PROGANOCHELYS QUENSTEDTI*, *CANDELARIA BARBOURI*, *PAPPOCHELYS ROSINAE*, AND *EUNOTOSAURUS AFRICANUS*)**

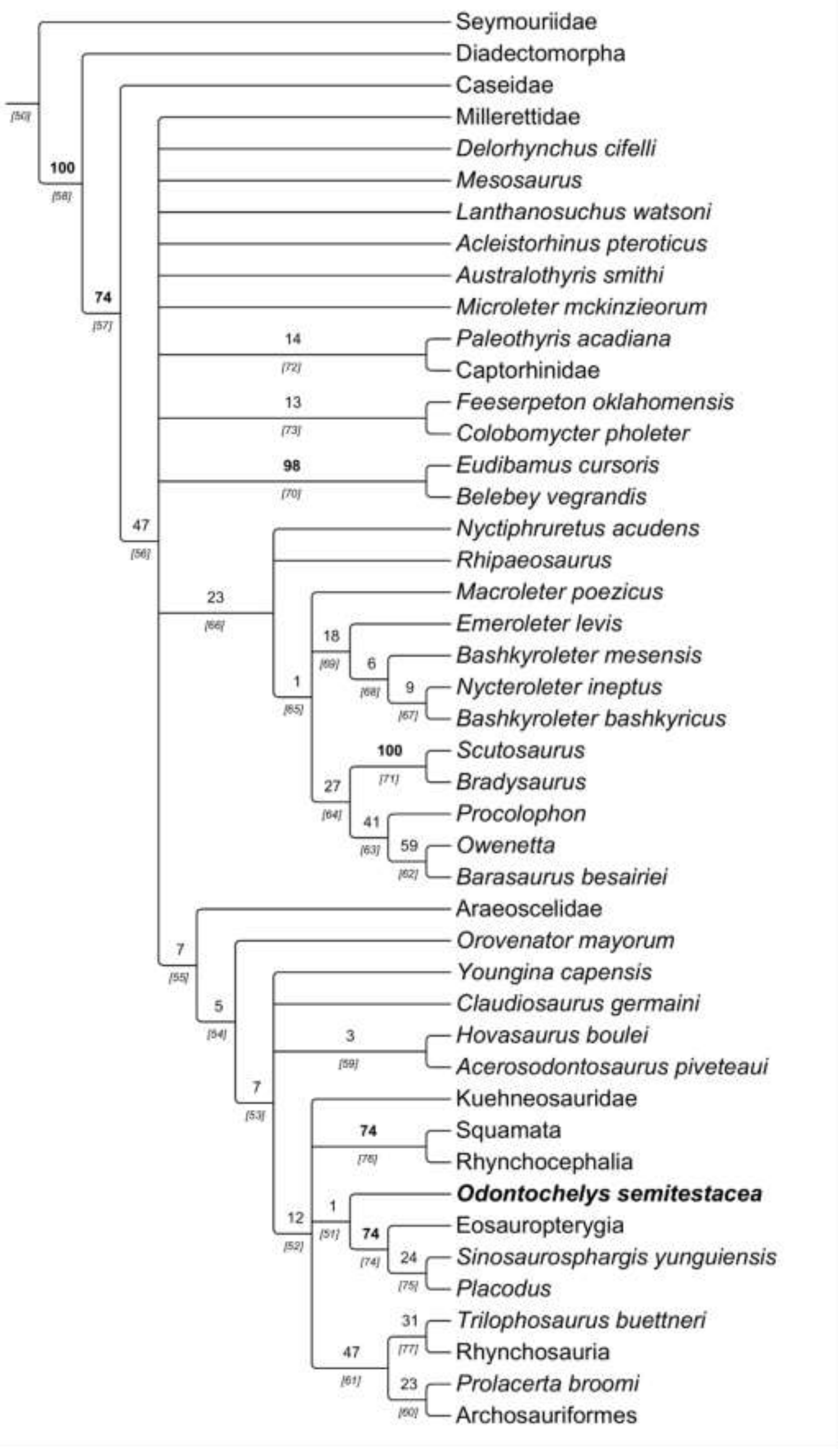

**Seymouriidae:**
Char. 51: 0 → 2
Char. 54: 1 → 0
Char. 71: 1 → 0
Char. 83: 0 → 1
Char. 85: 1 → 0
Char. 99: 1 → 0
Char. 107: 0 → 1
Char. 140: 0 → 2
Char. 154: 1 → 2
Char. 207: 0 → 1
Char. 225: 1 → 0
Char. 260: 2 → 0

***Odontochelys semitestacea*:**
Char. 46: 1 → 0
Char. 50: 0 → 1
Char. 58: 1 → 0
Char. 61: 1 → 0
Char. 62: 1 → 0
Char. 65: 0 → 1
Char. 89: 1 → 0
Char. 93: 1 → 0
Char. 109: 1 → 0
Char. 112: 1 → 0
Char. 124: 0 → 1
Char. 126: 1 → 0
Char. 150: 1 → 0
Char. 154: 0 → 1
Char. 167: 1 → 0
Char. 195: 0 → 2
Char. 201: 1 → 0
Char. 219: 1 → 0
Char. 226: 0 → 1
Char. 227: 0 → 1
Char. 238: 0 → 1
Char. 241: 0 → 1
Char. 246: 0 → 2
Char. 247: 0 → 1
Char. 252: 0 → 1
Char. 253: 0 → 2
Char. 254: 0 → 1
Char. 255: 0 → 1
Char. 256: 0 → 1
Char. 258: 0 → 1
Char. 259: 0 → 1
Char. 260: 1 → 2
Char. 265: 1 → 0
Char. 268: 0 → 1
Char. 269: 0 → 1
Char. 270: 0 → 1

***Acerosodontosaurus piveteaui*:**
Char. 81: 1 → 0
Char. 208: 0 → 1
Char. 224: 1 → 0

***Acleistorhinus pteroticus*:**
Char. 20: 0 → 1
Char. 21: 0 → 1
Char. 30: 0 → 1
Char. 33: 0 → 1
Char. 47: 0 → 1
Char. 55: 1 → 0
Char. 56: 0 → 1
Char. 64: 0 → 1
Char. 70: 0 → 1
Char. 79: 0 → 1
Char. 95: 0 → 1
Char. 110: 0 → 1
Char. 113: 0 → 1
Char. 114: 0 → 1
Char. 131: 0 → 1
Char. 137: 0 → 1
Char. 140: 0 → 2
Char. 146: 1 → 0
Char. 169: 1 → 0
Char. 170: 0 → 1
Char. 278: 3 → 1

**Araeoscelidae:**
Char. 5: 1 → 0
Char. 28: 0 → 1
Char. 106: 0 → 1
Char. 116: 1 → 0
Char. 169: 1 → 0
Char. 170: 0 → 1
Char. 197: 0 → 1

**Archosauriformes:**
Char. 32: 0 → 1
Char. 94: 1 → 0
Char. 112: 1 → 0
Char. 152: 0 → 1
Char. 154: 0 → 2
Char. 166: 1 → 0
Char. 171: 0 → 1
Char. 185: 0 → 1
Char. 204: 0 → 1
Char. 218: 0 → 3
Char. 242: 0 → 1

***Australothyris smithi*:**
Char. 23: 0 → 1
Char. 24: 0 → 1
Char. 34: 0 → 1
Char. 55: 1 → 0
Char. 57: 1 → 0
Char. 71: 1 → 0
Char. 79: 0 → 1
Char. 83: 0 → 1
Char. 85: 1 → 0
Char. 98: 1 → 0
Char. 100: 0 → 1
Char. 103: 0 → 1
Char. 110: 0 → 1
Char. 112: 0 → 1
Char. 123: 1 → 0
Char. 129: 0 → 1
Char. 131: 0 → 1
Char. 132: 1 → 0
Char. 144: 1 → 0
Char. 149: 1 → 0
Char. 150: 0 → 1

***Barasaurus besairiei*:**
Char. 33: 1 → 0
Char. 75: 1 → 0
Char. 216: 0 → 1

***Bashkyroleter bashkyricus*:**
Char. 275: 1 → 0

***Bashkyroleter mesensis*:**
Char. 169: 1 → 0

***Belebey vegrandis*:**
Char. 154: 1 → 0

***Bradysaurus* spp.:**
Char. 19: 0 → 1
Char. 73: 0 → 1
Char. 79: 0 → 1
Char. 135: 1 → 0
Char. 249: 0 → 1

**Captorhinidae:**
Char. 3: 0 → 1
Char. 23: 0 → 1
Char. 25: 1 → 0
Char. 26: 0 → 1
Char. 73: 0 → 1
Char. 75: 0 → 1
Char. 83: 0 → 1
Char. 108: 1 → 0
Char. 183: 1 → 0
Char. 201: 1 → 0
Char. 203: 1 → 2
Char. 216: 1 → 0

**Caseidae:**
Char. 24: 0 → 1
Char. 25: 1 → 0
Char. 36: 0 → 1
Char. 38: 1 → 0
Char. 46: 1 → 0
Char. 50: 1 → 0
Char. 56: 0 → 1
Char. 85: 1 → 0
Char. 96: 1 → 0
Char. 98: 1 → 0
Char. 170: 0 → 1
Char. 194: 0 → 1
Char. 273: 0 → 1
Char. 274: 0 → 1
Char. 278: 3 → 2

***Claudiosaurus germaini*:**
Char. 24: 0 → 1
Char. 34: 0 → 1
Char. 36: 0 → 1
Char. 43: 1 → 0
Char. 64: 0 → 1
Char. 73: 0 → 1
Char. 105: 0 → 1
Char. 106: 0 → 1
Char. 126: 0 → 1
Char. 130: 0 → 1
Char. 131: 0 → 1
Char. 144: 1 → 0
Char. 148: 0 → 1
Char. 182: 0 → 1
Char. 187: 1 → 0
Char. 199: 0 → 1
Char. 203: 1 → 2
Char. 204: 0 → 1
Char. 220: 0 → 1
Char. 222: 1 → 0
Char. 224: 1 → 0
Char. 234: 1 → 0
Char. 272: 2 → 1

***Colobomycter pholeter*:**
Char. 21: 0 → 1
Char. 25: 1 → 0
Char. 84: 1 → 0
Char. 154: 1 → 0
Char. 167: 0 → 1
Char. 267: 0 → 1

***Delorhynchus cifelli*:**
Char. 18: 0 → 1
Char. 20: 0 → 1
Char. 21: 0 → 1
Char. 24: 0 → 1
Char. 26: 0 → 1
Char. 28: 0 → 1
Char. 33: 0 → 2
Char. 39: 0 → 1
Char. 52: 0 → 1
Char. 100: 0 → 1
Char. 111: 0 → 1
Char. 116: 1 → 0
Char. 117: 0 → 1
Char. 131: 0 → 1
Char. 156: 1 → 0
Char. 167: 0 → 1
Char. 180: 0 → 1
Char. 189: 0 → 1
Char. 191: 0 → 1
Char. 204: 0 → 2
Char. 267: 0 → 1

**Diadectomorpha:**
Char. 0: 0 → 1
Char. 70: 0 → 1
Char. 75: 0 → 1
Char. 122: 0 → 1
Char. 123: 1 → 0
Char. 146: 1 → 0
Char. 275: 1 → 0
Char. 278: 3 → 0

***Emeroleter levis*:**
Char. 0: 0 → 2
Char. 51: 1 → 0

**Eosauropterygia:**
Char. 159: 1 → 0
Char. 166: 1 → 0
Char. 168: 0 → 1
Char. 178: 0 → 1
Char. 272: 1 → 0

***Eudibamus cursoris*:**
Char. 154: 1 → 2

***Feeserpeton oklahomensis*:**
Char. 51: 0 → 1
Char. 70: 0 → 1
Char. 157: 0 → 1
Char. 158: 0 → 1

***Hovasaurus boulei*:**
Char. 41: 0 → 1
Char. 77: 0 → 2
Char. 79: 0 → 1
Char. 93: 1 → 0
Char. 204: 0 → 2

**Kuehneosauridae:**
Char. 7: 0 → 1
Char. 24: 0 → 1
Char. 26: 0 → 1
Char. 27: 0 → 1
Char. 34: 0 → 1
Char. 36: 0 → 1
Char. 43: 1 → 0
Char. 44: 1 → 0
Char. 79: 0 → 2
Char. 82: 0 → 1
Char. 98: 1 → 0
Char. 108: 1 → 0
Char. 113: 0 → 1
Char. 128: 0 → 1
Char. 140: 1 → 0
Char. 147: 1 → 0
Char. 148: 1 → 0
Char. 159: 1 → 0
Char. 168: 0 → 1
Char. 181: 0 → 1
Char. 185: 0 → 1
Char. 206: 0 → 2
Char. 245: 0 → 1
Char. 278: 0 → 3

***Lanthanosuchus watsoni*:**
Char. 25: 1 → 0
Char. 51: 0 → 1
Char. 76: 0 → 1
Char. 86: 0 → 1
Char. 95: 0 → 1
Char. 98: 1 → 0
Char. 110: 0 → 1
Char. 113: 0 → 1
Char. 114: 0 → 1
Char. 131: 0 → 1
Char. 137: 0 → 1
Char. 138: 0 → 1
Char. 140: 0 → 2
Char. 144: 1 → 0
Char. 154: 1 → 2

***Macroleter poezicus*:**
Char. 0: 0 → 1
Char. 9: 0 → 1
Char. 26: 0 → 1
Char. 52: 0 → 1
Char. 66: 1 → 2

Char. 84: 1 → 0
Char. 87: 0 → 1
Char. 134: 0 → 2
Char. 139: 0 → 1
Char. 140: 0 → 1
Char. 146: 1 → 0
Char. 169: 1 → 0
Char. 235: 0 → 1

***Mesosaurus* spp.:**
Char. 0: 0 → 1
Char. 2: 0 → 1
Char. 5: 1 → 0
Char. 6: 0 → 1
Char. 8: 0 → 1
Char. 9: 0 → 1
Char. 13: 0 → 1
Char. 19: 0 → 1
Char. 23: 0 → 1
Char. 26: 0 → 1
Char. 29: 1 → 0
Char. 33: 0 → 1
Char. 38: 1 → 0
Char. 41: 0 → 1
Char. 50: 1 → 0
Char. 67: 0 → 1
Char. 76: 0 → 1
Char. 83: 0 → 1
Char. 84: 1 → 0
Char. 85: 1 → 0
Char. 94: 0 → 1
Char. 107: 0 → 1
Char. 109: 0 → 1
Char. 111: 0 → 1
Char. 115: 0 → 1
Char. 146: 1 → 0
Char. 148: 0 → 1
Char. 149: 1 → 0
Char. 164: 0 → 1
Char. 167: 0 → 1
Char. 176: 0 → 1
Char. 183: 1 → 0
Char. 184: 0 → 1
Char. 199: 0 → 1
Char. 202: 0 → 1
Char. 204: 0 → 2
Char. 206: 0 → 1
Char. 207: 0 → 1
Char. 209: 0 → 1
Char. 217: 0 → 1
Char. 219: 0 → 1
Char. 220: 0 → 1
Char. 231: 0 → 1
Char. 260: 2 → 0
Char. 272: 2 → 0
Char. 278: 3 → 0

***Microleter mckinzieorum*:**
Char. 0: 0 → 1
Char. 18: 0 → 1
Char. 24: 0 → 1
Char. 25: 1 → 0
Char. 36: 0 → 1
Char. 39: 0 → 1
Char. 51: 0 → 2
Char. 56: 0 → 1
Char. 57: 1 → 0
Char. 70: 0 → 1
Char. 76: 0 → 1
Char. 79: 0 → 1
Char. 83: 0 → 1
Char. 94: 0 → 1
Char. 106: 0 → 1
Char. 110: 0 → 1
Char. 132: 1 → 0
Char. 276: 0 → 1
Char. 278: 3 → 1

**Millerettidae:**
Char. 5: 1 → 0
Char. 24: 0 → 1
Char. 25: 1 → 0
Char. 44: 1 → 0
Char. 56: 0 → 1
Char. 57: 1 → 0
Char. 66: 0 → 2
Char. 78: 1 → 0
Char. 80: 1 → 0
Char. 84: 1 → 2
Char. 88: 1 → 0
Char. 96: 1 → 0
Char. 117: 0 → 1
Char. 124: 0 → 1
Char. 127: 0 → 1
Char. 135: 0 → 1
Char. 145: 0 → 1
Char. 202: 0 → 1
Char. 211: 0 → 1
Char. 230: 0 → 1
Char. 234: 0 → 1
Char. 248: 0 → 1
Char. 252: 0 → 1
Char. 253: 0 → 1

***Nycteroleter ineptus*:**
Char. 278: 0 → 3

***Nyctiphruretus acudens*:**
Char. 0: 0 → 1
Char. 21: 0 → 1
Char. 33: 1 → 2
Char. 41: 0 → 1
Char. 66: 1 → 2
Char. 81: 1 → 0
Char. 83: 0 → 1
Char. 84: 1 → 2
Char. 85: 1 → 0
Char. 94: 0 → 1
Char. 167: 0 → 1
Char. 224: 0 → 1
Char. 266: 0 → 1
Char. 272: 2 → 1
Char. 276: 0 → 1

***Orovenator mayorum*:**
Char. 8: 0 → 1
Char. 24: 0 → 1
Char. 36: 0 → 1
Char. 160: 0 → 1
Char. 165: 0 → 1

***Owenetta* spp.:**
Char. 169: 1 → 0

***Paleothyris acadiana*:**
Char. 38: 1 → 0
Char. 50: 1 → 0
Char. 66: 1 → 2
Char. 102: 0 → 1
Char. 146: 1 → 0
Char. 180: 0 → 1
Char. 237: 0 → 1

***Placodus* spp.:**
Char. 9: 0 → 1
Char. 12: 0 → 1
Char. 13: 0 → 1
Char. 19: 0 → 1
Char. 26: 0 → 1
Char. 31: 0 → 1
Char. 44: 1 → 0
Char. 46: 1 → 0
Char. 57: 0 → 1
Char. 78: 1 → 0
Char. 93: 1 → 0
Char. 109: 1 → 0
Char. 140: 1 → 0
Char. 155: 0 → 1
Char. 163: 1 → 0
Char. 164: 0 → 1

***Procolophon* spp.:**
Char. 41: 0 → 1
Char. 69: 0 → 1
Char. 79: 0 → 1
Char. 83: 0 → 1
Char. 88: 0 → 1
Char. 117: 1 → 0
Char. 149: 1 → 0
Char. 180: 0 → 1
Char. 204: 0 → 1
Char. 237: 0 → 1
Char. 238: 0 → 1
Char. 272: 2 → 1

***Prolacerta broomi*:**
Char. 58: 1 → 0
Char. 66: 1 → 0
Char. 67: 1 → 0
Char. 80: 1 → 0
Char. 139: 1 → 0
Char. 147: 1 → 0
Char. 192: 1 → 0
Char. 203: 1 → 2
Char. 206: 0 → 12
Char. 224: 1 → 0

***Rhipaeosaurus* spp.:**
Char. 172: 0 → 1
Char. 277: 0 → 1

**Rhynchocephalia:**
Char. 0: 1 → 2
Char. 23: 1 → 0
Char. 24: 0 → 1
Char. 77: 0 → 1
Char. 88: 1 → 0
Char. 94: 1 → 0
Char. 104: 0 → 1
Char. 117: 12 → 0
Char. 139: 1 → 0
Char. 167: 1 → 0
Char. 205: 1 → 0

**Rhynchosauria:**
Char. 0: 1 → 0
Char. 7: 0 → 1
Char. 9: 0 → 1
Char. 26: 0 → 1
Char. 44: 1 → 0
Char. 63: 1 → 0
Char. 68: 0 → 1
Char. 99: 1 → 0
Char. 150: 1 → 0
Char. 160: 0 → 1
Char. 161: 0 → 1
Char. 171: 0 → 2
Char. 241: 0 → 1

***Scutosaurus* spp.:**
Char. 175: 0 → 1
Char. 218: 0 → 1
Char. 243: 0 → 2
Char. 244: 0 → 1
Char. 251: 0 → 1

***Sinosaurosphargis yunguiensis*:**
Char. 8: 0 → 1
Char. 30: 0 → 1
Char. 53: 0 → 1
Char. 82: 0 → 1
Char. 89: 1 → 0
Char. 127: 1 → 0
Char. 150: 1 → 0
Char. 154: 0 → 2
Char. 167: 1 → 0
Char. 253: 0 → 1
Char. 255: 0 → 1

**Squamata:**
Char. 26: 0 → 1
Char. 45: 0 → 1
Char. 75: 0 → 1
Char. 79: 0 → 2
Char. 80: 1 → 0
Char. 82: 0 → 1
Char. 92: 1 → 0
Char. 106: 0 → 1
Char. 109: 1 → 0
Char. 122: 0 → 1
Char. 160: 0 → 1
Char. 200: 0 → 1
Char. 245: 0 → 1

***Trilophosaurus buettneri*:**
Char. 5: 1 → 0
Char. 11: 0 → 1
Char. 55: 1 → 0
Char. 93: 1 → 0
Char. 104: 0 → 1
Char. 106: 0 → 1
Char. 113: 0 → 1
Char. 122: 0 → 1
Char. 136: 1 → 0
Char. 144: 1 → 0
Char. 154: 0 → 1
Char. 157: 0 → 1
Char. 159: 1 → 0
Char. 177: 0 → 12
Char. 194: 0 → 1
Char. 203: 1 → 2
Char. 207: 1 → 0
Char. 208: 1 → 0
Char. 224: 1 → 0
Char. 272: 1 → 0

***Youngina capensis*:**
Char. 5: 1 → 0
Char. 23: 1 → 0
Char. 44: 1 → 0
Char. 75: 0 → 1
Char. 92: 1 → 0
Char. 94: 1 → 0
Char. 117: 12 → 0
Char. 163: 1 → 0
Char. 170: 0 → 1
Char. 201: 1 → 0
Char. 211: 0 → 1
Char. 215: 0 → 1
Char. 231: 0 → 1

**Node 50:**
Char. 15: 1 → 0
Char. 20: 1 → 0
Char. 25: 0 → 1
Char. 33: 2 → 0
Char. 73: 1 → 0
Char. 89: 1 → 0
Char. 94: 1 → 0
Char. 119: 1 → 0
Char. 127: 1 → 0
Char. 130: 1 → 0
Char. 134: 2 → 0
Char. 136: 1 → 0
Char. 141: 1 → 0
Char. 145: 1 → 0
Char. 147: 1 → 0
Char. 149: 1 → 0
Char. 151: 1 → 0
Char. 152: 1 → 0
Char. 155: 1 → 0
Char. 158: 1 → 0
Char. 161: 1 → 0
Char. 163: 1 → 0
Char. 166: 1 → 0
Char. 174: 1 → 0
Char. 180: 1 → 0
Char. 181: 1 → 0
Char. 187: 1 → 0
Char. 188: 1 → 0
Char. 194: 2 → 0

Char. 196: 1 → 0
Char. 203: 2 → 0
Char. 204: 2 → 0
Char. 217: 1 → 0
Char. 220: 1 → 0
Char. 221: 1 → 0
Char. 231: 1 → 0
Char. 234: 1 → 0
Char. 247: 1 → 0
Char. 251: 1 → 0
Char. 253: 2 → 0
Char. 266: 1 → 0

**Node 51:**
Char. 33: 1 → 2
Char. 88: 1 → 0
Char. 189: 0 → 1
Char. 193: 1 → 0
Char. 198: 0 → 1
Char. 206: 0 → 2
Char. 220: 0 → 1
Char. 235: 1 → 2
Char. 239: 1 → 0
Char. 240: 1 → 0

**Node 52:**
Char. 27: 1 → 0
Char. 35: 0 → 1
Char. 58: 0 → 1
Char. 61: 0 → 1
Char. 63: 0 → 1
Char. 66: 0 → 1
Char. 69: 0 → 1
Char. 73: 0 → 1
Char. 84: 0 → 1
Char. 126: 0 → 1
Char. 131: 0 → 1
Char. 140: 0 → 1
Char. 145: 0 → 1
Char. 147: 0 → 1
Char. 148: 0 → 1
Char. 150: 0 → 1
Char. 151: 0 → 1
Char. 167: 0 → 1
Char. 176: 0 → 1
Char. 184: 0 → 1
Char. 205: 0 → 1
Char. 208: 0 → 1
Char. 210: 0 → 1
Char. 230: 0 → 1
Char. 231: 0 → 1
Char. 232: 0 → 1

**Node 53:**
Char. 33: 0 → 1
Char. 94: 0 → 1
Char. 135: 0 → 1
Char. 267: 0 → 1
Char. 278: 1 → 0

**Node 54:**
Char. 20: 0 → 1
Char. 62: 0 → 1

**Node 55:**
Char. 0: 0 → 1
Char. 27: 0 → 1
Char. 29: 1 → 0
Char. 38: 1 → 0
Char. 40: 2 → 0
Char. 57: 1 → 0
Char. 59: 0 → 1
Char. 60: 0 → 1
Char. 67: 0 → 1
Char. 72: 1 → 2
Char. 84: 1 → 0
Char. 89: 0 → 1
Char. 111: 0 → 1
Char. 112: 0 → 1
Char. 117: 0 → 12
Char. 120: 0 → 1
Char. 154: 1 → 0
Char. 180: 0 → 1
Char. 193: 0 → 1
Char. 222: 0 → 1
Char. 224: 0 → 1
Char. 234: 0 → 1
Char. 237: 0 → 1
Char. 266: 0 → 1
Char. 278: 3 → 1

**Node 56:**
Char. 5: 0 → 1
Char. 29: 0 → 1
Char. 40: 0 → 2
Char. 74: 0 → 1
Char. 88: 0 → 1
Char. 116: 0 → 1
Char. 149: 0 → 1
Char. 169: 0 → 1
Char. 201: 0 → 1
Char. 203: 0 → 1
Char. 235: 0 → 1
Char. 276: 1 → 0

**Node 57:**
Char. 81: 0 → 1
Char. 97: 0 → 1

**Node 58:**
Char. 55: 0 → 1
Char. 78: 0 → 1
Char. 111: 1 → 0
Char. 126: 1 → 0
Char. 135: 1 → 0

**Node 59:**
Char. 78: 1 → 0
Char. 206: 0 → 2

**Node 60:**
Char. 19: 0 → 1
Char. 92: 1 → 0

**Node 61:**
Char. 4: 0 → 1
Char. 15: 0 → 1
Char. 29: 0 → 1
Char. 35: 1 → 0

Char. 48: 1 → 0
Char. 75: 0 → 1
Char. 210: 1 → 0
Char. 213: 0 → 1
Char. 226: 0 → 2
Char. 228: 0 → 1
Char. 275: 0 → 1

**Node 62:**
Char. 73: 0 → 1
Char. 131: 1 → 0
Char. 205: 0 → 1
Char. 276: 0 → 1

**Node 63:**
Char. 18: 0 → 1
Char. 37: 0 → 1
Char. 100: 1 → 0
Char. 107: 0 → 1
Char. 113: 1 → 0
Char. 118: 1 → 0
Char. 125: 1 → 0
Char. 150: 0 → 1

**Node 64:**
Char. 23: 0 → 1
Char. 71: 1 → 0
Char. 85: 1 → 0
Char. 102: 0 → 1
Char. 103: 0 → 1
Char. 106: 0 → 1
Char. 167: 0 → 1
Char. 214: 0 → 1
Char. 216: 1 → 0
Char. 235: 0 → 2
Char. 241: 0 → 1

**Node 65:**
Char. 100: 0 → 1
Char. 113: 0 → 1
Char. 132: 1 → 0
Char. 138: 0 → 1
Char. 141: 0 → 1
Char. 207: 0 → 1

**Node 66:**
Char. 20: 0 → 1
Char. 33: 0 → 1
Char. 38: 1 → 2
Char. 39: 0 → 1
Char. 44: 1 → 0
Char. 49: 0 → 1
Char. 51: 0 → 1
Char. 66: 0 → 1
Char. 70: 0 → 1
Char. 76: 0 → 1
Char. 80: 1 → 0
Char. 88: 1 → 0
Char. 95: 0 → 1
Char. 112: 0 → 1
Char. 117: 0 → 1
Char. 118: 0 → 1
Char. 125: 0 → 1
Char. 131: 0 → 1
Char. 137: 0 → 1

Char. 148: 0 → 1
Char. 158: 0 → 1
Char. 183: 1 → 2
Char. 194: 0 → 1
Char. 201: 1 → 0
Char. 211: 0 → 1
Char. 235: 1 → 0
Char. 252: 0 → 1

**Node 67:**
Char. 87: 0 → 1

**Node 68:**
Char. 25: 1 → 0
Char. 76: 1 → 0

**Node 69:**
Char. 79: 0 → 1
Char. 93: 1 → 0
Char. 133: 0 → 1

**Node 70:**
Char. 38: 1 → 2
Char. 39: 0 → 1
Char. 50: 1 → 0
Char. 58: 0 → 1
Char. 59: 0 → 1
Char. 60: 0 → 1
Char. 72: 1 → 2
Char. 83: 0 → 1
Char. 85: 1 → 0
Char. 88: 1 → 0
Char. 95: 0 → 1
Char. 104: 0 → 1
Char. 105: 0 → 1
Char. 106: 0 → 2
Char. 107: 0 → 1
Char. 109: 0 → 1
Char. 110: 0 → 1
Char. 146: 1 → 0
Char. 148: 0 → 1
Char. 155: 0 → 2
Char. 183: 1 → 2

**Node 71:**
Char. 0: 0 → 2
Char. 33: 1 → 0
Char. 38: 2 → 1
Char. 39: 1 → 0
Char. 42: 0 → 1
Char. 43: 0 → 1
Char. 46: 1 → 0
Char. 48: 1 → 0
Char. 49: 1 → 0
Char. 52: 0 → 1
Char. 83: 0 → 2
Char. 84: 1 → 0
Char. 87: 0 → 1
Char. 93: 1 → 0
Char. 132: 0 → 1
Char. 161: 0 → 1
Char. 163: 1 → 0
Char. 172: 0 → 2
Char. 174: 0 → 1
Char. 188: 0 → 1

Char. 189: 0 → 1
Char. 195: 0 → 1
Char. 204: 0 → 2
Char. 212: 0 → 1
Char. 236: 0 → 1
Char. 238: 0 → 2
Char. 242: 0 → 1
Char. 245: 0 → 1
Char. 274: 0 → 1
Char. 275: 1 → 0

**Node 72:**
Char. 5: 1 → 0
Char. 29: 1 → 0
Char. 44: 1 → 0
Char. 59: 0 → 1
Char. 60: 0 → 1
Char. 66: 0 → 1
Char. 67: 0 → 1
Char. 72: 1 → 2
Char. 78: 1 → 0
Char. 84: 1 → 0
Char. 88: 1 → 0
Char. 93: 1 → 0
Char. 111: 0 → 1
Char. 123: 1 → 0
Char. 129: 0 → 1
Char. 154: 1 → 0
Char. 169: 1 → 0
Char. 170: 0 → 1
Char. 197: 0 → 1

**Node 73:**
Char. 20: 0 → 1
Char. 47: 0 → 1
Char. 83: 0 → 1
Char. 85: 1 → 0
Char. 107: 0 → 1
Char. 110: 0 → 1
Char. 169: 1 → 0
Char. 170: 0 → 1

**Node 74:**
Char. 2: 0 → 1
Char. 6: 0 → 1
Char. 101: 0 → 1
Char. 104: 0 → 1
Char. 106: 0 → 1
Char. 113: 0 → 1
Char. 182: 0 → 1
Char. 186: 0 → 1
Char. 224: 1 → 0
Char. 229: 0 → 1
Char. 231: 1 → 0
Char. 232: 1 → 0
Char. 267: 1 → 0

**Node 75:**
Char. 17: 0 → 1
Char. 42: 0 → 1
Char. 264: 0 → 1

**Node 76:**
Char. 33: 1 → 2
Char. 41: 0 → 1

Char. 48: 1 → 0
Char. 61: 1 → 2
Char. 68: 0 → 1
Char. 112: 1 → 0
Char. 128: 0 → 1

Char. 138: 0 → 1
Char. 146: 1 → 0
Char. 155: 0 → 1
Char. 158: 0 → 1
Char. 192: 1 → 2

Char. 226: 0 → 1
Char. 227: 0 → 1
Char. 233: 0 → 1
Char. 235: 1 → 2
Char. 267: 1 → 2

**Node 77:**
Char. 61: 1 → 3
Char. 90: 0 → 1
Char. 91: 0 → 1
Char. 109: 1 → 0

Char. 155: 0 → 1
Char. 209: 1 → 0

**ANALYSIS 25**
**(NO *PROGANOCHELYS QUENSTEDTI*, *CANDELARIA BARBOURI*, *ODONTOCHELYS SEMITESTACEA*, AND *EUNOTOSAURUS AFRICANUS*)**

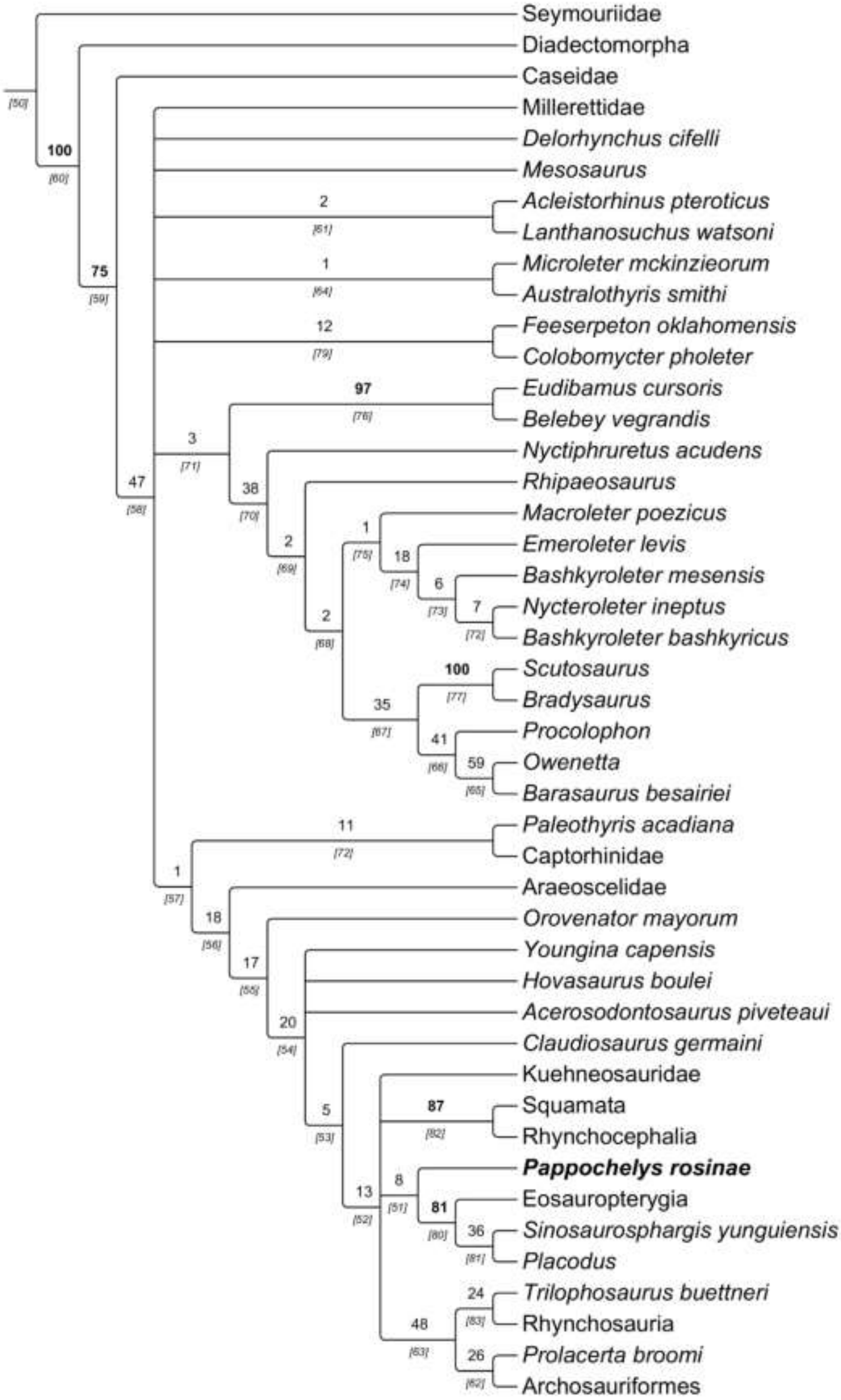

**Seymouriidae:**
Char. 23: 0 → 1
Char. 51: 0 → 2
Char. 54: 1 → 0
Char. 71: 1 → 0
Char. 83: 0 → 1
Char. 85: 1 → 0
Char. 99: 1 → 0
Char. 107: 0 → 1
Char. 140: 0 → 2
Char. 154: 1 → 2
Char. 225: 1 → 0
Char. 260: 2 → 0

***Pappochelys rosinae*:**
Char. 0: 1 → 0
Char. 5: 1 → 0
Char. 12: 0 → 1
Char. 43: 1 → 0
Char. 49: 0 → 1
Char. 61: 1 → 0
Char. 62: 1 → 0
Char. 65: 0 → 1
Char. 67: 1 → 0
Char. 91: 0 → 1
Char. 169: 1 → 0
Char. 201: 1 → 0
Char. 214: 1 → 0
Char. 219: 1 → 0
Char. 241: 0 → 1
Char. 246: 0 → 1
Char. 247: 0 → 1
Char. 252: 0 → 1
Char. 254: 0 → 1
Char. 255: 0 → 1
Char. 256: 0 → 1
Char. 260: 1 → 0
Char. 268: 0 → 1
Char. 269: 0 → 1

***Acerosodontosaurus piveteaui*:**
Char. 78: 1 → 0
Char. 81: 1 → 0
Char. 128: 0 → 1
Char. 155: 0 → 1
Char. 206: 0 → 2
Char. 208: 0 → 1

***Acleistorhinus pteroticus*:**
Char. 21: 0 → 1
Char. 119: 0 → 1
Char. 146: 1 → 0
Char. 169: 1 → 0
Char. 170: 0 → 1

**Araeoscelidae:**
Char. 28: 0 → 1
Char. 106: 0 → 1
Char. 239: 0 → 1

**Archosauriformes:**
Char. 32: 0 → 1
Char. 94: 1 → 0
Char. 112: 1 → 0
Char. 152: 0 → 1
Char. 154: 0 → 2
Char. 166: 1 → 0
Char. 171: 0 → 1
Char. 185: 0 → 1
Char. 204: 0 → 1
Char. 218: 0 → 3
Char. 242: 0 → 1

***Australothyris smithi*:**
Char. 23: 0 → 1
Char. 34: 0 → 1
Char. 55: 1 → 0
Char. 71: 1 → 0
Char. 85: 1 → 0
Char. 112: 0 → 1
Char. 129: 0 → 1
Char. 131: 0 → 1

***Barasaurus besairiei*:**
Char. 33: 1 → 0
Char. 75: 1 → 0
Char. 216: 0 → 1

***Bashkyroleter bashkyricus*:**
Char. 275: 1 → 0

***Bashkyroleter mesensis*:**
Char. 169: 1 → 0

***Belebey vegrandis*:**
Char. 154: 1 → 0

***Bradysaurus* spp.:**
Char. 19: 0 → 1
Char. 73: 0 → 1
Char. 79: 0 → 1
Char. 135: 1 → 0
Char. 249: 0 → 1

**Captorhinidae:**
Char. 3: 0 → 1
Char. 25: 1 → 0
Char. 26: 0 → 1
Char. 73: 0 → 1
Char. 75: 0 → 1
Char. 83: 0 → 1
Char. 108: 1 → 0
Char. 183: 1 → 0
Char. 201: 1 → 0
Char. 203: 1 → 2
Char. 216: 1 → 0

**Caseidae:**
Char. 24: 0 → 1
Char. 25: 1 → 0
Char. 36: 0 → 1
Char. 38: 1 → 0
Char. 46: 1 → 0
Char. 50: 1 → 0
Char. 56: 0 → 1
Char. 85: 1 → 0
Char. 96: 1 → 0
Char. 98: 1 → 0
Char. 170: 0 → 1
Char. 194: 0 → 1
Char. 273: 0 → 1
Char. 274: 0 → 1
Char. 278: 3 → 2

***Claudiosaurus germaini*:**
Char. 24: 0 → 1
Char. 34: 0 → 1
Char. 36: 0 → 1
Char. 43: 1 → 0
Char. 64: 0 → 1
Char. 105: 0 → 1
Char. 130: 0 → 1
Char. 144: 1 → 0
Char. 187: 1 → 0
Char. 199: 0 → 1
Char. 203: 1 → 2
Char. 204: 0 → 1
Char. 220: 0 → 1
Char. 222: 1 → 0

***Colobomycter pholeter*:**
Char. 21: 0 → 1
Char. 25: 1 → 0
Char. 84: 1 → 0
Char. 154: 1 → 0
Char. 167: 0 → 1
Char. 267: 0 → 1

***Delorhynchus cifelli*:**
Char. 18: 0 → 1
Char. 20: 0 → 1
Char. 21: 0 → 1
Char. 24: 0 → 1
Char. 26: 0 → 1
Char. 28: 0 → 1
Char. 33: 0 → 2
Char. 39: 0 → 1
Char. 52: 0 → 1
Char. 100: 0 → 1
Char. 111: 0 → 1
Char. 116: 1 → 0
Char. 117: 0 → 1
Char. 131: 0 → 1
Char. 156: 1 → 0
Char. 167: 0 → 1
Char. 180: 0 → 1
Char. 189: 0 → 1
Char. 191: 0 → 1
Char. 192: 0 → 1
Char. 267: 0 → 1

**Diadectomorpha:**
Char. 0: 0 → 1
Char. 70: 0 → 1
Char. 75: 0 → 1
Char. 122: 0 → 1
Char. 123: 1 → 0
Char. 146: 1 → 0
Char. 275: 1 → 0
Char. 278: 3 → 0

***Emeroleter levis*:**
Char. 51: 1 → 0

**Eosauropterygia:**
Char. 159: 1 → 0
Char. 166: 1 → 0
Char. 174: 0 → 1
Char. 178: 0 → 1
Char. 272: 1 → 0

***Eudibamus cursoris*:**
Char. 154: 1 → 2

***Feeserpeton oklahomensis*:**
Char. 51: 0 → 1
Char. 70: 0 → 1
Char. 157: 0 → 1
Char. 158: 0 → 1

***Hovasaurus boulei*:**
Char. 41: 0 → 1
Char. 55: 1 → 0
Char. 60: 1 → 0
Char. 72: 2 → 1
Char. 77: 0 → 2
Char. 78: 1 → 0
Char. 79: 0 → 1
Char. 93: 1 → 0
Char. 113: 0 → 1
Char. 138: 0 → 1
Char. 146: 1 → 0
Char. 154: 0 → 1
Char. 204: 0 → 2
Char. 206: 0 → 2
Char. 220: 0 → 1
Char. 278: 01 → 3

**Kuehneosauridae:**
Char. 7: 0 → 1
Char. 24: 0 → 1
Char. 26: 0 → 1
Char. 27: 0 → 1
Char. 34: 0 → 1
Char. 36: 0 → 1
Char. 43: 1 → 0
Char. 79: 0 → 2
Char. 82: 0 → 1
Char. 98: 1 → 0
Char. 107: 1 → 0
Char. 108: 1 → 0
Char. 113: 0 → 1
Char. 128: 0 → 1
Char. 140: 1 → 0
Char. 147: 1 → 0
Char. 148: 1 → 0
Char. 159: 1 → 0
Char. 181: 0 → 1
Char. 185: 0 → 1
Char. 206: 0 → 2
Char. 245: 0 → 1
Char. 272: 1 → 2
Char. 278: 0 → 3

***Lanthanosuchus watsoni*:**
Char. 25: 1 → 0
Char. 51: 0 → 1
Char. 76: 0 → 1
Char. 86: 0 → 1
Char. 98: 1 → 0
Char. 138: 0 → 1
Char. 144: 1 → 0
Char. 154: 1 → 2

***Macroleter poezicus*:**
Char. 52: 0 → 1
Char. 84: 1 → 0
Char. 87: 0 → 1
Char. 140: 0 → 1
Char. 146: 1 → 0
Char. 169: 1 → 0
Char. 235: 0 → 1

***Mesosaurus* spp.:**
Char. 0: 0 → 1
Char. 2: 0 → 1
Char. 6: 0 → 1
Char. 8: 0 → 1
Char. 9: 0 → 1
Char. 13: 0 → 1
Char. 19: 0 → 1
Char. 23: 0 → 1
Char. 26: 0 → 1
Char. 29: 1 → 0
Char. 33: 0 → 1
Char. 38: 1 → 0
Char. 41: 0 → 1
Char. 50: 1 → 0
Char. 67: 0 → 1
Char. 76: 0 → 1
Char. 83: 0 → 1
Char. 84: 1 → 0
Char. 85: 1 → 0
Char. 94: 0 → 1
Char. 107: 0 → 1
Char. 109: 0 → 1
Char. 111: 0 → 1
Char. 115: 0 → 1
Char. 146: 1 → 0
Char. 148: 0 → 1
Char. 149: 1 → 0
Char. 164: 0 → 1
Char. 167: 0 → 1
Char. 176: 0 → 1
Char. 183: 1 → 0
Char. 184: 0 → 1
Char. 199: 0 → 1
Char. 202: 0 → 1
Char. 206: 0 → 1
Char. 207: 0 → 1
Char. 209: 0 → 1
Char. 217: 0 → 1
Char. 219: 0 → 1
Char. 220: 0 → 1
Char. 231: 0 → 1
Char. 260: 2 → 0
Char. 272: 2 → 0
Char. 278: 3 → 0

***Microleter mckinzieorum*:**
Char. 0: 0 → 1
Char. 25: 1 → 0
Char. 36: 0 → 1

Char. 39: 0 → 1
Char. 51: 0 → 2
Char. 56: 0 → 1
Char. 70: 0 → 1
Char. 94: 0 → 1
Char. 106: 0 → 1
Char. 276: 0 → 1

**Millerettidae:**
Char. 24: 0 → 1
Char. 25: 1 → 0
Char. 44: 1 → 0
Char. 56: 0 → 1
Char. 57: 1 → 0
Char. 66: 0 → 2
Char. 78: 1 → 0
Char. 80: 1 → 0
Char. 84: 1 → 2
Char. 88: 1 → 0
Char. 96: 1 → 0
Char. 117: 0 → 1
Char. 119: 0 → 1
Char. 124: 0 → 1
Char. 127: 0 → 1
Char. 135: 0 → 1
Char. 145: 0 → 1
Char. 202: 0 → 1
Char. 211: 0 → 1
Char. 230: 0 → 1
Char. 234: 0 → 1
Char. 248: 0 → 1
Char. 252: 0 → 1
Char. 253: 0 → 1

***Nycteroleter ineptus*:**
Char. 278: 0 → 3

***Nyctiphruretus acudens*:**
Char. 21: 0 → 1
Char. 33: 1 → 2
Char. 41: 0 → 1
Char. 81: 1 → 0
Char. 84: 1 → 2
Char. 94: 0 → 1
Char. 167: 0 → 1
Char. 224: 0 → 1
Char. 266: 0 → 1
Char. 272: 2 → 1
Char. 276: 0 → 1

***Orovenator mayorum*:**
Char. 8: 0 → 1
Char. 24: 0 → 1
Char. 36: 0 → 1
Char. 160: 0 → 1
Char. 165: 0 → 1

***Owenetta* spp.:**
Char. 169: 1 → 0

***Paleothyris acadiana*:**
Char. 66: 1 → 2
Char. 102: 0 → 1
Char. 146: 1 → 0
Char. 239: 0 → 1

***Placodus* spp.:**
Char. 0: 1 → 2
Char. 9: 0 → 1
Char. 12: 0 → 1
Char. 13: 0 → 1
Char. 19: 0 → 1
Char. 26: 0 → 1
Char. 31: 0 → 1
Char. 46: 1 → 0
Char. 57: 0 → 1
Char. 93: 1 → 0
Char. 109: 1 → 0
Char. 140: 1 → 0
Char. 155: 0 → 1
Char. 163: 1 → 0
Char. 164: 0 → 1

***Procolophon* spp.:**
Char. 41: 0 → 1
Char. 69: 0 → 1
Char. 79: 0 → 1
Char. 88: 0 → 1
Char. 117: 1 → 0
Char. 149: 1 → 0
Char. 180: 0 → 1
Char. 237: 0 → 1
Char. 238: 0 → 1
Char. 272: 2 → 1
Char. 278: 3 → 0

***Prolacerta broomi*:**
Char. 58: 1 → 0
Char. 66: 1 → 0
Char. 67: 1 → 0
Char. 80: 1 → 0
Char. 123: 0 → 1
Char. 139: 1 → 0
Char. 147: 1 → 0
Char. 192: 1 → 0
Char. 203: 1 → 2
Char. 206: 0 → 12

***Rhipaeosaurus* spp.:**
Char. 172: 0 → 1
Char. 277: 0 → 1

**Rhynchocephalia:**
Char. 0: 1 → 2
Char. 23: 1 → 0
Char. 24: 0 → 1
Char. 77: 0 → 1
Char. 88: 1 → 0
Char. 94: 1 → 0
Char. 104: 0 → 1
Char. 117: 12 → 0
Char. 139: 1 → 0
Char. 167: 1 → 0
Char. 205: 1 → 0

**Rhynchosauria:**
Char. 0: 1 → 0
Char. 7: 0 → 1
Char. 9: 0 → 1
Char. 26: 0 → 1
Char. 63: 1 → 0
Char. 68: 0 → 1
Char. 99: 1 → 0
Char. 123: 0 → 1
Char. 150: 1 → 0
Char. 160: 0 → 1
Char. 161: 0 → 1
Char. 171: 0 → 2
Char. 182: 1 → 0
Char. 241: 0 → 1

***Scutosaurus* spp.:**
Char. 175: 0 → 1
Char. 218: 0 → 1
Char. 243: 0 → 2
Char. 244: 0 → 1
Char. 251: 0 → 1

***Sinosaurosphargis yunguiensis*:**
Char. 8: 0 → 1
Char. 30: 0 → 1
Char. 53: 0 → 1
Char. 82: 0 → 1
Char. 89: 1 → 0
Char. 127: 1 → 0
Char. 150: 1 → 0
Char. 154: 0 → 2
Char. 167: 1 → 0
Char. 253: 0 → 1
Char. 255: 0 → 1

**Squamata:**
Char. 26: 0 → 1
Char. 45: 0 → 1
Char. 79: 0 → 2
Char. 80: 1 → 0
Char. 82: 0 → 1
Char. 92: 1 → 0
Char. 109: 1 → 0
Char. 122: 0 → 1
Char. 160: 0 → 1
Char. 200: 0 → 1
Char. 245: 0 → 1

***Trilophosaurus buettneri*:**
Char. 5: 1 → 0
Char. 11: 0 → 1
Char. 55: 1 → 0
Char. 93: 1 → 0
Char. 104: 0 → 1
Char. 113: 0 → 1
Char. 122: 0 → 1
Char. 136: 1 → 0
Char. 144: 1 → 0
Char. 154: 0 → 1
Char. 157: 0 → 1
Char. 159: 1 → 0
Char. 177: 0 → 12
Char. 194: 0 → 1
Char. 203: 1 → 2
Char. 207: 1 → 0
Char. 208: 1 → 0
Char. 272: 1 → 0

***Youngina capensis*:**
Char. 75: 0 → 1
Char. 92: 1 → 0
Char. 170: 0 → 1
Char. 211: 0 → 1
Char. 231: 0 → 1
Char. 239: 0 → 1

**Node 50:**
Char. 0: 2 → 0
Char. 5: 1 → 0
Char. 15: 1 → 0
Char. 20: 1 → 0
Char. 25: 0 → 1
Char. 33: 2 → 0
Char. 73: 1 → 0
Char. 79: 2 → 1
Char. 94: 1 → 0
Char. 119: 1 → 0
Char. 127: 1 → 0
Char. 130: 1 → 0
Char. 134: 2 → 0
Char. 136: 1 → 0
Char. 141: 1 → 0
Char. 145: 1 → 0
Char. 147: 1 → 0
Char. 149: 1 → 0
Char. 151: 1 → 0
Char. 152: 1 → 0
Char. 155: 1 → 0
Char. 158: 1 → 0
Char. 161: 1 → 0
Char. 163: 1 → 0
Char. 166: 1 → 0
Char. 174: 1 → 0
Char. 180: 1 → 0
Char. 181: 1 → 0
Char. 187: 1 → 0
Char. 188: 1 → 0
Char. 189: 1 → 0
Char. 194: 2 → 0
Char. 196: 1 → 0
Char. 203: 2 → 0
Char. 204: 2 → 0
Char. 208: 1 → 0
Char. 217: 1 → 0
Char. 220: 1 → 0
Char. 221: 1 → 0
Char. 226: 1 → 0
Char. 227: 1 → 0
Char. 231: 1 → 0
Char. 234: 1 → 0
Char. 247: 1 → 0
Char. 251: 1 → 0
Char. 253: 2 → 0

**Node 51:**
Char. 1: 0 → 1
Char. 55: 1 → 0
Char. 193: 1 → 0
Char. 206: 0 → 2
Char. 220: 0 → 1
Char. 239: 1 → 0

**Node 52:**
Char. 27: 1 → 0
Char. 58: 0 → 1
Char. 61: 0 → 1
Char. 66: 0 → 1
Char. 69: 0 → 1
Char. 84: 0 → 1
Char. 107: 0 → 1
Char. 123: 1 → 0
Char. 140: 0 → 1
Char. 147: 0 → 1
Char. 150: 0 → 1
Char. 167: 0 → 1
Char. 184: 0 → 1
Char. 205: 0 → 1
Char. 208: 0 → 1
Char. 210: 0 → 1
Char. 230: 0 → 1
Char. 239: 0 → 1

**Node 53:**
Char. 70: 0 → 1
Char. 73: 0 → 1
Char. 126: 0 → 1
Char. 131: 0 → 1
Char. 148: 0 → 1
Char. 182: 0 → 1
Char. 190: 0 → 1
Char. 272: 2 → 1

**Node 54:**
Char. 33: 0 → 1
Char. 92: 0 → 1
Char. 135: 0 → 1
Char. 159: 0 → 1

**Node 55:**
Char. 20: 0 → 1
Char. 62: 0 → 1

**Node 56:**
Char. 0: 0 → 1
Char. 57: 1 → 0
Char. 89: 0 → 1
Char. 112: 0 → 1
Char. 120: 0 → 1
Char. 192: 0 → 1
Char. 193: 0 → 1
Char. 222: 0 → 1
Char. 234: 0 → 1
Char. 266: 0 → 1
Char. 278: 3 → 1

**Node 57:**
Char. 29: 1 → 0
Char. 59: 0 → 1
Char. 60: 0 → 1
Char. 67: 0 → 1
Char. 72: 1 → 2
Char. 84: 1 → 0
Char. 111: 0 → 1
Char. 119: 0 → 1
Char. 154: 1 → 0

**Node 58:**
Char. 29: 0 → 1
Char. 40: 0 → 2
Char. 74: 0 → 1
Char. 88: 0 → 1
Char. 116: 0 → 1
Char. 149: 0 → 1
Char. 169: 0 → 1
Char. 201: 0 → 1
Char. 203: 0 → 1
Char. 235: 0 → 1
Char. 276: 1 → 0

**Node 59:**
Char. 79: 1 → 0
Char. 81: 0 → 1
Char. 93: 0 → 1
Char. 97: 0 → 1

**Node 60:**
Char. 55: 0 → 1
Char. 78: 0 → 1
Char. 111: 1 → 0
Char. 135: 1 → 0

**Node 61:**
Char. 95: 0 → 1
Char. 110: 0 → 1
Char. 113: 0 → 1
Char. 114: 0 → 1
Char. 131: 0 → 1
Char. 137: 0 → 1
Char. 140: 0 → 2

**Node 62:**
Char. 19: 0 → 1
Char. 92: 1 → 0

**Node 63:**
Char. 4: 0 → 1
Char. 15: 0 → 1
Char. 29: 0 → 1
Char. 35: 1 → 0
Char. 48: 1 → 0
Char. 210: 1 → 0
Char. 213: 0 → 1
Char. 226: 0 → 2
Char. 228: 0 → 1
Char. 275: 0 → 1

**Node 64:**
Char. 24: 0 → 1
Char. 57: 1 → 0
Char. 79: 0 → 1
Char. 83: 0 → 1
Char. 110: 0 → 1
Char. 132: 1 → 0

**Node 65:**
Char. 73: 0 → 1
Char. 131: 1 → 0
Char. 205: 0 → 1
Char. 276: 0 → 1

**Node 66:**
Char. 18: 0 → 1
Char. 37: 0 → 1
Char. 107: 0 → 1
Char. 118: 1 → 0
Char. 125: 1 → 0
Char. 150: 0 → 1

**Node 67:**
Char. 23: 0 → 1
Char. 71: 1 → 0
Char. 102: 0 → 1
Char. 103: 0 → 1
Char. 106: 0 → 1
Char. 167: 0 → 1
Char. 214: 0 → 1
Char. 216: 1 → 0
Char. 235: 0 → 2
Char. 241: 0 → 1

**Node 68:**
Char. 207: 0 → 1

**Node 69:**
Char. 226: 0 → 1

**Node 70:**
Char. 20: 0 → 1
Char. 33: 0 → 1
Char. 44: 1 → 0
Char. 70: 0 → 1
Char. 80: 1 → 0
Char. 117: 0 → 1
Char. 118: 0 → 1
Char. 119: 0 → 1
Char. 125: 0 → 1
Char. 131: 0 → 1
Char. 194: 0 → 1
Char. 201: 1 → 0
Char. 211: 0 → 1

**Node 71:**
Char. 38: 1 → 2
Char. 39: 0 → 1
Char. 49: 0 → 1
Char. 66: 0 → 12
Char. 76: 0 → 1
Char. 85: 1 → 0
Char. 88: 1 → 0
Char. 95: 0 → 1
Char. 112: 0 → 1
Char. 148: 0 → 1
Char. 158: 0 → 1
Char. 183: 1 → 2
Char. 192: 0 → 1

**Node 72:**
Char. 87: 0 → 1

**Node 73:**
Char. 25: 1 → 0
Char. 76: 1 → 0

**Node 74:**
Char. 79: 0 → 1
Char. 93: 1 → 0
Char. 133: 0 → 1

**Node 75:**
Char. 85: 0 → 1
Char. 147: 1 → 2
Char. 154: 1 → 0
Char. 278: 3 → 0

**Node 76:**
Char. 50: 1 → 0
Char. 58: 0 → 1
Char. 59: 0 → 1
Char. 60: 0 → 1
Char. 72: 1 → 2
Char. 104: 0 → 1
Char. 105: 0 → 1
Char. 106: 0 → 2
Char. 107: 0 → 1
Char. 109: 0 → 1
Char. 110: 0 → 1
Char. 146: 1 → 0
Char. 155: 0 → 2

**Node 77:**
Char. 33: 1 → 0
Char. 38: 2 → 1
Char. 39: 1 → 0
Char. 42: 0 → 1
Char. 43: 0 → 1
Char. 46: 1 → 0
Char. 49: 1 → 0
Char. 52: 0 → 1
Char. 83: 01 → 2
Char. 84: 1 → 0
Char. 87: 0 → 1
Char. 93: 1 → 0
Char. 161: 0 → 1
Char. 163: 1 → 0
Char. 172: 0 → 2
Char. 174: 0 → 1
Char. 188: 0 → 1
Char. 189: 0 → 1
Char. 195: 0 → 1
Char. 212: 0 → 1
Char. 236: 0 → 1
Char. 238: 0 → 2
Char. 242: 0 → 1
Char. 274: 0 → 1
Char. 275: 1 → 0

**Node 78:**
Char. 44: 1 → 0
Char. 66: 0 → 1
Char. 78: 1 → 0
Char. 88: 1 → 0
Char. 93: 1 → 0
Char. 123: 1 → 0

**Node 79:**
Char. 20: 0 → 1
Char. 47: 0 → 1
Char. 83: 0 → 1
Char. 85: 1 → 0
Char. 107: 0 → 1
Char. 110: 0 → 1
Char. 169: 1 → 0
Char. 170: 0 → 1

**Node 80:**
Char. 2: 0 → 1
Char. 6: 0 → 1
Char. 198: 0 → 1
Char. 240: 1 → 0
Char. 266: 1 → 0
Char. 267: 1 → 0

**Node 81:**
Char. 17: 0 → 1
Char. 42: 0 → 1
Char. 251: 0 → 1
Char. 264: 0 → 1

**Node 82:**
Char. 33: 1 → 2
Char. 48: 1 → 0
Char. 61: 1 → 2
Char. 68: 0 → 1
Char. 112: 1 → 0
Char. 128: 0 → 1
Char. 138: 0 → 1
Char. 146: 1 → 0
Char. 155: 0 → 1
Char. 158: 0 → 1
Char. 182: 1 → 0
Char. 192: 1 → 2
Char. 226: 0 → 1
Char. 227: 0 → 1
Char. 233: 0 → 1
Char. 235: 1 → 2
Char. 267: 1 → 2

**Node 83:**
Char. 61: 1 → 3
Char. 90: 0 → 1
Char. 91: 0 → 1
Char. 109: 1 → 0
Char. 155: 0 → 1
Char. 209: 1 → 0

**ANALYSIS 26**

**(NO *PROGANOCHELYS QUENSTEDTI*, *PAPPOCHELYS ROSINAE*, *ODONTOCHELYS SEMITESTACEA*, AND *EUNOTOSAURUS AFRICANUS*)**

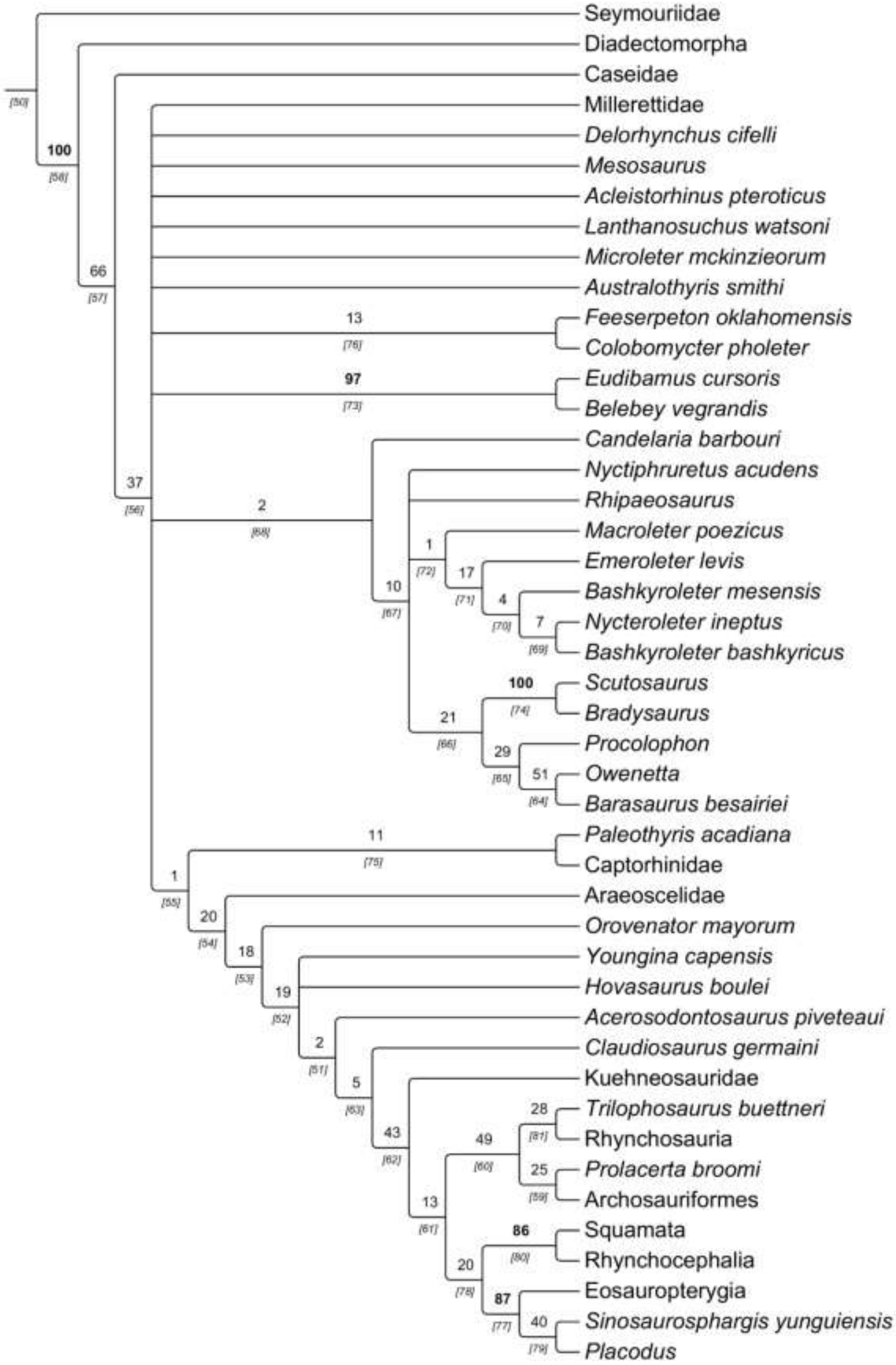

**Seymouriidae:**
Char. 23: 0 → 1
Char. 51: 0 → 2
Char. 54: 1 → 0
Char. 71: 1 → 0
Char. 83: 0 → 1
Char. 85: 1 → 0
Char. 99: 1 → 0
Char. 107: 0 → 1
Char. 140: 0 → 2
Char. 154: 1 → 2
Char. 225: 1 → 0
Char. 260: 2 → 0

***Acerosodontosaurus piveteaui*:**
Char. 78: 1 → 0
Char. 128: 0 → 1
Char. 155: 0 → 1
Char. 206: 0 → 2

***Acleistorhinus pteroticus*:**
Char. 20: 0 → 1
Char. 21: 0 → 1
Char. 30: 0 → 1
Char. 33: 0 → 1
Char. 47: 0 → 1
Char. 48: 0 → 1
Char. 55: 1 → 0
Char. 56: 0 → 1
Char. 64: 0 → 1
Char. 70: 0 → 1
Char. 79: 0 → 1
Char. 95: 0 → 1
Char. 113: 0 → 1
Char. 114: 0 → 1
Char. 119: 0 → 1
Char. 137: 0 → 1
Char. 140: 0 → 2
Char. 146: 1 → 0
Char. 169: 1 → 0
Char. 170: 0 → 1
Char. 278: 3 → 1

**Araeoscelidae:**
Char. 23: 0 → 1
Char. 28: 0 → 1
Char. 106: 0 → 1
Char. 239: 0 → 1

**Archosauriformes:**
Char. 32: 0 → 1
Char. 94: 1 → 0
Char. 112: 1 → 0
Char. 152: 0 → 1
Char. 154: 0 → 2
Char. 166: 1 → 0
Char. 171: 0 → 1
Char. 185: 0 → 1
Char. 204: 0 → 1
Char. 218: 0 → 3
Char. 224: 0 → 1
Char. 242: 0 → 1

***Australothyris smithi*:**
Char. 23: 0 → 1
Char. 24: 0 → 1
Char. 34: 0 → 1
Char. 55: 1 → 0
Char. 57: 1 → 0
Char. 71: 1 → 0
Char. 79: 0 → 1
Char. 85: 1 → 0
Char. 98: 1 → 0
Char. 100: 0 → 1
Char. 103: 0 → 1
Char. 112: 0 → 1
Char. 123: 1 → 0
Char. 129: 0 → 1
Char. 132: 1 → 0
Char. 144: 1 → 0
Char. 149: 1 → 0
Char. 150: 0 → 1

***Barasaurus besairiei*:**
Char. 33: 1 → 0
Char. 75: 1 → 0
Char. 216: 0 → 1

***Bashkyroleter bashkyricus*:**
Char. 275: 1 → 0

***Bashkyroleter mesensis*:**
Char. 169: 1 → 0

***Belebey vegrandis*:**
Char. 154: 1 → 0

***Bradysaurus* spp.:**
Char. 19: 0 → 1
Char. 73: 0 → 1
Char. 79: 0 → 1
Char. 135: 1 → 0
Char. 249: 0 → 1

***Candelaria barbouri*:**
Char. 1: 0 → 1
Char. 5: 1 → 0
Char. 8: 0 → 1
Char. 15: 0 → 1
Char. 23: 0 → 1
Char. 25: 1 → 0
Char. 29: 1 → 0
Char. 50: 1 → 0
Char. 55: 1 → 0
Char. 57: 1 → 0
Char. 67: 0 → 1
Char. 79: 0 → 2
Char. 89: 0 → 1
Char. 127: 0 → 1
Char. 154: 1 → 2
Char. 169: 1 → 0
Char. 277: 0 → 1

**Captorhinidae:**
Char. 3: 0 → 1
Char. 23: 0 → 1
Char. 25: 1 → 0
Char. 26: 0 → 1
Char. 73: 0 → 1
Char. 75: 0 → 1
Char. 108: 1 → 0
Char. 183: 1 → 0
Char. 201: 1 → 0
Char. 203: 1 → 2
Char. 216: 1 → 0

**Caseidae:**
Char. 24: 0 → 1
Char. 25: 1 → 0
Char. 36: 0 → 1
Char. 38: 1 → 0
Char. 46: 1 → 0
Char. 50: 1 → 0
Char. 56: 0 → 1
Char. 85: 1 → 0
Char. 96: 1 → 0
Char. 98: 1 → 0
Char. 170: 0 → 1
Char. 194: 0 → 1
Char. 273: 0 → 1
Char. 274: 0 → 1

***Claudiosaurus germaini*:**
Char. 64: 0 → 1
Char. 105: 0 → 1
Char. 130: 0 → 1
Char. 144: 1 → 0
Char. 187: 1 → 0
Char. 199: 0 → 1
Char. 203: 1 → 2
Char. 204: 0 → 1
Char. 220: 0 → 1
Char. 222: 1 → 0

***Colobomycter pholeter*:**
Char. 21: 0 → 1
Char. 25: 1 → 0
Char. 84: 1 → 0
Char. 154: 1 → 0
Char. 167: 0 → 1
Char. 267: 0 → 1

***Delorhynchus cifelli*:**
Char. 18: 0 → 1
Char. 20: 0 → 1
Char. 21: 0 → 1
Char. 24: 0 → 1
Char. 26: 0 → 1
Char. 28: 0 → 1
Char. 33: 0 → 2
Char. 39: 0 → 1
Char. 48: 0 → 1
Char. 52: 0 → 1
Char. 100: 0 → 1
Char. 111: 0 → 1
Char. 116: 1 → 0
Char. 117: 0 → 1
Char. 156: 1 → 0
Char. 167: 0 → 1
Char. 180: 0 → 1
Char. 189: 0 → 1
Char. 191: 0 → 1
Char. 267: 0 → 1

**Diadectomorpha:**
Char. 0: 0 → 1
Char. 70: 0 → 1
Char. 75: 0 → 1
Char. 122: 0 → 1
Char. 123: 1 → 0
Char. 146: 1 → 0
Char. 275: 1 → 0

***Emeroleter levis*:**
Char. 0: 0 → 2
Char. 51: 1 → 0

**Eosauropterygia:**
Char. 159: 1 → 0
Char. 166: 1 → 0
Char. 174: 0 → 1
Char. 194: 0 → 2
Char. 272: 1 → 0

***Eudibamus cursoris*:**
Char. 154: 1 → 2

***Feeserpeton oklahomensis*:**
Char. 51: 0 → 1
Char. 70: 0 → 1
Char. 157: 0 → 1
Char. 158: 0 → 1

***Hovasaurus boulei*:**
Char. 41: 0 → 1
Char. 55: 1 → 0
Char. 60: 1 → 0
Char. 72: 2 → 1
Char. 77: 0 → 2
Char. 78: 1 → 0
Char. 79: 0 → 1
Char. 93: 1 → 0
Char. 113: 0 → 1
Char. 138: 0 → 1
Char. 146: 1 → 0
Char. 154: 0 → 1
Char. 204: 0 → 2
Char. 206: 0 → 2
Char. 220: 0 → 1
Char. 278: 01 → 3

**Kuehneosauridae:**
Char. 7: 0 → 1
Char. 26: 0 → 1
Char. 44: 1 → 0
Char. 79: 0 → 2
Char. 82: 0 → 1
Char. 98: 1 → 0
Char. 108: 1 → 0
Char. 113: 0 → 1
Char. 128: 0 → 1
Char. 159: 1 → 0
Char. 181: 0 → 1
Char. 185: 0 → 1
Char. 206: 0 → 2
Char. 245: 0 → 1
Char. 278: 0 → 3

***Lanthanosuchus watsoni*:**
Char. 25: 1 → 0
Char. 51: 0 → 1
Char. 86: 0 → 1
Char. 95: 0 → 1
Char. 98: 1 → 0
Char. 113: 0 → 1
Char. 114: 0 → 1
Char. 137: 0 → 1
Char. 138: 0 → 1
Char. 140: 0 → 2
Char. 144: 1 → 0
Char. 154: 1 → 2

***Macroleter poezicus*:**
Char. 0: 0 → 1
Char. 52: 0 → 1
Char. 66: 1 → 2
Char. 75: 0 → 1
Char. 84: 1 → 0
Char. 87: 0 → 1
Char. 140: 0 → 1
Char. 146: 1 → 0
Char. 169: 1 → 0
Char. 235: 0 → 1

***Mesosaurus* spp.:**
Char. 0: 0 → 1
Char. 2: 0 → 1
Char. 5: 1 → 0
Char. 6: 0 → 1
Char. 8: 0 → 1
Char. 9: 0 → 1
Char. 13: 0 → 1
Char. 19: 0 → 1
Char. 23: 0 → 1
Char. 26: 0 → 1
Char. 29: 1 → 0
Char. 33: 0 → 1
Char. 38: 1 → 0
Char. 41: 0 → 1
Char. 48: 0 → 1
Char. 50: 1 → 0
Char. 67: 0 → 1
Char. 84: 1 → 0
Char. 85: 1 → 0
Char. 94: 0 → 1
Char. 107: 0 → 1
Char. 109: 0 → 1
Char. 111: 0 → 1
Char. 115: 0 → 1
Char. 146: 1 → 0
Char. 147: 1 → 0
Char. 148: 0 → 1
Char. 149: 1 → 0
Char. 164: 0 → 1
Char. 166: 1 → 0
Char. 167: 0 → 1
Char. 176: 0 → 1
Char. 183: 1 → 0
Char. 184: 0 → 1

Char. 199: 0 → 1
Char. 202: 0 → 1
Char. 206: 0 → 1
Char. 207: 0 → 1
Char. 209: 0 → 1
Char. 217: 0 → 1
Char. 219: 0 → 1
Char. 220: 0 → 1
Char. 231: 0 → 1
Char. 260: 2 → 0
Char. 272: 2 → 0
Char. 278: 3 → 0

***Microleter mckinzieorum*:**
Char. 0: 0 → 1
Char. 18: 0 → 1
Char. 24: 0 → 1
Char. 25: 1 → 0
Char. 36: 0 → 1
Char. 39: 0 → 1
Char. 51: 0 → 2
Char. 56: 0 → 1
Char. 57: 1 → 0
Char. 70: 0 → 1
Char. 79: 0 → 1
Char. 94: 0 → 1
Char. 106: 0 → 1
Char. 132: 1 → 0
Char. 166: 1 → 0
Char. 276: 0 → 1
Char. 278: 3 → 1

**Millerettidae:**
Char. 5: 1 → 0
Char. 24: 0 → 1
Char. 25: 1 → 0
Char. 44: 1 → 0
Char. 56: 0 → 1
Char. 57: 1 → 0
Char. 66: 0 → 2
Char. 78: 1 → 0
Char. 80: 1 → 0
Char. 84: 1 → 2
Char. 88: 1 → 0
Char. 96: 1 → 0
Char. 117: 0 → 1
Char. 119: 0 → 1
Char. 124: 0 → 1
Char. 127: 0 → 1
Char. 135: 0 → 1
Char. 145: 0 → 1
Char. 147: 1 → 0
Char. 166: 1 → 0
Char. 202: 0 → 1
Char. 211: 0 → 1
Char. 230: 0 → 1
Char. 234: 0 → 1
Char. 248: 0 → 1
Char. 252: 0 → 1
Char. 253: 0 → 1

***Nycteroleter ineptus*:**
Char. 278: 0 → 3

***Nyctiphruretus acudens*:**
Char. 0: 0 → 1
Char. 21: 0 → 1
Char. 41: 0 → 1
Char. 66: 1 → 2
Char. 81: 1 → 0
Char. 84: 1 → 2
Char. 166: 1 → 0
Char. 167: 0 → 1
Char. 215: 1 → 0
Char. 224: 0 → 1
Char. 226: 1 → 0
Char. 266: 0 → 1
Char. 272: 2 → 1

***Orovenator mayorum*:**
Char. 8: 0 → 1
Char. 24: 0 → 1
Char. 36: 0 → 1
Char. 160: 0 → 1
Char. 165: 0 → 1

***Owenetta* spp.:**
Char. 169: 1 → 0

***Paleothyris acadiana*:**
Char. 66: 1 → 2
Char. 102: 0 → 1
Char. 146: 1 → 0
Char. 239: 0 → 1

***Placodus* spp.:**
Char. 0: 1 → 2
Char. 9: 0 → 1
Char. 12: 0 → 1
Char. 13: 0 → 1
Char. 19: 0 → 1
Char. 26: 0 → 1
Char. 31: 0 → 1
Char. 44: 1 → 0
Char. 46: 1 → 0
Char. 57: 0 → 1
Char. 78: 1 → 0
Char. 93: 1 → 0
Char. 102: 1 → 2
Char. 109: 1 → 0
Char. 140: 1 → 0
Char. 155: 0 → 1
Char. 163: 1 → 0
Char. 164: 0 → 1

***Procolophon* spp.:**
Char. 41: 0 → 1
Char. 69: 0 → 1
Char. 79: 0 → 1
Char. 88: 0 → 1
Char. 117: 1 → 0
Char. 149: 1 → 0
Char. 180: 0 → 1
Char. 237: 0 → 1
Char. 238: 0 → 1
Char. 272: 2 → 1
Char. 278: 3 → 0

***Prolacerta broomi*:**
Char. 58: 1 → 0
Char. 66: 1 → 0
Char. 67: 1 → 0
Char. 80: 1 → 0
Char. 139: 1 → 0
Char. 147: 1 → 0
Char. 192: 1 → 0
Char. 203: 1 → 2
Char. 206: 0 → 12

***Rhipaeosaurus* spp.:**
Char. 172: 0 → 1
Char. 186: 1 → 0
Char. 277: 0 → 1

**Rhynchocephalia:**
Char. 0: 1 → 2
Char. 24: 0 → 1
Char. 75: 1 → 0
Char. 77: 0 → 1
Char. 94: 1 → 0
Char. 117: 12 → 0
Char. 139: 1 → 0
Char. 167: 1 → 0
Char. 205: 1 → 0

**Rhynchosauria:**
Char. 0: 1 → 0
Char. 7: 0 → 1
Char. 9: 0 → 1
Char. 26: 0 → 1
Char. 44: 1 → 0
Char. 68: 0 → 1
Char. 99: 1 → 0
Char. 150: 1 → 0
Char. 160: 0 → 1
Char. 161: 0 → 1
Char. 171: 0 → 2
Char. 182: 1 → 0
Char. 223: 0 → 1
Char. 224: 0 → 1
Char. 241: 0 → 1

***Scutosaurus* spp.:**
Char. 175: 0 → 1
Char. 218: 0 → 1
Char. 243: 0 → 2
Char. 244: 0 → 1
Char. 251: 0 → 1

***Sinosaurosphargis yunguiensis*:**
Char. 8: 0 → 1
Char. 30: 0 → 1
Char. 53: 0 → 1
Char. 82: 0 → 1
Char. 89: 1 → 0
Char. 127: 1 → 0
Char. 150: 1 → 0
Char. 154: 0 → 2
Char. 167: 1 → 0
Char. 253: 0 → 1
Char. 255: 0 → 1

**Squamata:**
Char. 26: 0 → 1
Char. 45: 0 → 1
Char. 79: 0 → 2
Char. 80: 1 → 0
Char. 82: 0 → 1
Char. 92: 1 → 0
Char. 109: 1 → 0
Char. 160: 0 → 1
Char. 245: 0 → 1

***Trilophosaurus buettneri*:**
Char. 5: 1 → 0
Char. 11: 0 → 1
Char. 55: 1 → 0
Char. 93: 1 → 0
Char. 104: 0 → 1
Char. 113: 0 → 1
Char. 122: 0 → 1
Char. 136: 1 → 0
Char. 144: 1 → 0
Char. 154: 0 → 1
Char. 157: 0 → 1
Char. 159: 1 → 0
Char. 177: 0 → 12
Char. 194: 0 → 1
Char. 203: 1 → 2
Char. 207: 1 → 0
Char. 208: 1 → 0
Char. 272: 1 → 0

***Youngina capensis*:**
Char. 21: 0 → 1
Char. 44: 1 → 0
Char. 75: 0 → 1
Char. 211: 0 → 1
Char. 231: 0 → 1
Char. 239: 0 → 1

**Node 50:**
Char. 0: 2 → 0
Char. 5: 1 → 0
Char. 15: 1 → 0
Char. 20: 1 → 0
Char. 21: 1 → 0
Char. 25: 0 → 1
Char. 33: 2 → 0
Char. 65: 1 → 0
Char. 72: 2 → 0
Char. 73: 1 → 0
Char. 84: 2 → 01
Char. 94: 1 → 0
Char. 119: 1 → 0
Char. 127: 1 → 0
Char. 130: 1 → 0
Char. 134: 2 → 0
Char. 136: 1 → 0
Char. 141: 1 → 0
Char. 145: 1 → 0
Char. 147: 1 → 0
Char. 149: 1 → 0
Char. 151: 1 → 0
Char. 152: 1 → 0
Char. 155: 1 → 0
Char. 158: 1 → 0
Char. 161: 1 → 0
Char. 163: 1 → 0
Char. 166: 1 → 0
Char. 174: 1 → 0
Char. 180: 1 → 0
Char. 181: 1 → 0
Char. 184: 1 → 0
Char. 187: 1 → 0
Char. 188: 1 → 0
Char. 189: 1 → 0
Char. 192: 1 → 0
Char. 194: 2 → 0
Char. 196: 1 → 0
Char. 203: 2 → 0
Char. 204: 2 → 0
Char. 205: 1 → 0
Char. 208: 1 → 0
Char. 210: 1 → 0
Char. 217: 1 → 0
Char. 220: 1 → 0
Char. 221: 1 → 0
Char. 222: 1 → 0
Char. 226: 1 → 0
Char. 227: 1 → 0
Char. 231: 1 → 0
Char. 234: 1 → 0
Char. 241: 1 → 0
Char. 246: 1 → 0
Char. 247: 1 → 0
Char. 251: 1 → 0
Char. 252: 1 → 0
Char. 253: 2 → 0
Char. 254: 1 → 0
Char. 255: 1 → 0
Char. 256: 1 → 0
Char. 266: 1 → 0
Char. 267: 1 → 0
Char. 268: 1 → 0
Char. 269: 1 → 0

**Node 51:**
Char. 23: 0 → 1
Char. 94: 0 → 1
Char. 224: 1 → 0

**Node 52:**
Char. 33: 0 → 1
Char. 135: 0 → 1
Char. 159: 0 → 1

**Node 53:**
Char. 20: 0 → 1
Char. 62: 0 → 1

**Node 54:**
Char. 0: 0 → 1
Char. 57: 1 → 0
Char. 89: 0 → 1
Char. 112: 0 → 1
Char. 120: 0 → 1
Char. 193: 0 → 1
Char. 222: 0 → 1
Char. 224: 0 → 1

Char. 234: 0 → 1
Char. 266: 0 → 1
Char. 278: 3 → 1

**Node 55:**
Char. 29: 1 → 0
Char. 59: 0 → 1
Char. 60: 0 → 1
Char. 67: 0 → 1
Char. 72: 1 → 2
Char. 84: 1 → 0
Char. 111: 0 → 1
Char. 119: 0 → 1
Char. 147: 1 → 0
Char. 154: 1 → 0

**Node 56:**
Char. 5: 0 → 1
Char. 29: 0 → 1
Char. 40: 0 → 2
Char. 74: 0 → 1
Char. 88: 0 → 1
Char. 116: 0 → 1
Char. 147: 0 → 1
Char. 149: 0 → 1
Char. 166: 0 → 1
Char. 169: 0 → 1
Char. 201: 0 → 1
Char. 203: 0 → 1
Char. 235: 0 → 1

**Node 57:**
Char. 72: 0 → 1
Char. 81: 0 → 1
Char. 93: 0 → 1
Char. 97: 0 → 1

**Node 58:**
Char. 55: 0 → 1
Char. 78: 0 → 1
Char. 111: 1 → 0
Char. 135: 1 → 0

**Node 59:**
Char. 19: 0 → 1
Char. 92: 1 → 0

**Node 60:**
Char. 4: 0 → 1
Char. 15: 0 → 1
Char. 29: 0 → 1
Char. 213: 0 → 1
Char. 226: 0 → 2
Char. 228: 0 → 1
Char. 275: 0 → 1

**Node 61:**
Char. 27: 1 → 0
Char. 75: 0 → 1
Char. 107: 0 → 1
Char. 140: 0 → 1
Char. 147: 0 → 1

**Node 62:**
Char. 58: 0 → 1
Char. 61: 0 → 1
Char. 66: 0 → 1
Char. 69: 0 → 1
Char. 150: 0 → 1
Char. 167: 0 → 1
Char. 205: 0 → 1
Char. 239: 0 → 1

**Node 63:**
Char. 126: 0 → 1
Char. 190: 0 → 1

**Node 64:**
Char. 73: 0 → 1
Char. 131: 1 → 0
Char. 205: 0 → 1
Char. 239: 0 → 1

**Node 65:**
Char. 18: 0 → 1
Char. 37: 0 → 1
Char. 48: 0 → 1
Char. 107: 0 → 1
Char. 118: 1 → 0
Char. 125: 1 → 0
Char. 150: 0 → 1

**Node 66:**
Char. 23: 0 → 1
Char. 71: 1 → 0
Char. 75: 0 → 1
Char. 102: 0 → 1
Char. 103: 0 → 1
Char. 106: 0 → 1
Char. 155: 0 → 1
Char. 167: 0 → 1
Char. 207: 0 → 1
Char. 214: 0 → 1
Char. 216: 1 → 0
Char. 235: 0 → 2
Char. 241: 0 → 1

**Node 67:**
Char. 66: 0 → 1

**Node 68:**
Char. 20: 0 → 1
Char. 33: 0 → 12
Char. 49: 0 → 1
Char. 88: 1 → 0
Char. 95: 0 → 1

**Node 69:**
Char. 87: 0 → 1

**Node 70:**
Char. 25: 1 → 0
Char. 76: 1 → 0

**Node 71:**
Char. 79: 0 → 1
Char. 86: 0 → 1
Char. 93: 1 → 0
Char. 133: 0 → 1

**Node 72:**
Char. 48: 0 → 1
Char. 100: 0 → 1
Char. 113: 0 → 1
Char. 147: 1 → 2
Char. 154: 1 → 0
Char. 207: 0 → 1
Char. 239: 0 → 1
Char. 278: 3 → 0

**Node 73:**
Char. 38: 1 → 2
Char. 39: 0 → 1
Char. 50: 1 → 0
Char. 58: 0 → 1
Char. 59: 0 → 1
Char. 60: 0 → 1
Char. 72: 1 → 2
Char. 85: 1 → 0
Char. 88: 1 → 0
Char. 95: 0 → 1
Char. 104: 0 → 1
Char. 105: 0 → 1
Char. 106: 0 → 2
Char. 107: 0 → 1
Char. 109: 0 → 1
Char. 146: 1 → 0
Char. 148: 0 → 1
Char. 155: 0 → 2
Char. 183: 1 → 2

**Node 74:**
Char. 0: 0 → 2
Char. 33: 1 → 0
Char. 38: 2 → 1
Char. 39: 1 → 0
Char. 42: 0 → 1
Char. 43: 0 → 1
Char. 46: 1 → 0
Char. 49: 1 → 0
Char. 52: 0 → 1
Char. 83: 01 → 2
Char. 84: 1 → 0
Char. 87: 0 → 1
Char. 93: 1 → 0
Char. 100: 0 → 1
Char. 113: 0 → 1
Char. 143: 0 → 1
Char. 161: 0 → 1
Char. 163: 1 → 0
Char. 172: 0 → 2
Char. 174: 0 → 1
Char. 188: 0 → 1
Char. 189: 0 → 1
Char. 195: 0 → 1
Char. 212: 0 → 1
Char. 236: 0 → 1
Char. 238: 0 → 2
Char. 242: 0 → 1
Char. 245: 0 → 1
Char. 274: 0 → 1
Char. 275: 1 → 0

**Node 75:**
Char. 44: 1 → 0
Char. 66: 0 → 1
Char. 78: 1 → 0
Char. 88: 1 → 0
Char. 93: 1 → 0
Char. 123: 1 → 0

**Node 76:**
Char. 20: 0 → 1
Char. 47: 0 → 1
Char. 85: 1 → 0
Char. 107: 0 → 1
Char. 169: 1 → 0
Char. 170: 0 → 1

**Node 77:**
Char. 2: 0 → 1
Char. 6: 0 → 1
Char. 98: 1 → 0
Char. 101: 0 → 1
Char. 113: 0 → 1
Char. 181: 0 → 1
Char. 186: 0 → 1
Char. 193: 1 → 0
Char. 198: 0 → 1
Char. 206: 0 → 2
Char. 220: 0 → 1
Char. 239: 1 → 0
Char. 240: 1 → 0
Char. 266: 1 → 0

**Node 78:**
Char. 33: 1 → 2
Char. 68: 0 → 1
Char. 102: 0 → 1
Char. 103: 0 → 1
Char. 223: 0 → 1
Char. 229: 0 → 1
Char. 235: 1 → 2

**Node 79:**
Char. 17: 0 → 1
Char. 42: 0 → 1
Char. 251: 0 → 1
Char. 264: 0 → 1

**Node 80:**
Char. 41: 0 → 1
Char. 61: 1 → 2
Char. 112: 1 → 0
Char. 128: 0 → 1
Char. 138: 0 → 1
Char. 146: 1 → 0
Char. 155: 0 → 1
Char. 182: 1 → 0
Char. 192: 1 → 2
Char. 224: 0 → 1
Char. 226: 0 → 1
Char. 227: 0 → 1
Char. 233: 0 → 1

**Node 81:**
Char. 61: 1 → 3
Char. 90: 0 → 1
Char. 91: 0 → 1
Char. 109: 1 → 0
Char. 155: 0 → 1
Char. 209: 1 → 0

## ANALYSIS 27
**(NO *CANDELARIA BARBOURI*, *PAPPOCHELYS ROSINAE*, *ODONTOCHELYS SEMITESTACEA*, AND *EUNOTOSAURUS AFRICANUS*)**

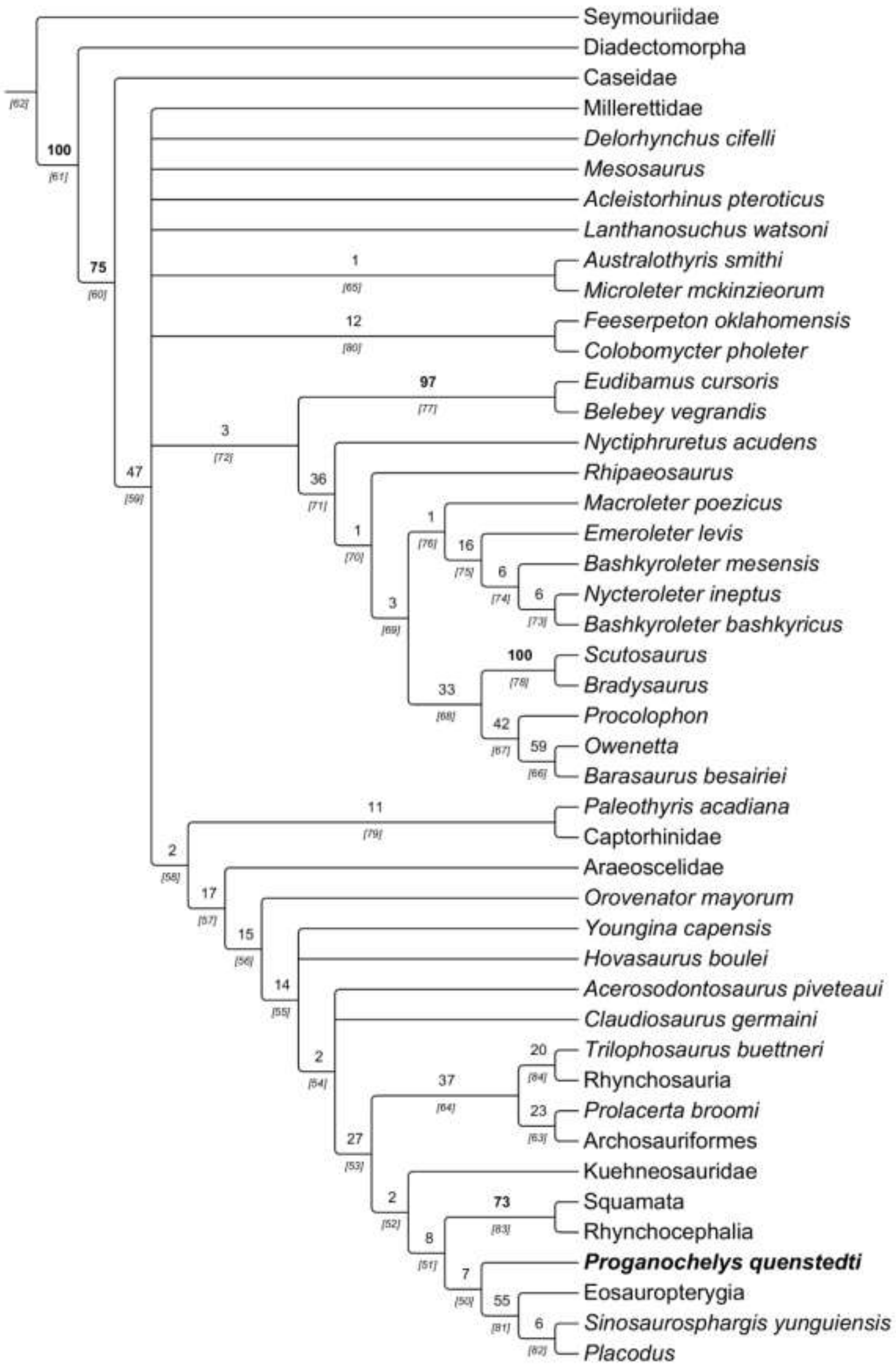

***Proganochelys quenstedti*:**
Char. 8: 0 → 1
Char. 11: 0 → 1
Char. 28: 0 → 1
Char. 35: 1 → 0
Char. 39: 0 → 1
Char. 43: 1 → 0
Char. 46: 1 → 0
Char. 50: 0 → 1
Char. 53: 0 → 1
Char. 57: 0 → 1
Char. 58: 1 → 0
Char. 61: 1 → 0
Char. 62: 1 → 0
Char. 65: 0 → 1
Char. 77: 0 → 1
Char. 84: 1 → 2
Char. 89: 1 → 0
Char. 93: 1 → 0
Char. 102: 1 → 2
Char. 110: 0 → 1
Char. 115: 0 → 1
Char. 129: 1 → 0
Char. 130: 0 → 1
Char. 142: 1 → 0
Char. 150: 1 → 0
Char. 152: 0 → 1
Char. 154: 0 → 1
Char. 160: 0 → 1
Char. 167: 1 → 0
Char. 175: 0 → 1
Char. 190: 1 → 0
Char. 195: 0 → 2
Char. 201: 1 → 0
Char. 202: 1 → 0
Char. 203: 1 → 2
Char. 214: 1 → 0
Char. 215: 0 → 1
Char. 218: 0 → 1
Char. 219: 1 → 0
Char. 235: 2 → 0
Char. 238: 0 → 2
Char. 241: 0 → 1
Char. 246: 0 → 2
Char. 247: 0 → 1
Char. 248: 0 → 1
Char. 250: 0 → 1
Char. 253: 0 → 2
Char. 254: 0 → 1
Char. 255: 0 → 1
Char. 256: 0 → 1
Char. 258: 0 → 1
Char. 259: 0 → 1
Char. 260: 1 → 2
Char. 262: 0 → 1
Char. 265: 1 → 0
Char. 268: 0 → 1
Char. 269: 0 → 1
Char. 270: 0 → 1

**Seymouriidae:**
Char. 51: 0 → 2
Char. 54: 1 → 0
Char. 71: 1 → 0
Char. 85: 1 → 0
Char. 99: 1 → 0
Char. 107: 0 → 1
Char. 140: 0 → 2
Char. 154: 1 → 2
Char. 225: 1 → 0
Char. 260: 2 → 0

***Acerosodontosaurus piveteaui*:**
Char. 78: 1 → 0
Char. 128: 0 → 1
Char. 155: 0 → 1
Char. 206: 0 → 2
Char. 267: 1 → 0
Char. 278: 0 → 1

***Acleistorhinus pteroticus*:**
Char. 20: 0 → 1
Char. 21: 0 → 1
Char. 30: 0 → 1
Char. 33: 0 → 1
Char. 47: 0 → 1
Char. 55: 1 → 0
Char. 56: 0 → 1
Char. 64: 0 → 1
Char. 70: 0 → 1
Char. 79: 0 → 1
Char. 95: 0 → 1
Char. 110: 0 → 1
Char. 113: 0 → 1
Char. 114: 0 → 1
Char. 119: 0 → 1
Char. 137: 0 → 1
Char. 140: 0 → 2
Char. 146: 1 → 0
Char. 169: 1 → 0
Char. 170: 0 → 1
Char. 278: 3 → 1

**Araeoscelidae:**
Char. 23: 0 → 1
Char. 28: 0 → 1
Char. 106: 0 → 1
Char. 239: 0 → 1

**Archosauriformes:**
Char. 32: 0 → 1
Char. 94: 1 → 0
Char. 112: 1 → 0
Char. 152: 0 → 1
Char. 154: 0 → 2
Char. 166: 1 → 0
Char. 171: 0 → 1
Char. 185: 0 → 1
Char. 204: 0 → 1
Char. 218: 0 → 3
Char. 242: 0 → 1

***Australothyris smithi*:**
Char. 23: 0 → 1
Char. 34: 0 → 1
Char. 55: 1 → 0
Char. 71: 1 → 0
Char. 85: 1 → 0
Char. 112: 0 → 1
Char. 129: 0 → 1

***Barasaurus besairiei*:**
Char. 33: 1 → 0
Char. 75: 1 → 0
Char. 216: 0 → 1

***Bashkyroleter bashkyricus*:**
Char. 275: 1 → 0

***Bashkyroleter mesensis*:**
Char. 169: 1 → 0

***Belebey vegrandis*:**
Char. 154: 1 → 0

***Bradysaurus* spp.:**
Char. 19: 0 → 1
Char. 73: 0 → 1
Char. 79: 0 → 1
Char. 135: 1 → 0
Char. 249: 0 → 1

**Captorhinidae:**
Char. 3: 0 → 1
Char. 23: 0 → 1
Char. 25: 1 → 0
Char. 26: 0 → 1
Char. 73: 0 → 1
Char. 75: 0 → 1
Char. 83: 0 → 1
Char. 108: 1 → 0
Char. 183: 1 → 0
Char. 201: 1 → 0
Char. 203: 1 → 2
Char. 216: 1 → 0

**Caseidae:**
Char. 24: 0 → 1
Char. 25: 1 → 0
Char. 36: 0 → 1
Char. 38: 1 → 0
Char. 46: 1 → 0
Char. 50: 1 → 0
Char. 56: 0 → 1
Char. 85: 1 → 0
Char. 96: 1 → 0
Char. 170: 0 → 1
Char. 194: 0 → 1
Char. 273: 0 → 1
Char. 274: 0 → 1
Char. 278: 3 → 2

***Claudiosaurus germaini*:**
Char. 24: 0 → 1
Char. 34: 0 → 1
Char. 36: 0 → 1
Char. 43: 1 → 0
Char. 64: 0 → 1
Char. 105: 0 → 1
Char. 130: 0 → 1
Char. 144: 1 → 0
Char. 187: 1 → 0
Char. 199: 0 → 1
Char. 203: 1 → 2
Char. 204: 0 → 1
Char. 220: 0 → 1
Char. 222: 1 → 0
Char. 234: 1 → 0

***Colobomycter pholeter*:**
Char. 21: 0 → 1
Char. 25: 1 → 0
Char. 84: 1 → 0
Char. 154: 1 → 0
Char. 167: 0 → 1
Char. 267: 0 → 1

***Delorhynchus cifelli*:**
Char. 18: 0 → 1
Char. 20: 0 → 1
Char. 21: 0 → 1
Char. 24: 0 → 1
Char. 26: 0 → 1
Char. 28: 0 → 1
Char. 33: 0 → 2
Char. 39: 0 → 1
Char. 52: 0 → 1
Char. 100: 0 → 1
Char. 111: 0 → 1
Char. 116: 1 → 0
Char. 117: 0 → 1
Char. 156: 1 → 0
Char. 167: 0 → 1
Char. 180: 0 → 1
Char. 189: 0 → 1
Char. 191: 0 → 1
Char. 192: 0 → 1
Char. 267: 0 → 1

**Diadectomorpha:**
Char. 0: 0 → 1
Char. 70: 0 → 1
Char. 75: 0 → 1
Char. 122: 0 → 1
Char. 123: 1 → 0
Char. 146: 1 → 0
Char. 275: 1 → 0
Char. 278: 3 → 0

***Emeroleter levis*:**
Char. 51: 1 → 0

**Eosauropterygia:**
Char. 158: 1 → 0
Char. 159: 1 → 0
Char. 166: 1 → 0
Char. 168: 0 → 1
Char. 178: 0 → 1
Char. 272: 1 → 0

***Eudibamus cursoris*:**
Char. 154: 1 → 2

***Feeserpeton oklahomensis*:**
Char. 51: 0 → 1
Char. 70: 0 → 1
Char. 157: 0 → 1
Char. 158: 0 → 1

***Hovasaurus boulei*:**
Char. 41: 0 → 1
Char. 55: 1 → 0
Char. 60: 1 → 0
Char. 72: 2 → 1
Char. 77: 0 → 2
Char. 78: 1 → 0
Char. 79: 0 → 1
Char. 93: 1 → 0
Char. 113: 0 → 1
Char. 138: 0 → 1
Char. 146: 1 → 0
Char. 154: 0 → 1
Char. 204: 0 → 2
Char. 206: 0 → 2
Char. 220: 0 → 1
Char. 278: 0 → 3

**Kuehneosauridae:**
Char. 7: 0 → 1
Char. 34: 0 → 1
Char. 36: 0 → 1
Char. 43: 1 → 0
Char. 82: 0 → 1
Char. 98: 1 → 0
Char. 113: 0 → 1
Char. 148: 1 → 0
Char. 159: 1 → 0
Char. 185: 0 → 1
Char. 206: 0 → 2
Char. 245: 0 → 1
Char. 272: 1 → 2
Char. 278: 0 → 3

***Lanthanosuchus watsoni*:**
Char. 25: 1 → 0
Char. 51: 0 → 1
Char. 76: 0 → 1
Char. 86: 0 → 1
Char. 95: 0 → 1
Char. 98: 1 → 0
Char. 110: 0 → 1
Char. 113: 0 → 1
Char. 114: 0 → 1
Char. 137: 0 → 1
Char. 138: 0 → 1
Char. 140: 0 → 2
Char. 144: 1 → 0
Char. 154: 1 → 2
Char. 192: 0 → 1

***Macroleter poezicus*:**
Char. 52: 0 → 1
Char. 84: 1 → 0
Char. 87: 0 → 1
Char. 140: 0 → 1
Char. 146: 1 → 0
Char. 169: 1 → 0
Char. 235: 0 → 1

***Mesosaurus* spp.:**
Char. 0: 0 → 1

Char. 2: 0 → 1
Char. 5: 1 → 0
Char. 6: 0 → 1
Char. 8: 0 → 1
Char. 9: 0 → 1
Char. 13: 0 → 1
Char. 19: 0 → 1
Char. 23: 0 → 1
Char. 26: 0 → 1
Char. 29: 1 → 0
Char. 33: 0 → 1
Char. 38: 1 → 0
Char. 41: 0 → 1
Char. 50: 1 → 0
Char. 67: 0 → 1
Char. 76: 0 → 1
Char. 83: 0 → 1
Char. 84: 1 → 0
Char. 85: 1 → 0
Char. 94: 0 → 1
Char. 107: 0 → 1
Char. 109: 0 → 1
Char. 111: 0 → 1
Char. 115: 0 → 1
Char. 146: 1 → 0
Char. 147: 1 → 0
Char. 148: 0 → 1
Char. 149: 1 → 0
Char. 164: 0 → 1
Char. 167: 0 → 1
Char. 176: 0 → 1
Char. 183: 1 → 0
Char. 184: 0 → 1
Char. 199: 0 → 1
Char. 202: 0 → 1
Char. 206: 0 → 1
Char. 207: 0 → 1
Char. 209: 0 → 1
Char. 217: 0 → 1
Char. 219: 0 → 1
Char. 220: 0 → 1
Char. 231: 0 → 1
Char. 260: 2 → 0
Char. 272: 2 → 0
Char. 278: 3 → 0

***Microleter mckinzieorum*:**
Char. 0: 0 → 1
Char. 25: 1 → 0
Char. 36: 0 → 1
Char. 39: 0 → 1
Char. 51: 0 → 2
Char. 56: 0 → 1
Char. 70: 0 → 1
Char. 94: 0 → 1
Char. 106: 0 → 1
Char. 276: 0 → 1

**Millerettidae:**
Char. 5: 1 → 0
Char. 24: 0 → 1
Char. 25: 1 → 0
Char. 44: 1 → 0
Char. 56: 0 → 1
Char. 57: 1 → 0
Char. 66: 0 → 2
Char. 78: 1 → 0
Char. 80: 1 → 0
Char. 84: 1 → 2
Char. 88: 1 → 0
Char. 96: 1 → 0
Char. 117: 0 → 1
Char. 119: 0 → 1
Char. 121: 0 → 1
Char. 124: 0 → 1
Char. 127: 0 → 1
Char. 135: 0 → 1
Char. 145: 0 → 1
Char. 147: 1 → 0
Char. 202: 0 → 1
Char. 211: 0 → 1
Char. 230: 0 → 1
Char. 234: 0 → 1
Char. 248: 0 → 1
Char. 252: 0 → 1
Char. 253: 0 → 1

***Nycteroleter ineptus*:**
Char. 278: 0 → 3

***Nyctiphruretus acudens*:**
Char. 21: 0 → 1
Char. 33: 1 → 2
Char. 41: 0 → 1
Char. 81: 1 → 0
Char. 84: 1 → 2
Char. 94: 0 → 1
Char. 167: 0 → 1
Char. 224: 0 → 1
Char. 266: 0 → 1
Char. 272: 2 → 1
Char. 276: 0 → 1

***Orovenator mayorum*:**
Char. 8: 0 → 1
Char. 24: 0 → 1
Char. 36: 0 → 1
Char. 160: 0 → 1
Char. 165: 0 → 1

***Owenetta* spp.:**
Char. 169: 1 → 0

***Paleothyris acadiana*:**
Char. 66: 1 → 2
Char. 102: 0 → 1
Char. 146: 1 → 0
Char. 239: 0 → 1

***Placodus* spp.:**
Char. 12: 0 → 1
Char. 13: 0 → 1
Char. 19: 0 → 1
Char. 31: 0 → 1
Char. 46: 1 → 0
Char. 57: 0 → 1
Char. 93: 1 → 0
Char. 102: 1 → 2
Char. 109: 1 → 0
Char. 163: 1 → 0
Char. 164: 0 → 1

***Procolophon* spp.:**
Char. 41: 0 → 1
Char. 69: 0 → 1
Char. 79: 0 → 1
Char. 88: 0 → 1
Char. 117: 1 → 0
Char. 149: 1 → 0
Char. 180: 0 → 1
Char. 237: 0 → 1
Char. 238: 0 → 1
Char. 272: 2 → 1
Char. 278: 3 → 0

***Prolacerta broomi*:**
Char. 58: 1 → 0
Char. 66: 1 → 0
Char. 67: 1 → 0
Char. 80: 1 → 0
Char. 139: 1 → 0
Char. 192: 1 → 0
Char. 203: 1 → 2
Char. 206: 0 → 12

***Rhipaeosaurus* spp.:**
Char. 172: 0 → 1
Char. 277: 0 → 1

**Rhynchocephalia:**
Char. 26: 1 → 0
Char. 77: 0 → 1
Char. 81: 0 → 1
Char. 94: 1 → 0
Char. 104: 0 → 1
Char. 117: 12 → 0
Char. 139: 1 → 0
Char. 167: 1 → 0
Char. 205: 1 → 0

**Rhynchosauria:**
Char. 0: 1 → 0
Char. 7: 0 → 1
Char. 9: 0 → 1
Char. 26: 0 → 1
Char. 68: 0 → 1
Char. 99: 1 → 0
Char. 150: 1 → 0
Char. 160: 0 → 1
Char. 161: 0 → 1
Char. 171: 0 → 2
Char. 223: 0 → 1
Char. 241: 0 → 1

***Scutosaurus* spp.:**
Char. 175: 0 → 1
Char. 218: 0 → 1
Char. 243: 0 → 2
Char. 244: 0 → 1
Char. 251: 0 → 1

***Sinosaurosphargis yunguiensis*:**
Char. 8: 0 → 1
Char. 23: 0 → 1
Char. 26: 1 → 0
Char. 30: 0 → 1
Char. 53: 0 → 1
Char. 82: 0 → 1
Char. 89: 1 → 0
Char. 127: 1 → 0
Char. 150: 1 → 0
Char. 154: 0 → 2
Char. 167: 1 → 0
Char. 253: 0 → 1
Char. 255: 0 → 1

**Squamata:**
Char. 45: 0 → 1
Char. 75: 0 → 1
Char. 80: 1 → 0
Char. 82: 0 → 1
Char. 92: 1 → 0
Char. 109: 1 → 0
Char. 122: 0 → 1
Char. 160: 0 → 1
Char. 245: 0 → 1

***Trilophosaurus buettneri*:**
Char. 5: 1 → 0
Char. 11: 0 → 1
Char. 55: 1 → 0
Char. 93: 1 → 0
Char. 104: 0 → 1
Char. 113: 0 → 1
Char. 122: 0 → 1
Char. 136: 1 → 0
Char. 144: 1 → 0
Char. 154: 0 → 1
Char. 157: 0 → 1
Char. 159: 1 → 0
Char. 177: 0 → 12
Char. 194: 0 → 1
Char. 203: 1 → 2
Char. 207: 1 → 0
Char. 208: 1 → 0
Char. 272: 1 → 0

***Youngina capensis*:**
Char. 21: 0 → 1
Char. 75: 0 → 1
Char. 211: 0 → 1
Char. 231: 0 → 1
Char. 239: 0 → 1

**Node 50:**
Char. 161: 0 → 1
Char. 189: 0 → 1
Char. 193: 1 → 0
Char. 198: 0 → 1
Char. 220: 0 → 1
Char. 239: 1 → 0
Char. 240: 1 → 0

**Node 51:**
Char. 21: 0 → 1
Char. 33: 1 → 2
Char. 235: 1 → 2

**Node 52:**
Char. 26: 0 → 1
Char. 35: 0 → 1
Char. 108: 1 → 0
Char. 128: 0 → 1
Char. 184: 0 → 1
Char. 210: 0 → 1

**Node 53:**
Char. 58: 0 → 1
Char. 61: 0 → 1
Char. 66: 0 → 1
Char. 69: 0 → 1
Char. 84: 0 → 1
Char. 150: 0 → 1
Char. 167: 0 → 1
Char. 176: 0 → 1
Char. 205: 0 → 1
Char. 230: 0 → 1
Char. 231: 0 → 1
Char. 232: 0 → 1
Char. 239: 0 → 1

**Node 54:**
Char. 23: 0 → 1
Char. 70: 0 → 1
Char. 73: 0 → 1
Char. 94: 0 → 1
Char. 131: 0 → 1
Char. 148: 0 → 1
Char. 272: 2 → 1

**Node 55:**
Char. 33: 0 → 1
Char. 135: 0 → 1
Char. 159: 0 → 1
Char. 267: 0 → 1
Char. 278: 1 → 0

**Node 56:**
Char. 20: 0 → 1
Char. 62: 0 → 1

**Node 57:**
Char. 0: 0 → 1
Char. 57: 1 → 0
Char. 89: 0 → 1
Char. 112: 0 → 1
Char. 120: 0 → 1
Char. 192: 0 → 1
Char. 193: 0 → 1
Char. 222: 0 → 1
Char. 224: 0 → 1
Char. 234: 0 → 1
Char. 266: 0 → 1
Char. 278: 3 → 1

**Node 58:**
Char. 29: 1 → 0
Char. 59: 0 → 1
Char. 60: 0 → 1
Char. 67: 0 → 1
Char. 72: 1 → 2
Char. 84: 1 → 0
Char. 111: 0 → 1

Char. 119: 0 → 1
Char. 147: 1 → 0
Char. 154: 1 → 0

**Node 59:**
Char. 5: 0 → 1
Char. 29: 0 → 1
Char. 40: 0 → 2
Char. 74: 0 → 1
Char. 88: 0 → 1
Char. 116: 0 → 1
Char. 147: 0 → 1
Char. 149: 0 → 1
Char. 169: 0 → 1
Char. 201: 0 → 1
Char. 203: 0 → 1
Char. 235: 0 → 1
Char. 276: 1 → 0

**Node 60:**
Char. 97: 0 → 1

**Node 61:**
Char. 23: 1 → 0
Char. 55: 0 → 1
Char. 78: 0 → 1
Char. 111: 1 → 0
Char. 135: 1 → 0
Char. 207: 1 → 0

**Node 62:**
Char. 15: 1 → 0
Char. 20: 1 → 0
Char. 25: 0 → 1
Char. 33: 2 → 0
Char. 73: 1 → 0
Char. 89: 1 → 0
Char. 94: 1 → 0
Char. 124: 1 → 0
Char. 127: 1 → 0
Char. 145: 1 → 0
Char. 147: 1 → 0
Char. 149: 1 → 0
Char. 174: 1 → 0
Char. 180: 1 → 0
Char. 187: 1 → 0
Char. 188: 1 → 0
Char. 194: 2 → 0
Char. 206: 2 → 0
Char. 217: 1 → 0
Char. 220: 1 → 0
Char. 221: 1 → 0
Char. 231: 1 → 0
Char. 247: 1 → 0
Char. 251: 1 → 0
Char. 252: 1 → 0
Char. 253: 2 → 0
Char. 277: 1 → 0

**Node 63:**
Char. 19: 0 → 1
Char. 92: 1 → 0

**Node 64:**
Char. 4: 0 → 1
Char. 29: 0 → 1
Char. 75: 0 → 1
Char. 213: 0 → 1
Char. 226: 0 → 2
Char. 228: 0 → 1
Char. 275: 0 → 1

**Node 65:**
Char. 24: 0 → 1
Char. 57: 1 → 0
Char. 79: 0 → 1
Char. 83: 0 → 1
Char. 110: 0 → 1
Char. 132: 1 → 0

**Node 66:**
Char. 73: 0 → 1
Char. 131: 1 → 0
Char. 205: 0 → 1
Char. 276: 0 → 1

**Node 67:**
Char. 18: 0 → 1
Char. 37: 0 → 1
Char. 107: 0 → 1
Char. 118: 1 → 0
Char. 125: 1 → 0
Char. 150: 0 → 1

**Node 68:**
Char. 23: 0 → 1
Char. 71: 1 → 0
Char. 102: 0 → 1
Char. 103: 0 → 1
Char. 106: 0 → 1
Char. 167: 0 → 1
Char. 214: 0 → 1
Char. 216: 1 → 0
Char. 235: 0 → 2
Char. 241: 0 → 1

**Node 69:**
Char. 207: 0 → 1

**Node 70:**
Char. 226: 0 → 1

**Node 71:**
Char. 20: 0 → 1
Char. 33: 0 → 1
Char. 44: 1 → 0
Char. 70: 0 → 1
Char. 80: 1 → 0
Char. 117: 0 → 1
Char. 118: 0 → 1
Char. 119: 0 → 1
Char. 125: 0 → 1
Char. 194: 0 → 1
Char. 201: 1 → 0
Char. 211: 0 → 1

**Node 72:**
Char. 38: 1 → 2
Char. 39: 0 → 1
Char. 49: 0 → 1
Char. 66: 0 → 12
Char. 76: 0 → 1
Char. 85: 1 → 0
Char. 88: 1 → 0
Char. 95: 0 → 1
Char. 112: 0 → 1
Char. 148: 0 → 1
Char. 158: 0 → 1
Char. 183: 1 → 2
Char. 192: 0 → 1

**Node 73:**
Char. 87: 0 → 1

**Node 74:**
Char. 25: 1 → 0
Char. 76: 1 → 0

**Node 75:**
Char. 79: 0 → 1
Char. 93: 1 → 0
Char. 133: 0 → 1

**Node 76:**
Char. 85: 0 → 1
Char. 147: 1 → 2
Char. 154: 1 → 0
Char. 278: 3 → 0

**Node 77:**
Char. 50: 1 → 0
Char. 58: 0 → 1
Char. 59: 0 → 1
Char. 60: 0 → 1
Char. 72: 1 → 2
Char. 104: 0 → 1
Char. 105: 0 → 1
Char. 106: 0 → 2
Char. 107: 0 → 1
Char. 109: 0 → 1
Char. 110: 0 → 1
Char. 146: 1 → 0
Char. 155: 0 → 2

**Node 78:**
Char. 33: 1 → 0
Char. 38: 2 → 1
Char. 39: 1 → 0
Char. 42: 0 → 1
Char. 43: 0 → 1
Char. 46: 1 → 0
Char. 49: 1 → 0
Char. 52: 0 → 1
Char. 83: 01 → 2
Char. 84: 1 → 0
Char. 87: 0 → 1
Char. 93: 1 → 0
Char. 161: 0 → 1
Char. 163: 1 → 0
Char. 172: 0 → 2
Char. 174: 0 → 1
Char. 188: 0 → 1
Char. 189: 0 → 1
Char. 195: 0 → 1
Char. 212: 0 → 1
Char. 236: 0 → 1
Char. 238: 0 → 2
Char. 242: 0 → 1
Char. 274: 0 → 1
Char. 275: 1 → 0

**Node 79:**
Char. 44: 1 → 0
Char. 66: 0 → 1
Char. 78: 1 → 0
Char. 88: 1 → 0
Char. 93: 1 → 0
Char. 123: 1 → 0

**Node 80:**
Char. 20: 0 → 1
Char. 47: 0 → 1
Char. 83: 0 → 1
Char. 85: 1 → 0
Char. 107: 0 → 1
Char. 110: 0 → 1
Char. 169: 1 → 0
Char. 170: 0 → 1

**Node 81:**
Char. 2: 0 → 1
Char. 6: 0 → 1
Char. 81: 0 → 1
Char. 98: 1 → 0
Char. 101: 0 → 1
Char. 104: 0 → 1
Char. 113: 0 → 1
Char. 128: 1 → 0
Char. 186: 0 → 1
Char. 206: 0 → 2
Char. 231: 1 → 0
Char. 232: 1 → 0
Char. 234: 1 → 0
Char. 266: 1 → 0
Char. 267: 1 → 0

**Node 82:**
Char. 17: 0 → 1
Char. 264: 0 → 1

**Node 83:**
Char. 41: 0 → 1
Char. 61: 1 → 2
Char. 112: 1 → 0
Char. 146: 1 → 0
Char. 192: 1 → 2
Char. 233: 0 → 1
Char. 267: 1 → 2

**Node 84:**
Char. 61: 1 → 3
Char. 90: 0 → 1
Char. 91: 0 → 1
Char. 109: 1 → 0
Char. 155: 0 → 1
Char. 209: 1 → 0

**ANALYSIS 28**
**(NO *PAPPOCHELYS ROSINAE*, *ODONTOCHELYS SEMITESTACEA*, AND *EUNOTOSAURUS AFRICANUS*)**

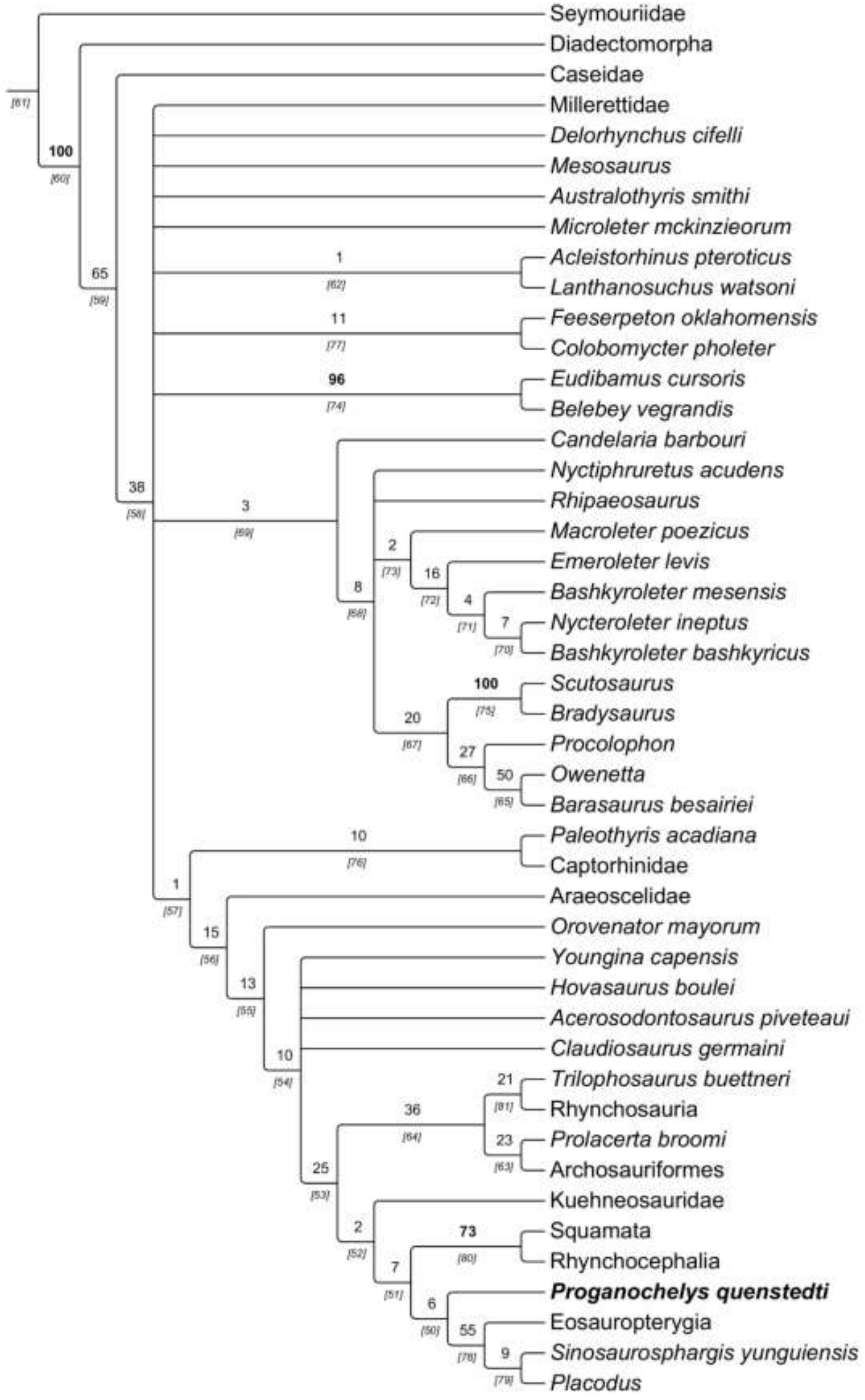

***Proganochelys quenstedti*:**
Char. 8: 0 → 1
Char. 11: 0 → 1
Char. 28: 0 → 1
Char. 35: 1 → 0
Char. 39: 0 → 1
Char. 43: 1 → 0
Char. 46: 1 → 0
Char. 50: 0 → 1
Char. 53: 0 → 1
Char. 57: 0 → 1
Char. 58: 1 → 0
Char. 61: 1 → 0
Char. 62: 1 → 0
Char. 65: 0 → 1
Char. 77: 0 → 1
Char. 84: 1 → 2
Char. 89: 1 → 0
Char. 93: 1 → 0
Char. 102: 1 → 2
Char. 110: 0 → 1
Char. 115: 0 → 1
Char. 129: 1 → 0
Char. 130: 0 → 1
Char. 142: 1 → 0
Char. 150: 1 → 0
Char. 152: 0 → 1
Char. 154: 0 → 1
Char. 160: 0 → 1
Char. 167: 1 → 0
Char. 175: 0 → 1
Char. 190: 1 → 0
Char. 195: 0 → 2
Char. 201: 1 → 0
Char. 202: 1 → 0
Char. 203: 1 → 2
Char. 214: 1 → 0
Char. 215: 0 → 1
Char. 218: 0 → 1
Char. 219: 1 → 0
Char. 235: 2 → 0
Char. 238: 0 → 2
Char. 241: 0 → 1
Char. 246: 0 → 2
Char. 247: 0 → 1
Char. 248: 0 → 1
Char. 250: 0 → 1
Char. 253: 0 → 2
Char. 254: 0 → 1
Char. 255: 0 → 1
Char. 256: 0 → 1
Char. 258: 0 → 1
Char. 259: 0 → 1
Char. 260: 1 → 2
Char. 262: 0 → 1
Char. 265: 1 → 0
Char. 268: 0 → 1
Char. 269: 0 → 1
Char. 270: 0 → 1

**Seymouriidae:**
Char. 51: 0 → 2
Char. 54: 1 → 0
Char. 71: 1 → 0
Char. 83: 0 → 1
Char. 85: 1 → 0
Char. 99: 1 → 0
Char. 107: 0 → 1
Char. 126: 0 → 1
Char. 140: 0 → 2
Char. 154: 1 → 2
Char. 225: 1 → 0
Char. 260: 2 → 0

***Acerosodontosaurus piveteaui*:**
Char. 78: 1 → 0
Char. 81: 1 → 0
Char. 128: 0 → 1
Char. 155: 0 → 1
Char. 206: 0 → 2
Char. 208: 0 → 1
Char. 224: 1 → 0
Char. 267: 1 → 0
Char. 278: 0 → 1

***Acleistorhinus pteroticus*:**
Char. 21: 0 → 1
Char. 119: 0 → 1
Char. 146: 1 → 0
Char. 169: 1 → 0
Char. 170: 0 → 1

**Araeoscelidae:**
Char. 28: 0 → 1
Char. 106: 0 → 1
Char. 239: 0 → 1

**Archosauriformes:**
Char. 32: 0 → 1
Char. 94: 1 → 0
Char. 112: 1 → 0
Char. 152: 0 → 1
Char. 154: 0 → 2
Char. 166: 1 → 0
Char. 171: 0 → 1
Char. 185: 0 → 1
Char. 204: 0 → 1
Char. 218: 0 → 3
Char. 242: 0 → 1

***Australothyris smithi*:**
Char. 23: 0 → 1
Char. 24: 0 → 1
Char. 34: 0 → 1
Char. 55: 1 → 0
Char. 57: 1 → 0
Char. 71: 1 → 0
Char. 79: 0 → 1
Char. 85: 1 → 0
Char. 98: 1 → 0
Char. 100: 0 → 1
Char. 103: 0 → 1
Char. 110: 0 → 1
Char. 112: 0 → 1
Char. 123: 1 → 0
Char. 129: 0 → 1
Char. 131: 0 → 1
Char. 132: 1 → 0
Char. 144: 1 → 0
Char. 149: 1 → 0
Char. 150: 0 → 1

***Barasaurus besairiei*:**
Char. 33: 1 → 0
Char. 75: 1 → 0
Char. 216: 0 → 1

***Bashkyroleter bashkyricus*:**
Char. 275: 1 → 0

***Bashkyroleter mesensis*:**
Char. 169: 1 → 0

***Belebey vegrandis*:**
Char. 154: 1 → 0

***Bradysaurus* spp.:**
Char. 19: 0 → 1
Char. 73: 0 → 1
Char. 79: 0 → 1
Char. 135: 1 → 0
Char. 249: 0 → 1

***Candelaria barbouri*:**
Char. 1: 0 → 1
Char. 8: 0 → 1
Char. 15: 0 → 1
Char. 23: 0 → 1
Char. 25: 1 → 0
Char. 29: 1 → 0
Char. 50: 1 → 0
Char. 55: 1 → 0
Char. 57: 1 → 0
Char. 67: 0 → 1
Char. 79: 0 → 2
Char. 89: 0 → 1
Char. 127: 0 → 1
Char. 154: 1 → 2
Char. 169: 1 → 0
Char. 277: 0 → 1

**Captorhinidae:**
Char. 3: 0 → 1
Char. 25: 1 → 0
Char. 26: 0 → 1
Char. 73: 0 → 1
Char. 75: 0 → 1
Char. 108: 1 → 0
Char. 183: 1 → 0
Char. 201: 1 → 0
Char. 203: 1 → 2
Char. 216: 1 → 0

**Caseidae:**
Char. 24: 0 → 1
Char. 25: 1 → 0
Char. 36: 0 → 1
Char. 38: 1 → 0
Char. 46: 1 → 0
Char. 50: 1 → 0
Char. 56: 0 → 1
Char. 85: 1 → 0
Char. 96: 1 → 0
Char. 170: 0 → 1
Char. 194: 0 → 1
Char. 273: 0 → 1
Char. 274: 0 → 1
Char. 278: 3 → 2

***Claudiosaurus germaini*:**
Char. 24: 0 → 1
Char. 34: 0 → 1
Char. 36: 0 → 1
Char. 43: 1 → 0
Char. 64: 0 → 1
Char. 73: 0 → 1
Char. 105: 0 → 1
Char. 106: 0 → 1
Char. 126: 0 → 1
Char. 130: 0 → 1
Char. 131: 0 → 1
Char. 144: 1 → 0
Char. 148: 0 → 1
Char. 182: 0 → 1
Char. 187: 1 → 0
Char. 190: 0 → 1
Char. 199: 0 → 1
Char. 203: 1 → 2
Char. 204: 0 → 1
Char. 220: 0 → 1
Char. 222: 1 → 0
Char. 224: 1 → 0
Char. 234: 1 → 0
Char. 272: 2 → 1

***Colobomycter pholeter*:**
Char. 21: 0 → 1
Char. 25: 1 → 0
Char. 84: 1 → 0
Char. 154: 1 → 0
Char. 167: 0 → 1
Char. 267: 0 → 1

***Delorhynchus cifelli*:**
Char. 18: 0 → 1
Char. 20: 0 → 1
Char. 21: 0 → 1
Char. 24: 0 → 1
Char. 26: 0 → 1
Char. 28: 0 → 1
Char. 33: 0 → 2
Char. 39: 0 → 1
Char. 48: 0 → 1
Char. 52: 0 → 1
Char. 83: 1 → 0
Char. 100: 0 → 1
Char. 111: 0 → 1
Char. 116: 1 → 0
Char. 117: 0 → 1
Char. 131: 0 → 1
Char. 156: 1 → 0
Char. 167: 0 → 1
Char. 180: 0 → 1
Char. 189: 0 → 1
Char. 191: 0 → 1
Char. 267: 0 → 1

**Diadectomorpha:**
Char. 0: 0 → 1
Char. 70: 0 → 1
Char. 75: 0 → 1
Char. 122: 0 → 1
Char. 123: 1 → 0
Char. 146: 1 → 0
Char. 275: 1 → 0
Char. 278: 3 → 0

***Emeroleter levis*:**
Char. 0: 0 → 2
Char. 51: 1 → 0

**Eosauropterygia:**
Char. 158: 1 → 0
Char. 159: 1 → 0
Char. 166: 1 → 0
Char. 168: 0 → 1
Char. 178: 0 → 1
Char. 272: 1 → 0

***Eudibamus cursoris*:**
Char. 154: 1 → 2

***Feeserpeton oklahomensis*:**
Char. 51: 0 → 1
Char. 70: 0 → 1
Char. 157: 0 → 1
Char. 158: 0 → 1

***Hovasaurus boulei*:**
Char. 41: 0 → 1
Char. 55: 1 → 0
Char. 60: 1 → 0
Char. 72: 2 → 1
Char. 77: 0 → 2
Char. 78: 1 → 0
Char. 79: 0 → 1
Char. 93: 1 → 0
Char. 113: 0 → 1
Char. 138: 0 → 1
Char. 146: 1 → 0
Char. 154: 0 → 1
Char. 204: 0 → 2
Char. 206: 0 → 2
Char. 214: 1 → 0
Char. 215: 0 → 1
Char. 219: 1 → 0
Char. 220: 0 → 1
Char. 278: 0 → 3

**Kuehneosauridae:**
Char. 7: 0 → 1
Char. 34: 0 → 1
Char. 36: 0 → 1
Char. 43: 1 → 0
Char. 82: 0 → 1
Char. 98: 1 → 0
Char. 113: 0 → 1
Char. 159: 1 → 0
Char. 185: 0 → 1
Char. 206: 0 → 2
Char. 245: 0 → 1
Char. 278: 0 → 3

***Lanthanosuchus watsoni*:**
Char. 25: 1 → 0
Char. 51: 0 → 1
Char. 86: 0 → 1
Char. 98: 1 → 0
Char. 138: 0 → 1
Char. 144: 1 → 0
Char. 154: 1 → 2

***Macroleter poezicus*:**
Char. 0: 0 → 1
Char. 52: 0 → 1
Char. 66: 1 → 2
Char. 75: 0 → 1
Char. 84: 1 → 0
Char. 87: 0 → 1
Char. 110: 0 → 1
Char. 121: 0 → 1
Char. 140: 0 → 1
Char. 146: 1 → 0
Char. 169: 1 → 0
Char. 235: 0 → 1

***Mesosaurus* spp.:**
Char. 0: 0 → 1
Char. 2: 0 → 1
Char. 6: 0 → 1
Char. 8: 0 → 1
Char. 9: 0 → 1
Char. 13: 0 → 1
Char. 19: 0 → 1
Char. 23: 0 → 1
Char. 26: 0 → 1
Char. 29: 1 → 0
Char. 33: 0 → 1
Char. 38: 1 → 0
Char. 41: 0 → 1
Char. 48: 0 → 1
Char. 50: 1 → 0
Char. 67: 0 → 1
Char. 84: 1 → 0
Char. 85: 1 → 0
Char. 94: 0 → 1
Char. 107: 0 → 1
Char. 109: 0 → 1
Char. 111: 0 → 1
Char. 115: 0 → 1
Char. 146: 1 → 0
Char. 148: 0 → 1
Char. 149: 1 → 0
Char. 164: 0 → 1
Char. 166: 1 → 0
Char. 167: 0 → 1
Char. 176: 0 → 1
Char. 183: 1 → 0
Char. 184: 0 → 1
Char. 199: 0 → 1
Char. 202: 0 → 1
Char. 206: 0 → 1
Char. 207: 0 → 1
Char. 209: 0 → 1
Char. 217: 0 → 1
Char. 219: 0 → 1
Char. 220: 0 → 1
Char. 231: 0 → 1
Char. 260: 2 → 0
Char. 272: 2 → 0
Char. 278: 3 → 0

***Microleter mckinzieorum*:**
Char. 0: 0 → 1
Char. 18: 0 → 1
Char. 24: 0 → 1
Char. 25: 1 → 0
Char. 36: 0 → 1
Char. 39: 0 → 1
Char. 51: 0 → 2
Char. 56: 0 → 1
Char. 57: 1 → 0
Char. 70: 0 → 1
Char. 79: 0 → 1
Char. 94: 0 → 1
Char. 106: 0 → 1
Char. 110: 0 → 1
Char. 132: 1 → 0
Char. 166: 1 → 0
Char. 276: 0 → 1
Char. 278: 3 → 1

**Millerettidae:**
Char. 24: 0 → 1
Char. 25: 1 → 0
Char. 44: 1 → 0
Char. 56: 0 → 1
Char. 57: 1 → 0
Char. 66: 0 → 2
Char. 78: 1 → 0
Char. 80: 1 → 0
Char. 83: 1 → 0
Char. 84: 1 → 2
Char. 88: 1 → 0
Char. 96: 1 → 0
Char. 117: 0 → 1
Char. 119: 0 → 1
Char. 121: 0 → 1
Char. 124: 0 → 1
Char. 127: 0 → 1
Char. 135: 0 → 1
Char. 145: 0 → 1
Char. 166: 1 → 0
Char. 202: 0 → 1
Char. 211: 0 → 1
Char. 230: 0 → 1
Char. 234: 0 → 1
Char. 248: 0 → 1
Char. 252: 0 → 1
Char. 253: 0 → 1

***Nycteroleter ineptus*:**
Char. 278: 0 → 3

***Nyctiphruretus acudens*:**
Char. 0: 0 → 1
Char. 21: 0 → 1
Char. 41: 0 → 1
Char. 66: 1 → 2
Char. 81: 1 → 0
Char. 84: 1 → 2
Char. 166: 1 → 0
Char. 167: 0 → 1
Char. 215: 1 → 0
Char. 224: 0 → 1
Char. 226: 1 → 0
Char. 266: 0 → 1
Char. 272: 2 → 1

***Orovenator mayorum*:**
Char. 8: 0 → 1
Char. 24: 0 → 1
Char. 36: 0 → 1
Char. 160: 0 → 1
Char. 165: 0 → 1

***Owenetta* spp.:**
Char. 169: 1 → 0

***Paleothyris acadiana*:**
Char. 66: 1 → 2
Char. 102: 0 → 1
Char. 146: 1 → 0
Char. 239: 0 → 1

***Placodus* spp.:**
Char. 12: 0 → 1
Char. 13: 0 → 1
Char. 19: 0 → 1
Char. 31: 0 → 1
Char. 46: 1 → 0
Char. 57: 0 → 1
Char. 93: 1 → 0
Char. 102: 1 → 2
Char. 109: 1 → 0
Char. 163: 1 → 0
Char. 164: 0 → 1

***Procolophon* spp.:**
Char. 41: 0 → 1
Char. 69: 0 → 1
Char. 79: 0 → 1
Char. 88: 0 → 1
Char. 117: 1 → 0
Char. 149: 1 → 0
Char. 180: 0 → 1
Char. 237: 0 → 1
Char. 238: 0 → 1
Char. 272: 2 → 1
Char. 278: 3 → 0

***Prolacerta broomi*:**
Char. 58: 1 → 0
Char. 66: 1 → 0
Char. 67: 1 → 0
Char. 80: 1 → 0
Char. 139: 1 → 0
Char. 192: 1 → 0
Char. 203: 1 → 2
Char. 206: 0 → 12
Char. 224: 1 → 0

***Rhipaeosaurus* spp.:**
Char. 172: 0 → 1
Char. 186: 1 → 0
Char. 277: 0 → 1

**Rhynchocephalia:**
Char. 26: 1 → 0
Char. 77: 0 → 1
Char. 81: 0 → 1
Char. 94: 1 → 0
Char. 104: 0 → 1
Char. 117: 12 → 0
Char. 139: 1 → 0
Char. 167: 1 → 0
Char. 205: 1 → 0

**Rhynchosauria:**
Char. 0: 1 → 0
Char. 7: 0 → 1
Char. 9: 0 → 1
Char. 26: 0 → 1
Char. 68: 0 → 1
Char. 99: 1 → 0
Char. 150: 1 → 0
Char. 160: 0 → 1
Char. 161: 0 → 1
Char. 171: 0 → 2
Char. 223: 0 → 1
Char. 241: 0 → 1

***Scutosaurus* spp.:**
Char. 175: 0 → 1
Char. 218: 0 → 1
Char. 243: 0 → 2
Char. 244: 0 → 1
Char. 251: 0 → 1

***Sinosaurosphargis yunguiensis*:**
Char. 8: 0 → 1
Char. 23: 0 → 1
Char. 26: 1 → 0
Char. 30: 0 → 1
Char. 53: 0 → 1
Char. 82: 0 → 1
Char. 89: 1 → 0
Char. 127: 1 → 0
Char. 150: 1 → 0
Char. 154: 0 → 2
Char. 167: 1 → 0
Char. 253: 0 → 1
Char. 255: 0 → 1

**Squamata:**
Char. 45: 0 → 1
Char. 75: 0 → 1
Char. 80: 1 → 0
Char. 82: 0 → 1
Char. 92: 1 → 0
Char. 109: 1 → 0
Char. 122: 0 → 1
Char. 160: 0 → 1
Char. 245: 0 → 1

***Trilophosaurus buettneri*:**
Char. 5: 1 → 0
Char. 11: 0 → 1
Char. 55: 1 → 0
Char. 93: 1 → 0
Char. 104: 0 → 1
Char. 106: 0 → 1
Char. 113: 0 → 1
Char. 122: 0 → 1
Char. 136: 1 → 0
Char. 144: 1 → 0
Char. 154: 0 → 1
Char. 157: 0 → 1
Char. 159: 1 → 0
Char. 177: 0 → 12
Char. 194: 0 → 1
Char. 203: 1 → 2
Char. 207: 1 → 0
Char. 208: 1 → 0
Char. 224: 1 → 0
Char. 272: 1 → 0

***Youngina capensis*:**
Char. 5: 1 → 0
Char. 21: 0 → 1
Char. 23: 1 → 0
Char. 75: 0 → 1
Char. 92: 1 → 0
Char. 94: 1 → 0
Char. 117: 12 → 0
Char. 163: 1 → 0
Char. 170: 0 → 1
Char. 211: 0 → 1
Char. 215: 0 → 1
Char. 231: 0 → 1
Char. 239: 0 → 1

**Node 50:**
Char. 161: 0 → 1
Char. 189: 0 → 1
Char. 193: 1 → 0
Char. 198: 0 → 1
Char. 220: 0 → 1
Char. 239: 1 → 0
Char. 240: 1 → 0

**Node 51:**
Char. 21: 0 → 1
Char. 33: 1 → 2
Char. 235: 1 → 2

**Node 52:**
Char. 26: 0 → 1
Char. 35: 0 → 1
Char. 108: 1 → 0
Char. 128: 0 → 1
Char. 184: 0 → 1
Char. 210: 0 → 1

**Node 53:**
Char. 58: 0 → 1
Char. 61: 0 → 1
Char. 66: 0 → 1
Char. 69: 0 → 1
Char. 73: 0 → 1
Char. 81: 1 → 0
Char. 84: 0 → 1
Char. 126: 0 → 1
Char. 131: 0 → 1
Char. 145: 0 → 1
Char. 150: 0 → 1

Char. 151: 0 → 1
Char. 167: 0 → 1
Char. 176: 0 → 1
Char. 190: 0 → 1
Char. 205: 0 → 1
Char. 208: 0 → 1
Char. 230: 0 → 1
Char. 231: 0 → 1
Char. 232: 0 → 1
Char. 239: 0 → 1

**Node 54:**
Char. 33: 0 → 1
Char. 92: 0 → 1
Char. 94: 0 → 1
Char. 135: 0 → 1
Char. 159: 0 → 1
Char. 267: 0 → 1
Char. 278: 1 → 0

**Node 55:**
Char. 20: 0 → 1
Char. 62: 0 → 1

**Node 56:**
Char. 0: 0 → 1
Char. 57: 1 → 0
Char. 89: 0 → 1
Char. 112: 0 → 1
Char. 120: 0 → 1
Char. 193: 0 → 1
Char. 222: 0 → 1
Char. 224: 0 → 1
Char. 234: 0 → 1
Char. 266: 0 → 1
Char. 278: 3 → 1

**Node 57:**
Char. 29: 1 → 0
Char. 59: 0 → 1
Char. 60: 0 → 1
Char. 67: 0 → 1
Char. 72: 1 → 2
Char. 84: 1 → 0
Char. 111: 0 → 1
Char. 119: 0 → 1
Char. 154: 1 → 0

**Node 58:**
Char. 29: 0 → 1
Char. 40: 0 → 2
Char. 74: 0 → 1
Char. 83: 0 → 1
Char. 88: 0 → 1
Char. 116: 0 → 1
Char. 149: 0 → 1
Char. 166: 0 → 1
Char. 169: 0 → 1
Char. 201: 0 → 1
Char. 203: 0 → 1
Char. 235: 0 → 1

**Node 59:**
Char. 81: 0 → 1
Char. 97: 0 → 1

**Node 60:**
Char. 55: 0 → 1
Char. 78: 0 → 1
Char. 111: 1 → 0
Char. 135: 1 → 0
Char. 207: 1 → 0

**Node 61:**
Char. 20: 1 → 0
Char. 25: 0 → 1
Char. 33: 2 → 0
Char. 73: 1 → 0
Char. 94: 1 → 0
Char. 124: 1 → 0
Char. 127: 1 → 0
Char. 145: 1 → 0
Char. 147: 1 → 0
Char. 149: 1 → 0
Char. 174: 1 → 0
Char. 180: 1 → 0
Char. 187: 1 → 0
Char. 188: 1 → 0
Char. 194: 2 → 0
Char. 206: 2 → 0
Char. 217: 1 → 0
Char. 220: 1 → 0
Char. 221: 1 → 0
Char. 231: 1 → 0
Char. 247: 1 → 0
Char. 251: 1 → 0
Char. 252: 1 → 0
Char. 253: 2 → 0

**Node 62:**
Char. 83: 1 → 0
Char. 95: 0 → 1
Char. 110: 0 → 1
Char. 113: 0 → 1
Char. 114: 0 → 1
Char. 131: 0 → 1
Char. 137: 0 → 1
Char. 140: 0 → 2

**Node 63:**
Char. 19: 0 → 1
Char. 92: 1 → 0

**Node 64:**
Char. 4: 0 → 1
Char. 29: 0 → 1
Char. 75: 0 → 1
Char. 213: 0 → 1
Char. 226: 0 → 2
Char. 228: 0 → 1
Char. 275: 0 → 1

**Node 65:**
Char. 73: 0 → 1
Char. 83: 1 → 0
Char. 131: 1 → 0
Char. 205: 0 → 1
Char. 239: 0 → 1

**Node 66:**
Char. 18: 0 → 1
Char. 37: 0 → 1
Char. 48: 0 → 1
Char. 107: 0 → 1
Char. 118: 1 → 0
Char. 125: 1 → 0
Char. 150: 0 → 1

**Node 67:**
Char. 23: 0 → 1
Char. 71: 1 → 0
Char. 75: 0 → 1
Char. 102: 0 → 1
Char. 103: 0 → 1
Char. 106: 0 → 1
Char. 110: 0 → 1
Char. 155: 0 → 1
Char. 167: 0 → 1
Char. 207: 0 → 1
Char. 214: 0 → 1
Char. 216: 1 → 0
Char. 235: 0 → 2
Char. 241: 0 → 1

**Node 68:**
Char. 66: 0 → 1

**Node 69:**
Char. 20: 0 → 1
Char. 33: 0 → 12
Char. 49: 0 → 1
Char. 88: 1 → 0
Char. 95: 0 → 1

**Node 70:**
Char. 87: 0 → 1
Char. 110: 0 → 1

**Node 71:**
Char. 25: 1 → 0
Char. 76: 1 → 0

**Node 72:**
Char. 79: 0 → 1
Char. 86: 0 → 1
Char. 93: 1 → 0
Char. 133: 0 → 1

**Node 73:**
Char. 48: 0 → 1
Char. 83: 1 → 0
Char. 100: 0 → 1
Char. 113: 0 → 1
Char. 147: 1 → 2

Char. 154: 1 → 0
Char. 207: 0 → 1
Char. 239: 0 → 1
Char. 278: 3 → 0

**Node 74:**
Char. 38: 1 → 2
Char. 39: 0 → 1
Char. 50: 1 → 0
Char. 58: 0 → 1
Char. 59: 0 → 1
Char. 60: 0 → 1
Char. 72: 1 → 2
Char. 85: 1 → 0
Char. 88: 1 → 0
Char. 95: 0 → 1
Char. 104: 0 → 1
Char. 105: 0 → 1
Char. 106: 0 → 2
Char. 107: 0 → 1
Char. 109: 0 → 1
Char. 110: 0 → 1
Char. 146: 1 → 0
Char. 148: 0 → 1
Char. 155: 0 → 2
Char. 183: 1 → 2

**Node 75:**
Char. 0: 0 → 2
Char. 33: 1 → 0
Char. 38: 2 → 1
Char. 39: 1 → 0
Char. 42: 0 → 1
Char. 43: 0 → 1
Char. 46: 1 → 0
Char. 49: 1 → 0
Char. 52: 0 → 1
Char. 83: 1 → 2
Char. 84: 1 → 0
Char. 87: 0 → 1
Char. 93: 1 → 0
Char. 100: 0 → 1
Char. 113: 0 → 1
Char. 143: 0 → 1
Char. 161: 0 → 1
Char. 163: 1 → 0
Char. 172: 0 → 2
Char. 174: 0 → 1
Char. 188: 0 → 1
Char. 189: 0 → 1
Char. 195: 0 → 1
Char. 212: 0 → 1
Char. 236: 0 → 1
Char. 238: 0 → 2
Char. 242: 0 → 1
Char. 245: 0 → 1
Char. 274: 0 → 1
Char. 275: 1 → 0

**Node 76:**
Char. 44: 1 → 0
Char. 66: 0 → 1
Char. 78: 1 → 0
Char. 88: 1 → 0
Char. 93: 1 → 0
Char. 123: 1 → 0

**Node 77:**
Char. 20: 0 → 1
Char. 47: 0 → 1
Char. 85: 1 → 0
Char. 107: 0 → 1
Char. 110: 0 → 1
Char. 169: 1 → 0
Char. 170: 0 → 1

**Node 78:**
Char. 2: 0 → 1
Char. 6: 0 → 1
Char. 81: 0 → 1
Char. 98: 1 → 0
Char. 101: 0 → 1
Char. 104: 0 → 1
Char. 113: 0 → 1
Char. 128: 1 → 0
Char. 182: 0 → 1
Char. 186: 0 → 1
Char. 206: 0 → 2
Char. 224: 1 → 0
Char. 231: 1 → 0
Char. 232: 1 → 0
Char. 234: 1 → 0
Char. 266: 1 → 0
Char. 267: 1 → 0

**Node 79:**
Char. 17: 0 → 1
Char. 264: 0 → 1

**Node 80:**
Char. 41: 0 → 1
Char. 61: 1 → 2
Char. 112: 1 → 0
Char. 146: 1 → 0
Char. 192: 1 → 2
Char. 233: 0 → 1
Char. 267: 1 → 2

**Node 81:**
Char. 61: 1 → 3
Char. 90: 0 → 1
Char. 91: 0 → 1
Char. 109: 1 → 0
Char. 155: 0 → 1
Char. 209: 1 → 0

**Analysis 29**

**(no *Candelaria barbouri*, *Odontochelys semitestacea*, and *Eunotosaurus africanus*)**

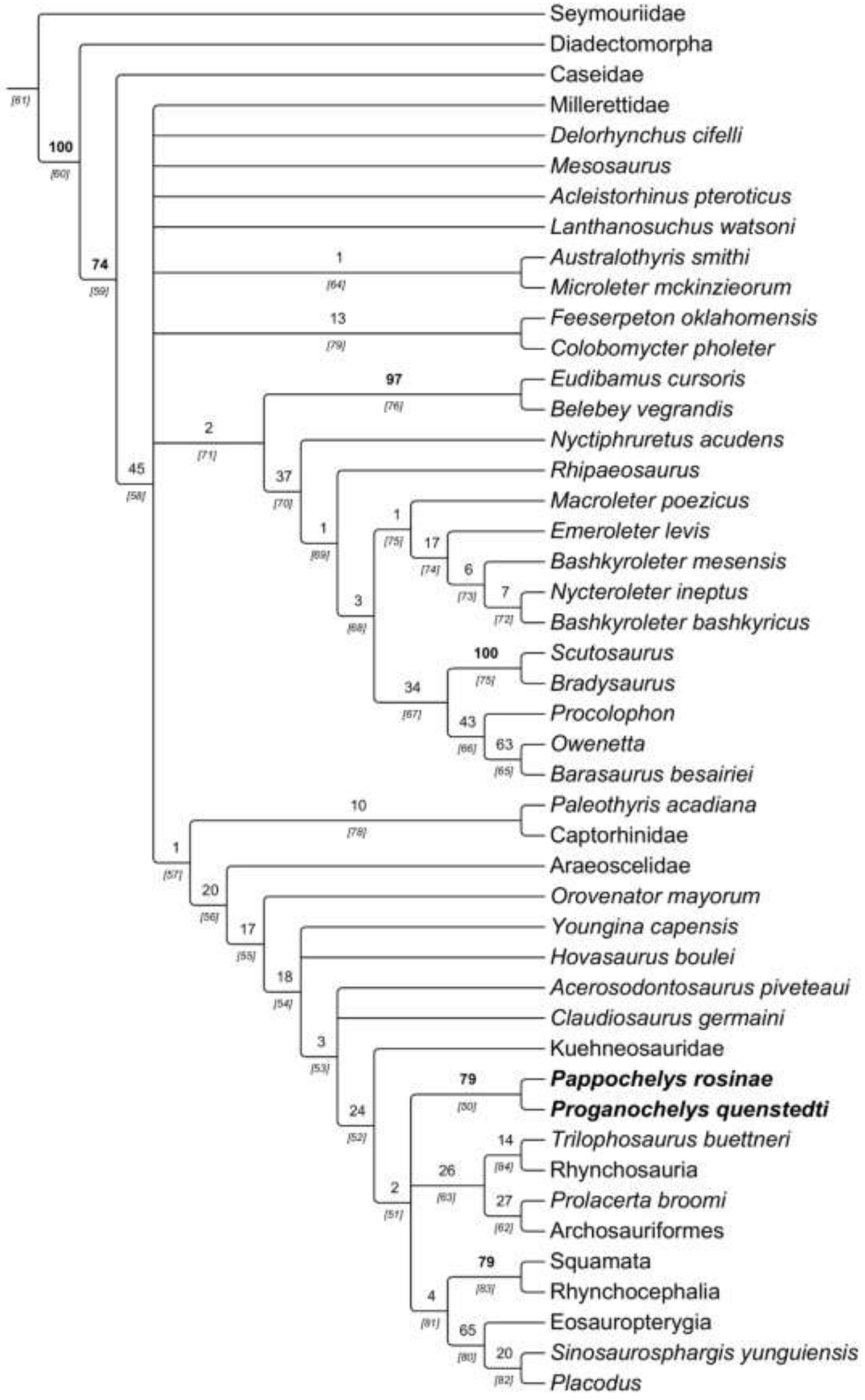

***Proganochelys quenstedti*:**
Char. 11: 0 → 1
Char. 23: 1 → 0
Char. 24: 0 → 1
Char. 42: 0 → 1
Char. 46: 1 → 0
Char. 50: 0 → 1
Char. 53: 0 → 1
Char. 57: 0 → 1
Char. 75: 1 → 0
Char. 77: 0 → 1
Char. 79: 0 → 2
Char. 88: 1 → 0
Char. 89: 1 → 0
Char. 93: 1 → 0
Char. 128: 0 → 1
Char. 129: 1 → 0
Char. 175: 0 → 1
Char. 195: 0 → 2
Char. 198: 0 → 1
Char. 202: 1 → 0
Char. 240: 1 → 0
Char. 246: 1 → 2
Char. 248: 0 → 1
Char. 250: 0 → 1
Char. 259: 0 → 1
Char. 262: 0 → 1
Char. 265: 1 → 0
Char. 270: 0 → 1

**Seymouriidae:**
Char. 51: 0 → 2
Char. 54: 1 → 0
Char. 71: 1 → 0
Char. 85: 1 → 0
Char. 99: 1 → 0
Char. 107: 0 → 1
Char. 126: 0 → 1
Char. 140: 0 → 2
Char. 154: 1 → 2
Char. 225: 1 → 0
Char. 260: 2 → 0

***Pappochelys rosinae*:**
Char. 1: 0 → 1
Char. 5: 1 → 0
Char. 41: 0 → 1
Char. 49: 0 → 1
Char. 55: 1 → 0
Char. 67: 1 → 0
Char. 176: 1 → 0
Char. 206: 0 → 2
Char. 252: 0 → 1

***Acerosodontosaurus piveteaui*:**
Char. 78: 1 → 0
Char. 128: 0 → 1
Char. 155: 0 → 1
Char. 206: 0 → 2
Char. 267: 1 → 0
Char. 278: 0 → 1

***Acleistorhinus pteroticus*:**
Char. 20: 0 → 1
Char. 21: 0 → 1
Char. 30: 0 → 1
Char. 33: 0 → 1
Char. 47: 0 → 1
Char. 55: 1 → 0
Char. 56: 0 → 1
Char. 64: 0 → 1
Char. 70: 0 → 1
Char. 79: 0 → 1
Char. 95: 0 → 1
Char. 110: 0 → 1
Char. 113: 0 → 1
Char. 114: 0 → 1
Char. 119: 0 → 1
Char. 137: 0 → 1
Char. 140: 0 → 2
Char. 146: 1 → 0
Char. 169: 1 → 0
Char. 170: 0 → 1
Char. 278: 3 → 1

**Araeoscelidae:**
Char. 23: 0 → 1
Char. 28: 0 → 1
Char. 106: 0 → 1
Char. 239: 0 → 1

**Archosauriformes:**
Char. 32: 0 → 1
Char. 94: 1 → 0
Char. 112: 1 → 0
Char. 123: 1 → 0
Char. 152: 0 → 1
Char. 154: 0 → 2
Char. 166: 1 → 0
Char. 171: 0 → 1
Char. 185: 0 → 1
Char. 204: 0 → 1
Char. 218: 0 → 3
Char. 242: 0 → 1

***Australothyris smithi*:**
Char. 23: 0 → 1
Char. 34: 0 → 1
Char. 55: 1 → 0
Char. 71: 1 → 0
Char. 85: 1 → 0
Char. 112: 0 → 1
Char. 129: 0 → 1

***Barasaurus besairiei*:**
Char. 33: 1 → 0
Char. 75: 1 → 0
Char. 216: 0 → 1

***Bashkyroleter bashkyricus*:**
Char. 275: 1 → 0

***Bashkyroleter mesensis*:**
Char. 169: 1 → 0

***Belebey vegrandis*:**
Char. 154: 1 → 0

***Bradysaurus* spp.:**
Char. 19: 0 → 1
Char. 73: 0 → 1
Char. 79: 0 → 1
Char. 135: 1 → 0
Char. 249: 0 → 1

**Captorhinidae:**
Char. 3: 0 → 1
Char. 23: 0 → 1
Char. 25: 1 → 0
Char. 26: 0 → 1
Char. 73: 0 → 1
Char. 75: 0 → 1
Char. 83: 0 → 1
Char. 108: 1 → 0
Char. 183: 1 → 0
Char. 201: 1 → 0
Char. 203: 1 → 2
Char. 216: 1 → 0

**Caseidae:**
Char. 24: 0 → 1
Char. 25: 1 → 0
Char. 36: 0 → 1
Char. 38: 1 → 0
Char. 46: 1 → 0
Char. 50: 1 → 0
Char. 56: 0 → 1
Char. 85: 1 → 0
Char. 96: 1 → 0
Char. 170: 0 → 1
Char. 194: 0 → 1
Char. 273: 0 → 1
Char. 274: 0 → 1
Char. 278: 3 → 2

***Claudiosaurus germaini*:**
Char. 24: 0 → 1
Char. 34: 0 → 1
Char. 36: 0 → 1
Char. 64: 0 → 1
Char. 105: 0 → 1
Char. 130: 0 → 1
Char. 144: 1 → 0
Char. 187: 1 → 0
Char. 199: 0 → 1
Char. 203: 1 → 2
Char. 204: 0 → 1
Char. 220: 0 → 1
Char. 222: 1 → 0
Char. 234: 1 → 0

***Colobomycter pholeter*:**
Char. 21: 0 → 1
Char. 25: 1 → 0
Char. 84: 1 → 0
Char. 154: 1 → 0
Char. 167: 0 → 1
Char. 267: 0 → 1

***Delorhynchus cifelli*:**
Char. 18: 0 → 1
Char. 20: 0 → 1
Char. 21: 0 → 1
Char. 24: 0 → 1
Char. 26: 0 → 1
Char. 28: 0 → 1
Char. 33: 0 → 2
Char. 39: 0 → 1
Char. 52: 0 → 1
Char. 100: 0 → 1
Char. 111: 0 → 1
Char. 116: 1 → 0
Char. 117: 0 → 1
Char. 156: 1 → 0
Char. 167: 0 → 1
Char. 180: 0 → 1
Char. 189: 0 → 1
Char. 191: 0 → 1
Char. 192: 0 → 1
Char. 267: 0 → 1

**Diadectomorpha:**
Char. 0: 0 → 1
Char. 70: 0 → 1
Char. 75: 0 → 1
Char. 122: 0 → 1
Char. 123: 1 → 0
Char. 146: 1 → 0
Char. 275: 1 → 0
Char. 278: 3 → 0

***Emeroleter levis*:**
Char. 51: 1 → 0

**Eosauropterygia:**
Char. 159: 1 → 0
Char. 166: 1 → 0
Char. 168: 0 → 1
Char. 174: 0 → 1
Char. 194: 0 → 2
Char. 272: 1 → 0

***Eudibamus cursoris*:**
Char. 154: 1 → 2

***Feeserpeton oklahomensis*:**
Char. 51: 0 → 1
Char. 70: 0 → 1
Char. 157: 0 → 1
Char. 158: 0 → 1

***Hovasaurus boulei*:**
Char. 41: 0 → 1
Char. 55: 1 → 0
Char. 60: 1 → 0
Char. 72: 2 → 1
Char. 77: 0 → 2
Char. 78: 1 → 0
Char. 79: 0 → 1
Char. 93: 1 → 0
Char. 113: 0 → 1
Char. 138: 0 → 1
Char. 146: 1 → 0
Char. 154: 0 → 1
Char. 204: 0 → 2
Char. 206: 0 → 2
Char. 220: 0 → 1
Char. 278: 0 → 3

**Kuehneosauridae:**
Char. 7: 0 → 1
Char. 24: 0 → 1
Char. 34: 0 → 1
Char. 35: 0 → 1
Char. 36: 0 → 1
Char. 79: 0 → 2
Char. 82: 0 → 1
Char. 98: 1 → 0
Char. 113: 0 → 1
Char. 128: 0 → 1
Char. 159: 1 → 0
Char. 165: 0 → 1
Char. 168: 0 → 1
Char. 185: 0 → 1
Char. 206: 0 → 2
Char. 245: 0 → 1
Char. 278: 0 → 3

***Lanthanosuchus watsoni*:**
Char. 25: 1 → 0
Char. 51: 0 → 1
Char. 76: 0 → 1
Char. 86: 0 → 1
Char. 95: 0 → 1
Char. 98: 1 → 0
Char. 110: 0 → 1
Char. 113: 0 → 1
Char. 114: 0 → 1
Char. 137: 0 → 1
Char. 138: 0 → 1
Char. 140: 0 → 2
Char. 144: 1 → 0
Char. 154: 1 → 2
Char. 192: 0 → 1

***Macroleter poezicus*:**
Char. 52: 0 → 1
Char. 84: 1 → 0
Char. 87: 0 → 1
Char. 140: 0 → 1
Char. 146: 1 → 0
Char. 169: 1 → 0
Char. 235: 0 → 1

***Mesosaurus* spp.:**
Char. 0: 0 → 1
Char. 2: 0 → 1
Char. 5: 1 → 0
Char. 6: 0 → 1
Char. 8: 0 → 1
Char. 9: 0 → 1
Char. 13: 0 → 1
Char. 19: 0 → 1
Char. 23: 0 → 1
Char. 26: 0 → 1
Char. 29: 1 → 0
Char. 33: 0 → 1
Char. 38: 1 → 0
Char. 41: 0 → 1
Char. 50: 1 → 0
Char. 67: 0 → 1
Char. 76: 0 → 1
Char. 83: 0 → 1

Char. 84: 1 → 0
Char. 85: 1 → 0
Char. 94: 0 → 1
Char. 107: 0 → 1
Char. 109: 0 → 1
Char. 111: 0 → 1
Char. 115: 0 → 1
Char. 146: 1 → 0
Char. 147: 1 → 0
Char. 148: 0 → 1
Char. 149: 1 → 0
Char. 164: 0 → 1
Char. 167: 0 → 1
Char. 176: 0 → 1
Char. 183: 1 → 0
Char. 184: 0 → 1
Char. 199: 0 → 1
Char. 202: 0 → 1
Char. 206: 0 → 1
Char. 207: 0 → 1
Char. 209: 0 → 1
Char. 217: 0 → 1
Char. 219: 0 → 1
Char. 220: 0 → 1
Char. 231: 0 → 1
Char. 260: 2 → 0
Char. 272: 2 → 0
Char. 278: 3 → 0

***Microleter mckinzieorum*:**
Char. 0: 0 → 1
Char. 25: 1 → 0
Char. 36: 0 → 1
Char. 39: 0 → 1
Char. 51: 0 → 2
Char. 56: 0 → 1
Char. 70: 0 → 1
Char. 94: 0 → 1
Char. 106: 0 → 1
Char. 276: 0 → 1

**Millerettidae:**
Char. 5: 1 → 0
Char. 24: 0 → 1
Char. 25: 1 → 0
Char. 44: 1 → 0
Char. 56: 0 → 1
Char. 57: 1 → 0
Char. 66: 0 → 2
Char. 78: 1 → 0
Char. 80: 1 → 0
Char. 84: 1 → 2
Char. 88: 1 → 0
Char. 96: 1 → 0
Char. 117: 0 → 1
Char. 119: 0 → 1
Char. 121: 0 → 1
Char. 124: 0 → 1
Char. 127: 0 → 1
Char. 135: 0 → 1
Char. 145: 0 → 1
Char. 147: 1 → 0
Char. 202: 0 → 1
Char. 211: 0 → 1

Char. 230: 0 → 1
Char. 234: 0 → 1
Char. 248: 0 → 1
Char. 252: 0 → 1
Char. 253: 0 → 1

***Nycteroleter ineptus*:**
Char. 278: 0 → 3

***Nyctiphruretus acudens*:**
Char. 21: 0 → 1
Char. 33: 1 → 2
Char. 41: 0 → 1
Char. 81: 1 → 0
Char. 84: 1 → 2
Char. 94: 0 → 1
Char. 167: 0 → 1
Char. 224: 0 → 1
Char. 266: 0 → 1
Char. 272: 2 → 1
Char. 276: 0 → 1

***Orovenator mayorum*:**
Char. 8: 0 → 1
Char. 24: 0 → 1
Char. 36: 0 → 1
Char. 160: 0 → 1
Char. 165: 0 → 1

***Owenetta* spp.:**
Char. 169: 1 → 0

***Paleothyris acadiana*:**
Char. 66: 1 → 2
Char. 102: 0 → 1
Char. 146: 1 → 0
Char. 239: 0 → 1

***Placodus* spp.:**
Char. 0: 1 → 2
Char. 9: 0 → 1
Char. 12: 0 → 1
Char. 13: 0 → 1
Char. 19: 0 → 1
Char. 31: 0 → 1
Char. 46: 1 → 0
Char. 57: 0 → 1
Char. 78: 1 → 0
Char. 93: 1 → 0
Char. 102: 1 → 2
Char. 109: 1 → 0
Char. 140: 1 → 0
Char. 163: 1 → 0
Char. 164: 0 → 1

***Procolophon* spp.:**
Char. 41: 0 → 1
Char. 69: 0 → 1
Char. 79: 0 → 1
Char. 88: 0 → 1
Char. 117: 1 → 0
Char. 149: 1 → 0
Char. 180: 0 → 1
Char. 237: 0 → 1
Char. 238: 0 → 1

Char. 272: 2 → 1
Char. 278: 3 → 0

***Prolacerta broomi*:**
Char. 58: 1 → 0
Char. 66: 1 → 0
Char. 67: 1 → 0
Char. 80: 1 → 0
Char. 139: 1 → 0
Char. 147: 1 → 0
Char. 192: 1 → 0
Char. 203: 1 → 2
Char. 206: 0 → 12

***Rhipaeosaurus* spp.:**
Char. 172: 0 → 1
Char. 277: 0 → 1

**Rhynchocephalia:**
Char. 0: 1 → 2
Char. 24: 0 → 1
Char. 75: 1 → 0
Char. 77: 0 → 1
Char. 94: 1 → 0
Char. 106: 1 → 0
Char. 117: 12 → 0
Char. 139: 1 → 0
Char. 167: 1 → 0
Char. 205: 1 → 0

**Rhynchosauria:**
Char. 0: 1 → 0
Char. 7: 0 → 1
Char. 9: 0 → 1
Char. 63: 1 → 0
Char. 68: 0 → 1
Char. 99: 1 → 0
Char. 150: 1 → 0
Char. 160: 0 → 1
Char. 161: 0 → 1
Char. 171: 0 → 2
Char. 241: 0 → 1

***Scutosaurus* spp.:**
Char. 175: 0 → 1
Char. 218: 0 → 1
Char. 243: 0 → 2
Char. 244: 0 → 1
Char. 251: 0 → 1

***Sinosaurosphargis yunguiensis*:**
Char. 8: 0 → 1
Char. 30: 0 → 1
Char. 53: 0 → 1
Char. 82: 0 → 1
Char. 89: 1 → 0
Char. 127: 1 → 0
Char. 150: 1 → 0
Char. 154: 0 → 2
Char. 167: 1 → 0
Char. 253: 0 → 1
Char. 255: 0 → 1

**Squamata:**
Char. 45: 0 → 1
Char. 79: 0 → 2
Char. 80: 1 → 0
Char. 82: 0 → 1
Char. 92: 1 → 0
Char. 109: 1 → 0
Char. 160: 0 → 1
Char. 245: 0 → 1

***Trilophosaurus buettneri*:**
Char. 5: 1 → 0
Char. 11: 0 → 1
Char. 55: 1 → 0
Char. 93: 1 → 0
Char. 104: 0 → 1
Char. 113: 0 → 1
Char. 122: 0 → 1
Char. 123: 1 → 0
Char. 136: 1 → 0
Char. 144: 1 → 0
Char. 154: 0 → 1
Char. 157: 0 → 1
Char. 159: 1 → 0
Char. 177: 0 → 12
Char. 194: 0 → 1
Char. 203: 1 → 2
Char. 207: 1 → 0
Char. 208: 1 → 0
Char. 272: 1 → 0

***Youngina capensis*:**
Char. 21: 0 → 1
Char. 75: 0 → 1
Char. 211: 0 → 1
Char. 231: 0 → 1
Char. 239: 0 → 1

**Node 50:**
Char. 61: 1 → 0
Char. 62: 1 → 0
Char. 78: 1 → 0
Char. 193: 1 → 0
Char. 194: 0 → 2
Char. 214: 1 → 0
Char. 219: 1 → 0
Char. 220: 0 → 1
Char. 241: 0 → 1
Char. 246: 0 → 1
Char. 247: 0 → 1
Char. 254: 0 → 1
Char. 255: 0 → 1
Char. 256: 0 → 1
Char. 268: 0 → 1
Char. 269: 0 → 1

**Node 51:**
Char. 21: 0 → 1
Char. 27: 1 → 0
Char. 75: 0 → 1
Char. 147: 0 → 1

**Node 52:**
Char. 58: 0 → 1
Char. 61: 0 → 1

Char. 66: 0 → 1
Char. 69: 0 → 1
Char. 150: 0 → 1
Char. 167: 0 → 1
Char. 176: 0 → 1
Char. 184: 0 → 1
Char. 205: 0 → 1
Char. 210: 0 → 1

**Node 53:**
Char. 23: 0 → 1
Char. 70: 0 → 1
Char. 73: 0 → 1
Char. 94: 0 → 1
Char. 106: 0 → 1
Char. 131: 0 → 1

**Node 54:**
Char. 33: 0 → 1
Char. 135: 0 → 1
Char. 159: 0 → 1
Char. 267: 0 → 1
Char. 278: 1 → 0

**Node 55:**
Char. 20: 0 → 1
Char. 62: 0 → 1

**Node 56:**
Char. 0: 0 → 1
Char. 57: 1 → 0
Char. 89: 0 → 1
Char. 112: 0 → 1
Char. 120: 0 → 1
Char. 192: 0 → 1
Char. 193: 0 → 1
Char. 222: 0 → 1
Char. 224: 0 → 1
Char. 234: 0 → 1
Char. 266: 0 → 1
Char. 278: 3 → 1

**Node 57:**
Char. 29: 1 → 0
Char. 59: 0 → 1
Char. 60: 0 → 1
Char. 67: 0 → 1
Char. 72: 1 → 2
Char. 84: 1 → 0
Char. 111: 0 → 1
Char. 119: 0 → 1
Char. 147: 1 → 0
Char. 154: 1 → 0

**Node 58:**
Char. 5: 0 → 1
Char. 29: 0 → 1
Char. 40: 0 → 2
Char. 74: 0 → 1
Char. 80: 0 → 1
Char. 88: 0 → 1
Char. 116: 0 → 1
Char. 147: 0 → 1
Char. 149: 0 → 1
Char. 169: 0 → 1

Char. 201: 0 → 1
Char. 203: 0 → 1
Char. 235: 0 → 1
Char. 276: 1 → 0

**Node 59:**
Char. 81: 0 → 1
Char. 97: 0 → 1

**Node 60:**
Char. 55: 0 → 1
Char. 111: 1 → 0
Char. 135: 1 → 0
Char. 207: 1 → 0

**Node 61:**
Char. 15: 1 → 0
Char. 20: 1 → 0
Char. 25: 0 → 1
Char. 33: 2 → 0
Char. 76: 1 → 0
Char. 94: 1 → 0
Char. 124: 1 → 0
Char. 127: 1 → 0
Char. 145: 1 → 0
Char. 147: 1 → 0
Char. 149: 1 → 0
Char. 174: 1 → 0
Char. 180: 1 → 0
Char. 188: 1 → 0
Char. 220: 1 → 0
Char. 221: 1 → 0
Char. 231: 1 → 0
Char. 247: 1 → 0
Char. 251: 1 → 0
Char. 252: 1 → 0
Char. 253: 2 → 0
Char. 277: 1 → 0

**Node 62:**
Char. 19: 0 → 1
Char. 21: 1 → 0
Char. 92: 1 → 0

**Node 63:**
Char. 4: 0 → 1
Char. 29: 0 → 1
Char. 210: 1 → 0
Char. 213: 0 → 1
Char. 226: 01 → 2

Char. 228: 0 → 1
Char. 275: 0 → 1

**Node 64:**
Char. 24: 0 → 1
Char. 57: 1 → 0
Char. 79: 0 → 1
Char. 83: 0 → 1
Char. 110: 0 → 1
Char. 132: 1 → 0

**Node 65:**
Char. 73: 0 → 1
Char. 131: 1 → 0
Char. 205: 0 → 1
Char. 276: 0 → 1

**Node 66:**
Char. 18: 0 → 1
Char. 37: 0 → 1
Char. 107: 0 → 1
Char. 118: 1 → 0
Char. 125: 1 → 0
Char. 150: 0 → 1

**Node 67:**
Char. 23: 0 → 1
Char. 71: 1 → 0
Char. 102: 0 → 1
Char. 103: 0 → 1
Char. 106: 0 → 1
Char. 167: 0 → 1
Char. 214: 0 → 1
Char. 216: 1 → 0
Char. 235: 0 → 2
Char. 241: 0 → 1

**Node 68:**
Char. 207: 0 → 1

**Node 69:**
Char. 226: 0 → 1

**Node 70:**
Char. 20: 0 → 1
Char. 33: 0 → 1
Char. 44: 1 → 0
Char. 70: 0 → 1
Char. 80: 1 → 0
Char. 117: 0 → 1
Char. 118: 0 → 1

Char. 119: 0 → 1
Char. 125: 0 → 1
Char. 194: 0 → 1
Char. 201: 1 → 0
Char. 211: 0 → 1

**Node 71:**
Char. 38: 1 → 2
Char. 39: 0 → 1
Char. 49: 0 → 1
Char. 66: 0 → 12
Char. 76: 0 → 1
Char. 85: 1 → 0
Char. 88: 1 → 0
Char. 95: 0 → 1
Char. 112: 0 → 1
Char. 148: 0 → 1
Char. 158: 0 → 1
Char. 183: 1 → 2
Char. 192: 0 → 1

**Node 72:**
Char. 87: 0 → 1

**Node 73:**
Char. 25: 1 → 0
Char. 76: 1 → 0

**Node 74:**
Char. 79: 0 → 1
Char. 93: 1 → 0
Char. 133: 0 → 1

**Node 75:**
Char. 85: 0 → 1
Char. 147: 1 → 2
Char. 154: 1 → 0
Char. 278: 3 → 0

**Node 76:**
Char. 50: 1 → 0
Char. 58: 0 → 1
Char. 59: 0 → 1
Char. 60: 0 → 1
Char. 72: 1 → 2
Char. 104: 0 → 1
Char. 105: 0 → 1
Char. 106: 0 → 2
Char. 107: 0 → 1
Char. 109: 0 → 1
Char. 110: 0 → 1

Char. 146: 1 → 0
Char. 155: 0 → 2

**Node 77:**
Char. 33: 1 → 0
Char. 38: 2 → 1
Char. 39: 1 → 0
Char. 42: 0 → 1
Char. 43: 0 → 1
Char. 46: 1 → 0
Char. 49: 1 → 0
Char. 52: 0 → 1
Char. 83: 01 → 2
Char. 84: 1 → 0
Char. 87: 0 → 1
Char. 93: 1 → 0
Char. 161: 0 → 1
Char. 163: 1 → 0
Char. 172: 0 → 2
Char. 174: 0 → 1
Char. 188: 0 → 1
Char. 189: 0 → 1
Char. 195: 0 → 1
Char. 212: 0 → 1
Char. 236: 0 → 1
Char. 238: 0 → 2
Char. 242: 0 → 1
Char. 274: 0 → 1
Char. 275: 1 → 0

**Node 78:**
Char. 44: 1 → 0
Char. 66: 0 → 1
Char. 78: 1 → 0
Char. 88: 1 → 0
Char. 93: 1 → 0
Char. 123: 1 → 0

**Node 79:**
Char. 20: 0 → 1
Char. 47: 0 → 1
Char. 83: 0 → 1
Char. 85: 1 → 0
Char. 107: 0 → 1
Char. 110: 0 → 1
Char. 169: 1 → 0
Char. 170: 0 → 1

**Node 80:**
Char. 2: 0 → 1
Char. 6: 0 → 1

Char. 98: 1 → 0
Char. 101: 0 → 1
Char. 113: 0 → 1
Char. 186: 0 → 1
Char. 193: 1 → 0
Char. 198: 0 → 1
Char. 206: 0 → 2
Char. 220: 0 → 1
Char. 231: 1 → 0
Char. 232: 1 → 0
Char. 234: 1 → 0
Char. 240: 1 → 0
Char. 266: 1 → 0

**Node 81:**
Char. 35: 0 → 1
Char. 68: 0 → 1
Char. 102: 0 → 1
Char. 103: 0 → 1
Char. 123: 1 → 0
Char. 229: 0 → 1
Char. 235: 1 → 2

**Node 82:**
Char. 17: 0 → 1
Char. 42: 0 → 1
Char. 251: 0 → 1
Char. 264: 0 → 1

**Node 83:**
Char. 41: 0 → 1
Char. 61: 1 → 2
Char. 112: 1 → 0
Char. 128: 0 → 1
Char. 138: 0 → 1
Char. 146: 1 → 0
Char. 165: 0 → 1
Char. 192: 1 → 2
Char. 227: 0 → 1
Char. 233: 0 → 1

**Node 84:**
Char. 61: 1 → 3
Char. 90: 0 → 1
Char. 91: 0 → 1
Char. 109: 1 → 0
Char. 209: 1 → 0

**ANALYSIS 30**
**(NO *CANDELARIA BARBOURI*, *PAPPOCHELYS ROSINAE*, AND *EUNOTOSAURUS AFRICANUS*)**

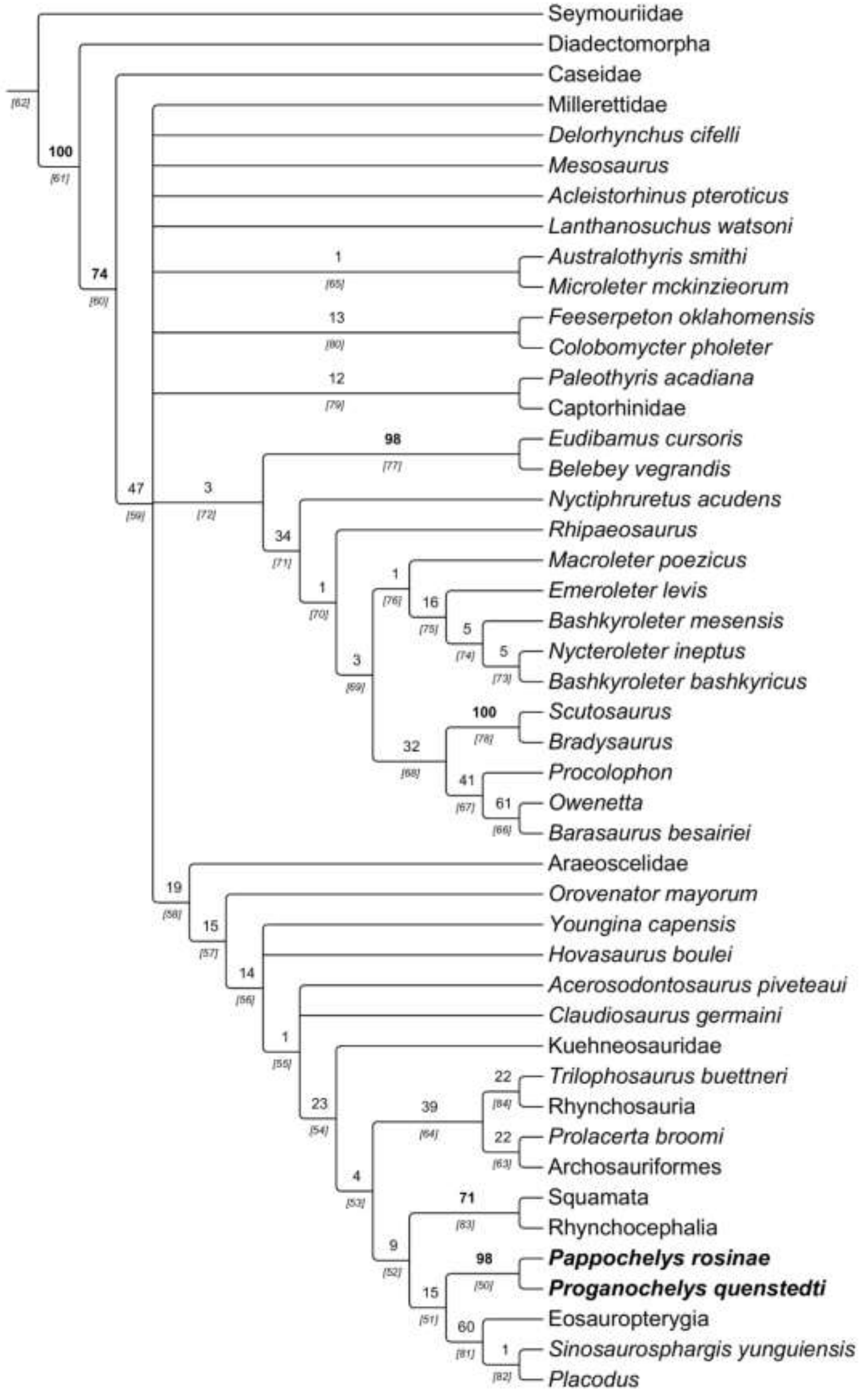

***Proganochelys quenstedti*:**
Char. 8: 0 → 1
Char. 11: 0 → 1
Char. 43: 1 → 0
Char. 108: 1 → 0
Char. 128: 0 → 1
Char. 175: 0 → 1
Char. 202: 1 → 0
Char. 207: 1 → 0
Char. 209: 1 → 0
Char. 235: 2 → 0
Char. 244: 0 → 1
Char. 248: 0 → 1
Char. 250: 0 → 1
Char. 262: 0 → 1

**Seymouriidae:**
Char. 51: 0 → 2
Char. 54: 1 → 0
Char. 71: 1 → 0
Char. 85: 1 → 0
Char. 99: 1 → 0
Char. 107: 0 → 1
Char. 140: 0 → 2
Char. 225: 1 → 0

***Odontochelys semitestacea*:**
Char. 109: 1 → 0
Char. 112: 1 → 0
Char. 124: 0 → 1
Char. 126: 1 → 0
Char. 252: 0 → 1

***Acerosodontosaurus piveteaui*:**
Char. 78: 1 → 0
Char. 128: 0 → 1
Char. 155: 0 → 1
Char. 206: 0 → 2
Char. 267: 1 → 0
Char. 278: 0 → 1

***Acleistorhinus pteroticus*:**
Char. 20: 0 → 1
Char. 21: 0 → 1
Char. 30: 0 → 1
Char. 33: 0 → 1
Char. 47: 0 → 1
Char. 55: 1 → 0
Char. 56: 0 → 1
Char. 64: 0 → 1
Char. 70: 0 → 1
Char. 79: 0 → 1
Char. 95: 0 → 1
Char. 110: 0 → 1
Char. 113: 0 → 1
Char. 114: 0 → 1
Char. 131: 0 → 1
Char. 137: 0 → 1
Char. 140: 0 → 2
Char. 146: 1 → 0
Char. 159: 0 → 1
Char. 169: 1 → 0
Char. 170: 0 → 1
Char. 278: 3 → 1

**Araeoscelidae:**
Char. 5: 1 → 0
Char. 23: 0 → 1
Char. 28: 0 → 1
Char. 106: 0 → 1
Char. 116: 1 → 0
Char. 169: 1 → 0
Char. 170: 0 → 1
Char. 197: 0 → 1
Char. 239: 0 → 1

**Archosauriformes:**
Char. 32: 0 → 1
Char. 94: 1 → 0
Char. 112: 1 → 0
Char. 152: 0 → 1
Char. 154: 0 → 2
Char. 166: 1 → 0
Char. 171: 0 → 1
Char. 185: 0 → 1
Char. 204: 0 → 1
Char. 218: 0 → 3
Char. 242: 0 → 1

***Australothyris smithi*:**
Char. 23: 0 → 1
Char. 34: 0 → 1
Char. 55: 1 → 0
Char. 71: 1 → 0
Char. 85: 1 → 0
Char. 112: 0 → 1
Char. 129: 0 → 1
Char. 131: 0 → 1

***Barasaurus besairiei*:**
Char. 33: 1 → 0
Char. 75: 1 → 0
Char. 216: 0 → 1

***Bashkyroleter bashkyricus*:**
Char. 275: 1 → 0

***Bashkyroleter mesensis*:**
Char. 169: 1 → 0

***Belebey vegrandis*:**
Char. 154: 1 → 0

***Bradysaurus* spp.:**
Char. 19: 0 → 1
Char. 73: 0 → 1
Char. 79: 0 → 1
Char. 135: 1 → 0
Char. 249: 0 → 1

**Captorhinidae:**
Char. 3: 0 → 1
Char. 23: 0 → 1
Char. 25: 1 → 0
Char. 26: 0 → 1
Char. 73: 0 → 1
Char. 75: 0 → 1
Char. 83: 0 → 1
Char. 108: 1 → 0
Char. 183: 1 → 0
Char. 201: 1 → 0
Char. 203: 1 → 2
Char. 216: 1 → 0

**Caseidae:**
Char. 24: 0 → 1
Char. 25: 1 → 0
Char. 36: 0 → 1
Char. 38: 1 → 0
Char. 46: 1 → 0
Char. 56: 0 → 1
Char. 85: 1 → 0
Char. 96: 1 → 0
Char. 170: 0 → 1
Char. 194: 0 → 1
Char. 273: 0 → 1
Char. 274: 0 → 1
Char. 278: 3 → 2

***Claudiosaurus germaini*:**
Char. 24: 0 → 1
Char. 34: 0 → 1
Char. 36: 0 → 1
Char. 64: 0 → 1
Char. 105: 0 → 1
Char. 130: 0 → 1
Char. 144: 1 → 0
Char. 187: 1 → 0
Char. 199: 0 → 1
Char. 203: 1 → 2
Char. 204: 0 → 1
Char. 220: 0 → 1
Char. 222: 1 → 0
Char. 234: 1 → 0

***Colobomycter pholeter*:**
Char. 21: 0 → 1
Char. 25: 1 → 0
Char. 84: 1 → 0
Char. 154: 1 → 0
Char. 167: 0 → 1
Char. 267: 0 → 1

***Delorhynchus cifelli*:**
Char. 18: 0 → 1
Char. 20: 0 → 1
Char. 21: 0 → 1
Char. 24: 0 → 1
Char. 26: 0 → 1
Char. 28: 0 → 1
Char. 33: 0 → 2
Char. 39: 0 → 1
Char. 52: 0 → 1
Char. 100: 0 → 1
Char. 111: 0 → 1
Char. 116: 1 → 0
Char. 117: 0 → 1
Char. 131: 0 → 1
Char. 156: 1 → 0
Char. 159: 0 → 1
Char. 167: 0 → 1
Char. 180: 0 → 1
Char. 189: 0 → 1
Char. 191: 0 → 1
Char. 192: 0 → 1
Char. 204: 0 → 2
Char. 267: 0 → 1

**Diadectomorpha:**
Char. 0: 0 → 1
Char. 70: 0 → 1
Char. 75: 0 → 1
Char. 122: 0 → 1
Char. 123: 1 → 0
Char. 146: 1 → 0
Char. 275: 1 → 0
Char. 278: 3 → 0

***Emeroleter levis*:**
Char. 51: 1 → 0

**Eosauropterygia:**
Char. 158: 1 → 0
Char. 159: 1 → 0
Char. 166: 1 → 0
Char. 168: 0 → 1
Char. 178: 0 → 1
Char. 272: 1 → 0

***Eudibamus cursoris*:**
Char. 154: 1 → 2

***Feeserpeton oklahomensis*:**
Char. 51: 0 → 1
Char. 70: 0 → 1
Char. 157: 0 → 1
Char. 158: 0 → 1

***Hovasaurus boulei*:**
Char. 41: 0 → 1
Char. 55: 1 → 0
Char. 60: 1 → 0
Char. 72: 2 → 1
Char. 77: 0 → 2
Char. 78: 1 → 0
Char. 79: 0 → 1
Char. 93: 1 → 0
Char. 113: 0 → 1
Char. 138: 0 → 1
Char. 146: 1 → 0
Char. 154: 0 → 1
Char. 204: 0 → 2
Char. 206: 0 → 2
Char. 220: 0 → 1
Char. 278: 0 → 3

**Kuehneosauridae:**
Char. 7: 0 → 1
Char. 24: 0 → 1
Char. 34: 0 → 1
Char. 36: 0 → 1
Char. 82: 0 → 1
Char. 98: 1 → 0
Char. 108: 1 → 0
Char. 113: 0 → 1
Char. 128: 0 → 1
Char. 159: 1 → 0
Char. 181: 0 → 1
Char. 185: 0 → 1
Char. 206: 0 → 2
Char. 245: 0 → 1
Char. 278: 0 → 3

***Lanthanosuchus watsoni*:**
Char. 25: 1 → 0
Char. 51: 0 → 1
Char. 76: 0 → 1
Char. 86: 0 → 1
Char. 95: 0 → 1
Char. 98: 1 → 0
Char. 110: 0 → 1
Char. 113: 0 → 1
Char. 114: 0 → 1
Char. 131: 0 → 1
Char. 137: 0 → 1
Char. 138: 0 → 1
Char. 140: 0 → 2
Char. 144: 1 → 0
Char. 154: 1 → 2
Char. 192: 0 → 1

***Macroleter poezicus*:**
Char. 52: 0 → 1
Char. 84: 1 → 0
Char. 87: 0 → 1
Char. 140: 0 → 1
Char. 146: 1 → 0
Char. 169: 1 → 0
Char. 235: 0 → 1

***Mesosaurus* spp.:**
Char. 0: 0 → 1
Char. 2: 0 → 1
Char. 5: 1 → 0
Char. 6: 0 → 1
Char. 8: 0 → 1
Char. 9: 0 → 1
Char. 13: 0 → 1
Char. 19: 0 → 1
Char. 23: 0 → 1
Char. 26: 0 → 1
Char. 29: 1 → 0
Char. 33: 0 → 1
Char. 38: 1 → 0
Char. 41: 0 → 1
Char. 50: 1 → 0
Char. 67: 0 → 1
Char. 76: 0 → 1
Char. 83: 0 → 1
Char. 84: 1 → 0
Char. 85: 1 → 0
Char. 94: 0 → 1
Char. 107: 0 → 1
Char. 109: 0 → 1
Char. 111: 0 → 1
Char. 115: 0 → 1
Char. 146: 1 → 0
Char. 148: 0 → 1
Char. 149: 1 → 0
Char. 164: 0 → 1
Char. 167: 0 → 1

Char. 176: 0 → 1
Char. 183: 1 → 0
Char. 184: 0 → 1
Char. 199: 0 → 1
Char. 202: 0 → 1
Char. 204: 0 → 2
Char. 206: 0 → 1
Char. 207: 0 → 1
Char. 209: 0 → 1
Char. 217: 0 → 1
Char. 219: 0 → 1
Char. 220: 0 → 1
Char. 231: 0 → 1
Char. 272: 2 → 0
Char. 278: 3 → 0

***Microleter mckinzieorum*:**
Char. 0: 0 → 1
Char. 25: 1 → 0
Char. 36: 0 → 1
Char. 39: 0 → 1
Char. 51: 0 → 2
Char. 56: 0 → 1
Char. 70: 0 → 1
Char. 94: 0 → 1
Char. 106: 0 → 1
Char. 159: 0 → 1
Char. 276: 0 → 1

**Millerettidae:**
Char. 5: 1 → 0
Char. 24: 0 → 1
Char. 25: 1 → 0
Char. 44: 1 → 0
Char. 56: 0 → 1
Char. 57: 1 → 0
Char. 66: 0 → 2
Char. 78: 1 → 0
Char. 80: 1 → 0
Char. 84: 1 → 2
Char. 88: 1 → 0
Char. 96: 1 → 0
Char. 117: 0 → 1
Char. 121: 0 → 1
Char. 124: 0 → 1
Char. 127: 0 → 1
Char. 135: 0 → 1
Char. 145: 0 → 1
Char. 202: 0 → 1
Char. 211: 0 → 1
Char. 230: 0 → 1
Char. 234: 0 → 1
Char. 248: 0 → 1
Char. 252: 0 → 1
Char. 253: 0 → 1

***Nycteroleter ineptus*:**
Char. 278: 0 → 3

***Nyctiphruretus acudens*:**
Char. 21: 0 → 1
Char. 33: 1 → 2
Char. 41: 0 → 1
Char. 81: 1 → 0
Char. 84: 1 → 2
Char. 94: 0 → 1
Char. 167: 0 → 1
Char. 224: 0 → 1
Char. 266: 0 → 1
Char. 272: 2 → 1
Char. 276: 0 → 1

***Orovenator mayorum*:**
Char. 8: 0 → 1
Char. 24: 0 → 1
Char. 36: 0 → 1
Char. 160: 0 → 1
Char. 165: 0 → 1

***Owenetta* spp.:**
Char. 169: 1 → 0

***Paleothyris acadiana*:**
Char. 38: 1 → 0
Char. 50: 1 → 0
Char. 66: 1 → 2
Char. 102: 0 → 1
Char. 146: 1 → 0
Char. 180: 0 → 1
Char. 237: 0 → 1
Char. 239: 0 → 1

***Placodus* spp.:**
Char. 12: 0 → 1
Char. 13: 0 → 1
Char. 19: 0 → 1
Char. 31: 0 → 1
Char. 46: 1 → 0
Char. 57: 0 → 1
Char. 93: 1 → 0
Char. 102: 1 → 2
Char. 109: 1 → 0
Char. 140: 1 → 0
Char. 163: 1 → 0
Char. 164: 0 → 1

***Procolophon* spp.:**
Char. 41: 0 → 1
Char. 69: 0 → 1
Char. 79: 0 → 1
Char. 88: 0 → 1
Char. 117: 1 → 0
Char. 149: 1 → 0
Char. 180: 0 → 1
Char. 204: 0 → 1
Char. 237: 0 → 1
Char. 238: 0 → 1
Char. 272: 2 → 1
Char. 278: 3 → 0

***Prolacerta broomi*:**
Char. 58: 1 → 0
Char. 66: 1 → 0
Char. 67: 1 → 0
Char. 80: 1 → 0
Char. 139: 1 → 0
Char. 147: 1 → 0
Char. 192: 1 → 0
Char. 203: 1 → 2
Char. 206: 0 → 12

***Rhipaeosaurus* spp.:**
Char. 172: 0 → 1
Char. 277: 0 → 1

**Rhynchocephalia:**
Char. 24: 0 → 1
Char. 81: 0 → 1
Char. 94: 1 → 0
Char. 104: 0 → 1
Char. 117: 12 → 0
Char. 139: 1 → 0
Char. 167: 1 → 0
Char. 205: 1 → 0

**Rhynchosauria:**
Char. 0: 1 → 0
Char. 7: 0 → 1
Char. 9: 0 → 1
Char. 68: 0 → 1
Char. 99: 1 → 0
Char. 150: 1 → 0
Char. 160: 0 → 1
Char. 161: 0 → 1
Char. 171: 0 → 2
Char. 223: 0 → 1
Char. 241: 0 → 1

***Scutosaurus* spp.:**
Char. 175: 0 → 1
Char. 218: 0 → 1
Char. 243: 0 → 2
Char. 244: 0 → 1
Char. 251: 0 → 1

***Sinosaurosphargis yunguiensis*:**
Char. 8: 0 → 1
Char. 23: 0 → 1
Char. 30: 0 → 1
Char. 53: 0 → 1
Char. 82: 0 → 1
Char. 89: 1 → 0
Char. 127: 1 → 0
Char. 150: 1 → 0
Char. 154: 0 → 2
Char. 167: 1 → 0
Char. 253: 0 → 1
Char. 255: 0 → 1

**Squamata:**
Char. 45: 0 → 1
Char. 80: 1 → 0
Char. 82: 0 → 1
Char. 92: 1 → 0
Char. 109: 1 → 0
Char. 122: 0 → 1
Char. 160: 0 → 1
Char. 245: 0 → 1

***Trilophosaurus buettneri*:**
Char. 5: 1 → 0
Char. 11: 0 → 1
Char. 55: 1 → 0
Char. 93: 1 → 0
Char. 104: 0 → 1
Char. 113: 0 → 1
Char. 122: 0 → 1
Char. 136: 1 → 0
Char. 144: 1 → 0
Char. 154: 0 → 1
Char. 157: 0 → 1
Char. 159: 1 → 0
Char. 177: 0 → 12
Char. 194: 0 → 1
Char. 203: 1 → 2
Char. 207: 1 → 0
Char. 208: 1 → 0
Char. 272: 1 → 0

***Youngina capensis*:**
Char. 5: 1 → 0
Char. 21: 0 → 1
Char. 75: 0 → 1
Char. 170: 0 → 1
Char. 211: 0 → 1
Char. 231: 0 → 1
Char. 239: 0 → 1

**Node 50:**
Char. 46: 1 → 0
Char. 50: 0 → 1
Char. 58: 1 → 0
Char. 61: 1 → 0
Char. 62: 1 → 0
Char. 65: 0 → 1
Char. 89: 1 → 0
Char. 93: 1 → 0
Char. 107: 1 → 0
Char. 150: 1 → 0
Char. 154: 0 → 1
Char. 167: 1 → 0
Char. 195: 0 → 2
Char. 201: 1 → 0
Char. 219: 1 → 0
Char. 241: 0 → 1
Char. 246: 0 → 2
Char. 247: 0 → 1
Char. 253: 0 → 2
Char. 254: 0 → 1
Char. 255: 0 → 1
Char. 256: 0 → 1
Char. 258: 0 → 1
Char. 259: 0 → 1
Char. 260: 1 → 2
Char. 265: 1 → 0
Char. 268: 0 → 1
Char. 269: 0 → 1
Char. 270: 0 → 1

**Node 51:**
Char. 161: 0 → 1
Char. 181: 0 → 1
Char. 189: 0 → 1
Char. 193: 1 → 0
Char. 198: 0 → 1
Char. 220: 0 → 1
Char. 239: 1 → 0
Char. 240: 1 → 0

**Node 52:**
Char. 33: 1 → 2
Char. 102: 0 → 1
Char. 158: 0 → 1
Char. 223: 0 → 1
Char. 235: 1 → 2

**Node 53:**
Char. 27: 1 → 0
Char. 48: 1 → 0
Char. 107: 0 → 1
Char. 140: 0 → 1
Char. 147: 0 → 1

**Node 54:**
Char. 58: 0 → 1
Char. 61: 0 → 1
Char. 66: 0 → 1
Char. 69: 0 → 1
Char. 150: 0 → 1
Char. 167: 0 → 1
Char. 176: 0 → 1
Char. 205: 0 → 1
Char. 239: 0 → 1

**Node 55:**
Char. 23: 0 → 1
Char. 70: 0 → 1
Char. 73: 0 → 1
Char. 94: 0 → 1
Char. 131: 0 → 1

**Node 56:**
Char. 33: 0 → 1
Char. 135: 0 → 1
Char. 159: 0 → 1
Char. 267: 0 → 1
Char. 278: 1 → 0

**Node 57:**
Char. 20: 0 → 1
Char. 62: 0 → 1

**Node 58:**
Char. 0: 0 → 1
Char. 27: 0 → 1
Char. 29: 1 → 0
Char. 38: 1 → 0
Char. 40: 2 → 0
Char. 57: 1 → 0
Char. 59: 0 → 1
Char. 60: 0 → 1
Char. 67: 0 → 1
Char. 72: 1 → 2
Char. 84: 1 → 0
Char. 89: 0 → 1
Char. 111: 0 → 1
Char. 112: 0 → 1
Char. 120: 0 → 1
Char. 154: 1 → 0
Char. 180: 0 → 1
Char. 192: 0 → 1

Char. 193: 0 → 1
Char. 222: 0 → 1
Char. 224: 0 → 1
Char. 234: 0 → 1
Char. 237: 0 → 1
Char. 266: 0 → 1
Char. 278: 3 → 1

**Node 59:**
Char. 5: 0 → 1
Char. 29: 0 → 1
Char. 40: 0 → 2
Char. 74: 0 → 1
Char. 88: 0 → 1
Char. 116: 0 → 1
Char. 132: 0 → 1
Char. 149: 0 → 1
Char. 169: 0 → 1
Char. 201: 0 → 1
Char. 203: 0 → 1
Char. 235: 0 → 1
Char. 276: 1 → 0

**Node 60:**
Char. 97: 0 → 1
Char. 144: 0 → 1

**Node 61:**
Char. 23: 1 → 0
Char. 55: 0 → 1
Char. 78: 0 → 1
Char. 111: 1 → 0
Char. 126: 1 → 0
Char. 135: 1 → 0

**Node 62:**
Char. 15: 1 → 0
Char. 20: 1 → 0
Char. 25: 0 → 1
Char. 33: 2 → 0
Char. 73: 1 → 0
Char. 89: 1 → 0
Char. 94: 1 → 0
Char. 127: 1 → 0
Char. 217: 1 → 0
Char. 220: 1 → 0
Char. 221: 1 → 0
Char. 247: 1 → 0
Char. 252: 1 → 0
Char. 277: 1 → 0

**Node 63:**
Char. 19: 0 → 1
Char. 92: 1 → 0

**Node 64:**
Char. 4: 0 → 1
Char. 29: 0 → 1
Char. 213: 0 → 1
Char. 228: 0 → 1
Char. 275: 0 → 1

**Node 65:**
Char. 24: 0 → 1
Char. 57: 1 → 0
Char. 79: 0 → 1
Char. 83: 0 → 1
Char. 110: 0 → 1
Char. 132: 1 → 0

**Node 66:**
Char. 73: 0 → 1
Char. 131: 1 → 0
Char. 205: 0 → 1
Char. 276: 0 → 1

**Node 67:**
Char. 18: 0 → 1
Char. 37: 0 → 1
Char. 107: 0 → 1
Char. 118: 1 → 0
Char. 125: 1 → 0
Char. 150: 0 → 1

**Node 68:**
Char. 23: 0 → 1
Char. 71: 1 → 0
Char. 102: 0 → 1
Char. 103: 0 → 1
Char. 106: 0 → 1
Char. 167: 0 → 1
Char. 214: 0 → 1
Char. 216: 1 → 0
Char. 235: 0 → 2
Char. 241: 0 → 1

**Node 69:**
Char. 207: 0 → 1

**Node 70:**
Char. 226: 0 → 1

**Node 71:**
Char. 20: 0 → 1
Char. 33: 0 → 1
Char. 44: 1 → 0
Char. 70: 0 → 1
Char. 80: 1 → 0
Char. 117: 0 → 1
Char. 118: 0 → 1
Char. 125: 0 → 1
Char. 131: 0 → 1
Char. 194: 0 → 1
Char. 201: 1 → 0
Char. 211: 0 → 1

**Node 72:**
Char. 38: 1 → 2
Char. 39: 0 → 1
Char. 49: 0 → 1
Char. 66: 0 → 12
Char. 76: 0 → 1
Char. 85: 1 → 0
Char. 88: 1 → 0
Char. 95: 0 → 1
Char. 112: 0 → 1
Char. 148: 0 → 1
Char. 158: 0 → 1
Char. 159: 0 → 1
Char. 183: 1 → 2
Char. 192: 0 → 1

**Node 73:**
Char. 87: 0 → 1

**Node 74:**
Char. 25: 1 → 0
Char. 76: 1 → 0

**Node 75:**
Char. 79: 0 → 1
Char. 93: 1 → 0
Char. 133: 0 → 1

**Node 76:**
Char. 85: 0 → 1
Char. 147: 1 → 2
Char. 154: 1 → 0
Char. 278: 3 → 0

**Node 77:**
Char. 50: 1 → 0
Char. 58: 0 → 1
Char. 59: 0 → 1
Char. 60: 0 → 1
Char. 72: 1 → 2
Char. 104: 0 → 1
Char. 105: 0 → 1
Char. 106: 0 → 2
Char. 107: 0 → 1
Char. 109: 0 → 1
Char. 110: 0 → 1
Char. 146: 1 → 0
Char. 155: 0 → 2

**Node 78:**
Char. 33: 1 → 0
Char. 38: 2 → 1
Char. 39: 1 → 0
Char. 42: 0 → 1
Char. 43: 0 → 1
Char. 46: 1 → 0
Char. 49: 1 → 0
Char. 52: 0 → 1
Char. 83: 01 → 2
Char. 84: 1 → 0
Char. 87: 0 → 1
Char. 93: 1 → 0
Char. 161: 0 → 1
Char. 163: 1 → 0
Char. 172: 0 → 2
Char. 174: 0 → 1
Char. 188: 0 → 1
Char. 189: 0 → 1
Char. 195: 0 → 1
Char. 204: 0 → 2
Char. 212: 0 → 1
Char. 236: 0 → 1
Char. 238: 0 → 2
Char. 242: 0 → 1
Char. 274: 0 → 1
Char. 275: 1 → 0

**Node 79:**
Char. 5: 1 → 0
Char. 29: 1 → 0
Char. 44: 1 → 0
Char. 59: 0 → 1
Char. 60: 0 → 1
Char. 66: 0 → 1
Char. 67: 0 → 1
Char. 72: 1 → 2
Char. 78: 1 → 0
Char. 84: 1 → 0
Char. 88: 1 → 0
Char. 93: 1 → 0
Char. 111: 0 → 1
Char. 123: 1 → 0
Char. 129: 0 → 1
Char. 154: 1 → 0
Char. 169: 1 → 0
Char. 170: 0 → 1
Char. 197: 0 → 1

**Node 80:**
Char. 20: 0 → 1
Char. 47: 0 → 1
Char. 83: 0 → 1
Char. 85: 1 → 0
Char. 107: 0 → 1
Char. 110: 0 → 1
Char. 169: 1 → 0
Char. 170: 0 → 1

**Node 81:**
Char. 2: 0 → 1
Char. 6: 0 → 1
Char. 81: 0 → 1
Char. 98: 1 → 0
Char. 101: 0 → 1
Char. 104: 0 → 1
Char. 113: 0 → 1
Char. 186: 0 → 1
Char. 231: 1 → 0
Char. 232: 1 → 0
Char. 234: 1 → 0
Char. 266: 1 → 0
Char. 267: 1 → 0

**Node 82:**
Char. 17: 0 → 1
Char. 264: 0 → 1

**Node 83:**
Char. 41: 0 → 1
Char. 61: 1 → 2
Char. 112: 1 → 0
Char. 128: 0 → 1
Char. 146: 1 → 0
Char. 192: 1 → 2
Char. 233: 0 → 1
Char. 267: 1 → 2

**Node 84:**
Char. 61: 1 → 3
Char. 90: 0 → 1
Char. 91: 0 → 1
Char. 109: 1 → 0
Char. 209: 1 → 0

## ANALYSIS 31
## (NO *CANDELARIA BARBOURI*, *PAPPOCHELYS ROSINAE*, AND *ODONTOCHELYS SEMITESTACEA*)

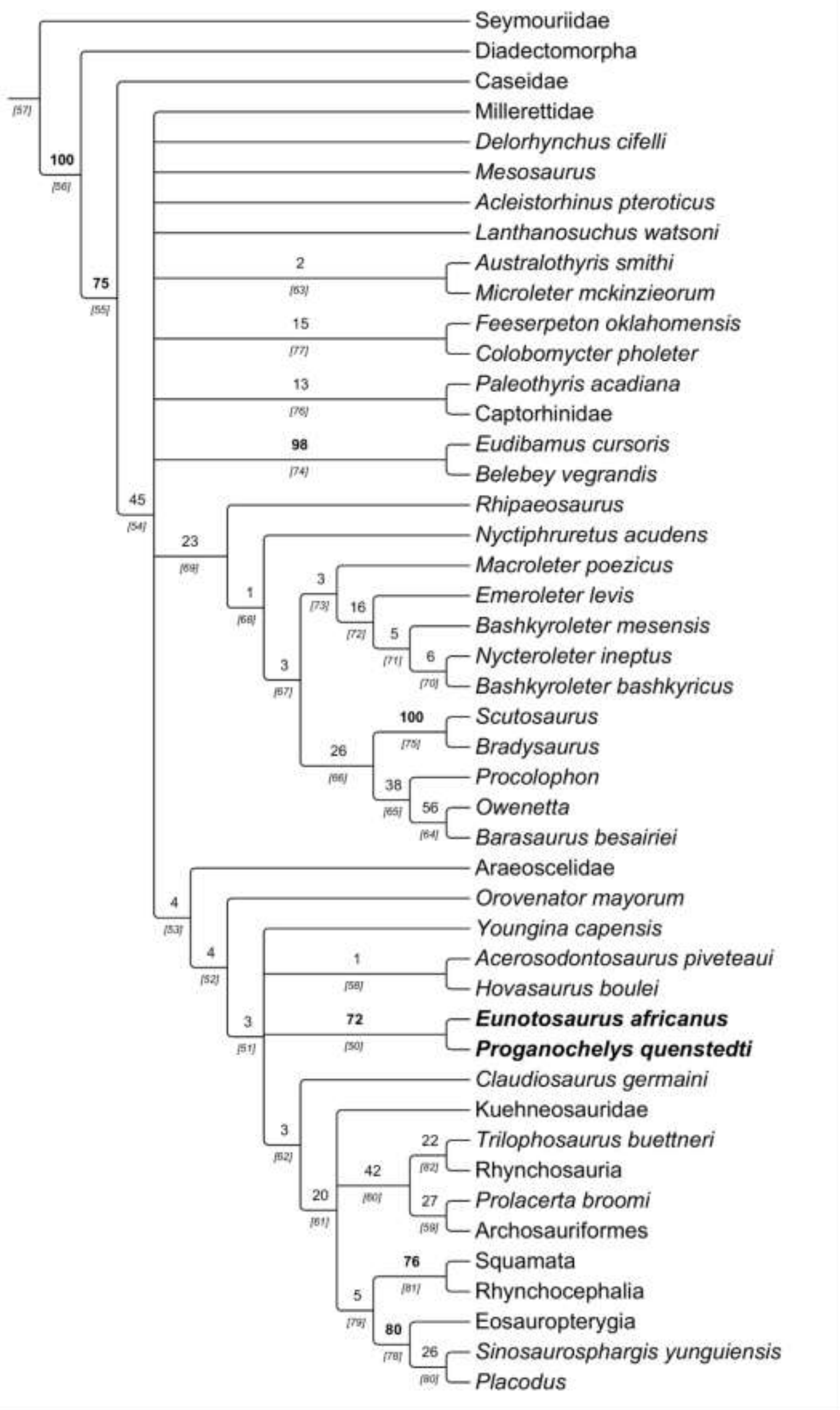

***Proganochelys quenstedti*:**
Char. 8: 0 → 1
Char. 9: 0 → 1
Char. 11: 0 → 1
Char. 26: 0 → 1
Char. 28: 0 → 1
Char. 39: 0 → 1
Char. 43: 1 → 0
Char. 46: 1 → 0
Char. 53: 0 → 1
Char. 63: 0 → 1
Char. 65: 0 → 1
Char. 69: 0 → 1
Char. 79: 0 → 2
Char. 88: 1 → 0
Char. 89: 1 → 0
Char. 93: 1 → 0
Char. 106: 0 → 1
Char. 108: 1 → 0
Char. 110: 0 → 1
Char. 115: 0 → 1
Char. 118: 0 → 1
Char. 121: 0 → 1
Char. 126: 0 → 1
Char. 128: 0 → 1
Char. 131: 0 → 1
Char. 138: 0 → 1
Char. 142: 1 → 0
Char. 160: 0 → 1
Char. 175: 0 → 1
Char. 176: 0 → 1
Char. 184: 0 → 1
Char. 195: 0 → 2
Char. 198: 0 → 1
Char. 200: 0 → 1
Char. 205: 0 → 1
Char. 210: 0 → 1
Char. 218: 0 → 1
Char. 223: 0 → 1
Char. 230: 0 → 1
Char. 232: 0 → 1
Char. 235: 1 → 0
Char. 238: 0 → 2
Char. 241: 0 → 1
Char. 246: 0 → 2
Char. 254: 0 → 1
Char. 255: 0 → 1
Char. 256: 0 → 1
Char. 258: 0 → 1
Char. 259: 0 → 1
Char. 262: 0 → 1
Char. 268: 0 → 1
Char. 269: 0 → 1
Char. 270: 0 → 1
Char. 271: 0 → 1
Char. 272: 2 → 1

**Seymouriidae:**
Char. 51: 0 → 2
Char. 54: 1 → 0
Char. 85: 1 → 0
Char. 99: 1 → 0
Char. 107: 0 → 1
Char. 225: 1 → 0

***Eunotosaurus africanus*:**
Char. 19: 0 → 1
Char. 55: 1 → 0
Char. 59: 1 → 0
Char. 60: 1 → 0
Char. 67: 1 → 0
Char. 72: 2 → 1
Char. 76: 0 → 1
Char. 97: 1 → 0
Char. 103: 0 → 1
Char. 109: 1 → 0
Char. 112: 1 → 0
Char. 124: 0 → 1
Char. 153: 1 → 0
Char. 191: 1 → 0
Char. 192: 1 → 0
Char. 211: 0 → 1
Char. 222: 1 → 0
Char. 237: 1 → 0
Char. 249: 0 → 2
Char. 252: 0 → 1
Char. 263: 0 → 1
Char. 273: 0 → 1
Char. 274: 0 → 1
Char. 275: 0 → 1
Char. 277: 0 → 1
Char. 278: 0 → 3

***Acerosodontosaurus piveteaui*:**
Char. 81: 1 → 0
Char. 127: 1 → 0
Char. 208: 0 → 1

***Acleistorhinus pteroticus*:**
Char. 20: 0 → 1
Char. 21: 0 → 1
Char. 30: 0 → 1
Char. 33: 0 → 1
Char. 47: 0 → 1
Char. 55: 1 → 0
Char. 56: 0 → 1
Char. 64: 0 → 1
Char. 70: 0 → 1
Char. 79: 0 → 1
Char. 95: 0 → 1
Char. 110: 0 → 1
Char. 113: 0 → 1
Char. 114: 0 → 1
Char. 131: 0 → 1
Char. 137: 0 → 1
Char. 140: 0 → 2
Char. 146: 1 → 0
Char. 169: 1 → 0
Char. 170: 0 → 1
Char. 278: 3 → 1

**Araeoscelidae:**
Char. 5: 1 → 0
Char. 23: 0 → 1
Char. 28: 0 → 1
Char. 106: 0 → 1
Char. 116: 1 → 0
Char. 117: 0 → 2
Char. 169: 1 → 0
Char. 170: 0 → 1
Char. 197: 0 → 1
Char. 239: 0 → 1

**Archosauriformes:**
Char. 32: 0 → 1
Char. 94: 1 → 0
Char. 112: 1 → 0
Char. 152: 0 → 1
Char. 154: 0 → 2
Char. 166: 1 → 0
Char. 171: 0 → 1
Char. 185: 0 → 1
Char. 204: 0 → 1
Char. 218: 0 → 3
Char. 242: 0 → 1

***Australothyris smithi*:**
Char. 23: 0 → 1
Char. 34: 0 → 1
Char. 55: 1 → 0
Char. 71: 1 → 0
Char. 85: 1 → 0
Char. 112: 0 → 1
Char. 129: 0 → 1
Char. 131: 0 → 1

***Barasaurus besairiei*:**
Char. 33: 1 → 0
Char. 75: 1 → 0
Char. 216: 0 → 1

***Bashkyroleter bashkyricus*:**
Char. 275: 1 → 0

***Bashkyroleter mesensis*:**
Char. 169: 1 → 0

***Belebey vegrandis*:**
Char. 154: 1 → 0

***Bradysaurus* spp.:**
Char. 19: 0 → 1
Char. 73: 0 → 1
Char. 79: 0 → 1
Char. 135: 1 → 0
Char. 249: 0 → 1

**Captorhinidae:**
Char. 3: 0 → 1
Char. 23: 0 → 1
Char. 25: 1 → 0
Char. 26: 0 → 1
Char. 73: 0 → 1
Char. 75: 0 → 1
Char. 83: 0 → 1
Char. 108: 1 → 0
Char. 183: 1 → 0
Char. 201: 1 → 0
Char. 203: 1 → 2
Char. 216: 1 → 0

**Caseidae:**
Char. 24: 0 → 1
Char. 25: 1 → 0
Char. 36: 0 → 1
Char. 38: 1 → 0
Char. 46: 1 → 0
Char. 56: 0 → 1
Char. 85: 1 → 0
Char. 96: 1 → 0
Char. 170: 0 → 1
Char. 194: 0 → 1
Char. 273: 0 → 1
Char. 274: 0 → 1
Char. 278: 3 → 2

***Claudiosaurus germaini*:**
Char. 24: 0 → 1
Char. 34: 0 → 1
Char. 36: 0 → 1
Char. 43: 1 → 0
Char. 64: 0 → 1
Char. 105: 0 → 1
Char. 127: 1 → 0
Char. 130: 0 → 1
Char. 141: 1 → 0
Char. 144: 1 → 0
Char. 166: 1 → 0
Char. 187: 1 → 0
Char. 199: 0 → 1
Char. 203: 1 → 2
Char. 204: 0 → 1
Char. 222: 1 → 0

***Colobomycter pholeter*:**
Char. 21: 0 → 1
Char. 25: 1 → 0
Char. 84: 1 → 0
Char. 154: 1 → 0
Char. 167: 0 → 1
Char. 267: 0 → 1

***Delorhynchus cifelli*:**
Char. 18: 0 → 1
Char. 20: 0 → 1
Char. 21: 0 → 1
Char. 24: 0 → 1
Char. 26: 0 → 1
Char. 28: 0 → 1
Char. 33: 0 → 2
Char. 39: 0 → 1
Char. 52: 0 → 1
Char. 100: 0 → 1
Char. 111: 0 → 1
Char. 116: 1 → 0
Char. 117: 0 → 1
Char. 131: 0 → 1
Char. 156: 1 → 0
Char. 167: 0 → 1
Char. 180: 0 → 1
Char. 189: 0 → 1
Char. 191: 0 → 1
Char. 204: 0 → 2
Char. 267: 0 → 1

**Diadectomorpha:**
Char. 0: 0 → 1
Char. 64: 0 → 1
Char. 70: 0 → 1
Char. 75: 0 → 1
Char. 122: 0 → 1
Char. 123: 1 → 0
Char. 146: 1 → 0
Char. 275: 1 → 0
Char. 278: 3 → 0

***Emeroleter levis*:**
Char. 0: 0 → 2
Char. 51: 1 → 0

**Eosauropterygia:**
Char. 159: 1 → 0
Char. 166: 1 → 0
Char. 174: 0 → 1
Char. 194: 0 → 2
Char. 272: 1 → 0

***Eudibamus cursoris*:**
Char. 154: 1 → 2

***Feeserpeton oklahomensis*:**
Char. 51: 0 → 1
Char. 70: 0 → 1
Char. 157: 0 → 1
Char. 158: 0 → 1

***Hovasaurus boulei*:**
Char. 41: 0 → 1
Char. 77: 0 → 2
Char. 79: 0 → 1
Char. 93: 1 → 0
Char. 204: 0 → 2

**Kuehneosauridae:**
Char. 7: 0 → 1
Char. 24: 0 → 1
Char. 26: 0 → 1
Char. 34: 0 → 1
Char. 36: 0 → 1
Char. 43: 1 → 0
Char. 79: 0 → 2
Char. 82: 0 → 1
Char. 98: 1 → 0
Char. 108: 1 → 0
Char. 113: 0 → 1
Char. 128: 0 → 1
Char. 148: 1 → 0
Char. 159: 1 → 0
Char. 181: 0 → 1
Char. 185: 0 → 1
Char. 206: 0 → 2
Char. 245: 0 → 1
Char. 272: 1 → 2
Char. 278: 0 → 3

***Lanthanosuchus watsoni*:**
Char. 25: 1 → 0
Char. 51: 0 → 1
Char. 76: 0 → 1
Char. 86: 0 → 1

Char. 95: 0 → 1
Char. 98: 1 → 0
Char. 110: 0 → 1
Char. 113: 0 → 1
Char. 114: 0 → 1
Char. 131: 0 → 1
Char. 137: 0 → 1
Char. 138: 0 → 1
Char. 140: 0 → 2
Char. 144: 1 → 0
Char. 154: 1 → 2

***Macroleter poezicus*:**
Char. 0: 0 → 1
Char. 52: 0 → 1
Char. 66: 1 → 2
Char. 84: 1 → 0
Char. 87: 0 → 1
Char. 140: 0 → 1
Char. 146: 1 → 0
Char. 169: 1 → 0
Char. 235: 0 → 1

***Mesosaurus* spp.:**
Char. 0: 0 → 1
Char. 2: 0 → 1
Char. 5: 1 → 0
Char. 6: 0 → 1
Char. 8: 0 → 1
Char. 9: 0 → 1
Char. 13: 0 → 1
Char. 19: 0 → 1
Char. 23: 0 → 1
Char. 26: 0 → 1
Char. 29: 1 → 0
Char. 33: 0 → 1
Char. 38: 1 → 0
Char. 41: 0 → 1
Char. 50: 1 → 0
Char. 67: 0 → 1
Char. 76: 0 → 1
Char. 83: 0 → 1
Char. 84: 1 → 0
Char. 85: 1 → 0
Char. 94: 0 → 1
Char. 107: 0 → 1
Char. 109: 0 → 1
Char. 111: 0 → 1
Char. 115: 0 → 1
Char. 146: 1 → 0
Char. 148: 0 → 1
Char. 149: 1 → 0
Char. 164: 0 → 1
Char. 167: 0 → 1
Char. 176: 0 → 1
Char. 183: 1 → 0
Char. 184: 0 → 1
Char. 199: 0 → 1
Char. 202: 0 → 1
Char. 204: 0 → 2
Char. 206: 0 → 1
Char. 207: 0 → 1
Char. 209: 0 → 1
Char. 217: 0 → 1
Char. 219: 0 → 1
Char. 220: 0 → 1
Char. 231: 0 → 1
Char. 260: 2 → 0
Char. 272: 2 → 0
Char. 278: 3 → 0

***Microleter mckinzieorum*:**
Char. 0: 0 → 1
Char. 25: 1 → 0
Char. 36: 0 → 1
Char. 39: 0 → 1
Char. 51: 0 → 2
Char. 56: 0 → 1
Char. 70: 0 → 1
Char. 94: 0 → 1
Char. 106: 0 → 1
Char. 276: 0 → 1

**Millerettidae:**
Char. 5: 1 → 0
Char. 24: 0 → 1
Char. 25: 1 → 0
Char. 44: 1 → 0
Char. 56: 0 → 1
Char. 57: 1 → 0
Char. 66: 0 → 2
Char. 78: 1 → 0
Char. 80: 1 → 0
Char. 84: 1 → 2
Char. 96: 1 → 0
Char. 117: 0 → 1
Char. 124: 0 → 1
Char. 127: 0 → 1
Char. 135: 0 → 1
Char. 145: 0 → 1
Char. 202: 0 → 1
Char. 211: 0 → 1
Char. 230: 0 → 1
Char. 234: 0 → 1
Char. 248: 0 → 1
Char. 252: 0 → 1
Char. 253: 0 → 1

***Nycteroleter ineptus*:**
Char. 278: 0 → 3

***Nyctiphruretus acudens*:**
Char. 0: 0 → 1
Char. 21: 0 → 1
Char. 33: 1 → 2
Char. 41: 0 → 1
Char. 66: 1 → 2
Char. 81: 1 → 0
Char. 83: 0 → 1
Char. 84: 1 → 2
Char. 94: 0 → 1
Char. 224: 0 → 1
Char. 266: 0 → 1
Char. 272: 2 → 1
Char. 276: 0 → 1

***Orovenator mayorum*:**
Char. 8: 0 → 1
Char. 24: 0 → 1
Char. 36: 0 → 1
Char. 160: 0 → 1
Char. 165: 0 → 1

***Owenetta* spp.:**
Char. 169: 1 → 0

***Paleothyris acadiana*:**
Char. 38: 1 → 0
Char. 50: 1 → 0
Char. 66: 1 → 2
Char. 102: 0 → 1
Char. 146: 1 → 0
Char. 180: 0 → 1
Char. 237: 0 → 1
Char. 239: 0 → 1

***Placodus* spp.:**
Char. 0: 1 → 2
Char. 9: 0 → 1
Char. 12: 0 → 1
Char. 13: 0 → 1
Char. 19: 0 → 1
Char. 26: 0 → 1
Char. 31: 0 → 1
Char. 46: 1 → 0
Char. 57: 0 → 1
Char. 78: 1 → 0
Char. 93: 1 → 0
Char. 102: 1 → 2
Char. 109: 1 → 0
Char. 140: 1 → 0
Char. 155: 0 → 1
Char. 163: 1 → 0
Char. 164: 0 → 1

***Procolophon* spp.:**
Char. 41: 0 → 1
Char. 69: 0 → 1
Char. 79: 0 → 1
Char. 83: 0 → 1
Char. 88: 0 → 1
Char. 117: 1 → 0
Char. 149: 1 → 0
Char. 180: 0 → 1
Char. 204: 0 → 1
Char. 237: 0 → 1
Char. 238: 0 → 1
Char. 272: 2 → 1
Char. 278: 3 → 0

***Prolacerta broomi*:**
Char. 58: 1 → 0
Char. 66: 1 → 0
Char. 67: 1 → 0
Char. 80: 1 → 0
Char. 139: 1 → 0
Char. 192: 1 → 0
Char. 203: 1 → 2
Char. 206: 0 → 12

***Rhipaeosaurus* spp.:**
Char. 172: 0 → 1
Char. 277: 0 → 1

**Rhynchocephalia:**
Char. 0: 1 → 2
Char. 24: 0 → 1
Char. 77: 0 → 1
Char. 94: 1 → 0
Char. 117: 12 → 0
Char. 139: 1 → 0
Char. 167: 1 → 0
Char. 205: 1 → 0

**Rhynchosauria:**
Char. 0: 1 → 0
Char. 7: 0 → 1
Char. 9: 0 → 1
Char. 26: 0 → 1
Char. 68: 0 → 1
Char. 99: 1 → 0
Char. 150: 1 → 0
Char. 160: 0 → 1
Char. 161: 0 → 1
Char. 171: 0 → 2
Char. 182: 1 → 0
Char. 223: 0 → 1
Char. 241: 0 → 1

***Scutosaurus* spp.:**
Char. 175: 0 → 1
Char. 218: 0 → 1
Char. 243: 0 → 2
Char. 244: 0 → 1
Char. 251: 0 → 1

***Sinosaurosphargis yunguiensis*:**
Char. 8: 0 → 1
Char. 30: 0 → 1
Char. 53: 0 → 1
Char. 82: 0 → 1
Char. 89: 1 → 0
Char. 127: 1 → 0
Char. 150: 1 → 0
Char. 154: 0 → 2
Char. 167: 1 → 0
Char. 253: 0 → 1
Char. 255: 0 → 1

**Squamata:**
Char. 26: 0 → 1
Char. 45: 0 → 1
Char. 79: 0 → 2
Char. 80: 1 → 0
Char. 82: 0 → 1
Char. 92: 1 → 0
Char. 109: 1 → 0
Char. 160: 0 → 1
Char. 245: 0 → 1

***Trilophosaurus buettneri*:**
Char. 5: 1 → 0
Char. 11: 0 → 1
Char. 55: 1 → 0
Char. 93: 1 → 0
Char. 104: 0 → 1
Char. 113: 0 → 1
Char. 122: 0 → 1
Char. 136: 1 → 0
Char. 144: 1 → 0
Char. 154: 0 → 1
Char. 157: 0 → 1
Char. 159: 1 → 0
Char. 177: 0 → 12
Char. 194: 0 → 1
Char. 203: 1 → 2
Char. 207: 1 → 0
Char. 208: 1 → 0
Char. 272: 1 → 0

***Youngina capensis*:**
Char. 5: 1 → 0
Char. 25: 0 → 1
Char. 56: 0 → 1
Char. 75: 0 → 1
Char. 92: 1 → 0
Char. 94: 1 → 0
Char. 163: 1 → 0
Char. 170: 0 → 1
Char. 211: 0 → 1
Char. 214: 0 → 1
Char. 239: 0 → 1

**Node 50:**
Char. 0: 1 → 2
Char. 15: 0 → 1
Char. 24: 0 → 1
Char. 33: 1 → 2
Char. 42: 0 → 1
Char. 48: 1 → 0
Char. 50: 0 → 1
Char. 57: 0 → 1
Char. 62: 1 → 0
Char. 64: 0 → 1
Char. 73: 0 → 1
Char. 81: 1 → 0
Char. 84: 01 → 2
Char. 129: 1 → 0
Char. 130: 0 → 1
Char. 134: 01 → 2
Char. 147: 0 → 1
Char. 148: 0 → 1
Char. 152: 0 → 1
Char. 155: 0 → 1
Char. 158: 0 → 1
Char. 161: 0 → 1
Char. 174: 0 → 1
Char. 181: 0 → 1
Char. 202: 1 → 0
Char. 203: 1 → 2
Char. 204: 0 → 2
Char. 240: 1 → 0
Char. 247: 0 → 1
Char. 248: 0 → 1
Char. 250: 0 → 1
Char. 251: 0 → 1
Char. 253: 0 → 2

**Node 51:**
Char. 33: 0 → 1
Char. 92: 0 → 1
Char. 94: 0 → 1

Char. 135: 0 → 1
Char. 278: 1 → 0

**Node 52:**
Char. 20: 0 → 1
Char. 25: 1 → 0
Char. 62: 0 → 1
Char. 141: 0 → 1

**Node 53:**
Char. 0: 0 → 1
Char. 29: 1 → 0
Char. 40: 2 → 0
Char. 57: 1 → 0
Char. 59: 0 → 1
Char. 60: 0 → 1
Char. 67: 0 → 1
Char. 72: 1 → 2
Char. 89: 0 → 1
Char. 111: 0 → 1
Char. 112: 0 → 1
Char. 120: 0 → 1
Char. 180: 0 → 1
Char. 193: 0 → 1
Char. 222: 0 → 1
Char. 234: 0 → 1
Char. 237: 0 → 1
Char. 266: 0 → 1
Char. 278: 3 → 1

**Node 54:**
Char. 5: 0 → 1
Char. 29: 0 → 1
Char. 40: 0 → 2
Char. 74: 0 → 1
Char. 116: 0 → 1
Char. 132: 0 → 1
Char. 149: 0 → 1
Char. 156: 0 → 1
Char. 169: 0 → 1
Char. 201: 0 → 1
Char. 203: 0 → 1
Char. 235: 0 → 1

**Node 55:**
Char. 97: 0 → 1

**Node 56:**
Char. 23: 1 → 0
Char. 55: 0 → 1
Char. 78: 0 → 1
Char. 83: 1 → 0
Char. 111: 1 → 0
Char. 126: 1 → 0
Char. 207: 1 → 0

**Node 57:**
Char. 20: 1 → 0
Char. 25: 0 → 1
Char. 33: 2 → 0
Char. 57: 0 → 1
Char. 94: 1 → 0
Char. 127: 1 → 0
Char. 184: 1 → 0
Char. 187: 1 → 0
Char. 192: 1 → 0
Char. 194: 2 → 0
Char. 202: 1 → 0
Char. 205: 1 → 0
Char. 206: 2 → 0
Char. 210: 1 → 0
Char. 220: 1 → 0
Char. 221: 1 → 0
Char. 222: 1 → 0
Char. 241: 1 → 0
Char. 246: 1 → 0
Char. 247: 1 → 0
Char. 252: 1 → 0
Char. 254: 1 → 0
Char. 255: 1 → 0
Char. 256: 1 → 0
Char. 268: 1 → 0
Char. 269: 1 → 0

**Node 58:**
Char. 78: 1 → 0
Char. 206: 0 → 2

**Node 59:**
Char. 19: 0 → 1
Char. 92: 1 → 0

**Node 60:**
Char. 4: 0 → 1
Char. 15: 0 → 1
Char. 29: 0 → 1
Char. 48: 1 → 0
Char. 213: 0 → 1
Char. 226: 0 → 2
Char. 228: 0 → 1
Char. 275: 0 → 1

**Node 61:**
Char. 58: 0 → 1
Char. 61: 0 → 1
Char. 66: 0 → 1
Char. 69: 0 → 1
Char. 150: 0 → 1
Char. 167: 0 → 1
Char. 205: 0 → 1
Char. 208: 0 → 1
Char. 230: 0 → 1
Char. 239: 0 → 1

**Node 62:**
Char. 23: 0 → 1
Char. 70: 0 → 1
Char. 73: 0 → 1
Char. 117: 0 → 12
Char. 126: 0 → 1
Char. 131: 0 → 1
Char. 148: 0 → 1
Char. 182: 0 → 1
Char. 190: 0 → 1
Char. 201: 0 → 1
Char. 214: 0 → 1
Char. 272: 2 → 1

**Node 63:**
Char. 24: 0 → 1
Char. 57: 1 → 0
Char. 79: 0 → 1
Char. 83: 0 → 1
Char. 110: 0 → 1
Char. 132: 1 → 0

**Node 64:**
Char. 73: 0 → 1
Char. 131: 1 → 0
Char. 205: 0 → 1
Char. 239: 0 → 1
Char. 276: 0 → 1

**Node 65:**
Char. 18: 0 → 1
Char. 37: 0 → 1
Char. 107: 0 → 1
Char. 118: 1 → 0
Char. 125: 1 → 0
Char. 150: 0 → 1

**Node 66:**
Char. 23: 0 → 1
Char. 71: 1 → 0
Char. 102: 0 → 1
Char. 103: 0 → 1
Char. 106: 0 → 1
Char. 214: 0 → 1
Char. 216: 1 → 0
Char. 235: 0 → 2
Char. 241: 0 → 1

**Node 67:**
Char. 138: 0 → 1
Char. 141: 0 → 1
Char. 207: 0 → 1

**Node 68:**
Char. 186: 0 → 1

**Node 69:**
Char. 148: 0 → 1
Char. 183: 1 → 2
Char. 194: 0 → 1
Char. 201: 1 → 0
Char. 211: 0 → 1
Char. 235: 1 → 0
Char. 252: 0 → 1

**Node 70:**
Char. 87: 0 → 1

**Node 71:**
Char. 25: 1 → 0
Char. 76: 1 → 0

**Node 72:**
Char. 79: 0 → 1
Char. 93: 1 → 0
Char. 133: 0 → 1

**Node 73:**
Char. 147: 1 → 2
Char. 154: 1 → 0
Char. 239: 0 → 1
Char. 278: 3 → 0

**Node 74:**
Char. 38: 1 → 2
Char. 39: 0 → 1
Char. 50: 1 → 0
Char. 58: 0 → 1
Char. 59: 0 → 1
Char. 60: 0 → 1
Char. 72: 1 → 2
Char. 83: 0 → 1
Char. 85: 1 → 0
Char. 95: 0 → 1
Char. 104: 0 → 1
Char. 105: 0 → 1
Char. 106: 0 → 2
Char. 107: 0 → 1
Char. 109: 0 → 1
Char. 110: 0 → 1
Char. 146: 1 → 0
Char. 148: 0 → 1
Char. 155: 0 → 2
Char. 183: 1 → 2

**Node 75:**
Char. 0: 0 → 2
Char. 33: 1 → 0
Char. 38: 2 → 1
Char. 39: 1 → 0
Char. 42: 0 → 1
Char. 43: 0 → 1
Char. 46: 1 → 0
Char. 49: 1 → 0
Char. 52: 0 → 1
Char. 83: 0 → 2
Char. 84: 1 → 0
Char. 87: 0 → 1
Char. 93: 1 → 0
Char. 161: 0 → 1
Char. 163: 1 → 0
Char. 172: 0 → 2
Char. 174: 0 → 1
Char. 188: 0 → 1
Char. 189: 0 → 1
Char. 195: 0 → 1
Char. 204: 0 → 2
Char. 212: 0 → 1
Char. 236: 0 → 1
Char. 238: 0 → 2
Char. 242: 0 → 1
Char. 245: 0 → 1
Char. 274: 0 → 1
Char. 275: 1 → 0

**Node 76:**
Char. 5: 1 → 0
Char. 29: 1 → 0
Char. 44: 1 → 0
Char. 59: 0 → 1
Char. 60: 0 → 1
Char. 66: 0 → 1
Char. 67: 0 → 1
Char. 72: 1 → 2
Char. 78: 1 → 0
Char. 84: 1 → 0
Char. 93: 1 → 0
Char. 111: 0 → 1
Char. 123: 1 → 0
Char. 129: 0 → 1
Char. 154: 1 → 0
Char. 169: 1 → 0
Char. 170: 0 → 1
Char. 197: 0 → 1

**Node 77:**
Char. 20: 0 → 1
Char. 47: 0 → 1
Char. 83: 0 → 1
Char. 85: 1 → 0
Char. 107: 0 → 1
Char. 110: 0 → 1
Char. 169: 1 → 0
Char. 170: 0 → 1

**Node 78:**
Char. 2: 0 → 1
Char. 6: 0 → 1
Char. 98: 1 → 0
Char. 101: 0 → 1
Char. 113: 0 → 1
Char. 181: 0 → 1
Char. 186: 0 → 1
Char. 193: 1 → 0
Char. 198: 0 → 1
Char. 206: 0 → 2
Char. 220: 0 → 1
Char. 239: 1 → 0
Char. 240: 1 → 0
Char. 266: 1 → 0

**Node 79:**
Char. 33: 1 → 2
Char. 68: 0 → 1
Char. 102: 0 → 1
Char. 103: 0 → 1
Char. 223: 0 → 1
Char. 229: 0 → 1
Char. 235: 1 → 2

**Node 80:**
Char. 17: 0 → 1
Char. 42: 0 → 1
Char. 251: 0 → 1
Char. 264: 0 → 1

**Node 81:**
Char. 41: 0 → 1
Char. 48: 1 → 0
Char. 61: 1 → 2
Char. 112: 1 → 0
Char. 128: 0 → 1
Char. 138: 0 → 1
Char. 146: 1 → 0
Char. 155: 0 → 1
Char. 182: 1 → 0
Char. 192: 1 → 2
Char. 226: 0 → 1
Char. 227: 0 → 1
Char. 233: 0 → 1

**Node 82:**
Char. 61: 1 → 3
Char. 90: 0 → 1
Char. 91: 0 → 1
Char. 109: 1 → 0
Char. 155: 0 → 1
Char. 209: 1 → 0

**ANALYSIS 32**

**(NO *PROGANOCHELYS QUENSTEDTI*, *CANDELARIA BARBOURI*, *PAPPOCHELYS ROSINAE*, *ODONTOCHELYS SEMITESTACEA*, AND *EUNOTOSAURUS AFRICANUS*)**

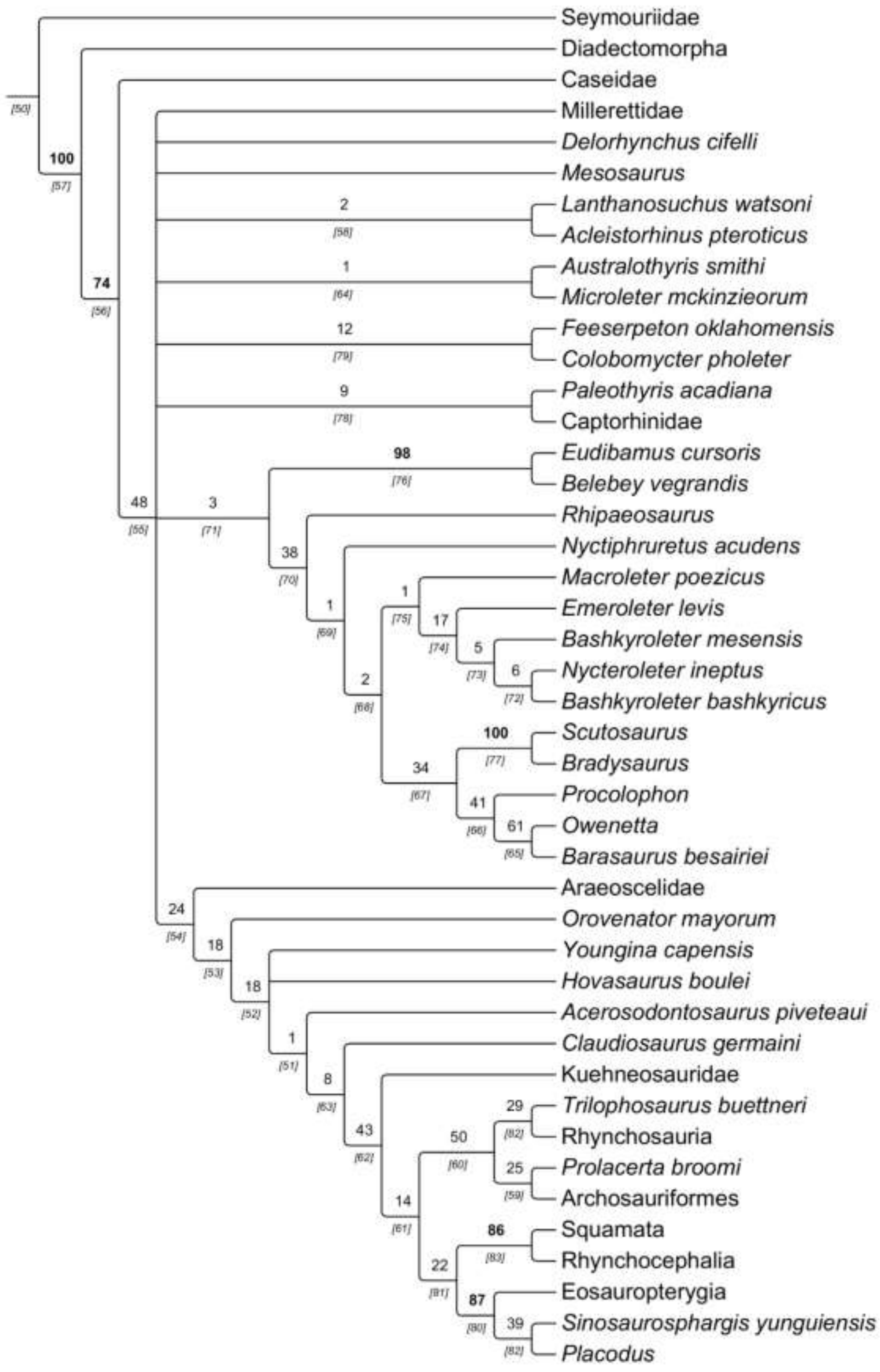

**Seymouriidae:**
Char. 51: 0 → 2
Char. 54: 1 → 0
Char. 71: 1 → 0
Char. 83: 0 → 1
Char. 85: 1 → 0
Char. 99: 1 → 0
Char. 107: 0 → 1
Char. 140: 0 → 2
Char. 154: 1 → 2
Char. 225: 1 → 0
Char. 260: 2 → 0

***Acerosodontosaurus piveteaui*:**
Char. 78: 1 → 0
Char. 128: 0 → 1
Char. 155: 0 → 1
Char. 206: 0 → 2

***Acleistorhinus pteroticus*:**
Char. 21: 0 → 1
Char. 146: 1 → 0
Char. 169: 1 → 0
Char. 170: 0 → 1

**Araeoscelidae:**
Char. 23: 0 → 1
Char. 28: 0 → 1
Char. 106: 0 → 1
Char. 116: 1 → 0
Char. 169: 1 → 0
Char. 170: 0 → 1
Char. 197: 0 → 1
Char. 239: 0 → 1

**Archosauriformes:**
Char. 32: 0 → 1
Char. 94: 1 → 0
Char. 112: 1 → 0
Char. 152: 0 → 1
Char. 154: 0 → 2
Char. 166: 1 → 0
Char. 171: 0 → 1
Char. 185: 0 → 1
Char. 204: 0 → 1
Char. 218: 0 → 3
Char. 224: 0 → 1
Char. 242: 0 → 1

***Australothyris smithi*:**
Char. 23: 0 → 1
Char. 34: 0 → 1
Char. 55: 1 → 0
Char. 71: 1 → 0
Char. 85: 1 → 0
Char. 112: 0 → 1
Char. 129: 0 → 1
Char. 131: 0 → 1

***Barasaurus besairiei*:**
Char. 33: 1 → 0
Char. 75: 1 → 0
Char. 216: 0 → 1

***Bashkyroleter bashkyricus*:**
Char. 275: 1 → 0

***Bashkyroleter mesensis*:**
Char. 169: 1 → 0

***Belebey vegrandis*:**
Char. 154: 1 → 0

***Bradysaurus* spp.:**
Char. 19: 0 → 1
Char. 73: 0 → 1
Char. 79: 0 → 1
Char. 135: 1 → 0
Char. 249: 0 → 1

**Captorhinidae:**
Char. 3: 0 → 1
Char. 23: 0 → 1
Char. 25: 1 → 0
Char. 26: 0 → 1
Char. 73: 0 → 1
Char. 75: 0 → 1
Char. 83: 0 → 1
Char. 108: 1 → 0
Char. 183: 1 → 0
Char. 201: 1 → 0
Char. 203: 1 → 2
Char. 216: 1 → 0

**Caseidae:**
Char. 24: 0 → 1
Char. 25: 1 → 0
Char. 36: 0 → 1
Char. 38: 1 → 0
Char. 46: 1 → 0
Char. 50: 1 → 0
Char. 56: 0 → 1
Char. 85: 1 → 0
Char. 96: 1 → 0
Char. 98: 1 → 0
Char. 170: 0 → 1
Char. 194: 0 → 1
Char. 273: 0 → 1
Char. 274: 0 → 1
Char. 278: 3 → 2

***Claudiosaurus germaini*:**
Char. 64: 0 → 1
Char. 105: 0 → 1
Char. 130: 0 → 1
Char. 144: 1 → 0
Char. 187: 1 → 0
Char. 199: 0 → 1
Char. 203: 1 → 2
Char. 204: 0 → 1
Char. 220: 0 → 1
Char. 222: 1 → 0

***Colobomycter pholeter*:**
Char. 21: 0 → 1
Char. 25: 1 → 0
Char. 84: 1 → 0
Char. 154: 1 → 0
Char. 167: 0 → 1
Char. 267: 0 → 1

***Delorhynchus cifelli*:**
Char. 18: 0 → 1
Char. 20: 0 → 1
Char. 21: 0 → 1
Char. 24: 0 → 1
Char. 26: 0 → 1
Char. 28: 0 → 1
Char. 33: 0 → 2
Char. 39: 0 → 1
Char. 52: 0 → 1
Char. 100: 0 → 1
Char. 111: 0 → 1
Char. 116: 1 → 0
Char. 117: 0 → 1
Char. 131: 0 → 1
Char. 147: 0 → 1
Char. 156: 1 → 0
Char. 159: 0 → 1
Char. 167: 0 → 1
Char. 180: 0 → 1
Char. 189: 0 → 1
Char. 191: 0 → 1
Char. 192: 0 → 1
Char. 204: 0 → 2
Char. 267: 0 → 1

**Diadectomorpha:**
Char. 0: 0 → 1
Char. 70: 0 → 1
Char. 75: 0 → 1
Char. 122: 0 → 1
Char. 123: 1 → 0
Char. 146: 1 → 0
Char. 275: 1 → 0
Char. 278: 3 → 0

***Emeroleter levis*:**
Char. 51: 1 → 0

**Eosauropterygia:**
Char. 159: 1 → 0
Char. 166: 1 → 0
Char. 174: 0 → 1
Char. 194: 0 → 2
Char. 272: 1 → 0

***Eudibamus cursoris*:**
Char. 154: 1 → 2

***Feeserpeton oklahomensis*:**
Char. 51: 0 → 1
Char. 70: 0 → 1
Char. 157: 0 → 1
Char. 158: 0 → 1

***Hovasaurus boulei*:**
Char. 41: 0 → 1
Char. 55: 1 → 0
Char. 60: 1 → 0
Char. 72: 2 → 1
Char. 77: 0 → 2
Char. 78: 1 → 0
Char. 79: 0 → 1
Char. 93: 1 → 0
Char. 113: 0 → 1
Char. 138: 0 → 1
Char. 146: 1 → 0
Char. 154: 0 → 1
Char. 204: 0 → 2
Char. 206: 0 → 2
Char. 220: 0 → 1
Char. 278: 01 → 3

**Kuehneosauridae:**
Char. 7: 0 → 1
Char. 26: 0 → 1
Char. 44: 1 → 0
Char. 79: 0 → 2
Char. 82: 0 → 1
Char. 98: 1 → 0
Char. 108: 1 → 0
Char. 113: 0 → 1
Char. 128: 0 → 1
Char. 159: 1 → 0
Char. 181: 0 → 1
Char. 185: 0 → 1
Char. 206: 0 → 2
Char. 245: 0 → 1
Char. 278: 0 → 3

***Lanthanosuchus watsoni*:**
Char. 25: 1 → 0
Char. 51: 0 → 1
Char. 76: 0 → 1
Char. 86: 0 → 1
Char. 98: 1 → 0
Char. 138: 0 → 1
Char. 144: 1 → 0
Char. 154: 1 → 2

***Macroleter poezicus*:**
Char. 52: 0 → 1
Char. 84: 1 → 0
Char. 87: 0 → 1
Char. 140: 0 → 1
Char. 146: 1 → 0
Char. 169: 1 → 0
Char. 235: 0 → 1

***Mesosaurus* spp.:**
Char. 0: 0 → 1
Char. 2: 0 → 1
Char. 6: 0 → 1
Char. 8: 0 → 1
Char. 9: 0 → 1
Char. 13: 0 → 1
Char. 19: 0 → 1
Char. 23: 0 → 1
Char. 26: 0 → 1
Char. 29: 1 → 0
Char. 33: 0 → 1
Char. 38: 1 → 0
Char. 41: 0 → 1
Char. 50: 1 → 0
Char. 67: 0 → 1
Char. 76: 0 → 1
Char. 83: 0 → 1
Char. 84: 1 → 0
Char. 85: 1 → 0
Char. 94: 0 → 1
Char. 107: 0 → 1
Char. 109: 0 → 1
Char. 111: 0 → 1
Char. 115: 0 → 1
Char. 146: 1 → 0
Char. 148: 0 → 1
Char. 149: 1 → 0
Char. 164: 0 → 1
Char. 167: 0 → 1
Char. 176: 0 → 1
Char. 183: 1 → 0
Char. 184: 0 → 1
Char. 199: 0 → 1
Char. 202: 0 → 1
Char. 204: 0 → 2
Char. 206: 0 → 1
Char. 207: 0 → 1
Char. 209: 0 → 1
Char. 217: 0 → 1
Char. 219: 0 → 1
Char. 220: 0 → 1
Char. 231: 0 → 1
Char. 260: 2 → 0
Char. 272: 2 → 0
Char. 278: 3 → 0

***Microleter mckinzieorum*:**
Char. 0: 0 → 1
Char. 25: 1 → 0
Char. 36: 0 → 1
Char. 39: 0 → 1
Char. 51: 0 → 2
Char. 56: 0 → 1
Char. 70: 0 → 1
Char. 94: 0 → 1
Char. 106: 0 → 1
Char. 159: 0 → 1
Char. 276: 0 → 1

**Millerettidae:**
Char. 24: 0 → 1
Char. 25: 1 → 0
Char. 44: 1 → 0
Char. 56: 0 → 1
Char. 57: 1 → 0
Char. 66: 0 → 2
Char. 78: 1 → 0
Char. 80: 1 → 0
Char. 84: 1 → 2
Char. 88: 1 → 0
Char. 96: 1 → 0
Char. 117: 0 → 1
Char. 124: 0 → 1
Char. 127: 0 → 1
Char. 135: 0 → 1
Char. 145: 0 → 1
Char. 202: 0 → 1
Char. 211: 0 → 1
Char. 230: 0 → 1
Char. 234: 0 → 1

Char. 248: 0 → 1
Char. 252: 0 → 1
Char. 253: 0 → 1

***Nycteroleter ineptus*:**
Char. 278: 0 → 3

***Nyctiphruretus acudens*:**
Char. 21: 0 → 1
Char. 33: 1 → 2
Char. 41: 0 → 1
Char. 81: 1 → 0
Char. 84: 1 → 2
Char. 94: 0 → 1
Char. 167: 0 → 1
Char. 224: 0 → 1
Char. 266: 0 → 1
Char. 272: 2 → 1
Char. 276: 0 → 1

***Orovenator mayorum*:**
Char. 8: 0 → 1
Char. 24: 0 → 1
Char. 36: 0 → 1
Char. 160: 0 → 1
Char. 165: 0 → 1

***Owenetta* spp.:**
Char. 169: 1 → 0

***Paleothyris acadiana*:**
Char. 38: 1 → 0
Char. 50: 1 → 0
Char. 66: 1 → 2
Char. 102: 0 → 1
Char. 146: 1 → 0
Char. 180: 0 → 1
Char. 237: 0 → 1
Char. 239: 0 → 1

***Placodus* spp.:**
Char. 0: 1 → 2
Char. 9: 0 → 1
Char. 12: 0 → 1
Char. 13: 0 → 1
Char. 19: 0 → 1
Char. 26: 0 → 1
Char. 31: 0 → 1
Char. 44: 1 → 0
Char. 46: 1 → 0
Char. 57: 0 → 1
Char. 78: 1 → 0
Char. 93: 1 → 0
Char. 102: 1 → 2
Char. 109: 1 → 0
Char. 140: 1 → 0
Char. 155: 0 → 1
Char. 163: 1 → 0
Char. 164: 0 → 1

***Procolophon* spp.:**
Char. 41: 0 → 1
Char. 69: 0 → 1
Char. 79: 0 → 1
Char. 88: 0 → 1
Char. 117: 1 → 0
Char. 149: 1 → 0
Char. 180: 0 → 1
Char. 204: 0 → 1
Char. 237: 0 → 1
Char. 238: 0 → 1
Char. 272: 2 → 1
Char. 278: 3 → 0

***Prolacerta broomi*:**
Char. 58: 1 → 0
Char. 66: 1 → 0
Char. 67: 1 → 0
Char. 80: 1 → 0
Char. 139: 1 → 0
Char. 147: 1 → 0
Char. 192: 1 → 0
Char. 203: 1 → 2
Char. 206: 0 → 12

***Rhipaeosaurus* spp.:**
Char. 172: 0 → 1
Char. 277: 0 → 1

**Rhynchocephalia:**
Char. 0: 1 → 2
Char. 24: 0 → 1
Char. 75: 1 → 0
Char. 77: 0 → 1
Char. 94: 1 → 0
Char. 117: 12 → 0
Char. 139: 1 → 0
Char. 167: 1 → 0
Char. 205: 1 → 0

**Rhynchosauria:**
Char. 0: 1 → 0
Char. 7: 0 → 1
Char. 9: 0 → 1
Char. 26: 0 → 1
Char. 44: 1 → 0
Char. 68: 0 → 1
Char. 99: 1 → 0
Char. 150: 1 → 0
Char. 160: 0 → 1
Char. 161: 0 → 1
Char. 171: 0 → 2
Char. 182: 1 → 0
Char. 223: 0 → 1
Char. 224: 0 → 1
Char. 241: 0 → 1

***Scutosaurus* spp.:**
Char. 175: 0 → 1
Char. 218: 0 → 1
Char. 243: 0 → 2
Char. 244: 0 → 1
Char. 251: 0 → 1

***Sinosaurosphargis yunguiensis*:**
Char. 8: 0 → 1
Char. 30: 0 → 1
Char. 53: 0 → 1
Char. 82: 0 → 1
Char. 89: 1 → 0
Char. 127: 1 → 0
Char. 150: 1 → 0
Char. 154: 0 → 2
Char. 167: 1 → 0
Char. 253: 0 → 1
Char. 255: 0 → 1

**Squamata:**
Char. 26: 0 → 1
Char. 45: 0 → 1
Char. 79: 0 → 2
Char. 80: 1 → 0
Char. 82: 0 → 1
Char. 92: 1 → 0
Char. 109: 1 → 0
Char. 160: 0 → 1
Char. 245: 0 → 1

***Trilophosaurus buettneri*:**
Char. 5: 1 → 0
Char. 11: 0 → 1
Char. 55: 1 → 0
Char. 93: 1 → 0
Char. 104: 0 → 1
Char. 113: 0 → 1
Char. 122: 0 → 1
Char. 136: 1 → 0
Char. 144: 1 → 0
Char. 154: 0 → 1
Char. 157: 0 → 1
Char. 159: 1 → 0
Char. 177: 0 → 12
Char. 194: 0 → 1
Char. 203: 1 → 2
Char. 207: 1 → 0
Char. 208: 1 → 0
Char. 272: 1 → 0

***Youngina capensis*:**
Char. 21: 0 → 1
Char. 44: 1 → 0
Char. 75: 0 → 1
Char. 170: 0 → 1
Char. 211: 0 → 1
Char. 231: 0 → 1
Char. 239: 0 → 1

**Node 50:**
Char. 15: 1 → 0
Char. 20: 1 → 0
Char. 25: 0 → 1
Char. 33: 2 → 0
Char. 73: 1 → 0
Char. 94: 1 → 0
Char. 119: 1 → 0
Char. 127: 1 → 0
Char. 130: 1 → 0
Char. 134: 2 → 0
Char. 136: 1 → 0
Char. 141: 1 → 0
Char. 145: 1 → 0
Char. 147: 1 → 0
Char. 149: 1 → 0
Char. 151: 1 → 0
Char. 152: 1 → 0
Char. 155: 1 → 0
Char. 158: 1 → 0
Char. 161: 1 → 0
Char. 163: 1 → 0
Char. 166: 1 → 0
Char. 174: 1 → 0
Char. 180: 1 → 0
Char. 181: 1 → 0
Char. 184: 1 → 0
Char. 187: 1 → 0
Char. 188: 1 → 0
Char. 189: 1 → 0
Char. 192: 1 → 0
Char. 194: 2 → 0
Char. 196: 1 → 0
Char. 203: 2 → 0
Char. 204: 2 → 0
Char. 205: 1 → 0
Char. 208: 1 → 0
Char. 210: 1 → 0
Char. 217: 1 → 0
Char. 220: 1 → 0
Char. 221: 1 → 0
Char. 222: 1 → 0
Char. 226: 1 → 0
Char. 227: 1 → 0
Char. 231: 1 → 0
Char. 234: 1 → 0
Char. 241: 1 → 0
Char. 246: 1 → 0
Char. 247: 1 → 0
Char. 251: 1 → 0
Char. 252: 1 → 0
Char. 253: 2 → 0
Char. 254: 1 → 0
Char. 255: 1 → 0
Char. 256: 1 → 0
Char. 266: 1 → 0
Char. 268: 1 → 0
Char. 269: 1 → 0

**Node 51:**
Char. 23: 0 → 1
Char. 94: 0 → 1
Char. 224: 1 → 0

**Node 52:**
Char. 33: 0 → 1
Char. 135: 0 → 1
Char. 159: 0 → 1

**Node 53:**
Char. 20: 0 → 1
Char. 62: 0 → 1

**Node 54:**
Char. 0: 0 → 1
Char. 27: 0 → 1
Char. 29: 1 → 0
Char. 38: 1 → 0
Char. 40: 2 → 0
Char. 57: 1 → 0
Char. 59: 0 → 1
Char. 60: 0 → 1
Char. 67: 0 → 1
Char. 72: 1 → 2
Char. 84: 1 → 0
Char. 89: 0 → 1
Char. 111: 0 → 1
Char. 112: 0 → 1
Char. 120: 0 → 1
Char. 154: 1 → 0
Char. 180: 0 → 1
Char. 192: 0 → 1
Char. 193: 0 → 1
Char. 222: 0 → 1
Char. 224: 0 → 1
Char. 234: 0 → 1
Char. 237: 0 → 1
Char. 266: 0 → 1
Char. 278: 3 → 1

**Node 55:**
Char. 29: 0 → 1
Char. 40: 0 → 2
Char. 74: 0 → 1
Char. 88: 0 → 1
Char. 116: 0 → 1
Char. 149: 0 → 1
Char. 169: 0 → 1
Char. 201: 0 → 1
Char. 203: 0 → 1
Char. 235: 0 → 1
Char. 276: 1 → 0

**Node 56:**
Char. 81: 0 → 1
Char. 97: 0 → 1

**Node 57:**
Char. 55: 0 → 1
Char. 78: 0 → 1
Char. 111: 1 → 0
Char. 126: 1 → 0
Char. 135: 1 → 0

**Node 58:**
Char. 95: 0 → 1
Char. 110: 0 → 1
Char. 113: 0 → 1
Char. 114: 0 → 1
Char. 131: 0 → 1
Char. 137: 0 → 1
Char. 140: 0 → 2
Char. 147: 0 → 1

**Node 59:**
Char. 19: 0 → 1
Char. 92: 1 → 0

**Node 60:**
Char. 4: 0 → 1
Char. 15: 0 → 1
Char. 29: 0 → 1
Char. 213: 0 → 1
Char. 226: 0 → 2

Char. 228: 0 → 1
Char. 275: 0 → 1

**Node 61:**
Char. 27: 1 → 0
Char. 75: 0 → 1
Char. 107: 0 → 1
Char. 140: 0 → 1
Char. 147: 0 → 1

**Node 62:**
Char. 58: 0 → 1
Char. 61: 0 → 1
Char. 66: 0 → 1
Char. 69: 0 → 1
Char. 150: 0 → 1
Char. 167: 0 → 1
Char. 205: 0 → 1
Char. 239: 0 → 1

**Node 63:**
Char. 126: 0 → 1
Char. 190: 0 → 1

**Node 64:**
Char. 24: 0 → 1
Char. 57: 1 → 0
Char. 79: 0 → 1
Char. 83: 0 → 1
Char. 110: 0 → 1
Char. 132: 1 → 0

**Node 65:**
Char. 73: 0 → 1
Char. 131: 1 → 0
Char. 205: 0 → 1
Char. 276: 0 → 1

**Node 66:**
Char. 18: 0 → 1
Char. 37: 0 → 1
Char. 107: 0 → 1
Char. 118: 1 → 0
Char. 125: 1 → 0
Char. 150: 0 → 1

**Node 67:**
Char. 23: 0 → 1
Char. 71: 1 → 0
Char. 102: 0 → 1
Char. 103: 0 → 1
Char. 106: 0 → 1
Char. 167: 0 → 1
Char. 214: 0 → 1
Char. 216: 1 → 0
Char. 235: 0 → 2
Char. 241: 0 → 1

**Node 68:**
Char. 207: 0 → 1

**Node 69:**
Char. 226: 0 → 1

**Node 70:**
Char. 20: 0 → 1
Char. 33: 0 → 1
Char. 44: 1 → 0
Char. 70: 0 → 1
Char. 80: 1 → 0
Char. 117: 0 → 1
Char. 118: 0 → 1
Char. 125: 0 → 1
Char. 131: 0 → 1
Char. 147: 0 → 1
Char. 194: 0 → 1
Char. 201: 1 → 0
Char. 211: 0 → 1

**Node 71:**
Char. 38: 1 → 2
Char. 39: 0 → 1
Char. 49: 0 → 1
Char. 66: 0 → 12
Char. 76: 0 → 1
Char. 85: 1 → 0
Char. 88: 1 → 0
Char. 95: 0 → 1
Char. 112: 0 → 1
Char. 148: 0 → 1
Char. 158: 0 → 1
Char. 159: 0 → 1
Char. 183: 1 → 2
Char. 192: 0 → 1

**Node 72:**
Char. 87: 0 → 1

**Node 73:**
Char. 25: 1 → 0
Char. 76: 1 → 0

**Node 74:**
Char. 79: 0 → 1
Char. 93: 1 → 0
Char. 133: 0 → 1

**Node 75:**
Char. 85: 0 → 1
Char. 147: 1 → 2
Char. 154: 1 → 0
Char. 278: 3 → 0

**Node 76:**
Char. 50: 1 → 0
Char. 58: 0 → 1
Char. 59: 0 → 1
Char. 60: 0 → 1
Char. 72: 1 → 2
Char. 104: 0 → 1
Char. 105: 0 → 1
Char. 106: 0 → 2
Char. 107: 0 → 1
Char. 109: 0 → 1
Char. 110: 0 → 1
Char. 146: 1 → 0
Char. 155: 0 → 2

**Node 77:**
Char. 33: 1 → 0
Char. 38: 2 → 1
Char. 39: 1 → 0
Char. 42: 0 → 1
Char. 43: 0 → 1
Char. 46: 1 → 0
Char. 49: 1 → 0
Char. 52: 0 → 1
Char. 83: 01 → 2
Char. 84: 1 → 0
Char. 87: 0 → 1
Char. 93: 1 → 0
Char. 161: 0 → 1
Char. 163: 1 → 0
Char. 172: 0 → 2
Char. 174: 0 → 1
Char. 188: 0 → 1
Char. 189: 0 → 1
Char. 195: 0 → 1
Char. 204: 0 → 2
Char. 212: 0 → 1
Char. 236: 0 → 1
Char. 238: 0 → 2
Char. 242: 0 → 1
Char. 274: 0 → 1
Char. 275: 1 → 0

**Node 78:**
Char. 29: 1 → 0
Char. 44: 1 → 0
Char. 59: 0 → 1
Char. 60: 0 → 1
Char. 66: 0 → 1
Char. 67: 0 → 1
Char. 72: 1 → 2
Char. 78: 1 → 0
Char. 84: 1 → 0
Char. 88: 1 → 0
Char. 93: 1 → 0
Char. 111: 0 → 1
Char. 123: 1 → 0
Char. 129: 0 → 1
Char. 154: 1 → 0
Char. 169: 1 → 0
Char. 170: 0 → 1
Char. 197: 0 → 1

**Node 79:**
Char. 20: 0 → 1
Char. 47: 0 → 1
Char. 83: 0 → 1
Char. 85: 1 → 0
Char. 107: 0 → 1
Char. 110: 0 → 1
Char. 169: 1 → 0
Char. 170: 0 → 1

**Node 80:**
Char. 2: 0 → 1
Char. 6: 0 → 1
Char. 98: 1 → 0
Char. 101: 0 → 1
Char. 113: 0 → 1
Char. 181: 0 → 1
Char. 186: 0 → 1
Char. 193: 1 → 0
Char. 198: 0 → 1
Char. 206: 0 → 2
Char. 220: 0 → 1
Char. 239: 1 → 0
Char. 240: 1 → 0
Char. 266: 1 → 0

**Node 81:**
Char. 33: 1 → 2
Char. 68: 0 → 1
Char. 102: 0 → 1
Char. 103: 0 → 1
Char. 223: 0 → 1
Char. 229: 0 → 1
Char. 235: 1 → 2

**Node 82:**
Char. 17: 0 → 1
Char. 42: 0 → 1
Char. 251: 0 → 1
Char. 264: 0 → 1

**Node 83:**
Char. 41: 0 → 1
Char. 61: 1 → 2
Char. 112: 1 → 0
Char. 128: 0 → 1
Char. 138: 0 → 1
Char. 146: 1 → 0
Char. 155: 0 → 1
Char. 182: 1 → 0
Char. 192: 1 → 2
Char. 224: 0 → 1
Char. 226: 0 → 1
Char. 227: 0 → 1
Char. 233: 0 → 1

**Node 84:**
Char. 61: 1 → 3
Char. 90: 0 → 1
Char. 91: 0 → 1
Char. 109: 1 → 0
Char. 155: 0 → 1
Char. 209: 1 → 0

**ANALYSIS 33**
**(ALL TAXA, IMPLIED WEIGHTING, K = 1)**

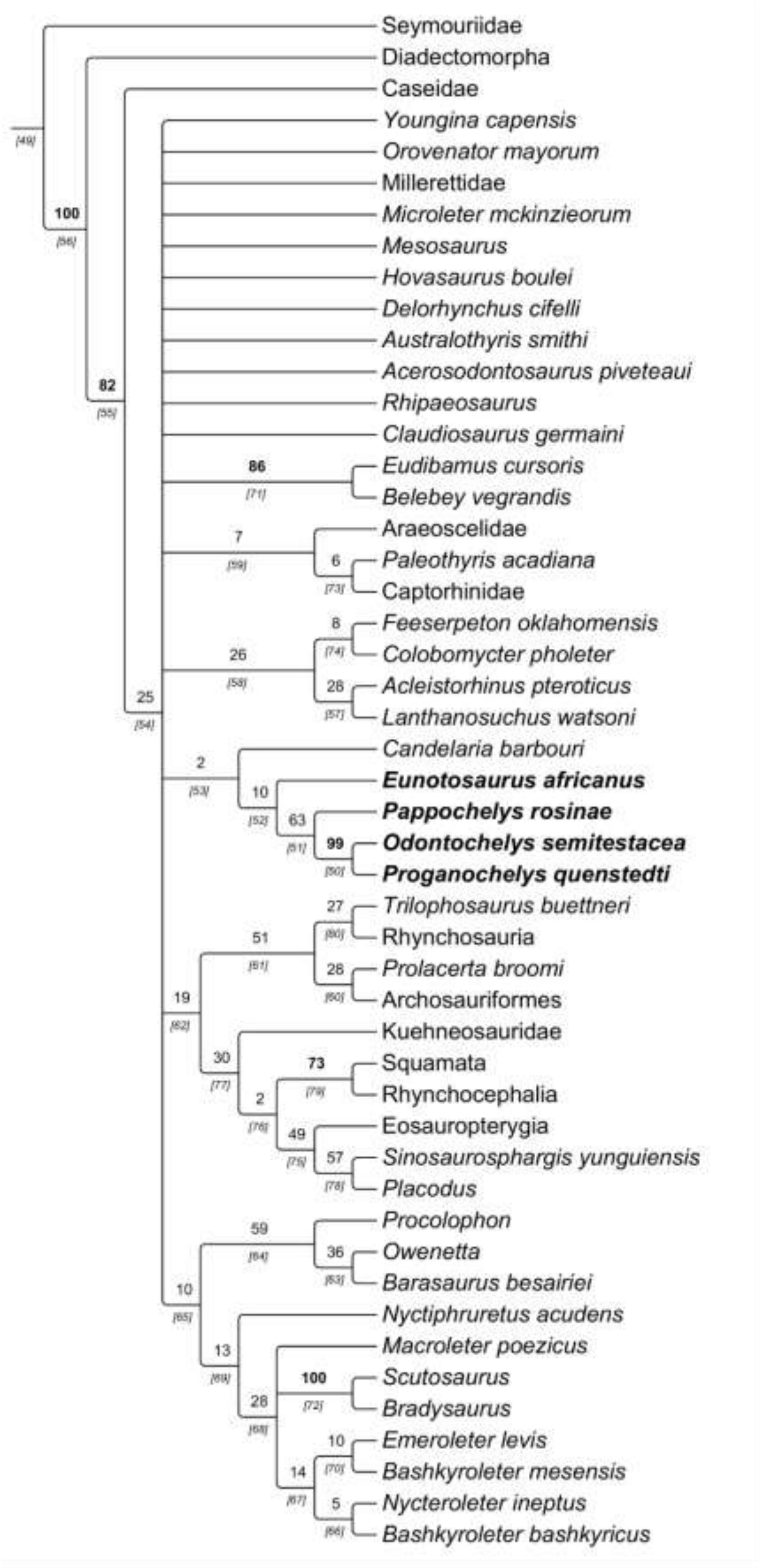

***Proganochelys quenstedti*:**
Char. 8: 0 → 1
Char. 11: 0 → 1
Char. 106: 0 → 1
Char. 108: 1 → 0
Char. 109: 0 → 1
Char. 128: 0 → 1
Char. 175: 0 → 1
Char. 202: 1 → 0
Char. 207: 1 → 0
Char. 209: 1 → 0
Char. 244: 0 → 1
Char. 248: 0 → 1
Char. 250: 0 → 1
Char. 252: 1 → 0
Char. 262: 0 → 1

***Pappochelys rosinae*:**
Char. 1: 0 → 1
Char. 5: 1 → 0
Char. 12: 0 → 1
Char. 41: 0 → 1
Char. 48: 0 → 1
Char. 49: 0 → 1
Char. 75: 0 → 1
Char. 129: 0 → 1
Char. 169: 1 → 0
Char. 260: 2 → 0
Char. 265: 0 → 1

***Odontochelys semitestacea*:**
Char. 43: 0 → 1

***Eunotosaurus africanus*:**
Char. 19: 0 → 1
Char. 43: 0 → 1
Char. 97: 1 → 0
Char. 103: 0 → 1
Char. 153: 1 → 0
Char. 192: 1 → 0
Char. 202: 1 → 0
Char. 211: 0 → 1
Char. 219: 0 → 1
Char. 248: 0 → 1
Char. 249: 0 → 2
Char. 250: 0 → 1
Char. 263: 0 → 1
Char. 273: 0 → 1
Char. 274: 0 → 1

***Acerosodontosaurus piveteaui*:**
Char. 59: 0 → 1
Char. 78: 1 → 0
Char. 81: 1 → 0
Char. 89: 0 → 1
Char. 94: 0 → 1
Char. 128: 0 → 1
Char. 129: 0 → 1
Char. 155: 0 → 1
Char. 206: 0 → 2
Char. 208: 0 → 1
Char. 265: 0 → 1
Char. 266: 0 → 1
Char. 278: 3 → 1

***Acleistorhinus pteroticus*:**
Char. 21: 0 → 1
Char. 146: 1 → 0

**Araeoscelidae:**
Char. 28: 0 → 1
Char. 43: 0 → 1
Char. 89: 0 → 1
Char. 106: 0 → 1
Char. 193: 0 → 1
Char. 222: 0 → 1
Char. 224: 0 → 1
Char. 266: 0 → 1
Char. 278: 3 → 1

**Archosauriformes:**
Char. 32: 0 → 1
Char. 94: 1 → 0
Char. 112: 1 → 0
Char. 152: 0 → 1
Char. 154: 01 → 2
Char. 166: 1 → 0
Char. 171: 0 → 1
Char. 185: 0 → 1
Char. 204: 0 → 1
Char. 218: 0 → 3
Char. 224: 0 → 1
Char. 242: 0 → 1

***Australothyris smithi*:**
Char. 0: 1 → 0
Char. 24: 0 → 1
Char. 34: 0 → 1
Char. 50: 0 → 1
Char. 55: 1 → 0
Char. 71: 1 → 0
Char. 79: 0 → 1
Char. 83: 0 → 1
Char. 85: 1 → 0
Char. 98: 1 → 0
Char. 100: 0 → 1
Char. 103: 0 → 1
Char. 110: 0 → 1
Char. 111: 1 → 0
Char. 123: 1 → 0
Char. 129: 0 → 1
Char. 131: 0 → 1
Char. 132: 1 → 0
Char. 144: 1 → 0
Char. 147: 0 → 1
Char. 149: 1 → 0
Char. 150: 0 → 1
Char. 159: 1 → 0
Char. 163: 1 → 0
Char. 192: 1 → 0

***Barasaurus besairiei*:**
Char. 33: 1 → 0
Char. 50: 0 → 1

***Bashkyroleter bashkyricus*:**
Char. 275: 1 → 0

***Bashkyroleter mesensis*:**
Char. 12: 0 → 1
Char. 66: 1 → 0
Char. 169: 1 → 0

***Belebey vegrandis*:**
Char. 154: 1 → 0

***Bradysaurus* spp.:**
Char. 19: 0 → 1
Char. 25: 1 → 0
Char. 73: 0 → 1
Char. 79: 0 → 1
Char. 135: 1 → 0
Char. 249: 0 → 1

***Candelaria barbouri*:**
Char. 1: 0 → 1
Char. 5: 1 → 0
Char. 8: 0 → 1
Char. 49: 0 → 1
Char. 79: 0 → 2
Char. 83: 0 → 1
Char. 88: 1 → 0
Char. 92: 1 → 0
Char. 95: 0 → 1
Char. 132: 1 → 0
Char. 154: 1 → 2
Char. 159: 1 → 0
Char. 169: 1 → 0

**Captorhinidae:**
Char. 3: 0 → 1
Char. 25: 1 → 0
Char. 26: 0 → 1
Char. 50: 0 → 1
Char. 73: 0 → 1
Char. 75: 0 → 1
Char. 83: 0 → 1
Char. 108: 1 → 0
Char. 180: 1 → 0
Char. 183: 1 → 0
Char. 203: 1 → 2
Char. 216: 1 → 0
Char. 240: 1 → 0

**Caseidae:**
Char. 24: 0 → 1
Char. 25: 1 → 0
Char. 36: 0 → 1
Char. 38: 1 → 0
Char. 46: 1 → 0
Char. 56: 0 → 1
Char. 170: 0 → 1
Char. 194: 0 → 1
Char. 273: 0 → 1
Char. 274: 0 → 1
Char. 278: 3 → 2

***Claudiosaurus germaini*:**
Char. 24: 0 → 1
Char. 27: 0 → 1
Char. 34: 0 → 1
Char. 36: 0 → 1
Char. 56: 0 → 1
Char. 59: 0 → 1
Char. 60: 0 → 1
Char. 62: 0 → 1
Char. 64: 0 → 1
Char. 70: 0 → 1
Char. 72: 1 → 2
Char. 73: 0 → 1
Char. 84: 1 → 0
Char. 89: 0 → 1
Char. 94: 0 → 1
Char. 105: 0 → 1
Char. 106: 0 → 1
Char. 109: 0 → 1
Char. 117: 01 → 2
Char. 126: 0 → 1
Char. 129: 0 → 1
Char. 130: 0 → 1
Char. 131: 0 → 1
Char. 144: 1 → 0
Char. 154: 1 → 0
Char. 166: 1 → 0
Char. 182: 0 → 1
Char. 188: 0 → 1
Char. 190: 0 → 1
Char. 199: 0 → 1
Char. 203: 1 → 2
Char. 204: 02 → 1
Char. 214: 0 → 1
Char. 219: 0 → 1
Char. 220: 0 → 1
Char. 265: 0 → 1
Char. 267: 0 → 1
Char. 272: 2 → 1
Char. 275: 1 → 0
Char. 278: 3 → 0

***Colobomycter pholeter*:**
Char. 21: 0 → 1
Char. 25: 1 → 0
Char. 84: 1 → 0
Char. 154: 1 → 0
Char. 167: 0 → 1
Char. 267: 0 → 1

***Delorhynchus cifelli*:**
Char. 0: 1 → 0
Char. 18: 0 → 1
Char. 21: 0 → 1
Char. 24: 0 → 1
Char. 26: 0 → 1
Char. 28: 0 → 1
Char. 33: 1 → 2
Char. 39: 0 → 1
Char. 50: 0 → 1
Char. 52: 0 → 1
Char. 57: 0 → 1
Char. 100: 0 → 1
Char. 116: 1 → 0
Char. 119: 1 → 0
Char. 131: 0 → 1
Char. 147: 0 → 1
Char. 156: 1 → 0
Char. 167: 0 → 1
Char. 189: 0 → 1
Char. 196: 1 → 0
Char. 202: 1 → 0
Char. 267: 0 → 1

**Diadectomorpha:**
Char. 64: 0 → 1
Char. 70: 0 → 1
Char. 122: 0 → 1
Char. 123: 1 → 0
Char. 146: 1 → 0
Char. 275: 1 → 0
Char. 278: 3 → 0

***Emeroleter levis*:**
Char. 51: 1 → 0

**Eosauropterygia:**
Char. 159: 1 → 0
Char. 166: 1 → 0
Char. 174: 0 → 1
Char. 194: 0 → 2
Char. 272: 1 → 0

***Eudibamus cursoris*:**
Char. 154: 1 → 2
Char. 163: 1 → 0

***Feeserpeton oklahomensis*:**
Char. 51: 0 → 1
Char. 157: 0 → 1
Char. 158: 0 → 1

***Hovasaurus boulei*:**
Char. 41: 0 → 1
Char. 43: 0 → 1
Char. 55: 1 → 0
Char. 59: 0 → 1
Char. 77: 0 → 2
Char. 78: 1 → 0
Char. 79: 0 → 1
Char. 89: 0 → 1
Char. 93: 1 → 0
Char. 113: 0 → 1
Char. 127: 0 → 1
Char. 135: 0 → 1
Char. 136: 0 → 1
Char. 138: 0 → 1
Char. 146: 1 → 0
Char. 188: 0 → 1
Char. 193: 0 → 1
Char. 206: 0 → 2
Char. 215: 0 → 1
Char. 220: 0 → 1
Char. 222: 0 → 1
Char. 224: 0 → 1
Char. 265: 0 → 1
Char. 275: 1 → 0

**Kuehneosauridae:**
Char. 7: 0 → 1
Char. 24: 0 → 1
Char. 27: 0 → 1
Char. 34: 0 → 1
Char. 36: 0 → 1
Char. 44: 1 → 0

Char. 79: 0 → 2
Char. 159: 1 → 0
Char. 185: 0 → 1
Char. 245: 0 → 1

***Lanthanosuchus watsoni*:**
Char. 25: 1 → 0
Char. 51: 0 → 1
Char. 76: 0 → 1
Char. 86: 0 → 1
Char. 98: 1 → 0
Char. 138: 0 → 1
Char. 144: 1 → 0
Char. 154: 1 → 2

***Macroleter poezicus*:**
Char. 9: 0 → 1
Char. 26: 0 → 1
Char. 66: 1 → 2
Char. 126: 0 → 1
Char. 134: 0 → 2
Char. 140: 0 → 1
Char. 146: 1 → 0
Char. 169: 1 → 0

***Mesosaurus* spp.:**
Char. 2: 0 → 1
Char. 5: 1 → 0
Char. 6: 0 → 1
Char. 8: 0 → 1
Char. 9: 0 → 1
Char. 13: 0 → 1
Char. 19: 0 → 1
Char. 20: 1 → 0
Char. 26: 0 → 1
Char. 38: 1 → 0
Char. 41: 0 → 1
Char. 76: 0 → 1
Char. 83: 0 → 1
Char. 84: 1 → 0
Char. 85: 1 → 0
Char. 94: 0 → 1
Char. 107: 0 → 1
Char. 109: 0 → 1
Char. 112: 1 → 0
Char. 115: 0 → 1
Char. 146: 1 → 0
Char. 149: 1 → 0
Char. 159: 1 → 0
Char. 163: 1 → 0
Char. 164: 0 → 1
Char. 166: 1 → 0
Char. 167: 0 → 1
Char. 176: 0 → 1
Char. 183: 1 → 0
Char. 184: 0 → 1
Char. 192: 1 → 0
Char. 196: 1 → 0
Char. 199: 0 → 1
Char. 206: 0 → 1
Char. 217: 0 → 1
Char. 219: 0 → 1
Char. 220: 0 → 1
Char. 231: 0 → 1

Char. 240: 1 → 0
Char. 260: 2 → 0
Char. 272: 2 → 0
Char. 278: 3 → 0

***Microleter mckinzieorum*:**
Char. 18: 0 → 1
Char. 20: 1 → 0
Char. 24: 0 → 1
Char. 25: 1 → 0
Char. 33: 1 → 0
Char. 36: 0 → 1
Char. 39: 0 → 1
Char. 48: 1 → 0
Char. 50: 0 → 1
Char. 51: 0 → 2
Char. 56: 0 → 1
Char. 70: 0 → 1
Char. 76: 0 → 1
Char. 79: 0 → 1
Char. 83: 0 → 1
Char. 94: 0 → 1
Char. 106: 0 → 1
Char. 110: 0 → 1
Char. 111: 1 → 0
Char. 112: 1 → 0
Char. 132: 1 → 0
Char. 166: 1 → 0
Char. 276: 0 → 1
Char. 278: 3 → 1

**Millerettidae:**
Char. 0: 1 → 0
Char. 5: 1 → 0
Char. 20: 1 → 0
Char. 24: 0 → 1
Char. 25: 1 → 0
Char. 33: 1 → 0
Char. 44: 1 → 0
Char. 48: 1 → 0
Char. 50: 0 → 1
Char. 56: 0 → 1
Char. 66: 0 → 2
Char. 78: 1 → 0
Char. 80: 1 → 0
Char. 84: 1 → 2
Char. 88: 1 → 0
Char. 96: 1 → 0
Char. 111: 1 → 0
Char. 112: 1 → 0
Char. 120: 1 → 0
Char. 121: 0 → 1
Char. 124: 0 → 1
Char. 127: 0 → 1
Char. 135: 0 → 1
Char. 145: 0 → 1
Char. 159: 1 → 0
Char. 163: 1 → 0
Char. 166: 1 → 0
Char. 180: 1 → 0
Char. 192: 1 → 0
Char. 211: 0 → 1
Char. 230: 0 → 1
Char. 248: 0 → 1

Char. 252: 0 → 1
Char. 253: 0 → 1

***Nycteroleter ineptus*:**
Char. 66: 1 → 0

***Nyctiphruretus acudens*:**
Char. 21: 0 → 1
Char. 33: 1 → 2
Char. 41: 0 → 1
Char. 48: 1 → 0
Char. 66: 1 → 2
Char. 81: 1 → 0
Char. 83: 0 → 1
Char. 84: 1 → 2
Char. 94: 0 → 1
Char. 166: 1 → 0
Char. 207: 1 → 0
Char. 224: 0 → 1
Char. 266: 0 → 1
Char. 272: 2 → 1
Char. 276: 0 → 1

***Orovenator mayorum*:**
Char. 8: 0 → 1
Char. 24: 0 → 1
Char. 25: 1 → 0
Char. 33: 1 → 0
Char. 36: 0 → 1
Char. 48: 1 → 0
Char. 50: 0 → 1
Char. 62: 0 → 1
Char. 72: 1 → 2
Char. 89: 0 → 1
Char. 92: 1 → 0
Char. 136: 0 → 1
Char. 141: 0 → 1
Char. 159: 1 → 0
Char. 160: 0 → 1
Char. 165: 0 → 1
Char. 278: 3 → 1

***Owenetta* spp.:**
Char. 142: 1 → 0
Char. 169: 1 → 0

***Paleothyris acadiana*:**
Char. 66: 1 → 2
Char. 102: 0 → 1
Char. 146: 1 → 0

***Placodus* spp.:**
Char. 0: 1 → 2
Char. 9: 0 → 1
Char. 12: 0 → 1
Char. 13: 0 → 1
Char. 19: 0 → 1
Char. 31: 0 → 1
Char. 44: 1 → 0
Char. 46: 1 → 0
Char. 57: 0 → 1
Char. 78: 1 → 0
Char. 93: 1 → 0
Char. 102: 1 → 2
Char. 140: 1 → 0

Char. 155: 0 → 1
Char. 163: 1 → 0
Char. 164: 0 → 1

***Procolophon* spp.:**
Char. 41: 0 → 1
Char. 69: 0 → 1
Char. 79: 0 → 1
Char. 83: 0 → 1
Char. 86: 0 → 1
Char. 101: 0 → 1
Char. 141: 0 → 1
Char. 149: 1 → 0
Char. 203: 1 → 2
Char. 215: 0 → 1
Char. 235: 1 → 2
Char. 238: 0 → 1
Char. 272: 2 → 1
Char. 278: 3 → 0

***Prolacerta broomi*:**
Char. 58: 1 → 0
Char. 66: 1 → 0
Char. 67: 1 → 0
Char. 80: 1 → 0
Char. 139: 1 → 0
Char. 192: 1 → 0
Char. 203: 1 → 2
Char. 206: 0 → 12

***Rhipaeosaurus* spp.:**
Char. 120: 1 → 0
Char. 172: 0 → 1
Char. 183: 1 → 2
Char. 194: 0 → 1
Char. 207: 1 → 0
Char. 211: 0 → 1
Char. 215: 0 → 1
Char. 226: 0 → 1
Char. 235: 1 → 0
Char. 240: 1 → 0
Char. 252: 0 → 1
Char. 277: 0 → 1

**Rhynchocephalia:**
Char. 0: 1 → 2
Char. 24: 0 → 1
Char. 77: 0 → 1
Char. 94: 1 → 0
Char. 139: 1 → 0
Char. 167: 1 → 0
Char. 205: 1 → 0

**Rhynchosauria:**
Char. 0: 1 → 0
Char. 7: 0 → 1
Char. 9: 0 → 1
Char. 26: 0 → 1
Char. 44: 1 → 0
Char. 68: 0 → 1
Char. 99: 1 → 0
Char. 150: 1 → 0
Char. 160: 0 → 1
Char. 161: 0 → 1
Char. 171: 0 → 2

Char. 223: 0 → 1
Char. 224: 0 → 1
Char. 241: 0 → 1

***Scutosaurus* spp.:**
Char. 175: 0 → 1
Char. 190: 0 → 1
Char. 218: 0 → 1
Char. 243: 0 → 2
Char. 244: 0 → 1
Char. 251: 0 → 1

***Sinosaurosphargis yunguiensis*:**
Char. 8: 0 → 1
Char. 30: 0 → 1
Char. 53: 0 → 1
Char. 89: 1 → 0
Char. 127: 1 → 0
Char. 150: 1 → 0
Char. 154: 0 → 2
Char. 167: 1 → 0
Char. 253: 0 → 1
Char. 255: 0 → 1

**Squamata:**
Char. 45: 0 → 1
Char. 79: 0 → 2
Char. 80: 1 → 0
Char. 92: 1 → 0
Char. 160: 0 → 1
Char. 245: 0 → 1

***Trilophosaurus buettneri*:**
Char. 5: 1 → 0
Char. 11: 0 → 1
Char. 55: 1 → 0
Char. 93: 1 → 0
Char. 104: 0 → 1
Char. 106: 0 → 1
Char. 113: 0 → 1
Char. 122: 0 → 1
Char. 144: 1 → 0
Char. 157: 0 → 1
Char. 159: 1 → 0
Char. 177: 0 → 12
Char. 194: 0 → 1
Char. 203: 1 → 2
Char. 207: 1 → 0
Char. 208: 1 → 0
Char. 272: 1 → 0

***Youngina capensis*:**
Char. 5: 1 → 0
Char. 21: 0 → 1
Char. 27: 0 → 1
Char. 38: 1 → 0
Char. 40: 2 → 0
Char. 43: 0 → 1
Char. 44: 1 → 0
Char. 56: 0 → 1
Char. 59: 0 → 1
Char. 60: 0 → 1
Char. 62: 0 → 1
Char. 72: 1 → 2

Char. 75: 0 → 1
Char. 84: 1 → 0
Char. 89: 0 → 1
Char. 92: 1 → 0
Char. 109: 0 → 1
Char. 127: 0 → 1
Char. 129: 0 → 1
Char. 134: 0 → 1
Char. 135: 0 → 1
Char. 141: 0 → 1
Char. 154: 1 → 0
Char. 163: 1 → 0
Char. 170: 0 → 1
Char. 193: 0 → 1
Char. 211: 0 → 1
Char. 214: 0 → 1
Char. 215: 0 → 1
Char. 219: 0 → 1
Char. 222: 0 → 1
Char. 224: 0 → 1
Char. 231: 0 → 1
Char. 239: 0 → 1
Char. 265: 0 → 1
Char. 267: 0 → 1
Char. 275: 1 → 0
Char. 278: 3 → 0

**Node 50:**
Char. 46: 1 → 0
Char. 88: 1 → 0
Char. 89: 1 → 0
Char. 93: 1 → 0
Char. 176: 0 → 1
Char. 195: 0 → 2
Char. 198: 0 → 1
Char. 246: 1 → 2
Char. 259: 0 → 1
Char. 270: 0 → 1

**Node 51:**
Char. 65: 0 → 1
Char. 72: 1 → 2
Char. 131: 0 → 1
Char. 184: 0 → 1
Char. 205: 0 → 1
Char. 210: 0 → 1
Char. 222: 0 → 1
Char. 241: 0 → 1
Char. 246: 0 → 1
Char. 254: 0 → 1
Char. 255: 0 → 1
Char. 256: 0 → 1
Char. 267: 0 → 1
Char. 268: 0 → 1
Char. 269: 0 → 1
Char. 278: 3 → 0

**Node 52:**
Char. 21: 0 → 1
Char. 84: 1 → 2

**Node 53:**
Char. 15: 0 → 1
Char. 25: 1 → 0
Char. 33: 1 → 2
Char. 48: 1 → 0
Char. 55: 1 → 0
Char. 89: 0 → 1
Char. 94: 0 → 1
Char. 127: 0 → 1

**Node 54:**
Char. 5: 0 → 1
Char. 20: 0 → 1
Char. 33: 0 → 1
Char. 40: 0 → 2
Char. 48: 0 → 1
Char. 57: 1 → 0
Char. 74: 0 → 1
Char. 80: 0 → 1
Char. 84: 0 → 1
Char. 88: 0 → 1
Char. 112: 0 → 1
Char. 116: 0 → 1
Char. 119: 0 → 1
Char. 132: 0 → 1
Char. 149: 0 → 1
Char. 156: 0 → 1
Char. 163: 0 → 1
Char. 166: 0 → 1
Char. 169: 0 → 1
Char. 179: 0 → 1
Char. 180: 0 → 1
Char. 192: 0 → 1
Char. 196: 0 → 1
Char. 202: 0 → 1
Char. 203: 0 → 1
Char. 209: 0 → 1
Char. 221: 0 → 1
Char. 235: 0 → 1

**Node 55:**
Char. 72: 0 → 1
Char. 79: 1 → 0
Char. 81: 0 → 1
Char. 93: 0 → 1
Char. 97: 0 → 1
Char. 104: 1 → 0
Char. 144: 0 → 1
Char. 173: 0 → 1
Char. 183: 0 → 1

**Node 57:**
Char. 95: 0 → 1
Char. 113: 0 → 1
Char. 114: 0 → 1

**Node 58:**
Char. 0: 1 → 0
Char. 47: 0 → 1
Char. 50: 0 → 1
Char. 57: 0 → 1
Char. 79: 0 → 1
Char. 110: 0 → 1
Char. 111: 1 → 0
Char. 112: 1 → 0
Char. 131: 0 → 1
Char. 137: 0 → 1
Char. 140: 0 → 2
Char. 147: 0 → 1

**Node 59:**
Char. 5: 1 → 0
Char. 20: 1 → 0
Char. 33: 1 → 0
Char. 59: 0 → 1
Char. 60: 0 → 1
Char. 72: 1 → 2
Char. 84: 1 → 0
Char. 116: 1 → 0
Char. 154: 1 → 0
Char. 159: 1 → 0
Char. 166: 1 → 0
Char. 169: 1 → 0
Char. 170: 0 → 1
Char. 179: 1 → 0
Char. 196: 1 → 0
Char. 197: 0 → 1
Char. 202: 1 → 0
Char. 207: 1 → 0
Char. 209: 1 → 0
Char. 221: 1 → 0

**Node 60:**
Char. 19: 0 → 1
Char. 92: 1 → 0

**Node 61:**
Char. 4: 0 → 1
Char. 15: 0 → 1
Char. 48: 1 → 0
Char. 213: 0 → 1
Char. 226: 0 → 2
Char. 228: 0 → 1

**Node 62:**
Char. 25: 1 → 0
Char. 58: 0 → 1
Char. 59: 0 → 1
Char. 60: 0 → 1
Char. 61: 0 → 1
Char. 62: 0 → 1
Char. 66: 0 → 1
Char. 69: 0 → 1
Char. 72: 1 → 2
Char. 73: 0 → 1
Char. 89: 0 → 1
Char. 94: 0 → 1
Char. 126: 0 → 1
Char. 127: 0 → 1
Char. 129: 0 → 1
Char. 131: 0 → 1
Char. 134: 0 → 1
Char. 135: 0 → 1
Char. 141: 0 → 1
Char. 145: 0 → 1
Char. 150: 0 → 1
Char. 151: 0 → 1
Char. 167: 0 → 1
Char. 176: 0 → 1
Char. 188: 0 → 1
Char. 190: 0 → 1
Char. 205: 0 → 1
Char. 208: 0 → 1
Char. 214: 0 → 1
Char. 219: 0 → 1
Char. 230: 0 → 1
Char. 239: 0 → 1
Char. 260: 2 → 1
Char. 265: 0 → 1

**Node 63:**
Char. 73: 0 → 1
Char. 205: 0 → 1
Char. 273: 0 → 1
Char. 276: 0 → 1

**Node 64:**
Char. 0: 1 → 0
Char. 18: 0 → 1
Char. 25: 1 → 0
Char. 37: 0 → 1
Char. 103: 0 → 1
Char. 106: 0 → 1
Char. 107: 0 → 1
Char. 126: 0 → 1
Char. 150: 0 → 1

**Node 65:**
Char. 38: 1 → 2
Char. 39: 0 → 1
Char. 44: 1 → 0
Char. 49: 0 → 1
Char. 57: 0 → 1
Char. 66: 0 → 1
Char. 76: 0 → 1
Char. 80: 1 → 0
Char. 95: 0 → 1
Char. 111: 1 → 0
Char. 120: 1 → 0
Char. 135: 0 → 1
Char. 137: 0 → 1
Char. 147: 0 → 1
Char. 157: 0 → 1
Char. 158: 0 → 1
Char. 179: 1 → 0
Char. 183: 1 → 2
Char. 186: 0 → 1
Char. 194: 0 → 1
Char. 197: 0 → 1
Char. 202: 1 → 0
Char. 209: 1 → 0
Char. 211: 0 → 1
Char. 240: 1 → 0
Char. 252: 0 → 1

**Node 67:**
Char. 133: 0 → 1

**Node 68:**
Char. 87: 0 → 1
Char. 100: 0 → 1
Char. 113: 0 → 1
Char. 141: 0 → 1
Char. 143: 0 → 1
Char. 215: 0 → 1

**Node 69:**
Char. 50: 0 → 1
Char. 70: 0 → 1
Char. 118: 0 → 1
Char. 125: 0 → 1

**Node 70:**
Char. 87: 1 → 0
Char. 110: 1 → 0

**Node 71:**
Char. 20: 1 → 0
Char. 38: 1 → 2
Char. 39: 0 → 1
Char. 57: 0 → 1
Char. 58: 0 → 1
Char. 59: 0 → 1
Char. 60: 0 → 1
Char. 72: 1 → 2
Char. 83: 0 → 1
Char. 85: 1 → 0
Char. 88: 1 → 0
Char. 95: 0 → 1
Char. 104: 0 → 1
Char. 105: 0 → 1
Char. 106: 0 → 2
Char. 107: 0 → 1
Char. 109: 0 → 1
Char. 110: 0 → 1
Char. 146: 1 → 0
Char. 155: 0 → 2
Char. 183: 1 → 2

**Node 72:**
Char. 23: 0 → 1
Char. 33: 1 → 0
Char. 38: 2 → 1
Char. 39: 1 → 0
Char. 42: 0 → 1
Char. 43: 0 → 1
Char. 46: 1 → 0
Char. 48: 1 → 0
Char. 49: 1 → 0
Char. 71: 1 → 0
Char. 83: 0 → 2
Char. 101: 0 → 1
Char. 103: 0 → 1
Char. 106: 0 → 1
Char. 161: 0 → 1
Char. 163: 1 → 0
Char. 172: 0 → 2
Char. 174: 0 → 1
Char. 188: 0 → 1
Char. 189: 0 → 1
Char. 203: 1 → 2
Char. 212: 0 → 1
Char. 216: 1 → 0
Char. 221: 1 → 0
Char. 235: 01 → 2
Char. 236: 0 → 1
Char. 238: 0 → 2
Char. 241: 0 → 1
Char. 242: 0 → 1
Char. 273: 0 → 1

Char. 274: 0 → 1
Char. 275: 1 → 0

**Node 73:**
Char. 0: 1 → 0
Char. 44: 1 → 0
Char. 48: 1 → 0
Char. 66: 0 → 1
Char. 78: 1 → 0
Char. 88: 1 → 0
Char. 93: 1 → 0
Char. 112: 1 → 0
Char. 123: 1 → 0
Char. 129: 0 → 1

Char. 163: 1 → 0
Char. 192: 1 → 0

**Node 74:**
Char. 33: 1 → 0
Char. 83: 0 → 1
Char. 85: 1 → 0
Char. 107: 0 → 1

**Node 75:**
Char. 2: 0 → 1
Char. 6: 0 → 1
Char. 101: 0 → 1
Char. 186: 0 → 1
Char. 198: 0 → 1

Char. 220: 0 → 1
Char. 239: 1 → 0
Char. 240: 1 → 0

**Node 76:**
Char. 21: 0 → 1
Char. 33: 1 → 2
Char. 68: 0 → 1
Char. 235: 1 → 2

**Node 77:**
Char. 35: 0 → 1
Char. 184: 0 → 1
Char. 210: 0 → 1

**Node 78:**
Char. 17: 0 → 1
Char. 42: 0 → 1
Char. 251: 0 → 1
Char. 264: 0 → 1

**Node 79:**
Char. 41: 0 → 1
Char. 48: 1 → 0
Char. 61: 1 → 2
Char. 112: 1 → 0
Char. 138: 0 → 1
Char. 146: 1 → 0
Char. 155: 0 → 1
Char. 192: 1 → 2

Char. 224: 0 → 1
Char. 226: 0 → 1
Char. 227: 0 → 1
Char. 233: 0 → 1

**Node 80:**
Char. 61: 1 → 3
Char. 90: 0 → 1
Char. 91: 0 → 1
Char. 155: 0 → 1
Char. 209: 1 → 0

Analysis 34
(all taxa, implied weighting, K = 2)

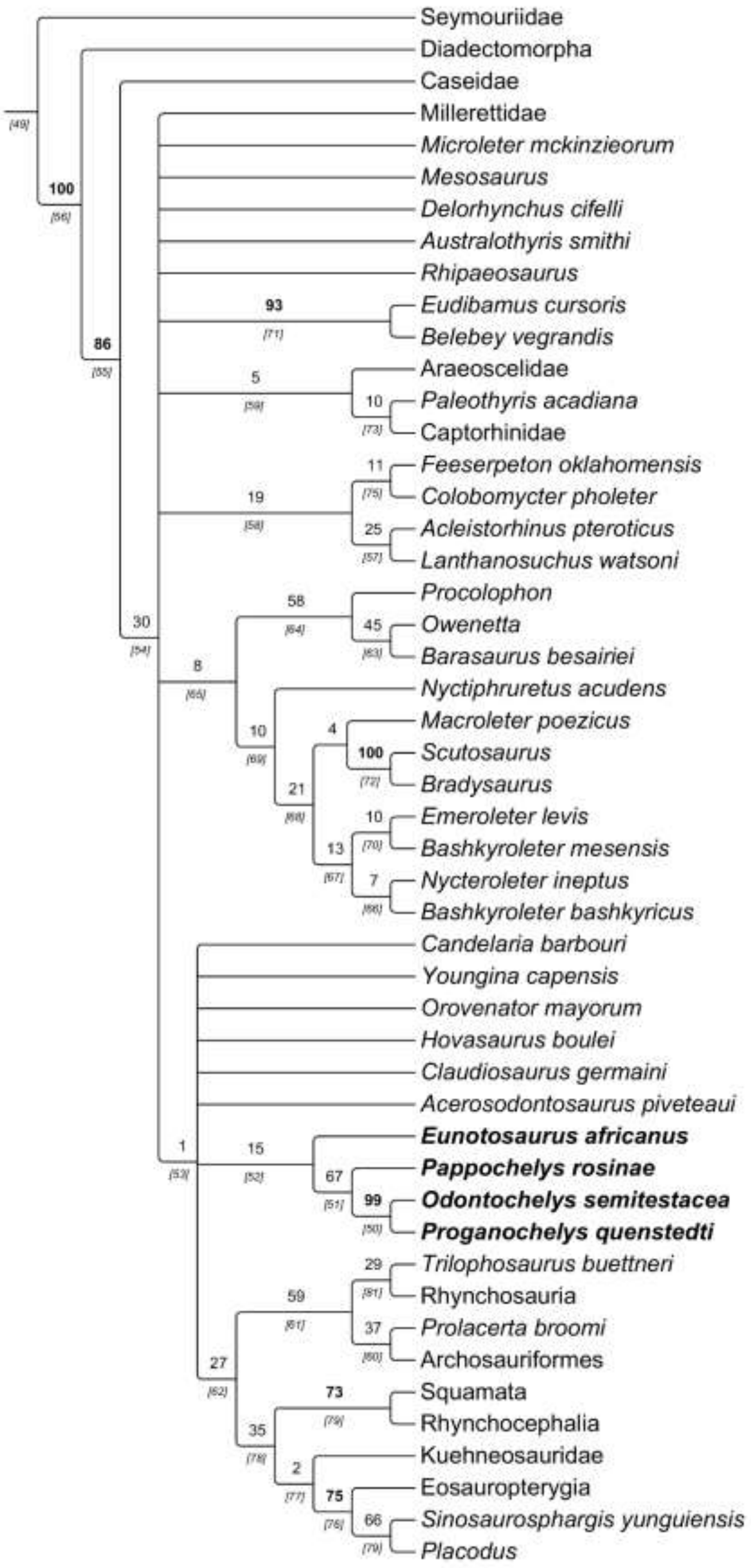

***Proganochelys quenstedti*:**
Char. 8: 0 → 1
Char. 11: 0 → 1
Char. 106: 0 → 1
Char. 108: 1 → 0
Char. 128: 0 → 1
Char. 175: 0 → 1
Char. 202: 1 → 0
Char. 207: 1 → 0
Char. 209: 1 → 0
Char. 244: 0 → 1
Char. 248: 0 → 1
Char. 250: 0 → 1
Char. 252: 1 → 0
Char. 262: 0 → 1

***Pappochelys rosinae*:**
Char. 0: 2 → 0
Char. 1: 0 → 1
Char. 5: 1 → 0
Char. 12: 0 → 1
Char. 41: 0 → 1
Char. 48: 0 → 1
Char. 49: 0 → 1
Char. 75: 0 → 1
Char. 169: 1 → 0
Char. 260: 2 → 0

***Odontochelys semitestacea*:**
Char. 43: 0 → 1

***Eunotosaurus africanus*:**
Char. 19: 0 → 1
Char. 43: 0 → 1
Char. 59: 1 → 0
Char. 72: 2 → 1
Char. 76: 0 → 1
Char. 97: 1 → 0
Char. 103: 0 → 1
Char. 153: 1 → 0
Char. 191: 1 → 0
Char. 192: 1 → 0
Char. 202: 1 → 0
Char. 211: 0 → 1
Char. 222: 1 → 0
Char. 237: 1 → 0
Char. 248: 0 → 1
Char. 249: 0 → 2
Char. 250: 0 → 1
Char. 263: 0 → 1
Char. 273: 0 → 1
Char. 274: 0 → 1
Char. 275: 0 → 1
Char. 276: 0 → 1
Char. 277: 0 → 1

***Acerosodontosaurus piveteaui*:**
Char. 78: 1 → 0
Char. 81: 1 → 0
Char. 127: 1 → 0
Char. 128: 0 → 1
Char. 155: 0 → 1
Char. 206: 0 → 2
Char. 208: 0 → 1
Char. 278: 03 → 1

***Acleistorhinus pteroticus*:**
Char. 21: 0 → 1
Char. 146: 1 → 0

**Araeoscelidae:**
Char. 0: 0 → 1
Char. 28: 0 → 1
Char. 43: 0 → 1
Char. 48: 0 → 1
Char. 57: 1 → 0
Char. 89: 0 → 1
Char. 106: 0 → 1
Char. 112: 0 → 1
Char. 120: 0 → 1
Char. 193: 0 → 1
Char. 222: 0 → 1
Char. 224: 0 → 1
Char. 234: 0 → 1
Char. 266: 0 → 1
Char. 278: 3 → 1

**Archosauriformes:**
Char. 32: 0 → 1
Char. 94: 1 → 0
Char. 112: 1 → 0
Char. 152: 0 → 1
Char. 154: 01 → 2
Char. 166: 1 → 0
Char. 171: 0 → 1
Char. 185: 0 → 1
Char. 201: 0 → 1
Char. 204: 0 → 1
Char. 218: 0 → 3
Char. 224: 0 → 1
Char. 242: 0 → 1

***Australothyris smithi*:**
Char. 24: 0 → 1
Char. 34: 0 → 1
Char. 55: 1 → 0
Char. 57: 1 → 0
Char. 71: 1 → 0
Char. 79: 0 → 1
Char. 83: 0 → 1
Char. 98: 1 → 0
Char. 100: 0 → 1
Char. 103: 0 → 1
Char. 110: 0 → 1
Char. 112: 0 → 1
Char. 123: 1 → 0
Char. 129: 0 → 1
Char. 131: 0 → 1
Char. 132: 1 → 0
Char. 144: 1 → 0
Char. 147: 0 → 1
Char. 149: 1 → 0
Char. 150: 0 → 1
Char. 159: 1 → 0
Char. 192: 1 → 0

***Barasaurus besairiei*:**
Char. 33: 1 → 0

***Bashkyroleter bashkyricus*:**
Char. 275: 1 → 0

***Bashkyroleter mesensis*:**
Char. 12: 0 → 1
Char. 66: 1 → 0
Char. 169: 1 → 0

***Belebey vegrandis*:**
Char. 154: 1 → 0

***Bradysaurus* spp.:**
Char. 19: 0 → 1
Char. 25: 1 → 0
Char. 73: 0 → 1
Char. 79: 0 → 1
Char. 135: 1 → 0
Char. 249: 0 → 1

***Candelaria barbouri*:**
Char. 0: 1 → 0
Char. 1: 0 → 1
Char. 5: 1 → 0
Char. 8: 0 → 1
Char. 15: 0 → 1
Char. 33: 1 → 2
Char. 49: 0 → 1
Char. 55: 1 → 0
Char. 59: 1 → 0
Char. 62: 1 → 0
Char. 72: 2 → 1
Char. 76: 0 → 1
Char. 79: 0 → 2
Char. 83: 0 → 1
Char. 88: 1 → 0
Char. 92: 1 → 0
Char. 95: 0 → 1
Char. 126: 0 → 1
Char. 132: 1 → 0
Char. 154: 1 → 2
Char. 159: 1 → 0
Char. 169: 1 → 0
Char. 265: 1 → 0
Char. 275: 0 → 1
Char. 276: 0 → 1
Char. 277: 0 → 1

**Captorhinidae:**
Char. 3: 0 → 1
Char. 25: 1 → 0
Char. 26: 0 → 1
Char. 73: 0 → 1
Char. 75: 0 → 1
Char. 83: 0 → 1
Char. 108: 1 → 0
Char. 180: 1 → 0
Char. 183: 1 → 0
Char. 201: 1 → 0
Char. 203: 1 → 2
Char. 216: 1 → 0

**Caseidae:**
Char. 24: 0 → 1
Char. 25: 1 → 0
Char. 36: 0 → 1
Char. 38: 1 → 0
Char. 46: 1 → 0
Char. 50: 1 → 0
Char. 56: 0 → 1
Char. 170: 0 → 1
Char. 194: 0 → 1
Char. 273: 0 → 1
Char. 274: 0 → 1
Char. 278: 3 → 2

***Claudiosaurus germaini*:**
Char. 24: 0 → 1
Char. 27: 0 → 1
Char. 34: 0 → 1
Char. 36: 0 → 1
Char. 48: 0 → 1
Char. 56: 0 → 1
Char. 64: 0 → 1
Char. 70: 0 → 1
Char. 84: 1 → 0
Char. 105: 0 → 1
Char. 106: 0 → 1
Char. 117: 01 → 2
Char. 126: 0 → 1
Char. 127: 1 → 0
Char. 130: 0 → 1
Char. 131: 0 → 1
Char. 141: 1 → 0
Char. 144: 1 → 0
Char. 154: 1 → 0
Char. 166: 1 → 0
Char. 182: 0 → 1
Char. 187: 1 → 0
Char. 190: 0 → 1
Char. 199: 0 → 1
Char. 201: 0 → 1
Char. 203: 1 → 2
Char. 204: 02 → 1
Char. 222: 1 → 0
Char. 234: 1 → 0
Char. 267: 0 → 1
Char. 272: 2 → 1

***Colobomycter pholeter*:**
Char. 21: 0 → 1
Char. 25: 1 → 0
Char. 84: 1 → 0
Char. 154: 1 → 0
Char. 167: 0 → 1
Char. 267: 0 → 1

***Delorhynchus cifelli*:**
Char. 18: 0 → 1
Char. 20: 0 → 1
Char. 21: 0 → 1
Char. 24: 0 → 1
Char. 26: 0 → 1
Char. 28: 0 → 1
Char. 33: 0 → 2
Char. 39: 0 → 1
Char. 48: 0 → 1
Char. 52: 0 → 1
Char. 100: 0 → 1
Char. 111: 0 → 1
Char. 116: 1 → 0
Char. 119: 1 → 0
Char. 131: 0 → 1
Char. 147: 0 → 1
Char. 156: 1 → 0
Char. 167: 0 → 1
Char. 189: 0 → 1
Char. 191: 0 → 1
Char. 267: 0 → 1

**Diadectomorpha:**
Char. 0: 0 → 1
Char. 64: 0 → 1
Char. 70: 0 → 1
Char. 122: 0 → 1
Char. 123: 1 → 0
Char. 146: 1 → 0
Char. 275: 1 → 0
Char. 278: 3 → 0

***Emeroleter levis*:**
Char. 51: 1 → 0

**Eosauropterygia:**
Char. 166: 1 → 0
Char. 174: 0 → 1
Char. 178: 0 → 1
Char. 194: 0 → 2
Char. 272: 1 → 0

***Eudibamus cursoris*:**
Char. 154: 1 → 2

***Feeserpeton oklahomensis*:**
Char. 51: 0 → 1
Char. 157: 0 → 1
Char. 158: 0 → 1

***Hovasaurus boulei*:**
Char. 41: 0 → 1
Char. 43: 0 → 1
Char. 48: 0 → 1
Char. 55: 1 → 0
Char. 72: 2 → 1
Char. 77: 0 → 2
Char. 78: 1 → 0
Char. 79: 0 → 1
Char. 93: 1 → 0
Char. 113: 0 → 1
Char. 138: 0 → 1
Char. 141: 1 → 0
Char. 146: 1 → 0
Char. 206: 0 → 2
Char. 215: 0 → 1
Char. 219: 1 → 0
Char. 224: 0 → 1

**Kuehneosauridae:**
Char. 7: 0 → 1
Char. 24: 0 → 1
Char. 27: 0 → 1

Char. 43: 1 → 0
Char. 79: 0 → 2
Char. 107: 1 → 0
Char. 140: 1 → 0
Char. 147: 1 → 0
Char. 148: 1 → 0
Char. 245: 0 → 1
Char. 278: 0 → 3

***Lanthanosuchus watsoni*:**
Char. 25: 1 → 0
Char. 51: 0 → 1
Char. 76: 0 → 1
Char. 86: 0 → 1
Char. 98: 1 → 0
Char. 138: 0 → 1
Char. 144: 1 → 0
Char. 154: 1 → 2

***Macroleter poezicus*:**
Char. 9: 0 → 1
Char. 26: 0 → 1
Char. 66: 1 → 2
Char. 126: 0 → 1
Char. 134: 0 → 2
Char. 140: 0 → 1
Char. 146: 1 → 0
Char. 169: 1 → 0

***Mesosaurus* spp.:**
Char. 0: 0 → 1
Char. 2: 0 → 1
Char. 5: 1 → 0
Char. 6: 0 → 1
Char. 8: 0 → 1
Char. 9: 0 → 1
Char. 13: 0 → 1
Char. 19: 0 → 1
Char. 26: 0 → 1
Char. 29: 1 → 0
Char. 33: 0 → 1
Char. 38: 1 → 0
Char. 41: 0 → 1
Char. 48: 0 → 1
Char. 50: 1 → 0
Char. 67: 0 → 1
Char. 76: 0 → 1
Char. 83: 0 → 1
Char. 84: 1 → 0
Char. 94: 0 → 1
Char. 107: 0 → 1
Char. 109: 0 → 1
Char. 111: 0 → 1
Char. 115: 0 → 1
Char. 146: 1 → 0
Char. 149: 1 → 0
Char. 159: 1 → 0
Char. 164: 0 → 1
Char. 166: 1 → 0
Char. 167: 0 → 1
Char. 176: 0 → 1
Char. 183: 1 → 0
Char. 184: 0 → 1
Char. 192: 1 → 0
Char. 199: 0 → 1
Char. 202: 0 → 1
Char. 206: 0 → 1
Char. 209: 0 → 1
Char. 217: 0 → 1
Char. 219: 0 → 1
Char. 220: 0 → 1
Char. 231: 0 → 1
Char. 260: 2 → 0
Char. 272: 2 → 0
Char. 278: 3 → 0

***Microleter mckinzieorum*:**
Char. 0: 0 → 1
Char. 18: 0 → 1
Char. 24: 0 → 1
Char. 25: 1 → 0
Char. 36: 0 → 1
Char. 39: 0 → 1
Char. 51: 0 → 2
Char. 56: 0 → 1
Char. 57: 1 → 0
Char. 70: 0 → 1
Char. 76: 0 → 1
Char. 79: 0 → 1
Char. 83: 0 → 1
Char. 94: 0 → 1
Char. 106: 0 → 1
Char. 110: 0 → 1
Char. 132: 1 → 0
Char. 166: 1 → 0
Char. 276: 0 → 1
Char. 278: 3 → 1

**Millerettidae:**
Char. 5: 1 → 0
Char. 24: 0 → 1
Char. 25: 1 → 0
Char. 44: 1 → 0
Char. 56: 0 → 1
Char. 57: 1 → 0
Char. 66: 0 → 2
Char. 78: 1 → 0
Char. 80: 1 → 0
Char. 84: 1 → 2
Char. 88: 1 → 0
Char. 96: 1 → 0
Char. 121: 0 → 1
Char. 124: 0 → 1
Char. 127: 0 → 1
Char. 135: 0 → 1
Char. 145: 0 → 1
Char. 159: 1 → 0
Char. 166: 1 → 0
Char. 180: 1 → 0
Char. 192: 1 → 0
Char. 202: 0 → 1
Char. 211: 0 → 1
Char. 230: 0 → 1
Char. 234: 0 → 1
Char. 248: 0 → 1
Char. 252: 0 → 1
Char. 253: 0 → 1

***Nycteroleter ineptus*:**
Char. 66: 1 → 0

***Nyctiphruretus acudens*:**
Char. 21: 0 → 1
Char. 41: 0 → 1
Char. 66: 1 → 2
Char. 81: 1 → 0
Char. 83: 0 → 1
Char. 84: 1 → 2
Char. 94: 0 → 1
Char. 166: 1 → 0
Char. 224: 0 → 1
Char. 266: 0 → 1
Char. 272: 2 → 1
Char. 276: 0 → 1

***Orovenator mayorum*:**
Char. 8: 0 → 1
Char. 24: 0 → 1
Char. 33: 1 → 0
Char. 36: 0 → 1
Char. 50: 0 → 1
Char. 92: 1 → 0
Char. 94: 1 → 0
Char. 135: 1 → 0
Char. 159: 1 → 0
Char. 160: 0 → 1
Char. 165: 0 → 1
Char. 278: 03 → 1

***Owenetta* spp.:**
Char. 142: 1 → 0
Char. 169: 1 → 0

***Paleothyris acadiana*:**
Char. 66: 1 → 2
Char. 102: 0 → 1
Char. 146: 1 → 0

***Placodus* spp.:**
Char. 0: 1 → 2
Char. 9: 0 → 1
Char. 12: 0 → 1
Char. 13: 0 → 1
Char. 19: 0 → 1
Char. 31: 0 → 1
Char. 46: 1 → 0
Char. 57: 0 → 1
Char. 78: 1 → 0
Char. 93: 1 → 0
Char. 102: 1 → 2
Char. 109: 1 → 0
Char. 140: 1 → 0
Char. 155: 0 → 1
Char. 163: 1 → 0
Char. 164: 0 → 1

***Procolophon* spp.:**
Char. 41: 0 → 1
Char. 69: 0 → 1
Char. 79: 0 → 1
Char. 83: 0 → 1
Char. 86: 0 → 1
Char. 101: 0 → 1
Char. 141: 0 → 1
Char. 149: 1 → 0
Char. 203: 1 → 2
Char. 215: 0 → 1
Char. 230: 0 → 1
Char. 235: 1 → 2
Char. 237: 0 → 1
Char. 238: 0 → 1
Char. 272: 2 → 1
Char. 278: 3 → 0

***Prolacerta broomi*:**
Char. 58: 1 → 0
Char. 66: 1 → 0
Char. 67: 1 → 0
Char. 80: 1 → 0
Char. 139: 1 → 0
Char. 147: 1 → 0
Char. 192: 1 → 0
Char. 203: 1 → 2
Char. 206: 0 → 12

***Rhipaeosaurus* spp.:**
Char. 172: 0 → 1
Char. 183: 1 → 2
Char. 194: 0 → 1
Char. 201: 1 → 0
Char. 211: 0 → 1
Char. 215: 0 → 1
Char. 226: 0 → 1
Char. 235: 1 → 0
Char. 252: 0 → 1
Char. 277: 0 → 1

**Rhynchocephalia:**
Char. 0: 1 → 2
Char. 24: 0 → 1
Char. 77: 0 → 1
Char. 88: 1 → 0
Char. 94: 1 → 0
Char. 139: 1 → 0
Char. 167: 1 → 0
Char. 205: 1 → 0

**Rhynchosauria:**
Char. 0: 1 → 0
Char. 7: 0 → 1
Char. 9: 0 → 1
Char. 26: 0 → 1
Char. 44: 1 → 0
Char. 68: 0 → 1
Char. 99: 1 → 0
Char. 150: 1 → 0
Char. 160: 0 → 1
Char. 161: 0 → 1
Char. 171: 0 → 2
Char. 223: 0 → 1
Char. 224: 0 → 1
Char. 241: 0 → 1

***Scutosaurus* spp.:**
Char. 175: 0 → 1
Char. 190: 0 → 1
Char. 218: 0 → 1
Char. 243: 0 → 2
Char. 244: 0 → 1
Char. 251: 0 → 1

***Sinosaurosphargis yunguiensis*:**
Char. 8: 0 → 1
Char. 30: 0 → 1
Char. 53: 0 → 1
Char. 89: 1 → 0
Char. 127: 1 → 0
Char. 150: 1 → 0
Char. 154: 0 → 2
Char. 167: 1 → 0
Char. 253: 0 → 1
Char. 255: 0 → 1

**Squamata:**
Char. 45: 0 → 1
Char. 79: 0 → 2
Char. 80: 1 → 0
Char. 92: 1 → 0
Char. 109: 1 → 0
Char. 160: 0 → 1
Char. 200: 0 → 1
Char. 245: 0 → 1

***Trilophosaurus buettneri*:**
Char. 5: 1 → 0
Char. 11: 0 → 1
Char. 55: 1 → 0
Char. 93: 1 → 0
Char. 104: 0 → 1
Char. 106: 0 → 1
Char. 113: 0 → 1
Char. 122: 0 → 1
Char. 136: 1 → 0
Char. 144: 1 → 0
Char. 157: 0 → 1
Char. 159: 1 → 0
Char. 177: 0 → 12
Char. 194: 0 → 1
Char. 203: 1 → 2
Char. 207: 1 → 0
Char. 208: 1 → 0
Char. 272: 1 → 0

***Youngina capensis*:**
Char. 5: 1 → 0
Char. 21: 0 → 1
Char. 25: 0 → 1
Char. 27: 0 → 1
Char. 38: 1 → 0
Char. 43: 0 → 1
Char. 44: 1 → 0
Char. 48: 0 → 1
Char. 56: 0 → 1
Char. 75: 0 → 1
Char. 84: 1 → 0
Char. 92: 1 → 0
Char. 94: 1 → 0
Char. 134: 0 → 1
Char. 154: 1 → 0
Char. 163: 1 → 0
Char. 170: 0 → 1

Char. 211: 0 → 1
Char. 215: 0 → 1
Char. 224: 0 → 1
Char. 239: 0 → 1
Char. 267: 0 → 1

**Node 50:**
Char. 46: 1 → 0
Char. 88: 1 → 0
Char. 89: 1 → 0
Char. 93: 1 → 0
Char. 176: 0 → 1
Char. 195: 0 → 2
Char. 198: 0 → 1
Char. 246: 1 → 2
Char. 259: 0 → 1
Char. 265: 1 → 0
Char. 270: 0 → 1

**Node 51:**
Char. 65: 0 → 1
Char. 131: 0 → 1
Char. 184: 0 → 1
Char. 205: 0 → 1
Char. 210: 0 → 1
Char. 219: 1 → 0
Char. 241: 0 → 1
Char. 246: 0 → 1
Char. 254: 0 → 1
Char. 255: 0 → 1
Char. 256: 0 → 1
Char. 267: 0 → 1
Char. 268: 0 → 1
Char. 269: 0 → 1

**Node 52:**
Char. 0: 1 → 2
Char. 15: 0 → 1
Char. 21: 0 → 1
Char. 33: 1 → 2
Char. 44: 1 → 0
Char. 62: 1 → 0
Char. 64: 0 → 1
Char. 84: 1 → 2
Char. 130: 0 → 1
Char. 134: 0 → 2
Char. 147: 0 → 1
Char. 152: 0 → 1
Char. 155: 0 → 1
Char. 158: 0 → 1
Char. 161: 0 → 1
Char. 174: 0 → 1
Char. 181: 0 → 1
Char. 203: 1 → 2
Char. 247: 0 → 1
Char. 251: 0 → 1
Char. 252: 0 → 1
Char. 253: 0 → 2

**Node 53:**
Char. 0: 0 → 1
Char. 20: 0 → 1
Char. 25: 1 → 0
Char. 29: 1 → 0
Char. 33: 0 → 1
Char. 40: 2 → 0
Char. 50: 1 → 0
Char. 57: 1 → 0
Char. 59: 0 → 1
Char. 62: 0 → 1
Char. 67: 0 → 1
Char. 72: 1 → 2
Char. 89: 0 → 1
Char. 94: 0 → 1
Char. 109: 0 → 1
Char. 111: 0 → 1
Char. 112: 0 → 1
Char. 120: 0 → 1
Char. 127: 0 → 1
Char. 129: 0 → 1
Char. 135: 0 → 1
Char. 136: 0 → 1
Char. 139: 0 → 1
Char. 141: 0 → 1
Char. 179: 0 → 1
Char. 187: 0 → 1
Char. 188: 0 → 1
Char. 191: 0 → 1
Char. 193: 0 → 1
Char. 196: 0 → 1
Char. 201: 1 → 0
Char. 202: 0 → 1
Char. 209: 0 → 1
Char. 219: 0 → 1
Char. 222: 0 → 1
Char. 234: 0 → 1
Char. 237: 0 → 1
Char. 265: 0 → 1
Char. 266: 0 → 1
Char. 275: 1 → 0

**Node 54:**
Char. 5: 0 → 1
Char. 29: 0 → 1
Char. 40: 0 → 2
Char. 74: 0 → 1
Char. 80: 0 → 1
Char. 84: 0 → 1
Char. 88: 0 → 1
Char. 116: 0 → 1
Char. 119: 0 → 1
Char. 132: 0 → 1
Char. 149: 0 → 1
Char. 156: 0 → 1
Char. 166: 0 → 1
Char. 169: 0 → 1
Char. 180: 0 → 1
Char. 192: 0 → 1
Char. 201: 0 → 1
Char. 203: 0 → 1
Char. 221: 0 → 1
Char. 235: 0 → 1

**Node 55:**
Char. 72: 0 → 1
Char. 79: 1 → 0
Char. 81: 0 → 1
Char. 93: 0 → 1
Char. 97: 0 → 1
Char. 104: 1 → 0
Char. 144: 0 → 1
Char. 173: 0 → 1
Char. 183: 0 → 1

**Node 57:**
Char. 95: 0 → 1
Char. 113: 0 → 1
Char. 114: 0 → 1

**Node 58:**
Char. 20: 0 → 1
Char. 47: 0 → 1
Char. 48: 0 → 1
Char. 79: 0 → 1
Char. 110: 0 → 1
Char. 131: 0 → 1
Char. 137: 0 → 1
Char. 140: 0 → 2
Char. 147: 0 → 1

**Node 59:**
Char. 5: 1 → 0
Char. 29: 1 → 0
Char. 59: 0 → 1
Char. 60: 0 → 1
Char. 67: 0 → 1
Char. 72: 1 → 2
Char. 84: 1 → 0
Char. 111: 0 → 1
Char. 116: 1 → 0
Char. 154: 1 → 0
Char. 159: 1 → 0
Char. 166: 1 → 0
Char. 169: 1 → 0
Char. 170: 0 → 1
Char. 197: 0 → 1
Char. 221: 1 → 0

**Node 60:**
Char. 19: 0 → 1
Char. 92: 1 → 0

**Node 61:**
Char. 4: 0 → 1
Char. 15: 0 → 1
Char. 29: 0 → 1
Char. 213: 0 → 1
Char. 226: 0 → 2
Char. 228: 0 → 1
Char. 275: 0 → 1

**Node 62:**
Char. 43: 0 → 1
Char. 58: 0 → 1
Char. 61: 0 → 1
Char. 66: 0 → 1
Char. 69: 0 → 1
Char. 107: 0 → 1
Char. 126: 0 → 1
Char. 131: 0 → 1
Char. 134: 0 → 1
Char. 140: 0 → 1
Char. 147: 0 → 1
Char. 150: 0 → 1
Char. 167: 0 → 1
Char. 176: 0 → 1
Char. 190: 0 → 1
Char. 205: 0 → 1
Char. 208: 0 → 1
Char. 230: 0 → 1
Char. 239: 0 → 1
Char. 260: 2 → 1

**Node 63:**
Char. 73: 0 → 1
Char. 205: 0 → 1
Char. 273: 0 → 1
Char. 276: 0 → 1

**Node 64:**
Char. 18: 0 → 1
Char. 25: 1 → 0
Char. 37: 0 → 1
Char. 103: 0 → 1
Char. 106: 0 → 1
Char. 107: 0 → 1
Char. 126: 0 → 1
Char. 150: 0 → 1

**Node 65:**
Char. 20: 0 → 1
Char. 33: 0 → 1
Char. 38: 1 → 2
Char. 39: 0 → 1
Char. 44: 1 → 0
Char. 49: 0 → 1
Char. 66: 0 → 1
Char. 76: 0 → 1
Char. 80: 1 → 0
Char. 95: 0 → 1
Char. 112: 0 → 1
Char. 135: 0 → 1
Char. 137: 0 → 1
Char. 147: 0 → 1
Char. 157: 0 → 1
Char. 158: 0 → 1
Char. 183: 1 → 2
Char. 186: 0 → 1
Char. 187: 0 → 1
Char. 194: 0 → 1
Char. 197: 0 → 1
Char. 201: 1 → 0
Char. 211: 0 → 1
Char. 252: 0 → 1

**Node 67:**
Char. 133: 0 → 1

**Node 68:**
Char. 100: 0 → 1
Char. 113: 0 → 1

**Node 69:**
Char. 70: 0 → 1
Char. 118: 0 → 1
Char. 125: 0 → 1

**Node 71:**
Char. 38: 1 → 2
Char. 39: 0 → 1
Char. 50: 1 → 0
Char. 58: 0 → 1
Char. 59: 0 → 1
Char. 60: 0 → 1
Char. 72: 1 → 2
Char. 83: 0 → 1
Char. 88: 1 → 0
Char. 95: 0 → 1
Char. 104: 0 → 1
Char. 105: 0 → 1
Char. 106: 0 → 2
Char. 107: 0 → 1
Char. 109: 0 → 1
Char. 110: 0 → 1
Char. 146: 1 → 0
Char. 155: 0 → 2
Char. 183: 1 → 2

**Node 72:**
Char. 23: 0 → 1
Char. 33: 1 → 0
Char. 38: 2 → 1
Char. 39: 1 → 0
Char. 42: 0 → 1
Char. 43: 0 → 1
Char. 46: 1 → 0
Char. 49: 1 → 0
Char. 71: 1 → 0
Char. 83: 0 → 2
Char. 101: 0 → 1
Char. 103: 0 → 1
Char. 106: 0 → 1
Char. 161: 0 → 1
Char. 163: 1 → 0
Char. 172: 0 → 2
Char. 174: 0 → 1
Char. 188: 0 → 1
Char. 203: 1 → 2
Char. 212: 0 → 1
Char. 216: 1 → 0
Char. 221: 1 → 0
Char. 236: 0 → 1
Char. 238: 0 → 2
Char. 241: 0 → 1
Char. 242: 0 → 1
Char. 273: 0 → 1
Char. 274: 0 → 1
Char. 275: 1 → 0

**Node 73:**
Char. 52: 0 → 1
Char. 75: 0 → 1
Char. 84: 1 → 0
Char. 121: 0 → 1
Char. 180: 1 → 0
Char. 230: 0 → 1

**Node 74:**
Char. 44: 1 → 0
Char. 66: 0 → 1
Char. 78: 1 → 0

Char. 88: 1 → 0
Char. 93: 1 → 0
Char. 123: 1 → 0
Char. 129: 0 → 1
Char. 192: 1 → 0

**Node 75:**
Char. 83: 0 → 1
Char. 107: 0 → 1

**Node 76:**
Char. 2: 0 → 1
Char. 6: 0 → 1
Char. 186: 0 → 1
Char. 198: 0 → 1
Char. 200: 0 → 1
Char. 220: 0 → 1
Char. 239: 1 → 0

**Node 77:**
Char. 48: 0 → 1
Char. 98: 1 → 0
Char. 113: 0 → 1
Char. 181: 0 → 1
Char. 206: 0 → 2

**Node 78:**
Char. 35: 0 → 1
Char. 102: 0 → 1
Char. 103: 0 → 1
Char. 184: 0 → 1
Char. 201: 0 → 1
Char. 210: 0 → 1
Char. 223: 0 → 1
Char. 229: 0 → 1

**Node 79:**
Char. 17: 0 → 1
Char. 42: 0 → 1
Char. 251: 0 → 1
Char. 264: 0 → 1

**Node 80:**
Char. 41: 0 → 1
Char. 61: 1 → 2
Char. 112: 1 → 0
Char. 138: 0 → 1
Char. 146: 1 → 0
Char. 155: 0 → 1
Char. 192: 1 → 2
Char. 224: 0 → 1
Char. 226: 0 → 1
Char. 227: 0 → 1
Char. 233: 0 → 1
Char. 267: 01 → 2

**Node 81:**
Char. 61: 1 → 3
Char. 90: 0 → 1
Char. 91: 0 → 1
Char. 109: 1 → 0
Char. 155: 0 → 1
Char. 209: 1 → 0

**ANALYSIS 35**
**(ALL TAXA, IMPLIED WEIGHTING, K = 2.375)**

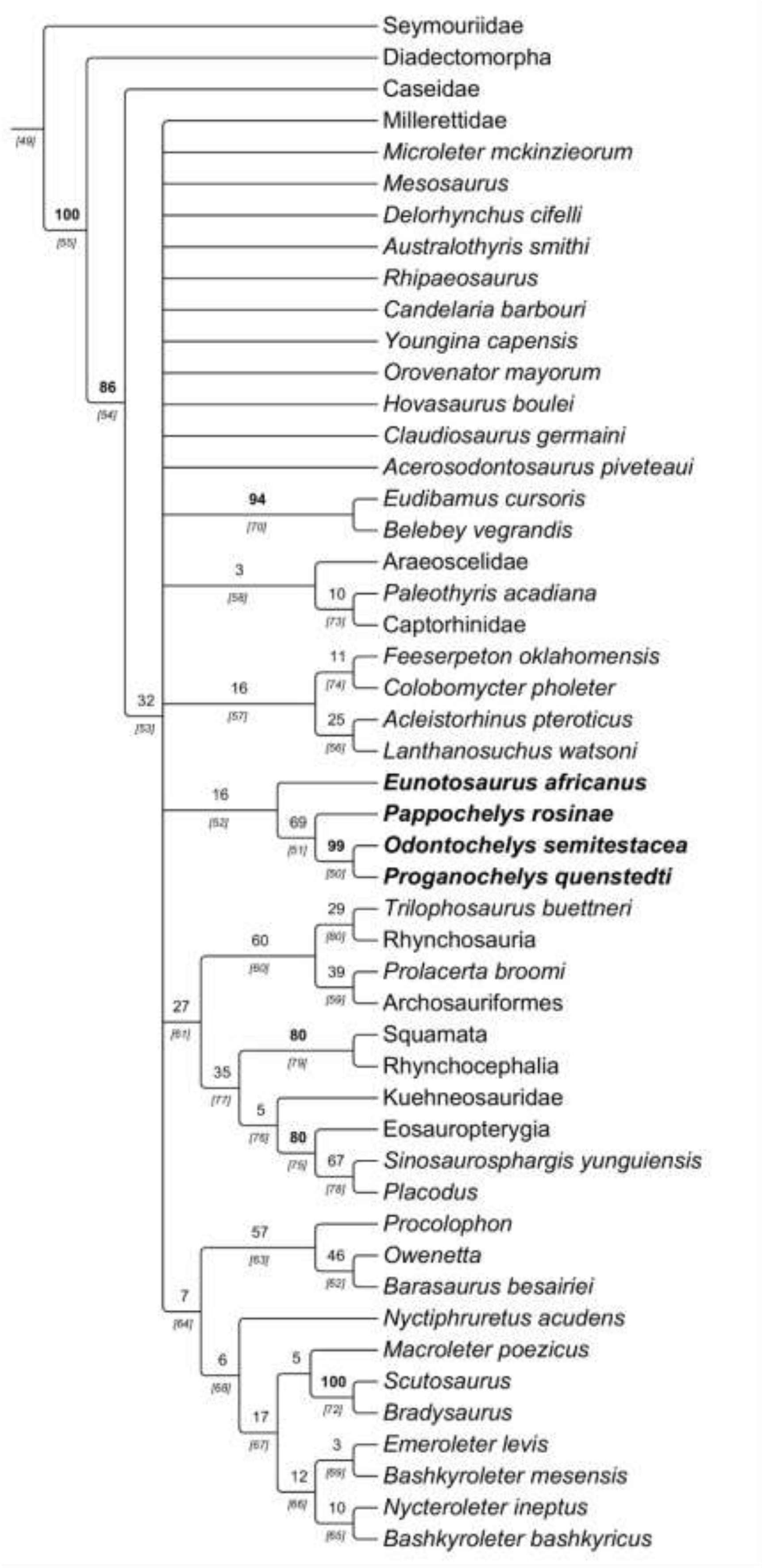

***Proganochelys quenstedti*:**
Char. 11: 0 → 1
Char. 46: 1 → 0
Char. 53: 0 → 1
Char. 55: 0 → 1
Char. 77: 0 → 1
Char. 79: 0 → 2
Char. 88: 1 → 0
Char. 89: 1 → 0
Char. 93: 1 → 0
Char. 128: 0 → 1
Char. 175: 0 → 1
Char. 176: 0 → 1
Char. 195: 0 → 2
Char. 198: 0 → 1
Char. 246: 1 → 2
Char. 259: 0 → 1
Char. 262: 0 → 1
Char. 270: 0 → 1

**Seymouriidae:**
Char. 51: 0 → 2
Char. 54: 1 → 0
Char. 99: 1 → 0
Char. 107: 0 → 1
Char. 126: 0 → 1
Char. 154: 1 → 2
Char. 225: 1 → 0
Char. 260: 2 → 0

***Pappochelys rosinae*:**
Char. 1: 0 → 1
Char. 5: 1 → 0
Char. 23: 0 → 1
Char. 41: 0 → 1
Char. 48: 0 → 1
Char. 49: 0 → 1
Char. 75: 0 → 1
Char. 129: 0 → 1
Char. 206: 0 → 2
Char. 260: 2 → 0
Char. 265: 0 → 1

***Eunotosaurus africanus*:**
Char. 19: 0 → 1
Char. 43: 0 → 1
Char. 97: 1 → 0
Char. 103: 0 → 1
Char. 112: 1 → 0
Char. 124: 0 → 1
Char. 153: 1 → 0
Char. 192: 1 → 0
Char. 211: 0 → 1
Char. 219: 0 → 1
Char. 237: 1 → 0
Char. 249: 0 → 2
Char. 263: 0 → 1
Char. 273: 0 → 1
Char. 274: 0 → 1

***Acerosodontosaurus piveteaui*:**
Char. 23: 0 → 1
Char. 59: 0 → 1
Char. 78: 1 → 0
Char. 81: 1 → 0
Char. 89: 0 → 1
Char. 94: 0 → 1
Char. 128: 0 → 1
Char. 129: 0 → 1
Char. 155: 0 → 1
Char. 206: 0 → 2
Char. 208: 0 → 1
Char. 265: 0 → 1
Char. 266: 0 → 1
Char. 278: 3 → 1

***Acleistorhinus pteroticus*:**
Char. 30: 0 → 1
Char. 55: 1 → 0
Char. 56: 0 → 1
Char. 64: 0 → 1
Char. 95: 0 → 1
Char. 113: 0 → 1
Char. 114: 0 → 1
Char. 146: 1 → 0
Char. 278: 3 → 1

**Araeoscelidae:**
Char. 5: 1 → 0
Char. 20: 1 → 0
Char. 23: 0 → 1
Char. 27: 0 → 1
Char. 28: 0 → 1
Char. 38: 1 → 0
Char. 40: 2 → 0
Char. 43: 0 → 1
Char. 59: 0 → 1
Char. 60: 0 → 1
Char. 72: 1 → 2
Char. 84: 1 → 0
Char. 89: 0 → 1
Char. 92: 1 → 0
Char. 106: 0 → 1
Char. 116: 1 → 0
Char. 117: 01 → 2
Char. 154: 1 → 0
Char. 169: 1 → 0
Char. 170: 0 → 1
Char. 193: 0 → 1
Char. 197: 0 → 1
Char. 202: 1 → 0
Char. 207: 1 → 0
Char. 209: 1 → 0
Char. 221: 1 → 0
Char. 224: 0 → 1
Char. 239: 0 → 1
Char. 266: 0 → 1
Char. 278: 3 → 1

**Archosauriformes:**
Char. 32: 0 → 1
Char. 94: 1 → 0
Char. 112: 1 → 0
Char. 152: 0 → 1
Char. 154: 0 → 2
Char. 166: 1 → 0
Char. 171: 0 → 1
Char. 185: 0 → 1
Char. 204: 0 → 1
Char. 218: 0 → 3
Char. 224: 0 → 1
Char. 242: 0 → 1

***Australothyris smithi*:**
Char. 23: 0 → 1
Char. 34: 0 → 1
Char. 55: 1 → 0
Char. 71: 1 → 0
Char. 85: 1 → 0
Char. 129: 0 → 1
Char. 131: 0 → 1

***Barasaurus besairiei*:**
Char. 33: 1 → 0
Char. 75: 1 → 0
Char. 216: 0 → 1

***Bashkyroleter bashkyricus*:**
Char. 275: 1 → 0

***Bashkyroleter mesensis*:**
Char. 169: 1 → 0

***Belebey vegrandis*:**
Char. 154: 1 → 0

***Bradysaurus* spp.:**
Char. 19: 0 → 1
Char. 73: 0 → 1
Char. 79: 0 → 1
Char. 135: 1 → 0
Char. 249: 0 → 1

***Candelaria barbouri*:**
Char. 1: 0 → 1
Char. 5: 1 → 0
Char. 23: 0 → 1
Char. 49: 0 → 1
Char. 79: 0 → 2
Char. 83: 0 → 1
Char. 88: 1 → 0
Char. 92: 1 → 0
Char. 95: 0 → 1
Char. 132: 1 → 0
Char. 154: 1 → 2

**Captorhinidae:**
Char. 3: 0 → 1
Char. 23: 0 → 1
Char. 25: 1 → 0
Char. 26: 0 → 1
Char. 73: 0 → 1
Char. 75: 0 → 1
Char. 83: 0 → 1
Char. 108: 1 → 0
Char. 180: 1 → 0
Char. 183: 1 → 0
Char. 201: 1 → 0
Char. 203: 1 → 2
Char. 216: 1 → 0
Char. 237: 1 → 0
Char. 240: 1 → 0

**Caseidae:**
Char. 24: 0 → 1
Char. 25: 1 → 0
Char. 36: 0 → 1
Char. 38: 1 → 0
Char. 46: 1 → 0
Char. 56: 0 → 1
Char. 170: 0 → 1
Char. 194: 0 → 1
Char. 273: 0 → 1
Char. 274: 0 → 1
Char. 278: 3 → 2

***Claudiosaurus germaini*:**
Char. 23: 0 → 1
Char. 24: 0 → 1
Char. 27: 0 → 1
Char. 34: 0 → 1
Char. 36: 0 → 1
Char. 56: 0 → 1
Char. 59: 0 → 1
Char. 60: 0 → 1
Char. 62: 0 → 1
Char. 64: 0 → 1
Char. 70: 0 → 1
Char. 72: 1 → 2
Char. 73: 0 → 1
Char. 84: 1 → 0
Char. 89: 0 → 1
Char. 94: 0 → 1
Char. 105: 0 → 1
Char. 106: 0 → 1
Char. 109: 0 → 1
Char. 117: 01 → 2
Char. 126: 0 → 1
Char. 129: 0 → 1
Char. 130: 0 → 1
Char. 131: 0 → 1
Char. 144: 1 → 0
Char. 148: 0 → 1
Char. 154: 1 → 0
Char. 182: 0 → 1
Char. 188: 0 → 1
Char. 190: 0 → 1
Char. 191: 0 → 1
Char. 199: 0 → 1
Char. 203: 1 → 2
Char. 204: 0 → 1
Char. 214: 0 → 1
Char. 219: 0 → 1
Char. 220: 0 → 1
Char. 234: 1 → 0
Char. 265: 0 → 1
Char. 267: 0 → 1
Char. 272: 2 → 1
Char. 275: 1 → 0
Char. 278: 3 → 0

***Colobomycter pholeter*:**
Char. 25: 1 → 0
Char. 84: 1 → 0
Char. 154: 1 → 0
Char. 167: 0 → 1
Char. 267: 0 → 1

***Delorhynchus cifelli*:**
Char. 18: 0 → 1
Char. 21: 0 → 1
Char. 24: 0 → 1
Char. 26: 0 → 1
Char. 28: 0 → 1
Char. 29: 0 → 1
Char. 33: 01 → 2
Char. 39: 0 → 1
Char. 52: 0 → 1
Char. 57: 0 → 1
Char. 67: 1 → 0
Char. 100: 0 → 1
Char. 116: 1 → 0
Char. 119: 1 → 0
Char. 131: 0 → 1
Char. 147: 0 → 1
Char. 156: 1 → 0
Char. 167: 0 → 1
Char. 189: 0 → 1
Char. 191: 0 → 1
Char. 202: 1 → 0
Char. 204: 0 → 2
Char. 267: 0 → 1

**Diadectomorpha:**
Char. 64: 0 → 1
Char. 70: 0 → 1
Char. 75: 0 → 1
Char. 122: 0 → 1
Char. 123: 1 → 0
Char. 146: 1 → 0
Char. 275: 1 → 0
Char. 278: 3 → 0

***Emeroleter levis*:**
Char. 51: 1 → 0

**Eosauropterygia:**
Char. 159: 1 → 0
Char. 166: 1 → 0
Char. 174: 0 → 1
Char. 194: 0 → 2
Char. 272: 1 → 0

***Eudibamus cursoris*:**
Char. 154: 1 → 2

***Feeserpeton oklahomensis*:**
Char. 51: 0 → 1
Char. 157: 0 → 1
Char. 158: 0 → 1

***Hovasaurus boulei*:**
Char. 41: 0 → 1
Char. 43: 0 → 1
Char. 55: 1 → 0
Char. 59: 0 → 1
Char. 77: 0 → 2

Char. 78: 1 → 0
Char. 79: 0 → 1
Char. 89: 0 → 1
Char. 93: 1 → 0
Char. 113: 0 → 1
Char. 127: 0 → 1
Char. 135: 0 → 1
Char. 136: 0 → 1
Char. 138: 0 → 1
Char. 146: 1 → 0
Char. 188: 0 → 1
Char. 191: 0 → 1
Char. 193: 0 → 1
Char. 201: 1 → 0
Char. 204: 0 → 2
Char. 206: 0 → 2
Char. 215: 0 → 1
Char. 220: 0 → 1
Char. 224: 0 → 1
Char. 265: 0 → 1
Char. 275: 1 → 0

**Kuehneosauridae:**
Char. 7: 0 → 1
Char. 24: 0 → 1
Char. 26: 0 → 1
Char. 27: 0 → 1
Char. 34: 0 → 1
Char. 36: 0 → 1
Char. 44: 1 → 0
Char. 56: 0 → 1
Char. 79: 0 → 2
Char. 82: 0 → 1
Char. 98: 1 → 0
Char. 108: 1 → 0
Char. 113: 0 → 1
Char. 128: 0 → 1
Char. 181: 0 → 1
Char. 185: 0 → 1
Char. 206: 0 → 2
Char. 245: 0 → 1

***Lanthanosuchus watsoni*:**
Char. 25: 1 → 0
Char. 29: 0 → 1
Char. 51: 0 → 1
Char. 76: 0 → 1
Char. 86: 0 → 1
Char. 95: 0 → 1
Char. 98: 1 → 0
Char. 110: 0 → 1
Char. 111: 1 → 0
Char. 113: 0 → 1
Char. 114: 0 → 1
Char. 119: 1 → 0
Char. 131: 0 → 1
Char. 137: 0 → 1
Char. 138: 0 → 1
Char. 140: 0 → 2
Char. 144: 1 → 0
Char. 147: 0 → 1
Char. 154: 1 → 2

***Macroleter poezicus*:**
Char. 52: 0 → 1
Char. 66: 1 → 2
Char. 84: 1 → 0
Char. 87: 0 → 1
Char. 140: 0 → 1
Char. 146: 1 → 0
Char. 169: 1 → 0
Char. 235: 0 → 1

***Mesosaurus* spp.:**
Char. 2: 0 → 1
Char. 5: 1 → 0
Char. 6: 0 → 1
Char. 8: 0 → 1
Char. 9: 0 → 1
Char. 13: 0 → 1
Char. 19: 0 → 1
Char. 41: 0 → 1
Char. 84: 1 → 0
Char. 94: 0 → 1
Char. 112: 1 → 0
Char. 115: 0 → 1
Char. 149: 1 → 0
Char. 164: 0 → 1
Char. 167: 0 → 1
Char. 192: 1 → 0
Char. 231: 0 → 1
Char. 234: 1 → 0
Char. 240: 1 → 0
Char. 272: 2 → 0

***Microleter mckinzieorum*:**
Char. 25: 1 → 0
Char. 36: 0 → 1
Char. 39: 0 → 1
Char. 51: 0 → 2
Char. 56: 0 → 1
Char. 70: 0 → 1
Char. 94: 0 → 1
Char. 106: 0 → 1
Char. 112: 1 → 0
Char. 276: 0 → 1

**Millerettidae:**
Char. 5: 1 → 0
Char. 20: 1 → 0
Char. 24: 0 → 1
Char. 25: 1 → 0
Char. 29: 0 → 1
Char. 44: 1 → 0
Char. 56: 0 → 1
Char. 66: 0 → 2
Char. 67: 1 → 0
Char. 78: 1 → 0
Char. 80: 1 → 0
Char. 84: 1 → 2
Char. 88: 1 → 0
Char. 96: 1 → 0
Char. 111: 1 → 0
Char. 112: 1 → 0
Char. 120: 1 → 0
Char. 121: 0 → 1

Char. 124: 0 → 1
Char. 127: 0 → 1
Char. 135: 0 → 1
Char. 145: 0 → 1
Char. 163: 1 → 0
Char. 180: 1 → 0
Char. 192: 1 → 0
Char. 211: 0 → 1
Char. 230: 0 → 1
Char. 248: 0 → 1
Char. 252: 0 → 1
Char. 253: 0 → 1

***Nycteroleter ineptus*:**
Char. 278: 0 → 3

***Nyctiphruretus acudens*:**
Char. 21: 0 → 1
Char. 33: 1 → 2
Char. 41: 0 → 1
Char. 66: 1 → 2
Char. 81: 1 → 0
Char. 83: 0 → 1
Char. 84: 1 → 2
Char. 94: 0 → 1
Char. 167: 0 → 1
Char. 224: 0 → 1
Char. 266: 0 → 1
Char. 272: 2 → 1
Char. 276: 0 → 1

***Orovenator mayorum*:**
Char. 8: 0 → 1
Char. 24: 0 → 1
Char. 25: 1 → 0
Char. 36: 0 → 1
Char. 62: 0 → 1
Char. 72: 1 → 2
Char. 89: 0 → 1
Char. 92: 1 → 0
Char. 136: 0 → 1
Char. 141: 0 → 1
Char. 160: 0 → 1
Char. 165: 0 → 1
Char. 278: 3 → 1

***Owenetta* spp.:**
Char. 169: 1 → 0

***Paleothyris acadiana*:**
Char. 38: 1 → 0
Char. 66: 1 → 2
Char. 102: 0 → 1
Char. 146: 1 → 0
Char. 239: 0 → 1

***Placodus* spp.:**
Char. 0: 1 → 2
Char. 9: 0 → 1
Char. 12: 0 → 1
Char. 13: 0 → 1
Char. 19: 0 → 1
Char. 26: 0 → 1
Char. 31: 0 → 1

Char. 44: 1 → 0
Char. 46: 1 → 0
Char. 57: 0 → 1
Char. 78: 1 → 0
Char. 93: 1 → 0
Char. 102: 1 → 2
Char. 140: 1 → 0
Char. 155: 0 → 1
Char. 163: 1 → 0
Char. 164: 0 → 1

***Procolophon* spp.:**
Char. 41: 0 → 1
Char. 69: 0 → 1
Char. 79: 0 → 1
Char. 83: 0 → 1
Char. 88: 0 → 1
Char. 117: 1 → 0
Char. 149: 1 → 0
Char. 204: 0 → 1
Char. 237: 0 → 1
Char. 238: 0 → 1
Char. 272: 2 → 1
Char. 278: 3 → 0

***Prolacerta broomi*:**
Char. 58: 1 → 0
Char. 66: 1 → 0
Char. 67: 1 → 0
Char. 80: 1 → 0
Char. 139: 1 → 0
Char. 147: 1 → 0
Char. 192: 1 → 0
Char. 203: 1 → 2
Char. 206: 0 → 12

***Rhipaeosaurus* spp.:**
Char. 172: 0 → 1
Char. 277: 0 → 1

**Rhynchocephalia:**
Char. 0: 1 → 2
Char. 24: 0 → 1
Char. 75: 1 → 0
Char. 77: 0 → 1
Char. 94: 1 → 0
Char. 139: 1 → 0
Char. 167: 1 → 0
Char. 205: 1 → 0

**Rhynchosauria:**
Char. 0: 1 → 0
Char. 7: 0 → 1
Char. 9: 0 → 1
Char. 26: 0 → 1
Char. 44: 1 → 0
Char. 68: 0 → 1
Char. 99: 1 → 0
Char. 150: 1 → 0
Char. 160: 0 → 1
Char. 161: 0 → 1
Char. 171: 0 → 2
Char. 223: 0 → 1

Char. 224: 0 → 1
Char. 241: 0 → 1

***Scutosaurus* spp.:**
Char. 175: 0 → 1
Char. 218: 0 → 1
Char. 243: 0 → 2
Char. 244: 0 → 1
Char. 251: 0 → 1

***Sinosaurosphargis yunguiensis*:**
Char. 8: 0 → 1
Char. 30: 0 → 1
Char. 53: 0 → 1
Char. 82: 0 → 1
Char. 89: 1 → 0
Char. 127: 1 → 0
Char. 150: 1 → 0
Char. 154: 0 → 2
Char. 167: 1 → 0
Char. 253: 0 → 1
Char. 255: 0 → 1

**Squamata:**
Char. 26: 0 → 1
Char. 45: 0 → 1
Char. 79: 0 → 2
Char. 80: 1 → 0
Char. 82: 0 → 1
Char. 92: 1 → 0
Char. 160: 0 → 1
Char. 245: 0 → 1

***Trilophosaurus buettneri*:**
Char. 5: 1 → 0
Char. 11: 0 → 1
Char. 55: 1 → 0
Char. 93: 1 → 0
Char. 104: 0 → 1
Char. 106: 0 → 1
Char. 113: 0 → 1
Char. 122: 0 → 1
Char. 144: 1 → 0
Char. 154: 0 → 1
Char. 157: 0 → 1
Char. 159: 1 → 0
Char. 177: 0 → 12
Char. 194: 0 → 1
Char. 203: 1 → 2
Char. 207: 1 → 0
Char. 208: 1 → 0
Char. 272: 1 → 0

***Youngina capensis*:**
Char. 5: 1 → 0
Char. 21: 0 → 1
Char. 27: 0 → 1
Char. 38: 1 → 0
Char. 40: 2 → 0
Char. 43: 0 → 1
Char. 44: 1 → 0
Char. 56: 0 → 1
Char. 59: 0 → 1

Char. 60: 0 → 1
Char. 62: 0 → 1
Char. 72: 1 → 2
Char. 75: 0 → 1
Char. 84: 1 → 0
Char. 89: 0 → 1
Char. 92: 1 → 0
Char. 109: 0 → 1
Char. 127: 0 → 1
Char. 129: 0 → 1
Char. 134: 0 → 1
Char. 135: 0 → 1
Char. 141: 0 → 1
Char. 154: 1 → 0
Char. 163: 1 → 0
Char. 170: 0 → 1
Char. 191: 0 → 1
Char. 193: 0 → 1
Char. 201: 1 → 0
Char. 211: 0 → 1
Char. 214: 0 → 1
Char. 215: 0 → 1
Char. 219: 0 → 1
Char. 224: 0 → 1
Char. 231: 0 → 1
Char. 239: 0 → 1
Char. 265: 0 → 1
Char. 267: 0 → 1
Char. 275: 1 → 0
Char. 278: 3 → 0

**Node 50:**
Char. 65: 0 → 1
Char. 72: 1 → 2
Char. 131: 0 → 1
Char. 184: 0 → 1
Char. 205: 0 → 1
Char. 210: 0 → 1
Char. 241: 0 → 1
Char. 246: 0 → 1
Char. 254: 0 → 1
Char. 255: 0 → 1
Char. 256: 0 → 1
Char. 267: 0 → 1
Char. 268: 0 → 1
Char. 269: 0 → 1
Char. 278: 3 → 0

**Node 51:**
Char. 21: 0 → 1
Char. 84: 1 → 2

**Node 52:**
Char. 15: 0 → 1
Char. 25: 1 → 0
Char. 33: 01 → 2
Char. 55: 1 → 0
Char. 89: 0 → 1
Char. 94: 0 → 1
Char. 127: 0 → 1

**Node 53:**
Char. 5: 0 → 1
Char. 20: 0 → 1
Char. 40: 0 → 2
Char. 57: 1 → 0
Char. 67: 0 → 1
Char. 74: 0 → 1
Char. 80: 0 → 1
Char. 84: 0 → 1
Char. 88: 0 → 1
Char. 112: 0 → 1
Char. 116: 0 → 1
Char. 119: 0 → 1
Char. 149: 0 → 1
Char. 156: 0 → 1
Char. 163: 0 → 1
Char. 169: 0 → 1
Char. 180: 0 → 1
Char. 192: 0 → 1
Char. 201: 0 → 1
Char. 202: 0 → 1
Char. 203: 0 → 1
Char. 209: 0 → 1
Char. 221: 0 → 1
Char. 234: 0 → 1
Char. 235: 0 → 1
Char. 237: 0 → 1

**Node 54:**
Char. 72: 0 → 1
Char. 79: 1 → 0
Char. 81: 0 → 1
Char. 93: 0 → 1
Char. 97: 0 → 1
Char. 183: 0 → 1

**Node 57:**
Char. 29: 0 → 1
Char. 47: 0 → 1
Char. 57: 0 → 1
Char. 67: 1 → 0
Char. 79: 0 → 1
Char. 110: 0 → 1
Char. 111: 1 → 0
Char. 112: 1 → 0
Char. 131: 0 → 1
Char. 137: 0 → 1
Char. 140: 0 → 2
Char. 147: 0 → 1
Char. 169: 1 → 0
Char. 170: 0 → 1

**Node 58:**
Char. 19: 0 → 1
Char. 92: 1 → 0

**Node 59:**
Char. 4: 0 → 1
Char. 15: 0 → 1
Char. 29: 0 → 1
Char. 213: 0 → 1
Char. 226: 0 → 2
Char. 228: 0 → 1

**Node 60:**
Char. 43: 0 → 1
Char. 75: 0 → 1
Char. 107: 0 → 1
Char. 140: 0 → 1
Char. 147: 0 → 1
Char. 148: 0 → 1
Char. 272: 2 → 1
Char. 278: 3 → 0

**Node 61:**
Char. 58: 0 → 1
Char. 59: 0 → 1
Char. 60: 0 → 1
Char. 61: 0 → 1
Char. 62: 0 → 1
Char. 66: 0 → 1
Char. 69: 0 → 1
Char. 72: 1 → 2
Char. 73: 0 → 1
Char. 89: 0 → 1
Char. 94: 0 → 1
Char. 126: 0 → 1
Char. 127: 0 → 1
Char. 129: 0 → 1
Char. 131: 0 → 1
Char. 141: 0 → 1
Char. 145: 0 → 1
Char. 150: 0 → 1
Char. 154: 1 → 0
Char. 167: 0 → 1
Char. 176: 0 → 1
Char. 188: 0 → 1
Char. 205: 0 → 1
Char. 208: 0 → 1
Char. 214: 0 → 1
Char. 219: 0 → 1
Char. 239: 0 → 1
Char. 265: 0 → 1

**Node 62:**
Char. 24: 0 → 1
Char. 29: 0 → 1
Char. 67: 1 → 0
Char. 79: 0 → 1
Char. 83: 0 → 1
Char. 110: 0 → 1
Char. 111: 1 → 0
Char. 132: 1 → 0

**Node 63:**
Char. 73: 0 → 1
Char. 131: 1 → 0
Char. 205: 0 → 1
Char. 239: 0 → 1
Char. 276: 0 → 1

**Node 64:**
Char. 18: 0 → 1
Char. 37: 0 → 1
Char. 107: 0 → 1
Char. 118: 1 → 0
Char. 125: 1 → 0
Char. 150: 0 → 1

**Node 65:**
Char. 23: 0 → 1
Char. 71: 1 → 0
Char. 102: 0 → 1
Char. 103: 0 → 1
Char. 106: 0 → 1
Char. 167: 0 → 1
Char. 214: 0 → 1
Char. 216: 1 → 0
Char. 235: 0 → 2
Char. 241: 0 → 1

**Node 66:**
Char. 138: 0 → 1
Char. 141: 0 → 1

**Node 67:**
Char. 29: 0 → 1
Char. 38: 1 → 2
Char. 39: 0 → 1
Char. 44: 1 → 0
Char. 49: 0 → 1
Char. 51: 0 → 1
Char. 57: 0 → 1
Char. 66: 0 → 1
Char. 67: 1 → 0
Char. 70: 0 → 1
Char. 76: 0 → 1
Char. 80: 1 → 0
Char. 88: 1 → 0
Char. 95: 0 → 1
Char. 111: 1 → 0
Char. 118: 0 → 1
Char. 120: 1 → 0
Char. 125: 0 → 1
Char. 131: 0 → 1
Char. 137: 0 → 1
Char. 147: 0 → 1
Char. 148: 0 → 1
Char. 158: 0 → 1
Char. 183: 1 → 2
Char. 194: 0 → 1
Char. 201: 1 → 0
Char. 211: 0 → 1
Char. 234: 1 → 0
Char. 235: 1 → 0
Char. 237: 1 → 0
Char. 240: 1 → 0
Char. 252: 0 → 1

**Node 68:**
Char. 87: 0 → 1

**Node 69:**
Char. 25: 1 → 0
Char. 76: 1 → 0

**Node 70:**
Char. 79: 0 → 1
Char. 93: 1 → 0
Char. 133: 0 → 1

**Node 71:**
Char. 147: 1 → 2
Char. 154: 1 → 0
Char. 239: 0 → 1
Char. 278: 3 → 0

**Node 72:**
Char. 39: 0 → 1
Char. 58: 0 → 1
Char. 59: 0 → 1
Char. 60: 0 → 1
Char. 72: 1 → 2
Char. 88: 1 → 0
Char. 95: 0 → 1
Char. 104: 0 → 1
Char. 105: 0 → 1
Char. 106: 0 → 2
Char. 110: 0 → 1
Char. 155: 0 → 2

**Node 73:**
Char. 20: 1 → 0
Char. 23: 0 → 1
Char. 26: 0 → 1
Char. 76: 0 → 1
Char. 83: 0 → 1
Char. 85: 1 → 0
Char. 107: 0 → 1
Char. 109: 0 → 1
Char. 146: 1 → 0
Char. 148: 0 → 1
Char. 199: 0 → 1
Char. 237: 1 → 0

**Node 74:**
Char. 33: 1 → 0
Char. 38: 2 → 1
Char. 39: 1 → 0
Char. 42: 0 → 1
Char. 43: 0 → 1
Char. 46: 1 → 0
Char. 49: 1 → 0
Char. 52: 0 → 1
Char. 83: 0 → 2
Char. 84: 1 → 0
Char. 87: 0 → 1
Char. 93: 1 → 0
Char. 161: 0 → 1
Char. 163: 1 → 0
Char. 172: 0 → 2
Char. 174: 0 → 1
Char. 188: 0 → 1
Char. 189: 0 → 1
Char. 195: 0 → 1
Char. 204: 0 → 2
Char. 212: 0 → 1
Char. 221: 1 → 0
Char. 236: 0 → 1
Char. 238: 0 → 2
Char. 242: 0 → 1
Char. 245: 0 → 1
Char. 274: 0 → 1
Char. 275: 1 → 0

**Node 75:**
Char. 5: 1 → 0
Char. 20: 1 → 0

Char. 44: 1 → 0
Char. 59: 0 → 1
Char. 60: 0 → 1
Char. 66: 0 → 1
Char. 72: 1 → 2
Char. 78: 1 → 0
Char. 84: 1 → 0
Char. 88: 1 → 0
Char. 93: 1 → 0
Char. 112: 1 → 0
Char. 123: 1 → 0
Char. 129: 0 → 1
Char. 154: 1 → 0
Char. 163: 1 → 0
Char. 169: 1 → 0
Char. 170: 0 → 1
Char. 192: 1 → 0
Char. 197: 0 → 1
Char. 202: 1 → 0
Char. 207: 1 → 0
Char. 209: 1 → 0
Char. 221: 1 → 0
Char. 234: 1 → 0

**Node 76:**
Char. 83: 0 → 1
Char. 85: 1 → 0
Char. 107: 0 → 1

**Node 77:**
Char. 2: 0 → 1
Char. 6: 0 → 1
Char. 98: 1 → 0
Char. 101: 0 → 1
Char. 113: 0 → 1
Char. 181: 0 → 1
Char. 186: 0 → 1
Char. 198: 0 → 1
Char. 206: 0 → 2
Char. 220: 0 → 1
Char. 234: 1 → 0
Char. 239: 1 → 0
Char. 240: 1 → 0

**Node 78:**
Char. 33: 1 → 2
Char. 68: 0 → 1
Char. 102: 0 → 1
Char. 103: 0 → 1
Char. 223: 0 → 1
Char. 229: 0 → 1
Char. 235: 1 → 2

**Node 79:**
Char. 17: 0 → 1
Char. 42: 0 → 1
Char. 251: 0 → 1
Char. 264: 0 → 1

**Node 80:**
Char. 41: 0 → 1
Char. 61: 1 → 2
Char. 112: 1 → 0
Char. 128: 0 → 1
Char. 138: 0 → 1
Char. 146: 1 → 0
Char. 155: 0 → 1
Char. 192: 1 → 2
Char. 224: 0 → 1
Char. 226: 0 → 1
Char. 227: 0 → 1
Char. 233: 0 → 1

**Node 81:**
Char. 61: 1 → 3
Char. 90: 0 → 1
Char. 91: 0 → 1
Char. 155: 0 → 1
Char. 209: 1 → 0

ANALYSIS 36

(ALL TAXA, IMPLIED WEIGHTING, K = 2.5)

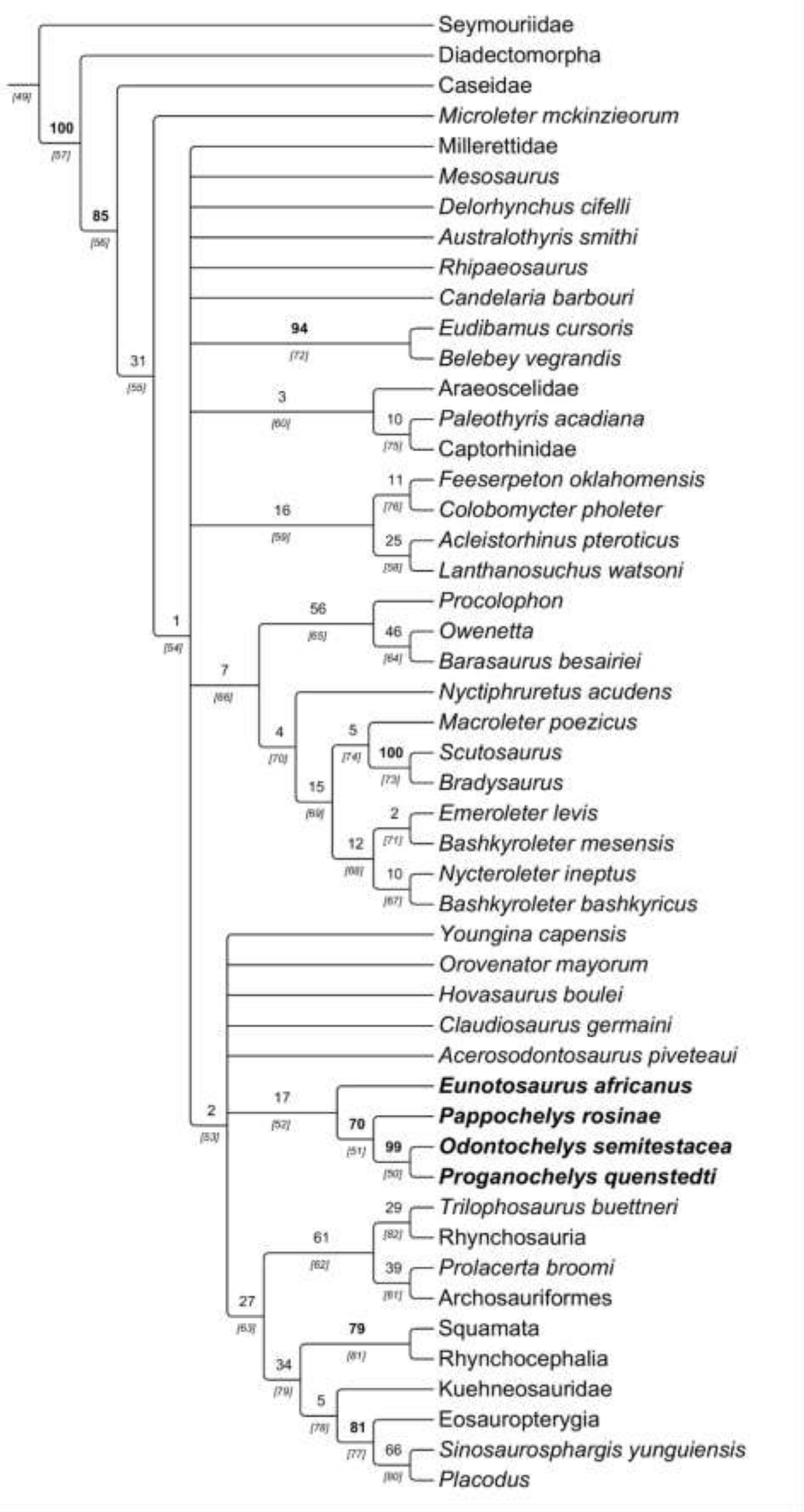

***Proganochelys quenstedti*:**
Char. 8: 0 → 1
Char. 11: 0 → 1
Char. 106: 0 → 1
Char. 108: 1 → 0
Char. 128: 0 → 1
Char. 175: 0 → 1
Char. 202: 1 → 0
Char. 207: 1 → 0
Char. 209: 1 → 0
Char. 244: 0 → 1
Char. 248: 0 → 1
Char. 250: 0 → 1
Char. 252: 1 → 0
Char. 262: 0 → 1

***Pappochelys rosinae*:**
Char. 0: 2 → 0
Char. 1: 0 → 1
Char. 5: 1 → 0
Char. 12: 0 → 1
Char. 41: 0 → 1
Char. 49: 0 → 1
Char. 75: 0 → 1
Char. 169: 1 → 0
Char. 260: 2 → 0

***Eunotosaurus africanus*:**
Char. 19: 0 → 1
Char. 59: 1 → 0
Char. 60: 1 → 0
Char. 72: 2 → 1
Char. 76: 0 → 1
Char. 97: 1 → 0
Char. 103: 0 → 1
Char. 153: 1 → 0
Char. 191: 1 → 0
Char. 192: 1 → 0
Char. 202: 1 → 0
Char. 211: 0 → 1
Char. 222: 1 → 0
Char. 237: 1 → 0
Char. 248: 0 → 1
Char. 249: 0 → 2
Char. 250: 0 → 1
Char. 263: 0 → 1
Char. 273: 0 → 1
Char. 274: 0 → 1
Char. 275: 0 → 1
Char. 276: 0 → 1
Char. 277: 0 → 1
Char. 278: 0 → 3

***Acerosodontosaurus piveteaui*:**
Char. 78: 1 → 0
Char. 81: 1 → 0
Char. 127: 1 → 0
Char. 128: 0 → 1
Char. 155: 0 → 1
Char. 206: 0 → 2
Char. 208: 0 → 1
Char. 278: 0 → 1

***Acleistorhinus pteroticus*:**
Char. 21: 0 → 1
Char. 146: 1 → 0

**Araeoscelidae:**
Char. 0: 0 → 1
Char. 28: 0 → 1
Char. 43: 0 → 1
Char. 89: 0 → 1
Char. 106: 0 → 1
Char. 120: 0 → 1
Char. 193: 0 → 1
Char. 222: 0 → 1
Char. 224: 0 → 1
Char. 234: 0 → 1
Char. 266: 0 → 1
Char. 278: 3 → 1

**Archosauriformes:**
Char. 32: 0 → 1
Char. 94: 1 → 0
Char. 112: 1 → 0
Char. 152: 0 → 1
Char. 154: 01 → 2
Char. 166: 1 → 0
Char. 171: 0 → 1
Char. 185: 0 → 1
Char. 201: 0 → 1
Char. 204: 0 → 1
Char. 218: 0 → 3
Char. 224: 0 → 1
Char. 242: 0 → 1

***Australothyris smithi*:**
Char. 24: 0 → 1
Char. 34: 0 → 1
Char. 55: 1 → 0
Char. 71: 1 → 0
Char. 79: 0 → 1
Char. 85: 1 → 0
Char. 98: 1 → 0
Char. 100: 0 → 1
Char. 103: 0 → 1
Char. 110: 0 → 1
Char. 123: 1 → 0
Char. 129: 0 → 1
Char. 131: 0 → 1
Char. 132: 1 → 0
Char. 144: 1 → 0
Char. 147: 0 → 1
Char. 149: 1 → 0
Char. 150: 0 → 1
Char. 159: 1 → 0
Char. 192: 1 → 0

***Barasaurus besairiei*:**
Char. 33: 1 → 0

***Bashkyroleter bashkyricus*:**
Char. 275: 1 → 0

***Bashkyroleter mesensis*:**
Char. 12: 0 → 1
Char. 66: 1 → 0
Char. 169: 1 → 0

***Belebey vegrandis*:**
Char. 154: 1 → 0

***Bradysaurus* spp.:**
Char. 19: 0 → 1
Char. 25: 1 → 0
Char. 73: 0 → 1
Char. 79: 0 → 1
Char. 135: 1 → 0
Char. 249: 0 → 1

***Candelaria barbouri*:**
Char. 1: 0 → 1
Char. 8: 0 → 1
Char. 15: 0 → 1
Char. 25: 1 → 0
Char. 29: 1 → 0
Char. 33: 1 → 2
Char. 49: 0 → 1
Char. 55: 1 → 0
Char. 67: 0 → 1
Char. 79: 0 → 2
Char. 88: 1 → 0
Char. 89: 0 → 1
Char. 94: 0 → 1
Char. 95: 0 → 1
Char. 126: 0 → 1
Char. 127: 0 → 1
Char. 132: 1 → 0
Char. 154: 1 → 2
Char. 159: 1 → 0
Char. 169: 1 → 0
Char. 276: 0 → 1
Char. 277: 0 → 1

**Captorhinidae:**
Char. 3: 0 → 1
Char. 25: 1 → 0
Char. 26: 0 → 1
Char. 73: 0 → 1
Char. 75: 0 → 1
Char. 108: 1 → 0
Char. 180: 1 → 0
Char. 183: 1 → 0
Char. 201: 1 → 0
Char. 203: 1 → 2
Char. 216: 1 → 0

**Caseidae:**
Char. 38: 1 → 0
Char. 46: 1 → 0
Char. 170: 0 → 1
Char. 194: 0 → 1
Char. 273: 0 → 1
Char. 274: 0 → 1
Char. 278: 3 → 2

***Claudiosaurus germaini*:**
Char. 24: 0 → 1
Char. 27: 0 → 1
Char. 34: 0 → 1
Char. 36: 0 → 1
Char. 56: 0 → 1
Char. 64: 0 → 1
Char. 70: 0 → 1
Char. 84: 1 → 0
Char. 105: 0 → 1
Char. 106: 0 → 1
Char. 117: 01 → 2
Char. 126: 0 → 1
Char. 127: 1 → 0
Char. 130: 0 → 1
Char. 131: 0 → 1
Char. 141: 1 → 0
Char. 144: 1 → 0
Char. 166: 1 → 0
Char. 182: 0 → 1
Char. 187: 1 → 0
Char. 190: 0 → 1
Char. 199: 0 → 1
Char. 201: 0 → 1
Char. 203: 1 → 2
Char. 204: 02 → 1
Char. 222: 1 → 0
Char. 234: 1 → 0
Char. 272: 2 → 1

***Colobomycter pholeter*:**
Char. 21: 0 → 1
Char. 25: 1 → 0
Char. 84: 1 → 0
Char. 154: 1 → 0
Char. 167: 0 → 1
Char. 267: 0 → 1

***Delorhynchus cifelli*:**
Char. 18: 0 → 1
Char. 21: 0 → 1
Char. 23: 1 → 0
Char. 24: 0 → 1
Char. 26: 0 → 1
Char. 28: 0 → 1
Char. 33: 1 → 2
Char. 39: 0 → 1
Char. 52: 0 → 1
Char. 100: 0 → 1
Char. 111: 0 → 1
Char. 116: 1 → 0
Char. 119: 1 → 0
Char. 131: 0 → 1
Char. 147: 0 → 1
Char. 156: 1 → 0
Char. 167: 0 → 1
Char. 189: 0 → 1
Char. 191: 0 → 1
Char. 267: 0 → 1

**Diadectomorpha:**
Char. 0: 0 → 1
Char. 64: 0 → 1
Char. 70: 0 → 1
Char. 122: 0 → 1
Char. 123: 1 → 0
Char. 146: 1 → 0
Char. 275: 1 → 0
Char. 278: 3 → 0

***Emeroleter levis*:**
Char. 51: 1 → 0

**Eosauropterygia:**
Char. 166: 1 → 0
Char. 174: 0 → 1
Char. 178: 0 → 1
Char. 194: 0 → 2
Char. 272: 1 → 0

***Eudibamus cursoris*:**
Char. 154: 1 → 2

***Feeserpeton oklahomensis*:**
Char. 51: 0 → 1
Char. 157: 0 → 1
Char. 158: 0 → 1

***Hovasaurus boulei*:**
Char. 41: 0 → 1
Char. 55: 1 → 0
Char. 60: 1 → 0
Char. 72: 2 → 1
Char. 77: 0 → 2
Char. 78: 1 → 0
Char. 79: 0 → 1
Char. 93: 1 → 0
Char. 113: 0 → 1
Char. 138: 0 → 1
Char. 141: 1 → 0
Char. 146: 1 → 0
Char. 206: 0 → 2
Char. 215: 0 → 1
Char. 219: 1 → 0
Char. 224: 0 → 1
Char. 278: 0 → 3

**Kuehneosauridae:**
Char. 7: 0 → 1
Char. 24: 0 → 1
Char. 27: 0 → 1
Char. 43: 1 → 0
Char. 79: 0 → 2
Char. 107: 1 → 0
Char. 140: 1 → 0
Char. 147: 1 → 0
Char. 148: 1 → 0
Char. 245: 0 → 1
Char. 278: 0 → 3

***Lanthanosuchus watsoni*:**
Char. 25: 1 → 0
Char. 51: 0 → 1
Char. 86: 0 → 1
Char. 98: 1 → 0
Char. 138: 0 → 1
Char. 144: 1 → 0
Char. 154: 1 → 2

***Macroleter poezicus*:**
Char. 9: 0 → 1
Char. 26: 0 → 1
Char. 66: 1 → 2
Char. 126: 0 → 1
Char. 134: 0 → 2
Char. 140: 0 → 1
Char. 146: 1 → 0
Char. 169: 1 → 0

***Mesosaurus* spp.:**
Char. 0: 0 → 1
Char. 2: 0 → 1
Char. 6: 0 → 1
Char. 8: 0 → 1
Char. 9: 0 → 1
Char. 13: 0 → 1
Char. 19: 0 → 1
Char. 26: 0 → 1
Char. 29: 1 → 0
Char. 38: 1 → 0
Char. 41: 0 → 1
Char. 67: 0 → 1
Char. 84: 1 → 0
Char. 85: 1 → 0
Char. 94: 0 → 1
Char. 107: 0 → 1
Char. 109: 0 → 1
Char. 111: 0 → 1
Char. 115: 0 → 1
Char. 146: 1 → 0
Char. 149: 1 → 0
Char. 159: 1 → 0
Char. 164: 0 → 1
Char. 166: 1 → 0
Char. 167: 0 → 1
Char. 176: 0 → 1
Char. 183: 1 → 0
Char. 184: 0 → 1
Char. 192: 1 → 0
Char. 199: 0 → 1
Char. 202: 0 → 1
Char. 206: 0 → 1
Char. 209: 0 → 1
Char. 217: 0 → 1
Char. 219: 0 → 1
Char. 220: 0 → 1
Char. 231: 0 → 1
Char. 260: 2 → 0
Char. 272: 2 → 0
Char. 278: 3 → 0

***Microleter mckinzieorum*:**
Char. 0: 0 → 1
Char. 18: 0 → 1
Char. 39: 0 → 1
Char. 70: 0 → 1
Char. 94: 0 → 1
Char. 106: 0 → 1
Char. 110: 0 → 1
Char. 278: 3 → 1

**Millerettidae:**
Char. 23: 1 → 0
Char. 24: 0 → 1
Char. 25: 1 → 0
Char. 33: 1 → 0
Char. 44: 1 → 0
Char. 56: 0 → 1
Char. 66: 0 → 2
Char. 78: 1 → 0
Char. 80: 1 → 0
Char. 84: 1 → 2
Char. 88: 1 → 0
Char. 96: 1 → 0
Char. 121: 0 → 1
Char. 124: 0 → 1
Char. 127: 0 → 1
Char. 135: 0 → 1
Char. 145: 0 → 1
Char. 159: 1 → 0
Char. 166: 1 → 0
Char. 180: 1 → 0
Char. 192: 1 → 0
Char. 202: 0 → 1
Char. 211: 0 → 1
Char. 230: 0 → 1
Char. 234: 0 → 1
Char. 248: 0 → 1
Char. 252: 0 → 1
Char. 253: 0 → 1

***Nycteroleter ineptus*:**
Char. 66: 1 → 0

***Nyctiphruretus acudens*:**
Char. 21: 0 → 1
Char. 41: 0 → 1
Char. 66: 1 → 2
Char. 81: 1 → 0
Char. 84: 1 → 2
Char. 94: 0 → 1
Char. 166: 1 → 0
Char. 224: 0 → 1
Char. 266: 0 → 1
Char. 272: 2 → 1
Char. 276: 0 → 1

***Orovenator mayorum*:**
Char. 8: 0 → 1
Char. 23: 1 → 0
Char. 24: 0 → 1
Char. 33: 1 → 0
Char. 36: 0 → 1
Char. 50: 0 → 1
Char. 92: 1 → 0
Char. 94: 1 → 0
Char. 135: 1 → 0
Char. 159: 1 → 0
Char. 160: 0 → 1
Char. 165: 0 → 1
Char. 278: 0 → 1

***Owenetta* spp.:**
Char. 142: 1 → 0
Char. 169: 1 → 0

***Paleothyris acadiana*:**
Char. 23: 1 → 0
Char. 66: 1 → 2
Char. 102: 0 → 1
Char. 146: 1 → 0

***Placodus* spp.:**
Char. 0: 1 → 2
Char. 9: 0 → 1
Char. 12: 0 → 1
Char. 13: 0 → 1
Char. 19: 0 → 1
Char. 31: 0 → 1
Char. 46: 1 → 0
Char. 57: 0 → 1
Char. 78: 1 → 0
Char. 93: 1 → 0
Char. 102: 1 → 2
Char. 109: 1 → 0
Char. 140: 1 → 0
Char. 155: 0 → 1
Char. 163: 1 → 0
Char. 164: 0 → 1

***Procolophon* spp.:**
Char. 41: 0 → 1
Char. 69: 0 → 1
Char. 79: 0 → 1
Char. 86: 0 → 1
Char. 101: 0 → 1
Char. 141: 0 → 1
Char. 149: 1 → 0
Char. 203: 1 → 2
Char. 215: 0 → 1
Char. 230: 0 → 1
Char. 235: 1 → 2
Char. 237: 0 → 1
Char. 238: 0 → 1
Char. 272: 2 → 1
Char. 278: 3 → 0

***Prolacerta broomi*:**
Char. 58: 1 → 0
Char. 66: 1 → 0
Char. 67: 1 → 0
Char. 80: 1 → 0
Char. 139: 1 → 0
Char. 147: 1 → 0
Char. 192: 1 → 0
Char. 203: 1 → 2
Char. 206: 0 → 12

***Rhipaeosaurus* spp.:**
Char. 172: 0 → 1
Char. 183: 1 → 2
Char. 194: 0 → 1
Char. 201: 1 → 0
Char. 211: 0 → 1
Char. 215: 0 → 1
Char. 226: 0 → 1
Char. 235: 1 → 0
Char. 252: 0 → 1
Char. 277: 0 → 1

**Rhynchocephalia:**
Char. 0: 1 → 2
Char. 23: 1 → 0
Char. 24: 0 → 1
Char. 77: 0 → 1
Char. 88: 1 → 0
Char. 94: 1 → 0
Char. 139: 1 → 0
Char. 167: 1 → 0
Char. 205: 1 → 0

**Rhynchosauria:**
Char. 0: 1 → 0
Char. 7: 0 → 1
Char. 9: 0 → 1
Char. 26: 0 → 1
Char. 44: 1 → 0
Char. 68: 0 → 1
Char. 99: 1 → 0
Char. 150: 1 → 0
Char. 160: 0 → 1
Char. 161: 0 → 1
Char. 171: 0 → 2
Char. 223: 0 → 1
Char. 224: 0 → 1
Char. 241: 0 → 1

***Scutosaurus* spp.:**
Char. 175: 0 → 1
Char. 190: 0 → 1
Char. 218: 0 → 1
Char. 243: 0 → 2
Char. 244: 0 → 1
Char. 251: 0 → 1

***Sinosaurosphargis yunguiensis*:**
Char. 8: 0 → 1
Char. 30: 0 → 1
Char. 53: 0 → 1
Char. 89: 1 → 0
Char. 127: 1 → 0
Char. 150: 1 → 0
Char. 154: 0 → 2
Char. 167: 1 → 0
Char. 253: 0 → 1
Char. 255: 0 → 1

**Squamata:**
Char. 45: 0 → 1
Char. 79: 0 → 2
Char. 80: 1 → 0
Char. 92: 1 → 0
Char. 109: 1 → 0
Char. 160: 0 → 1
Char. 200: 0 → 1
Char. 245: 0 → 1

***Trilophosaurus buettneri*:**
Char. 5: 1 → 0
Char. 11: 0 → 1
Char. 55: 1 → 0
Char. 93: 1 → 0
Char. 104: 0 → 1
Char. 106: 0 → 1
Char. 113: 0 → 1
Char. 122: 0 → 1
Char. 136: 1 → 0
Char. 144: 1 → 0
Char. 157: 0 → 1
Char. 159: 1 → 0
Char. 177: 0 → 12
Char. 194: 0 → 1
Char. 203: 1 → 2
Char. 207: 1 → 0
Char. 208: 1 → 0
Char. 272: 1 → 0

***Youngina capensis*:**
Char. 5: 1 → 0
Char. 23: 1 → 0
Char. 25: 0 → 1
Char. 27: 0 → 1
Char. 38: 1 → 0
Char. 44: 1 → 0
Char. 56: 0 → 1
Char. 75: 0 → 1
Char. 84: 1 → 0
Char. 92: 1 → 0
Char. 94: 1 → 0
Char. 134: 0 → 1
Char. 163: 1 → 0
Char. 170: 0 → 1
Char. 211: 0 → 1
Char. 215: 0 → 1
Char. 224: 0 → 1
Char. 239: 0 → 1

**Node 50:**
Char. 46: 1 → 0
Char. 88: 1 → 0
Char. 89: 1 → 0
Char. 93: 1 → 0
Char. 176: 0 → 1
Char. 195: 0 → 2
Char. 198: 0 → 1
Char. 246: 1 → 2
Char. 259: 0 → 1
Char. 265: 1 → 0
Char. 270: 0 → 1

**Node 51:**
Char. 65: 0 → 1
Char. 131: 0 → 1
Char. 184: 0 → 1
Char. 205: 0 → 1
Char. 210: 0 → 1
Char. 219: 1 → 0
Char. 241: 0 → 1
Char. 246: 0 → 1
Char. 254: 0 → 1
Char. 255: 0 → 1
Char. 256: 0 → 1
Char. 268: 0 → 1
Char. 269: 0 → 1

**Node 52:**
Char. 0: 1 → 2
Char. 15: 0 → 1
Char. 33: 1 → 2
Char. 44: 1 → 0
Char. 62: 1 → 0
Char. 64: 0 → 1
Char. 84: 1 → 2
Char. 130: 0 → 1
Char. 134: 0 → 2
Char. 147: 0 → 1
Char. 152: 0 → 1
Char. 155: 0 → 1
Char. 158: 0 → 1
Char. 161: 0 → 1
Char. 174: 0 → 1

Char. 181: 0 → 1
Char. 203: 1 → 2
Char. 247: 0 → 1
Char. 251: 0 → 1
Char. 252: 0 → 1
Char. 253: 0 → 2

**Node 53:**
Char. 0: 0 → 1
Char. 25: 1 → 0
Char. 29: 1 → 0
Char. 40: 2 → 0
Char. 59: 0 → 1
Char. 60: 0 → 1
Char. 62: 0 → 1
Char. 67: 0 → 1
Char. 72: 1 → 2
Char. 89: 0 → 1
Char. 92: 0 → 1
Char. 94: 0 → 1
Char. 109: 0 → 1
Char. 111: 0 → 1
Char. 120: 0 → 1
Char. 127: 0 → 1
Char. 129: 0 → 1
Char. 135: 0 → 1
Char. 136: 0 → 1
Char. 139: 0 → 1
Char. 141: 0 → 1
Char. 179: 0 → 1
Char. 187: 0 → 1
Char. 188: 0 → 1
Char. 191: 0 → 1
Char. 193: 0 → 1
Char. 196: 0 → 1
Char. 201: 1 → 0
Char. 202: 0 → 1
Char. 209: 0 → 1
Char. 219: 0 → 1
Char. 222: 0 → 1
Char. 234: 0 → 1
Char. 237: 0 → 1
Char. 265: 0 → 1
Char. 266: 0 → 1
Char. 275: 1 → 0
Char. 278: 3 → 0

**Node 54:**
Char. 23: 0 → 1
Char. 33: 0 → 1
Char. 132: 0 → 1
Char. 166: 0 → 1
Char. 276: 1 → 0

**Node 55:**
Char. 29: 0 → 1
Char. 84: 0 → 1
Char. 88: 0 → 1
Char. 169: 0 → 1

**Node 56:**
Char. 72: 0 → 1
Char. 81: 0 → 1
Char. 93: 0 → 1
Char. 97: 0 → 1
Char. 104: 1 → 0
Char. 144: 0 → 1
Char. 173: 0 → 1
Char. 183: 0 → 1

**Node 58:**
Char. 95: 0 → 1
Char. 113: 0 → 1
Char. 114: 0 → 1

**Node 59:**
Char. 23: 1 → 0
Char. 47: 0 → 1
Char. 79: 0 → 1
Char. 110: 0 → 1
Char. 131: 0 → 1
Char. 137: 0 → 1
Char. 140: 0 → 2
Char. 147: 0 → 1

**Node 60:**
Char. 29: 1 → 0
Char. 33: 1 → 0
Char. 59: 0 → 1
Char. 60: 0 → 1
Char. 67: 0 → 1
Char. 72: 1 → 2
Char. 84: 1 → 0
Char. 111: 0 → 1
Char. 116: 1 → 0
Char. 154: 1 → 0
Char. 159: 1 → 0
Char. 166: 1 → 0
Char. 169: 1 → 0
Char. 170: 0 → 1
Char. 197: 0 → 1
Char. 221: 1 → 0

**Node 61:**
Char. 19: 0 → 1
Char. 92: 1 → 0

**Node 62:**
Char. 4: 0 → 1
Char. 15: 0 → 1
Char. 29: 0 → 1
Char. 213: 0 → 1
Char. 226: 0 → 2
Char. 228: 0 → 1
Char. 275: 0 → 1

**Node 63:**
Char. 58: 0 → 1
Char. 61: 0 → 1
Char. 66: 0 → 1
Char. 69: 0 → 1
Char. 107: 0 → 1
Char. 126: 0 → 1
Char. 131: 0 → 1
Char. 134: 0 → 1
Char. 140: 0 → 1
Char. 147: 0 → 1
Char. 150: 0 → 1
Char. 167: 0 → 1
Char. 176: 0 → 1
Char. 190: 0 → 1
Char. 205: 0 → 1
Char. 208: 0 → 1
Char. 230: 0 → 1
Char. 239: 0 → 1
Char. 260: 2 → 1

**Node 64:**
Char. 73: 0 → 1
Char. 205: 0 → 1
Char. 273: 0 → 1
Char. 276: 0 → 1

**Node 65:**
Char. 18: 0 → 1
Char. 25: 1 → 0
Char. 37: 0 → 1
Char. 103: 0 → 1
Char. 106: 0 → 1
Char. 107: 0 → 1
Char. 126: 0 → 1
Char. 150: 0 → 1

**Node 66:**
Char. 38: 1 → 2
Char. 39: 0 → 1
Char. 44: 1 → 0
Char. 49: 0 → 1
Char. 66: 0 → 1
Char. 80: 1 → 0
Char. 95: 0 → 1
Char. 135: 0 → 1
Char. 137: 0 → 1
Char. 147: 0 → 1
Char. 157: 0 → 1
Char. 158: 0 → 1
Char. 183: 1 → 2
Char. 186: 0 → 1
Char. 187: 0 → 1
Char. 194: 0 → 1
Char. 197: 0 → 1
Char. 201: 1 → 0
Char. 211: 0 → 1
Char. 252: 0 → 1

**Node 68:**
Char. 133: 0 → 1

**Node 69:**
Char. 100: 0 → 1
Char. 113: 0 → 1

**Node 70:**
Char. 23: 1 → 0
Char. 70: 0 → 1
Char. 118: 0 → 1
Char. 125: 0 → 1

**Node 72:**
Char. 38: 1 → 2
Char. 39: 0 → 1
Char. 58: 0 → 1
Char. 59: 0 → 1
Char. 60: 0 → 1
Char. 72: 1 → 2
Char. 85: 1 → 0
Char. 88: 1 → 0
Char. 95: 0 → 1
Char. 104: 0 → 1
Char. 105: 0 → 1
Char. 106: 0 → 2
Char. 107: 0 → 1
Char. 109: 0 → 1
Char. 110: 0 → 1
Char. 146: 1 → 0
Char. 155: 0 → 2
Char. 183: 1 → 2

**Node 73:**
Char. 23: 0 → 1
Char. 33: 1 → 0
Char. 38: 2 → 1
Char. 39: 1 → 0
Char. 42: 0 → 1
Char. 43: 0 → 1
Char. 46: 1 → 0
Char. 49: 1 → 0
Char. 71: 1 → 0
Char. 83: 0 → 2
Char. 101: 0 → 1
Char. 103: 0 → 1
Char. 106: 0 → 1
Char. 161: 0 → 1
Char. 163: 1 → 0
Char. 172: 0 → 2
Char. 174: 0 → 1
Char. 188: 0 → 1
Char. 203: 1 → 2
Char. 212: 0 → 1
Char. 216: 1 → 0
Char. 221: 1 → 0
Char. 236: 0 → 1
Char. 238: 0 → 2
Char. 241: 0 → 1
Char. 242: 0 → 1
Char. 273: 0 → 1
Char. 274: 0 → 1
Char. 275: 1 → 0

**Node 74:**
Char. 52: 0 → 1
Char. 75: 0 → 1
Char. 84: 1 → 0
Char. 121: 0 → 1
Char. 180: 1 → 0
Char. 230: 0 → 1

**Node 75:**
Char. 44: 1 → 0
Char. 66: 0 → 1
Char. 78: 1 → 0
Char. 88: 1 → 0
Char. 93: 1 → 0
Char. 123: 1 → 0
Char. 129: 0 → 1
Char. 192: 1 → 0

**Node 76:**
Char. 33: 1 → 0
Char. 85: 1 → 0
Char. 107: 0 → 1

**Node 77:**
Char. 2: 0 → 1
Char. 6: 0 → 1
Char. 186: 0 → 1
Char. 198: 0 → 1
Char. 200: 0 → 1
Char. 220: 0 → 1
Char. 239: 1 → 0

**Node 78:**
Char. 98: 1 → 0
Char. 113: 0 → 1
Char. 181: 0 → 1
Char. 206: 0 → 2

**Node 79:**
Char. 35: 0 → 1
Char. 102: 0 → 1
Char. 103: 0 → 1
Char. 184: 0 → 1
Char. 201: 0 → 1
Char. 210: 0 → 1
Char. 223: 0 → 1
Char. 229: 0 → 1

**Node 80:**
Char. 17: 0 → 1
Char. 42: 0 → 1
Char. 251: 0 → 1
Char. 264: 0 → 1

**Node 81:**
Char. 41: 0 → 1
Char. 61: 1 → 2
Char. 112: 1 → 0
Char. 138: 0 → 1
Char. 146: 1 → 0
Char. 155: 0 → 1
Char. 192: 1 → 2
Char. 224: 0 → 1
Char. 226: 0 → 1
Char. 227: 0 → 1
Char. 233: 0 → 1
Char. 267: 01 → 2

**Node 82:**
Char. 61: 1 → 3
Char. 90: 0 → 1
Char. 91: 0 → 1
Char. 109: 1 → 0
Char. 155: 0 → 1
Char. 209: 1 → 0

## ANALYSIS 37
## (ALL TAXA, IMPLIED WEIGHTING, K = 2.625)

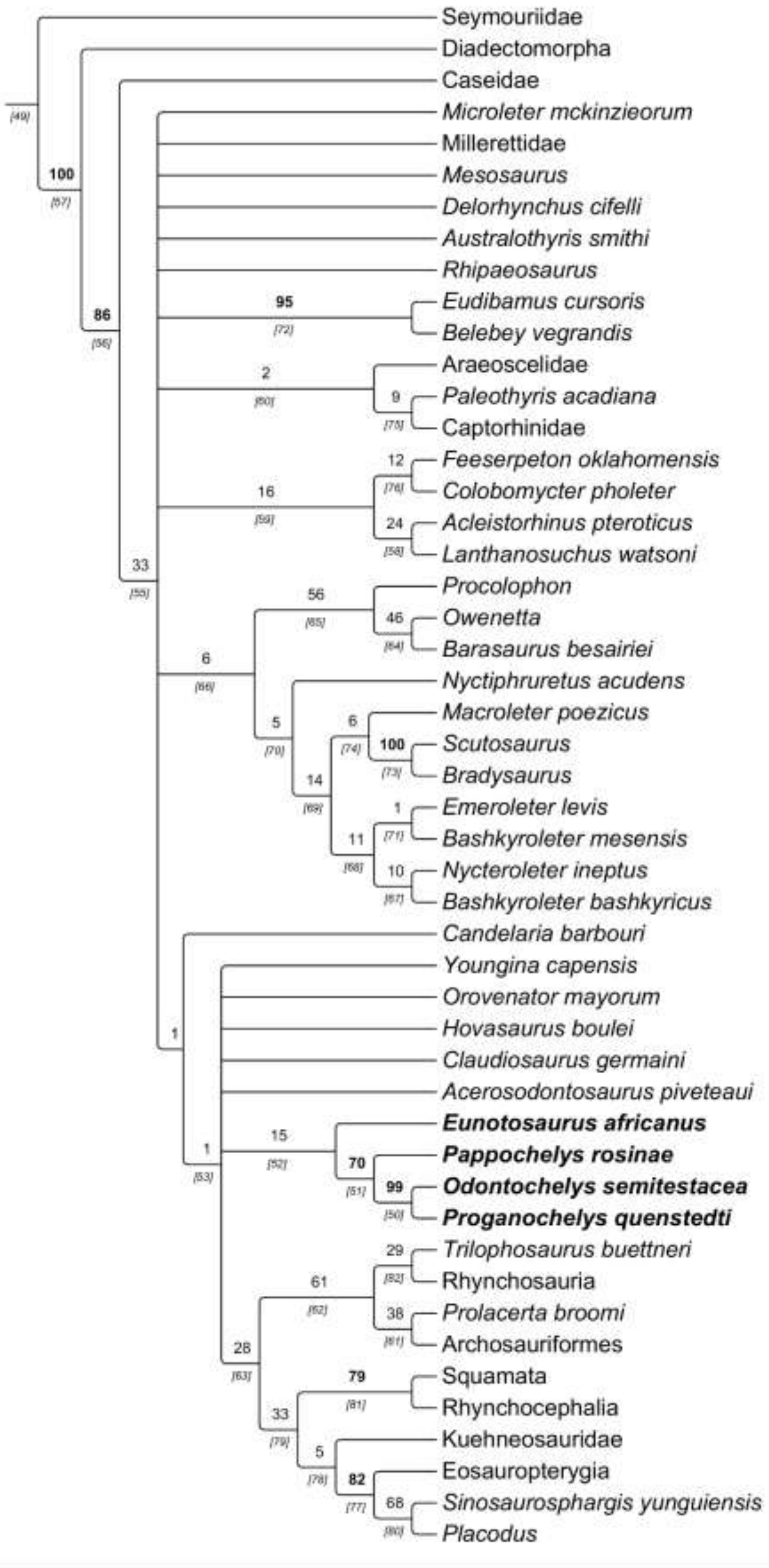

***Proganochelys quenstedti*:**
Char. 8: 0 → 1
Char. 11: 0 → 1
Char. 106: 0 → 1
Char. 108: 1 → 0
Char. 128: 0 → 1
Char. 175: 0 → 1
Char. 202: 1 → 0
Char. 207: 1 → 0
Char. 209: 1 → 0
Char. 244: 0 → 1
Char. 248: 0 → 1
Char. 250: 0 → 1
Char. 252: 1 → 0
Char. 262: 0 → 1

***Pappochelys rosinae*:**
Char. 0: 2 → 0
Char. 1: 0 → 1
Char. 5: 1 → 0
Char. 12: 0 → 1
Char. 41: 0 → 1
Char. 48: 0 → 1
Char. 49: 0 → 1
Char. 75: 0 → 1
Char. 169: 1 → 0
Char. 260: 2 → 0

***Eunotosaurus africanus*:**
Char. 19: 0 → 1
Char. 59: 1 → 0
Char. 60: 1 → 0
Char. 72: 2 → 1
Char. 76: 0 → 1
Char. 97: 1 → 0
Char. 103: 0 → 1
Char. 153: 1 → 0
Char. 191: 1 → 0
Char. 192: 1 → 0
Char. 202: 1 → 0
Char. 211: 0 → 1
Char. 222: 1 → 0
Char. 237: 1 → 0
Char. 248: 0 → 1
Char. 249: 0 → 2
Char. 250: 0 → 1
Char. 263: 0 → 1
Char. 273: 0 → 1
Char. 274: 0 → 1
Char. 275: 0 → 1
Char. 276: 0 → 1
Char. 277: 0 → 1
Char. 278: 0 → 3

***Acerosodontosaurus piveteaui*:**
Char. 78: 1 → 0
Char. 81: 1 → 0
Char. 127: 1 → 0
Char. 128: 0 → 1
Char. 155: 0 → 1
Char. 206: 0 → 2
Char. 208: 0 → 1
Char. 278: 0 → 1

***Acleistorhinus pteroticus*:**
Char. 21: 0 → 1
Char. 146: 1 → 0

**Araeoscelidae:**
Char. 0: 0 → 1
Char. 28: 0 → 1
Char. 43: 0 → 1
Char. 48: 0 → 1
Char. 57: 1 → 0
Char. 89: 0 → 1
Char. 106: 0 → 1
Char. 112: 0 → 1
Char. 120: 0 → 1
Char. 193: 0 → 1
Char. 222: 0 → 1
Char. 224: 0 → 1
Char. 234: 0 → 1
Char. 266: 0 → 1
Char. 278: 3 → 1

**Archosauriformes:**
Char. 32: 0 → 1
Char. 94: 1 → 0
Char. 112: 1 → 0
Char. 152: 0 → 1
Char. 154: 01 → 2
Char. 166: 1 → 0
Char. 171: 0 → 1
Char. 185: 0 → 1
Char. 201: 0 → 1
Char. 204: 0 → 1
Char. 218: 0 → 3
Char. 224: 0 → 1
Char. 242: 0 → 1

***Australothyris smithi*:**
Char. 24: 0 → 1
Char. 34: 0 → 1
Char. 55: 1 → 0
Char. 57: 1 → 0
Char. 71: 1 → 0
Char. 79: 0 → 1
Char. 98: 1 → 0
Char. 100: 0 → 1
Char. 103: 0 → 1
Char. 110: 0 → 1
Char. 112: 0 → 1
Char. 123: 1 → 0
Char. 129: 0 → 1
Char. 131: 0 → 1
Char. 132: 1 → 0
Char. 144: 1 → 0
Char. 147: 0 → 1
Char. 149: 1 → 0
Char. 150: 0 → 1
Char. 159: 1 → 0
Char. 192: 1 → 0

***Barasaurus besairiei*:**
Char. 33: 1 → 0

***Bashkyroleter bashkyricus*:**
Char. 275: 1 → 0

***Bashkyroleter mesensis*:**
Char. 12: 0 → 1
Char. 66: 1 → 0
Char. 169: 1 → 0

***Belebey vegrandis*:**
Char. 154: 1 → 0

***Bradysaurus* spp.:**
Char. 19: 0 → 1
Char. 25: 1 → 0
Char. 73: 0 → 1
Char. 79: 0 → 1
Char. 135: 1 → 0
Char. 249: 0 → 1

***Candelaria barbouri*:**
Char. 1: 0 → 1
Char. 8: 0 → 1
Char. 15: 0 → 1
Char. 33: 1 → 2
Char. 49: 0 → 1
Char. 55: 1 → 0
Char. 79: 0 → 2
Char. 88: 1 → 0
Char. 95: 0 → 1
Char. 126: 0 → 1
Char. 132: 1 → 0
Char. 154: 1 → 2
Char. 159: 1 → 0
Char. 169: 1 → 0
Char. 276: 0 → 1
Char. 277: 0 → 1

**Captorhinidae:**
Char. 3: 0 → 1
Char. 25: 1 → 0
Char. 26: 0 → 1
Char. 73: 0 → 1
Char. 75: 0 → 1
Char. 108: 1 → 0
Char. 180: 1 → 0
Char. 183: 1 → 0
Char. 201: 1 → 0
Char. 203: 1 → 2
Char. 216: 1 → 0

**Caseidae:**
Char. 24: 0 → 1
Char. 25: 1 → 0
Char. 36: 0 → 1
Char. 38: 1 → 0
Char. 46: 1 → 0
Char. 50: 1 → 0
Char. 56: 0 → 1
Char. 170: 0 → 1
Char. 194: 0 → 1
Char. 273: 0 → 1
Char. 274: 0 → 1
Char. 278: 3 → 2

***Claudiosaurus germaini*:**
Char. 24: 0 → 1
Char. 27: 0 → 1
Char. 34: 0 → 1
Char. 36: 0 → 1
Char. 48: 0 → 1
Char. 56: 0 → 1
Char. 64: 0 → 1
Char. 70: 0 → 1
Char. 84: 1 → 0
Char. 105: 0 → 1
Char. 106: 0 → 1
Char. 117: 01 → 2
Char. 126: 0 → 1
Char. 127: 1 → 0
Char. 130: 0 → 1
Char. 131: 0 → 1
Char. 141: 1 → 0
Char. 144: 1 → 0
Char. 166: 1 → 0
Char. 182: 0 → 1
Char. 187: 1 → 0
Char. 190: 0 → 1
Char. 199: 0 → 1
Char. 201: 0 → 1
Char. 203: 1 → 2
Char. 204: 02 → 1
Char. 222: 1 → 0
Char. 234: 1 → 0
Char. 272: 2 → 1

***Colobomycter pholeter*:**
Char. 21: 0 → 1
Char. 25: 1 → 0
Char. 84: 1 → 0
Char. 154: 1 → 0
Char. 167: 0 → 1
Char. 267: 0 → 1

***Delorhynchus cifelli*:**
Char. 18: 0 → 1
Char. 20: 0 → 1
Char. 21: 0 → 1
Char. 24: 0 → 1
Char. 26: 0 → 1
Char. 28: 0 → 1
Char. 33: 0 → 2
Char. 39: 0 → 1
Char. 48: 0 → 1
Char. 52: 0 → 1
Char. 100: 0 → 1
Char. 111: 0 → 1
Char. 116: 1 → 0
Char. 119: 1 → 0
Char. 131: 0 → 1
Char. 147: 0 → 1
Char. 156: 1 → 0
Char. 167: 0 → 1
Char. 189: 0 → 1
Char. 191: 0 → 1
Char. 267: 0 → 1

**Diadectomorpha:**
Char. 0: 0 → 1
Char. 64: 0 → 1
Char. 70: 0 → 1
Char. 122: 0 → 1
Char. 123: 1 → 0
Char. 146: 1 → 0
Char. 275: 1 → 0
Char. 278: 3 → 0

***Emeroleter levis*:**
Char. 51: 1 → 0

**Eosauropterygia:**
Char. 166: 1 → 0
Char. 174: 0 → 1
Char. 178: 0 → 1
Char. 194: 0 → 2
Char. 272: 1 → 0

***Eudibamus cursoris*:**
Char. 154: 1 → 2

***Feeserpeton oklahomensis*:**
Char. 51: 0 → 1
Char. 157: 0 → 1
Char. 158: 0 → 1

***Hovasaurus boulei*:**
Char. 41: 0 → 1
Char. 48: 0 → 1
Char. 55: 1 → 0
Char. 60: 1 → 0
Char. 72: 2 → 1
Char. 77: 0 → 2
Char. 78: 1 → 0
Char. 79: 0 → 1
Char. 93: 1 → 0
Char. 113: 0 → 1
Char. 138: 0 → 1
Char. 141: 1 → 0
Char. 146: 1 → 0
Char. 206: 0 → 2
Char. 215: 0 → 1
Char. 219: 1 → 0
Char. 224: 0 → 1
Char. 278: 0 → 3

**Kuehneosauridae:**
Char. 7: 0 → 1
Char. 24: 0 → 1
Char. 27: 0 → 1
Char. 43: 1 → 0
Char. 79: 0 → 2
Char. 107: 1 → 0
Char. 140: 1 → 0
Char. 147: 1 → 0
Char. 148: 1 → 0
Char. 245: 0 → 1
Char. 278: 0 → 3

***Lanthanosuchus watsoni*:**
Char. 25: 1 → 0
Char. 51: 0 → 1
Char. 86: 0 → 1
Char. 98: 1 → 0
Char. 138: 0 → 1
Char. 144: 1 → 0
Char. 154: 1 → 2

***Macroleter poezicus*:**
Char. 9: 0 → 1
Char. 26: 0 → 1
Char. 66: 1 → 2
Char. 126: 0 → 1
Char. 134: 0 → 2
Char. 140: 0 → 1
Char. 146: 1 → 0
Char. 169: 1 → 0

***Mesosaurus* spp.:**
Char. 0: 0 → 1
Char. 2: 0 → 1
Char. 6: 0 → 1
Char. 8: 0 → 1
Char. 9: 0 → 1
Char. 13: 0 → 1
Char. 19: 0 → 1
Char. 26: 0 → 1
Char. 29: 1 → 0
Char. 33: 0 → 1
Char. 38: 1 → 0
Char. 41: 0 → 1
Char. 48: 0 → 1
Char. 50: 1 → 0
Char. 67: 0 → 1
Char. 84: 1 → 0
Char. 94: 0 → 1
Char. 107: 0 → 1
Char. 109: 0 → 1
Char. 111: 0 → 1
Char. 115: 0 → 1
Char. 146: 1 → 0
Char. 149: 1 → 0
Char. 159: 1 → 0
Char. 164: 0 → 1
Char. 166: 1 → 0
Char. 167: 0 → 1
Char. 176: 0 → 1
Char. 183: 1 → 0
Char. 184: 0 → 1
Char. 192: 1 → 0
Char. 199: 0 → 1
Char. 202: 0 → 1
Char. 206: 0 → 1
Char. 209: 0 → 1
Char. 217: 0 → 1
Char. 219: 0 → 1
Char. 220: 0 → 1
Char. 231: 0 → 1
Char. 260: 2 → 0
Char. 272: 2 → 0
Char. 278: 3 → 0

***Microleter mckinzieorum*:**
Char. 0: 0 → 1
Char. 18: 0 → 1
Char. 24: 0 → 1
Char. 25: 1 → 0
Char. 36: 0 → 1
Char. 39: 0 → 1
Char. 51: 0 → 2
Char. 56: 0 → 1
Char. 57: 1 → 0
Char. 70: 0 → 1
Char. 79: 0 → 1
Char. 94: 0 → 1
Char. 106: 0 → 1
Char. 110: 0 → 1
Char. 132: 1 → 0
Char. 166: 1 → 0
Char. 276: 0 → 1
Char. 278: 3 → 1

**Millerettidae:**
Char. 24: 0 → 1
Char. 25: 1 → 0
Char. 44: 1 → 0
Char. 56: 0 → 1
Char. 57: 1 → 0
Char. 66: 0 → 2
Char. 78: 1 → 0
Char. 80: 1 → 0
Char. 84: 1 → 2
Char. 88: 1 → 0
Char. 96: 1 → 0
Char. 121: 0 → 1
Char. 124: 0 → 1
Char. 127: 0 → 1
Char. 135: 0 → 1
Char. 145: 0 → 1
Char. 159: 1 → 0
Char. 166: 1 → 0
Char. 180: 1 → 0
Char. 192: 1 → 0
Char. 202: 0 → 1
Char. 211: 0 → 1
Char. 230: 0 → 1
Char. 234: 0 → 1
Char. 248: 0 → 1
Char. 252: 0 → 1
Char. 253: 0 → 1

***Nycteroleter ineptus*:**
Char. 66: 1 → 0

***Nyctiphruretus acudens*:**
Char. 21: 0 → 1
Char. 41: 0 → 1
Char. 66: 1 → 2
Char. 81: 1 → 0
Char. 84: 1 → 2
Char. 94: 0 → 1
Char. 166: 1 → 0
Char. 224: 0 → 1
Char. 266: 0 → 1
Char. 272: 2 → 1
Char. 276: 0 → 1

***Orovenator mayorum*:**
Char. 8: 0 → 1
Char. 24: 0 → 1
Char. 33: 1 → 0
Char. 36: 0 → 1
Char. 50: 0 → 1
Char. 92: 1 → 0
Char. 94: 1 → 0
Char. 135: 1 → 0
Char. 159: 1 → 0
Char. 160: 0 → 1
Char. 165: 0 → 1
Char. 278: 0 → 1

***Owenetta* spp.:**
Char. 142: 1 → 0
Char. 169: 1 → 0

***Paleothyris acadiana*:**
Char. 66: 1 → 2
Char. 102: 0 → 1
Char. 146: 1 → 0

***Placodus* spp.:**
Char. 0: 1 → 2
Char. 9: 0 → 1
Char. 12: 0 → 1
Char. 13: 0 → 1
Char. 19: 0 → 1
Char. 31: 0 → 1
Char. 46: 1 → 0
Char. 57: 0 → 1
Char. 78: 1 → 0
Char. 93: 1 → 0
Char. 102: 1 → 2
Char. 109: 1 → 0
Char. 140: 1 → 0
Char. 155: 0 → 1
Char. 163: 1 → 0
Char. 164: 0 → 1

***Procolophon* spp.:**
Char. 41: 0 → 1
Char. 69: 0 → 1
Char. 79: 0 → 1
Char. 86: 0 → 1
Char. 101: 0 → 1
Char. 141: 0 → 1
Char. 149: 1 → 0
Char. 203: 1 → 2
Char. 215: 0 → 1
Char. 230: 0 → 1
Char. 235: 1 → 2
Char. 237: 0 → 1
Char. 238: 0 → 1
Char. 272: 2 → 1
Char. 278: 3 → 0

***Prolacerta broomi*:**
Char. 58: 1 → 0
Char. 66: 1 → 0
Char. 67: 1 → 0
Char. 80: 1 → 0
Char. 139: 1 → 0
Char. 147: 1 → 0
Char. 192: 1 → 0
Char. 203: 1 → 2
Char. 206: 0 → 12

***Rhipaeosaurus* spp.:**
Char. 172: 0 → 1
Char. 183: 1 → 2
Char. 194: 0 → 1
Char. 201: 1 → 0
Char. 211: 0 → 1
Char. 215: 0 → 1
Char. 226: 0 → 1
Char. 235: 1 → 0
Char. 252: 0 → 1
Char. 277: 0 → 1

**Rhynchocephalia:**
Char. 0: 1 → 2
Char. 24: 0 → 1
Char. 77: 0 → 1
Char. 88: 1 → 0
Char. 94: 1 → 0
Char. 139: 1 → 0
Char. 167: 1 → 0
Char. 205: 1 → 0

**Rhynchosauria:**
Char. 0: 1 → 0
Char. 7: 0 → 1
Char. 9: 0 → 1
Char. 26: 0 → 1
Char. 44: 1 → 0
Char. 68: 0 → 1
Char. 99: 1 → 0
Char. 150: 1 → 0
Char. 160: 0 → 1
Char. 161: 0 → 1
Char. 171: 0 → 2
Char. 223: 0 → 1
Char. 224: 0 → 1
Char. 241: 0 → 1

***Scutosaurus* spp.:**
Char. 175: 0 → 1
Char. 190: 0 → 1
Char. 218: 0 → 1
Char. 243: 0 → 2
Char. 244: 0 → 1
Char. 251: 0 → 1

***Sinosaurosphargis yunguiensis*:**
Char. 8: 0 → 1
Char. 30: 0 → 1
Char. 53: 0 → 1
Char. 89: 1 → 0
Char. 127: 1 → 0
Char. 150: 1 → 0
Char. 154: 0 → 2
Char. 167: 1 → 0
Char. 253: 0 → 1
Char. 255: 0 → 1

**Squamata:**
Char. 45: 0 → 1
Char. 79: 0 → 2
Char. 80: 1 → 0
Char. 92: 1 → 0
Char. 109: 1 → 0
Char. 160: 0 → 1
Char. 200: 0 → 1
Char. 245: 0 → 1

***Trilophosaurus buettneri*:**
Char. 5: 1 → 0
Char. 11: 0 → 1
Char. 55: 1 → 0
Char. 93: 1 → 0
Char. 104: 0 → 1
Char. 106: 0 → 1
Char. 113: 0 → 1
Char. 122: 0 → 1
Char. 136: 1 → 0
Char. 144: 1 → 0
Char. 157: 0 → 1
Char. 159: 1 → 0
Char. 177: 0 → 12
Char. 194: 0 → 1
Char. 203: 1 → 2
Char. 207: 1 → 0
Char. 208: 1 → 0
Char. 272: 1 → 0

***Youngina capensis*:**
Char. 5: 1 → 0
Char. 25: 0 → 1
Char. 27: 0 → 1
Char. 38: 1 → 0
Char. 44: 1 → 0
Char. 48: 0 → 1
Char. 56: 0 → 1
Char. 75: 0 → 1
Char. 84: 1 → 0
Char. 92: 1 → 0
Char. 94: 1 → 0
Char. 134: 0 → 1
Char. 163: 1 → 0
Char. 170: 0 → 1
Char. 211: 0 → 1
Char. 215: 0 → 1
Char. 224: 0 → 1
Char. 239: 0 → 1

**Node 50:**
Char. 46: 1 → 0
Char. 88: 1 → 0
Char. 89: 1 → 0
Char. 93: 1 → 0
Char. 176: 0 → 1
Char. 195: 0 → 2
Char. 198: 0 → 1
Char. 246: 1 → 2
Char. 259: 0 → 1
Char. 265: 1 → 0
Char. 270: 0 → 1

**Node 51:**
Char. 65: 0 → 1
Char. 131: 0 → 1
Char. 184: 0 → 1
Char. 205: 0 → 1
Char. 210: 0 → 1
Char. 219: 1 → 0
Char. 241: 0 → 1
Char. 246: 0 → 1
Char. 254: 0 → 1
Char. 255: 0 → 1

Char. 256: 0 → 1
Char. 268: 0 → 1
Char. 269: 0 → 1

**Node 52:**
Char. 0: 1 → 2
Char. 15: 0 → 1
Char. 33: 1 → 2
Char. 44: 1 → 0
Char. 62: 1 → 0
Char. 64: 0 → 1
Char. 84: 1 → 2
Char. 130: 0 → 1
Char. 134: 0 → 2
Char. 147: 0 → 1
Char. 152: 0 → 1
Char. 155: 0 → 1
Char. 158: 0 → 1
Char. 161: 0 → 1
Char. 174: 0 → 1
Char. 181: 0 → 1
Char. 203: 1 → 2
Char. 247: 0 → 1
Char. 251: 0 → 1
Char. 252: 0 → 1
Char. 253: 0 → 2

**Node 53:**
Char. 0: 0 → 1
Char. 59: 0 → 1
Char. 60: 0 → 1
Char. 62: 0 → 1
Char. 72: 1 → 2
Char. 92: 0 → 1
Char. 265: 0 → 1
Char. 275: 1 → 0
Char. 278: 3 → 0

**Node 54:**
Char. 20: 0 → 1
Char. 25: 1 → 0
Char. 29: 1 → 0
Char. 33: 0 → 1
Char. 50: 1 → 0
Char. 57: 1 → 0
Char. 67: 0 → 1
Char. 89: 0 → 1
Char. 94: 0 → 1
Char. 127: 0 → 1

**Node 55:**
Char. 29: 0 → 1
Char. 40: 0 → 2
Char. 74: 0 → 1
Char. 80: 0 → 1
Char. 84: 0 → 1
Char. 88: 0 → 1
Char. 116: 0 → 1
Char. 119: 0 → 1
Char. 132: 0 → 1
Char. 149: 0 → 1
Char. 156: 0 → 1
Char. 166: 0 → 1
Char. 169: 0 → 1
Char. 180: 0 → 1
Char. 192: 0 → 1
Char. 201: 0 → 1
Char. 203: 0 → 1
Char. 221: 0 → 1
Char. 235: 0 → 1

**Node 56:**
Char. 72: 0 → 1
Char. 79: 1 → 0
Char. 81: 0 → 1
Char. 93: 0 → 1
Char. 97: 0 → 1
Char. 104: 1 → 0
Char. 144: 0 → 1
Char. 173: 0 → 1
Char. 183: 0 → 1

**Node 58:**
Char. 95: 0 → 1
Char. 113: 0 → 1
Char. 114: 0 → 1

**Node 59:**
Char. 20: 0 → 1
Char. 47: 0 → 1
Char. 48: 0 → 1
Char. 79: 0 → 1
Char. 110: 0 → 1
Char. 131: 0 → 1
Char. 137: 0 → 1
Char. 140: 0 → 2
Char. 147: 0 → 1

**Node 60:**
Char. 29: 1 → 0
Char. 59: 0 → 1
Char. 60: 0 → 1
Char. 67: 0 → 1
Char. 72: 1 → 2
Char. 84: 1 → 0
Char. 111: 0 → 1
Char. 116: 1 → 0
Char. 154: 1 → 0
Char. 159: 1 → 0
Char. 166: 1 → 0
Char. 169: 1 → 0
Char. 170: 0 → 1
Char. 197: 0 → 1
Char. 221: 1 → 0

**Node 61:**
Char. 19: 0 → 1
Char. 92: 1 → 0

**Node 62:**
Char. 4: 0 → 1
Char. 15: 0 → 1
Char. 29: 0 → 1
Char. 213: 0 → 1
Char. 226: 0 → 2
Char. 228: 0 → 1
Char. 275: 0 → 1

**Node 63:**
Char. 58: 0 → 1
Char. 61: 0 → 1
Char. 66: 0 → 1
Char. 69: 0 → 1
Char. 107: 0 → 1
Char. 126: 0 → 1
Char. 131: 0 → 1
Char. 134: 0 → 1
Char. 140: 0 → 1
Char. 147: 0 → 1
Char. 150: 0 → 1
Char. 167: 0 → 1
Char. 176: 0 → 1
Char. 190: 0 → 1
Char. 205: 0 → 1
Char. 208: 0 → 1
Char. 230: 0 → 1
Char. 239: 0 → 1
Char. 260: 2 → 1

**Node 64:**
Char. 73: 0 → 1
Char. 205: 0 → 1
Char. 273: 0 → 1
Char. 276: 0 → 1

**Node 65:**
Char. 18: 0 → 1
Char. 25: 1 → 0
Char. 37: 0 → 1
Char. 103: 0 → 1
Char. 106: 0 → 1
Char. 107: 0 → 1
Char. 126: 0 → 1
Char. 150: 0 → 1

**Node 66:**
Char. 20: 0 → 1
Char. 33: 0 → 1
Char. 38: 1 → 2
Char. 39: 0 → 1
Char. 44: 1 → 0
Char. 49: 0 → 1
Char. 66: 0 → 1
Char. 80: 1 → 0
Char. 95: 0 → 1
Char. 112: 0 → 1
Char. 135: 0 → 1
Char. 137: 0 → 1
Char. 147: 0 → 1
Char. 157: 0 → 1
Char. 158: 0 → 1
Char. 183: 1 → 2
Char. 186: 0 → 1
Char. 187: 0 → 1
Char. 194: 0 → 1
Char. 197: 0 → 1
Char. 201: 1 → 0
Char. 211: 0 → 1
Char. 252: 0 → 1

**Node 68:**
Char. 133: 0 → 1

**Node 69:**
Char. 100: 0 → 1
Char. 113: 0 → 1

**Node 70:**
Char. 70: 0 → 1
Char. 118: 0 → 1
Char. 125: 0 → 1

**Node 72:**
Char. 38: 1 → 2
Char. 39: 0 → 1
Char. 50: 1 → 0
Char. 58: 0 → 1
Char. 59: 0 → 1
Char. 60: 0 → 1
Char. 72: 1 → 2
Char. 88: 1 → 0
Char. 95: 0 → 1
Char. 104: 0 → 1
Char. 105: 0 → 1
Char. 106: 0 → 2
Char. 107: 0 → 1
Char. 109: 0 → 1
Char. 110: 0 → 1
Char. 146: 1 → 0
Char. 155: 0 → 2
Char. 183: 1 → 2

**Node 73:**
Char. 23: 0 → 1
Char. 33: 1 → 0
Char. 38: 2 → 1
Char. 39: 1 → 0
Char. 42: 0 → 1
Char. 43: 0 → 1
Char. 46: 1 → 0
Char. 49: 1 → 0
Char. 71: 1 → 0
Char. 83: 0 → 2
Char. 101: 0 → 1
Char. 103: 0 → 1
Char. 106: 0 → 1
Char. 161: 0 → 1
Char. 163: 1 → 0
Char. 172: 0 → 2
Char. 174: 0 → 1
Char. 188: 0 → 1
Char. 203: 1 → 2
Char. 212: 0 → 1
Char. 216: 1 → 0
Char. 221: 1 → 0
Char. 236: 0 → 1
Char. 238: 0 → 2
Char. 241: 0 → 1
Char. 242: 0 → 1
Char. 273: 0 → 1
Char. 274: 0 → 1
Char. 275: 1 → 0

**Node 74:**
Char. 52: 0 → 1
Char. 75: 0 → 1
Char. 84: 1 → 0
Char. 121: 0 → 1
Char. 180: 1 → 0
Char. 230: 0 → 1

**Node 75:**
Char. 44: 1 → 0
Char. 66: 0 → 1
Char. 78: 1 → 0
Char. 88: 1 → 0
Char. 93: 1 → 0
Char. 123: 1 → 0
Char. 129: 0 → 1
Char. 192: 1 → 0

**Node 76:**
Char. 107: 0 → 1

**Node 77:**
Char. 2: 0 → 1
Char. 6: 0 → 1
Char. 186: 0 → 1
Char. 198: 0 → 1
Char. 200: 0 → 1
Char. 220: 0 → 1
Char. 239: 1 → 0

**Node 78:**
Char. 48: 0 → 1
Char. 98: 1 → 0
Char. 113: 0 → 1
Char. 181: 0 → 1
Char. 206: 0 → 2

**Node 79:**
Char. 35: 0 → 1
Char. 102: 0 → 1
Char. 103: 0 → 1
Char. 184: 0 → 1
Char. 201: 0 → 1
Char. 210: 0 → 1
Char. 223: 0 → 1
Char. 229: 0 → 1

**Node 80:**
Char. 17: 0 → 1
Char. 42: 0 → 1
Char. 251: 0 → 1
Char. 264: 0 → 1

**Node 81:**
Char. 41: 0 → 1
Char. 61: 1 → 2
Char. 112: 1 → 0
Char. 138: 0 → 1
Char. 146: 1 → 0
Char. 155: 0 → 1
Char. 192: 1 → 2
Char. 224: 0 → 1
Char. 226: 0 → 1
Char. 227: 0 → 1
Char. 233: 0 → 1
Char. 267: 01 → 2

**Node 82:**
Char. 61: 1 → 3

Char. 90: 0 → 1
Char. 91: 0 → 1
Char. 109: 1 → 0
Char. 155: 0 → 1
Char. 209: 1 → 0

## ANALYSIS 38
## (ALL TAXA, IMPLIED WEIGHTING, K = 2.75)

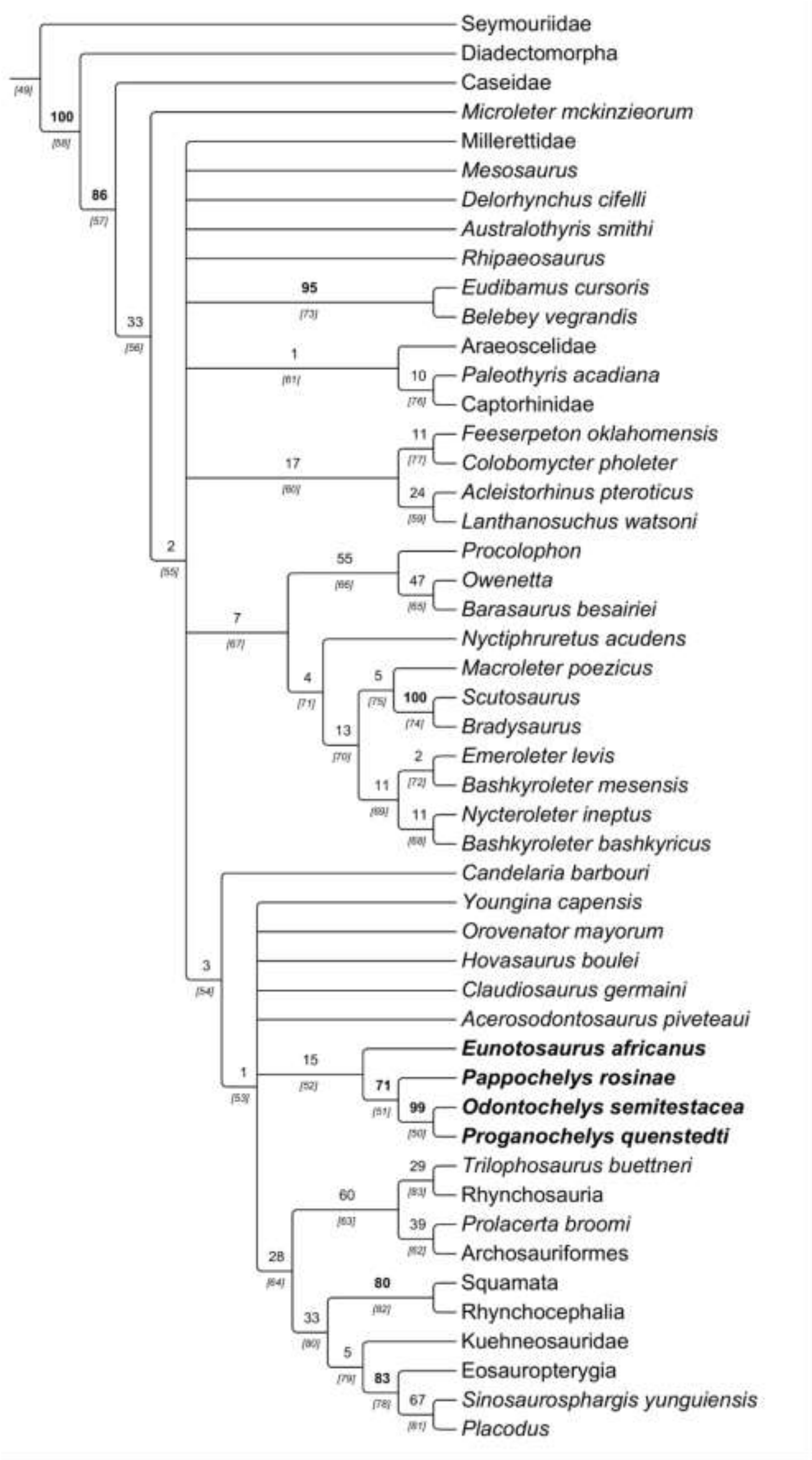

***Proganochelys quenstedti*:**
Char. 8: 0 → 1
Char. 11: 0 → 1
Char. 106: 0 → 1
Char. 108: 1 → 0
Char. 128: 0 → 1
Char. 175: 0 → 1
Char. 202: 1 → 0
Char. 207: 1 → 0
Char. 209: 1 → 0
Char. 244: 0 → 1
Char. 248: 0 → 1
Char. 250: 0 → 1
Char. 252: 1 → 0
Char. 262: 0 → 1

***Pappochelys rosinae*:**
Char. 0: 2 → 0
Char. 1: 0 → 1
Char. 5: 1 → 0
Char. 12: 0 → 1
Char. 41: 0 → 1
Char. 49: 0 → 1
Char. 75: 0 → 1
Char. 169: 1 → 0
Char. 260: 2 → 0

***Eunotosaurus africanus*:**
Char. 19: 0 → 1
Char. 59: 1 → 0
Char. 60: 1 → 0
Char. 72: 2 → 1
Char. 76: 0 → 1
Char. 97: 1 → 0
Char. 103: 0 → 1
Char. 153: 1 → 0
Char. 191: 1 → 0
Char. 192: 1 → 0
Char. 202: 1 → 0
Char. 211: 0 → 1
Char. 222: 1 → 0
Char. 237: 1 → 0
Char. 248: 0 → 1
Char. 249: 0 → 2
Char. 250: 0 → 1
Char. 263: 0 → 1
Char. 273: 0 → 1
Char. 274: 0 → 1
Char. 275: 0 → 1
Char. 276: 0 → 1
Char. 277: 0 → 1
Char. 278: 0 → 3

***Acerosodontosaurus piveteaui*:**
Char. 78: 1 → 0
Char. 81: 1 → 0
Char. 127: 1 → 0
Char. 128: 0 → 1
Char. 155: 0 → 1
Char. 206: 0 → 2
Char. 208: 0 → 1
Char. 278: 0 → 1

***Acleistorhinus pteroticus*:**
Char. 21: 0 → 1
Char. 146: 1 → 0

**Araeoscelidae:**
Char. 0: 0 → 1
Char. 28: 0 → 1
Char. 43: 0 → 1
Char. 57: 1 → 0
Char. 89: 0 → 1
Char. 106: 0 → 1
Char. 120: 0 → 1
Char. 193: 0 → 1
Char. 222: 0 → 1
Char. 224: 0 → 1
Char. 234: 0 → 1
Char. 266: 0 → 1
Char. 278: 3 → 1

**Archosauriformes:**
Char. 32: 0 → 1
Char. 94: 1 → 0
Char. 112: 1 → 0
Char. 152: 0 → 1
Char. 154: 01 → 2
Char. 166: 1 → 0
Char. 171: 0 → 1
Char. 185: 0 → 1
Char. 201: 0 → 1
Char. 204: 0 → 1
Char. 218: 0 → 3
Char. 224: 0 → 1
Char. 242: 0 → 1

***Australothyris smithi*:**
Char. 24: 0 → 1
Char. 34: 0 → 1
Char. 55: 1 → 0
Char. 57: 1 → 0
Char. 71: 1 → 0
Char. 79: 0 → 1
Char. 98: 1 → 0
Char. 100: 0 → 1
Char. 103: 0 → 1
Char. 110: 0 → 1
Char. 123: 1 → 0
Char. 129: 0 → 1
Char. 131: 0 → 1
Char. 132: 1 → 0
Char. 144: 1 → 0
Char. 147: 0 → 1
Char. 149: 1 → 0
Char. 150: 0 → 1
Char. 159: 1 → 0
Char. 192: 1 → 0

***Barasaurus besairiei*:**
Char. 33: 1 → 0

***Bashkyroleter bashkyricus*:**
Char. 275: 1 → 0

***Bashkyroleter mesensis*:**
Char. 12: 0 → 1
Char. 66: 1 → 0
Char. 169: 1 → 0

***Belebey vegrandis*:**
Char. 154: 1 → 0

***Bradysaurus* spp.:**
Char. 19: 0 → 1
Char. 25: 1 → 0
Char. 73: 0 → 1
Char. 79: 0 → 1
Char. 135: 1 → 0
Char. 249: 0 → 1

***Candelaria barbouri*:**
Char. 1: 0 → 1
Char. 8: 0 → 1
Char. 15: 0 → 1
Char. 33: 1 → 2
Char. 49: 0 → 1
Char. 55: 1 → 0
Char. 79: 0 → 2
Char. 88: 1 → 0
Char. 95: 0 → 1
Char. 126: 0 → 1
Char. 132: 1 → 0
Char. 154: 1 → 2
Char. 159: 1 → 0
Char. 169: 1 → 0
Char. 276: 0 → 1
Char. 277: 0 → 1

**Captorhinidae:**
Char. 3: 0 → 1
Char. 25: 1 → 0
Char. 26: 0 → 1
Char. 73: 0 → 1
Char. 75: 0 → 1
Char. 108: 1 → 0
Char. 180: 1 → 0
Char. 183: 1 → 0
Char. 201: 1 → 0
Char. 203: 1 → 2
Char. 216: 1 → 0

**Caseidae:**
Char. 38: 1 → 0
Char. 46: 1 → 0
Char. 50: 1 → 0
Char. 170: 0 → 1
Char. 194: 0 → 1
Char. 273: 0 → 1
Char. 274: 0 → 1
Char. 278: 3 → 2

***Claudiosaurus germaini*:**
Char. 24: 0 → 1
Char. 27: 0 → 1
Char. 34: 0 → 1
Char. 36: 0 → 1
Char. 56: 0 → 1
Char. 64: 0 → 1
Char. 70: 0 → 1
Char. 84: 1 → 0
Char. 105: 0 → 1
Char. 106: 0 → 1
Char. 117: 01 → 2
Char. 126: 0 → 1
Char. 127: 1 → 0
Char. 130: 0 → 1
Char. 131: 0 → 1
Char. 141: 1 → 0
Char. 144: 1 → 0
Char. 166: 1 → 0
Char. 182: 0 → 1
Char. 187: 1 → 0
Char. 190: 0 → 1
Char. 199: 0 → 1
Char. 201: 0 → 1
Char. 203: 1 → 2
Char. 204: 02 → 1
Char. 222: 1 → 0
Char. 234: 1 → 0
Char. 272: 2 → 1

***Colobomycter pholeter*:**
Char. 21: 0 → 1
Char. 25: 1 → 0
Char. 84: 1 → 0
Char. 154: 1 → 0
Char. 167: 0 → 1
Char. 267: 0 → 1

***Delorhynchus cifelli*:**
Char. 18: 0 → 1
Char. 20: 0 → 1
Char. 21: 0 → 1
Char. 23: 1 → 0
Char. 24: 0 → 1
Char. 26: 0 → 1
Char. 28: 0 → 1
Char. 33: 01 → 2
Char. 39: 0 → 1
Char. 52: 0 → 1
Char. 100: 0 → 1
Char. 111: 0 → 1
Char. 116: 1 → 0
Char. 119: 1 → 0
Char. 131: 0 → 1
Char. 147: 0 → 1
Char. 156: 1 → 0
Char. 167: 0 → 1
Char. 189: 0 → 1
Char. 191: 0 → 1
Char. 267: 0 → 1

**Diadectomorpha:**
Char. 0: 0 → 1
Char. 64: 0 → 1
Char. 70: 0 → 1
Char. 122: 0 → 1
Char. 123: 1 → 0
Char. 146: 1 → 0
Char. 275: 1 → 0
Char. 278: 3 → 0

***Emeroleter levis*:**
Char. 51: 1 → 0

**Eosauropterygia:**
Char. 166: 1 → 0
Char. 174: 0 → 1
Char. 178: 0 → 1
Char. 194: 0 → 2
Char. 272: 1 → 0

***Eudibamus cursoris*:**
Char. 154: 1 → 2

***Feeserpeton oklahomensis*:**
Char. 51: 0 → 1
Char. 157: 0 → 1
Char. 158: 0 → 1

***Hovasaurus boulei*:**
Char. 41: 0 → 1
Char. 55: 1 → 0
Char. 60: 1 → 0
Char. 72: 2 → 1
Char. 77: 0 → 2
Char. 78: 1 → 0
Char. 79: 0 → 1
Char. 93: 1 → 0
Char. 113: 0 → 1
Char. 138: 0 → 1
Char. 141: 1 → 0
Char. 146: 1 → 0
Char. 206: 0 → 2
Char. 215: 0 → 1
Char. 219: 1 → 0
Char. 224: 0 → 1
Char. 278: 0 → 3

**Kuehneosauridae:**
Char. 7: 0 → 1
Char. 24: 0 → 1
Char. 27: 0 → 1
Char. 43: 1 → 0
Char. 79: 0 → 2
Char. 107: 1 → 0
Char. 140: 1 → 0
Char. 147: 1 → 0
Char. 148: 1 → 0
Char. 245: 0 → 1
Char. 278: 0 → 3

***Lanthanosuchus watsoni*:**
Char. 25: 1 → 0
Char. 51: 0 → 1
Char. 86: 0 → 1
Char. 98: 1 → 0
Char. 138: 0 → 1
Char. 144: 1 → 0
Char. 154: 1 → 2

***Macroleter poezicus*:**
Char. 9: 0 → 1
Char. 26: 0 → 1
Char. 66: 1 → 2
Char. 126: 0 → 1
Char. 134: 0 → 2
Char. 140: 0 → 1
Char. 146: 1 → 0
Char. 169: 1 → 0

***Mesosaurus* spp.:**
Char. 0: 0 → 1
Char. 2: 0 → 1
Char. 6: 0 → 1
Char. 8: 0 → 1
Char. 9: 0 → 1
Char. 13: 0 → 1
Char. 19: 0 → 1
Char. 26: 0 → 1
Char. 29: 1 → 0
Char. 38: 1 → 0
Char. 41: 0 → 1
Char. 50: 1 → 0
Char. 67: 0 → 1
Char. 84: 1 → 0
Char. 94: 0 → 1
Char. 107: 0 → 1
Char. 109: 0 → 1
Char. 111: 0 → 1
Char. 115: 0 → 1
Char. 146: 1 → 0
Char. 149: 1 → 0
Char. 159: 1 → 0
Char. 164: 0 → 1
Char. 166: 1 → 0
Char. 167: 0 → 1
Char. 176: 0 → 1
Char. 183: 1 → 0
Char. 184: 0 → 1
Char. 192: 1 → 0
Char. 199: 0 → 1
Char. 202: 0 → 1
Char. 206: 0 → 1
Char. 209: 0 → 1
Char. 217: 0 → 1
Char. 219: 0 → 1
Char. 220: 0 → 1
Char. 231: 0 → 1
Char. 260: 2 → 0
Char. 272: 2 → 0
Char. 278: 3 → 0

***Microleter mckinzieorum*:**
Char. 0: 0 → 1
Char. 18: 0 → 1
Char. 39: 0 → 1
Char. 57: 1 → 0
Char. 70: 0 → 1
Char. 94: 0 → 1
Char. 106: 0 → 1
Char. 110: 0 → 1
Char. 278: 3 → 1

**Millerettidae:**
Char. 23: 1 → 0
Char. 24: 0 → 1
Char. 25: 1 → 0
Char. 44: 1 → 0
Char. 56: 0 → 1
Char. 57: 1 → 0
Char. 66: 0 → 2
Char. 78: 1 → 0
Char. 80: 1 → 0
Char. 84: 1 → 2
Char. 88: 1 → 0
Char. 96: 1 → 0
Char. 121: 0 → 1
Char. 124: 0 → 1
Char. 127: 0 → 1
Char. 135: 0 → 1
Char. 145: 0 → 1
Char. 159: 1 → 0
Char. 166: 1 → 0
Char. 180: 1 → 0
Char. 192: 1 → 0
Char. 202: 0 → 1
Char. 211: 0 → 1
Char. 230: 0 → 1
Char. 234: 0 → 1
Char. 248: 0 → 1
Char. 252: 0 → 1
Char. 253: 0 → 1

***Nycteroleter ineptus*:**
Char. 66: 1 → 0

***Nyctiphruretus acudens*:**
Char. 21: 0 → 1
Char. 41: 0 → 1
Char. 66: 1 → 2
Char. 81: 1 → 0
Char. 84: 1 → 2
Char. 94: 0 → 1
Char. 166: 1 → 0
Char. 224: 0 → 1
Char. 266: 0 → 1
Char. 272: 2 → 1
Char. 276: 0 → 1

***Orovenator mayorum*:**
Char. 8: 0 → 1
Char. 23: 1 → 0
Char. 24: 0 → 1
Char. 33: 1 → 0
Char. 36: 0 → 1
Char. 50: 0 → 1
Char. 92: 1 → 0
Char. 94: 1 → 0
Char. 135: 1 → 0
Char. 159: 1 → 0
Char. 160: 0 → 1
Char. 165: 0 → 1
Char. 278: 0 → 1

***Owenetta* spp.:**
Char. 142: 1 → 0
Char. 169: 1 → 0

***Paleothyris acadiana*:**
Char. 23: 1 → 0
Char. 66: 1 → 2
Char. 102: 0 → 1
Char. 146: 1 → 0

***Placodus* spp.:**
Char. 0: 1 → 2
Char. 9: 0 → 1
Char. 12: 0 → 1
Char. 13: 0 → 1
Char. 19: 0 → 1
Char. 31: 0 → 1
Char. 46: 1 → 0
Char. 57: 0 → 1
Char. 78: 1 → 0
Char. 93: 1 → 0
Char. 102: 1 → 2
Char. 109: 1 → 0
Char. 140: 1 → 0
Char. 155: 0 → 1
Char. 163: 1 → 0
Char. 164: 0 → 1

***Procolophon* spp.:**
Char. 41: 0 → 1
Char. 69: 0 → 1
Char. 79: 0 → 1
Char. 86: 0 → 1
Char. 101: 0 → 1
Char. 141: 0 → 1
Char. 149: 1 → 0
Char. 203: 1 → 2
Char. 215: 0 → 1
Char. 230: 0 → 1
Char. 235: 1 → 2
Char. 237: 0 → 1
Char. 238: 0 → 1
Char. 272: 2 → 1
Char. 278: 3 → 0

***Prolacerta broomi*:**
Char. 58: 1 → 0
Char. 66: 1 → 0
Char. 67: 1 → 0
Char. 80: 1 → 0
Char. 139: 1 → 0
Char. 147: 1 → 0
Char. 192: 1 → 0
Char. 203: 1 → 2
Char. 206: 0 → 12

***Rhipaeosaurus* spp.:**
Char. 172: 0 → 1
Char. 183: 1 → 2
Char. 194: 0 → 1
Char. 201: 1 → 0
Char. 211: 0 → 1
Char. 215: 0 → 1
Char. 226: 0 → 1
Char. 235: 1 → 0
Char. 252: 0 → 1
Char. 277: 0 → 1

**Rhynchocephalia:**
Char. 0: 1 → 2
Char. 23: 1 → 0
Char. 24: 0 → 1
Char. 77: 0 → 1
Char. 88: 1 → 0
Char. 94: 1 → 0
Char. 139: 1 → 0
Char. 167: 1 → 0
Char. 205: 1 → 0

**Rhynchosauria:**
Char. 0: 1 → 0
Char. 7: 0 → 1
Char. 9: 0 → 1
Char. 26: 0 → 1
Char. 44: 1 → 0
Char. 68: 0 → 1
Char. 99: 1 → 0
Char. 150: 1 → 0
Char. 160: 0 → 1
Char. 161: 0 → 1
Char. 171: 0 → 2
Char. 223: 0 → 1
Char. 224: 0 → 1
Char. 241: 0 → 1

***Scutosaurus* spp.:**
Char. 175: 0 → 1
Char. 190: 0 → 1
Char. 218: 0 → 1
Char. 243: 0 → 2
Char. 244: 0 → 1
Char. 251: 0 → 1

***Sinosaurosphargis yunguiensis*:**
Char. 8: 0 → 1
Char. 30: 0 → 1
Char. 53: 0 → 1
Char. 89: 1 → 0
Char. 127: 1 → 0
Char. 150: 1 → 0
Char. 154: 0 → 2
Char. 167: 1 → 0
Char. 253: 0 → 1
Char. 255: 0 → 1

**Squamata:**
Char. 45: 0 → 1
Char. 79: 0 → 2
Char. 80: 1 → 0
Char. 92: 1 → 0
Char. 109: 1 → 0
Char. 160: 0 → 1
Char. 200: 0 → 1
Char. 245: 0 → 1

***Trilophosaurus buettneri*:**
Char. 5: 1 → 0
Char. 11: 0 → 1
Char. 55: 1 → 0
Char. 93: 1 → 0
Char. 104: 0 → 1
Char. 106: 0 → 1
Char. 113: 0 → 1
Char. 122: 0 → 1
Char. 136: 1 → 0
Char. 144: 1 → 0
Char. 157: 0 → 1
Char. 159: 1 → 0
Char. 177: 0 → 12
Char. 194: 0 → 1
Char. 203: 1 → 2
Char. 207: 1 → 0
Char. 208: 1 → 0
Char. 272: 1 → 0

***Youngina capensis*:**
Char. 5: 1 → 0
Char. 23: 1 → 0
Char. 25: 0 → 1
Char. 27: 0 → 1
Char. 38: 1 → 0
Char. 44: 1 → 0
Char. 56: 0 → 1
Char. 75: 0 → 1
Char. 84: 1 → 0
Char. 92: 1 → 0
Char. 94: 1 → 0
Char. 134: 0 → 1
Char. 163: 1 → 0
Char. 170: 0 → 1
Char. 211: 0 → 1
Char. 215: 0 → 1
Char. 224: 0 → 1
Char. 239: 0 → 1

**Node 50:**
Char. 46: 1 → 0
Char. 88: 1 → 0
Char. 89: 1 → 0
Char. 93: 1 → 0
Char. 176: 0 → 1
Char. 195: 0 → 2
Char. 198: 0 → 1
Char. 246: 1 → 2
Char. 259: 0 → 1
Char. 265: 1 → 0
Char. 270: 0 → 1

**Node 51:**
Char. 65: 0 → 1
Char. 131: 0 → 1
Char. 184: 0 → 1
Char. 205: 0 → 1
Char. 210: 0 → 1
Char. 219: 1 → 0
Char. 241: 0 → 1
Char. 246: 0 → 1
Char. 254: 0 → 1
Char. 255: 0 → 1
Char. 256: 0 → 1
Char. 268: 0 → 1
Char. 269: 0 → 1

**Node 52:**
Char. 0: 1 → 2
Char. 15: 0 → 1
Char. 33: 1 → 2
Char. 44: 1 → 0
Char. 62: 1 → 0
Char. 64: 0 → 1
Char. 84: 1 → 2
Char. 130: 0 → 1
Char. 134: 0 → 2
Char. 147: 0 → 1
Char. 152: 0 → 1
Char. 155: 0 → 1

Char. 158: 0 → 1
Char. 161: 0 → 1
Char. 174: 0 → 1
Char. 181: 0 → 1
Char. 203: 1 → 2
Char. 247: 0 → 1
Char. 251: 0 → 1
Char. 252: 0 → 1
Char. 253: 0 → 2

**Node 53:**
Char. 0: 0 → 1
Char. 59: 0 → 1
Char. 60: 0 → 1
Char. 62: 0 → 1
Char. 72: 1 → 2
Char. 92: 0 → 1
Char. 265: 0 → 1
Char. 275: 1 → 0
Char. 278: 3 → 0

**Node 54:**
Char. 20: 0 → 1
Char. 25: 1 → 0
Char. 29: 1 → 0
Char. 50: 1 → 0
Char. 57: 1 → 0
Char. 67: 0 → 1
Char. 89: 0 → 1
Char. 94: 0 → 1
Char. 127: 0 → 1

**Node 55:**
Char. 23: 0 → 1
Char. 132: 0 → 1
Char. 166: 0 → 1
Char. 276: 1 → 0

**Node 56:**
Char. 29: 0 → 1
Char. 84: 0 → 1
Char. 88: 0 → 1
Char. 169: 0 → 1

**Node 57:**
Char. 72: 0 → 1
Char. 81: 0 → 1
Char. 93: 0 → 1
Char. 97: 0 → 1
Char. 104: 1 → 0
Char. 144: 0 → 1
Char. 173: 0 → 1
Char. 183: 0 → 1

**Node 59:**
Char. 95: 0 → 1
Char. 113: 0 → 1
Char. 114: 0 → 1

**Node 60:**
Char. 20: 0 → 1
Char. 23: 1 → 0
Char. 47: 0 → 1
Char. 79: 0 → 1
Char. 110: 0 → 1
Char. 131: 0 → 1
Char. 137: 0 → 1
Char. 140: 0 → 2
Char. 147: 0 → 1

**Node 61:**
Char. 29: 1 → 0
Char. 59: 0 → 1
Char. 60: 0 → 1
Char. 67: 0 → 1
Char. 72: 1 → 2
Char. 84: 1 → 0
Char. 111: 0 → 1
Char. 116: 1 → 0
Char. 154: 1 → 0
Char. 159: 1 → 0
Char. 166: 1 → 0
Char. 169: 1 → 0
Char. 170: 0 → 1
Char. 197: 0 → 1
Char. 221: 1 → 0

**Node 62:**
Char. 19: 0 → 1
Char. 92: 1 → 0

**Node 63:**
Char. 4: 0 → 1
Char. 15: 0 → 1
Char. 29: 0 → 1
Char. 213: 0 → 1
Char. 226: 0 → 2
Char. 228: 0 → 1
Char. 275: 0 → 1

**Node 64:**
Char. 58: 0 → 1
Char. 61: 0 → 1
Char. 66: 0 → 1
Char. 69: 0 → 1
Char. 107: 0 → 1
Char. 126: 0 → 1
Char. 131: 0 → 1
Char. 134: 0 → 1
Char. 140: 0 → 1
Char. 147: 0 → 1
Char. 150: 0 → 1
Char. 167: 0 → 1
Char. 176: 0 → 1
Char. 190: 0 → 1
Char. 205: 0 → 1
Char. 208: 0 → 1
Char. 230: 0 → 1
Char. 239: 0 → 1
Char. 260: 2 → 1

**Node 65:**
Char. 73: 0 → 1
Char. 205: 0 → 1
Char. 273: 0 → 1
Char. 276: 0 → 1

**Node 66:**
Char. 18: 0 → 1
Char. 25: 1 → 0
Char. 37: 0 → 1
Char. 103: 0 → 1
Char. 106: 0 → 1
Char. 107: 0 → 1
Char. 126: 0 → 1
Char. 150: 0 → 1

**Node 67:**
Char. 20: 0 → 1
Char. 38: 1 → 2
Char. 39: 0 → 1
Char. 44: 1 → 0
Char. 49: 0 → 1
Char. 66: 0 → 1
Char. 80: 1 → 0
Char. 95: 0 → 1
Char. 135: 0 → 1
Char. 137: 0 → 1
Char. 147: 0 → 1
Char. 157: 0 → 1
Char. 158: 0 → 1
Char. 183: 1 → 2
Char. 186: 0 → 1
Char. 187: 0 → 1
Char. 194: 0 → 1
Char. 197: 0 → 1
Char. 201: 1 → 0
Char. 211: 0 → 1
Char. 252: 0 → 1

**Node 69:**
Char. 133: 0 → 1

**Node 70:**
Char. 100: 0 → 1
Char. 113: 0 → 1

**Node 71:**
Char. 23: 1 → 0
Char. 70: 0 → 1
Char. 118: 0 → 1
Char. 125: 0 → 1

**Node 73:**
Char. 38: 1 → 2
Char. 39: 0 → 1
Char. 50: 1 → 0
Char. 58: 0 → 1
Char. 59: 0 → 1
Char. 60: 0 → 1
Char. 72: 1 → 2
Char. 88: 1 → 0
Char. 95: 0 → 1
Char. 104: 0 → 1
Char. 105: 0 → 1
Char. 106: 0 → 2
Char. 107: 0 → 1
Char. 109: 0 → 1
Char. 110: 0 → 1
Char. 146: 1 → 0
Char. 155: 0 → 2
Char. 183: 1 → 2

**Node 74:**
Char. 23: 0 → 1
Char. 33: 1 → 0
Char. 38: 2 → 1
Char. 39: 1 → 0
Char. 42: 0 → 1
Char. 43: 0 → 1
Char. 46: 1 → 0
Char. 49: 1 → 0
Char. 71: 1 → 0
Char. 83: 0 → 2
Char. 101: 0 → 1
Char. 103: 0 → 1
Char. 106: 0 → 1
Char. 161: 0 → 1
Char. 163: 1 → 0
Char. 172: 0 → 2
Char. 174: 0 → 1
Char. 188: 0 → 1
Char. 203: 1 → 2
Char. 212: 0 → 1
Char. 216: 1 → 0
Char. 221: 1 → 0
Char. 236: 0 → 1
Char. 238: 0 → 2
Char. 241: 0 → 1
Char. 242: 0 → 1
Char. 273: 0 → 1
Char. 274: 0 → 1
Char. 275: 1 → 0

**Node 75:**
Char. 52: 0 → 1
Char. 75: 0 → 1
Char. 84: 1 → 0
Char. 121: 0 → 1
Char. 180: 1 → 0
Char. 230: 0 → 1

**Node 76:**
Char. 44: 1 → 0
Char. 66: 0 → 1
Char. 78: 1 → 0
Char. 88: 1 → 0
Char. 93: 1 → 0
Char. 123: 1 → 0
Char. 129: 0 → 1
Char. 192: 1 → 0

**Node 77:**
Char. 107: 0 → 1

**Node 78:**
Char. 2: 0 → 1
Char. 6: 0 → 1
Char. 186: 0 → 1
Char. 198: 0 → 1
Char. 200: 0 → 1
Char. 220: 0 → 1
Char. 239: 1 → 0

**Node 79:**
Char. 98: 1 → 0
Char. 113: 0 → 1
Char. 181: 0 → 1
Char. 206: 0 → 2

**Node 80:**
Char. 35: 0 → 1
Char. 102: 0 → 1
Char. 103: 0 → 1
Char. 184: 0 → 1
Char. 201: 0 → 1
Char. 210: 0 → 1
Char. 223: 0 → 1
Char. 229: 0 → 1

**Node 81:**
Char. 17: 0 → 1
Char. 42: 0 → 1
Char. 251: 0 → 1
Char. 264: 0 → 1

**Node 82:**
Char. 41: 0 → 1
Char. 61: 1 → 2
Char. 112: 1 → 0
Char. 138: 0 → 1
Char. 146: 1 → 0
Char. 155: 0 → 1
Char. 192: 1 → 2
Char. 224: 0 → 1
Char. 226: 0 → 1
Char. 227: 0 → 1
Char. 233: 0 → 1
Char. 267: 01 → 2

**Node 83:**
Char. 61: 1 → 3
Char. 90: 0 → 1
Char. 91: 0 → 1
Char. 109: 1 → 0
Char. 155: 0 → 1
Char. 209: 1 → 0

**ANALYSIS 39**
**(ALL TAXA, IMPLIED WEIGHTING, K = 2.875)**

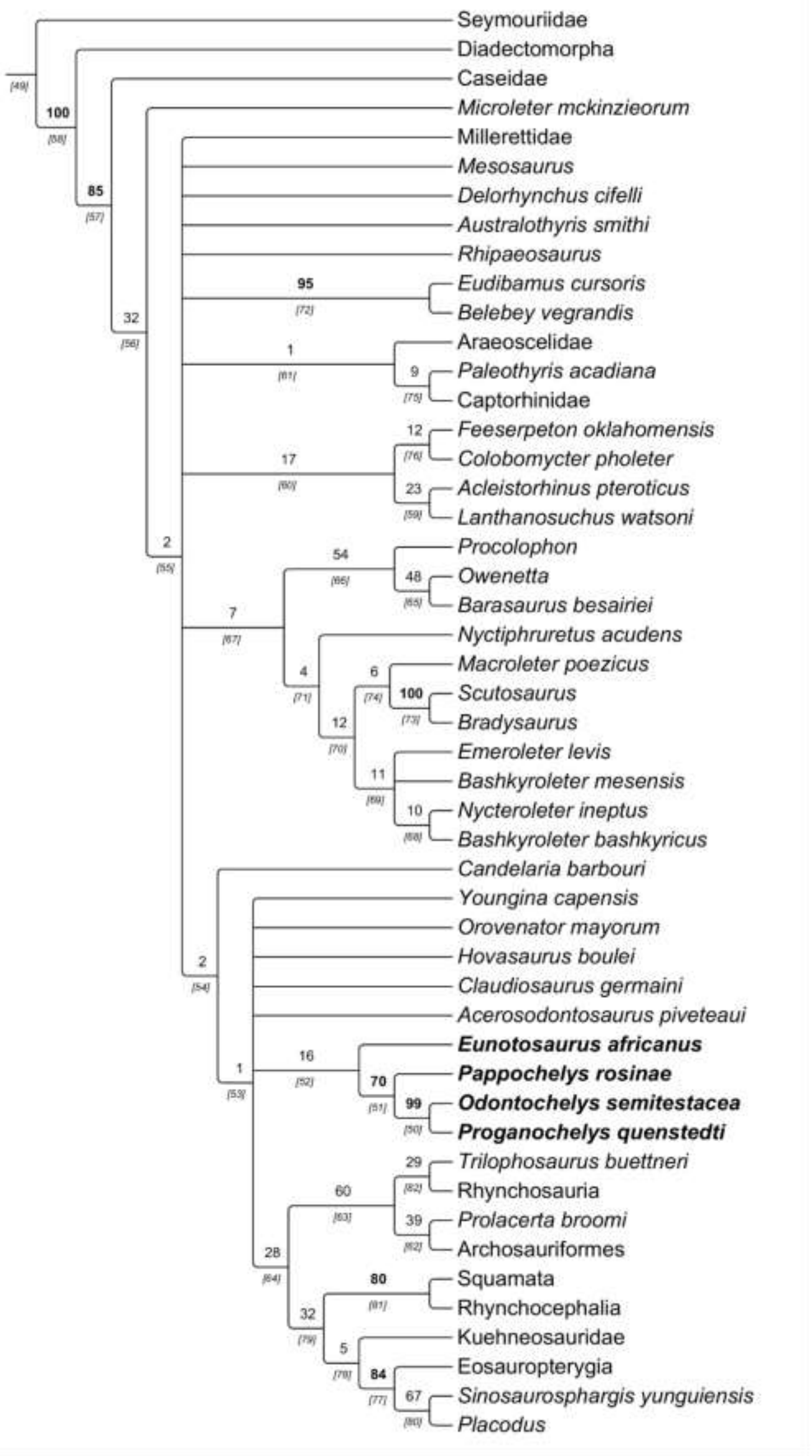

***Proganochelys quenstedti*:**
Char. 8: 0 → 1
Char. 11: 0 → 1
Char. 106: 0 → 1
Char. 108: 1 → 0
Char. 128: 0 → 1
Char. 175: 0 → 1
Char. 202: 1 → 0
Char. 207: 1 → 0
Char. 209: 1 → 0
Char. 244: 0 → 1
Char. 248: 0 → 1
Char. 250: 0 → 1
Char. 252: 1 → 0
Char. 262: 0 → 1

***Pappochelys rosinae*:**
Char. 0: 2 → 0
Char. 1: 0 → 1
Char. 5: 1 → 0
Char. 12: 0 → 1
Char. 41: 0 → 1
Char. 49: 0 → 1
Char. 75: 0 → 1
Char. 169: 1 → 0
Char. 260: 2 → 0

***Eunotosaurus africanus*:**
Char. 19: 0 → 1
Char. 59: 1 → 0
Char. 60: 1 → 0
Char. 72: 2 → 1
Char. 76: 0 → 1
Char. 97: 1 → 0
Char. 103: 0 → 1
Char. 153: 1 → 0
Char. 191: 1 → 0
Char. 192: 1 → 0
Char. 202: 1 → 0
Char. 211: 0 → 1
Char. 222: 1 → 0
Char. 237: 1 → 0
Char. 248: 0 → 1
Char. 249: 0 → 2
Char. 250: 0 → 1
Char. 263: 0 → 1
Char. 273: 0 → 1
Char. 274: 0 → 1
Char. 275: 0 → 1
Char. 276: 0 → 1
Char. 277: 0 → 1
Char. 278: 0 → 3

***Acerosodontosaurus piveteaui*:**
Char. 78: 1 → 0
Char. 81: 1 → 0
Char. 127: 1 → 0
Char. 128: 0 → 1
Char. 155: 0 → 1
Char. 206: 0 → 2
Char. 208: 0 → 1
Char. 278: 0 → 1

***Acleistorhinus pteroticus*:**
Char. 21: 0 → 1
Char. 146: 1 → 0

**Araeoscelidae:**
Char. 0: 0 → 1
Char. 28: 0 → 1
Char. 43: 0 → 1
Char. 57: 1 → 0
Char. 89: 0 → 1
Char. 106: 0 → 1
Char. 120: 0 → 1
Char. 193: 0 → 1
Char. 222: 0 → 1
Char. 224: 0 → 1
Char. 234: 0 → 1
Char. 266: 0 → 1
Char. 278: 3 → 1

**Archosauriformes:**
Char. 32: 0 → 1
Char. 94: 1 → 0
Char. 112: 1 → 0
Char. 152: 0 → 1
Char. 154: 01 → 2
Char. 166: 1 → 0
Char. 171: 0 → 1
Char. 185: 0 → 1
Char. 201: 0 → 1
Char. 204: 0 → 1
Char. 218: 0 → 3
Char. 224: 0 → 1
Char. 242: 0 → 1

***Australothyris smithi*:**
Char. 24: 0 → 1
Char. 34: 0 → 1
Char. 55: 1 → 0
Char. 57: 1 → 0
Char. 71: 1 → 0
Char. 79: 0 → 1
Char. 98: 1 → 0
Char. 100: 0 → 1
Char. 103: 0 → 1
Char. 110: 0 → 1
Char. 123: 1 → 0
Char. 129: 0 → 1
Char. 131: 0 → 1
Char. 132: 1 → 0
Char. 144: 1 → 0
Char. 147: 0 → 1
Char. 149: 1 → 0
Char. 150: 0 → 1
Char. 159: 1 → 0
Char. 192: 1 → 0

***Barasaurus besairiei*:**
Char. 33: 1 → 0

***Bashkyroleter bashkyricus*:**
Char. 275: 1 → 0

***Bashkyroleter mesensis*:**
Char. 12: 0 → 1
Char. 66: 1 → 0
Char. 169: 1 → 0

***Belebey vegrandis*:**
Char. 154: 1 → 0

***Bradysaurus* spp.:**
Char. 19: 0 → 1
Char. 25: 1 → 0
Char. 73: 0 → 1
Char. 79: 0 → 1
Char. 135: 1 → 0
Char. 249: 0 → 1

***Candelaria barbouri*:**
Char. 1: 0 → 1
Char. 8: 0 → 1
Char. 15: 0 → 1
Char. 33: 1 → 2
Char. 49: 0 → 1
Char. 55: 1 → 0
Char. 79: 0 → 2
Char. 88: 1 → 0
Char. 95: 0 → 1
Char. 126: 0 → 1
Char. 132: 1 → 0
Char. 154: 1 → 2
Char. 159: 1 → 0
Char. 169: 1 → 0
Char. 276: 0 → 1
Char. 277: 0 → 1

**Captorhinidae:**
Char. 3: 0 → 1
Char. 25: 1 → 0
Char. 26: 0 → 1
Char. 73: 0 → 1
Char. 75: 0 → 1
Char. 108: 1 → 0
Char. 180: 1 → 0
Char. 183: 1 → 0
Char. 201: 1 → 0
Char. 203: 1 → 2
Char. 216: 1 → 0

**Caseidae:**
Char. 38: 1 → 0
Char. 46: 1 → 0
Char. 50: 1 → 0
Char. 170: 0 → 1
Char. 194: 0 → 1
Char. 273: 0 → 1
Char. 274: 0 → 1
Char. 278: 3 → 2

***Claudiosaurus germaini*:**
Char. 24: 0 → 1
Char. 27: 0 → 1
Char. 34: 0 → 1
Char. 36: 0 → 1
Char. 56: 0 → 1
Char. 64: 0 → 1
Char. 70: 0 → 1
Char. 84: 1 → 0
Char. 105: 0 → 1
Char. 106: 0 → 1
Char. 117: 01 → 2
Char. 126: 0 → 1
Char. 127: 1 → 0
Char. 130: 0 → 1
Char. 131: 0 → 1
Char. 141: 1 → 0
Char. 144: 1 → 0
Char. 166: 1 → 0
Char. 182: 0 → 1
Char. 187: 1 → 0
Char. 190: 0 → 1
Char. 199: 0 → 1
Char. 201: 0 → 1
Char. 203: 1 → 2
Char. 204: 02 → 1
Char. 222: 1 → 0
Char. 234: 1 → 0
Char. 272: 2 → 1

***Colobomycter pholeter*:**
Char. 21: 0 → 1
Char. 25: 1 → 0
Char. 84: 1 → 0
Char. 154: 1 → 0
Char. 167: 0 → 1
Char. 267: 0 → 1

***Delorhynchus cifelli*:**
Char. 18: 0 → 1
Char. 20: 0 → 1
Char. 21: 0 → 1
Char. 23: 1 → 0
Char. 24: 0 → 1
Char. 26: 0 → 1
Char. 28: 0 → 1
Char. 33: 01 → 2
Char. 39: 0 → 1
Char. 52: 0 → 1
Char. 100: 0 → 1
Char. 111: 0 → 1
Char. 116: 1 → 0
Char. 119: 1 → 0
Char. 131: 0 → 1
Char. 147: 0 → 1
Char. 156: 1 → 0
Char. 167: 0 → 1
Char. 189: 0 → 1
Char. 191: 0 → 1
Char. 267: 0 → 1

**Diadectomorpha:**
Char. 0: 0 → 1
Char. 64: 0 → 1
Char. 70: 0 → 1
Char. 122: 0 → 1
Char. 123: 1 → 0
Char. 146: 1 → 0
Char. 275: 1 → 0
Char. 278: 3 → 0

***Emeroleter levis*:**
Char. 51: 1 → 0

**Eosauropterygia:**
Char. 166: 1 → 0
Char. 174: 0 → 1
Char. 178: 0 → 1
Char. 194: 0 → 2
Char. 272: 1 → 0

***Eudibamus cursoris*:**
Char. 154: 1 → 2

***Feeserpeton oklahomensis*:**
Char. 51: 0 → 1
Char. 157: 0 → 1
Char. 158: 0 → 1

***Hovasaurus boulei*:**
Char. 41: 0 → 1
Char. 55: 1 → 0
Char. 60: 1 → 0
Char. 72: 2 → 1
Char. 77: 0 → 2
Char. 78: 1 → 0
Char. 79: 0 → 1
Char. 93: 1 → 0
Char. 113: 0 → 1
Char. 138: 0 → 1
Char. 141: 1 → 0
Char. 146: 1 → 0
Char. 206: 0 → 2
Char. 215: 0 → 1
Char. 219: 1 → 0
Char. 224: 0 → 1
Char. 278: 0 → 3

**Kuehneosauridae:**
Char. 7: 0 → 1
Char. 24: 0 → 1
Char. 27: 0 → 1
Char. 43: 1 → 0
Char. 79: 0 → 2
Char. 107: 1 → 0
Char. 140: 1 → 0
Char. 147: 1 → 0
Char. 148: 1 → 0
Char. 245: 0 → 1
Char. 278: 0 → 3

***Lanthanosuchus watsoni*:**
Char. 25: 1 → 0
Char. 51: 0 → 1
Char. 86: 0 → 1
Char. 98: 1 → 0
Char. 138: 0 → 1
Char. 144: 1 → 0
Char. 154: 1 → 2

***Macroleter poezicus*:**
Char. 9: 0 → 1
Char. 26: 0 → 1
Char. 66: 1 → 2
Char. 126: 0 → 1
Char. 134: 0 → 2
Char. 140: 0 → 1
Char. 146: 1 → 0
Char. 169: 1 → 0

***Mesosaurus* spp.:**
Char. 0: 0 → 1
Char. 2: 0 → 1
Char. 6: 0 → 1
Char. 8: 0 → 1
Char. 9: 0 → 1
Char. 13: 0 → 1
Char. 19: 0 → 1
Char. 26: 0 → 1
Char. 29: 1 → 0
Char. 38: 1 → 0
Char. 41: 0 → 1
Char. 50: 1 → 0
Char. 67: 0 → 1
Char. 84: 1 → 0
Char. 94: 0 → 1
Char. 107: 0 → 1
Char. 109: 0 → 1
Char. 111: 0 → 1
Char. 115: 0 → 1
Char. 146: 1 → 0
Char. 149: 1 → 0
Char. 159: 1 → 0
Char. 164: 0 → 1
Char. 166: 1 → 0
Char. 167: 0 → 1
Char. 176: 0 → 1
Char. 183: 1 → 0
Char. 184: 0 → 1
Char. 192: 1 → 0
Char. 199: 0 → 1
Char. 202: 0 → 1
Char. 206: 0 → 1
Char. 209: 0 → 1
Char. 217: 0 → 1
Char. 219: 0 → 1
Char. 220: 0 → 1
Char. 231: 0 → 1
Char. 260: 2 → 0
Char. 272: 2 → 0
Char. 278: 3 → 0

***Microleter mckinzieorum*:**
Char. 0: 0 → 1
Char. 18: 0 → 1
Char. 39: 0 → 1
Char. 57: 1 → 0
Char. 70: 0 → 1
Char. 94: 0 → 1
Char. 106: 0 → 1
Char. 110: 0 → 1
Char. 278: 3 → 1

**Millerettidae:**
Char. 23: 1 → 0
Char. 24: 0 → 1
Char. 25: 1 → 0
Char. 44: 1 → 0
Char. 56: 0 → 1
Char. 57: 1 → 0
Char. 66: 0 → 2
Char. 78: 1 → 0
Char. 80: 1 → 0
Char. 84: 1 → 2
Char. 88: 1 → 0
Char. 96: 1 → 0
Char. 121: 0 → 1
Char. 124: 0 → 1
Char. 127: 0 → 1
Char. 135: 0 → 1
Char. 145: 0 → 1
Char. 159: 1 → 0
Char. 166: 1 → 0
Char. 180: 1 → 0
Char. 192: 1 → 0
Char. 202: 0 → 1
Char. 211: 0 → 1
Char. 230: 0 → 1
Char. 234: 0 → 1
Char. 248: 0 → 1
Char. 252: 0 → 1
Char. 253: 0 → 1

***Nycteroleter ineptus*:**
Char. 66: 1 → 0
Char. 278: 0 → 3

***Nyctiphruretus acudens*:**
Char. 21: 0 → 1
Char. 41: 0 → 1
Char. 66: 1 → 2
Char. 81: 1 → 0
Char. 84: 1 → 2
Char. 94: 0 → 1
Char. 166: 1 → 0
Char. 224: 0 → 1
Char. 266: 0 → 1
Char. 272: 2 → 1
Char. 276: 0 → 1

***Orovenator mayorum*:**
Char. 8: 0 → 1
Char. 23: 1 → 0
Char. 24: 0 → 1
Char. 33: 1 → 0
Char. 36: 0 → 1
Char. 50: 0 → 1
Char. 92: 1 → 0
Char. 94: 1 → 0
Char. 135: 1 → 0
Char. 159: 1 → 0
Char. 160: 0 → 1
Char. 165: 0 → 1
Char. 278: 0 → 1

***Owenetta* spp.:**
Char. 142: 1 → 0
Char. 169: 1 → 0

***Paleothyris acadiana*:**
Char. 23: 1 → 0
Char. 66: 1 → 2
Char. 102: 0 → 1
Char. 146: 1 → 0

***Placodus* spp.:**
Char. 0: 1 → 2
Char. 9: 0 → 1
Char. 12: 0 → 1
Char. 13: 0 → 1
Char. 19: 0 → 1
Char. 31: 0 → 1
Char. 46: 1 → 0
Char. 57: 0 → 1
Char. 78: 1 → 0
Char. 93: 1 → 0
Char. 102: 1 → 2
Char. 109: 1 → 0
Char. 140: 1 → 0
Char. 155: 0 → 1
Char. 163: 1 → 0
Char. 164: 0 → 1

***Procolophon* spp.:**
Char. 41: 0 → 1
Char. 69: 0 → 1
Char. 79: 0 → 1
Char. 86: 0 → 1
Char. 101: 0 → 1
Char. 141: 0 → 1
Char. 149: 1 → 0
Char. 203: 1 → 2
Char. 215: 0 → 1
Char. 230: 0 → 1
Char. 235: 1 → 2
Char. 237: 0 → 1
Char. 238: 0 → 1
Char. 272: 2 → 1
Char. 278: 3 → 0

***Prolacerta broomi*:**
Char. 58: 1 → 0
Char. 66: 1 → 0
Char. 67: 1 → 0
Char. 80: 1 → 0
Char. 139: 1 → 0
Char. 147: 1 → 0
Char. 192: 1 → 0
Char. 203: 1 → 2
Char. 206: 0 → 12

***Rhipaeosaurus* spp.:**
Char. 172: 0 → 1
Char. 183: 1 → 2
Char. 194: 0 → 1
Char. 201: 1 → 0
Char. 211: 0 → 1
Char. 215: 0 → 1
Char. 226: 0 → 1
Char. 235: 1 → 0
Char. 252: 0 → 1
Char. 277: 0 → 1

**Rhynchocephalia:**
Char. 0: 1 → 2
Char. 23: 1 → 0
Char. 24: 0 → 1
Char. 77: 0 → 1
Char. 88: 1 → 0
Char. 94: 1 → 0
Char. 139: 1 → 0
Char. 167: 1 → 0
Char. 205: 1 → 0

**Rhynchosauria:**
Char. 0: 1 → 0
Char. 7: 0 → 1
Char. 9: 0 → 1
Char. 26: 0 → 1
Char. 44: 1 → 0
Char. 68: 0 → 1
Char. 99: 1 → 0
Char. 150: 1 → 0
Char. 160: 0 → 1
Char. 161: 0 → 1
Char. 171: 0 → 2
Char. 223: 0 → 1
Char. 224: 0 → 1
Char. 241: 0 → 1

***Scutosaurus* spp.:**
Char. 175: 0 → 1
Char. 190: 0 → 1
Char. 218: 0 → 1
Char. 243: 0 → 2
Char. 244: 0 → 1
Char. 251: 0 → 1

***Sinosaurosphargis yunguiensis*:**
Char. 8: 0 → 1
Char. 30: 0 → 1
Char. 53: 0 → 1
Char. 89: 1 → 0
Char. 127: 1 → 0
Char. 150: 1 → 0
Char. 154: 0 → 2
Char. 167: 1 → 0
Char. 253: 0 → 1
Char. 255: 0 → 1

**Squamata:**
Char. 45: 0 → 1
Char. 79: 0 → 2
Char. 80: 1 → 0
Char. 92: 1 → 0
Char. 109: 1 → 0
Char. 160: 0 → 1
Char. 200: 0 → 1
Char. 245: 0 → 1

***Trilophosaurus buettneri*:**
Char. 5: 1 → 0
Char. 11: 0 → 1
Char. 55: 1 → 0
Char. 93: 1 → 0
Char. 104: 0 → 1
Char. 106: 0 → 1
Char. 113: 0 → 1
Char. 122: 0 → 1
Char. 136: 1 → 0
Char. 144: 1 → 0
Char. 157: 0 → 1
Char. 159: 1 → 0
Char. 177: 0 → 12
Char. 194: 0 → 1
Char. 203: 1 → 2
Char. 207: 1 → 0
Char. 208: 1 → 0
Char. 272: 1 → 0

***Youngina capensis*:**
Char. 5: 1 → 0
Char. 23: 1 → 0
Char. 25: 0 → 1
Char. 27: 0 → 1
Char. 38: 1 → 0
Char. 44: 1 → 0
Char. 56: 0 → 1
Char. 75: 0 → 1
Char. 84: 1 → 0
Char. 92: 1 → 0
Char. 94: 1 → 0
Char. 134: 0 → 1
Char. 163: 1 → 0
Char. 170: 0 → 1
Char. 211: 0 → 1
Char. 215: 0 → 1
Char. 224: 0 → 1
Char. 239: 0 → 1

**Node 50:**
Char. 46: 1 → 0
Char. 88: 1 → 0
Char. 89: 1 → 0
Char. 93: 1 → 0
Char. 176: 0 → 1
Char. 195: 0 → 2
Char. 198: 0 → 1
Char. 246: 1 → 2
Char. 259: 0 → 1
Char. 265: 1 → 0
Char. 270: 0 → 1

**Node 51:**
Char. 65: 0 → 1
Char. 131: 0 → 1
Char. 184: 0 → 1
Char. 205: 0 → 1
Char. 210: 0 → 1
Char. 219: 1 → 0
Char. 241: 0 → 1
Char. 246: 0 → 1
Char. 254: 0 → 1
Char. 255: 0 → 1
Char. 256: 0 → 1
Char. 268: 0 → 1
Char. 269: 0 → 1

**Node 52:**
Char. 0: 1 → 2
Char. 15: 0 → 1
Char. 33: 1 → 2
Char. 44: 1 → 0
Char. 62: 1 → 0
Char. 64: 0 → 1
Char. 84: 1 → 2
Char. 130: 0 → 1
Char. 134: 0 → 2
Char. 147: 0 → 1
Char. 152: 0 → 1
Char. 155: 0 → 1

Char. 158: 0 → 1
Char. 161: 0 → 1
Char. 174: 0 → 1
Char. 181: 0 → 1
Char. 203: 1 → 2
Char. 247: 0 → 1
Char. 251: 0 → 1
Char. 252: 0 → 1
Char. 253: 0 → 2

**Node 53:**
Char. 0: 0 → 1
Char. 59: 0 → 1
Char. 60: 0 → 1
Char. 62: 0 → 1
Char. 72: 1 → 2
Char. 92: 0 → 1
Char. 265: 0 → 1
Char. 275: 1 → 0
Char. 278: 3 → 0

**Node 54:**
Char. 20: 0 → 1
Char. 25: 1 → 0
Char. 29: 1 → 0
Char. 50: 1 → 0
Char. 57: 1 → 0
Char. 67: 0 → 1
Char. 89: 0 → 1
Char. 94: 0 → 1
Char. 127: 0 → 1

**Node 55:**
Char. 23: 0 → 1
Char. 132: 0 → 1
Char. 166: 0 → 1
Char. 276: 1 → 0

**Node 56:**
Char. 29: 0 → 1
Char. 84: 0 → 1
Char. 88: 0 → 1
Char. 169: 0 → 1

**Node 57:**
Char. 72: 0 → 1
Char. 81: 0 → 1
Char. 93: 0 → 1
Char. 97: 0 → 1
Char. 104: 1 → 0
Char. 144: 0 → 1
Char. 173: 0 → 1
Char. 183: 0 → 1

**Node 59:**
Char. 95: 0 → 1
Char. 113: 0 → 1
Char. 114: 0 → 1

**Node 60:**
Char. 20: 0 → 1
Char. 23: 1 → 0
Char. 47: 0 → 1
Char. 79: 0 → 1
Char. 110: 0 → 1
Char. 131: 0 → 1
Char. 137: 0 → 1
Char. 140: 0 → 2
Char. 147: 0 → 1

**Node 61:**
Char. 29: 1 → 0
Char. 59: 0 → 1
Char. 60: 0 → 1
Char. 67: 0 → 1
Char. 72: 1 → 2
Char. 84: 1 → 0
Char. 111: 0 → 1
Char. 116: 1 → 0
Char. 154: 1 → 0
Char. 159: 1 → 0
Char. 166: 1 → 0
Char. 169: 1 → 0
Char. 170: 0 → 1
Char. 197: 0 → 1
Char. 221: 1 → 0

**Node 62:**
Char. 19: 0 → 1
Char. 92: 1 → 0

**Node 63:**
Char. 4: 0 → 1
Char. 15: 0 → 1
Char. 29: 0 → 1
Char. 213: 0 → 1
Char. 226: 0 → 2
Char. 228: 0 → 1
Char. 275: 0 → 1

**Node 64:**
Char. 58: 0 → 1
Char. 61: 0 → 1
Char. 66: 0 → 1
Char. 69: 0 → 1
Char. 107: 0 → 1
Char. 126: 0 → 1
Char. 131: 0 → 1
Char. 134: 0 → 1
Char. 140: 0 → 1
Char. 147: 0 → 1
Char. 150: 0 → 1
Char. 167: 0 → 1
Char. 176: 0 → 1
Char. 190: 0 → 1
Char. 205: 0 → 1
Char. 208: 0 → 1
Char. 230: 0 → 1
Char. 239: 0 → 1
Char. 260: 2 → 1

**Node 65:**
Char. 73: 0 → 1
Char. 205: 0 → 1
Char. 273: 0 → 1
Char. 276: 0 → 1

**Node 66:**
Char. 18: 0 → 1
Char. 25: 1 → 0
Char. 37: 0 → 1
Char. 103: 0 → 1
Char. 106: 0 → 1
Char. 107: 0 → 1
Char. 110: 0 → 1
Char. 126: 0 → 1
Char. 150: 0 → 1

**Node 67:**
Char. 20: 0 → 1
Char. 38: 1 → 2
Char. 39: 0 → 1
Char. 44: 1 → 0
Char. 49: 0 → 1
Char. 66: 0 → 1
Char. 80: 1 → 0
Char. 95: 0 → 1
Char. 135: 0 → 1
Char. 137: 0 → 1
Char. 147: 0 → 1
Char. 157: 0 → 1
Char. 158: 0 → 1
Char. 183: 1 → 2
Char. 186: 0 → 1
Char. 187: 0 → 1
Char. 194: 0 → 1
Char. 197: 0 → 1
Char. 201: 1 → 0
Char. 211: 0 → 1
Char. 252: 0 → 1

**Node 68:**
Char. 87: 0 → 1
Char. 110: 0 → 1

**Node 69:**
Char. 79: 0 → 1
Char. 133: 0 → 1

**Node 70:**
Char. 100: 0 → 1
Char. 113: 0 → 1

**Node 71:**
Char. 23: 1 → 0
Char. 70: 0 → 1
Char. 118: 0 → 1
Char. 125: 0 → 1

**Node 72:**
Char. 38: 1 → 2
Char. 39: 0 → 1
Char. 50: 1 → 0
Char. 58: 0 → 1
Char. 59: 0 → 1
Char. 60: 0 → 1
Char. 72: 1 → 2
Char. 88: 1 → 0
Char. 95: 0 → 1
Char. 104: 0 → 1
Char. 105: 0 → 1
Char. 106: 0 → 2
Char. 107: 0 → 1
Char. 109: 0 → 1
Char. 110: 0 → 1
Char. 146: 1 → 0
Char. 155: 0 → 2
Char. 183: 1 → 2

**Node 73:**
Char. 23: 0 → 1
Char. 33: 1 → 0
Char. 38: 2 → 1
Char. 39: 1 → 0
Char. 42: 0 → 1
Char. 43: 0 → 1
Char. 46: 1 → 0
Char. 49: 1 → 0
Char. 71: 1 → 0
Char. 83: 0 → 2
Char. 101: 0 → 1
Char. 103: 0 → 1
Char. 106: 0 → 1
Char. 161: 0 → 1
Char. 163: 1 → 0
Char. 172: 0 → 2
Char. 174: 0 → 1
Char. 188: 0 → 1
Char. 203: 1 → 2
Char. 212: 0 → 1
Char. 216: 1 → 0
Char. 221: 1 → 0
Char. 236: 0 → 1
Char. 238: 0 → 2
Char. 241: 0 → 1
Char. 242: 0 → 1
Char. 273: 0 → 1
Char. 274: 0 → 1
Char. 275: 1 → 0

**Node 74:**
Char. 52: 0 → 1
Char. 75: 0 → 1
Char. 84: 1 → 0
Char. 87: 0 → 1
Char. 110: 0 → 1
Char. 121: 0 → 1
Char. 180: 1 → 0
Char. 230: 0 → 1

**Node 75:**
Char. 44: 1 → 0
Char. 66: 0 → 1
Char. 78: 1 → 0
Char. 88: 1 → 0
Char. 93: 1 → 0
Char. 123: 1 → 0
Char. 129: 0 → 1
Char. 192: 1 → 0

**Node 76:**
Char. 107: 0 → 1

**Node 77:**
Char. 2: 0 → 1
Char. 6: 0 → 1
Char. 186: 0 → 1
Char. 198: 0 → 1
Char. 200: 0 → 1
Char. 220: 0 → 1
Char. 239: 1 → 0

**Node 78:**
Char. 98: 1 → 0
Char. 113: 0 → 1
Char. 181: 0 → 1
Char. 206: 0 → 2

**Node 79:**
Char. 35: 0 → 1
Char. 102: 0 → 1
Char. 103: 0 → 1
Char. 184: 0 → 1
Char. 201: 0 → 1
Char. 210: 0 → 1
Char. 223: 0 → 1
Char. 229: 0 → 1

**Node 80:**
Char. 17: 0 → 1
Char. 42: 0 → 1
Char. 251: 0 → 1
Char. 264: 0 → 1

**Node 81:**
Char. 41: 0 → 1
Char. 61: 1 → 2
Char. 112: 1 → 0
Char. 138: 0 → 1
Char. 146: 1 → 0
Char. 155: 0 → 1
Char. 192: 1 → 2
Char. 224: 0 → 1
Char. 226: 0 → 1
Char. 227: 0 → 1
Char. 233: 0 → 1
Char. 267: 01 → 2

**Node 82:**
Char. 61: 1 → 3
Char. 90: 0 → 1
Char. 91: 0 → 1
Char. 109: 1 → 0
Char. 155: 0 → 1
Char. 209: 1 → 0

**Analysis 40**
**(all taxa, implied weighting, K = 3)**

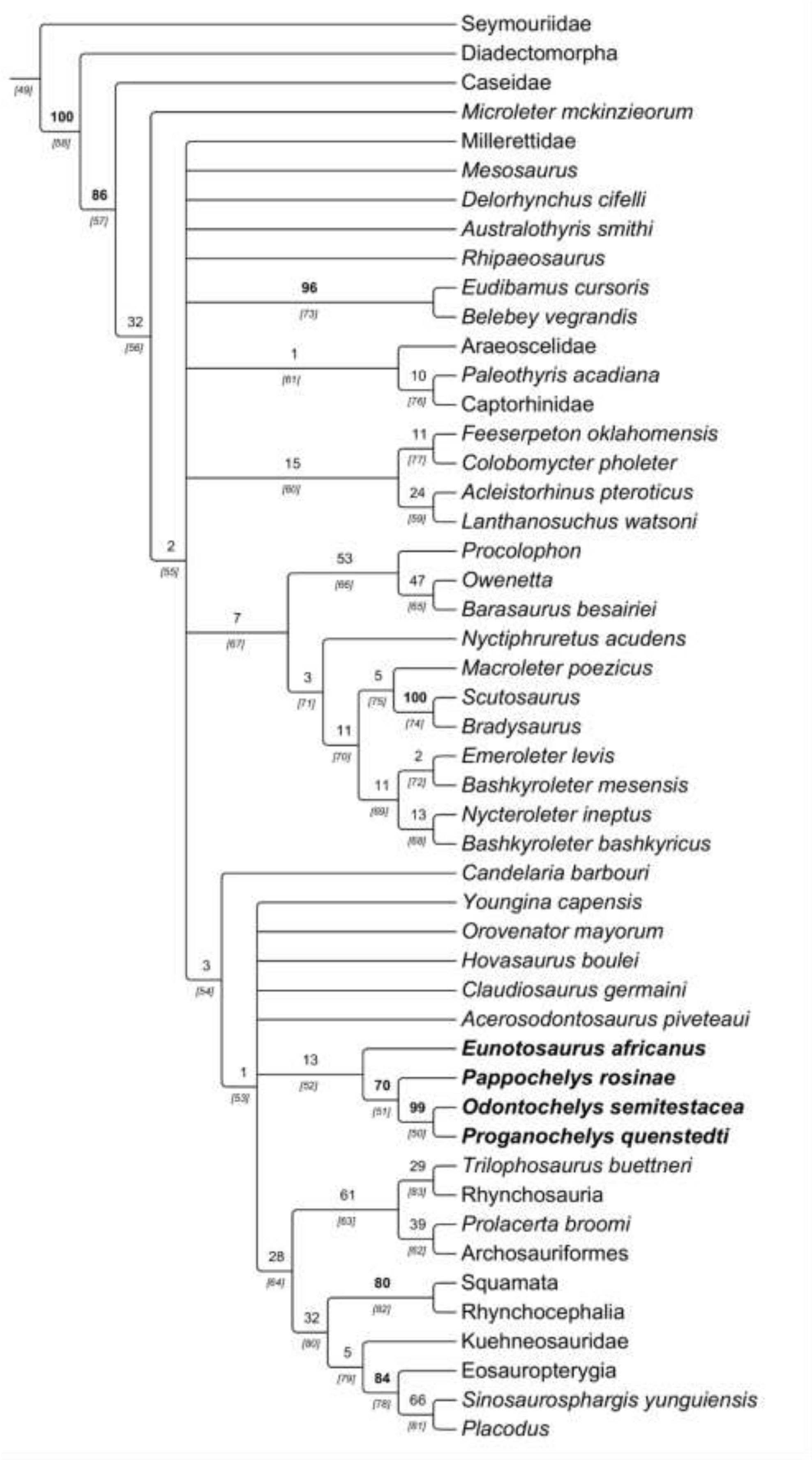

***Proganochelys quenstedti*:**
Char. 8: 0 → 1
Char. 11: 0 → 1
Char. 106: 0 → 1
Char. 108: 1 → 0
Char. 128: 0 → 1
Char. 175: 0 → 1
Char. 202: 1 → 0
Char. 207: 1 → 0
Char. 209: 1 → 0
Char. 244: 0 → 1
Char. 248: 0 → 1
Char. 250: 0 → 1
Char. 252: 1 → 0
Char. 262: 0 → 1

***Pappochelys rosinae*:**
Char. 0: 2 → 0
Char. 1: 0 → 1
Char. 5: 1 → 0
Char. 12: 0 → 1
Char. 41: 0 → 1
Char. 49: 0 → 1
Char. 75: 0 → 1
Char. 169: 1 → 0
Char. 260: 2 → 0

***Eunotosaurus africanus*:**
Char. 19: 0 → 1
Char. 59: 1 → 0
Char. 60: 1 → 0
Char. 72: 2 → 1
Char. 76: 0 → 1
Char. 97: 1 → 0
Char. 103: 0 → 1
Char. 153: 1 → 0
Char. 191: 1 → 0
Char. 192: 1 → 0
Char. 202: 1 → 0
Char. 211: 0 → 1
Char. 222: 1 → 0
Char. 237: 1 → 0
Char. 248: 0 → 1
Char. 249: 0 → 2
Char. 250: 0 → 1
Char. 263: 0 → 1
Char. 273: 0 → 1
Char. 274: 0 → 1
Char. 275: 0 → 1
Char. 276: 0 → 1
Char. 277: 0 → 1
Char. 278: 0 → 3

***Acerosodontosaurus piveteaui*:**
Char. 78: 1 → 0
Char. 81: 1 → 0
Char. 127: 1 → 0
Char. 128: 0 → 1
Char. 155: 0 → 1
Char. 206: 0 → 2
Char. 208: 0 → 1
Char. 278: 0 → 1

***Acleistorhinus pteroticus*:**
Char. 21: 0 → 1
Char. 146: 1 → 0

**Araeoscelidae:**
Char. 0: 0 → 1
Char. 28: 0 → 1
Char. 43: 0 → 1
Char. 57: 1 → 0
Char. 89: 0 → 1
Char. 106: 0 → 1
Char. 120: 0 → 1
Char. 193: 0 → 1
Char. 222: 0 → 1
Char. 224: 0 → 1
Char. 234: 0 → 1
Char. 266: 0 → 1
Char. 278: 3 → 1

**Archosauriformes:**
Char. 32: 0 → 1
Char. 94: 1 → 0
Char. 112: 1 → 0
Char. 152: 0 → 1
Char. 154: 01 → 2
Char. 166: 1 → 0
Char. 171: 0 → 1
Char. 185: 0 → 1
Char. 201: 0 → 1
Char. 204: 0 → 1
Char. 218: 0 → 3
Char. 224: 0 → 1
Char. 242: 0 → 1

***Australothyris smithi*:**
Char. 24: 0 → 1
Char. 34: 0 → 1
Char. 55: 1 → 0
Char. 57: 1 → 0
Char. 71: 1 → 0
Char. 79: 0 → 1
Char. 98: 1 → 0
Char. 100: 0 → 1
Char. 103: 0 → 1
Char. 110: 0 → 1
Char. 123: 1 → 0
Char. 129: 0 → 1
Char. 131: 0 → 1
Char. 132: 1 → 0
Char. 144: 1 → 0
Char. 147: 0 → 1
Char. 149: 1 → 0
Char. 150: 0 → 1
Char. 159: 1 → 0
Char. 192: 1 → 0

***Barasaurus besairiei*:**
Char. 33: 1 → 0

***Bashkyroleter bashkyricus*:**
Char. 275: 1 → 0

***Bashkyroleter mesensis*:**
Char. 12: 0 → 1
Char. 66: 1 → 0
Char. 169: 1 → 0

***Belebey vegrandis*:**
Char. 154: 1 → 0

***Bradysaurus* spp.:**
Char. 19: 0 → 1
Char. 25: 1 → 0
Char. 73: 0 → 1
Char. 79: 0 → 1
Char. 135: 1 → 0
Char. 249: 0 → 1

***Candelaria barbouri*:**
Char. 1: 0 → 1
Char. 8: 0 → 1
Char. 15: 0 → 1
Char. 33: 1 → 2
Char. 49: 0 → 1
Char. 55: 1 → 0
Char. 79: 0 → 2
Char. 88: 1 → 0
Char. 95: 0 → 1
Char. 126: 0 → 1
Char. 132: 1 → 0
Char. 154: 1 → 2
Char. 159: 1 → 0
Char. 169: 1 → 0
Char. 276: 0 → 1
Char. 277: 0 → 1

**Captorhinidae:**
Char. 3: 0 → 1
Char. 25: 1 → 0
Char. 26: 0 → 1
Char. 73: 0 → 1
Char. 75: 0 → 1
Char. 108: 1 → 0
Char. 180: 1 → 0
Char. 183: 1 → 0
Char. 201: 1 → 0
Char. 203: 1 → 2
Char. 216: 1 → 0

**Caseidae:**
Char. 38: 1 → 0
Char. 46: 1 → 0
Char. 50: 1 → 0
Char. 170: 0 → 1
Char. 194: 0 → 1
Char. 273: 0 → 1
Char. 274: 0 → 1
Char. 278: 3 → 2

***Claudiosaurus germaini*:**
Char. 24: 0 → 1
Char. 27: 0 → 1
Char. 34: 0 → 1
Char. 36: 0 → 1
Char. 56: 0 → 1
Char. 64: 0 → 1
Char. 70: 0 → 1
Char. 84: 1 → 0
Char. 105: 0 → 1
Char. 106: 0 → 1
Char. 117: 01 → 2
Char. 126: 0 → 1
Char. 127: 1 → 0
Char. 130: 0 → 1
Char. 131: 0 → 1
Char. 141: 1 → 0
Char. 144: 1 → 0
Char. 166: 1 → 0
Char. 182: 0 → 1
Char. 187: 1 → 0
Char. 190: 0 → 1
Char. 199: 0 → 1
Char. 201: 0 → 1
Char. 203: 1 → 2
Char. 204: 02 → 1
Char. 222: 1 → 0
Char. 234: 1 → 0
Char. 272: 2 → 1

***Colobomycter pholeter*:**
Char. 21: 0 → 1
Char. 25: 1 → 0
Char. 84: 1 → 0
Char. 154: 1 → 0
Char. 167: 0 → 1
Char. 267: 0 → 1

***Delorhynchus cifelli*:**
Char. 18: 0 → 1
Char. 20: 0 → 1
Char. 21: 0 → 1
Char. 23: 1 → 0
Char. 24: 0 → 1
Char. 26: 0 → 1
Char. 28: 0 → 1
Char. 33: 01 → 2
Char. 39: 0 → 1
Char. 52: 0 → 1
Char. 100: 0 → 1
Char. 111: 0 → 1
Char. 116: 1 → 0
Char. 119: 1 → 0
Char. 131: 0 → 1
Char. 147: 0 → 1
Char. 156: 1 → 0
Char. 167: 0 → 1
Char. 189: 0 → 1
Char. 191: 0 → 1
Char. 267: 0 → 1

**Diadectomorpha:**
Char. 0: 0 → 1
Char. 64: 0 → 1
Char. 70: 0 → 1
Char. 122: 0 → 1
Char. 123: 1 → 0
Char. 146: 1 → 0
Char. 275: 1 → 0
Char. 278: 3 → 0

***Emeroleter levis*:**
Char. 51: 1 → 0

**Eosauropterygia:**
Char. 166: 1 → 0
Char. 174: 0 → 1
Char. 178: 0 → 1
Char. 194: 0 → 2
Char. 272: 1 → 0

***Eudibamus cursoris*:**
Char. 154: 1 → 2

***Feeserpeton oklahomensis*:**
Char. 51: 0 → 1
Char. 157: 0 → 1
Char. 158: 0 → 1

***Hovasaurus boulei*:**
Char. 41: 0 → 1
Char. 55: 1 → 0
Char. 60: 1 → 0
Char. 72: 2 → 1
Char. 77: 0 → 2
Char. 78: 1 → 0
Char. 79: 0 → 1
Char. 93: 1 → 0
Char. 113: 0 → 1
Char. 138: 0 → 1
Char. 141: 1 → 0
Char. 146: 1 → 0
Char. 206: 0 → 2
Char. 215: 0 → 1
Char. 219: 1 → 0
Char. 224: 0 → 1
Char. 278: 0 → 3

**Kuehneosauridae:**
Char. 7: 0 → 1
Char. 24: 0 → 1
Char. 27: 0 → 1
Char. 43: 1 → 0
Char. 79: 0 → 2
Char. 107: 1 → 0
Char. 140: 1 → 0
Char. 147: 1 → 0
Char. 148: 1 → 0
Char. 245: 0 → 1
Char. 278: 0 → 3

***Lanthanosuchus watsoni*:**
Char. 25: 1 → 0
Char. 51: 0 → 1
Char. 86: 0 → 1
Char. 98: 1 → 0
Char. 138: 0 → 1
Char. 144: 1 → 0
Char. 154: 1 → 2

***Macroleter poezicus*:**
Char. 9: 0 → 1
Char. 26: 0 → 1
Char. 66: 1 → 2
Char. 126: 0 → 1
Char. 134: 0 → 2
Char. 140: 0 → 1
Char. 146: 1 → 0
Char. 169: 1 → 0

***Mesosaurus* spp.:**
Char. 0: 0 → 1
Char. 2: 0 → 1
Char. 6: 0 → 1
Char. 8: 0 → 1
Char. 9: 0 → 1
Char. 13: 0 → 1
Char. 19: 0 → 1
Char. 26: 0 → 1
Char. 29: 1 → 0
Char. 38: 1 → 0
Char. 41: 0 → 1
Char. 50: 1 → 0
Char. 67: 0 → 1
Char. 84: 1 → 0
Char. 94: 0 → 1
Char. 107: 0 → 1
Char. 109: 0 → 1
Char. 111: 0 → 1
Char. 115: 0 → 1
Char. 146: 1 → 0
Char. 149: 1 → 0
Char. 159: 1 → 0
Char. 164: 0 → 1
Char. 166: 1 → 0
Char. 167: 0 → 1
Char. 176: 0 → 1
Char. 183: 1 → 0
Char. 184: 0 → 1
Char. 192: 1 → 0
Char. 199: 0 → 1
Char. 202: 0 → 1
Char. 206: 0 → 1
Char. 209: 0 → 1
Char. 217: 0 → 1
Char. 219: 0 → 1
Char. 220: 0 → 1
Char. 231: 0 → 1
Char. 260: 2 → 0
Char. 272: 2 → 0
Char. 278: 3 → 0

***Microleter mckinzieorum*:**
Char. 0: 0 → 1
Char. 18: 0 → 1
Char. 39: 0 → 1
Char. 57: 1 → 0
Char. 70: 0 → 1
Char. 94: 0 → 1
Char. 106: 0 → 1
Char. 110: 0 → 1
Char. 278: 3 → 1

**Millerettidae:**
Char. 23: 1 → 0
Char. 24: 0 → 1
Char. 25: 1 → 0
Char. 44: 1 → 0
Char. 56: 0 → 1
Char. 57: 1 → 0
Char. 66: 0 → 2
Char. 78: 1 → 0
Char. 80: 1 → 0
Char. 84: 1 → 2
Char. 88: 1 → 0
Char. 96: 1 → 0
Char. 121: 0 → 1
Char. 124: 0 → 1
Char. 127: 0 → 1
Char. 135: 0 → 1
Char. 145: 0 → 1
Char. 159: 1 → 0
Char. 166: 1 → 0
Char. 180: 1 → 0
Char. 192: 1 → 0
Char. 202: 0 → 1
Char. 211: 0 → 1
Char. 230: 0 → 1
Char. 234: 0 → 1
Char. 248: 0 → 1
Char. 252: 0 → 1
Char. 253: 0 → 1

***Nycteroleter ineptus*:**
Char. 66: 1 → 0

***Nyctiphruretus acudens*:**
Char. 21: 0 → 1
Char. 41: 0 → 1
Char. 66: 1 → 2
Char. 81: 1 → 0
Char. 84: 1 → 2
Char. 94: 0 → 1
Char. 166: 1 → 0
Char. 224: 0 → 1
Char. 266: 0 → 1
Char. 272: 2 → 1
Char. 276: 0 → 1

***Orovenator mayorum*:**
Char. 8: 0 → 1
Char. 23: 1 → 0
Char. 24: 0 → 1
Char. 33: 1 → 0
Char. 36: 0 → 1
Char. 50: 0 → 1
Char. 92: 1 → 0
Char. 94: 1 → 0
Char. 135: 1 → 0
Char. 159: 1 → 0
Char. 160: 0 → 1
Char. 165: 0 → 1
Char. 278: 0 → 1

***Owenetta* spp.:**
Char. 142: 1 → 0
Char. 169: 1 → 0

***Paleothyris acadiana*:**
Char. 23: 1 → 0
Char. 66: 1 → 2
Char. 102: 0 → 1
Char. 146: 1 → 0

***Placodus* spp.:**
Char. 0: 1 → 2
Char. 9: 0 → 1
Char. 12: 0 → 1
Char. 13: 0 → 1
Char. 19: 0 → 1
Char. 31: 0 → 1
Char. 46: 1 → 0
Char. 57: 0 → 1
Char. 78: 1 → 0
Char. 93: 1 → 0
Char. 102: 1 → 2
Char. 109: 1 → 0
Char. 140: 1 → 0
Char. 155: 0 → 1
Char. 163: 1 → 0
Char. 164: 0 → 1

***Procolophon* spp.:**
Char. 41: 0 → 1
Char. 69: 0 → 1
Char. 79: 0 → 1
Char. 86: 0 → 1
Char. 101: 0 → 1
Char. 141: 0 → 1
Char. 149: 1 → 0
Char. 203: 1 → 2
Char. 215: 0 → 1
Char. 230: 0 → 1
Char. 235: 1 → 2
Char. 237: 0 → 1
Char. 238: 0 → 1
Char. 272: 2 → 1
Char. 278: 3 → 0

***Prolacerta broomi*:**
Char. 58: 1 → 0
Char. 66: 1 → 0
Char. 67: 1 → 0
Char. 80: 1 → 0
Char. 139: 1 → 0
Char. 147: 1 → 0
Char. 192: 1 → 0
Char. 203: 1 → 2
Char. 206: 0 → 12

***Rhipaeosaurus* spp.:**
Char. 172: 0 → 1
Char. 183: 1 → 2
Char. 194: 0 → 1
Char. 201: 1 → 0
Char. 211: 0 → 1
Char. 215: 0 → 1
Char. 226: 0 → 1
Char. 235: 1 → 0
Char. 252: 0 → 1
Char. 277: 0 → 1

**Rhynchocephalia:**
Char. 0: 1 → 2
Char. 23: 1 → 0
Char. 24: 0 → 1
Char. 77: 0 → 1
Char. 88: 1 → 0
Char. 94: 1 → 0
Char. 139: 1 → 0
Char. 167: 1 → 0
Char. 205: 1 → 0

**Rhynchosauria:**
Char. 0: 1 → 0
Char. 7: 0 → 1
Char. 9: 0 → 1
Char. 26: 0 → 1
Char. 44: 1 → 0
Char. 68: 0 → 1
Char. 99: 1 → 0
Char. 150: 1 → 0
Char. 160: 0 → 1
Char. 161: 0 → 1
Char. 171: 0 → 2
Char. 223: 0 → 1
Char. 224: 0 → 1
Char. 241: 0 → 1

***Scutosaurus* spp.:**
Char. 175: 0 → 1
Char. 190: 0 → 1
Char. 218: 0 → 1
Char. 243: 0 → 2
Char. 244: 0 → 1
Char. 251: 0 → 1

***Sinosaurosphargis yunguiensis*:**
Char. 8: 0 → 1
Char. 30: 0 → 1
Char. 53: 0 → 1
Char. 89: 1 → 0
Char. 127: 1 → 0
Char. 150: 1 → 0
Char. 154: 0 → 2
Char. 167: 1 → 0
Char. 253: 0 → 1
Char. 255: 0 → 1

**Squamata:**
Char. 45: 0 → 1
Char. 79: 0 → 2
Char. 80: 1 → 0
Char. 92: 1 → 0
Char. 109: 1 → 0
Char. 160: 0 → 1
Char. 200: 0 → 1
Char. 245: 0 → 1

***Trilophosaurus buettneri*:**
Char. 5: 1 → 0
Char. 11: 0 → 1
Char. 55: 1 → 0
Char. 93: 1 → 0
Char. 104: 0 → 1
Char. 106: 0 → 1
Char. 113: 0 → 1
Char. 122: 0 → 1
Char. 136: 1 → 0
Char. 144: 1 → 0
Char. 157: 0 → 1
Char. 159: 1 → 0
Char. 177: 0 → 12
Char. 194: 0 → 1
Char. 203: 1 → 2
Char. 207: 1 → 0
Char. 208: 1 → 0
Char. 272: 1 → 0

***Youngina capensis*:**
Char. 5: 1 → 0
Char. 23: 1 → 0
Char. 25: 0 → 1
Char. 27: 0 → 1
Char. 38: 1 → 0
Char. 44: 1 → 0
Char. 56: 0 → 1
Char. 75: 0 → 1
Char. 84: 1 → 0
Char. 92: 1 → 0
Char. 94: 1 → 0
Char. 134: 0 → 1
Char. 163: 1 → 0
Char. 170: 0 → 1
Char. 211: 0 → 1
Char. 215: 0 → 1
Char. 224: 0 → 1
Char. 239: 0 → 1

**Node 50:**
Char. 46: 1 → 0
Char. 88: 1 → 0
Char. 89: 1 → 0
Char. 93: 1 → 0
Char. 176: 0 → 1
Char. 195: 0 → 2
Char. 198: 0 → 1
Char. 246: 1 → 2
Char. 259: 0 → 1
Char. 265: 1 → 0
Char. 270: 0 → 1

**Node 51:**
Char. 65: 0 → 1
Char. 131: 0 → 1
Char. 184: 0 → 1
Char. 205: 0 → 1
Char. 210: 0 → 1
Char. 219: 1 → 0
Char. 241: 0 → 1
Char. 246: 0 → 1
Char. 254: 0 → 1
Char. 255: 0 → 1
Char. 256: 0 → 1
Char. 268: 0 → 1
Char. 269: 0 → 1

**Node 52:**
Char. 0: 1 → 2
Char. 15: 0 → 1
Char. 33: 1 → 2
Char. 44: 1 → 0
Char. 62: 1 → 0
Char. 64: 0 → 1
Char. 84: 1 → 2
Char. 130: 0 → 1
Char. 134: 0 → 2
Char. 147: 0 → 1
Char. 152: 0 → 1
Char. 155: 0 → 1

Char. 158: 0 → 1
Char. 161: 0 → 1
Char. 174: 0 → 1
Char. 181: 0 → 1
Char. 203: 1 → 2
Char. 247: 0 → 1
Char. 251: 0 → 1
Char. 252: 0 → 1
Char. 253: 0 → 2

**Node 53:**
Char. 0: 0 → 1
Char. 59: 0 → 1
Char. 60: 0 → 1
Char. 62: 0 → 1
Char. 72: 1 → 2
Char. 92: 0 → 1
Char. 265: 0 → 1
Char. 275: 1 → 0
Char. 278: 3 → 0

**Node 54:**
Char. 20: 0 → 1
Char. 25: 1 → 0
Char. 29: 1 → 0
Char. 50: 1 → 0
Char. 57: 1 → 0
Char. 67: 0 → 1
Char. 89: 0 → 1
Char. 94: 0 → 1
Char. 127: 0 → 1

**Node 55:**
Char. 23: 0 → 1
Char. 132: 0 → 1
Char. 166: 0 → 1
Char. 276: 1 → 0

**Node 56:**
Char. 29: 0 → 1
Char. 84: 0 → 1
Char. 88: 0 → 1
Char. 169: 0 → 1

**Node 57:**
Char. 72: 0 → 1
Char. 81: 0 → 1
Char. 93: 0 → 1
Char. 97: 0 → 1
Char. 104: 1 → 0
Char. 144: 0 → 1
Char. 173: 0 → 1
Char. 183: 0 → 1

**Node 59:**
Char. 95: 0 → 1
Char. 113: 0 → 1
Char. 114: 0 → 1

**Node 60:**
Char. 20: 0 → 1
Char. 23: 1 → 0
Char. 47: 0 → 1
Char. 79: 0 → 1
Char. 110: 0 → 1
Char. 131: 0 → 1
Char. 137: 0 → 1
Char. 140: 0 → 2
Char. 147: 0 → 1

**Node 61:**
Char. 29: 1 → 0
Char. 59: 0 → 1
Char. 60: 0 → 1
Char. 67: 0 → 1
Char. 72: 1 → 2
Char. 84: 1 → 0
Char. 111: 0 → 1
Char. 116: 1 → 0
Char. 154: 1 → 0
Char. 159: 1 → 0
Char. 166: 1 → 0
Char. 169: 1 → 0
Char. 170: 0 → 1
Char. 197: 0 → 1
Char. 221: 1 → 0

**Node 62:**
Char. 19: 0 → 1
Char. 92: 1 → 0

**Node 63:**
Char. 4: 0 → 1
Char. 15: 0 → 1
Char. 29: 0 → 1
Char. 213: 0 → 1
Char. 226: 0 → 2
Char. 228: 0 → 1
Char. 275: 0 → 1

**Node 64:**
Char. 58: 0 → 1
Char. 61: 0 → 1
Char. 66: 0 → 1
Char. 69: 0 → 1
Char. 107: 0 → 1
Char. 126: 0 → 1
Char. 131: 0 → 1
Char. 134: 0 → 1
Char. 140: 0 → 1
Char. 147: 0 → 1
Char. 150: 0 → 1
Char. 167: 0 → 1
Char. 176: 0 → 1
Char. 190: 0 → 1
Char. 205: 0 → 1
Char. 208: 0 → 1
Char. 230: 0 → 1
Char. 239: 0 → 1
Char. 260: 2 → 1

**Node 65:**
Char. 73: 0 → 1
Char. 205: 0 → 1
Char. 273: 0 → 1
Char. 276: 0 → 1

**Node 66:**
Char. 18: 0 → 1
Char. 25: 1 → 0
Char. 37: 0 → 1
Char. 103: 0 → 1
Char. 106: 0 → 1
Char. 107: 0 → 1
Char. 126: 0 → 1
Char. 150: 0 → 1

**Node 67:**
Char. 20: 0 → 1
Char. 38: 1 → 2
Char. 39: 0 → 1
Char. 44: 1 → 0
Char. 49: 0 → 1
Char. 66: 0 → 1
Char. 80: 1 → 0
Char. 95: 0 → 1
Char. 135: 0 → 1
Char. 137: 0 → 1
Char. 147: 0 → 1
Char. 157: 0 → 1
Char. 158: 0 → 1
Char. 183: 1 → 2
Char. 186: 0 → 1
Char. 187: 0 → 1
Char. 194: 0 → 1
Char. 197: 0 → 1
Char. 201: 1 → 0
Char. 211: 0 → 1
Char. 252: 0 → 1

**Node 69:**
Char. 133: 0 → 1

**Node 70:**
Char. 100: 0 → 1
Char. 113: 0 → 1

**Node 71:**
Char. 23: 1 → 0
Char. 70: 0 → 1
Char. 118: 0 → 1
Char. 125: 0 → 1

**Node 73:**
Char. 38: 1 → 2
Char. 39: 0 → 1
Char. 50: 1 → 0
Char. 58: 0 → 1
Char. 59: 0 → 1
Char. 60: 0 → 1
Char. 72: 1 → 2
Char. 88: 1 → 0
Char. 95: 0 → 1
Char. 104: 0 → 1
Char. 105: 0 → 1
Char. 106: 0 → 2
Char. 107: 0 → 1
Char. 109: 0 → 1
Char. 110: 0 → 1
Char. 146: 1 → 0
Char. 155: 0 → 2
Char. 183: 1 → 2

**Node 74:**
Char. 23: 0 → 1
Char. 33: 1 → 0
Char. 38: 2 → 1
Char. 39: 1 → 0
Char. 42: 0 → 1
Char. 43: 0 → 1
Char. 46: 1 → 0
Char. 49: 1 → 0
Char. 71: 1 → 0
Char. 83: 0 → 2
Char. 101: 0 → 1
Char. 103: 0 → 1
Char. 106: 0 → 1
Char. 161: 0 → 1
Char. 163: 1 → 0
Char. 172: 0 → 2
Char. 174: 0 → 1
Char. 188: 0 → 1
Char. 203: 1 → 2
Char. 212: 0 → 1
Char. 216: 1 → 0
Char. 221: 1 → 0
Char. 236: 0 → 1
Char. 238: 0 → 2
Char. 241: 0 → 1
Char. 242: 0 → 1
Char. 273: 0 → 1
Char. 274: 0 → 1
Char. 275: 1 → 0

**Node 75:**
Char. 52: 0 → 1
Char. 75: 0 → 1
Char. 84: 1 → 0
Char. 121: 0 → 1
Char. 180: 1 → 0
Char. 230: 0 → 1

**Node 76:**
Char. 44: 1 → 0
Char. 66: 0 → 1
Char. 78: 1 → 0
Char. 88: 1 → 0
Char. 93: 1 → 0
Char. 123: 1 → 0
Char. 129: 0 → 1
Char. 192: 1 → 0

**Node 77:**
Char. 107: 0 → 1

**Node 78:**
Char. 2: 0 → 1
Char. 6: 0 → 1
Char. 186: 0 → 1
Char. 198: 0 → 1
Char. 200: 0 → 1
Char. 220: 0 → 1
Char. 239: 1 → 0

**Node 79:**
Char. 98: 1 → 0
Char. 113: 0 → 1
Char. 181: 0 → 1
Char. 206: 0 → 2

**Node 80:**
Char. 35: 0 → 1
Char. 102: 0 → 1
Char. 103: 0 → 1
Char. 184: 0 → 1
Char. 201: 0 → 1
Char. 210: 0 → 1
Char. 223: 0 → 1
Char. 229: 0 → 1

**Node 81:**
Char. 17: 0 → 1
Char. 42: 0 → 1
Char. 251: 0 → 1
Char. 264: 0 → 1

**Node 82:**
Char. 41: 0 → 1
Char. 61: 1 → 2
Char. 112: 1 → 0
Char. 138: 0 → 1
Char. 146: 1 → 0
Char. 155: 0 → 1
Char. 192: 1 → 2
Char. 224: 0 → 1
Char. 226: 0 → 1
Char. 227: 0 → 1
Char. 233: 0 → 1
Char. 267: 01 → 2

**Node 83:**
Char. 61: 1 → 3
Char. 90: 0 → 1
Char. 91: 0 → 1
Char. 109: 1 → 0
Char. 155: 0 → 1
Char. 209: 1 → 0

## Analysis 41
## (all taxa, implied weighting, K = 3.125)

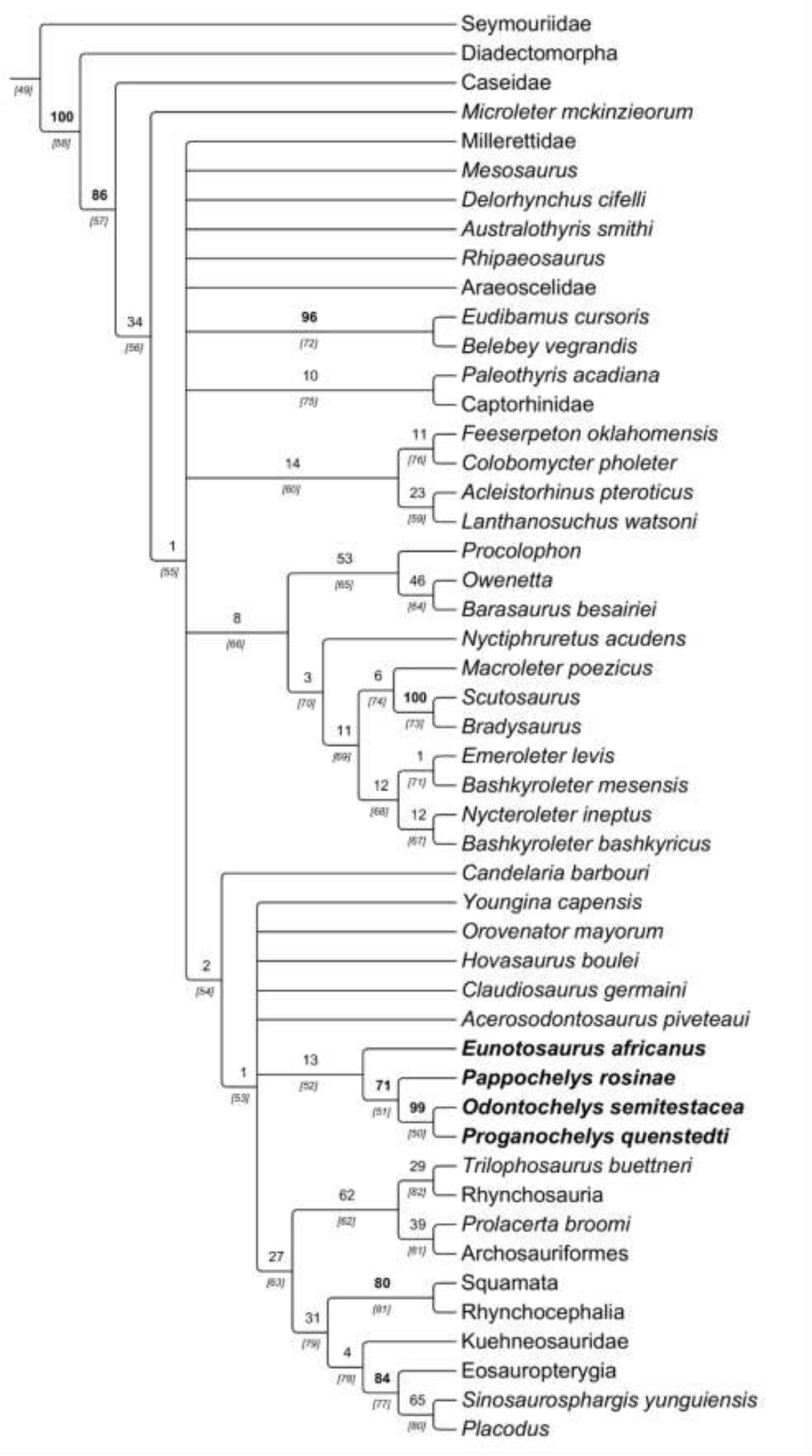

***Proganochelys quenstedti*:**
Char. 8: 0 → 1
Char. 11: 0 → 1
Char. 106: 0 → 1
Char. 108: 1 → 0
Char. 128: 0 → 1
Char. 175: 0 → 1
Char. 202: 1 → 0
Char. 207: 1 → 0
Char. 209: 1 → 0
Char. 244: 0 → 1
Char. 248: 0 → 1
Char. 250: 0 → 1
Char. 252: 1 → 0
Char. 262: 0 → 1

***Pappochelys rosinae*:**
Char. 0: 2 → 0
Char. 1: 0 → 1
Char. 5: 1 → 0
Char. 12: 0 → 1
Char. 41: 0 → 1
Char. 49: 0 → 1
Char. 75: 0 → 1
Char. 169: 1 → 0
Char. 260: 2 → 0

***Eunotosaurus africanus*:**
Char. 19: 0 → 1
Char. 59: 1 → 0
Char. 60: 1 → 0
Char. 72: 2 → 1
Char. 76: 0 → 1
Char. 97: 1 → 0
Char. 103: 0 → 1
Char. 153: 1 → 0
Char. 191: 1 → 0
Char. 192: 1 → 0
Char. 202: 1 → 0
Char. 211: 0 → 1
Char. 222: 1 → 0
Char. 237: 1 → 0
Char. 248: 0 → 1
Char. 249: 0 → 2
Char. 250: 0 → 1
Char. 263: 0 → 1
Char. 273: 0 → 1
Char. 274: 0 → 1
Char. 275: 0 → 1
Char. 276: 0 → 1
Char. 277: 0 → 1
Char. 278: 0 → 3

***Acerosodontosaurus piveteaui*:**
Char. 78: 1 → 0
Char. 81: 1 → 0
Char. 127: 1 → 0
Char. 128: 0 → 1
Char. 155: 0 → 1
Char. 206: 0 → 2
Char. 208: 0 → 1
Char. 278: 0 → 1

***Acleistorhinus pteroticus*:**
Char. 21: 0 → 1
Char. 146: 1 → 0

**Araeoscelidae:**
Char. 0: 0 → 1
Char. 27: 0 → 1
Char. 28: 0 → 1
Char. 29: 1 → 0
Char. 38: 1 → 0
Char. 40: 2 → 0
Char. 43: 0 → 1
Char. 50: 1 → 0
Char. 57: 1 → 0
Char. 59: 0 → 1
Char. 60: 0 → 1
Char. 67: 0 → 1
Char. 72: 1 → 2
Char. 84: 1 → 0
Char. 89: 0 → 1
Char. 106: 0 → 1
Char. 111: 0 → 1
Char. 116: 1 → 0
Char. 117: 01 → 2
Char. 120: 0 → 1
Char. 154: 1 → 0
Char. 166: 1 → 0
Char. 169: 1 → 0
Char. 170: 0 → 1
Char. 193: 0 → 1
Char. 197: 0 → 1
Char. 222: 0 → 1
Char. 224: 0 → 1
Char. 234: 0 → 1
Char. 237: 0 → 1
Char. 239: 0 → 1
Char. 266: 0 → 1
Char. 278: 3 → 1

**Archosauriformes:**
Char. 32: 0 → 1
Char. 94: 1 → 0
Char. 112: 1 → 0
Char. 152: 0 → 1
Char. 154: 01 → 2
Char. 166: 1 → 0
Char. 171: 0 → 1
Char. 185: 0 → 1
Char. 201: 0 → 1
Char. 204: 0 → 1
Char. 218: 0 → 3
Char. 224: 0 → 1
Char. 242: 0 → 1

***Australothyris smithi*:**
Char. 24: 0 → 1
Char. 34: 0 → 1
Char. 55: 1 → 0
Char. 57: 1 → 0
Char. 71: 1 → 0
Char. 79: 0 → 1
Char. 85: 1 → 0
Char. 98: 1 → 0
Char. 100: 0 → 1
Char. 103: 0 → 1
Char. 110: 0 → 1
Char. 123: 1 → 0
Char. 129: 0 → 1
Char. 131: 0 → 1
Char. 132: 1 → 0
Char. 144: 1 → 0
Char. 147: 0 → 1
Char. 149: 1 → 0
Char. 150: 0 → 1
Char. 192: 1 → 0

***Barasaurus besairiei*:**
Char. 33: 1 → 0

***Bashkyroleter bashkyricus*:**
Char. 275: 1 → 0

***Bashkyroleter mesensis*:**
Char. 12: 0 → 1
Char. 66: 1 → 0
Char. 169: 1 → 0

***Belebey vegrandis*:**
Char. 154: 1 → 0

***Bradysaurus* spp.:**
Char. 19: 0 → 1
Char. 25: 1 → 0
Char. 73: 0 → 1
Char. 79: 0 → 1
Char. 135: 1 → 0
Char. 249: 0 → 1

***Candelaria barbouri*:**
Char. 1: 0 → 1
Char. 8: 0 → 1
Char. 15: 0 → 1
Char. 33: 1 → 2
Char. 49: 0 → 1
Char. 55: 1 → 0
Char. 76: 0 → 1
Char. 79: 0 → 2
Char. 88: 1 → 0
Char. 95: 0 → 1
Char. 126: 0 → 1
Char. 132: 1 → 0
Char. 154: 1 → 2
Char. 169: 1 → 0
Char. 276: 0 → 1
Char. 277: 0 → 1

**Captorhinidae:**
Char. 3: 0 → 1
Char. 25: 1 → 0
Char. 26: 0 → 1
Char. 73: 0 → 1
Char. 75: 0 → 1
Char. 108: 1 → 0
Char. 180: 1 → 0
Char. 183: 1 → 0
Char. 201: 1 → 0
Char. 203: 1 → 2
Char. 216: 1 → 0

**Caseidae:**
Char. 38: 1 → 0
Char. 46: 1 → 0
Char. 50: 1 → 0
Char. 170: 0 → 1
Char. 194: 0 → 1
Char. 273: 0 → 1
Char. 274: 0 → 1
Char. 278: 3 → 2

***Claudiosaurus germaini*:**
Char. 24: 0 → 1
Char. 27: 0 → 1
Char. 34: 0 → 1
Char. 36: 0 → 1
Char. 56: 0 → 1
Char. 64: 0 → 1
Char. 70: 0 → 1
Char. 84: 1 → 0
Char. 105: 0 → 1
Char. 106: 0 → 1
Char. 117: 01 → 2
Char. 126: 0 → 1
Char. 127: 1 → 0
Char. 130: 0 → 1
Char. 131: 0 → 1
Char. 141: 1 → 0
Char. 144: 1 → 0
Char. 166: 1 → 0
Char. 182: 0 → 1
Char. 187: 1 → 0
Char. 190: 0 → 1
Char. 199: 0 → 1
Char. 201: 0 → 1
Char. 203: 1 → 2
Char. 204: 0 → 1
Char. 222: 1 → 0
Char. 234: 1 → 0
Char. 272: 2 → 1

***Colobomycter pholeter*:**
Char. 21: 0 → 1
Char. 25: 1 → 0
Char. 84: 1 → 0
Char. 154: 1 → 0
Char. 167: 0 → 1
Char. 267: 0 → 1

***Delorhynchus cifelli*:**
Char. 5: 0 → 1
Char. 18: 0 → 1
Char. 20: 0 → 1
Char. 21: 0 → 1
Char. 23: 1 → 0
Char. 24: 0 → 1
Char. 26: 0 → 1
Char. 28: 0 → 1
Char. 33: 0 → 2
Char. 39: 0 → 1
Char. 52: 0 → 1
Char. 100: 0 → 1
Char. 111: 0 → 1
Char. 116: 1 → 0
Char. 119: 1 → 0
Char. 131: 0 → 1
Char. 147: 0 → 1
Char. 156: 1 → 0
Char. 167: 0 → 1
Char. 189: 0 → 1
Char. 191: 0 → 1
Char. 204: 0 → 2
Char. 267: 0 → 1

**Diadectomorpha:**
Char. 0: 0 → 1
Char. 64: 0 → 1
Char. 70: 0 → 1
Char. 122: 0 → 1
Char. 123: 1 → 0
Char. 146: 1 → 0
Char. 275: 1 → 0
Char. 278: 3 → 0

***Emeroleter levis*:**
Char. 51: 1 → 0

**Eosauropterygia:**
Char. 166: 1 → 0
Char. 174: 0 → 1
Char. 178: 0 → 1
Char. 194: 0 → 2
Char. 272: 1 → 0

***Eudibamus cursoris*:**
Char. 154: 1 → 2

***Feeserpeton oklahomensis*:**
Char. 51: 0 → 1
Char. 157: 0 → 1
Char. 158: 0 → 1

***Hovasaurus boulei*:**
Char. 41: 0 → 1
Char. 55: 1 → 0
Char. 60: 1 → 0
Char. 72: 2 → 1
Char. 77: 0 → 2
Char. 78: 1 → 0
Char. 79: 0 → 1
Char. 93: 1 → 0
Char. 113: 0 → 1
Char. 138: 0 → 1
Char. 141: 1 → 0
Char. 146: 1 → 0
Char. 204: 0 → 2
Char. 206: 0 → 2
Char. 215: 0 → 1
Char. 219: 1 → 0
Char. 224: 0 → 1
Char. 278: 0 → 3

**Kuehneosauridae:**
Char. 7: 0 → 1
Char. 24: 0 → 1
Char. 27: 0 → 1
Char. 43: 1 → 0
Char. 79: 0 → 2
Char. 107: 1 → 0
Char. 140: 1 → 0

Char. 147: 1 → 0
Char. 148: 1 → 0
Char. 245: 0 → 1
Char. 278: 0 → 3

***Lanthanosuchus watsoni*:**
Char. 25: 1 → 0
Char. 51: 0 → 1
Char. 76: 0 → 1
Char. 86: 0 → 1
Char. 98: 1 → 0
Char. 138: 0 → 1
Char. 144: 1 → 0
Char. 154: 1 → 2

***Macroleter poezicus*:**
Char. 9: 0 → 1
Char. 26: 0 → 1
Char. 66: 1 → 2
Char. 126: 0 → 1
Char. 134: 0 → 2
Char. 140: 0 → 1
Char. 146: 1 → 0
Char. 169: 1 → 0

***Mesosaurus* spp.:**
Char. 0: 0 → 1
Char. 2: 0 → 1
Char. 6: 0 → 1
Char. 8: 0 → 1
Char. 9: 0 → 1
Char. 13: 0 → 1
Char. 19: 0 → 1
Char. 26: 0 → 1
Char. 29: 1 → 0
Char. 33: 0 → 1
Char. 38: 1 → 0
Char. 41: 0 → 1
Char. 50: 1 → 0
Char. 67: 0 → 1
Char. 76: 0 → 1
Char. 84: 1 → 0
Char. 85: 1 → 0
Char. 94: 0 → 1
Char. 107: 0 → 1
Char. 109: 0 → 1
Char. 111: 0 → 1
Char. 115: 0 → 1
Char. 146: 1 → 0
Char. 149: 1 → 0
Char. 164: 0 → 1
Char. 166: 1 → 0
Char. 167: 0 → 1
Char. 176: 0 → 1
Char. 183: 1 → 0
Char. 184: 0 → 1
Char. 192: 1 → 0
Char. 199: 0 → 1
Char. 202: 0 → 1
Char. 204: 0 → 2
Char. 206: 0 → 1
Char. 207: 0 → 1
Char. 209: 0 → 1
Char. 217: 0 → 1
Char. 219: 0 → 1
Char. 220: 0 → 1
Char. 231: 0 → 1
Char. 260: 2 → 0
Char. 272: 2 → 0
Char. 278: 3 → 0

***Microleter mckinzieorum*:**
Char. 0: 0 → 1
Char. 18: 0 → 1
Char. 39: 0 → 1
Char. 57: 1 → 0
Char. 70: 0 → 1
Char. 76: 0 → 1
Char. 94: 0 → 1
Char. 106: 0 → 1
Char. 110: 0 → 1
Char. 278: 3 → 1

**Millerettidae:**
Char. 23: 1 → 0
Char. 24: 0 → 1
Char. 25: 1 → 0
Char. 44: 1 → 0
Char. 56: 0 → 1
Char. 57: 1 → 0
Char. 66: 0 → 2
Char. 78: 1 → 0
Char. 80: 1 → 0
Char. 84: 1 → 2
Char. 88: 1 → 0
Char. 96: 1 → 0
Char. 121: 0 → 1
Char. 124: 0 → 1
Char. 127: 0 → 1
Char. 135: 0 → 1
Char. 145: 0 → 1
Char. 166: 1 → 0
Char. 180: 1 → 0
Char. 192: 1 → 0
Char. 202: 0 → 1
Char. 211: 0 → 1
Char. 230: 0 → 1
Char. 234: 0 → 1
Char. 248: 0 → 1
Char. 252: 0 → 1
Char. 253: 0 → 1

***Nycteroleter ineptus*:**
Char. 66: 1 → 0

***Nyctiphruretus acudens*:**
Char. 21: 0 → 1
Char. 41: 0 → 1
Char. 66: 1 → 2
Char. 81: 1 → 0
Char. 84: 1 → 2
Char. 94: 0 → 1
Char. 166: 1 → 0
Char. 224: 0 → 1
Char. 266: 0 → 1
Char. 272: 2 → 1
Char. 276: 0 → 1

***Orovenator mayorum*:**
Char. 8: 0 → 1
Char. 23: 1 → 0
Char. 24: 0 → 1
Char. 33: 1 → 0
Char. 36: 0 → 1
Char. 50: 0 → 1
Char. 92: 1 → 0
Char. 94: 1 → 0
Char. 135: 1 → 0
Char. 159: 1 → 0
Char. 160: 0 → 1
Char. 165: 0 → 1
Char. 278: 0 → 1

***Owenetta* spp.:**
Char. 142: 1 → 0
Char. 169: 1 → 0

***Paleothyris acadiana*:**
Char. 23: 1 → 0
Char. 38: 1 → 0
Char. 50: 1 → 0
Char. 66: 1 → 2
Char. 102: 0 → 1
Char. 146: 1 → 0
Char. 237: 0 → 1
Char. 239: 0 → 1

***Placodus* spp.:**
Char. 0: 1 → 2
Char. 9: 0 → 1
Char. 12: 0 → 1
Char. 13: 0 → 1
Char. 19: 0 → 1
Char. 31: 0 → 1
Char. 46: 1 → 0
Char. 57: 0 → 1
Char. 78: 1 → 0
Char. 93: 1 → 0
Char. 102: 1 → 2
Char. 109: 1 → 0
Char. 140: 1 → 0
Char. 155: 0 → 1
Char. 163: 1 → 0
Char. 164: 0 → 1

***Procolophon* spp.:**
Char. 41: 0 → 1
Char. 69: 0 → 1
Char. 79: 0 → 1
Char. 86: 0 → 1
Char. 101: 0 → 1
Char. 141: 0 → 1
Char. 149: 1 → 0
Char. 203: 1 → 2
Char. 204: 0 → 1
Char. 215: 0 → 1
Char. 230: 0 → 1
Char. 235: 1 → 2
Char. 237: 0 → 1
Char. 238: 0 → 1
Char. 272: 2 → 1
Char. 278: 3 → 0

***Prolacerta broomi*:**
Char. 58: 1 → 0
Char. 66: 1 → 0
Char. 67: 1 → 0
Char. 80: 1 → 0
Char. 139: 1 → 0
Char. 147: 1 → 0
Char. 192: 1 → 0
Char. 203: 1 → 2
Char. 206: 0 → 12

***Rhipaeosaurus* spp.:**
Char. 172: 0 → 1
Char. 183: 1 → 2
Char. 194: 0 → 1
Char. 201: 1 → 0
Char. 211: 0 → 1
Char. 215: 0 → 1
Char. 226: 0 → 1
Char. 235: 1 → 0
Char. 252: 0 → 1
Char. 277: 0 → 1

**Rhynchocephalia:**
Char. 0: 1 → 2
Char. 23: 1 → 0
Char. 24: 0 → 1
Char. 77: 0 → 1
Char. 88: 1 → 0
Char. 94: 1 → 0
Char. 139: 1 → 0
Char. 167: 1 → 0
Char. 205: 1 → 0

**Rhynchosauria:**
Char. 0: 1 → 0
Char. 7: 0 → 1
Char. 9: 0 → 1
Char. 26: 0 → 1
Char. 44: 1 → 0
Char. 68: 0 → 1
Char. 99: 1 → 0
Char. 150: 1 → 0
Char. 160: 0 → 1
Char. 161: 0 → 1
Char. 171: 0 → 2
Char. 223: 0 → 1
Char. 224: 0 → 1
Char. 241: 0 → 1

***Scutosaurus* spp.:**
Char. 175: 0 → 1
Char. 190: 0 → 1
Char. 218: 0 → 1
Char. 243: 0 → 2
Char. 244: 0 → 1
Char. 251: 0 → 1

***Sinosaurosphargis yunguiensis*:**
Char. 8: 0 → 1
Char. 30: 0 → 1
Char. 53: 0 → 1
Char. 89: 1 → 0
Char. 127: 1 → 0
Char. 150: 1 → 0
Char. 154: 0 → 2
Char. 167: 1 → 0
Char. 253: 0 → 1
Char. 255: 0 → 1

**Squamata:**
Char. 45: 0 → 1
Char. 79: 0 → 2
Char. 80: 1 → 0
Char. 92: 1 → 0
Char. 109: 1 → 0
Char. 160: 0 → 1
Char. 200: 0 → 1
Char. 245: 0 → 1

***Trilophosaurus buettneri*:**
Char. 5: 1 → 0
Char. 11: 0 → 1
Char. 55: 1 → 0
Char. 93: 1 → 0
Char. 104: 0 → 1
Char. 106: 0 → 1
Char. 113: 0 → 1
Char. 122: 0 → 1
Char. 136: 1 → 0
Char. 144: 1 → 0
Char. 157: 0 → 1
Char. 159: 1 → 0
Char. 177: 0 → 12
Char. 194: 0 → 1
Char. 203: 1 → 2
Char. 207: 1 → 0
Char. 208: 1 → 0
Char. 272: 1 → 0

***Youngina capensis*:**
Char. 5: 1 → 0
Char. 23: 1 → 0
Char. 25: 0 → 1
Char. 27: 0 → 1
Char. 38: 1 → 0
Char. 44: 1 → 0
Char. 56: 0 → 1
Char. 75: 0 → 1
Char. 84: 1 → 0
Char. 92: 1 → 0
Char. 94: 1 → 0
Char. 134: 0 → 1
Char. 163: 1 → 0
Char. 170: 0 → 1
Char. 211: 0 → 1
Char. 215: 0 → 1
Char. 224: 0 → 1
Char. 239: 0 → 1

**Node 50:**
Char. 46: 1 → 0
Char. 88: 1 → 0
Char. 89: 1 → 0
Char. 93: 1 → 0
Char. 176: 0 → 1
Char. 195: 0 → 2
Char. 198: 0 → 1

Char. 246: 1 → 2
Char. 259: 0 → 1
Char. 265: 1 → 0
Char. 270: 0 → 1

**Node 51:**
Char. 65: 0 → 1
Char. 131: 0 → 1
Char. 184: 0 → 1
Char. 205: 0 → 1
Char. 210: 0 → 1
Char. 219: 1 → 0
Char. 241: 0 → 1
Char. 246: 0 → 1
Char. 254: 0 → 1
Char. 255: 0 → 1
Char. 256: 0 → 1
Char. 268: 0 → 1
Char. 269: 0 → 1

**Node 52:**
Char. 0: 1 → 2
Char. 15: 0 → 1
Char. 33: 1 → 2
Char. 44: 1 → 0
Char. 62: 1 → 0
Char. 64: 0 → 1
Char. 84: 1 → 2
Char. 130: 0 → 1
Char. 134: 0 → 2
Char. 147: 0 → 1
Char. 152: 0 → 1
Char. 155: 0 → 1
Char. 158: 0 → 1
Char. 161: 0 → 1
Char. 174: 0 → 1
Char. 181: 0 → 1
Char. 203: 1 → 2
Char. 204: 0 → 2
Char. 247: 0 → 1
Char. 251: 0 → 1
Char. 252: 0 → 1
Char. 253: 0 → 2

**Node 53:**
Char. 0: 0 → 1
Char. 5: 0 → 1
Char. 59: 0 → 1
Char. 60: 0 → 1
Char. 62: 0 → 1
Char. 72: 1 → 2
Char. 92: 0 → 1
Char. 265: 0 → 1
Char. 275: 1 → 0
Char. 278: 3 → 0

**Node 54:**
Char. 20: 0 → 1
Char. 25: 1 → 0
Char. 29: 1 → 0
Char. 33: 0 → 1
Char. 50: 1 → 0
Char. 57: 1 → 0
Char. 67: 0 → 1
Char. 89: 0 → 1
Char. 94: 0 → 1
Char. 127: 0 → 1

**Node 55:**
Char. 23: 0 → 1
Char. 132: 0 → 1
Char. 166: 0 → 1
Char. 276: 1 → 0

**Node 56:**
Char. 29: 0 → 1
Char. 84: 0 → 1
Char. 88: 0 → 1
Char. 169: 0 → 1

**Node 57:**
Char. 72: 0 → 1
Char. 81: 0 → 1
Char. 93: 0 → 1
Char. 97: 0 → 1
Char. 104: 1 → 0
Char. 144: 0 → 1
Char. 173: 0 → 1
Char. 183: 0 → 1

**Node 59:**
Char. 95: 0 → 1
Char. 113: 0 → 1
Char. 114: 0 → 1

**Node 60:**
Char. 5: 0 → 1
Char. 20: 0 → 1
Char. 23: 1 → 0
Char. 47: 0 → 1
Char. 79: 0 → 1
Char. 110: 0 → 1
Char. 131: 0 → 1
Char. 137: 0 → 1
Char. 140: 0 → 2
Char. 147: 0 → 1

**Node 61:**
Char. 19: 0 → 1
Char. 92: 1 → 0

**Node 62:**
Char. 4: 0 → 1
Char. 15: 0 → 1
Char. 29: 0 → 1
Char. 213: 0 → 1
Char. 226: 0 → 2
Char. 228: 0 → 1
Char. 275: 0 → 1

**Node 63:**
Char. 58: 0 → 1
Char. 61: 0 → 1
Char. 66: 0 → 1
Char. 69: 0 → 1
Char. 107: 0 → 1
Char. 126: 0 → 1
Char. 131: 0 → 1
Char. 134: 0 → 1
Char. 140: 0 → 1
Char. 147: 0 → 1
Char. 150: 0 → 1
Char. 167: 0 → 1
Char. 176: 0 → 1
Char. 190: 0 → 1
Char. 205: 0 → 1
Char. 208: 0 → 1
Char. 230: 0 → 1
Char. 239: 0 → 1
Char. 260: 2 → 1

**Node 64:**
Char. 73: 0 → 1
Char. 205: 0 → 1
Char. 273: 0 → 1
Char. 276: 0 → 1

**Node 65:**
Char. 18: 0 → 1
Char. 25: 1 → 0
Char. 37: 0 → 1
Char. 103: 0 → 1
Char. 106: 0 → 1
Char. 107: 0 → 1
Char. 126: 0 → 1
Char. 150: 0 → 1

**Node 66:**
Char. 5: 0 → 1
Char. 20: 0 → 1
Char. 33: 0 → 1
Char. 38: 1 → 2
Char. 39: 0 → 1
Char. 44: 1 → 0
Char. 49: 0 → 1
Char. 66: 0 → 1
Char. 76: 0 → 1
Char. 80: 1 → 0
Char. 95: 0 → 1
Char. 135: 0 → 1
Char. 137: 0 → 1
Char. 147: 0 → 1
Char. 157: 0 → 1
Char. 158: 0 → 1
Char. 183: 1 → 2
Char. 186: 0 → 1
Char. 187: 0 → 1
Char. 194: 0 → 1
Char. 197: 0 → 1
Char. 201: 1 → 0
Char. 211: 0 → 1
Char. 252: 0 → 1

**Node 68:**
Char. 133: 0 → 1

**Node 69:**
Char. 100: 0 → 1
Char. 113: 0 → 1

**Node 70:**
Char. 23: 1 → 0
Char. 70: 0 → 1
Char. 118: 0 → 1
Char. 125: 0 → 1

**Node 72:**
Char. 5: 0 → 1
Char. 38: 1 → 2
Char. 39: 0 → 1
Char. 50: 1 → 0
Char. 58: 0 → 1
Char. 59: 0 → 1
Char. 60: 0 → 1
Char. 72: 1 → 2
Char. 85: 1 → 0
Char. 88: 1 → 0
Char. 95: 0 → 1
Char. 104: 0 → 1
Char. 105: 0 → 1
Char. 106: 0 → 2
Char. 107: 0 → 1
Char. 109: 0 → 1
Char. 110: 0 → 1
Char. 146: 1 → 0
Char. 155: 0 → 2
Char. 183: 1 → 2

**Node 73:**
Char. 23: 0 → 1
Char. 33: 1 → 0
Char. 38: 2 → 1
Char. 39: 1 → 0
Char. 42: 0 → 1
Char. 43: 0 → 1
Char. 46: 1 → 0
Char. 49: 1 → 0
Char. 71: 1 → 0
Char. 83: 0 → 2
Char. 101: 0 → 1
Char. 103: 0 → 1
Char. 106: 0 → 1
Char. 161: 0 → 1
Char. 163: 1 → 0
Char. 172: 0 → 2
Char. 174: 0 → 1
Char. 188: 0 → 1
Char. 203: 1 → 2
Char. 212: 0 → 1
Char. 216: 1 → 0
Char. 236: 0 → 1
Char. 238: 0 → 2
Char. 241: 0 → 1
Char. 242: 0 → 1
Char. 273: 0 → 1
Char. 274: 0 → 1
Char. 275: 1 → 0

**Node 74:**
Char. 52: 0 → 1
Char. 75: 0 → 1
Char. 84: 1 → 0
Char. 121: 0 → 1
Char. 180: 1 → 0
Char. 230: 0 → 1

**Node 75:**
Char. 29: 1 → 0
Char. 44: 1 → 0
Char. 59: 0 → 1
Char. 60: 0 → 1
Char. 66: 0 → 1
Char. 67: 0 → 1
Char. 72: 1 → 2
Char. 78: 1 → 0
Char. 84: 1 → 0
Char. 88: 1 → 0
Char. 93: 1 → 0
Char. 111: 0 → 1
Char. 123: 1 → 0
Char. 129: 0 → 1
Char. 154: 1 → 0
Char. 169: 1 → 0
Char. 170: 0 → 1
Char. 192: 1 → 0
Char. 197: 0 → 1

**Node 76:**
Char. 85: 1 → 0
Char. 107: 0 → 1

**Node 77:**
Char. 2: 0 → 1
Char. 6: 0 → 1
Char. 186: 0 → 1
Char. 198: 0 → 1
Char. 200: 0 → 1
Char. 220: 0 → 1
Char. 239: 1 → 0

**Node 78:**
Char. 98: 1 → 0
Char. 113: 0 → 1
Char. 181: 0 → 1
Char. 206: 0 → 2

**Node 79:**
Char. 35: 0 → 1
Char. 102: 0 → 1
Char. 103: 0 → 1
Char. 184: 0 → 1
Char. 201: 0 → 1
Char. 210: 0 → 1
Char. 223: 0 → 1
Char. 229: 0 → 1

**Node 80:**
Char. 17: 0 → 1
Char. 42: 0 → 1
Char. 251: 0 → 1
Char. 264: 0 → 1

**Node 81:**
Char. 41: 0 → 1
Char. 61: 1 → 2
Char. 112: 1 → 0
Char. 138: 0 → 1
Char. 146: 1 → 0
Char. 155: 0 → 1
Char. 192: 1 → 2
Char. 224: 0 → 1

Char. 226: 0 → 1
Char. 227: 0 → 1
Char. 233: 0 → 1
Char. 267: 01 → 2

**Node 82:**
Char. 61: 1 → 3
Char. 90: 0 → 1
Char. 91: 0 → 1

Char. 109: 1 → 0
Char. 155: 0 → 1
Char. 209: 1 → 0

**Analysis 42**
**(all taxa, implied weighting, K = 3.25)**

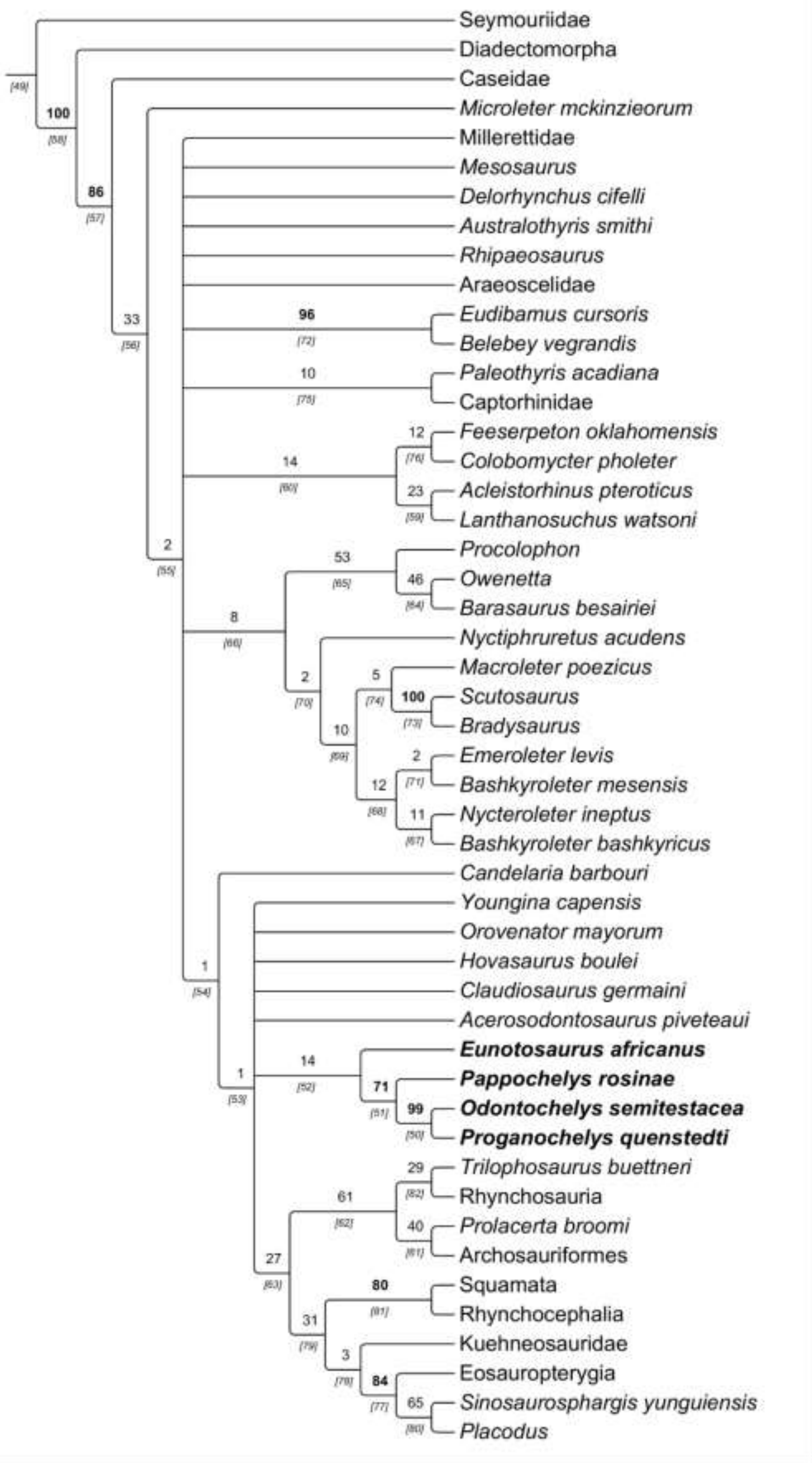

***Proganochelys quenstedti*:**
Char. 8: 0 → 1
Char. 11: 0 → 1
Char. 106: 0 → 1
Char. 108: 1 → 0
Char. 128: 0 → 1
Char. 175: 0 → 1
Char. 202: 1 → 0
Char. 207: 1 → 0
Char. 209: 1 → 0
Char. 244: 0 → 1
Char. 248: 0 → 1
Char. 250: 0 → 1
Char. 252: 1 → 0
Char. 262: 0 → 1

***Pappochelys rosinae*:**
Char. 0: 2 → 0
Char. 1: 0 → 1
Char. 5: 1 → 0
Char. 12: 0 → 1
Char. 41: 0 → 1
Char. 49: 0 → 1
Char. 75: 0 → 1
Char. 169: 1 → 0
Char. 260: 2 → 0

***Eunotosaurus africanus*:**
Char. 19: 0 → 1
Char. 59: 1 → 0
Char. 60: 1 → 0
Char. 72: 2 → 1
Char. 76: 0 → 1
Char. 97: 1 → 0
Char. 103: 0 → 1
Char. 153: 1 → 0
Char. 191: 1 → 0
Char. 192: 1 → 0
Char. 202: 1 → 0
Char. 211: 0 → 1
Char. 222: 1 → 0
Char. 237: 1 → 0
Char. 248: 0 → 1
Char. 249: 0 → 2
Char. 250: 0 → 1
Char. 263: 0 → 1
Char. 273: 0 → 1
Char. 274: 0 → 1
Char. 275: 0 → 1
Char. 276: 0 → 1
Char. 277: 0 → 1
Char. 278: 0 → 3

***Acerosodontosaurus piveteaui*:**
Char. 78: 1 → 0
Char. 81: 1 → 0
Char. 127: 1 → 0
Char. 128: 0 → 1
Char. 155: 0 → 1
Char. 206: 0 → 2
Char. 208: 0 → 1
Char. 278: 0 → 1

***Acleistorhinus pteroticus*:**
Char. 21: 0 → 1
Char. 146: 1 → 0

**Araeoscelidae:**
Char. 0: 0 → 1
Char. 27: 0 → 1
Char. 28: 0 → 1
Char. 29: 1 → 0
Char. 38: 1 → 0
Char. 40: 2 → 0
Char. 43: 0 → 1
Char. 50: 1 → 0
Char. 57: 1 → 0
Char. 59: 0 → 1
Char. 60: 0 → 1
Char. 67: 0 → 1
Char. 72: 1 → 2
Char. 84: 1 → 0
Char. 89: 0 → 1
Char. 106: 0 → 1
Char. 111: 0 → 1
Char. 116: 1 → 0
Char. 117: 01 → 2
Char. 120: 0 → 1
Char. 154: 1 → 0
Char. 166: 1 → 0
Char. 169: 1 → 0
Char. 170: 0 → 1
Char. 193: 0 → 1
Char. 197: 0 → 1
Char. 222: 0 → 1
Char. 224: 0 → 1
Char. 234: 0 → 1
Char. 237: 0 → 1
Char. 239: 0 → 1
Char. 266: 0 → 1
Char. 278: 3 → 1

**Archosauriformes:**
Char. 32: 0 → 1
Char. 94: 1 → 0
Char. 112: 1 → 0
Char. 152: 0 → 1
Char. 154: 01 → 2
Char. 166: 1 → 0
Char. 171: 0 → 1
Char. 185: 0 → 1
Char. 201: 0 → 1
Char. 204: 0 → 1
Char. 218: 0 → 3
Char. 224: 0 → 1
Char. 242: 0 → 1

***Australothyris smithi*:**
Char. 24: 0 → 1
Char. 34: 0 → 1
Char. 55: 1 → 0
Char. 57: 1 → 0
Char. 71: 1 → 0
Char. 79: 0 → 1
Char. 85: 1 → 0
Char. 98: 1 → 0
Char. 100: 0 → 1
Char. 103: 0 → 1
Char. 110: 0 → 1
Char. 123: 1 → 0
Char. 129: 0 → 1
Char. 131: 0 → 1
Char. 132: 1 → 0
Char. 144: 1 → 0
Char. 147: 0 → 1
Char. 149: 1 → 0
Char. 150: 0 → 1
Char. 192: 1 → 0

***Barasaurus besairiei*:**
Char. 33: 1 → 0

***Bashkyroleter bashkyricus*:**
Char. 275: 1 → 0

***Bashkyroleter mesensis*:**
Char. 12: 0 → 1
Char. 66: 1 → 0
Char. 169: 1 → 0

***Belebey vegrandis*:**
Char. 154: 1 → 0

***Bradysaurus* spp.:**
Char. 19: 0 → 1
Char. 25: 1 → 0
Char. 73: 0 → 1
Char. 79: 0 → 1
Char. 135: 1 → 0
Char. 249: 0 → 1

***Candelaria barbouri*:**
Char. 1: 0 → 1
Char. 8: 0 → 1
Char. 15: 0 → 1
Char. 33: 1 → 2
Char. 49: 0 → 1
Char. 55: 1 → 0
Char. 76: 0 → 1
Char. 79: 0 → 2
Char. 88: 1 → 0
Char. 95: 0 → 1
Char. 126: 0 → 1
Char. 132: 1 → 0
Char. 154: 1 → 2
Char. 169: 1 → 0
Char. 276: 0 → 1
Char. 277: 0 → 1

**Captorhinidae:**
Char. 3: 0 → 1
Char. 25: 1 → 0
Char. 26: 0 → 1
Char. 73: 0 → 1
Char. 75: 0 → 1
Char. 108: 1 → 0
Char. 180: 1 → 0
Char. 183: 1 → 0
Char. 201: 1 → 0
Char. 203: 1 → 2
Char. 216: 1 → 0

**Caseidae:**
Char. 38: 1 → 0
Char. 46: 1 → 0
Char. 50: 1 → 0
Char. 170: 0 → 1
Char. 194: 0 → 1
Char. 273: 0 → 1
Char. 274: 0 → 1
Char. 278: 3 → 2

***Claudiosaurus germaini*:**
Char. 24: 0 → 1
Char. 27: 0 → 1
Char. 34: 0 → 1
Char. 36: 0 → 1
Char. 56: 0 → 1
Char. 64: 0 → 1
Char. 70: 0 → 1
Char. 84: 1 → 0
Char. 105: 0 → 1
Char. 106: 0 → 1
Char. 117: 01 → 2
Char. 126: 0 → 1
Char. 127: 1 → 0
Char. 130: 0 → 1
Char. 131: 0 → 1
Char. 141: 1 → 0
Char. 144: 1 → 0
Char. 166: 1 → 0
Char. 182: 0 → 1
Char. 187: 1 → 0
Char. 190: 0 → 1
Char. 199: 0 → 1
Char. 201: 0 → 1
Char. 203: 1 → 2
Char. 204: 0 → 1
Char. 222: 1 → 0
Char. 234: 1 → 0
Char. 272: 2 → 1

***Colobomycter pholeter*:**
Char. 21: 0 → 1
Char. 25: 1 → 0
Char. 84: 1 → 0
Char. 154: 1 → 0
Char. 167: 0 → 1
Char. 267: 0 → 1

***Delorhynchus cifelli*:**
Char. 5: 0 → 1
Char. 18: 0 → 1
Char. 20: 0 → 1
Char. 21: 0 → 1
Char. 23: 1 → 0
Char. 24: 0 → 1
Char. 26: 0 → 1
Char. 28: 0 → 1
Char. 33: 0 → 2
Char. 39: 0 → 1
Char. 52: 0 → 1
Char. 100: 0 → 1
Char. 111: 0 → 1
Char. 116: 1 → 0
Char. 119: 1 → 0
Char. 131: 0 → 1
Char. 147: 0 → 1
Char. 156: 1 → 0
Char. 167: 0 → 1
Char. 189: 0 → 1
Char. 191: 0 → 1
Char. 204: 0 → 2
Char. 267: 0 → 1

**Diadectomorpha:**
Char. 0: 0 → 1
Char. 64: 0 → 1
Char. 70: 0 → 1
Char. 122: 0 → 1
Char. 123: 1 → 0
Char. 146: 1 → 0
Char. 275: 1 → 0
Char. 278: 3 → 0

***Emeroleter levis*:**
Char. 51: 1 → 0

**Eosauropterygia:**
Char. 166: 1 → 0
Char. 174: 0 → 1
Char. 178: 0 → 1
Char. 194: 0 → 2
Char. 272: 1 → 0

***Eudibamus cursoris*:**
Char. 154: 1 → 2

***Feeserpeton oklahomensis*:**
Char. 51: 0 → 1
Char. 157: 0 → 1
Char. 158: 0 → 1

***Hovasaurus boulei*:**
Char. 41: 0 → 1
Char. 55: 1 → 0
Char. 60: 1 → 0
Char. 72: 2 → 1
Char. 77: 0 → 2
Char. 78: 1 → 0
Char. 79: 0 → 1
Char. 93: 1 → 0
Char. 113: 0 → 1
Char. 138: 0 → 1
Char. 141: 1 → 0
Char. 146: 1 → 0
Char. 204: 0 → 2
Char. 206: 0 → 2
Char. 215: 0 → 1
Char. 219: 1 → 0
Char. 224: 0 → 1
Char. 278: 0 → 3

**Kuehneosauridae:**
Char. 7: 0 → 1
Char. 24: 0 → 1
Char. 27: 0 → 1
Char. 43: 1 → 0
Char. 79: 0 → 2
Char. 107: 1 → 0
Char. 140: 1 → 0

Char. 147: 1 → 0
Char. 148: 1 → 0
Char. 245: 0 → 1
Char. 278: 0 → 3

***Lanthanosuchus watsoni*:**
Char. 25: 1 → 0
Char. 51: 0 → 1
Char. 76: 0 → 1
Char. 86: 0 → 1
Char. 98: 1 → 0
Char. 138: 0 → 1
Char. 144: 1 → 0
Char. 154: 1 → 2

***Macroleter poezicus*:**
Char. 9: 0 → 1
Char. 26: 0 → 1
Char. 66: 1 → 2
Char. 126: 0 → 1
Char. 134: 0 → 2
Char. 140: 0 → 1
Char. 146: 1 → 0
Char. 169: 1 → 0

***Mesosaurus* spp.:**
Char. 0: 0 → 1
Char. 2: 0 → 1
Char. 6: 0 → 1
Char. 8: 0 → 1
Char. 9: 0 → 1
Char. 13: 0 → 1
Char. 19: 0 → 1
Char. 26: 0 → 1
Char. 29: 1 → 0
Char. 33: 0 → 1
Char. 38: 1 → 0
Char. 41: 0 → 1
Char. 50: 1 → 0
Char. 67: 0 → 1
Char. 76: 0 → 1
Char. 84: 1 → 0
Char. 85: 1 → 0
Char. 94: 0 → 1
Char. 107: 0 → 1
Char. 109: 0 → 1
Char. 111: 0 → 1
Char. 115: 0 → 1
Char. 146: 1 → 0
Char. 149: 1 → 0
Char. 164: 0 → 1
Char. 166: 1 → 0
Char. 167: 0 → 1
Char. 176: 0 → 1
Char. 183: 1 → 0
Char. 184: 0 → 1
Char. 192: 1 → 0
Char. 199: 0 → 1
Char. 202: 0 → 1
Char. 204: 0 → 2
Char. 206: 0 → 1
Char. 207: 0 → 1
Char. 209: 0 → 1
Char. 217: 0 → 1
Char. 219: 0 → 1
Char. 220: 0 → 1
Char. 231: 0 → 1
Char. 260: 2 → 0
Char. 272: 2 → 0
Char. 278: 3 → 0

***Microleter mckinzieorum*:**
Char. 0: 0 → 1
Char. 18: 0 → 1
Char. 39: 0 → 1
Char. 57: 1 → 0
Char. 70: 0 → 1
Char. 76: 0 → 1
Char. 94: 0 → 1
Char. 106: 0 → 1
Char. 110: 0 → 1
Char. 278: 3 → 1

**Millerettidae:**
Char. 23: 1 → 0
Char. 24: 0 → 1
Char. 25: 1 → 0
Char. 44: 1 → 0
Char. 56: 0 → 1
Char. 57: 1 → 0
Char. 66: 0 → 2
Char. 78: 1 → 0
Char. 80: 1 → 0
Char. 84: 1 → 2
Char. 88: 1 → 0
Char. 96: 1 → 0
Char. 121: 0 → 1
Char. 124: 0 → 1
Char. 127: 0 → 1
Char. 135: 0 → 1
Char. 145: 0 → 1
Char. 166: 1 → 0
Char. 180: 1 → 0
Char. 192: 1 → 0
Char. 202: 0 → 1
Char. 211: 0 → 1
Char. 230: 0 → 1
Char. 234: 0 → 1
Char. 248: 0 → 1
Char. 252: 0 → 1
Char. 253: 0 → 1

***Nycteroleter ineptus*:**
Char. 66: 1 → 0

***Nyctiphruretus acudens*:**
Char. 21: 0 → 1
Char. 41: 0 → 1
Char. 66: 1 → 2
Char. 81: 1 → 0
Char. 84: 1 → 2
Char. 94: 0 → 1
Char. 166: 1 → 0
Char. 224: 0 → 1
Char. 266: 0 → 1
Char. 272: 2 → 1
Char. 276: 0 → 1

***Orovenator mayorum*:**
Char. 8: 0 → 1
Char. 23: 1 → 0
Char. 24: 0 → 1
Char. 33: 1 → 0
Char. 36: 0 → 1
Char. 50: 0 → 1
Char. 92: 1 → 0
Char. 94: 1 → 0
Char. 135: 1 → 0
Char. 159: 1 → 0
Char. 160: 0 → 1
Char. 165: 0 → 1
Char. 278: 0 → 1

***Owenetta* spp.:**
Char. 142: 1 → 0
Char. 169: 1 → 0

***Paleothyris acadiana*:**
Char. 23: 1 → 0
Char. 38: 1 → 0
Char. 50: 1 → 0
Char. 66: 1 → 2
Char. 102: 0 → 1
Char. 146: 1 → 0
Char. 237: 0 → 1
Char. 239: 0 → 1

***Placodus* spp.:**
Char. 0: 1 → 2
Char. 9: 0 → 1
Char. 12: 0 → 1
Char. 13: 0 → 1
Char. 19: 0 → 1
Char. 31: 0 → 1
Char. 46: 1 → 0
Char. 57: 0 → 1
Char. 78: 1 → 0
Char. 93: 1 → 0
Char. 102: 1 → 2
Char. 109: 1 → 0
Char. 140: 1 → 0
Char. 155: 0 → 1
Char. 163: 1 → 0
Char. 164: 0 → 1

***Procolophon* spp.:**
Char. 41: 0 → 1
Char. 69: 0 → 1
Char. 79: 0 → 1
Char. 86: 0 → 1
Char. 101: 0 → 1
Char. 141: 0 → 1
Char. 149: 1 → 0
Char. 203: 1 → 2
Char. 204: 0 → 1
Char. 215: 0 → 1
Char. 230: 0 → 1
Char. 235: 1 → 2
Char. 237: 0 → 1
Char. 238: 0 → 1
Char. 272: 2 → 1
Char. 278: 3 → 0

***Prolacerta broomi*:**
Char. 58: 1 → 0
Char. 66: 1 → 0
Char. 67: 1 → 0
Char. 80: 1 → 0
Char. 139: 1 → 0
Char. 147: 1 → 0
Char. 192: 1 → 0
Char. 203: 1 → 2
Char. 206: 0 → 12

***Rhipaeosaurus* spp.:**
Char. 172: 0 → 1
Char. 183: 1 → 2
Char. 194: 0 → 1
Char. 201: 1 → 0
Char. 211: 0 → 1
Char. 215: 0 → 1
Char. 226: 0 → 1
Char. 235: 1 → 0
Char. 252: 0 → 1
Char. 277: 0 → 1

**Rhynchocephalia:**
Char. 0: 1 → 2
Char. 23: 1 → 0
Char. 24: 0 → 1
Char. 77: 0 → 1
Char. 88: 1 → 0
Char. 94: 1 → 0
Char. 139: 1 → 0
Char. 167: 1 → 0
Char. 205: 1 → 0

**Rhynchosauria:**
Char. 0: 1 → 0
Char. 7: 0 → 1
Char. 9: 0 → 1
Char. 26: 0 → 1
Char. 44: 1 → 0
Char. 68: 0 → 1
Char. 99: 1 → 0
Char. 150: 1 → 0
Char. 160: 0 → 1
Char. 161: 0 → 1
Char. 171: 0 → 2
Char. 223: 0 → 1
Char. 224: 0 → 1
Char. 241: 0 → 1

***Scutosaurus* spp.:**
Char. 175: 0 → 1
Char. 190: 0 → 1
Char. 218: 0 → 1
Char. 243: 0 → 2
Char. 244: 0 → 1
Char. 251: 0 → 1

***Sinosaurosphargis yunguiensis*:**
Char. 8: 0 → 1
Char. 30: 0 → 1
Char. 53: 0 → 1
Char. 89: 1 → 0
Char. 127: 1 → 0
Char. 150: 1 → 0
Char. 154: 0 → 2
Char. 167: 1 → 0
Char. 253: 0 → 1
Char. 255: 0 → 1

**Squamata:**
Char. 45: 0 → 1
Char. 79: 0 → 2
Char. 80: 1 → 0
Char. 92: 1 → 0
Char. 109: 1 → 0
Char. 160: 0 → 1
Char. 200: 0 → 1
Char. 245: 0 → 1

***Trilophosaurus buettneri*:**
Char. 5: 1 → 0
Char. 11: 0 → 1
Char. 55: 1 → 0
Char. 93: 1 → 0
Char. 104: 0 → 1
Char. 106: 0 → 1
Char. 113: 0 → 1
Char. 122: 0 → 1
Char. 136: 1 → 0
Char. 144: 1 → 0
Char. 157: 0 → 1
Char. 159: 1 → 0
Char. 177: 0 → 12
Char. 194: 0 → 1
Char. 203: 1 → 2
Char. 207: 1 → 0
Char. 208: 1 → 0
Char. 272: 1 → 0

***Youngina capensis*:**
Char. 5: 1 → 0
Char. 23: 1 → 0
Char. 25: 0 → 1
Char. 27: 0 → 1
Char. 38: 1 → 0
Char. 44: 1 → 0
Char. 56: 0 → 1
Char. 75: 0 → 1
Char. 84: 1 → 0
Char. 92: 1 → 0
Char. 94: 1 → 0
Char. 134: 0 → 1
Char. 163: 1 → 0
Char. 170: 0 → 1
Char. 211: 0 → 1
Char. 215: 0 → 1
Char. 224: 0 → 1
Char. 239: 0 → 1

**Node 50:**
Char. 46: 1 → 0
Char. 88: 1 → 0
Char. 89: 1 → 0
Char. 93: 1 → 0
Char. 176: 0 → 1
Char. 195: 0 → 2
Char. 198: 0 → 1

Char. 246: 1 → 2
Char. 259: 0 → 1
Char. 265: 1 → 0
Char. 270: 0 → 1

**Node 51:**
Char. 65: 0 → 1
Char. 131: 0 → 1
Char. 184: 0 → 1
Char. 205: 0 → 1
Char. 210: 0 → 1
Char. 219: 1 → 0
Char. 241: 0 → 1
Char. 246: 0 → 1
Char. 254: 0 → 1
Char. 255: 0 → 1
Char. 256: 0 → 1
Char. 268: 0 → 1
Char. 269: 0 → 1

**Node 52:**
Char. 0: 1 → 2
Char. 15: 0 → 1
Char. 33: 1 → 2
Char. 44: 1 → 0
Char. 62: 1 → 0
Char. 64: 0 → 1
Char. 84: 1 → 2
Char. 130: 0 → 1
Char. 134: 0 → 2
Char. 147: 0 → 1
Char. 152: 0 → 1
Char. 155: 0 → 1
Char. 158: 0 → 1
Char. 161: 0 → 1
Char. 174: 0 → 1
Char. 181: 0 → 1
Char. 203: 1 → 2
Char. 204: 0 → 2
Char. 247: 0 → 1
Char. 251: 0 → 1
Char. 252: 0 → 1
Char. 253: 0 → 2

**Node 53:**
Char. 0: 0 → 1
Char. 5: 0 → 1
Char. 59: 0 → 1
Char. 60: 0 → 1
Char. 62: 0 → 1
Char. 72: 1 → 2
Char. 92: 0 → 1
Char. 265: 0 → 1
Char. 275: 1 → 0
Char. 278: 3 → 0

**Node 54:**
Char. 20: 0 → 1
Char. 25: 1 → 0
Char. 29: 1 → 0
Char. 33: 0 → 1
Char. 50: 1 → 0
Char. 57: 1 → 0
Char. 67: 0 → 1
Char. 89: 0 → 1
Char. 94: 0 → 1
Char. 127: 0 → 1

**Node 55:**
Char. 23: 0 → 1
Char. 132: 0 → 1
Char. 166: 0 → 1
Char. 276: 1 → 0

**Node 56:**
Char. 29: 0 → 1
Char. 84: 0 → 1
Char. 88: 0 → 1
Char. 169: 0 → 1

**Node 57:**
Char. 72: 0 → 1
Char. 81: 0 → 1
Char. 93: 0 → 1
Char. 97: 0 → 1
Char. 104: 1 → 0
Char. 144: 0 → 1
Char. 173: 0 → 1
Char. 183: 0 → 1

**Node 59:**
Char. 95: 0 → 1
Char. 113: 0 → 1
Char. 114: 0 → 1

**Node 60:**
Char. 5: 0 → 1
Char. 20: 0 → 1
Char. 23: 1 → 0
Char. 47: 0 → 1
Char. 79: 0 → 1
Char. 110: 0 → 1
Char. 131: 0 → 1
Char. 137: 0 → 1
Char. 140: 0 → 2
Char. 147: 0 → 1

**Node 61:**
Char. 19: 0 → 1
Char. 92: 1 → 0

**Node 62:**
Char. 4: 0 → 1
Char. 15: 0 → 1
Char. 29: 0 → 1
Char. 213: 0 → 1
Char. 226: 0 → 2
Char. 228: 0 → 1
Char. 275: 0 → 1

**Node 63:**
Char. 58: 0 → 1
Char. 61: 0 → 1
Char. 66: 0 → 1
Char. 69: 0 → 1
Char. 107: 0 → 1
Char. 126: 0 → 1
Char. 131: 0 → 1
Char. 134: 0 → 1
Char. 140: 0 → 1
Char. 147: 0 → 1
Char. 150: 0 → 1
Char. 167: 0 → 1
Char. 176: 0 → 1
Char. 190: 0 → 1
Char. 205: 0 → 1
Char. 208: 0 → 1
Char. 230: 0 → 1
Char. 239: 0 → 1
Char. 260: 2 → 1

**Node 64:**
Char. 73: 0 → 1
Char. 205: 0 → 1
Char. 273: 0 → 1
Char. 276: 0 → 1

**Node 65:**
Char. 18: 0 → 1
Char. 25: 1 → 0
Char. 37: 0 → 1
Char. 103: 0 → 1
Char. 106: 0 → 1
Char. 107: 0 → 1
Char. 126: 0 → 1
Char. 150: 0 → 1

**Node 66:**
Char. 5: 0 → 1
Char. 20: 0 → 1
Char. 33: 0 → 1
Char. 38: 1 → 2
Char. 39: 0 → 1
Char. 44: 1 → 0
Char. 49: 0 → 1
Char. 66: 0 → 1
Char. 76: 0 → 1
Char. 80: 1 → 0
Char. 95: 0 → 1
Char. 135: 0 → 1
Char. 137: 0 → 1
Char. 147: 0 → 1
Char. 157: 0 → 1
Char. 158: 0 → 1
Char. 183: 1 → 2
Char. 186: 0 → 1
Char. 187: 0 → 1
Char. 194: 0 → 1
Char. 197: 0 → 1
Char. 201: 1 → 0
Char. 211: 0 → 1
Char. 252: 0 → 1

**Node 68:**
Char. 133: 0 → 1

**Node 69:**
Char. 100: 0 → 1
Char. 113: 0 → 1

**Node 70:**
Char. 23: 1 → 0
Char. 70: 0 → 1
Char. 118: 0 → 1
Char. 125: 0 → 1

**Node 72:**
Char. 5: 0 → 1
Char. 38: 1 → 2
Char. 39: 0 → 1
Char. 50: 1 → 0
Char. 58: 0 → 1
Char. 59: 0 → 1
Char. 60: 0 → 1
Char. 72: 1 → 2
Char. 85: 1 → 0
Char. 88: 1 → 0
Char. 95: 0 → 1
Char. 104: 0 → 1
Char. 105: 0 → 1
Char. 106: 0 → 2
Char. 107: 0 → 1
Char. 109: 0 → 1
Char. 110: 0 → 1
Char. 146: 1 → 0
Char. 155: 0 → 2
Char. 183: 1 → 2

**Node 73:**
Char. 23: 0 → 1
Char. 33: 1 → 0
Char. 38: 2 → 1
Char. 39: 1 → 0
Char. 42: 0 → 1
Char. 43: 0 → 1
Char. 46: 1 → 0
Char. 49: 1 → 0
Char. 71: 1 → 0
Char. 83: 0 → 2
Char. 101: 0 → 1
Char. 103: 0 → 1
Char. 106: 0 → 1
Char. 161: 0 → 1
Char. 163: 1 → 0
Char. 172: 0 → 2
Char. 174: 0 → 1
Char. 188: 0 → 1
Char. 203: 1 → 2
Char. 212: 0 → 1
Char. 216: 1 → 0
Char. 236: 0 → 1
Char. 238: 0 → 2
Char. 241: 0 → 1
Char. 242: 0 → 1
Char. 273: 0 → 1
Char. 274: 0 → 1
Char. 275: 1 → 0

**Node 74:**
Char. 52: 0 → 1
Char. 75: 0 → 1
Char. 84: 1 → 0
Char. 121: 0 → 1
Char. 180: 1 → 0
Char. 230: 0 → 1

**Node 75:**
Char. 29: 1 → 0
Char. 44: 1 → 0
Char. 59: 0 → 1
Char. 60: 0 → 1
Char. 66: 0 → 1
Char. 67: 0 → 1
Char. 72: 1 → 2
Char. 78: 1 → 0
Char. 84: 1 → 0
Char. 88: 1 → 0
Char. 93: 1 → 0
Char. 111: 0 → 1
Char. 123: 1 → 0
Char. 129: 0 → 1
Char. 154: 1 → 0
Char. 169: 1 → 0
Char. 170: 0 → 1
Char. 192: 1 → 0
Char. 197: 0 → 1

**Node 76:**
Char. 85: 1 → 0
Char. 107: 0 → 1

**Node 77:**
Char. 2: 0 → 1
Char. 6: 0 → 1
Char. 186: 0 → 1
Char. 198: 0 → 1
Char. 200: 0 → 1
Char. 220: 0 → 1
Char. 239: 1 → 0

**Node 78:**
Char. 98: 1 → 0
Char. 113: 0 → 1
Char. 181: 0 → 1
Char. 206: 0 → 2

**Node 79:**
Char. 35: 0 → 1
Char. 102: 0 → 1
Char. 103: 0 → 1
Char. 184: 0 → 1
Char. 201: 0 → 1
Char. 210: 0 → 1
Char. 223: 0 → 1
Char. 229: 0 → 1

**Node 80:**
Char. 17: 0 → 1
Char. 42: 0 → 1
Char. 251: 0 → 1
Char. 264: 0 → 1

**Node 81:**
Char. 41: 0 → 1
Char. 61: 1 → 2
Char. 112: 1 → 0
Char. 138: 0 → 1
Char. 146: 1 → 0
Char. 155: 0 → 1
Char. 192: 1 → 2
Char. 224: 0 → 1

Char. 226: 0 → 1
Char. 227: 0 → 1

Char. 233: 0 → 1
Char. 267: 01 → 2

**Node 82:**
Char. 61: 1 → 3
Char. 90: 0 → 1

Char. 91: 0 → 1
Char. 109: 1 → 0

**Analysis 43**
**(all taxa, implied weighting, K = 3.375)**

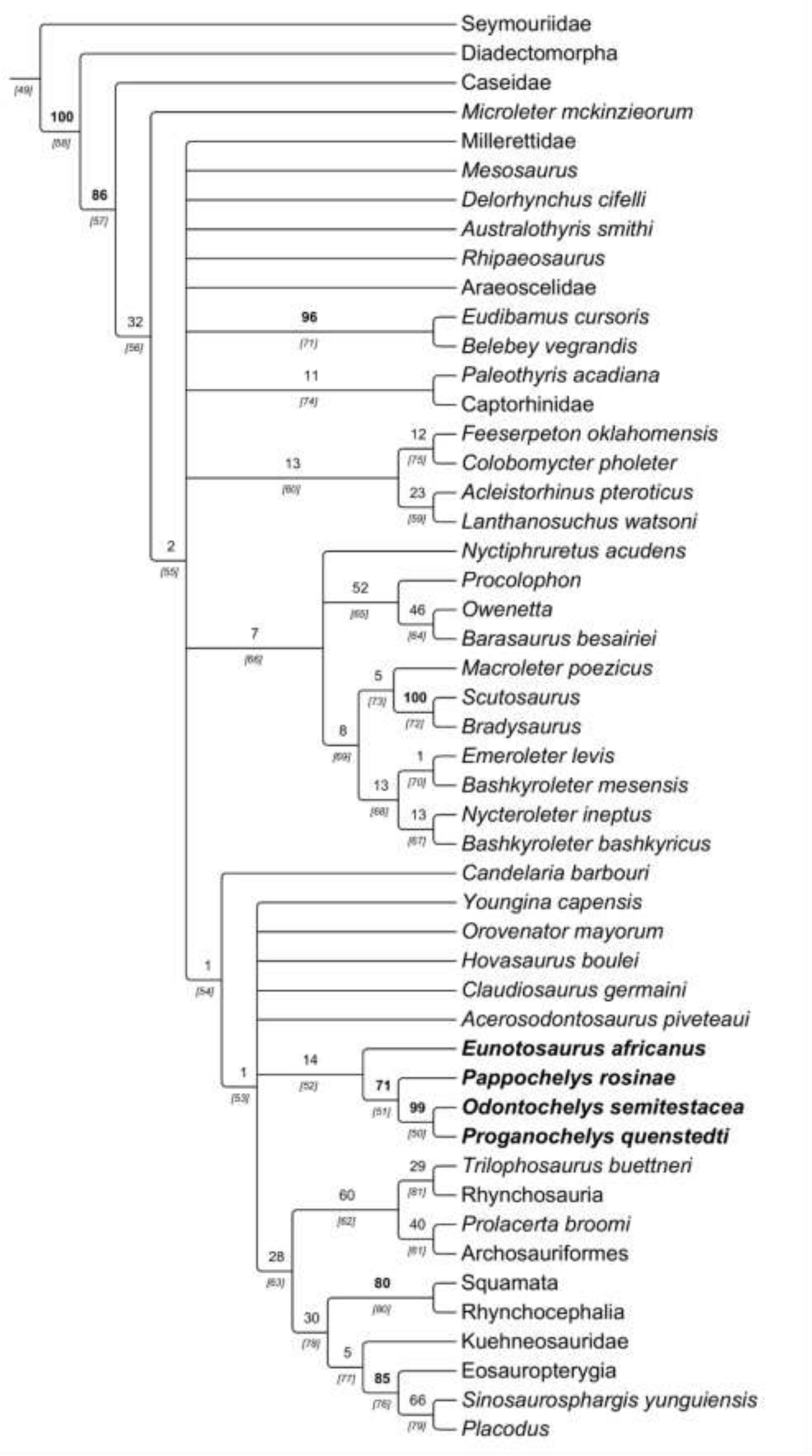

***Proganochelys quenstedti*:**
Char. 8: 0 → 1
Char. 11: 0 → 1
Char. 106: 0 → 1
Char. 108: 1 → 0
Char. 128: 0 → 1
Char. 175: 0 → 1
Char. 202: 1 → 0
Char. 207: 1 → 0
Char. 209: 1 → 0
Char. 244: 0 → 1
Char. 248: 0 → 1
Char. 250: 0 → 1
Char. 252: 1 → 0
Char. 262: 0 → 1

***Pappochelys rosinae*:**
Char. 0: 2 → 0
Char. 1: 0 → 1
Char. 5: 1 → 0
Char. 12: 0 → 1
Char. 41: 0 → 1
Char. 49: 0 → 1
Char. 75: 0 → 1
Char. 169: 1 → 0
Char. 260: 2 → 0

***Eunotosaurus africanus*:**
Char. 19: 0 → 1
Char. 59: 1 → 0
Char. 60: 1 → 0
Char. 72: 2 → 1
Char. 76: 0 → 1
Char. 97: 1 → 0
Char. 103: 0 → 1
Char. 153: 1 → 0
Char. 191: 1 → 0
Char. 192: 1 → 0
Char. 202: 1 → 0
Char. 211: 0 → 1
Char. 222: 1 → 0
Char. 237: 1 → 0
Char. 248: 0 → 1
Char. 249: 0 → 2
Char. 250: 0 → 1
Char. 263: 0 → 1
Char. 273: 0 → 1
Char. 274: 0 → 1
Char. 275: 0 → 1
Char. 276: 0 → 1
Char. 277: 0 → 1
Char. 278: 0 → 3

***Acerosodontosaurus piveteaui*:**
Char. 78: 1 → 0
Char. 81: 1 → 0
Char. 127: 1 → 0
Char. 128: 0 → 1
Char. 155: 0 → 1
Char. 206: 0 → 2
Char. 208: 0 → 1
Char. 278: 0 → 1

***Acleistorhinus pteroticus*:**
Char. 21: 0 → 1
Char. 146: 1 → 0

**Araeoscelidae:**
Char. 0: 0 → 1
Char. 27: 0 → 1
Char. 28: 0 → 1
Char. 29: 1 → 0
Char. 38: 1 → 0
Char. 40: 2 → 0
Char. 43: 0 → 1
Char. 50: 1 → 0
Char. 57: 1 → 0
Char. 59: 0 → 1
Char. 60: 0 → 1
Char. 67: 0 → 1
Char. 72: 1 → 2
Char. 84: 1 → 0
Char. 89: 0 → 1
Char. 106: 0 → 1
Char. 111: 0 → 1
Char. 116: 1 → 0
Char. 117: 01 → 2
Char. 120: 0 → 1
Char. 154: 1 → 0
Char. 166: 1 → 0
Char. 169: 1 → 0
Char. 170: 0 → 1
Char. 193: 0 → 1
Char. 197: 0 → 1
Char. 222: 0 → 1
Char. 224: 0 → 1
Char. 234: 0 → 1
Char. 237: 0 → 1
Char. 239: 0 → 1
Char. 266: 0 → 1
Char. 278: 3 → 1

**Archosauriformes:**
Char. 32: 0 → 1
Char. 94: 1 → 0
Char. 112: 1 → 0
Char. 152: 0 → 1
Char. 154: 01 → 2
Char. 166: 1 → 0
Char. 171: 0 → 1
Char. 185: 0 → 1
Char. 201: 0 → 1
Char. 204: 0 → 1
Char. 218: 0 → 3
Char. 224: 0 → 1
Char. 242: 0 → 1

***Australothyris smithi*:**
Char. 24: 0 → 1
Char. 34: 0 → 1
Char. 55: 1 → 0
Char. 57: 1 → 0
Char. 71: 1 → 0
Char. 79: 0 → 1
Char. 85: 1 → 0
Char. 98: 1 → 0
Char. 100: 0 → 1
Char. 103: 0 → 1
Char. 110: 0 → 1
Char. 123: 1 → 0
Char. 129: 0 → 1
Char. 131: 0 → 1
Char. 132: 1 → 0
Char. 144: 1 → 0
Char. 147: 0 → 1
Char. 149: 1 → 0
Char. 150: 0 → 1
Char. 192: 1 → 0

***Barasaurus besairiei*:**
Char. 33: 1 → 0

***Bashkyroleter bashkyricus*:**
Char. 275: 1 → 0

***Bashkyroleter mesensis*:**
Char. 12: 0 → 1
Char. 66: 1 → 0
Char. 169: 1 → 0

***Belebey vegrandis*:**
Char. 154: 1 → 0

***Bradysaurus* spp.:**
Char. 19: 0 → 1
Char. 25: 1 → 0
Char. 73: 0 → 1
Char. 79: 0 → 1
Char. 135: 1 → 0
Char. 249: 0 → 1

***Candelaria barbouri*:**
Char. 1: 0 → 1
Char. 8: 0 → 1
Char. 15: 0 → 1
Char. 33: 1 → 2
Char. 49: 0 → 1
Char. 55: 1 → 0
Char. 76: 0 → 1
Char. 79: 0 → 2
Char. 88: 1 → 0
Char. 95: 0 → 1
Char. 126: 0 → 1
Char. 132: 1 → 0
Char. 154: 1 → 2
Char. 169: 1 → 0
Char. 276: 0 → 1
Char. 277: 0 → 1

**Captorhinidae:**
Char. 3: 0 → 1
Char. 25: 1 → 0
Char. 26: 0 → 1
Char. 73: 0 → 1
Char. 75: 0 → 1
Char. 108: 1 → 0
Char. 180: 1 → 0
Char. 183: 1 → 0
Char. 201: 1 → 0
Char. 203: 1 → 2
Char. 216: 1 → 0

**Caseidae:**
Char. 38: 1 → 0
Char. 46: 1 → 0
Char. 50: 1 → 0
Char. 170: 0 → 1
Char. 194: 0 → 1
Char. 273: 0 → 1
Char. 274: 0 → 1
Char. 278: 3 → 2

***Claudiosaurus germaini*:**
Char. 24: 0 → 1
Char. 27: 0 → 1
Char. 34: 0 → 1
Char. 36: 0 → 1
Char. 56: 0 → 1
Char. 64: 0 → 1
Char. 70: 0 → 1
Char. 84: 1 → 0
Char. 105: 0 → 1
Char. 106: 0 → 1
Char. 117: 01 → 2
Char. 126: 0 → 1
Char. 127: 1 → 0
Char. 130: 0 → 1
Char. 131: 0 → 1
Char. 141: 1 → 0
Char. 144: 1 → 0
Char. 166: 1 → 0
Char. 182: 0 → 1
Char. 187: 1 → 0
Char. 190: 0 → 1
Char. 199: 0 → 1
Char. 201: 0 → 1
Char. 203: 1 → 2
Char. 204: 0 → 1
Char. 222: 1 → 0
Char. 234: 1 → 0
Char. 272: 2 → 1

***Colobomycter pholeter*:**
Char. 21: 0 → 1
Char. 25: 1 → 0
Char. 84: 1 → 0
Char. 154: 1 → 0
Char. 167: 0 → 1
Char. 267: 0 → 1

***Delorhynchus cifelli*:**
Char. 5: 0 → 1
Char. 18: 0 → 1
Char. 20: 0 → 1
Char. 21: 0 → 1
Char. 24: 0 → 1
Char. 26: 0 → 1
Char. 28: 0 → 1
Char. 33: 0 → 2
Char. 39: 0 → 1
Char. 52: 0 → 1
Char. 100: 0 → 1
Char. 111: 0 → 1
Char. 116: 1 → 0
Char. 119: 1 → 0
Char. 131: 0 → 1
Char. 147: 0 → 1
Char. 156: 1 → 0
Char. 167: 0 → 1
Char. 189: 0 → 1
Char. 191: 0 → 1
Char. 204: 0 → 2
Char. 267: 0 → 1

**Diadectomorpha:**
Char. 0: 0 → 1
Char. 64: 0 → 1
Char. 70: 0 → 1
Char. 122: 0 → 1
Char. 123: 1 → 0
Char. 146: 1 → 0
Char. 275: 1 → 0
Char. 278: 3 → 0

***Emeroleter levis*:**
Char. 51: 1 → 0

**Eosauropterygia:**
Char. 166: 1 → 0
Char. 174: 0 → 1
Char. 178: 0 → 1
Char. 194: 0 → 2
Char. 272: 1 → 0

***Eudibamus cursoris*:**
Char. 154: 1 → 2

***Feeserpeton oklahomensis*:**
Char. 51: 0 → 1
Char. 157: 0 → 1
Char. 158: 0 → 1

***Hovasaurus boulei*:**
Char. 41: 0 → 1
Char. 55: 1 → 0
Char. 60: 1 → 0
Char. 72: 2 → 1
Char. 77: 0 → 2
Char. 78: 1 → 0
Char. 79: 0 → 1
Char. 93: 1 → 0
Char. 113: 0 → 1
Char. 138: 0 → 1
Char. 141: 1 → 0
Char. 146: 1 → 0
Char. 204: 0 → 2
Char. 206: 0 → 2
Char. 215: 0 → 1
Char. 219: 1 → 0
Char. 224: 0 → 1
Char. 278: 0 → 3

**Kuehneosauridae:**
Char. 7: 0 → 1
Char. 24: 0 → 1
Char. 27: 0 → 1
Char. 43: 1 → 0
Char. 79: 0 → 2
Char. 107: 1 → 0
Char. 140: 1 → 0
Char. 147: 1 → 0

Char. 148: 1 → 0
Char. 245: 0 → 1
Char. 278: 0 → 3

***Lanthanosuchus watsoni*:**
Char. 25: 1 → 0
Char. 51: 0 → 1
Char. 76: 0 → 1
Char. 86: 0 → 1
Char. 98: 1 → 0
Char. 138: 0 → 1
Char. 144: 1 → 0
Char. 154: 1 → 2

***Macroleter poezicus*:**
Char. 9: 0 → 1
Char. 26: 0 → 1
Char. 66: 1 → 2
Char. 126: 0 → 1
Char. 134: 0 → 2
Char. 140: 0 → 1
Char. 146: 1 → 0
Char. 169: 1 → 0

***Mesosaurus* spp.:**
Char. 0: 0 → 1
Char. 2: 0 → 1
Char. 6: 0 → 1
Char. 8: 0 → 1
Char. 9: 0 → 1
Char. 13: 0 → 1
Char. 19: 0 → 1
Char. 26: 0 → 1
Char. 29: 1 → 0
Char. 33: 0 → 1
Char. 38: 1 → 0
Char. 41: 0 → 1
Char. 50: 1 → 0
Char. 67: 0 → 1
Char. 76: 0 → 1
Char. 84: 1 → 0
Char. 85: 1 → 0
Char. 94: 0 → 1
Char. 107: 0 → 1
Char. 109: 0 → 1
Char. 111: 0 → 1
Char. 115: 0 → 1
Char. 146: 1 → 0
Char. 149: 1 → 0
Char. 164: 0 → 1
Char. 166: 1 → 0
Char. 167: 0 → 1
Char. 176: 0 → 1
Char. 183: 1 → 0
Char. 184: 0 → 1
Char. 192: 1 → 0
Char. 199: 0 → 1
Char. 202: 0 → 1
Char. 204: 0 → 2
Char. 206: 0 → 1
Char. 207: 0 → 1
Char. 209: 0 → 1
Char. 217: 0 → 1
Char. 219: 0 → 1
Char. 220: 0 → 1
Char. 231: 0 → 1
Char. 260: 2 → 0
Char. 272: 2 → 0
Char. 278: 3 → 0

***Microleter mckinzieorum*:**
Char. 0: 0 → 1
Char. 18: 0 → 1
Char. 39: 0 → 1
Char. 57: 1 → 0
Char. 70: 0 → 1
Char. 76: 0 → 1
Char. 94: 0 → 1
Char. 106: 0 → 1
Char. 110: 0 → 1
Char. 278: 3 → 1

**Millerettidae:**
Char. 24: 0 → 1
Char. 25: 1 → 0
Char. 44: 1 → 0
Char. 56: 0 → 1
Char. 57: 1 → 0
Char. 66: 0 → 2
Char. 78: 1 → 0
Char. 80: 1 → 0
Char. 84: 1 → 2
Char. 88: 1 → 0
Char. 96: 1 → 0
Char. 121: 0 → 1
Char. 124: 0 → 1
Char. 127: 0 → 1
Char. 135: 0 → 1
Char. 145: 0 → 1
Char. 166: 1 → 0
Char. 180: 1 → 0
Char. 192: 1 → 0
Char. 202: 0 → 1
Char. 211: 0 → 1
Char. 230: 0 → 1
Char. 234: 0 → 1
Char. 248: 0 → 1
Char. 252: 0 → 1
Char. 253: 0 → 1

***Nycteroleter ineptus*:**
Char. 66: 1 → 0

***Nyctiphruretus acudens*:**
Char. 0: 0 → 1
Char. 21: 0 → 1
Char. 33: 1 → 2
Char. 41: 0 → 1
Char. 66: 1 → 2
Char. 81: 1 → 0
Char. 84: 1 → 2
Char. 94: 0 → 1
Char. 166: 1 → 0
Char. 224: 0 → 1
Char. 235: 1 → 0
Char. 266: 0 → 1
Char. 272: 2 → 1
Char. 276: 0 → 1

***Orovenator mayorum*:**
Char. 8: 0 → 1
Char. 24: 0 → 1
Char. 33: 1 → 0
Char. 36: 0 → 1
Char. 50: 0 → 1
Char. 92: 1 → 0
Char. 94: 1 → 0
Char. 135: 1 → 0
Char. 159: 1 → 0
Char. 160: 0 → 1
Char. 165: 0 → 1
Char. 278: 0 → 1

***Owenetta* spp.:**
Char. 142: 1 → 0
Char. 169: 1 → 0

***Paleothyris acadiana*:**
Char. 38: 1 → 0
Char. 50: 1 → 0
Char. 66: 1 → 2
Char. 102: 0 → 1
Char. 146: 1 → 0
Char. 237: 0 → 1
Char. 239: 0 → 1

***Placodus* spp.:**
Char. 0: 1 → 2
Char. 9: 0 → 1
Char. 12: 0 → 1
Char. 13: 0 → 1
Char. 19: 0 → 1
Char. 31: 0 → 1
Char. 46: 1 → 0
Char. 57: 0 → 1
Char. 78: 1 → 0
Char. 93: 1 → 0
Char. 102: 1 → 2
Char. 109: 1 → 0
Char. 140: 1 → 0
Char. 155: 0 → 1
Char. 163: 1 → 0
Char. 164: 0 → 1

***Procolophon* spp.:**
Char. 41: 0 → 1
Char. 69: 0 → 1
Char. 79: 0 → 1
Char. 86: 0 → 1
Char. 88: 0 → 1
Char. 101: 0 → 1
Char. 117: 1 → 0
Char. 141: 0 → 1
Char. 149: 1 → 0
Char. 203: 1 → 2
Char. 204: 0 → 1
Char. 215: 0 → 1
Char. 230: 0 → 1
Char. 235: 1 → 2
Char. 237: 0 → 1
Char. 238: 0 → 1
Char. 272: 2 → 1
Char. 278: 3 → 0

***Prolacerta broomi*:**
Char. 58: 1 → 0
Char. 66: 1 → 0
Char. 67: 1 → 0
Char. 80: 1 → 0
Char. 139: 1 → 0
Char. 147: 1 → 0
Char. 192: 1 → 0
Char. 203: 1 → 2
Char. 206: 0 → 12

***Rhipaeosaurus* spp.:**
Char. 172: 0 → 1
Char. 183: 1 → 2
Char. 194: 0 → 1
Char. 201: 1 → 0
Char. 211: 0 → 1
Char. 215: 0 → 1
Char. 226: 0 → 1
Char. 235: 1 → 0
Char. 252: 0 → 1
Char. 277: 0 → 1

**Rhynchocephalia:**
Char. 0: 1 → 2
Char. 24: 0 → 1
Char. 77: 0 → 1
Char. 88: 1 → 0
Char. 94: 1 → 0
Char. 139: 1 → 0
Char. 167: 1 → 0
Char. 205: 1 → 0

**Rhynchosauria:**
Char. 0: 1 → 0
Char. 7: 0 → 1
Char. 9: 0 → 1
Char. 26: 0 → 1
Char. 44: 1 → 0
Char. 68: 0 → 1
Char. 99: 1 → 0
Char. 150: 1 → 0
Char. 160: 0 → 1
Char. 161: 0 → 1
Char. 171: 0 → 2
Char. 223: 0 → 1
Char. 224: 0 → 1
Char. 241: 0 → 1

***Scutosaurus* spp.:**
Char. 175: 0 → 1
Char. 190: 0 → 1
Char. 218: 0 → 1
Char. 243: 0 → 2
Char. 244: 0 → 1
Char. 251: 0 → 1

***Sinosaurosphargis yunguiensis*:**
Char. 8: 0 → 1
Char. 30: 0 → 1
Char. 53: 0 → 1
Char. 89: 1 → 0
Char. 127: 1 → 0
Char. 150: 1 → 0
Char. 154: 0 → 2
Char. 167: 1 → 0
Char. 253: 0 → 1
Char. 255: 0 → 1

**Squamata:**
Char. 45: 0 → 1
Char. 79: 0 → 2
Char. 80: 1 → 0
Char. 92: 1 → 0
Char. 109: 1 → 0
Char. 160: 0 → 1
Char. 200: 0 → 1
Char. 245: 0 → 1

***Trilophosaurus buettneri*:**
Char. 5: 1 → 0
Char. 11: 0 → 1
Char. 55: 1 → 0
Char. 93: 1 → 0
Char. 104: 0 → 1
Char. 106: 0 → 1
Char. 113: 0 → 1
Char. 122: 0 → 1
Char. 136: 1 → 0
Char. 144: 1 → 0
Char. 157: 0 → 1
Char. 159: 1 → 0
Char. 177: 0 → 12
Char. 194: 0 → 1
Char. 203: 1 → 2
Char. 207: 1 → 0
Char. 208: 1 → 0
Char. 272: 1 → 0

***Youngina capensis*:**
Char. 5: 1 → 0
Char. 25: 0 → 1
Char. 27: 0 → 1
Char. 38: 1 → 0
Char. 44: 1 → 0
Char. 56: 0 → 1
Char. 75: 0 → 1
Char. 84: 1 → 0
Char. 92: 1 → 0
Char. 94: 1 → 0
Char. 134: 0 → 1
Char. 163: 1 → 0
Char. 170: 0 → 1
Char. 211: 0 → 1
Char. 215: 0 → 1
Char. 224: 0 → 1
Char. 239: 0 → 1

**Node 50:**
Char. 46: 1 → 0
Char. 88: 1 → 0
Char. 89: 1 → 0
Char. 93: 1 → 0
Char. 176: 0 → 1
Char. 195: 0 → 2
Char. 198: 0 → 1
Char. 246: 1 → 2
Char. 259: 0 → 1

Char. 265: 1 → 0
Char. 270: 0 → 1

**Node 51:**
Char. 65: 0 → 1
Char. 131: 0 → 1
Char. 184: 0 → 1
Char. 205: 0 → 1
Char. 210: 0 → 1
Char. 219: 1 → 0
Char. 241: 0 → 1
Char. 246: 0 → 1
Char. 254: 0 → 1
Char. 255: 0 → 1
Char. 256: 0 → 1
Char. 268: 0 → 1
Char. 269: 0 → 1

**Node 52:**
Char. 0: 1 → 2
Char. 15: 0 → 1
Char. 33: 1 → 2
Char. 44: 1 → 0
Char. 62: 1 → 0
Char. 64: 0 → 1
Char. 84: 1 → 2
Char. 130: 0 → 1
Char. 134: 0 → 2
Char. 147: 0 → 1
Char. 152: 0 → 1
Char. 155: 0 → 1
Char. 158: 0 → 1
Char. 161: 0 → 1
Char. 174: 0 → 1
Char. 181: 0 → 1
Char. 203: 1 → 2
Char. 204: 0 → 2
Char. 247: 0 → 1
Char. 251: 0 → 1
Char. 252: 0 → 1
Char. 253: 0 → 2

**Node 53:**
Char. 0: 0 → 1
Char. 5: 0 → 1
Char. 59: 0 → 1
Char. 60: 0 → 1
Char. 62: 0 → 1
Char. 72: 1 → 2
Char. 92: 0 → 1
Char. 265: 0 → 1
Char. 275: 1 → 0
Char. 278: 3 → 0

**Node 54:**
Char. 20: 0 → 1
Char. 25: 1 → 0
Char. 29: 1 → 0
Char. 33: 0 → 1
Char. 50: 1 → 0
Char. 57: 1 → 0
Char. 67: 0 → 1
Char. 89: 0 → 1
Char. 94: 0 → 1
Char. 127: 0 → 1

**Node 55:**
Char. 132: 0 → 1
Char. 166: 0 → 1
Char. 276: 1 → 0

**Node 56:**
Char. 29: 0 → 1
Char. 84: 0 → 1
Char. 88: 0 → 1
Char. 169: 0 → 1

**Node 57:**
Char. 72: 0 → 1
Char. 81: 0 → 1
Char. 93: 0 → 1
Char. 97: 0 → 1
Char. 104: 1 → 0
Char. 144: 0 → 1
Char. 173: 0 → 1
Char. 183: 0 → 1

**Node 59:**
Char. 95: 0 → 1
Char. 113: 0 → 1
Char. 114: 0 → 1

**Node 60:**
Char. 5: 0 → 1
Char. 20: 0 → 1
Char. 47: 0 → 1
Char. 79: 0 → 1
Char. 110: 0 → 1
Char. 131: 0 → 1
Char. 137: 0 → 1
Char. 140: 0 → 2
Char. 147: 0 → 1

**Node 61:**
Char. 19: 0 → 1
Char. 92: 1 → 0

**Node 62:**
Char. 4: 0 → 1
Char. 15: 0 → 1
Char. 29: 0 → 1
Char. 213: 0 → 1
Char. 226: 0 → 2
Char. 228: 0 → 1
Char. 275: 0 → 1

**Node 63:**
Char. 58: 0 → 1
Char. 61: 0 → 1
Char. 66: 0 → 1
Char. 69: 0 → 1
Char. 107: 0 → 1
Char. 126: 0 → 1
Char. 131: 0 → 1
Char. 134: 0 → 1
Char. 140: 0 → 1
Char. 147: 0 → 1
Char. 150: 0 → 1
Char. 167: 0 → 1
Char. 176: 0 → 1
Char. 190: 0 → 1
Char. 205: 0 → 1
Char. 208: 0 → 1
Char. 230: 0 → 1
Char. 239: 0 → 1
Char. 260: 2 → 1

**Node 64:**
Char. 73: 0 → 1
Char. 131: 1 → 0
Char. 205: 0 → 1
Char. 273: 0 → 1
Char. 276: 0 → 1

**Node 65:**
Char. 18: 0 → 1
Char. 25: 1 → 0
Char. 37: 0 → 1
Char. 103: 0 → 1
Char. 106: 0 → 1
Char. 107: 0 → 1
Char. 126: 0 → 1
Char. 150: 0 → 1

**Node 66:**
Char. 5: 0 → 1
Char. 20: 0 → 1
Char. 33: 0 → 1
Char. 38: 1 → 2
Char. 39: 0 → 1
Char. 44: 1 → 0
Char. 49: 0 → 1
Char. 51: 0 → 1
Char. 66: 0 → 1
Char. 76: 0 → 1
Char. 80: 1 → 0
Char. 88: 1 → 0
Char. 95: 0 → 1
Char. 131: 0 → 1
Char. 135: 0 → 1
Char. 137: 0 → 1
Char. 147: 0 → 1
Char. 157: 0 → 1
Char. 158: 0 → 1
Char. 183: 1 → 2
Char. 186: 0 → 1
Char. 187: 0 → 1
Char. 194: 0 → 1
Char. 197: 0 → 1
Char. 201: 1 → 0
Char. 211: 0 → 1
Char. 252: 0 → 1

**Node 68:**
Char. 133: 0 → 1

**Node 69:**
Char. 100: 0 → 1
Char. 113: 0 → 1

**Node 71:**
Char. 5: 0 → 1
Char. 38: 1 → 2
Char. 39: 0 → 1
Char. 50: 1 → 0
Char. 58: 0 → 1
Char. 59: 0 → 1
Char. 60: 0 → 1
Char. 72: 1 → 2
Char. 85: 1 → 0
Char. 88: 1 → 0
Char. 95: 0 → 1
Char. 104: 0 → 1
Char. 105: 0 → 1
Char. 106: 0 → 2
Char. 107: 0 → 1
Char. 109: 0 → 1
Char. 110: 0 → 1
Char. 146: 1 → 0
Char. 155: 0 → 2
Char. 183: 1 → 2

**Node 72:**
Char. 33: 1 → 0
Char. 38: 2 → 1
Char. 39: 1 → 0
Char. 42: 0 → 1
Char. 43: 0 → 1
Char. 46: 1 → 0
Char. 49: 1 → 0
Char. 71: 1 → 0
Char. 83: 0 → 2
Char. 101: 0 → 1
Char. 103: 0 → 1
Char. 106: 0 → 1
Char. 161: 0 → 1
Char. 163: 1 → 0
Char. 172: 0 → 2
Char. 174: 0 → 1
Char. 188: 0 → 1
Char. 203: 1 → 2
Char. 212: 0 → 1
Char. 216: 1 → 0
Char. 235: 1 → 2
Char. 236: 0 → 1
Char. 238: 0 → 2
Char. 241: 0 → 1
Char. 242: 0 → 1
Char. 273: 0 → 1
Char. 274: 0 → 1
Char. 275: 1 → 0

**Node 73:**
Char. 52: 0 → 1
Char. 75: 0 → 1
Char. 84: 1 → 0
Char. 121: 0 → 1
Char. 180: 1 → 0
Char. 230: 0 → 1

**Node 74:**
Char. 29: 1 → 0
Char. 44: 1 → 0
Char. 59: 0 → 1
Char. 60: 0 → 1
Char. 66: 0 → 1
Char. 67: 0 → 1
Char. 72: 1 → 2
Char. 78: 1 → 0
Char. 84: 1 → 0
Char. 88: 1 → 0
Char. 93: 1 → 0
Char. 111: 0 → 1
Char. 123: 1 → 0
Char. 129: 0 → 1
Char. 154: 1 → 0
Char. 169: 1 → 0
Char. 170: 0 → 1
Char. 192: 1 → 0
Char. 197: 0 → 1

**Node 75:**
Char. 85: 1 → 0
Char. 107: 0 → 1

**Node 76:**
Char. 2: 0 → 1
Char. 6: 0 → 1
Char. 186: 0 → 1
Char. 198: 0 → 1
Char. 200: 0 → 1
Char. 220: 0 → 1
Char. 239: 1 → 0

**Node 77:**
Char. 98: 1 → 0
Char. 113: 0 → 1
Char. 181: 0 → 1
Char. 206: 0 → 2

**Node 78:**
Char. 35: 0 → 1
Char. 102: 0 → 1
Char. 103: 0 → 1
Char. 184: 0 → 1
Char. 201: 0 → 1
Char. 210: 0 → 1
Char. 223: 0 → 1
Char. 229: 0 → 1

**Node 79:**
Char. 17: 0 → 1
Char. 42: 0 → 1
Char. 251: 0 → 1
Char. 264: 0 → 1

**Node 80:**
Char. 41: 0 → 1
Char. 61: 1 → 2
Char. 112: 1 → 0
Char. 138: 0 → 1
Char. 146: 1 → 0
Char. 155: 0 → 1
Char. 192: 1 → 2
Char. 224: 0 → 1
Char. 226: 0 → 1
Char. 227: 0 → 1
Char. 233: 0 → 1
Char. 267: 01 → 2

**Node 81:**
Char. 61: 1 → 3

Char. 90: 0 → 1
Char. 91: 0 → 1
Char. 109: 1 → 0
Char. 155: 0 → 1
Char. 209: 1 → 0

**Analysis 44**
**(all taxa, implied weighting, K = 3.5)**

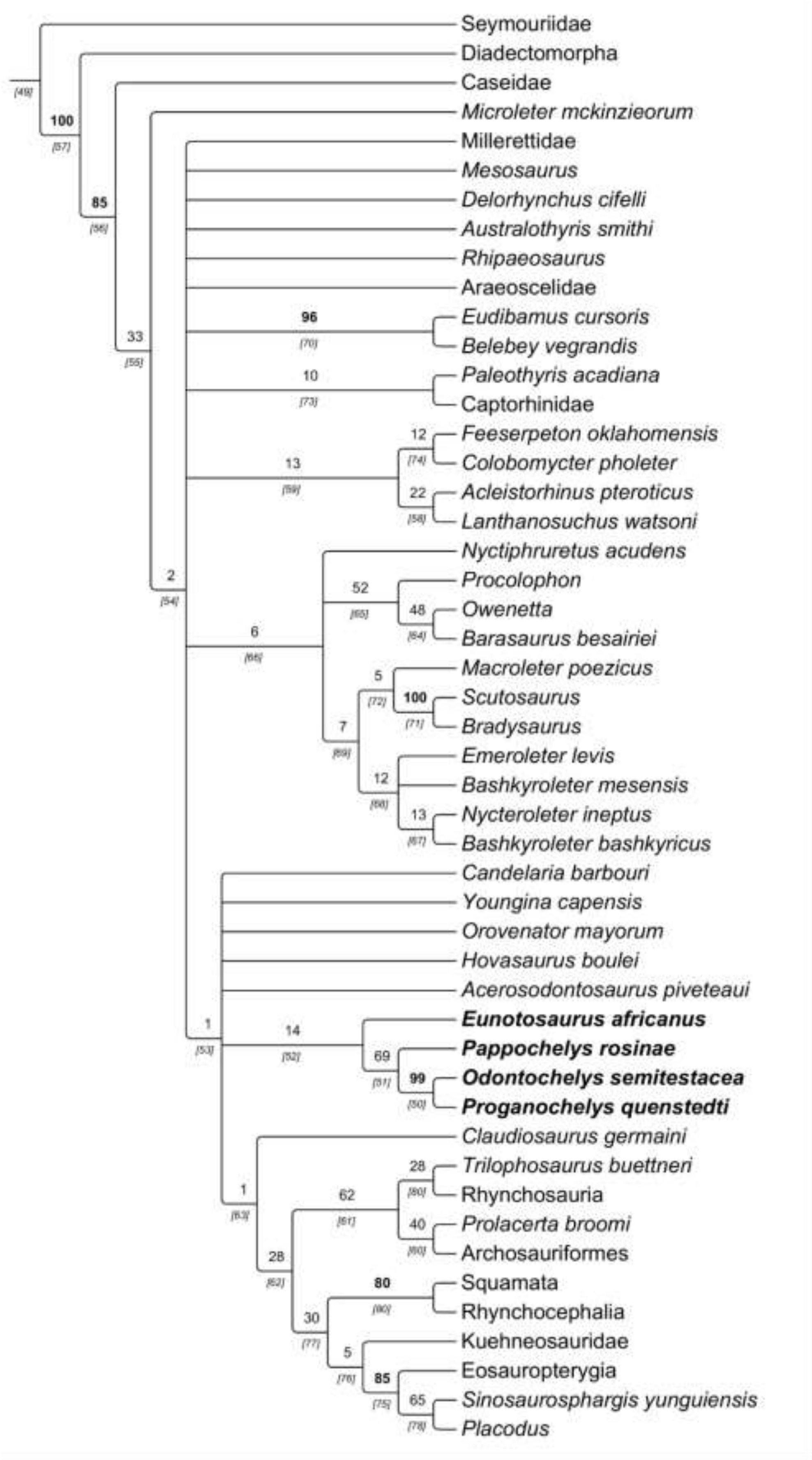

***Proganochelys quenstedti*:**
Char. 8: 0 → 1
Char. 11: 0 → 1
Char. 106: 0 → 1
Char. 108: 1 → 0
Char. 128: 0 → 1
Char. 175: 0 → 1
Char. 202: 1 → 0
Char. 207: 1 → 0
Char. 209: 1 → 0
Char. 244: 0 → 1
Char. 248: 0 → 1
Char. 250: 0 → 1
Char. 252: 1 → 0
Char. 262: 0 → 1

***Pappochelys rosinae*:**
Char. 0: 2 → 0
Char. 1: 0 → 1
Char. 12: 0 → 1
Char. 23: 0 → 1
Char. 41: 0 → 1
Char. 49: 0 → 1
Char. 75: 0 → 1
Char. 169: 1 → 0
Char. 260: 2 → 0

***Odontochelys semitestacea*:**
Char. 43: 0 → 1

***Eunotosaurus africanus*:**
Char. 19: 0 → 1
Char. 43: 0 → 1
Char. 59: 1 → 0
Char. 76: 0 → 1
Char. 97: 1 → 0
Char. 103: 0 → 1
Char. 153: 1 → 0
Char. 191: 1 → 0
Char. 192: 1 → 0
Char. 202: 1 → 0
Char. 211: 0 → 1
Char. 222: 1 → 0
Char. 237: 1 → 0
Char. 248: 0 → 1
Char. 249: 0 → 2
Char. 250: 0 → 1
Char. 263: 0 → 1
Char. 273: 0 → 1
Char. 274: 0 → 1
Char. 275: 0 → 1
Char. 276: 0 → 1
Char. 277: 0 → 1

***Acerosodontosaurus piveteaui*:**
Char. 23: 0 → 1
Char. 78: 1 → 0
Char. 81: 1 → 0
Char. 127: 1 → 0
Char. 128: 0 → 1
Char. 155: 0 → 1
Char. 208: 0 → 1
Char. 278: 3 → 1

***Acleistorhinus pteroticus*:**
Char. 21: 0 → 1
Char. 146: 1 → 0

**Araeoscelidae:**
Char. 0: 0 → 1
Char. 23: 0 → 1
Char. 27: 0 → 1
Char. 28: 0 → 1
Char. 29: 1 → 0
Char. 38: 1 → 0
Char. 40: 2 → 0
Char. 43: 0 → 1
Char. 50: 1 → 0
Char. 57: 1 → 0
Char. 59: 0 → 1
Char. 60: 0 → 1
Char. 67: 0 → 1
Char. 72: 1 → 2
Char. 84: 1 → 0
Char. 89: 0 → 1
Char. 106: 0 → 1
Char. 111: 0 → 1
Char. 116: 1 → 0
Char. 117: 0 → 2
Char. 120: 0 → 1
Char. 154: 1 → 0
Char. 159: 1 → 0
Char. 166: 1 → 0
Char. 169: 1 → 0
Char. 170: 0 → 1
Char. 193: 0 → 1
Char. 197: 0 → 1
Char. 222: 0 → 1
Char. 224: 0 → 1
Char. 234: 0 → 1
Char. 237: 0 → 1
Char. 239: 0 → 1
Char. 266: 0 → 1
Char. 278: 3 → 1

**Archosauriformes:**
Char. 32: 0 → 1
Char. 94: 1 → 0
Char. 112: 1 → 0
Char. 152: 0 → 1
Char. 154: 0 → 2
Char. 166: 1 → 0
Char. 171: 0 → 1
Char. 185: 0 → 1
Char. 204: 0 → 1
Char. 218: 0 → 3
Char. 224: 0 → 1
Char. 242: 0 → 1

***Australothyris smithi*:**
Char. 23: 0 → 1
Char. 24: 0 → 1
Char. 34: 0 → 1
Char. 55: 1 → 0
Char. 57: 1 → 0
Char. 71: 1 → 0
Char. 79: 0 → 1
Char. 83: 0 → 1
Char. 85: 1 → 0
Char. 98: 1 → 0
Char. 100: 0 → 1
Char. 103: 0 → 1
Char. 110: 0 → 1
Char. 123: 1 → 0
Char. 129: 0 → 1
Char. 131: 0 → 1
Char. 132: 1 → 0
Char. 144: 1 → 0
Char. 147: 0 → 1
Char. 149: 1 → 0
Char. 150: 0 → 1
Char. 159: 1 → 0
Char. 192: 1 → 0

***Barasaurus besairiei*:**
Char. 33: 1 → 0

***Bashkyroleter bashkyricus*:**
Char. 275: 1 → 0

***Bashkyroleter mesensis*:**
Char. 12: 0 → 1
Char. 66: 1 → 0
Char. 169: 1 → 0

***Belebey vegrandis*:**
Char. 154: 1 → 0

***Bradysaurus* spp.:**
Char. 19: 0 → 1
Char. 25: 1 → 0
Char. 73: 0 → 1
Char. 79: 0 → 1
Char. 135: 1 → 0
Char. 249: 0 → 1

***Candelaria barbouri*:**
Char. 0: 1 → 0
Char. 1: 0 → 1
Char. 8: 0 → 1
Char. 15: 0 → 1
Char. 23: 0 → 1
Char. 33: 1 → 2
Char. 49: 0 → 1
Char. 59: 1 → 0
Char. 76: 0 → 1
Char. 79: 0 → 2
Char. 83: 0 → 1
Char. 88: 1 → 0
Char. 95: 0 → 1
Char. 126: 0 → 1
Char. 132: 1 → 0
Char. 154: 1 → 2
Char. 159: 1 → 0
Char. 169: 1 → 0
Char. 265: 1 → 0
Char. 275: 0 → 1
Char. 276: 0 → 1
Char. 277: 0 → 1

**Captorhinidae:**
Char. 3: 0 → 1
Char. 23: 0 → 1
Char. 25: 1 → 0
Char. 26: 0 → 1
Char. 73: 0 → 1
Char. 75: 0 → 1
Char. 83: 0 → 1
Char. 108: 1 → 0
Char. 180: 1 → 0
Char. 183: 1 → 0
Char. 201: 1 → 0
Char. 203: 1 → 2
Char. 216: 1 → 0

**Caseidae:**
Char. 38: 1 → 0
Char. 46: 1 → 0
Char. 50: 1 → 0
Char. 170: 0 → 1
Char. 194: 0 → 1
Char. 273: 0 → 1
Char. 274: 0 → 1
Char. 278: 3 → 2

***Claudiosaurus germaini*:**
Char. 24: 0 → 1
Char. 27: 0 → 1
Char. 34: 0 → 1
Char. 36: 0 → 1
Char. 64: 0 → 1
Char. 84: 1 → 0
Char. 105: 0 → 1
Char. 127: 1 → 0
Char. 130: 0 → 1
Char. 141: 1 → 0
Char. 144: 1 → 0
Char. 166: 1 → 0
Char. 187: 1 → 0
Char. 199: 0 → 1
Char. 203: 1 → 2
Char. 204: 0 → 1
Char. 222: 1 → 0

***Colobomycter pholeter*:**
Char. 21: 0 → 1
Char. 25: 1 → 0
Char. 84: 1 → 0
Char. 154: 1 → 0
Char. 167: 0 → 1
Char. 267: 0 → 1

***Delorhynchus cifelli*:**
Char. 18: 0 → 1
Char. 20: 0 → 1
Char. 21: 0 → 1
Char. 24: 0 → 1
Char. 26: 0 → 1
Char. 28: 0 → 1
Char. 33: 0 → 2
Char. 39: 0 → 1
Char. 52: 0 → 1
Char. 100: 0 → 1
Char. 111: 0 → 1
Char. 116: 1 → 0
Char. 117: 0 → 1
Char. 119: 1 → 0
Char. 131: 0 → 1
Char. 147: 0 → 1
Char. 156: 1 → 0
Char. 167: 0 → 1
Char. 189: 0 → 1
Char. 191: 0 → 1
Char. 204: 0 → 2
Char. 267: 0 → 1

**Diadectomorpha:**
Char. 0: 0 → 1
Char. 64: 0 → 1
Char. 70: 0 → 1
Char. 122: 0 → 1
Char. 123: 1 → 0
Char. 146: 1 → 0
Char. 275: 1 → 0
Char. 278: 3 → 0

***Emeroleter levis*:**
Char. 51: 1 → 0

**Eosauropterygia:**
Char. 166: 1 → 0
Char. 174: 0 → 1
Char. 178: 0 → 1
Char. 194: 0 → 2
Char. 272: 1 → 0

***Eudibamus cursoris*:**
Char. 154: 1 → 2

***Feeserpeton oklahomensis*:**
Char. 51: 0 → 1
Char. 157: 0 → 1
Char. 158: 0 → 1

***Hovasaurus boulei*:**
Char. 41: 0 → 1
Char. 43: 0 → 1
Char. 77: 0 → 2
Char. 78: 1 → 0
Char. 79: 0 → 1
Char. 93: 1 → 0
Char. 113: 0 → 1
Char. 138: 0 → 1
Char. 141: 1 → 0
Char. 146: 1 → 0
Char. 204: 0 → 2
Char. 224: 0 → 1

**Kuehneosauridae:**
Char. 7: 0 → 1
Char. 24: 0 → 1
Char. 27: 0 → 1
Char. 43: 1 → 0
Char. 79: 0 → 2
Char. 107: 1 → 0
Char. 140: 1 → 0
Char. 147: 1 → 0
Char. 148: 1 → 0
Char. 245: 0 → 1

Char. 272: 1 → 2
Char. 278: 0 → 3

***Lanthanosuchus watsoni*:**
Char. 25: 1 → 0
Char. 51: 0 → 1
Char. 76: 0 → 1
Char. 86: 0 → 1
Char. 98: 1 → 0
Char. 138: 0 → 1
Char. 144: 1 → 0
Char. 154: 1 → 2

***Macroleter poezicus*:**
Char. 9: 0 → 1
Char. 26: 0 → 1
Char. 66: 1 → 2
Char. 126: 0 → 1
Char. 134: 0 → 2
Char. 140: 0 → 1
Char. 146: 1 → 0
Char. 169: 1 → 0

***Mesosaurus* spp.:**
Char. 0: 0 → 1
Char. 2: 0 → 1
Char. 6: 0 → 1
Char. 8: 0 → 1
Char. 9: 0 → 1
Char. 13: 0 → 1
Char. 19: 0 → 1
Char. 23: 0 → 1
Char. 26: 0 → 1
Char. 29: 1 → 0
Char. 33: 0 → 1
Char. 38: 1 → 0
Char. 41: 0 → 1
Char. 50: 1 → 0
Char. 67: 0 → 1
Char. 76: 0 → 1
Char. 83: 0 → 1
Char. 84: 1 → 0
Char. 85: 1 → 0
Char. 94: 0 → 1
Char. 107: 0 → 1
Char. 109: 0 → 1
Char. 111: 0 → 1
Char. 115: 0 → 1
Char. 146: 1 → 0
Char. 148: 0 → 1
Char. 149: 1 → 0
Char. 159: 1 → 0
Char. 164: 0 → 1
Char. 166: 1 → 0
Char. 167: 0 → 1
Char. 176: 0 → 1
Char. 183: 1 → 0
Char. 184: 0 → 1
Char. 192: 1 → 0
Char. 199: 0 → 1
Char. 202: 0 → 1
Char. 204: 0 → 2
Char. 206: 0 → 1
Char. 207: 0 → 1
Char. 209: 0 → 1
Char. 217: 0 → 1
Char. 219: 0 → 1
Char. 220: 0 → 1
Char. 231: 0 → 1
Char. 260: 2 → 0
Char. 272: 2 → 0
Char. 278: 3 → 0

***Microleter mckinzieorum*:**
Char. 0: 0 → 1
Char. 18: 0 → 1
Char. 39: 0 → 1
Char. 57: 1 → 0
Char. 70: 0 → 1
Char. 76: 0 → 1
Char. 83: 0 → 1
Char. 94: 0 → 1
Char. 106: 0 → 1
Char. 110: 0 → 1
Char. 278: 3 → 1

**Millerettidae:**
Char. 24: 0 → 1
Char. 25: 1 → 0
Char. 44: 1 → 0
Char. 56: 0 → 1
Char. 57: 1 → 0
Char. 66: 0 → 2
Char. 78: 1 → 0
Char. 80: 1 → 0
Char. 84: 1 → 2
Char. 88: 1 → 0
Char. 96: 1 → 0
Char. 117: 0 → 1
Char. 121: 0 → 1
Char. 124: 0 → 1
Char. 127: 0 → 1
Char. 135: 0 → 1
Char. 145: 0 → 1
Char. 159: 1 → 0
Char. 166: 1 → 0
Char. 180: 1 → 0
Char. 192: 1 → 0
Char. 202: 0 → 1
Char. 211: 0 → 1
Char. 230: 0 → 1
Char. 234: 0 → 1
Char. 248: 0 → 1
Char. 252: 0 → 1
Char. 253: 0 → 1

***Nycteroleter ineptus*:**
Char. 66: 1 → 0
Char. 278: 0 → 3

***Nyctiphruretus acudens*:**
Char. 0: 0 → 1
Char. 21: 0 → 1
Char. 33: 1 → 2
Char. 41: 0 → 1
Char. 66: 1 → 2
Char. 81: 1 → 0
Char. 83: 0 → 1
Char. 84: 1 → 2
Char. 94: 0 → 1
Char. 166: 1 → 0
Char. 224: 0 → 1
Char. 235: 1 → 0
Char. 266: 0 → 1
Char. 272: 2 → 1
Char. 276: 0 → 1

***Orovenator mayorum*:**
Char. 8: 0 → 1
Char. 24: 0 → 1
Char. 33: 1 → 0
Char. 36: 0 → 1
Char. 50: 0 → 1
Char. 94: 1 → 0
Char. 135: 1 → 0
Char. 159: 1 → 0
Char. 160: 0 → 1
Char. 165: 0 → 1
Char. 278: 3 → 1

***Owenetta* spp.:**
Char. 142: 1 → 0
Char. 169: 1 → 0

***Paleothyris acadiana*:**
Char. 38: 1 → 0
Char. 50: 1 → 0
Char. 66: 1 → 2
Char. 102: 0 → 1
Char. 146: 1 → 0
Char. 237: 0 → 1
Char. 239: 0 → 1

***Placodus* spp.:**
Char. 0: 1 → 2
Char. 9: 0 → 1
Char. 12: 0 → 1
Char. 13: 0 → 1
Char. 19: 0 → 1
Char. 31: 0 → 1
Char. 46: 1 → 0
Char. 57: 0 → 1
Char. 78: 1 → 0
Char. 93: 1 → 0
Char. 102: 1 → 2
Char. 109: 1 → 0
Char. 140: 1 → 0
Char. 155: 0 → 1
Char. 163: 1 → 0
Char. 164: 0 → 1

***Procolophon* spp.:**
Char. 41: 0 → 1
Char. 69: 0 → 1
Char. 79: 0 → 1
Char. 83: 0 → 1
Char. 86: 0 → 1
Char. 88: 0 → 1
Char. 101: 0 → 1
Char. 117: 1 → 0
Char. 141: 0 → 1
Char. 149: 1 → 0
Char. 203: 1 → 2
Char. 204: 0 → 1
Char. 215: 0 → 1
Char. 230: 0 → 1
Char. 235: 1 → 2
Char. 237: 0 → 1
Char. 238: 0 → 1
Char. 272: 2 → 1
Char. 278: 3 → 0

***Prolacerta broomi*:**
Char. 58: 1 → 0
Char. 66: 1 → 0
Char. 67: 1 → 0
Char. 80: 1 → 0
Char. 139: 1 → 0
Char. 147: 1 → 0
Char. 192: 1 → 0
Char. 203: 1 → 2
Char. 206: 0 → 12

***Rhipaeosaurus* spp.:**
Char. 148: 0 → 1
Char. 172: 0 → 1
Char. 183: 1 → 2
Char. 194: 0 → 1
Char. 201: 1 → 0
Char. 211: 0 → 1
Char. 215: 0 → 1
Char. 226: 0 → 1
Char. 235: 1 → 0
Char. 252: 0 → 1
Char. 277: 0 → 1

**Rhynchocephalia:**
Char. 0: 1 → 2
Char. 23: 1 → 0
Char. 24: 0 → 1
Char. 77: 0 → 1
Char. 88: 1 → 0
Char. 94: 1 → 0
Char. 117: 12 → 0
Char. 139: 1 → 0
Char. 167: 1 → 0
Char. 205: 1 → 0

**Rhynchosauria:**
Char. 0: 1 → 0
Char. 7: 0 → 1
Char. 9: 0 → 1
Char. 26: 0 → 1
Char. 44: 1 → 0
Char. 68: 0 → 1
Char. 99: 1 → 0
Char. 150: 1 → 0
Char. 160: 0 → 1
Char. 161: 0 → 1
Char. 171: 0 → 2
Char. 182: 1 → 0
Char. 223: 0 → 1
Char. 224: 0 → 1
Char. 241: 0 → 1

***Scutosaurus* spp.:**
Char. 175: 0 → 1
Char. 190: 0 → 1
Char. 218: 0 → 1
Char. 243: 0 → 2
Char. 244: 0 → 1
Char. 251: 0 → 1

***Sinosaurosphargis yunguiensis*:**
Char. 8: 0 → 1
Char. 30: 0 → 1
Char. 53: 0 → 1
Char. 89: 1 → 0
Char. 127: 1 → 0
Char. 150: 1 → 0
Char. 154: 0 → 2
Char. 167: 1 → 0
Char. 253: 0 → 1
Char. 255: 0 → 1

**Squamata:**
Char. 45: 0 → 1
Char. 79: 0 → 2
Char. 80: 1 → 0
Char. 92: 1 → 0
Char. 109: 1 → 0
Char. 160: 0 → 1
Char. 200: 0 → 1
Char. 245: 0 → 1

***Trilophosaurus buettneri*:**
Char. 5: 1 → 0
Char. 11: 0 → 1
Char. 55: 1 → 0
Char. 93: 1 → 0
Char. 104: 0 → 1
Char. 113: 0 → 1
Char. 122: 0 → 1
Char. 136: 1 → 0
Char. 144: 1 → 0
Char. 154: 0 → 1
Char. 157: 0 → 1
Char. 159: 1 → 0
Char. 177: 0 → 12
Char. 194: 0 → 1
Char. 203: 1 → 2
Char. 207: 1 → 0
Char. 208: 1 → 0
Char. 272: 1 → 0

***Youngina capensis*:**
Char. 21: 0 → 1
Char. 25: 0 → 1
Char. 27: 0 → 1
Char. 38: 1 → 0
Char. 43: 0 → 1
Char. 44: 1 → 0
Char. 56: 0 → 1
Char. 60: 0 → 1
Char. 75: 0 → 1
Char. 84: 1 → 0
Char. 94: 1 → 0
Char. 134: 0 → 1
Char. 154: 1 → 0
Char. 170: 0 → 1
Char. 211: 0 → 1

Char. 214: 0 → 1
Char. 224: 0 → 1
Char. 239: 0 → 1
Char. 267: 0 → 1
Char. 278: 3 → 0

**Node 50:**
Char. 46: 1 → 0
Char. 88: 1 → 0
Char. 89: 1 → 0
Char. 93: 1 → 0
Char. 176: 0 → 1
Char. 195: 0 → 2
Char. 198: 0 → 1
Char. 246: 1 → 2
Char. 259: 0 → 1
Char. 265: 1 → 0
Char. 270: 0 → 1

**Node 51:**
Char. 65: 0 → 1
Char. 131: 0 → 1
Char. 184: 0 → 1
Char. 205: 0 → 1
Char. 210: 0 → 1
Char. 241: 0 → 1
Char. 246: 0 → 1
Char. 254: 0 → 1
Char. 255: 0 → 1
Char. 256: 0 → 1
Char. 267: 0 → 1
Char. 268: 0 → 1
Char. 269: 0 → 1
Char. 278: 3 → 0

**Node 52:**
Char. 0: 1 → 2
Char. 15: 0 → 1
Char. 21: 0 → 1
Char. 33: 1 → 2
Char. 44: 1 → 0
Char. 64: 0 → 1
Char. 73: 0 → 1
Char. 84: 1 → 2
Char. 130: 0 → 1
Char. 134: 0 → 2
Char. 147: 0 → 1
Char. 148: 0 → 1
Char. 152: 0 → 1
Char. 155: 0 → 1
Char. 158: 0 → 1
Char. 161: 0 → 1
Char. 174: 0 → 1
Char. 181: 0 → 1
Char. 203: 1 → 2
Char. 204: 0 → 2
Char. 247: 0 → 1
Char. 251: 0 → 1
Char. 252: 0 → 1
Char. 253: 0 → 2

**Node 53:**
Char. 0: 0 → 1
Char. 20: 0 → 1
Char. 25: 1 → 0
Char. 29: 1 → 0
Char. 33: 0 → 1
Char. 40: 2 → 0
Char. 50: 1 → 0
Char. 57: 1 → 0
Char. 59: 0 → 1
Char. 67: 0 → 1
Char. 89: 0 → 1
Char. 94: 0 → 1
Char. 111: 0 → 1
Char. 120: 0 → 1
Char. 127: 0 → 1
Char. 129: 0 → 1
Char. 135: 0 → 1
Char. 136: 0 → 1
Char. 139: 0 → 1
Char. 141: 0 → 1
Char. 179: 0 → 1
Char. 187: 0 → 1
Char. 188: 0 → 1
Char. 191: 0 → 1
Char. 193: 0 → 1
Char. 196: 0 → 1
Char. 201: 1 → 0
Char. 202: 0 → 1
Char. 207: 0 → 1
Char. 209: 0 → 1
Char. 222: 0 → 1
Char. 234: 0 → 1
Char. 237: 0 → 1
Char. 265: 0 → 1
Char. 266: 0 → 1
Char. 275: 1 → 0

**Node 54:**
Char. 132: 0 → 1
Char. 166: 0 → 1
Char. 276: 1 → 0

**Node 55:**
Char. 29: 0 → 1
Char. 84: 0 → 1
Char. 88: 0 → 1
Char. 169: 0 → 1

**Node 56:**
Char. 72: 0 → 1
Char. 81: 0 → 1
Char. 93: 0 → 1
Char. 97: 0 → 1
Char. 104: 1 → 0
Char. 144: 0 → 1
Char. 173: 0 → 1
Char. 183: 0 → 1

**Node 58:**
Char. 95: 0 → 1
Char. 113: 0 → 1
Char. 114: 0 → 1

**Node 59:**
Char. 20: 0 → 1
Char. 47: 0 → 1
Char. 79: 0 → 1
Char. 110: 0 → 1
Char. 131: 0 → 1
Char. 137: 0 → 1
Char. 140: 0 → 2
Char. 147: 0 → 1

**Node 60:**
Char. 19: 0 → 1
Char. 92: 1 → 0

**Node 61:**
Char. 4: 0 → 1
Char. 15: 0 → 1
Char. 29: 0 → 1
Char. 213: 0 → 1
Char. 226: 0 → 2
Char. 228: 0 → 1
Char. 275: 0 → 1

**Node 62:**
Char. 43: 0 → 1
Char. 58: 0 → 1
Char. 61: 0 → 1
Char. 66: 0 → 1
Char. 69: 0 → 1
Char. 107: 0 → 1
Char. 140: 0 → 1
Char. 147: 0 → 1
Char. 150: 0 → 1
Char. 167: 0 → 1
Char. 205: 0 → 1
Char. 208: 0 → 1
Char. 230: 0 → 1
Char. 239: 0 → 1

**Node 63:**
Char. 23: 0 → 1
Char. 60: 0 → 1
Char. 70: 0 → 1
Char. 73: 0 → 1
Char. 117: 0 → 12
Char. 126: 0 → 1
Char. 131: 0 → 1
Char. 148: 0 → 1
Char. 154: 1 → 0
Char. 182: 0 → 1
Char. 190: 0 → 1
Char. 214: 0 → 1
Char. 267: 0 → 1
Char. 272: 2 → 1
Char. 278: 3 → 0

**Node 64:**
Char. 73: 0 → 1
Char. 131: 1 → 0
Char. 205: 0 → 1
Char. 273: 0 → 1
Char. 276: 0 → 1

**Node 65:**
Char. 18: 0 → 1
Char. 23: 0 → 1
Char. 25: 1 → 0
Char. 37: 0 → 1
Char. 103: 0 → 1
Char. 106: 0 → 1
Char. 107: 0 → 1
Char. 110: 0 → 1
Char. 126: 0 → 1
Char. 150: 0 → 1

**Node 66:**
Char. 20: 0 → 1
Char. 33: 0 → 1
Char. 38: 1 → 2
Char. 39: 0 → 1
Char. 44: 1 → 0
Char. 49: 0 → 1
Char. 51: 0 → 1
Char. 66: 0 → 1
Char. 76: 0 → 1
Char. 80: 1 → 0
Char. 88: 1 → 0
Char. 95: 0 → 1
Char. 117: 0 → 1
Char. 131: 0 → 1
Char. 135: 0 → 1
Char. 137: 0 → 1
Char. 147: 0 → 1
Char. 148: 0 → 1
Char. 157: 0 → 1
Char. 158: 0 → 1
Char. 183: 1 → 2
Char. 186: 0 → 1
Char. 187: 0 → 1
Char. 194: 0 → 1
Char. 197: 0 → 1
Char. 201: 1 → 0
Char. 211: 0 → 1
Char. 252: 0 → 1

**Node 67:**
Char. 87: 0 → 1
Char. 110: 0 → 1

**Node 68:**
Char. 79: 0 → 1
Char. 133: 0 → 1

**Node 69:**
Char. 100: 0 → 1
Char. 113: 0 → 1

**Node 70:**
Char. 38: 1 → 2
Char. 39: 0 → 1
Char. 50: 1 → 0
Char. 58: 0 → 1
Char. 59: 0 → 1
Char. 60: 0 → 1
Char. 72: 1 → 2
Char. 83: 0 → 1
Char. 85: 1 → 0
Char. 88: 1 → 0
Char. 95: 0 → 1
Char. 104: 0 → 1
Char. 105: 0 → 1
Char. 106: 0 → 2
Char. 107: 0 → 1
Char. 109: 0 → 1
Char. 110: 0 → 1
Char. 146: 1 → 0
Char. 148: 0 → 1
Char. 155: 0 → 2
Char. 183: 1 → 2

**Node 71:**
Char. 23: 0 → 1
Char. 33: 1 → 0
Char. 38: 2 → 1
Char. 39: 1 → 0
Char. 42: 0 → 1
Char. 43: 0 → 1
Char. 46: 1 → 0
Char. 49: 1 → 0
Char. 71: 1 → 0
Char. 83: 0 → 2
Char. 101: 0 → 1
Char. 103: 0 → 1
Char. 106: 0 → 1
Char. 161: 0 → 1
Char. 163: 1 → 0
Char. 172: 0 → 2
Char. 174: 0 → 1
Char. 188: 0 → 1
Char. 203: 1 → 2
Char. 212: 0 → 1
Char. 216: 1 → 0
Char. 235: 1 → 2
Char. 236: 0 → 1
Char. 238: 0 → 2
Char. 241: 0 → 1
Char. 242: 0 → 1
Char. 273: 0 → 1
Char. 274: 0 → 1
Char. 275: 1 → 0

**Node 72:**
Char. 52: 0 → 1
Char. 75: 0 → 1
Char. 84: 1 → 0
Char. 87: 0 → 1
Char. 110: 0 → 1
Char. 121: 0 → 1
Char. 180: 1 → 0
Char. 230: 0 → 1

**Node 73:**
Char. 29: 1 → 0
Char. 44: 1 → 0
Char. 59: 0 → 1
Char. 60: 0 → 1
Char. 66: 0 → 1
Char. 67: 0 → 1
Char. 72: 1 → 2
Char. 78: 1 → 0
Char. 84: 1 → 0
Char. 88: 1 → 0
Char. 93: 1 → 0
Char. 111: 0 → 1
Char. 123: 1 → 0
Char. 129: 0 → 1
Char. 154: 1 → 0
Char. 159: 1 → 0

Char. 169: 1 → 0
Char. 170: 0 → 1
Char. 192: 1 → 0
Char. 197: 0 → 1

**Node 74:**
Char. 83: 0 → 1
Char. 85: 1 → 0
Char. 107: 0 → 1

**Node 75:**
Char. 2: 0 → 1
Char. 6: 0 → 1
Char. 186: 0 → 1

Char. 198: 0 → 1
Char. 200: 0 → 1
Char. 220: 0 → 1
Char. 239: 1 → 0
Char. 267: 1 → 0

**Node 76:**
Char. 98: 1 → 0
Char. 113: 0 → 1
Char. 181: 0 → 1
Char. 206: 0 → 2

**Node 77:**
Char. 35: 0 → 1

Char. 102: 0 → 1
Char. 103: 0 → 1
Char. 184: 0 → 1
Char. 210: 0 → 1
Char. 223: 0 → 1
Char. 229: 0 → 1

**Node 78:**
Char. 17: 0 → 1
Char. 42: 0 → 1
Char. 251: 0 → 1
Char. 264: 0 → 1

**Node 79:**
Char. 41: 0 → 1
Char. 61: 1 → 2
Char. 112: 1 → 0
Char. 138: 0 → 1
Char. 146: 1 → 0
Char. 155: 0 → 1
Char. 182: 1 → 0
Char. 192: 1 → 2
Char. 224: 0 → 1
Char. 226: 0 → 1
Char. 227: 0 → 1
Char. 233: 0 → 1
Char. 267: 1 → 2

**Node 80:**
Char. 61: 1 → 3
Char. 90: 0 → 1
Char. 91: 0 → 1
Char. 109: 1 → 0
Char. 155: 0 → 1
Char. 209: 1 → 0

**ANALYSIS 45**
**(ALL TAXA, IMPLIED WEIGHTING, K = 3.625)**

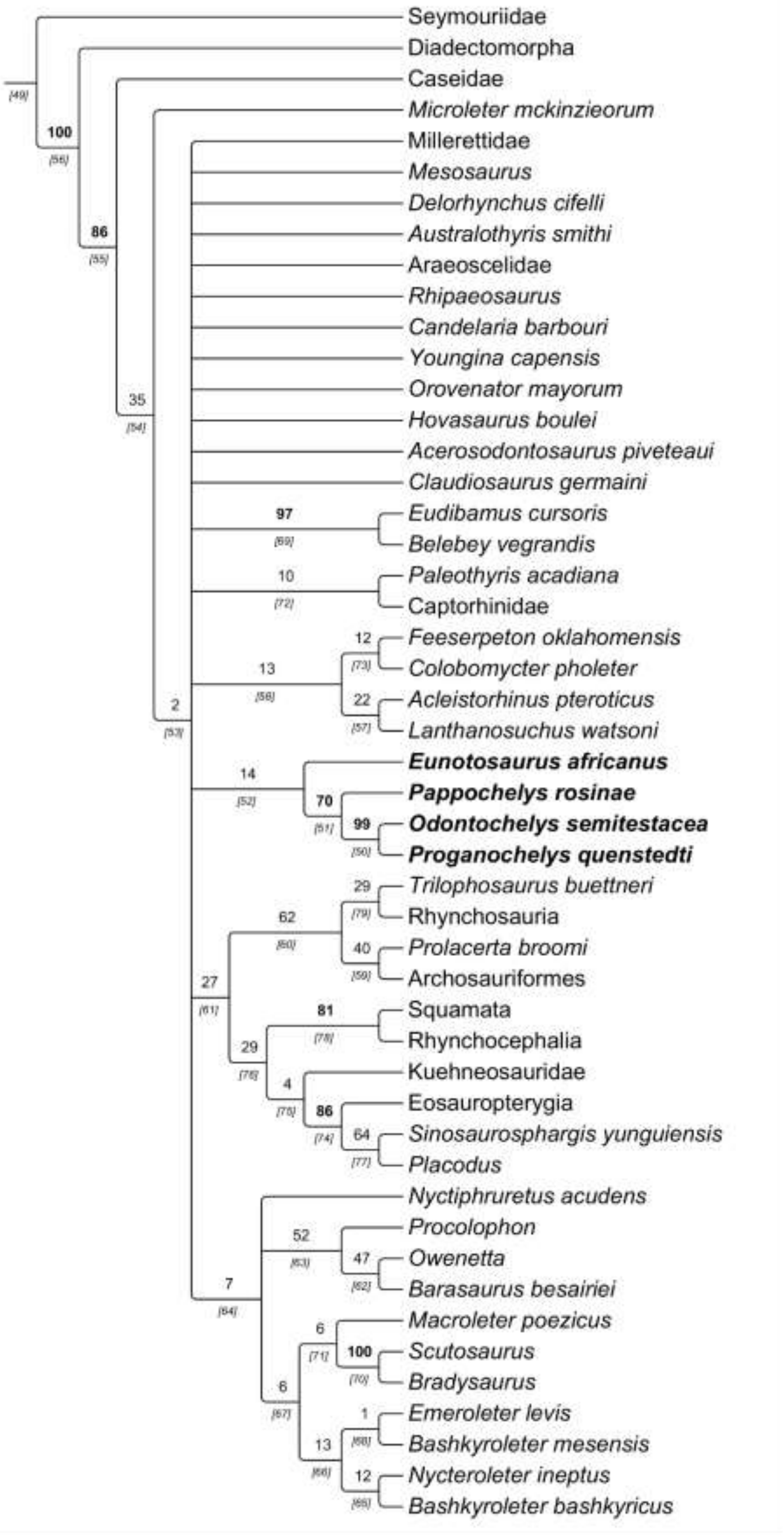

***Proganochelys quenstedti*:**
Char. 8: 0 → 1
Char. 11: 0 → 1
Char. 106: 0 → 1
Char. 108: 1 → 0
Char. 109: 0 → 1
Char. 128: 0 → 1
Char. 175: 0 → 1
Char. 202: 1 → 0
Char. 207: 1 → 0
Char. 209: 1 → 0
Char. 244: 0 → 1
Char. 248: 0 → 1
Char. 250: 0 → 1
Char. 252: 1 → 0
Char. 262: 0 → 1

***Pappochelys rosinae*:**
Char. 0: 2 → 0
Char. 1: 0 → 1
Char. 5: 1 → 0
Char. 12: 0 → 1
Char. 41: 0 → 1
Char. 49: 0 → 1
Char. 75: 0 → 1
Char. 129: 0 → 1
Char. 169: 1 → 0
Char. 260: 2 → 0
Char. 265: 0 → 1

***Odontochelys semitestacea*:**
Char. 43: 0 → 1

***Eunotosaurus africanus*:**
Char. 19: 0 → 1
Char. 43: 0 → 1
Char. 76: 0 → 1
Char. 97: 1 → 0
Char. 103: 0 → 1
Char. 153: 1 → 0
Char. 192: 1 → 0
Char. 202: 1 → 0
Char. 211: 0 → 1
Char. 219: 0 → 1
Char. 248: 0 → 1
Char. 249: 0 → 2
Char. 250: 0 → 1
Char. 263: 0 → 1
Char. 273: 0 → 1
Char. 274: 0 → 1
Char. 276: 0 → 1
Char. 277: 0 → 1

***Acerosodontosaurus piveteaui*:**
Char. 78: 1 → 0
Char. 81: 1 → 0
Char. 89: 0 → 1
Char. 94: 0 → 1
Char. 128: 0 → 1
Char. 129: 0 → 1
Char. 155: 0 → 1
Char. 206: 0 → 2
Char. 208: 0 → 1
Char. 265: 0 → 1
Char. 266: 0 → 1
Char. 278: 3 → 1

***Acleistorhinus pteroticus*:**
Char. 21: 0 → 1
Char. 146: 1 → 0

**Araeoscelidae:**
Char. 5: 1 → 0
Char. 20: 1 → 0
Char. 27: 0 → 1
Char. 28: 0 → 1
Char. 33: 1 → 0
Char. 38: 1 → 0
Char. 40: 2 → 0
Char. 43: 0 → 1
Char. 60: 0 → 1
Char. 72: 1 → 2
Char. 84: 1 → 0
Char. 89: 0 → 1
Char. 92: 1 → 0
Char. 106: 0 → 1
Char. 116: 1 → 0
Char. 117: 01 → 2
Char. 154: 1 → 0
Char. 159: 1 → 0
Char. 166: 1 → 0
Char. 169: 1 → 0
Char. 170: 0 → 1
Char. 193: 0 → 1
Char. 197: 0 → 1
Char. 202: 1 → 0
Char. 207: 1 → 0
Char. 209: 1 → 0
Char. 221: 1 → 0
Char. 222: 0 → 1
Char. 224: 0 → 1
Char. 239: 0 → 1
Char. 266: 0 → 1
Char. 278: 3 → 1

**Archosauriformes:**
Char. 32: 0 → 1
Char. 94: 1 → 0
Char. 112: 1 → 0
Char. 152: 0 → 1
Char. 154: 01 → 2
Char. 166: 1 → 0
Char. 171: 0 → 1
Char. 185: 0 → 1
Char. 204: 0 → 1
Char. 218: 0 → 3
Char. 224: 0 → 1
Char. 242: 0 → 1

***Australothyris smithi*:**
Char. 0: 1 → 0
Char. 24: 0 → 1
Char. 29: 0 → 1
Char. 34: 0 → 1
Char. 50: 0 → 1
Char. 55: 1 → 0
Char. 67: 1 → 0
Char. 71: 1 → 0
Char. 79: 0 → 1
Char. 83: 0 → 1
Char. 85: 1 → 0
Char. 98: 1 → 0
Char. 100: 0 → 1
Char. 103: 0 → 1
Char. 110: 0 → 1
Char. 111: 1 → 0
Char. 123: 1 → 0
Char. 129: 0 → 1
Char. 131: 0 → 1
Char. 132: 1 → 0
Char. 144: 1 → 0
Char. 147: 0 → 1
Char. 149: 1 → 0
Char. 150: 0 → 1
Char. 159: 1 → 0
Char. 163: 1 → 0
Char. 192: 1 → 0

***Barasaurus besairiei*:**
Char. 33: 1 → 0

***Bashkyroleter bashkyricus*:**
Char. 275: 1 → 0

***Bashkyroleter mesensis*:**
Char. 12: 0 → 1
Char. 66: 1 → 0
Char. 169: 1 → 0

***Belebey vegrandis*:**
Char. 154: 1 → 0

***Bradysaurus* spp.:**
Char. 19: 0 → 1
Char. 73: 0 → 1
Char. 79: 0 → 1
Char. 135: 1 → 0
Char. 249: 0 → 1

***Candelaria barbouri*:**
Char. 0: 1 → 0
Char. 1: 0 → 1
Char. 5: 1 → 0
Char. 8: 0 → 1
Char. 15: 0 → 1
Char. 33: 1 → 2
Char. 49: 0 → 1
Char. 55: 1 → 0
Char. 76: 0 → 1
Char. 79: 0 → 2
Char. 83: 0 → 1
Char. 88: 1 → 0
Char. 89: 0 → 1
Char. 92: 1 → 0
Char. 94: 0 → 1
Char. 95: 0 → 1
Char. 126: 0 → 1
Char. 127: 0 → 1
Char. 132: 1 → 0
Char. 154: 1 → 2
Char. 159: 1 → 0
Char. 169: 1 → 0
Char. 276: 0 → 1
Char. 277: 0 → 1

**Captorhinidae:**
Char. 3: 0 → 1
Char. 26: 0 → 1
Char. 50: 0 → 1
Char. 73: 0 → 1
Char. 75: 0 → 1
Char. 83: 0 → 1
Char. 108: 1 → 0
Char. 180: 1 → 0
Char. 183: 1 → 0
Char. 203: 1 → 2
Char. 216: 1 → 0
Char. 240: 1 → 0

**Caseidae:**
Char. 38: 1 → 0
Char. 46: 1 → 0
Char. 170: 0 → 1
Char. 194: 0 → 1
Char. 273: 0 → 1
Char. 274: 0 → 1
Char. 278: 3 → 2

***Claudiosaurus germaini*:**
Char. 24: 0 → 1
Char. 27: 0 → 1
Char. 34: 0 → 1
Char. 36: 0 → 1
Char. 56: 0 → 1
Char. 60: 0 → 1
Char. 62: 0 → 1
Char. 64: 0 → 1
Char. 70: 0 → 1
Char. 72: 1 → 2
Char. 73: 0 → 1
Char. 84: 1 → 0
Char. 89: 0 → 1
Char. 94: 0 → 1
Char. 105: 0 → 1
Char. 106: 0 → 1
Char. 109: 0 → 1
Char. 117: 01 → 2
Char. 126: 0 → 1
Char. 129: 0 → 1
Char. 130: 0 → 1
Char. 131: 0 → 1
Char. 144: 1 → 0
Char. 154: 1 → 0
Char. 166: 1 → 0
Char. 182: 0 → 1
Char. 188: 0 → 1
Char. 190: 0 → 1
Char. 191: 0 → 1
Char. 199: 0 → 1
Char. 203: 1 → 2
Char. 204: 0 → 1
Char. 214: 0 → 1
Char. 219: 0 → 1
Char. 220: 0 → 1
Char. 265: 0 → 1
Char. 267: 0 → 1
Char. 272: 2 → 1
Char. 275: 1 → 0
Char. 278: 3 → 0

***Colobomycter pholeter*:**
Char. 21: 0 → 1
Char. 84: 1 → 0
Char. 154: 1 → 0
Char. 167: 0 → 1
Char. 267: 0 → 1

***Delorhynchus cifelli*:**
Char. 0: 1 → 0
Char. 18: 0 → 1
Char. 21: 0 → 1
Char. 24: 0 → 1
Char. 26: 0 → 1
Char. 28: 0 → 1
Char. 29: 0 → 1
Char. 33: 1 → 2
Char. 39: 0 → 1
Char. 50: 0 → 1
Char. 52: 0 → 1
Char. 57: 0 → 1
Char. 67: 1 → 0
Char. 100: 0 → 1
Char. 116: 1 → 0
Char. 119: 1 → 0
Char. 131: 0 → 1
Char. 147: 0 → 1
Char. 156: 1 → 0
Char. 167: 0 → 1
Char. 189: 0 → 1
Char. 191: 0 → 1
Char. 202: 1 → 0
Char. 204: 0 → 2
Char. 267: 0 → 1

**Diadectomorpha:**
Char. 64: 0 → 1
Char. 70: 0 → 1
Char. 122: 0 → 1
Char. 123: 1 → 0
Char. 146: 1 → 0
Char. 275: 1 → 0
Char. 278: 3 → 0

***Emeroleter levis*:**
Char. 51: 1 → 0

**Eosauropterygia:**
Char. 166: 1 → 0
Char. 174: 0 → 1
Char. 178: 0 → 1
Char. 194: 0 → 2
Char. 272: 1 → 0

***Eudibamus cursoris*:**
Char. 154: 1 → 2
Char. 163: 1 → 0

***Feeserpeton oklahomensis*:**
Char. 51: 0 → 1

Char. 157: 0 → 1
Char. 158: 0 → 1

***Hovasaurus boulei*:**
Char. 41: 0 → 1
Char. 43: 0 → 1
Char. 55: 1 → 0
Char. 77: 0 → 2
Char. 78: 1 → 0
Char. 79: 0 → 1
Char. 89: 0 → 1
Char. 93: 1 → 0
Char. 113: 0 → 1
Char. 127: 0 → 1
Char. 135: 0 → 1
Char. 136: 0 → 1
Char. 138: 0 → 1
Char. 146: 1 → 0
Char. 187: 0 → 1
Char. 188: 0 → 1
Char. 191: 0 → 1
Char. 193: 0 → 1
Char. 204: 0 → 2
Char. 206: 0 → 2
Char. 215: 0 → 1
Char. 220: 0 → 1
Char. 222: 0 → 1
Char. 224: 0 → 1
Char. 265: 0 → 1
Char. 275: 1 → 0

**Kuehneosauridae:**
Char. 7: 0 → 1
Char. 24: 0 → 1
Char. 27: 0 → 1
Char. 43: 1 → 0
Char. 79: 0 → 2
Char. 107: 1 → 0
Char. 140: 1 → 0
Char. 147: 1 → 0
Char. 148: 1 → 0
Char. 245: 0 → 1
Char. 278: 0 → 3

***Lanthanosuchus watsoni*:**
Char. 51: 0 → 1
Char. 76: 0 → 1
Char. 86: 0 → 1
Char. 98: 1 → 0
Char. 138: 0 → 1
Char. 144: 1 → 0
Char. 154: 1 → 2

***Macroleter poezicus*:**
Char. 9: 0 → 1
Char. 26: 0 → 1
Char. 66: 1 → 2
Char. 126: 0 → 1
Char. 134: 0 → 2
Char. 140: 0 → 1
Char. 146: 1 → 0
Char. 169: 1 → 0

***Mesosaurus* spp.:**
Char. 2: 0 → 1
Char. 5: 1 → 0
Char. 6: 0 → 1
Char. 8: 0 → 1
Char. 9: 0 → 1
Char. 13: 0 → 1
Char. 19: 0 → 1
Char. 20: 1 → 0
Char. 26: 0 → 1
Char. 38: 1 → 0
Char. 41: 0 → 1
Char. 76: 0 → 1
Char. 83: 0 → 1
Char. 84: 1 → 0
Char. 85: 1 → 0
Char. 94: 0 → 1
Char. 107: 0 → 1
Char. 109: 0 → 1
Char. 112: 1 → 0
Char. 115: 0 → 1
Char. 146: 1 → 0
Char. 149: 1 → 0
Char. 159: 1 → 0
Char. 163: 1 → 0
Char. 164: 0 → 1
Char. 166: 1 → 0
Char. 167: 0 → 1
Char. 176: 0 → 1
Char. 183: 1 → 0
Char. 184: 0 → 1
Char. 192: 1 → 0
Char. 199: 0 → 1
Char. 204: 0 → 2
Char. 206: 0 → 1
Char. 217: 0 → 1
Char. 219: 0 → 1
Char. 220: 0 → 1
Char. 231: 0 → 1
Char. 240: 1 → 0
Char. 260: 2 → 0
Char. 272: 2 → 0
Char. 278: 3 → 0

***Microleter mckinzieorum*:**
Char. 18: 0 → 1
Char. 29: 0 → 1
Char. 39: 0 → 1
Char. 70: 0 → 1
Char. 76: 0 → 1
Char. 83: 0 → 1
Char. 94: 0 → 1
Char. 106: 0 → 1
Char. 110: 0 → 1
Char. 278: 3 → 1

**Millerettidae:**
Char. 0: 1 → 0
Char. 5: 1 → 0
Char. 20: 1 → 0
Char. 24: 0 → 1
Char. 29: 0 → 1
Char. 33: 1 → 0
Char. 44: 1 → 0
Char. 50: 0 → 1
Char. 56: 0 → 1
Char. 66: 0 → 2
Char. 67: 1 → 0
Char. 78: 1 → 0
Char. 80: 1 → 0
Char. 84: 1 → 2
Char. 88: 1 → 0
Char. 96: 1 → 0
Char. 111: 1 → 0
Char. 112: 1 → 0
Char. 120: 1 → 0
Char. 121: 0 → 1
Char. 124: 0 → 1
Char. 127: 0 → 1
Char. 135: 0 → 1
Char. 145: 0 → 1
Char. 159: 1 → 0
Char. 163: 1 → 0
Char. 166: 1 → 0
Char. 180: 1 → 0
Char. 192: 1 → 0
Char. 211: 0 → 1
Char. 230: 0 → 1
Char. 248: 0 → 1
Char. 252: 0 → 1
Char. 253: 0 → 1

***Nycteroleter ineptus*:**
Char. 66: 1 → 0

***Nyctiphruretus acudens*:**
Char. 21: 0 → 1
Char. 33: 1 → 2
Char. 41: 0 → 1
Char. 66: 1 → 2
Char. 81: 1 → 0
Char. 83: 0 → 1
Char. 84: 1 → 2
Char. 94: 0 → 1
Char. 166: 1 → 0
Char. 207: 1 → 0
Char. 224: 0 → 1
Char. 235: 1 → 0
Char. 266: 0 → 1
Char. 272: 2 → 1
Char. 276: 0 → 1

***Orovenator mayorum*:**
Char. 8: 0 → 1
Char. 24: 0 → 1
Char. 33: 1 → 0
Char. 36: 0 → 1
Char. 50: 0 → 1
Char. 62: 0 → 1
Char. 72: 1 → 2
Char. 89: 0 → 1
Char. 92: 1 → 0
Char. 136: 0 → 1
Char. 141: 0 → 1
Char. 159: 1 → 0
Char. 160: 0 → 1
Char. 165: 0 → 1
Char. 278: 3 → 1

***Owenetta* spp.:**
Char. 142: 1 → 0
Char. 169: 1 → 0

***Paleothyris acadiana*:**
Char. 38: 1 → 0
Char. 66: 1 → 2
Char. 102: 0 → 1
Char. 146: 1 → 0
Char. 239: 0 → 1

***Placodus* spp.:**
Char. 0: 1 → 2
Char. 9: 0 → 1
Char. 12: 0 → 1
Char. 13: 0 → 1
Char. 19: 0 → 1
Char. 31: 0 → 1
Char. 46: 1 → 0
Char. 57: 0 → 1
Char. 78: 1 → 0
Char. 93: 1 → 0
Char. 102: 1 → 2
Char. 140: 1 → 0
Char. 155: 0 → 1
Char. 163: 1 → 0
Char. 164: 0 → 1

***Procolophon* spp.:**
Char. 41: 0 → 1
Char. 69: 0 → 1
Char. 79: 0 → 1
Char. 83: 0 → 1
Char. 86: 0 → 1
Char. 88: 0 → 1
Char. 101: 0 → 1
Char. 117: 1 → 0
Char. 141: 0 → 1
Char. 149: 1 → 0
Char. 203: 1 → 2
Char. 204: 0 → 1
Char. 215: 0 → 1
Char. 230: 0 → 1
Char. 235: 1 → 2
Char. 238: 0 → 1
Char. 272: 2 → 1
Char. 278: 3 → 0

***Prolacerta broomi*:**
Char. 58: 1 → 0
Char. 66: 1 → 0
Char. 67: 1 → 0
Char. 80: 1 → 0
Char. 139: 1 → 0
Char. 147: 1 → 0
Char. 192: 1 → 0
Char. 203: 1 → 2
Char. 206: 0 → 12

***Rhipaeosaurus* spp.:**
Char. 120: 1 → 0
Char. 172: 0 → 1
Char. 183: 1 → 2
Char. 194: 0 → 1
Char. 207: 1 → 0
Char. 211: 0 → 1
Char. 215: 0 → 1
Char. 226: 0 → 1
Char. 235: 1 → 0
Char. 240: 1 → 0
Char. 252: 0 → 1
Char. 277: 0 → 1

**Rhynchocephalia:**
Char. 0: 1 → 2
Char. 24: 0 → 1
Char. 77: 0 → 1
Char. 88: 1 → 0
Char. 94: 1 → 0
Char. 139: 1 → 0
Char. 167: 1 → 0
Char. 205: 1 → 0

**Rhynchosauria:**
Char. 0: 1 → 0
Char. 7: 0 → 1
Char. 9: 0 → 1
Char. 26: 0 → 1
Char. 44: 1 → 0
Char. 68: 0 → 1
Char. 99: 1 → 0
Char. 150: 1 → 0
Char. 160: 0 → 1
Char. 161: 0 → 1
Char. 171: 0 → 2
Char. 223: 0 → 1
Char. 224: 0 → 1
Char. 241: 0 → 1

***Scutosaurus* spp.:**
Char. 175: 0 → 1
Char. 190: 0 → 1
Char. 218: 0 → 1
Char. 243: 0 → 2
Char. 244: 0 → 1
Char. 251: 0 → 1

***Sinosaurosphargis yunguiensis*:**
Char. 8: 0 → 1
Char. 30: 0 → 1
Char. 53: 0 → 1
Char. 89: 1 → 0
Char. 127: 1 → 0
Char. 150: 1 → 0
Char. 154: 0 → 2
Char. 167: 1 → 0
Char. 253: 0 → 1
Char. 255: 0 → 1

**Squamata:**
Char. 45: 0 → 1
Char. 79: 0 → 2
Char. 80: 1 → 0
Char. 92: 1 → 0
Char. 160: 0 → 1
Char. 200: 0 → 1
Char. 245: 0 → 1

***Trilophosaurus buettneri*:**
Char. 5: 1 → 0
Char. 11: 0 → 1
Char. 55: 1 → 0
Char. 93: 1 → 0
Char. 104: 0 → 1
Char. 106: 0 → 1
Char. 113: 0 → 1
Char. 122: 0 → 1
Char. 144: 1 → 0
Char. 157: 0 → 1
Char. 159: 1 → 0
Char. 177: 0 → 12
Char. 194: 0 → 1
Char. 203: 1 → 2
Char. 207: 1 → 0
Char. 208: 1 → 0
Char. 272: 1 → 0

***Youngina capensis*:**
Char. 5: 1 → 0
Char. 21: 0 → 1
Char. 27: 0 → 1
Char. 38: 1 → 0
Char. 40: 2 → 0
Char. 43: 0 → 1
Char. 44: 1 → 0
Char. 56: 0 → 1
Char. 60: 0 → 1
Char. 62: 0 → 1
Char. 72: 1 → 2
Char. 75: 0 → 1
Char. 84: 1 → 0
Char. 89: 0 → 1
Char. 92: 1 → 0
Char. 109: 0 → 1
Char. 127: 0 → 1
Char. 129: 0 → 1
Char. 134: 0 → 1
Char. 135: 0 → 1
Char. 141: 0 → 1
Char. 154: 1 → 0
Char. 163: 1 → 0
Char. 170: 0 → 1
Char. 187: 0 → 1
Char. 191: 0 → 1
Char. 193: 0 → 1
Char. 211: 0 → 1
Char. 214: 0 → 1
Char. 215: 0 → 1
Char. 219: 0 → 1
Char. 222: 0 → 1
Char. 224: 0 → 1
Char. 231: 0 → 1
Char. 239: 0 → 1
Char. 265: 0 → 1
Char. 267: 0 → 1
Char. 275: 1 → 0
Char. 278: 3 → 0

**Node 50:**
Char. 46: 1 → 0
Char. 88: 1 → 0
Char. 93: 1 → 0
Char. 176: 0 → 1
Char. 195: 0 → 2
Char. 198: 0 → 1
Char. 246: 1 → 2
Char. 259: 0 → 1
Char. 270: 0 → 1

**Node 51:**
Char. 65: 0 → 1
Char. 72: 1 → 2
Char. 131: 0 → 1
Char. 184: 0 → 1
Char. 205: 0 → 1
Char. 210: 0 → 1
Char. 222: 0 → 1
Char. 241: 0 → 1
Char. 246: 0 → 1
Char. 254: 0 → 1
Char. 255: 0 → 1
Char. 256: 0 → 1
Char. 267: 0 → 1
Char. 268: 0 → 1
Char. 269: 0 → 1
Char. 278: 3 → 0

**Node 52:**
Char. 0: 1 → 2
Char. 15: 0 → 1
Char. 21: 0 → 1
Char. 33: 1 → 2
Char. 44: 1 → 0
Char. 64: 0 → 1
Char. 73: 0 → 1
Char. 84: 1 → 2
Char. 94: 0 → 1
Char. 127: 0 → 1
Char. 130: 0 → 1
Char. 134: 0 → 2
Char. 135: 0 → 1
Char. 136: 0 → 1
Char. 141: 0 → 1
Char. 145: 0 → 1
Char. 147: 0 → 1
Char. 151: 0 → 1
Char. 152: 0 → 1
Char. 155: 0 → 1
Char. 158: 0 → 1
Char. 161: 0 → 1
Char. 174: 0 → 1
Char. 181: 0 → 1
Char. 188: 0 → 1
Char. 203: 1 → 2
Char. 204: 0 → 2
Char. 217: 0 → 1
Char. 220: 0 → 1
Char. 231: 0 → 1
Char. 247: 0 → 1
Char. 251: 0 → 1
Char. 252: 0 → 1
Char. 253: 0 → 2

**Node 53:**
Char. 20: 0 → 1
Char. 33: 0 → 1
Char. 67: 0 → 1
Char. 111: 0 → 1
Char. 112: 0 → 1
Char. 132: 0 → 1
Char. 166: 0 → 1
Char. 276: 1 → 0

**Node 54:**
Char. 57: 1 → 0
Char. 84: 0 → 1
Char. 88: 0 → 1
Char. 169: 0 → 1

**Node 55:**
Char. 72: 0 → 1
Char. 81: 0 → 1
Char. 93: 0 → 1
Char. 97: 0 → 1
Char. 104: 1 → 0
Char. 144: 0 → 1
Char. 173: 0 → 1
Char. 183: 0 → 1

**Node 57:**
Char. 95: 0 → 1
Char. 113: 0 → 1
Char. 114: 0 → 1

**Node 58:**
Char. 0: 1 → 0
Char. 29: 0 → 1
Char. 47: 0 → 1
Char. 50: 0 → 1
Char. 57: 0 → 1
Char. 67: 1 → 0
Char. 79: 0 → 1
Char. 110: 0 → 1
Char. 111: 1 → 0
Char. 112: 1 → 0
Char. 131: 0 → 1
Char. 137: 0 → 1
Char. 140: 0 → 2
Char. 147: 0 → 1

**Node 59:**
Char. 19: 0 → 1
Char. 92: 1 → 0

**Node 60:**
Char. 4: 0 → 1
Char. 15: 0 → 1
Char. 29: 0 → 1
Char. 213: 0 → 1
Char. 226: 0 → 2
Char. 228: 0 → 1

**Node 61:**
Char. 43: 0 → 1
Char. 58: 0 → 1
Char. 60: 0 → 1
Char. 61: 0 → 1
Char. 62: 0 → 1
Char. 66: 0 → 1
Char. 69: 0 → 1
Char. 72: 1 → 2
Char. 73: 0 → 1
Char. 89: 0 → 1
Char. 94: 0 → 1
Char. 107: 0 → 1
Char. 126: 0 → 1
Char. 127: 0 → 1
Char. 129: 0 → 1
Char. 131: 0 → 1
Char. 134: 0 → 1
Char. 135: 0 → 1
Char. 140: 0 → 1
Char. 141: 0 → 1
Char. 145: 0 → 1
Char. 147: 0 → 1
Char. 150: 0 → 1
Char. 151: 0 → 1
Char. 167: 0 → 1
Char. 176: 0 → 1
Char. 188: 0 → 1
Char. 190: 0 → 1
Char. 191: 0 → 1
Char. 205: 0 → 1
Char. 208: 0 → 1
Char. 214: 0 → 1
Char. 219: 0 → 1
Char. 230: 0 → 1
Char. 239: 0 → 1
Char. 260: 2 → 1
Char. 265: 0 → 1
Char. 278: 3 → 0

**Node 62:**
Char. 73: 0 → 1
Char. 131: 1 → 0
Char. 205: 0 → 1
Char. 273: 0 → 1
Char. 276: 0 → 1

**Node 63:**
Char. 0: 1 → 0
Char. 18: 0 → 1
Char. 37: 0 → 1
Char. 103: 0 → 1
Char. 106: 0 → 1
Char. 107: 0 → 1
Char. 126: 0 → 1
Char. 150: 0 → 1

**Node 64:**
Char. 29: 0 → 1
Char. 38: 1 → 2
Char. 39: 0 → 1
Char. 44: 1 → 0
Char. 49: 0 → 1
Char. 51: 0 → 1
Char. 57: 0 → 1
Char. 66: 0 → 1
Char. 67: 1 → 0
Char. 76: 0 → 1
Char. 80: 1 → 0
Char. 88: 1 → 0
Char. 95: 0 → 1
Char. 111: 1 → 0
Char. 120: 1 → 0
Char. 131: 0 → 1
Char. 135: 0 → 1
Char. 137: 0 → 1
Char. 147: 0 → 1
Char. 157: 0 → 1
Char. 158: 0 → 1
Char. 183: 1 → 2
Char. 186: 0 → 1
Char. 187: 0 → 1
Char. 194: 0 → 1
Char. 197: 0 → 1
Char. 209: 1 → 0
Char. 211: 0 → 1
Char. 240: 1 → 0
Char. 252: 0 → 1

**Node 66:**
Char. 133: 0 → 1

**Node 67:**
Char. 100: 0 → 1
Char. 113: 0 → 1

**Node 69:**
Char. 20: 1 → 0
Char. 38: 1 → 2
Char. 39: 0 → 1
Char. 57: 0 → 1
Char. 58: 0 → 1
Char. 60: 0 → 1
Char. 72: 1 → 2
Char. 83: 0 → 1
Char. 85: 1 → 0
Char. 88: 1 → 0
Char. 95: 0 → 1
Char. 104: 0 → 1
Char. 105: 0 → 1
Char. 106: 0 → 2
Char. 107: 0 → 1
Char. 109: 0 → 1
Char. 110: 0 → 1
Char. 146: 1 → 0
Char. 155: 0 → 2
Char. 183: 1 → 2

**Node 70:**
Char. 33: 1 → 0
Char. 38: 2 → 1
Char. 39: 1 → 0
Char. 42: 0 → 1
Char. 43: 0 → 1
Char. 46: 1 → 0
Char. 49: 1 → 0
Char. 71: 1 → 0
Char. 83: 0 → 2
Char. 101: 0 → 1
Char. 103: 0 → 1
Char. 106: 0 → 1
Char. 161: 0 → 1
Char. 163: 1 → 0
Char. 172: 0 → 2
Char. 174: 0 → 1
Char. 188: 0 → 1
Char. 203: 1 → 2

Char. 212: 0 → 1
Char. 216: 1 → 0
Char. 221: 1 → 0
Char. 235: 1 → 2
Char. 236: 0 → 1
Char. 238: 0 → 2
Char. 241: 0 → 1
Char. 242: 0 → 1
Char. 273: 0 → 1
Char. 274: 0 → 1
Char. 275: 1 → 0

**Node 71:**
Char. 52: 0 → 1
Char. 75: 0 → 1
Char. 84: 1 → 0
Char. 121: 0 → 1
Char. 180: 1 → 0
Char. 230: 0 → 1

**Node 72:**
Char. 0: 1 → 0
Char. 5: 1 → 0
Char. 20: 1 → 0
Char. 33: 1 → 0
Char. 44: 1 → 0
Char. 60: 0 → 1
Char. 66: 0 → 1
Char. 72: 1 → 2
Char. 78: 1 → 0
Char. 84: 1 → 0
Char. 88: 1 → 0
Char. 93: 1 → 0
Char. 112: 1 → 0
Char. 123: 1 → 0
Char. 129: 0 → 1
Char. 154: 1 → 0
Char. 159: 1 → 0
Char. 163: 1 → 0
Char. 169: 1 → 0
Char. 170: 0 → 1
Char. 192: 1 → 0
Char. 197: 0 → 1
Char. 202: 1 → 0
Char. 207: 1 → 0
Char. 209: 1 → 0
Char. 221: 1 → 0

**Node 73:**
Char. 33: 1 → 0
Char. 83: 0 → 1
Char. 85: 1 → 0
Char. 107: 0 → 1

**Node 74:**
Char. 2: 0 → 1
Char. 6: 0 → 1
Char. 186: 0 → 1
Char. 198: 0 → 1
Char. 200: 0 → 1
Char. 220: 0 → 1
Char. 239: 1 → 0

**Node 75:**
Char. 98: 1 → 0
Char. 113: 0 → 1
Char. 181: 0 → 1
Char. 206: 0 → 2

**Node 76:**
Char. 35: 0 → 1
Char. 102: 0 → 1
Char. 103: 0 → 1
Char. 184: 0 → 1
Char. 210: 0 → 1
Char. 223: 0 → 1
Char. 229: 0 → 1

**Node 77:**
Char. 17: 0 → 1
Char. 42: 0 → 1
Char. 251: 0 → 1
Char. 264: 0 → 1

**Node 78:**
Char. 41: 0 → 1
Char. 61: 1 → 2
Char. 112: 1 → 0
Char. 138: 0 → 1
Char. 146: 1 → 0
Char. 155: 0 → 1
Char. 192: 1 → 2
Char. 224: 0 → 1
Char. 226: 0 → 1
Char. 227: 0 → 1
Char. 233: 0 → 1
Char. 267: 01 → 2

**Node 79:**
Char. 61: 1 → 3
Char. 90: 0 → 1
Char. 91: 0 → 1
Char. 155: 0 → 1
Char. 209: 1 → 0

## ANALYSIS 46
**(ALL TAXA, IMPLIED WEIGHTING, K = 3.75)**

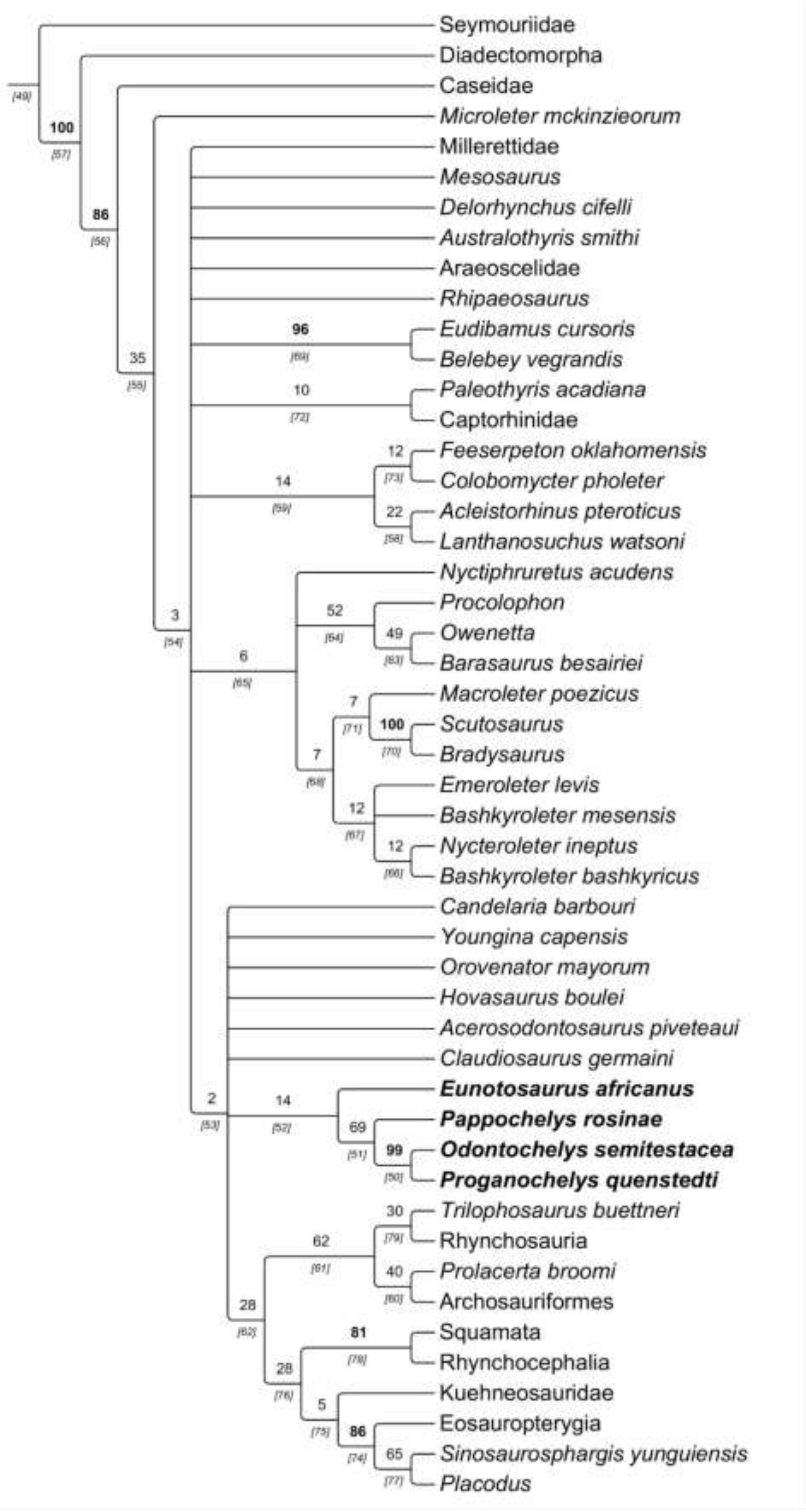

***Proganochelys quenstedti*:**
Char. 8: 0 → 1
Char. 11: 0 → 1
Char. 106: 0 → 1
Char. 108: 1 → 0
Char. 128: 0 → 1
Char. 175: 0 → 1
Char. 202: 1 → 0
Char. 207: 1 → 0
Char. 209: 1 → 0
Char. 244: 0 → 1
Char. 248: 0 → 1
Char. 250: 0 → 1
Char. 252: 1 → 0
Char. 262: 0 → 1

***Pappochelys rosinae*:**
Char. 0: 2 → 0
Char. 1: 0 → 1
Char. 5: 1 → 0
Char. 12: 0 → 1
Char. 41: 0 → 1
Char. 49: 0 → 1
Char. 75: 0 → 1
Char. 169: 1 → 0
Char. 260: 2 → 0

***Odontochelys semitestacea*:**
Char. 43: 0 → 1

***Eunotosaurus africanus*:**
Char. 19: 0 → 1
Char. 43: 0 → 1
Char. 59: 1 → 0
Char. 72: 2 → 1
Char. 76: 0 → 1
Char. 97: 1 → 0
Char. 103: 0 → 1
Char. 153: 1 → 0
Char. 191: 1 → 0
Char. 192: 1 → 0
Char. 202: 1 → 0
Char. 211: 0 → 1
Char. 222: 1 → 0
Char. 237: 1 → 0
Char. 248: 0 → 1
Char. 249: 0 → 2
Char. 250: 0 → 1
Char. 263: 0 → 1
Char. 273: 0 → 1
Char. 274: 0 → 1
Char. 275: 0 → 1
Char. 276: 0 → 1
Char. 277: 0 → 1

***Acerosodontosaurus piveteaui*:**
Char. 78: 1 → 0
Char. 81: 1 → 0
Char. 127: 1 → 0
Char. 128: 0 → 1
Char. 155: 0 → 1
Char. 206: 0 → 2
Char. 208: 0 → 1
Char. 278: 03 → 1

***Acleistorhinus pteroticus*:**
Char. 21: 0 → 1
Char. 146: 1 → 0

**Araeoscelidae:**
Char. 0: 0 → 1
Char. 27: 0 → 1
Char. 28: 0 → 1
Char. 29: 1 → 0
Char. 38: 1 → 0
Char. 40: 2 → 0
Char. 43: 0 → 1
Char. 50: 1 → 0
Char. 57: 1 → 0
Char. 59: 0 → 1
Char. 60: 0 → 1
Char. 67: 0 → 1
Char. 72: 1 → 2
Char. 84: 1 → 0
Char. 89: 0 → 1
Char. 106: 0 → 1
Char. 111: 0 → 1
Char. 116: 1 → 0
Char. 117: 01 → 2
Char. 120: 0 → 1
Char. 154: 1 → 0
Char. 159: 1 → 0
Char. 166: 1 → 0
Char. 169: 1 → 0
Char. 170: 0 → 1
Char. 193: 0 → 1
Char. 197: 0 → 1
Char. 222: 0 → 1
Char. 224: 0 → 1
Char. 234: 0 → 1
Char. 237: 0 → 1
Char. 239: 0 → 1
Char. 266: 0 → 1
Char. 278: 3 → 1

**Archosauriformes:**
Char. 32: 0 → 1
Char. 94: 1 → 0
Char. 112: 1 → 0
Char. 152: 0 → 1
Char. 154: 01 → 2
Char. 166: 1 → 0
Char. 171: 0 → 1
Char. 185: 0 → 1
Char. 201: 0 → 1
Char. 204: 0 → 1
Char. 218: 0 → 3
Char. 224: 0 → 1
Char. 242: 0 → 1

***Australothyris smithi*:**
Char. 24: 0 → 1
Char. 34: 0 → 1
Char. 55: 1 → 0
Char. 57: 1 → 0
Char. 71: 1 → 0
Char. 79: 0 → 1
Char. 83: 0 → 1
Char. 85: 1 → 0
Char. 98: 1 → 0
Char. 100: 0 → 1
Char. 103: 0 → 1
Char. 110: 0 → 1
Char. 123: 1 → 0
Char. 129: 0 → 1
Char. 131: 0 → 1
Char. 132: 1 → 0
Char. 144: 1 → 0
Char. 147: 0 → 1
Char. 149: 1 → 0
Char. 150: 0 → 1
Char. 159: 1 → 0
Char. 192: 1 → 0

***Barasaurus besairiei*:**
Char. 33: 1 → 0

***Bashkyroleter bashkyricus*:**
Char. 275: 1 → 0

***Bashkyroleter mesensis*:**
Char. 12: 0 → 1
Char. 66: 1 → 0
Char. 169: 1 → 0

***Belebey vegrandis*:**
Char. 154: 1 → 0

***Bradysaurus* spp.:**
Char. 19: 0 → 1
Char. 25: 1 → 0
Char. 73: 0 → 1
Char. 79: 0 → 1
Char. 135: 1 → 0
Char. 249: 0 → 1

***Candelaria barbouri*:**
Char. 0: 1 → 0
Char. 1: 0 → 1
Char. 5: 1 → 0
Char. 8: 0 → 1
Char. 15: 0 → 1
Char. 33: 1 → 2
Char. 49: 0 → 1
Char. 55: 1 → 0
Char. 59: 1 → 0
Char. 62: 1 → 0
Char. 72: 2 → 1
Char. 76: 0 → 1
Char. 79: 0 → 2
Char. 83: 0 → 1
Char. 88: 1 → 0
Char. 92: 1 → 0
Char. 95: 0 → 1
Char. 126: 0 → 1
Char. 132: 1 → 0
Char. 154: 1 → 2
Char. 159: 1 → 0
Char. 169: 1 → 0
Char. 265: 1 → 0
Char. 275: 0 → 1
Char. 276: 0 → 1
Char. 277: 0 → 1

**Captorhinidae:**
Char. 3: 0 → 1
Char. 25: 1 → 0
Char. 26: 0 → 1
Char. 73: 0 → 1
Char. 75: 0 → 1
Char. 83: 0 → 1
Char. 108: 1 → 0
Char. 180: 1 → 0
Char. 183: 1 → 0
Char. 201: 1 → 0
Char. 203: 1 → 2
Char. 216: 1 → 0

**Caseidae:**
Char. 38: 1 → 0
Char. 46: 1 → 0
Char. 50: 1 → 0
Char. 170: 0 → 1
Char. 194: 0 → 1
Char. 273: 0 → 1
Char. 274: 0 → 1
Char. 278: 3 → 2

***Claudiosaurus germaini*:**
Char. 24: 0 → 1
Char. 27: 0 → 1
Char. 34: 0 → 1
Char. 36: 0 → 1
Char. 56: 0 → 1
Char. 64: 0 → 1
Char. 70: 0 → 1
Char. 84: 1 → 0
Char. 105: 0 → 1
Char. 106: 0 → 1
Char. 117: 01 → 2
Char. 126: 0 → 1
Char. 127: 1 → 0
Char. 130: 0 → 1
Char. 131: 0 → 1
Char. 141: 1 → 0
Char. 144: 1 → 0
Char. 154: 1 → 0
Char. 166: 1 → 0
Char. 182: 0 → 1
Char. 187: 1 → 0
Char. 190: 0 → 1
Char. 199: 0 → 1
Char. 201: 0 → 1
Char. 203: 1 → 2
Char. 204: 0 → 1
Char. 222: 1 → 0
Char. 234: 1 → 0
Char. 267: 0 → 1
Char. 272: 2 → 1

***Colobomycter pholeter*:**
Char. 21: 0 → 1
Char. 25: 1 → 0
Char. 84: 1 → 0
Char. 154: 1 → 0
Char. 167: 0 → 1
Char. 267: 0 → 1

***Delorhynchus cifelli*:**
Char. 18: 0 → 1
Char. 20: 0 → 1
Char. 21: 0 → 1
Char. 24: 0 → 1
Char. 26: 0 → 1
Char. 28: 0 → 1
Char. 33: 0 → 2
Char. 39: 0 → 1
Char. 52: 0 → 1
Char. 100: 0 → 1
Char. 111: 0 → 1
Char. 116: 1 → 0
Char. 119: 1 → 0
Char. 131: 0 → 1
Char. 147: 0 → 1
Char. 156: 1 → 0
Char. 167: 0 → 1
Char. 189: 0 → 1
Char. 191: 0 → 1
Char. 204: 0 → 2
Char. 267: 0 → 1

**Diadectomorpha:**
Char. 0: 0 → 1
Char. 64: 0 → 1
Char. 70: 0 → 1
Char. 122: 0 → 1
Char. 123: 1 → 0
Char. 146: 1 → 0
Char. 275: 1 → 0
Char. 278: 3 → 0

***Emeroleter levis*:**
Char. 51: 1 → 0

**Eosauropterygia:**
Char. 166: 1 → 0
Char. 174: 0 → 1
Char. 178: 0 → 1
Char. 194: 0 → 2
Char. 272: 1 → 0

***Eudibamus cursoris*:**
Char. 154: 1 → 2

***Feeserpeton oklahomensis*:**
Char. 51: 0 → 1
Char. 157: 0 → 1
Char. 158: 0 → 1

***Hovasaurus boulei*:**
Char. 41: 0 → 1
Char. 43: 0 → 1
Char. 55: 1 → 0
Char. 72: 2 → 1
Char. 77: 0 → 2
Char. 78: 1 → 0
Char. 79: 0 → 1
Char. 93: 1 → 0
Char. 113: 0 → 1
Char. 138: 0 → 1

Char. 141: 1 → 0
Char. 146: 1 → 0
Char. 204: 0 → 2
Char. 206: 0 → 2
Char. 215: 0 → 1
Char. 219: 1 → 0
Char. 224: 0 → 1

**Kuehneosauridae:**
Char. 7: 0 → 1
Char. 24: 0 → 1
Char. 27: 0 → 1
Char. 43: 1 → 0
Char. 79: 0 → 2
Char. 107: 1 → 0
Char. 140: 1 → 0
Char. 147: 1 → 0
Char. 148: 1 → 0
Char. 245: 0 → 1
Char. 278: 0 → 3

***Lanthanosuchus watsoni*:**
Char. 25: 1 → 0
Char. 51: 0 → 1
Char. 76: 0 → 1
Char. 86: 0 → 1
Char. 98: 1 → 0
Char. 138: 0 → 1
Char. 144: 1 → 0
Char. 154: 1 → 2

***Macroleter poezicus*:**
Char. 9: 0 → 1
Char. 26: 0 → 1
Char. 66: 1 → 2
Char. 126: 0 → 1
Char. 134: 0 → 2
Char. 140: 0 → 1
Char. 146: 1 → 0
Char. 169: 1 → 0

***Mesosaurus* spp.:**
Char. 0: 0 → 1
Char. 2: 0 → 1
Char. 6: 0 → 1
Char. 8: 0 → 1
Char. 9: 0 → 1
Char. 13: 0 → 1
Char. 19: 0 → 1
Char. 26: 0 → 1
Char. 29: 1 → 0
Char. 33: 0 → 1
Char. 38: 1 → 0
Char. 41: 0 → 1
Char. 50: 1 → 0
Char. 67: 0 → 1
Char. 76: 0 → 1
Char. 83: 0 → 1
Char. 84: 1 → 0
Char. 85: 1 → 0
Char. 94: 0 → 1
Char. 107: 0 → 1
Char. 109: 0 → 1
Char. 111: 0 → 1
Char. 115: 0 → 1
Char. 146: 1 → 0
Char. 149: 1 → 0
Char. 159: 1 → 0
Char. 164: 0 → 1
Char. 166: 1 → 0
Char. 167: 0 → 1
Char. 176: 0 → 1
Char. 183: 1 → 0
Char. 184: 0 → 1
Char. 192: 1 → 0
Char. 199: 0 → 1
Char. 202: 0 → 1
Char. 204: 0 → 2
Char. 206: 0 → 1
Char. 207: 0 → 1
Char. 209: 0 → 1
Char. 217: 0 → 1
Char. 219: 0 → 1
Char. 220: 0 → 1
Char. 231: 0 → 1
Char. 260: 2 → 0
Char. 272: 2 → 0
Char. 278: 3 → 0

***Microleter mckinzieorum*:**
Char. 0: 0 → 1
Char. 18: 0 → 1
Char. 39: 0 → 1
Char. 57: 1 → 0
Char. 70: 0 → 1
Char. 76: 0 → 1
Char. 83: 0 → 1
Char. 94: 0 → 1
Char. 106: 0 → 1
Char. 110: 0 → 1
Char. 278: 3 → 1

**Millerettidae:**
Char. 24: 0 → 1
Char. 25: 1 → 0
Char. 44: 1 → 0
Char. 56: 0 → 1
Char. 57: 1 → 0
Char. 66: 0 → 2
Char. 78: 1 → 0
Char. 80: 1 → 0
Char. 84: 1 → 2
Char. 88: 1 → 0
Char. 96: 1 → 0
Char. 121: 0 → 1
Char. 124: 0 → 1
Char. 127: 0 → 1
Char. 135: 0 → 1
Char. 145: 0 → 1
Char. 159: 1 → 0
Char. 166: 1 → 0
Char. 180: 1 → 0
Char. 192: 1 → 0
Char. 202: 0 → 1
Char. 211: 0 → 1
Char. 230: 0 → 1
Char. 234: 0 → 1
Char. 248: 0 → 1
Char. 252: 0 → 1
Char. 253: 0 → 1

***Nycteroleter ineptus*:**
Char. 66: 1 → 0
Char. 278: 0 → 3

***Nyctiphruretus acudens*:**
Char. 0: 0 → 1
Char. 21: 0 → 1
Char. 33: 1 → 2
Char. 41: 0 → 1
Char. 66: 1 → 2
Char. 81: 1 → 0
Char. 83: 0 → 1
Char. 84: 1 → 2
Char. 94: 0 → 1
Char. 166: 1 → 0
Char. 224: 0 → 1
Char. 235: 1 → 0
Char. 266: 0 → 1
Char. 272: 2 → 1
Char. 276: 0 → 1

***Orovenator mayorum*:**
Char. 8: 0 → 1
Char. 24: 0 → 1
Char. 33: 1 → 0
Char. 36: 0 → 1
Char. 50: 0 → 1
Char. 92: 1 → 0
Char. 94: 1 → 0
Char. 135: 1 → 0
Char. 159: 1 → 0
Char. 160: 0 → 1
Char. 165: 0 → 1
Char. 278: 03 → 1

***Owenetta* spp.:**
Char. 142: 1 → 0
Char. 169: 1 → 0

***Paleothyris acadiana*:**
Char. 38: 1 → 0
Char. 50: 1 → 0
Char. 66: 1 → 2
Char. 102: 0 → 1
Char. 146: 1 → 0
Char. 237: 0 → 1
Char. 239: 0 → 1

***Placodus* spp.:**
Char. 0: 1 → 2
Char. 9: 0 → 1
Char. 12: 0 → 1
Char. 13: 0 → 1
Char. 19: 0 → 1
Char. 31: 0 → 1
Char. 46: 1 → 0
Char. 57: 0 → 1
Char. 78: 1 → 0
Char. 93: 1 → 0
Char. 102: 1 → 2
Char. 109: 1 → 0
Char. 140: 1 → 0
Char. 155: 0 → 1
Char. 163: 1 → 0
Char. 164: 0 → 1

***Procolophon* spp.:**
Char. 41: 0 → 1
Char. 69: 0 → 1
Char. 79: 0 → 1
Char. 83: 0 → 1
Char. 86: 0 → 1
Char. 88: 0 → 1
Char. 101: 0 → 1
Char. 117: 1 → 0
Char. 141: 0 → 1
Char. 149: 1 → 0
Char. 203: 1 → 2
Char. 204: 0 → 1
Char. 215: 0 → 1
Char. 230: 0 → 1
Char. 235: 1 → 2
Char. 237: 0 → 1
Char. 238: 0 → 1
Char. 272: 2 → 1
Char. 278: 3 → 0

***Prolacerta broomi*:**
Char. 58: 1 → 0
Char. 66: 1 → 0
Char. 67: 1 → 0
Char. 80: 1 → 0
Char. 139: 1 → 0
Char. 147: 1 → 0
Char. 192: 1 → 0
Char. 203: 1 → 2
Char. 206: 0 → 12

***Rhipaeosaurus* spp.:**
Char. 172: 0 → 1
Char. 183: 1 → 2
Char. 194: 0 → 1
Char. 201: 1 → 0
Char. 211: 0 → 1
Char. 215: 0 → 1
Char. 226: 0 → 1
Char. 235: 1 → 0
Char. 252: 0 → 1
Char. 277: 0 → 1

**Rhynchocephalia:**
Char. 0: 1 → 2
Char. 24: 0 → 1
Char. 77: 0 → 1
Char. 88: 1 → 0
Char. 94: 1 → 0
Char. 139: 1 → 0
Char. 167: 1 → 0
Char. 205: 1 → 0

**Rhynchosauria:**
Char. 0: 1 → 0
Char. 7: 0 → 1
Char. 9: 0 → 1
Char. 26: 0 → 1
Char. 44: 1 → 0
Char. 68: 0 → 1
Char. 99: 1 → 0
Char. 150: 1 → 0
Char. 160: 0 → 1
Char. 161: 0 → 1
Char. 171: 0 → 2
Char. 223: 0 → 1
Char. 224: 0 → 1
Char. 241: 0 → 1

***Scutosaurus* spp.:**
Char. 175: 0 → 1
Char. 190: 0 → 1
Char. 218: 0 → 1
Char. 243: 0 → 2
Char. 244: 0 → 1
Char. 251: 0 → 1

***Sinosaurosphargis yunguiensis*:**
Char. 8: 0 → 1
Char. 30: 0 → 1
Char. 53: 0 → 1
Char. 89: 1 → 0
Char. 127: 1 → 0
Char. 150: 1 → 0
Char. 154: 0 → 2
Char. 167: 1 → 0
Char. 253: 0 → 1
Char. 255: 0 → 1

**Squamata:**
Char. 45: 0 → 1
Char. 79: 0 → 2
Char. 80: 1 → 0
Char. 92: 1 → 0
Char. 109: 1 → 0
Char. 160: 0 → 1
Char. 200: 0 → 1
Char. 245: 0 → 1

***Trilophosaurus buettneri*:**
Char. 5: 1 → 0
Char. 11: 0 → 1
Char. 55: 1 → 0
Char. 93: 1 → 0
Char. 104: 0 → 1
Char. 106: 0 → 1
Char. 113: 0 → 1
Char. 122: 0 → 1
Char. 136: 1 → 0
Char. 144: 1 → 0
Char. 157: 0 → 1
Char. 159: 1 → 0
Char. 177: 0 → 12
Char. 194: 0 → 1
Char. 203: 1 → 2
Char. 207: 1 → 0
Char. 208: 1 → 0
Char. 272: 1 → 0

***Youngina capensis*:**
Char. 5: 1 → 0
Char. 21: 0 → 1
Char. 25: 0 → 1
Char. 27: 0 → 1

Char. 38: 1 → 0
Char. 43: 0 → 1
Char. 44: 1 → 0
Char. 56: 0 → 1
Char. 75: 0 → 1
Char. 84: 1 → 0
Char. 92: 1 → 0
Char. 94: 1 → 0
Char. 134: 0 → 1
Char. 154: 1 → 0
Char. 163: 1 → 0
Char. 170: 0 → 1
Char. 211: 0 → 1
Char. 215: 0 → 1
Char. 224: 0 → 1
Char. 239: 0 → 1
Char. 267: 0 → 1

**Node 50:**
Char. 46: 1 → 0
Char. 88: 1 → 0
Char. 89: 1 → 0
Char. 93: 1 → 0
Char. 176: 0 → 1
Char. 195: 0 → 2
Char. 198: 0 → 1
Char. 246: 1 → 2
Char. 259: 0 → 1
Char. 265: 1 → 0
Char. 270: 0 → 1

**Node 51:**
Char. 65: 0 → 1
Char. 131: 0 → 1
Char. 184: 0 → 1
Char. 205: 0 → 1
Char. 210: 0 → 1
Char. 219: 1 → 0
Char. 241: 0 → 1
Char. 246: 0 → 1
Char. 254: 0 → 1
Char. 255: 0 → 1
Char. 256: 0 → 1
Char. 267: 0 → 1
Char. 268: 0 → 1
Char. 269: 0 → 1

**Node 52:**
Char. 0: 1 → 2
Char. 15: 0 → 1
Char. 21: 0 → 1
Char. 33: 1 → 2
Char. 44: 1 → 0
Char. 62: 1 → 0
Char. 64: 0 → 1
Char. 84: 1 → 2
Char. 130: 0 → 1
Char. 134: 0 → 2
Char. 147: 0 → 1
Char. 152: 0 → 1
Char. 155: 0 → 1
Char. 158: 0 → 1
Char. 161: 0 → 1
Char. 174: 0 → 1
Char. 181: 0 → 1
Char. 203: 1 → 2
Char. 204: 0 → 2
Char. 247: 0 → 1
Char. 251: 0 → 1
Char. 252: 0 → 1
Char. 253: 0 → 2

**Node 53:**
Char. 0: 0 → 1
Char. 20: 0 → 1
Char. 25: 1 → 0
Char. 29: 1 → 0
Char. 33: 0 → 1
Char. 40: 2 → 0
Char. 50: 1 → 0
Char. 57: 1 → 0
Char. 59: 0 → 1
Char. 62: 0 → 1
Char. 67: 0 → 1
Char. 72: 1 → 2
Char. 89: 0 → 1
Char. 94: 0 → 1
Char. 109: 0 → 1
Char. 111: 0 → 1
Char. 120: 0 → 1
Char. 127: 0 → 1
Char. 129: 0 → 1
Char. 135: 0 → 1
Char. 136: 0 → 1
Char. 139: 0 → 1
Char. 141: 0 → 1
Char. 179: 0 → 1
Char. 187: 0 → 1
Char. 188: 0 → 1
Char. 191: 0 → 1
Char. 193: 0 → 1
Char. 196: 0 → 1
Char. 201: 1 → 0
Char. 202: 0 → 1
Char. 207: 0 → 1
Char. 209: 0 → 1
Char. 219: 0 → 1
Char. 222: 0 → 1
Char. 234: 0 → 1
Char. 237: 0 → 1
Char. 265: 0 → 1
Char. 266: 0 → 1
Char. 275: 1 → 0

**Node 54:**
Char. 132: 0 → 1
Char. 166: 0 → 1
Char. 276: 1 → 0

**Node 55:**
Char. 29: 0 → 1
Char. 84: 0 → 1
Char. 88: 0 → 1
Char. 169: 0 → 1

**Node 56:**
Char. 72: 0 → 1
Char. 81: 0 → 1
Char. 93: 0 → 1
Char. 97: 0 → 1
Char. 104: 1 → 0
Char. 144: 0 → 1
Char. 173: 0 → 1
Char. 183: 0 → 1

**Node 58:**
Char. 95: 0 → 1
Char. 113: 0 → 1
Char. 114: 0 → 1

**Node 59:**
Char. 20: 0 → 1
Char. 47: 0 → 1
Char. 79: 0 → 1
Char. 110: 0 → 1
Char. 131: 0 → 1
Char. 137: 0 → 1
Char. 140: 0 → 2
Char. 147: 0 → 1

**Node 60:**
Char. 19: 0 → 1
Char. 92: 1 → 0

**Node 61:**
Char. 4: 0 → 1
Char. 15: 0 → 1
Char. 29: 0 → 1
Char. 213: 0 → 1
Char. 226: 0 → 2
Char. 228: 0 → 1
Char. 275: 0 → 1

**Node 62:**
Char. 43: 0 → 1
Char. 58: 0 → 1
Char. 61: 0 → 1
Char. 66: 0 → 1
Char. 69: 0 → 1
Char. 107: 0 → 1
Char. 126: 0 → 1
Char. 131: 0 → 1
Char. 134: 0 → 1
Char. 140: 0 → 1
Char. 147: 0 → 1
Char. 150: 0 → 1
Char. 167: 0 → 1
Char. 176: 0 → 1
Char. 190: 0 → 1
Char. 205: 0 → 1
Char. 208: 0 → 1
Char. 230: 0 → 1
Char. 239: 0 → 1
Char. 260: 2 → 1

**Node 63:**
Char. 73: 0 → 1
Char. 131: 1 → 0
Char. 205: 0 → 1
Char. 273: 0 → 1
Char. 276: 0 → 1

**Node 64:**
Char. 18: 0 → 1
Char. 25: 1 → 0
Char. 37: 0 → 1
Char. 103: 0 → 1
Char. 106: 0 → 1
Char. 107: 0 → 1
Char. 110: 0 → 1
Char. 126: 0 → 1
Char. 150: 0 → 1

**Node 65:**
Char. 20: 0 → 1
Char. 33: 0 → 1
Char. 38: 1 → 2
Char. 39: 0 → 1
Char. 44: 1 → 0
Char. 49: 0 → 1
Char. 51: 0 → 1
Char. 66: 0 → 1
Char. 76: 0 → 1
Char. 80: 1 → 0
Char. 88: 1 → 0
Char. 95: 0 → 1
Char. 131: 0 → 1
Char. 135: 0 → 1
Char. 137: 0 → 1
Char. 147: 0 → 1
Char. 157: 0 → 1
Char. 158: 0 → 1
Char. 183: 1 → 2
Char. 186: 0 → 1
Char. 187: 0 → 1
Char. 194: 0 → 1
Char. 197: 0 → 1
Char. 201: 1 → 0
Char. 211: 0 → 1
Char. 252: 0 → 1

**Node 66:**
Char. 87: 0 → 1
Char. 110: 0 → 1

**Node 67:**
Char. 79: 0 → 1
Char. 133: 0 → 1

**Node 68:**
Char. 100: 0 → 1
Char. 113: 0 → 1

**Node 69:**
Char. 38: 1 → 2
Char. 39: 0 → 1
Char. 50: 1 → 0
Char. 58: 0 → 1
Char. 59: 0 → 1
Char. 60: 0 → 1
Char. 72: 1 → 2
Char. 83: 0 → 1
Char. 85: 1 → 0
Char. 88: 1 → 0
Char. 95: 0 → 1
Char. 104: 0 → 1
Char. 105: 0 → 1
Char. 106: 0 → 2
Char. 107: 0 → 1
Char. 109: 0 → 1
Char. 110: 0 → 1
Char. 146: 1 → 0
Char. 155: 0 → 2
Char. 183: 1 → 2

**Node 70:**
Char. 33: 1 → 0
Char. 38: 2 → 1
Char. 39: 1 → 0
Char. 42: 0 → 1
Char. 43: 0 → 1
Char. 46: 1 → 0
Char. 49: 1 → 0
Char. 71: 1 → 0
Char. 83: 0 → 2
Char. 101: 0 → 1
Char. 103: 0 → 1
Char. 106: 0 → 1
Char. 161: 0 → 1
Char. 163: 1 → 0
Char. 172: 0 → 2
Char. 174: 0 → 1
Char. 188: 0 → 1
Char. 203: 1 → 2
Char. 212: 0 → 1
Char. 216: 1 → 0
Char. 235: 1 → 2
Char. 236: 0 → 1
Char. 238: 0 → 2
Char. 241: 0 → 1
Char. 242: 0 → 1
Char. 273: 0 → 1
Char. 274: 0 → 1
Char. 275: 1 → 0

**Node 71:**
Char. 52: 0 → 1
Char. 75: 0 → 1
Char. 84: 1 → 0
Char. 87: 0 → 1
Char. 110: 0 → 1
Char. 121: 0 → 1
Char. 180: 1 → 0
Char. 230: 0 → 1

**Node 72:**
Char. 29: 1 → 0
Char. 44: 1 → 0
Char. 59: 0 → 1
Char. 60: 0 → 1
Char. 66: 0 → 1
Char. 67: 0 → 1
Char. 72: 1 → 2
Char. 78: 1 → 0
Char. 84: 1 → 0
Char. 88: 1 → 0
Char. 93: 1 → 0
Char. 111: 0 → 1
Char. 123: 1 → 0
Char. 129: 0 → 1
Char. 154: 1 → 0

Char. 159: 1 → 0
Char. 169: 1 → 0
Char. 170: 0 → 1
Char. 192: 1 → 0
Char. 197: 0 → 1

**Node 73:**
Char. 83: 0 → 1
Char. 85: 1 → 0
Char. 107: 0 → 1

**Node 74:**
Char. 2: 0 → 1
Char. 6: 0 → 1
Char. 186: 0 → 1
Char. 198: 0 → 1
Char. 200: 0 → 1
Char. 220: 0 → 1
Char. 239: 1 → 0

**Node 75:**
Char. 98: 1 → 0
Char. 113: 0 → 1
Char. 181: 0 → 1
Char. 206: 0 → 2

**Node 76:**
Char. 35: 0 → 1
Char. 102: 0 → 1
Char. 103: 0 → 1
Char. 184: 0 → 1
Char. 201: 0 → 1
Char. 210: 0 → 1
Char. 223: 0 → 1
Char. 229: 0 → 1

**Node 77:**
Char. 17: 0 → 1
Char. 42: 0 → 1
Char. 251: 0 → 1
Char. 264: 0 → 1

**Node 78:**
Char. 41: 0 → 1
Char. 61: 1 → 2
Char. 112: 1 → 0
Char. 138: 0 → 1
Char. 146: 1 → 0
Char. 155: 0 → 1
Char. 192: 1 → 2
Char. 224: 0 → 1
Char. 226: 0 → 1
Char. 227: 0 → 1
Char. 233: 0 → 1
Char. 267: 01 → 2

**Node 79:**
Char. 61: 1 → 3
Char. 90: 0 → 1
Char. 91: 0 → 1
Char. 109: 1 → 0
Char. 155: 0 → 1
Char. 209: 1 → 0

**ANALYSIS 47**
**(ALL TAXA, IMPLIED WEIGHTING, K = 3.875)**

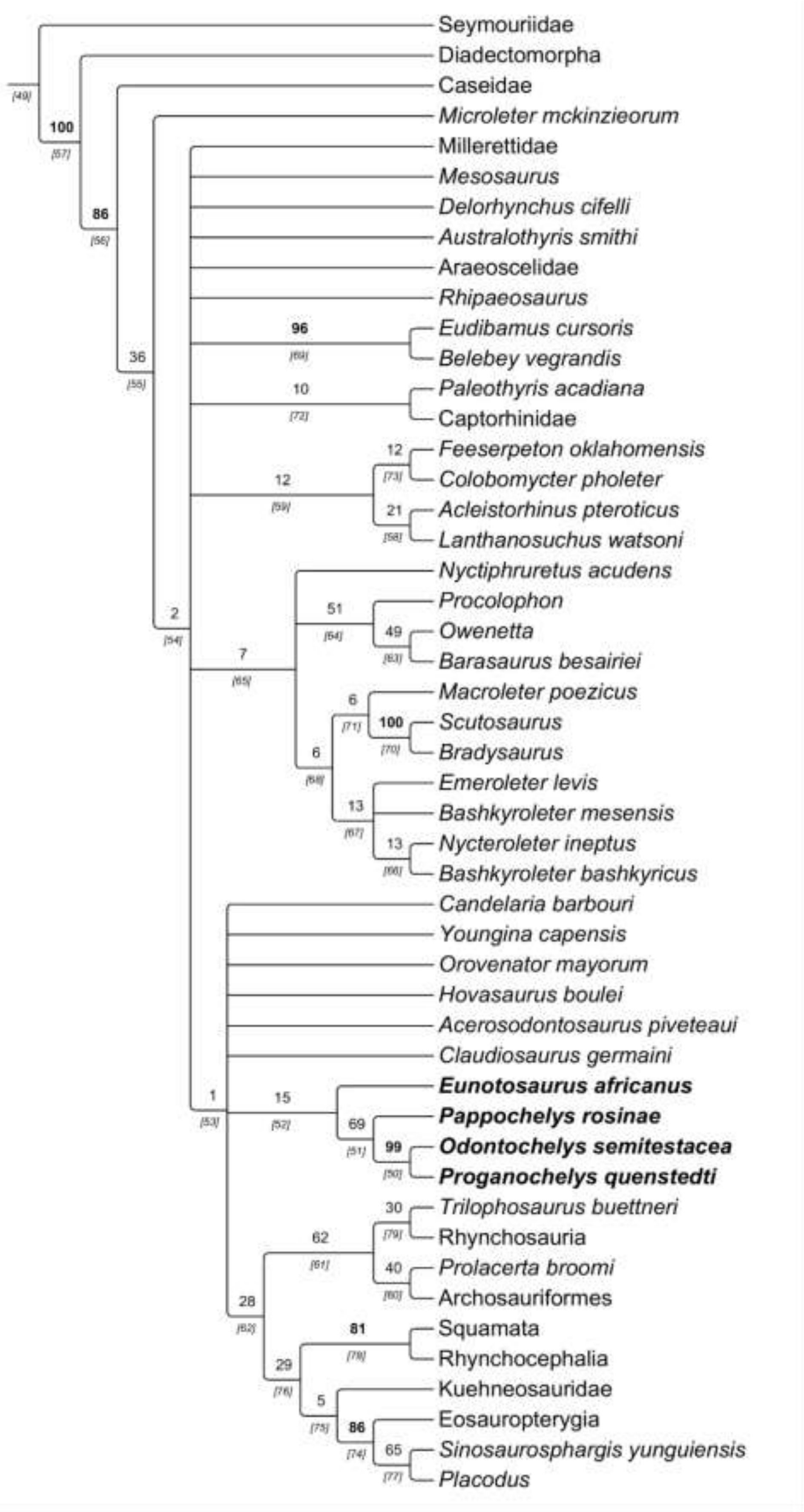

***Proganochelys quenstedti*:**
Char. 8: 0 → 1
Char. 11: 0 → 1
Char. 106: 0 → 1
Char. 108: 1 → 0
Char. 128: 0 → 1
Char. 175: 0 → 1
Char. 202: 1 → 0
Char. 207: 1 → 0
Char. 209: 1 → 0
Char. 244: 0 → 1
Char. 248: 0 → 1
Char. 250: 0 → 1
Char. 252: 1 → 0
Char. 262: 0 → 1

***Pappochelys rosinae*:**
Char. 0: 2 → 0
Char. 1: 0 → 1
Char. 5: 1 → 0
Char. 12: 0 → 1
Char. 41: 0 → 1
Char. 49: 0 → 1
Char. 75: 0 → 1
Char. 169: 1 → 0
Char. 260: 2 → 0

***Odontochelys semitestacea*:**
Char. 43: 0 → 1

***Eunotosaurus africanus*:**
Char. 19: 0 → 1
Char. 43: 0 → 1
Char. 59: 1 → 0
Char. 72: 2 → 1
Char. 76: 0 → 1
Char. 97: 1 → 0
Char. 103: 0 → 1
Char. 153: 1 → 0
Char. 191: 1 → 0
Char. 192: 1 → 0
Char. 202: 1 → 0
Char. 211: 0 → 1
Char. 222: 1 → 0
Char. 237: 1 → 0
Char. 248: 0 → 1
Char. 249: 0 → 2
Char. 250: 0 → 1
Char. 263: 0 → 1
Char. 273: 0 → 1
Char. 274: 0 → 1
Char. 275: 0 → 1
Char. 276: 0 → 1
Char. 277: 0 → 1

***Acerosodontosaurus piveteaui*:**
Char. 78: 1 → 0
Char. 81: 1 → 0
Char. 127: 1 → 0
Char. 128: 0 → 1
Char. 155: 0 → 1
Char. 206: 0 → 2
Char. 208: 0 → 1
Char. 278: 03 → 1

***Acleistorhinus pteroticus*:**
Char. 21: 0 → 1
Char. 146: 1 → 0

**Araeoscelidae:**
Char. 0: 0 → 1
Char. 27: 0 → 1
Char. 28: 0 → 1
Char. 29: 1 → 0
Char. 38: 1 → 0
Char. 40: 2 → 0
Char. 43: 0 → 1
Char. 50: 1 → 0
Char. 57: 1 → 0
Char. 59: 0 → 1
Char. 60: 0 → 1
Char. 67: 0 → 1
Char. 72: 1 → 2
Char. 84: 1 → 0
Char. 89: 0 → 1
Char. 106: 0 → 1
Char. 111: 0 → 1
Char. 116: 1 → 0
Char. 117: 01 → 2
Char. 120: 0 → 1
Char. 154: 1 → 0
Char. 159: 1 → 0
Char. 166: 1 → 0
Char. 169: 1 → 0
Char. 170: 0 → 1
Char. 193: 0 → 1
Char. 197: 0 → 1
Char. 222: 0 → 1
Char. 224: 0 → 1
Char. 234: 0 → 1
Char. 237: 0 → 1
Char. 239: 0 → 1
Char. 266: 0 → 1
Char. 278: 3 → 1

**Archosauriformes:**
Char. 32: 0 → 1
Char. 94: 1 → 0
Char. 112: 1 → 0
Char. 152: 0 → 1
Char. 154: 01 → 2
Char. 166: 1 → 0
Char. 171: 0 → 1
Char. 185: 0 → 1
Char. 201: 0 → 1
Char. 204: 0 → 1
Char. 218: 0 → 3
Char. 224: 0 → 1
Char. 242: 0 → 1

***Australothyris smithi*:**
Char. 24: 0 → 1
Char. 34: 0 → 1
Char. 55: 1 → 0
Char. 57: 1 → 0
Char. 71: 1 → 0
Char. 79: 0 → 1
Char. 83: 0 → 1
Char. 85: 1 → 0
Char. 98: 1 → 0
Char. 100: 0 → 1
Char. 103: 0 → 1
Char. 110: 0 → 1
Char. 123: 1 → 0
Char. 129: 0 → 1
Char. 131: 0 → 1
Char. 132: 1 → 0
Char. 144: 1 → 0
Char. 147: 0 → 1
Char. 149: 1 → 0
Char. 150: 0 → 1
Char. 159: 1 → 0
Char. 192: 1 → 0

***Barasaurus besairiei*:**
Char. 33: 1 → 0

***Bashkyroleter bashkyricus*:**
Char. 275: 1 → 0

***Bashkyroleter mesensis*:**
Char. 12: 0 → 1
Char. 66: 1 → 0
Char. 169: 1 → 0

***Belebey vegrandis*:**
Char. 154: 1 → 0

***Bradysaurus* spp.:**
Char. 19: 0 → 1
Char. 25: 1 → 0
Char. 73: 0 → 1
Char. 79: 0 → 1
Char. 135: 1 → 0
Char. 249: 0 → 1

***Candelaria barbouri*:**
Char. 0: 1 → 0
Char. 1: 0 → 1
Char. 5: 1 → 0
Char. 8: 0 → 1
Char. 15: 0 → 1
Char. 33: 1 → 2
Char. 49: 0 → 1
Char. 55: 1 → 0
Char. 59: 1 → 0
Char. 62: 1 → 0
Char. 72: 2 → 1
Char. 76: 0 → 1
Char. 79: 0 → 2
Char. 83: 0 → 1
Char. 88: 1 → 0
Char. 92: 1 → 0
Char. 95: 0 → 1
Char. 126: 0 → 1
Char. 132: 1 → 0
Char. 154: 1 → 2
Char. 159: 1 → 0
Char. 169: 1 → 0
Char. 265: 1 → 0
Char. 275: 0 → 1
Char. 276: 0 → 1
Char. 277: 0 → 1

**Captorhinidae:**
Char. 3: 0 → 1
Char. 25: 1 → 0
Char. 26: 0 → 1
Char. 73: 0 → 1
Char. 75: 0 → 1
Char. 83: 0 → 1
Char. 108: 1 → 0
Char. 180: 1 → 0
Char. 183: 1 → 0
Char. 201: 1 → 0
Char. 203: 1 → 2
Char. 216: 1 → 0

**Caseidae:**
Char. 38: 1 → 0
Char. 46: 1 → 0
Char. 50: 1 → 0
Char. 170: 0 → 1
Char. 194: 0 → 1
Char. 273: 0 → 1
Char. 274: 0 → 1
Char. 278: 3 → 2

***Claudiosaurus germaini*:**
Char. 24: 0 → 1
Char. 27: 0 → 1
Char. 34: 0 → 1
Char. 36: 0 → 1
Char. 56: 0 → 1
Char. 64: 0 → 1
Char. 70: 0 → 1
Char. 84: 1 → 0
Char. 105: 0 → 1
Char. 106: 0 → 1
Char. 117: 01 → 2
Char. 126: 0 → 1
Char. 127: 1 → 0
Char. 130: 0 → 1
Char. 131: 0 → 1
Char. 141: 1 → 0
Char. 144: 1 → 0
Char. 154: 1 → 0
Char. 166: 1 → 0
Char. 182: 0 → 1
Char. 187: 1 → 0
Char. 190: 0 → 1
Char. 199: 0 → 1
Char. 201: 0 → 1
Char. 203: 1 → 2
Char. 204: 0 → 1
Char. 222: 1 → 0
Char. 234: 1 → 0
Char. 267: 0 → 1
Char. 272: 2 → 1

***Colobomycter pholeter*:**
Char. 21: 0 → 1
Char. 25: 1 → 0
Char. 84: 1 → 0
Char. 154: 1 → 0
Char. 167: 0 → 1
Char. 267: 0 → 1

***Delorhynchus cifelli*:**
Char. 18: 0 → 1
Char. 20: 0 → 1
Char. 21: 0 → 1
Char. 24: 0 → 1
Char. 26: 0 → 1
Char. 28: 0 → 1
Char. 33: 0 → 2
Char. 39: 0 → 1
Char. 52: 0 → 1
Char. 100: 0 → 1
Char. 111: 0 → 1
Char. 116: 1 → 0
Char. 119: 1 → 0
Char. 131: 0 → 1
Char. 147: 0 → 1
Char. 156: 1 → 0
Char. 167: 0 → 1
Char. 189: 0 → 1
Char. 191: 0 → 1
Char. 204: 0 → 2
Char. 267: 0 → 1

**Diadectomorpha:**
Char. 0: 0 → 1
Char. 64: 0 → 1
Char. 70: 0 → 1
Char. 122: 0 → 1
Char. 123: 1 → 0
Char. 146: 1 → 0
Char. 275: 1 → 0
Char. 278: 3 → 0

***Emeroleter levis*:**
Char. 51: 1 → 0

**Eosauropterygia:**
Char. 166: 1 → 0
Char. 174: 0 → 1
Char. 178: 0 → 1
Char. 194: 0 → 2
Char. 272: 1 → 0

***Eudibamus cursoris*:**
Char. 154: 1 → 2

***Feeserpeton oklahomensis*:**
Char. 51: 0 → 1
Char. 157: 0 → 1
Char. 158: 0 → 1

***Hovasaurus boulei*:**
Char. 41: 0 → 1
Char. 43: 0 → 1
Char. 55: 1 → 0
Char. 72: 2 → 1
Char. 77: 0 → 2
Char. 78: 1 → 0
Char. 79: 0 → 1
Char. 93: 1 → 0
Char. 113: 0 → 1
Char. 138: 0 → 1

Char. 141: 1 → 0
Char. 146: 1 → 0
Char. 204: 0 → 2
Char. 206: 0 → 2
Char. 215: 0 → 1
Char. 219: 1 → 0
Char. 224: 0 → 1

**Kuehneosauridae:**
Char. 7: 0 → 1
Char. 24: 0 → 1
Char. 27: 0 → 1
Char. 43: 1 → 0
Char. 79: 0 → 2
Char. 107: 1 → 0
Char. 140: 1 → 0
Char. 147: 1 → 0
Char. 148: 1 → 0
Char. 245: 0 → 1
Char. 278: 0 → 3

***Lanthanosuchus watsoni*:**
Char. 25: 1 → 0
Char. 51: 0 → 1
Char. 76: 0 → 1
Char. 86: 0 → 1
Char. 98: 1 → 0
Char. 138: 0 → 1
Char. 144: 1 → 0
Char. 154: 1 → 2

***Macroleter poezicus*:**
Char. 9: 0 → 1
Char. 26: 0 → 1
Char. 66: 1 → 2
Char. 126: 0 → 1
Char. 134: 0 → 2
Char. 140: 0 → 1
Char. 146: 1 → 0
Char. 169: 1 → 0

***Mesosaurus* spp.:**
Char. 0: 0 → 1
Char. 2: 0 → 1
Char. 6: 0 → 1
Char. 8: 0 → 1
Char. 9: 0 → 1
Char. 13: 0 → 1
Char. 19: 0 → 1
Char. 26: 0 → 1
Char. 29: 1 → 0
Char. 33: 0 → 1
Char. 38: 1 → 0
Char. 41: 0 → 1
Char. 50: 1 → 0
Char. 67: 0 → 1
Char. 76: 0 → 1
Char. 83: 0 → 1
Char. 84: 1 → 0
Char. 85: 1 → 0
Char. 94: 0 → 1
Char. 107: 0 → 1
Char. 109: 0 → 1
Char. 111: 0 → 1
Char. 115: 0 → 1
Char. 146: 1 → 0
Char. 149: 1 → 0
Char. 159: 1 → 0
Char. 164: 0 → 1
Char. 166: 1 → 0
Char. 167: 0 → 1
Char. 176: 0 → 1
Char. 183: 1 → 0
Char. 184: 0 → 1
Char. 192: 1 → 0
Char. 199: 0 → 1
Char. 202: 0 → 1
Char. 204: 0 → 2
Char. 206: 0 → 1
Char. 207: 0 → 1
Char. 209: 0 → 1
Char. 217: 0 → 1
Char. 219: 0 → 1
Char. 220: 0 → 1
Char. 231: 0 → 1
Char. 260: 2 → 0
Char. 272: 2 → 0
Char. 278: 3 → 0

***Microleter mckinzieorum*:**
Char. 0: 0 → 1
Char. 18: 0 → 1
Char. 39: 0 → 1
Char. 57: 1 → 0
Char. 70: 0 → 1
Char. 76: 0 → 1
Char. 83: 0 → 1
Char. 94: 0 → 1
Char. 106: 0 → 1
Char. 110: 0 → 1
Char. 278: 3 → 1

**Millerettidae:**
Char. 24: 0 → 1
Char. 25: 1 → 0
Char. 44: 1 → 0
Char. 56: 0 → 1
Char. 57: 1 → 0
Char. 66: 0 → 2
Char. 78: 1 → 0
Char. 80: 1 → 0
Char. 84: 1 → 2
Char. 88: 1 → 0
Char. 96: 1 → 0
Char. 121: 0 → 1
Char. 124: 0 → 1
Char. 127: 0 → 1
Char. 135: 0 → 1
Char. 145: 0 → 1
Char. 159: 1 → 0
Char. 166: 1 → 0
Char. 180: 1 → 0
Char. 192: 1 → 0
Char. 202: 0 → 1
Char. 211: 0 → 1
Char. 230: 0 → 1
Char. 234: 0 → 1
Char. 248: 0 → 1
Char. 252: 0 → 1
Char. 253: 0 → 1

***Nycteroleter ineptus*:**
Char. 66: 1 → 0
Char. 278: 0 → 3

***Nyctiphruretus acudens*:**
Char. 0: 0 → 1
Char. 21: 0 → 1
Char. 33: 1 → 2
Char. 41: 0 → 1
Char. 66: 1 → 2
Char. 81: 1 → 0
Char. 83: 0 → 1
Char. 84: 1 → 2
Char. 94: 0 → 1
Char. 166: 1 → 0
Char. 224: 0 → 1
Char. 235: 1 → 0
Char. 266: 0 → 1
Char. 272: 2 → 1
Char. 276: 0 → 1

***Orovenator mayorum*:**
Char. 8: 0 → 1
Char. 24: 0 → 1
Char. 33: 1 → 0
Char. 36: 0 → 1
Char. 50: 0 → 1
Char. 92: 1 → 0
Char. 94: 1 → 0
Char. 135: 1 → 0
Char. 159: 1 → 0
Char. 160: 0 → 1
Char. 165: 0 → 1
Char. 278: 03 → 1

***Owenetta* spp.:**
Char. 142: 1 → 0
Char. 169: 1 → 0

***Paleothyris acadiana*:**
Char. 38: 1 → 0
Char. 50: 1 → 0
Char. 66: 1 → 2
Char. 102: 0 → 1
Char. 146: 1 → 0
Char. 237: 0 → 1
Char. 239: 0 → 1

***Placodus* spp.:**
Char. 0: 1 → 2
Char. 9: 0 → 1
Char. 12: 0 → 1
Char. 13: 0 → 1
Char. 19: 0 → 1
Char. 31: 0 → 1
Char. 46: 1 → 0
Char. 57: 0 → 1
Char. 78: 1 → 0
Char. 93: 1 → 0
Char. 102: 1 → 2
Char. 109: 1 → 0
Char. 140: 1 → 0
Char. 155: 0 → 1
Char. 163: 1 → 0
Char. 164: 0 → 1

***Procolophon* spp.:**
Char. 41: 0 → 1
Char. 69: 0 → 1
Char. 79: 0 → 1
Char. 83: 0 → 1
Char. 86: 0 → 1
Char. 88: 0 → 1
Char. 101: 0 → 1
Char. 117: 1 → 0
Char. 141: 0 → 1
Char. 149: 1 → 0
Char. 203: 1 → 2
Char. 204: 0 → 1
Char. 215: 0 → 1
Char. 230: 0 → 1
Char. 235: 1 → 2
Char. 237: 0 → 1
Char. 238: 0 → 1
Char. 272: 2 → 1
Char. 278: 3 → 0

***Prolacerta broomi*:**
Char. 58: 1 → 0
Char. 66: 1 → 0
Char. 67: 1 → 0
Char. 80: 1 → 0
Char. 139: 1 → 0
Char. 147: 1 → 0
Char. 192: 1 → 0
Char. 203: 1 → 2
Char. 206: 0 → 12

***Rhipaeosaurus* spp.:**
Char. 172: 0 → 1
Char. 183: 1 → 2
Char. 194: 0 → 1
Char. 201: 1 → 0
Char. 211: 0 → 1
Char. 215: 0 → 1
Char. 226: 0 → 1
Char. 235: 1 → 0
Char. 252: 0 → 1
Char. 277: 0 → 1

**Rhynchocephalia:**
Char. 0: 1 → 2
Char. 24: 0 → 1
Char. 77: 0 → 1
Char. 88: 1 → 0
Char. 94: 1 → 0
Char. 139: 1 → 0
Char. 167: 1 → 0
Char. 205: 1 → 0

**Rhynchosauria:**
Char. 0: 1 → 0
Char. 7: 0 → 1
Char. 9: 0 → 1
Char. 26: 0 → 1
Char. 44: 1 → 0
Char. 68: 0 → 1
Char. 99: 1 → 0
Char. 150: 1 → 0
Char. 160: 0 → 1
Char. 161: 0 → 1
Char. 171: 0 → 2
Char. 223: 0 → 1
Char. 224: 0 → 1
Char. 241: 0 → 1

***Scutosaurus* spp.:**
Char. 175: 0 → 1
Char. 190: 0 → 1
Char. 218: 0 → 1
Char. 243: 0 → 2
Char. 244: 0 → 1
Char. 251: 0 → 1

***Sinosaurosphargis yunguiensis*:**
Char. 8: 0 → 1
Char. 30: 0 → 1
Char. 53: 0 → 1
Char. 89: 1 → 0
Char. 127: 1 → 0
Char. 150: 1 → 0
Char. 154: 0 → 2
Char. 167: 1 → 0
Char. 253: 0 → 1
Char. 255: 0 → 1

**Squamata:**
Char. 45: 0 → 1
Char. 79: 0 → 2
Char. 80: 1 → 0
Char. 92: 1 → 0
Char. 109: 1 → 0
Char. 160: 0 → 1
Char. 200: 0 → 1
Char. 245: 0 → 1

***Trilophosaurus buettneri*:**
Char. 5: 1 → 0
Char. 11: 0 → 1
Char. 55: 1 → 0
Char. 93: 1 → 0
Char. 104: 0 → 1
Char. 106: 0 → 1
Char. 113: 0 → 1
Char. 122: 0 → 1
Char. 136: 1 → 0
Char. 144: 1 → 0
Char. 157: 0 → 1
Char. 159: 1 → 0
Char. 177: 0 → 12
Char. 194: 0 → 1
Char. 203: 1 → 2
Char. 207: 1 → 0
Char. 208: 1 → 0
Char. 272: 1 → 0

***Youngina capensis*:**
Char. 5: 1 → 0
Char. 21: 0 → 1
Char. 25: 0 → 1
Char. 27: 0 → 1

Char. 38: 1 → 0
Char. 43: 0 → 1
Char. 44: 1 → 0
Char. 56: 0 → 1
Char. 75: 0 → 1
Char. 84: 1 → 0
Char. 92: 1 → 0
Char. 94: 1 → 0
Char. 134: 0 → 1
Char. 154: 1 → 0
Char. 163: 1 → 0
Char. 170: 0 → 1
Char. 211: 0 → 1
Char. 215: 0 → 1
Char. 224: 0 → 1
Char. 239: 0 → 1
Char. 267: 0 → 1

**Node 50:**
Char. 46: 1 → 0
Char. 88: 1 → 0
Char. 89: 1 → 0
Char. 93: 1 → 0
Char. 176: 0 → 1
Char. 195: 0 → 2
Char. 198: 0 → 1
Char. 246: 1 → 2
Char. 259: 0 → 1
Char. 265: 1 → 0
Char. 270: 0 → 1

**Node 51:**
Char. 65: 0 → 1
Char. 131: 0 → 1
Char. 184: 0 → 1
Char. 205: 0 → 1
Char. 210: 0 → 1
Char. 219: 1 → 0
Char. 241: 0 → 1
Char. 246: 0 → 1
Char. 254: 0 → 1
Char. 255: 0 → 1
Char. 256: 0 → 1
Char. 267: 0 → 1
Char. 268: 0 → 1
Char. 269: 0 → 1

**Node 52:**
Char. 0: 1 → 2
Char. 15: 0 → 1
Char. 21: 0 → 1
Char. 33: 1 → 2
Char. 44: 1 → 0
Char. 62: 1 → 0
Char. 64: 0 → 1
Char. 84: 1 → 2
Char. 130: 0 → 1
Char. 134: 0 → 2
Char. 147: 0 → 1
Char. 152: 0 → 1
Char. 155: 0 → 1
Char. 158: 0 → 1
Char. 161: 0 → 1
Char. 174: 0 → 1
Char. 181: 0 → 1
Char. 203: 1 → 2
Char. 204: 0 → 2
Char. 247: 0 → 1
Char. 251: 0 → 1
Char. 252: 0 → 1
Char. 253: 0 → 2

**Node 53:**
Char. 0: 0 → 1
Char. 20: 0 → 1
Char. 25: 1 → 0
Char. 29: 1 → 0
Char. 33: 0 → 1
Char. 40: 2 → 0
Char. 50: 1 → 0
Char. 57: 1 → 0
Char. 59: 0 → 1
Char. 62: 0 → 1
Char. 67: 0 → 1
Char. 72: 1 → 2
Char. 89: 0 → 1
Char. 94: 0 → 1
Char. 109: 0 → 1
Char. 111: 0 → 1
Char. 120: 0 → 1
Char. 127: 0 → 1
Char. 129: 0 → 1
Char. 135: 0 → 1
Char. 136: 0 → 1
Char. 139: 0 → 1
Char. 141: 0 → 1
Char. 179: 0 → 1
Char. 187: 0 → 1
Char. 188: 0 → 1
Char. 191: 0 → 1
Char. 193: 0 → 1
Char. 196: 0 → 1
Char. 201: 1 → 0
Char. 202: 0 → 1
Char. 207: 0 → 1
Char. 209: 0 → 1
Char. 219: 0 → 1
Char. 222: 0 → 1
Char. 234: 0 → 1
Char. 237: 0 → 1
Char. 265: 0 → 1
Char. 266: 0 → 1
Char. 275: 1 → 0

**Node 54:**
Char. 132: 0 → 1
Char. 166: 0 → 1
Char. 276: 1 → 0

**Node 55:**
Char. 29: 0 → 1
Char. 84: 0 → 1
Char. 88: 0 → 1
Char. 169: 0 → 1

**Node 56:**
Char. 72: 0 → 1
Char. 81: 0 → 1
Char. 93: 0 → 1
Char. 97: 0 → 1
Char. 104: 1 → 0
Char. 144: 0 → 1
Char. 173: 0 → 1
Char. 183: 0 → 1

**Node 58:**
Char. 95: 0 → 1
Char. 113: 0 → 1
Char. 114: 0 → 1

**Node 59:**
Char. 20: 0 → 1
Char. 47: 0 → 1
Char. 79: 0 → 1
Char. 110: 0 → 1
Char. 131: 0 → 1
Char. 137: 0 → 1
Char. 140: 0 → 2
Char. 147: 0 → 1

**Node 60:**
Char. 19: 0 → 1
Char. 92: 1 → 0

**Node 61:**
Char. 4: 0 → 1
Char. 15: 0 → 1
Char. 29: 0 → 1
Char. 213: 0 → 1
Char. 226: 0 → 2
Char. 228: 0 → 1
Char. 275: 0 → 1

**Node 62:**
Char. 43: 0 → 1
Char. 58: 0 → 1
Char. 61: 0 → 1
Char. 66: 0 → 1
Char. 69: 0 → 1
Char. 107: 0 → 1
Char. 126: 0 → 1
Char. 131: 0 → 1
Char. 134: 0 → 1
Char. 140: 0 → 1
Char. 147: 0 → 1
Char. 150: 0 → 1
Char. 167: 0 → 1
Char. 176: 0 → 1
Char. 190: 0 → 1
Char. 205: 0 → 1
Char. 208: 0 → 1
Char. 230: 0 → 1
Char. 239: 0 → 1
Char. 260: 2 → 1

**Node 63:**
Char. 73: 0 → 1
Char. 131: 1 → 0
Char. 205: 0 → 1
Char. 273: 0 → 1
Char. 276: 0 → 1

**Node 64:**
Char. 18: 0 → 1
Char. 25: 1 → 0
Char. 37: 0 → 1
Char. 103: 0 → 1
Char. 106: 0 → 1
Char. 107: 0 → 1
Char. 110: 0 → 1
Char. 126: 0 → 1
Char. 150: 0 → 1

**Node 65:**
Char. 20: 0 → 1
Char. 33: 0 → 1
Char. 38: 1 → 2
Char. 39: 0 → 1
Char. 44: 1 → 0
Char. 49: 0 → 1
Char. 51: 0 → 1
Char. 66: 0 → 1
Char. 76: 0 → 1
Char. 80: 1 → 0
Char. 88: 1 → 0
Char. 95: 0 → 1
Char. 131: 0 → 1
Char. 135: 0 → 1
Char. 137: 0 → 1
Char. 147: 0 → 1
Char. 157: 0 → 1
Char. 158: 0 → 1
Char. 183: 1 → 2
Char. 186: 0 → 1
Char. 187: 0 → 1
Char. 194: 0 → 1
Char. 197: 0 → 1
Char. 201: 1 → 0
Char. 211: 0 → 1
Char. 252: 0 → 1

**Node 66:**
Char. 87: 0 → 1
Char. 110: 0 → 1

**Node 67:**
Char. 79: 0 → 1
Char. 133: 0 → 1

**Node 68:**
Char. 100: 0 → 1
Char. 113: 0 → 1

**Node 69:**
Char. 38: 1 → 2
Char. 39: 0 → 1
Char. 50: 1 → 0
Char. 58: 0 → 1
Char. 59: 0 → 1
Char. 60: 0 → 1
Char. 72: 1 → 2
Char. 83: 0 → 1
Char. 85: 1 → 0
Char. 88: 1 → 0
Char. 95: 0 → 1
Char. 104: 0 → 1
Char. 105: 0 → 1
Char. 106: 0 → 2
Char. 107: 0 → 1
Char. 109: 0 → 1
Char. 110: 0 → 1
Char. 146: 1 → 0
Char. 155: 0 → 2
Char. 183: 1 → 2

**Node 70:**
Char. 33: 1 → 0
Char. 38: 2 → 1
Char. 39: 1 → 0
Char. 42: 0 → 1
Char. 43: 0 → 1
Char. 46: 1 → 0
Char. 49: 1 → 0
Char. 71: 1 → 0
Char. 83: 0 → 2
Char. 101: 0 → 1
Char. 103: 0 → 1
Char. 106: 0 → 1
Char. 161: 0 → 1
Char. 163: 1 → 0
Char. 172: 0 → 2
Char. 174: 0 → 1
Char. 188: 0 → 1
Char. 203: 1 → 2
Char. 212: 0 → 1
Char. 216: 1 → 0
Char. 235: 1 → 2
Char. 236: 0 → 1
Char. 238: 0 → 2
Char. 241: 0 → 1
Char. 242: 0 → 1
Char. 273: 0 → 1
Char. 274: 0 → 1
Char. 275: 1 → 0

**Node 71:**
Char. 52: 0 → 1
Char. 75: 0 → 1
Char. 84: 1 → 0
Char. 87: 0 → 1
Char. 110: 0 → 1
Char. 121: 0 → 1
Char. 180: 1 → 0
Char. 230: 0 → 1

**Node 72:**
Char. 29: 1 → 0
Char. 44: 1 → 0
Char. 59: 0 → 1
Char. 60: 0 → 1
Char. 66: 0 → 1
Char. 67: 0 → 1
Char. 72: 1 → 2
Char. 78: 1 → 0
Char. 84: 1 → 0
Char. 88: 1 → 0
Char. 93: 1 → 0
Char. 111: 0 → 1
Char. 123: 1 → 0
Char. 129: 0 → 1
Char. 154: 1 → 0

Char. 159: 1 → 0
Char. 169: 1 → 0
Char. 170: 0 → 1
Char. 192: 1 → 0
Char. 197: 0 → 1

**Node 73:**
Char. 83: 0 → 1
Char. 85: 1 → 0
Char. 107: 0 → 1

**Node 74:**
Char. 2: 0 → 1
Char. 6: 0 → 1
Char. 186: 0 → 1
Char. 198: 0 → 1
Char. 200: 0 → 1
Char. 220: 0 → 1
Char. 239: 1 → 0

**Node 75:**
Char. 98: 1 → 0
Char. 113: 0 → 1
Char. 181: 0 → 1
Char. 206: 0 → 2

**Node 76:**
Char. 35: 0 → 1
Char. 102: 0 → 1
Char. 103: 0 → 1
Char. 184: 0 → 1
Char. 201: 0 → 1
Char. 210: 0 → 1
Char. 223: 0 → 1
Char. 229: 0 → 1

**Node 77:**
Char. 17: 0 → 1
Char. 42: 0 → 1
Char. 251: 0 → 1
Char. 264: 0 → 1

**Node 78:**
Char. 41: 0 → 1
Char. 61: 1 → 2
Char. 112: 1 → 0
Char. 138: 0 → 1
Char. 146: 1 → 0
Char. 155: 0 → 1
Char. 192: 1 → 2
Char. 224: 0 → 1
Char. 226: 0 → 1
Char. 227: 0 → 1
Char. 233: 0 → 1
Char. 267: 01 → 2

**Node 79:**
Char. 61: 1 → 3
Char. 90: 0 → 1
Char. 91: 0 → 1
Char. 109: 1 → 0
Char. 155: 0 → 1
Char. 209: 1 → 0

### ANALYSIS 48
### (ALL TAXA, IMPLIED WEIGHTING, K = 4)

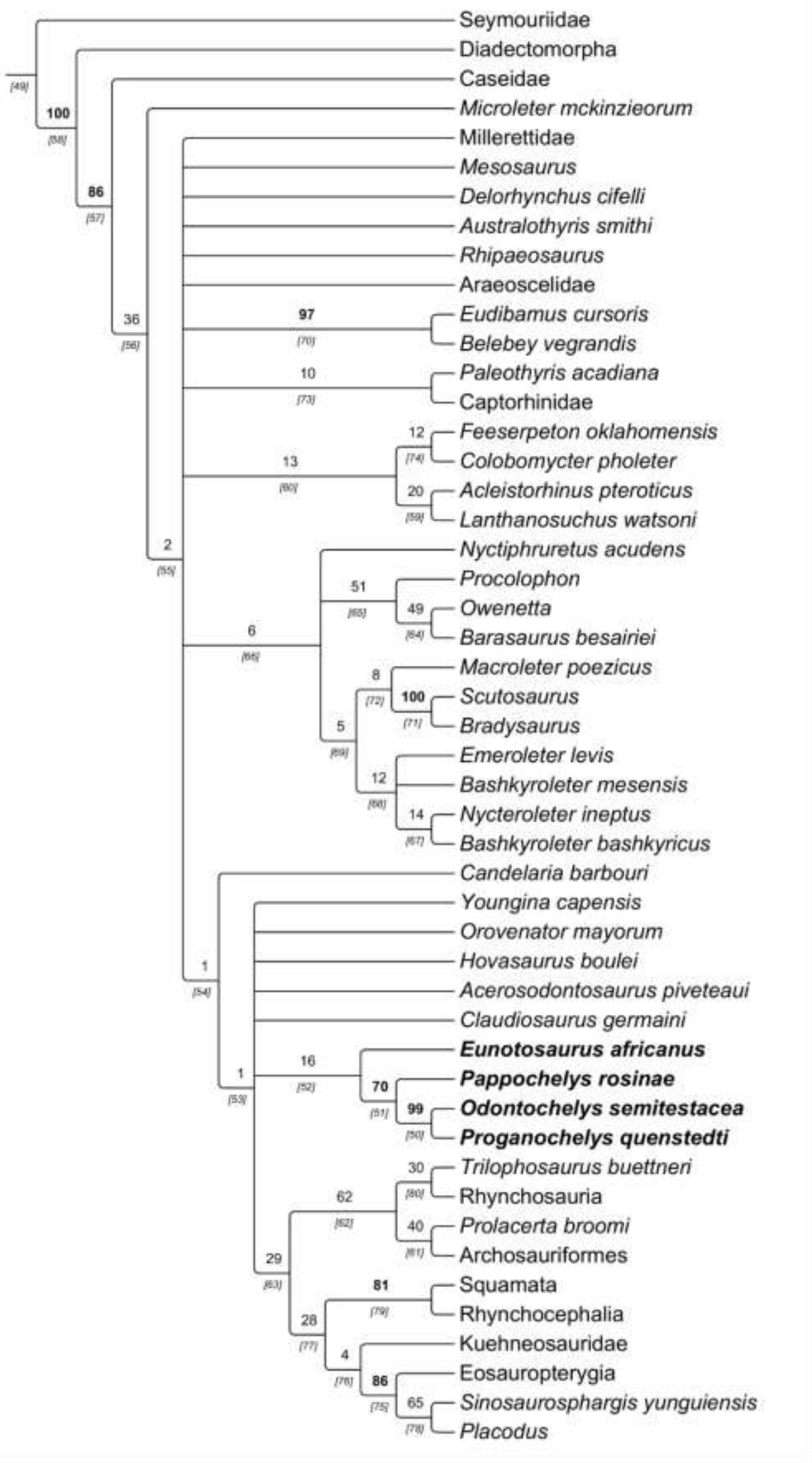

***Proganochelys quenstedti*:**
Char. 8: 0 → 1
Char. 11: 0 → 1
Char. 106: 0 → 1
Char. 108: 1 → 0
Char. 128: 0 → 1
Char. 175: 0 → 1
Char. 202: 1 → 0
Char. 207: 1 → 0
Char. 209: 1 → 0
Char. 244: 0 → 1
Char. 248: 0 → 1
Char. 250: 0 → 1
Char. 252: 1 → 0
Char. 262: 0 → 1

***Pappochelys rosinae*:**
Char. 0: 2 → 0
Char. 1: 0 → 1
Char. 5: 1 → 0
Char. 12: 0 → 1
Char. 41: 0 → 1
Char. 49: 0 → 1
Char. 75: 0 → 1
Char. 169: 1 → 0
Char. 260: 2 → 0

***Eunotosaurus africanus*:**
Char. 19: 0 → 1
Char. 59: 1 → 0
Char. 60: 1 → 0
Char. 72: 2 → 1
Char. 76: 0 → 1
Char. 97: 1 → 0
Char. 103: 0 → 1
Char. 153: 1 → 0
Char. 191: 1 → 0
Char. 192: 1 → 0
Char. 202: 1 → 0
Char. 211: 0 → 1
Char. 222: 1 → 0
Char. 237: 1 → 0
Char. 248: 0 → 1
Char. 249: 0 → 2
Char. 250: 0 → 1
Char. 263: 0 → 1
Char. 273: 0 → 1
Char. 274: 0 → 1
Char. 275: 0 → 1
Char. 276: 0 → 1
Char. 277: 0 → 1
Char. 278: 0 → 3

***Acerosodontosaurus piveteaui*:**
Char. 78: 1 → 0
Char. 81: 1 → 0
Char. 127: 1 → 0
Char. 128: 0 → 1
Char. 155: 0 → 1
Char. 206: 0 → 2
Char. 208: 0 → 1
Char. 278: 0 → 1

***Acleistorhinus pteroticus*:**
Char. 21: 0 → 1
Char. 146: 1 → 0

**Araeoscelidae:**
Char. 0: 0 → 1
Char. 27: 0 → 1
Char. 28: 0 → 1
Char. 29: 1 → 0
Char. 38: 1 → 0
Char. 40: 2 → 0
Char. 43: 0 → 1
Char. 50: 1 → 0
Char. 57: 1 → 0
Char. 59: 0 → 1
Char. 60: 0 → 1
Char. 67: 0 → 1
Char. 72: 1 → 2
Char. 84: 1 → 0
Char. 89: 0 → 1
Char. 106: 0 → 1
Char. 111: 0 → 1
Char. 116: 1 → 0
Char. 117: 01 → 2
Char. 120: 0 → 1
Char. 154: 1 → 0
Char. 166: 1 → 0
Char. 169: 1 → 0
Char. 170: 0 → 1
Char. 193: 0 → 1
Char. 197: 0 → 1
Char. 222: 0 → 1
Char. 224: 0 → 1
Char. 234: 0 → 1
Char. 237: 0 → 1
Char. 239: 0 → 1
Char. 266: 0 → 1
Char. 278: 3 → 1

**Archosauriformes:**
Char. 32: 0 → 1
Char. 94: 1 → 0
Char. 112: 1 → 0
Char. 152: 0 → 1
Char. 154: 01 → 2
Char. 166: 1 → 0
Char. 171: 0 → 1
Char. 185: 0 → 1
Char. 201: 0 → 1
Char. 204: 0 → 1
Char. 218: 0 → 3
Char. 224: 0 → 1
Char. 242: 0 → 1

***Australothyris smithi*:**
Char. 24: 0 → 1
Char. 34: 0 → 1
Char. 55: 1 → 0
Char. 57: 1 → 0
Char. 71: 1 → 0
Char. 79: 0 → 1
Char. 85: 1 → 0
Char. 98: 1 → 0
Char. 100: 0 → 1
Char. 103: 0 → 1
Char. 110: 0 → 1
Char. 123: 1 → 0
Char. 129: 0 → 1
Char. 131: 0 → 1
Char. 132: 1 → 0
Char. 144: 1 → 0
Char. 147: 0 → 1
Char. 149: 1 → 0
Char. 150: 0 → 1
Char. 192: 1 → 0

***Barasaurus besairiei*:**
Char. 33: 1 → 0

***Bashkyroleter bashkyricus*:**
Char. 275: 1 → 0

***Bashkyroleter mesensis*:**
Char. 12: 0 → 1
Char. 66: 1 → 0
Char. 169: 1 → 0

***Belebey vegrandis*:**
Char. 154: 1 → 0

***Bradysaurus* spp.:**
Char. 19: 0 → 1
Char. 25: 1 → 0
Char. 73: 0 → 1
Char. 79: 0 → 1
Char. 135: 1 → 0
Char. 249: 0 → 1

***Candelaria barbouri*:**
Char. 1: 0 → 1
Char. 8: 0 → 1
Char. 15: 0 → 1
Char. 33: 1 → 2
Char. 49: 0 → 1
Char. 55: 1 → 0
Char. 76: 0 → 1
Char. 79: 0 → 2
Char. 88: 1 → 0
Char. 95: 0 → 1
Char. 126: 0 → 1
Char. 132: 1 → 0
Char. 154: 1 → 2
Char. 169: 1 → 0
Char. 276: 0 → 1
Char. 277: 0 → 1

**Captorhinidae:**
Char. 3: 0 → 1
Char. 25: 1 → 0
Char. 26: 0 → 1
Char. 73: 0 → 1
Char. 75: 0 → 1
Char. 108: 1 → 0
Char. 180: 1 → 0
Char. 183: 1 → 0
Char. 201: 1 → 0
Char. 203: 1 → 2
Char. 216: 1 → 0

**Caseidae:**
Char. 38: 1 → 0
Char. 46: 1 → 0
Char. 50: 1 → 0
Char. 170: 0 → 1
Char. 194: 0 → 1
Char. 273: 0 → 1
Char. 274: 0 → 1
Char. 278: 3 → 2

***Claudiosaurus germaini*:**
Char. 24: 0 → 1
Char. 27: 0 → 1
Char. 34: 0 → 1
Char. 36: 0 → 1
Char. 56: 0 → 1
Char. 64: 0 → 1
Char. 70: 0 → 1
Char. 84: 1 → 0
Char. 105: 0 → 1
Char. 106: 0 → 1
Char. 117: 01 → 2
Char. 126: 0 → 1
Char. 127: 1 → 0
Char. 130: 0 → 1
Char. 131: 0 → 1
Char. 141: 1 → 0
Char. 144: 1 → 0
Char. 166: 1 → 0
Char. 182: 0 → 1
Char. 187: 1 → 0
Char. 190: 0 → 1
Char. 199: 0 → 1
Char. 201: 0 → 1
Char. 203: 1 → 2
Char. 204: 0 → 1
Char. 222: 1 → 0
Char. 234: 1 → 0
Char. 272: 2 → 1

***Colobomycter pholeter*:**
Char. 21: 0 → 1
Char. 25: 1 → 0
Char. 84: 1 → 0
Char. 154: 1 → 0
Char. 167: 0 → 1
Char. 267: 0 → 1

***Delorhynchus cifelli*:**
Char. 5: 0 → 1
Char. 18: 0 → 1
Char. 20: 0 → 1
Char. 21: 0 → 1
Char. 24: 0 → 1
Char. 26: 0 → 1
Char. 28: 0 → 1
Char. 33: 0 → 2
Char. 39: 0 → 1
Char. 52: 0 → 1
Char. 100: 0 → 1
Char. 111: 0 → 1
Char. 116: 1 → 0
Char. 119: 1 → 0
Char. 131: 0 → 1
Char. 147: 0 → 1
Char. 156: 1 → 0
Char. 167: 0 → 1
Char. 189: 0 → 1
Char. 191: 0 → 1
Char. 204: 0 → 2
Char. 267: 0 → 1

**Diadectomorpha:**
Char. 0: 0 → 1
Char. 64: 0 → 1
Char. 70: 0 → 1
Char. 122: 0 → 1
Char. 123: 1 → 0
Char. 146: 1 → 0
Char. 275: 1 → 0
Char. 278: 3 → 0

***Emeroleter levis*:**
Char. 51: 1 → 0

**Eosauropterygia:**
Char. 166: 1 → 0
Char. 174: 0 → 1
Char. 178: 0 → 1
Char. 194: 0 → 2
Char. 272: 1 → 0

***Eudibamus cursoris*:**
Char. 154: 1 → 2

***Feeserpeton oklahomensis*:**
Char. 51: 0 → 1
Char. 157: 0 → 1
Char. 158: 0 → 1

***Hovasaurus boulei*:**
Char. 41: 0 → 1
Char. 55: 1 → 0
Char. 60: 1 → 0
Char. 72: 2 → 1
Char. 77: 0 → 2
Char. 78: 1 → 0
Char. 79: 0 → 1
Char. 93: 1 → 0
Char. 113: 0 → 1
Char. 138: 0 → 1
Char. 141: 1 → 0
Char. 146: 1 → 0
Char. 204: 0 → 2
Char. 206: 0 → 2
Char. 215: 0 → 1
Char. 219: 1 → 0
Char. 224: 0 → 1
Char. 278: 0 → 3

**Kuehneosauridae:**
Char. 7: 0 → 1
Char. 24: 0 → 1
Char. 27: 0 → 1
Char. 43: 1 → 0
Char. 79: 0 → 2
Char. 107: 1 → 0
Char. 140: 1 → 0
Char. 147: 1 → 0

Char. 148: 1 → 0
Char. 245: 0 → 1
Char. 278: 0 → 3

***Lanthanosuchus watsoni*:**
Char. 25: 1 → 0
Char. 51: 0 → 1
Char. 76: 0 → 1
Char. 86: 0 → 1
Char. 98: 1 → 0
Char. 138: 0 → 1
Char. 144: 1 → 0
Char. 154: 1 → 2

***Macroleter poezicus*:**
Char. 9: 0 → 1
Char. 26: 0 → 1
Char. 66: 1 → 2
Char. 126: 0 → 1
Char. 134: 0 → 2
Char. 140: 0 → 1
Char. 146: 1 → 0
Char. 169: 1 → 0

***Mesosaurus* spp.:**
Char. 0: 0 → 1
Char. 2: 0 → 1
Char. 6: 0 → 1
Char. 8: 0 → 1
Char. 9: 0 → 1
Char. 13: 0 → 1
Char. 19: 0 → 1
Char. 26: 0 → 1
Char. 29: 1 → 0
Char. 33: 0 → 1
Char. 38: 1 → 0
Char. 41: 0 → 1
Char. 50: 1 → 0
Char. 67: 0 → 1
Char. 76: 0 → 1
Char. 84: 1 → 0
Char. 85: 1 → 0
Char. 94: 0 → 1
Char. 107: 0 → 1
Char. 109: 0 → 1
Char. 111: 0 → 1
Char. 115: 0 → 1
Char. 146: 1 → 0
Char. 149: 1 → 0
Char. 164: 0 → 1
Char. 166: 1 → 0
Char. 167: 0 → 1
Char. 176: 0 → 1
Char. 183: 1 → 0
Char. 184: 0 → 1
Char. 192: 1 → 0
Char. 199: 0 → 1
Char. 202: 0 → 1
Char. 204: 0 → 2
Char. 206: 0 → 1
Char. 207: 0 → 1
Char. 209: 0 → 1
Char. 217: 0 → 1
Char. 219: 0 → 1
Char. 220: 0 → 1
Char. 231: 0 → 1
Char. 260: 2 → 0
Char. 272: 2 → 0
Char. 278: 3 → 0

***Microleter mckinzieorum*:**
Char. 0: 0 → 1
Char. 18: 0 → 1
Char. 39: 0 → 1
Char. 57: 1 → 0
Char. 70: 0 → 1
Char. 76: 0 → 1
Char. 94: 0 → 1
Char. 106: 0 → 1
Char. 110: 0 → 1
Char. 278: 3 → 1

**Millerettidae:**
Char. 24: 0 → 1
Char. 25: 1 → 0
Char. 44: 1 → 0
Char. 56: 0 → 1
Char. 57: 1 → 0
Char. 66: 0 → 2
Char. 78: 1 → 0
Char. 80: 1 → 0
Char. 84: 1 → 2
Char. 88: 1 → 0
Char. 96: 1 → 0
Char. 121: 0 → 1
Char. 124: 0 → 1
Char. 127: 0 → 1
Char. 135: 0 → 1
Char. 145: 0 → 1
Char. 166: 1 → 0
Char. 180: 1 → 0
Char. 192: 1 → 0
Char. 202: 0 → 1
Char. 211: 0 → 1
Char. 230: 0 → 1
Char. 234: 0 → 1
Char. 248: 0 → 1
Char. 252: 0 → 1
Char. 253: 0 → 1

***Nycteroleter ineptus*:**
Char. 66: 1 → 0
Char. 278: 0 → 3

***Nyctiphruretus acudens*:**
Char. 0: 0 → 1
Char. 21: 0 → 1
Char. 33: 1 → 2
Char. 41: 0 → 1
Char. 66: 1 → 2
Char. 81: 1 → 0
Char. 84: 1 → 2
Char. 94: 0 → 1
Char. 166: 1 → 0
Char. 224: 0 → 1
Char. 235: 1 → 0
Char. 266: 0 → 1
Char. 272: 2 → 1
Char. 276: 0 → 1

***Orovenator mayorum*:**
Char. 8: 0 → 1
Char. 24: 0 → 1
Char. 33: 1 → 0
Char. 36: 0 → 1
Char. 50: 0 → 1
Char. 92: 1 → 0
Char. 94: 1 → 0
Char. 135: 1 → 0
Char. 159: 1 → 0
Char. 160: 0 → 1
Char. 165: 0 → 1
Char. 278: 0 → 1

***Owenetta* spp.:**
Char. 142: 1 → 0
Char. 169: 1 → 0

***Paleothyris acadiana*:**
Char. 38: 1 → 0
Char. 50: 1 → 0
Char. 66: 1 → 2
Char. 102: 0 → 1
Char. 146: 1 → 0
Char. 237: 0 → 1
Char. 239: 0 → 1

***Placodus* spp.:**
Char. 0: 1 → 2
Char. 9: 0 → 1
Char. 12: 0 → 1
Char. 13: 0 → 1
Char. 19: 0 → 1
Char. 31: 0 → 1
Char. 46: 1 → 0
Char. 57: 0 → 1
Char. 78: 1 → 0
Char. 93: 1 → 0
Char. 102: 1 → 2
Char. 109: 1 → 0
Char. 140: 1 → 0
Char. 155: 0 → 1
Char. 163: 1 → 0
Char. 164: 0 → 1

***Procolophon* spp.:**
Char. 41: 0 → 1
Char. 69: 0 → 1
Char. 79: 0 → 1
Char. 86: 0 → 1
Char. 88: 0 → 1
Char. 101: 0 → 1
Char. 117: 1 → 0
Char. 141: 0 → 1
Char. 149: 1 → 0
Char. 203: 1 → 2
Char. 204: 0 → 1
Char. 215: 0 → 1
Char. 230: 0 → 1
Char. 235: 1 → 2
Char. 237: 0 → 1
Char. 238: 0 → 1
Char. 272: 2 → 1
Char. 278: 3 → 0

***Prolacerta broomi*:**
Char. 58: 1 → 0
Char. 66: 1 → 0
Char. 67: 1 → 0
Char. 80: 1 → 0
Char. 139: 1 → 0
Char. 147: 1 → 0
Char. 192: 1 → 0
Char. 203: 1 → 2
Char. 206: 0 → 12

***Rhipaeosaurus* spp.:**
Char. 172: 0 → 1
Char. 183: 1 → 2
Char. 194: 0 → 1
Char. 201: 1 → 0
Char. 211: 0 → 1
Char. 215: 0 → 1
Char. 226: 0 → 1
Char. 235: 1 → 0
Char. 252: 0 → 1
Char. 277: 0 → 1

**Rhynchocephalia:**
Char. 0: 1 → 2
Char. 24: 0 → 1
Char. 77: 0 → 1
Char. 88: 1 → 0
Char. 94: 1 → 0
Char. 139: 1 → 0
Char. 167: 1 → 0
Char. 205: 1 → 0

**Rhynchosauria:**
Char. 0: 1 → 0
Char. 7: 0 → 1
Char. 9: 0 → 1
Char. 26: 0 → 1
Char. 44: 1 → 0
Char. 68: 0 → 1
Char. 99: 1 → 0
Char. 150: 1 → 0
Char. 160: 0 → 1
Char. 161: 0 → 1
Char. 171: 0 → 2
Char. 223: 0 → 1
Char. 224: 0 → 1
Char. 241: 0 → 1

***Scutosaurus* spp.:**
Char. 175: 0 → 1
Char. 190: 0 → 1
Char. 218: 0 → 1
Char. 243: 0 → 2
Char. 244: 0 → 1
Char. 251: 0 → 1

***Sinosaurosphargis yunguiensis*:**
Char. 8: 0 → 1
Char. 30: 0 → 1
Char. 53: 0 → 1
Char. 89: 1 → 0
Char. 127: 1 → 0
Char. 150: 1 → 0
Char. 154: 0 → 2
Char. 167: 1 → 0
Char. 253: 0 → 1
Char. 255: 0 → 1

**Squamata:**
Char. 45: 0 → 1
Char. 79: 0 → 2
Char. 80: 1 → 0
Char. 92: 1 → 0
Char. 109: 1 → 0
Char. 160: 0 → 1
Char. 200: 0 → 1
Char. 245: 0 → 1

***Trilophosaurus buettneri*:**
Char. 5: 1 → 0
Char. 11: 0 → 1
Char. 55: 1 → 0
Char. 93: 1 → 0
Char. 104: 0 → 1
Char. 106: 0 → 1
Char. 113: 0 → 1
Char. 122: 0 → 1
Char. 136: 1 → 0
Char. 144: 1 → 0
Char. 157: 0 → 1
Char. 159: 1 → 0
Char. 177: 0 → 12
Char. 194: 0 → 1
Char. 203: 1 → 2
Char. 207: 1 → 0
Char. 208: 1 → 0
Char. 272: 1 → 0

***Youngina capensis*:**
Char. 5: 1 → 0
Char. 25: 0 → 1
Char. 27: 0 → 1
Char. 38: 1 → 0
Char. 44: 1 → 0
Char. 56: 0 → 1
Char. 75: 0 → 1
Char. 84: 1 → 0
Char. 92: 1 → 0
Char. 94: 1 → 0
Char. 134: 0 → 1
Char. 163: 1 → 0
Char. 170: 0 → 1
Char. 211: 0 → 1
Char. 215: 0 → 1
Char. 224: 0 → 1
Char. 239: 0 → 1

**Node 50:**
Char. 46: 1 → 0
Char. 88: 1 → 0
Char. 89: 1 → 0
Char. 93: 1 → 0
Char. 176: 0 → 1
Char. 195: 0 → 2

Char. 198: 0 → 1
Char. 246: 1 → 2
Char. 259: 0 → 1
Char. 265: 1 → 0
Char. 270: 0 → 1

**Node 51:**
Char. 65: 0 → 1
Char. 131: 0 → 1
Char. 184: 0 → 1
Char. 205: 0 → 1
Char. 210: 0 → 1
Char. 219: 1 → 0
Char. 241: 0 → 1
Char. 246: 0 → 1
Char. 254: 0 → 1
Char. 255: 0 → 1
Char. 256: 0 → 1
Char. 268: 0 → 1
Char. 269: 0 → 1

**Node 52:**
Char. 0: 1 → 2
Char. 15: 0 → 1
Char. 33: 1 → 2
Char. 44: 1 → 0
Char. 62: 1 → 0
Char. 64: 0 → 1
Char. 84: 1 → 2
Char. 130: 0 → 1
Char. 134: 0 → 2
Char. 147: 0 → 1
Char. 152: 0 → 1
Char. 155: 0 → 1
Char. 158: 0 → 1
Char. 161: 0 → 1
Char. 174: 0 → 1
Char. 181: 0 → 1
Char. 203: 1 → 2
Char. 204: 0 → 2
Char. 247: 0 → 1
Char. 251: 0 → 1
Char. 252: 0 → 1
Char. 253: 0 → 2

**Node 53:**
Char. 0: 0 → 1
Char. 5: 0 → 1
Char. 59: 0 → 1
Char. 60: 0 → 1
Char. 62: 0 → 1
Char. 72: 1 → 2
Char. 92: 0 → 1
Char. 265: 0 → 1
Char. 275: 1 → 0
Char. 278: 3 → 0

**Node 54:**
Char. 20: 0 → 1
Char. 25: 1 → 0
Char. 29: 1 → 0
Char. 33: 0 → 1
Char. 50: 1 → 0
Char. 57: 1 → 0
Char. 67: 0 → 1
Char. 89: 0 → 1
Char. 94: 0 → 1
Char. 127: 0 → 1

**Node 55:**
Char. 132: 0 → 1
Char. 166: 0 → 1
Char. 276: 1 → 0

**Node 56:**
Char. 29: 0 → 1
Char. 84: 0 → 1
Char. 88: 0 → 1
Char. 169: 0 → 1

**Node 57:**
Char. 72: 0 → 1
Char. 81: 0 → 1
Char. 93: 0 → 1
Char. 97: 0 → 1
Char. 104: 1 → 0
Char. 144: 0 → 1
Char. 173: 0 → 1
Char. 183: 0 → 1

**Node 59:**
Char. 95: 0 → 1
Char. 113: 0 → 1
Char. 114: 0 → 1

**Node 60:**
Char. 5: 0 → 1
Char. 20: 0 → 1
Char. 47: 0 → 1
Char. 79: 0 → 1
Char. 110: 0 → 1
Char. 131: 0 → 1
Char. 137: 0 → 1
Char. 140: 0 → 2
Char. 147: 0 → 1

**Node 61:**
Char. 19: 0 → 1
Char. 92: 1 → 0

**Node 62:**
Char. 4: 0 → 1
Char. 15: 0 → 1
Char. 29: 0 → 1
Char. 213: 0 → 1
Char. 226: 0 → 2
Char. 228: 0 → 1
Char. 275: 0 → 1

**Node 63:**
Char. 58: 0 → 1
Char. 61: 0 → 1
Char. 66: 0 → 1
Char. 69: 0 → 1
Char. 107: 0 → 1
Char. 126: 0 → 1
Char. 131: 0 → 1
Char. 134: 0 → 1
Char. 140: 0 → 1
Char. 147: 0 → 1
Char. 150: 0 → 1
Char. 167: 0 → 1
Char. 176: 0 → 1
Char. 190: 0 → 1
Char. 205: 0 → 1
Char. 208: 0 → 1
Char. 230: 0 → 1
Char. 239: 0 → 1
Char. 260: 2 → 1

**Node 64:**
Char. 73: 0 → 1
Char. 131: 1 → 0
Char. 205: 0 → 1
Char. 273: 0 → 1
Char. 276: 0 → 1

**Node 65:**
Char. 18: 0 → 1
Char. 25: 1 → 0
Char. 37: 0 → 1
Char. 103: 0 → 1
Char. 106: 0 → 1
Char. 107: 0 → 1
Char. 110: 0 → 1
Char. 126: 0 → 1
Char. 150: 0 → 1

**Node 66:**
Char. 5: 0 → 1
Char. 20: 0 → 1
Char. 33: 0 → 1
Char. 38: 1 → 2
Char. 39: 0 → 1
Char. 44: 1 → 0
Char. 49: 0 → 1
Char. 51: 0 → 1
Char. 66: 0 → 1
Char. 76: 0 → 1
Char. 80: 1 → 0
Char. 88: 1 → 0
Char. 95: 0 → 1
Char. 131: 0 → 1
Char. 135: 0 → 1
Char. 137: 0 → 1
Char. 147: 0 → 1
Char. 157: 0 → 1
Char. 158: 0 → 1
Char. 183: 1 → 2
Char. 186: 0 → 1
Char. 187: 0 → 1
Char. 194: 0 → 1
Char. 197: 0 → 1
Char. 201: 1 → 0
Char. 211: 0 → 1
Char. 252: 0 → 1

**Node 67:**
Char. 87: 0 → 1
Char. 110: 0 → 1

**Node 68:**
Char. 79: 0 → 1
Char. 133: 0 → 1

**Node 69:**
Char. 100: 0 → 1
Char. 113: 0 → 1

**Node 70:**
Char. 5: 0 → 1
Char. 38: 1 → 2
Char. 39: 0 → 1
Char. 50: 1 → 0
Char. 58: 0 → 1
Char. 59: 0 → 1
Char. 60: 0 → 1
Char. 72: 1 → 2
Char. 85: 1 → 0
Char. 88: 1 → 0
Char. 95: 0 → 1
Char. 104: 0 → 1
Char. 105: 0 → 1
Char. 106: 0 → 2
Char. 107: 0 → 1
Char. 109: 0 → 1
Char. 110: 0 → 1
Char. 146: 1 → 0
Char. 155: 0 → 2
Char. 183: 1 → 2

**Node 71:**
Char. 33: 1 → 0
Char. 38: 2 → 1
Char. 39: 1 → 0
Char. 42: 0 → 1
Char. 43: 0 → 1
Char. 46: 1 → 0
Char. 49: 1 → 0
Char. 71: 1 → 0
Char. 83: 0 → 2
Char. 101: 0 → 1
Char. 103: 0 → 1
Char. 106: 0 → 1
Char. 161: 0 → 1
Char. 163: 1 → 0
Char. 172: 0 → 2
Char. 174: 0 → 1
Char. 188: 0 → 1
Char. 203: 1 → 2
Char. 212: 0 → 1
Char. 216: 1 → 0
Char. 235: 1 → 2
Char. 236: 0 → 1
Char. 238: 0 → 2
Char. 241: 0 → 1
Char. 242: 0 → 1
Char. 273: 0 → 1
Char. 274: 0 → 1
Char. 275: 1 → 0

**Node 72:**
Char. 52: 0 → 1
Char. 75: 0 → 1
Char. 84: 1 → 0
Char. 87: 0 → 1
Char. 110: 0 → 1
Char. 121: 0 → 1
Char. 180: 1 → 0
Char. 230: 0 → 1

**Node 73:**
Char. 29: 1 → 0
Char. 44: 1 → 0
Char. 59: 0 → 1
Char. 60: 0 → 1
Char. 66: 0 → 1
Char. 67: 0 → 1
Char. 72: 1 → 2
Char. 78: 1 → 0
Char. 84: 1 → 0
Char. 88: 1 → 0
Char. 93: 1 → 0
Char. 111: 0 → 1
Char. 123: 1 → 0
Char. 129: 0 → 1
Char. 154: 1 → 0
Char. 169: 1 → 0
Char. 170: 0 → 1
Char. 192: 1 → 0
Char. 197: 0 → 1

**Node 74:**
Char. 85: 1 → 0
Char. 107: 0 → 1

**Node 75:**
Char. 2: 0 → 1
Char. 6: 0 → 1
Char. 186: 0 → 1
Char. 198: 0 → 1
Char. 200: 0 → 1
Char. 220: 0 → 1
Char. 239: 1 → 0

**Node 76:**
Char. 98: 1 → 0
Char. 113: 0 → 1
Char. 181: 0 → 1
Char. 206: 0 → 2

**Node 77:**
Char. 35: 0 → 1
Char. 102: 0 → 1
Char. 103: 0 → 1
Char. 184: 0 → 1
Char. 201: 0 → 1
Char. 210: 0 → 1
Char. 223: 0 → 1
Char. 229: 0 → 1

**Node 78:**
Char. 17: 0 → 1
Char. 42: 0 → 1
Char. 251: 0 → 1
Char. 264: 0 → 1

**Node 79:**
Char. 41: 0 → 1
Char. 61: 1 → 2
Char. 112: 1 → 0
Char. 138: 0 → 1
Char. 146: 1 → 0

Char. 155: 0 → 1
Char. 192: 1 → 2
Char. 224: 0 → 1
Char. 226: 0 → 1

Char. 227: 0 → 1
Char. 233: 0 → 1
Char. 267: 01 → 2

**Node 80:**
Char. 61: 1 → 3
Char. 90: 0 → 1
Char. 91: 0 → 1

Char. 109: 1 → 0
Char. 155: 0 → 1
Char. 209: 1 → 0

**ANALYSIS 49**
**(ALL TAXA, IMPLIED WEIGHTING, K = 4.125)**

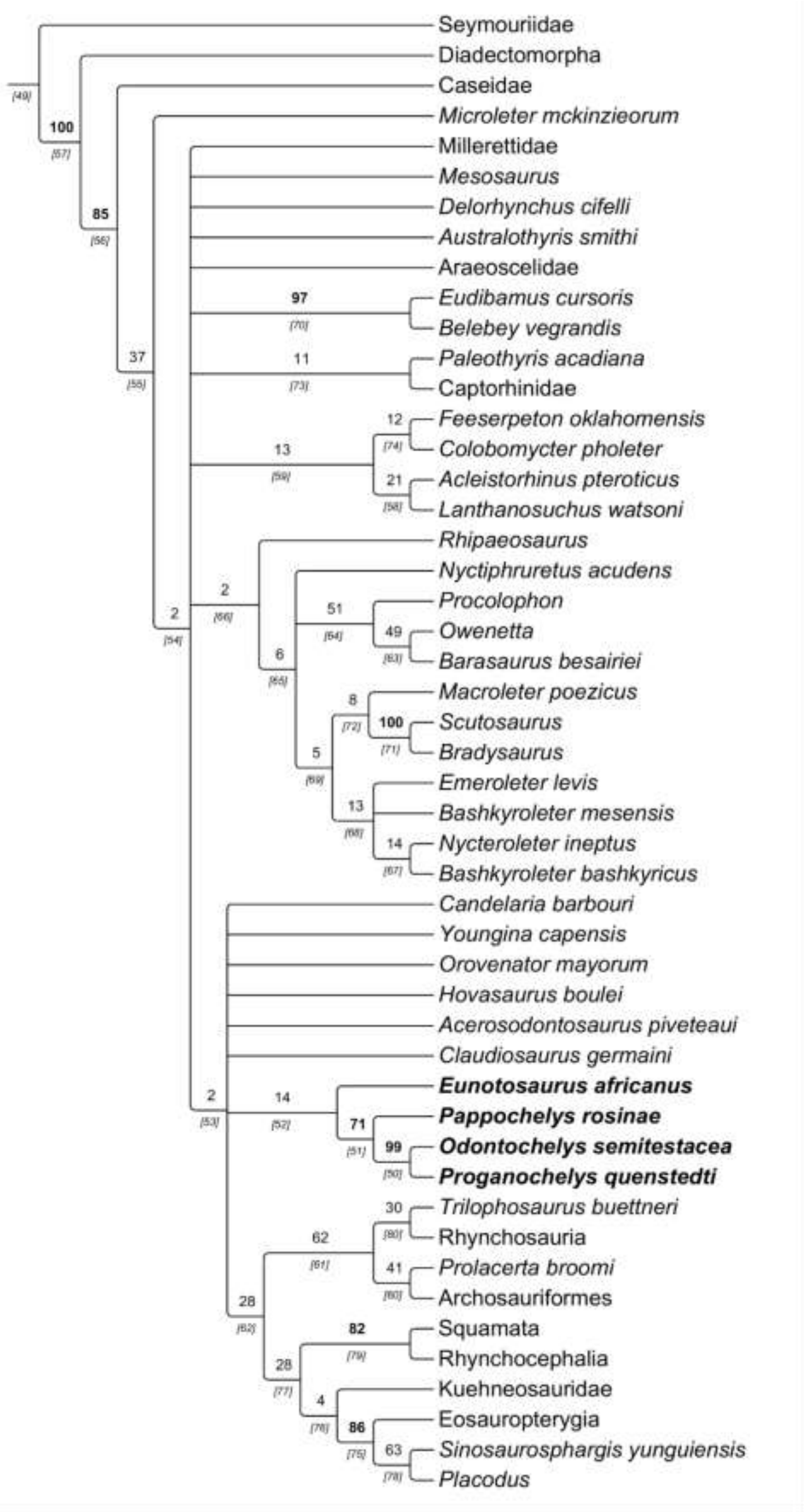

***Proganochelys quenstedti*:**
Char. 8: 0 → 1
Char. 11: 0 → 1
Char. 106: 0 → 1
Char. 108: 1 → 0
Char. 128: 0 → 1
Char. 175: 0 → 1
Char. 202: 1 → 0
Char. 207: 1 → 0
Char. 209: 1 → 0
Char. 244: 0 → 1
Char. 248: 0 → 1
Char. 250: 0 → 1
Char. 252: 1 → 0
Char. 262: 0 → 1

***Pappochelys rosinae*:**
Char. 0: 2 → 0
Char. 1: 0 → 1
Char. 5: 1 → 0
Char. 12: 0 → 1
Char. 41: 0 → 1
Char. 49: 0 → 1
Char. 75: 0 → 1
Char. 169: 1 → 0
Char. 260: 2 → 0

***Odontochelys semitestacea*:**
Char. 43: 0 → 1

***Eunotosaurus africanus*:**
Char. 19: 0 → 1
Char. 43: 0 → 1
Char. 59: 1 → 0
Char. 72: 2 → 1
Char. 76: 0 → 1
Char. 97: 1 → 0
Char. 103: 0 → 1
Char. 153: 1 → 0
Char. 191: 1 → 0
Char. 192: 1 → 0
Char. 202: 1 → 0
Char. 211: 0 → 1
Char. 222: 1 → 0
Char. 237: 1 → 0
Char. 248: 0 → 1
Char. 249: 0 → 2
Char. 250: 0 → 1
Char. 263: 0 → 1
Char. 273: 0 → 1
Char. 274: 0 → 1
Char. 275: 0 → 1
Char. 276: 0 → 1
Char. 277: 0 → 1

***Acerosodontosaurus piveteaui*:**
Char. 78: 1 → 0
Char. 81: 1 → 0
Char. 127: 1 → 0
Char. 128: 0 → 1
Char. 155: 0 → 1
Char. 206: 0 → 2
Char. 208: 0 → 1
Char. 278: 03 → 1

***Acleistorhinus pteroticus*:**
Char. 21: 0 → 1
Char. 146: 1 → 0

**Araeoscelidae:**
Char. 0: 0 → 1
Char. 27: 0 → 1
Char. 28: 0 → 1
Char. 29: 1 → 0
Char. 38: 1 → 0
Char. 40: 2 → 0
Char. 43: 0 → 1
Char. 50: 1 → 0
Char. 57: 1 → 0
Char. 59: 0 → 1
Char. 60: 0 → 1
Char. 67: 0 → 1
Char. 72: 1 → 2
Char. 84: 1 → 0
Char. 89: 0 → 1
Char. 106: 0 → 1
Char. 111: 0 → 1
Char. 116: 1 → 0
Char. 117: 01 → 2
Char. 120: 0 → 1
Char. 154: 1 → 0
Char. 166: 1 → 0
Char. 169: 1 → 0
Char. 170: 0 → 1
Char. 193: 0 → 1
Char. 197: 0 → 1
Char. 222: 0 → 1
Char. 224: 0 → 1
Char. 237: 0 → 1
Char. 239: 0 → 1
Char. 266: 0 → 1
Char. 278: 3 → 1

**Archosauriformes:**
Char. 32: 0 → 1
Char. 94: 1 → 0
Char. 112: 1 → 0
Char. 152: 0 → 1
Char. 154: 01 → 2
Char. 166: 1 → 0
Char. 171: 0 → 1
Char. 185: 0 → 1
Char. 201: 0 → 1
Char. 204: 0 → 1
Char. 218: 0 → 3
Char. 224: 0 → 1
Char. 242: 0 → 1

***Australothyris smithi*:**
Char. 24: 0 → 1
Char. 34: 0 → 1
Char. 55: 1 → 0
Char. 57: 1 → 0
Char. 71: 1 → 0
Char. 79: 0 → 1
Char. 83: 0 → 1
Char. 85: 1 → 0
Char. 98: 1 → 0
Char. 100: 0 → 1
Char. 103: 0 → 1
Char. 110: 0 → 1
Char. 123: 1 → 0
Char. 129: 0 → 1
Char. 131: 0 → 1
Char. 132: 1 → 0
Char. 144: 1 → 0
Char. 147: 0 → 1
Char. 149: 1 → 0
Char. 150: 0 → 1
Char. 192: 1 → 0

***Barasaurus besairiei*:**
Char. 33: 1 → 0

***Bashkyroleter bashkyricus*:**
Char. 275: 1 → 0

***Bashkyroleter mesensis*:**
Char. 12: 0 → 1
Char. 66: 1 → 0
Char. 169: 1 → 0

***Belebey vegrandis*:**
Char. 154: 1 → 0

***Bradysaurus* spp.:**
Char. 19: 0 → 1
Char. 25: 1 → 0
Char. 73: 0 → 1
Char. 79: 0 → 1
Char. 135: 1 → 0
Char. 249: 0 → 1

***Candelaria barbouri*:**
Char. 0: 1 → 0
Char. 1: 0 → 1
Char. 5: 1 → 0
Char. 8: 0 → 1
Char. 15: 0 → 1
Char. 33: 1 → 2
Char. 49: 0 → 1
Char. 55: 1 → 0
Char. 59: 1 → 0
Char. 62: 1 → 0
Char. 72: 2 → 1
Char. 76: 0 → 1
Char. 79: 0 → 2
Char. 83: 0 → 1
Char. 88: 1 → 0
Char. 92: 1 → 0
Char. 95: 0 → 1
Char. 126: 0 → 1
Char. 132: 1 → 0
Char. 154: 1 → 2
Char. 159: 1 → 0
Char. 169: 1 → 0
Char. 265: 1 → 0
Char. 275: 0 → 1
Char. 276: 0 → 1
Char. 277: 0 → 1

**Captorhinidae:**
Char. 3: 0 → 1
Char. 25: 1 → 0
Char. 26: 0 → 1
Char. 73: 0 → 1
Char. 75: 0 → 1
Char. 83: 0 → 1
Char. 108: 1 → 0
Char. 180: 1 → 0
Char. 183: 1 → 0
Char. 201: 1 → 0
Char. 203: 1 → 2
Char. 216: 1 → 0
Char. 240: 1 → 0

**Caseidae:**
Char. 38: 1 → 0
Char. 46: 1 → 0
Char. 50: 1 → 0
Char. 170: 0 → 1
Char. 194: 0 → 1
Char. 273: 0 → 1
Char. 274: 0 → 1
Char. 278: 3 → 2

***Claudiosaurus germaini*:**
Char. 24: 0 → 1
Char. 27: 0 → 1
Char. 34: 0 → 1
Char. 36: 0 → 1
Char. 56: 0 → 1
Char. 64: 0 → 1
Char. 70: 0 → 1
Char. 84: 1 → 0
Char. 105: 0 → 1
Char. 106: 0 → 1
Char. 117: 01 → 2
Char. 126: 0 → 1
Char. 127: 1 → 0
Char. 130: 0 → 1
Char. 131: 0 → 1
Char. 141: 1 → 0
Char. 144: 1 → 0
Char. 148: 0 → 1
Char. 154: 1 → 0
Char. 166: 1 → 0
Char. 182: 0 → 1
Char. 187: 1 → 0
Char. 190: 0 → 1
Char. 199: 0 → 1
Char. 201: 0 → 1
Char. 203: 1 → 2
Char. 204: 0 → 1
Char. 222: 1 → 0
Char. 234: 1 → 0
Char. 267: 0 → 1
Char. 272: 2 → 1

***Colobomycter pholeter*:**
Char. 21: 0 → 1
Char. 25: 1 → 0
Char. 84: 1 → 0
Char. 154: 1 → 0
Char. 167: 0 → 1
Char. 267: 0 → 1

***Delorhynchus cifelli*:**
Char. 18: 0 → 1
Char. 20: 0 → 1
Char. 21: 0 → 1
Char. 24: 0 → 1
Char. 26: 0 → 1
Char. 28: 0 → 1
Char. 33: 0 → 2
Char. 39: 0 → 1
Char. 52: 0 → 1
Char. 100: 0 → 1
Char. 111: 0 → 1
Char. 116: 1 → 0
Char. 119: 1 → 0
Char. 131: 0 → 1
Char. 147: 0 → 1
Char. 156: 1 → 0
Char. 167: 0 → 1
Char. 189: 0 → 1
Char. 191: 0 → 1
Char. 204: 0 → 2
Char. 267: 0 → 1

**Diadectomorpha:**
Char. 0: 0 → 1
Char. 64: 0 → 1
Char. 70: 0 → 1
Char. 122: 0 → 1
Char. 123: 1 → 0
Char. 146: 1 → 0
Char. 275: 1 → 0
Char. 278: 3 → 0

***Emeroleter levis*:**
Char. 51: 1 → 0

**Eosauropterygia:**
Char. 166: 1 → 0
Char. 174: 0 → 1
Char. 178: 0 → 1
Char. 194: 0 → 2
Char. 272: 1 → 0

***Eudibamus cursoris*:**
Char. 154: 1 → 2

***Feeserpeton oklahomensis*:**
Char. 51: 0 → 1
Char. 157: 0 → 1
Char. 158: 0 → 1

***Hovasaurus boulei*:**
Char. 41: 0 → 1
Char. 43: 0 → 1
Char. 55: 1 → 0
Char. 72: 2 → 1
Char. 77: 0 → 2
Char. 78: 1 → 0
Char. 79: 0 → 1
Char. 93: 1 → 0
Char. 113: 0 → 1
Char. 138: 0 → 1

Char. 141: 1 → 0
Char. 146: 1 → 0
Char. 204: 0 → 2
Char. 206: 0 → 2
Char. 215: 0 → 1
Char. 219: 1 → 0
Char. 224: 0 → 1

**Kuehneosauridae:**
Char. 7: 0 → 1
Char. 24: 0 → 1
Char. 27: 0 → 1
Char. 43: 1 → 0
Char. 79: 0 → 2
Char. 107: 1 → 0
Char. 140: 1 → 0
Char. 147: 1 → 0
Char. 148: 1 → 0
Char. 245: 0 → 1
Char. 278: 0 → 3

***Lanthanosuchus watsoni*:**
Char. 25: 1 → 0
Char. 51: 0 → 1
Char. 76: 0 → 1
Char. 86: 0 → 1
Char. 98: 1 → 0
Char. 138: 0 → 1
Char. 144: 1 → 0
Char. 154: 1 → 2

***Macroleter poezicus*:**
Char. 9: 0 → 1
Char. 26: 0 → 1
Char. 66: 1 → 2
Char. 126: 0 → 1
Char. 134: 0 → 2
Char. 140: 0 → 1
Char. 146: 1 → 0
Char. 169: 1 → 0

***Mesosaurus* spp.:**
Char. 0: 0 → 1
Char. 2: 0 → 1
Char. 6: 0 → 1
Char. 8: 0 → 1
Char. 9: 0 → 1
Char. 13: 0 → 1
Char. 19: 0 → 1
Char. 26: 0 → 1
Char. 29: 1 → 0
Char. 33: 0 → 1
Char. 38: 1 → 0
Char. 41: 0 → 1
Char. 50: 1 → 0
Char. 67: 0 → 1
Char. 76: 0 → 1
Char. 83: 0 → 1
Char. 84: 1 → 0
Char. 85: 1 → 0
Char. 94: 0 → 1
Char. 107: 0 → 1
Char. 109: 0 → 1
Char. 111: 0 → 1
Char. 115: 0 → 1
Char. 146: 1 → 0
Char. 148: 0 → 1
Char. 149: 1 → 0
Char. 164: 0 → 1
Char. 166: 1 → 0
Char. 167: 0 → 1
Char. 176: 0 → 1
Char. 183: 1 → 0
Char. 184: 0 → 1
Char. 192: 1 → 0
Char. 199: 0 → 1
Char. 202: 0 → 1
Char. 204: 0 → 2
Char. 206: 0 → 1
Char. 207: 0 → 1
Char. 209: 0 → 1
Char. 217: 0 → 1
Char. 219: 0 → 1
Char. 220: 0 → 1
Char. 231: 0 → 1
Char. 240: 1 → 0
Char. 260: 2 → 0
Char. 272: 2 → 0
Char. 278: 3 → 0

***Microleter mckinzieorum*:**
Char. 0: 0 → 1
Char. 18: 0 → 1
Char. 39: 0 → 1
Char. 57: 1 → 0
Char. 70: 0 → 1
Char. 76: 0 → 1
Char. 83: 0 → 1
Char. 94: 0 → 1
Char. 106: 0 → 1
Char. 110: 0 → 1
Char. 278: 3 → 1

**Millerettidae:**
Char. 24: 0 → 1
Char. 25: 1 → 0
Char. 44: 1 → 0
Char. 56: 0 → 1
Char. 57: 1 → 0
Char. 66: 0 → 2
Char. 78: 1 → 0
Char. 80: 1 → 0
Char. 84: 1 → 2
Char. 88: 1 → 0
Char. 96: 1 → 0
Char. 121: 0 → 1
Char. 124: 0 → 1
Char. 127: 0 → 1
Char. 135: 0 → 1
Char. 145: 0 → 1
Char. 166: 1 → 0
Char. 180: 1 → 0
Char. 192: 1 → 0
Char. 202: 0 → 1
Char. 211: 0 → 1
Char. 230: 0 → 1
Char. 248: 0 → 1
Char. 252: 0 → 1
Char. 253: 0 → 1

***Nycteroleter ineptus*:**
Char. 66: 1 → 0
Char. 278: 0 → 3

***Nyctiphruretus acudens*:**
Char. 0: 0 → 1
Char. 21: 0 → 1
Char. 33: 1 → 2
Char. 41: 0 → 1
Char. 66: 1 → 2
Char. 81: 1 → 0
Char. 83: 0 → 1
Char. 84: 1 → 2
Char. 94: 0 → 1
Char. 166: 1 → 0
Char. 224: 0 → 1
Char. 226: 1 → 0
Char. 266: 0 → 1
Char. 272: 2 → 1
Char. 276: 0 → 1

***Orovenator mayorum*:**
Char. 8: 0 → 1
Char. 24: 0 → 1
Char. 33: 1 → 0
Char. 36: 0 → 1
Char. 50: 0 → 1
Char. 92: 1 → 0
Char. 94: 1 → 0
Char. 135: 1 → 0
Char. 159: 1 → 0
Char. 160: 0 → 1
Char. 165: 0 → 1
Char. 278: 03 → 1

***Owenetta* spp.:**
Char. 142: 1 → 0
Char. 169: 1 → 0

***Paleothyris acadiana*:**
Char. 38: 1 → 0
Char. 50: 1 → 0
Char. 66: 1 → 2
Char. 102: 0 → 1
Char. 146: 1 → 0
Char. 237: 0 → 1
Char. 239: 0 → 1

***Placodus* spp.:**
Char. 0: 1 → 2
Char. 9: 0 → 1
Char. 12: 0 → 1
Char. 13: 0 → 1
Char. 19: 0 → 1
Char. 31: 0 → 1
Char. 46: 1 → 0
Char. 57: 0 → 1
Char. 78: 1 → 0
Char. 93: 1 → 0
Char. 102: 1 → 2
Char. 109: 1 → 0
Char. 140: 1 → 0
Char. 155: 0 → 1
Char. 163: 1 → 0
Char. 164: 0 → 1

***Procolophon* spp.:**
Char. 41: 0 → 1
Char. 69: 0 → 1
Char. 79: 0 → 1
Char. 83: 0 → 1
Char. 86: 0 → 1
Char. 88: 0 → 1
Char. 101: 0 → 1
Char. 117: 1 → 0
Char. 141: 0 → 1
Char. 149: 1 → 0
Char. 203: 1 → 2
Char. 204: 0 → 1
Char. 230: 0 → 1
Char. 237: 0 → 1
Char. 238: 0 → 1
Char. 272: 2 → 1
Char. 278: 3 → 0

***Prolacerta broomi*:**
Char. 58: 1 → 0
Char. 66: 1 → 0
Char. 67: 1 → 0
Char. 80: 1 → 0
Char. 139: 1 → 0
Char. 147: 1 → 0
Char. 192: 1 → 0
Char. 203: 1 → 2
Char. 206: 0 → 12

***Rhipaeosaurus* spp.:**
Char. 172: 0 → 1
Char. 277: 0 → 1

**Rhynchocephalia:**
Char. 0: 1 → 2
Char. 24: 0 → 1
Char. 77: 0 → 1
Char. 88: 1 → 0
Char. 94: 1 → 0
Char. 139: 1 → 0
Char. 167: 1 → 0
Char. 205: 1 → 0

**Rhynchosauria:**
Char. 0: 1 → 0
Char. 7: 0 → 1
Char. 9: 0 → 1
Char. 26: 0 → 1
Char. 44: 1 → 0
Char. 68: 0 → 1
Char. 99: 1 → 0
Char. 150: 1 → 0
Char. 160: 0 → 1
Char. 161: 0 → 1
Char. 171: 0 → 2
Char. 223: 0 → 1
Char. 224: 0 → 1
Char. 241: 0 → 1

***Scutosaurus* spp.:**
Char. 175: 0 → 1
Char. 190: 0 → 1
Char. 218: 0 → 1
Char. 243: 0 → 2
Char. 244: 0 → 1
Char. 251: 0 → 1

***Sinosaurosphargis yunguiensis*:**
Char. 8: 0 → 1
Char. 30: 0 → 1
Char. 53: 0 → 1
Char. 89: 1 → 0
Char. 127: 1 → 0
Char. 150: 1 → 0
Char. 154: 0 → 2
Char. 167: 1 → 0
Char. 253: 0 → 1
Char. 255: 0 → 1

**Squamata:**
Char. 45: 0 → 1
Char. 79: 0 → 2
Char. 80: 1 → 0
Char. 92: 1 → 0
Char. 109: 1 → 0
Char. 160: 0 → 1
Char. 200: 0 → 1
Char. 245: 0 → 1

***Trilophosaurus buettneri*:**
Char. 5: 1 → 0
Char. 11: 0 → 1
Char. 55: 1 → 0
Char. 93: 1 → 0
Char. 104: 0 → 1
Char. 106: 0 → 1
Char. 113: 0 → 1
Char. 122: 0 → 1
Char. 136: 1 → 0
Char. 144: 1 → 0
Char. 157: 0 → 1
Char. 159: 1 → 0
Char. 177: 0 → 12
Char. 194: 0 → 1
Char. 203: 1 → 2
Char. 207: 1 → 0
Char. 208: 1 → 0
Char. 272: 1 → 0

***Youngina capensis*:**
Char. 5: 1 → 0
Char. 21: 0 → 1
Char. 25: 0 → 1
Char. 27: 0 → 1
Char. 38: 1 → 0
Char. 43: 0 → 1
Char. 44: 1 → 0
Char. 56: 0 → 1
Char. 75: 0 → 1
Char. 84: 1 → 0
Char. 92: 1 → 0
Char. 94: 1 → 0

Char. 134: 0 → 1
Char. 154: 1 → 0
Char. 163: 1 → 0
Char. 170: 0 → 1
Char. 211: 0 → 1
Char. 215: 0 → 1
Char. 224: 0 → 1
Char. 239: 0 → 1
Char. 267: 0 → 1

**Node 50:**
Char. 46: 1 → 0
Char. 88: 1 → 0
Char. 89: 1 → 0
Char. 93: 1 → 0
Char. 176: 0 → 1
Char. 195: 0 → 2
Char. 198: 0 → 1
Char. 246: 1 → 2
Char. 259: 0 → 1
Char. 265: 1 → 0
Char. 270: 0 → 1

**Node 51:**
Char. 65: 0 → 1
Char. 131: 0 → 1
Char. 184: 0 → 1
Char. 205: 0 → 1
Char. 210: 0 → 1
Char. 219: 1 → 0
Char. 241: 0 → 1
Char. 246: 0 → 1
Char. 254: 0 → 1
Char. 255: 0 → 1
Char. 256: 0 → 1
Char. 267: 0 → 1
Char. 268: 0 → 1
Char. 269: 0 → 1

**Node 52:**
Char. 0: 1 → 2
Char. 15: 0 → 1
Char. 21: 0 → 1
Char. 33: 1 → 2
Char. 44: 1 → 0
Char. 62: 1 → 0
Char. 64: 0 → 1
Char. 84: 1 → 2
Char. 130: 0 → 1
Char. 134: 0 → 2
Char. 147: 0 → 1
Char. 148: 0 → 1
Char. 152: 0 → 1
Char. 155: 0 → 1
Char. 158: 0 → 1
Char. 161: 0 → 1
Char. 174: 0 → 1
Char. 181: 0 → 1
Char. 203: 1 → 2
Char. 204: 0 → 2
Char. 247: 0 → 1
Char. 251: 0 → 1
Char. 252: 0 → 1
Char. 253: 0 → 2

**Node 53:**
Char. 0: 0 → 1
Char. 20: 0 → 1
Char. 25: 1 → 0
Char. 29: 1 → 0
Char. 33: 0 → 1
Char. 40: 2 → 0
Char. 50: 1 → 0
Char. 57: 1 → 0
Char. 59: 0 → 1
Char. 62: 0 → 1
Char. 67: 0 → 1
Char. 72: 1 → 2
Char. 89: 0 → 1
Char. 94: 0 → 1
Char. 109: 0 → 1
Char. 111: 0 → 1
Char. 120: 0 → 1
Char. 127: 0 → 1
Char. 129: 0 → 1
Char. 135: 0 → 1
Char. 136: 0 → 1
Char. 139: 0 → 1
Char. 141: 0 → 1
Char. 179: 0 → 1
Char. 187: 0 → 1
Char. 188: 0 → 1
Char. 191: 0 → 1
Char. 193: 0 → 1
Char. 196: 0 → 1
Char. 201: 1 → 0
Char. 202: 0 → 1
Char. 207: 0 → 1
Char. 209: 0 → 1
Char. 219: 0 → 1
Char. 222: 0 → 1
Char. 237: 0 → 1
Char. 265: 0 → 1
Char. 266: 0 → 1
Char. 275: 1 → 0

**Node 54:**
Char. 132: 0 → 1
Char. 166: 0 → 1
Char. 276: 1 → 0

**Node 55:**
Char. 29: 0 → 1
Char. 84: 0 → 1
Char. 88: 0 → 1
Char. 169: 0 → 1

**Node 56:**
Char. 72: 0 → 1
Char. 81: 0 → 1
Char. 93: 0 → 1
Char. 97: 0 → 1
Char. 104: 1 → 0
Char. 144: 0 → 1
Char. 173: 0 → 1
Char. 183: 0 → 1

**Node 58:**
Char. 95: 0 → 1
Char. 113: 0 → 1
Char. 114: 0 → 1

**Node 59:**
Char. 20: 0 → 1
Char. 47: 0 → 1
Char. 79: 0 → 1
Char. 110: 0 → 1
Char. 131: 0 → 1
Char. 137: 0 → 1
Char. 140: 0 → 2
Char. 147: 0 → 1

**Node 60:**
Char. 19: 0 → 1
Char. 92: 1 → 0

**Node 61:**
Char. 4: 0 → 1
Char. 15: 0 → 1
Char. 29: 0 → 1
Char. 213: 0 → 1
Char. 226: 0 → 2
Char. 228: 0 → 1
Char. 275: 0 → 1

**Node 62:**
Char. 43: 0 → 1
Char. 58: 0 → 1
Char. 61: 0 → 1
Char. 66: 0 → 1
Char. 69: 0 → 1
Char. 107: 0 → 1
Char. 126: 0 → 1
Char. 131: 0 → 1
Char. 134: 0 → 1
Char. 140: 0 → 1
Char. 147: 0 → 1
Char. 148: 0 → 1
Char. 150: 0 → 1
Char. 167: 0 → 1
Char. 176: 0 → 1
Char. 190: 0 → 1
Char. 205: 0 → 1
Char. 208: 0 → 1
Char. 230: 0 → 1
Char. 239: 0 → 1
Char. 260: 2 → 1

**Node 63:**
Char. 73: 0 → 1
Char. 131: 1 → 0
Char. 205: 0 → 1
Char. 273: 0 → 1
Char. 276: 0 → 1

**Node 64:**
Char. 18: 0 → 1
Char. 25: 1 → 0
Char. 37: 0 → 1
Char. 103: 0 → 1
Char. 106: 0 → 1
Char. 107: 0 → 1
Char. 110: 0 → 1
Char. 126: 0 → 1
Char. 150: 0 → 1

**Node 65:**
Char. 186: 0 → 1

**Node 66:**
Char. 148: 0 → 1
Char. 183: 1 → 2
Char. 194: 0 → 1
Char. 201: 1 → 0
Char. 211: 0 → 1
Char. 226: 0 → 1
Char. 240: 1 → 0
Char. 252: 0 → 1

**Node 67:**
Char. 87: 0 → 1
Char. 110: 0 → 1

**Node 68:**
Char. 79: 0 → 1
Char. 133: 0 → 1

**Node 69:**
Char. 100: 0 → 1
Char. 113: 0 → 1

**Node 70:**
Char. 38: 1 → 2
Char. 39: 0 → 1
Char. 50: 1 → 0
Char. 58: 0 → 1
Char. 59: 0 → 1
Char. 60: 0 → 1
Char. 72: 1 → 2
Char. 83: 0 → 1
Char. 85: 1 → 0
Char. 88: 1 → 0
Char. 95: 0 → 1
Char. 104: 0 → 1
Char. 105: 0 → 1
Char. 106: 0 → 2
Char. 107: 0 → 1
Char. 109: 0 → 1
Char. 110: 0 → 1
Char. 146: 1 → 0
Char. 148: 0 → 1
Char. 155: 0 → 2
Char. 183: 1 → 2

**Node 71:**
Char. 33: 1 → 0
Char. 38: 2 → 1
Char. 39: 1 → 0
Char. 42: 0 → 1
Char. 43: 0 → 1
Char. 46: 1 → 0
Char. 49: 1 → 0
Char. 71: 1 → 0
Char. 83: 0 → 2
Char. 101: 0 → 1
Char. 103: 0 → 1
Char. 106: 0 → 1
Char. 161: 0 → 1
Char. 163: 1 → 0
Char. 172: 0 → 2
Char. 174: 0 → 1
Char. 188: 0 → 1
Char. 203: 1 → 2
Char. 212: 0 → 1
Char. 216: 1 → 0
Char. 236: 0 → 1
Char. 238: 0 → 2
Char. 241: 0 → 1
Char. 242: 0 → 1
Char. 273: 0 → 1
Char. 274: 0 → 1
Char. 275: 1 → 0

**Node 72:**
Char. 52: 0 → 1
Char. 75: 0 → 1
Char. 84: 1 → 0
Char. 87: 0 → 1
Char. 110: 0 → 1
Char. 121: 0 → 1
Char. 180: 1 → 0
Char. 230: 0 → 1

**Node 73:**
Char. 29: 1 → 0
Char. 44: 1 → 0
Char. 59: 0 → 1
Char. 60: 0 → 1
Char. 66: 0 → 1
Char. 67: 0 → 1
Char. 72: 1 → 2
Char. 78: 1 → 0
Char. 84: 1 → 0
Char. 88: 1 → 0
Char. 93: 1 → 0
Char. 111: 0 → 1
Char. 123: 1 → 0
Char. 129: 0 → 1
Char. 154: 1 → 0
Char. 169: 1 → 0
Char. 170: 0 → 1
Char. 192: 1 → 0
Char. 197: 0 → 1

**Node 74:**
Char. 83: 0 → 1
Char. 85: 1 → 0
Char. 107: 0 → 1

**Node 75:**
Char. 2: 0 → 1
Char. 6: 0 → 1
Char. 186: 0 → 1
Char. 198: 0 → 1
Char. 200: 0 → 1
Char. 220: 0 → 1
Char. 239: 1 → 0

**Node 76:**
Char. 98: 1 → 0
Char. 113: 0 → 1
Char. 181: 0 → 1
Char. 206: 0 → 2

**Node 77:**
Char. 35: 0 → 1
Char. 102: 0 → 1
Char. 103: 0 → 1
Char. 184: 0 → 1
Char. 201: 0 → 1
Char. 210: 0 → 1
Char. 223: 0 → 1
Char. 229: 0 → 1

**Node 78:**
Char. 17: 0 → 1
Char. 42: 0 → 1
Char. 251: 0 → 1
Char. 264: 0 → 1

**Node 79:**
Char. 41: 0 → 1
Char. 61: 1 → 2
Char. 112: 1 → 0
Char. 138: 0 → 1
Char. 146: 1 → 0
Char. 155: 0 → 1
Char. 192: 1 → 2
Char. 224: 0 → 1
Char. 226: 0 → 1
Char. 227: 0 → 1
Char. 233: 0 → 1
Char. 267: 01 → 2

**Node 80:**
Char. 61: 1 → 3
Char. 90: 0 → 1
Char. 91: 0 → 1
Char. 109: 1 → 0
Char. 155: 0 → 1
Char. 209: 1 → 0

**ANALYSIS 50**
**(ALL TAXA, IMPLIED WEIGHTING, K = 4.25)**

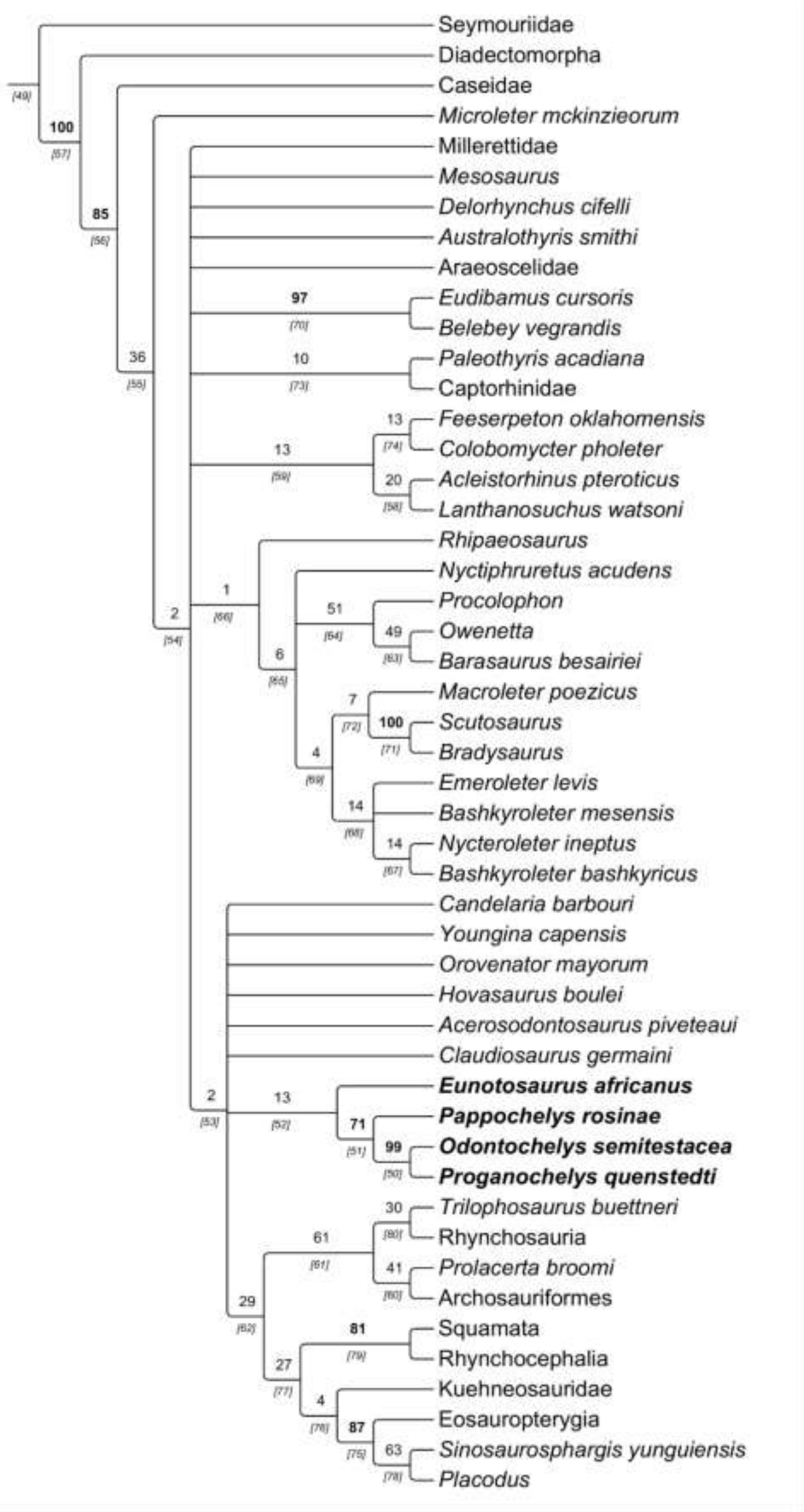

***Proganochelys quenstedti*:**
Char. 8: 0 → 1
Char. 11: 0 → 1
Char. 106: 0 → 1
Char. 108: 1 → 0
Char. 128: 0 → 1
Char. 175: 0 → 1
Char. 202: 1 → 0
Char. 207: 1 → 0
Char. 209: 1 → 0
Char. 244: 0 → 1
Char. 248: 0 → 1
Char. 250: 0 → 1
Char. 252: 1 → 0
Char. 262: 0 → 1

***Pappochelys rosinae*:**
Char. 0: 2 → 0
Char. 1: 0 → 1
Char. 5: 1 → 0
Char. 12: 0 → 1
Char. 41: 0 → 1
Char. 49: 0 → 1
Char. 75: 0 → 1
Char. 169: 1 → 0
Char. 260: 2 → 0

***Odontochelys semitestacea*:**
Char. 43: 0 → 1

***Eunotosaurus africanus*:**
Char. 19: 0 → 1
Char. 43: 0 → 1
Char. 59: 1 → 0
Char. 72: 2 → 1
Char. 76: 0 → 1
Char. 97: 1 → 0
Char. 103: 0 → 1
Char. 153: 1 → 0
Char. 191: 1 → 0
Char. 192: 1 → 0
Char. 202: 1 → 0
Char. 211: 0 → 1
Char. 222: 1 → 0
Char. 237: 1 → 0
Char. 248: 0 → 1
Char. 249: 0 → 2
Char. 250: 0 → 1
Char. 263: 0 → 1
Char. 273: 0 → 1
Char. 274: 0 → 1
Char. 275: 0 → 1
Char. 276: 0 → 1
Char. 277: 0 → 1

***Acerosodontosaurus piveteaui*:**
Char. 78: 1 → 0
Char. 81: 1 → 0
Char. 127: 1 → 0
Char. 128: 0 → 1
Char. 155: 0 → 1
Char. 206: 0 → 2
Char. 208: 0 → 1
Char. 278: 03 → 1

***Acleistorhinus pteroticus*:**
Char. 21: 0 → 1
Char. 146: 1 → 0

**Araeoscelidae:**
Char. 0: 0 → 1
Char. 27: 0 → 1
Char. 28: 0 → 1
Char. 29: 1 → 0
Char. 38: 1 → 0
Char. 40: 2 → 0
Char. 43: 0 → 1
Char. 50: 1 → 0
Char. 57: 1 → 0
Char. 59: 0 → 1
Char. 60: 0 → 1
Char. 67: 0 → 1
Char. 72: 1 → 2
Char. 84: 1 → 0
Char. 89: 0 → 1
Char. 106: 0 → 1
Char. 111: 0 → 1
Char. 116: 1 → 0
Char. 117: 01 → 2
Char. 120: 0 → 1
Char. 154: 1 → 0
Char. 166: 1 → 0
Char. 169: 1 → 0
Char. 170: 0 → 1
Char. 193: 0 → 1
Char. 197: 0 → 1
Char. 222: 0 → 1
Char. 224: 0 → 1
Char. 237: 0 → 1
Char. 239: 0 → 1
Char. 266: 0 → 1
Char. 278: 3 → 1

**Archosauriformes:**
Char. 32: 0 → 1
Char. 94: 1 → 0
Char. 112: 1 → 0
Char. 152: 0 → 1
Char. 154: 01 → 2
Char. 166: 1 → 0
Char. 171: 0 → 1
Char. 185: 0 → 1
Char. 201: 0 → 1
Char. 204: 0 → 1
Char. 218: 0 → 3
Char. 224: 0 → 1
Char. 242: 0 → 1

***Australothyris smithi*:**
Char. 24: 0 → 1
Char. 34: 0 → 1
Char. 55: 1 → 0
Char. 57: 1 → 0
Char. 71: 1 → 0
Char. 79: 0 → 1
Char. 83: 0 → 1
Char. 85: 1 → 0
Char. 98: 1 → 0
Char. 100: 0 → 1
Char. 103: 0 → 1
Char. 110: 0 → 1
Char. 123: 1 → 0
Char. 129: 0 → 1
Char. 131: 0 → 1
Char. 132: 1 → 0
Char. 144: 1 → 0
Char. 147: 0 → 1
Char. 149: 1 → 0
Char. 150: 0 → 1
Char. 192: 1 → 0

***Barasaurus besairiei*:**
Char. 33: 1 → 0

***Bashkyroleter bashkyricus*:**
Char. 275: 1 → 0

***Bashkyroleter mesensis*:**
Char. 12: 0 → 1
Char. 66: 1 → 0
Char. 169: 1 → 0

***Belebey vegrandis*:**
Char. 154: 1 → 0

***Bradysaurus* spp.:**
Char. 19: 0 → 1
Char. 25: 1 → 0
Char. 73: 0 → 1
Char. 79: 0 → 1
Char. 135: 1 → 0
Char. 249: 0 → 1

***Candelaria barbouri*:**
Char. 0: 1 → 0
Char. 1: 0 → 1
Char. 5: 1 → 0
Char. 8: 0 → 1
Char. 15: 0 → 1
Char. 33: 1 → 2
Char. 49: 0 → 1
Char. 55: 1 → 0
Char. 59: 1 → 0
Char. 62: 1 → 0
Char. 72: 2 → 1
Char. 76: 0 → 1
Char. 79: 0 → 2
Char. 83: 0 → 1
Char. 88: 1 → 0
Char. 92: 1 → 0
Char. 95: 0 → 1
Char. 126: 0 → 1
Char. 132: 1 → 0
Char. 154: 1 → 2
Char. 159: 1 → 0
Char. 169: 1 → 0
Char. 265: 1 → 0
Char. 275: 0 → 1
Char. 276: 0 → 1
Char. 277: 0 → 1

**Captorhinidae:**
Char. 3: 0 → 1
Char. 25: 1 → 0
Char. 26: 0 → 1
Char. 73: 0 → 1
Char. 75: 0 → 1
Char. 83: 0 → 1
Char. 108: 1 → 0
Char. 180: 1 → 0
Char. 183: 1 → 0
Char. 201: 1 → 0
Char. 203: 1 → 2
Char. 216: 1 → 0
Char. 240: 1 → 0

**Caseidae:**
Char. 38: 1 → 0
Char. 46: 1 → 0
Char. 50: 1 → 0
Char. 170: 0 → 1
Char. 194: 0 → 1
Char. 273: 0 → 1
Char. 274: 0 → 1
Char. 278: 3 → 2

***Claudiosaurus germaini*:**
Char. 24: 0 → 1
Char. 27: 0 → 1
Char. 34: 0 → 1
Char. 36: 0 → 1
Char. 56: 0 → 1
Char. 64: 0 → 1
Char. 70: 0 → 1
Char. 84: 1 → 0
Char. 105: 0 → 1
Char. 106: 0 → 1
Char. 117: 01 → 2
Char. 126: 0 → 1
Char. 127: 1 → 0
Char. 130: 0 → 1
Char. 131: 0 → 1
Char. 141: 1 → 0
Char. 144: 1 → 0
Char. 148: 0 → 1
Char. 154: 1 → 0
Char. 166: 1 → 0
Char. 182: 0 → 1
Char. 187: 1 → 0
Char. 190: 0 → 1
Char. 199: 0 → 1
Char. 201: 0 → 1
Char. 203: 1 → 2
Char. 204: 0 → 1
Char. 222: 1 → 0
Char. 234: 1 → 0
Char. 267: 0 → 1
Char. 272: 2 → 1

***Colobomycter pholeter*:**
Char. 21: 0 → 1
Char. 25: 1 → 0
Char. 84: 1 → 0
Char. 154: 1 → 0
Char. 167: 0 → 1
Char. 267: 0 → 1

***Delorhynchus cifelli*:**
Char. 18: 0 → 1
Char. 20: 0 → 1
Char. 21: 0 → 1
Char. 24: 0 → 1
Char. 26: 0 → 1
Char. 28: 0 → 1
Char. 33: 0 → 2
Char. 39: 0 → 1
Char. 52: 0 → 1
Char. 100: 0 → 1
Char. 111: 0 → 1
Char. 116: 1 → 0
Char. 119: 1 → 0
Char. 131: 0 → 1
Char. 147: 0 → 1
Char. 156: 1 → 0
Char. 167: 0 → 1
Char. 189: 0 → 1
Char. 191: 0 → 1
Char. 204: 0 → 2
Char. 267: 0 → 1

**Diadectomorpha:**
Char. 0: 0 → 1
Char. 64: 0 → 1
Char. 70: 0 → 1
Char. 122: 0 → 1
Char. 123: 1 → 0
Char. 146: 1 → 0
Char. 275: 1 → 0
Char. 278: 3 → 0

***Emeroleter levis*:**
Char. 51: 1 → 0

**Eosauropterygia:**
Char. 166: 1 → 0
Char. 174: 0 → 1
Char. 178: 0 → 1
Char. 194: 0 → 2
Char. 272: 1 → 0

***Eudibamus cursoris*:**
Char. 154: 1 → 2

***Feeserpeton oklahomensis*:**
Char. 51: 0 → 1
Char. 157: 0 → 1
Char. 158: 0 → 1

***Hovasaurus boulei*:**
Char. 41: 0 → 1
Char. 43: 0 → 1
Char. 55: 1 → 0
Char. 72: 2 → 1
Char. 77: 0 → 2
Char. 78: 1 → 0
Char. 79: 0 → 1
Char. 93: 1 → 0
Char. 113: 0 → 1
Char. 138: 0 → 1

Char. 141: 1 → 0
Char. 146: 1 → 0
Char. 204: 0 → 2
Char. 206: 0 → 2
Char. 215: 0 → 1
Char. 219: 1 → 0
Char. 224: 0 → 1

**Kuehneosauridae:**
Char. 7: 0 → 1
Char. 24: 0 → 1
Char. 27: 0 → 1
Char. 43: 1 → 0
Char. 79: 0 → 2
Char. 107: 1 → 0
Char. 140: 1 → 0
Char. 147: 1 → 0
Char. 148: 1 → 0
Char. 245: 0 → 1
Char. 278: 0 → 3

***Lanthanosuchus watsoni*:**
Char. 25: 1 → 0
Char. 51: 0 → 1
Char. 76: 0 → 1
Char. 86: 0 → 1
Char. 98: 1 → 0
Char. 138: 0 → 1
Char. 144: 1 → 0
Char. 154: 1 → 2

***Macroleter poezicus*:**
Char. 9: 0 → 1
Char. 26: 0 → 1
Char. 66: 1 → 2
Char. 126: 0 → 1
Char. 134: 0 → 2
Char. 140: 0 → 1
Char. 146: 1 → 0
Char. 169: 1 → 0

***Mesosaurus* spp.:**
Char. 0: 0 → 1
Char. 2: 0 → 1
Char. 6: 0 → 1
Char. 8: 0 → 1
Char. 9: 0 → 1
Char. 13: 0 → 1
Char. 19: 0 → 1
Char. 26: 0 → 1
Char. 29: 1 → 0
Char. 33: 0 → 1
Char. 38: 1 → 0
Char. 41: 0 → 1
Char. 50: 1 → 0
Char. 67: 0 → 1
Char. 76: 0 → 1
Char. 83: 0 → 1
Char. 84: 1 → 0
Char. 85: 1 → 0
Char. 94: 0 → 1
Char. 107: 0 → 1
Char. 109: 0 → 1
Char. 111: 0 → 1
Char. 115: 0 → 1
Char. 146: 1 → 0
Char. 148: 0 → 1
Char. 149: 1 → 0
Char. 164: 0 → 1
Char. 166: 1 → 0
Char. 167: 0 → 1
Char. 176: 0 → 1
Char. 183: 1 → 0
Char. 184: 0 → 1
Char. 192: 1 → 0
Char. 199: 0 → 1
Char. 202: 0 → 1
Char. 204: 0 → 2
Char. 206: 0 → 1
Char. 207: 0 → 1
Char. 209: 0 → 1
Char. 217: 0 → 1
Char. 219: 0 → 1
Char. 220: 0 → 1
Char. 231: 0 → 1
Char. 240: 1 → 0
Char. 260: 2 → 0
Char. 272: 2 → 0
Char. 278: 3 → 0

***Microleter mckinzieorum*:**
Char. 0: 0 → 1
Char. 18: 0 → 1
Char. 39: 0 → 1
Char. 57: 1 → 0
Char. 70: 0 → 1
Char. 76: 0 → 1
Char. 83: 0 → 1
Char. 94: 0 → 1
Char. 106: 0 → 1
Char. 110: 0 → 1
Char. 278: 3 → 1

**Millerettidae:**
Char. 24: 0 → 1
Char. 25: 1 → 0
Char. 44: 1 → 0
Char. 56: 0 → 1
Char. 57: 1 → 0
Char. 66: 0 → 2
Char. 78: 1 → 0
Char. 80: 1 → 0
Char. 84: 1 → 2
Char. 88: 1 → 0
Char. 96: 1 → 0
Char. 121: 0 → 1
Char. 124: 0 → 1
Char. 127: 0 → 1
Char. 135: 0 → 1
Char. 145: 0 → 1
Char. 166: 1 → 0
Char. 180: 1 → 0
Char. 192: 1 → 0
Char. 202: 0 → 1
Char. 211: 0 → 1
Char. 230: 0 → 1
Char. 248: 0 → 1
Char. 252: 0 → 1
Char. 253: 0 → 1

***Nycteroleter ineptus*:**
Char. 66: 1 → 0
Char. 278: 0 → 3

***Nyctiphruretus acudens*:**
Char. 0: 0 → 1
Char. 21: 0 → 1
Char. 33: 1 → 2
Char. 41: 0 → 1
Char. 66: 1 → 2
Char. 81: 1 → 0
Char. 83: 0 → 1
Char. 84: 1 → 2
Char. 94: 0 → 1
Char. 166: 1 → 0
Char. 224: 0 → 1
Char. 226: 1 → 0
Char. 266: 0 → 1
Char. 272: 2 → 1
Char. 276: 0 → 1

***Orovenator mayorum*:**
Char. 8: 0 → 1
Char. 24: 0 → 1
Char. 33: 1 → 0
Char. 36: 0 → 1
Char. 50: 0 → 1
Char. 92: 1 → 0
Char. 94: 1 → 0
Char. 135: 1 → 0
Char. 159: 1 → 0
Char. 160: 0 → 1
Char. 165: 0 → 1
Char. 278: 03 → 1

***Owenetta* spp.:**
Char. 142: 1 → 0
Char. 169: 1 → 0

***Paleothyris acadiana*:**
Char. 38: 1 → 0
Char. 50: 1 → 0
Char. 66: 1 → 2
Char. 102: 0 → 1
Char. 146: 1 → 0
Char. 237: 0 → 1
Char. 239: 0 → 1

***Placodus* spp.:**
Char. 0: 1 → 2
Char. 9: 0 → 1
Char. 12: 0 → 1
Char. 13: 0 → 1
Char. 19: 0 → 1
Char. 31: 0 → 1
Char. 46: 1 → 0
Char. 57: 0 → 1
Char. 78: 1 → 0
Char. 93: 1 → 0
Char. 102: 1 → 2
Char. 109: 1 → 0
Char. 140: 1 → 0
Char. 155: 0 → 1
Char. 163: 1 → 0
Char. 164: 0 → 1

***Procolophon* spp.:**
Char. 41: 0 → 1
Char. 69: 0 → 1
Char. 79: 0 → 1
Char. 83: 0 → 1
Char. 86: 0 → 1
Char. 88: 0 → 1
Char. 101: 0 → 1
Char. 117: 1 → 0
Char. 141: 0 → 1
Char. 149: 1 → 0
Char. 203: 1 → 2
Char. 204: 0 → 1
Char. 230: 0 → 1
Char. 237: 0 → 1
Char. 238: 0 → 1
Char. 272: 2 → 1
Char. 278: 3 → 0

***Prolacerta broomi*:**
Char. 58: 1 → 0
Char. 66: 1 → 0
Char. 67: 1 → 0
Char. 80: 1 → 0
Char. 139: 1 → 0
Char. 147: 1 → 0
Char. 192: 1 → 0
Char. 203: 1 → 2
Char. 206: 0 → 12

***Rhipaeosaurus* spp.:**
Char. 172: 0 → 1
Char. 277: 0 → 1

**Rhynchocephalia:**
Char. 0: 1 → 2
Char. 24: 0 → 1
Char. 77: 0 → 1
Char. 88: 1 → 0
Char. 94: 1 → 0
Char. 139: 1 → 0
Char. 167: 1 → 0
Char. 205: 1 → 0

**Rhynchosauria:**
Char. 0: 1 → 0
Char. 7: 0 → 1
Char. 9: 0 → 1
Char. 26: 0 → 1
Char. 44: 1 → 0
Char. 68: 0 → 1
Char. 99: 1 → 0
Char. 150: 1 → 0
Char. 160: 0 → 1
Char. 161: 0 → 1
Char. 171: 0 → 2
Char. 223: 0 → 1
Char. 224: 0 → 1
Char. 241: 0 → 1

***Scutosaurus* spp.:**
Char. 175: 0 → 1
Char. 190: 0 → 1
Char. 218: 0 → 1
Char. 243: 0 → 2
Char. 244: 0 → 1
Char. 251: 0 → 1

***Sinosaurosphargis yunguiensis*:**
Char. 8: 0 → 1
Char. 30: 0 → 1
Char. 53: 0 → 1
Char. 89: 1 → 0
Char. 127: 1 → 0
Char. 150: 1 → 0
Char. 154: 0 → 2
Char. 167: 1 → 0
Char. 253: 0 → 1
Char. 255: 0 → 1

**Squamata:**
Char. 45: 0 → 1
Char. 79: 0 → 2
Char. 80: 1 → 0
Char. 92: 1 → 0
Char. 109: 1 → 0
Char. 160: 0 → 1
Char. 200: 0 → 1
Char. 245: 0 → 1

***Trilophosaurus buettneri*:**
Char. 5: 1 → 0
Char. 11: 0 → 1
Char. 55: 1 → 0
Char. 93: 1 → 0
Char. 104: 0 → 1
Char. 106: 0 → 1
Char. 113: 0 → 1
Char. 122: 0 → 1
Char. 136: 1 → 0
Char. 144: 1 → 0
Char. 157: 0 → 1
Char. 159: 1 → 0
Char. 177: 0 → 12
Char. 194: 0 → 1
Char. 203: 1 → 2
Char. 207: 1 → 0
Char. 208: 1 → 0
Char. 272: 1 → 0

***Youngina capensis*:**
Char. 5: 1 → 0
Char. 21: 0 → 1
Char. 25: 0 → 1
Char. 27: 0 → 1
Char. 38: 1 → 0
Char. 43: 0 → 1
Char. 44: 1 → 0
Char. 56: 0 → 1
Char. 75: 0 → 1
Char. 84: 1 → 0
Char. 92: 1 → 0
Char. 94: 1 → 0

Char. 134: 0 → 1
Char. 154: 1 → 0
Char. 163: 1 → 0
Char. 170: 0 → 1
Char. 211: 0 → 1
Char. 215: 0 → 1
Char. 224: 0 → 1
Char. 239: 0 → 1
Char. 267: 0 → 1

**Node 50:**
Char. 46: 1 → 0
Char. 88: 1 → 0
Char. 89: 1 → 0
Char. 93: 1 → 0
Char. 176: 0 → 1
Char. 195: 0 → 2
Char. 198: 0 → 1
Char. 246: 1 → 2
Char. 259: 0 → 1
Char. 265: 1 → 0
Char. 270: 0 → 1

**Node 51:**
Char. 65: 0 → 1
Char. 131: 0 → 1
Char. 184: 0 → 1
Char. 205: 0 → 1
Char. 210: 0 → 1
Char. 219: 1 → 0
Char. 241: 0 → 1
Char. 246: 0 → 1
Char. 254: 0 → 1
Char. 255: 0 → 1
Char. 256: 0 → 1
Char. 267: 0 → 1
Char. 268: 0 → 1
Char. 269: 0 → 1

**Node 52:**
Char. 0: 1 → 2
Char. 15: 0 → 1
Char. 21: 0 → 1
Char. 33: 1 → 2
Char. 44: 1 → 0
Char. 62: 1 → 0
Char. 64: 0 → 1
Char. 84: 1 → 2
Char. 130: 0 → 1
Char. 134: 0 → 2
Char. 147: 0 → 1
Char. 148: 0 → 1
Char. 152: 0 → 1
Char. 155: 0 → 1
Char. 158: 0 → 1
Char. 161: 0 → 1
Char. 174: 0 → 1
Char. 181: 0 → 1
Char. 203: 1 → 2
Char. 204: 0 → 2
Char. 247: 0 → 1
Char. 251: 0 → 1
Char. 252: 0 → 1
Char. 253: 0 → 2

**Node 53:**
Char. 0: 0 → 1
Char. 20: 0 → 1
Char. 25: 1 → 0
Char. 29: 1 → 0
Char. 33: 0 → 1
Char. 40: 2 → 0
Char. 50: 1 → 0
Char. 57: 1 → 0
Char. 59: 0 → 1
Char. 62: 0 → 1
Char. 67: 0 → 1
Char. 72: 1 → 2
Char. 89: 0 → 1
Char. 94: 0 → 1
Char. 109: 0 → 1
Char. 111: 0 → 1
Char. 120: 0 → 1
Char. 127: 0 → 1
Char. 129: 0 → 1
Char. 135: 0 → 1
Char. 136: 0 → 1
Char. 139: 0 → 1
Char. 141: 0 → 1
Char. 179: 0 → 1
Char. 187: 0 → 1
Char. 188: 0 → 1
Char. 191: 0 → 1
Char. 193: 0 → 1
Char. 196: 0 → 1
Char. 201: 1 → 0
Char. 202: 0 → 1
Char. 207: 0 → 1
Char. 209: 0 → 1
Char. 219: 0 → 1
Char. 222: 0 → 1
Char. 237: 0 → 1
Char. 265: 0 → 1
Char. 266: 0 → 1
Char. 275: 1 → 0

**Node 54:**
Char. 132: 0 → 1
Char. 166: 0 → 1
Char. 276: 1 → 0

**Node 55:**
Char. 29: 0 → 1
Char. 84: 0 → 1
Char. 88: 0 → 1
Char. 169: 0 → 1

**Node 56:**
Char. 72: 0 → 1
Char. 81: 0 → 1
Char. 93: 0 → 1
Char. 97: 0 → 1
Char. 104: 1 → 0
Char. 144: 0 → 1
Char. 173: 0 → 1
Char. 183: 0 → 1

**Node 58:**
Char. 95: 0 → 1
Char. 113: 0 → 1
Char. 114: 0 → 1

**Node 59:**
Char. 20: 0 → 1
Char. 47: 0 → 1
Char. 79: 0 → 1
Char. 110: 0 → 1
Char. 131: 0 → 1
Char. 137: 0 → 1
Char. 140: 0 → 2
Char. 147: 0 → 1

**Node 60:**
Char. 19: 0 → 1
Char. 92: 1 → 0

**Node 61:**
Char. 4: 0 → 1
Char. 15: 0 → 1
Char. 29: 0 → 1
Char. 213: 0 → 1
Char. 226: 0 → 2
Char. 228: 0 → 1
Char. 275: 0 → 1

**Node 62:**
Char. 43: 0 → 1
Char. 58: 0 → 1
Char. 61: 0 → 1
Char. 66: 0 → 1
Char. 69: 0 → 1
Char. 107: 0 → 1
Char. 126: 0 → 1
Char. 131: 0 → 1
Char. 134: 0 → 1
Char. 140: 0 → 1
Char. 147: 0 → 1
Char. 148: 0 → 1
Char. 150: 0 → 1
Char. 167: 0 → 1
Char. 176: 0 → 1
Char. 190: 0 → 1
Char. 205: 0 → 1
Char. 208: 0 → 1
Char. 230: 0 → 1
Char. 239: 0 → 1
Char. 260: 2 → 1

**Node 63:**
Char. 73: 0 → 1
Char. 131: 1 → 0
Char. 205: 0 → 1
Char. 273: 0 → 1
Char. 276: 0 → 1

**Node 64:**
Char. 18: 0 → 1
Char. 25: 1 → 0
Char. 37: 0 → 1
Char. 103: 0 → 1
Char. 106: 0 → 1
Char. 107: 0 → 1
Char. 110: 0 → 1
Char. 126: 0 → 1
Char. 150: 0 → 1

**Node 65:**
Char. 186: 0 → 1

**Node 66:**
Char. 148: 0 → 1
Char. 183: 1 → 2
Char. 194: 0 → 1
Char. 201: 1 → 0
Char. 211: 0 → 1
Char. 226: 0 → 1
Char. 240: 1 → 0
Char. 252: 0 → 1

**Node 67:**
Char. 87: 0 → 1
Char. 110: 0 → 1

**Node 68:**
Char. 79: 0 → 1
Char. 133: 0 → 1

**Node 69:**
Char. 100: 0 → 1
Char. 113: 0 → 1

**Node 70:**
Char. 38: 1 → 2
Char. 39: 0 → 1
Char. 50: 1 → 0
Char. 58: 0 → 1
Char. 59: 0 → 1
Char. 60: 0 → 1
Char. 72: 1 → 2
Char. 83: 0 → 1
Char. 85: 1 → 0
Char. 88: 1 → 0
Char. 95: 0 → 1
Char. 104: 0 → 1
Char. 105: 0 → 1
Char. 106: 0 → 2
Char. 107: 0 → 1
Char. 109: 0 → 1
Char. 110: 0 → 1
Char. 146: 1 → 0
Char. 148: 0 → 1
Char. 155: 0 → 2
Char. 183: 1 → 2

**Node 71:**
Char. 33: 1 → 0
Char. 38: 2 → 1
Char. 39: 1 → 0
Char. 42: 0 → 1
Char. 43: 0 → 1
Char. 46: 1 → 0
Char. 49: 1 → 0
Char. 71: 1 → 0
Char. 83: 0 → 2
Char. 101: 0 → 1
Char. 103: 0 → 1
Char. 106: 0 → 1
Char. 161: 0 → 1
Char. 163: 1 → 0
Char. 172: 0 → 2
Char. 174: 0 → 1
Char. 188: 0 → 1
Char. 203: 1 → 2
Char. 212: 0 → 1
Char. 216: 1 → 0
Char. 236: 0 → 1
Char. 238: 0 → 2
Char. 241: 0 → 1
Char. 242: 0 → 1
Char. 273: 0 → 1
Char. 274: 0 → 1
Char. 275: 1 → 0

**Node 72:**
Char. 52: 0 → 1
Char. 75: 0 → 1
Char. 84: 1 → 0
Char. 87: 0 → 1
Char. 110: 0 → 1
Char. 121: 0 → 1
Char. 180: 1 → 0
Char. 230: 0 → 1

**Node 73:**
Char. 29: 1 → 0
Char. 44: 1 → 0
Char. 59: 0 → 1
Char. 60: 0 → 1
Char. 66: 0 → 1
Char. 67: 0 → 1
Char. 72: 1 → 2
Char. 78: 1 → 0
Char. 84: 1 → 0
Char. 88: 1 → 0
Char. 93: 1 → 0
Char. 111: 0 → 1
Char. 123: 1 → 0
Char. 129: 0 → 1
Char. 154: 1 → 0
Char. 169: 1 → 0
Char. 170: 0 → 1
Char. 192: 1 → 0
Char. 197: 0 → 1

**Node 74:**
Char. 83: 0 → 1
Char. 85: 1 → 0
Char. 107: 0 → 1

**Node 75:**
Char. 2: 0 → 1
Char. 6: 0 → 1
Char. 186: 0 → 1
Char. 198: 0 → 1
Char. 200: 0 → 1
Char. 220: 0 → 1
Char. 239: 1 → 0

**Node 76:**
Char. 98: 1 → 0
Char. 113: 0 → 1
Char. 181: 0 → 1
Char. 206: 0 → 2

**Node 77:**
Char. 35: 0 → 1
Char. 102: 0 → 1
Char. 103: 0 → 1
Char. 184: 0 → 1
Char. 201: 0 → 1
Char. 210: 0 → 1
Char. 223: 0 → 1
Char. 229: 0 → 1

**Node 78:**
Char. 17: 0 → 1
Char. 42: 0 → 1
Char. 251: 0 → 1
Char. 264: 0 → 1

**Node 79:**
Char. 41: 0 → 1
Char. 61: 1 → 2
Char. 112: 1 → 0
Char. 138: 0 → 1
Char. 146: 1 → 0
Char. 155: 0 → 1
Char. 192: 1 → 2
Char. 224: 0 → 1
Char. 226: 0 → 1
Char. 227: 0 → 1
Char. 233: 0 → 1
Char. 267: 01 → 2

**Node 80:**
Char. 61: 1 → 3
Char. 90: 0 → 1
Char. 91: 0 → 1
Char. 109: 1 → 0
Char. 155: 0 → 1
Char. 209: 1 → 0

**Analysis 51**
**(all taxa, implied weighting, K = 4.375)**

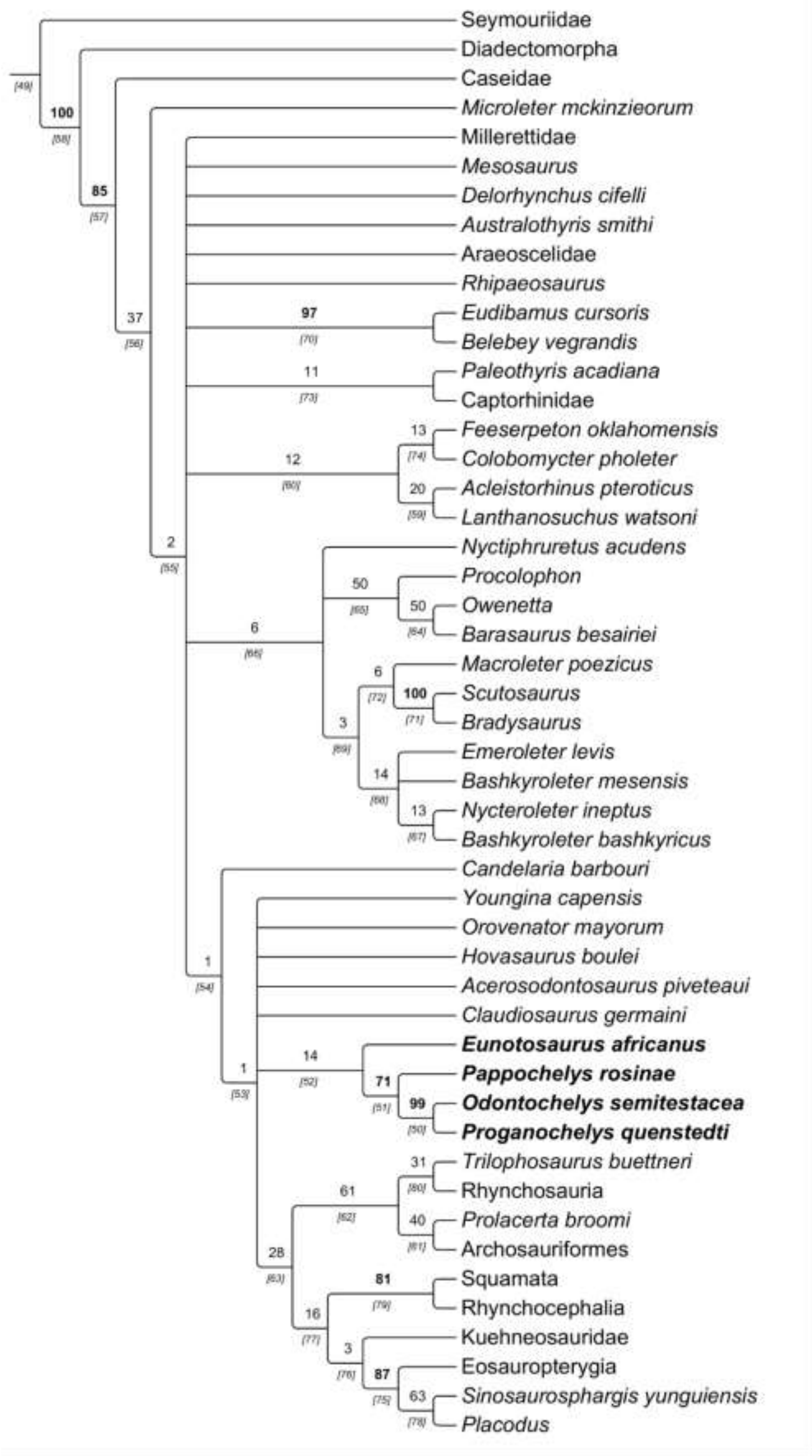

***Proganochelys quenstedti*:**
Char. 8: 0 → 1
Char. 11: 0 → 1
Char. 106: 0 → 1
Char. 108: 1 → 0
Char. 128: 0 → 1
Char. 175: 0 → 1
Char. 202: 1 → 0
Char. 207: 1 → 0
Char. 209: 1 → 0
Char. 244: 0 → 1
Char. 248: 0 → 1
Char. 250: 0 → 1
Char. 252: 1 → 0
Char. 262: 0 → 1

***Pappochelys rosinae*:**
Char. 0: 2 → 0
Char. 1: 0 → 1
Char. 5: 1 → 0
Char. 12: 0 → 1
Char. 41: 0 → 1
Char. 49: 0 → 1
Char. 75: 0 → 1
Char. 169: 1 → 0
Char. 260: 2 → 0

***Eunotosaurus africanus*:**
Char. 19: 0 → 1
Char. 59: 1 → 0
Char. 60: 1 → 0
Char. 72: 2 → 1
Char. 76: 0 → 1
Char. 97: 1 → 0
Char. 103: 0 → 1
Char. 153: 1 → 0
Char. 191: 1 → 0
Char. 192: 1 → 0
Char. 202: 1 → 0
Char. 211: 0 → 1
Char. 222: 1 → 0
Char. 237: 1 → 0
Char. 248: 0 → 1
Char. 249: 0 → 2
Char. 250: 0 → 1
Char. 263: 0 → 1
Char. 273: 0 → 1
Char. 274: 0 → 1
Char. 275: 0 → 1
Char. 276: 0 → 1
Char. 277: 0 → 1
Char. 278: 0 → 3

***Acerosodontosaurus piveteaui*:**
Char. 78: 1 → 0
Char. 81: 1 → 0
Char. 127: 1 → 0
Char. 128: 0 → 1
Char. 155: 0 → 1
Char. 206: 0 → 2
Char. 208: 0 → 1
Char. 278: 0 → 1

***Acleistorhinus pteroticus*:**
Char. 21: 0 → 1
Char. 146: 1 → 0

**Araeoscelidae:**
Char. 0: 0 → 1
Char. 27: 0 → 1
Char. 28: 0 → 1
Char. 29: 1 → 0
Char. 38: 1 → 0
Char. 40: 2 → 0
Char. 43: 0 → 1
Char. 50: 1 → 0
Char. 57: 1 → 0
Char. 59: 0 → 1
Char. 60: 0 → 1
Char. 67: 0 → 1
Char. 72: 1 → 2
Char. 84: 1 → 0
Char. 89: 0 → 1
Char. 106: 0 → 1
Char. 111: 0 → 1
Char. 116: 1 → 0
Char. 117: 01 → 2
Char. 120: 0 → 1
Char. 154: 1 → 0
Char. 166: 1 → 0
Char. 169: 1 → 0
Char. 170: 0 → 1
Char. 193: 0 → 1
Char. 197: 0 → 1
Char. 222: 0 → 1
Char. 224: 0 → 1
Char. 234: 0 → 1
Char. 237: 0 → 1
Char. 239: 0 → 1
Char. 266: 0 → 1
Char. 278: 3 → 1

**Archosauriformes:**
Char. 32: 0 → 1
Char. 94: 1 → 0
Char. 112: 1 → 0
Char. 152: 0 → 1
Char. 154: 01 → 2
Char. 166: 1 → 0
Char. 171: 0 → 1
Char. 185: 0 → 1
Char. 201: 0 → 1
Char. 204: 0 → 1
Char. 218: 0 → 3
Char. 224: 0 → 1
Char. 242: 0 → 1

***Australothyris smithi*:**
Char. 24: 0 → 1
Char. 34: 0 → 1
Char. 55: 1 → 0
Char. 57: 1 → 0
Char. 71: 1 → 0
Char. 79: 0 → 1
Char. 85: 1 → 0
Char. 98: 1 → 0
Char. 100: 0 → 1
Char. 103: 0 → 1
Char. 110: 0 → 1
Char. 123: 1 → 0
Char. 129: 0 → 1
Char. 131: 0 → 1
Char. 132: 1 → 0
Char. 144: 1 → 0
Char. 147: 0 → 1
Char. 149: 1 → 0
Char. 150: 0 → 1
Char. 192: 1 → 0

***Barasaurus besairiei*:**
Char. 33: 1 → 0

***Bashkyroleter bashkyricus*:**
Char. 275: 1 → 0

***Bashkyroleter mesensis*:**
Char. 12: 0 → 1
Char. 66: 1 → 0
Char. 169: 1 → 0

***Belebey vegrandis*:**
Char. 154: 1 → 0

***Bradysaurus* spp.:**
Char. 19: 0 → 1
Char. 25: 1 → 0
Char. 73: 0 → 1
Char. 79: 0 → 1
Char. 135: 1 → 0
Char. 249: 0 → 1

***Candelaria barbouri*:**
Char. 1: 0 → 1
Char. 8: 0 → 1
Char. 15: 0 → 1
Char. 33: 1 → 2
Char. 49: 0 → 1
Char. 55: 1 → 0
Char. 76: 0 → 1
Char. 79: 0 → 2
Char. 88: 1 → 0
Char. 95: 0 → 1
Char. 126: 0 → 1
Char. 132: 1 → 0
Char. 154: 1 → 2
Char. 169: 1 → 0
Char. 276: 0 → 1
Char. 277: 0 → 1

**Captorhinidae:**
Char. 3: 0 → 1
Char. 25: 1 → 0
Char. 26: 0 → 1
Char. 73: 0 → 1
Char. 75: 0 → 1
Char. 108: 1 → 0
Char. 180: 1 → 0
Char. 183: 1 → 0
Char. 201: 1 → 0
Char. 203: 1 → 2
Char. 216: 1 → 0

**Caseidae:**
Char. 38: 1 → 0
Char. 46: 1 → 0
Char. 50: 1 → 0
Char. 170: 0 → 1
Char. 194: 0 → 1
Char. 273: 0 → 1
Char. 274: 0 → 1
Char. 278: 3 → 2

***Claudiosaurus germaini*:**
Char. 24: 0 → 1
Char. 27: 0 → 1
Char. 34: 0 → 1
Char. 36: 0 → 1
Char. 56: 0 → 1
Char. 64: 0 → 1
Char. 70: 0 → 1
Char. 84: 1 → 0
Char. 105: 0 → 1
Char. 106: 0 → 1
Char. 117: 01 → 2
Char. 126: 0 → 1
Char. 127: 1 → 0
Char. 130: 0 → 1
Char. 131: 0 → 1
Char. 141: 1 → 0
Char. 144: 1 → 0
Char. 166: 1 → 0
Char. 182: 0 → 1
Char. 187: 1 → 0
Char. 190: 0 → 1
Char. 199: 0 → 1
Char. 201: 0 → 1
Char. 203: 1 → 2
Char. 204: 0 → 1
Char. 222: 1 → 0
Char. 234: 1 → 0
Char. 272: 2 → 1

***Colobomycter pholeter*:**
Char. 21: 0 → 1
Char. 25: 1 → 0
Char. 84: 1 → 0
Char. 154: 1 → 0
Char. 167: 0 → 1
Char. 267: 0 → 1

***Delorhynchus cifelli*:**
Char. 5: 0 → 1
Char. 18: 0 → 1
Char. 20: 0 → 1
Char. 21: 0 → 1
Char. 24: 0 → 1
Char. 26: 0 → 1
Char. 28: 0 → 1
Char. 33: 0 → 2
Char. 39: 0 → 1
Char. 52: 0 → 1
Char. 100: 0 → 1
Char. 111: 0 → 1
Char. 116: 1 → 0
Char. 119: 1 → 0
Char. 131: 0 → 1
Char. 147: 0 → 1
Char. 156: 1 → 0
Char. 167: 0 → 1
Char. 189: 0 → 1
Char. 191: 0 → 1
Char. 204: 0 → 2
Char. 267: 0 → 1

**Diadectomorpha:**
Char. 0: 0 → 1
Char. 64: 0 → 1
Char. 70: 0 → 1
Char. 122: 0 → 1
Char. 123: 1 → 0
Char. 146: 1 → 0
Char. 275: 1 → 0
Char. 278: 3 → 0

***Emeroleter levis*:**
Char. 51: 1 → 0

**Eosauropterygia:**
Char. 166: 1 → 0
Char. 174: 0 → 1
Char. 178: 0 → 1
Char. 194: 0 → 2
Char. 272: 1 → 0

***Eudibamus cursoris*:**
Char. 154: 1 → 2

***Feeserpeton oklahomensis*:**
Char. 51: 0 → 1
Char. 157: 0 → 1
Char. 158: 0 → 1

***Hovasaurus boulei*:**
Char. 41: 0 → 1
Char. 55: 1 → 0
Char. 60: 1 → 0
Char. 72: 2 → 1
Char. 77: 0 → 2
Char. 78: 1 → 0
Char. 79: 0 → 1
Char. 93: 1 → 0
Char. 113: 0 → 1
Char. 138: 0 → 1
Char. 141: 1 → 0
Char. 146: 1 → 0
Char. 204: 0 → 2
Char. 206: 0 → 2
Char. 215: 0 → 1
Char. 219: 1 → 0
Char. 224: 0 → 1
Char. 278: 0 → 3

**Kuehneosauridae:**
Char. 7: 0 → 1
Char. 24: 0 → 1
Char. 27: 0 → 1
Char. 43: 1 → 0
Char. 79: 0 → 2
Char. 107: 1 → 0
Char. 140: 1 → 0
Char. 147: 1 → 0

Char. 148: 1 → 0
Char. 245: 0 → 1
Char. 278: 0 → 3

***Lanthanosuchus watsoni*:**
Char. 25: 1 → 0
Char. 51: 0 → 1
Char. 76: 0 → 1
Char. 86: 0 → 1
Char. 98: 1 → 0
Char. 138: 0 → 1
Char. 144: 1 → 0
Char. 154: 1 → 2

***Macroleter poezicus*:**
Char. 9: 0 → 1
Char. 26: 0 → 1
Char. 66: 1 → 2
Char. 126: 0 → 1
Char. 134: 0 → 2
Char. 140: 0 → 1
Char. 146: 1 → 0
Char. 169: 1 → 0

***Mesosaurus* spp.:**
Char. 0: 0 → 1
Char. 2: 0 → 1
Char. 6: 0 → 1
Char. 8: 0 → 1
Char. 9: 0 → 1
Char. 13: 0 → 1
Char. 19: 0 → 1
Char. 26: 0 → 1
Char. 29: 1 → 0
Char. 33: 0 → 1
Char. 38: 1 → 0
Char. 41: 0 → 1
Char. 50: 1 → 0
Char. 67: 0 → 1
Char. 76: 0 → 1
Char. 84: 1 → 0
Char. 85: 1 → 0
Char. 94: 0 → 1
Char. 107: 0 → 1
Char. 109: 0 → 1
Char. 111: 0 → 1
Char. 115: 0 → 1
Char. 146: 1 → 0
Char. 149: 1 → 0
Char. 164: 0 → 1
Char. 166: 1 → 0
Char. 167: 0 → 1
Char. 176: 0 → 1
Char. 183: 1 → 0
Char. 184: 0 → 1
Char. 192: 1 → 0
Char. 199: 0 → 1
Char. 202: 0 → 1
Char. 204: 0 → 2
Char. 206: 0 → 1
Char. 207: 0 → 1
Char. 209: 0 → 1
Char. 217: 0 → 1
Char. 219: 0 → 1
Char. 220: 0 → 1
Char. 231: 0 → 1
Char. 260: 2 → 0
Char. 272: 2 → 0
Char. 278: 3 → 0

***Microleter mckinzieorum*:**
Char. 0: 0 → 1
Char. 18: 0 → 1
Char. 39: 0 → 1
Char. 57: 1 → 0
Char. 70: 0 → 1
Char. 76: 0 → 1
Char. 94: 0 → 1
Char. 106: 0 → 1
Char. 110: 0 → 1
Char. 278: 3 → 1

**Millerettidae:**
Char. 24: 0 → 1
Char. 25: 1 → 0
Char. 44: 1 → 0
Char. 56: 0 → 1
Char. 57: 1 → 0
Char. 66: 0 → 2
Char. 78: 1 → 0
Char. 80: 1 → 0
Char. 84: 1 → 2
Char. 88: 1 → 0
Char. 96: 1 → 0
Char. 121: 0 → 1
Char. 124: 0 → 1
Char. 127: 0 → 1
Char. 135: 0 → 1
Char. 145: 0 → 1
Char. 166: 1 → 0
Char. 180: 1 → 0
Char. 192: 1 → 0
Char. 202: 0 → 1
Char. 211: 0 → 1
Char. 230: 0 → 1
Char. 234: 0 → 1
Char. 248: 0 → 1
Char. 252: 0 → 1
Char. 253: 0 → 1

***Nycteroleter ineptus*:**
Char. 66: 1 → 0
Char. 278: 0 → 3

***Nyctiphruretus acudens*:**
Char. 0: 0 → 1
Char. 21: 0 → 1
Char. 33: 1 → 2
Char. 41: 0 → 1
Char. 66: 1 → 2
Char. 81: 1 → 0
Char. 84: 1 → 2
Char. 94: 0 → 1
Char. 166: 1 → 0
Char. 224: 0 → 1
Char. 235: 1 → 0
Char. 266: 0 → 1
Char. 272: 2 → 1
Char. 276: 0 → 1

***Orovenator mayorum*:**
Char. 8: 0 → 1
Char. 24: 0 → 1
Char. 33: 1 → 0
Char. 36: 0 → 1
Char. 50: 0 → 1
Char. 92: 1 → 0
Char. 94: 1 → 0
Char. 135: 1 → 0
Char. 159: 1 → 0
Char. 160: 0 → 1
Char. 165: 0 → 1
Char. 278: 0 → 1

***Owenetta* spp.:**
Char. 142: 1 → 0
Char. 169: 1 → 0

***Paleothyris acadiana*:**
Char. 38: 1 → 0
Char. 50: 1 → 0
Char. 66: 1 → 2
Char. 102: 0 → 1
Char. 146: 1 → 0
Char. 237: 0 → 1
Char. 239: 0 → 1

***Placodus* spp.:**
Char. 0: 1 → 2
Char. 9: 0 → 1
Char. 12: 0 → 1
Char. 13: 0 → 1
Char. 19: 0 → 1
Char. 31: 0 → 1
Char. 46: 1 → 0
Char. 57: 0 → 1
Char. 78: 1 → 0
Char. 93: 1 → 0
Char. 102: 1 → 2
Char. 109: 1 → 0
Char. 140: 1 → 0
Char. 155: 0 → 1
Char. 163: 1 → 0
Char. 164: 0 → 1

***Procolophon* spp.:**
Char. 41: 0 → 1
Char. 69: 0 → 1
Char. 79: 0 → 1
Char. 86: 0 → 1
Char. 88: 0 → 1
Char. 101: 0 → 1
Char. 117: 1 → 0
Char. 141: 0 → 1
Char. 149: 1 → 0
Char. 203: 1 → 2
Char. 204: 0 → 1
Char. 215: 0 → 1
Char. 230: 0 → 1
Char. 235: 1 → 2
Char. 237: 0 → 1
Char. 238: 0 → 1
Char. 272: 2 → 1
Char. 278: 3 → 0

***Prolacerta broomi*:**
Char. 58: 1 → 0
Char. 66: 1 → 0
Char. 67: 1 → 0
Char. 80: 1 → 0
Char. 139: 1 → 0
Char. 147: 1 → 0
Char. 192: 1 → 0
Char. 203: 1 → 2
Char. 206: 0 → 12

***Rhipaeosaurus* spp.:**
Char. 172: 0 → 1
Char. 183: 1 → 2
Char. 194: 0 → 1
Char. 201: 1 → 0
Char. 211: 0 → 1
Char. 215: 0 → 1
Char. 226: 0 → 1
Char. 235: 1 → 0
Char. 252: 0 → 1
Char. 277: 0 → 1

**Rhynchocephalia:**
Char. 0: 1 → 2
Char. 24: 0 → 1
Char. 77: 0 → 1
Char. 88: 1 → 0
Char. 94: 1 → 0
Char. 139: 1 → 0
Char. 167: 1 → 0
Char. 205: 1 → 0

**Rhynchosauria:**
Char. 0: 1 → 0
Char. 7: 0 → 1
Char. 9: 0 → 1
Char. 26: 0 → 1
Char. 44: 1 → 0
Char. 68: 0 → 1
Char. 99: 1 → 0
Char. 150: 1 → 0
Char. 160: 0 → 1
Char. 161: 0 → 1
Char. 171: 0 → 2
Char. 223: 0 → 1
Char. 224: 0 → 1
Char. 241: 0 → 1

***Scutosaurus* spp.:**
Char. 175: 0 → 1
Char. 190: 0 → 1
Char. 218: 0 → 1
Char. 243: 0 → 2
Char. 244: 0 → 1
Char. 251: 0 → 1

***Sinosaurosphargis yunguiensis*:**
Char. 8: 0 → 1
Char. 30: 0 → 1
Char. 53: 0 → 1
Char. 89: 1 → 0
Char. 127: 1 → 0
Char. 150: 1 → 0
Char. 154: 0 → 2
Char. 167: 1 → 0
Char. 253: 0 → 1
Char. 255: 0 → 1

**Squamata:**
Char. 45: 0 → 1
Char. 79: 0 → 2
Char. 80: 1 → 0
Char. 92: 1 → 0
Char. 109: 1 → 0
Char. 160: 0 → 1
Char. 200: 0 → 1
Char. 245: 0 → 1

***Trilophosaurus buettneri*:**
Char. 5: 1 → 0
Char. 11: 0 → 1
Char. 55: 1 → 0
Char. 93: 1 → 0
Char. 104: 0 → 1
Char. 106: 0 → 1
Char. 113: 0 → 1
Char. 122: 0 → 1
Char. 136: 1 → 0
Char. 144: 1 → 0
Char. 157: 0 → 1
Char. 159: 1 → 0
Char. 177: 0 → 12
Char. 194: 0 → 1
Char. 203: 1 → 2
Char. 207: 1 → 0
Char. 208: 1 → 0
Char. 272: 1 → 0

***Youngina capensis*:**
Char. 5: 1 → 0
Char. 25: 0 → 1
Char. 27: 0 → 1
Char. 38: 1 → 0
Char. 44: 1 → 0
Char. 56: 0 → 1
Char. 75: 0 → 1
Char. 84: 1 → 0
Char. 92: 1 → 0
Char. 94: 1 → 0
Char. 134: 0 → 1
Char. 163: 1 → 0
Char. 170: 0 → 1
Char. 211: 0 → 1
Char. 215: 0 → 1
Char. 224: 0 → 1
Char. 239: 0 → 1

**Node 50:**
Char. 46: 1 → 0
Char. 88: 1 → 0
Char. 89: 1 → 0
Char. 93: 1 → 0
Char. 176: 0 → 1
Char. 195: 0 → 2

Char. 198: 0 → 1
Char. 246: 1 → 2
Char. 259: 0 → 1
Char. 265: 1 → 0
Char. 270: 0 → 1

**Node 51:**
Char. 65: 0 → 1
Char. 131: 0 → 1
Char. 184: 0 → 1
Char. 205: 0 → 1
Char. 210: 0 → 1
Char. 219: 1 → 0
Char. 241: 0 → 1
Char. 246: 0 → 1
Char. 254: 0 → 1
Char. 255: 0 → 1
Char. 256: 0 → 1
Char. 268: 0 → 1
Char. 269: 0 → 1

**Node 52:**
Char. 0: 1 → 2
Char. 15: 0 → 1
Char. 33: 1 → 2
Char. 44: 1 → 0
Char. 62: 1 → 0
Char. 64: 0 → 1
Char. 84: 1 → 2
Char. 130: 0 → 1
Char. 134: 0 → 2
Char. 147: 0 → 1
Char. 152: 0 → 1
Char. 155: 0 → 1
Char. 158: 0 → 1
Char. 161: 0 → 1
Char. 174: 0 → 1
Char. 181: 0 → 1
Char. 203: 1 → 2
Char. 204: 0 → 2
Char. 247: 0 → 1
Char. 251: 0 → 1
Char. 252: 0 → 1
Char. 253: 0 → 2

**Node 53:**
Char. 0: 0 → 1
Char. 5: 0 → 1
Char. 59: 0 → 1
Char. 60: 0 → 1
Char. 62: 0 → 1
Char. 72: 1 → 2
Char. 92: 0 → 1
Char. 265: 0 → 1
Char. 275: 1 → 0
Char. 278: 3 → 0

**Node 54:**
Char. 20: 0 → 1
Char. 25: 1 → 0
Char. 29: 1 → 0
Char. 33: 0 → 1
Char. 50: 1 → 0
Char. 57: 1 → 0
Char. 67: 0 → 1
Char. 89: 0 → 1
Char. 94: 0 → 1
Char. 127: 0 → 1

**Node 55:**
Char. 132: 0 → 1
Char. 166: 0 → 1
Char. 276: 1 → 0

**Node 56:**
Char. 29: 0 → 1
Char. 84: 0 → 1
Char. 88: 0 → 1
Char. 169: 0 → 1

**Node 57:**
Char. 72: 0 → 1
Char. 81: 0 → 1
Char. 93: 0 → 1
Char. 97: 0 → 1
Char. 104: 1 → 0
Char. 144: 0 → 1
Char. 173: 0 → 1
Char. 183: 0 → 1

**Node 59:**
Char. 95: 0 → 1
Char. 113: 0 → 1
Char. 114: 0 → 1

**Node 60:**
Char. 5: 0 → 1
Char. 20: 0 → 1
Char. 47: 0 → 1
Char. 79: 0 → 1
Char. 110: 0 → 1
Char. 131: 0 → 1
Char. 137: 0 → 1
Char. 140: 0 → 2
Char. 147: 0 → 1

**Node 61:**
Char. 19: 0 → 1
Char. 92: 1 → 0

**Node 62:**
Char. 4: 0 → 1
Char. 15: 0 → 1
Char. 29: 0 → 1
Char. 213: 0 → 1
Char. 226: 0 → 2
Char. 228: 0 → 1
Char. 275: 0 → 1

**Node 63:**
Char. 58: 0 → 1
Char. 61: 0 → 1
Char. 66: 0 → 1
Char. 69: 0 → 1
Char. 107: 0 → 1
Char. 126: 0 → 1
Char. 131: 0 → 1
Char. 134: 0 → 1
Char. 140: 0 → 1
Char. 147: 0 → 1
Char. 150: 0 → 1
Char. 167: 0 → 1
Char. 176: 0 → 1
Char. 190: 0 → 1
Char. 205: 0 → 1
Char. 208: 0 → 1
Char. 230: 0 → 1
Char. 239: 0 → 1
Char. 260: 2 → 1

**Node 64:**
Char. 73: 0 → 1
Char. 131: 1 → 0
Char. 205: 0 → 1
Char. 273: 0 → 1
Char. 276: 0 → 1

**Node 65:**
Char. 18: 0 → 1
Char. 25: 1 → 0
Char. 37: 0 → 1
Char. 103: 0 → 1
Char. 106: 0 → 1
Char. 107: 0 → 1
Char. 110: 0 → 1
Char. 126: 0 → 1
Char. 150: 0 → 1

**Node 66:**
Char. 5: 0 → 1
Char. 20: 0 → 1
Char. 33: 0 → 1
Char. 38: 1 → 2
Char. 39: 0 → 1
Char. 44: 1 → 0
Char. 49: 0 → 1
Char. 51: 0 → 1
Char. 66: 0 → 1
Char. 76: 0 → 1
Char. 80: 1 → 0
Char. 88: 1 → 0
Char. 95: 0 → 1
Char. 131: 0 → 1
Char. 135: 0 → 1
Char. 137: 0 → 1
Char. 147: 0 → 1
Char. 157: 0 → 1
Char. 158: 0 → 1
Char. 183: 1 → 2
Char. 186: 0 → 1
Char. 187: 0 → 1
Char. 194: 0 → 1
Char. 197: 0 → 1
Char. 201: 1 → 0
Char. 211: 0 → 1
Char. 252: 0 → 1

**Node 67:**
Char. 87: 0 → 1
Char. 110: 0 → 1

**Node 68:**
Char. 79: 0 → 1
Char. 133: 0 → 1

**Node 69:**
Char. 100: 0 → 1
Char. 113: 0 → 1

**Node 70:**
Char. 5: 0 → 1
Char. 38: 1 → 2
Char. 39: 0 → 1
Char. 50: 1 → 0
Char. 58: 0 → 1
Char. 59: 0 → 1
Char. 60: 0 → 1
Char. 72: 1 → 2
Char. 85: 1 → 0
Char. 88: 1 → 0
Char. 95: 0 → 1
Char. 104: 0 → 1
Char. 105: 0 → 1
Char. 106: 0 → 2
Char. 107: 0 → 1
Char. 109: 0 → 1
Char. 110: 0 → 1
Char. 146: 1 → 0
Char. 155: 0 → 2
Char. 183: 1 → 2

**Node 71:**
Char. 33: 1 → 0
Char. 38: 2 → 1
Char. 39: 1 → 0
Char. 42: 0 → 1
Char. 43: 0 → 1
Char. 46: 1 → 0
Char. 49: 1 → 0
Char. 71: 1 → 0
Char. 83: 0 → 2
Char. 101: 0 → 1
Char. 103: 0 → 1
Char. 106: 0 → 1
Char. 161: 0 → 1
Char. 163: 1 → 0
Char. 172: 0 → 2
Char. 174: 0 → 1
Char. 188: 0 → 1
Char. 203: 1 → 2
Char. 212: 0 → 1
Char. 216: 1 → 0
Char. 235: 1 → 2
Char. 236: 0 → 1
Char. 238: 0 → 2
Char. 241: 0 → 1
Char. 242: 0 → 1
Char. 273: 0 → 1
Char. 274: 0 → 1
Char. 275: 1 → 0

**Node 72:**
Char. 52: 0 → 1
Char. 75: 0 → 1
Char. 84: 1 → 0
Char. 87: 0 → 1
Char. 110: 0 → 1
Char. 121: 0 → 1
Char. 180: 1 → 0
Char. 230: 0 → 1

**Node 73:**
Char. 29: 1 → 0
Char. 44: 1 → 0
Char. 59: 0 → 1
Char. 60: 0 → 1
Char. 66: 0 → 1
Char. 67: 0 → 1
Char. 72: 1 → 2
Char. 78: 1 → 0
Char. 84: 1 → 0
Char. 88: 1 → 0
Char. 93: 1 → 0
Char. 111: 0 → 1
Char. 123: 1 → 0
Char. 129: 0 → 1
Char. 154: 1 → 0
Char. 169: 1 → 0
Char. 170: 0 → 1
Char. 192: 1 → 0
Char. 197: 0 → 1

**Node 74:**
Char. 85: 1 → 0
Char. 107: 0 → 1

**Node 75:**
Char. 2: 0 → 1
Char. 6: 0 → 1
Char. 186: 0 → 1
Char. 198: 0 → 1
Char. 200: 0 → 1
Char. 220: 0 → 1
Char. 239: 1 → 0

**Node 76:**
Char. 98: 1 → 0
Char. 113: 0 → 1
Char. 181: 0 → 1
Char. 206: 0 → 2

**Node 77:**
Char. 35: 0 → 1
Char. 102: 0 → 1
Char. 103: 0 → 1
Char. 184: 0 → 1
Char. 201: 0 → 1
Char. 210: 0 → 1
Char. 223: 0 → 1
Char. 229: 0 → 1

**Node 78:**
Char. 17: 0 → 1
Char. 42: 0 → 1
Char. 251: 0 → 1
Char. 264: 0 → 1

**Node 79:**
Char. 41: 0 → 1
Char. 61: 1 → 2
Char. 112: 1 → 0
Char. 138: 0 → 1
Char. 146: 1 → 0

Char. 155: 0 → 1
Char. 192: 1 → 2
Char. 224: 0 → 1
Char. 226: 0 → 1

Char. 227: 0 → 1
Char. 233: 0 → 1
Char. 267: 01 → 2

**Node 80:**
Char. 61: 1 → 3
Char. 90: 0 → 1
Char. 91: 0 → 1

Char. 109: 1 → 0
Char. 155: 0 → 1
Char. 209: 1 → 0

**ANALYSIS 52**
**(ALL TAXA, IMPLIED WEIGHTING, K = 4.5)**

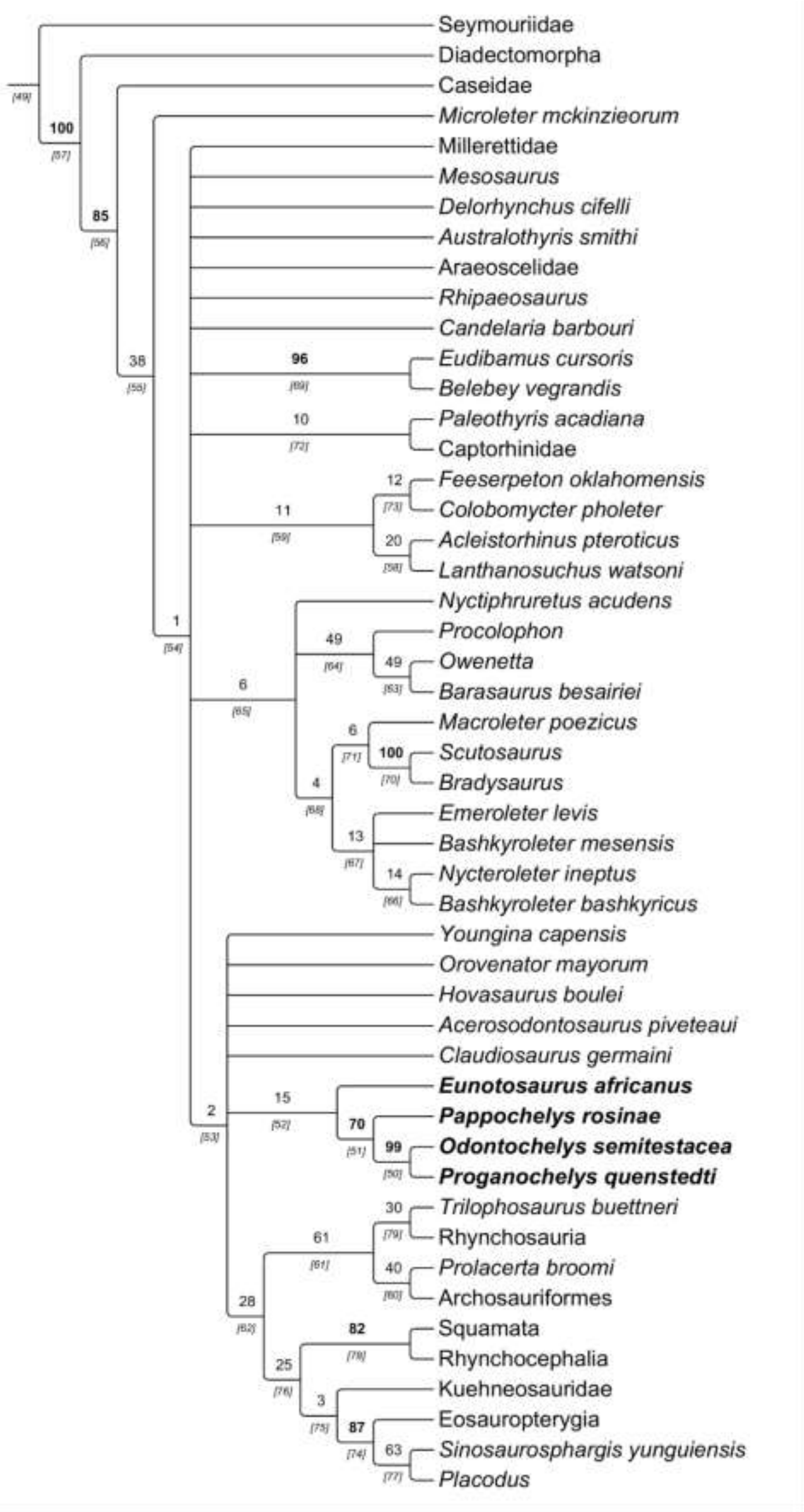

***Proganochelys quenstedti*:**
Char. 8: 0 → 1
Char. 11: 0 → 1
Char. 106: 0 → 1
Char. 108: 1 → 0
Char. 128: 0 → 1
Char. 175: 0 → 1
Char. 202: 1 → 0
Char. 207: 1 → 0
Char. 209: 1 → 0
Char. 244: 0 → 1
Char. 248: 0 → 1
Char. 250: 0 → 1
Char. 252: 1 → 0
Char. 262: 0 → 1

***Pappochelys rosinae*:**
Char. 0: 2 → 0
Char. 1: 0 → 1
Char. 5: 1 → 0
Char. 12: 0 → 1
Char. 41: 0 → 1
Char. 49: 0 → 1
Char. 75: 0 → 1
Char. 169: 1 → 0
Char. 260: 2 → 0

***Eunotosaurus africanus*:**
Char. 19: 0 → 1
Char. 59: 1 → 0
Char. 60: 1 → 0
Char. 72: 2 → 1
Char. 76: 0 → 1
Char. 97: 1 → 0
Char. 103: 0 → 1
Char. 153: 1 → 0
Char. 191: 1 → 0
Char. 192: 1 → 0
Char. 202: 1 → 0
Char. 211: 0 → 1
Char. 222: 1 → 0
Char. 237: 1 → 0
Char. 248: 0 → 1
Char. 249: 0 → 2
Char. 250: 0 → 1
Char. 263: 0 → 1
Char. 273: 0 → 1
Char. 274: 0 → 1
Char. 275: 0 → 1
Char. 276: 0 → 1
Char. 277: 0 → 1
Char. 278: 0 → 3

***Acerosodontosaurus piveteaui*:**
Char. 78: 1 → 0
Char. 81: 1 → 0
Char. 127: 1 → 0
Char. 128: 0 → 1
Char. 155: 0 → 1
Char. 206: 0 → 2
Char. 208: 0 → 1
Char. 278: 0 → 1

***Acleistorhinus pteroticus*:**
Char. 21: 0 → 1
Char. 146: 1 → 0

**Araeoscelidae:**
Char. 0: 0 → 1
Char. 27: 0 → 1
Char. 28: 0 → 1
Char. 29: 1 → 0
Char. 38: 1 → 0
Char. 40: 2 → 0
Char. 43: 0 → 1
Char. 50: 1 → 0
Char. 59: 0 → 1
Char. 60: 0 → 1
Char. 72: 1 → 2
Char. 84: 1 → 0
Char. 89: 0 → 1
Char. 106: 0 → 1
Char. 111: 0 → 1
Char. 116: 1 → 0
Char. 117: 01 → 2
Char. 120: 0 → 1
Char. 154: 1 → 0
Char. 166: 1 → 0
Char. 169: 1 → 0
Char. 170: 0 → 1
Char. 193: 0 → 1
Char. 197: 0 → 1
Char. 222: 0 → 1
Char. 224: 0 → 1
Char. 234: 0 → 1
Char. 237: 0 → 1
Char. 239: 0 → 1
Char. 266: 0 → 1
Char. 278: 3 → 1

**Archosauriformes:**
Char. 32: 0 → 1
Char. 94: 1 → 0
Char. 112: 1 → 0
Char. 152: 0 → 1
Char. 154: 01 → 2
Char. 166: 1 → 0
Char. 171: 0 → 1
Char. 185: 0 → 1
Char. 201: 0 → 1
Char. 204: 0 → 1
Char. 218: 0 → 3
Char. 224: 0 → 1
Char. 242: 0 → 1

***Australothyris smithi*:**
Char. 24: 0 → 1
Char. 34: 0 → 1
Char. 55: 1 → 0
Char. 71: 1 → 0
Char. 79: 0 → 1
Char. 85: 1 → 0
Char. 98: 1 → 0
Char. 100: 0 → 1
Char. 103: 0 → 1
Char. 110: 0 → 1
Char. 123: 1 → 0
Char. 129: 0 → 1
Char. 131: 0 → 1
Char. 132: 1 → 0
Char. 144: 1 → 0
Char. 147: 0 → 1
Char. 149: 1 → 0
Char. 150: 0 → 1
Char. 192: 1 → 0

***Barasaurus besairiei*:**
Char. 33: 1 → 0

***Bashkyroleter bashkyricus*:**
Char. 275: 1 → 0

***Bashkyroleter mesensis*:**
Char. 12: 0 → 1
Char. 66: 1 → 0
Char. 169: 1 → 0

***Belebey vegrandis*:**
Char. 154: 1 → 0

***Bradysaurus* spp.:**
Char. 19: 0 → 1
Char. 25: 1 → 0
Char. 73: 0 → 1
Char. 79: 0 → 1
Char. 135: 1 → 0
Char. 249: 0 → 1

***Candelaria barbouri*:**
Char. 1: 0 → 1
Char. 8: 0 → 1
Char. 15: 0 → 1
Char. 20: 0 → 1
Char. 25: 1 → 0
Char. 29: 1 → 0
Char. 33: 01 → 2
Char. 49: 0 → 1
Char. 50: 1 → 0
Char. 55: 1 → 0
Char. 76: 0 → 1
Char. 79: 0 → 2
Char. 88: 1 → 0
Char. 89: 0 → 1
Char. 94: 0 → 1
Char. 95: 0 → 1
Char. 126: 0 → 1
Char. 127: 0 → 1
Char. 132: 1 → 0
Char. 154: 1 → 2
Char. 169: 1 → 0
Char. 276: 0 → 1
Char. 277: 0 → 1

**Captorhinidae:**
Char. 3: 0 → 1
Char. 25: 1 → 0
Char. 26: 0 → 1
Char. 73: 0 → 1
Char. 75: 0 → 1
Char. 108: 1 → 0
Char. 180: 1 → 0
Char. 183: 1 → 0
Char. 201: 1 → 0
Char. 203: 1 → 2
Char. 216: 1 → 0

**Caseidae:**
Char. 38: 1 → 0
Char. 46: 1 → 0
Char. 50: 1 → 0
Char. 170: 0 → 1
Char. 194: 0 → 1
Char. 273: 0 → 1
Char. 274: 0 → 1
Char. 278: 3 → 2

***Claudiosaurus germaini*:**
Char. 24: 0 → 1
Char. 27: 0 → 1
Char. 34: 0 → 1
Char. 36: 0 → 1
Char. 56: 0 → 1
Char. 64: 0 → 1
Char. 70: 0 → 1
Char. 84: 1 → 0
Char. 105: 0 → 1
Char. 106: 0 → 1
Char. 117: 01 → 2
Char. 126: 0 → 1
Char. 127: 1 → 0
Char. 130: 0 → 1
Char. 131: 0 → 1
Char. 141: 1 → 0
Char. 144: 1 → 0
Char. 166: 1 → 0
Char. 182: 0 → 1
Char. 187: 1 → 0
Char. 190: 0 → 1
Char. 199: 0 → 1
Char. 201: 0 → 1
Char. 203: 1 → 2
Char. 204: 0 → 1
Char. 222: 1 → 0
Char. 234: 1 → 0
Char. 272: 2 → 1

***Colobomycter pholeter*:**
Char. 21: 0 → 1
Char. 25: 1 → 0
Char. 84: 1 → 0
Char. 154: 1 → 0
Char. 167: 0 → 1
Char. 267: 0 → 1

***Delorhynchus cifelli*:**
Char. 5: 0 → 1
Char. 18: 0 → 1
Char. 20: 0 → 1
Char. 21: 0 → 1
Char. 24: 0 → 1
Char. 26: 0 → 1
Char. 28: 0 → 1
Char. 33: 01 → 2
Char. 39: 0 → 1
Char. 52: 0 → 1
Char. 100: 0 → 1
Char. 111: 0 → 1
Char. 116: 1 → 0
Char. 119: 1 → 0
Char. 131: 0 → 1
Char. 147: 0 → 1
Char. 156: 1 → 0
Char. 167: 0 → 1
Char. 189: 0 → 1
Char. 191: 0 → 1
Char. 204: 0 → 2
Char. 267: 0 → 1

**Diadectomorpha:**
Char. 0: 0 → 1
Char. 64: 0 → 1
Char. 70: 0 → 1
Char. 122: 0 → 1
Char. 123: 1 → 0
Char. 146: 1 → 0
Char. 275: 1 → 0
Char. 278: 3 → 0

***Emeroleter levis*:**
Char. 51: 1 → 0

**Eosauropterygia:**
Char. 166: 1 → 0
Char. 174: 0 → 1
Char. 178: 0 → 1
Char. 194: 0 → 2
Char. 272: 1 → 0

***Eudibamus cursoris*:**
Char. 154: 1 → 2

***Feeserpeton oklahomensis*:**
Char. 51: 0 → 1
Char. 157: 0 → 1
Char. 158: 0 → 1

***Hovasaurus boulei*:**
Char. 41: 0 → 1
Char. 55: 1 → 0
Char. 60: 1 → 0
Char. 72: 2 → 1
Char. 77: 0 → 2
Char. 78: 1 → 0
Char. 79: 0 → 1
Char. 93: 1 → 0
Char. 113: 0 → 1
Char. 138: 0 → 1
Char. 141: 1 → 0
Char. 146: 1 → 0
Char. 204: 0 → 2
Char. 206: 0 → 2
Char. 215: 0 → 1
Char. 219: 1 → 0
Char. 224: 0 → 1
Char. 278: 0 → 3

**Kuehneosauridae:**
Char. 7: 0 → 1
Char. 24: 0 → 1
Char. 27: 0 → 1
Char. 43: 1 → 0

Char. 79: 0 → 2
Char. 107: 1 → 0
Char. 140: 1 → 0
Char. 147: 1 → 0
Char. 148: 1 → 0
Char. 245: 0 → 1
Char. 278: 0 → 3

***Lanthanosuchus watsoni*:**
Char. 25: 1 → 0
Char. 51: 0 → 1
Char. 76: 0 → 1
Char. 86: 0 → 1
Char. 98: 1 → 0
Char. 138: 0 → 1
Char. 144: 1 → 0
Char. 154: 1 → 2

***Macroleter poezicus*:**
Char. 9: 0 → 1
Char. 26: 0 → 1
Char. 66: 1 → 2
Char. 126: 0 → 1
Char. 134: 0 → 2
Char. 140: 0 → 1
Char. 146: 1 → 0
Char. 169: 1 → 0

***Mesosaurus* spp.:**
Char. 0: 0 → 1
Char. 2: 0 → 1
Char. 6: 0 → 1
Char. 8: 0 → 1
Char. 9: 0 → 1
Char. 13: 0 → 1
Char. 19: 0 → 1
Char. 26: 0 → 1
Char. 29: 1 → 0
Char. 38: 1 → 0
Char. 41: 0 → 1
Char. 50: 1 → 0
Char. 76: 0 → 1
Char. 84: 1 → 0
Char. 85: 1 → 0
Char. 94: 0 → 1
Char. 107: 0 → 1
Char. 109: 0 → 1
Char. 111: 0 → 1
Char. 115: 0 → 1
Char. 146: 1 → 0
Char. 149: 1 → 0
Char. 164: 0 → 1
Char. 166: 1 → 0
Char. 167: 0 → 1
Char. 176: 0 → 1
Char. 183: 1 → 0
Char. 184: 0 → 1
Char. 192: 1 → 0
Char. 199: 0 → 1
Char. 202: 0 → 1
Char. 204: 0 → 2
Char. 206: 0 → 1
Char. 207: 0 → 1
Char. 209: 0 → 1

Char. 217: 0 → 1
Char. 219: 0 → 1
Char. 220: 0 → 1
Char. 231: 0 → 1
Char. 260: 2 → 0
Char. 272: 2 → 0
Char. 278: 3 → 0

***Microleter mckinzieorum*:**
Char. 0: 0 → 1
Char. 18: 0 → 1
Char. 39: 0 → 1
Char. 70: 0 → 1
Char. 76: 0 → 1
Char. 94: 0 → 1
Char. 106: 0 → 1
Char. 110: 0 → 1
Char. 278: 3 → 1

**Millerettidae:**
Char. 24: 0 → 1
Char. 25: 1 → 0
Char. 44: 1 → 0
Char. 56: 0 → 1
Char. 66: 0 → 2
Char. 78: 1 → 0
Char. 80: 1 → 0
Char. 84: 1 → 2
Char. 88: 1 → 0
Char. 96: 1 → 0
Char. 121: 0 → 1
Char. 124: 0 → 1
Char. 127: 0 → 1
Char. 135: 0 → 1
Char. 145: 0 → 1
Char. 166: 1 → 0
Char. 180: 1 → 0
Char. 192: 1 → 0
Char. 202: 0 → 1
Char. 211: 0 → 1
Char. 230: 0 → 1
Char. 234: 0 → 1
Char. 248: 0 → 1
Char. 252: 0 → 1
Char. 253: 0 → 1

***Nycteroleter ineptus*:**
Char. 66: 1 → 0
Char. 278: 0 → 3

***Nyctiphruretus acudens*:**
Char. 0: 0 → 1
Char. 21: 0 → 1
Char. 33: 1 → 2
Char. 41: 0 → 1
Char. 66: 1 → 2
Char. 81: 1 → 0
Char. 84: 1 → 2
Char. 94: 0 → 1
Char. 166: 1 → 0
Char. 224: 0 → 1
Char. 235: 1 → 0
Char. 266: 0 → 1

Char. 272: 2 → 1
Char. 276: 0 → 1

***Orovenator mayorum*:**
Char. 8: 0 → 1
Char. 24: 0 → 1
Char. 33: 1 → 0
Char. 36: 0 → 1
Char. 50: 0 → 1
Char. 92: 1 → 0
Char. 94: 1 → 0
Char. 135: 1 → 0
Char. 159: 1 → 0
Char. 160: 0 → 1
Char. 165: 0 → 1
Char. 278: 0 → 1

***Owenetta* spp.:**
Char. 142: 1 → 0
Char. 169: 1 → 0

***Paleothyris acadiana*:**
Char. 38: 1 → 0
Char. 50: 1 → 0
Char. 66: 1 → 2
Char. 102: 0 → 1
Char. 146: 1 → 0
Char. 237: 0 → 1
Char. 239: 0 → 1

***Placodus* spp.:**
Char. 0: 1 → 2
Char. 9: 0 → 1
Char. 12: 0 → 1
Char. 13: 0 → 1
Char. 19: 0 → 1
Char. 31: 0 → 1
Char. 46: 1 → 0
Char. 57: 0 → 1
Char. 78: 1 → 0
Char. 93: 1 → 0
Char. 102: 1 → 2
Char. 109: 1 → 0
Char. 140: 1 → 0
Char. 155: 0 → 1
Char. 163: 1 → 0
Char. 164: 0 → 1

***Procolophon* spp.:**
Char. 41: 0 → 1
Char. 69: 0 → 1
Char. 79: 0 → 1
Char. 86: 0 → 1
Char. 88: 0 → 1
Char. 101: 0 → 1
Char. 117: 1 → 0
Char. 141: 0 → 1
Char. 149: 1 → 0
Char. 203: 1 → 2
Char. 204: 0 → 1
Char. 215: 0 → 1
Char. 230: 0 → 1
Char. 235: 1 → 2
Char. 237: 0 → 1
Char. 238: 0 → 1

Char. 272: 2 → 1
Char. 278: 3 → 0

***Prolacerta broomi*:**
Char. 58: 1 → 0
Char. 66: 1 → 0
Char. 67: 1 → 0
Char. 80: 1 → 0
Char. 139: 1 → 0
Char. 147: 1 → 0
Char. 192: 1 → 0
Char. 203: 1 → 2
Char. 206: 0 → 12

***Rhipaeosaurus* spp.:**
Char. 172: 0 → 1
Char. 183: 1 → 2
Char. 194: 0 → 1
Char. 201: 1 → 0
Char. 211: 0 → 1
Char. 215: 0 → 1
Char. 226: 0 → 1
Char. 235: 1 → 0
Char. 252: 0 → 1
Char. 277: 0 → 1

**Rhynchocephalia:**
Char. 0: 1 → 2
Char. 24: 0 → 1
Char. 77: 0 → 1
Char. 88: 1 → 0
Char. 94: 1 → 0
Char. 139: 1 → 0
Char. 167: 1 → 0
Char. 205: 1 → 0

**Rhynchosauria:**
Char. 0: 1 → 0
Char. 7: 0 → 1
Char. 9: 0 → 1
Char. 26: 0 → 1
Char. 44: 1 → 0
Char. 68: 0 → 1
Char. 99: 1 → 0
Char. 150: 1 → 0
Char. 160: 0 → 1
Char. 161: 0 → 1
Char. 171: 0 → 2
Char. 223: 0 → 1
Char. 224: 0 → 1
Char. 241: 0 → 1

***Scutosaurus* spp.:**
Char. 175: 0 → 1
Char. 190: 0 → 1
Char. 218: 0 → 1
Char. 243: 0 → 2
Char. 244: 0 → 1
Char. 251: 0 → 1

***Sinosaurosphargis yunguiensis*:**
Char. 8: 0 → 1
Char. 30: 0 → 1
Char. 53: 0 → 1

Char. 89: 1 → 0
Char. 127: 1 → 0
Char. 150: 1 → 0
Char. 154: 0 → 2
Char. 167: 1 → 0
Char. 253: 0 → 1
Char. 255: 0 → 1

**Squamata:**
Char. 45: 0 → 1
Char. 79: 0 → 2
Char. 80: 1 → 0
Char. 92: 1 → 0
Char. 109: 1 → 0
Char. 160: 0 → 1
Char. 200: 0 → 1
Char. 245: 0 → 1

***Trilophosaurus buettneri*:**
Char. 5: 1 → 0
Char. 11: 0 → 1
Char. 55: 1 → 0
Char. 93: 1 → 0
Char. 104: 0 → 1
Char. 106: 0 → 1
Char. 113: 0 → 1
Char. 122: 0 → 1
Char. 136: 1 → 0
Char. 144: 1 → 0
Char. 157: 0 → 1
Char. 159: 1 → 0
Char. 177: 0 → 12
Char. 194: 0 → 1
Char. 203: 1 → 2
Char. 207: 1 → 0
Char. 208: 1 → 0
Char. 272: 1 → 0

***Youngina capensis*:**
Char. 5: 1 → 0
Char. 25: 0 → 1
Char. 27: 0 → 1
Char. 38: 1 → 0
Char. 44: 1 → 0
Char. 56: 0 → 1
Char. 75: 0 → 1
Char. 84: 1 → 0
Char. 92: 1 → 0
Char. 94: 1 → 0
Char. 134: 0 → 1
Char. 163: 1 → 0
Char. 170: 0 → 1
Char. 211: 0 → 1
Char. 215: 0 → 1
Char. 224: 0 → 1
Char. 239: 0 → 1

**Node 50:**
Char. 46: 1 → 0
Char. 88: 1 → 0
Char. 89: 1 → 0
Char. 93: 1 → 0
Char. 176: 0 → 1
Char. 195: 0 → 2

Char. 198: 0 → 1
Char. 246: 1 → 2
Char. 259: 0 → 1
Char. 265: 1 → 0
Char. 270: 0 → 1

**Node 51:**
Char. 65: 0 → 1
Char. 131: 0 → 1
Char. 184: 0 → 1
Char. 205: 0 → 1
Char. 210: 0 → 1
Char. 219: 1 → 0
Char. 241: 0 → 1
Char. 246: 0 → 1
Char. 254: 0 → 1
Char. 255: 0 → 1
Char. 256: 0 → 1
Char. 268: 0 → 1
Char. 269: 0 → 1

**Node 52:**
Char. 0: 1 → 2
Char. 15: 0 → 1
Char. 33: 1 → 2
Char. 44: 1 → 0
Char. 62: 1 → 0
Char. 64: 0 → 1
Char. 84: 1 → 2
Char. 130: 0 → 1
Char. 134: 0 → 2
Char. 147: 0 → 1
Char. 152: 0 → 1
Char. 155: 0 → 1
Char. 158: 0 → 1
Char. 161: 0 → 1
Char. 174: 0 → 1
Char. 181: 0 → 1
Char. 203: 1 → 2
Char. 204: 0 → 2
Char. 247: 0 → 1
Char. 251: 0 → 1
Char. 252: 0 → 1
Char. 253: 0 → 2

**Node 53:**
Char. 0: 0 → 1
Char. 5: 0 → 1
Char. 20: 0 → 1
Char. 25: 1 → 0
Char. 29: 1 → 0
Char. 40: 2 → 0
Char. 50: 1 → 0
Char. 59: 0 → 1
Char. 60: 0 → 1
Char. 62: 0 → 1
Char. 72: 1 → 2
Char. 89: 0 → 1
Char. 92: 0 → 1
Char. 94: 0 → 1
Char. 109: 0 → 1
Char. 111: 0 → 1
Char. 120: 0 → 1
Char. 127: 0 → 1
Char. 129: 0 → 1
Char. 135: 0 → 1
Char. 136: 0 → 1
Char. 139: 0 → 1
Char. 141: 0 → 1
Char. 179: 0 → 1
Char. 187: 0 → 1
Char. 188: 0 → 1
Char. 191: 0 → 1
Char. 193: 0 → 1
Char. 196: 0 → 1
Char. 201: 1 → 0
Char. 202: 0 → 1
Char. 207: 0 → 1
Char. 209: 0 → 1
Char. 219: 0 → 1
Char. 222: 0 → 1
Char. 234: 0 → 1
Char. 237: 0 → 1
Char. 265: 0 → 1
Char. 266: 0 → 1
Char. 275: 1 → 0
Char. 278: 3 → 0

**Node 54:**
Char. 132: 0 → 1
Char. 166: 0 → 1
Char. 276: 1 → 0

**Node 55:**
Char. 29: 0 → 1
Char. 84: 0 → 1
Char. 88: 0 → 1
Char. 169: 0 → 1

**Node 56:**
Char. 72: 0 → 1
Char. 81: 0 → 1
Char. 93: 0 → 1
Char. 97: 0 → 1
Char. 104: 1 → 0
Char. 144: 0 → 1
Char. 173: 0 → 1
Char. 183: 0 → 1

**Node 58:**
Char. 95: 0 → 1
Char. 113: 0 → 1
Char. 114: 0 → 1

**Node 59:**
Char. 5: 0 → 1
Char. 20: 0 → 1
Char. 47: 0 → 1
Char. 79: 0 → 1
Char. 110: 0 → 1
Char. 131: 0 → 1
Char. 137: 0 → 1
Char. 140: 0 → 2
Char. 147: 0 → 1

**Node 60:**
Char. 19: 0 → 1
Char. 92: 1 → 0

**Node 61:**
Char. 4: 0 → 1
Char. 15: 0 → 1
Char. 29: 0 → 1
Char. 213: 0 → 1
Char. 226: 0 → 2
Char. 228: 0 → 1
Char. 275: 0 → 1

**Node 62:**
Char. 58: 0 → 1
Char. 61: 0 → 1
Char. 66: 0 → 1
Char. 69: 0 → 1
Char. 107: 0 → 1
Char. 126: 0 → 1
Char. 131: 0 → 1
Char. 134: 0 → 1
Char. 140: 0 → 1
Char. 147: 0 → 1
Char. 150: 0 → 1
Char. 167: 0 → 1
Char. 176: 0 → 1
Char. 190: 0 → 1
Char. 205: 0 → 1
Char. 208: 0 → 1
Char. 230: 0 → 1
Char. 239: 0 → 1
Char. 260: 2 → 1

**Node 63:**
Char. 73: 0 → 1
Char. 131: 1 → 0
Char. 205: 0 → 1
Char. 273: 0 → 1
Char. 276: 0 → 1

**Node 64:**
Char. 18: 0 → 1
Char. 25: 1 → 0
Char. 37: 0 → 1
Char. 103: 0 → 1
Char. 106: 0 → 1
Char. 107: 0 → 1
Char. 110: 0 → 1
Char. 126: 0 → 1
Char. 150: 0 → 1

**Node 65:**
Char. 5: 0 → 1
Char. 20: 0 → 1
Char. 38: 1 → 2
Char. 39: 0 → 1
Char. 44: 1 → 0
Char. 49: 0 → 1
Char. 51: 0 → 1
Char. 66: 0 → 1
Char. 76: 0 → 1
Char. 80: 1 → 0
Char. 88: 1 → 0
Char. 95: 0 → 1
Char. 131: 0 → 1
Char. 135: 0 → 1
Char. 137: 0 → 1
Char. 147: 0 → 1
Char. 157: 0 → 1
Char. 158: 0 → 1
Char. 183: 1 → 2
Char. 186: 0 → 1
Char. 187: 0 → 1
Char. 194: 0 → 1
Char. 197: 0 → 1
Char. 201: 1 → 0
Char. 211: 0 → 1
Char. 252: 0 → 1

**Node 66:**
Char. 87: 0 → 1
Char. 110: 0 → 1

**Node 67:**
Char. 79: 0 → 1
Char. 133: 0 → 1

**Node 68:**
Char. 100: 0 → 1
Char. 113: 0 → 1

**Node 69:**
Char. 5: 0 → 1
Char. 38: 1 → 2
Char. 39: 0 → 1
Char. 50: 1 → 0
Char. 58: 0 → 1
Char. 59: 0 → 1
Char. 60: 0 → 1
Char. 72: 1 → 2
Char. 85: 1 → 0
Char. 88: 1 → 0
Char. 95: 0 → 1
Char. 104: 0 → 1
Char. 105: 0 → 1
Char. 106: 0 → 2
Char. 107: 0 → 1
Char. 109: 0 → 1
Char. 110: 0 → 1
Char. 146: 1 → 0
Char. 155: 0 → 2
Char. 183: 1 → 2

**Node 70:**
Char. 33: 1 → 0
Char. 38: 2 → 1
Char. 39: 1 → 0
Char. 42: 0 → 1
Char. 43: 0 → 1
Char. 46: 1 → 0
Char. 49: 1 → 0
Char. 71: 1 → 0
Char. 83: 0 → 2
Char. 101: 0 → 1
Char. 103: 0 → 1
Char. 106: 0 → 1
Char. 161: 0 → 1
Char. 163: 1 → 0
Char. 172: 0 → 2
Char. 174: 0 → 1
Char. 188: 0 → 1
Char. 203: 1 → 2
Char. 212: 0 → 1
Char. 216: 1 → 0
Char. 235: 1 → 2
Char. 236: 0 → 1
Char. 238: 0 → 2
Char. 241: 0 → 1
Char. 242: 0 → 1
Char. 273: 0 → 1
Char. 274: 0 → 1
Char. 275: 1 → 0

**Node 71:**
Char. 52: 0 → 1
Char. 75: 0 → 1
Char. 84: 1 → 0
Char. 87: 0 → 1
Char. 110: 0 → 1
Char. 121: 0 → 1
Char. 180: 1 → 0
Char. 230: 0 → 1

**Node 72:**
Char. 29: 1 → 0
Char. 44: 1 → 0
Char. 59: 0 → 1
Char. 60: 0 → 1
Char. 66: 0 → 1
Char. 72: 1 → 2
Char. 78: 1 → 0
Char. 84: 1 → 0
Char. 88: 1 → 0
Char. 93: 1 → 0
Char. 111: 0 → 1
Char. 123: 1 → 0
Char. 129: 0 → 1
Char. 154: 1 → 0
Char. 169: 1 → 0
Char. 170: 0 → 1
Char. 192: 1 → 0
Char. 197: 0 → 1

**Node 73:**
Char. 85: 1 → 0
Char. 107: 0 → 1

**Node 74:**
Char. 2: 0 → 1
Char. 6: 0 → 1
Char. 186: 0 → 1
Char. 198: 0 → 1
Char. 200: 0 → 1
Char. 220: 0 → 1
Char. 239: 1 → 0

**Node 75:**
Char. 98: 1 → 0
Char. 113: 0 → 1
Char. 181: 0 → 1
Char. 206: 0 → 2

**Node 76:**
Char. 35: 0 → 1
Char. 102: 0 → 1
Char. 103: 0 → 1
Char. 184: 0 → 1

Char. 201: 0 → 1
Char. 210: 0 → 1
Char. 223: 0 → 1
Char. 229: 0 → 1

**Node 77:**
Char. 17: 0 → 1
Char. 42: 0 → 1
Char. 251: 0 → 1
Char. 264: 0 → 1

**Node 78:**
Char. 41: 0 → 1
Char. 61: 1 → 2
Char. 112: 1 → 0
Char. 138: 0 → 1
Char. 146: 1 → 0
Char. 155: 0 → 1
Char. 192: 1 → 2
Char. 224: 0 → 1
Char. 226: 0 → 1
Char. 227: 0 → 1
Char. 233: 0 → 1
Char. 267: 01 → 2

**Node 79:**
Char. 61: 1 → 3
Char. 90: 0 → 1
Char. 91: 0 → 1
Char. 109: 1 → 0
Char. 155: 0 → 1
Char. 209: 1 → 0

**Analysis 53**
**(all taxa, implied weighting, K = 4.625)**

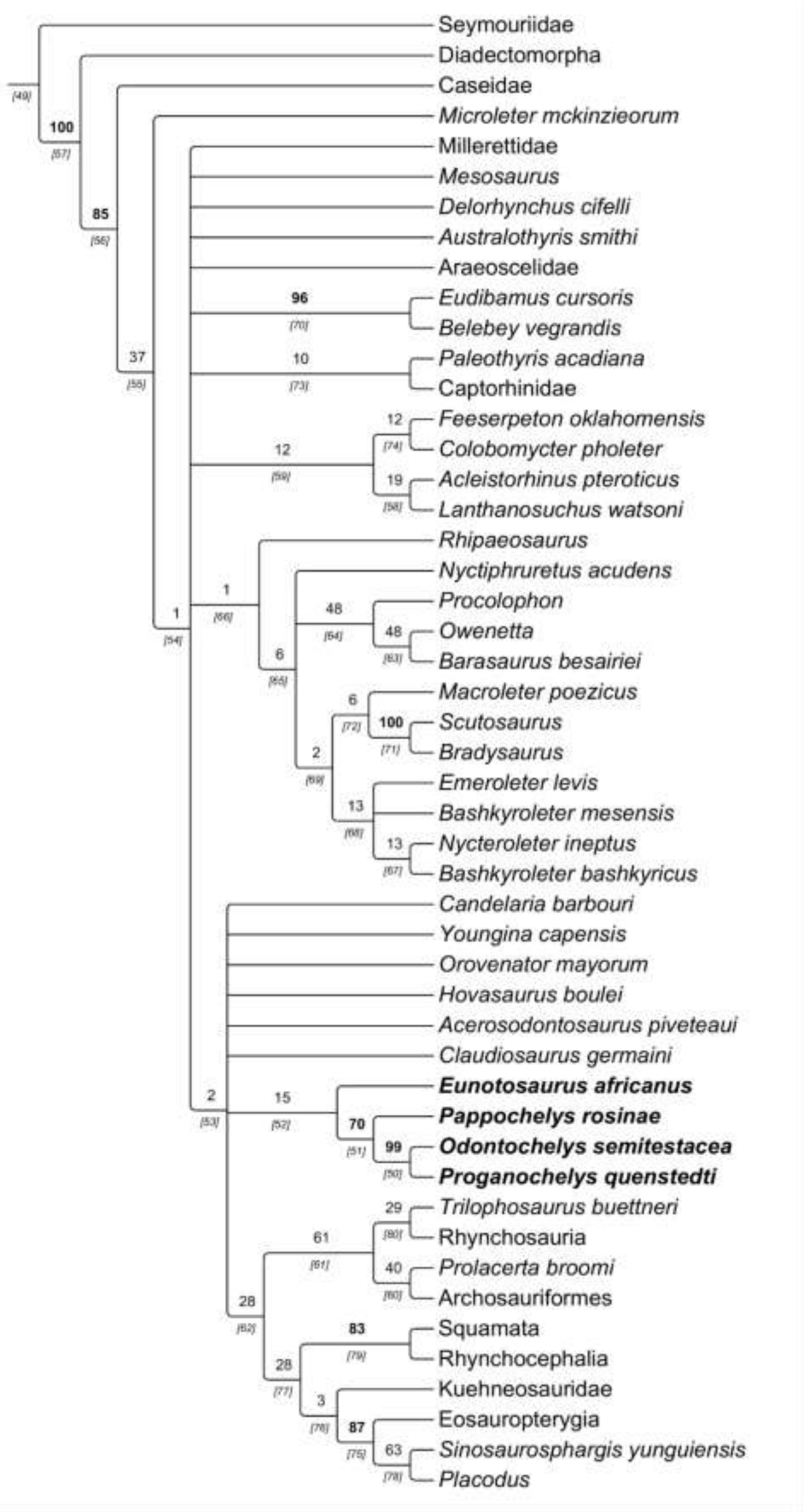

***Proganochelys quenstedti*:**
Char. 8: 0 → 1
Char. 11: 0 → 1
Char. 106: 0 → 1
Char. 108: 1 → 0
Char. 128: 0 → 1
Char. 175: 0 → 1
Char. 202: 1 → 0
Char. 207: 1 → 0
Char. 209: 1 → 0
Char. 244: 0 → 1
Char. 248: 0 → 1
Char. 250: 0 → 1
Char. 252: 1 → 0
Char. 262: 0 → 1

***Pappochelys rosinae*:**
Char. 0: 2 → 0
Char. 1: 0 → 1
Char. 5: 1 → 0
Char. 12: 0 → 1
Char. 41: 0 → 1
Char. 49: 0 → 1
Char. 75: 0 → 1
Char. 169: 1 → 0
Char. 260: 2 → 0

***Odontochelys semitestacea*:**
Char. 43: 0 → 1

***Eunotosaurus africanus*:**
Char. 19: 0 → 1
Char. 43: 0 → 1
Char. 59: 1 → 0
Char. 72: 2 → 1
Char. 76: 0 → 1
Char. 97: 1 → 0
Char. 103: 0 → 1
Char. 153: 1 → 0
Char. 191: 1 → 0
Char. 192: 1 → 0
Char. 202: 1 → 0
Char. 211: 0 → 1
Char. 222: 1 → 0
Char. 237: 1 → 0
Char. 248: 0 → 1
Char. 249: 0 → 2
Char. 250: 0 → 1
Char. 263: 0 → 1
Char. 273: 0 → 1
Char. 274: 0 → 1
Char. 275: 0 → 1
Char. 276: 0 → 1
Char. 277: 0 → 1

***Acerosodontosaurus piveteaui*:**
Char. 78: 1 → 0
Char. 81: 1 → 0
Char. 127: 1 → 0
Char. 128: 0 → 1
Char. 155: 0 → 1
Char. 206: 0 → 2
Char. 208: 0 → 1
Char. 278: 03 → 1

***Acleistorhinus pteroticus*:**
Char. 21: 0 → 1
Char. 146: 1 → 0

**Araeoscelidae:**
Char. 0: 0 → 1
Char. 27: 0 → 1
Char. 28: 0 → 1
Char. 29: 1 → 0
Char. 38: 1 → 0
Char. 40: 2 → 0
Char. 43: 0 → 1
Char. 50: 1 → 0
Char. 57: 1 → 0
Char. 59: 0 → 1
Char. 60: 0 → 1
Char. 67: 0 → 1
Char. 72: 1 → 2
Char. 84: 1 → 0
Char. 89: 0 → 1
Char. 106: 0 → 1
Char. 111: 0 → 1
Char. 116: 1 → 0
Char. 117: 01 → 2
Char. 120: 0 → 1
Char. 154: 1 → 0
Char. 166: 1 → 0
Char. 169: 1 → 0
Char. 170: 0 → 1
Char. 193: 0 → 1
Char. 197: 0 → 1
Char. 222: 0 → 1
Char. 224: 0 → 1
Char. 237: 0 → 1
Char. 239: 0 → 1
Char. 266: 0 → 1
Char. 278: 3 → 1

**Archosauriformes:**
Char. 32: 0 → 1
Char. 94: 1 → 0
Char. 112: 1 → 0
Char. 152: 0 → 1
Char. 154: 01 → 2
Char. 166: 1 → 0
Char. 171: 0 → 1
Char. 185: 0 → 1
Char. 201: 0 → 1
Char. 204: 0 → 1
Char. 218: 0 → 3
Char. 224: 0 → 1
Char. 242: 0 → 1

***Australothyris smithi*:**
Char. 24: 0 → 1
Char. 34: 0 → 1
Char. 55: 1 → 0
Char. 57: 1 → 0
Char. 71: 1 → 0
Char. 79: 0 → 1
Char. 83: 0 → 1
Char. 85: 1 → 0
Char. 98: 1 → 0
Char. 100: 0 → 1
Char. 103: 0 → 1
Char. 110: 0 → 1
Char. 123: 1 → 0
Char. 129: 0 → 1
Char. 131: 0 → 1
Char. 132: 1 → 0
Char. 144: 1 → 0
Char. 147: 0 → 1
Char. 149: 1 → 0
Char. 150: 0 → 1
Char. 192: 1 → 0

***Barasaurus besairiei*:**
Char. 33: 1 → 0

***Bashkyroleter bashkyricus*:**
Char. 275: 1 → 0

***Bashkyroleter mesensis*:**
Char. 12: 0 → 1
Char. 66: 1 → 0
Char. 169: 1 → 0

***Belebey vegrandis*:**
Char. 154: 1 → 0

***Bradysaurus* spp.:**
Char. 19: 0 → 1
Char. 25: 1 → 0
Char. 73: 0 → 1
Char. 79: 0 → 1
Char. 135: 1 → 0
Char. 249: 0 → 1

***Candelaria barbouri*:**
Char. 0: 1 → 0
Char. 1: 0 → 1
Char. 5: 1 → 0
Char. 8: 0 → 1
Char. 15: 0 → 1
Char. 33: 1 → 2
Char. 49: 0 → 1
Char. 55: 1 → 0
Char. 59: 1 → 0
Char. 62: 1 → 0
Char. 72: 2 → 1
Char. 76: 0 → 1
Char. 79: 0 → 2
Char. 83: 0 → 1
Char. 88: 1 → 0
Char. 92: 1 → 0
Char. 95: 0 → 1
Char. 126: 0 → 1
Char. 132: 1 → 0
Char. 154: 1 → 2
Char. 159: 1 → 0
Char. 169: 1 → 0
Char. 265: 1 → 0
Char. 275: 0 → 1
Char. 276: 0 → 1
Char. 277: 0 → 1

**Captorhinidae:**
Char. 3: 0 → 1
Char. 25: 1 → 0
Char. 26: 0 → 1
Char. 73: 0 → 1
Char. 75: 0 → 1
Char. 83: 0 → 1
Char. 108: 1 → 0
Char. 180: 1 → 0
Char. 183: 1 → 0
Char. 201: 1 → 0
Char. 203: 1 → 2
Char. 216: 1 → 0
Char. 240: 1 → 0

**Caseidae:**
Char. 38: 1 → 0
Char. 46: 1 → 0
Char. 50: 1 → 0
Char. 170: 0 → 1
Char. 194: 0 → 1
Char. 273: 0 → 1
Char. 274: 0 → 1
Char. 278: 3 → 2

***Claudiosaurus germaini*:**
Char. 24: 0 → 1
Char. 27: 0 → 1
Char. 34: 0 → 1
Char. 36: 0 → 1
Char. 56: 0 → 1
Char. 64: 0 → 1
Char. 70: 0 → 1
Char. 84: 1 → 0
Char. 105: 0 → 1
Char. 106: 0 → 1
Char. 117: 01 → 2
Char. 126: 0 → 1
Char. 127: 1 → 0
Char. 130: 0 → 1
Char. 131: 0 → 1
Char. 141: 1 → 0
Char. 144: 1 → 0
Char. 148: 0 → 1
Char. 154: 1 → 0
Char. 166: 1 → 0
Char. 182: 0 → 1
Char. 187: 1 → 0
Char. 190: 0 → 1
Char. 199: 0 → 1
Char. 201: 0 → 1
Char. 203: 1 → 2
Char. 204: 0 → 1
Char. 222: 1 → 0
Char. 234: 1 → 0
Char. 267: 0 → 1
Char. 272: 2 → 1

***Colobomycter pholeter*:**
Char. 21: 0 → 1
Char. 25: 1 → 0
Char. 84: 1 → 0
Char. 154: 1 → 0
Char. 167: 0 → 1
Char. 267: 0 → 1

***Delorhynchus cifelli*:**
Char. 18: 0 → 1
Char. 20: 0 → 1
Char. 21: 0 → 1
Char. 24: 0 → 1
Char. 26: 0 → 1
Char. 28: 0 → 1
Char. 33: 0 → 2
Char. 39: 0 → 1
Char. 52: 0 → 1
Char. 100: 0 → 1
Char. 111: 0 → 1
Char. 116: 1 → 0
Char. 119: 1 → 0
Char. 131: 0 → 1
Char. 147: 0 → 1
Char. 156: 1 → 0
Char. 167: 0 → 1
Char. 189: 0 → 1
Char. 191: 0 → 1
Char. 204: 0 → 2
Char. 267: 0 → 1

**Diadectomorpha:**
Char. 0: 0 → 1
Char. 64: 0 → 1
Char. 70: 0 → 1
Char. 122: 0 → 1
Char. 123: 1 → 0
Char. 146: 1 → 0
Char. 275: 1 → 0
Char. 278: 3 → 0

***Emeroleter levis*:**
Char. 51: 1 → 0

**Eosauropterygia:**
Char. 166: 1 → 0
Char. 174: 0 → 1
Char. 178: 0 → 1
Char. 194: 0 → 2
Char. 272: 1 → 0

***Eudibamus cursoris*:**
Char. 154: 1 → 2

***Feeserpeton oklahomensis*:**
Char. 51: 0 → 1
Char. 157: 0 → 1
Char. 158: 0 → 1

***Hovasaurus boulei*:**
Char. 41: 0 → 1
Char. 43: 0 → 1
Char. 55: 1 → 0
Char. 72: 2 → 1
Char. 77: 0 → 2
Char. 78: 1 → 0
Char. 79: 0 → 1
Char. 93: 1 → 0
Char. 113: 0 → 1
Char. 138: 0 → 1

Char. 141: 1 → 0
Char. 146: 1 → 0
Char. 204: 0 → 2
Char. 206: 0 → 2
Char. 215: 0 → 1
Char. 219: 1 → 0
Char. 224: 0 → 1

**Kuehneosauridae:**
Char. 7: 0 → 1
Char. 24: 0 → 1
Char. 27: 0 → 1
Char. 43: 1 → 0
Char. 79: 0 → 2
Char. 107: 1 → 0
Char. 140: 1 → 0
Char. 147: 1 → 0
Char. 148: 1 → 0
Char. 245: 0 → 1
Char. 278: 0 → 3

***Lanthanosuchus watsoni*:**
Char. 25: 1 → 0
Char. 51: 0 → 1
Char. 76: 0 → 1
Char. 86: 0 → 1
Char. 98: 1 → 0
Char. 138: 0 → 1
Char. 144: 1 → 0
Char. 154: 1 → 2

***Macroleter poezicus*:**
Char. 9: 0 → 1
Char. 26: 0 → 1
Char. 66: 1 → 2
Char. 126: 0 → 1
Char. 134: 0 → 2
Char. 140: 0 → 1
Char. 146: 1 → 0
Char. 169: 1 → 0

***Mesosaurus* spp.:**
Char. 0: 0 → 1
Char. 2: 0 → 1
Char. 6: 0 → 1
Char. 8: 0 → 1
Char. 9: 0 → 1
Char. 13: 0 → 1
Char. 19: 0 → 1
Char. 26: 0 → 1
Char. 29: 1 → 0
Char. 33: 0 → 1
Char. 38: 1 → 0
Char. 41: 0 → 1
Char. 50: 1 → 0
Char. 67: 0 → 1
Char. 76: 0 → 1
Char. 83: 0 → 1
Char. 84: 1 → 0
Char. 85: 1 → 0
Char. 94: 0 → 1
Char. 107: 0 → 1
Char. 109: 0 → 1
Char. 111: 0 → 1
Char. 115: 0 → 1
Char. 146: 1 → 0
Char. 148: 0 → 1
Char. 149: 1 → 0
Char. 164: 0 → 1
Char. 166: 1 → 0
Char. 167: 0 → 1
Char. 176: 0 → 1
Char. 183: 1 → 0
Char. 184: 0 → 1
Char. 192: 1 → 0
Char. 199: 0 → 1
Char. 202: 0 → 1
Char. 204: 0 → 2
Char. 206: 0 → 1
Char. 207: 0 → 1
Char. 209: 0 → 1
Char. 217: 0 → 1
Char. 219: 0 → 1
Char. 220: 0 → 1
Char. 231: 0 → 1
Char. 240: 1 → 0
Char. 260: 2 → 0
Char. 272: 2 → 0
Char. 278: 3 → 0

***Microleter mckinzieorum*:**
Char. 0: 0 → 1
Char. 18: 0 → 1
Char. 39: 0 → 1
Char. 57: 1 → 0
Char. 70: 0 → 1
Char. 76: 0 → 1
Char. 83: 0 → 1
Char. 94: 0 → 1
Char. 106: 0 → 1
Char. 110: 0 → 1
Char. 278: 3 → 1

**Millerettidae:**
Char. 24: 0 → 1
Char. 25: 1 → 0
Char. 44: 1 → 0
Char. 56: 0 → 1
Char. 57: 1 → 0
Char. 66: 0 → 2
Char. 78: 1 → 0
Char. 80: 1 → 0
Char. 84: 1 → 2
Char. 88: 1 → 0
Char. 96: 1 → 0
Char. 121: 0 → 1
Char. 124: 0 → 1
Char. 127: 0 → 1
Char. 135: 0 → 1
Char. 145: 0 → 1
Char. 166: 1 → 0
Char. 180: 1 → 0
Char. 192: 1 → 0
Char. 202: 0 → 1
Char. 211: 0 → 1
Char. 230: 0 → 1
Char. 248: 0 → 1
Char. 252: 0 → 1
Char. 253: 0 → 1

***Nycteroleter ineptus*:**
Char. 66: 1 → 0
Char. 278: 0 → 3

***Nyctiphruretus acudens*:**
Char. 0: 0 → 1
Char. 21: 0 → 1
Char. 33: 1 → 2
Char. 41: 0 → 1
Char. 66: 1 → 2
Char. 81: 1 → 0
Char. 83: 0 → 1
Char. 84: 1 → 2
Char. 94: 0 → 1
Char. 166: 1 → 0
Char. 224: 0 → 1
Char. 226: 1 → 0
Char. 266: 0 → 1
Char. 272: 2 → 1
Char. 276: 0 → 1

***Orovenator mayorum*:**
Char. 8: 0 → 1
Char. 24: 0 → 1
Char. 33: 1 → 0
Char. 36: 0 → 1
Char. 50: 0 → 1
Char. 92: 1 → 0
Char. 94: 1 → 0
Char. 135: 1 → 0
Char. 159: 1 → 0
Char. 160: 0 → 1
Char. 165: 0 → 1
Char. 278: 03 → 1

***Owenetta* spp.:**
Char. 142: 1 → 0
Char. 169: 1 → 0

***Paleothyris acadiana*:**
Char. 38: 1 → 0
Char. 50: 1 → 0
Char. 66: 1 → 2
Char. 102: 0 → 1
Char. 146: 1 → 0
Char. 237: 0 → 1
Char. 239: 0 → 1

***Placodus* spp.:**
Char. 0: 1 → 2
Char. 9: 0 → 1
Char. 12: 0 → 1
Char. 13: 0 → 1
Char. 19: 0 → 1
Char. 31: 0 → 1
Char. 46: 1 → 0
Char. 57: 0 → 1
Char. 78: 1 → 0
Char. 93: 1 → 0
Char. 102: 1 → 2
Char. 109: 1 → 0
Char. 140: 1 → 0
Char. 155: 0 → 1
Char. 163: 1 → 0
Char. 164: 0 → 1

***Procolophon* spp.:**
Char. 41: 0 → 1
Char. 69: 0 → 1
Char. 79: 0 → 1
Char. 83: 0 → 1
Char. 86: 0 → 1
Char. 88: 0 → 1
Char. 101: 0 → 1
Char. 117: 1 → 0
Char. 141: 0 → 1
Char. 149: 1 → 0
Char. 203: 1 → 2
Char. 204: 0 → 1
Char. 230: 0 → 1
Char. 237: 0 → 1
Char. 238: 0 → 1
Char. 272: 2 → 1
Char. 278: 3 → 0

***Prolacerta broomi*:**
Char. 58: 1 → 0
Char. 66: 1 → 0
Char. 67: 1 → 0
Char. 80: 1 → 0
Char. 139: 1 → 0
Char. 147: 1 → 0
Char. 192: 1 → 0
Char. 203: 1 → 2
Char. 206: 0 → 12

***Rhipaeosaurus* spp.:**
Char. 172: 0 → 1
Char. 277: 0 → 1

**Rhynchocephalia:**
Char. 0: 1 → 2
Char. 24: 0 → 1
Char. 77: 0 → 1
Char. 88: 1 → 0
Char. 94: 1 → 0
Char. 139: 1 → 0
Char. 167: 1 → 0
Char. 205: 1 → 0

**Rhynchosauria:**
Char. 0: 1 → 0
Char. 7: 0 → 1
Char. 9: 0 → 1
Char. 26: 0 → 1
Char. 44: 1 → 0
Char. 68: 0 → 1
Char. 99: 1 → 0
Char. 150: 1 → 0
Char. 160: 0 → 1
Char. 161: 0 → 1
Char. 171: 0 → 2
Char. 223: 0 → 1
Char. 224: 0 → 1
Char. 241: 0 → 1

***Scutosaurus* spp.:**
Char. 175: 0 → 1
Char. 190: 0 → 1
Char. 218: 0 → 1
Char. 243: 0 → 2
Char. 244: 0 → 1
Char. 251: 0 → 1

***Sinosaurosphargis yunguiensis*:**
Char. 8: 0 → 1
Char. 30: 0 → 1
Char. 53: 0 → 1
Char. 89: 1 → 0
Char. 127: 1 → 0
Char. 150: 1 → 0
Char. 154: 0 → 2
Char. 167: 1 → 0
Char. 253: 0 → 1
Char. 255: 0 → 1

**Squamata:**
Char. 45: 0 → 1
Char. 79: 0 → 2
Char. 80: 1 → 0
Char. 92: 1 → 0
Char. 109: 1 → 0
Char. 160: 0 → 1
Char. 200: 0 → 1
Char. 245: 0 → 1

***Trilophosaurus buettneri*:**
Char. 5: 1 → 0
Char. 11: 0 → 1
Char. 55: 1 → 0
Char. 93: 1 → 0
Char. 104: 0 → 1
Char. 106: 0 → 1
Char. 113: 0 → 1
Char. 122: 0 → 1
Char. 136: 1 → 0
Char. 144: 1 → 0
Char. 157: 0 → 1
Char. 159: 1 → 0
Char. 177: 0 → 12
Char. 194: 0 → 1
Char. 203: 1 → 2
Char. 207: 1 → 0
Char. 208: 1 → 0
Char. 272: 1 → 0

***Youngina capensis*:**
Char. 5: 1 → 0
Char. 21: 0 → 1
Char. 25: 0 → 1
Char. 27: 0 → 1
Char. 38: 1 → 0
Char. 43: 0 → 1
Char. 44: 1 → 0
Char. 56: 0 → 1
Char. 75: 0 → 1
Char. 84: 1 → 0
Char. 92: 1 → 0
Char. 94: 1 → 0

Char. 134: 0 → 1
Char. 154: 1 → 0
Char. 163: 1 → 0
Char. 170: 0 → 1
Char. 211: 0 → 1
Char. 215: 0 → 1
Char. 224: 0 → 1
Char. 239: 0 → 1
Char. 267: 0 → 1

**Node 50:**
Char. 46: 1 → 0
Char. 88: 1 → 0
Char. 89: 1 → 0
Char. 93: 1 → 0
Char. 176: 0 → 1
Char. 195: 0 → 2
Char. 198: 0 → 1
Char. 246: 1 → 2
Char. 259: 0 → 1
Char. 265: 1 → 0
Char. 270: 0 → 1

**Node 51:**
Char. 65: 0 → 1
Char. 131: 0 → 1
Char. 184: 0 → 1
Char. 205: 0 → 1
Char. 210: 0 → 1
Char. 219: 1 → 0
Char. 241: 0 → 1
Char. 246: 0 → 1
Char. 254: 0 → 1
Char. 255: 0 → 1
Char. 256: 0 → 1
Char. 267: 0 → 1
Char. 268: 0 → 1
Char. 269: 0 → 1

**Node 52:**
Char. 0: 1 → 2
Char. 15: 0 → 1
Char. 21: 0 → 1
Char. 33: 1 → 2
Char. 44: 1 → 0
Char. 62: 1 → 0
Char. 64: 0 → 1
Char. 84: 1 → 2
Char. 130: 0 → 1
Char. 134: 0 → 2
Char. 147: 0 → 1
Char. 148: 0 → 1
Char. 152: 0 → 1
Char. 155: 0 → 1
Char. 158: 0 → 1
Char. 161: 0 → 1
Char. 174: 0 → 1
Char. 181: 0 → 1
Char. 203: 1 → 2
Char. 204: 0 → 2
Char. 247: 0 → 1
Char. 251: 0 → 1
Char. 252: 0 → 1
Char. 253: 0 → 2

**Node 53:**
Char. 0: 0 → 1
Char. 20: 0 → 1
Char. 25: 1 → 0
Char. 29: 1 → 0
Char. 33: 0 → 1
Char. 40: 2 → 0
Char. 50: 1 → 0
Char. 57: 1 → 0
Char. 59: 0 → 1
Char. 62: 0 → 1
Char. 67: 0 → 1
Char. 72: 1 → 2
Char. 89: 0 → 1
Char. 94: 0 → 1
Char. 109: 0 → 1
Char. 111: 0 → 1
Char. 120: 0 → 1
Char. 127: 0 → 1
Char. 129: 0 → 1
Char. 135: 0 → 1
Char. 136: 0 → 1
Char. 139: 0 → 1
Char. 141: 0 → 1
Char. 179: 0 → 1
Char. 187: 0 → 1
Char. 188: 0 → 1
Char. 191: 0 → 1
Char. 193: 0 → 1
Char. 196: 0 → 1
Char. 201: 1 → 0
Char. 202: 0 → 1
Char. 207: 0 → 1
Char. 209: 0 → 1
Char. 219: 0 → 1
Char. 222: 0 → 1
Char. 237: 0 → 1
Char. 265: 0 → 1
Char. 266: 0 → 1
Char. 275: 1 → 0

**Node 54:**
Char. 132: 0 → 1
Char. 166: 0 → 1
Char. 276: 1 → 0

**Node 55:**
Char. 29: 0 → 1
Char. 84: 0 → 1
Char. 88: 0 → 1
Char. 169: 0 → 1

**Node 56:**
Char. 72: 0 → 1
Char. 81: 0 → 1
Char. 93: 0 → 1
Char. 97: 0 → 1
Char. 104: 1 → 0
Char. 144: 0 → 1
Char. 173: 0 → 1
Char. 183: 0 → 1

**Node 58:**
Char. 95: 0 → 1
Char. 113: 0 → 1
Char. 114: 0 → 1

**Node 59:**
Char. 20: 0 → 1
Char. 47: 0 → 1
Char. 79: 0 → 1
Char. 110: 0 → 1
Char. 131: 0 → 1
Char. 137: 0 → 1
Char. 140: 0 → 2
Char. 147: 0 → 1

**Node 60:**
Char. 19: 0 → 1
Char. 92: 1 → 0

**Node 61:**
Char. 4: 0 → 1
Char. 15: 0 → 1
Char. 29: 0 → 1
Char. 213: 0 → 1
Char. 226: 0 → 2
Char. 228: 0 → 1
Char. 275: 0 → 1

**Node 62:**
Char. 43: 0 → 1
Char. 58: 0 → 1
Char. 61: 0 → 1
Char. 66: 0 → 1
Char. 69: 0 → 1
Char. 107: 0 → 1
Char. 126: 0 → 1
Char. 131: 0 → 1
Char. 134: 0 → 1
Char. 140: 0 → 1
Char. 147: 0 → 1
Char. 148: 0 → 1
Char. 150: 0 → 1
Char. 167: 0 → 1
Char. 176: 0 → 1
Char. 190: 0 → 1
Char. 205: 0 → 1
Char. 208: 0 → 1
Char. 230: 0 → 1
Char. 239: 0 → 1
Char. 260: 2 → 1

**Node 63:**
Char. 73: 0 → 1
Char. 131: 1 → 0
Char. 205: 0 → 1
Char. 273: 0 → 1
Char. 276: 0 → 1

**Node 64:**
Char. 18: 0 → 1
Char. 25: 1 → 0
Char. 37: 0 → 1
Char. 103: 0 → 1
Char. 106: 0 → 1
Char. 107: 0 → 1
Char. 110: 0 → 1
Char. 126: 0 → 1
Char. 150: 0 → 1

**Node 65:**
Char. 186: 0 → 1

**Node 66:**
Char. 148: 0 → 1
Char. 183: 1 → 2
Char. 194: 0 → 1
Char. 201: 1 → 0
Char. 211: 0 → 1
Char. 226: 0 → 1
Char. 240: 1 → 0
Char. 252: 0 → 1

**Node 67:**
Char. 87: 0 → 1
Char. 110: 0 → 1

**Node 68:**
Char. 79: 0 → 1
Char. 133: 0 → 1

**Node 69:**
Char. 100: 0 → 1
Char. 113: 0 → 1

**Node 70:**
Char. 38: 1 → 2
Char. 39: 0 → 1
Char. 50: 1 → 0
Char. 58: 0 → 1
Char. 59: 0 → 1
Char. 60: 0 → 1
Char. 72: 1 → 2
Char. 83: 0 → 1
Char. 85: 1 → 0
Char. 88: 1 → 0
Char. 95: 0 → 1
Char. 104: 0 → 1
Char. 105: 0 → 1
Char. 106: 0 → 2
Char. 107: 0 → 1
Char. 109: 0 → 1
Char. 110: 0 → 1
Char. 146: 1 → 0
Char. 148: 0 → 1
Char. 155: 0 → 2
Char. 183: 1 → 2

**Node 71:**
Char. 33: 1 → 0
Char. 38: 2 → 1
Char. 39: 1 → 0
Char. 42: 0 → 1
Char. 43: 0 → 1
Char. 46: 1 → 0
Char. 49: 1 → 0
Char. 71: 1 → 0
Char. 83: 0 → 2
Char. 101: 0 → 1
Char. 103: 0 → 1
Char. 106: 0 → 1
Char. 161: 0 → 1
Char. 163: 1 → 0
Char. 172: 0 → 2
Char. 174: 0 → 1
Char. 188: 0 → 1
Char. 203: 1 → 2
Char. 212: 0 → 1
Char. 216: 1 → 0
Char. 236: 0 → 1
Char. 238: 0 → 2
Char. 241: 0 → 1
Char. 242: 0 → 1
Char. 273: 0 → 1
Char. 274: 0 → 1
Char. 275: 1 → 0

**Node 72:**
Char. 52: 0 → 1
Char. 75: 0 → 1
Char. 84: 1 → 0
Char. 87: 0 → 1
Char. 110: 0 → 1
Char. 121: 0 → 1
Char. 180: 1 → 0
Char. 230: 0 → 1

**Node 73:**
Char. 29: 1 → 0
Char. 44: 1 → 0
Char. 59: 0 → 1
Char. 60: 0 → 1
Char. 66: 0 → 1
Char. 67: 0 → 1
Char. 72: 1 → 2
Char. 78: 1 → 0
Char. 84: 1 → 0
Char. 88: 1 → 0
Char. 93: 1 → 0
Char. 111: 0 → 1
Char. 123: 1 → 0
Char. 129: 0 → 1
Char. 154: 1 → 0
Char. 169: 1 → 0
Char. 170: 0 → 1
Char. 192: 1 → 0
Char. 197: 0 → 1

**Node 74:**
Char. 83: 0 → 1
Char. 85: 1 → 0
Char. 107: 0 → 1

**Node 75:**
Char. 2: 0 → 1
Char. 6: 0 → 1
Char. 186: 0 → 1
Char. 198: 0 → 1
Char. 200: 0 → 1
Char. 220: 0 → 1
Char. 239: 1 → 0

**Node 76:**
Char. 98: 1 → 0
Char. 113: 0 → 1
Char. 181: 0 → 1
Char. 206: 0 → 2

**Node 77:**
Char. 35: 0 → 1
Char. 102: 0 → 1
Char. 103: 0 → 1
Char. 184: 0 → 1
Char. 201: 0 → 1
Char. 210: 0 → 1
Char. 223: 0 → 1
Char. 229: 0 → 1

**Node 78:**
Char. 17: 0 → 1
Char. 42: 0 → 1
Char. 251: 0 → 1
Char. 264: 0 → 1

**Node 79:**
Char. 41: 0 → 1
Char. 61: 1 → 2
Char. 112: 1 → 0
Char. 138: 0 → 1
Char. 146: 1 → 0
Char. 155: 0 → 1
Char. 192: 1 → 2
Char. 224: 0 → 1
Char. 226: 0 → 1
Char. 227: 0 → 1
Char. 233: 0 → 1
Char. 267: 01 → 2

**Node 80:**
Char. 61: 1 → 3
Char. 90: 0 → 1
Char. 91: 0 → 1
Char. 109: 1 → 0
Char. 155: 0 → 1
Char. 209: 1 →

**ANALYSIS 54**
**(ALL TAXA, IMPLIED WEIGHTING, K = 4.75)**

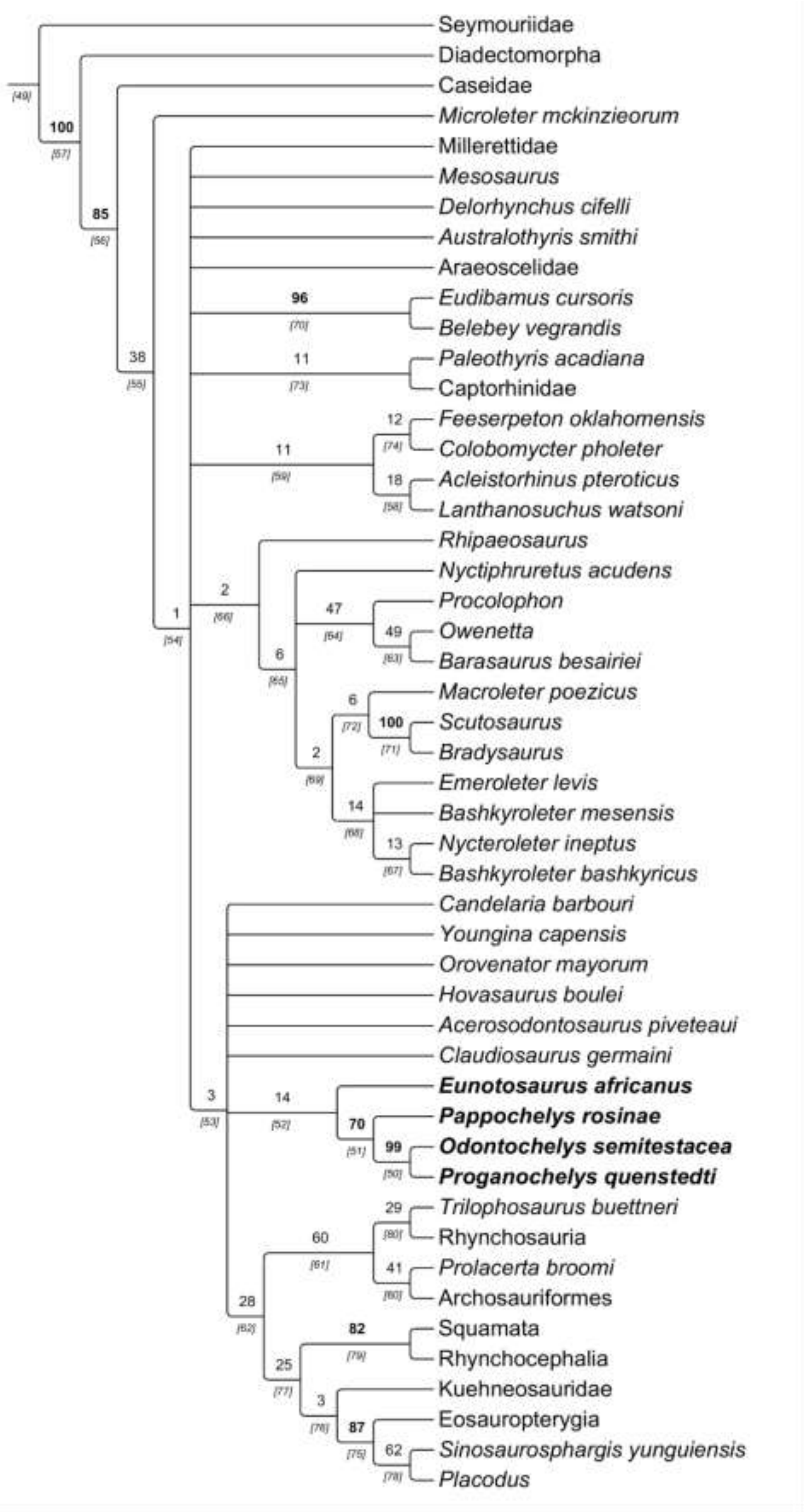

***Proganochelys quenstedti*:**
Char. 8: 0 → 1
Char. 11: 0 → 1
Char. 106: 0 → 1
Char. 108: 1 → 0
Char. 128: 0 → 1
Char. 175: 0 → 1
Char. 202: 1 → 0
Char. 207: 1 → 0
Char. 209: 1 → 0
Char. 244: 0 → 1
Char. 248: 0 → 1
Char. 250: 0 → 1
Char. 252: 1 → 0
Char. 262: 0 → 1

***Pappochelys rosinae*:**
Char. 0: 2 → 0
Char. 1: 0 → 1
Char. 5: 1 → 0
Char. 12: 0 → 1
Char. 41: 0 → 1
Char. 49: 0 → 1
Char. 75: 0 → 1
Char. 169: 1 → 0
Char. 260: 2 → 0

***Odontochelys semitestacea*:**
Char. 43: 0 → 1

***Eunotosaurus africanus*:**
Char. 19: 0 → 1
Char. 43: 0 → 1
Char. 59: 1 → 0
Char. 72: 2 → 1
Char. 76: 0 → 1
Char. 97: 1 → 0
Char. 103: 0 → 1
Char. 153: 1 → 0
Char. 191: 1 → 0
Char. 192: 1 → 0
Char. 202: 1 → 0
Char. 211: 0 → 1
Char. 222: 1 → 0
Char. 237: 1 → 0
Char. 248: 0 → 1
Char. 249: 0 → 2
Char. 250: 0 → 1
Char. 263: 0 → 1
Char. 273: 0 → 1
Char. 274: 0 → 1
Char. 275: 0 → 1
Char. 276: 0 → 1
Char. 277: 0 → 1

***Acerosodontosaurus piveteaui*:**
Char. 78: 1 → 0
Char. 81: 1 → 0
Char. 127: 1 → 0
Char. 128: 0 → 1
Char. 155: 0 → 1
Char. 206: 0 → 2
Char. 208: 0 → 1
Char. 278: 03 → 1

***Acleistorhinus pteroticus*:**
Char. 21: 0 → 1
Char. 146: 1 → 0

**Araeoscelidae:**
Char. 0: 0 → 1
Char. 27: 0 → 1
Char. 28: 0 → 1
Char. 29: 1 → 0
Char. 38: 1 → 0
Char. 40: 2 → 0
Char. 43: 0 → 1
Char. 50: 1 → 0
Char. 57: 1 → 0
Char. 59: 0 → 1
Char. 60: 0 → 1
Char. 67: 0 → 1
Char. 72: 1 → 2
Char. 84: 1 → 0
Char. 89: 0 → 1
Char. 106: 0 → 1
Char. 111: 0 → 1
Char. 116: 1 → 0
Char. 117: 01 → 2
Char. 120: 0 → 1
Char. 154: 1 → 0
Char. 166: 1 → 0
Char. 169: 1 → 0
Char. 170: 0 → 1
Char. 193: 0 → 1
Char. 197: 0 → 1
Char. 222: 0 → 1
Char. 224: 0 → 1
Char. 237: 0 → 1
Char. 239: 0 → 1
Char. 266: 0 → 1
Char. 278: 3 → 1

**Archosauriformes:**
Char. 32: 0 → 1
Char. 94: 1 → 0
Char. 112: 1 → 0
Char. 152: 0 → 1
Char. 154: 01 → 2
Char. 166: 1 → 0
Char. 171: 0 → 1
Char. 185: 0 → 1
Char. 201: 0 → 1
Char. 204: 0 → 1
Char. 218: 0 → 3
Char. 224: 0 → 1
Char. 242: 0 → 1

***Australothyris smithi*:**
Char. 24: 0 → 1
Char. 34: 0 → 1
Char. 55: 1 → 0
Char. 57: 1 → 0
Char. 71: 1 → 0
Char. 79: 0 → 1
Char. 83: 0 → 1
Char. 85: 1 → 0
Char. 98: 1 → 0
Char. 100: 0 → 1
Char. 103: 0 → 1
Char. 110: 0 → 1
Char. 123: 1 → 0
Char. 129: 0 → 1
Char. 131: 0 → 1
Char. 132: 1 → 0
Char. 144: 1 → 0
Char. 147: 0 → 1
Char. 149: 1 → 0
Char. 150: 0 → 1
Char. 192: 1 → 0

***Barasaurus besairiei*:**
Char. 33: 1 → 0

***Bashkyroleter bashkyricus*:**
Char. 275: 1 → 0

***Bashkyroleter mesensis*:**
Char. 12: 0 → 1
Char. 66: 1 → 0
Char. 169: 1 → 0

***Belebey vegrandis*:**
Char. 154: 1 → 0

***Bradysaurus* spp.:**
Char. 19: 0 → 1
Char. 25: 1 → 0
Char. 73: 0 → 1
Char. 79: 0 → 1
Char. 135: 1 → 0
Char. 249: 0 → 1

***Candelaria barbouri*:**
Char. 0: 1 → 0
Char. 1: 0 → 1
Char. 5: 1 → 0
Char. 8: 0 → 1
Char. 15: 0 → 1
Char. 33: 1 → 2
Char. 49: 0 → 1
Char. 55: 1 → 0
Char. 59: 1 → 0
Char. 62: 1 → 0
Char. 72: 2 → 1
Char. 76: 0 → 1
Char. 79: 0 → 2
Char. 83: 0 → 1
Char. 88: 1 → 0
Char. 92: 1 → 0
Char. 95: 0 → 1
Char. 126: 0 → 1
Char. 132: 1 → 0
Char. 154: 1 → 2
Char. 159: 1 → 0
Char. 169: 1 → 0
Char. 265: 1 → 0
Char. 275: 0 → 1
Char. 276: 0 → 1
Char. 277: 0 → 1

**Captorhinidae:**
Char. 3: 0 → 1
Char. 25: 1 → 0
Char. 26: 0 → 1
Char. 73: 0 → 1
Char. 75: 0 → 1
Char. 83: 0 → 1
Char. 108: 1 → 0
Char. 180: 1 → 0
Char. 183: 1 → 0
Char. 201: 1 → 0
Char. 203: 1 → 2
Char. 216: 1 → 0
Char. 240: 1 → 0

**Caseidae:**
Char. 38: 1 → 0
Char. 46: 1 → 0
Char. 50: 1 → 0
Char. 170: 0 → 1
Char. 194: 0 → 1
Char. 273: 0 → 1
Char. 274: 0 → 1
Char. 278: 3 → 2

***Claudiosaurus germaini*:**
Char. 24: 0 → 1
Char. 27: 0 → 1
Char. 34: 0 → 1
Char. 36: 0 → 1
Char. 56: 0 → 1
Char. 64: 0 → 1
Char. 70: 0 → 1
Char. 84: 1 → 0
Char. 105: 0 → 1
Char. 106: 0 → 1
Char. 117: 01 → 2
Char. 126: 0 → 1
Char. 127: 1 → 0
Char. 130: 0 → 1
Char. 131: 0 → 1
Char. 141: 1 → 0
Char. 144: 1 → 0
Char. 148: 0 → 1
Char. 154: 1 → 0
Char. 166: 1 → 0
Char. 182: 0 → 1
Char. 187: 1 → 0
Char. 190: 0 → 1
Char. 199: 0 → 1
Char. 201: 0 → 1
Char. 203: 1 → 2
Char. 204: 0 → 1
Char. 222: 1 → 0
Char. 234: 1 → 0
Char. 267: 0 → 1
Char. 272: 2 → 1

***Colobomycter pholeter*:**
Char. 21: 0 → 1
Char. 25: 1 → 0
Char. 84: 1 → 0
Char. 154: 1 → 0
Char. 167: 0 → 1
Char. 267: 0 → 1

***Delorhynchus cifelli*:**
Char. 18: 0 → 1
Char. 20: 0 → 1
Char. 21: 0 → 1
Char. 24: 0 → 1
Char. 26: 0 → 1
Char. 28: 0 → 1
Char. 33: 0 → 2
Char. 39: 0 → 1
Char. 52: 0 → 1
Char. 100: 0 → 1
Char. 111: 0 → 1
Char. 116: 1 → 0
Char. 119: 1 → 0
Char. 131: 0 → 1
Char. 147: 0 → 1
Char. 156: 1 → 0
Char. 167: 0 → 1
Char. 189: 0 → 1
Char. 191: 0 → 1
Char. 204: 0 → 2
Char. 267: 0 → 1

**Diadectomorpha:**
Char. 0: 0 → 1
Char. 64: 0 → 1
Char. 70: 0 → 1
Char. 122: 0 → 1
Char. 123: 1 → 0
Char. 146: 1 → 0
Char. 275: 1 → 0
Char. 278: 3 → 0

***Emeroleter levis*:**
Char. 51: 1 → 0

**Eosauropterygia:**
Char. 166: 1 → 0
Char. 174: 0 → 1
Char. 178: 0 → 1
Char. 194: 0 → 2
Char. 272: 1 → 0

***Eudibamus cursoris*:**
Char. 154: 1 → 2

***Feeserpeton oklahomensis*:**
Char. 51: 0 → 1
Char. 157: 0 → 1
Char. 158: 0 → 1

***Hovasaurus boulei*:**
Char. 41: 0 → 1
Char. 43: 0 → 1
Char. 55: 1 → 0
Char. 72: 2 → 1
Char. 77: 0 → 2
Char. 78: 1 → 0
Char. 79: 0 → 1
Char. 93: 1 → 0
Char. 113: 0 → 1
Char. 138: 0 → 1

Char. 141: 1 → 0
Char. 146: 1 → 0
Char. 204: 0 → 2
Char. 206: 0 → 2
Char. 215: 0 → 1
Char. 219: 1 → 0
Char. 224: 0 → 1

**Kuehneosauridae:**
Char. 7: 0 → 1
Char. 24: 0 → 1
Char. 27: 0 → 1
Char. 43: 1 → 0
Char. 79: 0 → 2
Char. 107: 1 → 0
Char. 140: 1 → 0
Char. 147: 1 → 0
Char. 148: 1 → 0
Char. 245: 0 → 1
Char. 278: 0 → 3

***Lanthanosuchus watsoni*:**
Char. 25: 1 → 0
Char. 51: 0 → 1
Char. 76: 0 → 1
Char. 86: 0 → 1
Char. 98: 1 → 0
Char. 138: 0 → 1
Char. 144: 1 → 0
Char. 154: 1 → 2

***Macroleter poezicus*:**
Char. 9: 0 → 1
Char. 26: 0 → 1
Char. 66: 1 → 2
Char. 126: 0 → 1
Char. 134: 0 → 2
Char. 140: 0 → 1
Char. 146: 1 → 0
Char. 169: 1 → 0

***Mesosaurus* spp.:**
Char. 0: 0 → 1
Char. 2: 0 → 1
Char. 6: 0 → 1
Char. 8: 0 → 1
Char. 9: 0 → 1
Char. 13: 0 → 1
Char. 19: 0 → 1
Char. 26: 0 → 1
Char. 29: 1 → 0
Char. 33: 0 → 1
Char. 38: 1 → 0
Char. 41: 0 → 1
Char. 50: 1 → 0
Char. 67: 0 → 1
Char. 76: 0 → 1
Char. 83: 0 → 1
Char. 84: 1 → 0
Char. 85: 1 → 0
Char. 94: 0 → 1
Char. 107: 0 → 1
Char. 109: 0 → 1
Char. 111: 0 → 1
Char. 115: 0 → 1
Char. 146: 1 → 0
Char. 148: 0 → 1
Char. 149: 1 → 0
Char. 164: 0 → 1
Char. 166: 1 → 0
Char. 167: 0 → 1
Char. 176: 0 → 1
Char. 183: 1 → 0
Char. 184: 0 → 1
Char. 192: 1 → 0
Char. 199: 0 → 1
Char. 202: 0 → 1
Char. 204: 0 → 2
Char. 206: 0 → 1
Char. 207: 0 → 1
Char. 209: 0 → 1
Char. 217: 0 → 1
Char. 219: 0 → 1
Char. 220: 0 → 1
Char. 231: 0 → 1
Char. 240: 1 → 0
Char. 260: 2 → 0
Char. 272: 2 → 0
Char. 278: 3 → 0

***Microleter mckinzieorum*:**
Char. 0: 0 → 1
Char. 18: 0 → 1
Char. 39: 0 → 1
Char. 57: 1 → 0
Char. 70: 0 → 1
Char. 76: 0 → 1
Char. 83: 0 → 1
Char. 94: 0 → 1
Char. 106: 0 → 1
Char. 110: 0 → 1
Char. 278: 3 → 1

**Millerettidae:**
Char. 24: 0 → 1
Char. 25: 1 → 0
Char. 44: 1 → 0
Char. 56: 0 → 1
Char. 57: 1 → 0
Char. 66: 0 → 2
Char. 78: 1 → 0
Char. 80: 1 → 0
Char. 84: 1 → 2
Char. 88: 1 → 0
Char. 96: 1 → 0
Char. 121: 0 → 1
Char. 124: 0 → 1
Char. 127: 0 → 1
Char. 135: 0 → 1
Char. 145: 0 → 1
Char. 166: 1 → 0
Char. 180: 1 → 0
Char. 192: 1 → 0
Char. 202: 0 → 1
Char. 211: 0 → 1
Char. 230: 0 → 1
Char. 248: 0 → 1
Char. 252: 0 → 1
Char. 253: 0 → 1

***Nycteroleter ineptus*:**
Char. 66: 1 → 0
Char. 278: 0 → 3

***Nyctiphruretus acudens*:**
Char. 0: 0 → 1
Char. 21: 0 → 1
Char. 33: 1 → 2
Char. 41: 0 → 1
Char. 66: 1 → 2
Char. 81: 1 → 0
Char. 83: 0 → 1
Char. 84: 1 → 2
Char. 94: 0 → 1
Char. 166: 1 → 0
Char. 224: 0 → 1
Char. 226: 1 → 0
Char. 266: 0 → 1
Char. 272: 2 → 1
Char. 276: 0 → 1

***Orovenator mayorum*:**
Char. 8: 0 → 1
Char. 24: 0 → 1
Char. 33: 1 → 0
Char. 36: 0 → 1
Char. 50: 0 → 1
Char. 92: 1 → 0
Char. 94: 1 → 0
Char. 135: 1 → 0
Char. 159: 1 → 0
Char. 160: 0 → 1
Char. 165: 0 → 1
Char. 278: 03 → 1

***Owenetta* spp.:**
Char. 142: 1 → 0
Char. 169: 1 → 0

***Paleothyris acadiana*:**
Char. 38: 1 → 0
Char. 50: 1 → 0
Char. 66: 1 → 2
Char. 102: 0 → 1
Char. 146: 1 → 0
Char. 237: 0 → 1
Char. 239: 0 → 1

***Placodus* spp.:**
Char. 0: 1 → 2
Char. 9: 0 → 1
Char. 12: 0 → 1
Char. 13: 0 → 1
Char. 19: 0 → 1
Char. 31: 0 → 1
Char. 46: 1 → 0
Char. 57: 0 → 1
Char. 78: 1 → 0
Char. 93: 1 → 0
Char. 102: 1 → 2
Char. 109: 1 → 0
Char. 140: 1 → 0
Char. 155: 0 → 1
Char. 163: 1 → 0
Char. 164: 0 → 1

***Procolophon* spp.:**
Char. 41: 0 → 1
Char. 69: 0 → 1
Char. 79: 0 → 1
Char. 83: 0 → 1
Char. 86: 0 → 1
Char. 88: 0 → 1
Char. 101: 0 → 1
Char. 117: 1 → 0
Char. 141: 0 → 1
Char. 149: 1 → 0
Char. 203: 1 → 2
Char. 204: 0 → 1
Char. 230: 0 → 1
Char. 237: 0 → 1
Char. 238: 0 → 1
Char. 272: 2 → 1
Char. 278: 3 → 0

***Prolacerta broomi*:**
Char. 58: 1 → 0
Char. 66: 1 → 0
Char. 67: 1 → 0
Char. 80: 1 → 0
Char. 139: 1 → 0
Char. 147: 1 → 0
Char. 192: 1 → 0
Char. 203: 1 → 2
Char. 206: 0 → 12

***Rhipaeosaurus* spp.:**
Char. 172: 0 → 1
Char. 277: 0 → 1

**Rhynchocephalia:**
Char. 0: 1 → 2
Char. 24: 0 → 1
Char. 77: 0 → 1
Char. 88: 1 → 0
Char. 94: 1 → 0
Char. 139: 1 → 0
Char. 167: 1 → 0
Char. 205: 1 → 0

**Rhynchosauria:**
Char. 0: 1 → 0
Char. 7: 0 → 1
Char. 9: 0 → 1
Char. 26: 0 → 1
Char. 44: 1 → 0
Char. 68: 0 → 1
Char. 99: 1 → 0
Char. 150: 1 → 0
Char. 160: 0 → 1
Char. 161: 0 → 1
Char. 171: 0 → 2
Char. 223: 0 → 1
Char. 224: 0 → 1
Char. 241: 0 → 1

***Scutosaurus* spp.:**
Char. 175: 0 → 1
Char. 190: 0 → 1
Char. 218: 0 → 1
Char. 243: 0 → 2
Char. 244: 0 → 1
Char. 251: 0 → 1

***Sinosaurosphargis yunguiensis*:**
Char. 8: 0 → 1
Char. 30: 0 → 1
Char. 53: 0 → 1
Char. 89: 1 → 0
Char. 127: 1 → 0
Char. 150: 1 → 0
Char. 154: 0 → 2
Char. 167: 1 → 0
Char. 253: 0 → 1
Char. 255: 0 → 1

**Squamata:**
Char. 45: 0 → 1
Char. 79: 0 → 2
Char. 80: 1 → 0
Char. 92: 1 → 0
Char. 109: 1 → 0
Char. 160: 0 → 1
Char. 200: 0 → 1
Char. 245: 0 → 1

***Trilophosaurus buettneri*:**
Char. 5: 1 → 0
Char. 11: 0 → 1
Char. 55: 1 → 0
Char. 93: 1 → 0
Char. 104: 0 → 1
Char. 106: 0 → 1
Char. 113: 0 → 1
Char. 122: 0 → 1
Char. 136: 1 → 0
Char. 144: 1 → 0
Char. 157: 0 → 1
Char. 159: 1 → 0
Char. 177: 0 → 12
Char. 194: 0 → 1
Char. 203: 1 → 2
Char. 207: 1 → 0
Char. 208: 1 → 0
Char. 272: 1 → 0

***Youngina capensis*:**
Char. 5: 1 → 0
Char. 21: 0 → 1
Char. 25: 0 → 1
Char. 27: 0 → 1
Char. 38: 1 → 0
Char. 43: 0 → 1
Char. 44: 1 → 0
Char. 56: 0 → 1
Char. 75: 0 → 1
Char. 84: 1 → 0
Char. 92: 1 → 0
Char. 94: 1 → 0

Char. 134: 0 → 1
Char. 154: 1 → 0
Char. 163: 1 → 0
Char. 170: 0 → 1
Char. 211: 0 → 1
Char. 215: 0 → 1
Char. 224: 0 → 1
Char. 239: 0 → 1
Char. 267: 0 → 1

**Node 50:**
Char. 46: 1 → 0
Char. 88: 1 → 0
Char. 89: 1 → 0
Char. 93: 1 → 0
Char. 176: 0 → 1
Char. 195: 0 → 2
Char. 198: 0 → 1
Char. 246: 1 → 2
Char. 259: 0 → 1
Char. 265: 1 → 0
Char. 270: 0 → 1

**Node 51:**
Char. 65: 0 → 1
Char. 131: 0 → 1
Char. 184: 0 → 1
Char. 205: 0 → 1
Char. 210: 0 → 1
Char. 219: 1 → 0
Char. 241: 0 → 1
Char. 246: 0 → 1
Char. 254: 0 → 1
Char. 255: 0 → 1
Char. 256: 0 → 1
Char. 267: 0 → 1
Char. 268: 0 → 1
Char. 269: 0 → 1

**Node 52:**
Char. 0: 1 → 2
Char. 15: 0 → 1
Char. 21: 0 → 1
Char. 33: 1 → 2
Char. 44: 1 → 0
Char. 62: 1 → 0
Char. 64: 0 → 1
Char. 84: 1 → 2
Char. 130: 0 → 1
Char. 134: 0 → 2
Char. 147: 0 → 1
Char. 148: 0 → 1
Char. 152: 0 → 1
Char. 155: 0 → 1
Char. 158: 0 → 1
Char. 161: 0 → 1
Char. 174: 0 → 1
Char. 181: 0 → 1
Char. 203: 1 → 2
Char. 204: 0 → 2
Char. 247: 0 → 1
Char. 251: 0 → 1
Char. 252: 0 → 1
Char. 253: 0 → 2

**Node 53:**
Char. 0: 0 → 1
Char. 20: 0 → 1
Char. 25: 1 → 0
Char. 29: 1 → 0
Char. 33: 0 → 1
Char. 40: 2 → 0
Char. 50: 1 → 0
Char. 57: 1 → 0
Char. 59: 0 → 1
Char. 62: 0 → 1
Char. 67: 0 → 1
Char. 72: 1 → 2
Char. 89: 0 → 1
Char. 94: 0 → 1
Char. 109: 0 → 1
Char. 111: 0 → 1
Char. 120: 0 → 1
Char. 127: 0 → 1
Char. 129: 0 → 1
Char. 135: 0 → 1
Char. 136: 0 → 1
Char. 139: 0 → 1
Char. 141: 0 → 1
Char. 179: 0 → 1
Char. 187: 0 → 1
Char. 188: 0 → 1
Char. 191: 0 → 1
Char. 193: 0 → 1
Char. 196: 0 → 1
Char. 201: 1 → 0
Char. 202: 0 → 1
Char. 207: 0 → 1
Char. 209: 0 → 1
Char. 219: 0 → 1
Char. 222: 0 → 1
Char. 237: 0 → 1
Char. 265: 0 → 1
Char. 266: 0 → 1
Char. 275: 1 → 0

**Node 54:**
Char. 132: 0 → 1
Char. 166: 0 → 1
Char. 276: 1 → 0

**Node 55:**
Char. 29: 0 → 1
Char. 84: 0 → 1
Char. 88: 0 → 1
Char. 169: 0 → 1

**Node 56:**
Char. 72: 0 → 1
Char. 81: 0 → 1
Char. 93: 0 → 1
Char. 97: 0 → 1
Char. 104: 1 → 0
Char. 144: 0 → 1
Char. 173: 0 → 1
Char. 183: 0 → 1

**Node 58:**
Char. 95: 0 → 1
Char. 113: 0 → 1
Char. 114: 0 → 1

**Node 59:**
Char. 20: 0 → 1
Char. 47: 0 → 1
Char. 79: 0 → 1
Char. 110: 0 → 1
Char. 131: 0 → 1
Char. 137: 0 → 1
Char. 140: 0 → 2
Char. 147: 0 → 1

**Node 60:**
Char. 19: 0 → 1
Char. 92: 1 → 0

**Node 61:**
Char. 4: 0 → 1
Char. 15: 0 → 1
Char. 29: 0 → 1
Char. 213: 0 → 1
Char. 226: 0 → 2
Char. 228: 0 → 1
Char. 275: 0 → 1

**Node 62:**
Char. 43: 0 → 1
Char. 58: 0 → 1
Char. 61: 0 → 1
Char. 66: 0 → 1
Char. 69: 0 → 1
Char. 107: 0 → 1
Char. 126: 0 → 1
Char. 131: 0 → 1
Char. 134: 0 → 1
Char. 140: 0 → 1
Char. 147: 0 → 1
Char. 148: 0 → 1
Char. 150: 0 → 1
Char. 167: 0 → 1
Char. 176: 0 → 1
Char. 190: 0 → 1
Char. 205: 0 → 1
Char. 208: 0 → 1
Char. 230: 0 → 1
Char. 239: 0 → 1
Char. 260: 2 → 1

**Node 63:**
Char. 73: 0 → 1
Char. 131: 1 → 0
Char. 205: 0 → 1
Char. 273: 0 → 1
Char. 276: 0 → 1

**Node 64:**
Char. 18: 0 → 1
Char. 25: 1 → 0
Char. 37: 0 → 1
Char. 103: 0 → 1
Char. 106: 0 → 1
Char. 107: 0 → 1
Char. 110: 0 → 1
Char. 126: 0 → 1
Char. 150: 0 → 1

**Node 65:**
Char. 186: 0 → 1

**Node 66:**
Char. 148: 0 → 1
Char. 183: 1 → 2
Char. 194: 0 → 1
Char. 201: 1 → 0
Char. 211: 0 → 1
Char. 226: 0 → 1
Char. 240: 1 → 0
Char. 252: 0 → 1

**Node 67:**
Char. 87: 0 → 1
Char. 110: 0 → 1

**Node 68:**
Char. 79: 0 → 1
Char. 133: 0 → 1

**Node 69:**
Char. 100: 0 → 1
Char. 113: 0 → 1

**Node 70:**
Char. 38: 1 → 2
Char. 39: 0 → 1
Char. 50: 1 → 0
Char. 58: 0 → 1
Char. 59: 0 → 1
Char. 60: 0 → 1
Char. 72: 1 → 2
Char. 83: 0 → 1
Char. 85: 1 → 0
Char. 88: 1 → 0
Char. 95: 0 → 1
Char. 104: 0 → 1
Char. 105: 0 → 1
Char. 106: 0 → 2
Char. 107: 0 → 1
Char. 109: 0 → 1
Char. 110: 0 → 1
Char. 146: 1 → 0
Char. 148: 0 → 1
Char. 155: 0 → 2
Char. 183: 1 → 2

**Node 71:**
Char. 33: 1 → 0
Char. 38: 2 → 1
Char. 39: 1 → 0
Char. 42: 0 → 1
Char. 43: 0 → 1
Char. 46: 1 → 0
Char. 49: 1 → 0
Char. 71: 1 → 0
Char. 83: 0 → 2
Char. 101: 0 → 1
Char. 103: 0 → 1
Char. 106: 0 → 1
Char. 161: 0 → 1
Char. 163: 1 → 0
Char. 172: 0 → 2
Char. 174: 0 → 1
Char. 188: 0 → 1
Char. 203: 1 → 2
Char. 212: 0 → 1
Char. 216: 1 → 0
Char. 236: 0 → 1
Char. 238: 0 → 2
Char. 241: 0 → 1
Char. 242: 0 → 1
Char. 273: 0 → 1
Char. 274: 0 → 1
Char. 275: 1 → 0

**Node 72:**
Char. 52: 0 → 1
Char. 75: 0 → 1
Char. 84: 1 → 0
Char. 87: 0 → 1
Char. 110: 0 → 1
Char. 121: 0 → 1
Char. 180: 1 → 0
Char. 230: 0 → 1

**Node 73:**
Char. 29: 1 → 0
Char. 44: 1 → 0
Char. 59: 0 → 1
Char. 60: 0 → 1
Char. 66: 0 → 1
Char. 67: 0 → 1
Char. 72: 1 → 2
Char. 78: 1 → 0
Char. 84: 1 → 0
Char. 88: 1 → 0
Char. 93: 1 → 0
Char. 111: 0 → 1
Char. 123: 1 → 0
Char. 129: 0 → 1
Char. 154: 1 → 0
Char. 169: 1 → 0
Char. 170: 0 → 1
Char. 192: 1 → 0
Char. 197: 0 → 1

**Node 74:**
Char. 83: 0 → 1
Char. 85: 1 → 0
Char. 107: 0 → 1

**Node 75:**
Char. 2: 0 → 1
Char. 6: 0 → 1
Char. 186: 0 → 1
Char. 198: 0 → 1
Char. 200: 0 → 1
Char. 220: 0 → 1
Char. 239: 1 → 0

**Node 76:**
Char. 98: 1 → 0
Char. 113: 0 → 1
Char. 181: 0 → 1
Char. 206: 0 → 2

**Node 77:**
Char. 35: 0 → 1
Char. 102: 0 → 1
Char. 103: 0 → 1
Char. 184: 0 → 1
Char. 201: 0 → 1
Char. 210: 0 → 1
Char. 223: 0 → 1
Char. 229: 0 → 1

**Node 78:**
Char. 17: 0 → 1
Char. 42: 0 → 1
Char. 251: 0 → 1
Char. 264: 0 → 1

**Node 79:**
Char. 41: 0 → 1
Char. 61: 1 → 2
Char. 112: 1 → 0
Char. 138: 0 → 1
Char. 146: 1 → 0
Char. 155: 0 → 1
Char. 192: 1 → 2
Char. 224: 0 → 1
Char. 226: 0 → 1
Char. 227: 0 → 1
Char. 233: 0 → 1
Char. 267: 01 → 2

**Node 80:**
Char. 61: 1 → 3
Char. 90: 0 → 1
Char. 91: 0 → 1
Char. 109: 1 → 0
Char. 155: 0 → 1
Char. 209: 1 → 0

**Analysis 55**
**(all taxa, implied weighting, K = 4.875)**

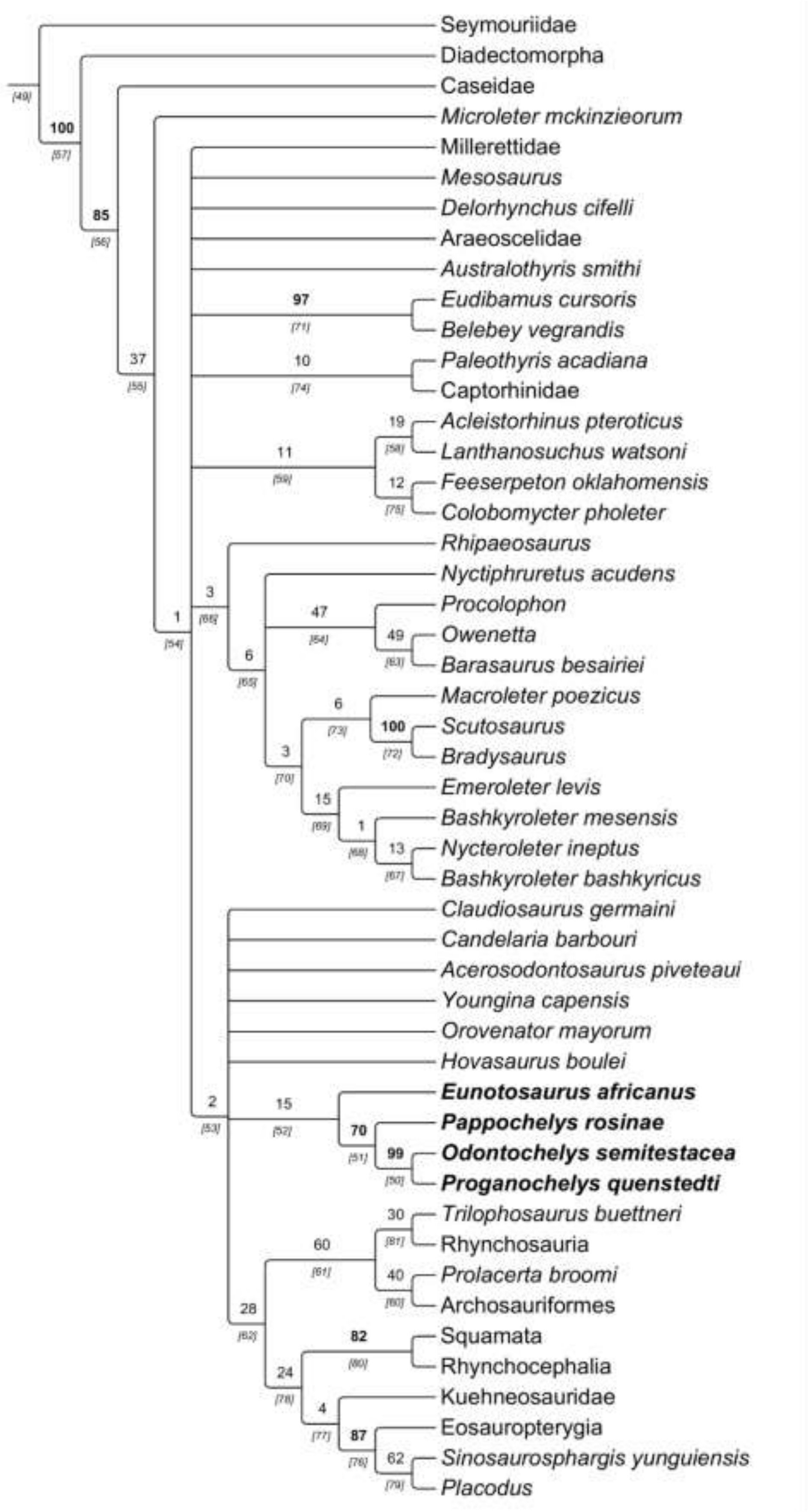

***Proganochelys quenstedti*:**
Char. 8: 0 → 1
Char. 11: 0 → 1
Char. 106: 0 → 1
Char. 108: 1 → 0
Char. 128: 0 → 1
Char. 175: 0 → 1
Char. 202: 1 → 0
Char. 207: 1 → 0
Char. 209: 1 → 0
Char. 244: 0 → 1
Char. 248: 0 → 1
Char. 250: 0 → 1
Char. 252: 1 → 0
Char. 262: 0 → 1

***Pappochelys rosinae*:**
Char. 0: 2 → 0
Char. 1: 0 → 1
Char. 5: 1 → 0
Char. 12: 0 → 1
Char. 41: 0 → 1
Char. 49: 0 → 1
Char. 75: 0 → 1
Char. 169: 1 → 0
Char. 260: 2 → 0

***Odontochelys semitestacea*:**
Char. 43: 0 → 1

***Eunotosaurus africanus*:**
Char. 19: 0 → 1
Char. 43: 0 → 1
Char. 59: 1 → 0
Char. 72: 2 → 1
Char. 76: 0 → 1
Char. 97: 1 → 0
Char. 103: 0 → 1
Char. 153: 1 → 0
Char. 191: 1 → 0
Char. 192: 1 → 0
Char. 202: 1 → 0
Char. 211: 0 → 1
Char. 222: 1 → 0
Char. 237: 1 → 0
Char. 248: 0 → 1
Char. 249: 0 → 2
Char. 250: 0 → 1
Char. 263: 0 → 1
Char. 273: 0 → 1
Char. 274: 0 → 1
Char. 275: 0 → 1
Char. 276: 0 → 1
Char. 277: 0 → 1

***Acerosodontosaurus piveteaui*:**
Char. 78: 1 → 0
Char. 81: 1 → 0
Char. 127: 1 → 0
Char. 128: 0 → 1
Char. 155: 0 → 1
Char. 206: 0 → 2
Char. 208: 0 → 1
Char. 278: 03 → 1

***Acleistorhinus pteroticus*:**
Char. 21: 0 → 1
Char. 146: 1 → 0

**Araeoscelidae:**
Char. 0: 0 → 1
Char. 27: 0 → 1
Char. 28: 0 → 1
Char. 29: 1 → 0
Char. 38: 1 → 0
Char. 40: 2 → 0
Char. 43: 0 → 1
Char. 50: 1 → 0
Char. 57: 1 → 0
Char. 59: 0 → 1
Char. 60: 0 → 1
Char. 67: 0 → 1
Char. 72: 1 → 2
Char. 84: 1 → 0
Char. 89: 0 → 1
Char. 106: 0 → 1
Char. 111: 0 → 1
Char. 116: 1 → 0
Char. 117: 01 → 2
Char. 120: 0 → 1
Char. 154: 1 → 0
Char. 166: 1 → 0
Char. 169: 1 → 0
Char. 170: 0 → 1
Char. 193: 0 → 1
Char. 197: 0 → 1
Char. 222: 0 → 1
Char. 224: 0 → 1
Char. 237: 0 → 1
Char. 239: 0 → 1
Char. 266: 0 → 1
Char. 278: 3 → 1

**Archosauriformes:**
Char. 32: 0 → 1
Char. 94: 1 → 0
Char. 112: 1 → 0
Char. 152: 0 → 1
Char. 154: 01 → 2
Char. 166: 1 → 0
Char. 171: 0 → 1
Char. 185: 0 → 1
Char. 201: 0 → 1
Char. 204: 0 → 1
Char. 218: 0 → 3
Char. 224: 0 → 1
Char. 242: 0 → 1

***Australothyris smithi*:**
Char. 24: 0 → 1
Char. 34: 0 → 1
Char. 55: 1 → 0
Char. 57: 1 → 0
Char. 71: 1 → 0
Char. 79: 0 → 1
Char. 83: 0 → 1
Char. 85: 1 → 0
Char. 98: 1 → 0
Char. 100: 0 → 1
Char. 103: 0 → 1
Char. 110: 0 → 1
Char. 123: 1 → 0
Char. 129: 0 → 1
Char. 131: 0 → 1
Char. 132: 1 → 0
Char. 144: 1 → 0
Char. 147: 0 → 1
Char. 149: 1 → 0
Char. 150: 0 → 1
Char. 192: 1 → 0

***Barasaurus besairiei*:**
Char. 33: 1 → 0

***Bashkyroleter bashkyricus*:**
Char. 275: 1 → 0

***Bashkyroleter mesensis*:**
Char. 169: 1 → 0

***Belebey vegrandis*:**
Char. 154: 1 → 0

***Bradysaurus* spp.:**
Char. 19: 0 → 1
Char. 25: 1 → 0
Char. 73: 0 → 1
Char. 79: 0 → 1
Char. 135: 1 → 0
Char. 249: 0 → 1

***Candelaria barbouri*:**
Char. 0: 1 → 0
Char. 1: 0 → 1
Char. 5: 1 → 0
Char. 8: 0 → 1
Char. 15: 0 → 1
Char. 33: 1 → 2
Char. 49: 0 → 1
Char. 55: 1 → 0
Char. 59: 1 → 0
Char. 62: 1 → 0
Char. 72: 2 → 1
Char. 76: 0 → 1
Char. 79: 0 → 2
Char. 83: 0 → 1
Char. 88: 1 → 0
Char. 92: 1 → 0
Char. 95: 0 → 1
Char. 126: 0 → 1
Char. 132: 1 → 0
Char. 154: 1 → 2
Char. 159: 1 → 0
Char. 169: 1 → 0
Char. 265: 1 → 0
Char. 275: 0 → 1
Char. 276: 0 → 1
Char. 277: 0 → 1

**Captorhinidae:**
Char. 3: 0 → 1
Char. 25: 1 → 0
Char. 26: 0 → 1
Char. 73: 0 → 1
Char. 75: 0 → 1
Char. 83: 0 → 1
Char. 108: 1 → 0
Char. 180: 1 → 0
Char. 183: 1 → 0
Char. 201: 1 → 0
Char. 203: 1 → 2
Char. 216: 1 → 0
Char. 240: 1 → 0

**Caseidae:**
Char. 38: 1 → 0
Char. 46: 1 → 0
Char. 50: 1 → 0
Char. 170: 0 → 1
Char. 194: 0 → 1
Char. 273: 0 → 1
Char. 274: 0 → 1
Char. 278: 3 → 2

***Claudiosaurus germaini*:**
Char. 24: 0 → 1
Char. 27: 0 → 1
Char. 34: 0 → 1
Char. 36: 0 → 1
Char. 56: 0 → 1
Char. 64: 0 → 1
Char. 70: 0 → 1
Char. 84: 1 → 0
Char. 105: 0 → 1
Char. 106: 0 → 1
Char. 117: 01 → 2
Char. 126: 0 → 1
Char. 127: 1 → 0
Char. 130: 0 → 1
Char. 131: 0 → 1
Char. 141: 1 → 0
Char. 144: 1 → 0
Char. 148: 0 → 1
Char. 154: 1 → 0
Char. 166: 1 → 0
Char. 182: 0 → 1
Char. 187: 1 → 0
Char. 190: 0 → 1
Char. 199: 0 → 1
Char. 201: 0 → 1
Char. 203: 1 → 2
Char. 204: 0 → 1
Char. 222: 1 → 0
Char. 234: 1 → 0
Char. 267: 0 → 1
Char. 272: 2 → 1

***Colobomycter pholeter*:**
Char. 21: 0 → 1
Char. 25: 1 → 0
Char. 84: 1 → 0
Char. 154: 1 → 0
Char. 167: 0 → 1
Char. 267: 0 → 1

***Delorhynchus cifelli*:**
Char. 18: 0 → 1
Char. 20: 0 → 1
Char. 21: 0 → 1
Char. 24: 0 → 1
Char. 26: 0 → 1
Char. 28: 0 → 1
Char. 33: 0 → 2
Char. 39: 0 → 1
Char. 52: 0 → 1
Char. 100: 0 → 1
Char. 111: 0 → 1
Char. 116: 1 → 0
Char. 119: 1 → 0
Char. 131: 0 → 1
Char. 147: 0 → 1
Char. 156: 1 → 0
Char. 167: 0 → 1
Char. 189: 0 → 1
Char. 191: 0 → 1
Char. 204: 0 → 2
Char. 267: 0 → 1

**Diadectomorpha:**
Char. 0: 0 → 1
Char. 64: 0 → 1
Char. 70: 0 → 1
Char. 122: 0 → 1
Char. 123: 1 → 0
Char. 146: 1 → 0
Char. 275: 1 → 0
Char. 278: 3 → 0

***Emeroleter levis*:**
Char. 51: 1 → 0

**Eosauropterygia:**
Char. 166: 1 → 0
Char. 174: 0 → 1
Char. 178: 0 → 1
Char. 194: 0 → 2
Char. 272: 1 → 0

***Eudibamus cursoris*:**
Char. 154: 1 → 2

***Feeserpeton oklahomensis*:**
Char. 51: 0 → 1
Char. 157: 0 → 1
Char. 158: 0 → 1

***Hovasaurus boulei*:**
Char. 41: 0 → 1
Char. 43: 0 → 1
Char. 55: 1 → 0
Char. 72: 2 → 1
Char. 77: 0 → 2
Char. 78: 1 → 0
Char. 79: 0 → 1
Char. 93: 1 → 0
Char. 113: 0 → 1
Char. 138: 0 → 1
Char. 141: 1 → 0
Char. 146: 1 → 0
Char. 204: 0 → 2

Char. 206: 0 → 2
Char. 215: 0 → 1
Char. 219: 1 → 0
Char. 224: 0 → 1

**Kuehneosauridae:**
Char. 7: 0 → 1
Char. 24: 0 → 1
Char. 27: 0 → 1
Char. 43: 1 → 0
Char. 79: 0 → 2
Char. 107: 1 → 0
Char. 140: 1 → 0
Char. 147: 1 → 0
Char. 148: 1 → 0
Char. 245: 0 → 1
Char. 278: 0 → 3

***Lanthanosuchus watsoni*:**
Char. 25: 1 → 0
Char. 51: 0 → 1
Char. 76: 0 → 1
Char. 86: 0 → 1
Char. 98: 1 → 0
Char. 138: 0 → 1
Char. 144: 1 → 0
Char. 154: 1 → 2

***Macroleter poezicus*:**
Char. 9: 0 → 1
Char. 26: 0 → 1
Char. 66: 1 → 2
Char. 126: 0 → 1
Char. 134: 0 → 2
Char. 140: 0 → 1
Char. 146: 1 → 0
Char. 169: 1 → 0

***Mesosaurus* spp.:**
Char. 0: 0 → 1
Char. 2: 0 → 1
Char. 6: 0 → 1
Char. 8: 0 → 1
Char. 9: 0 → 1
Char. 13: 0 → 1
Char. 19: 0 → 1
Char. 26: 0 → 1
Char. 29: 1 → 0
Char. 33: 0 → 1
Char. 38: 1 → 0
Char. 41: 0 → 1
Char. 50: 1 → 0
Char. 67: 0 → 1
Char. 76: 0 → 1
Char. 83: 0 → 1
Char. 84: 1 → 0
Char. 85: 1 → 0
Char. 94: 0 → 1
Char. 107: 0 → 1
Char. 109: 0 → 1
Char. 111: 0 → 1
Char. 115: 0 → 1
Char. 146: 1 → 0
Char. 148: 0 → 1
Char. 149: 1 → 0
Char. 164: 0 → 1
Char. 166: 1 → 0
Char. 167: 0 → 1
Char. 176: 0 → 1
Char. 183: 1 → 0
Char. 184: 0 → 1
Char. 192: 1 → 0
Char. 199: 0 → 1
Char. 202: 0 → 1
Char. 204: 0 → 2
Char. 206: 0 → 1
Char. 207: 0 → 1
Char. 209: 0 → 1
Char. 217: 0 → 1
Char. 219: 0 → 1
Char. 220: 0 → 1
Char. 231: 0 → 1
Char. 240: 1 → 0
Char. 260: 2 → 0
Char. 272: 2 → 0
Char. 278: 3 → 0

***Microleter mckinzieorum*:**
Char. 0: 0 → 1
Char. 18: 0 → 1
Char. 39: 0 → 1
Char. 57: 1 → 0
Char. 70: 0 → 1
Char. 76: 0 → 1
Char. 83: 0 → 1
Char. 94: 0 → 1
Char. 106: 0 → 1
Char. 110: 0 → 1
Char. 278: 3 → 1

**Millerettidae:**
Char. 24: 0 → 1
Char. 25: 1 → 0
Char. 44: 1 → 0
Char. 56: 0 → 1
Char. 57: 1 → 0
Char. 66: 0 → 2
Char. 78: 1 → 0
Char. 80: 1 → 0
Char. 84: 1 → 2
Char. 88: 1 → 0
Char. 96: 1 → 0
Char. 121: 0 → 1
Char. 124: 0 → 1
Char. 127: 0 → 1
Char. 135: 0 → 1
Char. 145: 0 → 1
Char. 166: 1 → 0
Char. 180: 1 → 0
Char. 192: 1 → 0
Char. 202: 0 → 1
Char. 211: 0 → 1
Char. 230: 0 → 1
Char. 248: 0 → 1
Char. 252: 0 → 1
Char. 253: 0 → 1

***Nycteroleter ineptus*:**
Char. 278: 0 → 3

***Nyctiphruretus acudens*:**
Char. 0: 0 → 1
Char. 21: 0 → 1
Char. 33: 1 → 2
Char. 41: 0 → 1
Char. 66: 1 → 2
Char. 81: 1 → 0
Char. 83: 0 → 1
Char. 84: 1 → 2
Char. 94: 0 → 1
Char. 166: 1 → 0
Char. 224: 0 → 1
Char. 226: 1 → 0
Char. 266: 0 → 1
Char. 272: 2 → 1
Char. 276: 0 → 1

***Orovenator mayorum*:**
Char. 8: 0 → 1
Char. 24: 0 → 1
Char. 33: 1 → 0
Char. 36: 0 → 1
Char. 50: 0 → 1
Char. 92: 1 → 0
Char. 94: 1 → 0
Char. 135: 1 → 0
Char. 159: 1 → 0
Char. 160: 0 → 1
Char. 165: 0 → 1
Char. 278: 03 → 1

***Owenetta* spp.:**
Char. 142: 1 → 0
Char. 169: 1 → 0

***Paleothyris acadiana*:**
Char. 38: 1 → 0
Char. 50: 1 → 0
Char. 66: 1 → 2
Char. 102: 0 → 1
Char. 146: 1 → 0
Char. 237: 0 → 1
Char. 239: 0 → 1

***Placodus* spp.:**
Char. 0: 1 → 2
Char. 9: 0 → 1
Char. 12: 0 → 1
Char. 13: 0 → 1
Char. 19: 0 → 1
Char. 31: 0 → 1
Char. 46: 1 → 0
Char. 57: 0 → 1
Char. 78: 1 → 0
Char. 93: 1 → 0
Char. 102: 1 → 2
Char. 109: 1 → 0
Char. 140: 1 → 0
Char. 155: 0 → 1
Char. 163: 1 → 0
Char. 164: 0 → 1

***Procolophon* spp.:**
Char. 41: 0 → 1
Char. 69: 0 → 1
Char. 79: 0 → 1
Char. 83: 0 → 1
Char. 86: 0 → 1
Char. 88: 0 → 1
Char. 101: 0 → 1
Char. 117: 1 → 0
Char. 141: 0 → 1
Char. 149: 1 → 0
Char. 203: 1 → 2
Char. 204: 0 → 1
Char. 230: 0 → 1
Char. 237: 0 → 1
Char. 238: 0 → 1
Char. 272: 2 → 1
Char. 278: 3 → 0

***Prolacerta broomi*:**
Char. 58: 1 → 0
Char. 66: 1 → 0
Char. 67: 1 → 0
Char. 80: 1 → 0
Char. 139: 1 → 0
Char. 147: 1 → 0
Char. 192: 1 → 0
Char. 203: 1 → 2
Char. 206: 0 → 12

***Rhipaeosaurus* spp.:**
Char. 172: 0 → 1
Char. 277: 0 → 1

**Rhynchocephalia:**
Char. 0: 1 → 2
Char. 24: 0 → 1
Char. 77: 0 → 1
Char. 88: 1 → 0
Char. 94: 1 → 0
Char. 139: 1 → 0
Char. 167: 1 → 0
Char. 205: 1 → 0

**Rhynchosauria:**
Char. 0: 1 → 0
Char. 7: 0 → 1
Char. 9: 0 → 1
Char. 26: 0 → 1
Char. 44: 1 → 0
Char. 68: 0 → 1
Char. 99: 1 → 0
Char. 150: 1 → 0
Char. 160: 0 → 1
Char. 161: 0 → 1
Char. 171: 0 → 2
Char. 223: 0 → 1
Char. 224: 0 → 1
Char. 241: 0 → 1

***Scutosaurus* spp.:**
Char. 175: 0 → 1
Char. 190: 0 → 1
Char. 218: 0 → 1
Char. 243: 0 → 2
Char. 244: 0 → 1
Char. 251: 0 → 1

***Sinosaurosphargis yunguiensis*:**
Char. 8: 0 → 1
Char. 30: 0 → 1
Char. 53: 0 → 1
Char. 89: 1 → 0
Char. 127: 1 → 0
Char. 150: 1 → 0
Char. 154: 0 → 2
Char. 167: 1 → 0
Char. 253: 0 → 1
Char. 255: 0 → 1

**Squamata:**
Char. 45: 0 → 1
Char. 79: 0 → 2
Char. 80: 1 → 0
Char. 92: 1 → 0
Char. 109: 1 → 0
Char. 160: 0 → 1
Char. 200: 0 → 1
Char. 245: 0 → 1

***Trilophosaurus buettneri*:**
Char. 5: 1 → 0
Char. 11: 0 → 1
Char. 55: 1 → 0
Char. 93: 1 → 0
Char. 104: 0 → 1
Char. 106: 0 → 1
Char. 113: 0 → 1
Char. 122: 0 → 1
Char. 136: 1 → 0
Char. 144: 1 → 0
Char. 157: 0 → 1
Char. 159: 1 → 0
Char. 177: 0 → 12
Char. 194: 0 → 1
Char. 203: 1 → 2
Char. 207: 1 → 0
Char. 208: 1 → 0
Char. 272: 1 → 0

***Youngina capensis*:**
Char. 5: 1 → 0
Char. 21: 0 → 1
Char. 25: 0 → 1
Char. 27: 0 → 1
Char. 38: 1 → 0
Char. 43: 0 → 1
Char. 44: 1 → 0
Char. 56: 0 → 1
Char. 75: 0 → 1
Char. 84: 1 → 0
Char. 92: 1 → 0
Char. 94: 1 → 0
Char. 134: 0 → 1
Char. 154: 1 → 0
Char. 163: 1 → 0
Char. 170: 0 → 1
Char. 211: 0 → 1

Char. 215: 0 → 1
Char. 224: 0 → 1
Char. 239: 0 → 1
Char. 267: 0 → 1

**Node 50:**
Char. 46: 1 → 0
Char. 88: 1 → 0
Char. 89: 1 → 0
Char. 93: 1 → 0
Char. 176: 0 → 1
Char. 195: 0 → 2
Char. 198: 0 → 1
Char. 246: 1 → 2
Char. 259: 0 → 1
Char. 265: 1 → 0
Char. 270: 0 → 1

**Node 51:**
Char. 65: 0 → 1
Char. 131: 0 → 1
Char. 184: 0 → 1
Char. 205: 0 → 1
Char. 210: 0 → 1
Char. 219: 1 → 0
Char. 241: 0 → 1
Char. 246: 0 → 1
Char. 254: 0 → 1
Char. 255: 0 → 1
Char. 256: 0 → 1
Char. 267: 0 → 1
Char. 268: 0 → 1
Char. 269: 0 → 1

**Node 52:**
Char. 0: 1 → 2
Char. 15: 0 → 1
Char. 21: 0 → 1
Char. 33: 1 → 2
Char. 44: 1 → 0
Char. 62: 1 → 0
Char. 64: 0 → 1
Char. 84: 1 → 2
Char. 130: 0 → 1
Char. 134: 0 → 2
Char. 147: 0 → 1
Char. 148: 0 → 1
Char. 152: 0 → 1
Char. 155: 0 → 1
Char. 158: 0 → 1
Char. 161: 0 → 1
Char. 174: 0 → 1
Char. 181: 0 → 1
Char. 203: 1 → 2
Char. 204: 0 → 2
Char. 247: 0 → 1
Char. 251: 0 → 1
Char. 252: 0 → 1
Char. 253: 0 → 2

**Node 53:**
Char. 0: 0 → 1
Char. 20: 0 → 1
Char. 25: 1 → 0
Char. 29: 1 → 0
Char. 33: 0 → 1
Char. 40: 2 → 0
Char. 50: 1 → 0
Char. 57: 1 → 0
Char. 59: 0 → 1
Char. 62: 0 → 1
Char. 67: 0 → 1
Char. 72: 1 → 2
Char. 89: 0 → 1
Char. 94: 0 → 1
Char. 109: 0 → 1
Char. 111: 0 → 1
Char. 120: 0 → 1
Char. 127: 0 → 1
Char. 129: 0 → 1
Char. 135: 0 → 1
Char. 136: 0 → 1
Char. 139: 0 → 1
Char. 141: 0 → 1
Char. 179: 0 → 1
Char. 187: 0 → 1
Char. 188: 0 → 1
Char. 191: 0 → 1
Char. 193: 0 → 1
Char. 196: 0 → 1
Char. 201: 1 → 0
Char. 202: 0 → 1
Char. 207: 0 → 1
Char. 209: 0 → 1
Char. 219: 0 → 1
Char. 222: 0 → 1
Char. 237: 0 → 1
Char. 265: 0 → 1
Char. 266: 0 → 1
Char. 275: 1 → 0

**Node 54:**
Char. 132: 0 → 1
Char. 166: 0 → 1
Char. 276: 1 → 0

**Node 55:**
Char. 29: 0 → 1
Char. 84: 0 → 1
Char. 88: 0 → 1
Char. 169: 0 → 1

**Node 56:**
Char. 72: 0 → 1
Char. 81: 0 → 1
Char. 93: 0 → 1
Char. 97: 0 → 1
Char. 104: 1 → 0
Char. 144: 0 → 1
Char. 173: 0 → 1
Char. 183: 0 → 1

**Node 58:**
Char. 95: 0 → 1
Char. 113: 0 → 1
Char. 114: 0 → 1

**Node 59:**
Char. 20: 0 → 1
Char. 47: 0 → 1
Char. 79: 0 → 1
Char. 110: 0 → 1
Char. 131: 0 → 1
Char. 137: 0 → 1
Char. 140: 0 → 2
Char. 147: 0 → 1

**Node 60:**
Char. 19: 0 → 1
Char. 92: 1 → 0

**Node 61:**
Char. 4: 0 → 1
Char. 15: 0 → 1
Char. 29: 0 → 1
Char. 213: 0 → 1
Char. 226: 0 → 2
Char. 228: 0 → 1
Char. 275: 0 → 1

**Node 62:**
Char. 43: 0 → 1
Char. 58: 0 → 1
Char. 61: 0 → 1
Char. 66: 0 → 1
Char. 69: 0 → 1
Char. 107: 0 → 1
Char. 126: 0 → 1
Char. 131: 0 → 1
Char. 134: 0 → 1
Char. 140: 0 → 1
Char. 147: 0 → 1
Char. 148: 0 → 1
Char. 150: 0 → 1
Char. 167: 0 → 1
Char. 176: 0 → 1
Char. 190: 0 → 1
Char. 205: 0 → 1
Char. 208: 0 → 1
Char. 230: 0 → 1
Char. 239: 0 → 1
Char. 260: 2 → 1

**Node 63:**
Char. 73: 0 → 1
Char. 131: 1 → 0
Char. 205: 0 → 1
Char. 273: 0 → 1
Char. 276: 0 → 1

**Node 64:**
Char. 18: 0 → 1
Char. 25: 1 → 0
Char. 37: 0 → 1
Char. 103: 0 → 1
Char. 106: 0 → 1
Char. 107: 0 → 1
Char. 110: 0 → 1
Char. 126: 0 → 1
Char. 150: 0 → 1

**Node 65:**
Char. 186: 0 → 1

**Node 66:**
Char. 148: 0 → 1
Char. 183: 1 → 2
Char. 194: 0 → 1
Char. 201: 1 → 0
Char. 211: 0 → 1
Char. 226: 0 → 1
Char. 240: 1 → 0
Char. 252: 0 → 1

**Node 67:**
Char. 87: 0 → 1
Char. 110: 0 → 1

**Node 68:**
Char. 25: 1 → 0
Char. 76: 1 → 0

**Node 69:**
Char. 79: 0 → 1
Char. 133: 0 → 1

**Node 70:**
Char. 100: 0 → 1
Char. 113: 0 → 1

**Node 71:**
Char. 38: 1 → 2
Char. 39: 0 → 1
Char. 50: 1 → 0
Char. 58: 0 → 1
Char. 59: 0 → 1
Char. 60: 0 → 1
Char. 72: 1 → 2
Char. 83: 0 → 1
Char. 85: 1 → 0
Char. 88: 1 → 0
Char. 95: 0 → 1
Char. 104: 0 → 1
Char. 105: 0 → 1
Char. 106: 0 → 2
Char. 107: 0 → 1
Char. 109: 0 → 1
Char. 110: 0 → 1
Char. 146: 1 → 0
Char. 148: 0 → 1
Char. 155: 0 → 2
Char. 183: 1 → 2

**Node 72:**
Char. 33: 1 → 0
Char. 38: 2 → 1
Char. 39: 1 → 0
Char. 42: 0 → 1
Char. 43: 0 → 1
Char. 46: 1 → 0
Char. 49: 1 → 0
Char. 71: 1 → 0
Char. 83: 0 → 2
Char. 101: 0 → 1
Char. 103: 0 → 1
Char. 106: 0 → 1
Char. 161: 0 → 1
Char. 163: 1 → 0
Char. 172: 0 → 2
Char. 174: 0 → 1
Char. 188: 0 → 1
Char. 203: 1 → 2
Char. 212: 0 → 1
Char. 216: 1 → 0
Char. 236: 0 → 1
Char. 238: 0 → 2
Char. 241: 0 → 1
Char. 242: 0 → 1
Char. 273: 0 → 1
Char. 274: 0 → 1
Char. 275: 1 → 0

**Node 73:**
Char. 52: 0 → 1
Char. 75: 0 → 1
Char. 84: 1 → 0
Char. 87: 0 → 1
Char. 110: 0 → 1
Char. 121: 0 → 1
Char. 180: 1 → 0
Char. 230: 0 → 1

**Node 74:**
Char. 29: 1 → 0
Char. 44: 1 → 0
Char. 59: 0 → 1
Char. 60: 0 → 1
Char. 66: 0 → 1
Char. 67: 0 → 1
Char. 72: 1 → 2
Char. 78: 1 → 0
Char. 84: 1 → 0
Char. 88: 1 → 0
Char. 93: 1 → 0
Char. 111: 0 → 1
Char. 123: 1 → 0
Char. 129: 0 → 1
Char. 154: 1 → 0
Char. 169: 1 → 0
Char. 170: 0 → 1
Char. 192: 1 → 0
Char. 197: 0 → 1

**Node 75:**
Char. 83: 0 → 1
Char. 85: 1 → 0
Char. 107: 0 → 1

**Node 76:**
Char. 2: 0 → 1
Char. 6: 0 → 1
Char. 186: 0 → 1
Char. 198: 0 → 1
Char. 200: 0 → 1
Char. 220: 0 → 1
Char. 239: 1 → 0

**Node 77:**
Char. 98: 1 → 0
Char. 113: 0 → 1
Char. 181: 0 → 1
Char. 206: 0 → 2

**Node 78:**
Char. 35: 0 → 1
Char. 102: 0 → 1
Char. 103: 0 → 1
Char. 184: 0 → 1
Char. 201: 0 → 1
Char. 210: 0 → 1
Char. 223: 0 → 1
Char. 229: 0 → 1

**Node 79:**
Char. 17: 0 → 1
Char. 42: 0 → 1
Char. 251: 0 → 1
Char. 264: 0 → 1

**Node 80:**
Char. 41: 0 → 1
Char. 61: 1 → 2
Char. 112: 1 → 0
Char. 138: 0 → 1
Char. 146: 1 → 0
Char. 155: 0 → 1
Char. 192: 1 → 2
Char. 224: 0 → 1
Char. 226: 0 → 1
Char. 227: 0 → 1
Char. 233: 0 → 1
Char. 267: 01 → 2

**Node 81:**
Char. 61: 1 → 3
Char. 90: 0 → 1
Char. 91: 0 → 1
Char. 109: 1 → 0
Char. 155: 0 → 1
Char. 209: 1 → 0

**ANALYSIS 56**
**(ALL TAXA, IMPLIED WEIGHTING, K = 5)**

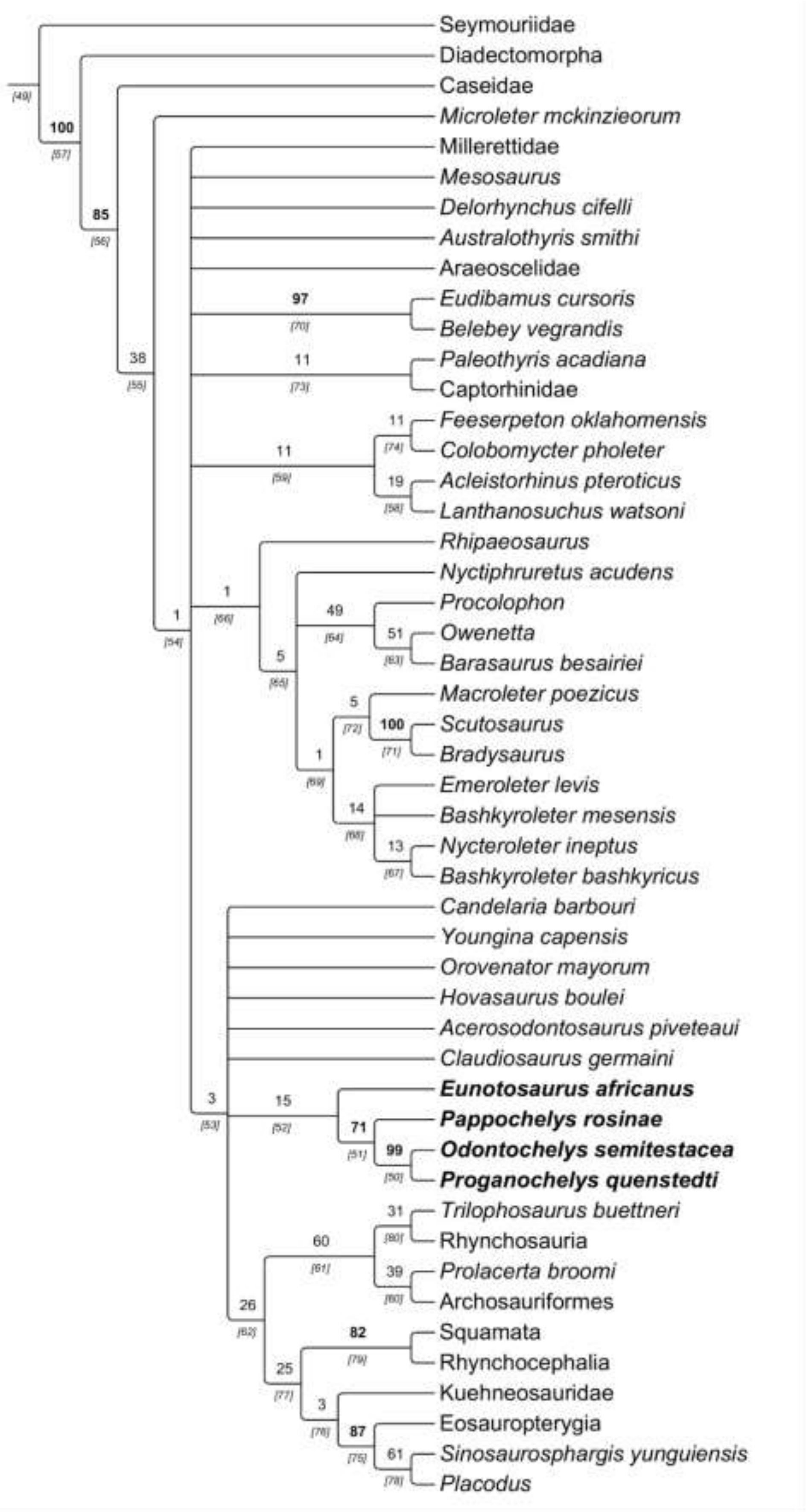

***Proganochelys quenstedti*:**
Char. 8: 0 → 1
Char. 11: 0 → 1
Char. 106: 0 → 1
Char. 108: 1 → 0
Char. 128: 0 → 1
Char. 175: 0 → 1
Char. 202: 1 → 0
Char. 207: 1 → 0
Char. 209: 1 → 0
Char. 244: 0 → 1
Char. 248: 0 → 1
Char. 250: 0 → 1
Char. 252: 1 → 0
Char. 262: 0 → 1

***Pappochelys rosinae*:**
Char. 0: 2 → 0
Char. 1: 0 → 1
Char. 5: 1 → 0
Char. 12: 0 → 1
Char. 41: 0 → 1
Char. 49: 0 → 1
Char. 75: 0 → 1
Char. 169: 1 → 0
Char. 260: 2 → 0

***Odontochelys semitestacea*:**
Char. 43: 0 → 1

***Eunotosaurus africanus*:**
Char. 19: 0 → 1
Char. 43: 0 → 1
Char. 59: 1 → 0
Char. 72: 2 → 1
Char. 76: 0 → 1
Char. 97: 1 → 0
Char. 103: 0 → 1
Char. 153: 1 → 0
Char. 191: 1 → 0
Char. 192: 1 → 0
Char. 202: 1 → 0
Char. 211: 0 → 1
Char. 222: 1 → 0
Char. 237: 1 → 0
Char. 248: 0 → 1
Char. 249: 0 → 2
Char. 250: 0 → 1
Char. 263: 0 → 1
Char. 273: 0 → 1
Char. 274: 0 → 1
Char. 275: 0 → 1
Char. 276: 0 → 1
Char. 277: 0 → 1

***Acerosodontosaurus piveteaui*:**
Char. 78: 1 → 0
Char. 81: 1 → 0
Char. 127: 1 → 0
Char. 128: 0 → 1
Char. 155: 0 → 1
Char. 206: 0 → 2
Char. 208: 0 → 1
Char. 278: 03 → 1

***Acleistorhinus pteroticus*:**
Char. 21: 0 → 1
Char. 146: 1 → 0

**Araeoscelidae:**
Char. 0: 0 → 1
Char. 27: 0 → 1
Char. 28: 0 → 1
Char. 29: 1 → 0
Char. 38: 1 → 0
Char. 40: 2 → 0
Char. 43: 0 → 1
Char. 50: 1 → 0
Char. 57: 1 → 0
Char. 59: 0 → 1
Char. 60: 0 → 1
Char. 67: 0 → 1
Char. 72: 1 → 2
Char. 84: 1 → 0
Char. 89: 0 → 1
Char. 106: 0 → 1
Char. 111: 0 → 1
Char. 116: 1 → 0
Char. 117: 01 → 2
Char. 120: 0 → 1
Char. 154: 1 → 0
Char. 166: 1 → 0
Char. 169: 1 → 0
Char. 170: 0 → 1
Char. 193: 0 → 1
Char. 197: 0 → 1
Char. 222: 0 → 1
Char. 224: 0 → 1
Char. 237: 0 → 1
Char. 239: 0 → 1
Char. 266: 0 → 1
Char. 278: 3 → 1

**Archosauriformes:**
Char. 32: 0 → 1
Char. 94: 1 → 0
Char. 112: 1 → 0
Char. 152: 0 → 1
Char. 154: 01 → 2
Char. 166: 1 → 0
Char. 171: 0 → 1
Char. 185: 0 → 1
Char. 201: 0 → 1
Char. 204: 0 → 1
Char. 218: 0 → 3
Char. 224: 0 → 1
Char. 242: 0 → 1

***Australothyris smithi*:**
Char. 24: 0 → 1
Char. 34: 0 → 1
Char. 55: 1 → 0
Char. 57: 1 → 0
Char. 71: 1 → 0
Char. 79: 0 → 1
Char. 83: 0 → 1
Char. 85: 1 → 0
Char. 98: 1 → 0
Char. 100: 0 → 1
Char. 103: 0 → 1
Char. 110: 0 → 1
Char. 123: 1 → 0
Char. 129: 0 → 1
Char. 131: 0 → 1
Char. 132: 1 → 0
Char. 144: 1 → 0
Char. 147: 0 → 1
Char. 149: 1 → 0
Char. 150: 0 → 1
Char. 192: 1 → 0

***Barasaurus besairiei*:**
Char. 33: 1 → 0

***Bashkyroleter bashkyricus*:**
Char. 275: 1 → 0

***Bashkyroleter mesensis*:**
Char. 12: 0 → 1
Char. 66: 1 → 0
Char. 169: 1 → 0

***Belebey vegrandis*:**
Char. 154: 1 → 0

***Bradysaurus* spp.:**
Char. 19: 0 → 1
Char. 25: 1 → 0
Char. 73: 0 → 1
Char. 79: 0 → 1
Char. 135: 1 → 0
Char. 249: 0 → 1

***Candelaria barbouri*:**
Char. 0: 1 → 0
Char. 1: 0 → 1
Char. 5: 1 → 0
Char. 8: 0 → 1
Char. 15: 0 → 1
Char. 33: 1 → 2
Char. 49: 0 → 1
Char. 55: 1 → 0
Char. 59: 1 → 0
Char. 62: 1 → 0
Char. 72: 2 → 1
Char. 76: 0 → 1
Char. 79: 0 → 2
Char. 83: 0 → 1
Char. 88: 1 → 0
Char. 92: 1 → 0
Char. 95: 0 → 1
Char. 126: 0 → 1
Char. 132: 1 → 0
Char. 154: 1 → 2
Char. 159: 1 → 0
Char. 169: 1 → 0
Char. 265: 1 → 0
Char. 275: 0 → 1
Char. 276: 0 → 1
Char. 277: 0 → 1

**Captorhinidae:**
Char. 3: 0 → 1
Char. 25: 1 → 0
Char. 26: 0 → 1
Char. 73: 0 → 1
Char. 75: 0 → 1
Char. 83: 0 → 1
Char. 108: 1 → 0
Char. 180: 1 → 0
Char. 183: 1 → 0
Char. 201: 1 → 0
Char. 203: 1 → 2
Char. 216: 1 → 0
Char. 240: 1 → 0

**Caseidae:**
Char. 38: 1 → 0
Char. 46: 1 → 0
Char. 50: 1 → 0
Char. 170: 0 → 1
Char. 194: 0 → 1
Char. 273: 0 → 1
Char. 274: 0 → 1
Char. 278: 3 → 2

***Claudiosaurus germaini*:**
Char. 24: 0 → 1
Char. 27: 0 → 1
Char. 34: 0 → 1
Char. 36: 0 → 1
Char. 56: 0 → 1
Char. 64: 0 → 1
Char. 70: 0 → 1
Char. 84: 1 → 0
Char. 105: 0 → 1
Char. 106: 0 → 1
Char. 117: 01 → 2
Char. 126: 0 → 1
Char. 127: 1 → 0
Char. 130: 0 → 1
Char. 131: 0 → 1
Char. 141: 1 → 0
Char. 144: 1 → 0
Char. 148: 0 → 1
Char. 154: 1 → 0
Char. 166: 1 → 0
Char. 182: 0 → 1
Char. 187: 1 → 0
Char. 190: 0 → 1
Char. 199: 0 → 1
Char. 201: 0 → 1
Char. 203: 1 → 2
Char. 204: 0 → 1
Char. 222: 1 → 0
Char. 234: 1 → 0
Char. 267: 0 → 1
Char. 272: 2 → 1

***Colobomycter pholeter*:**
Char. 21: 0 → 1
Char. 25: 1 → 0
Char. 84: 1 → 0
Char. 154: 1 → 0
Char. 167: 0 → 1
Char. 267: 0 → 1

***Delorhynchus cifelli*:**
Char. 18: 0 → 1
Char. 20: 0 → 1
Char. 21: 0 → 1
Char. 24: 0 → 1
Char. 26: 0 → 1
Char. 28: 0 → 1
Char. 33: 0 → 2
Char. 39: 0 → 1
Char. 52: 0 → 1
Char. 100: 0 → 1
Char. 111: 0 → 1
Char. 116: 1 → 0
Char. 119: 1 → 0
Char. 131: 0 → 1
Char. 147: 0 → 1
Char. 156: 1 → 0
Char. 167: 0 → 1
Char. 189: 0 → 1
Char. 191: 0 → 1
Char. 204: 0 → 2
Char. 267: 0 → 1

**Diadectomorpha:**
Char. 0: 0 → 1
Char. 64: 0 → 1
Char. 70: 0 → 1
Char. 122: 0 → 1
Char. 123: 1 → 0
Char. 146: 1 → 0
Char. 275: 1 → 0
Char. 278: 3 → 0

***Emeroleter levis*:**
Char. 51: 1 → 0

**Eosauropterygia:**
Char. 166: 1 → 0
Char. 174: 0 → 1
Char. 178: 0 → 1
Char. 194: 0 → 2
Char. 272: 1 → 0

***Eudibamus cursoris*:**
Char. 154: 1 → 2

***Feeserpeton oklahomensis*:**
Char. 51: 0 → 1
Char. 157: 0 → 1
Char. 158: 0 → 1

***Hovasaurus boulei*:**
Char. 41: 0 → 1
Char. 43: 0 → 1
Char. 55: 1 → 0
Char. 72: 2 → 1
Char. 77: 0 → 2
Char. 78: 1 → 0
Char. 79: 0 → 1
Char. 93: 1 → 0
Char. 113: 0 → 1
Char. 138: 0 → 1

Char. 141: 1 → 0
Char. 146: 1 → 0
Char. 204: 0 → 2
Char. 206: 0 → 2
Char. 215: 0 → 1
Char. 219: 1 → 0
Char. 224: 0 → 1

**Kuehneosauridae:**
Char. 7: 0 → 1
Char. 24: 0 → 1
Char. 27: 0 → 1
Char. 43: 1 → 0
Char. 79: 0 → 2
Char. 107: 1 → 0
Char. 140: 1 → 0
Char. 147: 1 → 0
Char. 148: 1 → 0
Char. 245: 0 → 1
Char. 278: 0 → 3

***Lanthanosuchus watsoni*:**
Char. 25: 1 → 0
Char. 51: 0 → 1
Char. 76: 0 → 1
Char. 86: 0 → 1
Char. 98: 1 → 0
Char. 138: 0 → 1
Char. 144: 1 → 0
Char. 154: 1 → 2

***Macroleter poezicus*:**
Char. 9: 0 → 1
Char. 26: 0 → 1
Char. 66: 1 → 2
Char. 126: 0 → 1
Char. 134: 0 → 2
Char. 140: 0 → 1
Char. 146: 1 → 0
Char. 169: 1 → 0

***Mesosaurus* spp.:**
Char. 0: 0 → 1
Char. 2: 0 → 1
Char. 6: 0 → 1
Char. 8: 0 → 1
Char. 9: 0 → 1
Char. 13: 0 → 1
Char. 19: 0 → 1
Char. 26: 0 → 1
Char. 29: 1 → 0
Char. 33: 0 → 1
Char. 38: 1 → 0
Char. 41: 0 → 1
Char. 50: 1 → 0
Char. 67: 0 → 1
Char. 76: 0 → 1
Char. 83: 0 → 1
Char. 84: 1 → 0
Char. 85: 1 → 0
Char. 94: 0 → 1
Char. 107: 0 → 1
Char. 109: 0 → 1
Char. 111: 0 → 1
Char. 115: 0 → 1
Char. 146: 1 → 0
Char. 148: 0 → 1
Char. 149: 1 → 0
Char. 164: 0 → 1
Char. 166: 1 → 0
Char. 167: 0 → 1
Char. 176: 0 → 1
Char. 183: 1 → 0
Char. 184: 0 → 1
Char. 192: 1 → 0
Char. 199: 0 → 1
Char. 202: 0 → 1
Char. 204: 0 → 2
Char. 206: 0 → 1
Char. 207: 0 → 1
Char. 209: 0 → 1
Char. 217: 0 → 1
Char. 219: 0 → 1
Char. 220: 0 → 1
Char. 231: 0 → 1
Char. 240: 1 → 0
Char. 260: 2 → 0
Char. 272: 2 → 0
Char. 278: 3 → 0

***Microleter mckinzieorum*:**
Char. 0: 0 → 1
Char. 18: 0 → 1
Char. 39: 0 → 1
Char. 57: 1 → 0
Char. 70: 0 → 1
Char. 76: 0 → 1
Char. 83: 0 → 1
Char. 94: 0 → 1
Char. 106: 0 → 1
Char. 110: 0 → 1
Char. 278: 3 → 1

**Millerettidae:**
Char. 24: 0 → 1
Char. 25: 1 → 0
Char. 44: 1 → 0
Char. 56: 0 → 1
Char. 57: 1 → 0
Char. 66: 0 → 2
Char. 78: 1 → 0
Char. 80: 1 → 0
Char. 84: 1 → 2
Char. 88: 1 → 0
Char. 96: 1 → 0
Char. 121: 0 → 1
Char. 124: 0 → 1
Char. 127: 0 → 1
Char. 135: 0 → 1
Char. 145: 0 → 1
Char. 166: 1 → 0
Char. 180: 1 → 0
Char. 192: 1 → 0
Char. 202: 0 → 1
Char. 211: 0 → 1
Char. 230: 0 → 1
Char. 248: 0 → 1
Char. 252: 0 → 1
Char. 253: 0 → 1

***Nycteroleter ineptus*:**
Char. 66: 1 → 0
Char. 278: 0 → 3

***Nyctiphruretus acudens*:**
Char. 0: 0 → 1
Char. 21: 0 → 1
Char. 33: 1 → 2
Char. 41: 0 → 1
Char. 66: 1 → 2
Char. 81: 1 → 0
Char. 83: 0 → 1
Char. 84: 1 → 2
Char. 94: 0 → 1
Char. 166: 1 → 0
Char. 224: 0 → 1
Char. 226: 1 → 0
Char. 266: 0 → 1
Char. 272: 2 → 1
Char. 276: 0 → 1

***Orovenator mayorum*:**
Char. 8: 0 → 1
Char. 24: 0 → 1
Char. 33: 1 → 0
Char. 36: 0 → 1
Char. 50: 0 → 1
Char. 92: 1 → 0
Char. 94: 1 → 0
Char. 135: 1 → 0
Char. 159: 1 → 0
Char. 160: 0 → 1
Char. 165: 0 → 1
Char. 278: 03 → 1

***Owenetta* spp.:**
Char. 142: 1 → 0
Char. 169: 1 → 0

***Paleothyris acadiana*:**
Char. 38: 1 → 0
Char. 50: 1 → 0
Char. 66: 1 → 2
Char. 102: 0 → 1
Char. 146: 1 → 0
Char. 237: 0 → 1
Char. 239: 0 → 1

***Placodus* spp.:**
Char. 0: 1 → 2
Char. 9: 0 → 1
Char. 12: 0 → 1
Char. 13: 0 → 1
Char. 19: 0 → 1
Char. 31: 0 → 1
Char. 46: 1 → 0
Char. 57: 0 → 1
Char. 78: 1 → 0
Char. 93: 1 → 0
Char. 102: 1 → 2
Char. 109: 1 → 0
Char. 140: 1 → 0
Char. 155: 0 → 1
Char. 163: 1 → 0
Char. 164: 0 → 1

***Procolophon* spp.:**
Char. 41: 0 → 1
Char. 69: 0 → 1
Char. 79: 0 → 1
Char. 83: 0 → 1
Char. 86: 0 → 1
Char. 88: 0 → 1
Char. 101: 0 → 1
Char. 117: 1 → 0
Char. 141: 0 → 1
Char. 149: 1 → 0
Char. 203: 1 → 2
Char. 204: 0 → 1
Char. 230: 0 → 1
Char. 237: 0 → 1
Char. 238: 0 → 1
Char. 272: 2 → 1
Char. 278: 3 → 0

***Prolacerta broomi*:**
Char. 58: 1 → 0
Char. 66: 1 → 0
Char. 67: 1 → 0
Char. 80: 1 → 0
Char. 139: 1 → 0
Char. 147: 1 → 0
Char. 192: 1 → 0
Char. 203: 1 → 2
Char. 206: 0 → 12

***Rhipaeosaurus* spp.:**
Char. 172: 0 → 1
Char. 277: 0 → 1

**Rhynchocephalia:**
Char. 0: 1 → 2
Char. 24: 0 → 1
Char. 77: 0 → 1
Char. 88: 1 → 0
Char. 94: 1 → 0
Char. 139: 1 → 0
Char. 167: 1 → 0
Char. 205: 1 → 0

**Rhynchosauria:**
Char. 0: 1 → 0
Char. 7: 0 → 1
Char. 9: 0 → 1
Char. 26: 0 → 1
Char. 44: 1 → 0
Char. 68: 0 → 1
Char. 99: 1 → 0
Char. 150: 1 → 0
Char. 160: 0 → 1
Char. 161: 0 → 1
Char. 171: 0 → 2
Char. 223: 0 → 1
Char. 224: 0 → 1
Char. 241: 0 → 1

***Scutosaurus* spp.:**
Char. 175: 0 → 1
Char. 190: 0 → 1
Char. 218: 0 → 1
Char. 243: 0 → 2
Char. 244: 0 → 1
Char. 251: 0 → 1

***Sinosaurosphargis yunguiensis*:**
Char. 8: 0 → 1
Char. 30: 0 → 1
Char. 53: 0 → 1
Char. 89: 1 → 0
Char. 127: 1 → 0
Char. 150: 1 → 0
Char. 154: 0 → 2
Char. 167: 1 → 0
Char. 253: 0 → 1
Char. 255: 0 → 1

**Squamata:**
Char. 45: 0 → 1
Char. 79: 0 → 2
Char. 80: 1 → 0
Char. 92: 1 → 0
Char. 109: 1 → 0
Char. 160: 0 → 1
Char. 200: 0 → 1
Char. 245: 0 → 1

***Trilophosaurus buettneri*:**
Char. 5: 1 → 0
Char. 11: 0 → 1
Char. 55: 1 → 0
Char. 93: 1 → 0
Char. 104: 0 → 1
Char. 106: 0 → 1
Char. 113: 0 → 1
Char. 122: 0 → 1
Char. 136: 1 → 0
Char. 144: 1 → 0
Char. 157: 0 → 1
Char. 159: 1 → 0
Char. 177: 0 → 12
Char. 194: 0 → 1
Char. 203: 1 → 2
Char. 207: 1 → 0
Char. 208: 1 → 0
Char. 272: 1 → 0

***Youngina capensis*:**
Char. 5: 1 → 0
Char. 21: 0 → 1
Char. 25: 0 → 1
Char. 27: 0 → 1
Char. 38: 1 → 0
Char. 43: 0 → 1
Char. 44: 1 → 0
Char. 56: 0 → 1
Char. 75: 0 → 1
Char. 84: 1 → 0
Char. 92: 1 → 0
Char. 94: 1 → 0

Char. 134: 0 → 1
Char. 154: 1 → 0
Char. 163: 1 → 0
Char. 170: 0 → 1
Char. 211: 0 → 1
Char. 215: 0 → 1
Char. 224: 0 → 1
Char. 239: 0 → 1
Char. 267: 0 → 1

**Node 50:**
Char. 46: 1 → 0
Char. 88: 1 → 0
Char. 89: 1 → 0
Char. 93: 1 → 0
Char. 176: 0 → 1
Char. 195: 0 → 2
Char. 198: 0 → 1
Char. 246: 1 → 2
Char. 259: 0 → 1
Char. 265: 1 → 0
Char. 270: 0 → 1

**Node 51:**
Char. 65: 0 → 1
Char. 131: 0 → 1
Char. 184: 0 → 1
Char. 205: 0 → 1
Char. 210: 0 → 1
Char. 219: 1 → 0
Char. 241: 0 → 1
Char. 246: 0 → 1
Char. 254: 0 → 1
Char. 255: 0 → 1
Char. 256: 0 → 1
Char. 267: 0 → 1
Char. 268: 0 → 1
Char. 269: 0 → 1

**Node 52:**
Char. 0: 1 → 2
Char. 15: 0 → 1
Char. 21: 0 → 1
Char. 33: 1 → 2
Char. 44: 1 → 0
Char. 62: 1 → 0
Char. 64: 0 → 1
Char. 84: 1 → 2
Char. 130: 0 → 1
Char. 134: 0 → 2
Char. 147: 0 → 1
Char. 148: 0 → 1
Char. 152: 0 → 1
Char. 155: 0 → 1
Char. 158: 0 → 1
Char. 161: 0 → 1
Char. 174: 0 → 1
Char. 181: 0 → 1
Char. 203: 1 → 2
Char. 204: 0 → 2
Char. 247: 0 → 1
Char. 251: 0 → 1
Char. 252: 0 → 1
Char. 253: 0 → 2

**Node 53:**
Char. 0: 0 → 1
Char. 20: 0 → 1
Char. 25: 1 → 0
Char. 29: 1 → 0
Char. 33: 0 → 1
Char. 40: 2 → 0
Char. 50: 1 → 0
Char. 57: 1 → 0
Char. 59: 0 → 1
Char. 62: 0 → 1
Char. 67: 0 → 1
Char. 72: 1 → 2
Char. 89: 0 → 1
Char. 94: 0 → 1
Char. 109: 0 → 1
Char. 111: 0 → 1
Char. 120: 0 → 1
Char. 127: 0 → 1
Char. 129: 0 → 1
Char. 135: 0 → 1
Char. 136: 0 → 1
Char. 139: 0 → 1
Char. 141: 0 → 1
Char. 179: 0 → 1
Char. 187: 0 → 1
Char. 188: 0 → 1
Char. 191: 0 → 1
Char. 193: 0 → 1
Char. 196: 0 → 1
Char. 201: 1 → 0
Char. 202: 0 → 1
Char. 207: 0 → 1
Char. 209: 0 → 1
Char. 219: 0 → 1
Char. 222: 0 → 1
Char. 237: 0 → 1
Char. 265: 0 → 1
Char. 266: 0 → 1
Char. 275: 1 → 0

**Node 54:**
Char. 132: 0 → 1
Char. 166: 0 → 1
Char. 276: 1 → 0

**Node 55:**
Char. 29: 0 → 1
Char. 84: 0 → 1
Char. 88: 0 → 1
Char. 169: 0 → 1

**Node 56:**
Char. 72: 0 → 1
Char. 81: 0 → 1
Char. 93: 0 → 1
Char. 97: 0 → 1
Char. 104: 1 → 0
Char. 144: 0 → 1
Char. 173: 0 → 1
Char. 183: 0 → 1

**Node 58:**
Char. 95: 0 → 1
Char. 113: 0 → 1
Char. 114: 0 → 1

**Node 59:**
Char. 20: 0 → 1
Char. 47: 0 → 1
Char. 79: 0 → 1
Char. 110: 0 → 1
Char. 131: 0 → 1
Char. 137: 0 → 1
Char. 140: 0 → 2
Char. 147: 0 → 1

**Node 60:**
Char. 19: 0 → 1
Char. 92: 1 → 0

**Node 61:**
Char. 4: 0 → 1
Char. 15: 0 → 1
Char. 29: 0 → 1
Char. 213: 0 → 1
Char. 226: 0 → 2
Char. 228: 0 → 1
Char. 275: 0 → 1

**Node 62:**
Char. 43: 0 → 1
Char. 58: 0 → 1
Char. 61: 0 → 1
Char. 66: 0 → 1
Char. 69: 0 → 1
Char. 107: 0 → 1
Char. 126: 0 → 1
Char. 131: 0 → 1
Char. 134: 0 → 1
Char. 140: 0 → 1
Char. 147: 0 → 1
Char. 148: 0 → 1
Char. 150: 0 → 1
Char. 167: 0 → 1
Char. 176: 0 → 1
Char. 190: 0 → 1
Char. 205: 0 → 1
Char. 208: 0 → 1
Char. 230: 0 → 1
Char. 239: 0 → 1
Char. 260: 2 → 1

**Node 63:**
Char. 73: 0 → 1
Char. 131: 1 → 0
Char. 205: 0 → 1
Char. 273: 0 → 1
Char. 276: 0 → 1

**Node 64:**
Char. 18: 0 → 1
Char. 25: 1 → 0
Char. 37: 0 → 1
Char. 103: 0 → 1
Char. 106: 0 → 1
Char. 107: 0 → 1
Char. 110: 0 → 1
Char. 126: 0 → 1
Char. 150: 0 → 1

**Node 65:**
Char. 186: 0 → 1

**Node 66:**
Char. 148: 0 → 1
Char. 183: 1 → 2
Char. 194: 0 → 1
Char. 201: 1 → 0
Char. 211: 0 → 1
Char. 226: 0 → 1
Char. 240: 1 → 0
Char. 252: 0 → 1

**Node 67:**
Char. 87: 0 → 1
Char. 110: 0 → 1

**Node 68:**
Char. 79: 0 → 1
Char. 133: 0 → 1

**Node 69:**
Char. 100: 0 → 1
Char. 113: 0 → 1

**Node 70:**
Char. 38: 1 → 2
Char. 39: 0 → 1
Char. 50: 1 → 0
Char. 58: 0 → 1
Char. 59: 0 → 1
Char. 60: 0 → 1
Char. 72: 1 → 2
Char. 83: 0 → 1
Char. 85: 1 → 0
Char. 88: 1 → 0
Char. 95: 0 → 1
Char. 104: 0 → 1
Char. 105: 0 → 1
Char. 106: 0 → 2
Char. 107: 0 → 1
Char. 109: 0 → 1
Char. 110: 0 → 1
Char. 146: 1 → 0
Char. 148: 0 → 1
Char. 155: 0 → 2
Char. 183: 1 → 2

**Node 71:**
Char. 33: 1 → 0
Char. 38: 2 → 1
Char. 39: 1 → 0
Char. 42: 0 → 1
Char. 43: 0 → 1
Char. 46: 1 → 0
Char. 49: 1 → 0
Char. 71: 1 → 0
Char. 83: 0 → 2
Char. 101: 0 → 1
Char. 103: 0 → 1
Char. 106: 0 → 1
Char. 161: 0 → 1
Char. 163: 1 → 0
Char. 172: 0 → 2
Char. 174: 0 → 1
Char. 188: 0 → 1
Char. 203: 1 → 2
Char. 212: 0 → 1
Char. 216: 1 → 0
Char. 236: 0 → 1
Char. 238: 0 → 2
Char. 241: 0 → 1
Char. 242: 0 → 1
Char. 273: 0 → 1
Char. 274: 0 → 1
Char. 275: 1 → 0

**Node 72:**
Char. 52: 0 → 1
Char. 75: 0 → 1
Char. 84: 1 → 0
Char. 87: 0 → 1
Char. 110: 0 → 1
Char. 121: 0 → 1
Char. 180: 1 → 0
Char. 230: 0 → 1

**Node 73:**
Char. 29: 1 → 0
Char. 44: 1 → 0
Char. 59: 0 → 1
Char. 60: 0 → 1
Char. 66: 0 → 1
Char. 67: 0 → 1
Char. 72: 1 → 2
Char. 78: 1 → 0
Char. 84: 1 → 0
Char. 88: 1 → 0
Char. 93: 1 → 0
Char. 111: 0 → 1
Char. 123: 1 → 0
Char. 129: 0 → 1
Char. 154: 1 → 0
Char. 169: 1 → 0
Char. 170: 0 → 1
Char. 192: 1 → 0
Char. 197: 0 → 1

**Node 74:**
Char. 83: 0 → 1
Char. 85: 1 → 0
Char. 107: 0 → 1

**Node 75:**
Char. 2: 0 → 1
Char. 6: 0 → 1
Char. 186: 0 → 1
Char. 198: 0 → 1
Char. 200: 0 → 1
Char. 220: 0 → 1
Char. 239: 1 → 0

**Node 76:**
Char. 98: 1 → 0
Char. 113: 0 → 1
Char. 181: 0 → 1
Char. 206: 0 → 2

**Node 77:**
Char. 35: 0 → 1
Char. 102: 0 → 1
Char. 103: 0 → 1
Char. 184: 0 → 1
Char. 201: 0 → 1
Char. 210: 0 → 1
Char. 223: 0 → 1
Char. 229: 0 → 1

**Node 78:**
Char. 17: 0 → 1
Char. 42: 0 → 1
Char. 251: 0 → 1
Char. 264: 0 → 1

**Node 79:**
Char. 41: 0 → 1
Char. 61: 1 → 2
Char. 112: 1 → 0
Char. 138: 0 → 1
Char. 146: 1 → 0
Char. 155: 0 → 1
Char. 192: 1 → 2
Char. 224: 0 → 1
Char. 226: 0 → 1
Char. 227: 0 → 1
Char. 233: 0 → 1
Char. 267: 01 → 2

**Node 80:**
Char. 61: 1 → 3
Char. 90: 0 → 1
Char. 91: 0 → 1
Char. 109: 1 → 0
Char. 155: 0 → 1
Char. 209: 1 → 0

**ANALYSIS 57**
**(ALL TAXA, IMPLIED WEIGHTING, K = 5.125)**

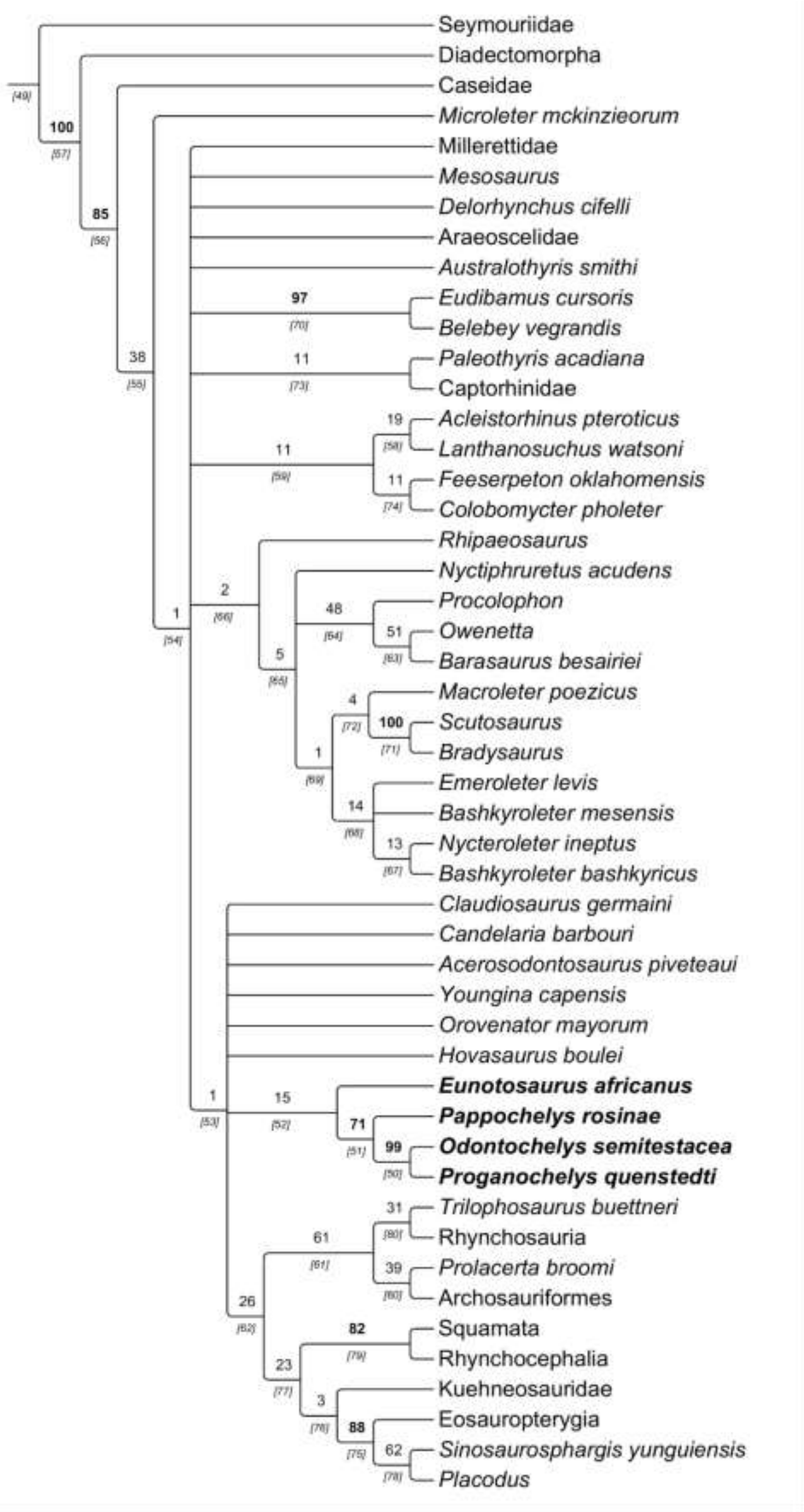

***Proganochelys quenstedti*:**
Char. 8: 0 → 1
Char. 11: 0 → 1
Char. 106: 0 → 1
Char. 108: 1 → 0
Char. 128: 0 → 1
Char. 175: 0 → 1
Char. 202: 1 → 0
Char. 207: 1 → 0
Char. 209: 1 → 0
Char. 244: 0 → 1
Char. 248: 0 → 1
Char. 250: 0 → 1
Char. 252: 1 → 0
Char. 262: 0 → 1

***Pappochelys rosinae*:**
Char. 0: 2 → 0
Char. 1: 0 → 1
Char. 5: 1 → 0
Char. 12: 0 → 1
Char. 41: 0 → 1
Char. 49: 0 → 1
Char. 75: 0 → 1
Char. 169: 1 → 0
Char. 260: 2 → 0

***Odontochelys semitestacea*:**
Char. 43: 0 → 1

***Eunotosaurus africanus*:**
Char. 19: 0 → 1
Char. 43: 0 → 1
Char. 59: 1 → 0
Char. 72: 2 → 1
Char. 76: 0 → 1
Char. 97: 1 → 0
Char. 103: 0 → 1
Char. 153: 1 → 0
Char. 191: 1 → 0
Char. 192: 1 → 0
Char. 202: 1 → 0
Char. 211: 0 → 1
Char. 222: 1 → 0
Char. 237: 1 → 0
Char. 248: 0 → 1
Char. 249: 0 → 2
Char. 250: 0 → 1
Char. 263: 0 → 1
Char. 273: 0 → 1
Char. 274: 0 → 1
Char. 275: 0 → 1
Char. 276: 0 → 1
Char. 277: 0 → 1

***Acerosodontosaurus piveteaui*:**
Char. 78: 1 → 0
Char. 81: 1 → 0
Char. 127: 1 → 0
Char. 128: 0 → 1
Char. 155: 0 → 1
Char. 206: 0 → 2
Char. 208: 0 → 1
Char. 278: 03 → 1

***Acleistorhinus pteroticus*:**
Char. 21: 0 → 1
Char. 146: 1 → 0

**Araeoscelidae:**
Char. 0: 0 → 1
Char. 27: 0 → 1
Char. 28: 0 → 1
Char. 29: 1 → 0
Char. 38: 1 → 0
Char. 40: 2 → 0
Char. 43: 0 → 1
Char. 50: 1 → 0
Char. 57: 1 → 0
Char. 59: 0 → 1
Char. 60: 0 → 1
Char. 67: 0 → 1
Char. 72: 1 → 2
Char. 84: 1 → 0
Char. 89: 0 → 1
Char. 106: 0 → 1
Char. 111: 0 → 1
Char. 116: 1 → 0
Char. 117: 01 → 2
Char. 120: 0 → 1
Char. 154: 1 → 0
Char. 166: 1 → 0
Char. 169: 1 → 0
Char. 170: 0 → 1
Char. 193: 0 → 1
Char. 197: 0 → 1
Char. 222: 0 → 1
Char. 224: 0 → 1
Char. 237: 0 → 1
Char. 239: 0 → 1
Char. 266: 0 → 1
Char. 278: 3 → 1

**Archosauriformes:**
Char. 32: 0 → 1
Char. 94: 1 → 0
Char. 112: 1 → 0
Char. 152: 0 → 1
Char. 154: 01 → 2
Char. 166: 1 → 0
Char. 171: 0 → 1
Char. 185: 0 → 1
Char. 201: 0 → 1
Char. 204: 0 → 1
Char. 218: 0 → 3
Char. 224: 0 → 1
Char. 242: 0 → 1

***Australothyris smithi*:**
Char. 24: 0 → 1
Char. 34: 0 → 1
Char. 55: 1 → 0
Char. 57: 1 → 0
Char. 71: 1 → 0
Char. 79: 0 → 1
Char. 83: 0 → 1
Char. 85: 1 → 0
Char. 98: 1 → 0
Char. 100: 0 → 1
Char. 103: 0 → 1
Char. 110: 0 → 1
Char. 123: 1 → 0
Char. 129: 0 → 1
Char. 131: 0 → 1
Char. 132: 1 → 0
Char. 144: 1 → 0
Char. 147: 0 → 1
Char. 149: 1 → 0
Char. 150: 0 → 1
Char. 192: 1 → 0

***Barasaurus besairiei*:**
Char. 33: 1 → 0

***Bashkyroleter bashkyricus*:**
Char. 275: 1 → 0

***Bashkyroleter mesensis*:**
Char. 12: 0 → 1
Char. 66: 1 → 0
Char. 169: 1 → 0

***Belebey vegrandis*:**
Char. 154: 1 → 0

***Bradysaurus* spp.:**
Char. 19: 0 → 1
Char. 25: 1 → 0
Char. 73: 0 → 1
Char. 79: 0 → 1
Char. 135: 1 → 0
Char. 249: 0 → 1

***Candelaria barbouri*:**
Char. 0: 1 → 0
Char. 1: 0 → 1
Char. 5: 1 → 0
Char. 8: 0 → 1
Char. 15: 0 → 1
Char. 33: 1 → 2
Char. 49: 0 → 1
Char. 55: 1 → 0
Char. 59: 1 → 0
Char. 62: 1 → 0
Char. 72: 2 → 1
Char. 76: 0 → 1
Char. 79: 0 → 2
Char. 83: 0 → 1
Char. 88: 1 → 0
Char. 92: 1 → 0
Char. 95: 0 → 1
Char. 126: 0 → 1
Char. 132: 1 → 0
Char. 154: 1 → 2
Char. 159: 1 → 0
Char. 169: 1 → 0
Char. 265: 1 → 0
Char. 275: 0 → 1
Char. 276: 0 → 1
Char. 277: 0 → 1

**Captorhinidae:**
Char. 3: 0 → 1
Char. 25: 1 → 0
Char. 26: 0 → 1
Char. 73: 0 → 1
Char. 75: 0 → 1
Char. 83: 0 → 1
Char. 108: 1 → 0
Char. 180: 1 → 0
Char. 183: 1 → 0
Char. 201: 1 → 0
Char. 203: 1 → 2
Char. 216: 1 → 0
Char. 240: 1 → 0

**Caseidae:**
Char. 38: 1 → 0
Char. 46: 1 → 0
Char. 50: 1 → 0
Char. 170: 0 → 1
Char. 194: 0 → 1
Char. 273: 0 → 1
Char. 274: 0 → 1
Char. 278: 3 → 2

***Claudiosaurus germaini*:**
Char. 24: 0 → 1
Char. 27: 0 → 1
Char. 34: 0 → 1
Char. 36: 0 → 1
Char. 56: 0 → 1
Char. 64: 0 → 1
Char. 70: 0 → 1
Char. 84: 1 → 0
Char. 105: 0 → 1
Char. 106: 0 → 1
Char. 117: 01 → 2
Char. 126: 0 → 1
Char. 127: 1 → 0
Char. 130: 0 → 1
Char. 131: 0 → 1
Char. 141: 1 → 0
Char. 144: 1 → 0
Char. 148: 0 → 1
Char. 154: 1 → 0
Char. 166: 1 → 0
Char. 182: 0 → 1
Char. 187: 1 → 0
Char. 190: 0 → 1
Char. 199: 0 → 1
Char. 201: 0 → 1
Char. 203: 1 → 2
Char. 204: 0 → 1
Char. 222: 1 → 0
Char. 234: 1 → 0
Char. 267: 0 → 1
Char. 272: 2 → 1

***Colobomycter pholeter*:**
Char. 21: 0 → 1
Char. 25: 1 → 0
Char. 84: 1 → 0
Char. 154: 1 → 0
Char. 167: 0 → 1
Char. 267: 0 → 1

***Delorhynchus cifelli*:**
Char. 18: 0 → 1
Char. 20: 0 → 1
Char. 21: 0 → 1
Char. 24: 0 → 1
Char. 26: 0 → 1
Char. 28: 0 → 1
Char. 33: 0 → 2
Char. 39: 0 → 1
Char. 52: 0 → 1
Char. 100: 0 → 1
Char. 111: 0 → 1
Char. 116: 1 → 0
Char. 119: 1 → 0
Char. 131: 0 → 1
Char. 147: 0 → 1
Char. 156: 1 → 0
Char. 167: 0 → 1
Char. 189: 0 → 1
Char. 191: 0 → 1
Char. 204: 0 → 2
Char. 267: 0 → 1

**Diadectomorpha:**
Char. 0: 0 → 1
Char. 64: 0 → 1
Char. 70: 0 → 1
Char. 122: 0 → 1
Char. 123: 1 → 0
Char. 146: 1 → 0
Char. 275: 1 → 0
Char. 278: 3 → 0

***Emeroleter levis*:**
Char. 51: 1 → 0

**Eosauropterygia:**
Char. 166: 1 → 0
Char. 174: 0 → 1
Char. 178: 0 → 1
Char. 194: 0 → 2
Char. 272: 1 → 0

***Eudibamus cursoris*:**
Char. 154: 1 → 2

***Feeserpeton oklahomensis*:**
Char. 51: 0 → 1
Char. 157: 0 → 1
Char. 158: 0 → 1

***Hovasaurus boulei*:**
Char. 41: 0 → 1
Char. 43: 0 → 1
Char. 55: 1 → 0
Char. 72: 2 → 1
Char. 77: 0 → 2
Char. 78: 1 → 0
Char. 79: 0 → 1
Char. 93: 1 → 0
Char. 113: 0 → 1
Char. 138: 0 → 1

Char. 141: 1 → 0
Char. 146: 1 → 0
Char. 204: 0 → 2
Char. 206: 0 → 2
Char. 215: 0 → 1
Char. 219: 1 → 0
Char. 224: 0 → 1

**Kuehneosauridae:**
Char. 7: 0 → 1
Char. 24: 0 → 1
Char. 27: 0 → 1
Char. 43: 1 → 0
Char. 79: 0 → 2
Char. 107: 1 → 0
Char. 140: 1 → 0
Char. 147: 1 → 0
Char. 148: 1 → 0
Char. 245: 0 → 1
Char. 278: 0 → 3

***Lanthanosuchus watsoni*:**
Char. 25: 1 → 0
Char. 51: 0 → 1
Char. 76: 0 → 1
Char. 86: 0 → 1
Char. 98: 1 → 0
Char. 138: 0 → 1
Char. 144: 1 → 0
Char. 154: 1 → 2

***Macroleter poezicus*:**
Char. 9: 0 → 1
Char. 26: 0 → 1
Char. 66: 1 → 2
Char. 126: 0 → 1
Char. 134: 0 → 2
Char. 140: 0 → 1
Char. 146: 1 → 0
Char. 169: 1 → 0

***Mesosaurus* spp.:**
Char. 0: 0 → 1
Char. 2: 0 → 1
Char. 6: 0 → 1
Char. 8: 0 → 1
Char. 9: 0 → 1
Char. 13: 0 → 1
Char. 19: 0 → 1
Char. 26: 0 → 1
Char. 29: 1 → 0
Char. 33: 0 → 1
Char. 38: 1 → 0
Char. 41: 0 → 1
Char. 50: 1 → 0
Char. 67: 0 → 1
Char. 76: 0 → 1
Char. 83: 0 → 1
Char. 84: 1 → 0
Char. 85: 1 → 0
Char. 94: 0 → 1
Char. 107: 0 → 1
Char. 109: 0 → 1
Char. 111: 0 → 1
Char. 115: 0 → 1
Char. 146: 1 → 0
Char. 148: 0 → 1
Char. 149: 1 → 0
Char. 164: 0 → 1
Char. 166: 1 → 0
Char. 167: 0 → 1
Char. 176: 0 → 1
Char. 183: 1 → 0
Char. 184: 0 → 1
Char. 192: 1 → 0
Char. 199: 0 → 1
Char. 202: 0 → 1
Char. 204: 0 → 2
Char. 206: 0 → 1
Char. 207: 0 → 1
Char. 209: 0 → 1
Char. 217: 0 → 1
Char. 219: 0 → 1
Char. 220: 0 → 1
Char. 231: 0 → 1
Char. 240: 1 → 0
Char. 260: 2 → 0
Char. 272: 2 → 0
Char. 278: 3 → 0

***Microleter mckinzieorum*:**
Char. 0: 0 → 1
Char. 18: 0 → 1
Char. 39: 0 → 1
Char. 57: 1 → 0
Char. 70: 0 → 1
Char. 76: 0 → 1
Char. 83: 0 → 1
Char. 94: 0 → 1
Char. 106: 0 → 1
Char. 110: 0 → 1
Char. 278: 3 → 1

**Millerettidae:**
Char. 24: 0 → 1
Char. 25: 1 → 0
Char. 44: 1 → 0
Char. 56: 0 → 1
Char. 57: 1 → 0
Char. 66: 0 → 2
Char. 78: 1 → 0
Char. 80: 1 → 0
Char. 84: 1 → 2
Char. 88: 1 → 0
Char. 96: 1 → 0
Char. 121: 0 → 1
Char. 124: 0 → 1
Char. 127: 0 → 1
Char. 135: 0 → 1
Char. 145: 0 → 1
Char. 166: 1 → 0
Char. 180: 1 → 0
Char. 192: 1 → 0
Char. 202: 0 → 1
Char. 211: 0 → 1
Char. 230: 0 → 1
Char. 248: 0 → 1
Char. 252: 0 → 1
Char. 253: 0 → 1

***Nycteroleter ineptus*:**
Char. 66: 1 → 0
Char. 278: 0 → 3

***Nyctiphruretus acudens*:**
Char. 0: 0 → 1
Char. 21: 0 → 1
Char. 33: 1 → 2
Char. 41: 0 → 1
Char. 66: 1 → 2
Char. 81: 1 → 0
Char. 83: 0 → 1
Char. 84: 1 → 2
Char. 94: 0 → 1
Char. 166: 1 → 0
Char. 224: 0 → 1
Char. 226: 1 → 0
Char. 266: 0 → 1
Char. 272: 2 → 1
Char. 276: 0 → 1

***Orovenator mayorum*:**
Char. 8: 0 → 1
Char. 24: 0 → 1
Char. 33: 1 → 0
Char. 36: 0 → 1
Char. 50: 0 → 1
Char. 92: 1 → 0
Char. 94: 1 → 0
Char. 135: 1 → 0
Char. 159: 1 → 0
Char. 160: 0 → 1
Char. 165: 0 → 1
Char. 278: 03 → 1

***Owenetta* spp.:**
Char. 142: 1 → 0
Char. 169: 1 → 0

***Paleothyris acadiana*:**
Char. 38: 1 → 0
Char. 50: 1 → 0
Char. 66: 1 → 2
Char. 102: 0 → 1
Char. 146: 1 → 0
Char. 237: 0 → 1
Char. 239: 0 → 1

***Placodus* spp.:**
Char. 0: 1 → 2
Char. 9: 0 → 1
Char. 12: 0 → 1
Char. 13: 0 → 1
Char. 19: 0 → 1
Char. 31: 0 → 1
Char. 46: 1 → 0
Char. 57: 0 → 1
Char. 78: 1 → 0
Char. 93: 1 → 0
Char. 102: 1 → 2
Char. 109: 1 → 0
Char. 140: 1 → 0
Char. 155: 0 → 1
Char. 163: 1 → 0
Char. 164: 0 → 1

***Procolophon* spp.:**
Char. 41: 0 → 1
Char. 69: 0 → 1
Char. 79: 0 → 1
Char. 83: 0 → 1
Char. 86: 0 → 1
Char. 88: 0 → 1
Char. 101: 0 → 1
Char. 117: 1 → 0
Char. 141: 0 → 1
Char. 149: 1 → 0
Char. 203: 1 → 2
Char. 204: 0 → 1
Char. 230: 0 → 1
Char. 237: 0 → 1
Char. 238: 0 → 1
Char. 272: 2 → 1
Char. 278: 3 → 0

***Prolacerta broomi*:**
Char. 58: 1 → 0
Char. 66: 1 → 0
Char. 67: 1 → 0
Char. 80: 1 → 0
Char. 139: 1 → 0
Char. 147: 1 → 0
Char. 192: 1 → 0
Char. 203: 1 → 2
Char. 206: 0 → 12

***Rhipaeosaurus* spp.:**
Char. 172: 0 → 1
Char. 277: 0 → 1

**Rhynchocephalia:**
Char. 0: 1 → 2
Char. 24: 0 → 1
Char. 77: 0 → 1
Char. 88: 1 → 0
Char. 94: 1 → 0
Char. 139: 1 → 0
Char. 167: 1 → 0
Char. 205: 1 → 0

**Rhynchosauria:**
Char. 0: 1 → 0
Char. 7: 0 → 1
Char. 9: 0 → 1
Char. 26: 0 → 1
Char. 44: 1 → 0
Char. 68: 0 → 1
Char. 99: 1 → 0
Char. 150: 1 → 0
Char. 160: 0 → 1
Char. 161: 0 → 1
Char. 171: 0 → 2
Char. 223: 0 → 1
Char. 224: 0 → 1
Char. 241: 0 → 1

***Scutosaurus* spp.:**
Char. 175: 0 → 1
Char. 190: 0 → 1
Char. 218: 0 → 1
Char. 243: 0 → 2
Char. 244: 0 → 1
Char. 251: 0 → 1

***Sinosaurosphargis yunguiensis*:**
Char. 8: 0 → 1
Char. 30: 0 → 1
Char. 53: 0 → 1
Char. 89: 1 → 0
Char. 127: 1 → 0
Char. 150: 1 → 0
Char. 154: 0 → 2
Char. 167: 1 → 0
Char. 253: 0 → 1
Char. 255: 0 → 1

**Squamata:**
Char. 45: 0 → 1
Char. 79: 0 → 2
Char. 80: 1 → 0
Char. 92: 1 → 0
Char. 109: 1 → 0
Char. 160: 0 → 1
Char. 200: 0 → 1
Char. 245: 0 → 1

***Trilophosaurus buettneri*:**
Char. 5: 1 → 0
Char. 11: 0 → 1
Char. 55: 1 → 0
Char. 93: 1 → 0
Char. 104: 0 → 1
Char. 106: 0 → 1
Char. 113: 0 → 1
Char. 122: 0 → 1
Char. 136: 1 → 0
Char. 144: 1 → 0
Char. 157: 0 → 1
Char. 159: 1 → 0
Char. 177: 0 → 12
Char. 194: 0 → 1
Char. 203: 1 → 2
Char. 207: 1 → 0
Char. 208: 1 → 0
Char. 272: 1 → 0

***Youngina capensis*:**
Char. 5: 1 → 0
Char. 21: 0 → 1
Char. 25: 0 → 1
Char. 27: 0 → 1
Char. 38: 1 → 0
Char. 43: 0 → 1
Char. 44: 1 → 0
Char. 56: 0 → 1
Char. 75: 0 → 1
Char. 84: 1 → 0
Char. 92: 1 → 0
Char. 94: 1 → 0

Char. 134: 0 → 1
Char. 154: 1 → 0
Char. 163: 1 → 0
Char. 170: 0 → 1
Char. 211: 0 → 1
Char. 215: 0 → 1
Char. 224: 0 → 1
Char. 239: 0 → 1
Char. 267: 0 → 1

**Node 50:**
Char. 46: 1 → 0
Char. 88: 1 → 0
Char. 89: 1 → 0
Char. 93: 1 → 0
Char. 176: 0 → 1
Char. 195: 0 → 2
Char. 198: 0 → 1
Char. 246: 1 → 2
Char. 259: 0 → 1
Char. 265: 1 → 0
Char. 270: 0 → 1

**Node 51:**
Char. 65: 0 → 1
Char. 131: 0 → 1
Char. 184: 0 → 1
Char. 205: 0 → 1
Char. 210: 0 → 1
Char. 219: 1 → 0
Char. 241: 0 → 1
Char. 246: 0 → 1
Char. 254: 0 → 1
Char. 255: 0 → 1
Char. 256: 0 → 1
Char. 267: 0 → 1
Char. 268: 0 → 1
Char. 269: 0 → 1

**Node 52:**
Char. 0: 1 → 2
Char. 15: 0 → 1
Char. 21: 0 → 1
Char. 33: 1 → 2
Char. 44: 1 → 0
Char. 62: 1 → 0
Char. 64: 0 → 1
Char. 84: 1 → 2
Char. 130: 0 → 1
Char. 134: 0 → 2
Char. 147: 0 → 1
Char. 148: 0 → 1
Char. 152: 0 → 1
Char. 155: 0 → 1
Char. 158: 0 → 1
Char. 161: 0 → 1
Char. 174: 0 → 1
Char. 181: 0 → 1
Char. 203: 1 → 2
Char. 204: 0 → 2
Char. 247: 0 → 1
Char. 251: 0 → 1
Char. 252: 0 → 1
Char. 253: 0 → 2

**Node 53:**
Char. 0: 0 → 1
Char. 20: 0 → 1
Char. 25: 1 → 0
Char. 29: 1 → 0
Char. 33: 0 → 1
Char. 40: 2 → 0
Char. 50: 1 → 0
Char. 57: 1 → 0
Char. 59: 0 → 1
Char. 62: 0 → 1
Char. 67: 0 → 1
Char. 72: 1 → 2
Char. 89: 0 → 1
Char. 94: 0 → 1
Char. 109: 0 → 1
Char. 111: 0 → 1
Char. 120: 0 → 1
Char. 127: 0 → 1
Char. 129: 0 → 1
Char. 135: 0 → 1
Char. 136: 0 → 1
Char. 139: 0 → 1
Char. 141: 0 → 1
Char. 179: 0 → 1
Char. 187: 0 → 1
Char. 188: 0 → 1
Char. 191: 0 → 1
Char. 193: 0 → 1
Char. 196: 0 → 1
Char. 201: 1 → 0
Char. 202: 0 → 1
Char. 207: 0 → 1
Char. 209: 0 → 1
Char. 219: 0 → 1
Char. 222: 0 → 1
Char. 237: 0 → 1
Char. 265: 0 → 1
Char. 266: 0 → 1
Char. 275: 1 → 0

**Node 54:**
Char. 132: 0 → 1
Char. 166: 0 → 1
Char. 276: 1 → 0

**Node 55:**
Char. 29: 0 → 1
Char. 84: 0 → 1
Char. 88: 0 → 1
Char. 169: 0 → 1

**Node 56:**
Char. 72: 0 → 1
Char. 81: 0 → 1
Char. 93: 0 → 1
Char. 97: 0 → 1
Char. 104: 1 → 0
Char. 144: 0 → 1
Char. 173: 0 → 1
Char. 183: 0 → 1

**Node 58:**
Char. 95: 0 → 1
Char. 113: 0 → 1
Char. 114: 0 → 1

**Node 59:**
Char. 20: 0 → 1
Char. 47: 0 → 1
Char. 79: 0 → 1
Char. 110: 0 → 1
Char. 131: 0 → 1
Char. 137: 0 → 1
Char. 140: 0 → 2
Char. 147: 0 → 1

**Node 60:**
Char. 19: 0 → 1
Char. 92: 1 → 0

**Node 61:**
Char. 4: 0 → 1
Char. 15: 0 → 1
Char. 29: 0 → 1
Char. 213: 0 → 1
Char. 226: 0 → 2
Char. 228: 0 → 1
Char. 275: 0 → 1

**Node 62:**
Char. 43: 0 → 1
Char. 58: 0 → 1
Char. 61: 0 → 1
Char. 66: 0 → 1
Char. 69: 0 → 1
Char. 107: 0 → 1
Char. 126: 0 → 1
Char. 131: 0 → 1
Char. 134: 0 → 1
Char. 140: 0 → 1
Char. 147: 0 → 1
Char. 148: 0 → 1
Char. 150: 0 → 1
Char. 167: 0 → 1
Char. 176: 0 → 1
Char. 190: 0 → 1
Char. 205: 0 → 1
Char. 208: 0 → 1
Char. 230: 0 → 1
Char. 239: 0 → 1
Char. 260: 2 → 1

**Node 63:**
Char. 73: 0 → 1
Char. 131: 1 → 0
Char. 205: 0 → 1
Char. 273: 0 → 1
Char. 276: 0 → 1

**Node 64:**
Char. 18: 0 → 1
Char. 25: 1 → 0
Char. 37: 0 → 1
Char. 103: 0 → 1
Char. 106: 0 → 1
Char. 107: 0 → 1
Char. 110: 0 → 1
Char. 126: 0 → 1
Char. 150: 0 → 1

**Node 65:**
Char. 186: 0 → 1

**Node 66:**
Char. 148: 0 → 1
Char. 183: 1 → 2
Char. 194: 0 → 1
Char. 201: 1 → 0
Char. 211: 0 → 1
Char. 226: 0 → 1
Char. 240: 1 → 0
Char. 252: 0 → 1

**Node 67:**
Char. 87: 0 → 1
Char. 110: 0 → 1

**Node 68:**
Char. 79: 0 → 1
Char. 133: 0 → 1

**Node 69:**
Char. 100: 0 → 1
Char. 113: 0 → 1

**Node 70:**
Char. 38: 1 → 2
Char. 39: 0 → 1
Char. 50: 1 → 0
Char. 58: 0 → 1
Char. 59: 0 → 1
Char. 60: 0 → 1
Char. 72: 1 → 2
Char. 83: 0 → 1
Char. 85: 1 → 0
Char. 88: 1 → 0
Char. 95: 0 → 1
Char. 104: 0 → 1
Char. 105: 0 → 1
Char. 106: 0 → 2
Char. 107: 0 → 1
Char. 109: 0 → 1
Char. 110: 0 → 1
Char. 146: 1 → 0
Char. 148: 0 → 1
Char. 155: 0 → 2
Char. 183: 1 → 2

**Node 71:**
Char. 33: 1 → 0
Char. 38: 2 → 1
Char. 39: 1 → 0
Char. 42: 0 → 1
Char. 43: 0 → 1
Char. 46: 1 → 0
Char. 49: 1 → 0
Char. 71: 1 → 0
Char. 83: 0 → 2
Char. 101: 0 → 1
Char. 103: 0 → 1
Char. 106: 0 → 1
Char. 161: 0 → 1
Char. 163: 1 → 0
Char. 172: 0 → 2
Char. 174: 0 → 1
Char. 188: 0 → 1
Char. 203: 1 → 2
Char. 212: 0 → 1
Char. 216: 1 → 0
Char. 236: 0 → 1
Char. 238: 0 → 2
Char. 241: 0 → 1
Char. 242: 0 → 1
Char. 273: 0 → 1
Char. 274: 0 → 1
Char. 275: 1 → 0

**Node 72:**
Char. 52: 0 → 1
Char. 75: 0 → 1
Char. 84: 1 → 0
Char. 87: 0 → 1
Char. 110: 0 → 1
Char. 121: 0 → 1
Char. 180: 1 → 0
Char. 230: 0 → 1

**Node 73:**
Char. 29: 1 → 0
Char. 44: 1 → 0
Char. 59: 0 → 1
Char. 60: 0 → 1
Char. 66: 0 → 1
Char. 67: 0 → 1
Char. 72: 1 → 2
Char. 78: 1 → 0
Char. 84: 1 → 0
Char. 88: 1 → 0
Char. 93: 1 → 0
Char. 111: 0 → 1
Char. 123: 1 → 0
Char. 129: 0 → 1
Char. 154: 1 → 0
Char. 169: 1 → 0
Char. 170: 0 → 1
Char. 192: 1 → 0
Char. 197: 0 → 1

**Node 74:**
Char. 83: 0 → 1
Char. 85: 1 → 0
Char. 107: 0 → 1

**Node 75:**
Char. 2: 0 → 1
Char. 6: 0 → 1
Char. 186: 0 → 1
Char. 198: 0 → 1
Char. 200: 0 → 1
Char. 220: 0 → 1
Char. 239: 1 → 0

**Node 76:**
Char. 98: 1 → 0
Char. 113: 0 → 1
Char. 181: 0 → 1
Char. 206: 0 → 2

**Node 77:**
Char. 35: 0 → 1
Char. 102: 0 → 1
Char. 103: 0 → 1
Char. 184: 0 → 1
Char. 201: 0 → 1
Char. 210: 0 → 1
Char. 223: 0 → 1
Char. 229: 0 → 1

**Node 78:**
Char. 17: 0 → 1
Char. 42: 0 → 1
Char. 251: 0 → 1
Char. 264: 0 → 1

**Node 79:**
Char. 41: 0 → 1
Char. 61: 1 → 2
Char. 112: 1 → 0
Char. 138: 0 → 1
Char. 146: 1 → 0
Char. 155: 0 → 1
Char. 192: 1 → 2
Char. 224: 0 → 1
Char. 226: 0 → 1
Char. 227: 0 → 1
Char. 233: 0 → 1
Char. 267: 01 → 2

**Node 80:**
Char. 61: 1 → 3
Char. 90: 0 → 1
Char. 91: 0 → 1
Char. 109: 1 → 0
Char. 155: 0 → 1
Char. 209: 1 → 0

**ANALYSIS 58**
**(ALL TAXA, IMPLIED WEIGHTING, K = 5.25)**

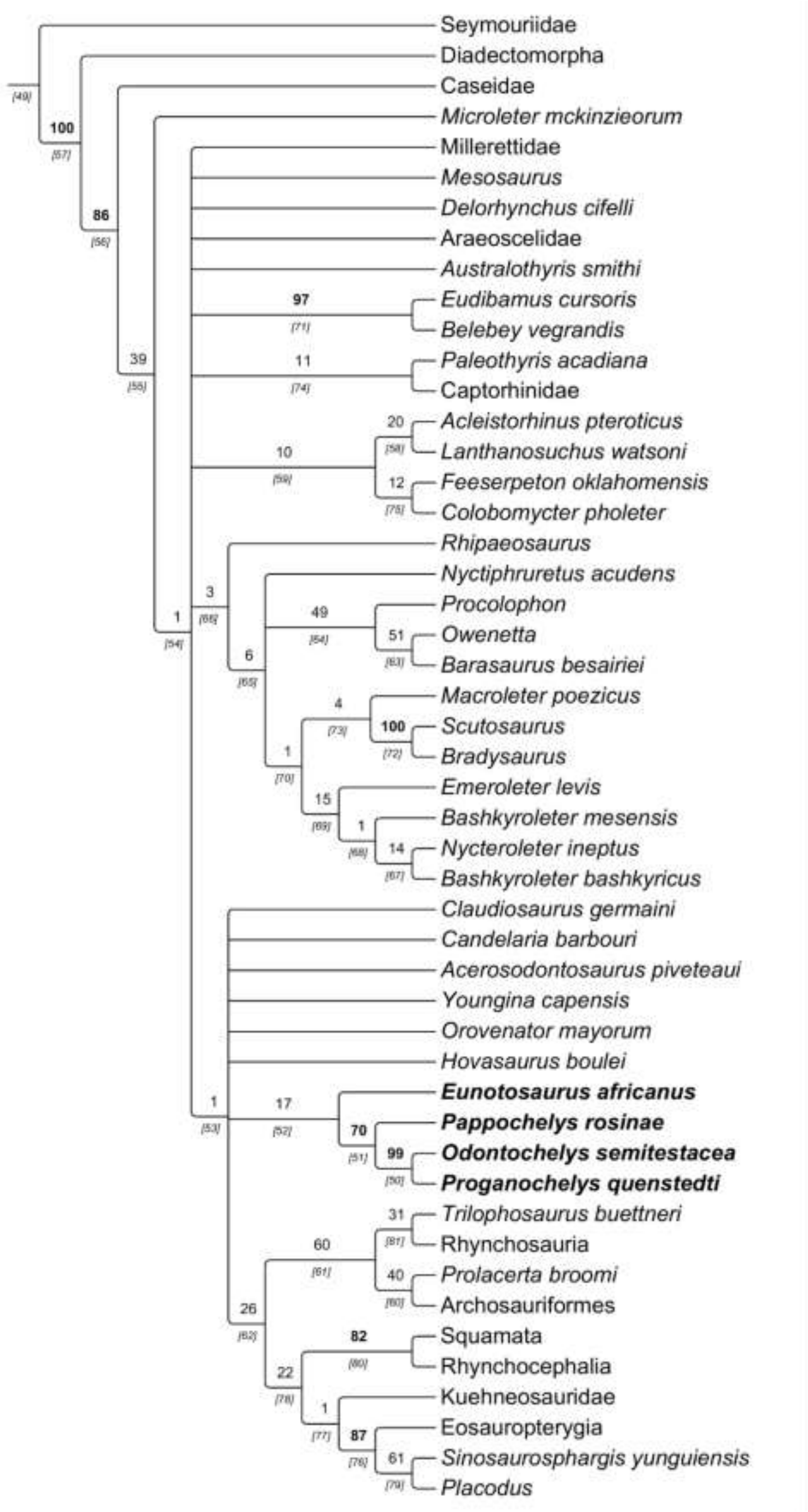

***Proganochelys quenstedti*:**
Char. 8: 0 → 1
Char. 11: 0 → 1
Char. 106: 0 → 1
Char. 108: 1 → 0
Char. 128: 0 → 1
Char. 175: 0 → 1
Char. 202: 1 → 0
Char. 207: 1 → 0
Char. 209: 1 → 0
Char. 244: 0 → 1
Char. 248: 0 → 1
Char. 250: 0 → 1
Char. 252: 1 → 0
Char. 262: 0 → 1

***Pappochelys rosinae*:**
Char. 0: 2 → 0
Char. 1: 0 → 1
Char. 5: 1 → 0
Char. 12: 0 → 1
Char. 41: 0 → 1
Char. 49: 0 → 1
Char. 75: 0 → 1
Char. 169: 1 → 0
Char. 260: 2 → 0

***Odontochelys semitestacea*:**
Char. 43: 0 → 1

***Eunotosaurus africanus*:**
Char. 19: 0 → 1
Char. 43: 0 → 1
Char. 59: 1 → 0
Char. 72: 2 → 1
Char. 76: 0 → 1
Char. 97: 1 → 0
Char. 103: 0 → 1
Char. 153: 1 → 0
Char. 191: 1 → 0
Char. 192: 1 → 0
Char. 202: 1 → 0
Char. 211: 0 → 1
Char. 222: 1 → 0
Char. 237: 1 → 0
Char. 248: 0 → 1
Char. 249: 0 → 2
Char. 250: 0 → 1
Char. 263: 0 → 1
Char. 273: 0 → 1
Char. 274: 0 → 1
Char. 275: 0 → 1
Char. 276: 0 → 1
Char. 277: 0 → 1

***Acerosodontosaurus piveteaui*:**
Char. 78: 1 → 0
Char. 81: 1 → 0
Char. 127: 1 → 0
Char. 128: 0 → 1
Char. 155: 0 → 1
Char. 206: 0 → 2
Char. 208: 0 → 1
Char. 278: 03 → 1

***Acleistorhinus pteroticus*:**
Char. 21: 0 → 1
Char. 146: 1 → 0

**Araeoscelidae:**
Char. 0: 0 → 1
Char. 27: 0 → 1
Char. 28: 0 → 1
Char. 29: 1 → 0
Char. 38: 1 → 0
Char. 40: 2 → 0
Char. 43: 0 → 1
Char. 50: 1 → 0
Char. 57: 1 → 0
Char. 59: 0 → 1
Char. 60: 0 → 1
Char. 67: 0 → 1
Char. 72: 1 → 2
Char. 84: 1 → 0
Char. 89: 0 → 1
Char. 106: 0 → 1
Char. 111: 0 → 1
Char. 116: 1 → 0
Char. 117: 01 → 2
Char. 120: 0 → 1
Char. 154: 1 → 0
Char. 166: 1 → 0
Char. 169: 1 → 0
Char. 170: 0 → 1
Char. 193: 0 → 1
Char. 197: 0 → 1
Char. 222: 0 → 1
Char. 224: 0 → 1
Char. 237: 0 → 1
Char. 239: 0 → 1
Char. 266: 0 → 1
Char. 278: 3 → 1

**Archosauriformes:**
Char. 32: 0 → 1
Char. 94: 1 → 0
Char. 112: 1 → 0
Char. 152: 0 → 1
Char. 154: 01 → 2
Char. 166: 1 → 0
Char. 171: 0 → 1
Char. 185: 0 → 1
Char. 201: 0 → 1
Char. 204: 0 → 1
Char. 218: 0 → 3
Char. 224: 0 → 1
Char. 242: 0 → 1

***Australothyris smithi*:**
Char. 24: 0 → 1
Char. 34: 0 → 1
Char. 55: 1 → 0
Char. 57: 1 → 0
Char. 71: 1 → 0
Char. 79: 0 → 1
Char. 83: 0 → 1
Char. 85: 1 → 0
Char. 98: 1 → 0
Char. 100: 0 → 1
Char. 103: 0 → 1
Char. 110: 0 → 1
Char. 123: 1 → 0
Char. 129: 0 → 1
Char. 131: 0 → 1
Char. 132: 1 → 0
Char. 144: 1 → 0
Char. 147: 0 → 1
Char. 149: 1 → 0
Char. 150: 0 → 1
Char. 192: 1 → 0

***Barasaurus besairiei*:**
Char. 33: 1 → 0

***Bashkyroleter bashkyricus*:**
Char. 275: 1 → 0

***Bashkyroleter mesensis*:**
Char. 169: 1 → 0

***Belebey vegrandis*:**
Char. 154: 1 → 0

***Bradysaurus* spp.:**
Char. 19: 0 → 1
Char. 25: 1 → 0
Char. 73: 0 → 1
Char. 79: 0 → 1
Char. 135: 1 → 0
Char. 249: 0 → 1

***Candelaria barbouri*:**
Char. 0: 1 → 0
Char. 1: 0 → 1
Char. 5: 1 → 0
Char. 8: 0 → 1
Char. 15: 0 → 1
Char. 33: 1 → 2
Char. 49: 0 → 1
Char. 55: 1 → 0
Char. 59: 1 → 0
Char. 62: 1 → 0
Char. 72: 2 → 1
Char. 76: 0 → 1
Char. 79: 0 → 2
Char. 83: 0 → 1
Char. 88: 1 → 0
Char. 92: 1 → 0
Char. 95: 0 → 1
Char. 126: 0 → 1
Char. 132: 1 → 0
Char. 154: 1 → 2
Char. 159: 1 → 0
Char. 169: 1 → 0
Char. 265: 1 → 0
Char. 275: 0 → 1
Char. 276: 0 → 1
Char. 277: 0 → 1

**Captorhinidae:**
Char. 3: 0 → 1
Char. 25: 1 → 0
Char. 26: 0 → 1
Char. 73: 0 → 1
Char. 75: 0 → 1
Char. 83: 0 → 1
Char. 108: 1 → 0
Char. 180: 1 → 0
Char. 183: 1 → 0
Char. 201: 1 → 0
Char. 203: 1 → 2
Char. 216: 1 → 0
Char. 240: 1 → 0

**Caseidae:**
Char. 38: 1 → 0
Char. 46: 1 → 0
Char. 50: 1 → 0
Char. 170: 0 → 1
Char. 194: 0 → 1
Char. 273: 0 → 1
Char. 274: 0 → 1
Char. 278: 3 → 2

***Claudiosaurus germaini*:**
Char. 24: 0 → 1
Char. 27: 0 → 1
Char. 34: 0 → 1
Char. 36: 0 → 1
Char. 56: 0 → 1
Char. 64: 0 → 1
Char. 70: 0 → 1
Char. 84: 1 → 0
Char. 105: 0 → 1
Char. 106: 0 → 1
Char. 117: 01 → 2
Char. 126: 0 → 1
Char. 127: 1 → 0
Char. 130: 0 → 1
Char. 131: 0 → 1
Char. 141: 1 → 0
Char. 144: 1 → 0
Char. 148: 0 → 1
Char. 154: 1 → 0
Char. 166: 1 → 0
Char. 182: 0 → 1
Char. 187: 1 → 0
Char. 190: 0 → 1
Char. 199: 0 → 1
Char. 201: 0 → 1
Char. 203: 1 → 2
Char. 204: 0 → 1
Char. 222: 1 → 0
Char. 234: 1 → 0
Char. 267: 0 → 1
Char. 272: 2 → 1

***Colobomycter pholeter*:**
Char. 21: 0 → 1
Char. 25: 1 → 0
Char. 84: 1 → 0
Char. 154: 1 → 0
Char. 167: 0 → 1
Char. 267: 0 → 1

***Delorhynchus cifelli*:**
Char. 18: 0 → 1
Char. 20: 0 → 1
Char. 21: 0 → 1
Char. 24: 0 → 1
Char. 26: 0 → 1
Char. 28: 0 → 1
Char. 33: 0 → 2
Char. 39: 0 → 1
Char. 52: 0 → 1
Char. 100: 0 → 1
Char. 111: 0 → 1
Char. 116: 1 → 0
Char. 119: 1 → 0
Char. 131: 0 → 1
Char. 147: 0 → 1
Char. 156: 1 → 0
Char. 167: 0 → 1
Char. 189: 0 → 1
Char. 191: 0 → 1
Char. 204: 0 → 2
Char. 267: 0 → 1

**Diadectomorpha:**
Char. 0: 0 → 1
Char. 64: 0 → 1
Char. 70: 0 → 1
Char. 122: 0 → 1
Char. 123: 1 → 0
Char. 146: 1 → 0
Char. 275: 1 → 0
Char. 278: 3 → 0

***Emeroleter levis*:**
Char. 51: 1 → 0

**Eosauropterygia:**
Char. 166: 1 → 0
Char. 174: 0 → 1
Char. 178: 0 → 1
Char. 194: 0 → 2
Char. 272: 1 → 0

***Eudibamus cursoris*:**
Char. 154: 1 → 2

***Feeserpeton oklahomensis*:**
Char. 51: 0 → 1
Char. 157: 0 → 1
Char. 158: 0 → 1

***Hovasaurus boulei*:**
Char. 41: 0 → 1
Char. 43: 0 → 1
Char. 55: 1 → 0
Char. 72: 2 → 1
Char. 77: 0 → 2
Char. 78: 1 → 0
Char. 79: 0 → 1
Char. 93: 1 → 0
Char. 113: 0 → 1
Char. 138: 0 → 1
Char. 141: 1 → 0
Char. 146: 1 → 0
Char. 204: 0 → 2

Char. 206: 0 → 2
Char. 215: 0 → 1
Char. 219: 1 → 0
Char. 224: 0 → 1

**Kuehneosauridae:**
Char. 7: 0 → 1
Char. 24: 0 → 1
Char. 27: 0 → 1
Char. 43: 1 → 0
Char. 79: 0 → 2
Char. 107: 1 → 0
Char. 140: 1 → 0
Char. 147: 1 → 0
Char. 148: 1 → 0
Char. 245: 0 → 1
Char. 278: 0 → 3

***Lanthanosuchus watsoni*:**
Char. 25: 1 → 0
Char. 51: 0 → 1
Char. 76: 0 → 1
Char. 86: 0 → 1
Char. 98: 1 → 0
Char. 138: 0 → 1
Char. 144: 1 → 0
Char. 154: 1 → 2

***Macroleter poezicus*:**
Char. 9: 0 → 1
Char. 26: 0 → 1
Char. 66: 1 → 2
Char. 126: 0 → 1
Char. 134: 0 → 2
Char. 140: 0 → 1
Char. 146: 1 → 0
Char. 169: 1 → 0

***Mesosaurus* spp.:**
Char. 0: 0 → 1
Char. 2: 0 → 1
Char. 6: 0 → 1
Char. 8: 0 → 1
Char. 9: 0 → 1
Char. 13: 0 → 1
Char. 19: 0 → 1
Char. 26: 0 → 1
Char. 29: 1 → 0
Char. 33: 0 → 1
Char. 38: 1 → 0
Char. 41: 0 → 1
Char. 50: 1 → 0
Char. 67: 0 → 1
Char. 76: 0 → 1
Char. 83: 0 → 1
Char. 84: 1 → 0
Char. 85: 1 → 0
Char. 94: 0 → 1
Char. 107: 0 → 1
Char. 109: 0 → 1
Char. 111: 0 → 1
Char. 115: 0 → 1
Char. 146: 1 → 0
Char. 148: 0 → 1

Char. 149: 1 → 0
Char. 164: 0 → 1
Char. 166: 1 → 0
Char. 167: 0 → 1
Char. 176: 0 → 1
Char. 183: 1 → 0
Char. 184: 0 → 1
Char. 192: 1 → 0
Char. 199: 0 → 1
Char. 202: 0 → 1
Char. 204: 0 → 2
Char. 206: 0 → 1
Char. 207: 0 → 1
Char. 209: 0 → 1
Char. 217: 0 → 1
Char. 219: 0 → 1
Char. 220: 0 → 1
Char. 231: 0 → 1
Char. 240: 1 → 0
Char. 260: 2 → 0
Char. 272: 2 → 0
Char. 278: 3 → 0

***Microleter mckinzieorum*:**
Char. 0: 0 → 1
Char. 18: 0 → 1
Char. 39: 0 → 1
Char. 57: 1 → 0
Char. 70: 0 → 1
Char. 76: 0 → 1
Char. 83: 0 → 1
Char. 94: 0 → 1
Char. 106: 0 → 1
Char. 110: 0 → 1
Char. 278: 3 → 1

**Millerettidae:**
Char. 24: 0 → 1
Char. 25: 1 → 0
Char. 44: 1 → 0
Char. 56: 0 → 1
Char. 57: 1 → 0
Char. 66: 0 → 2
Char. 78: 1 → 0
Char. 80: 1 → 0
Char. 84: 1 → 2
Char. 88: 1 → 0
Char. 96: 1 → 0
Char. 121: 0 → 1
Char. 124: 0 → 1
Char. 127: 0 → 1
Char. 135: 0 → 1
Char. 145: 0 → 1
Char. 166: 1 → 0
Char. 180: 1 → 0
Char. 192: 1 → 0
Char. 202: 0 → 1
Char. 211: 0 → 1
Char. 230: 0 → 1
Char. 248: 0 → 1
Char. 252: 0 → 1
Char. 253: 0 → 1

***Nycteroleter ineptus*:**
Char. 278: 0 → 3

***Nyctiphruretus acudens*:**
Char. 0: 0 → 1
Char. 21: 0 → 1
Char. 33: 1 → 2
Char. 41: 0 → 1
Char. 66: 1 → 2
Char. 81: 1 → 0
Char. 83: 0 → 1
Char. 84: 1 → 2
Char. 94: 0 → 1
Char. 166: 1 → 0
Char. 224: 0 → 1
Char. 226: 1 → 0
Char. 266: 0 → 1
Char. 272: 2 → 1
Char. 276: 0 → 1

***Orovenator mayorum*:**
Char. 8: 0 → 1
Char. 24: 0 → 1
Char. 33: 1 → 0
Char. 36: 0 → 1
Char. 50: 0 → 1
Char. 92: 1 → 0
Char. 94: 1 → 0
Char. 135: 1 → 0
Char. 159: 1 → 0
Char. 160: 0 → 1
Char. 165: 0 → 1
Char. 278: 03 → 1

***Owenetta* spp.:**
Char. 142: 1 → 0
Char. 169: 1 → 0

***Paleothyris acadiana*:**
Char. 38: 1 → 0
Char. 50: 1 → 0
Char. 66: 1 → 2
Char. 102: 0 → 1
Char. 146: 1 → 0
Char. 237: 0 → 1
Char. 239: 0 → 1

***Placodus* spp.:**
Char. 0: 1 → 2
Char. 9: 0 → 1
Char. 12: 0 → 1
Char. 13: 0 → 1
Char. 19: 0 → 1
Char. 31: 0 → 1
Char. 46: 1 → 0
Char. 57: 0 → 1
Char. 78: 1 → 0
Char. 93: 1 → 0
Char. 102: 1 → 2
Char. 109: 1 → 0
Char. 140: 1 → 0
Char. 155: 0 → 1
Char. 163: 1 → 0
Char. 164: 0 → 1

***Procolophon* spp.:**
Char. 41: 0 → 1
Char. 69: 0 → 1
Char. 79: 0 → 1
Char. 83: 0 → 1
Char. 86: 0 → 1
Char. 88: 0 → 1
Char. 101: 0 → 1
Char. 117: 1 → 0
Char. 141: 0 → 1
Char. 149: 1 → 0
Char. 203: 1 → 2
Char. 204: 0 → 1
Char. 230: 0 → 1
Char. 237: 0 → 1
Char. 238: 0 → 1
Char. 272: 2 → 1
Char. 278: 3 → 0

***Prolacerta broomi*:**
Char. 58: 1 → 0
Char. 66: 1 → 0
Char. 67: 1 → 0
Char. 80: 1 → 0
Char. 139: 1 → 0
Char. 147: 1 → 0
Char. 192: 1 → 0
Char. 203: 1 → 2
Char. 206: 0 → 12

***Rhipaeosaurus* spp.:**
Char. 172: 0 → 1
Char. 277: 0 → 1

**Rhynchocephalia:**
Char. 0: 1 → 2
Char. 24: 0 → 1
Char. 77: 0 → 1
Char. 88: 1 → 0
Char. 94: 1 → 0
Char. 139: 1 → 0
Char. 167: 1 → 0
Char. 205: 1 → 0

**Rhynchosauria:**
Char. 0: 1 → 0
Char. 7: 0 → 1
Char. 9: 0 → 1
Char. 26: 0 → 1
Char. 44: 1 → 0
Char. 68: 0 → 1
Char. 99: 1 → 0
Char. 150: 1 → 0
Char. 160: 0 → 1
Char. 161: 0 → 1
Char. 171: 0 → 2
Char. 223: 0 → 1
Char. 224: 0 → 1
Char. 241: 0 → 1

***Scutosaurus* spp.:**
Char. 175: 0 → 1
Char. 190: 0 → 1
Char. 218: 0 → 1
Char. 243: 0 → 2

Char. 244: 0 → 1
Char. 251: 0 → 1

***Sinosaurosphargis yunguiensis*:**
Char. 8: 0 → 1
Char. 30: 0 → 1
Char. 53: 0 → 1
Char. 89: 1 → 0
Char. 127: 1 → 0
Char. 150: 1 → 0
Char. 154: 0 → 2
Char. 167: 1 → 0
Char. 253: 0 → 1
Char. 255: 0 → 1

**Squamata:**
Char. 45: 0 → 1
Char. 79: 0 → 2
Char. 80: 1 → 0
Char. 92: 1 → 0
Char. 109: 1 → 0
Char. 160: 0 → 1
Char. 200: 0 → 1
Char. 245: 0 → 1

***Trilophosaurus buettneri*:**
Char. 5: 1 → 0
Char. 11: 0 → 1
Char. 55: 1 → 0
Char. 93: 1 → 0
Char. 104: 0 → 1
Char. 106: 0 → 1
Char. 113: 0 → 1
Char. 122: 0 → 1
Char. 136: 1 → 0
Char. 144: 1 → 0
Char. 157: 0 → 1
Char. 159: 1 → 0
Char. 177: 0 → 12
Char. 194: 0 → 1
Char. 203: 1 → 2
Char. 207: 1 → 0
Char. 208: 1 → 0
Char. 272: 1 → 0

***Youngina capensis*:**
Char. 5: 1 → 0
Char. 21: 0 → 1
Char. 25: 0 → 1
Char. 27: 0 → 1
Char. 38: 1 → 0
Char. 43: 0 → 1
Char. 44: 1 → 0
Char. 56: 0 → 1
Char. 75: 0 → 1
Char. 84: 1 → 0
Char. 92: 1 → 0
Char. 94: 1 → 0
Char. 134: 0 → 1
Char. 154: 1 → 0
Char. 163: 1 → 0
Char. 170: 0 → 1
Char. 211: 0 → 1

Char. 215: 0 → 1
Char. 224: 0 → 1
Char. 239: 0 → 1
Char. 267: 0 → 1

**Node 50:**
Char. 46: 1 → 0
Char. 88: 1 → 0
Char. 89: 1 → 0
Char. 93: 1 → 0
Char. 176: 0 → 1
Char. 195: 0 → 2
Char. 198: 0 → 1
Char. 246: 1 → 2
Char. 259: 0 → 1
Char. 265: 1 → 0
Char. 270: 0 → 1

**Node 51:**
Char. 65: 0 → 1
Char. 131: 0 → 1
Char. 184: 0 → 1
Char. 205: 0 → 1
Char. 210: 0 → 1
Char. 219: 1 → 0
Char. 241: 0 → 1
Char. 246: 0 → 1
Char. 254: 0 → 1
Char. 255: 0 → 1
Char. 256: 0 → 1
Char. 267: 0 → 1
Char. 268: 0 → 1
Char. 269: 0 → 1

**Node 52:**
Char. 0: 1 → 2
Char. 15: 0 → 1
Char. 21: 0 → 1
Char. 33: 1 → 2
Char. 44: 1 → 0
Char. 62: 1 → 0
Char. 64: 0 → 1
Char. 84: 1 → 2
Char. 130: 0 → 1
Char. 134: 0 → 2
Char. 147: 0 → 1
Char. 148: 0 → 1
Char. 152: 0 → 1
Char. 155: 0 → 1
Char. 158: 0 → 1
Char. 161: 0 → 1
Char. 174: 0 → 1
Char. 181: 0 → 1
Char. 203: 1 → 2
Char. 204: 0 → 2
Char. 247: 0 → 1
Char. 251: 0 → 1
Char. 252: 0 → 1
Char. 253: 0 → 2

**Node 53:**
Char. 0: 0 → 1
Char. 20: 0 → 1
Char. 25: 1 → 0
Char. 29: 1 → 0
Char. 33: 0 → 1
Char. 40: 2 → 0
Char. 50: 1 → 0
Char. 57: 1 → 0
Char. 59: 0 → 1
Char. 62: 0 → 1
Char. 67: 0 → 1
Char. 72: 1 → 2
Char. 89: 0 → 1
Char. 94: 0 → 1
Char. 109: 0 → 1
Char. 111: 0 → 1
Char. 120: 0 → 1
Char. 127: 0 → 1
Char. 129: 0 → 1
Char. 135: 0 → 1
Char. 136: 0 → 1
Char. 139: 0 → 1
Char. 141: 0 → 1
Char. 179: 0 → 1
Char. 187: 0 → 1
Char. 188: 0 → 1
Char. 191: 0 → 1
Char. 193: 0 → 1
Char. 196: 0 → 1
Char. 201: 1 → 0
Char. 202: 0 → 1
Char. 207: 0 → 1
Char. 209: 0 → 1
Char. 219: 0 → 1
Char. 222: 0 → 1
Char. 237: 0 → 1
Char. 265: 0 → 1
Char. 266: 0 → 1
Char. 275: 1 → 0

**Node 54:**
Char. 132: 0 → 1
Char. 166: 0 → 1
Char. 276: 1 → 0

**Node 55:**
Char. 29: 0 → 1
Char. 84: 0 → 1
Char. 88: 0 → 1
Char. 169: 0 → 1

**Node 56:**
Char. 72: 0 → 1
Char. 81: 0 → 1
Char. 93: 0 → 1
Char. 97: 0 → 1
Char. 104: 1 → 0
Char. 144: 0 → 1
Char. 173: 0 → 1
Char. 183: 0 → 1

**Node 58:**
Char. 95: 0 → 1
Char. 113: 0 → 1
Char. 114: 0 → 1

**Node 59:**
Char. 20: 0 → 1
Char. 47: 0 → 1
Char. 79: 0 → 1
Char. 110: 0 → 1
Char. 131: 0 → 1
Char. 137: 0 → 1
Char. 140: 0 → 2
Char. 147: 0 → 1

**Node 60:**
Char. 19: 0 → 1
Char. 92: 1 → 0

**Node 61:**
Char. 4: 0 → 1
Char. 15: 0 → 1
Char. 29: 0 → 1
Char. 213: 0 → 1
Char. 226: 0 → 2
Char. 228: 0 → 1
Char. 275: 0 → 1

**Node 62:**
Char. 43: 0 → 1
Char. 58: 0 → 1
Char. 61: 0 → 1
Char. 66: 0 → 1
Char. 69: 0 → 1
Char. 107: 0 → 1
Char. 126: 0 → 1
Char. 131: 0 → 1
Char. 134: 0 → 1
Char. 140: 0 → 1
Char. 147: 0 → 1
Char. 148: 0 → 1
Char. 150: 0 → 1
Char. 167: 0 → 1
Char. 176: 0 → 1
Char. 190: 0 → 1
Char. 205: 0 → 1
Char. 208: 0 → 1
Char. 230: 0 → 1
Char. 239: 0 → 1
Char. 260: 2 → 1

**Node 63:**
Char. 73: 0 → 1
Char. 131: 1 → 0
Char. 205: 0 → 1
Char. 273: 0 → 1
Char. 276: 0 → 1

**Node 64:**
Char. 18: 0 → 1
Char. 25: 1 → 0
Char. 37: 0 → 1
Char. 103: 0 → 1
Char. 106: 0 → 1
Char. 107: 0 → 1
Char. 110: 0 → 1
Char. 126: 0 → 1
Char. 150: 0 → 1

**Node 65:**
Char. 186: 0 → 1

**Node 66:**
Char. 148: 0 → 1
Char. 183: 1 → 2
Char. 194: 0 → 1
Char. 201: 1 → 0
Char. 211: 0 → 1
Char. 226: 0 → 1
Char. 240: 1 → 0
Char. 252: 0 → 1

**Node 67:**
Char. 87: 0 → 1
Char. 110: 0 → 1

**Node 68:**
Char. 25: 1 → 0
Char. 76: 1 → 0

**Node 69:**
Char. 79: 0 → 1
Char. 133: 0 → 1

**Node 70:**
Char. 100: 0 → 1
Char. 113: 0 → 1

**Node 71:**
Char. 38: 1 → 2
Char. 39: 0 → 1
Char. 50: 1 → 0
Char. 58: 0 → 1
Char. 59: 0 → 1
Char. 60: 0 → 1
Char. 72: 1 → 2
Char. 83: 0 → 1
Char. 85: 1 → 0
Char. 88: 1 → 0
Char. 95: 0 → 1
Char. 104: 0 → 1
Char. 105: 0 → 1
Char. 106: 0 → 2
Char. 107: 0 → 1
Char. 109: 0 → 1
Char. 110: 0 → 1
Char. 146: 1 → 0
Char. 148: 0 → 1
Char. 155: 0 → 2
Char. 183: 1 → 2

**Node 72:**
Char. 33: 1 → 0
Char. 38: 2 → 1
Char. 39: 1 → 0
Char. 42: 0 → 1
Char. 43: 0 → 1
Char. 46: 1 → 0
Char. 49: 1 → 0
Char. 71: 1 → 0
Char. 83: 0 → 2
Char. 101: 0 → 1
Char. 103: 0 → 1
Char. 106: 0 → 1
Char. 161: 0 → 1
Char. 163: 1 → 0
Char. 172: 0 → 2
Char. 174: 0 → 1
Char. 188: 0 → 1
Char. 203: 1 → 2
Char. 212: 0 → 1
Char. 216: 1 → 0
Char. 236: 0 → 1
Char. 238: 0 → 2
Char. 241: 0 → 1
Char. 242: 0 → 1
Char. 273: 0 → 1
Char. 274: 0 → 1
Char. 275: 1 → 0

**Node 73:**
Char. 52: 0 → 1
Char. 75: 0 → 1
Char. 84: 1 → 0
Char. 87: 0 → 1
Char. 110: 0 → 1
Char. 121: 0 → 1
Char. 180: 1 → 0
Char. 230: 0 → 1

**Node 74:**
Char. 29: 1 → 0
Char. 44: 1 → 0
Char. 59: 0 → 1
Char. 60: 0 → 1
Char. 66: 0 → 1
Char. 67: 0 → 1
Char. 72: 1 → 2
Char. 78: 1 → 0
Char. 84: 1 → 0
Char. 88: 1 → 0
Char. 93: 1 → 0
Char. 111: 0 → 1
Char. 123: 1 → 0
Char. 129: 0 → 1
Char. 154: 1 → 0
Char. 169: 1 → 0
Char. 170: 0 → 1
Char. 192: 1 → 0
Char. 197: 0 → 1

**Node 75:**
Char. 83: 0 → 1
Char. 85: 1 → 0
Char. 107: 0 → 1

**Node 76:**
Char. 2: 0 → 1
Char. 6: 0 → 1
Char. 186: 0 → 1
Char. 198: 0 → 1
Char. 200: 0 → 1
Char. 220: 0 → 1
Char. 239: 1 → 0

**Node 77:**
Char. 98: 1 → 0
Char. 113: 0 → 1
Char. 181: 0 → 1
Char. 206: 0 → 2

**Node 78:**
Char. 35: 0 → 1
Char. 102: 0 → 1
Char. 103: 0 → 1
Char. 184: 0 → 1
Char. 201: 0 → 1
Char. 210: 0 → 1
Char. 223: 0 → 1
Char. 229: 0 → 1

**Node 79:**
Char. 17: 0 → 1
Char. 42: 0 → 1
Char. 251: 0 → 1
Char. 264: 0 → 1

**Node 80:**
Char. 41: 0 → 1
Char. 61: 1 → 2
Char. 112: 1 → 0
Char. 138: 0 → 1
Char. 146: 1 → 0
Char. 155: 0 → 1
Char. 192: 1 → 2
Char. 224: 0 → 1
Char. 226: 0 → 1
Char. 227: 0 → 1
Char. 233: 0 → 1
Char. 267: 01 → 2

**Node 81:**
Char. 61: 1 → 3
Char. 90: 0 → 1
Char. 91: 0 → 1
Char. 109: 1 → 0
Char. 155: 0 → 1
Char. 209: 1 → 0

**ANALYSIS 59**
**(ALL TAXA, IMPLIED WEIGHTING, K = 5.375)**

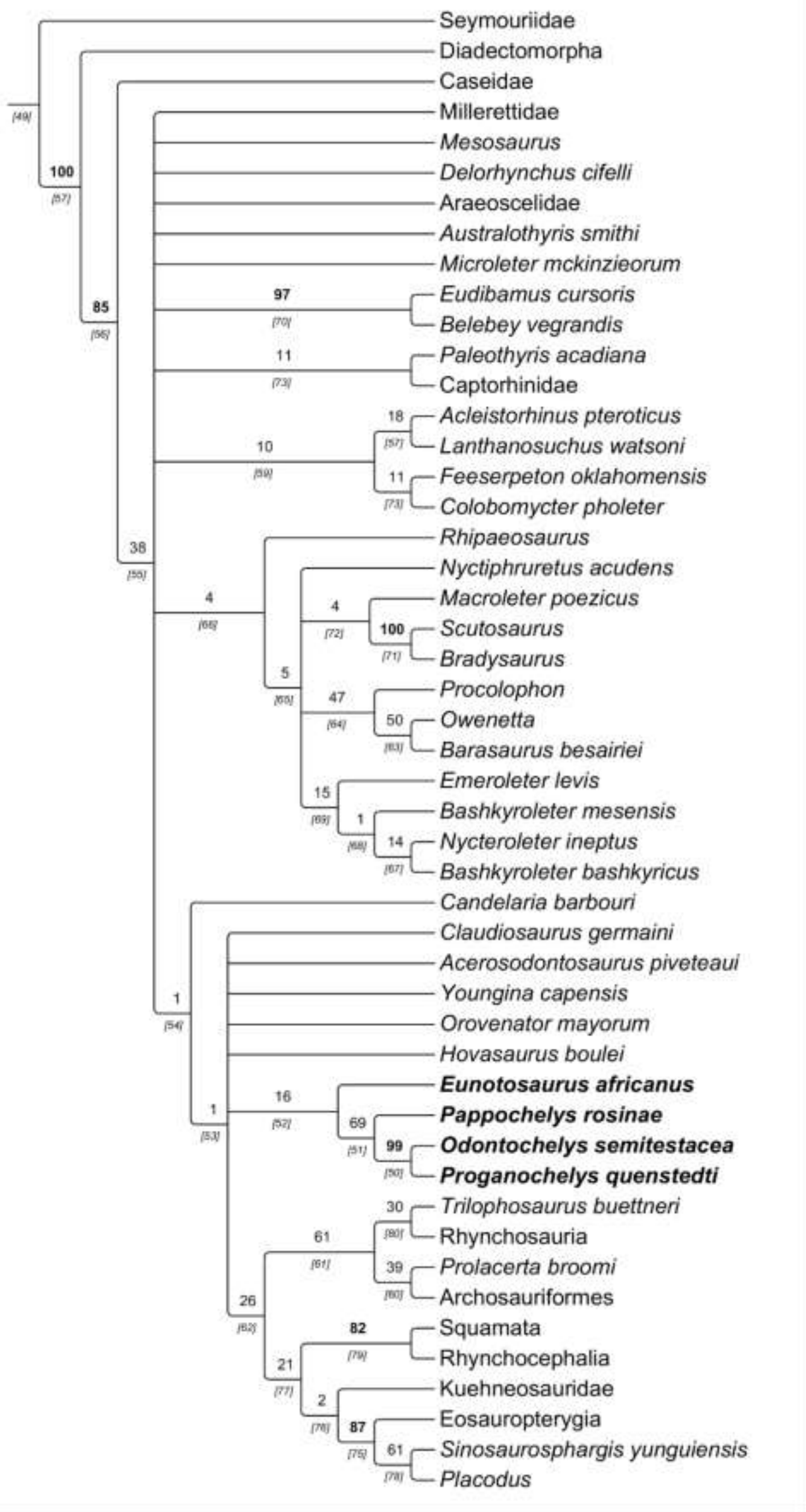

***Proganochelys quenstedti*:**
Char. 8: 0 → 1
Char. 11: 0 → 1
Char. 106: 0 → 1
Char. 108: 1 → 0
Char. 128: 0 → 1
Char. 175: 0 → 1
Char. 202: 1 → 0
Char. 207: 1 → 0
Char. 209: 1 → 0
Char. 244: 0 → 1
Char. 248: 0 → 1
Char. 250: 0 → 1
Char. 252: 1 → 0
Char. 262: 0 → 1

***Pappochelys rosinae*:**
Char. 0: 2 → 0
Char. 1: 0 → 1
Char. 5: 1 → 0
Char. 12: 0 → 1
Char. 41: 0 → 1
Char. 48: 0 → 1
Char. 49: 0 → 1
Char. 75: 0 → 1
Char. 169: 1 → 0
Char. 260: 2 → 0

***Eunotosaurus africanus*:**
Char. 19: 0 → 1
Char. 59: 1 → 0
Char. 60: 1 → 0
Char. 72: 2 → 1
Char. 76: 0 → 1
Char. 97: 1 → 0
Char. 103: 0 → 1
Char. 153: 1 → 0
Char. 191: 1 → 0
Char. 192: 1 → 0
Char. 202: 1 → 0
Char. 211: 0 → 1
Char. 222: 1 → 0
Char. 237: 1 → 0
Char. 248: 0 → 1
Char. 249: 0 → 2
Char. 250: 0 → 1
Char. 263: 0 → 1
Char. 273: 0 → 1
Char. 274: 0 → 1
Char. 275: 0 → 1
Char. 276: 0 → 1
Char. 277: 0 → 1
Char. 278: 0 → 3

***Acerosodontosaurus piveteaui*:**
Char. 78: 1 → 0
Char. 81: 1 → 0
Char. 127: 1 → 0
Char. 128: 0 → 1
Char. 155: 0 → 1
Char. 206: 0 → 2
Char. 208: 0 → 1
Char. 278: 0 → 1

***Acleistorhinus pteroticus*:**
Char. 21: 0 → 1
Char. 146: 1 → 0

**Araeoscelidae:**
Char. 0: 0 → 1
Char. 23: 0 → 1
Char. 27: 0 → 1
Char. 28: 0 → 1
Char. 29: 1 → 0
Char. 38: 1 → 0
Char. 40: 2 → 0
Char. 43: 0 → 1
Char. 48: 0 → 1
Char. 50: 1 → 0
Char. 57: 1 → 0
Char. 59: 0 → 1
Char. 60: 0 → 1
Char. 67: 0 → 1
Char. 72: 1 → 2
Char. 84: 1 → 0
Char. 89: 0 → 1
Char. 106: 0 → 1
Char. 111: 0 → 1
Char. 112: 0 → 1
Char. 116: 1 → 0
Char. 117: 01 → 2
Char. 120: 0 → 1
Char. 154: 1 → 0
Char. 169: 1 → 0
Char. 170: 0 → 1
Char. 193: 0 → 1
Char. 197: 0 → 1
Char. 222: 0 → 1
Char. 224: 0 → 1
Char. 237: 0 → 1
Char. 239: 0 → 1
Char. 266: 0 → 1
Char. 278: 3 → 1

**Archosauriformes:**
Char. 32: 0 → 1
Char. 94: 1 → 0
Char. 112: 1 → 0
Char. 152: 0 → 1
Char. 154: 01 → 2
Char. 166: 1 → 0
Char. 171: 0 → 1
Char. 185: 0 → 1
Char. 201: 0 → 1
Char. 204: 0 → 1
Char. 218: 0 → 3
Char. 224: 0 → 1
Char. 242: 0 → 1

***Australothyris smithi*:**
Char. 23: 0 → 1
Char. 24: 0 → 1
Char. 34: 0 → 1
Char. 55: 1 → 0
Char. 57: 1 → 0
Char. 71: 1 → 0
Char. 79: 0 → 1
Char. 83: 0 → 1
Char. 85: 1 → 0
Char. 98: 1 → 0
Char. 100: 0 → 1
Char. 103: 0 → 1
Char. 110: 0 → 1
Char. 112: 0 → 1
Char. 123: 1 → 0
Char. 129: 0 → 1
Char. 131: 0 → 1
Char. 132: 1 → 0
Char. 144: 1 → 0
Char. 147: 0 → 1
Char. 149: 1 → 0
Char. 150: 0 → 1
Char. 192: 1 → 0

***Barasaurus besairiei*:**
Char. 33: 1 → 0

***Bashkyroleter bashkyricus*:**
Char. 275: 1 → 0

***Bashkyroleter mesensis*:**
Char. 169: 1 → 0

***Belebey vegrandis*:**
Char. 154: 1 → 0

***Bradysaurus* spp.:**
Char. 19: 0 → 1
Char. 25: 1 → 0
Char. 73: 0 → 1
Char. 79: 0 → 1
Char. 135: 1 → 0
Char. 249: 0 → 1

***Candelaria barbouri*:**
Char. 1: 0 → 1
Char. 8: 0 → 1
Char. 15: 0 → 1
Char. 33: 1 → 2
Char. 49: 0 → 1
Char. 55: 1 → 0
Char. 76: 0 → 1
Char. 79: 0 → 2
Char. 83: 0 → 1
Char. 88: 1 → 0
Char. 95: 0 → 1
Char. 126: 0 → 1
Char. 132: 1 → 0
Char. 154: 1 → 2
Char. 169: 1 → 0
Char. 276: 0 → 1
Char. 277: 0 → 1

**Captorhinidae:**
Char. 3: 0 → 1
Char. 23: 0 → 1
Char. 25: 1 → 0
Char. 26: 0 → 1
Char. 73: 0 → 1
Char. 75: 0 → 1
Char. 83: 0 → 1
Char. 108: 1 → 0
Char. 180: 1 → 0
Char. 183: 1 → 0
Char. 201: 1 → 0
Char. 203: 1 → 2
Char. 216: 1 → 0
Char. 240: 1 → 0

**Caseidae:**
Char. 24: 0 → 1
Char. 25: 1 → 0
Char. 36: 0 → 1
Char. 38: 1 → 0
Char. 46: 1 → 0
Char. 50: 1 → 0
Char. 56: 0 → 1
Char. 170: 0 → 1
Char. 194: 0 → 1
Char. 273: 0 → 1
Char. 274: 0 → 1
Char. 278: 3 → 2

***Claudiosaurus germaini*:**
Char. 24: 0 → 1
Char. 27: 0 → 1
Char. 34: 0 → 1
Char. 36: 0 → 1
Char. 48: 0 → 1
Char. 56: 0 → 1
Char. 64: 0 → 1
Char. 70: 0 → 1
Char. 84: 1 → 0
Char. 105: 0 → 1
Char. 106: 0 → 1
Char. 117: 01 → 2
Char. 126: 0 → 1
Char. 127: 1 → 0
Char. 130: 0 → 1
Char. 131: 0 → 1
Char. 141: 1 → 0
Char. 144: 1 → 0
Char. 148: 0 → 1
Char. 166: 1 → 0
Char. 182: 0 → 1
Char. 187: 1 → 0
Char. 190: 0 → 1
Char. 199: 0 → 1
Char. 201: 0 → 1
Char. 203: 1 → 2
Char. 204: 0 → 1
Char. 222: 1 → 0
Char. 234: 1 → 0
Char. 272: 2 → 1

***Colobomycter pholeter*:**
Char. 21: 0 → 1
Char. 25: 1 → 0
Char. 84: 1 → 0
Char. 154: 1 → 0
Char. 167: 0 → 1
Char. 267: 0 → 1

***Delorhynchus cifelli*:**
Char. 5: 0 → 1
Char. 18: 0 → 1
Char. 20: 0 → 1
Char. 21: 0 → 1
Char. 24: 0 → 1
Char. 26: 0 → 1
Char. 28: 0 → 1
Char. 33: 0 → 2
Char. 39: 0 → 1
Char. 48: 0 → 1
Char. 52: 0 → 1
Char. 100: 0 → 1
Char. 111: 0 → 1
Char. 116: 1 → 0
Char. 119: 1 → 0
Char. 131: 0 → 1
Char. 147: 0 → 1
Char. 156: 1 → 0
Char. 159: 0 → 1
Char. 167: 0 → 1
Char. 189: 0 → 1
Char. 191: 0 → 1
Char. 204: 0 → 2
Char. 267: 0 → 1

**Diadectomorpha:**
Char. 0: 0 → 1
Char. 64: 0 → 1
Char. 70: 0 → 1
Char. 122: 0 → 1
Char. 123: 1 → 0
Char. 146: 1 → 0
Char. 275: 1 → 0
Char. 278: 3 → 0

***Emeroleter levis*:**
Char. 0: 0 → 2
Char. 51: 1 → 0

**Eosauropterygia:**
Char. 166: 1 → 0
Char. 174: 0 → 1
Char. 178: 0 → 1
Char. 194: 0 → 2
Char. 272: 1 → 0

***Eudibamus cursoris*:**
Char. 154: 1 → 2

***Feeserpeton oklahomensis*:**
Char. 51: 0 → 1
Char. 157: 0 → 1
Char. 158: 0 → 1

***Hovasaurus boulei*:**
Char. 41: 0 → 1
Char. 48: 0 → 1
Char. 55: 1 → 0
Char. 60: 1 → 0
Char. 72: 2 → 1
Char. 77: 0 → 2
Char. 78: 1 → 0
Char. 79: 0 → 1
Char. 93: 1 → 0
Char. 113: 0 → 1
Char. 138: 0 → 1
Char. 141: 1 → 0
Char. 146: 1 → 0

Char. 204: 0 → 2
Char. 206: 0 → 2
Char. 215: 0 → 1
Char. 219: 1 → 0
Char. 224: 0 → 1
Char. 278: 0 → 3

**Kuehneosauridae:**
Char. 7: 0 → 1
Char. 24: 0 → 1
Char. 27: 0 → 1
Char. 43: 1 → 0
Char. 79: 0 → 2
Char. 107: 1 → 0
Char. 140: 1 → 0
Char. 147: 1 → 0
Char. 148: 1 → 0
Char. 245: 0 → 1
Char. 278: 0 → 3

***Lanthanosuchus watsoni*:**
Char. 25: 1 → 0
Char. 51: 0 → 1
Char. 76: 0 → 1
Char. 86: 0 → 1
Char. 98: 1 → 0
Char. 138: 0 → 1
Char. 144: 1 → 0
Char. 154: 1 → 2

***Macroleter poezicus*:**
Char. 9: 0 → 1
Char. 26: 0 → 1
Char. 66: 1 → 2
Char. 126: 0 → 1
Char. 134: 0 → 2
Char. 140: 0 → 1
Char. 146: 1 → 0
Char. 147: 1 → 2
Char. 154: 1 → 0
Char. 169: 1 → 0
Char. 278: 3 → 0

***Mesosaurus* spp.:**
Char. 0: 0 → 1
Char. 2: 0 → 1
Char. 6: 0 → 1
Char. 8: 0 → 1
Char. 9: 0 → 1
Char. 13: 0 → 1
Char. 19: 0 → 1
Char. 23: 0 → 1
Char. 26: 0 → 1
Char. 29: 1 → 0
Char. 33: 0 → 1
Char. 38: 1 → 0
Char. 41: 0 → 1
Char. 48: 0 → 1
Char. 50: 1 → 0
Char. 67: 0 → 1
Char. 76: 0 → 1
Char. 83: 0 → 1
Char. 84: 1 → 0
Char. 85: 1 → 0
Char. 94: 0 → 1
Char. 107: 0 → 1
Char. 109: 0 → 1
Char. 111: 0 → 1
Char. 115: 0 → 1
Char. 146: 1 → 0
Char. 148: 0 → 1
Char. 149: 1 → 0
Char. 164: 0 → 1
Char. 167: 0 → 1
Char. 176: 0 → 1
Char. 183: 1 → 0
Char. 184: 0 → 1
Char. 192: 1 → 0
Char. 199: 0 → 1
Char. 202: 0 → 1
Char. 204: 0 → 2
Char. 206: 0 → 1
Char. 207: 0 → 1
Char. 209: 0 → 1
Char. 217: 0 → 1
Char. 219: 0 → 1
Char. 220: 0 → 1
Char. 231: 0 → 1
Char. 240: 1 → 0
Char. 260: 2 → 0
Char. 272: 2 → 0
Char. 278: 3 → 0

***Microleter mckinzieorum*:**
Char. 0: 0 → 1
Char. 18: 0 → 1
Char. 24: 0 → 1
Char. 25: 1 → 0
Char. 36: 0 → 1
Char. 39: 0 → 1
Char. 51: 0 → 2
Char. 56: 0 → 1
Char. 57: 1 → 0
Char. 70: 0 → 1
Char. 76: 0 → 1
Char. 79: 0 → 1
Char. 83: 0 → 1
Char. 94: 0 → 1
Char. 106: 0 → 1
Char. 110: 0 → 1
Char. 132: 1 → 0
Char. 159: 0 → 1
Char. 276: 0 → 1
Char. 278: 3 → 1

**Millerettidae:**
Char. 24: 0 → 1
Char. 25: 1 → 0
Char. 44: 1 → 0
Char. 56: 0 → 1
Char. 57: 1 → 0
Char. 66: 0 → 2
Char. 78: 1 → 0
Char. 80: 1 → 0
Char. 84: 1 → 2
Char. 88: 1 → 0
Char. 96: 1 → 0
Char. 121: 0 → 1
Char. 124: 0 → 1
Char. 127: 0 → 1
Char. 135: 0 → 1
Char. 145: 0 → 1
Char. 180: 1 → 0
Char. 192: 1 → 0
Char. 202: 0 → 1
Char. 211: 0 → 1
Char. 230: 0 → 1
Char. 248: 0 → 1
Char. 252: 0 → 1
Char. 253: 0 → 1

***Nycteroleter ineptus*:**
Char. 278: 0 → 3

***Nyctiphruretus acudens*:**
Char. 0: 0 → 1
Char. 21: 0 → 1
Char. 33: 1 → 2
Char. 41: 0 → 1
Char. 66: 1 → 2
Char. 81: 1 → 0
Char. 83: 0 → 1
Char. 84: 1 → 2
Char. 94: 0 → 1
Char. 166: 1 → 0
Char. 207: 1 → 0
Char. 224: 0 → 1
Char. 226: 1 → 0
Char. 266: 0 → 1
Char. 272: 2 → 1
Char. 276: 0 → 1

***Orovenator mayorum*:**
Char. 8: 0 → 1
Char. 24: 0 → 1
Char. 33: 1 → 0
Char. 36: 0 → 1
Char. 50: 0 → 1
Char. 92: 1 → 0
Char. 94: 1 → 0
Char. 135: 1 → 0
Char. 159: 1 → 0
Char. 160: 0 → 1
Char. 165: 0 → 1
Char. 278: 0 → 1

***Owenetta* spp.:**
Char. 142: 1 → 0
Char. 169: 1 → 0

***Paleothyris acadiana*:**
Char. 38: 1 → 0
Char. 50: 1 → 0
Char. 66: 1 → 2
Char. 102: 0 → 1
Char. 146: 1 → 0
Char. 237: 0 → 1
Char. 239: 0 → 1

***Placodus* spp.:**
Char. 0: 1 → 2
Char. 9: 0 → 1
Char. 12: 0 → 1
Char. 13: 0 → 1
Char. 19: 0 → 1
Char. 31: 0 → 1
Char. 46: 1 → 0
Char. 57: 0 → 1
Char. 78: 1 → 0
Char. 93: 1 → 0
Char. 102: 1 → 2
Char. 109: 1 → 0
Char. 140: 1 → 0
Char. 155: 0 → 1
Char. 163: 1 → 0
Char. 164: 0 → 1

***Procolophon* spp.:**
Char. 41: 0 → 1
Char. 69: 0 → 1
Char. 79: 0 → 1
Char. 83: 0 → 1
Char. 86: 0 → 1
Char. 88: 0 → 1
Char. 101: 0 → 1
Char. 117: 1 → 0
Char. 141: 0 → 1
Char. 149: 1 → 0
Char. 203: 1 → 2
Char. 204: 0 → 1
Char. 230: 0 → 1
Char. 237: 0 → 1
Char. 238: 0 → 1
Char. 272: 2 → 1
Char. 278: 3 → 0

***Prolacerta broomi*:**
Char. 58: 1 → 0
Char. 66: 1 → 0
Char. 67: 1 → 0
Char. 80: 1 → 0
Char. 139: 1 → 0
Char. 147: 1 → 0
Char. 192: 1 → 0
Char. 203: 1 → 2
Char. 206: 0 → 12

***Rhipaeosaurus* spp.:**
Char. 172: 0 → 1
Char. 277: 0 → 1

**Rhynchocephalia:**
Char. 0: 1 → 2
Char. 24: 0 → 1
Char. 77: 0 → 1
Char. 88: 1 → 0
Char. 94: 1 → 0
Char. 139: 1 → 0
Char. 167: 1 → 0
Char. 205: 1 → 0

**Rhynchosauria:**
Char. 0: 1 → 0
Char. 7: 0 → 1
Char. 9: 0 → 1
Char. 26: 0 → 1
Char. 44: 1 → 0
Char. 68: 0 → 1
Char. 99: 1 → 0
Char. 150: 1 → 0
Char. 160: 0 → 1
Char. 161: 0 → 1
Char. 171: 0 → 2
Char. 223: 0 → 1
Char. 224: 0 → 1
Char. 241: 0 → 1

***Scutosaurus* spp.:**
Char. 175: 0 → 1
Char. 190: 0 → 1
Char. 218: 0 → 1
Char. 243: 0 → 2
Char. 244: 0 → 1
Char. 251: 0 → 1

***Sinosaurosphargis yunguiensis*:**
Char. 8: 0 → 1
Char. 30: 0 → 1
Char. 53: 0 → 1
Char. 89: 1 → 0
Char. 127: 1 → 0
Char. 150: 1 → 0
Char. 154: 0 → 2
Char. 167: 1 → 0
Char. 253: 0 → 1
Char. 255: 0 → 1

**Squamata:**
Char. 45: 0 → 1
Char. 79: 0 → 2
Char. 80: 1 → 0
Char. 92: 1 → 0
Char. 109: 1 → 0
Char. 160: 0 → 1
Char. 200: 0 → 1
Char. 245: 0 → 1

***Trilophosaurus buettneri*:**
Char. 5: 1 → 0
Char. 11: 0 → 1
Char. 55: 1 → 0
Char. 93: 1 → 0
Char. 104: 0 → 1
Char. 106: 0 → 1
Char. 113: 0 → 1
Char. 122: 0 → 1
Char. 136: 1 → 0
Char. 144: 1 → 0
Char. 157: 0 → 1
Char. 159: 1 → 0
Char. 177: 0 → 12
Char. 194: 0 → 1
Char. 203: 1 → 2
Char. 207: 1 → 0
Char. 208: 1 → 0
Char. 272: 1 → 0

***Youngina capensis*:**
Char. 5: 1 → 0
Char. 25: 0 → 1
Char. 27: 0 → 1
Char. 38: 1 → 0

Char. 44: 1 → 0
Char. 48: 0 → 1
Char. 56: 0 → 1
Char. 75: 0 → 1
Char. 84: 1 → 0
Char. 92: 1 → 0
Char. 94: 1 → 0
Char. 134: 0 → 1
Char. 163: 1 → 0
Char. 170: 0 → 1
Char. 211: 0 → 1
Char. 215: 0 → 1
Char. 224: 0 → 1
Char. 239: 0 → 1

**Node 50:**
Char. 46: 1 → 0
Char. 88: 1 → 0
Char. 89: 1 → 0
Char. 93: 1 → 0
Char. 176: 0 → 1
Char. 195: 0 → 2
Char. 198: 0 → 1
Char. 246: 1 → 2
Char. 259: 0 → 1
Char. 265: 1 → 0
Char. 270: 0 → 1

**Node 51:**
Char. 65: 0 → 1
Char. 131: 0 → 1
Char. 184: 0 → 1
Char. 205: 0 → 1
Char. 210: 0 → 1
Char. 219: 1 → 0
Char. 241: 0 → 1
Char. 246: 0 → 1
Char. 254: 0 → 1
Char. 255: 0 → 1
Char. 256: 0 → 1
Char. 268: 0 → 1
Char. 269: 0 → 1

**Node 52:**
Char. 0: 1 → 2
Char. 15: 0 → 1
Char. 33: 1 → 2
Char. 44: 1 → 0
Char. 62: 1 → 0
Char. 64: 0 → 1
Char. 84: 1 → 2
Char. 130: 0 → 1
Char. 134: 0 → 2
Char. 147: 0 → 1
Char. 148: 0 → 1
Char. 152: 0 → 1
Char. 155: 0 → 1
Char. 158: 0 → 1
Char. 161: 0 → 1
Char. 174: 0 → 1
Char. 181: 0 → 1
Char. 203: 1 → 2
Char. 204: 0 → 2
Char. 247: 0 → 1
Char. 251: 0 → 1
Char. 252: 0 → 1
Char. 253: 0 → 2

**Node 53:**
Char. 0: 0 → 1
Char. 5: 0 → 1
Char. 59: 0 → 1
Char. 60: 0 → 1
Char. 62: 0 → 1
Char. 72: 1 → 2
Char. 92: 0 → 1
Char. 159: 0 → 1
Char. 265: 0 → 1
Char. 275: 1 → 0
Char. 278: 3 → 0

**Node 54:**
Char. 20: 0 → 1
Char. 25: 1 → 0
Char. 29: 1 → 0
Char. 33: 0 → 1
Char. 50: 1 → 0
Char. 57: 1 → 0
Char. 67: 0 → 1
Char. 89: 0 → 1
Char. 94: 0 → 1
Char. 127: 0 → 1

**Node 55:**
Char. 29: 0 → 1
Char. 40: 0 → 2
Char. 74: 0 → 1
Char. 80: 0 → 1
Char. 84: 0 → 1
Char. 88: 0 → 1
Char. 116: 0 → 1
Char. 119: 0 → 1
Char. 132: 0 → 1
Char. 149: 0 → 1
Char. 156: 0 → 1
Char. 169: 0 → 1
Char. 180: 0 → 1
Char. 192: 0 → 1
Char. 201: 0 → 1
Char. 203: 0 → 1
Char. 235: 0 → 1

**Node 56:**
Char. 72: 0 → 1
Char. 79: 1 → 0
Char. 81: 0 → 1
Char. 93: 0 → 1
Char. 97: 0 → 1
Char. 104: 1 → 0
Char. 144: 0 → 1
Char. 173: 0 → 1
Char. 183: 0 → 1

**Node 58:**
Char. 95: 0 → 1
Char. 113: 0 → 1
Char. 114: 0 → 1

**Node 59:**
Char. 5: 0 → 1
Char. 20: 0 → 1
Char. 47: 0 → 1
Char. 48: 0 → 1
Char. 79: 0 → 1
Char. 110: 0 → 1
Char. 131: 0 → 1
Char. 137: 0 → 1
Char. 140: 0 → 2
Char. 147: 0 → 1
Char. 159: 0 → 1

**Node 60:**
Char. 19: 0 → 1
Char. 92: 1 → 0

**Node 61:**
Char. 4: 0 → 1
Char. 15: 0 → 1
Char. 29: 0 → 1
Char. 213: 0 → 1
Char. 226: 0 → 2
Char. 228: 0 → 1
Char. 275: 0 → 1

**Node 62:**
Char. 58: 0 → 1
Char. 61: 0 → 1
Char. 66: 0 → 1
Char. 69: 0 → 1
Char. 107: 0 → 1
Char. 126: 0 → 1
Char. 131: 0 → 1
Char. 134: 0 → 1
Char. 140: 0 → 1
Char. 147: 0 → 1
Char. 148: 0 → 1
Char. 150: 0 → 1
Char. 167: 0 → 1
Char. 176: 0 → 1
Char. 190: 0 → 1
Char. 205: 0 → 1
Char. 208: 0 → 1
Char. 230: 0 → 1
Char. 239: 0 → 1
Char. 260: 2 → 1

**Node 63:**
Char. 73: 0 → 1
Char. 131: 1 → 0
Char. 205: 0 → 1
Char. 273: 0 → 1
Char. 276: 0 → 1

**Node 64:**
Char. 18: 0 → 1
Char. 23: 0 → 1
Char. 25: 1 → 0
Char. 37: 0 → 1
Char. 103: 0 → 1
Char. 106: 0 → 1
Char. 107: 0 → 1
Char. 110: 0 → 1
Char. 118: 1 → 0
Char. 125: 1 → 0
Char. 126: 0 → 1
Char. 150: 0 → 1

**Node 65:**
Char. 186: 0 → 1
Char. 207: 0 → 1

**Node 66:**
Char. 148: 0 → 1
Char. 159: 0 → 1
Char. 183: 1 → 2
Char. 194: 0 → 1
Char. 201: 1 → 0
Char. 211: 0 → 1
Char. 226: 0 → 1
Char. 240: 1 → 0
Char. 252: 0 → 1

**Node 67:**
Char. 87: 0 → 1
Char. 110: 0 → 1

**Node 68:**
Char. 25: 1 → 0
Char. 76: 1 → 0

**Node 69:**
Char. 79: 0 → 1
Char. 86: 0 → 1
Char. 93: 1 → 0
Char. 100: 0 → 1
Char. 113: 0 → 1
Char. 133: 0 → 1
Char. 147: 1 → 2
Char. 154: 1 → 0
Char. 278: 3 → 0

**Node 70:**
Char. 5: 0 → 1
Char. 38: 1 → 2
Char. 39: 0 → 1
Char. 50: 1 → 0
Char. 58: 0 → 1
Char. 59: 0 → 1
Char. 60: 0 → 1
Char. 72: 1 → 2
Char. 83: 0 → 1
Char. 85: 1 → 0
Char. 88: 1 → 0
Char. 95: 0 → 1
Char. 104: 0 → 1
Char. 105: 0 → 1
Char. 106: 0 → 2
Char. 107: 0 → 1
Char. 109: 0 → 1
Char. 110: 0 → 1
Char. 146: 1 → 0
Char. 148: 0 → 1
Char. 155: 0 → 2
Char. 183: 1 → 2

**Node 71:**
Char. 23: 0 → 1
Char. 33: 1 → 0
Char. 38: 2 → 1
Char. 39: 1 → 0
Char. 42: 0 → 1
Char. 43: 0 → 1
Char. 46: 1 → 0
Char. 49: 1 → 0
Char. 71: 1 → 0
Char. 83: 0 → 2
Char. 86: 0 → 1
Char. 93: 1 → 0
Char. 101: 0 → 1
Char. 103: 0 → 1
Char. 106: 0 → 1
Char. 161: 0 → 1
Char. 163: 1 → 0
Char. 172: 0 → 2
Char. 174: 0 → 1
Char. 188: 0 → 1
Char. 195: 0 → 1
Char. 203: 1 → 2
Char. 204: 0 → 2
Char. 212: 0 → 1
Char. 216: 1 → 0
Char. 236: 0 → 1
Char. 238: 0 → 2
Char. 241: 0 → 1
Char. 242: 0 → 1
Char. 273: 0 → 1
Char. 274: 0 → 1
Char. 275: 1 → 0

**Node 72:**
Char. 52: 0 → 1
Char. 75: 0 → 1
Char. 84: 1 → 0
Char. 87: 0 → 1
Char. 100: 0 → 1
Char. 110: 0 → 1
Char. 113: 0 → 1
Char. 121: 0 → 1
Char. 141: 0 → 1
Char. 142: 1 → 0
Char. 143: 0 → 1
Char. 180: 1 → 0
Char. 230: 0 → 1

**Node 73:**
Char. 29: 1 → 0
Char. 44: 1 → 0
Char. 59: 0 → 1
Char. 60: 0 → 1
Char. 66: 0 → 1
Char. 67: 0 → 1
Char. 72: 1 → 2
Char. 78: 1 → 0
Char. 84: 1 → 0
Char. 88: 1 → 0
Char. 93: 1 → 0
Char. 111: 0 → 1
Char. 123: 1 → 0
Char. 129: 0 → 1
Char. 154: 1 → 0
Char. 169: 1 → 0
Char. 170: 0 → 1

Char. 192: 1 → 0
Char. 197: 0 → 1

**Node 74:**
Char. 83: 0 → 1
Char. 85: 1 → 0
Char. 107: 0 → 1

**Node 75:**
Char. 2: 0 → 1
Char. 6: 0 → 1
Char. 186: 0 → 1
Char. 198: 0 → 1
Char. 200: 0 → 1
Char. 220: 0 → 1
Char. 239: 1 → 0

**Node 76:**
Char. 48: 0 → 1
Char. 98: 1 → 0
Char. 113: 0 → 1
Char. 181: 0 → 1
Char. 206: 0 → 2

**Node 77:**
Char. 35: 0 → 1
Char. 102: 0 → 1
Char. 103: 0 → 1
Char. 184: 0 → 1
Char. 201: 0 → 1
Char. 210: 0 → 1
Char. 223: 0 → 1
Char. 229: 0 → 1

**Node 78:**
Char. 17: 0 → 1
Char. 42: 0 → 1
Char. 251: 0 → 1
Char. 264: 0 → 1

**Node 79:**
Char. 41: 0 → 1
Char. 61: 1 → 2
Char. 112: 1 → 0
Char. 138: 0 → 1
Char. 146: 1 → 0
Char. 155: 0 → 1
Char. 192: 1 → 2
Char. 224: 0 → 1
Char. 226: 0 → 1
Char. 227: 0 → 1
Char. 233: 0 → 1
Char. 267: 01 → 2

**Node 80:**
Char. 61: 1 → 3
Char. 90: 0 → 1
Char. 91: 0 → 1
Char. 109: 1 → 0
Char. 155: 0 → 1
Char. 209: 1 → 0

**ANALYSIS 60**
**(ALL TAXA, IMPLIED WEIGHTING, K = 5.5)**

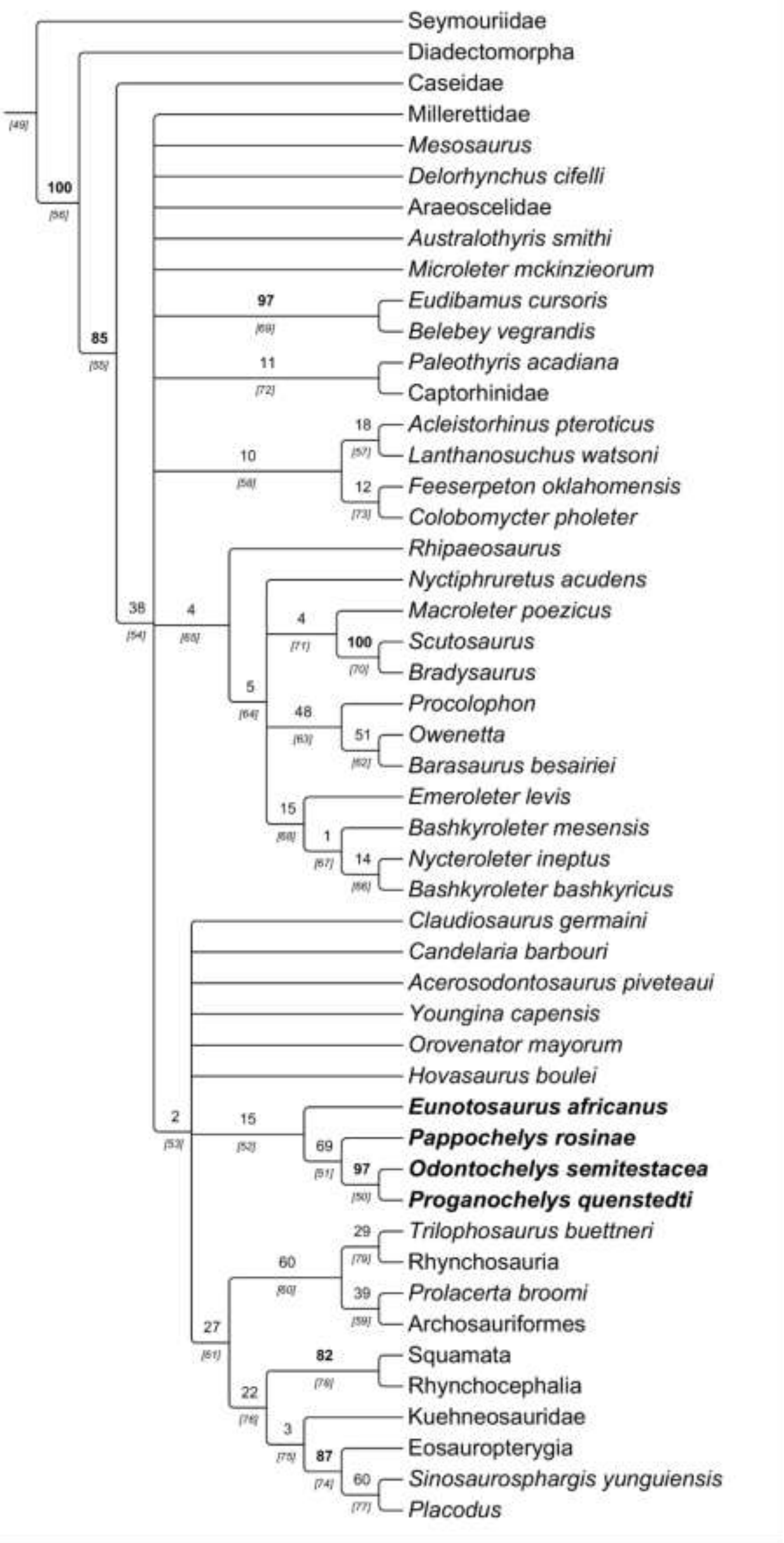

***Proganochelys quenstedti*:**
Char. 8: 0 → 1
Char. 11: 0 → 1
Char. 106: 0 → 1
Char. 108: 1 → 0
Char. 128: 0 → 1
Char. 175: 0 → 1
Char. 202: 1 → 0
Char. 207: 1 → 0
Char. 209: 1 → 0
Char. 244: 0 → 1
Char. 248: 0 → 1
Char. 250: 0 → 1
Char. 252: 1 → 0
Char. 262: 0 → 1

***Pappochelys rosinae*:**
Char. 0: 2 → 0
Char. 1: 0 → 1
Char. 5: 1 → 0
Char. 12: 0 → 1
Char. 41: 0 → 1
Char. 48: 0 → 1
Char. 49: 0 → 1
Char. 75: 0 → 1
Char. 169: 1 → 0
Char. 260: 2 → 0

***Odontochelys semitestacea*:**
Char. 43: 0 → 1

***Eunotosaurus africanus*:**
Char. 19: 0 → 1
Char. 43: 0 → 1
Char. 59: 1 → 0
Char. 72: 2 → 1
Char. 76: 0 → 1
Char. 97: 1 → 0
Char. 103: 0 → 1
Char. 153: 1 → 0
Char. 191: 1 → 0
Char. 192: 1 → 0
Char. 202: 1 → 0
Char. 211: 0 → 1
Char. 222: 1 → 0
Char. 237: 1 → 0
Char. 248: 0 → 1
Char. 249: 0 → 2
Char. 250: 0 → 1
Char. 263: 0 → 1
Char. 273: 0 → 1
Char. 274: 0 → 1
Char. 275: 0 → 1
Char. 276: 0 → 1
Char. 277: 0 → 1

***Acerosodontosaurus piveteaui*:**
Char. 78: 1 → 0
Char. 81: 1 → 0
Char. 127: 1 → 0
Char. 128: 0 → 1
Char. 155: 0 → 1
Char. 206: 0 → 2
Char. 208: 0 → 1
Char. 278: 03 → 1

***Acleistorhinus pteroticus*:**
Char. 21: 0 → 1
Char. 146: 1 → 0

**Araeoscelidae:**
Char. 0: 0 → 1
Char. 23: 0 → 1
Char. 27: 0 → 1
Char. 28: 0 → 1
Char. 29: 1 → 0
Char. 38: 1 → 0
Char. 40: 2 → 0
Char. 43: 0 → 1
Char. 48: 0 → 1
Char. 50: 1 → 0
Char. 57: 1 → 0
Char. 59: 0 → 1
Char. 60: 0 → 1
Char. 67: 0 → 1
Char. 72: 1 → 2
Char. 84: 1 → 0
Char. 89: 0 → 1
Char. 106: 0 → 1
Char. 111: 0 → 1
Char. 112: 0 → 1
Char. 116: 1 → 0
Char. 117: 01 → 2
Char. 120: 0 → 1
Char. 154: 1 → 0
Char. 169: 1 → 0
Char. 170: 0 → 1
Char. 193: 0 → 1
Char. 197: 0 → 1
Char. 222: 0 → 1
Char. 224: 0 → 1
Char. 237: 0 → 1
Char. 239: 0 → 1
Char. 266: 0 → 1
Char. 278: 3 → 1

**Archosauriformes:**
Char. 32: 0 → 1
Char. 94: 1 → 0
Char. 112: 1 → 0
Char. 152: 0 → 1
Char. 154: 01 → 2
Char. 166: 1 → 0
Char. 171: 0 → 1
Char. 185: 0 → 1
Char. 201: 0 → 1
Char. 204: 0 → 1
Char. 218: 0 → 3
Char. 224: 0 → 1
Char. 242: 0 → 1

***Australothyris smithi*:**
Char. 23: 0 → 1
Char. 24: 0 → 1
Char. 34: 0 → 1
Char. 55: 1 → 0
Char. 57: 1 → 0
Char. 71: 1 → 0
Char. 79: 0 → 1
Char. 83: 0 → 1
Char. 85: 1 → 0
Char. 98: 1 → 0
Char. 100: 0 → 1
Char. 103: 0 → 1
Char. 110: 0 → 1
Char. 112: 0 → 1
Char. 123: 1 → 0
Char. 129: 0 → 1
Char. 131: 0 → 1
Char. 132: 1 → 0
Char. 144: 1 → 0
Char. 147: 0 → 1
Char. 149: 1 → 0
Char. 150: 0 → 1
Char. 192: 1 → 0

***Barasaurus besairiei*:**
Char. 33: 1 → 0

***Bashkyroleter bashkyricus*:**
Char. 275: 1 → 0

***Bashkyroleter mesensis*:**
Char. 169: 1 → 0

***Belebey vegrandis*:**
Char. 154: 1 → 0

***Bradysaurus* spp.:**
Char. 19: 0 → 1
Char. 25: 1 → 0
Char. 73: 0 → 1
Char. 79: 0 → 1
Char. 135: 1 → 0
Char. 249: 0 → 1

***Candelaria barbouri*:**
Char. 0: 1 → 0
Char. 1: 0 → 1
Char. 5: 1 → 0
Char. 8: 0 → 1
Char. 15: 0 → 1
Char. 33: 1 → 2
Char. 49: 0 → 1
Char. 55: 1 → 0
Char. 59: 1 → 0
Char. 62: 1 → 0
Char. 72: 2 → 1
Char. 76: 0 → 1
Char. 79: 0 → 2
Char. 83: 0 → 1
Char. 88: 1 → 0
Char. 92: 1 → 0
Char. 95: 0 → 1
Char. 126: 0 → 1
Char. 132: 1 → 0
Char. 154: 1 → 2
Char. 159: 1 → 0
Char. 169: 1 → 0
Char. 265: 1 → 0
Char. 275: 0 → 1
Char. 276: 0 → 1
Char. 277: 0 → 1

**Captorhinidae:**
Char. 3: 0 → 1
Char. 23: 0 → 1
Char. 25: 1 → 0
Char. 26: 0 → 1
Char. 73: 0 → 1
Char. 75: 0 → 1
Char. 83: 0 → 1
Char. 108: 1 → 0
Char. 180: 1 → 0
Char. 183: 1 → 0
Char. 201: 1 → 0
Char. 203: 1 → 2
Char. 216: 1 → 0
Char. 240: 1 → 0

**Caseidae:**
Char. 24: 0 → 1
Char. 25: 1 → 0
Char. 36: 0 → 1
Char. 38: 1 → 0
Char. 46: 1 → 0
Char. 50: 1 → 0
Char. 56: 0 → 1
Char. 170: 0 → 1
Char. 194: 0 → 1
Char. 273: 0 → 1
Char. 274: 0 → 1
Char. 278: 3 → 2

***Claudiosaurus germaini*:**
Char. 24: 0 → 1
Char. 27: 0 → 1
Char. 34: 0 → 1
Char. 36: 0 → 1
Char. 48: 0 → 1
Char. 56: 0 → 1
Char. 64: 0 → 1
Char. 70: 0 → 1
Char. 84: 1 → 0
Char. 105: 0 → 1
Char. 106: 0 → 1
Char. 117: 01 → 2
Char. 126: 0 → 1
Char. 127: 1 → 0
Char. 130: 0 → 1
Char. 131: 0 → 1
Char. 141: 1 → 0
Char. 144: 1 → 0
Char. 148: 0 → 1
Char. 154: 1 → 0
Char. 166: 1 → 0
Char. 182: 0 → 1
Char. 187: 1 → 0
Char. 190: 0 → 1
Char. 199: 0 → 1
Char. 201: 0 → 1
Char. 203: 1 → 2
Char. 204: 0 → 1
Char. 222: 1 → 0
Char. 234: 1 → 0
Char. 267: 0 → 1
Char. 272: 2 → 1

***Colobomycter pholeter*:**
Char. 21: 0 → 1
Char. 25: 1 → 0
Char. 84: 1 → 0
Char. 154: 1 → 0
Char. 167: 0 → 1
Char. 267: 0 → 1

***Delorhynchus cifelli*:**
Char. 18: 0 → 1
Char. 20: 0 → 1
Char. 21: 0 → 1
Char. 24: 0 → 1
Char. 26: 0 → 1
Char. 28: 0 → 1
Char. 33: 0 → 2
Char. 39: 0 → 1
Char. 48: 0 → 1
Char. 52: 0 → 1
Char. 100: 0 → 1
Char. 111: 0 → 1
Char. 116: 1 → 0
Char. 119: 1 → 0
Char. 131: 0 → 1
Char. 147: 0 → 1
Char. 156: 1 → 0
Char. 167: 0 → 1
Char. 189: 0 → 1
Char. 191: 0 → 1
Char. 204: 0 → 2
Char. 267: 0 → 1

**Diadectomorpha:**
Char. 0: 0 → 1
Char. 64: 0 → 1
Char. 70: 0 → 1
Char. 122: 0 → 1
Char. 123: 1 → 0
Char. 146: 1 → 0
Char. 275: 1 → 0
Char. 278: 3 → 0

***Emeroleter levis*:**
Char. 0: 0 → 2
Char. 51: 1 → 0

**Eosauropterygia:**
Char. 166: 1 → 0
Char. 174: 0 → 1
Char. 178: 0 → 1
Char. 194: 0 → 2
Char. 272: 1 → 0

***Eudibamus cursoris*:**
Char. 154: 1 → 2

***Feeserpeton oklahomensis*:**
Char. 51: 0 → 1
Char. 157: 0 → 1
Char. 158: 0 → 1

***Hovasaurus boulei*:**
Char. 41: 0 → 1
Char. 43: 0 → 1
Char. 48: 0 → 1
Char. 55: 1 → 0
Char. 72: 2 → 1
Char. 77: 0 → 2
Char. 78: 1 → 0
Char. 79: 0 → 1
Char. 93: 1 → 0
Char. 113: 0 → 1
Char. 138: 0 → 1
Char. 141: 1 → 0
Char. 146: 1 → 0
Char. 204: 0 → 2
Char. 206: 0 → 2
Char. 215: 0 → 1
Char. 219: 1 → 0
Char. 224: 0 → 1

**Kuehneosauridae:**
Char. 7: 0 → 1
Char. 24: 0 → 1
Char. 27: 0 → 1
Char. 43: 1 → 0
Char. 79: 0 → 2
Char. 107: 1 → 0
Char. 140: 1 → 0
Char. 147: 1 → 0
Char. 148: 1 → 0
Char. 245: 0 → 1
Char. 278: 0 → 3

***Lanthanosuchus watsoni*:**
Char. 25: 1 → 0
Char. 51: 0 → 1
Char. 76: 0 → 1
Char. 86: 0 → 1
Char. 98: 1 → 0
Char. 138: 0 → 1
Char. 144: 1 → 0
Char. 154: 1 → 2

***Macroleter poezicus*:**
Char. 9: 0 → 1
Char. 26: 0 → 1
Char. 66: 1 → 2
Char. 126: 0 → 1
Char. 134: 0 → 2
Char. 140: 0 → 1
Char. 146: 1 → 0
Char. 147: 1 → 2
Char. 154: 1 → 0
Char. 169: 1 → 0
Char. 278: 3 → 0

***Mesosaurus* spp.:**
Char. 0: 0 → 1
Char. 2: 0 → 1
Char. 6: 0 → 1
Char. 8: 0 → 1
Char. 9: 0 → 1
Char. 13: 0 → 1
Char. 19: 0 → 1
Char. 23: 0 → 1
Char. 26: 0 → 1
Char. 29: 1 → 0
Char. 33: 0 → 1
Char. 38: 1 → 0
Char. 41: 0 → 1
Char. 48: 0 → 1
Char. 50: 1 → 0
Char. 67: 0 → 1
Char. 76: 0 → 1
Char. 83: 0 → 1
Char. 84: 1 → 0
Char. 85: 1 → 0
Char. 94: 0 → 1
Char. 107: 0 → 1
Char. 109: 0 → 1
Char. 111: 0 → 1
Char. 115: 0 → 1
Char. 146: 1 → 0
Char. 148: 0 → 1
Char. 149: 1 → 0
Char. 164: 0 → 1
Char. 167: 0 → 1
Char. 176: 0 → 1
Char. 183: 1 → 0
Char. 184: 0 → 1
Char. 192: 1 → 0
Char. 199: 0 → 1
Char. 202: 0 → 1
Char. 204: 0 → 2
Char. 206: 0 → 1
Char. 207: 0 → 1
Char. 209: 0 → 1
Char. 217: 0 → 1
Char. 219: 0 → 1
Char. 220: 0 → 1
Char. 231: 0 → 1
Char. 240: 1 → 0
Char. 260: 2 → 0
Char. 272: 2 → 0
Char. 278: 3 → 0

***Microleter mckinzieorum*:**
Char. 0: 0 → 1
Char. 18: 0 → 1
Char. 24: 0 → 1
Char. 25: 1 → 0
Char. 36: 0 → 1
Char. 39: 0 → 1
Char. 51: 0 → 2
Char. 56: 0 → 1
Char. 57: 1 → 0
Char. 70: 0 → 1
Char. 76: 0 → 1
Char. 79: 0 → 1
Char. 83: 0 → 1
Char. 94: 0 → 1
Char. 106: 0 → 1
Char. 110: 0 → 1
Char. 132: 1 → 0
Char. 276: 0 → 1
Char. 278: 3 → 1

**Millerettidae:**
Char. 24: 0 → 1
Char. 25: 1 → 0
Char. 44: 1 → 0
Char. 56: 0 → 1
Char. 57: 1 → 0
Char. 66: 0 → 2
Char. 78: 1 → 0
Char. 80: 1 → 0
Char. 84: 1 → 2
Char. 88: 1 → 0
Char. 96: 1 → 0
Char. 121: 0 → 1
Char. 124: 0 → 1
Char. 127: 0 → 1
Char. 135: 0 → 1
Char. 145: 0 → 1
Char. 180: 1 → 0
Char. 192: 1 → 0
Char. 202: 0 → 1
Char. 211: 0 → 1
Char. 230: 0 → 1
Char. 248: 0 → 1
Char. 252: 0 → 1
Char. 253: 0 → 1

***Nycteroleter ineptus*:**
Char. 278: 0 → 3

***Nyctiphruretus acudens*:**
Char. 0: 0 → 1
Char. 21: 0 → 1
Char. 33: 1 → 2
Char. 41: 0 → 1
Char. 66: 1 → 2
Char. 81: 1 → 0
Char. 83: 0 → 1
Char. 84: 1 → 2
Char. 94: 0 → 1
Char. 166: 1 → 0
Char. 207: 1 → 0
Char. 224: 0 → 1
Char. 226: 1 → 0
Char. 266: 0 → 1
Char. 272: 2 → 1
Char. 276: 0 → 1

***Orovenator mayorum*:**
Char. 8: 0 → 1
Char. 24: 0 → 1
Char. 33: 1 → 0
Char. 36: 0 → 1
Char. 50: 0 → 1
Char. 92: 1 → 0
Char. 94: 1 → 0
Char. 135: 1 → 0
Char. 159: 1 → 0
Char. 160: 0 → 1
Char. 165: 0 → 1
Char. 278: 03 → 1

***Owenetta* spp.:**
Char. 142: 1 → 0
Char. 169: 1 → 0

***Paleothyris acadiana*:**
Char. 38: 1 → 0
Char. 50: 1 → 0
Char. 66: 1 → 2
Char. 102: 0 → 1
Char. 146: 1 → 0
Char. 237: 0 → 1
Char. 239: 0 → 1

***Placodus* spp.:**
Char. 0: 1 → 2
Char. 9: 0 → 1
Char. 12: 0 → 1
Char. 13: 0 → 1
Char. 19: 0 → 1
Char. 31: 0 → 1
Char. 46: 1 → 0
Char. 57: 0 → 1
Char. 78: 1 → 0
Char. 93: 1 → 0
Char. 102: 1 → 2
Char. 109: 1 → 0
Char. 140: 1 → 0
Char. 155: 0 → 1
Char. 163: 1 → 0
Char. 164: 0 → 1

***Procolophon* spp.:**
Char. 41: 0 → 1
Char. 69: 0 → 1
Char. 79: 0 → 1
Char. 83: 0 → 1
Char. 86: 0 → 1
Char. 88: 0 → 1
Char. 101: 0 → 1
Char. 117: 1 → 0
Char. 141: 0 → 1
Char. 149: 1 → 0
Char. 203: 1 → 2
Char. 204: 0 → 1
Char. 230: 0 → 1
Char. 237: 0 → 1
Char. 238: 0 → 1
Char. 272: 2 → 1
Char. 278: 3 → 0

***Prolacerta broomi*:**
Char. 58: 1 → 0
Char. 66: 1 → 0
Char. 67: 1 → 0
Char. 80: 1 → 0
Char. 139: 1 → 0
Char. 147: 1 → 0
Char. 192: 1 → 0
Char. 203: 1 → 2
Char. 206: 0 → 12

***Rhipaeosaurus* spp.:**
Char. 172: 0 → 1
Char. 277: 0 → 1

**Rhynchocephalia:**
Char. 0: 1 → 2
Char. 24: 0 → 1
Char. 77: 0 → 1
Char. 88: 1 → 0
Char. 94: 1 → 0
Char. 139: 1 → 0
Char. 167: 1 → 0
Char. 205: 1 → 0

**Rhynchosauria:**
Char. 0: 1 → 0
Char. 7: 0 → 1
Char. 9: 0 → 1
Char. 26: 0 → 1
Char. 44: 1 → 0
Char. 68: 0 → 1
Char. 99: 1 → 0
Char. 150: 1 → 0
Char. 160: 0 → 1
Char. 161: 0 → 1
Char. 171: 0 → 2
Char. 223: 0 → 1
Char. 224: 0 → 1
Char. 241: 0 → 1

***Scutosaurus* spp.:**
Char. 175: 0 → 1
Char. 190: 0 → 1
Char. 218: 0 → 1
Char. 243: 0 → 2
Char. 244: 0 → 1
Char. 251: 0 → 1

***Sinosaurosphargis yunguiensis*:**
Char. 8: 0 → 1
Char. 30: 0 → 1
Char. 53: 0 → 1
Char. 89: 1 → 0
Char. 127: 1 → 0
Char. 150: 1 → 0
Char. 154: 0 → 2
Char. 167: 1 → 0
Char. 253: 0 → 1
Char. 255: 0 → 1

**Squamata:**
Char. 45: 0 → 1
Char. 79: 0 → 2
Char. 80: 1 → 0
Char. 92: 1 → 0
Char. 109: 1 → 0
Char. 160: 0 → 1
Char. 200: 0 → 1
Char. 245: 0 → 1

***Trilophosaurus buettneri*:**
Char. 5: 1 → 0
Char. 11: 0 → 1
Char. 55: 1 → 0
Char. 93: 1 → 0
Char. 104: 0 → 1
Char. 106: 0 → 1
Char. 113: 0 → 1
Char. 122: 0 → 1
Char. 136: 1 → 0
Char. 144: 1 → 0
Char. 157: 0 → 1

Char. 159: 1 → 0
Char. 177: 0 → 12
Char. 194: 0 → 1
Char. 203: 1 → 2
Char. 207: 1 → 0
Char. 208: 1 → 0
Char. 272: 1 → 0

***Youngina capensis*:**
Char. 5: 1 → 0
Char. 21: 0 → 1
Char. 25: 0 → 1
Char. 27: 0 → 1
Char. 38: 1 → 0
Char. 43: 0 → 1
Char. 44: 1 → 0
Char. 48: 0 → 1
Char. 56: 0 → 1
Char. 75: 0 → 1
Char. 84: 1 → 0
Char. 92: 1 → 0
Char. 94: 1 → 0
Char. 134: 0 → 1
Char. 154: 1 → 0
Char. 163: 1 → 0
Char. 170: 0 → 1
Char. 211: 0 → 1
Char. 215: 0 → 1
Char. 224: 0 → 1
Char. 239: 0 → 1
Char. 267: 0 → 1

**Node 50:**
Char. 46: 1 → 0
Char. 88: 1 → 0
Char. 89: 1 → 0
Char. 93: 1 → 0
Char. 176: 0 → 1
Char. 195: 0 → 2
Char. 198: 0 → 1
Char. 246: 1 → 2
Char. 259: 0 → 1
Char. 265: 1 → 0
Char. 270: 0 → 1

**Node 51:**
Char. 65: 0 → 1
Char. 131: 0 → 1
Char. 184: 0 → 1
Char. 205: 0 → 1
Char. 210: 0 → 1
Char. 219: 1 → 0
Char. 241: 0 → 1
Char. 246: 0 → 1
Char. 254: 0 → 1
Char. 255: 0 → 1
Char. 256: 0 → 1
Char. 267: 0 → 1
Char. 268: 0 → 1
Char. 269: 0 → 1

**Node 52:**
Char. 0: 1 → 2
Char. 15: 0 → 1
Char. 21: 0 → 1
Char. 33: 1 → 2
Char. 44: 1 → 0
Char. 62: 1 → 0
Char. 64: 0 → 1
Char. 84: 1 → 2
Char. 130: 0 → 1
Char. 134: 0 → 2
Char. 147: 0 → 1
Char. 148: 0 → 1
Char. 152: 0 → 1
Char. 155: 0 → 1
Char. 158: 0 → 1
Char. 161: 0 → 1
Char. 174: 0 → 1
Char. 181: 0 → 1
Char. 203: 1 → 2
Char. 204: 0 → 2
Char. 247: 0 → 1
Char. 251: 0 → 1
Char. 252: 0 → 1
Char. 253: 0 → 2

**Node 53:**
Char. 0: 0 → 1
Char. 20: 0 → 1
Char. 25: 1 → 0
Char. 29: 1 → 0
Char. 33: 0 → 1
Char. 40: 2 → 0
Char. 50: 1 → 0
Char. 57: 1 → 0
Char. 59: 0 → 1
Char. 62: 0 → 1
Char. 67: 0 → 1
Char. 72: 1 → 2
Char. 89: 0 → 1
Char. 94: 0 → 1
Char. 109: 0 → 1
Char. 111: 0 → 1
Char. 112: 0 → 1
Char. 120: 0 → 1
Char. 127: 0 → 1
Char. 129: 0 → 1
Char. 135: 0 → 1
Char. 136: 0 → 1
Char. 139: 0 → 1
Char. 141: 0 → 1
Char. 179: 0 → 1
Char. 187: 0 → 1
Char. 188: 0 → 1
Char. 191: 0 → 1
Char. 193: 0 → 1
Char. 196: 0 → 1
Char. 201: 1 → 0
Char. 202: 0 → 1
Char. 207: 0 → 1
Char. 209: 0 → 1
Char. 219: 0 → 1
Char. 222: 0 → 1
Char. 237: 0 → 1
Char. 265: 0 → 1
Char. 266: 0 → 1
Char. 275: 1 → 0

**Node 54:**
Char. 29: 0 → 1
Char. 40: 0 → 2
Char. 74: 0 → 1
Char. 80: 0 → 1
Char. 84: 0 → 1
Char. 88: 0 → 1
Char. 116: 0 → 1
Char. 119: 0 → 1
Char. 132: 0 → 1
Char. 149: 0 → 1
Char. 156: 0 → 1
Char. 169: 0 → 1
Char. 180: 0 → 1
Char. 192: 0 → 1
Char. 201: 0 → 1
Char. 203: 0 → 1
Char. 235: 0 → 1

**Node 55:**
Char. 72: 0 → 1
Char. 79: 1 → 0
Char. 81: 0 → 1
Char. 93: 0 → 1
Char. 97: 0 → 1
Char. 104: 1 → 0
Char. 144: 0 → 1
Char. 173: 0 → 1
Char. 183: 0 → 1

**Node 57:**
Char. 95: 0 → 1
Char. 113: 0 → 1
Char. 114: 0 → 1

**Node 58:**
Char. 20: 0 → 1
Char. 47: 0 → 1
Char. 48: 0 → 1
Char. 79: 0 → 1
Char. 110: 0 → 1
Char. 131: 0 → 1
Char. 137: 0 → 1
Char. 140: 0 → 2
Char. 147: 0 → 1

**Node 59:**
Char. 19: 0 → 1
Char. 92: 1 → 0

**Node 60:**
Char. 4: 0 → 1
Char. 15: 0 → 1
Char. 29: 0 → 1
Char. 213: 0 → 1
Char. 226: 0 → 2
Char. 228: 0 → 1
Char. 275: 0 → 1

**Node 61:**
Char. 43: 0 → 1
Char. 58: 0 → 1
Char. 61: 0 → 1
Char. 66: 0 → 1
Char. 69: 0 → 1
Char. 107: 0 → 1
Char. 126: 0 → 1
Char. 131: 0 → 1
Char. 134: 0 → 1
Char. 140: 0 → 1
Char. 147: 0 → 1
Char. 148: 0 → 1
Char. 150: 0 → 1
Char. 167: 0 → 1
Char. 176: 0 → 1
Char. 190: 0 → 1
Char. 205: 0 → 1
Char. 208: 0 → 1
Char. 230: 0 → 1
Char. 239: 0 → 1
Char. 260: 2 → 1

**Node 62:**
Char. 73: 0 → 1
Char. 131: 1 → 0
Char. 205: 0 → 1
Char. 273: 0 → 1
Char. 276: 0 → 1

**Node 63:**
Char. 18: 0 → 1
Char. 23: 0 → 1
Char. 25: 1 → 0
Char. 37: 0 → 1
Char. 103: 0 → 1
Char. 106: 0 → 1
Char. 107: 0 → 1
Char. 110: 0 → 1
Char. 118: 1 → 0
Char. 125: 1 → 0
Char. 126: 0 → 1
Char. 150: 0 → 1

**Node 64:**
Char. 186: 0 → 1
Char. 207: 0 → 1

**Node 65:**
Char. 148: 0 → 1
Char. 183: 1 → 2
Char. 194: 0 → 1
Char. 201: 1 → 0
Char. 211: 0 → 1
Char. 226: 0 → 1
Char. 240: 1 → 0
Char. 252: 0 → 1

**Node 66:**
Char. 87: 0 → 1
Char. 110: 0 → 1

**Node 67:**
Char. 25: 1 → 0
Char. 76: 1 → 0

**Node 68:**
Char. 79: 0 → 1
Char. 86: 0 → 1
Char. 93: 1 → 0
Char. 100: 0 → 1
Char. 113: 0 → 1
Char. 133: 0 → 1
Char. 147: 1 → 2
Char. 154: 1 → 0
Char. 278: 3 → 0

**Node 69:**
Char. 38: 1 → 2
Char. 39: 0 → 1
Char. 50: 1 → 0
Char. 58: 0 → 1
Char. 59: 0 → 1
Char. 60: 0 → 1
Char. 72: 1 → 2
Char. 83: 0 → 1
Char. 85: 1 → 0
Char. 88: 1 → 0
Char. 95: 0 → 1
Char. 104: 0 → 1
Char. 105: 0 → 1
Char. 106: 0 → 2
Char. 107: 0 → 1
Char. 109: 0 → 1
Char. 110: 0 → 1
Char. 146: 1 → 0
Char. 148: 0 → 1
Char. 155: 0 → 2
Char. 183: 1 → 2

**Node 70:**
Char. 23: 0 → 1
Char. 33: 1 → 0
Char. 38: 2 → 1
Char. 39: 1 → 0
Char. 42: 0 → 1
Char. 43: 0 → 1
Char. 46: 1 → 0
Char. 49: 1 → 0
Char. 71: 1 → 0
Char. 83: 0 → 2
Char. 86: 0 → 1
Char. 93: 1 → 0
Char. 101: 0 → 1
Char. 103: 0 → 1
Char. 106: 0 → 1
Char. 161: 0 → 1
Char. 163: 1 → 0
Char. 172: 0 → 2
Char. 174: 0 → 1
Char. 188: 0 → 1
Char. 195: 0 → 1
Char. 203: 1 → 2
Char. 204: 0 → 2
Char. 212: 0 → 1
Char. 216: 1 → 0
Char. 236: 0 → 1
Char. 238: 0 → 2
Char. 241: 0 → 1
Char. 242: 0 → 1
Char. 273: 0 → 1

Char. 274: 0 → 1
Char. 275: 1 → 0

**Node 71:**
Char. 52: 0 → 1
Char. 75: 0 → 1
Char. 84: 1 → 0
Char. 87: 0 → 1
Char. 100: 0 → 1
Char. 110: 0 → 1
Char. 113: 0 → 1
Char. 121: 0 → 1
Char. 141: 0 → 1
Char. 142: 1 → 0
Char. 143: 0 → 1
Char. 180: 1 → 0
Char. 230: 0 → 1

**Node 72:**
Char. 29: 1 → 0
Char. 44: 1 → 0
Char. 59: 0 → 1
Char. 60: 0 → 1
Char. 66: 0 → 1
Char. 67: 0 → 1
Char. 72: 1 → 2
Char. 78: 1 → 0
Char. 84: 1 → 0
Char. 88: 1 → 0
Char. 93: 1 → 0
Char. 111: 0 → 1
Char. 123: 1 → 0
Char. 129: 0 → 1
Char. 154: 1 → 0
Char. 169: 1 → 0
Char. 170: 0 → 1
Char. 192: 1 → 0
Char. 197: 0 → 1

**Node 73:**
Char. 83: 0 → 1
Char. 85: 1 → 0
Char. 107: 0 → 1

**Node 74:**
Char. 2: 0 → 1
Char. 6: 0 → 1
Char. 186: 0 → 1
Char. 198: 0 → 1
Char. 200: 0 → 1
Char. 220: 0 → 1
Char. 239: 1 → 0

**Node 75:**
Char. 48: 0 → 1
Char. 98: 1 → 0
Char. 113: 0 → 1
Char. 181: 0 → 1
Char. 206: 0 → 2

**Node 76:**
Char. 35: 0 → 1
Char. 102: 0 → 1
Char. 103: 0 → 1
Char. 184: 0 → 1
Char. 201: 0 → 1
Char. 210: 0 → 1
Char. 223: 0 → 1
Char. 229: 0 → 1

**Node 77:**
Char. 17: 0 → 1
Char. 42: 0 → 1
Char. 251: 0 → 1
Char. 264: 0 → 1

**Node 78:**
Char. 41: 0 → 1
Char. 61: 1 → 2
Char. 112: 1 → 0
Char. 138: 0 → 1
Char. 146: 1 → 0
Char. 155: 0 → 1
Char. 192: 1 → 2
Char. 224: 0 → 1
Char. 226: 0 → 1
Char. 227: 0 → 1
Char. 233: 0 → 1
Char. 267: 01 → 2

**Node 79:**
Char. 61: 1 → 3
Char. 90: 0 → 1
Char. 91: 0 → 1
Char. 109: 1 → 0
Char. 155: 0 → 1
Char. 209: 1 → 0

**ANALYSIS 61**
**(ALL TAXA, IMPLIED WEIGHTING, K = 6)**

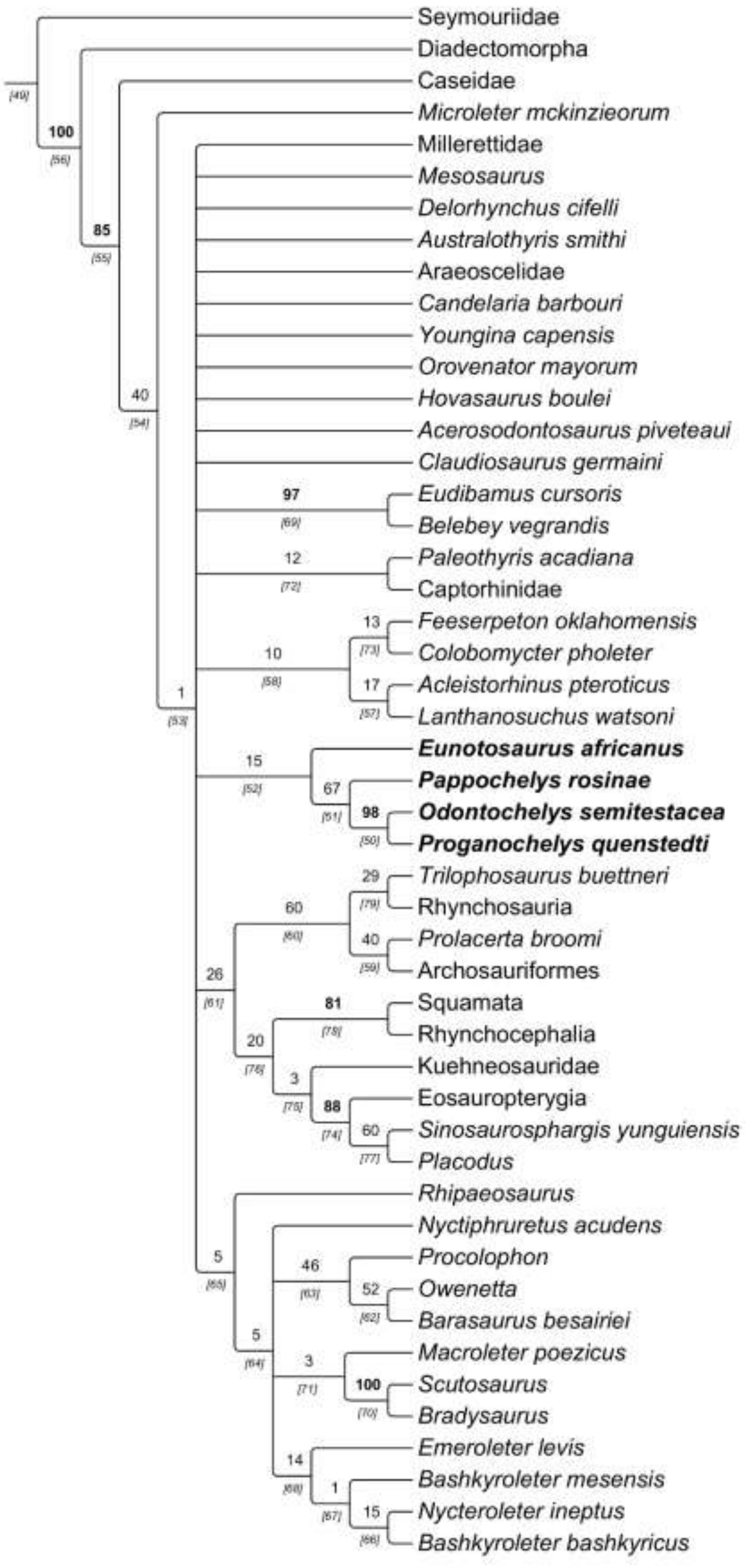

***Proganochelys quenstedti*:**
Char. 8: 0 → 1
Char. 11: 0 → 1
Char. 106: 0 → 1
Char. 108: 1 → 0
Char. 109: 0 → 1
Char. 128: 0 → 1
Char. 175: 0 → 1
Char. 202: 1 → 0
Char. 207: 1 → 0
Char. 209: 1 → 0
Char. 244: 0 → 1
Char. 248: 0 → 1
Char. 250: 0 → 1
Char. 252: 1 → 0
Char. 262: 0 → 1

***Pappochelys rosinae*:**
Char. 0: 2 → 0
Char. 1: 0 → 1
Char. 5: 1 → 0
Char. 12: 0 → 1
Char. 41: 0 → 1
Char. 49: 0 → 1
Char. 75: 0 → 1
Char. 129: 0 → 1
Char. 169: 1 → 0
Char. 260: 2 → 0
Char. 265: 0 → 1

***Odontochelys semitestacea*:**
Char. 43: 0 → 1

***Eunotosaurus africanus*:**
Char. 19: 0 → 1
Char. 43: 0 → 1
Char. 76: 0 → 1
Char. 97: 1 → 0
Char. 103: 0 → 1
Char. 153: 1 → 0
Char. 192: 1 → 0
Char. 202: 1 → 0
Char. 211: 0 → 1
Char. 219: 0 → 1
Char. 237: 1 → 0
Char. 248: 0 → 1
Char. 249: 0 → 2
Char. 250: 0 → 1
Char. 263: 0 → 1
Char. 273: 0 → 1
Char. 274: 0 → 1
Char. 276: 0 → 1
Char. 277: 0 → 1

***Acerosodontosaurus piveteaui*:**
Char. 78: 1 → 0
Char. 81: 1 → 0
Char. 94: 0 → 1
Char. 128: 0 → 1
Char. 129: 0 → 1
Char. 155: 0 → 1
Char. 206: 0 → 2
Char. 208: 0 → 1
Char. 265: 0 → 1
Char. 266: 0 → 1
Char. 278: 3 → 1

***Acleistorhinus pteroticus*:**
Char. 21: 0 → 1
Char. 146: 1 → 0

**Araeoscelidae:**
Char. 5: 1 → 0
Char. 20: 1 → 0
Char. 27: 0 → 1
Char. 28: 0 → 1
Char. 33: 1 → 0
Char. 38: 1 → 0
Char. 40: 2 → 0
Char. 43: 0 → 1
Char. 60: 0 → 1
Char. 72: 1 → 2
Char. 84: 1 → 0
Char. 92: 1 → 0
Char. 106: 0 → 1
Char. 116: 1 → 0
Char. 117: 01 → 2
Char. 154: 1 → 0
Char. 159: 1 → 0
Char. 166: 1 → 0
Char. 169: 1 → 0
Char. 170: 0 → 1
Char. 193: 0 → 1
Char. 197: 0 → 1
Char. 202: 1 → 0
Char. 207: 1 → 0
Char. 209: 1 → 0
Char. 221: 1 → 0
Char. 222: 0 → 1
Char. 224: 0 → 1
Char. 239: 0 → 1
Char. 266: 0 → 1
Char. 278: 3 → 1

**Archosauriformes:**
Char. 32: 0 → 1
Char. 94: 1 → 0
Char. 112: 1 → 0
Char. 152: 0 → 1
Char. 154: 01 → 2
Char. 166: 1 → 0
Char. 171: 0 → 1
Char. 185: 0 → 1
Char. 204: 0 → 1
Char. 218: 0 → 3
Char. 224: 0 → 1
Char. 242: 0 → 1

***Australothyris smithi*:**
Char. 0: 1 → 0
Char. 24: 0 → 1
Char. 29: 0 → 1
Char. 34: 0 → 1
Char. 50: 0 → 1
Char. 55: 1 → 0
Char. 67: 1 → 0
Char. 71: 1 → 0
Char. 79: 0 → 1
Char. 83: 0 → 1
Char. 85: 1 → 0
Char. 98: 1 → 0
Char. 100: 0 → 1
Char. 103: 0 → 1
Char. 110: 0 → 1
Char. 111: 1 → 0
Char. 123: 1 → 0
Char. 129: 0 → 1
Char. 131: 0 → 1
Char. 132: 1 → 0
Char. 144: 1 → 0
Char. 147: 0 → 1
Char. 149: 1 → 0
Char. 150: 0 → 1
Char. 159: 1 → 0
Char. 163: 1 → 0
Char. 192: 1 → 0

***Barasaurus besairiei*:**
Char. 33: 1 → 0

***Bashkyroleter bashkyricus*:**
Char. 275: 1 → 0

***Bashkyroleter mesensis*:**
Char. 169: 1 → 0

***Belebey vegrandis*:**
Char. 154: 1 → 0

***Bradysaurus* spp.:**
Char. 19: 0 → 1
Char. 25: 1 → 0
Char. 73: 0 → 1
Char. 79: 0 → 1
Char. 135: 1 → 0
Char. 249: 0 → 1

***Candelaria barbouri*:**
Char. 0: 1 → 0
Char. 1: 0 → 1
Char. 5: 1 → 0
Char. 8: 0 → 1
Char. 15: 0 → 1
Char. 25: 1 → 0
Char. 33: 1 → 2
Char. 49: 0 → 1
Char. 55: 1 → 0
Char. 76: 0 → 1
Char. 79: 0 → 2
Char. 83: 0 → 1
Char. 88: 1 → 0
Char. 92: 1 → 0
Char. 94: 0 → 1
Char. 95: 0 → 1
Char. 126: 0 → 1
Char. 127: 0 → 1
Char. 132: 1 → 0
Char. 154: 1 → 2
Char. 159: 1 → 0
Char. 169: 1 → 0
Char. 276: 0 → 1
Char. 277: 0 → 1

**Captorhinidae:**
Char. 3: 0 → 1
Char. 25: 1 → 0
Char. 26: 0 → 1
Char. 50: 0 → 1
Char. 73: 0 → 1
Char. 75: 0 → 1
Char. 83: 0 → 1
Char. 108: 1 → 0
Char. 180: 1 → 0
Char. 183: 1 → 0
Char. 201: 1 → 0
Char. 203: 1 → 2
Char. 216: 1 → 0
Char. 237: 1 → 0
Char. 240: 1 → 0

**Caseidae:**
Char. 38: 1 → 0
Char. 46: 1 → 0
Char. 170: 0 → 1
Char. 194: 0 → 1
Char. 273: 0 → 1
Char. 274: 0 → 1
Char. 278: 3 → 2

***Claudiosaurus germaini*:**
Char. 24: 0 → 1
Char. 27: 0 → 1
Char. 34: 0 → 1
Char. 36: 0 → 1
Char. 56: 0 → 1
Char. 60: 0 → 1
Char. 62: 0 → 1
Char. 64: 0 → 1
Char. 70: 0 → 1
Char. 72: 1 → 2
Char. 73: 0 → 1
Char. 84: 1 → 0
Char. 94: 0 → 1
Char. 105: 0 → 1
Char. 106: 0 → 1
Char. 109: 0 → 1
Char. 117: 01 → 2
Char. 126: 0 → 1
Char. 129: 0 → 1
Char. 130: 0 → 1
Char. 131: 0 → 1
Char. 144: 1 → 0
Char. 148: 0 → 1
Char. 154: 1 → 0
Char. 166: 1 → 0
Char. 182: 0 → 1
Char. 188: 0 → 1
Char. 190: 0 → 1
Char. 191: 0 → 1
Char. 199: 0 → 1
Char. 203: 1 → 2
Char. 204: 0 → 1
Char. 214: 0 → 1
Char. 219: 0 → 1
Char. 220: 0 → 1
Char. 234: 1 → 0
Char. 265: 0 → 1
Char. 267: 0 → 1
Char. 272: 2 → 1
Char. 275: 1 → 0
Char. 278: 3 → 0

***Colobomycter pholeter*:**
Char. 21: 0 → 1
Char. 25: 1 → 0
Char. 84: 1 → 0
Char. 154: 1 → 0
Char. 167: 0 → 1
Char. 267: 0 → 1

***Delorhynchus cifelli*:**
Char. 0: 1 → 0
Char. 18: 0 → 1
Char. 21: 0 → 1
Char. 24: 0 → 1
Char. 26: 0 → 1
Char. 28: 0 → 1
Char. 29: 0 → 1
Char. 33: 1 → 2
Char. 39: 0 → 1
Char. 50: 0 → 1
Char. 52: 0 → 1
Char. 57: 0 → 1
Char. 67: 1 → 0
Char. 100: 0 → 1
Char. 116: 1 → 0
Char. 119: 1 → 0
Char. 131: 0 → 1
Char. 147: 0 → 1
Char. 156: 1 → 0
Char. 167: 0 → 1
Char. 189: 0 → 1
Char. 191: 0 → 1
Char. 202: 1 → 0
Char. 204: 0 → 2
Char. 267: 0 → 1

**Diadectomorpha:**
Char. 64: 0 → 1
Char. 70: 0 → 1
Char. 122: 0 → 1
Char. 123: 1 → 0
Char. 146: 1 → 0
Char. 275: 1 → 0
Char. 278: 3 → 0

***Emeroleter levis*:**
Char. 51: 1 → 0

**Eosauropterygia:**
Char. 166: 1 → 0
Char. 174: 0 → 1
Char. 178: 0 → 1
Char. 194: 0 → 2
Char. 272: 1 → 0

***Eudibamus cursoris*:**
Char. 154: 1 → 2
Char. 163: 1 → 0

***Feeserpeton oklahomensis*:**
Char. 51: 0 → 1
Char. 157: 0 → 1
Char. 158: 0 → 1

***Hovasaurus boulei*:**
Char. 41: 0 → 1
Char. 43: 0 → 1
Char. 55: 1 → 0
Char. 77: 0 → 2
Char. 78: 1 → 0
Char. 79: 0 → 1
Char. 93: 1 → 0
Char. 113: 0 → 1
Char. 127: 0 → 1
Char. 135: 0 → 1
Char. 136: 0 → 1
Char. 138: 0 → 1
Char. 146: 1 → 0
Char. 187: 0 → 1
Char. 188: 0 → 1
Char. 191: 0 → 1
Char. 193: 0 → 1
Char. 201: 1 → 0
Char. 204: 0 → 2
Char. 206: 0 → 2
Char. 215: 0 → 1
Char. 220: 0 → 1
Char. 222: 0 → 1
Char. 224: 0 → 1
Char. 265: 0 → 1
Char. 275: 1 → 0

**Kuehneosauridae:**
Char. 7: 0 → 1
Char. 24: 0 → 1
Char. 27: 0 → 1
Char. 43: 1 → 0
Char. 79: 0 → 2
Char. 107: 1 → 0
Char. 140: 1 → 0
Char. 147: 1 → 0
Char. 148: 1 → 0
Char. 245: 0 → 1
Char. 278: 0 → 3

***Lanthanosuchus watsoni*:**
Char. 25: 1 → 0
Char. 51: 0 → 1
Char. 76: 0 → 1
Char. 86: 0 → 1
Char. 98: 1 → 0
Char. 138: 0 → 1
Char. 144: 1 → 0
Char. 154: 1 → 2

***Macroleter poezicus*:**
Char. 9: 0 → 1
Char. 26: 0 → 1
Char. 66: 1 → 2
Char. 126: 0 → 1
Char. 134: 0 → 2
Char. 140: 0 → 1
Char. 146: 1 → 0
Char. 147: 1 → 2
Char. 154: 1 → 0
Char. 169: 1 → 0
Char. 278: 3 → 0

***Mesosaurus* spp.:**
Char. 2: 0 → 1
Char. 5: 1 → 0
Char. 6: 0 → 1
Char. 8: 0 → 1
Char. 9: 0 → 1
Char. 13: 0 → 1
Char. 19: 0 → 1
Char. 20: 1 → 0
Char. 26: 0 → 1
Char. 38: 1 → 0
Char. 41: 0 → 1
Char. 76: 0 → 1
Char. 83: 0 → 1
Char. 84: 1 → 0
Char. 85: 1 → 0
Char. 94: 0 → 1
Char. 107: 0 → 1
Char. 109: 0 → 1
Char. 112: 1 → 0
Char. 115: 0 → 1
Char. 146: 1 → 0
Char. 148: 0 → 1
Char. 149: 1 → 0
Char. 159: 1 → 0
Char. 163: 1 → 0
Char. 164: 0 → 1
Char. 166: 1 → 0
Char. 167: 0 → 1
Char. 176: 0 → 1
Char. 183: 1 → 0
Char. 184: 0 → 1
Char. 192: 1 → 0
Char. 199: 0 → 1
Char. 204: 0 → 2
Char. 206: 0 → 1
Char. 217: 0 → 1
Char. 219: 0 → 1
Char. 220: 0 → 1
Char. 231: 0 → 1
Char. 234: 1 → 0
Char. 237: 1 → 0
Char. 240: 1 → 0
Char. 260: 2 → 0
Char. 272: 2 → 0
Char. 278: 3 → 0

***Microleter mckinzieorum*:**
Char. 18: 0 → 1
Char. 29: 0 → 1
Char. 39: 0 → 1
Char. 70: 0 → 1
Char. 76: 0 → 1
Char. 83: 0 → 1
Char. 94: 0 → 1
Char. 106: 0 → 1
Char. 110: 0 → 1
Char. 278: 3 → 1

**Millerettidae:**
Char. 0: 1 → 0
Char. 5: 1 → 0
Char. 20: 1 → 0
Char. 24: 0 → 1
Char. 25: 1 → 0
Char. 29: 0 → 1
Char. 33: 1 → 0
Char. 44: 1 → 0
Char. 50: 0 → 1
Char. 56: 0 → 1
Char. 66: 0 → 2
Char. 67: 1 → 0
Char. 78: 1 → 0
Char. 80: 1 → 0
Char. 84: 1 → 2
Char. 88: 1 → 0
Char. 96: 1 → 0
Char. 111: 1 → 0
Char. 112: 1 → 0
Char. 120: 1 → 0
Char. 121: 0 → 1
Char. 124: 0 → 1
Char. 127: 0 → 1
Char. 135: 0 → 1
Char. 145: 0 → 1
Char. 159: 1 → 0
Char. 163: 1 → 0
Char. 166: 1 → 0
Char. 180: 1 → 0
Char. 192: 1 → 0
Char. 211: 0 → 1
Char. 230: 0 → 1
Char. 248: 0 → 1
Char. 252: 0 → 1
Char. 253: 0 → 1

***Nycteroleter ineptus*:**
Char. 278: 0 → 3

***Nyctiphruretus acudens*:**
Char. 21: 0 → 1
Char. 33: 1 → 2
Char. 41: 0 → 1
Char. 66: 1 → 2
Char. 81: 1 → 0
Char. 83: 0 → 1
Char. 84: 1 → 2
Char. 94: 0 → 1
Char. 166: 1 → 0
Char. 207: 1 → 0
Char. 224: 0 → 1
Char. 226: 1 → 0
Char. 266: 0 → 1
Char. 272: 2 → 1
Char. 276: 0 → 1

***Orovenator mayorum*:**
Char. 8: 0 → 1
Char. 24: 0 → 1
Char. 25: 1 → 0
Char. 33: 1 → 0
Char. 36: 0 → 1
Char. 50: 0 → 1
Char. 62: 0 → 1
Char. 72: 1 → 2
Char. 92: 1 → 0
Char. 136: 0 → 1
Char. 141: 0 → 1
Char. 159: 1 → 0
Char. 160: 0 → 1
Char. 165: 0 → 1
Char. 278: 3 → 1

***Owenetta* spp.:**
Char. 142: 1 → 0
Char. 169: 1 → 0

***Paleothyris acadiana*:**
Char. 38: 1 → 0
Char. 66: 1 → 2
Char. 102: 0 → 1
Char. 146: 1 → 0
Char. 239: 0 → 1

***Placodus* spp.:**
Char. 0: 1 → 2
Char. 9: 0 → 1
Char. 12: 0 → 1
Char. 13: 0 → 1
Char. 19: 0 → 1
Char. 31: 0 → 1
Char. 46: 1 → 0
Char. 57: 0 → 1
Char. 78: 1 → 0
Char. 93: 1 → 0
Char. 102: 1 → 2
Char. 140: 1 → 0
Char. 155: 0 → 1
Char. 163: 1 → 0
Char. 164: 0 → 1

***Procolophon* spp.:**
Char. 41: 0 → 1
Char. 69: 0 → 1
Char. 79: 0 → 1
Char. 83: 0 → 1
Char. 86: 0 → 1
Char. 88: 0 → 1
Char. 101: 0 → 1
Char. 117: 1 → 0
Char. 141: 0 → 1
Char. 149: 1 → 0
Char. 203: 1 → 2
Char. 204: 0 → 1
Char. 230: 0 → 1
Char. 237: 0 → 1
Char. 238: 0 → 1
Char. 272: 2 → 1
Char. 278: 3 → 0

***Prolacerta broomi*:**
Char. 58: 1 → 0
Char. 66: 1 → 0
Char. 67: 1 → 0
Char. 80: 1 → 0
Char. 139: 1 → 0
Char. 147: 1 → 0
Char. 192: 1 → 0
Char. 203: 1 → 2
Char. 206: 0 → 12

***Rhipaeosaurus* spp.:**
Char. 172: 0 → 1
Char. 207: 1 → 0
Char. 277: 0 → 1

**Rhynchocephalia:**
Char. 0: 1 → 2
Char. 24: 0 → 1
Char. 77: 0 → 1
Char. 88: 1 → 0
Char. 94: 1 → 0
Char. 139: 1 → 0
Char. 167: 1 → 0
Char. 205: 1 → 0

**Rhynchosauria:**
Char. 0: 1 → 0
Char. 7: 0 → 1
Char. 9: 0 → 1
Char. 26: 0 → 1
Char. 44: 1 → 0
Char. 68: 0 → 1
Char. 99: 1 → 0
Char. 150: 1 → 0
Char. 160: 0 → 1
Char. 161: 0 → 1
Char. 171: 0 → 2
Char. 223: 0 → 1
Char. 224: 0 → 1
Char. 241: 0 → 1

***Scutosaurus* spp.:**
Char. 175: 0 → 1
Char. 190: 0 → 1
Char. 218: 0 → 1
Char. 243: 0 → 2
Char. 244: 0 → 1
Char. 251: 0 → 1

***Sinosaurosphargis yunguiensis*:**
Char. 8: 0 → 1
Char. 30: 0 → 1
Char. 53: 0 → 1
Char. 89: 1 → 0
Char. 127: 1 → 0
Char. 150: 1 → 0
Char. 154: 0 → 2
Char. 167: 1 → 0
Char. 253: 0 → 1
Char. 255: 0 → 1

**Squamata:**
Char. 45: 0 → 1
Char. 79: 0 → 2
Char. 80: 1 → 0
Char. 92: 1 → 0
Char. 160: 0 → 1
Char. 200: 0 → 1
Char. 245: 0 → 1

***Trilophosaurus buettneri*:**
Char. 5: 1 → 0
Char. 11: 0 → 1
Char. 55: 1 → 0
Char. 93: 1 → 0
Char. 104: 0 → 1
Char. 106: 0 → 1
Char. 113: 0 → 1
Char. 122: 0 → 1
Char. 144: 1 → 0
Char. 157: 0 → 1
Char. 159: 1 → 0
Char. 177: 0 → 12
Char. 194: 0 → 1
Char. 203: 1 → 2
Char. 207: 1 → 0
Char. 208: 1 → 0
Char. 272: 1 → 0

***Youngina capensis*:**
Char. 5: 1 → 0
Char. 21: 0 → 1
Char. 27: 0 → 1
Char. 38: 1 → 0
Char. 40: 2 → 0
Char. 43: 0 → 1
Char. 44: 1 → 0
Char. 56: 0 → 1
Char. 60: 0 → 1
Char. 62: 0 → 1
Char. 72: 1 → 2
Char. 75: 0 → 1
Char. 84: 1 → 0
Char. 92: 1 → 0
Char. 109: 0 → 1
Char. 127: 0 → 1
Char. 129: 0 → 1
Char. 134: 0 → 1
Char. 135: 0 → 1
Char. 141: 0 → 1
Char. 154: 1 → 0
Char. 163: 1 → 0
Char. 170: 0 → 1
Char. 187: 0 → 1
Char. 191: 0 → 1
Char. 193: 0 → 1
Char. 201: 1 → 0
Char. 211: 0 → 1
Char. 214: 0 → 1
Char. 215: 0 → 1
Char. 219: 0 → 1
Char. 222: 0 → 1
Char. 224: 0 → 1
Char. 231: 0 → 1
Char. 239: 0 → 1
Char. 265: 0 → 1
Char. 267: 0 → 1
Char. 275: 1 → 0
Char. 278: 3 → 0

**Node 50:**
Char. 46: 1 → 0
Char. 88: 1 → 0
Char. 93: 1 → 0
Char. 176: 0 → 1
Char. 195: 0 → 2
Char. 198: 0 → 1
Char. 246: 1 → 2
Char. 259: 0 → 1
Char. 270: 0 → 1

**Node 51:**
Char. 65: 0 → 1
Char. 72: 1 → 2
Char. 131: 0 → 1
Char. 184: 0 → 1
Char. 205: 0 → 1
Char. 210: 0 → 1
Char. 222: 0 → 1
Char. 241: 0 → 1
Char. 246: 0 → 1
Char. 254: 0 → 1
Char. 255: 0 → 1
Char. 256: 0 → 1
Char. 267: 0 → 1
Char. 268: 0 → 1
Char. 269: 0 → 1
Char. 278: 3 → 0

**Node 52:**
Char. 0: 1 → 2
Char. 15: 0 → 1
Char. 21: 0 → 1
Char. 25: 1 → 0
Char. 33: 1 → 2
Char. 44: 1 → 0
Char. 64: 0 → 1
Char. 73: 0 → 1
Char. 84: 1 → 2
Char. 94: 0 → 1
Char. 127: 0 → 1
Char. 130: 0 → 1
Char. 134: 0 → 2
Char. 135: 0 → 1
Char. 136: 0 → 1
Char. 141: 0 → 1
Char. 145: 0 → 1
Char. 147: 0 → 1
Char. 148: 0 → 1
Char. 151: 0 → 1
Char. 152: 0 → 1
Char. 155: 0 → 1
Char. 158: 0 → 1
Char. 161: 0 → 1
Char. 174: 0 → 1
Char. 181: 0 → 1
Char. 188: 0 → 1
Char. 201: 1 → 0
Char. 203: 1 → 2
Char. 204: 0 → 2
Char. 217: 0 → 1
Char. 220: 0 → 1
Char. 231: 0 → 1
Char. 247: 0 → 1
Char. 251: 0 → 1
Char. 252: 0 → 1
Char. 253: 0 → 2

**Node 53:**
Char. 20: 0 → 1
Char. 33: 0 → 1
Char. 67: 0 → 1
Char. 111: 0 → 1
Char. 112: 0 → 1
Char. 132: 0 → 1
Char. 166: 0 → 1
Char. 276: 1 → 0

**Node 54:**
Char. 57: 1 → 0
Char. 84: 0 → 1
Char. 88: 0 → 1
Char. 169: 0 → 1

**Node 55:**
Char. 72: 0 → 1
Char. 81: 0 → 1
Char. 93: 0 → 1
Char. 97: 0 → 1
Char. 104: 1 → 0
Char. 144: 0 → 1
Char. 173: 0 → 1
Char. 183: 0 → 1

**Node 57:**
Char. 95: 0 → 1
Char. 113: 0 → 1
Char. 114: 0 → 1

**Node 58:**
Char. 0: 1 → 0
Char. 29: 0 → 1
Char. 47: 0 → 1
Char. 50: 0 → 1
Char. 57: 0 → 1
Char. 67: 1 → 0
Char. 79: 0 → 1
Char. 110: 0 → 1
Char. 111: 1 → 0
Char. 112: 1 → 0
Char. 131: 0 → 1
Char. 137: 0 → 1
Char. 140: 0 → 2
Char. 147: 0 → 1

**Node 59:**
Char. 19: 0 → 1
Char. 92: 1 → 0

**Node 60:**
Char. 4: 0 → 1
Char. 15: 0 → 1
Char. 29: 0 → 1
Char. 213: 0 → 1
Char. 226: 0 → 2
Char. 228: 0 → 1

**Node 61:**
Char. 25: 1 → 0
Char. 43: 0 → 1
Char. 58: 0 → 1
Char. 60: 0 → 1
Char. 61: 0 → 1
Char. 62: 0 → 1
Char. 66: 0 → 1
Char. 69: 0 → 1
Char. 72: 1 → 2
Char. 73: 0 → 1
Char. 94: 0 → 1
Char. 107: 0 → 1
Char. 126: 0 → 1
Char. 127: 0 → 1
Char. 129: 0 → 1
Char. 131: 0 → 1
Char. 134: 0 → 1
Char. 135: 0 → 1
Char. 140: 0 → 1
Char. 141: 0 → 1
Char. 145: 0 → 1
Char. 147: 0 → 1
Char. 148: 0 → 1
Char. 150: 0 → 1
Char. 151: 0 → 1
Char. 167: 0 → 1
Char. 176: 0 → 1
Char. 188: 0 → 1
Char. 190: 0 → 1
Char. 191: 0 → 1
Char. 205: 0 → 1
Char. 208: 0 → 1
Char. 214: 0 → 1
Char. 219: 0 → 1
Char. 230: 0 → 1
Char. 239: 0 → 1
Char. 260: 2 → 1
Char. 265: 0 → 1
Char. 278: 3 → 0

**Node 62:**
Char. 73: 0 → 1
Char. 131: 1 → 0
Char. 205: 0 → 1
Char. 273: 0 → 1
Char. 276: 0 → 1

**Node 63:**
Char. 0: 1 → 0
Char. 18: 0 → 1
Char. 25: 1 → 0
Char. 37: 0 → 1
Char. 103: 0 → 1
Char. 106: 0 → 1
Char. 107: 0 → 1
Char. 110: 0 → 1
Char. 118: 1 → 0
Char. 125: 1 → 0
Char. 126: 0 → 1
Char. 150: 0 → 1

**Node 64:**
Char. 186: 0 → 1

**Node 65:**
Char. 120: 1 → 0
Char. 148: 0 → 1
Char. 183: 1 → 2
Char. 194: 0 → 1
Char. 201: 1 → 0
Char. 211: 0 → 1
Char. 226: 0 → 1
Char. 234: 1 → 0
Char. 237: 1 → 0
Char. 240: 1 → 0
Char. 252: 0 → 1

**Node 66:**
Char. 87: 0 → 1
Char. 110: 0 → 1

**Node 67:**
Char. 25: 1 → 0
Char. 76: 1 → 0

**Node 68:**
Char. 79: 0 → 1
Char. 86: 0 → 1
Char. 93: 1 → 0
Char. 100: 0 → 1
Char. 113: 0 → 1
Char. 133: 0 → 1
Char. 147: 1 → 2
Char. 154: 1 → 0
Char. 278: 3 → 0

**Node 69:**
Char. 20: 1 → 0
Char. 38: 1 → 2
Char. 39: 0 → 1
Char. 57: 0 → 1
Char. 58: 0 → 1
Char. 60: 0 → 1
Char. 72: 1 → 2
Char. 83: 0 → 1
Char. 85: 1 → 0
Char. 88: 1 → 0
Char. 95: 0 → 1
Char. 104: 0 → 1
Char. 105: 0 → 1
Char. 106: 0 → 2
Char. 107: 0 → 1
Char. 109: 0 → 1
Char. 110: 0 → 1
Char. 146: 1 → 0
Char. 148: 0 → 1
Char. 155: 0 → 2
Char. 183: 1 → 2

**Node 70:**
Char. 0: 1 → 2
Char. 33: 1 → 0
Char. 38: 2 → 1
Char. 39: 1 → 0
Char. 42: 0 → 1
Char. 43: 0 → 1
Char. 46: 1 → 0
Char. 49: 1 → 0
Char. 71: 1 → 0
Char. 83: 0 → 2
Char. 86: 0 → 1
Char. 93: 1 → 0
Char. 101: 0 → 1
Char. 103: 0 → 1

Char. 106: 0 → 1
Char. 161: 0 → 1
Char. 163: 1 → 0
Char. 172: 0 → 2
Char. 174: 0 → 1
Char. 188: 0 → 1
Char. 195: 0 → 1
Char. 203: 1 → 2
Char. 204: 0 → 2
Char. 212: 0 → 1
Char. 216: 1 → 0
Char. 221: 1 → 0
Char. 236: 0 → 1
Char. 238: 0 → 2
Char. 241: 0 → 1
Char. 242: 0 → 1
Char. 273: 0 → 1
Char. 274: 0 → 1
Char. 275: 1 → 0

**Node 71:**
Char. 52: 0 → 1
Char. 75: 0 → 1
Char. 84: 1 → 0
Char. 87: 0 → 1
Char. 100: 0 → 1
Char. 110: 0 → 1
Char. 113: 0 → 1
Char. 121: 0 → 1
Char. 141: 0 → 1
Char. 142: 1 → 0
Char. 143: 0 → 1
Char. 180: 1 → 0
Char. 230: 0 → 1

**Node 72:**
Char. 0: 1 → 0
Char. 5: 1 → 0
Char. 20: 1 → 0
Char. 33: 1 → 0
Char. 44: 1 → 0
Char. 60: 0 → 1
Char. 66: 0 → 1
Char. 72: 1 → 2
Char. 78: 1 → 0
Char. 84: 1 → 0
Char. 88: 1 → 0
Char. 93: 1 → 0
Char. 112: 1 → 0
Char. 123: 1 → 0
Char. 129: 0 → 1
Char. 154: 1 → 0
Char. 159: 1 → 0
Char. 163: 1 → 0
Char. 169: 1 → 0
Char. 170: 0 → 1
Char. 192: 1 → 0
Char. 197: 0 → 1
Char. 202: 1 → 0
Char. 207: 1 → 0
Char. 209: 1 → 0
Char. 221: 1 → 0
Char. 234: 1 → 0

**Node 73:**
Char. 33: 1 → 0
Char. 83: 0 → 1
Char. 85: 1 → 0
Char. 107: 0 → 1

**Node 74:**
Char. 2: 0 → 1
Char. 6: 0 → 1
Char. 186: 0 → 1
Char. 198: 0 → 1
Char. 200: 0 → 1
Char. 220: 0 → 1
Char. 239: 1 → 0

**Node 75:**
Char. 98: 1 → 0
Char. 113: 0 → 1
Char. 181: 0 → 1
Char. 206: 0 → 2

**Node 76:**
Char. 35: 0 → 1
Char. 102: 0 → 1
Char. 103: 0 → 1
Char. 184: 0 → 1
Char. 210: 0 → 1
Char. 223: 0 → 1
Char. 229: 0 → 1

**Node 77:**
Char. 17: 0 → 1
Char. 42: 0 → 1
Char. 251: 0 → 1
Char. 264: 0 → 1

**Node 78:**
Char. 41: 0 → 1
Char. 61: 1 → 2
Char. 112: 1 → 0
Char. 138: 0 → 1
Char. 146: 1 → 0
Char. 155: 0 → 1
Char. 192: 1 → 2
Char. 224: 0 → 1
Char. 226: 0 → 1
Char. 227: 0 → 1
Char. 233: 0 → 1
Char. 267: 01 → 2

**Node 79:**
Char. 61: 1 → 3
Char. 90: 0 → 1
Char. 91: 0 → 1
Char. 155: 0 → 1
Char. 209: 1 → 0

## ANALYSIS 62
## (ALL TAXA, IMPLIED WEIGHTING, K = 10)

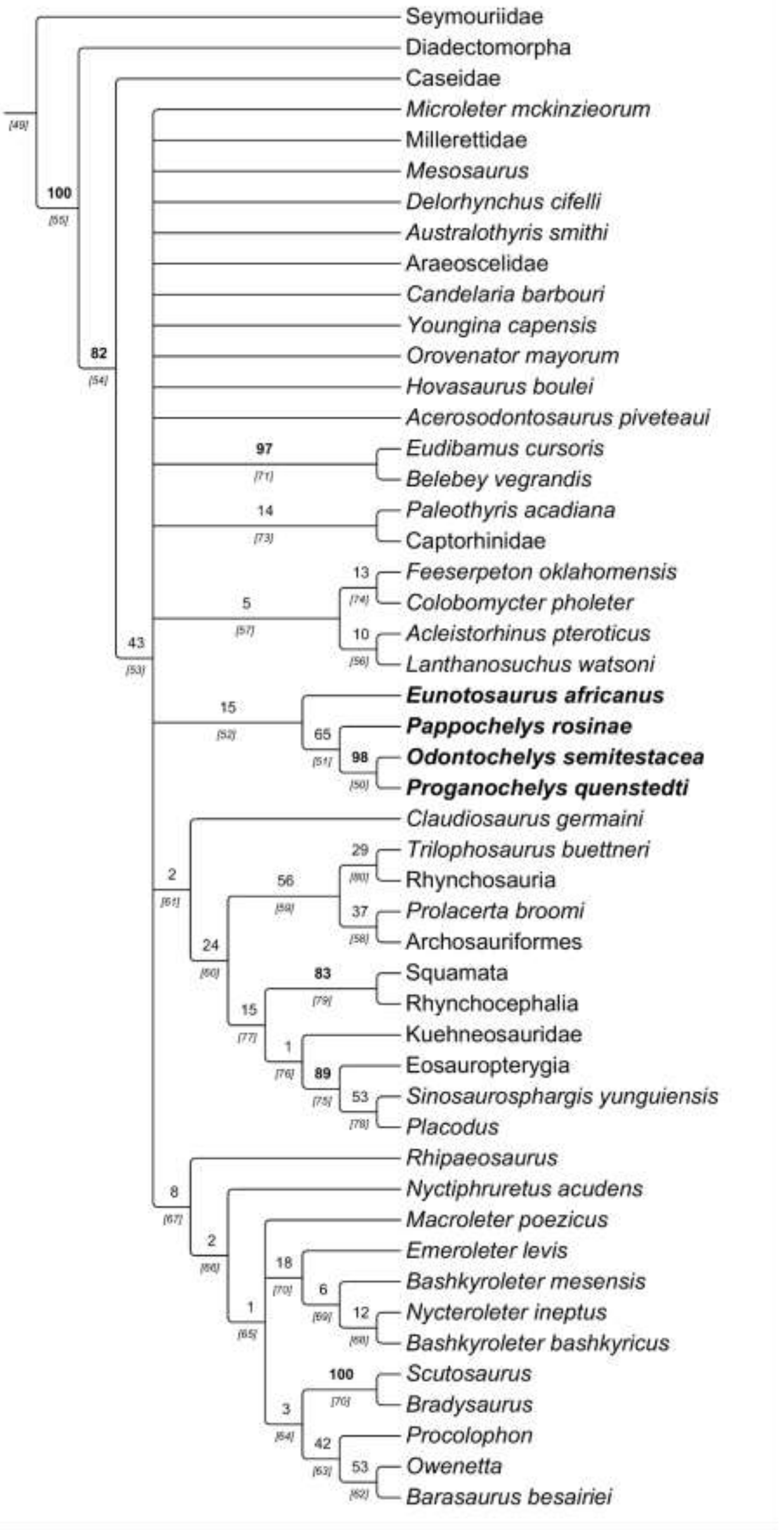

***Proganochelys quenstedti*:**
Char. 8: 0 → 1
Char. 11: 0 → 1
Char. 106: 0 → 1
Char. 108: 1 → 0
Char. 109: 0 → 1
Char. 128: 0 → 1
Char. 175: 0 → 1
Char. 202: 1 → 0
Char. 207: 1 → 0
Char. 209: 1 → 0
Char. 244: 0 → 1
Char. 248: 0 → 1
Char. 250: 0 → 1
Char. 252: 1 → 0
Char. 262: 0 → 1

***Pappochelys rosinae*:**
Char. 1: 0 → 1
Char. 12: 0 → 1
Char. 23: 0 → 1
Char. 41: 0 → 1
Char. 48: 0 → 1
Char. 49: 0 → 1
Char. 75: 0 → 1
Char. 129: 0 → 1
Char. 169: 1 → 0
Char. 260: 2 → 0
Char. 265: 0 → 1

***Odontochelys semitestacea*:**
Char. 43: 0 → 1

***Eunotosaurus africanus*:**
Char. 19: 0 → 1
Char. 43: 0 → 1
Char. 76: 0 → 1
Char. 97: 1 → 0
Char. 103: 0 → 1
Char. 153: 1 → 0
Char. 192: 1 → 0
Char. 202: 1 → 0
Char. 211: 0 → 1
Char. 219: 0 → 1
Char. 248: 0 → 1
Char. 249: 0 → 2
Char. 250: 0 → 1
Char. 263: 0 → 1
Char. 273: 0 → 1
Char. 274: 0 → 1
Char. 276: 0 → 1
Char. 277: 0 → 1

***Acerosodontosaurus piveteaui*:**
Char. 23: 0 → 1
Char. 59: 0 → 1
Char. 78: 1 → 0
Char. 81: 1 → 0
Char. 89: 0 → 1
Char. 94: 0 → 1
Char. 128: 0 → 1
Char. 129: 0 → 1
Char. 155: 0 → 1
Char. 206: 0 → 2
Char. 208: 0 → 1
Char. 265: 0 → 1
Char. 266: 0 → 1
Char. 278: 3 → 1

***Acleistorhinus pteroticus*:**
Char. 21: 0 → 1
Char. 146: 1 → 0

**Araeoscelidae:**
Char. 20: 1 → 0
Char. 23: 0 → 1
Char. 27: 0 → 1
Char. 28: 0 → 1
Char. 38: 1 → 0
Char. 40: 2 → 0
Char. 43: 0 → 1
Char. 48: 0 → 1
Char. 59: 0 → 1
Char. 60: 0 → 1
Char. 72: 1 → 2
Char. 84: 1 → 0
Char. 89: 0 → 1
Char. 106: 0 → 1
Char. 116: 1 → 0
Char. 117: 0 → 2
Char. 154: 1 → 0
Char. 169: 1 → 0
Char. 170: 0 → 1
Char. 193: 0 → 1
Char. 197: 0 → 1
Char. 202: 1 → 0
Char. 207: 1 → 0
Char. 209: 1 → 0
Char. 221: 1 → 0
Char. 224: 0 → 1
Char. 239: 0 → 1
Char. 266: 0 → 1
Char. 278: 3 → 1

**Archosauriformes:**
Char. 32: 0 → 1
Char. 94: 1 → 0
Char. 112: 1 → 0
Char. 152: 0 → 1
Char. 154: 0 → 2
Char. 166: 1 → 0
Char. 171: 0 → 1
Char. 185: 0 → 1
Char. 204: 0 → 1
Char. 218: 0 → 3
Char. 224: 0 → 1
Char. 242: 0 → 1

***Australothyris smithi*:**
Char. 23: 0 → 1
Char. 24: 0 → 1
Char. 29: 0 → 1
Char. 34: 0 → 1
Char. 55: 1 → 0
Char. 71: 1 → 0
Char. 79: 0 → 1
Char. 83: 0 → 1
Char. 85: 1 → 0
Char. 98: 1 → 0
Char. 100: 0 → 1
Char. 103: 0 → 1
Char. 110: 0 → 1
Char. 111: 1 → 0
Char. 123: 1 → 0
Char. 129: 0 → 1
Char. 131: 0 → 1
Char. 132: 1 → 0
Char. 144: 1 → 0
Char. 147: 0 → 1
Char. 149: 1 → 0
Char. 150: 0 → 1
Char. 192: 1 → 0

***Barasaurus besairiei*:**
Char. 33: 1 → 0
Char. 75: 1 → 0
Char. 216: 0 → 1

***Bashkyroleter bashkyricus*:**
Char. 275: 1 → 0

***Bashkyroleter mesensis*:**
Char. 169: 1 → 0

***Belebey vegrandis*:**
Char. 154: 1 → 0

***Bradysaurus* spp.:**
Char. 19: 0 → 1
Char. 73: 0 → 1
Char. 79: 0 → 1
Char. 135: 1 → 0
Char. 249: 0 → 1

***Candelaria barbouri*:**
Char. 1: 0 → 1
Char. 8: 0 → 1
Char. 15: 0 → 1
Char. 23: 0 → 1
Char. 25: 1 → 0
Char. 33: 01 → 2
Char. 49: 0 → 1
Char. 55: 1 → 0
Char. 76: 0 → 1
Char. 79: 0 → 2
Char. 83: 0 → 1
Char. 88: 1 → 0
Char. 89: 0 → 1
Char. 94: 0 → 1
Char. 95: 0 → 1
Char. 126: 0 → 1
Char. 127: 0 → 1
Char. 132: 1 → 0
Char. 154: 1 → 2
Char. 169: 1 → 0
Char. 276: 0 → 1
Char. 277: 0 → 1

**Captorhinidae:**
Char. 3: 0 → 1
Char. 23: 0 → 1
Char. 25: 1 → 0
Char. 26: 0 → 1
Char. 73: 0 → 1
Char. 75: 0 → 1
Char. 83: 0 → 1
Char. 108: 1 → 0
Char. 180: 1 → 0
Char. 183: 1 → 0
Char. 201: 1 → 0
Char. 203: 1 → 2
Char. 216: 1 → 0
Char. 240: 1 → 0

**Caseidae:**
Char. 24: 0 → 1
Char. 25: 1 → 0
Char. 36: 0 → 1
Char. 38: 1 → 0
Char. 46: 1 → 0
Char. 56: 0 → 1
Char. 170: 0 → 1
Char. 194: 0 → 1
Char. 273: 0 → 1
Char. 274: 0 → 1
Char. 278: 3 → 2

***Claudiosaurus germaini*:**
Char. 24: 0 → 1
Char. 27: 0 → 1
Char. 34: 0 → 1
Char. 36: 0 → 1
Char. 48: 0 → 1
Char. 64: 0 → 1
Char. 84: 1 → 0
Char. 105: 0 → 1
Char. 130: 0 → 1
Char. 144: 1 → 0
Char. 199: 0 → 1
Char. 203: 1 → 2
Char. 204: 0 → 1
Char. 220: 0 → 1

***Colobomycter pholeter*:**
Char. 21: 0 → 1
Char. 25: 1 → 0
Char. 84: 1 → 0
Char. 154: 1 → 0
Char. 167: 0 → 1
Char. 267: 0 → 1

***Delorhynchus cifelli*:**
Char. 18: 0 → 1
Char. 21: 0 → 1
Char. 24: 0 → 1
Char. 26: 0 → 1
Char. 28: 0 → 1
Char. 29: 0 → 1
Char. 33: 01 → 2
Char. 39: 0 → 1
Char. 48: 0 → 1
Char. 52: 0 → 1
Char. 57: 0 → 1
Char. 100: 0 → 1
Char. 116: 1 → 0
Char. 117: 0 → 1
Char. 119: 1 → 0
Char. 131: 0 → 1
Char. 147: 0 → 1
Char. 156: 1 → 0
Char. 167: 0 → 1
Char. 189: 0 → 1
Char. 191: 0 → 1
Char. 202: 1 → 0
Char. 204: 0 → 2
Char. 267: 0 → 1

**Diadectomorpha:**
Char. 64: 0 → 1
Char. 70: 0 → 1
Char. 122: 0 → 1
Char. 123: 1 → 0
Char. 146: 1 → 0
Char. 275: 1 → 0
Char. 278: 3 → 0

***Emeroleter levis*:**
Char. 51: 1 → 0

**Eosauropterygia:**
Char. 166: 1 → 0
Char. 174: 0 → 1
Char. 178: 0 → 1
Char. 194: 0 → 2
Char. 272: 1 → 0

***Eudibamus cursoris*:**
Char. 154: 1 → 2

***Feeserpeton oklahomensis*:**
Char. 51: 0 → 1
Char. 157: 0 → 1
Char. 158: 0 → 1

***Hovasaurus boulei*:**
Char. 41: 0 → 1
Char. 43: 0 → 1
Char. 48: 0 → 1
Char. 55: 1 → 0
Char. 59: 0 → 1
Char. 77: 0 → 2
Char. 78: 1 → 0
Char. 79: 0 → 1
Char. 89: 0 → 1
Char. 93: 1 → 0
Char. 113: 0 → 1
Char. 127: 0 → 1
Char. 135: 0 → 1
Char. 136: 0 → 1
Char. 138: 0 → 1
Char. 146: 1 → 0
Char. 187: 0 → 1
Char. 188: 0 → 1
Char. 191: 0 → 1
Char. 193: 0 → 1
Char. 196: 0 → 1
Char. 201: 1 → 0
Char. 204: 0 → 2
Char. 206: 0 → 2
Char. 215: 0 → 1

Char. 220: 0 → 1
Char. 224: 0 → 1
Char. 265: 0 → 1
Char. 275: 1 → 0

**Kuehneosauridae:**
Char. 7: 0 → 1
Char. 24: 0 → 1
Char. 27: 0 → 1
Char. 43: 1 → 0
Char. 79: 0 → 2
Char. 107: 1 → 0
Char. 140: 1 → 0
Char. 147: 1 → 0
Char. 148: 1 → 0
Char. 245: 0 → 1
Char. 272: 1 → 2
Char. 278: 0 → 3

***Lanthanosuchus watsoni*:**
Char. 25: 1 → 0
Char. 51: 0 → 1
Char. 76: 0 → 1
Char. 86: 0 → 1
Char. 98: 1 → 0
Char. 138: 0 → 1
Char. 144: 1 → 0
Char. 154: 1 → 2

***Macroleter poezicus*:**
Char. 9: 0 → 1
Char. 26: 0 → 1
Char. 52: 0 → 1
Char. 66: 1 → 2
Char. 84: 1 → 0
Char. 87: 0 → 1
Char. 134: 0 → 2
Char. 140: 0 → 1
Char. 146: 1 → 0
Char. 169: 1 → 0
Char. 235: 0 → 1

***Mesosaurus* spp.:**
Char. 2: 0 → 1
Char. 6: 0 → 1
Char. 8: 0 → 1
Char. 9: 0 → 1
Char. 13: 0 → 1
Char. 19: 0 → 1
Char. 20: 1 → 0
Char. 23: 0 → 1
Char. 26: 0 → 1
Char. 38: 1 → 0
Char. 41: 0 → 1
Char. 48: 0 → 1
Char. 76: 0 → 1
Char. 83: 0 → 1
Char. 84: 1 → 0
Char. 85: 1 → 0
Char. 94: 0 → 1
Char. 107: 0 → 1
Char. 109: 0 → 1
Char. 112: 1 → 0
Char. 115: 0 → 1
Char. 146: 1 → 0
Char. 148: 0 → 1
Char. 149: 1 → 0
Char. 164: 0 → 1
Char. 167: 0 → 1
Char. 176: 0 → 1
Char. 183: 1 → 0
Char. 184: 0 → 1
Char. 192: 1 → 0
Char. 199: 0 → 1
Char. 204: 0 → 2
Char. 206: 0 → 1
Char. 217: 0 → 1
Char. 219: 0 → 1
Char. 220: 0 → 1
Char. 231: 0 → 1
Char. 234: 1 → 0
Char. 240: 1 → 0
Char. 260: 2 → 0
Char. 272: 2 → 0
Char. 278: 3 → 0

***Microleter mckinzieorum*:**
Char. 18: 0 → 1
Char. 20: 1 → 0
Char. 24: 0 → 1
Char. 25: 1 → 0
Char. 29: 0 → 1
Char. 36: 0 → 1
Char. 39: 0 → 1
Char. 51: 0 → 2
Char. 56: 0 → 1
Char. 70: 0 → 1
Char. 76: 0 → 1
Char. 79: 0 → 1
Char. 83: 0 → 1
Char. 94: 0 → 1
Char. 106: 0 → 1
Char. 110: 0 → 1
Char. 111: 1 → 0
Char. 112: 1 → 0
Char. 132: 1 → 0
Char. 276: 0 → 1
Char. 278: 3 → 1

**Millerettidae:**
Char. 20: 1 → 0
Char. 24: 0 → 1
Char. 25: 1 → 0
Char. 29: 0 → 1
Char. 44: 1 → 0
Char. 56: 0 → 1
Char. 66: 0 → 2
Char. 78: 1 → 0
Char. 80: 1 → 0
Char. 84: 1 → 2
Char. 88: 1 → 0
Char. 96: 1 → 0
Char. 111: 1 → 0
Char. 112: 1 → 0
Char. 117: 0 → 1
Char. 120: 1 → 0
Char. 121: 0 → 1
Char. 124: 0 → 1
Char. 127: 0 → 1
Char. 135: 0 → 1
Char. 145: 0 → 1
Char. 180: 1 → 0
Char. 192: 1 → 0
Char. 211: 0 → 1
Char. 230: 0 → 1
Char. 248: 0 → 1
Char. 252: 0 → 1
Char. 253: 0 → 1

***Nycteroleter ineptus*:**
Char. 278: 0 → 3

***Nyctiphruretus acudens*:**
Char. 21: 0 → 1
Char. 33: 1 → 2
Char. 41: 0 → 1
Char. 66: 1 → 2
Char. 81: 1 → 0
Char. 83: 0 → 1
Char. 84: 1 → 2
Char. 85: 1 → 0
Char. 94: 0 → 1
Char. 167: 0 → 1
Char. 224: 0 → 1
Char. 266: 0 → 1
Char. 272: 2 → 1
Char. 276: 0 → 1

***Orovenator mayorum*:**
Char. 8: 0 → 1
Char. 24: 0 → 1
Char. 25: 1 → 0
Char. 36: 0 → 1
Char. 62: 0 → 1
Char. 72: 1 → 2
Char. 89: 0 → 1
Char. 136: 0 → 1
Char. 141: 0 → 1
Char. 160: 0 → 1
Char. 165: 0 → 1
Char. 278: 3 → 1

***Owenetta* spp.:**
Char. 169: 1 → 0

***Paleothyris acadiana*:**
Char. 38: 1 → 0
Char. 66: 1 → 2
Char. 102: 0 → 1
Char. 146: 1 → 0
Char. 239: 0 → 1

***Placodus* spp.:**
Char. 0: 1 → 2
Char. 9: 0 → 1
Char. 12: 0 → 1
Char. 13: 0 → 1
Char. 19: 0 → 1
Char. 31: 0 → 1
Char. 46: 1 → 0
Char. 57: 0 → 1
Char. 78: 1 → 0
Char. 93: 1 → 0
Char. 102: 1 → 2
Char. 109: 1 → 0
Char. 140: 1 → 0
Char. 155: 0 → 1
Char. 163: 1 → 0
Char. 164: 0 → 1

***Procolophon* spp.:**
Char. 41: 0 → 1
Char. 69: 0 → 1
Char. 79: 0 → 1
Char. 83: 0 → 1
Char. 88: 0 → 1
Char. 117: 1 → 0
Char. 149: 1 → 0
Char. 204: 0 → 1
Char. 237: 0 → 1
Char. 238: 0 → 1
Char. 272: 2 → 1

***Prolacerta broomi*:**
Char. 58: 1 → 0
Char. 66: 1 → 0
Char. 67: 1 → 0
Char. 80: 1 → 0
Char. 139: 1 → 0
Char. 147: 1 → 0
Char. 192: 1 → 0
Char. 203: 1 → 2
Char. 206: 0 → 12

***Rhipaeosaurus* spp.:**
Char. 172: 0 → 1
Char. 277: 0 → 1

**Rhynchocephalia:**
Char. 0: 1 → 2
Char. 23: 1 → 0
Char. 24: 0 → 1
Char. 77: 0 → 1
Char. 88: 1 → 0
Char. 94: 1 → 0
Char. 117: 12 → 0
Char. 139: 1 → 0
Char. 167: 1 → 0
Char. 205: 1 → 0

**Rhynchosauria:**
Char. 0: 1 → 0
Char. 7: 0 → 1
Char. 9: 0 → 1
Char. 26: 0 → 1
Char. 44: 1 → 0
Char. 68: 0 → 1
Char. 99: 1 → 0
Char. 150: 1 → 0
Char. 160: 0 → 1
Char. 161: 0 → 1
Char. 171: 0 → 2
Char. 182: 1 → 0
Char. 223: 0 → 1
Char. 224: 0 → 1
Char. 241: 0 → 1

***Scutosaurus* spp.:**
Char. 175: 0 → 1
Char. 218: 0 → 1
Char. 243: 0 → 2
Char. 244: 0 → 1
Char. 251: 0 → 1

***Sinosaurosphargis yunguiensis*:**
Char. 8: 0 → 1
Char. 30: 0 → 1
Char. 53: 0 → 1
Char. 89: 1 → 0
Char. 127: 1 → 0
Char. 150: 1 → 0
Char. 154: 0 → 2
Char. 167: 1 → 0
Char. 253: 0 → 1
Char. 255: 0 → 1

**Squamata:**
Char. 45: 0 → 1
Char. 79: 0 → 2
Char. 80: 1 → 0
Char. 92: 1 → 0
Char. 109: 1 → 0
Char. 160: 0 → 1
Char. 200: 0 → 1
Char. 245: 0 → 1

***Trilophosaurus buettneri*:**
Char. 5: 1 → 0
Char. 11: 0 → 1
Char. 55: 1 → 0
Char. 93: 1 → 0
Char. 104: 0 → 1
Char. 113: 0 → 1
Char. 122: 0 → 1
Char. 144: 1 → 0
Char. 154: 0 → 1
Char. 157: 0 → 1
Char. 159: 1 → 0
Char. 177: 0 → 12
Char. 194: 0 → 1
Char. 203: 1 → 2
Char. 207: 1 → 0
Char. 208: 1 → 0
Char. 272: 1 → 0

***Youngina capensis*:**
Char. 21: 0 → 1
Char. 27: 0 → 1
Char. 38: 1 → 0
Char. 40: 2 → 0
Char. 43: 0 → 1
Char. 44: 1 → 0
Char. 48: 0 → 1
Char. 56: 0 → 1
Char. 59: 0 → 1
Char. 60: 0 → 1
Char. 62: 0 → 1
Char. 72: 1 → 2
Char. 75: 0 → 1
Char. 84: 1 → 0

Char. 89: 0 → 1
Char. 109: 0 → 1
Char. 127: 0 → 1
Char. 129: 0 → 1
Char. 134: 0 → 1
Char. 135: 0 → 1
Char. 141: 0 → 1
Char. 154: 1 → 0
Char. 170: 0 → 1
Char. 179: 0 → 1
Char. 187: 0 → 1
Char. 191: 0 → 1
Char. 193: 0 → 1
Char. 196: 0 → 1
Char. 201: 1 → 0
Char. 211: 0 → 1
Char. 214: 0 → 1
Char. 215: 0 → 1
Char. 219: 0 → 1
Char. 224: 0 → 1
Char. 231: 0 → 1
Char. 239: 0 → 1
Char. 265: 0 → 1
Char. 267: 0 → 1
Char. 275: 1 → 0
Char. 278: 3 → 0

**Node 50:**
Char. 46: 1 → 0
Char. 88: 1 → 0
Char. 93: 1 → 0
Char. 176: 0 → 1
Char. 195: 0 → 2
Char. 198: 0 → 1
Char. 246: 1 → 2
Char. 259: 0 → 1
Char. 270: 0 → 1

**Node 51:**
Char. 65: 0 → 1
Char. 72: 1 → 2
Char. 131: 0 → 1
Char. 184: 0 → 1
Char. 205: 0 → 1
Char. 210: 0 → 1
Char. 241: 0 → 1
Char. 246: 0 → 1
Char. 254: 0 → 1
Char. 255: 0 → 1
Char. 256: 0 → 1
Char. 267: 0 → 1
Char. 268: 0 → 1
Char. 269: 0 → 1
Char. 278: 3 → 0

**Node 52:**
Char. 15: 0 → 1
Char. 21: 0 → 1
Char. 25: 1 → 0
Char. 33: 01 → 2
Char. 44: 1 → 0
Char. 64: 0 → 1
Char. 73: 0 → 1
Char. 84: 1 → 2

Char. 94: 0 → 1
Char. 127: 0 → 1
Char. 130: 0 → 1
Char. 134: 0 → 2
Char. 135: 0 → 1
Char. 136: 0 → 1
Char. 141: 0 → 1
Char. 145: 0 → 1
Char. 147: 0 → 1
Char. 148: 0 → 1
Char. 151: 0 → 1
Char. 152: 0 → 1
Char. 155: 0 → 1
Char. 158: 0 → 1
Char. 161: 0 → 1
Char. 174: 0 → 1
Char. 181: 0 → 1
Char. 188: 0 → 1
Char. 196: 0 → 1
Char. 201: 1 → 0
Char. 203: 1 → 2
Char. 204: 0 → 2
Char. 217: 0 → 1
Char. 220: 0 → 1
Char. 231: 0 → 1
Char. 247: 0 → 1
Char. 251: 0 → 1
Char. 252: 0 → 1
Char. 253: 0 → 2

**Node 53:**
Char. 20: 0 → 1
Char. 40: 0 → 2
Char. 57: 1 → 0
Char. 74: 0 → 1
Char. 80: 0 → 1
Char. 84: 0 → 1
Char. 88: 0 → 1
Char. 112: 0 → 1
Char. 116: 0 → 1
Char. 119: 0 → 1
Char. 132: 0 → 1
Char. 149: 0 → 1
Char. 156: 0 → 1
Char. 169: 0 → 1
Char. 180: 0 → 1
Char. 192: 0 → 1
Char. 201: 0 → 1
Char. 202: 0 → 1
Char. 203: 0 → 1
Char. 209: 0 → 1
Char. 221: 0 → 1
Char. 234: 0 → 1
Char. 235: 0 → 1

**Node 54:**
Char. 72: 0 → 1
Char. 79: 1 → 0
Char. 81: 0 → 1
Char. 93: 0 → 1
Char. 97: 0 → 1
Char. 104: 1 → 0
Char. 144: 0 → 1

Char. 173: 0 → 1
Char. 183: 0 → 1

**Node 56:**
Char. 95: 0 → 1
Char. 113: 0 → 1
Char. 114: 0 → 1

**Node 57:**
Char. 29: 0 → 1
Char. 47: 0 → 1
Char. 48: 0 → 1
Char. 57: 0 → 1
Char. 79: 0 → 1
Char. 110: 0 → 1
Char. 111: 1 → 0
Char. 112: 1 → 0
Char. 131: 0 → 1
Char. 137: 0 → 1
Char. 140: 0 → 2
Char. 147: 0 → 1

**Node 58:**
Char. 19: 0 → 1
Char. 92: 1 → 0

**Node 59:**
Char. 4: 0 → 1
Char. 15: 0 → 1
Char. 29: 0 → 1
Char. 213: 0 → 1
Char. 226: 0 → 2
Char. 228: 0 → 1

**Node 60:**
Char. 43: 0 → 1
Char. 58: 0 → 1
Char. 61: 0 → 1
Char. 66: 0 → 1
Char. 69: 0 → 1
Char. 107: 0 → 1
Char. 127: 0 → 1
Char. 140: 0 → 1
Char. 141: 0 → 1
Char. 147: 0 → 1
Char. 150: 0 → 1
Char. 167: 0 → 1
Char. 205: 0 → 1
Char. 208: 0 → 1
Char. 230: 0 → 1
Char. 239: 0 → 1

**Node 61:**
Char. 23: 0 → 1
Char. 59: 0 → 1
Char. 60: 0 → 1
Char. 62: 0 → 1
Char. 70: 0 → 1
Char. 72: 1 → 2
Char. 73: 0 → 1
Char. 89: 0 → 1
Char. 94: 0 → 1
Char. 109: 0 → 1
Char. 117: 0 → 12
Char. 126: 0 → 1

Char. 129: 0 → 1
Char. 131: 0 → 1
Char. 148: 0 → 1
Char. 154: 1 → 0
Char. 179: 0 → 1
Char. 182: 0 → 1
Char. 188: 0 → 1
Char. 190: 0 → 1
Char. 191: 0 → 1
Char. 196: 0 → 1
Char. 214: 0 → 1
Char. 219: 0 → 1
Char. 265: 0 → 1
Char. 267: 0 → 1
Char. 272: 2 → 1
Char. 278: 3 → 0

**Node 62:**
Char. 73: 0 → 1
Char. 131: 1 → 0
Char. 205: 0 → 1
Char. 276: 0 → 1

**Node 63:**
Char. 18: 0 → 1
Char. 37: 0 → 1
Char. 100: 1 → 0
Char. 107: 0 → 1
Char. 113: 1 → 0
Char. 118: 1 → 0
Char. 125: 1 → 0
Char. 150: 0 → 1

**Node 64:**
Char. 23: 0 → 1
Char. 71: 1 → 0
Char. 85: 1 → 0
Char. 102: 0 → 1
Char. 103: 0 → 1
Char. 106: 0 → 1
Char. 167: 0 → 1
Char. 214: 0 → 1
Char. 216: 1 → 0
Char. 235: 0 → 2
Char. 241: 0 → 1

**Node 65:**
Char. 48: 0 → 1
Char. 100: 0 → 1
Char. 113: 0 → 1
Char. 132: 1 → 0
Char. 138: 0 → 1
Char. 141: 0 → 1

**Node 66:**
Char. 186: 0 → 1

**Node 67:**
Char. 120: 1 → 0
Char. 148: 0 → 1
Char. 183: 1 → 2
Char. 194: 0 → 1
Char. 201: 1 → 0
Char. 211: 0 → 1
Char. 234: 1 → 0

Char. 235: 1 → 0
Char. 240: 1 → 0
Char. 252: 0 → 1

**Node 68:**
Char. 87: 0 → 1

**Node 69:**
Char. 25: 1 → 0
Char. 76: 1 → 0

**Node 70:**
Char. 79: 0 → 1
Char. 93: 1 → 0
Char. 133: 0 → 1

**Node 71:**
Char. 20: 1 → 0
Char. 38: 1 → 2
Char. 39: 0 → 1
Char. 57: 0 → 1
Char. 58: 0 → 1
Char. 59: 0 → 1
Char. 60: 0 → 1
Char. 72: 1 → 2
Char. 83: 0 → 1
Char. 85: 1 → 0
Char. 88: 1 → 0
Char. 95: 0 → 1
Char. 104: 0 → 1
Char. 105: 0 → 1
Char. 106: 0 → 2
Char. 107: 0 → 1
Char. 109: 0 → 1
Char. 110: 0 → 1
Char. 146: 1 → 0
Char. 148: 0 → 1
Char. 155: 0 → 2
Char. 183: 1 → 2

**Node 72:**
Char. 33: 1 → 0
Char. 38: 2 → 1
Char. 39: 1 → 0
Char. 42: 0 → 1
Char. 43: 0 → 1
Char. 46: 1 → 0
Char. 48: 1 → 0
Char. 49: 1 → 0
Char. 52: 0 → 1
Char. 83: 0 → 2
Char. 84: 1 → 0
Char. 87: 0 → 1
Char. 93: 1 → 0
Char. 132: 0 → 1
Char. 161: 0 → 1
Char. 163: 1 → 0
Char. 172: 0 → 2
Char. 174: 0 → 1
Char. 188: 0 → 1
Char. 189: 0 → 1
Char. 195: 0 → 1
Char. 204: 0 → 2
Char. 212: 0 → 1
Char. 221: 1 → 0

Char. 236: 0 → 1
Char. 238: 0 → 2
Char. 242: 0 → 1
Char. 245: 0 → 1
Char. 274: 0 → 1
Char. 275: 1 → 0

**Node 73:**
Char. 20: 1 → 0
Char. 44: 1 → 0
Char. 59: 0 → 1
Char. 60: 0 → 1
Char. 66: 0 → 1
Char. 72: 1 → 2
Char. 78: 1 → 0
Char. 84: 1 → 0
Char. 88: 1 → 0
Char. 93: 1 → 0
Char. 112: 1 → 0
Char. 123: 1 → 0
Char. 129: 0 → 1
Char. 154: 1 → 0
Char. 169: 1 → 0
Char. 170: 0 → 1
Char. 192: 1 → 0
Char. 197: 0 → 1
Char. 202: 1 → 0
Char. 207: 1 → 0
Char. 209: 1 → 0
Char. 221: 1 → 0
Char. 234: 1 → 0

**Node 74:**
Char. 83: 0 → 1
Char. 85: 1 → 0
Char. 107: 0 → 1

**Node 75:**
Char. 2: 0 → 1
Char. 6: 0 → 1
Char. 186: 0 → 1
Char. 198: 0 → 1
Char. 200: 0 → 1
Char. 220: 0 → 1
Char. 239: 1 → 0
Char. 267: 1 → 0

**Node 76:**
Char. 48: 0 → 1
Char. 98: 1 → 0
Char. 113: 0 → 1
Char. 181: 0 → 1
Char. 206: 0 → 2

**Node 77:**
Char. 35: 0 → 1
Char. 102: 0 → 1
Char. 103: 0 → 1
Char. 184: 0 → 1
Char. 210: 0 → 1
Char. 223: 0 → 1
Char. 229: 0 → 1

**Node 78:**
Char. 17: 0 → 1
Char. 42: 0 → 1
Char. 251: 0 → 1
Char. 264: 0 → 1

**Node 79:**
Char. 41: 0 → 1
Char. 61: 1 → 2
Char. 112: 1 → 0
Char. 138: 0 → 1
Char. 146: 1 → 0
Char. 155: 0 → 1
Char. 182: 1 → 0
Char. 192: 1 → 2
Char. 224: 0 → 1
Char. 226: 0 → 1
Char. 227: 0 → 1
Char. 233: 0 → 1
Char. 267: 1 → 2

**Node 80:**
Char. 61: 1 → 3
Char. 90: 0 → 1
Char. 91: 0 → 1
Char. 109: 1 → 0
Char. 155: 0 → 1
Char. 209: 1 → 0

**ANALYSIS 63**
**(ALL TAXA, IMPLIED WEIGHTING, K = 100)**

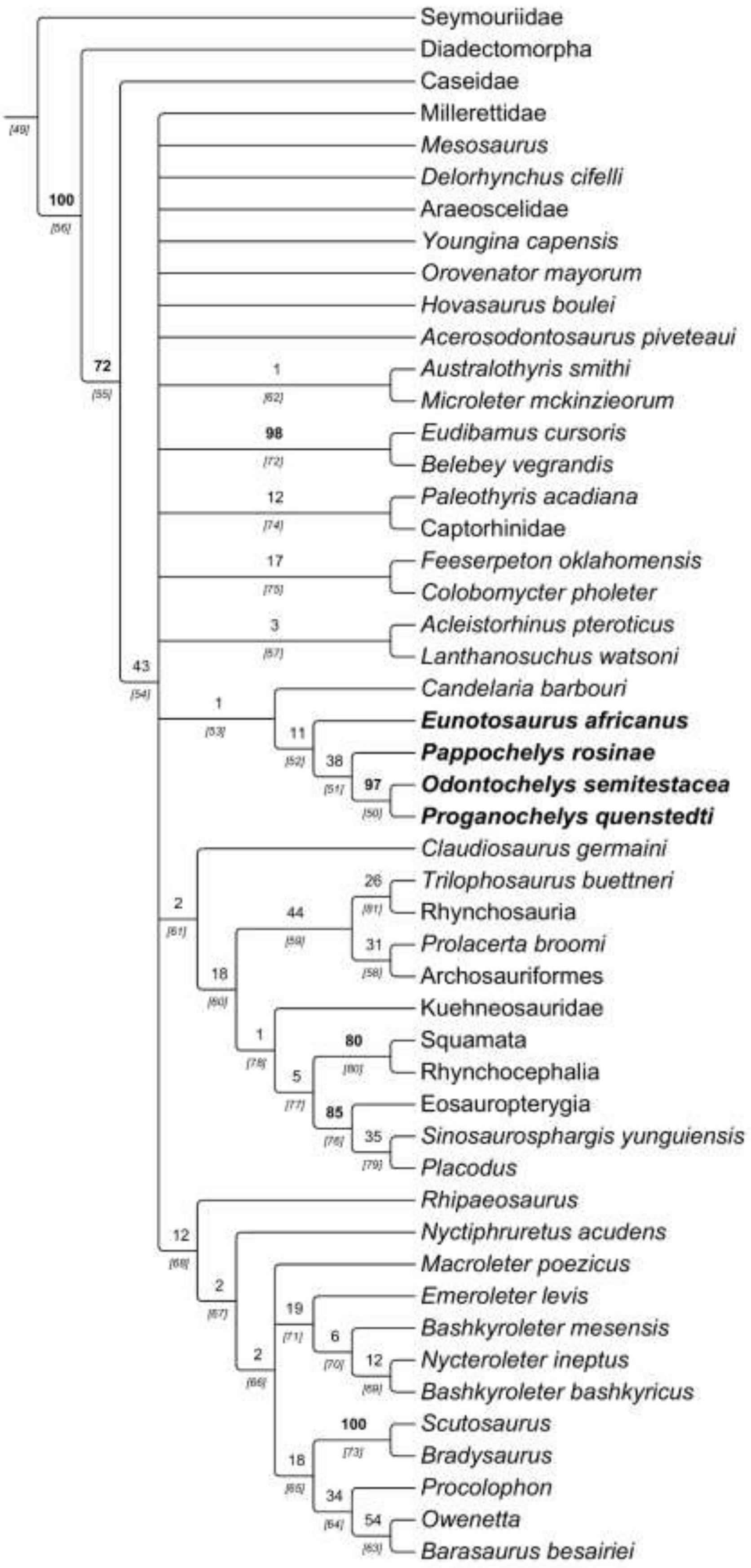

***Proganochelys quenstedti*:**
Char. 8: 0 → 1
Char. 11: 0 → 1
Char. 106: 0 → 1
Char. 108: 1 → 0
Char. 109: 0 → 1
Char. 128: 0 → 1
Char. 175: 0 → 1
Char. 202: 1 → 0
Char. 207: 1 → 0
Char. 209: 1 → 0
Char. 244: 0 → 1
Char. 248: 0 → 1
Char. 250: 0 → 1
Char. 252: 1 → 0
Char. 262: 0 → 1

***Pappochelys rosinae*:**
Char. 1: 0 → 1
Char. 5: 1 → 0
Char. 12: 0 → 1
Char. 23: 0 → 1
Char. 41: 0 → 1
Char. 48: 0 → 1
Char. 49: 0 → 1
Char. 75: 0 → 1
Char. 129: 0 → 1
Char. 169: 1 → 0
Char. 260: 2 → 0
Char. 265: 0 → 1

***Odontochelys semitestacea*:**
Char. 43: 0 → 1

***Eunotosaurus africanus*:**
Char. 19: 0 → 1
Char. 43: 0 → 1
Char. 97: 1 → 0
Char. 103: 0 → 1
Char. 153: 1 → 0
Char. 192: 1 → 0
Char. 202: 1 → 0
Char. 211: 0 → 1
Char. 219: 0 → 1
Char. 248: 0 → 1
Char. 249: 0 → 2
Char. 250: 0 → 1
Char. 263: 0 → 1
Char. 273: 0 → 1
Char. 274: 0 → 1

***Acerosodontosaurus piveteaui*:**
Char. 23: 0 → 1
Char. 59: 0 → 1
Char. 78: 1 → 0
Char. 81: 1 → 0
Char. 89: 0 → 1
Char. 94: 0 → 1
Char. 128: 0 → 1
Char. 129: 0 → 1
Char. 155: 0 → 1
Char. 206: 0 → 2
Char. 208: 0 → 1
Char. 265: 0 → 1
Char. 266: 0 → 1
Char. 278: 3 → 1

***Acleistorhinus pteroticus*:**
Char. 21: 0 → 1
Char. 146: 1 → 0
Char. 169: 1 → 0
Char. 170: 0 → 1

**Araeoscelidae:**
Char. 0: 0 → 1
Char. 5: 1 → 0
Char. 20: 1 → 0
Char. 23: 0 → 1
Char. 27: 0 → 1
Char. 28: 0 → 1
Char. 38: 1 → 0
Char. 40: 2 → 0
Char. 43: 0 → 1
Char. 59: 0 → 1
Char. 60: 0 → 1
Char. 72: 1 → 2
Char. 84: 1 → 0
Char. 89: 0 → 1
Char. 106: 0 → 1
Char. 116: 1 → 0
Char. 117: 0 → 2
Char. 154: 1 → 0
Char. 159: 1 → 0
Char. 166: 1 → 0
Char. 169: 1 → 0
Char. 170: 0 → 1
Char. 193: 0 → 1
Char. 197: 0 → 1
Char. 202: 1 → 0
Char. 207: 1 → 0
Char. 209: 1 → 0
Char. 221: 1 → 0
Char. 224: 0 → 1
Char. 239: 0 → 1
Char. 266: 0 → 1
Char. 278: 3 → 1

**Archosauriformes:**
Char. 32: 0 → 1
Char. 94: 1 → 0
Char. 112: 1 → 0
Char. 152: 0 → 1
Char. 154: 0 → 2
Char. 166: 1 → 0
Char. 171: 0 → 1
Char. 185: 0 → 1
Char. 204: 0 → 1
Char. 218: 0 → 3
Char. 224: 0 → 1
Char. 242: 0 → 1

***Australothyris smithi*:**
Char. 23: 0 → 1
Char. 34: 0 → 1
Char. 55: 1 → 0
Char. 71: 1 → 0
Char. 85: 1 → 0
Char. 129: 0 → 1
Char. 131: 0 → 1
Char. 159: 1 → 0

***Barasaurus besairiei*:**
Char. 33: 1 → 0
Char. 75: 1 → 0
Char. 216: 0 → 1

***Bashkyroleter bashkyricus*:**
Char. 275: 1 → 0

***Bashkyroleter mesensis*:**
Char. 169: 1 → 0

***Belebey vegrandis*:**
Char. 154: 1 → 0

***Bradysaurus* spp.:**
Char. 19: 0 → 1
Char. 73: 0 → 1
Char. 79: 0 → 1
Char. 135: 1 → 0
Char. 249: 0 → 1

***Candelaria barbouri*:**
Char. 1: 0 → 1
Char. 5: 1 → 0
Char. 8: 0 → 1
Char. 23: 0 → 1
Char. 49: 0 → 1
Char. 79: 0 → 2
Char. 83: 0 → 1
Char. 88: 1 → 0
Char. 95: 0 → 1
Char. 132: 1 → 0
Char. 154: 1 → 2
Char. 159: 1 → 0
Char. 169: 1 → 0

**Captorhinidae:**
Char. 3: 0 → 1
Char. 23: 0 → 1
Char. 25: 1 → 0
Char. 26: 0 → 1
Char. 73: 0 → 1
Char. 75: 0 → 1
Char. 83: 0 → 1
Char. 108: 1 → 0
Char. 180: 1 → 0
Char. 183: 1 → 0
Char. 201: 1 → 0
Char. 203: 1 → 2
Char. 216: 1 → 0
Char. 240: 1 → 0

**Caseidae:**
Char. 24: 0 → 1
Char. 25: 1 → 0
Char. 36: 0 → 1
Char. 38: 1 → 0
Char. 46: 1 → 0
Char. 56: 0 → 1
Char. 170: 0 → 1
Char. 194: 0 → 1
Char. 273: 0 → 1
Char. 274: 0 → 1
Char. 278: 3 → 2

***Claudiosaurus germaini*:**
Char. 24: 0 → 1
Char. 27: 0 → 1
Char. 34: 0 → 1
Char. 36: 0 → 1
Char. 64: 0 → 1
Char. 84: 1 → 0
Char. 105: 0 → 1
Char. 130: 0 → 1
Char. 144: 1 → 0
Char. 166: 1 → 0
Char. 199: 0 → 1
Char. 203: 1 → 2
Char. 204: 0 → 1
Char. 220: 0 → 1

***Colobomycter pholeter*:**
Char. 21: 0 → 1
Char. 25: 1 → 0
Char. 84: 1 → 0
Char. 154: 1 → 0
Char. 167: 0 → 1
Char. 267: 0 → 1

***Delorhynchus cifelli*:**
Char. 18: 0 → 1
Char. 21: 0 → 1
Char. 24: 0 → 1
Char. 26: 0 → 1
Char. 28: 0 → 1
Char. 29: 0 → 1
Char. 33: 0 → 2
Char. 39: 0 → 1
Char. 52: 0 → 1
Char. 100: 0 → 1
Char. 116: 1 → 0
Char. 117: 0 → 1
Char. 119: 1 → 0
Char. 131: 0 → 1
Char. 147: 0 → 1
Char. 156: 1 → 0
Char. 167: 0 → 1
Char. 189: 0 → 1
Char. 191: 0 → 1
Char. 202: 1 → 0
Char. 204: 0 → 2
Char. 267: 0 → 1

**Diadectomorpha:**
Char. 0: 0 → 1
Char. 64: 0 → 1
Char. 70: 0 → 1
Char. 122: 0 → 1
Char. 123: 1 → 0
Char. 146: 1 → 0
Char. 275: 1 → 0
Char. 278: 3 → 0

***Emeroleter levis*:**
Char. 0: 0 → 2
Char. 51: 1 → 0

**Eosauropterygia:**
Char. 159: 1 → 0
Char. 166: 1 → 0
Char. 174: 0 → 1
Char. 194: 0 → 2
Char. 272: 1 → 0

***Eudibamus cursoris*:**
Char. 154: 1 → 2
Char. 163: 1 → 0

***Feeserpeton oklahomensis*:**
Char. 51: 0 → 1
Char. 70: 0 → 1
Char. 157: 0 → 1
Char. 158: 0 → 1

***Hovasaurus boulei*:**
Char. 41: 0 → 1
Char. 43: 0 → 1
Char. 55: 1 → 0
Char. 59: 0 → 1
Char. 77: 0 → 2
Char. 78: 1 → 0
Char. 79: 0 → 1
Char. 89: 0 → 1
Char. 93: 1 → 0
Char. 113: 0 → 1
Char. 127: 0 → 1
Char. 135: 0 → 1
Char. 136: 0 → 1
Char. 138: 0 → 1
Char. 146: 1 → 0
Char. 187: 0 → 1
Char. 188: 0 → 1
Char. 191: 0 → 1
Char. 193: 0 → 1
Char. 196: 0 → 1
Char. 201: 1 → 0
Char. 204: 0 → 2
Char. 206: 0 → 2
Char. 215: 0 → 1
Char. 220: 0 → 1
Char. 224: 0 → 1
Char. 265: 0 → 1
Char. 275: 1 → 0

**Kuehneosauridae:**
Char. 7: 0 → 1
Char. 24: 0 → 1
Char. 27: 0 → 1
Char. 34: 0 → 1
Char. 36: 0 → 1
Char. 44: 1 → 0
Char. 79: 0 → 2
Char. 148: 1 → 0
Char. 159: 1 → 0
Char. 185: 0 → 1
Char. 245: 0 → 1
Char. 272: 1 → 2
Char. 278: 0 → 3

***Lanthanosuchus watsoni*:**
Char. 25: 1 → 0
Char. 51: 0 → 1
Char. 76: 0 → 1
Char. 86: 0 → 1
Char. 98: 1 → 0
Char. 119: 1 → 0
Char. 138: 0 → 1
Char. 144: 1 → 0
Char. 154: 1 → 2

***Macroleter poezicus*:**
Char. 0: 0 → 1
Char. 9: 0 → 1
Char. 26: 0 → 1
Char. 52: 0 → 1
Char. 66: 1 → 2
Char. 84: 1 → 0
Char. 87: 0 → 1
Char. 134: 0 → 2
Char. 140: 0 → 1
Char. 146: 1 → 0
Char. 169: 1 → 0
Char. 235: 0 → 1

***Mesosaurus* spp.:**
Char. 0: 0 → 1
Char. 2: 0 → 1
Char. 5: 1 → 0
Char. 6: 0 → 1
Char. 8: 0 → 1
Char. 9: 0 → 1
Char. 13: 0 → 1
Char. 19: 0 → 1
Char. 20: 1 → 0
Char. 23: 0 → 1
Char. 26: 0 → 1
Char. 33: 0 → 1
Char. 38: 1 → 0
Char. 41: 0 → 1
Char. 76: 0 → 1
Char. 83: 0 → 1
Char. 84: 1 → 0
Char. 85: 1 → 0
Char. 94: 0 → 1
Char. 107: 0 → 1
Char. 109: 0 → 1
Char. 115: 0 → 1
Char. 146: 1 → 0
Char. 148: 0 → 1
Char. 149: 1 → 0
Char. 159: 1 → 0
Char. 163: 1 → 0
Char. 164: 0 → 1
Char. 166: 1 → 0
Char. 167: 0 → 1
Char. 176: 0 → 1
Char. 183: 1 → 0
Char. 184: 0 → 1
Char. 192: 1 → 0
Char. 199: 0 → 1
Char. 204: 0 → 2
Char. 206: 0 → 1
Char. 217: 0 → 1
Char. 219: 0 → 1
Char. 220: 0 → 1
Char. 231: 0 → 1
Char. 234: 1 → 0
Char. 240: 1 → 0
Char. 260: 2 → 0
Char. 272: 2 → 0
Char. 278: 3 → 0

***Microleter mckinzieorum*:**
Char. 0: 0 → 1
Char. 25: 1 → 0
Char. 36: 0 → 1
Char. 39: 0 → 1
Char. 51: 0 → 2
Char. 56: 0 → 1
Char. 70: 0 → 1
Char. 94: 0 → 1
Char. 106: 0 → 1
Char. 166: 1 → 0
Char. 276: 0 → 1

**Millerettidae:**
Char. 5: 1 → 0
Char. 20: 1 → 0
Char. 24: 0 → 1
Char. 25: 1 → 0
Char. 29: 0 → 1
Char. 44: 1 → 0
Char. 48: 1 → 0
Char. 56: 0 → 1
Char. 66: 0 → 2
Char. 78: 1 → 0
Char. 80: 1 → 0
Char. 84: 1 → 2
Char. 88: 1 → 0
Char. 96: 1 → 0
Char. 111: 1 → 0
Char. 117: 0 → 1
Char. 120: 1 → 0
Char. 121: 0 → 1
Char. 124: 0 → 1
Char. 127: 0 → 1
Char. 135: 0 → 1
Char. 145: 0 → 1
Char. 159: 1 → 0
Char. 163: 1 → 0
Char. 166: 1 → 0
Char. 180: 1 → 0
Char. 192: 1 → 0
Char. 211: 0 → 1
Char. 230: 0 → 1
Char. 248: 0 → 1
Char. 252: 0 → 1
Char. 253: 0 → 1

***Nycteroleter ineptus*:**
Char. 278: 0 → 3

***Nyctiphruretus acudens*:**
Char. 0: 0 → 1
Char. 21: 0 → 1
Char. 33: 1 → 2
Char. 41: 0 → 1
Char. 48: 1 → 0
Char. 66: 1 → 2
Char. 81: 1 → 0
Char. 83: 0 → 1
Char. 84: 1 → 2
Char. 85: 1 → 0
Char. 94: 0 → 1
Char. 166: 1 → 0
Char. 167: 0 → 1
Char. 224: 0 → 1
Char. 266: 0 → 1
Char. 272: 2 → 1
Char. 276: 0 → 1

***Orovenator mayorum*:**
Char. 0: 0 → 1
Char. 8: 0 → 1
Char. 24: 0 → 1
Char. 25: 1 → 0
Char. 36: 0 → 1
Char. 48: 1 → 0
Char. 62: 0 → 1
Char. 72: 1 → 2
Char. 89: 0 → 1
Char. 136: 0 → 1
Char. 141: 0 → 1
Char. 159: 1 → 0
Char. 160: 0 → 1
Char. 165: 0 → 1
Char. 278: 3 → 1

***Owenetta* spp.:**
Char. 169: 1 → 0

***Paleothyris acadiana*:**
Char. 38: 1 → 0
Char. 66: 1 → 2
Char. 102: 0 → 1
Char. 146: 1 → 0
Char. 239: 0 → 1

***Placodus* spp.:**
Char. 0: 1 → 2
Char. 9: 0 → 1
Char. 12: 0 → 1
Char. 13: 0 → 1
Char. 19: 0 → 1
Char. 31: 0 → 1
Char. 44: 1 → 0
Char. 46: 1 → 0
Char. 57: 0 → 1
Char. 78: 1 → 0
Char. 93: 1 → 0
Char. 102: 1 → 2
Char. 109: 1 → 0
Char. 140: 1 → 0
Char. 155: 0 → 1
Char. 163: 1 → 0
Char. 164: 0 → 1

***Procolophon* spp.:**
Char. 41: 0 → 1
Char. 69: 0 → 1
Char. 79: 0 → 1
Char. 83: 0 → 1
Char. 88: 0 → 1
Char. 117: 1 → 0
Char. 149: 1 → 0
Char. 204: 0 → 1
Char. 237: 0 → 1
Char. 238: 0 → 1
Char. 272: 2 → 1

***Prolacerta broomi*:**
Char. 58: 1 → 0
Char. 66: 1 → 0
Char. 67: 1 → 0
Char. 80: 1 → 0
Char. 139: 1 → 0
Char. 192: 1 → 0
Char. 203: 1 → 2
Char. 206: 0 → 12

***Rhipaeosaurus* spp.:**
Char. 172: 0 → 1
Char. 277: 0 → 1

**Rhynchocephalia:**
Char. 0: 1 → 2
Char. 24: 0 → 1
Char. 77: 0 → 1
Char. 94: 1 → 0
Char. 117: 12 → 0
Char. 139: 1 → 0
Char. 167: 1 → 0
Char. 205: 1 → 0

**Rhynchosauria:**
Char. 0: 1 → 0
Char. 7: 0 → 1
Char. 9: 0 → 1
Char. 26: 0 → 1
Char. 44: 1 → 0
Char. 68: 0 → 1
Char. 99: 1 → 0
Char. 150: 1 → 0
Char. 160: 0 → 1
Char. 161: 0 → 1
Char. 171: 0 → 2
Char. 182: 1 → 0
Char. 223: 0 → 1
Char. 224: 0 → 1
Char. 241: 0 → 1

***Scutosaurus* spp.:**
Char. 175: 0 → 1
Char. 218: 0 → 1
Char. 243: 0 → 2
Char. 244: 0 → 1
Char. 251: 0 → 1

***Sinosaurosphargis yunguiensis*:**
Char. 8: 0 → 1
Char. 30: 0 → 1
Char. 53: 0 → 1
Char. 89: 1 → 0
Char. 127: 1 → 0
Char. 150: 1 → 0
Char. 154: 0 → 2
Char. 167: 1 → 0
Char. 253: 0 → 1
Char. 255: 0 → 1

**Squamata:**
Char. 45: 0 → 1
Char. 79: 0 → 2
Char. 80: 1 → 0
Char. 92: 1 → 0
Char. 109: 1 → 0
Char. 160: 0 → 1
Char. 245: 0 → 1

***Trilophosaurus buettneri*:**
Char. 5: 1 → 0
Char. 11: 0 → 1
Char. 55: 1 → 0
Char. 93: 1 → 0
Char. 104: 0 → 1
Char. 113: 0 → 1
Char. 122: 0 → 1
Char. 144: 1 → 0
Char. 154: 0 → 1
Char. 157: 0 → 1
Char. 159: 1 → 0
Char. 177: 0 → 12
Char. 194: 0 → 1
Char. 203: 1 → 2
Char. 207: 1 → 0
Char. 208: 1 → 0
Char. 272: 1 → 0

***Youngina capensis*:**
Char. 0: 0 → 1
Char. 5: 1 → 0
Char. 21: 0 → 1
Char. 27: 0 → 1
Char. 33: 0 → 1
Char. 38: 1 → 0
Char. 40: 2 → 0
Char. 43: 0 → 1
Char. 44: 1 → 0
Char. 56: 0 → 1
Char. 59: 0 → 1
Char. 60: 0 → 1
Char. 62: 0 → 1
Char. 72: 1 → 2
Char. 75: 0 → 1
Char. 84: 1 → 0
Char. 89: 0 → 1
Char. 109: 0 → 1
Char. 127: 0 → 1
Char. 129: 0 → 1
Char. 134: 0 → 1
Char. 135: 0 → 1
Char. 141: 0 → 1
Char. 154: 1 → 0
Char. 163: 1 → 0
Char. 170: 0 → 1
Char. 179: 0 → 1
Char. 187: 0 → 1
Char. 191: 0 → 1
Char. 193: 0 → 1
Char. 196: 0 → 1

Char. 201: 1 → 0
Char. 211: 0 → 1
Char. 214: 0 → 1
Char. 215: 0 → 1
Char. 219: 0 → 1
Char. 224: 0 → 1
Char. 231: 0 → 1
Char. 239: 0 → 1
Char. 265: 0 → 1
Char. 267: 0 → 1
Char. 275: 1 → 0
Char. 278: 3 → 0

**Node 50:**
Char. 46: 1 → 0
Char. 88: 1 → 0
Char. 89: 1 → 0
Char. 93: 1 → 0
Char. 176: 0 → 1
Char. 195: 0 → 2
Char. 198: 0 → 1
Char. 246: 1 → 2
Char. 259: 0 → 1
Char. 270: 0 → 1

**Node 51:**
Char. 65: 0 → 1
Char. 72: 1 → 2
Char. 131: 0 → 1
Char. 184: 0 → 1
Char. 205: 0 → 1
Char. 210: 0 → 1
Char. 241: 0 → 1
Char. 246: 0 → 1
Char. 254: 0 → 1
Char. 255: 0 → 1
Char. 256: 0 → 1
Char. 267: 0 → 1
Char. 268: 0 → 1
Char. 269: 0 → 1
Char. 278: 3 → 0

**Node 52:**
Char. 21: 0 → 1
Char. 84: 1 → 2

**Node 53:**
Char. 15: 0 → 1
Char. 25: 1 → 0
Char. 33: 0 → 2
Char. 48: 1 → 0
Char. 55: 1 → 0
Char. 89: 0 → 1
Char. 94: 0 → 1
Char. 127: 0 → 1

**Node 54:**
Char. 5: 0 → 1
Char. 20: 0 → 1
Char. 40: 0 → 2
Char. 48: 0 → 1
Char. 74: 0 → 1
Char. 80: 0 → 1
Char. 84: 0 → 1
Char. 88: 0 → 1
Char. 116: 0 → 1
Char. 119: 0 → 1
Char. 132: 0 → 1
Char. 149: 0 → 1
Char. 156: 0 → 1
Char. 163: 0 → 1
Char. 166: 0 → 1
Char. 169: 0 → 1
Char. 180: 0 → 1
Char. 192: 0 → 1
Char. 201: 0 → 1
Char. 202: 0 → 1
Char. 203: 0 → 1
Char. 209: 0 → 1
Char. 221: 0 → 1
Char. 234: 0 → 1
Char. 235: 0 → 1

**Node 55:**
Char. 72: 0 → 1
Char. 79: 1 → 0
Char. 81: 0 → 1
Char. 93: 0 → 1
Char. 97: 0 → 1
Char. 104: 1 → 0
Char. 144: 0 → 1
Char. 173: 0 → 1
Char. 183: 0 → 1

**Node 57:**
Char. 29: 0 → 1
Char. 95: 0 → 1
Char. 110: 0 → 1
Char. 111: 1 → 0
Char. 113: 0 → 1
Char. 114: 0 → 1
Char. 131: 0 → 1
Char. 137: 0 → 1
Char. 140: 0 → 2
Char. 147: 0 → 1

**Node 58:**
Char. 19: 0 → 1
Char. 92: 1 → 0

**Node 59:**
Char. 4: 0 → 1
Char. 15: 0 → 1
Char. 29: 0 → 1
Char. 48: 1 → 0
Char. 213: 0 → 1
Char. 226: 0 → 2
Char. 228: 0 → 1

**Node 60:**
Char. 58: 0 → 1
Char. 61: 0 → 1
Char. 66: 0 → 1
Char. 69: 0 → 1
Char. 127: 0 → 1
Char. 141: 0 → 1
Char. 150: 0 → 1
Char. 167: 0 → 1
Char. 205: 0 → 1
Char. 208: 0 → 1
Char. 230: 0 → 1
Char. 239: 0 → 1

**Node 61:**
Char. 0: 0 → 1
Char. 23: 0 → 1
Char. 33: 0 → 1
Char. 59: 0 → 1
Char. 60: 0 → 1
Char. 62: 0 → 1
Char. 70: 0 → 1
Char. 72: 1 → 2
Char. 73: 0 → 1
Char. 89: 0 → 1
Char. 94: 0 → 1
Char. 109: 0 → 1
Char. 117: 0 → 12
Char. 126: 0 → 1
Char. 129: 0 → 1
Char. 131: 0 → 1
Char. 148: 0 → 1
Char. 154: 1 → 0
Char. 179: 0 → 1
Char. 182: 0 → 1
Char. 188: 0 → 1
Char. 190: 0 → 1
Char. 191: 0 → 1
Char. 196: 0 → 1
Char. 214: 0 → 1
Char. 219: 0 → 1
Char. 265: 0 → 1
Char. 267: 0 → 1
Char. 272: 2 → 1
Char. 278: 3 → 0

**Node 62:**
Char. 24: 0 → 1
Char. 29: 0 → 1
Char. 79: 0 → 1
Char. 83: 0 → 1
Char. 110: 0 → 1
Char. 111: 1 → 0
Char. 132: 1 → 0

**Node 63:**
Char. 73: 0 → 1
Char. 131: 1 → 0
Char. 205: 0 → 1
Char. 276: 0 → 1

**Node 64:**
Char. 18: 0 → 1
Char. 37: 0 → 1
Char. 100: 1 → 0
Char. 107: 0 → 1
Char. 113: 1 → 0
Char. 118: 1 → 0
Char. 125: 1 → 0
Char. 150: 0 → 1

**Node 65:**
Char. 23: 0 → 1
Char. 71: 1 → 0
Char. 85: 1 → 0
Char. 102: 0 → 1
Char. 103: 0 → 1
Char. 106: 0 → 1
Char. 167: 0 → 1
Char. 214: 0 → 1
Char. 216: 1 → 0
Char. 235: 0 → 2
Char. 241: 0 → 1

**Node 66:**
Char. 100: 0 → 1
Char. 113: 0 → 1
Char. 132: 1 → 0
Char. 138: 0 → 1
Char. 141: 0 → 1

**Node 67:**
Char. 186: 0 → 1

**Node 68:**
Char. 120: 1 → 0
Char. 148: 0 → 1
Char. 183: 1 → 2
Char. 194: 0 → 1
Char. 201: 1 → 0
Char. 211: 0 → 1
Char. 234: 1 → 0
Char. 235: 1 → 0
Char. 240: 1 → 0
Char. 252: 0 → 1

**Node 69:**
Char. 87: 0 → 1

**Node 70:**
Char. 25: 1 → 0
Char. 76: 1 → 0

**Node 71:**
Char. 79: 0 → 1
Char. 93: 1 → 0
Char. 133: 0 → 1

**Node 72:**
Char. 20: 1 → 0
Char. 38: 1 → 2
Char. 39: 0 → 1
Char. 58: 0 → 1
Char. 59: 0 → 1
Char. 60: 0 → 1
Char. 72: 1 → 2
Char. 83: 0 → 1
Char. 85: 1 → 0
Char. 88: 1 → 0
Char. 95: 0 → 1
Char. 104: 0 → 1
Char. 105: 0 → 1
Char. 106: 0 → 2
Char. 107: 0 → 1
Char. 109: 0 → 1
Char. 110: 0 → 1
Char. 146: 1 → 0
Char. 148: 0 → 1
Char. 155: 0 → 2
Char. 183: 1 → 2

**Node 73:**
Char. 0: 0 → 2
Char. 33: 1 → 0
Char. 38: 2 → 1
Char. 39: 1 → 0
Char. 42: 0 → 1
Char. 43: 0 → 1
Char. 46: 1 → 0
Char. 48: 1 → 0
Char. 49: 1 → 0
Char. 52: 0 → 1
Char. 83: 0 → 2
Char. 84: 1 → 0
Char. 87: 0 → 1
Char. 93: 1 → 0
Char. 132: 0 → 1
Char. 161: 0 → 1
Char. 163: 1 → 0
Char. 172: 0 → 2
Char. 174: 0 → 1
Char. 188: 0 → 1
Char. 189: 0 → 1
Char. 195: 0 → 1
Char. 204: 0 → 2
Char. 212: 0 → 1
Char. 221: 1 → 0
Char. 236: 0 → 1
Char. 238: 0 → 2
Char. 242: 0 → 1
Char. 245: 0 → 1
Char. 274: 0 → 1
Char. 275: 1 → 0

**Node 74:**
Char. 5: 1 → 0
Char. 20: 1 → 0
Char. 44: 1 → 0
Char. 48: 1 → 0
Char. 59: 0 → 1
Char. 60: 0 → 1
Char. 66: 0 → 1
Char. 72: 1 → 2
Char. 78: 1 → 0
Char. 84: 1 → 0
Char. 88: 1 → 0
Char. 93: 1 → 0
Char. 123: 1 → 0
Char. 129: 0 → 1
Char. 154: 1 → 0
Char. 159: 1 → 0
Char. 163: 1 → 0
Char. 169: 1 → 0
Char. 170: 0 → 1
Char. 192: 1 → 0
Char. 197: 0 → 1
Char. 202: 1 → 0
Char. 207: 1 → 0
Char. 209: 1 → 0
Char. 221: 1 → 0
Char. 234: 1 → 0

**Node 75:**
Char. 29: 0 → 1
Char. 47: 0 → 1

Char. 83: 0 → 1
Char. 85: 1 → 0
Char. 107: 0 → 1
Char. 110: 0 → 1
Char. 111: 1 → 0
Char. 169: 1 → 0
Char. 170: 0 → 1

**Node 76:**
Char. 2: 0 → 1
Char. 6: 0 → 1
Char. 101: 0 → 1
Char. 186: 0 → 1
Char. 198: 0 → 1
Char. 220: 0 → 1
Char. 239: 1 → 0
Char. 240: 1 → 0

**Node 77:**
Char. 21: 0 → 1
Char. 33: 1 → 2
Char. 68: 0 → 1
Char. 235: 1 → 2

**Node 78:**
Char. 35: 0 → 1
Char. 184: 0 → 1
Char. 210: 0 → 1

**Node 79:**
Char. 17: 0 → 1
Char. 42: 0 → 1
Char. 251: 0 → 1
Char. 264: 0 → 1

**Node 80:**
Char. 41: 0 → 1
Char. 48: 1 → 0
Char. 61: 1 → 2
Char. 112: 1 → 0
Char. 138: 0 → 1
Char. 146: 1 → 0
Char. 155: 0 → 1
Char. 182: 1 → 0
Char. 192: 1 → 2
Char. 224: 0 → 1
Char. 226: 0 → 1
Char. 227: 0 → 1
Char. 233: 0 → 1

**Node 81:**
Char. 61: 1 → 3
Char. 90: 0 → 1
Char. 91: 0 → 1
Char. 109: 1 → 0
Char. 155: 0 → 1
Char. 209: 1 → 0

**Analysis 64**
**(all taxa, implied weighting, K = 1000)**

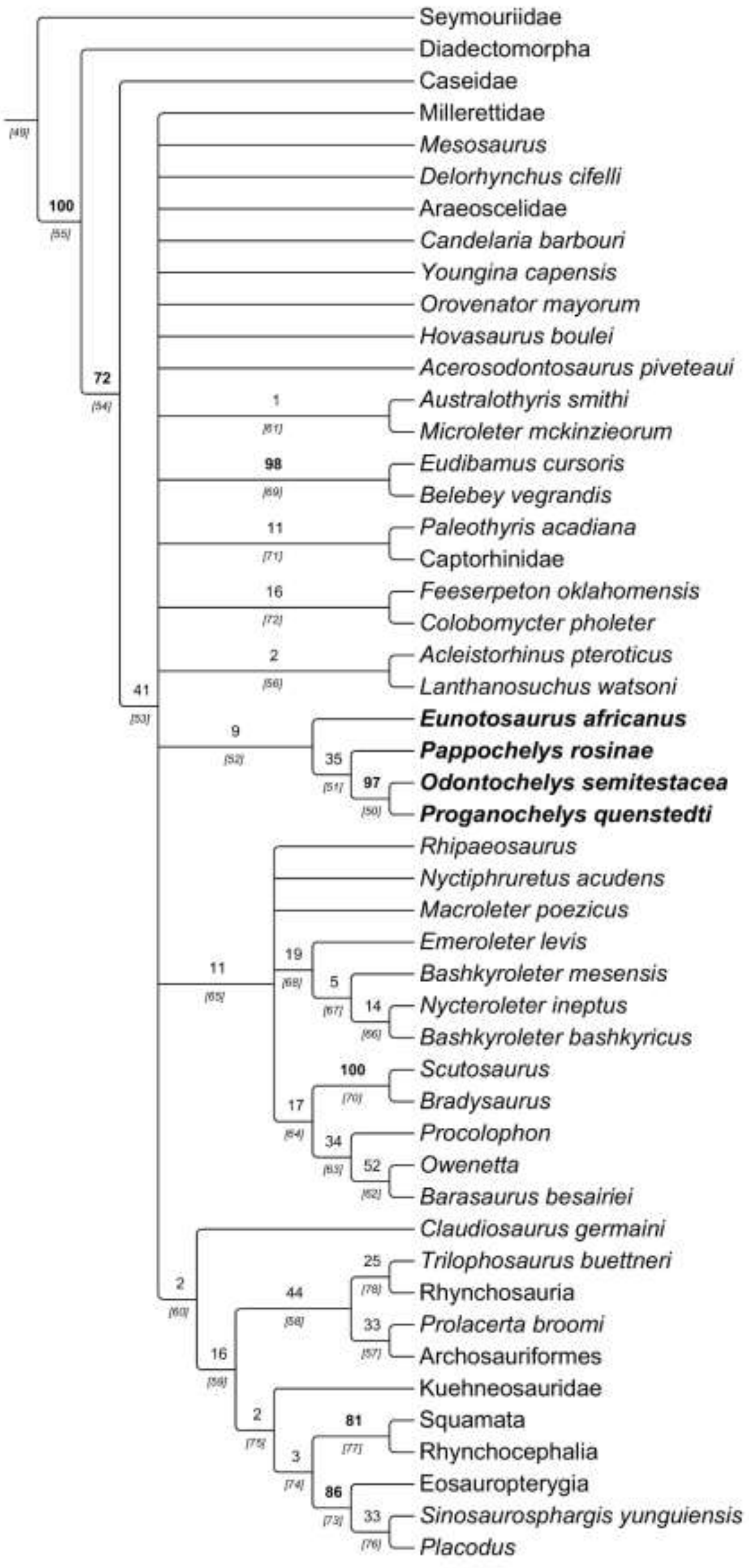

***Proganochelys quenstedti*:**
Char. 8: 0 → 1
Char. 11: 0 → 1
Char. 106: 0 → 1
Char. 108: 1 → 0
Char. 109: 0 → 1
Char. 128: 0 → 1
Char. 175: 0 → 1
Char. 202: 1 → 0
Char. 207: 1 → 0
Char. 209: 1 → 0
Char. 244: 0 → 1
Char. 248: 0 → 1
Char. 250: 0 → 1
Char. 252: 1 → 0
Char. 262: 0 → 1

***Pappochelys rosinae*:**
Char. 1: 0 → 1
Char. 5: 1 → 0
Char. 12: 0 → 1
Char. 23: 0 → 1
Char. 41: 0 → 1
Char. 49: 0 → 1
Char. 75: 0 → 1
Char. 129: 0 → 1
Char. 169: 1 → 0
Char. 260: 2 → 0
Char. 265: 0 → 1

***Odontochelys semitestacea*:**
Char. 43: 0 → 1

***Eunotosaurus africanus*:**
Char. 19: 0 → 1
Char. 43: 0 → 1
Char. 76: 0 → 1
Char. 97: 1 → 0
Char. 103: 0 → 1
Char. 153: 1 → 0
Char. 192: 1 → 0
Char. 202: 1 → 0
Char. 211: 0 → 1
Char. 219: 0 → 1
Char. 248: 0 → 1
Char. 249: 0 → 2
Char. 250: 0 → 1
Char. 263: 0 → 1
Char. 273: 0 → 1
Char. 274: 0 → 1
Char. 276: 0 → 1
Char. 277: 0 → 1

***Acerosodontosaurus piveteaui*:**
Char. 23: 0 → 1
Char. 59: 0 → 1
Char. 78: 1 → 0
Char. 81: 1 → 0
Char. 89: 0 → 1
Char. 94: 0 → 1
Char. 128: 0 → 1
Char. 129: 0 → 1
Char. 155: 0 → 1
Char. 206: 0 → 2
Char. 208: 0 → 1
Char. 265: 0 → 1
Char. 266: 0 → 1
Char. 278: 3 → 1

***Acleistorhinus pteroticus*:**
Char. 21: 0 → 1
Char. 146: 1 → 0
Char. 169: 1 → 0
Char. 170: 0 → 1

**Araeoscelidae:**
Char. 0: 0 → 1
Char. 5: 1 → 0
Char. 20: 1 → 0
Char. 23: 0 → 1
Char. 27: 0 → 1
Char. 28: 0 → 1
Char. 38: 1 → 0
Char. 40: 2 → 0
Char. 43: 0 → 1
Char. 59: 0 → 1
Char. 60: 0 → 1
Char. 72: 1 → 2
Char. 84: 1 → 0
Char. 89: 0 → 1
Char. 106: 0 → 1
Char. 116: 1 → 0
Char. 117: 0 → 2
Char. 154: 1 → 0
Char. 159: 1 → 0
Char. 166: 1 → 0
Char. 169: 1 → 0
Char. 170: 0 → 1
Char. 193: 0 → 1
Char. 197: 0 → 1
Char. 202: 1 → 0
Char. 207: 1 → 0
Char. 209: 1 → 0
Char. 221: 1 → 0
Char. 224: 0 → 1
Char. 239: 0 → 1
Char. 266: 0 → 1
Char. 278: 3 → 1

**Archosauriformes:**
Char. 32: 0 → 1
Char. 94: 1 → 0
Char. 112: 1 → 0
Char. 152: 0 → 1
Char. 154: 0 → 2
Char. 166: 1 → 0
Char. 171: 0 → 1
Char. 185: 0 → 1
Char. 204: 0 → 1
Char. 218: 0 → 3
Char. 224: 0 → 1
Char. 242: 0 → 1

***Australothyris smithi*:**
Char. 23: 0 → 1
Char. 34: 0 → 1
Char. 55: 1 → 0
Char. 71: 1 → 0
Char. 85: 1 → 0
Char. 129: 0 → 1
Char. 131: 0 → 1
Char. 159: 1 → 0

***Barasaurus besairiei*:**
Char. 33: 1 → 0
Char. 75: 1 → 0
Char. 216: 0 → 1

***Bashkyroleter bashkyricus*:**
Char. 275: 1 → 0

***Bashkyroleter mesensis*:**
Char. 169: 1 → 0

***Belebey vegrandis*:**
Char. 154: 1 → 0

***Bradysaurus* spp.:**
Char. 19: 0 → 1
Char. 73: 0 → 1
Char. 79: 0 → 1
Char. 135: 1 → 0
Char. 249: 0 → 1

***Candelaria barbouri*:**
Char. 1: 0 → 1
Char. 5: 1 → 0
Char. 8: 0 → 1
Char. 15: 0 → 1
Char. 23: 0 → 1
Char. 25: 1 → 0
Char. 33: 01 → 2
Char. 48: 1 → 0
Char. 49: 0 → 1
Char. 55: 1 → 0
Char. 76: 0 → 1
Char. 79: 0 → 2
Char. 83: 0 → 1
Char. 88: 1 → 0
Char. 89: 0 → 1
Char. 94: 0 → 1
Char. 95: 0 → 1
Char. 126: 0 → 1
Char. 127: 0 → 1
Char. 132: 1 → 0
Char. 154: 1 → 2
Char. 159: 1 → 0
Char. 169: 1 → 0
Char. 276: 0 → 1
Char. 277: 0 → 1

**Captorhinidae:**
Char. 3: 0 → 1
Char. 23: 0 → 1
Char. 25: 1 → 0
Char. 26: 0 → 1
Char. 73: 0 → 1
Char. 75: 0 → 1
Char. 83: 0 → 1
Char. 108: 1 → 0
Char. 180: 1 → 0
Char. 183: 1 → 0
Char. 201: 1 → 0
Char. 203: 1 → 2
Char. 216: 1 → 0
Char. 240: 1 → 0

**Caseidae:**
Char. 24: 0 → 1
Char. 25: 1 → 0
Char. 36: 0 → 1
Char. 38: 1 → 0
Char. 46: 1 → 0
Char. 56: 0 → 1
Char. 170: 0 → 1
Char. 194: 0 → 1
Char. 273: 0 → 1
Char. 274: 0 → 1
Char. 278: 3 → 2

***Claudiosaurus germaini*:**
Char. 24: 0 → 1
Char. 27: 0 → 1
Char. 34: 0 → 1
Char. 36: 0 → 1
Char. 64: 0 → 1
Char. 84: 1 → 0
Char. 105: 0 → 1
Char. 130: 0 → 1
Char. 144: 1 → 0
Char. 166: 1 → 0
Char. 199: 0 → 1
Char. 203: 1 → 2
Char. 204: 0 → 1
Char. 220: 0 → 1

***Colobomycter pholeter*:**
Char. 21: 0 → 1
Char. 25: 1 → 0
Char. 84: 1 → 0
Char. 154: 1 → 0
Char. 167: 0 → 1
Char. 267: 0 → 1

***Delorhynchus cifelli*:**
Char. 18: 0 → 1
Char. 21: 0 → 1
Char. 24: 0 → 1
Char. 26: 0 → 1
Char. 28: 0 → 1
Char. 29: 0 → 1
Char. 33: 01 → 2
Char. 39: 0 → 1
Char. 52: 0 → 1
Char. 100: 0 → 1
Char. 116: 1 → 0
Char. 117: 0 → 1
Char. 119: 1 → 0
Char. 131: 0 → 1
Char. 147: 0 → 1
Char. 156: 1 → 0
Char. 167: 0 → 1
Char. 189: 0 → 1
Char. 191: 0 → 1
Char. 202: 1 → 0
Char. 204: 0 → 2
Char. 267: 0 → 1

**Diadectomorpha:**
Char. 0: 0 → 1
Char. 64: 0 → 1
Char. 70: 0 → 1
Char. 122: 0 → 1
Char. 123: 1 → 0
Char. 146: 1 → 0
Char. 275: 1 → 0
Char. 278: 3 → 0

***Emeroleter levis*:**
Char. 0: 0 → 2
Char. 51: 1 → 0

**Eosauropterygia:**
Char. 159: 1 → 0
Char. 166: 1 → 0
Char. 174: 0 → 1
Char. 194: 0 → 2
Char. 272: 1 → 0

***Eudibamus cursoris*:**
Char. 154: 1 → 2
Char. 163: 1 → 0

***Feeserpeton oklahomensis*:**
Char. 51: 0 → 1
Char. 70: 0 → 1
Char. 157: 0 → 1
Char. 158: 0 → 1

***Hovasaurus boulei*:**
Char. 41: 0 → 1
Char. 43: 0 → 1
Char. 55: 1 → 0
Char. 59: 0 → 1
Char. 77: 0 → 2
Char. 78: 1 → 0
Char. 79: 0 → 1
Char. 89: 0 → 1
Char. 93: 1 → 0
Char. 113: 0 → 1
Char. 127: 0 → 1
Char. 135: 0 → 1
Char. 136: 0 → 1
Char. 138: 0 → 1
Char. 146: 1 → 0
Char. 187: 0 → 1
Char. 188: 0 → 1
Char. 191: 0 → 1
Char. 193: 0 → 1
Char. 196: 0 → 1
Char. 201: 1 → 0
Char. 204: 0 → 2
Char. 206: 0 → 2
Char. 215: 0 → 1
Char. 220: 0 → 1
Char. 224: 0 → 1
Char. 265: 0 → 1
Char. 275: 1 → 0

**Kuehneosauridae:**
Char. 7: 0 → 1
Char. 24: 0 → 1
Char. 27: 0 → 1
Char. 34: 0 → 1
Char. 36: 0 → 1
Char. 44: 1 → 0
Char. 79: 0 → 2
Char. 148: 1 → 0
Char. 159: 1 → 0
Char. 185: 0 → 1
Char. 245: 0 → 1
Char. 272: 1 → 2
Char. 278: 0 → 3

***Lanthanosuchus watsoni*:**
Char. 25: 1 → 0
Char. 51: 0 → 1
Char. 76: 0 → 1
Char. 86: 0 → 1
Char. 98: 1 → 0
Char. 119: 1 → 0
Char. 138: 0 → 1
Char. 144: 1 → 0
Char. 154: 1 → 2

***Macroleter poezicus*:**
Char. 0: 0 → 1
Char. 9: 0 → 1
Char. 26: 0 → 1
Char. 52: 0 → 1
Char. 66: 1 → 2
Char. 75: 0 → 1
Char. 84: 1 → 0
Char. 87: 0 → 1
Char. 110: 0 → 1
Char. 121: 0 → 1
Char. 126: 0 → 1
Char. 134: 0 → 2
Char. 140: 0 → 1
Char. 143: 0 → 1
Char. 146: 1 → 0
Char. 147: 1 → 2
Char. 154: 1 → 0
Char. 169: 1 → 0
Char. 235: 0 → 1
Char. 278: 3 → 0

***Mesosaurus* spp.:**
Char. 0: 0 → 1
Char. 2: 0 → 1
Char. 5: 1 → 0
Char. 6: 0 → 1
Char. 8: 0 → 1
Char. 9: 0 → 1
Char. 13: 0 → 1
Char. 19: 0 → 1
Char. 20: 1 → 0
Char. 23: 0 → 1
Char. 26: 0 → 1
Char. 38: 1 → 0
Char. 41: 0 → 1
Char. 76: 0 → 1
Char. 83: 0 → 1
Char. 84: 1 → 0
Char. 85: 1 → 0
Char. 94: 0 → 1
Char. 107: 0 → 1
Char. 109: 0 → 1
Char. 115: 0 → 1
Char. 146: 1 → 0
Char. 148: 0 → 1
Char. 149: 1 → 0
Char. 159: 1 → 0
Char. 163: 1 → 0
Char. 164: 0 → 1
Char. 166: 1 → 0
Char. 167: 0 → 1
Char. 176: 0 → 1
Char. 183: 1 → 0
Char. 184: 0 → 1
Char. 192: 1 → 0
Char. 199: 0 → 1
Char. 204: 0 → 2
Char. 206: 0 → 1
Char. 217: 0 → 1
Char. 219: 0 → 1
Char. 220: 0 → 1
Char. 231: 0 → 1
Char. 234: 1 → 0
Char. 240: 1 → 0
Char. 260: 2 → 0
Char. 272: 2 → 0
Char. 278: 3 → 0

***Microleter mckinzieorum*:**
Char. 0: 0 → 1
Char. 25: 1 → 0
Char. 36: 0 → 1
Char. 39: 0 → 1
Char. 51: 0 → 2
Char. 56: 0 → 1
Char. 70: 0 → 1
Char. 94: 0 → 1
Char. 106: 0 → 1
Char. 166: 1 → 0
Char. 276: 0 → 1

**Millerettidae:**
Char. 5: 1 → 0
Char. 20: 1 → 0
Char. 24: 0 → 1
Char. 25: 1 → 0
Char. 29: 0 → 1
Char. 44: 1 → 0
Char. 48: 1 → 0
Char. 56: 0 → 1
Char. 66: 0 → 2
Char. 78: 1 → 0
Char. 80: 1 → 0
Char. 84: 1 → 2
Char. 88: 1 → 0
Char. 96: 1 → 0
Char. 111: 1 → 0
Char. 117: 0 → 1
Char. 120: 1 → 0
Char. 121: 0 → 1
Char. 124: 0 → 1
Char. 127: 0 → 1
Char. 135: 0 → 1
Char. 145: 0 → 1
Char. 159: 1 → 0
Char. 163: 1 → 0
Char. 166: 1 → 0
Char. 180: 1 → 0
Char. 192: 1 → 0
Char. 211: 0 → 1
Char. 230: 0 → 1
Char. 248: 0 → 1
Char. 252: 0 → 1
Char. 253: 0 → 1

***Nycteroleter ineptus*:**
Char. 278: 0 → 3

***Nyctiphruretus acudens*:**
Char. 0: 0 → 1
Char. 21: 0 → 1
Char. 33: 1 → 2
Char. 41: 0 → 1
Char. 48: 1 → 0
Char. 66: 1 → 2
Char. 81: 1 → 0
Char. 83: 0 → 1
Char. 84: 1 → 2
Char. 85: 1 → 0
Char. 94: 0 → 1
Char. 166: 1 → 0
Char. 167: 0 → 1
Char. 207: 1 → 0
Char. 215: 1 → 0
Char. 224: 0 → 1
Char. 226: 1 → 0
Char. 266: 0 → 1
Char. 272: 2 → 1
Char. 276: 0 → 1

***Orovenator mayorum*:**
Char. 0: 0 → 1
Char. 8: 0 → 1
Char. 24: 0 → 1
Char. 25: 1 → 0
Char. 36: 0 → 1
Char. 48: 1 → 0
Char. 62: 0 → 1
Char. 72: 1 → 2
Char. 89: 0 → 1
Char. 136: 0 → 1
Char. 141: 0 → 1
Char. 159: 1 → 0
Char. 160: 0 → 1
Char. 165: 0 → 1
Char. 278: 3 → 1

***Owenetta* spp.:**
Char. 169: 1 → 0

***Paleothyris acadiana*:**
Char. 38: 1 → 0
Char. 66: 1 → 2
Char. 102: 0 → 1
Char. 146: 1 → 0
Char. 239: 0 → 1

***Placodus* spp.:**
Char. 0: 1 → 2
Char. 9: 0 → 1
Char. 12: 0 → 1
Char. 13: 0 → 1
Char. 19: 0 → 1
Char. 31: 0 → 1
Char. 44: 1 → 0
Char. 46: 1 → 0
Char. 57: 0 → 1
Char. 78: 1 → 0
Char. 93: 1 → 0
Char. 102: 1 → 2
Char. 109: 1 → 0
Char. 140: 1 → 0
Char. 155: 0 → 1
Char. 163: 1 → 0
Char. 164: 0 → 1

***Procolophon* spp.:**
Char. 41: 0 → 1
Char. 69: 0 → 1
Char. 79: 0 → 1
Char. 83: 0 → 1
Char. 88: 0 → 1
Char. 117: 1 → 0
Char. 149: 1 → 0
Char. 204: 0 → 1
Char. 237: 0 → 1
Char. 238: 0 → 1
Char. 272: 2 → 1
Char. 278: 3 → 0

***Prolacerta broomi*:**
Char. 58: 1 → 0
Char. 66: 1 → 0
Char. 67: 1 → 0
Char. 80: 1 → 0
Char. 139: 1 → 0
Char. 192: 1 → 0
Char. 203: 1 → 2
Char. 206: 0 → 12

***Rhipaeosaurus* spp.:**
Char. 172: 0 → 1
Char. 186: 1 → 0
Char. 207: 1 → 0
Char. 277: 0 → 1

**Rhynchocephalia:**
Char. 0: 1 → 2
Char. 24: 0 → 1
Char. 77: 0 → 1
Char. 94: 1 → 0
Char. 117: 12 → 0
Char. 139: 1 → 0
Char. 167: 1 → 0
Char. 205: 1 → 0

**Rhynchosauria:**
Char. 0: 1 → 0
Char. 7: 0 → 1
Char. 9: 0 → 1
Char. 26: 0 → 1
Char. 44: 1 → 0
Char. 68: 0 → 1
Char. 99: 1 → 0
Char. 150: 1 → 0
Char. 160: 0 → 1
Char. 161: 0 → 1
Char. 171: 0 → 2
Char. 182: 1 → 0
Char. 223: 0 → 1
Char. 224: 0 → 1
Char. 241: 0 → 1

***Scutosaurus* spp.:**
Char. 175: 0 → 1
Char. 218: 0 → 1
Char. 243: 0 → 2
Char. 244: 0 → 1
Char. 251: 0 → 1

***Sinosaurosphargis yunguiensis*:**
Char. 8: 0 → 1
Char. 30: 0 → 1
Char. 53: 0 → 1
Char. 89: 1 → 0
Char. 127: 1 → 0
Char. 150: 1 → 0
Char. 154: 0 → 2
Char. 167: 1 → 0
Char. 253: 0 → 1
Char. 255: 0 → 1

**Squamata:**
Char. 45: 0 → 1
Char. 79: 0 → 2
Char. 80: 1 → 0
Char. 92: 1 → 0
Char. 109: 1 → 0
Char. 160: 0 → 1
Char. 245: 0 → 1

***Trilophosaurus buettneri*:**
Char. 5: 1 → 0
Char. 11: 0 → 1
Char. 55: 1 → 0
Char. 93: 1 → 0
Char. 104: 0 → 1
Char. 113: 0 → 1
Char. 122: 0 → 1
Char. 144: 1 → 0
Char. 154: 0 → 1
Char. 157: 0 → 1
Char. 159: 1 → 0
Char. 177: 0 → 12
Char. 194: 0 → 1
Char. 203: 1 → 2
Char. 207: 1 → 0
Char. 208: 1 → 0
Char. 272: 1 → 0

***Youngina capensis*:**
Char. 0: 0 → 1
Char. 5: 1 → 0
Char. 21: 0 → 1
Char. 27: 0 → 1
Char. 38: 1 → 0

Char. 40: 2 → 0
Char. 43: 0 → 1
Char. 44: 1 → 0
Char. 56: 0 → 1
Char. 59: 0 → 1
Char. 60: 0 → 1
Char. 62: 0 → 1
Char. 72: 1 → 2
Char. 75: 0 → 1
Char. 84: 1 → 0
Char. 89: 0 → 1
Char. 109: 0 → 1
Char. 127: 0 → 1
Char. 129: 0 → 1
Char. 134: 0 → 1
Char. 135: 0 → 1
Char. 141: 0 → 1
Char. 154: 1 → 0
Char. 163: 1 → 0
Char. 170: 0 → 1
Char. 179: 0 → 1
Char. 187: 0 → 1
Char. 191: 0 → 1
Char. 193: 0 → 1
Char. 196: 0 → 1
Char. 201: 1 → 0
Char. 211: 0 → 1
Char. 214: 0 → 1
Char. 215: 0 → 1
Char. 219: 0 → 1
Char. 224: 0 → 1
Char. 231: 0 → 1
Char. 239: 0 → 1
Char. 265: 0 → 1
Char. 267: 0 → 1
Char. 275: 1 → 0
Char. 278: 3 → 0

**Node 50:**
Char. 46: 1 → 0
Char. 88: 1 → 0
Char. 93: 1 → 0
Char. 176: 0 → 1
Char. 195: 0 → 2
Char. 198: 0 → 1
Char. 246: 1 → 2
Char. 259: 0 → 1
Char. 270: 0 → 1

**Node 51:**
Char. 65: 0 → 1
Char. 72: 1 → 2
Char. 131: 0 → 1
Char. 184: 0 → 1
Char. 205: 0 → 1
Char. 210: 0 → 1
Char. 241: 0 → 1
Char. 246: 0 → 1
Char. 254: 0 → 1
Char. 255: 0 → 1
Char. 256: 0 → 1
Char. 267: 0 → 1
Char. 268: 0 → 1
Char. 269: 0 → 1
Char. 278: 3 → 0

**Node 52:**
Char. 15: 0 → 1
Char. 21: 0 → 1
Char. 25: 1 → 0
Char. 33: 01 → 2
Char. 44: 1 → 0
Char. 64: 0 → 1
Char. 73: 0 → 1
Char. 84: 1 → 2
Char. 94: 0 → 1
Char. 127: 0 → 1
Char. 130: 0 → 1
Char. 134: 0 → 2
Char. 135: 0 → 1
Char. 136: 0 → 1
Char. 141: 0 → 1
Char. 145: 0 → 1
Char. 147: 0 → 1
Char. 148: 0 → 1
Char. 151: 0 → 1
Char. 152: 0 → 1
Char. 155: 0 → 1
Char. 158: 0 → 1
Char. 161: 0 → 1
Char. 174: 0 → 1
Char. 181: 0 → 1
Char. 188: 0 → 1
Char. 196: 0 → 1
Char. 201: 1 → 0
Char. 203: 1 → 2
Char. 204: 0 → 2
Char. 217: 0 → 1
Char. 220: 0 → 1
Char. 231: 0 → 1
Char. 247: 0 → 1
Char. 251: 0 → 1
Char. 252: 0 → 1
Char. 253: 0 → 2

**Node 53:**
Char. 5: 0 → 1
Char. 20: 0 → 1
Char. 40: 0 → 2
Char. 48: 0 → 1
Char. 74: 0 → 1
Char. 80: 0 → 1
Char. 84: 0 → 1
Char. 88: 0 → 1
Char. 116: 0 → 1
Char. 119: 0 → 1
Char. 132: 0 → 1
Char. 149: 0 → 1
Char. 156: 0 → 1
Char. 163: 0 → 1
Char. 166: 0 → 1
Char. 169: 0 → 1
Char. 180: 0 → 1
Char. 192: 0 → 1
Char. 201: 0 → 1
Char. 202: 0 → 1
Char. 203: 0 → 1
Char. 209: 0 → 1
Char. 221: 0 → 1
Char. 234: 0 → 1
Char. 235: 0 → 1

**Node 54:**
Char. 72: 0 → 1
Char. 79: 1 → 0
Char. 81: 0 → 1
Char. 93: 0 → 1
Char. 97: 0 → 1
Char. 104: 1 → 0
Char. 144: 0 → 1
Char. 173: 0 → 1
Char. 183: 0 → 1

**Node 56:**
Char. 29: 0 → 1
Char. 95: 0 → 1
Char. 110: 0 → 1
Char. 111: 1 → 0
Char. 113: 0 → 1
Char. 114: 0 → 1
Char. 131: 0 → 1
Char. 137: 0 → 1
Char. 140: 0 → 2
Char. 147: 0 → 1

**Node 57:**
Char. 19: 0 → 1
Char. 92: 1 → 0

**Node 58:**
Char. 4: 0 → 1
Char. 15: 0 → 1
Char. 29: 0 → 1
Char. 48: 1 → 0
Char. 213: 0 → 1
Char. 226: 0 → 2
Char. 228: 0 → 1

**Node 59:**
Char. 58: 0 → 1
Char. 61: 0 → 1
Char. 66: 0 → 1
Char. 69: 0 → 1
Char. 127: 0 → 1
Char. 141: 0 → 1
Char. 150: 0 → 1
Char. 167: 0 → 1
Char. 205: 0 → 1
Char. 208: 0 → 1
Char. 230: 0 → 1
Char. 239: 0 → 1

**Node 60:**
Char. 0: 0 → 1
Char. 23: 0 → 1
Char. 59: 0 → 1
Char. 60: 0 → 1
Char. 62: 0 → 1
Char. 70: 0 → 1
Char. 72: 1 → 2
Char. 73: 0 → 1
Char. 89: 0 → 1
Char. 94: 0 → 1
Char. 109: 0 → 1
Char. 117: 0 → 12
Char. 126: 0 → 1
Char. 129: 0 → 1
Char. 131: 0 → 1
Char. 148: 0 → 1
Char. 154: 1 → 0
Char. 179: 0 → 1
Char. 182: 0 → 1
Char. 188: 0 → 1
Char. 190: 0 → 1
Char. 191: 0 → 1
Char. 196: 0 → 1
Char. 214: 0 → 1
Char. 219: 0 → 1
Char. 265: 0 → 1
Char. 267: 0 → 1
Char. 272: 2 → 1
Char. 278: 3 → 0

**Node 61:**
Char. 24: 0 → 1
Char. 29: 0 → 1
Char. 79: 0 → 1
Char. 83: 0 → 1
Char. 110: 0 → 1
Char. 111: 1 → 0
Char. 132: 1 → 0

**Node 62:**
Char. 73: 0 → 1
Char. 131: 1 → 0
Char. 205: 0 → 1
Char. 276: 0 → 1

**Node 63:**
Char. 18: 0 → 1
Char. 37: 0 → 1
Char. 107: 0 → 1
Char. 118: 1 → 0
Char. 125: 1 → 0
Char. 126: 0 → 1
Char. 150: 0 → 1

**Node 64:**
Char. 23: 0 → 1
Char. 71: 1 → 0
Char. 75: 0 → 1
Char. 85: 1 → 0
Char. 102: 0 → 1
Char. 103: 0 → 1
Char. 106: 0 → 1
Char. 110: 0 → 1
Char. 155: 0 → 1
Char. 167: 0 → 1
Char. 214: 0 → 1
Char. 216: 1 → 0
Char. 235: 0 → 2
Char. 241: 0 → 1

**Node 65:**
Char. 29: 0 → 1
Char. 38: 1 → 2
Char. 39: 0 → 1
Char. 44: 1 → 0
Char. 49: 0 → 1
Char. 51: 0 → 1
Char. 66: 0 → 1
Char. 70: 0 → 1
Char. 76: 0 → 1
Char. 80: 1 → 0
Char. 88: 1 → 0
Char. 95: 0 → 1
Char. 111: 1 → 0
Char. 117: 0 → 1
Char. 118: 0 → 1
Char. 120: 1 → 0
Char. 125: 0 → 1
Char. 131: 0 → 1
Char. 135: 0 → 1
Char. 137: 0 → 1
Char. 147: 0 → 1
Char. 148: 0 → 1
Char. 157: 0 → 1
Char. 158: 0 → 1
Char. 183: 1 → 2
Char. 186: 0 → 1
Char. 194: 0 → 1
Char. 197: 0 → 1
Char. 201: 1 → 0
Char. 209: 1 → 0
Char. 211: 0 → 1
Char. 215: 0 → 1
Char. 226: 0 → 1
Char. 234: 1 → 0
Char. 235: 1 → 0
Char. 240: 1 → 0
Char. 252: 0 → 1

**Node 66:**
Char. 87: 0 → 1
Char. 110: 0 → 1

**Node 67:**
Char. 25: 1 → 0
Char. 76: 1 → 0

**Node 68:**
Char. 79: 0 → 1
Char. 86: 0 → 1
Char. 93: 1 → 0
Char. 133: 0 → 1
Char. 147: 1 → 2
Char. 154: 1 → 0
Char. 278: 3 → 0

**Node 69:**
Char. 20: 1 → 0
Char. 38: 1 → 2
Char. 39: 0 → 1
Char. 58: 0 → 1
Char. 59: 0 → 1
Char. 60: 0 → 1
Char. 72: 1 → 2
Char. 83: 0 → 1
Char. 85: 1 → 0
Char. 88: 1 → 0
Char. 95: 0 → 1

Char. 104: 0 → 1
Char. 105: 0 → 1
Char. 106: 0 → 2
Char. 107: 0 → 1
Char. 109: 0 → 1
Char. 110: 0 → 1
Char. 146: 1 → 0
Char. 148: 0 → 1
Char. 155: 0 → 2
Char. 183: 1 → 2

**Node 70:**
Char. 0: 0 → 2
Char. 33: 1 → 0
Char. 38: 2 → 1
Char. 39: 1 → 0
Char. 42: 0 → 1
Char. 43: 0 → 1
Char. 46: 1 → 0
Char. 48: 1 → 0
Char. 49: 1 → 0
Char. 52: 0 → 1
Char. 83: 0 → 2
Char. 84: 1 → 0
Char. 87: 0 → 1
Char. 93: 1 → 0
Char. 143: 0 → 1
Char. 161: 0 → 1
Char. 163: 1 → 0
Char. 172: 0 → 2
Char. 174: 0 → 1
Char. 188: 0 → 1
Char. 189: 0 → 1
Char. 195: 0 → 1
Char. 204: 0 → 2
Char. 212: 0 → 1
Char. 221: 1 → 0
Char. 236: 0 → 1
Char. 238: 0 → 2
Char. 242: 0 → 1
Char. 245: 0 → 1
Char. 274: 0 → 1
Char. 275: 1 → 0

**Node 71:**
Char. 5: 1 → 0
Char. 20: 1 → 0
Char. 44: 1 → 0
Char. 48: 1 → 0
Char. 59: 0 → 1
Char. 60: 0 → 1
Char. 66: 0 → 1
Char. 72: 1 → 2
Char. 78: 1 → 0
Char. 84: 1 → 0
Char. 88: 1 → 0
Char. 93: 1 → 0
Char. 123: 1 → 0
Char. 129: 0 → 1
Char. 154: 1 → 0
Char. 159: 1 → 0
Char. 163: 1 → 0
Char. 169: 1 → 0
Char. 170: 0 → 1
Char. 192: 1 → 0
Char. 197: 0 → 1
Char. 202: 1 → 0
Char. 207: 1 → 0
Char. 209: 1 → 0
Char. 221: 1 → 0
Char. 234: 1 → 0

**Node 72:**
Char. 29: 0 → 1
Char. 47: 0 → 1
Char. 83: 0 → 1
Char. 85: 1 → 0
Char. 107: 0 → 1
Char. 110: 0 → 1
Char. 111: 1 → 0
Char. 169: 1 → 0
Char. 170: 0 → 1

**Node 73:**
Char. 2: 0 → 1
Char. 6: 0 → 1
Char. 101: 0 → 1
Char. 186: 0 → 1
Char. 198: 0 → 1
Char. 220: 0 → 1
Char. 239: 1 → 0
Char. 240: 1 → 0

**Node 74:**
Char. 21: 0 → 1
Char. 33: 1 → 2
Char. 68: 0 → 1
Char. 235: 1 → 2

**Node 75:**
Char. 35: 0 → 1
Char. 184: 0 → 1
Char. 210: 0 → 1

**Node 76:**
Char. 17: 0 → 1
Char. 42: 0 → 1
Char. 251: 0 → 1
Char. 264: 0 → 1

**Node 77:**
Char. 41: 0 → 1
Char. 48: 1 → 0
Char. 61: 1 → 2
Char. 112: 1 → 0
Char. 138: 0 → 1
Char. 146: 1 → 0
Char. 155: 0 → 1
Char. 182: 1 → 0
Char. 192: 1 → 2
Char. 224: 0 → 1
Char. 226: 0 → 1
Char. 227: 0 → 1
Char. 233: 0 → 1

**Node 78:**
Char. 61: 1 → 3
Char. 90: 0 → 1
Char. 91: 0 → 1
Char. 109: 1 → 0
Char. 155: 0 → 1
Char. 209: 1 → 0

**ANALYSIS 65**
**(NO *CANDELARIA BARBOURI* AND *EUNOTOSAURUS AFRICANUS*, IMPLIED WEIGHTING, K = 2.75)**

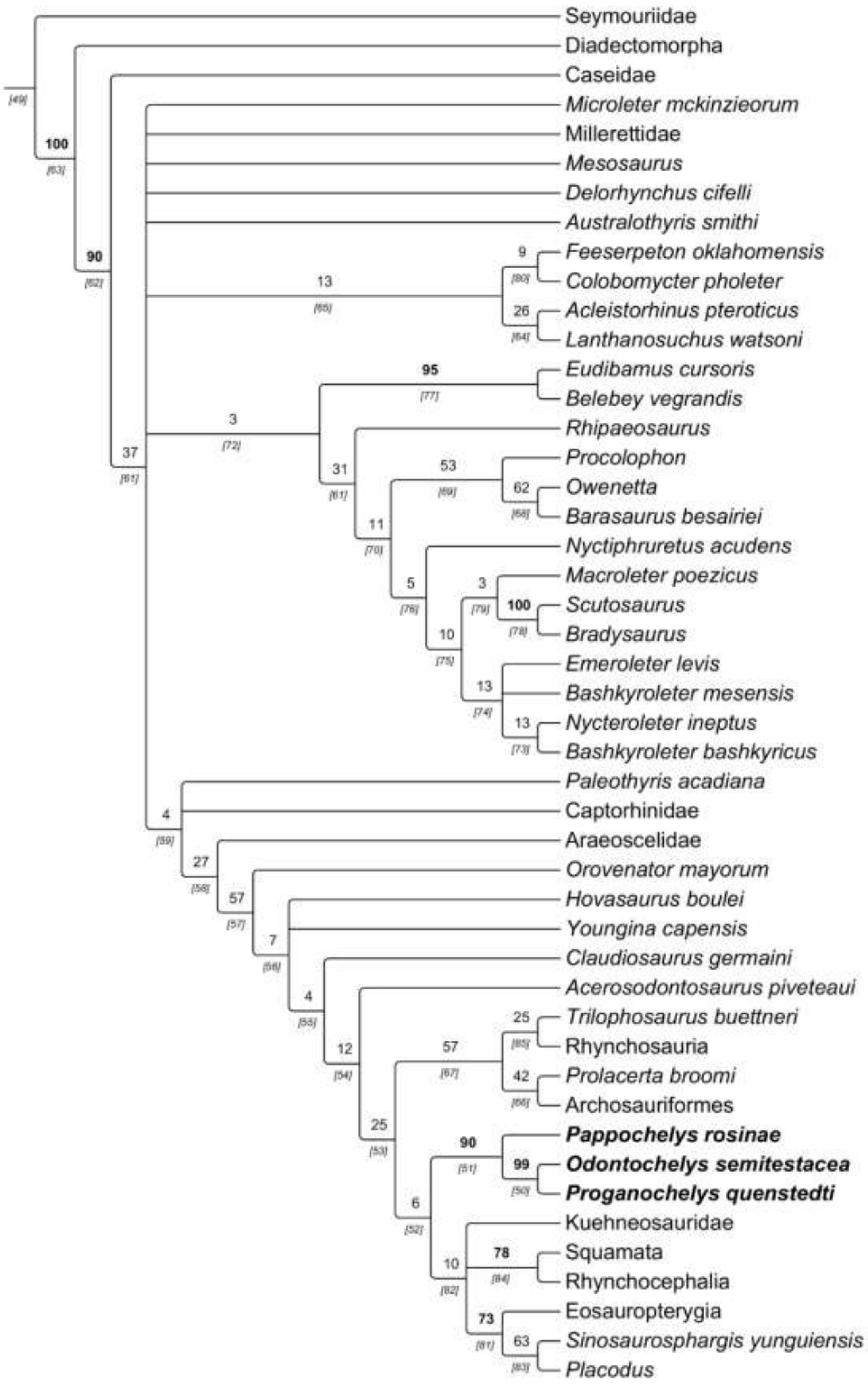

***Proganochelys quenstedti*:**
Char. 8: 0 → 1
Char. 11: 0 → 1
Char. 128: 0 → 1
Char. 175: 0 → 1
Char. 202: 1 → 0
Char. 206: 2 → 0
Char. 207: 1 → 0
Char. 209: 1 → 0
Char. 235: 2 → 0
Char. 244: 0 → 1
Char. 248: 0 → 1
Char. 250: 0 → 1
Char. 262: 0 → 1

***Pappochelys rosinae*:**
Char. 1: 0 → 1
Char. 5: 1 → 0
Char. 12: 0 → 1
Char. 41: 0 → 1
Char. 49: 0 → 1
Char. 55: 1 → 0
Char. 67: 1 → 0
Char. 81: 0 → 1
Char. 169: 1 → 0
Char. 176: 1 → 0

***Odontochelys semitestacea*:**
Char. 109: 1 → 0
Char. 112: 1 → 0
Char. 124: 0 → 1
Char. 126: 1 → 0

***Acerosodontosaurus piveteaui*:**
Char. 78: 1 → 0
Char. 128: 0 → 1
Char. 267: 1 → 0
Char. 278: 0 → 1

***Acleistorhinus pteroticus*:**
Char. 21: 0 → 1
Char. 119: 0 → 1
Char. 146: 1 → 0

**Araeoscelidae:**
Char. 23: 0 → 1
Char. 28: 0 → 1
Char. 106: 0 → 1
Char. 129: 1 → 0
Char. 239: 0 → 1

**Archosauriformes:**
Char. 32: 0 → 1
Char. 94: 1 → 0
Char. 112: 1 → 0
Char. 123: 1 → 0
Char. 152: 0 → 1
Char. 154: 0 → 2
Char. 166: 1 → 0
Char. 171: 0 → 1
Char. 185: 0 → 1
Char. 201: 0 → 1
Char. 204: 0 → 1
Char. 218: 0 → 3
Char. 242: 0 → 1

***Australothyris smithi*:**
Char. 23: 0 → 1
Char. 34: 0 → 1
Char. 55: 1 → 0
Char. 57: 1 → 0
Char. 71: 1 → 0
Char. 79: 0 → 1
Char. 98: 1 → 0
Char. 100: 0 → 1
Char. 103: 0 → 1
Char. 110: 0 → 1
Char. 112: 0 → 1
Char. 123: 1 → 0
Char. 129: 0 → 1
Char. 131: 0 → 1
Char. 132: 1 → 0
Char. 144: 1 → 0
Char. 147: 0 → 1
Char. 149: 1 → 0
Char. 150: 0 → 1
Char. 166: 0 → 1

***Barasaurus besairiei*:**
Char. 33: 1 → 0

***Bashkyroleter bashkyricus*:**
Char. 275: 1 → 0

***Bashkyroleter mesensis*:**
Char. 12: 0 → 1
Char. 66: 1 → 0
Char. 169: 1 → 0

***Belebey vegrandis*:**
Char. 154: 1 → 0

***Bradysaurus* spp.:**
Char. 19: 0 → 1
Char. 25: 1 → 0
Char. 73: 0 → 1
Char. 79: 0 → 1
Char. 135: 1 → 0
Char. 249: 0 → 1

**Captorhinidae:**
Char. 3: 0 → 1
Char. 23: 0 → 1
Char. 25: 1 → 0
Char. 26: 0 → 1
Char. 51: 0 → 2
Char. 73: 0 → 1
Char. 75: 0 → 1
Char. 108: 1 → 0
Char. 115: 0 → 1
Char. 121: 0 → 1
Char. 183: 1 → 0
Char. 201: 1 → 0
Char. 203: 1 → 2
Char. 216: 1 → 0

**Caseidae:**
Char. 25: 1 → 0
Char. 36: 0 → 1
Char. 38: 1 → 0
Char. 46: 1 → 0
Char. 50: 1 → 0
Char. 56: 0 → 1
Char. 170: 0 → 1
Char. 194: 0 → 1
Char. 273: 0 → 1
Char. 274: 0 → 1
Char. 278: 3 → 2

***Claudiosaurus germaini*:**
Char. 24: 0 → 1
Char. 34: 0 → 1
Char. 36: 0 → 1
Char. 43: 1 → 0
Char. 56: 0 → 1
Char. 64: 0 → 1
Char. 105: 0 → 1
Char. 130: 0 → 1
Char. 144: 1 → 0
Char. 187: 1 → 0
Char. 199: 0 → 1
Char. 201: 0 → 1
Char. 204: 0 → 1
Char. 220: 0 → 1
Char. 222: 1 → 0
Char. 234: 1 → 0

***Colobomycter pholeter*:**
Char. 21: 0 → 1
Char. 25: 1 → 0
Char. 84: 1 → 0
Char. 154: 1 → 0
Char. 167: 0 → 1
Char. 267: 0 → 1

***Delorhynchus cifelli*:**
Char. 5: 0 → 1
Char. 18: 0 → 1
Char. 20: 0 → 1
Char. 21: 0 → 1
Char. 26: 0 → 1
Char. 28: 0 → 1
Char. 33: 0 → 2
Char. 39: 0 → 1
Char. 48: 0 → 1
Char. 52: 0 → 1
Char. 100: 0 → 1
Char. 111: 0 → 1
Char. 116: 1 → 0
Char. 131: 0 → 1
Char. 147: 0 → 1
Char. 156: 1 → 0
Char. 159: 0 → 1
Char. 163: 0 → 1
Char. 166: 0 → 1
Char. 167: 0 → 1
Char. 189: 0 → 1
Char. 191: 0 → 1
Char. 192: 0 → 1
Char. 267: 0 → 1

**Diadectomorpha:**
Char. 0: 0 → 1
Char. 64: 0 → 1
Char. 70: 0 → 1
Char. 122: 0 → 1
Char. 123: 1 → 0
Char. 146: 1 → 0
Char. 275: 1 → 0
Char. 278: 3 → 0

***Emeroleter levis*:**
Char. 51: 1 → 0

**Eosauropterygia:**
Char. 158: 1 → 0
Char. 159: 1 → 0
Char. 166: 1 → 0
Char. 168: 0 → 1
Char. 174: 0 → 1
Char. 178: 0 → 1
Char. 194: 0 → 2
Char. 272: 1 → 0

***Eudibamus cursoris*:**
Char. 154: 1 → 2

***Feeserpeton oklahomensis*:**
Char. 51: 0 → 1
Char. 157: 0 → 1
Char. 158: 0 → 1

***Hovasaurus boulei*:**
Char. 41: 0 → 1
Char. 55: 1 → 0
Char. 60: 1 → 0
Char. 72: 2 → 1
Char. 77: 0 → 2
Char. 78: 1 → 0
Char. 79: 0 → 1
Char. 93: 1 → 0
Char. 113: 0 → 1
Char. 138: 0 → 1
Char. 146: 1 → 0
Char. 154: 0 → 1
Char. 204: 0 → 2
Char. 206: 0 → 2
Char. 220: 0 → 1
Char. 278: 0 → 3

**Kuehneosauridae:**
Char. 7: 0 → 1
Char. 21: 1 → 0
Char. 24: 0 → 1
Char. 27: 0 → 1
Char. 33: 2 → 1
Char. 34: 0 → 1
Char. 36: 0 → 1
Char. 43: 1 → 0
Char. 79: 0 → 2
Char. 147: 1 → 0
Char. 148: 1 → 0
Char. 159: 1 → 0
Char. 168: 0 → 1
Char. 185: 0 → 1
Char. 235: 2 → 1
Char. 245: 0 → 1
Char. 272: 1 → 2
Char. 278: 0 → 3

***Lanthanosuchus watsoni*:**
Char. 25: 1 → 0
Char. 51: 0 → 1
Char. 76: 0 → 1
Char. 86: 0 → 1
Char. 98: 1 → 0
Char. 138: 0 → 1
Char. 144: 1 → 0
Char. 154: 1 → 2

***Macroleter poezicus*:**
Char. 9: 0 → 1
Char. 26: 0 → 1
Char. 66: 1 → 2
Char. 126: 0 → 1
Char. 134: 0 → 2
Char. 140: 0 → 1
Char. 146: 1 → 0
Char. 169: 1 → 0

***Mesosaurus* spp.:**
Char. 0: 0 → 1
Char. 2: 0 → 1
Char. 6: 0 → 1
Char. 8: 0 → 1
Char. 9: 0 → 1
Char. 13: 0 → 1
Char. 19: 0 → 1
Char. 23: 0 → 1
Char. 26: 0 → 1
Char. 29: 1 → 0
Char. 33: 0 → 1
Char. 38: 1 → 0
Char. 41: 0 → 1
Char. 48: 0 → 1
Char. 50: 1 → 0
Char. 67: 0 → 1
Char. 76: 0 → 1
Char. 84: 1 → 0
Char. 94: 0 → 1
Char. 107: 0 → 1
Char. 109: 0 → 1
Char. 111: 0 → 1
Char. 115: 0 → 1
Char. 146: 1 → 0
Char. 148: 0 → 1
Char. 149: 1 → 0
Char. 164: 0 → 1
Char. 167: 0 → 1
Char. 176: 0 → 1
Char. 183: 1 → 0
Char. 184: 0 → 1
Char. 199: 0 → 1
Char. 202: 0 → 1
Char. 206: 0 → 1
Char. 207: 0 → 1
Char. 209: 0 → 1
Char. 217: 0 → 1
Char. 219: 0 → 1
Char. 220: 0 → 1

Char. 231: 0 → 1
Char. 272: 2 → 0
Char. 278: 3 → 0

***Microleter mckinzieorum*:**
Char. 0: 0 → 1
Char. 18: 0 → 1
Char. 25: 1 → 0
Char. 36: 0 → 1
Char. 39: 0 → 1
Char. 51: 0 → 2
Char. 56: 0 → 1
Char. 57: 1 → 0
Char. 70: 0 → 1
Char. 76: 0 → 1
Char. 79: 0 → 1
Char. 94: 0 → 1
Char. 106: 0 → 1
Char. 110: 0 → 1
Char. 132: 1 → 0
Char. 159: 0 → 1
Char. 276: 0 → 1
Char. 278: 3 → 1

**Millerettidae:**
Char. 25: 1 → 0
Char. 44: 1 → 0
Char. 56: 0 → 1
Char. 57: 1 → 0
Char. 66: 0 → 2
Char. 80: 1 → 0
Char. 84: 1 → 2
Char. 88: 1 → 0
Char. 96: 1 → 0
Char. 119: 0 → 1
Char. 121: 0 → 1
Char. 124: 0 → 1
Char. 127: 0 → 1
Char. 135: 0 → 1
Char. 145: 0 → 1
Char. 202: 0 → 1
Char. 211: 0 → 1
Char. 230: 0 → 1
Char. 234: 0 → 1
Char. 248: 0 → 1
Char. 252: 0 → 1
Char. 253: 0 → 1

***Nycteroleter ineptus*:**
Char. 66: 1 → 0
Char. 278: 0 → 3

***Nyctiphruretus acudens*:**
Char. 21: 0 → 1
Char. 41: 0 → 1
Char. 66: 1 → 2
Char. 81: 1 → 0
Char. 84: 1 → 2
Char. 94: 0 → 1
Char. 166: 1 → 0
Char. 215: 1 → 0
Char. 224: 0 → 1
Char. 226: 1 → 0
Char. 266: 0 → 1

Char. 272: 2 → 1
Char. 276: 0 → 1

***Orovenator mayorum*:**
Char. 8: 0 → 1
Char. 24: 0 → 1
Char. 36: 0 → 1
Char. 160: 0 → 1
Char. 165: 0 → 1

***Owenetta* spp.:**
Char. 142: 1 → 0
Char. 169: 1 → 0

***Paleothyris acadiana*:**
Char. 66: 01 → 2
Char. 102: 0 → 1
Char. 146: 1 → 0
Char. 239: 0 → 1

***Placodus* spp.:**
Char. 0: 1 → 2
Char. 9: 0 → 1
Char. 12: 0 → 1
Char. 13: 0 → 1
Char. 19: 0 → 1
Char. 31: 0 → 1
Char. 46: 1 → 0
Char. 57: 0 → 1
Char. 78: 1 → 0
Char. 93: 1 → 0
Char. 102: 1 → 2
Char. 109: 1 → 0
Char. 163: 1 → 0
Char. 164: 0 → 1

***Procolophon* spp.:**
Char. 41: 0 → 1
Char. 69: 0 → 1
Char. 79: 0 → 1
Char. 86: 0 → 1
Char. 88: 0 → 1
Char. 101: 0 → 1
Char. 141: 0 → 1
Char. 149: 1 → 0
Char. 203: 1 → 2
Char. 230: 0 → 1
Char. 237: 0 → 1
Char. 238: 0 → 1
Char. 239: 1 → 0
Char. 272: 2 → 1
Char. 278: 3 → 0

***Prolacerta broomi*:**
Char. 66: 1 → 0
Char. 67: 1 → 0
Char. 80: 1 → 0
Char. 139: 1 → 0
Char. 147: 1 → 0
Char. 192: 1 → 0

***Rhipaeosaurus* spp.:**
Char. 172: 0 → 1
Char. 277: 0 → 1

**Rhynchocephalia:**
Char. 0: 1 → 2
Char. 23: 1 → 0
Char. 24: 0 → 1
Char. 26: 1 → 0
Char. 77: 0 → 1
Char. 81: 0 → 1
Char. 88: 1 → 0
Char. 94: 1 → 0
Char. 117: 12 → 0
Char. 139: 1 → 0
Char. 167: 1 → 0
Char. 205: 1 → 0

**Rhynchosauria:**
Char. 0: 1 → 0
Char. 7: 0 → 1
Char. 9: 0 → 1
Char. 26: 0 → 1
Char. 68: 0 → 1
Char. 99: 1 → 0
Char. 160: 0 → 1
Char. 161: 0 → 1
Char. 171: 0 → 2
Char. 223: 0 → 1
Char. 241: 0 → 1

***Scutosaurus* spp.:**
Char. 175: 0 → 1
Char. 190: 0 → 1
Char. 218: 0 → 1
Char. 243: 0 → 2
Char. 244: 0 → 1
Char. 251: 0 → 1

***Sinosaurosphargis yunguiensis*:**
Char. 8: 0 → 1
Char. 26: 1 → 0
Char. 30: 0 → 1
Char. 53: 0 → 1
Char. 89: 1 → 0
Char. 127: 1 → 0
Char. 150: 1 → 0
Char. 154: 0 → 2
Char. 167: 1 → 0
Char. 253: 0 → 1
Char. 255: 0 → 1

**Squamata:**
Char. 45: 0 → 1
Char. 79: 0 → 2
Char. 80: 1 → 0
Char. 92: 1 → 0
Char. 109: 1 → 0
Char. 160: 0 → 1
Char. 245: 0 → 1

***Trilophosaurus buettneri*:**
Char. 5: 1 → 0
Char. 11: 0 → 1
Char. 55: 1 → 0
Char. 93: 1 → 0
Char. 104: 0 → 1
Char. 113: 0 → 1

Char. 122: 0 → 1
Char. 123: 1 → 0
Char. 136: 1 → 0
Char. 144: 1 → 0
Char. 154: 0 → 1
Char. 157: 0 → 1
Char. 159: 1 → 0
Char. 177: 0 → 12
Char. 194: 0 → 1
Char. 207: 1 → 0
Char. 208: 1 → 0
Char. 272: 1 → 0

***Youngina capensis*:**
Char. 56: 0 → 1
Char. 75: 0 → 1
Char. 211: 0 → 1
Char. 231: 0 → 1
Char. 239: 0 → 1

**Node 48:**
Char. 46: 1 → 0
Char. 50: 0 → 1
Char. 88: 1 → 0
Char. 89: 1 → 0
Char. 93: 1 → 0
Char. 195: 0 → 2
Char. 198: 0 → 1
Char. 240: 1 → 0
Char. 246: 1 → 2
Char. 259: 0 → 1
Char. 265: 1 → 0
Char. 270: 0 → 1

**Node 49:**
Char. 62: 1 → 0
Char. 78: 1 → 0
Char. 194: 0 → 2
Char. 214: 1 → 0
Char. 219: 1 → 0
Char. 220: 0 → 1
Char. 241: 0 → 1
Char. 246: 0 → 1
Char. 247: 0 → 1
Char. 254: 0 → 1
Char. 255: 0 → 1
Char. 256: 0 → 1
Char. 268: 0 → 1
Char. 269: 0 → 1

**Node 50:**
Char. 26: 0 → 1
Char. 33: 1 → 2
Char. 158: 0 → 1
Char. 181: 0 → 1
Char. 184: 0 → 1
Char. 210: 0 → 1
Char. 223: 0 → 1
Char. 235: 1 → 2

**Node 51:**
Char. 176: 0 → 1
Char. 205: 0 → 1

**Node 52:**
Char. 81: 1 → 0
Char. 166: 0 → 1
Char. 208: 0 → 1

**Node 53:**
Char. 23: 0 → 1
Char. 70: 0 → 1
Char. 73: 0 → 1
Char. 94: 0 → 1
Char. 131: 0 → 1
Char. 148: 0 → 1
Char. 272: 2 → 1

**Node 54:**
Char. 33: 0 → 1
Char. 135: 0 → 1
Char. 159: 0 → 1
Char. 267: 0 → 1
Char. 278: 1 → 0

**Node 55:**
Char. 20: 0 → 1
Char. 62: 0 → 1
Char. 169: 0 → 1

**Node 56:**
Char. 0: 0 → 1
Char. 57: 1 → 0
Char. 89: 0 → 1
Char. 112: 0 → 1
Char. 120: 0 → 1
Char. 192: 0 → 1
Char. 193: 0 → 1
Char. 222: 0 → 1
Char. 224: 0 → 1
Char. 234: 0 → 1
Char. 266: 0 → 1
Char. 278: 3 → 1

**Node 57:**
Char. 29: 1 → 0
Char. 59: 0 → 1
Char. 60: 0 → 1
Char. 67: 0 → 1
Char. 72: 1 → 2
Char. 84: 1 → 0
Char. 111: 0 → 1
Char. 119: 0 → 1
Char. 129: 0 → 1
Char. 154: 1 → 0
Char. 169: 1 → 0
Char. 170: 0 → 1
Char. 197: 0 → 1

**Node 58:**
Char. 29: 0 → 1
Char. 40: 0 → 2
Char. 74: 0 → 1
Char. 80: 0 → 1
Char. 84: 0 → 1
Char. 88: 0 → 1
Char. 116: 0 → 1
Char. 132: 0 → 1
Char. 149: 0 → 1

Char. 156: 0 → 1
Char. 169: 0 → 1
Char. 201: 0 → 1
Char. 203: 0 → 1
Char. 235: 0 → 1

**Node 59:**
Char. 72: 0 → 1
Char. 79: 1 → 0
Char. 81: 0 → 1
Char. 93: 0 → 1
Char. 97: 0 → 1
Char. 104: 1 → 0
Char. 144: 0 → 1
Char. 173: 0 → 1
Char. 183: 0 → 1

**Node 61:**
Char. 95: 0 → 1
Char. 113: 0 → 1
Char. 114: 0 → 1

**Node 62:**
Char. 5: 0 → 1
Char. 20: 0 → 1
Char. 47: 0 → 1
Char. 48: 0 → 1
Char. 79: 0 → 1
Char. 110: 0 → 1
Char. 131: 0 → 1
Char. 137: 0 → 1
Char. 140: 0 → 2
Char. 147: 0 → 1
Char. 159: 0 → 1
Char. 163: 0 → 1
Char. 166: 0 → 1

**Node 63:**
Char. 19: 0 → 1
Char. 92: 1 → 0

**Node 64:**
Char. 4: 0 → 1
Char. 29: 0 → 1
Char. 48: 1 → 0
Char. 213: 0 → 1
Char. 226: 0 → 2
Char. 228: 0 → 1
Char. 275: 0 → 1

**Node 65:**
Char. 73: 0 → 1
Char. 205: 0 → 1
Char. 273: 0 → 1
Char. 276: 0 → 1

**Node 66:**
Char. 18: 0 → 1
Char. 25: 1 → 0
Char. 37: 0 → 1
Char. 106: 0 → 1
Char. 107: 0 → 1
Char. 110: 0 → 1
Char. 126: 0 → 1
Char. 150: 0 → 1

**Node 67:**
Char. 186: 0 → 1

**Node 68:**
Char. 119: 0 → 1
Char. 194: 0 → 1
Char. 201: 1 → 0
Char. 211: 0 → 1
Char. 226: 0 → 1

**Node 69:**
Char. 5: 0 → 1
Char. 38: 1 → 2
Char. 39: 0 → 1
Char. 49: 0 → 1
Char. 66: 0 → 1
Char. 76: 0 → 1
Char. 88: 1 → 0
Char. 95: 0 → 1
Char. 112: 0 → 1
Char. 148: 0 → 1
Char. 158: 0 → 1
Char. 159: 0 → 1
Char. 166: 0 → 1
Char. 183: 1 → 2
Char. 192: 0 → 1
Char. 215: 0 → 1
Char. 239: 0 → 1

**Node 70:**
Char. 87: 0 → 1
Char. 110: 0 → 1

**Node 71:**
Char. 79: 0 → 1
Char. 133: 0 → 1

**Node 72:**
Char. 100: 0 → 1
Char. 113: 0 → 1

**Node 73:**
Char. 70: 0 → 1
Char. 118: 0 → 1
Char. 125: 0 → 1

**Node 74:**
Char. 58: 0 → 1
Char. 59: 0 → 1
Char. 60: 0 → 1
Char. 72: 1 → 2
Char. 104: 0 → 1
Char. 105: 0 → 1
Char. 106: 0 → 2
Char. 107: 0 → 1
Char. 109: 0 → 1
Char. 110: 0 → 1
Char. 146: 1 → 0

**Node 75:**
Char. 23: 0 → 1
Char. 33: 1 → 0
Char. 38: 2 → 1
Char. 39: 1 → 0
Char. 42: 0 → 1
Char. 43: 0 → 1
Char. 46: 1 → 0
Char. 49: 1 → 0
Char. 71: 1 → 0
Char. 83: 0 → 2
Char. 101: 0 → 1
Char. 103: 0 → 1
Char. 106: 0 → 1
Char. 161: 0 → 1
Char. 163: 1 → 0
Char. 172: 0 → 2
Char. 174: 0 → 1
Char. 188: 0 → 1
Char. 203: 1 → 2
Char. 212: 0 → 1
Char. 216: 1 → 0
Char. 236: 0 → 1
Char. 238: 0 → 2
Char. 239: 1 → 0
Char. 241: 0 → 1
Char. 242: 0 → 1
Char. 273: 0 → 1
Char. 274: 0 → 1
Char. 275: 1 → 0

**Node 76:**
Char. 52: 0 → 1
Char. 75: 0 → 1
Char. 84: 1 → 0
Char. 87: 0 → 1
Char. 110: 0 → 1
Char. 121: 0 → 1
Char. 230: 0 → 1

**Node 77:**
Char. 107: 0 → 1

**Node 78:**
Char. 2: 0 → 1
Char. 6: 0 → 1
Char. 81: 0 → 1
Char. 101: 0 → 1
Char. 186: 0 → 1
Char. 198: 0 → 1
Char. 220: 0 → 1
Char. 231: 1 → 0
Char. 232: 1 → 0
Char. 234: 1 → 0
Char. 240: 1 → 0
Char. 266: 1 → 0
Char. 267: 1 → 0

**Node 79:**
Char. 35: 0 → 1
Char. 103: 0 → 1
Char. 123: 1 → 0
Char. 201: 0 → 1
Char. 229: 0 → 1

**Node 80:**
Char. 17: 0 → 1
Char. 42: 0 → 1
Char. 251: 0 → 1
Char. 264: 0 → 1

**Node 81:**
Char. 41: 0 → 1
Char. 48: 1 → 0
Char. 61: 1 → 2
Char. 112: 1 → 0
Char. 138: 0 → 1
Char. 146: 1 → 0
Char. 181: 1 → 0
Char. 192: 1 → 2
Char. 206: 2 → 0
Char. 226: 0 → 1
Char. 233: 0 → 1
Char. 267: 1 → 2

**Node 82:**
Char. 61: 1 → 3
Char. 90: 0 → 1
Char. 91: 0 → 1
Char. 109: 1 → 0
Char. 209: 1 → 0

**ANALYSIS 66**
**(NO *PROGANOCHELYS QUENSTEDTI*, *CANDELARIA BARBOURI*, AND *EUNOTOSAURUS AFRICANUS*, IMPLIED WEIGHTING, K = 2.75)**

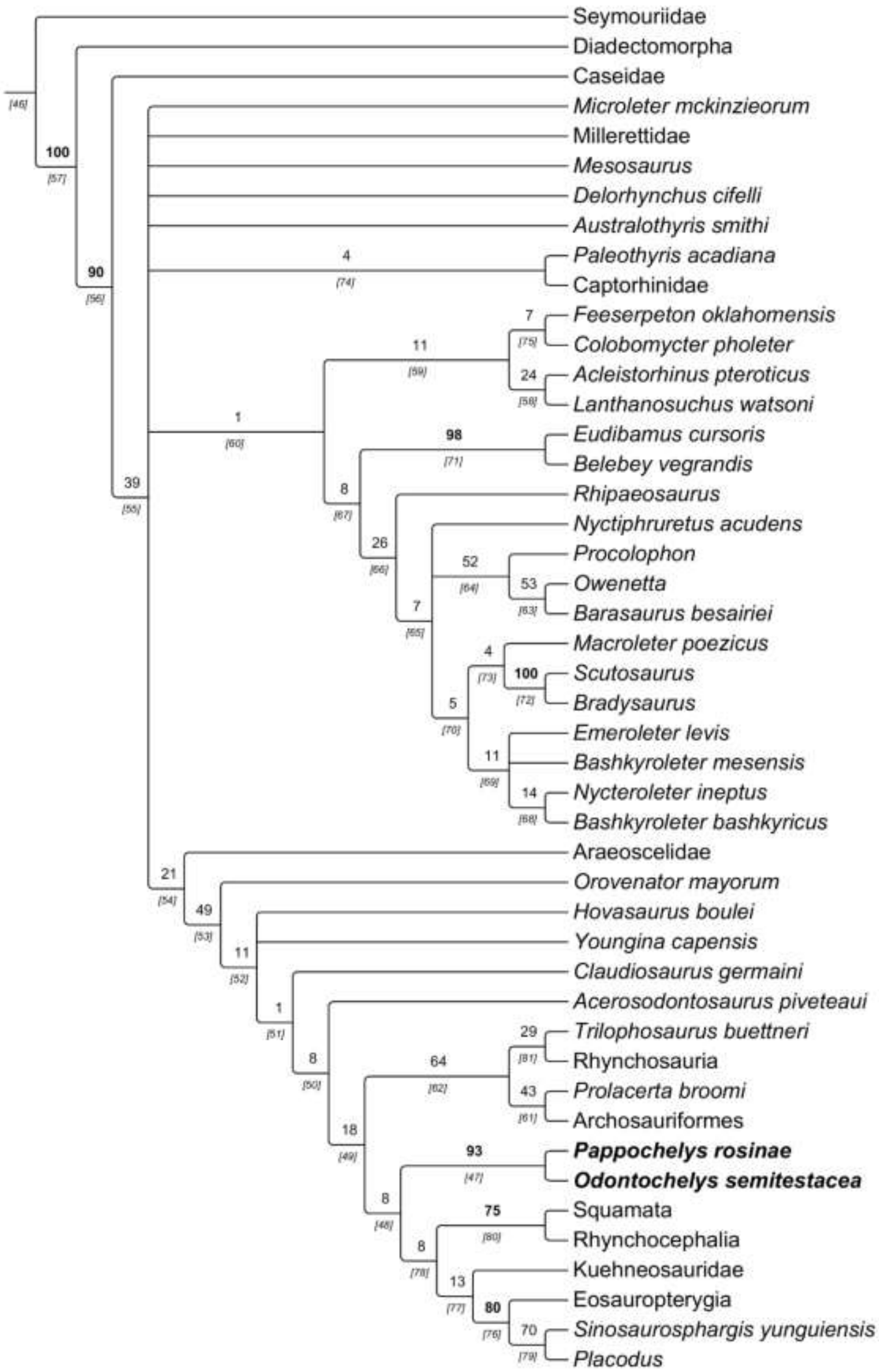

***Pappochelys rosinae*:**
Char. 1: 0 → 1
Char. 5: 1 → 0
Char. 12: 0 → 1
Char. 41: 0 → 1
Char. 43: 1 → 0
Char. 49: 0 → 1
Char. 169: 1 → 0
Char. 176: 1 → 0

***Odontochelys semitestacea*:**
Char. 46: 1 →oc 0
Char. 50: 0 → 1
Char. 88: 1 → 0
Char. 89: 1 → 0
Char. 93: 1 → 0
Char. 126: 1 → 0
Char. 195: 0 → 2
Char. 198: 0 → 1
Char. 240: 1 → 0
Char. 246: 1 → 2
Char. 259: 0 → 1
Char. 265: 1 → 0
Char. 270: 0 → 1

***Acerosodontosaurus piveteaui*:**
Char. 78: 1 → 0
Char. 128: 0 → 1
Char. 267: 1 → 0
Char. 278: 0 → 1

***Acleistorhinus pteroticus*:**
Char. 21: 0 → 1
Char. 146: 1 → 0

**Araeoscelidae:**
Char. 23: 0 → 1
Char. 28: 0 → 1
Char. 106: 0 → 1
Char. 169: 1 → 0
Char. 170: 0 → 1
Char. 197: 0 → 1
Char. 239: 0 → 1

**Archosauriformes:**
Char. 32: 0 → 1
Char. 94: 1 → 0
Char. 112: 1 → 0
Char. 123: 1 → 0
Char. 152: 0 → 1
Char. 154: 0 → 2
Char. 166: 1 → 0
Char. 171: 0 → 1
Char. 185: 0 → 1
Char. 201: 0 → 1
Char. 204: 0 → 1
Char. 218: 0 → 3
Char. 242: 0 → 1

***Australothyris smithi*:**
Char. 23: 0 → 1
Char. 34: 0 → 1
Char. 55: 1 → 0
Char. 71: 1 → 0
Char. 79: 0 → 1
Char. 85: 1 → 0
Char. 98: 1 → 0
Char. 100: 0 → 1
Char. 103: 0 → 1
Char. 110: 0 → 1
Char. 112: 0 → 1
Char. 123: 1 → 0
Char. 129: 0 → 1
Char. 131: 0 → 1
Char. 132: 1 → 0
Char. 144: 1 → 0
Char. 147: 0 → 1
Char. 149: 1 → 0
Char. 150: 0 → 1
Char. 166: 0 → 1

***Barasaurus besairiei*:**
Char. 33: 1 → 0

***Bashkyroleter bashkyricus*:**
Char. 275: 1 → 0

***Bashkyroleter mesensis*:**
Char. 12: 0 → 1
Char. 66: 1 → 0
Char. 169: 1 → 0

***Belebey vegrandis*:**
Char. 154: 1 → 0

***Bradysaurus* spp.:**
Char. 19: 0 → 1
Char. 25: 1 → 0
Char. 73: 0 → 1
Char. 79: 0 → 1
Char. 135: 1 → 0
Char. 249: 0 → 1

**Captorhinidae:**
Char. 3: 0 → 1
Char. 23: 0 → 1
Char. 25: 1 → 0
Char. 26: 0 → 1
Char. 73: 0 → 1
Char. 75: 0 → 1
Char. 108: 1 → 0
Char. 183: 1 → 0
Char. 201: 1 → 0
Char. 203: 1 → 2
Char. 216: 1 → 0

**Caseidae:**
Char. 25: 1 → 0
Char. 36: 0 → 1
Char. 38: 1 → 0
Char. 46: 1 → 0
Char. 50: 1 → 0
Char. 56: 0 → 1
Char. 170: 0 → 1
Char. 194: 0 → 1
Char. 273: 0 → 1
Char. 274: 0 → 1
Char. 278: 3 → 2

***Claudiosaurus germaini*:**
Char. 34: 0 → 1
Char. 36: 0 → 1
Char. 43: 1 → 0
Char. 56: 0 → 1
Char. 64: 0 → 1
Char. 105: 0 → 1
Char. 106: 0 → 1
Char. 130: 0 → 1
Char. 144: 1 → 0
Char. 187: 1 → 0
Char. 199: 0 → 1
Char. 201: 0 → 1
Char. 203: 1 → 2
Char. 204: 0 → 1
Char. 220: 0 → 1
Char. 222: 1 → 0

***Colobomycter pholeter*:**
Char. 21: 0 → 1
Char. 25: 1 → 0
Char. 84: 1 → 0
Char. 154: 1 → 0
Char. 167: 0 → 1
Char. 267: 0 → 1

***Delorhynchus cifelli*:**
Char. 5: 0 → 1
Char. 18: 0 → 1
Char. 20: 0 → 1
Char. 21: 0 → 1
Char. 26: 0 → 1
Char. 28: 0 → 1
Char. 33: 0 → 2
Char. 39: 0 → 1
Char. 52: 0 → 1
Char. 100: 0 → 1
Char. 111: 0 → 1
Char. 131: 0 → 1
Char. 147: 0 → 1
Char. 156: 1 → 0
Char. 159: 0 → 1
Char. 163: 0 → 1
Char. 166: 0 → 1
Char. 167: 0 → 1
Char. 189: 0 → 1
Char. 191: 0 → 1
Char. 192: 0 → 1
Char. 204: 0 → 2
Char. 267: 0 → 1

**Diadectomorpha:**
Char. 0: 0 → 1
Char. 64: 0 → 1
Char. 70: 0 → 1
Char. 122: 0 → 1
Char. 123: 1 → 0
Char. 146: 1 → 0
Char. 275: 1 → 0
Char. 278: 3 → 0

***Emeroleter levis*:**
Char. 51: 1 → 0

**Eosauropterygia:**
Char. 166: 1 → 0
Char. 174: 0 → 1
Char. 178: 0 → 1
Char. 194: 0 → 2
Char. 272: 1 → 0

***Eudibamus cursoris*:**
Char. 154: 1 → 2
Char. 163: 1 → 0

***Feeserpeton oklahomensis*:**
Char. 157: 0 → 1

***Hovasaurus boulei*:**
Char. 41: 0 → 1
Char. 55: 1 → 0
Char. 60: 1 → 0
Char. 72: 2 → 1
Char. 77: 0 → 2
Char. 78: 1 → 0
Char. 79: 0 → 1
Char. 93: 1 → 0
Char. 113: 0 → 1
Char. 138: 0 → 1
Char. 146: 1 → 0
Char. 154: 0 → 1
Char. 204: 0 → 2
Char. 206: 0 → 2
Char. 220: 0 → 1
Char. 278: 0 → 3

**Kuehneosauridae:**
Char. 7: 0 → 1
Char. 24: 0 → 1
Char. 27: 0 → 1
Char. 33: 2 → 1
Char. 43: 1 → 0
Char. 79: 0 → 2
Char. 140: 1 → 0
Char. 147: 1 → 0
Char. 148: 1 → 0
Char. 235: 2 → 1
Char. 245: 0 → 1
Char. 272: 1 → 2
Char. 278: 0 → 3

***Lanthanosuchus watsoni*:**
Char. 25: 1 → 0
Char. 86: 0 → 1
Char. 98: 1 → 0
Char. 138: 0 → 1
Char. 144: 1 → 0
Char. 154: 1 → 2

***Macroleter poezicus*:**
Char. 9: 0 → 1
Char. 26: 0 → 1
Char. 66: 1 → 2
Char. 126: 0 → 1
Char. 134: 0 → 2
Char. 140: 0 → 1
Char. 146: 1 → 0
Char. 169: 1 → 0

***Mesosaurus* spp.:**
Char. 0: 0 → 1
Char. 2: 0 → 1
Char. 6: 0 → 1
Char. 8: 0 → 1
Char. 9: 0 → 1
Char. 13: 0 → 1
Char. 19: 0 → 1
Char. 23: 0 → 1
Char. 26: 0 → 1
Char. 29: 1 → 0
Char. 33: 0 → 1
Char. 38: 1 → 0
Char. 41: 0 → 1
Char. 50: 1 → 0
Char. 67: 0 → 1
Char. 76: 0 → 1
Char. 84: 1 → 0
Char. 85: 1 → 0
Char. 94: 0 → 1
Char. 107: 0 → 1
Char. 109: 0 → 1
Char. 111: 0 → 1
Char. 115: 0 → 1
Char. 146: 1 → 0
Char. 148: 0 → 1
Char. 149: 1 → 0
Char. 164: 0 → 1
Char. 167: 0 → 1
Char. 176: 0 → 1
Char. 183: 1 → 0
Char. 184: 0 → 1
Char. 199: 0 → 1
Char. 202: 0 → 1
Char. 204: 0 → 2
Char. 206: 0 → 1
Char. 207: 0 → 1
Char. 209: 0 → 1
Char. 217: 0 → 1
Char. 219: 0 → 1
Char. 220: 0 → 1
Char. 231: 0 → 1
Char. 272: 2 → 0
Char. 278: 3 → 0

***Microleter mckinzieorum*:**
Char. 0: 0 → 1
Char. 18: 0 → 1
Char. 25: 1 → 0
Char. 36: 0 → 1
Char. 39: 0 → 1
Char. 51: 0 → 2
Char. 56: 0 → 1
Char. 70: 0 → 1
Char. 76: 0 → 1
Char. 79: 0 → 1
Char. 94: 0 → 1
Char. 106: 0 → 1
Char. 110: 0 → 1
Char. 132: 1 → 0
Char. 159: 0 → 1
Char. 276: 0 → 1
Char. 278: 3 → 1

**Millerettidae:**
Char. 25: 1 → 0
Char. 44: 1 → 0
Char. 56: 0 → 1
Char. 66: 0 → 2
Char. 78: 1 → 0
Char. 80: 1 → 0
Char. 84: 1 → 2
Char. 88: 1 → 0
Char. 96: 1 → 0
Char. 124: 0 → 1
Char. 127: 0 → 1
Char. 135: 0 → 1
Char. 145: 0 → 1
Char. 202: 0 → 1
Char. 211: 0 → 1
Char. 230: 0 → 1
Char. 234: 0 → 1
Char. 248: 0 → 1
Char. 252: 0 → 1
Char. 253: 0 → 1

***Nycteroleter ineptus*:**
Char. 66: 1 → 0
Char. 278: 0 → 3

***Nyctiphruretus acudens*:**
Char. 21: 0 → 1
Char. 33: 1 → 2
Char. 41: 0 → 1
Char. 66: 1 → 2
Char. 81: 1 → 0
Char. 84: 1 → 2
Char. 94: 0 → 1
Char. 166: 1 → 0
Char. 215: 1 → 0
Char. 224: 0 → 1
Char. 226: 1 → 0
Char. 266: 0 → 1
Char. 272: 2 → 1
Char. 276: 0 → 1

***Orovenator mayorum*:**
Char. 8: 0 → 1
Char. 36: 0 → 1
Char. 160: 0 → 1
Char. 165: 0 → 1

***Owenetta* spp.:**
Char. 142: 1 → 0
Char. 169: 1 → 0

***Paleothyris acadiana*:**
Char. 38: 1 → 0
Char. 50: 1 → 0
Char. 66: 1 → 2
Char. 102: 0 → 1
Char. 146: 1 → 0
Char. 237: 0 → 1
Char. 239: 0 → 1

***Placodus* spp.:**
Char. 0: 1 → 2
Char. 9: 0 → 1
Char. 12: 0 → 1
Char. 13: 0 → 1
Char. 19: 0 → 1
Char. 31: 0 → 1
Char. 46: 1 → 0
Char. 57: 0 → 1
Char. 78: 1 → 0
Char. 93: 1 → 0
Char. 102: 1 → 2
Char. 109: 1 → 0
Char. 140: 1 → 0
Char. 155: 0 → 1
Char. 163: 1 → 0
Char. 164: 0 → 1

***Procolophon* spp.:**
Char. 41: 0 → 1
Char. 69: 0 → 1
Char. 79: 0 → 1
Char. 86: 0 → 1
Char. 88: 0 → 1
Char. 101: 0 → 1
Char. 117: 1 → 0
Char. 141: 0 → 1
Char. 149: 1 → 0
Char. 203: 1 → 2
Char. 204: 0 → 1
Char. 230: 0 → 1
Char. 237: 0 → 1
Char. 238: 0 → 1
Char. 239: 1 → 0
Char. 272: 2 → 1
Char. 278: 3 → 0

***Prolacerta broomi*:**
Char. 66: 1 → 0
Char. 67: 1 → 0
Char. 80: 1 → 0
Char. 139: 1 → 0
Char. 147: 1 → 0
Char. 192: 1 → 0
Char. 203: 1 → 2

***Rhipaeosaurus* spp.:**
Char. 172: 0 → 1
Char. 277: 0 → 1

**Rhynchocephalia:**
Char. 0: 1 → 2
Char. 23: 1 → 0
Char. 24: 0 → 1
Char. 77: 0 → 1
Char. 88: 1 → 0
Char. 94: 1 → 0
Char. 117: 12 → 0
Char. 139: 1 → 0
Char. 205: 1 → 0

**Rhynchosauria:**
Char. 0: 1 → 0
Char. 7: 0 → 1
Char. 9: 0 → 1
Char. 26: 0 → 1
Char. 68: 0 → 1
Char. 99: 1 → 0
Char. 160: 0 → 1
Char. 161: 0 → 1
Char. 171: 0 → 2
Char. 223: 0 → 1
Char. 241: 0 → 1

***Scutosaurus* spp.:**
Char. 175: 0 → 1
Char. 190: 0 → 1
Char. 218: 0 → 1
Char. 243: 0 → 2
Char. 244: 0 → 1
Char. 251: 0 → 1

***Sinosaurosphargis yunguiensis*:**
Char. 8: 0 → 1
Char. 30: 0 → 1
Char. 53: 0 → 1
Char. 89: 1 → 0
Char. 127: 1 → 0
Char. 150: 1 → 0
Char. 154: 0 → 2
Char. 167: 1 → 0
Char. 253: 0 → 1
Char. 255: 0 → 1

**Squamata:**
Char. 45: 0 → 1
Char. 79: 0 → 2
Char. 80: 1 → 0
Char. 92: 1 → 0
Char. 109: 1 → 0
Char. 160: 0 → 1
Char. 200: 0 → 1
Char. 245: 0 → 1

***Trilophosaurus buettneri*:**
Char. 5: 1 → 0
Char. 11: 0 → 1
Char. 55: 1 → 0
Char. 93: 1 → 0
Char. 104: 0 → 1
Char. 106: 0 → 1
Char. 113: 0 → 1
Char. 122: 0 → 1
Char. 123: 1 → 0
Char. 136: 1 → 0
Char. 144: 1 → 0
Char. 154: 0 → 1
Char. 157: 0 → 1
Char. 159: 1 → 0
Char. 177: 0 → 12
Char. 194: 0 → 1
Char. 203: 1 → 2
Char. 207: 1 → 0
Char. 208: 1 → 0
Char. 272: 1 → 0

***Youngina capensis*:**
Char. 56: 0 → 1
Char. 75: 0 → 1
Char. 170: 0 → 1
Char. 211: 0 → 1
Char. 231: 0 → 1
Char. 239: 0 → 1

**Node 47:**
Char. 62: 1 → 0
Char. 194: 0 → 2
Char. 219: 1 → 0
Char. 220: 0 → 1
Char. 241: 0 → 1
Char. 246: 0 → 1
Char. 247: 0 → 1
Char. 252: 0 → 1
Char. 254: 0 → 1
Char. 255: 0 → 1
Char. 256: 0 → 1
Char. 268: 0 → 1
Char. 269: 0 → 1

**Node 48:**
Char. 33: 1 → 2
Char. 184: 0 → 1
Char. 210: 0 → 1
Char. 223: 0 → 1
Char. 235: 1 → 2

**Node 49:**
Char. 176: 0 → 1
Char. 205: 0 → 1

**Node 50:**
Char. 166: 0 → 1
Char. 208: 0 → 1

**Node 51:**
Char. 23: 0 → 1
Char. 70: 0 → 1
Char. 73: 0 → 1
Char. 94: 0 → 1
Char. 131: 0 → 1
Char. 148: 0 → 1
Char. 272: 2 → 1

**Node 52:**
Char. 33: 0 → 1
Char. 135: 0 → 1
Char. 159: 0 → 1
Char. 267: 0 → 1
Char. 278: 1 → 0

**Node 53:**
Char. 20: 0 → 1
Char. 62: 0 → 1

**Node 54:**
Char. 0: 0 → 1
Char. 27: 0 → 1
Char. 29: 1 → 0
Char. 38: 1 → 0
Char. 40: 2 → 0
Char. 59: 0 → 1
Char. 60: 0 → 1
Char. 67: 0 → 1
Char. 72: 1 → 2
Char. 84: 1 → 0
Char. 89: 0 → 1
Char. 111: 0 → 1
Char. 112: 0 → 1
Char. 120: 0 → 1
Char. 154: 1 → 0
Char. 192: 0 → 1
Char. 193: 0 → 1
Char. 222: 0 → 1
Char. 224: 0 → 1
Char. 234: 0 → 1
Char. 237: 0 → 1
Char. 266: 0 → 1
Char. 278: 3 → 1

**Node 55:**
Char. 29: 0 → 1
Char. 40: 0 → 2
Char. 74: 0 → 1
Char. 80: 0 → 1
Char. 84: 0 → 1
Char. 88: 0 → 1
Char. 132: 0 → 1
Char. 149: 0 → 1
Char. 156: 0 → 1
Char. 169: 0 → 1
Char. 201: 0 → 1
Char. 203: 0 → 1
Char. 235: 0 → 1

**Node 56:**
Char. 72: 0 → 1
Char. 79: 1 → 0
Char. 81: 0 → 1
Char. 93: 0 → 1
Char. 97: 0 → 1
Char. 104: 1 → 0
Char. 144: 0 → 1
Char. 173: 0 → 1
Char. 183: 0 → 1

**Node 58:**
Char. 113: 0 → 1
Char. 114: 0 → 1

**Node 59:**
Char. 47: 0 → 1
Char. 79: 0 → 1
Char. 140: 0 → 2

**Node 60:**
Char. 5: 0 → 1
Char. 137: 0 → 1
Char. 159: 0 → 1
Char. 163: 0 → 1
Char. 166: 0 → 1
Char. 192: 0 → 1

**Node 61:**
Char. 19: 0 → 1
Char. 92: 1 → 0

**Node 62:**
Char. 4: 0 → 1
Char. 15: 0 → 1
Char. 29: 0 → 1
Char. 48: 1 → 0
Char. 213: 0 → 1
Char. 228: 0 → 1
Char. 275: 0 → 1

**Node 63:**
Char. 73: 0 → 1
Char. 131: 1 → 0
Char. 205: 0 → 1
Char. 273: 0 → 1
Char. 276: 0 → 1

**Node 64:**
Char. 18: 0 → 1
Char. 23: 0 → 1
Char. 25: 1 → 0
Char. 37: 0 → 1
Char. 103: 0 → 1
Char. 106: 0 → 1
Char. 107: 0 → 1
Char. 126: 0 → 1
Char. 150: 0 → 1

**Node 65:**
Char. 186: 0 → 1

**Node 66:**
Char. 194: 0 → 1
Char. 201: 1 → 0
Char. 211: 0 → 1
Char. 226: 0 → 1

**Node 67:**
Char. 38: 1 → 2
Char. 39: 0 → 1
Char. 49: 0 → 1
Char. 66: 0 → 1
Char. 88: 1 → 0
Char. 112: 0 → 1
Char. 148: 0 → 1

**Node 68:**
Char. 87: 0 → 1

**Node 69:**
Char. 79: 0 → 1
Char. 133: 0 → 1

**Node 70:**
Char. 100: 0 → 1
Char. 113: 0 → 1

**Node 71:**
Char. 50: 1 → 0
Char. 58: 0 → 1
Char. 59: 0 → 1
Char. 60: 0 → 1
Char. 72: 1 → 2
Char. 104: 0 → 1
Char. 105: 0 → 1
Char. 106: 0 → 2
Char. 107: 0 → 1
Char. 109: 0 → 1
Char. 146: 1 → 0

**Node 72:**
Char. 23: 0 → 1
Char. 33: 1 → 0
Char. 38: 2 → 1
Char. 39: 1 → 0
Char. 42: 0 → 1
Char. 43: 0 → 1
Char. 46: 1 → 0
Char. 49: 1 → 0
Char. 71: 1 → 0
Char. 83: 0 → 2
Char. 101: 0 → 1
Char. 103: 0 → 1
Char. 106: 0 → 1
Char. 161: 0 → 1
Char. 163: 1 → 0
Char. 172: 0 → 2
Char. 174: 0 → 1
Char. 188: 0 → 1
Char. 203: 1 → 2
Char. 212: 0 → 1
Char. 216: 1 → 0
Char. 236: 0 → 1
Char. 238: 0 → 2
Char. 239: 1 → 0
Char. 241: 0 → 1
Char. 242: 0 → 1
Char. 273: 0 → 1
Char. 274: 0 → 1
Char. 275: 1 → 0

**Node 73:**
Char. 52: 0 → 1
Char. 75: 0 → 1
Char. 84: 1 → 0
Char. 87: 0 → 1
Char. 121: 0 → 1
Char. 230: 0 → 1

**Node 74:**
Char. 29: 1 → 0
Char. 44: 1 → 0
Char. 59: 0 → 1
Char. 60: 0 → 1
Char. 66: 0 → 1
Char. 67: 0 → 1
Char. 72: 1 → 2
Char. 78: 1 → 0
Char. 84: 1 → 0
Char. 88: 1 → 0
Char. 93: 1 → 0
Char. 111: 0 → 1
Char. 123: 1 → 0
Char. 129: 0 → 1
Char. 154: 1 → 0
Char. 169: 1 → 0
Char. 170: 0 → 1
Char. 197: 0 → 1

**Node 75:**
Char. 107: 0 → 1

**Node 76:**
Char. 2: 0 → 1
Char. 6: 0 → 1
Char. 186: 0 → 1
Char. 198: 0 → 1
Char. 200: 0 → 1
Char. 220: 0 → 1
Char. 267: 1 → 0

**Node 77:**
Char. 98: 1 → 0
Char. 113: 0 → 1
Char. 181: 0 → 1

**Node 78:**
Char. 123: 1 → 0
Char. 201: 0 → 1
Char. 229: 0 → 1

**Node 79:**
Char. 17: 0 → 1
Char. 42: 0 → 1
Char. 251: 0 → 1
Char. 264: 0 → 1

**Node 80:**
Char. 41: 0 → 1
Char. 48: 1 → 0
Char. 61: 1 → 2
Char. 138: 0 → 1
Char. 146: 1 → 0
Char. 192: 1 → 2
Char. 233: 0 → 1
Char. 267: 1 → 2

**Node 81:**
Char. 61: 1 → 3
Char. 90: 0 → 1
Char. 91: 0 → 1
Char. 109: 1 → 0
Char. 209: 1 → 0

**ANALYSIS 67**
**(NO *CANDELARIA BARBOURI* AND *PAPPOCHELYS ROSINAE*, IMPLIED WEIGHTING, K = 2.5)**

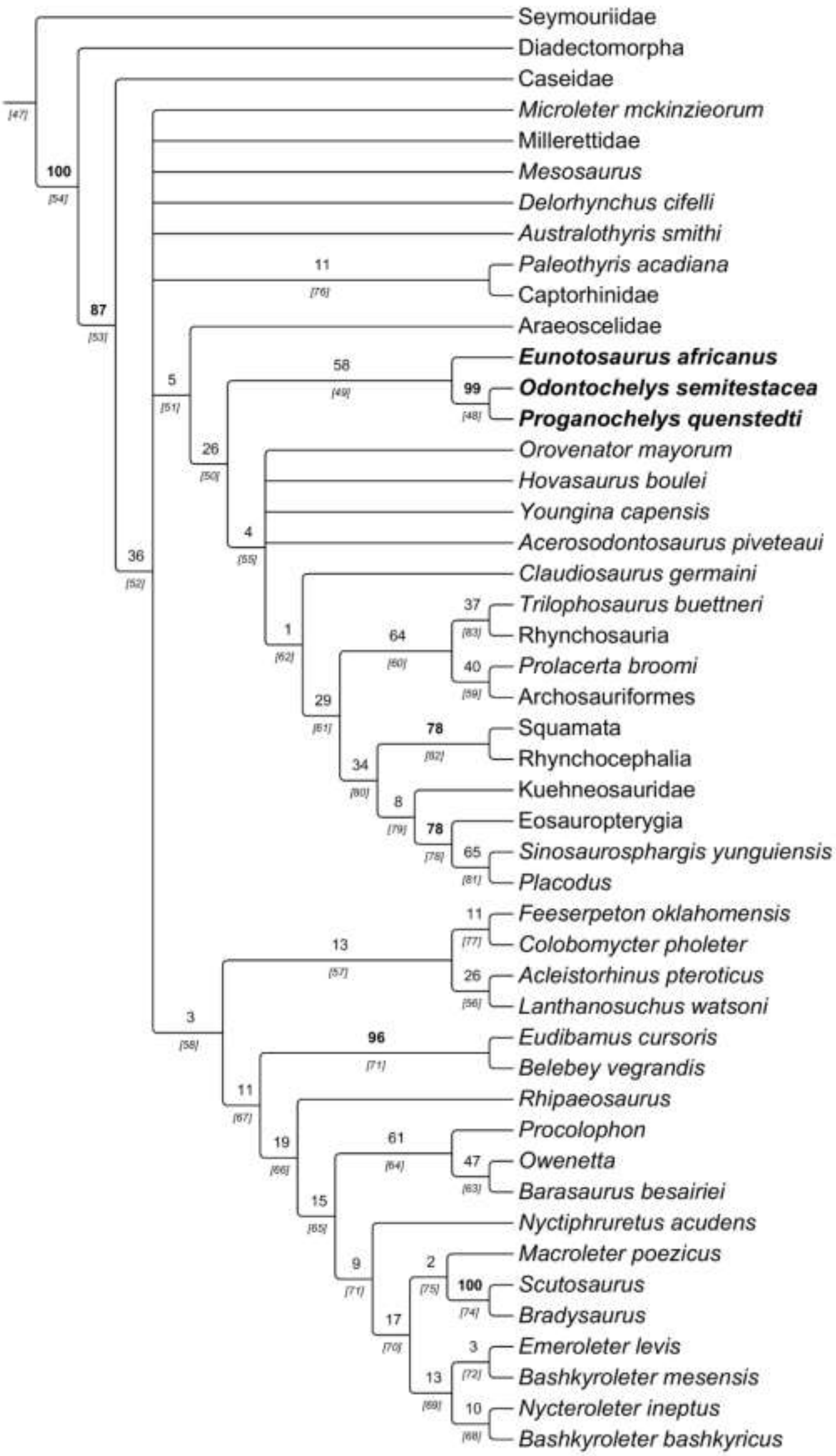

***Proganochelys quenstedti*:**
Char. 8: 0 → 1
Char. 11: 0 → 1
Char. 43: 1 → 0
Char. 106: 0 → 1
Char. 108: 1 → 0
Char. 109: 0 → 1
Char. 126: 0 → 1
Char. 128: 0 → 1
Char. 175: 0 → 1
Char. 244: 0 → 1
Char. 262: 0 → 1

***Odontochelys semitestacea*:**
Char. 202: 0 → 1
Char. 206: 0 → 2

***Eunotosaurus africanus*:**
Char. 19: 0 → 1
Char. 55: 1 → 0
Char. 59: 1 → 0
Char. 60: 1 → 0
Char. 67: 1 → 0
Char. 72: 2 → 1
Char. 76: 0 → 1
Char. 97: 1 → 0
Char. 103: 0 → 1
Char. 153: 1 → 0
Char. 192: 1 → 0
Char. 211: 0 → 1
Char. 222: 1 → 0
Char. 237: 1 → 0
Char. 249: 0 → 2
Char. 263: 0 → 1
Char. 273: 0 → 1
Char. 274: 0 → 1
Char. 277: 0 → 1

***Acerosodontosaurus piveteaui*:**
Char. 23: 0 → 1
Char. 78: 1 → 0
Char. 81: 1 → 0
Char. 127: 1 → 0
Char. 128: 0 → 1
Char. 155: 0 → 1
Char. 206: 0 → 2
Char. 208: 0 → 1

***Acleistorhinus pteroticus*:**
Char. 21: 0 → 1
Char. 146: 1 → 0

**Araeoscelidae:**
Char. 23: 0 → 1
Char. 28: 0 → 1
Char. 106: 0 → 1
Char. 117: 0 → 2
Char. 169: 1 → 0
Char. 170: 0 → 1
Char. 197: 0 → 1
Char. 239: 0 → 1

**Archosauriformes:**
Char. 32: 0 → 1
Char. 94: 1 → 0
Char. 112: 1 → 0
Char. 152: 0 → 1
Char. 154: 0 → 2
Char. 166: 1 → 0
Char. 171: 0 → 1
Char. 185: 0 → 1
Char. 204: 0 → 1
Char. 218: 0 → 3
Char. 242: 0 → 1

***Australothyris smithi*:**
Char. 23: 0 → 1
Char. 34: 0 → 1
Char. 55: 1 → 0
Char. 71: 1 → 0
Char. 79: 0 → 1
Char. 85: 1 → 0
Char. 98: 1 → 0
Char. 100: 0 → 1
Char. 103: 0 → 1
Char. 110: 0 → 1
Char. 112: 0 → 1
Char. 123: 1 → 0
Char. 129: 0 → 1
Char. 131: 0 → 1
Char. 132: 1 → 0
Char. 144: 1 → 0
Char. 147: 0 → 1
Char. 149: 1 → 0
Char. 150: 0 → 1
Char. 166: 0 → 1

***Barasaurus besairiei*:**
Char. 33: 1 → 0

***Bashkyroleter bashkyricus*:**
Char. 275: 1 → 0

***Bashkyroleter mesensis*:**
Char. 12: 0 → 1
Char. 66: 1 → 0
Char. 169: 1 → 0

***Belebey vegrandis*:**
Char. 154: 1 → 0

***Bradysaurus* spp.:**
Char. 19: 0 → 1
Char. 25: 1 → 0
Char. 73: 0 → 1
Char. 79: 0 → 1
Char. 135: 1 → 0
Char. 249: 0 → 1

**Captorhinidae:**
Char. 3: 0 → 1
Char. 23: 0 → 1
Char. 25: 1 → 0
Char. 26: 0 → 1
Char. 73: 0 → 1
Char. 75: 0 → 1
Char. 108: 1 → 0
Char. 183: 1 → 0
Char. 201: 1 → 0
Char. 203: 1 → 2
Char. 216: 1 → 0

**Caseidae:**
Char. 25: 1 → 0
Char. 36: 0 → 1
Char. 38: 1 → 0
Char. 46: 1 → 0
Char. 50: 1 → 0
Char. 56: 0 → 1
Char. 170: 0 → 1
Char. 194: 0 → 1
Char. 273: 0 → 1
Char. 274: 0 → 1
Char. 278: 3 → 2

***Claudiosaurus germaini*:**
Char. 34: 0 → 1
Char. 36: 0 → 1
Char. 43: 1 → 0
Char. 64: 0 → 1
Char. 105: 0 → 1
Char. 127: 1 → 0
Char. 130: 0 → 1
Char. 141: 1 → 0
Char. 144: 1 → 0
Char. 166: 1 → 0
Char. 187: 1 → 0
Char. 199: 0 → 1
Char. 203: 1 → 2
Char. 204: 0 → 1
Char. 222: 1 → 0

***Colobomycter pholeter*:**
Char. 21: 0 → 1
Char. 25: 1 → 0
Char. 84: 1 → 0
Char. 154: 1 → 0
Char. 167: 0 → 1
Char. 267: 0 → 1

***Delorhynchus cifelli*:**
Char. 5: 0 → 1
Char. 18: 0 → 1
Char. 20: 0 → 1
Char. 21: 0 → 1
Char. 26: 0 → 1
Char. 28: 0 → 1
Char. 33: 0 → 2
Char. 39: 0 → 1
Char. 52: 0 → 1
Char. 100: 0 → 1
Char. 111: 0 → 1
Char. 117: 0 → 1
Char. 131: 0 → 1
Char. 147: 0 → 1
Char. 156: 1 → 0
Char. 159: 0 → 1
Char. 163: 0 → 1
Char. 166: 0 → 1
Char. 167: 0 → 1
Char. 189: 0 → 1
Char. 191: 0 → 1
Char. 192: 0 → 1
Char. 204: 0 → 2
Char. 267: 0 → 1

**Diadectomorpha:**
Char. 0: 0 → 1
Char. 64: 0 → 1
Char. 70: 0 → 1
Char. 122: 0 → 1
Char. 123: 1 → 0
Char. 146: 1 → 0
Char. 275: 1 → 0
Char. 278: 3 → 0

***Emeroleter levis*:**
Char. 51: 1 → 0

**Eosauropterygia:**
Char. 166: 1 → 0
Char. 174: 0 → 1
Char. 178: 0 → 1
Char. 194: 0 → 2
Char. 272: 1 → 0

***Eudibamus cursoris*:**
Char. 154: 1 → 2
Char. 163: 1 → 0

***Feeserpeton oklahomensis*:**
Char. 157: 0 → 1

***Hovasaurus boulei*:**
Char. 41: 0 → 1
Char. 55: 1 → 0
Char. 60: 1 → 0
Char. 72: 2 → 1
Char. 77: 0 → 2
Char. 78: 1 → 0
Char. 79: 0 → 1
Char. 93: 1 → 0
Char. 113: 0 → 1
Char. 138: 0 → 1
Char. 141: 1 → 0
Char. 146: 1 → 0
Char. 204: 0 → 2
Char. 206: 0 → 2

**Kuehneosauridae:**
Char. 7: 0 → 1
Char. 27: 0 → 1
Char. 43: 1 → 0
Char. 79: 0 → 2
Char. 107: 1 → 0
Char. 140: 1 → 0
Char. 147: 1 → 0
Char. 148: 1 → 0
Char. 245: 0 → 1
Char. 272: 1 → 2
Char. 278: 0 → 3

***Lanthanosuchus watsoni*:**
Char. 25: 1 → 0
Char. 86: 0 → 1
Char. 98: 1 → 0
Char. 138: 0 → 1
Char. 144: 1 → 0
Char. 154: 1 → 2

***Macroleter poezicus*:**
Char. 9: 0 → 1
Char. 26: 0 → 1
Char. 66: 1 → 2
Char. 126: 0 → 1
Char. 134: 0 → 2
Char. 140: 0 → 1
Char. 146: 1 → 0
Char. 169: 1 → 0

***Mesosaurus* spp.:**
Char. 0: 0 → 1
Char. 2: 0 → 1
Char. 6: 0 → 1
Char. 8: 0 → 1
Char. 9: 0 → 1
Char. 13: 0 → 1
Char. 19: 0 → 1
Char. 23: 0 → 1
Char. 26: 0 → 1
Char. 29: 1 → 0
Char. 33: 0 → 1
Char. 38: 1 → 0
Char. 41: 0 → 1
Char. 50: 1 → 0
Char. 67: 0 → 1
Char. 76: 0 → 1
Char. 84: 1 → 0
Char. 85: 1 → 0
Char. 94: 0 → 1
Char. 107: 0 → 1
Char. 109: 0 → 1
Char. 111: 0 → 1
Char. 115: 0 → 1
Char. 146: 1 → 0
Char. 148: 0 → 1
Char. 149: 1 → 0
Char. 164: 0 → 1
Char. 167: 0 → 1
Char. 176: 0 → 1
Char. 183: 1 → 0
Char. 184: 0 → 1
Char. 199: 0 → 1
Char. 202: 0 → 1
Char. 204: 0 → 2
Char. 206: 0 → 1
Char. 207: 0 → 1
Char. 209: 0 → 1
Char. 217: 0 → 1
Char. 219: 0 → 1
Char. 220: 0 → 1
Char. 231: 0 → 1
Char. 260: 2 → 0
Char. 272: 2 → 0
Char. 278: 3 → 0

***Microleter mckinzieorum*:**
Char. 0: 0 → 1
Char. 18: 0 → 1
Char. 25: 1 → 0

Char. 36: 0 → 1
Char. 39: 0 → 1
Char. 51: 0 → 2
Char. 56: 0 → 1
Char. 70: 0 → 1
Char. 76: 0 → 1
Char. 79: 0 → 1
Char. 94: 0 → 1
Char. 106: 0 → 1
Char. 110: 0 → 1
Char. 132: 1 → 0
Char. 159: 0 → 1
Char. 276: 0 → 1
Char. 278: 3 → 1

**Millerettidae:**
Char. 25: 1 → 0
Char. 44: 1 → 0
Char. 56: 0 → 1
Char. 66: 0 → 2
Char. 78: 1 → 0
Char. 80: 1 → 0
Char. 84: 1 → 2
Char. 88: 1 → 0
Char. 96: 1 → 0
Char. 117: 0 → 1
Char. 124: 0 → 1
Char. 127: 0 → 1
Char. 135: 0 → 1
Char. 145: 0 → 1
Char. 202: 0 → 1
Char. 211: 0 → 1
Char. 230: 0 → 1
Char. 234: 0 → 1
Char. 248: 0 → 1
Char. 252: 0 → 1
Char. 253: 0 → 1

***Nycteroleter ineptus*:**
Char. 66: 1 → 0

***Nyctiphruretus acudens*:**
Char. 21: 0 → 1
Char. 41: 0 → 1
Char. 66: 1 → 2
Char. 81: 1 → 0
Char. 84: 1 → 2
Char. 94: 0 → 1
Char. 110: 1 → 0
Char. 166: 1 → 0
Char. 215: 1 → 0
Char. 224: 0 → 1
Char. 226: 1 → 0
Char. 266: 0 → 1
Char. 272: 2 → 1
Char. 276: 0 → 1

***Orovenator mayorum*:**
Char. 8: 0 → 1
Char. 33: 1 → 0
Char. 36: 0 → 1
Char. 43: 1 → 0
Char. 50: 0 → 1
Char. 92: 1 → 0
Char. 135: 1 → 0
Char. 159: 1 → 0
Char. 160: 0 → 1
Char. 165: 0 → 1

***Owenetta* spp.:**
Char. 142: 1 → 0
Char. 169: 1 → 0

***Paleothyris acadiana*:**
Char. 38: 1 → 0
Char. 50: 1 → 0
Char. 66: 1 → 2
Char. 102: 0 → 1
Char. 146: 1 → 0
Char. 237: 0 → 1
Char. 239: 0 → 1

***Placodus* spp.:**
Char. 0: 1 → 2
Char. 9: 0 → 1
Char. 12: 0 → 1
Char. 13: 0 → 1
Char. 19: 0 → 1
Char. 31: 0 → 1
Char. 46: 1 → 0
Char. 57: 0 → 1
Char. 78: 1 → 0
Char. 93: 1 → 0
Char. 102: 1 → 2
Char. 109: 1 → 0
Char. 140: 1 → 0
Char. 155: 0 → 1
Char. 163: 1 → 0
Char. 164: 0 → 1

***Procolophon* spp.:**
Char. 41: 0 → 1
Char. 69: 0 → 1
Char. 79: 0 → 1
Char. 86: 0 → 1
Char. 88: 0 → 1
Char. 101: 0 → 1
Char. 141: 0 → 1
Char. 149: 1 → 0
Char. 203: 1 → 2
Char. 204: 0 → 1
Char. 230: 0 → 1
Char. 237: 0 → 1
Char. 238: 0 → 1
Char. 239: 1 → 0
Char. 272: 2 → 1
Char. 278: 3 → 0

***Prolacerta broomi*:**
Char. 58: 1 → 0
Char. 66: 1 → 0
Char. 67: 1 → 0
Char. 80: 1 → 0
Char. 139: 1 → 0
Char. 147: 1 → 0
Char. 192: 1 → 0
Char. 203: 1 → 2
Char. 206: 0 → 12

***Rhipaeosaurus* spp.:**
Char. 110: 1 → 0
Char. 172: 0 → 1
Char. 277: 0 → 1

**Rhynchocephalia:**
Char. 0: 1 → 2
Char. 23: 1 → 0
Char. 77: 0 → 1
Char. 88: 1 → 0
Char. 94: 1 → 0
Char. 117: 12 → 0
Char. 139: 1 → 0
Char. 167: 1 → 0
Char. 205: 1 → 0

**Rhynchosauria:**
Char. 0: 1 → 0
Char. 7: 0 → 1
Char. 9: 0 → 1
Char. 26: 0 → 1
Char. 44: 1 → 0
Char. 68: 0 → 1
Char. 99: 1 → 0
Char. 150: 1 → 0
Char. 160: 0 → 1
Char. 161: 0 → 1
Char. 171: 0 → 2
Char. 182: 1 → 0
Char. 223: 0 → 1
Char. 241: 0 → 1

***Scutosaurus* spp.:**
Char. 175: 0 → 1
Char. 190: 0 → 1
Char. 218: 0 → 1
Char. 243: 0 → 2
Char. 244: 0 → 1
Char. 251: 0 → 1

***Sinosaurosphargis yunguiensis*:**
Char. 8: 0 → 1
Char. 30: 0 → 1
Char. 53: 0 → 1
Char. 89: 1 → 0
Char. 127: 1 → 0
Char. 150: 1 → 0
Char. 154: 0 → 2
Char. 167: 1 → 0
Char. 253: 0 → 1
Char. 255: 0 → 1

**Squamata:**
Char. 45: 0 → 1
Char. 79: 0 → 2
Char. 80: 1 → 0
Char. 92: 1 → 0
Char. 109: 1 → 0
Char. 160: 0 → 1
Char. 200: 0 → 1
Char. 245: 0 → 1

***Trilophosaurus buettneri*:**
Char. 5: 1 → 0
Char. 11: 0 → 1
Char. 55: 1 → 0
Char. 93: 1 → 0
Char. 104: 0 → 1
Char. 113: 0 → 1
Char. 122: 0 → 1
Char. 136: 1 → 0
Char. 144: 1 → 0
Char. 154: 0 → 1
Char. 157: 0 → 1
Char. 159: 1 → 0
Char. 177: 0 → 12
Char. 194: 0 → 1
Char. 203: 1 → 2
Char. 207: 1 → 0
Char. 208: 1 → 0
Char. 272: 1 → 0

***Youngina capensis*:**
Char. 5: 1 → 0
Char. 25: 0 → 1
Char. 44: 1 → 0
Char. 75: 0 → 1
Char. 92: 1 → 0
Char. 134: 0 → 1
Char. 163: 1 → 0
Char. 170: 0 → 1
Char. 211: 0 → 1
Char. 239: 0 → 1
Char. 267: 0 → 1

**Node 48:**
Char. 46: 1 → 0
Char. 65: 0 → 1
Char. 88: 1 → 0
Char. 89: 1 → 0
Char. 93: 1 → 0
Char. 176: 0 → 1
Char. 184: 0 → 1
Char. 195: 0 → 2
Char. 198: 0 → 1
Char. 205: 0 → 1
Char. 210: 0 → 1
Char. 223: 0 → 1
Char. 232: 0 → 1
Char. 241: 0 → 1
Char. 246: 0 → 2
Char. 254: 0 → 1
Char. 255: 0 → 1
Char. 256: 0 → 1
Char. 258: 0 → 1
Char. 259: 0 → 1
Char. 267: 0 → 1
Char. 268: 0 → 1
Char. 269: 0 → 1
Char. 270: 0 → 1
Char. 272: 2 → 1

**Node 49:**
Char. 0: 1 → 2
Char. 15: 0 → 1
Char. 33: 1 → 2
Char. 42: 0 → 1
Char. 44: 1 → 0
Char. 64: 0 → 1
Char. 73: 0 → 1
Char. 81: 1 → 0
Char. 84: 01 → 2
Char. 130: 0 → 1
Char. 134: 0 → 2
Char. 147: 0 → 1
Char. 148: 0 → 1
Char. 152: 0 → 1
Char. 155: 0 → 1
Char. 158: 0 → 1
Char. 161: 0 → 1
Char. 174: 0 → 1
Char. 181: 0 → 1
Char. 203: 1 → 2
Char. 204: 0 → 2
Char. 247: 0 → 1
Char. 251: 0 → 1
Char. 253: 0 → 2

**Node 50:**
Char. 5: 0 → 1
Char. 20: 0 → 1
Char. 25: 1 → 0
Char. 33: 0 → 1
Char. 127: 0 → 1
Char. 135: 0 → 1
Char. 141: 0 → 1
Char. 159: 0 → 1
Char. 166: 0 → 1
Char. 179: 0 → 1
Char. 187: 0 → 1
Char. 188: 0 → 1
Char. 196: 0 → 1
Char. 201: 1 → 0

**Node 51:**
Char. 0: 0 → 1
Char. 29: 1 → 0
Char. 40: 2 → 0
Char. 43: 0 → 1
Char. 59: 0 → 1
Char. 60: 0 → 1
Char. 67: 0 → 1
Char. 72: 1 → 2
Char. 89: 0 → 1
Char. 111: 0 → 1
Char. 120: 0 → 1
Char. 163: 0 → 1
Char. 192: 0 → 1
Char. 222: 0 → 1
Char. 234: 0 → 1
Char. 237: 0 → 1
Char. 266: 0 → 1

**Node 52:**
Char. 29: 0 → 1
Char. 40: 0 → 2
Char. 74: 0 → 1
Char. 80: 0 → 1
Char. 84: 0 → 1
Char. 88: 0 → 1
Char. 132: 0 → 1
Char. 149: 0 → 1

Char. 156: 0 → 1
Char. 169: 0 → 1
Char. 201: 0 → 1
Char. 203: 0 → 1
Char. 235: 0 → 1

**Node 53:**
Char. 72: 0 → 1
Char. 79: 1 → 0
Char. 81: 0 → 1
Char. 93: 0 → 1
Char. 97: 0 → 1
Char. 104: 1 → 0
Char. 144: 0 → 1
Char. 173: 0 → 1
Char. 183: 0 → 1

**Node 55:**
Char. 62: 0 → 1
Char. 109: 0 → 1
Char. 129: 0 → 1
Char. 202: 0 → 1
Char. 265: 0 → 1

**Node 56:**
Char. 113: 0 → 1
Char. 114: 0 → 1

**Node 57:**
Char. 47: 0 → 1
Char. 79: 0 → 1
Char. 140: 0 → 2

**Node 58:**
Char. 5: 0 → 1
Char. 110: 0 → 1
Char. 137: 0 → 1
Char. 159: 0 → 1
Char. 163: 0 → 1
Char. 166: 0 → 1
Char. 192: 0 → 1

**Node 59:**
Char. 19: 0 → 1
Char. 92: 1 → 0

**Node 60:**
Char. 4: 0 → 1
Char. 15: 0 → 1
Char. 29: 0 → 1
Char. 213: 0 → 1
Char. 226: 0 → 2

Char. 228: 0 → 1
Char. 275: 0 → 1

**Node 61:**
Char. 58: 0 → 1
Char. 61: 0 → 1
Char. 66: 0 → 1
Char. 69: 0 → 1
Char. 107: 0 → 1
Char. 140: 0 → 1
Char. 147: 0 → 1
Char. 150: 0 → 1
Char. 167: 0 → 1
Char. 205: 0 → 1
Char. 208: 0 → 1
Char. 230: 0 → 1
Char. 239: 0 → 1

**Node 62:**
Char. 23: 0 → 1
Char. 70: 0 → 1
Char. 73: 0 → 1
Char. 117: 0 → 12
Char. 126: 0 → 1
Char. 131: 0 → 1
Char. 148: 0 → 1
Char. 182: 0 → 1
Char. 190: 0 → 1
Char. 267: 0 → 1
Char. 272: 2 → 1

**Node 63:**
Char. 73: 0 → 1
Char. 205: 0 → 1
Char. 273: 0 → 1
Char. 276: 0 → 1

**Node 64:**
Char. 18: 0 → 1
Char. 25: 1 → 0
Char. 37: 0 → 1
Char. 106: 0 → 1
Char. 107: 0 → 1
Char. 126: 0 → 1
Char. 150: 0 → 1

**Node 65:**
Char. 186: 0 → 1

**Node 66:**
Char. 194: 0 → 1
Char. 201: 1 → 0

Char. 211: 0 → 1
Char. 226: 0 → 1

**Node 67:**
Char. 38: 1 → 2
Char. 39: 0 → 1
Char. 49: 0 → 1
Char. 66: 0 → 1
Char. 88: 1 → 0
Char. 112: 0 → 1
Char. 148: 0 → 1

**Node 69:**
Char. 133: 0 → 1

**Node 70:**
Char. 100: 0 → 1
Char. 113: 0 → 1

**Node 71:**
Char. 70: 0 → 1
Char. 118: 0 → 1
Char. 125: 0 → 1

**Node 72:**
Char. 110: 1 → 0

**Node 73:**
Char. 58: 0 → 1
Char. 59: 0 → 1
Char. 60: 0 → 1
Char. 72: 1 → 2
Char. 104: 0 → 1
Char. 105: 0 → 1
Char. 106: 0 → 2
Char. 107: 0 → 1
Char. 109: 0 → 1
Char. 146: 1 → 0

**Node 74:**
Char. 23: 0 → 1
Char. 33: 1 → 0
Char. 38: 2 → 1
Char. 39: 1 → 0
Char. 42: 0 → 1
Char. 43: 0 → 1
Char. 46: 1 → 0
Char. 49: 1 → 0
Char. 71: 1 → 0
Char. 83: 0 → 2
Char. 101: 0 → 1
Char. 103: 0 → 1

Char. 106: 0 → 1
Char. 161: 0 → 1
Char. 163: 1 → 0
Char. 172: 0 → 2
Char. 174: 0 → 1
Char. 188: 0 → 1
Char. 203: 1 → 2
Char. 212: 0 → 1
Char. 216: 1 → 0
Char. 236: 0 → 1
Char. 238: 0 → 2
Char. 239: 1 → 0
Char. 241: 0 → 1
Char. 242: 0 → 1
Char. 273: 0 → 1
Char. 274: 0 → 1
Char. 275: 1 → 0

**Node 75:**
Char. 52: 0 → 1
Char. 75: 0 → 1
Char. 84: 1 → 0
Char. 121: 0 → 1
Char. 230: 0 → 1

**Node 76:**
Char. 29: 1 → 0
Char. 44: 1 → 0
Char. 59: 0 → 1
Char. 60: 0 → 1
Char. 66: 0 → 1
Char. 67: 0 → 1
Char. 72: 1 → 2
Char. 78: 1 → 0
Char. 84: 1 → 0
Char. 88: 1 → 0
Char. 93: 1 → 0
Char. 111: 0 → 1
Char. 123: 1 → 0
Char. 129: 0 → 1
Char. 154: 1 → 0
Char. 169: 1 → 0
Char. 170: 0 → 1
Char. 197: 0 → 1

**Node 77:**
Char. 107: 0 → 1

**Node 78:**
Char. 2: 0 → 1
Char. 6: 0 → 1
Char. 186: 0 → 1

Char. 198: 0 → 1
Char. 200: 0 → 1
Char. 220: 0 → 1
Char. 239: 1 → 0
Char. 267: 1 → 0

**Node 79:**
Char. 98: 1 → 0
Char. 113: 0 → 1
Char. 181: 0 → 1
Char. 206: 0 → 2

**Node 80:**
Char. 35: 0 → 1
Char. 102: 0 → 1
Char. 103: 0 → 1
Char. 184: 0 → 1
Char. 210: 0 → 1
Char. 223: 0 → 1
Char. 229: 0 → 1

**Node 81:**
Char. 17: 0 → 1
Char. 42: 0 → 1
Char. 251: 0 → 1
Char. 264: 0 → 1

**Node 82:**
Char. 41: 0 → 1
Char. 61: 1 → 2
Char. 112: 1 → 0
Char. 138: 0 → 1
Char. 146: 1 → 0
Char. 155: 0 → 1
Char. 182: 1 → 0
Char. 192: 1 → 2
Char. 226: 0 → 1
Char. 227: 0 → 1
Char. 233: 0 → 1
Char. 267: 1 → 2

**Node 83:**
Char. 61: 1 → 3
Char. 90: 0 → 1
Char. 91: 0 → 1
Char. 109: 1 → 0
Char. 155: 0 → 1
Char. 209: 1 → 0

**ANALYSIS 68**

**(NO *CANDELARIA BARBOURI*, *PAPPOCHELYS ROSINAE*, AND *ODONTOCHELYS SEMITESTACEA*, IMPLIED WEIGHTING, K = 2.5)**

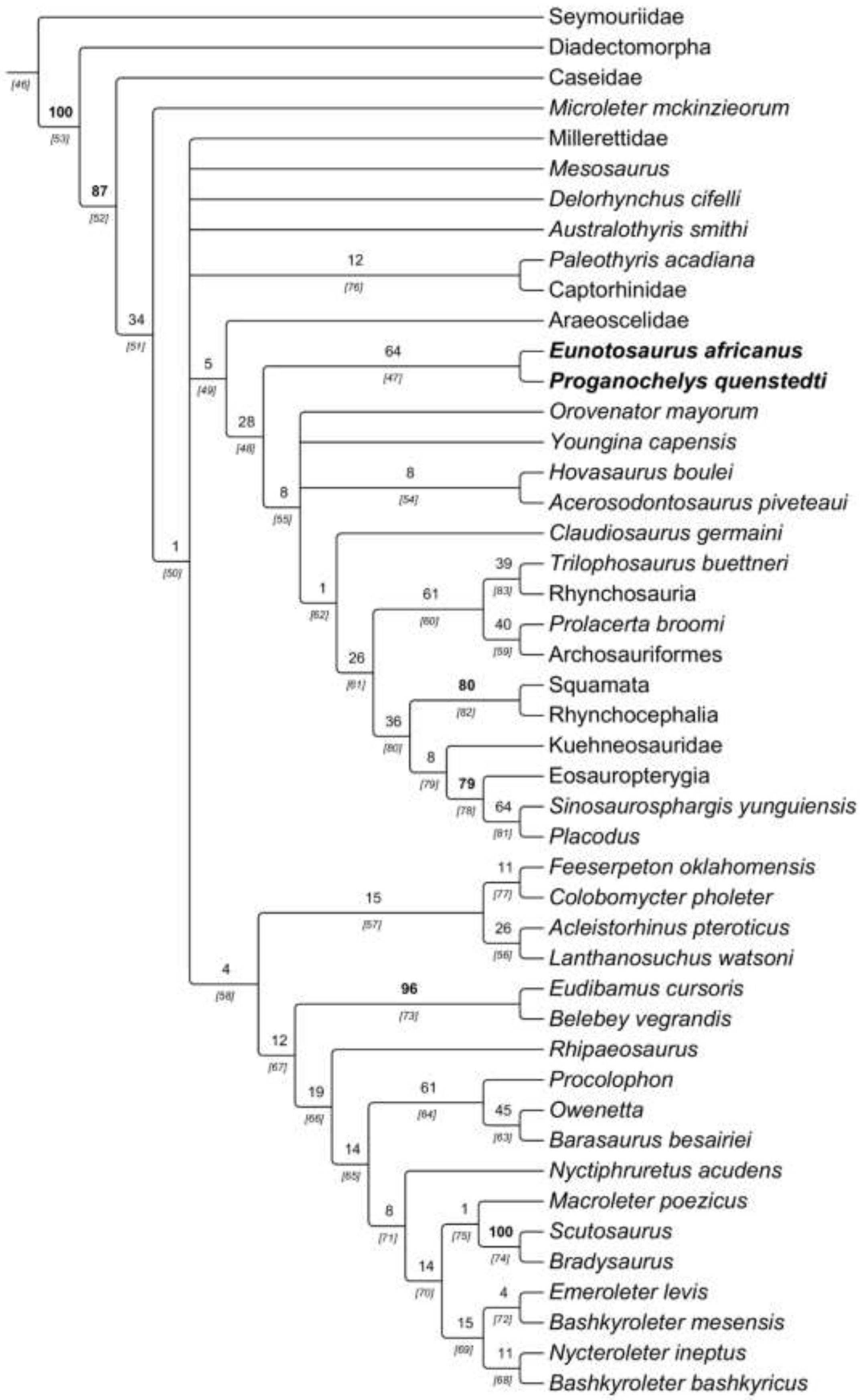

***Proganochelys quenstedti*:**
Char. 8: 0 → 1
Char. 9: 0 → 1
Char. 11: 0 → 1
Char. 26: 0 → 1
Char. 28: 0 → 1
Char. 39: 0 → 1
Char. 43: 1 → 0
Char. 46: 1 → 0
Char. 53: 0 → 1
Char. 63: 0 → 1
Char. 65: 0 → 1
Char. 69: 0 → 1
Char. 79: 0 → 2
Char. 88: 1 → 0
Char. 89: 1 → 0
Char. 93: 1 → 0
Char. 106: 0 → 1
Char. 108: 1 → 0
Char. 110: 0 → 1
Char. 115: 0 → 1
Char. 118: 0 → 1
Char. 121: 0 → 1
Char. 126: 0 → 1
Char. 128: 0 → 1
Char. 131: 0 → 1
Char. 138: 0 → 1
Char. 142: 1 → 0
Char. 160: 0 → 1
Char. 175: 0 → 1
Char. 176: 0 → 1
Char. 184: 0 → 1
Char. 195: 0 → 2
Char. 198: 0 → 1
Char. 200: 0 → 1
Char. 205: 0 → 1
Char. 210: 0 → 1
Char. 218: 0 → 1
Char. 223: 0 → 1
Char. 230: 0 → 1
Char. 232: 0 → 1
Char. 235: 1 → 0
Char. 238: 0 → 2
Char. 241: 0 → 1
Char. 246: 0 → 2
Char. 254: 0 → 1
Char. 255: 0 → 1
Char. 256: 0 → 1
Char. 258: 0 → 1
Char. 259: 0 → 1
Char. 262: 0 → 1
Char. 267: 0 → 1
Char. 268: 0 → 1
Char. 269: 0 → 1
Char. 270: 0 → 1
Char. 271: 0 → 1
Char. 272: 2 → 1

***Eunotosaurus africanus*:**
Char. 19: 0 → 1
Char. 55: 1 → 0
Char. 59: 1 → 0
Char. 60: 1 → 0
Char. 67: 1 → 0
Char. 72: 2 → 1
Char. 76: 0 → 1
Char. 97: 1 → 0
Char. 103: 0 → 1
Char. 112: 1 → 0
Char. 124: 0 → 1
Char. 153: 1 → 0
Char. 192: 1 → 0
Char. 211: 0 → 1
Char. 222: 1 → 0
Char. 237: 1 → 0
Char. 249: 0 → 2
Char. 252: 0 → 1
Char. 263: 0 → 1
Char. 273: 0 → 1
Char. 274: 0 → 1
Char. 277: 0 → 1

***Acerosodontosaurus piveteaui*:**
Char. 81: 1 → 0
Char. 127: 1 → 0
Char. 208: 0 → 1

***Acleistorhinus pteroticus*:**
Char. 21: 0 → 1
Char. 146: 1 → 0

**Araeoscelidae:**
Char. 23: 0 → 1
Char. 28: 0 → 1
Char. 106: 0 → 1
Char. 117: 0 → 2
Char. 169: 1 → 0
Char. 170: 0 → 1
Char. 197: 0 → 1
Char. 239: 0 → 1

**Archosauriformes:**
Char. 32: 0 → 1
Char. 94: 1 → 0
Char. 112: 1 → 0
Char. 152: 0 → 1
Char. 154: 0 → 2
Char. 166: 1 → 0
Char. 171: 0 → 1
Char. 185: 0 → 1
Char. 204: 0 → 1
Char. 218: 0 → 3
Char. 242: 0 → 1

***Australothyris smithi*:**
Char. 23: 0 → 1
Char. 34: 0 → 1
Char. 55: 1 → 0
Char. 71: 1 → 0
Char. 79: 0 → 1
Char. 85: 1 → 0
Char. 98: 1 → 0
Char. 100: 0 → 1
Char. 103: 0 → 1
Char. 110: 0 → 1
Char. 112: 0 → 1
Char. 123: 1 → 0
Char. 129: 0 → 1
Char. 131: 0 → 1
Char. 132: 1 → 0
Char. 144: 1 → 0
Char. 147: 0 → 1
Char. 149: 1 → 0
Char. 150: 0 → 1
Char. 166: 0 → 1

***Barasaurus besairiei*:**
Char. 33: 1 → 0

***Bashkyroleter bashkyricus*:**
Char. 275: 1 → 0

***Bashkyroleter mesensis*:**
Char. 12: 0 → 1
Char. 66: 1 → 0
Char. 169: 1 → 0

***Belebey vegrandis*:**
Char. 154: 1 → 0

***Bradysaurus* spp.:**
Char. 19: 0 → 1
Char. 25: 1 → 0
Char. 73: 0 → 1
Char. 79: 0 → 1
Char. 135: 1 → 0
Char. 249: 0 → 1

**Captorhinidae:**
Char. 3: 0 → 1
Char. 23: 0 → 1
Char. 25: 1 → 0
Char. 26: 0 → 1
Char. 73: 0 → 1
Char. 75: 0 → 1
Char. 108: 1 → 0
Char. 183: 1 → 0
Char. 201: 1 → 0
Char. 203: 1 → 2
Char. 216: 1 → 0

**Caseidae:**
Char. 38: 1 → 0
Char. 46: 1 → 0
Char. 50: 1 → 0
Char. 170: 0 → 1
Char. 194: 0 → 1
Char. 273: 0 → 1
Char. 274: 0 → 1
Char. 278: 3 → 2

***Claudiosaurus germaini*:**
Char. 34: 0 → 1
Char. 36: 0 → 1
Char. 43: 1 → 0
Char. 64: 0 → 1
Char. 105: 0 → 1
Char. 127: 1 → 0
Char. 130: 0 → 1
Char. 141: 1 → 0
Char. 144: 1 → 0
Char. 166: 1 → 0
Char. 187: 1 → 0
Char. 199: 0 → 1
Char. 203: 1 → 2
Char. 204: 0 → 1
Char. 222: 1 → 0

***Colobomycter pholeter*:**
Char. 21: 0 → 1
Char. 25: 1 → 0
Char. 84: 1 → 0
Char. 154: 1 → 0
Char. 167: 0 → 1
Char. 267: 0 → 1

***Delorhynchus cifelli*:**
Char. 5: 0 → 1
Char. 18: 0 → 1
Char. 20: 0 → 1
Char. 21: 0 → 1
Char. 26: 0 → 1
Char. 28: 0 → 1
Char. 33: 0 → 2
Char. 39: 0 → 1
Char. 52: 0 → 1
Char. 100: 0 → 1
Char. 117: 0 → 1
Char. 131: 0 → 1
Char. 147: 0 → 1
Char. 156: 1 → 0
Char. 159: 0 → 1
Char. 163: 0 → 1
Char. 166: 0 → 1
Char. 167: 0 → 1
Char. 189: 0 → 1
Char. 191: 0 → 1
Char. 192: 0 → 1
Char. 204: 0 → 2
Char. 267: 0 → 1

**Diadectomorpha:**
Char. 0: 0 → 1
Char. 64: 0 → 1
Char. 70: 0 → 1
Char. 122: 0 → 1
Char. 123: 1 → 0
Char. 146: 1 → 0
Char. 275: 1 → 0
Char. 278: 3 → 0

***Emeroleter levis*:**
Char. 51: 1 → 0

**Eosauropterygia:**
Char. 166: 1 → 0
Char. 174: 0 → 1
Char. 178: 0 → 1
Char. 194: 0 → 2
Char. 272: 1 → 0

***Eudibamus cursoris*:**
Char. 154: 1 → 2
Char. 163: 1 → 0

***Feeserpeton oklahomensis*:**
Char. 157: 0 → 1

***Hovasaurus boulei*:**
Char. 41: 0 → 1
Char. 77: 0 → 2
Char. 79: 0 → 1
Char. 93: 1 → 0
Char. 204: 0 → 2

**Kuehneosauridae:**
Char. 7: 0 → 1
Char. 27: 0 → 1
Char. 43: 1 → 0
Char. 79: 0 → 2
Char. 107: 1 → 0
Char. 140: 1 → 0
Char. 147: 1 → 0
Char. 148: 1 → 0
Char. 245: 0 → 1
Char. 272: 1 → 2
Char. 278: 0 → 3

***Lanthanosuchus watsoni*:**
Char. 25: 1 → 0
Char. 86: 0 → 1
Char. 98: 1 → 0
Char. 138: 0 → 1
Char. 144: 1 → 0
Char. 154: 1 → 2

***Macroleter poezicus*:**
Char. 9: 0 → 1
Char. 26: 0 → 1
Char. 66: 1 → 2
Char. 126: 0 → 1
Char. 134: 0 → 2
Char. 140: 0 → 1
Char. 146: 1 → 0
Char. 169: 1 → 0

***Mesosaurus* spp.:**
Char. 0: 0 → 1
Char. 2: 0 → 1
Char. 6: 0 → 1
Char. 8: 0 → 1
Char. 9: 0 → 1
Char. 13: 0 → 1
Char. 19: 0 → 1
Char. 23: 0 → 1
Char. 26: 0 → 1
Char. 29: 1 → 0
Char. 33: 0 → 1
Char. 38: 1 → 0
Char. 41: 0 → 1
Char. 50: 1 → 0
Char. 67: 0 → 1
Char. 76: 0 → 1
Char. 84: 1 → 0
Char. 85: 1 → 0
Char. 94: 0 → 1
Char. 107: 0 → 1
Char. 109: 0 → 1
Char. 115: 0 → 1
Char. 146: 1 → 0
Char. 148: 0 → 1
Char. 149: 1 → 0

Char. 164: 0 → 1
Char. 167: 0 → 1
Char. 176: 0 → 1
Char. 183: 1 → 0
Char. 184: 0 → 1
Char. 199: 0 → 1
Char. 202: 0 → 1
Char. 204: 0 → 2
Char. 206: 0 → 1
Char. 207: 0 → 1
Char. 209: 0 → 1
Char. 217: 0 → 1
Char. 219: 0 → 1
Char. 220: 0 → 1
Char. 231: 0 → 1
Char. 260: 2 → 0
Char. 272: 2 → 0
Char. 278: 3 → 0

***Microleter mckinzieorum*:**
Char. 0: 0 → 1
Char. 18: 0 → 1
Char. 39: 0 → 1
Char. 70: 0 → 1
Char. 76: 0 → 1
Char. 94: 0 → 1
Char. 106: 0 → 1
Char. 110: 0 → 1
Char. 159: 0 → 1
Char. 278: 3 → 1

**Millerettidae:**
Char. 25: 1 → 0
Char. 44: 1 → 0
Char. 48: 1 → 0
Char. 56: 0 → 1
Char. 66: 0 → 2
Char. 78: 1 → 0
Char. 80: 1 → 0
Char. 84: 1 → 2
Char. 88: 1 → 0
Char. 96: 1 → 0
Char. 117: 0 → 1
Char. 124: 0 → 1
Char. 127: 0 → 1
Char. 135: 0 → 1
Char. 145: 0 → 1
Char. 202: 0 → 1
Char. 211: 0 → 1
Char. 230: 0 → 1
Char. 234: 0 → 1
Char. 248: 0 → 1
Char. 252: 0 → 1
Char. 253: 0 → 1

***Nycteroleter ineptus*:**
Char. 66: 1 → 0

***Nyctiphruretus acudens*:**
Char. 21: 0 → 1
Char. 41: 0 → 1
Char. 48: 1 → 0
Char. 66: 1 → 2
Char. 81: 1 → 0
Char. 84: 1 → 2
Char. 94: 0 → 1
Char. 110: 1 → 0
Char. 166: 1 → 0
Char. 215: 1 → 0
Char. 224: 0 → 1
Char. 226: 1 → 0
Char. 266: 0 → 1
Char. 272: 2 → 1
Char. 276: 0 → 1

***Orovenator mayorum*:**
Char. 8: 0 → 1
Char. 33: 1 → 0
Char. 36: 0 → 1
Char. 43: 1 → 0
Char. 48: 1 → 0
Char. 50: 0 → 1
Char. 135: 1 → 0
Char. 159: 1 → 0
Char. 160: 0 → 1
Char. 165: 0 → 1

***Owenetta* spp.:**
Char. 142: 1 → 0
Char. 169: 1 → 0

***Paleothyris acadiana*:**
Char. 38: 1 → 0
Char. 50: 1 → 0
Char. 66: 1 → 2
Char. 102: 0 → 1
Char. 146: 1 → 0
Char. 237: 0 → 1
Char. 239: 0 → 1

***Placodus* spp.:**
Char. 0: 1 → 2
Char. 9: 0 → 1
Char. 12: 0 → 1
Char. 13: 0 → 1
Char. 19: 0 → 1
Char. 31: 0 → 1
Char. 46: 1 → 0
Char. 57: 0 → 1
Char. 78: 1 → 0
Char. 93: 1 → 0
Char. 102: 1 → 2
Char. 109: 1 → 0
Char. 140: 1 → 0
Char. 155: 0 → 1
Char. 163: 1 → 0
Char. 164: 0 → 1

***Procolophon* spp.:**
Char. 41: 0 → 1
Char. 69: 0 → 1
Char. 79: 0 → 1
Char. 86: 0 → 1
Char. 88: 0 → 1
Char. 101: 0 → 1
Char. 141: 0 → 1
Char. 149: 1 → 0
Char. 203: 1 → 2
Char. 204: 0 → 1
Char. 230: 0 → 1
Char. 237: 0 → 1
Char. 238: 0 → 1
Char. 239: 1 → 0
Char. 272: 2 → 1
Char. 278: 3 → 0

***Prolacerta broomi*:**
Char. 58: 1 → 0
Char. 66: 1 → 0
Char. 67: 1 → 0
Char. 80: 1 → 0
Char. 139: 1 → 0
Char. 147: 1 → 0
Char. 192: 1 → 0
Char. 203: 1 → 2
Char. 206: 0 → 12

***Rhipaeosaurus* spp.:**
Char. 110: 1 → 0
Char. 172: 0 → 1
Char. 277: 0 → 1

**Rhynchocephalia:**
Char. 0: 1 → 2
Char. 23: 1 → 0
Char. 77: 0 → 1
Char. 88: 1 → 0
Char. 94: 1 → 0
Char. 117: 12 → 0
Char. 139: 1 → 0
Char. 167: 1 → 0
Char. 205: 1 → 0

**Rhynchosauria:**
Char. 0: 1 → 0
Char. 7: 0 → 1
Char. 9: 0 → 1
Char. 26: 0 → 1
Char. 44: 1 → 0
Char. 68: 0 → 1
Char. 99: 1 → 0
Char. 150: 1 → 0
Char. 160: 0 → 1
Char. 161: 0 → 1
Char. 171: 0 → 2
Char. 182: 1 → 0
Char. 223: 0 → 1
Char. 241: 0 → 1

***Scutosaurus* spp.:**
Char. 175: 0 → 1
Char. 190: 0 → 1
Char. 218: 0 → 1
Char. 243: 0 → 2
Char. 244: 0 → 1
Char. 251: 0 → 1

***Sinosaurosphargis yunguiensis*:**
Char. 8: 0 → 1
Char. 30: 0 → 1
Char. 53: 0 → 1
Char. 89: 1 → 0
Char. 127: 1 → 0
Char. 150: 1 → 0
Char. 154: 0 → 2
Char. 167: 1 → 0
Char. 253: 0 → 1
Char. 255: 0 → 1

**Squamata:**
Char. 45: 0 → 1
Char. 79: 0 → 2
Char. 80: 1 → 0
Char. 92: 1 → 0
Char. 109: 1 → 0
Char. 160: 0 → 1
Char. 200: 0 → 1
Char. 245: 0 → 1

***Trilophosaurus buettneri*:**
Char. 5: 1 → 0
Char. 11: 0 → 1
Char. 55: 1 → 0
Char. 93: 1 → 0
Char. 104: 0 → 1
Char. 113: 0 → 1
Char. 122: 0 → 1
Char. 136: 1 → 0
Char. 144: 1 → 0
Char. 154: 0 → 1
Char. 157: 0 → 1
Char. 159: 1 → 0
Char. 177: 0 → 12
Char. 194: 0 → 1
Char. 203: 1 → 2
Char. 207: 1 → 0
Char. 208: 1 → 0
Char. 272: 1 → 0

***Youngina capensis*:**
Char. 5: 1 → 0
Char. 25: 0 → 1
Char. 44: 1 → 0
Char. 75: 0 → 1
Char. 134: 0 → 1
Char. 163: 1 → 0
Char. 170: 0 → 1
Char. 211: 0 → 1
Char. 239: 0 → 1
Char. 267: 0 → 1

**Node 47:**
Char. 0: 1 → 2
Char. 15: 0 → 1
Char. 33: 1 → 2
Char. 42: 0 → 1
Char. 44: 1 → 0
Char. 48: 1 → 0
Char. 64: 0 → 1
Char. 73: 0 → 1
Char. 81: 1 → 0
Char. 84: 01 → 2
Char. 130: 0 → 1
Char. 134: 0 → 2
Char. 147: 0 → 1
Char. 148: 0 → 1
Char. 152: 0 → 1
Char. 155: 0 → 1
Char. 158: 0 → 1
Char. 161: 0 → 1
Char. 174: 0 → 1
Char. 181: 0 → 1
Char. 203: 1 → 2
Char. 204: 0 → 2
Char. 247: 0 → 1
Char. 248: 0 → 1
Char. 250: 0 → 1
Char. 251: 0 → 1
Char. 253: 0 → 2

**Node 48:**
Char. 5: 0 → 1
Char. 20: 0 → 1
Char. 25: 1 → 0
Char. 33: 0 → 1
Char. 127: 0 → 1
Char. 135: 0 → 1
Char. 141: 0 → 1
Char. 159: 0 → 1
Char. 166: 0 → 1
Char. 179: 0 → 1
Char. 187: 0 → 1
Char. 188: 0 → 1
Char. 196: 0 → 1
Char. 201: 1 → 0

**Node 49:**
Char. 0: 0 → 1
Char. 29: 1 → 0
Char. 40: 2 → 0
Char. 43: 0 → 1
Char. 59: 0 → 1
Char. 60: 0 → 1
Char. 67: 0 → 1
Char. 72: 1 → 2
Char. 89: 0 → 1
Char. 112: 0 → 1
Char. 120: 0 → 1
Char. 163: 0 → 1
Char. 192: 0 → 1
Char. 222: 0 → 1
Char. 234: 0 → 1
Char. 237: 0 → 1
Char. 266: 0 → 1

**Node 50:**
Char. 48: 0 → 1
Char. 132: 0 → 1
Char. 276: 1 → 0

**Node 51:**
Char. 29: 0 → 1
Char. 84: 0 → 1
Char. 88: 0 → 1
Char. 169: 0 → 1

**Node 52:**
Char. 72: 0 → 1
Char. 81: 0 → 1
Char. 93: 0 → 1
Char. 97: 0 → 1
Char. 104: 1 → 0

Char. 144: 0 → 1
Char. 173: 0 → 1
Char. 183: 0 → 1

**Node 54:**
Char. 78: 1 → 0
Char. 206: 0 → 2

**Node 55:**
Char. 62: 0 → 1
Char. 129: 0 → 1
Char. 202: 0 → 1
Char. 207: 0 → 1
Char. 209: 0 → 1
Char. 265: 0 → 1

**Node 56:**
Char. 113: 0 → 1
Char. 114: 0 → 1

**Node 57:**
Char. 47: 0 → 1
Char. 79: 0 → 1
Char. 140: 0 → 2

**Node 58:**
Char. 5: 0 → 1
Char. 110: 0 → 1
Char. 137: 0 → 1
Char. 159: 0 → 1
Char. 163: 0 → 1
Char. 166: 0 → 1
Char. 192: 0 → 1

**Node 59:**
Char. 19: 0 → 1
Char. 92: 1 → 0

**Node 60:**
Char. 4: 0 → 1
Char. 15: 0 → 1
Char. 29: 0 → 1
Char. 213: 0 → 1
Char. 226: 0 → 2
Char. 228: 0 → 1
Char. 275: 0 → 1

**Node 61:**
Char. 58: 0 → 1
Char. 61: 0 → 1
Char. 66: 0 → 1
Char. 69: 0 → 1
Char. 107: 0 → 1
Char. 140: 0 → 1
Char. 147: 0 → 1
Char. 150: 0 → 1
Char. 167: 0 → 1
Char. 205: 0 → 1
Char. 208: 0 → 1
Char. 230: 0 → 1
Char. 239: 0 → 1

**Node 62:**
Char. 23: 0 → 1
Char. 70: 0 → 1
Char. 73: 0 → 1
Char. 117: 0 → 12
Char. 126: 0 → 1
Char. 131: 0 → 1
Char. 148: 0 → 1
Char. 182: 0 → 1
Char. 190: 0 → 1
Char. 267: 0 → 1
Char. 272: 2 → 1

**Node 63:**
Char. 73: 0 → 1
Char. 205: 0 → 1
Char. 273: 0 → 1
Char. 276: 0 → 1

**Node 64:**
Char. 18: 0 → 1
Char. 25: 1 → 0
Char. 37: 0 → 1
Char. 106: 0 → 1
Char. 107: 0 → 1
Char. 126: 0 → 1
Char. 150: 0 → 1

**Node 65:**
Char. 186: 0 → 1

**Node 66:**
Char. 194: 0 → 1
Char. 201: 1 → 0
Char. 211: 0 → 1
Char. 226: 0 → 1

**Node 67:**
Char. 38: 1 → 2
Char. 39: 0 → 1
Char. 49: 0 → 1
Char. 66: 0 → 1
Char. 88: 1 → 0
Char. 112: 0 → 1
Char. 148: 0 → 1

**Node 69:**
Char. 133: 0 → 1

**Node 70:**
Char. 100: 0 → 1
Char. 113: 0 → 1

**Node 71:**
Char. 70: 0 → 1
Char. 118: 0 → 1
Char. 125: 0 → 1

**Node 72:**
Char. 110: 1 → 0

**Node 73:**
Char. 58: 0 → 1
Char. 59: 0 → 1
Char. 60: 0 → 1
Char. 72: 1 → 2
Char. 104: 0 → 1
Char. 105: 0 → 1
Char. 106: 0 → 2
Char. 107: 0 → 1
Char. 109: 0 → 1
Char. 146: 1 → 0

**Node 74:**
Char. 23: 0 → 1
Char. 33: 1 → 0
Char. 38: 2 → 1
Char. 39: 1 → 0
Char. 42: 0 → 1
Char. 43: 0 → 1
Char. 46: 1 → 0
Char. 48: 1 → 0
Char. 49: 1 → 0
Char. 71: 1 → 0
Char. 83: 0 → 2
Char. 101: 0 → 1
Char. 103: 0 → 1
Char. 106: 0 → 1
Char. 161: 0 → 1
Char. 163: 1 → 0
Char. 172: 0 → 2
Char. 174: 0 → 1
Char. 188: 0 → 1
Char. 203: 1 → 2
Char. 212: 0 → 1
Char. 216: 1 → 0
Char. 236: 0 → 1
Char. 238: 0 → 2
Char. 239: 1 → 0
Char. 241: 0 → 1
Char. 242: 0 → 1
Char. 273: 0 → 1
Char. 274: 0 → 1
Char. 275: 1 → 0

**Node 75:**
Char. 52: 0 → 1
Char. 75: 0 → 1
Char. 84: 1 → 0
Char. 121: 0 → 1
Char. 230: 0 → 1

**Node 76:**
Char. 29: 1 → 0
Char. 44: 1 → 0
Char. 48: 1 → 0
Char. 59: 0 → 1
Char. 60: 0 → 1
Char. 66: 0 → 1
Char. 67: 0 → 1
Char. 72: 1 → 2
Char. 78: 1 → 0
Char. 84: 1 → 0
Char. 88: 1 → 0
Char. 93: 1 → 0
Char. 123: 1 → 0
Char. 129: 0 → 1
Char. 154: 1 → 0
Char. 169: 1 → 0
Char. 170: 0 → 1
Char. 197: 0 → 1

**Node 77:**
Char. 107: 0 → 1

**Node 78:**
Char. 2: 0 → 1
Char. 6: 0 → 1
Char. 186: 0 → 1
Char. 198: 0 → 1
Char. 200: 0 → 1
Char. 220: 0 → 1
Char. 239: 1 → 0
Char. 267: 1 → 0

**Node 79:**
Char. 98: 1 → 0
Char. 113: 0 → 1
Char. 181: 0 → 1
Char. 206: 0 → 2

**Node 80:**
Char. 35: 0 → 1
Char. 102: 0 → 1
Char. 103: 0 → 1
Char. 184: 0 → 1
Char. 210: 0 → 1
Char. 223: 0 → 1
Char. 229: 0 → 1

**Node 81:**
Char. 17: 0 → 1
Char. 42: 0 → 1
Char. 251: 0 → 1
Char. 264: 0 → 1

**Node 82:**
Char. 41: 0 → 1
Char. 61: 1 → 2
Char. 112: 1 → 0
Char. 138: 0 → 1
Char. 146: 1 → 0
Char. 155: 0 → 1
Char. 182: 1 → 0
Char. 192: 1 → 2
Char. 226: 0 → 1
Char. 227: 0 → 1
Char. 233: 0 → 1
Char. 267: 1 → 2

**Node 83:**
Char. 61: 1 → 3
Char. 90: 0 → 1
Char. 91: 0 → 1
Char. 109: 1 → 0
Char. 155: 0 → 1
Char. 209: 1 → 0

**ANALYSIS 69**

**(NO *PROGANOCHELYS QUENSTEDTI*, *CANDELARIA BARBOURI*, AND *ODONTOCHELYS SEMITESTACEA*, IMPLIED WEIGHTING, K = 2.75)**

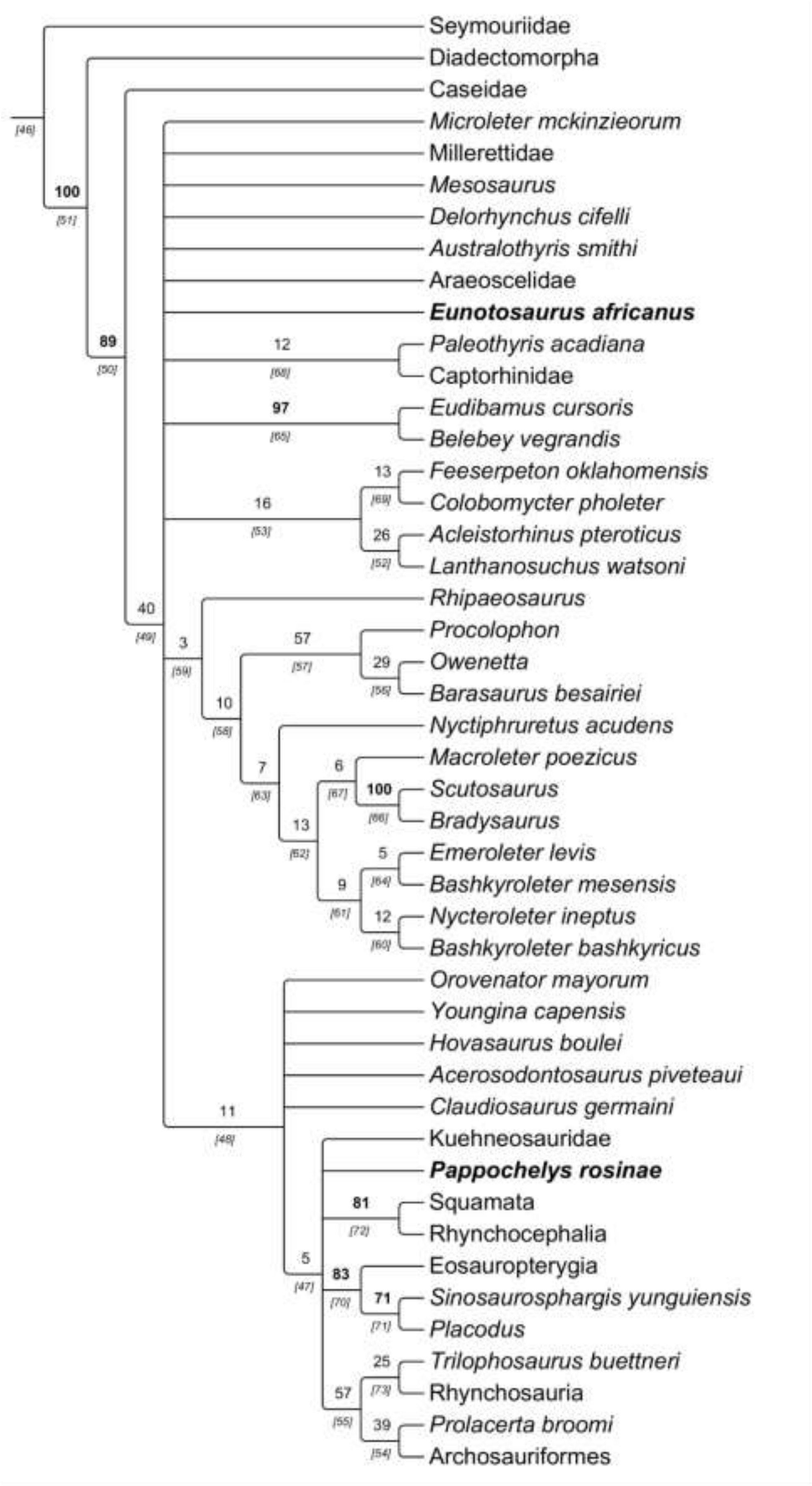

***Pappochelys rosinae*:**
Char. 0: 1 → 0
Char. 1: 0 → 1
Char. 5: 1 → 0
Char. 12: 0 → 1
Char. 49: 0 → 1
Char. 55: 1 → 0
Char. 61: 1 → 0
Char. 62: 1 → 0
Char. 65: 0 → 1
Char. 67: 1 → 0
Char. 78: 1 → 0
Char. 91: 0 → 1
Char. 169: 1 → 0
Char. 176: 1 → 0
Char. 193: 1 → 0
Char. 194: 0 → 2
Char. 201: 1 → 0
Char. 214: 1 → 0
Char. 219: 1 → 0
Char. 220: 0 → 1
Char. 241: 0 → 1
Char. 246: 0 → 1
Char. 247: 0 → 1
Char. 252: 0 → 1
Char. 254: 0 → 1
Char. 255: 0 → 1
Char. 256: 0 → 1
Char. 260: 1 → 0
Char. 268: 0 → 1
Char. 269: 0 → 1

***Eunotosaurus africanus*:**
Char. 0: 0 → 2
Char. 15: 0 → 1
Char. 19: 0 → 1
Char. 20: 0 → 1
Char. 21: 0 → 1
Char. 24: 0 → 1
Char. 25: 1 → 0
Char. 29: 1 → 0
Char. 33: 0 → 2
Char. 40: 2 → 0
Char. 42: 0 → 1
Char. 43: 0 → 1
Char. 44: 1 → 0
Char. 55: 1 → 0
Char. 64: 0 → 1
Char. 73: 0 → 1
Char. 76: 0 → 1
Char. 81: 1 → 0
Char. 84: 1 → 2
Char. 89: 0 → 1
Char. 94: 0 → 1
Char. 97: 1 → 0
Char. 102: 0 → 1
Char. 103: 0 → 1
Char. 111: 0 → 1
Char. 120: 0 → 1
Char. 124: 0 → 1
Char. 127: 0 → 1
Char. 130: 0 → 1
Char. 134: 0 → 2
Char. 135: 0 → 1
Char. 136: 0 → 1
Char. 141: 0 → 1
Char. 145: 0 → 1
Char. 147: 0 → 1
Char. 148: 0 → 1
Char. 151: 0 → 1
Char. 152: 0 → 1
Char. 155: 0 → 1
Char. 158: 0 → 1
Char. 161: 0 → 1
Char. 174: 0 → 1
Char. 181: 0 → 1
Char. 188: 0 → 1
Char. 194: 0 → 1
Char. 196: 0 → 1
Char. 201: 1 → 0
Char. 203: 1 → 2
Char. 204: 0 → 2
Char. 211: 0 → 1
Char. 217: 0 → 1
Char. 219: 0 → 1
Char. 220: 0 → 1
Char. 231: 0 → 1
Char. 247: 0 → 1
Char. 248: 0 → 1
Char. 249: 0 → 2
Char. 250: 0 → 1
Char. 251: 0 → 1
Char. 252: 0 → 1
Char. 253: 0 → 2
Char. 263: 0 → 1
Char. 273: 0 → 1
Char. 274: 0 → 1
Char. 276: 0 → 1
Char. 277: 0 → 1

***Acerosodontosaurus piveteaui*:**
Char. 78: 1 → 0
Char. 81: 1 → 0
Char. 128: 0 → 1
Char. 155: 0 → 1
Char. 208: 0 → 1
Char. 278: 0 → 1

***Acleistorhinus pteroticus*:**
Char. 21: 0 → 1
Char. 146: 1 → 0

**Araeoscelidae:**
Char. 0: 0 → 1
Char. 5: 1 → 0
Char. 23: 0 → 1
Char. 27: 0 → 1
Char. 28: 0 → 1
Char. 29: 1 → 0
Char. 38: 1 → 0
Char. 40: 2 → 0
Char. 43: 0 → 1
Char. 50: 1 → 0
Char. 57: 1 → 0
Char. 59: 0 → 1
Char. 60: 0 → 1
Char. 67: 0 → 1
Char. 72: 1 → 2
Char. 84: 1 → 0
Char. 89: 0 → 1
Char. 92: 1 → 0
Char. 106: 0 → 1
Char. 111: 0 → 1
Char. 112: 0 → 1
Char. 116: 1 → 0
Char. 117: 01 → 2
Char. 120: 0 → 1
Char. 154: 1 → 0
Char. 159: 1 → 0
Char. 166: 1 → 0
Char. 169: 1 → 0
Char. 170: 0 → 1
Char. 193: 0 → 1
Char. 197: 0 → 1
Char. 221: 1 → 0
Char. 222: 0 → 1
Char. 224: 0 → 1
Char. 237: 0 → 1
Char. 239: 0 → 1
Char. 266: 0 → 1
Char. 278: 3 → 1

**Archosauriformes:**
Char. 32: 0 → 1
Char. 94: 1 → 0
Char. 112: 1 → 0
Char. 152: 0 → 1
Char. 154: 0 → 2
Char. 166: 1 → 0
Char. 171: 0 → 1
Char. 185: 0 → 1
Char. 204: 0 → 1
Char. 218: 0 → 3
Char. 224: 0 → 1
Char. 242: 0 → 1

***Australothyris smithi*:**
Char. 23: 0 → 1
Char. 24: 0 → 1
Char. 34: 0 → 1
Char. 55: 1 → 0
Char. 57: 1 → 0
Char. 71: 1 → 0
Char. 79: 0 → 1
Char. 83: 0 → 1
Char. 85: 1 → 0
Char. 98: 1 → 0
Char. 100: 0 → 1
Char. 103: 0 → 1
Char. 110: 0 → 1
Char. 112: 0 → 1
Char. 123: 1 → 0
Char. 129: 0 → 1
Char. 131: 0 → 1
Char. 132: 1 → 0
Char. 144: 1 → 0
Char. 147: 0 → 1
Char. 149: 1 → 0
Char. 150: 0 → 1
Char. 159: 1 → 0
Char. 163: 1 → 0

***Barasaurus besairiei*:**
Char. 33: 1 → 0

***Bashkyroleter bashkyricus*:**
Char. 275: 1 → 0

***Bashkyroleter mesensis*:**
Char. 12: 0 → 1
Char. 66: 1 → 0
Char. 169: 1 → 0

***Belebey vegrandis*:**
Char. 154: 1 → 0

***Bradysaurus* spp.:**
Char. 19: 0 → 1
Char. 25: 1 → 0
Char. 73: 0 → 1
Char. 79: 0 → 1
Char. 135: 1 → 0
Char. 249: 0 → 1

**Captorhinidae:**
Char. 3: 0 → 1
Char. 23: 0 → 1
Char. 25: 1 → 0
Char. 26: 0 → 1
Char. 73: 0 → 1
Char. 75: 0 → 1
Char. 83: 0 → 1
Char. 108: 1 → 0
Char. 180: 1 → 0
Char. 183: 1 → 0
Char. 201: 1 → 0
Char. 203: 1 → 2
Char. 216: 1 → 0

**Caseidae:**
Char. 24: 0 → 1
Char. 25: 1 → 0
Char. 36: 0 → 1
Char. 38: 1 → 0
Char. 46: 1 → 0
Char. 50: 1 → 0
Char. 56: 0 → 1
Char. 170: 0 → 1
Char. 194: 0 → 1
Char. 273: 0 → 1
Char. 274: 0 → 1
Char. 278: 3 → 2

***Claudiosaurus germaini*:**
Char. 24: 0 → 1
Char. 34: 0 → 1
Char. 36: 0 → 1
Char. 56: 0 → 1
Char. 64: 0 → 1
Char. 73: 0 → 1
Char. 105: 0 → 1
Char. 106: 0 → 1
Char. 117: 01 → 2
Char. 126: 0 → 1
Char. 130: 0 → 1
Char. 131: 0 → 1
Char. 144: 1 → 0
Char. 148: 0 → 1
Char. 166: 1 → 0
Char. 182: 0 → 1
Char. 187: 1 → 0
Char. 190: 0 → 1
Char. 199: 0 → 1
Char. 203: 1 → 2
Char. 204: 0 → 1
Char. 220: 0 → 1
Char. 222: 1 → 0
Char. 234: 1 → 0
Char. 272: 2 → 1

***Colobomycter pholeter*:**
Char. 21: 0 → 1
Char. 25: 1 → 0
Char. 84: 1 → 0
Char. 154: 1 → 0
Char. 167: 0 → 1
Char. 267: 0 → 1

***Delorhynchus cifelli*:**
Char. 18: 0 → 1
Char. 20: 0 → 1
Char. 21: 0 → 1
Char. 24: 0 → 1
Char. 26: 0 → 1
Char. 28: 0 → 1
Char. 33: 0 → 2
Char. 39: 0 → 1
Char. 52: 0 → 1
Char. 100: 0 → 1
Char. 111: 0 → 1
Char. 116: 1 → 0
Char. 119: 1 → 0
Char. 131: 0 → 1
Char. 147: 0 → 1
Char. 156: 1 → 0
Char. 167: 0 → 1
Char. 189: 0 → 1
Char. 191: 0 → 1
Char. 204: 0 → 2
Char. 267: 0 → 1

**Diadectomorpha:**
Char. 0: 0 → 1
Char. 64: 0 → 1
Char. 70: 0 → 1
Char. 122: 0 → 1
Char. 123: 1 → 0
Char. 146: 1 → 0
Char. 275: 1 → 0
Char. 278: 3 → 0

***Emeroleter levis*:**
Char. 51: 1 → 0

**Eosauropterygia:**
Char. 159: 1 → 0
Char. 166: 1 → 0
Char. 174: 0 → 1
Char. 178: 0 → 1

Char. 194: 0 → 2
Char. 272: 1 → 0

***Eudibamus cursoris*:**
Char. 154: 1 → 2
Char. 163: 1 → 0

***Feeserpeton oklahomensis*:**
Char. 51: 0 → 1
Char. 157: 0 → 1
Char. 158: 0 → 1

***Hovasaurus boulei*:**
Char. 41: 0 → 1
Char. 55: 1 → 0
Char. 60: 1 → 0
Char. 72: 2 → 1
Char. 77: 0 → 2
Char. 78: 1 → 0
Char. 79: 0 → 1
Char. 93: 1 → 0
Char. 113: 0 → 1
Char. 138: 0 → 1
Char. 146: 1 → 0
Char. 154: 0 → 1
Char. 201: 1 → 0
Char. 204: 0 → 2
Char. 214: 1 → 0
Char. 215: 0 → 1
Char. 219: 1 → 0
Char. 220: 0 → 1
Char. 224: 0 → 1
Char. 278: 0 → 3

**Kuehneosauridae:**
Char. 7: 0 → 1
Char. 24: 0 → 1
Char. 26: 0 → 1
Char. 27: 0 → 1
Char. 34: 0 → 1
Char. 36: 0 → 1
Char. 56: 0 → 1
Char. 75: 1 → 0
Char. 79: 0 → 2
Char. 81: 1 → 0
Char. 82: 0 → 1
Char. 98: 1 → 0
Char. 107: 1 → 0
Char. 108: 1 → 0
Char. 113: 0 → 1
Char. 128: 0 → 1
Char. 140: 1 → 0
Char. 147: 1 → 0
Char. 148: 1 → 0
Char. 159: 1 → 0
Char. 181: 0 → 1
Char. 185: 0 → 1
Char. 245: 0 → 1
Char. 278: 0 → 3

***Lanthanosuchus watsoni*:**
Char. 25: 1 → 0
Char. 51: 0 → 1
Char. 76: 0 → 1
Char. 86: 0 → 1
Char. 98: 1 → 0
Char. 138: 0 → 1
Char. 144: 1 → 0
Char. 154: 1 → 2

***Macroleter poezicus*:**
Char. 9: 0 → 1
Char. 26: 0 → 1
Char. 66: 1 → 2
Char. 126: 0 → 1
Char. 134: 0 → 2
Char. 140: 0 → 1
Char. 146: 1 → 0
Char. 169: 1 → 0

***Mesosaurus* spp.:**
Char. 0: 0 → 1
Char. 2: 0 → 1
Char. 5: 1 → 0
Char. 6: 0 → 1
Char. 8: 0 → 1
Char. 9: 0 → 1
Char. 13: 0 → 1
Char. 19: 0 → 1
Char. 23: 0 → 1
Char. 26: 0 → 1
Char. 29: 1 → 0
Char. 33: 0 → 1
Char. 38: 1 → 0
Char. 41: 0 → 1
Char. 50: 1 → 0
Char. 67: 0 → 1
Char. 76: 0 → 1
Char. 83: 0 → 1
Char. 84: 1 → 0
Char. 85: 1 → 0
Char. 94: 0 → 1
Char. 107: 0 → 1
Char. 109: 0 → 1
Char. 111: 0 → 1
Char. 115: 0 → 1
Char. 146: 1 → 0
Char. 148: 0 → 1
Char. 149: 1 → 0
Char. 159: 1 → 0
Char. 163: 1 → 0
Char. 164: 0 → 1
Char. 166: 1 → 0
Char. 167: 0 → 1
Char. 176: 0 → 1
Char. 183: 1 → 0
Char. 184: 0 → 1
Char. 199: 0 → 1
Char. 202: 0 → 1
Char. 204: 0 → 2
Char. 206: 0 → 1
Char. 207: 0 → 1
Char. 209: 0 → 1
Char. 217: 0 → 1
Char. 219: 0 → 1
Char. 220: 0 → 1
Char. 231: 0 → 1
Char. 234: 1 → 0
Char. 260: 2 → 0
Char. 272: 2 → 0
Char. 278: 3 → 0

***Microleter mckinzieorum*:**
Char. 0: 0 → 1
Char. 18: 0 → 1
Char. 24: 0 → 1
Char. 25: 1 → 0
Char. 36: 0 → 1
Char. 39: 0 → 1
Char. 51: 0 → 2
Char. 56: 0 → 1
Char. 57: 1 → 0
Char. 70: 0 → 1
Char. 76: 0 → 1
Char. 79: 0 → 1
Char. 83: 0 → 1
Char. 94: 0 → 1
Char. 106: 0 → 1
Char. 110: 0 → 1
Char. 132: 1 → 0
Char. 166: 1 → 0
Char. 276: 0 → 1
Char. 278: 3 → 1

**Millerettidae:**
Char. 5: 1 → 0
Char. 24: 0 → 1
Char. 25: 1 → 0
Char. 44: 1 → 0
Char. 56: 0 → 1
Char. 57: 1 → 0
Char. 66: 0 → 2
Char. 78: 1 → 0
Char. 80: 1 → 0
Char. 84: 1 → 2
Char. 88: 1 → 0
Char. 96: 1 → 0
Char. 121: 0 → 1
Char. 124: 0 → 1
Char. 127: 0 → 1
Char. 135: 0 → 1
Char. 145: 0 → 1
Char. 159: 1 → 0
Char. 163: 1 → 0
Char. 166: 1 → 0
Char. 180: 1 → 0
Char. 202: 0 → 1
Char. 211: 0 → 1
Char. 230: 0 → 1
Char. 248: 0 → 1
Char. 252: 0 → 1
Char. 253: 0 → 1

***Nycteroleter ineptus*:**
Char. 66: 1 → 0

***Nyctiphruretus acudens*:**
Char. 21: 0 → 1
Char. 41: 0 → 1
Char. 66: 1 → 2
Char. 81: 1 → 0
Char. 83: 0 → 1
Char. 84: 1 → 2
Char. 94: 0 → 1
Char. 166: 1 → 0
Char. 224: 0 → 1
Char. 226: 1 → 0
Char. 266: 0 → 1
Char. 272: 2 → 1
Char. 276: 0 → 1

***Orovenator mayorum*:**
Char. 8: 0 → 1
Char. 24: 0 → 1
Char. 33: 1 → 0
Char. 36: 0 → 1
Char. 48: 1 → 0
Char. 50: 0 → 1
Char. 92: 1 → 0
Char. 135: 1 → 0
Char. 159: 1 → 0
Char. 160: 0 → 1
Char. 165: 0 → 1
Char. 278: 0 → 1

***Owenetta* spp.:**
Char. 142: 1 → 0
Char. 169: 1 → 0

***Paleothyris acadiana*:**
Char. 38: 1 → 0
Char. 50: 1 → 0
Char. 66: 1 → 2
Char. 102: 0 → 1
Char. 146: 1 → 0
Char. 237: 0 → 1
Char. 239: 0 → 1

***Placodus* spp.:**
Char. 0: 1 → 2
Char. 9: 0 → 1
Char. 12: 0 → 1
Char. 13: 0 → 1
Char. 19: 0 → 1
Char. 26: 0 → 1
Char. 31: 0 → 1
Char. 46: 1 → 0
Char. 57: 0 → 1
Char. 78: 1 → 0
Char. 93: 1 → 0
Char. 109: 1 → 0
Char. 140: 1 → 0
Char. 155: 0 → 1
Char. 163: 1 → 0
Char. 164: 0 → 1

***Procolophon* spp.:**
Char. 41: 0 → 1
Char. 69: 0 → 1
Char. 79: 0 → 1
Char. 83: 0 → 1
Char. 86: 0 → 1
Char. 101: 0 → 1
Char. 141: 0 → 1
Char. 149: 1 → 0
Char. 203: 1 → 2
Char. 204: 0 → 1
Char. 230: 0 → 1
Char. 237: 0 → 1
Char. 238: 0 → 1
Char. 272: 2 → 1
Char. 278: 3 → 0

***Prolacerta broomi*:**
Char. 58: 1 → 0
Char. 66: 1 → 0
Char. 67: 1 → 0
Char. 80: 1 → 0
Char. 123: 0 → 1
Char. 139: 1 → 0
Char. 147: 1 → 0
Char. 192: 1 → 0
Char. 203: 1 → 2

***Rhipaeosaurus* spp.:**
Char. 172: 0 → 1
Char. 277: 0 → 1

**Rhynchocephalia:**
Char. 0: 1 → 2
Char. 23: 1 → 0
Char. 24: 0 → 1
Char. 75: 1 → 0
Char. 77: 0 → 1
Char. 88: 1 → 0
Char. 94: 1 → 0
Char. 104: 0 → 1
Char. 139: 1 → 0
Char. 167: 1 → 0
Char. 205: 1 → 0

**Rhynchosauria:**
Char. 0: 1 → 0
Char. 7: 0 → 1
Char. 9: 0 → 1
Char. 26: 0 → 1
Char. 63: 1 → 0
Char. 68: 0 → 1
Char. 99: 1 → 0
Char. 123: 0 → 1
Char. 150: 1 → 0
Char. 160: 0 → 1
Char. 161: 0 → 1
Char. 171: 0 → 2
Char. 224: 0 → 1
Char. 241: 0 → 1

***Scutosaurus* spp.:**
Char. 175: 0 → 1
Char. 190: 0 → 1
Char. 218: 0 → 1
Char. 243: 0 → 2
Char. 244: 0 → 1
Char. 251: 0 → 1

***Sinosaurosphargis yunguiensis*:**
Char. 8: 0 → 1
Char. 30: 0 → 1
Char. 53: 0 → 1
Char. 82: 0 → 1
Char. 89: 1 → 0
Char. 127: 1 → 0

Char. 150: 1 → 0
Char. 154: 0 → 2
Char. 167: 1 → 0
Char. 253: 0 → 1
Char. 255: 0 → 1

**Squamata:**
Char. 26: 0 → 1
Char. 45: 0 → 1
Char. 79: 0 → 2
Char. 80: 1 → 0
Char. 81: 1 → 0
Char. 82: 0 → 1
Char. 92: 1 → 0
Char. 106: 0 → 1
Char. 109: 1 → 0
Char. 117: 01 → 2
Char. 122: 0 → 1
Char. 160: 0 → 1
Char. 200: 0 → 1
Char. 245: 0 → 1

***Trilophosaurus buettneri*:**
Char. 5: 1 → 0
Char. 11: 0 → 1
Char. 55: 1 → 0
Char. 93: 1 → 0
Char. 104: 0 → 1
Char. 106: 0 → 1
Char. 113: 0 → 1
Char. 122: 0 → 1
Char. 136: 1 → 0
Char. 144: 1 → 0
Char. 154: 0 → 1
Char. 157: 0 → 1
Char. 159: 1 → 0
Char. 177: 0 → 12
Char. 194: 0 → 1
Char. 203: 1 → 2
Char. 207: 1 → 0
Char. 208: 1 → 0
Char. 272: 1 → 0

***Youngina capensis*:**
Char. 5: 1 → 0
Char. 21: 0 → 1
Char. 44: 1 → 0
Char. 56: 0 → 1
Char. 75: 0 → 1
Char. 92: 1 → 0
Char. 134: 0 → 1
Char. 163: 1 → 0
Char. 170: 0 → 1
Char. 201: 1 → 0
Char. 211: 0 → 1
Char. 215: 0 → 1
Char. 224: 0 → 1
Char. 231: 0 → 1
Char. 239: 0 → 1

**Node 47:**
Char. 35: 0 → 1
Char. 58: 0 → 1
Char. 61: 0 → 1
Char. 63: 0 → 1
Char. 66: 0 → 1
Char. 69: 0 → 1
Char. 73: 0 → 1
Char. 75: 0 → 1
Char. 107: 0 → 1
Char. 123: 1 → 0
Char. 126: 0 → 1
Char. 131: 0 → 1
Char. 134: 0 → 1
Char. 140: 0 → 1
Char. 145: 0 → 1
Char. 147: 0 → 1
Char. 148: 0 → 1
Char. 150: 0 → 1
Char. 151: 0 → 1
Char. 167: 0 → 1
Char. 176: 0 → 1
Char. 184: 0 → 1
Char. 190: 0 → 1
Char. 205: 0 → 1
Char. 208: 0 → 1
Char. 210: 0 → 1
Char. 217: 0 → 1
Char. 230: 0 → 1

**Node 48:**
Char. 0: 0 → 1
Char. 20: 0 → 1
Char. 29: 1 → 0
Char. 33: 0 → 1
Char. 50: 1 → 0
Char. 57: 1 → 0
Char. 59: 0 → 1
Char. 60: 0 → 1
Char. 62: 0 → 1
Char. 67: 0 → 1
Char. 72: 1 → 2
Char. 89: 0 → 1
Char. 109: 0 → 1
Char. 111: 0 → 1
Char. 112: 0 → 1
Char. 120: 0 → 1
Char. 129: 0 → 1
Char. 135: 0 → 1
Char. 136: 0 → 1
Char. 139: 0 → 1
Char. 154: 1 → 0
Char. 179: 0 → 1
Char. 187: 0 → 1
Char. 188: 0 → 1
Char. 191: 0 → 1
Char. 193: 0 → 1
Char. 196: 0 → 1
Char. 202: 0 → 1
Char. 207: 0 → 1
Char. 209: 0 → 1
Char. 214: 0 → 1
Char. 219: 0 → 1
Char. 222: 0 → 1
Char. 237: 0 → 1
Char. 265: 0 → 1
Char. 266: 0 → 1
Char. 275: 1 → 0
Char. 278: 3 → 0

**Node 49:**
Char. 5: 0 → 1
Char. 29: 0 → 1
Char. 40: 0 → 2
Char. 74: 0 → 1
Char. 80: 0 → 1
Char. 84: 0 → 1
Char. 88: 0 → 1
Char. 116: 0 → 1
Char. 119: 0 → 1
Char. 132: 0 → 1
Char. 149: 0 → 1
Char. 156: 0 → 1
Char. 163: 0 → 1
Char. 166: 0 → 1
Char. 169: 0 → 1
Char. 180: 0 → 1
Char. 201: 0 → 1
Char. 203: 0 → 1
Char. 221: 0 → 1
Char. 234: 0 → 1
Char. 235: 0 → 1

**Node 50:**
Char. 72: 0 → 1
Char. 79: 1 → 0
Char. 81: 0 → 1
Char. 93: 0 → 1
Char. 97: 0 → 1
Char. 104: 1 → 0
Char. 144: 0 → 1
Char. 173: 0 → 1
Char. 183: 0 → 1

**Node 52:**
Char. 95: 0 → 1
Char. 113: 0 → 1
Char. 114: 0 → 1

**Node 53:**
Char. 20: 0 → 1
Char. 47: 0 → 1
Char. 79: 0 → 1
Char. 110: 0 → 1
Char. 131: 0 → 1
Char. 137: 0 → 1
Char. 140: 0 → 2
Char. 147: 0 → 1

**Node 54:**
Char. 19: 0 → 1
Char. 92: 1 → 0

**Node 55:**
Char. 4: 0 → 1
Char. 15: 0 → 1
Char. 29: 0 → 1
Char. 35: 1 → 0
Char. 48: 1 → 0
Char. 81: 1 → 0
Char. 210: 1 → 0
Char. 213: 0 → 1
Char. 226: 0 → 2
Char. 228: 0 → 1
Char. 275: 0 → 1

**Node 56:**
Char. 73: 0 → 1
Char. 205: 0 → 1
Char. 273: 0 → 1
Char. 276: 0 → 1

**Node 57:**
Char. 18: 0 → 1
Char. 23: 0 → 1
Char. 25: 1 → 0
Char. 37: 0 → 1
Char. 103: 0 → 1
Char. 106: 0 → 1
Char. 107: 0 → 1
Char. 126: 0 → 1
Char. 150: 0 → 1

**Node 58:**
Char. 186: 0 → 1

**Node 59:**
Char. 148: 0 → 1
Char. 183: 1 → 2
Char. 194: 0 → 1
Char. 201: 1 → 0
Char. 211: 0 → 1
Char. 226: 0 → 1
Char. 234: 1 → 0
Char. 252: 0 → 1

**Node 61:**
Char. 133: 0 → 1

**Node 62:**
Char. 100: 0 → 1
Char. 113: 0 → 1

**Node 63:**
Char. 70: 0 → 1
Char. 118: 0 → 1
Char. 125: 0 → 1

**Node 65:**
Char. 38: 1 → 2
Char. 39: 0 → 1
Char. 50: 1 → 0
Char. 58: 0 → 1
Char. 59: 0 → 1
Char. 60: 0 → 1
Char. 72: 1 → 2
Char. 83: 0 → 1
Char. 85: 1 → 0
Char. 88: 1 → 0
Char. 95: 0 → 1
Char. 104: 0 → 1
Char. 105: 0 → 1
Char. 106: 0 → 2
Char. 107: 0 → 1
Char. 109: 0 → 1
Char. 110: 0 → 1
Char. 146: 1 → 0
Char. 148: 0 → 1
Char. 155: 0 → 2
Char. 183: 1 → 2

**Node 66:**
Char. 23: 0 → 1
Char. 33: 1 → 0
Char. 38: 2 → 1
Char. 39: 1 → 0
Char. 42: 0 → 1
Char. 43: 0 → 1
Char. 46: 1 → 0
Char. 49: 1 → 0
Char. 71: 1 → 0
Char. 83: 0 → 2
Char. 101: 0 → 1
Char. 103: 0 → 1
Char. 106: 0 → 1
Char. 161: 0 → 1
Char. 163: 1 → 0
Char. 172: 0 → 2
Char. 174: 0 → 1
Char. 188: 0 → 1
Char. 203: 1 → 2
Char. 212: 0 → 1
Char. 216: 1 → 0
Char. 221: 1 → 0
Char. 236: 0 → 1
Char. 238: 0 → 2
Char. 241: 0 → 1
Char. 242: 0 → 1
Char. 273: 0 → 1
Char. 274: 0 → 1
Char. 275: 1 → 0

**Node 67:**
Char. 52: 0 → 1
Char. 75: 0 → 1
Char. 84: 1 → 0
Char. 121: 0 → 1
Char. 180: 1 → 0
Char. 230: 0 → 1

**Node 68:**
Char. 5: 1 → 0
Char. 29: 1 → 0
Char. 44: 1 → 0
Char. 59: 0 → 1
Char. 60: 0 → 1
Char. 66: 0 → 1
Char. 67: 0 → 1
Char. 72: 1 → 2
Char. 78: 1 → 0
Char. 84: 1 → 0
Char. 88: 1 → 0
Char. 93: 1 → 0
Char. 111: 0 → 1
Char. 123: 1 → 0
Char. 129: 0 → 1
Char. 154: 1 → 0
Char. 159: 1 → 0
Char. 163: 1 → 0
Char. 169: 1 → 0
Char. 170: 0 → 1

Char. 197: 0 → 1
Char. 221: 1 → 0
Char. 234: 1 → 0

**Node 69:**
Char. 83: 0 → 1
Char. 85: 1 → 0
Char. 107: 0 → 1

**Node 70:**
Char. 2: 0 → 1
Char. 6: 0 → 1
Char. 33: 1 → 2
Char. 98: 1 → 0
Char. 101: 0 → 1
Char. 104: 0 → 1
Char. 106: 0 → 1
Char. 113: 0 → 1
Char. 117: 01 → 2
Char. 181: 0 → 1
Char. 186: 0 → 1
Char. 193: 1 → 0
Char. 198: 0 → 1
Char. 200: 0 → 1
Char. 220: 0 → 1
Char. 234: 1 → 0
Char. 240: 1 → 0
Char. 266: 1 → 0
Char. 267: 1 → 0

**Node 71:**
Char. 17: 0 → 1
Char. 42: 0 → 1
Char. 251: 0 → 1
Char. 264: 0 → 1

**Node 72:**
Char. 33: 1 → 2
Char. 48: 1 → 0
Char. 61: 1 → 2
Char. 68: 0 → 1
Char. 112: 1 → 0
Char. 128: 0 → 1
Char. 138: 0 → 1
Char. 146: 1 → 0
Char. 155: 0 → 1
Char. 158: 0 → 1
Char. 192: 1 → 2
Char. 224: 0 → 1
Char. 226: 0 → 1
Char. 227: 0 → 1
Char. 233: 0 → 1
Char. 235: 1 → 2
Char. 267: 1 → 2

**Node 73:**
Char. 61: 1 → 3
Char. 90: 0 → 1
Char. 91: 0 → 1
Char. 109: 1 → 0
Char. 155: 0 → 1
Char. 209: 1 → 0

**ANALYSIS 70**
**(NO *CANDELARIA BARBOURI*, IMPLIED WEIGHTING, K = 2.75)**

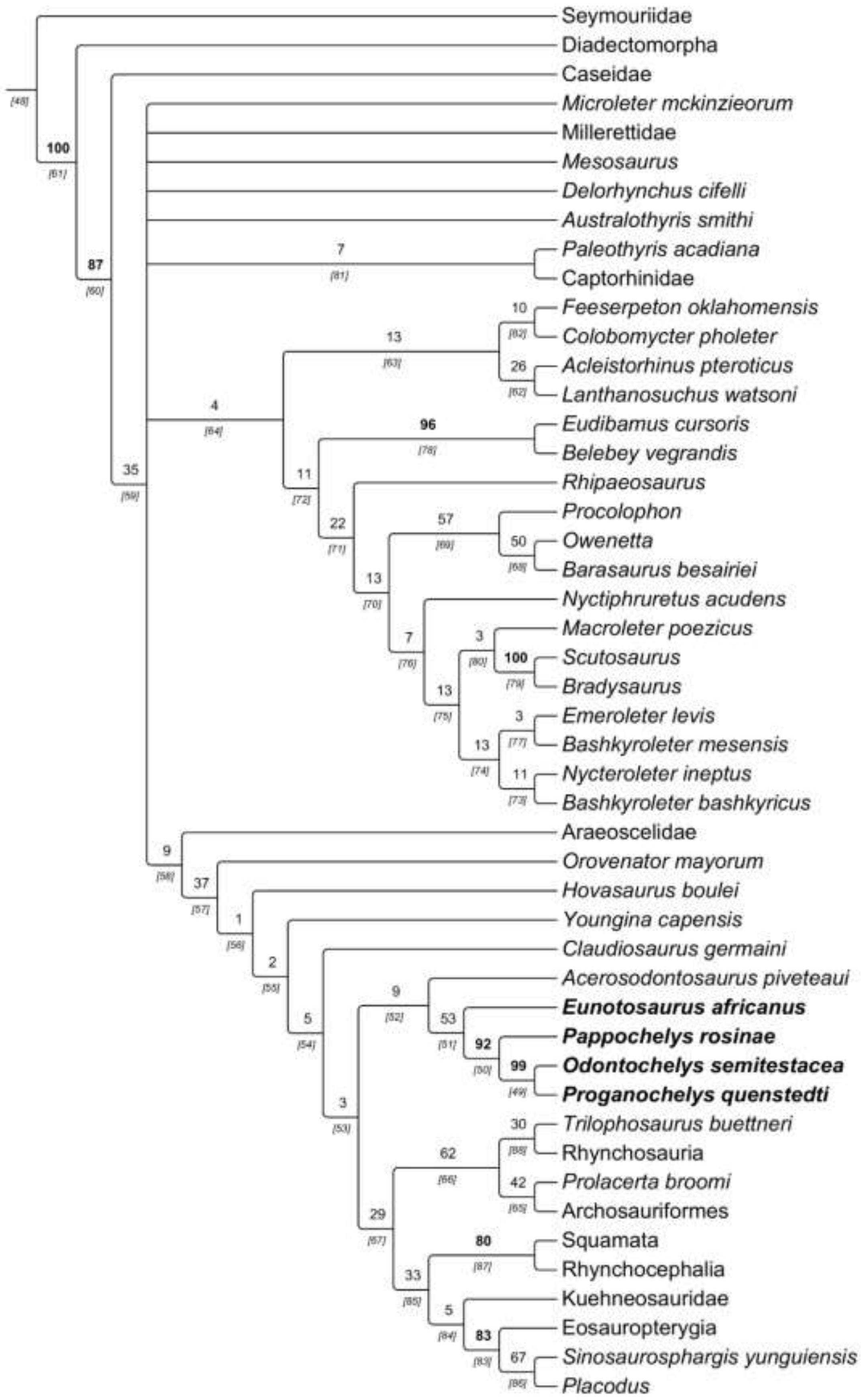

***Proganochelys quenstedti*:**
Char. 8: 0 → 1
Char. 11: 0 → 1
Char. 106: 0 → 1
Char. 108: 1 → 0
Char. 128: 0 → 1
Char. 175: 0 → 1
Char. 202: 1 → 0
Char. 206: 2 → 0
Char. 207: 1 → 0
Char. 209: 1 → 0
Char. 244: 0 → 1
Char. 248: 0 → 1
Char. 250: 0 → 1
Char. 252: 1 → 0
Char. 262: 0 → 1

***Pappochelys rosinae*:**
Char. 0: 2 → 0
Char. 1: 0 → 1
Char. 5: 1 → 0
Char. 12: 0 → 1
Char. 41: 0 → 1
Char. 48: 0 → 1
Char. 49: 0 → 1
Char. 75: 0 → 1
Char. 81: 0 → 1
Char. 169: 1 → 0
Char. 260: 2 → 0

***Eunotosaurus africanus*:**
Char. 19: 0 → 1
Char. 59: 1 → 0
Char. 60: 1 → 0
Char. 72: 2 → 1
Char. 76: 0 → 1
Char. 97: 1 → 0
Char. 103: 0 → 1
Char. 131: 1 → 0
Char. 153: 1 → 0
Char. 191: 1 → 0
Char. 192: 1 → 0
Char. 202: 1 → 0
Char. 211: 0 → 1
Char. 222: 1 → 0
Char. 237: 1 → 0
Char. 248: 0 → 1
Char. 249: 0 → 2
Char. 250: 0 → 1
Char. 263: 0 → 1
Char. 272: 1 → 2
Char. 273: 0 → 1
Char. 274: 0 → 1
Char. 275: 0 → 1
Char. 276: 0 → 1
Char. 277: 0 → 1
Char. 278: 0 → 3

***Acerosodontosaurus piveteaui*:**
Char. 127: 1 → 0
Char. 128: 0 → 1
Char. 278: 0 → 1

***Acleistorhinus pteroticus*:**
Char. 21: 0 → 1
Char. 146: 1 → 0

**Araeoscelidae:**
Char. 23: 0 → 1
Char. 28: 0 → 1
Char. 106: 0 → 1
Char. 117: 01 → 2
Char. 169: 1 → 0
Char. 170: 0 → 1
Char. 197: 0 → 1
Char. 239: 0 → 1

**Archosauriformes:**
Char. 32: 0 → 1
Char. 94: 1 → 0
Char. 112: 1 → 0
Char. 152: 0 → 1
Char. 154: 0 → 2
Char. 166: 1 → 0
Char. 171: 0 → 1
Char. 185: 0 → 1
Char. 201: 0 → 1
Char. 204: 0 → 1
Char. 218: 0 → 3
Char. 224: 0 → 1
Char. 242: 0 → 1

***Australothyris smithi*:**
Char. 23: 0 → 1
Char. 34: 0 → 1
Char. 55: 1 → 0
Char. 71: 1 → 0
Char. 79: 0 → 1
Char. 85: 1 → 0
Char. 98: 1 → 0
Char. 100: 0 → 1
Char. 103: 0 → 1
Char. 110: 0 → 1
Char. 112: 0 → 1
Char. 123: 1 → 0
Char. 129: 0 → 1
Char. 131: 0 → 1
Char. 132: 1 → 0
Char. 144: 1 → 0
Char. 147: 0 → 1
Char. 149: 1 → 0
Char. 150: 0 → 1
Char. 166: 0 → 1

***Barasaurus besairiei*:**
Char. 33: 1 → 0

***Bashkyroleter bashkyricus*:**
Char. 275: 1 → 0

***Bashkyroleter mesensis*:**
Char. 12: 0 → 1
Char. 66: 1 → 0
Char. 169: 1 → 0

***Belebey vegrandis*:**
Char. 154: 1 → 0

***Bradysaurus* spp.:**
Char. 19: 0 → 1
Char. 25: 1 → 0
Char. 73: 0 → 1
Char. 79: 0 → 1
Char. 135: 1 → 0
Char. 249: 0 → 1

**Captorhinidae:**
Char. 3: 0 → 1
Char. 23: 0 → 1
Char. 25: 1 → 0
Char. 26: 0 → 1
Char. 73: 0 → 1
Char. 75: 0 → 1
Char. 108: 1 → 0
Char. 183: 1 → 0
Char. 201: 1 → 0
Char. 203: 1 → 2
Char. 216: 1 → 0

**Caseidae:**
Char. 25: 1 → 0
Char. 36: 0 → 1
Char. 38: 1 → 0
Char. 46: 1 → 0
Char. 50: 1 → 0
Char. 56: 0 → 1
Char. 170: 0 → 1
Char. 194: 0 → 1
Char. 273: 0 → 1
Char. 274: 0 → 1
Char. 278: 3 → 2

***Claudiosaurus germaini*:**
Char. 34: 0 → 1
Char. 36: 0 → 1
Char. 43: 1 → 0
Char. 105: 0 → 1
Char. 106: 0 → 1
Char. 117: 01 → 2
Char. 127: 1 → 0
Char. 144: 1 → 0
Char. 187: 1 → 0
Char. 199: 0 → 1
Char. 201: 0 → 1
Char. 204: 0 → 1
Char. 222: 1 → 0
Char. 234: 1 → 0

***Colobomycter pholeter*:**
Char. 21: 0 → 1
Char. 25: 1 → 0
Char. 84: 1 → 0
Char. 154: 1 → 0
Char. 167: 0 → 1
Char. 267: 0 → 1

***Delorhynchus cifelli*:**
Char. 5: 0 → 1
Char. 18: 0 → 1
Char. 20: 0 → 1
Char. 21: 0 → 1
Char. 26: 0 → 1
Char. 28: 0 → 1
Char. 33: 0 → 2
Char. 39: 0 → 1
Char. 52: 0 → 1
Char. 100: 0 → 1
Char. 111: 0 → 1
Char. 131: 0 → 1
Char. 147: 0 → 1
Char. 156: 1 → 0
Char. 159: 0 → 1
Char. 163: 0 → 1
Char. 166: 0 → 1
Char. 167: 0 → 1
Char. 189: 0 → 1
Char. 191: 0 → 1
Char. 192: 0 → 1
Char. 204: 0 → 2
Char. 267: 0 → 1

**Diadectomorpha:**
Char. 0: 0 → 1
Char. 64: 0 → 1
Char. 70: 0 → 1
Char. 122: 0 → 1
Char. 123: 1 → 0
Char. 146: 1 → 0
Char. 275: 1 → 0
Char. 278: 3 → 0

***Emeroleter levis*:**
Char. 51: 1 → 0

**Eosauropterygia:**
Char. 166: 1 → 0
Char. 174: 0 → 1
Char. 178: 0 → 1
Char. 194: 0 → 2
Char. 272: 1 → 0

***Eudibamus cursoris*:**
Char. 154: 1 → 2
Char. 163: 1 → 0

***Feeserpeton oklahomensis*:**
Char. 157: 0 → 1

***Hovasaurus boulei*:**
Char. 41: 0 → 1
Char. 55: 1 → 0
Char. 72: 2 → 1
Char. 77: 0 → 2
Char. 78: 1 → 0
Char. 79: 0 → 1
Char. 93: 1 → 0
Char. 113: 0 → 1
Char. 138: 0 → 1
Char. 146: 1 → 0
Char. 204: 0 → 2
Char. 206: 0 → 2

**Kuehneosauridae:**
Char. 7: 0 → 1
Char. 21: 1 → 0
Char. 27: 0 → 1
Char. 43: 1 → 0
Char. 79: 0 → 2
Char. 107: 1 → 0
Char. 140: 1 → 0
Char. 147: 1 → 0
Char. 148: 1 → 0
Char. 245: 0 → 1
Char. 272: 1 → 2
Char. 278: 0 → 3

***Lanthanosuchus watsoni*:**
Char. 25: 1 → 0
Char. 86: 0 → 1
Char. 98: 1 → 0
Char. 138: 0 → 1
Char. 144: 1 → 0
Char. 154: 1 → 2

***Macroleter poezicus*:**
Char. 9: 0 → 1
Char. 26: 0 → 1
Char. 66: 1 → 2
Char. 126: 0 → 1
Char. 134: 0 → 2
Char. 140: 0 → 1
Char. 146: 1 → 0
Char. 169: 1 → 0

***Mesosaurus* spp.:**
Char. 0: 0 → 1
Char. 2: 0 → 1
Char. 6: 0 → 1
Char. 8: 0 → 1
Char. 9: 0 → 1
Char. 13: 0 → 1
Char. 19: 0 → 1
Char. 23: 0 → 1
Char. 26: 0 → 1
Char. 29: 1 → 0
Char. 33: 0 → 1
Char. 38: 1 → 0
Char. 41: 0 → 1
Char. 50: 1 → 0
Char. 67: 0 → 1
Char. 76: 0 → 1
Char. 84: 1 → 0
Char. 85: 1 → 0
Char. 94: 0 → 1
Char. 107: 0 → 1
Char. 109: 0 → 1
Char. 111: 0 → 1
Char. 115: 0 → 1
Char. 146: 1 → 0
Char. 148: 0 → 1
Char. 149: 1 → 0
Char. 164: 0 → 1
Char. 167: 0 → 1
Char. 176: 0 → 1
Char. 183: 1 → 0
Char. 184: 0 → 1
Char. 199: 0 → 1
Char. 202: 0 → 1
Char. 204: 0 → 2
Char. 206: 0 → 1
Char. 207: 0 → 1
Char. 209: 0 → 1

Char. 217: 0 → 1
Char. 219: 0 → 1
Char. 220: 0 → 1
Char. 231: 0 → 1
Char. 260: 2 → 0
Char. 272: 2 → 0
Char. 278: 3 → 0

***Microleter mckinzieorum*:**
Char. 0: 0 → 1
Char. 18: 0 → 1
Char. 25: 1 → 0
Char. 36: 0 → 1
Char. 39: 0 → 1
Char. 51: 0 → 2
Char. 56: 0 → 1
Char. 70: 0 → 1
Char. 76: 0 → 1
Char. 79: 0 → 1
Char. 94: 0 → 1
Char. 106: 0 → 1
Char. 110: 0 → 1
Char. 132: 1 → 0
Char. 159: 0 → 1
Char. 276: 0 → 1
Char. 278: 3 → 1

**Millerettidae:**
Char. 25: 1 → 0
Char. 44: 1 → 0
Char. 56: 0 → 1
Char. 66: 0 → 2
Char. 78: 1 → 0
Char. 80: 1 → 0
Char. 84: 1 → 2
Char. 88: 1 → 0
Char. 96: 1 → 0
Char. 124: 0 → 1
Char. 127: 0 → 1
Char. 135: 0 → 1
Char. 145: 0 → 1
Char. 202: 0 → 1
Char. 211: 0 → 1
Char. 230: 0 → 1
Char. 234: 0 → 1
Char. 248: 0 → 1
Char. 252: 0 → 1
Char. 253: 0 → 1

***Nycteroleter ineptus*:**
Char. 66: 1 → 0

***Nyctiphruretus acudens*:**
Char. 21: 0 → 1
Char. 41: 0 → 1
Char. 66: 1 → 2
Char. 81: 1 → 0
Char. 84: 1 → 2
Char. 94: 0 → 1
Char. 110: 1 → 0
Char. 166: 1 → 0
Char. 215: 1 → 0
Char. 224: 0 → 1
Char. 226: 1 → 0

Char. 266: 0 → 1
Char. 272: 2 → 1
Char. 276: 0 → 1

***Orovenator mayorum*:**
Char. 8: 0 → 1
Char. 36: 0 → 1
Char. 160: 0 → 1
Char. 165: 0 → 1

***Owenetta* spp.:**
Char. 142: 1 → 0
Char. 169: 1 → 0

***Paleothyris acadiana*:**
Char. 38: 1 → 0
Char. 50: 1 → 0
Char. 66: 1 → 2
Char. 102: 0 → 1
Char. 146: 1 → 0
Char. 237: 0 → 1
Char. 239: 0 → 1

***Placodus* spp.:**
Char. 0: 1 → 2
Char. 9: 0 → 1
Char. 12: 0 → 1
Char. 13: 0 → 1
Char. 19: 0 → 1
Char. 31: 0 → 1
Char. 46: 1 → 0
Char. 57: 0 → 1
Char. 78: 1 → 0
Char. 93: 1 → 0
Char. 102: 1 → 2
Char. 109: 1 → 0
Char. 140: 1 → 0
Char. 155: 0 → 1
Char. 163: 1 → 0
Char. 164: 0 → 1

***Procolophon* spp.:**
Char. 41: 0 → 1
Char. 69: 0 → 1
Char. 79: 0 → 1
Char. 86: 0 → 1
Char. 88: 0 → 1
Char. 101: 0 → 1
Char. 141: 0 → 1
Char. 149: 1 → 0
Char. 203: 1 → 2
Char. 204: 0 → 1
Char. 230: 0 → 1
Char. 237: 0 → 1
Char. 238: 0 → 1
Char. 239: 1 → 0
Char. 272: 2 → 1
Char. 278: 3 → 0

***Prolacerta broomi*:**
Char. 58: 1 → 0
Char. 66: 1 → 0
Char. 67: 1 → 0
Char. 80: 1 → 0
Char. 139: 1 → 0

Char. 147: 1 → 0
Char. 192: 1 → 0
Char. 206: 0 → 12

***Rhipaeosaurus* spp.:**
Char. 110: 1 → 0
Char. 172: 0 → 1
Char. 277: 0 → 1

**Rhynchocephalia:**
Char. 0: 1 → 2
Char. 23: 1 → 0
Char. 77: 0 → 1
Char. 81: 0 → 1
Char. 88: 1 → 0
Char. 94: 1 → 0
Char. 139: 1 → 0
Char. 167: 1 → 0
Char. 205: 1 → 0

**Rhynchosauria:**
Char. 0: 1 → 0
Char. 7: 0 → 1
Char. 9: 0 → 1
Char. 26: 0 → 1
Char. 68: 0 → 1
Char. 99: 1 → 0
Char. 150: 1 → 0
Char. 160: 0 → 1
Char. 161: 0 → 1
Char. 171: 0 → 2
Char. 223: 0 → 1
Char. 224: 0 → 1
Char. 241: 0 → 1

***Scutosaurus* spp.:**
Char. 175: 0 → 1
Char. 190: 0 → 1
Char. 218: 0 → 1
Char. 243: 0 → 2
Char. 244: 0 → 1
Char. 251: 0 → 1

***Sinosaurosphargis yunguiensis*:**
Char. 8: 0 → 1
Char. 30: 0 → 1
Char. 44: 0 → 1
Char. 53: 0 → 1
Char. 89: 1 → 0
Char. 127: 1 → 0
Char. 150: 1 → 0
Char. 154: 0 → 2
Char. 167: 1 → 0
Char. 253: 0 → 1
Char. 255: 0 → 1

**Squamata:**
Char. 45: 0 → 1
Char. 79: 0 → 2
Char. 80: 1 → 0
Char. 92: 1 → 0
Char. 109: 1 → 0
Char. 160: 0 → 1

Char. 200: 0 → 1
Char. 245: 0 → 1

***Trilophosaurus buettneri*:**
Char. 5: 1 → 0
Char. 11: 0 → 1
Char. 44: 0 → 1
Char. 55: 1 → 0
Char. 93: 1 → 0
Char. 104: 0 → 1
Char. 106: 0 → 1
Char. 113: 0 → 1
Char. 122: 0 → 1
Char. 136: 1 → 0
Char. 144: 1 → 0
Char. 154: 0 → 1
Char. 157: 0 → 1
Char. 159: 1 → 0
Char. 177: 0 → 12
Char. 194: 0 → 1
Char. 207: 1 → 0
Char. 208: 1 → 0
Char. 272: 1 → 0

***Youngina capensis*:**
Char. 75: 0 → 1
Char. 170: 0 → 1
Char. 211: 0 → 1
Char. 239: 0 → 1

**Node 49:**
Char. 46: 1 → 0
Char. 88: 1 → 0
Char. 89: 1 → 0
Char. 93: 1 → 0
Char. 176: 0 → 1
Char. 195: 0 → 2
Char. 198: 0 → 1
Char. 246: 1 → 2
Char. 259: 0 → 1
Char. 265: 1 → 0
Char. 270: 0 → 1

**Node 50:**
Char. 65: 0 → 1
Char. 184: 0 → 1
Char. 205: 0 → 1
Char. 210: 0 → 1
Char. 219: 1 → 0
Char. 241: 0 → 1
Char. 246: 0 → 1
Char. 254: 0 → 1
Char. 255: 0 → 1
Char. 256: 0 → 1
Char. 268: 0 → 1
Char. 269: 0 → 1

**Node 51:**
Char. 174: 0 → 1
Char. 204: 0 → 2
Char. 247: 0 → 1
Char. 251: 0 → 1
Char. 253: 0 → 2

**Node 52:**
Char. 78: 1 → 0
Char. 206: 0 → 2

**Node 53:**
Char. 27: 1 → 0
Char. 48: 1 → 0
Char. 81: 1 → 0
Char. 84: 0 → 1
Char. 147: 0 → 1
Char. 166: 0 → 1
Char. 208: 0 → 1

**Node 54:**
Char. 23: 0 → 1
Char. 73: 0 → 1
Char. 94: 0 → 1
Char. 131: 0 → 1
Char. 148: 0 → 1
Char. 224: 1 → 0
Char. 272: 2 → 1

**Node 55:**
Char. 44: 1 → 0
Char. 134: 0 → 1
Char. 214: 0 → 1
Char. 219: 0 → 1

**Node 56:**
Char. 135: 0 → 1

**Node 57:**
Char. 20: 0 → 1
Char. 62: 0 → 1

**Node 58:**
Char. 0: 0 → 1
Char. 27: 0 → 1
Char. 29: 1 → 0
Char. 40: 2 → 0
Char. 59: 0 → 1
Char. 67: 0 → 1
Char. 72: 1 → 2
Char. 84: 1 → 0
Char. 89: 0 → 1
Char. 111: 0 → 1
Char. 112: 0 → 1
Char. 120: 0 → 1
Char. 192: 0 → 1
Char. 193: 0 → 1
Char. 222: 0 → 1
Char. 224: 0 → 1
Char. 234: 0 → 1
Char. 237: 0 → 1
Char. 266: 0 → 1

**Node 59:**
Char. 29: 0 → 1
Char. 40: 0 → 2
Char. 74: 0 → 1
Char. 80: 0 → 1
Char. 84: 0 → 1
Char. 88: 0 → 1
Char. 132: 0 → 1
Char. 149: 0 → 1

Char. 156: 0 → 1
Char. 169: 0 → 1
Char. 201: 0 → 1
Char. 203: 0 → 1
Char. 235: 0 → 1

**Node 60:**
Char. 72: 0 → 1
Char. 79: 1 → 0
Char. 81: 0 → 1
Char. 93: 0 → 1
Char. 97: 0 → 1
Char. 104: 1 → 0
Char. 144: 0 → 1
Char. 173: 0 → 1
Char. 183: 0 → 1

**Node 62:**
Char. 113: 0 → 1
Char. 114: 0 → 1

**Node 63:**
Char. 47: 0 → 1
Char. 79: 0 → 1
Char. 140: 0 → 2

**Node 64:**
Char. 5: 0 → 1
Char. 110: 0 → 1
Char. 137: 0 → 1
Char. 159: 0 → 1
Char. 163: 0 → 1
Char. 166: 0 → 1
Char. 192: 0 → 1

**Node 65:**
Char. 19: 0 → 1
Char. 21: 1 → 0
Char. 92: 1 → 0

**Node 66:**
Char. 4: 0 → 1
Char. 29: 0 → 1
Char. 213: 0 → 1
Char. 226: 01 → 2
Char. 228: 0 → 1
Char. 275: 0 → 1

**Node 67:**
Char. 58: 0 → 1
Char. 61: 0 → 1
Char. 66: 0 → 1
Char. 107: 0 → 1
Char. 140: 0 → 1
Char. 150: 0 → 1
Char. 167: 0 → 1
Char. 176: 0 → 1
Char. 205: 0 → 1
Char. 239: 0 → 1
Char. 260: 2 → 1

**Node 68:**
Char. 73: 0 → 1
Char. 205: 0 → 1
Char. 273: 0 → 1
Char. 276: 0 → 1

**Node 69:**
Char. 18: 0 → 1
Char. 25: 1 → 0
Char. 37: 0 → 1
Char. 106: 0 → 1
Char. 107: 0 → 1
Char. 126: 0 → 1
Char. 150: 0 → 1

**Node 70:**
Char. 186: 0 → 1

**Node 71:**
Char. 194: 0 → 1
Char. 201: 1 → 0
Char. 211: 0 → 1
Char. 226: 0 → 1

**Node 72:**
Char. 38: 1 → 2
Char. 39: 0 → 1
Char. 49: 0 → 1
Char. 66: 0 → 1
Char. 88: 1 → 0
Char. 112: 0 → 1
Char. 148: 0 → 1

**Node 74:**
Char. 133: 0 → 1

**Node 75:**
Char. 100: 0 → 1
Char. 113: 0 → 1

**Node 76:**
Char. 70: 0 → 1
Char. 118: 0 → 1
Char. 125: 0 → 1

**Node 77:**
Char. 110: 1 → 0

**Node 78:**
Char. 58: 0 → 1
Char. 59: 0 → 1
Char. 60: 0 → 1
Char. 72: 1 → 2
Char. 104: 0 → 1
Char. 105: 0 → 1
Char. 106: 0 → 2
Char. 107: 0 → 1
Char. 109: 0 → 1
Char. 146: 1 → 0

**Node 79:**
Char. 23: 0 → 1
Char. 33: 1 → 0
Char. 38: 2 → 1
Char. 39: 1 → 0
Char. 42: 0 → 1
Char. 43: 0 → 1
Char. 46: 1 → 0
Char. 49: 1 → 0
Char. 71: 1 → 0
Char. 83: 0 → 2
Char. 101: 0 → 1
Char. 103: 0 → 1
Char. 106: 0 → 1
Char. 161: 0 → 1
Char. 163: 1 → 0
Char. 172: 0 → 2
Char. 174: 0 → 1
Char. 188: 0 → 1
Char. 203: 1 → 2
Char. 212: 0 → 1
Char. 216: 1 → 0
Char. 236: 0 → 1
Char. 238: 0 → 2

Char. 239: 1 → 0
Char. 241: 0 → 1
Char. 242: 0 → 1
Char. 273: 0 → 1
Char. 274: 0 → 1
Char. 275: 1 → 0

**Node 80:**
Char. 52: 0 → 1
Char. 75: 0 → 1
Char. 84: 1 → 0
Char. 121: 0 → 1
Char. 230: 0 → 1

**Node 81:**
Char. 29: 1 → 0
Char. 44: 1 → 0
Char. 59: 0 → 1
Char. 60: 0 → 1
Char. 66: 0 → 1
Char. 67: 0 → 1
Char. 72: 1 → 2
Char. 78: 1 → 0
Char. 84: 1 → 0
Char. 88: 1 → 0
Char. 93: 1 → 0
Char. 111: 0 → 1
Char. 123: 1 → 0
Char. 129: 0 → 1
Char. 154: 1 → 0
Char. 169: 1 → 0
Char. 170: 0 → 1
Char. 197: 0 → 1

**Node 82:**
Char. 107: 0 → 1

**Node 83:**
Char. 2: 0 → 1
Char. 6: 0 → 1
Char. 81: 0 → 1
Char. 186: 0 → 1
Char. 198: 0 → 1
Char. 200: 0 → 1
Char. 220: 0 → 1
Char. 239: 1 → 0
Char. 267: 1 → 0

**Node 84:**
Char. 48: 0 → 1
Char. 98: 1 → 0
Char. 113: 0 → 1
Char. 181: 0 → 1
Char. 206: 0 → 2

**Node 85:**
Char. 35: 0 → 1
Char. 103: 0 → 1
Char. 184: 0 → 1
Char. 201: 0 → 1
Char. 210: 0 → 1
Char. 223: 0 → 1
Char. 229: 0 → 1

**Node 86:**
Char. 17: 0 → 1
Char. 42: 0 → 1
Char. 251: 0 → 1
Char. 264: 0 → 1

**Node 87:**
Char. 41: 0 → 1
Char. 44: 0 → 1
Char. 61: 1 → 2
Char. 112: 1 → 0
Char. 138: 0 → 1
Char. 146: 1 → 0
Char. 192: 1 → 2
Char. 224: 0 → 1
Char. 227: 0 → 1
Char. 233: 0 → 1
Char. 267: 1 → 2

**Node 88:**
Char. 61: 1 → 3
Char. 90: 0 → 1
Char. 91: 0 → 1
Char. 109: 1 → 0
Char. 209: 1 → 0

**ANALYSIS 71**
**(ALL TAXA, NO CHARACTER 253)**

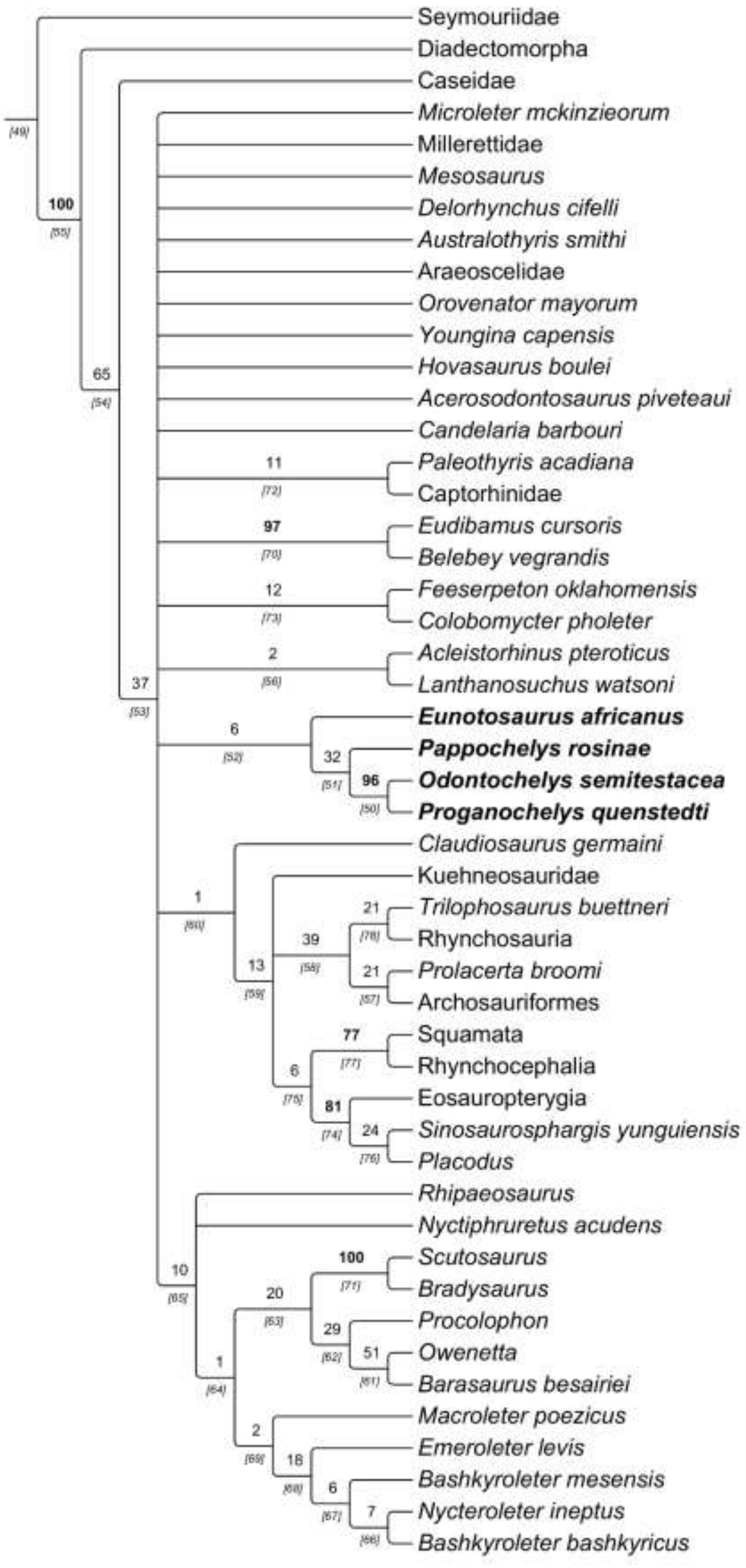

***Proganochelys quenstedti*:**
Char. 8: 0 → 1
Char. 11: 0 → 1
Char. 106: 0 → 1
Char. 108: 1 → 0
Char. 109: 0 → 1
Char. 128: 0 → 1
Char. 175: 0 → 1
Char. 202: 1 → 0
Char. 207: 1 → 0
Char. 209: 1 → 0
Char. 244: 0 → 1
Char. 248: 0 → 1
Char. 250: 0 → 1
Char. 252: 1 → 0
Char. 262: 0 → 1

***Pappochelys rosinae*:**
Char. 1: 0 → 1
Char. 5: 1 → 0
Char. 12: 0 → 1
Char. 23: 0 → 1
Char. 41: 0 → 1
Char. 49: 0 → 1
Char. 50: 1 → 0
Char. 75: 0 → 1
Char. 129: 0 → 1
Char. 169: 1 → 0
Char. 260: 2 → 0
Char. 265: 0 → 1

***Odontochelys semitestacea*:**
Char. 43: 0 → 1

***Eunotosaurus africanus*:**
Char. 19: 0 → 1
Char. 43: 0 → 1
Char. 76: 0 → 1
Char. 97: 1 → 0
Char. 103: 0 → 1
Char. 153: 1 → 0
Char. 192: 1 → 0
Char. 202: 1 → 0
Char. 211: 0 → 1
Char. 219: 0 → 1
Char. 248: 0 → 1
Char. 249: 0 → 2
Char. 250: 0 → 1
Char. 263: 0 → 1
Char. 273: 0 → 1
Char. 274: 0 → 1
Char. 276: 0 → 1
Char. 277: 0 → 1

***Acerosodontosaurus piveteaui*:**
Char. 23: 0 → 1
Char. 50: 1 → 0
Char. 59: 0 → 1
Char. 78: 1 → 0
Char. 81: 1 → 0
Char. 89: 0 → 1
Char. 94: 0 → 1
Char. 128: 0 → 1
Char. 129: 0 → 1
Char. 155: 0 → 1
Char. 206: 0 → 2
Char. 208: 0 → 1
Char. 265: 0 → 1
Char. 266: 0 → 1
Char. 278: 3 → 1

***Acleistorhinus pteroticus*:**
Char. 21: 0 → 1
Char. 146: 1 → 0
Char. 169: 1 → 0
Char. 170: 0 → 1

**Araeoscelidae:**
Char. 0: 0 → 1
Char. 5: 1 → 0
Char. 20: 1 → 0
Char. 23: 0 → 1
Char. 27: 0 → 1
Char. 28: 0 → 1
Char. 38: 1 → 0
Char. 40: 2 → 0
Char. 43: 0 → 1
Char. 50: 1 → 0
Char. 59: 0 → 1
Char. 60: 0 → 1
Char. 67: 0 → 1
Char. 72: 1 → 2
Char. 84: 1 → 0
Char. 89: 0 → 1
Char. 106: 0 → 1
Char. 116: 1 → 0
Char. 117: 0 → 2
Char. 154: 1 → 0
Char. 159: 1 → 0
Char. 166: 1 → 0
Char. 169: 1 → 0
Char. 170: 0 → 1
Char. 193: 0 → 1
Char. 197: 0 → 1
Char. 202: 1 → 0
Char. 207: 1 → 0
Char. 209: 1 → 0
Char. 221: 1 → 0
Char. 224: 0 → 1
Char. 239: 0 → 1
Char. 266: 0 → 1
Char. 278: 3 → 1

**Archosauriformes:**
Char. 32: 0 → 1
Char. 94: 1 → 0
Char. 112: 1 → 0
Char. 152: 0 → 1
Char. 154: 0 → 2
Char. 166: 1 → 0
Char. 171: 0 → 1
Char. 185: 0 → 1
Char. 204: 0 → 1
Char. 218: 0 → 3
Char. 224: 0 → 1
Char. 242: 0 → 1

***Australothyris smithi*:**
Char. 23: 0 → 1
Char. 24: 0 → 1
Char. 29: 0 → 1
Char. 34: 0 → 1
Char. 55: 1 → 0
Char. 71: 1 → 0
Char. 79: 0 → 1
Char. 83: 0 → 1
Char. 85: 1 → 0
Char. 98: 1 → 0
Char. 100: 0 → 1
Char. 103: 0 → 1
Char. 110: 0 → 1
Char. 111: 1 → 0
Char. 123: 1 → 0
Char. 129: 0 → 1
Char. 131: 0 → 1
Char. 132: 1 → 0
Char. 144: 1 → 0
Char. 147: 0 → 1
Char. 149: 1 → 0
Char. 150: 0 → 1
Char. 159: 1 → 0
Char. 163: 1 → 0
Char. 192: 1 → 0

***Barasaurus besairiei*:**
Char. 33: 1 → 0
Char. 75: 1 → 0
Char. 216: 0 → 1

***Bashkyroleter bashkyricus*:**
Char. 275: 1 → 0

***Bashkyroleter mesensis*:**
Char. 169: 1 → 0

***Belebey vegrandis*:**
Char. 154: 1 → 0

***Bradysaurus* spp.:**
Char. 19: 0 → 1
Char. 73: 0 → 1
Char. 79: 0 → 1
Char. 135: 1 → 0
Char. 249: 0 → 1

***Candelaria barbouri*:**
Char. 1: 0 → 1
Char. 5: 1 → 0
Char. 8: 0 → 1
Char. 15: 0 → 1
Char. 23: 0 → 1
Char. 25: 1 → 0
Char. 33: 01 → 2
Char. 49: 0 → 1
Char. 50: 1 → 0
Char. 55: 1 → 0
Char. 67: 0 → 1
Char. 76: 0 → 1
Char. 79: 0 → 2
Char. 83: 0 → 1
Char. 88: 1 → 0
Char. 89: 0 → 1
Char. 94: 0 → 1
Char. 95: 0 → 1
Char. 126: 0 → 1
Char. 127: 0 → 1
Char. 132: 1 → 0
Char. 154: 1 → 2
Char. 159: 1 → 0
Char. 169: 1 → 0
Char. 276: 0 → 1
Char. 277: 0 → 1

**Captorhinidae:**
Char. 3: 0 → 1
Char. 23: 0 → 1
Char. 25: 1 → 0
Char. 26: 0 → 1
Char. 73: 0 → 1
Char. 75: 0 → 1
Char. 83: 0 → 1
Char. 108: 1 → 0
Char. 180: 1 → 0
Char. 183: 1 → 0
Char. 201: 1 → 0
Char. 203: 1 → 2
Char. 216: 1 → 0
Char. 240: 1 → 0

**Caseidae:**
Char. 24: 0 → 1
Char. 25: 1 → 0
Char. 36: 0 → 1
Char. 38: 1 → 0
Char. 46: 1 → 0
Char. 50: 1 → 0
Char. 56: 0 → 1
Char. 170: 0 → 1
Char. 194: 0 → 1
Char. 273: 0 → 1
Char. 274: 0 → 1
Char. 278: 3 → 2

***Claudiosaurus germaini*:**
Char. 24: 0 → 1
Char. 27: 0 → 1
Char. 34: 0 → 1
Char. 36: 0 → 1
Char. 64: 0 → 1
Char. 84: 1 → 0
Char. 105: 0 → 1
Char. 130: 0 → 1
Char. 144: 1 → 0
Char. 166: 1 → 0
Char. 199: 0 → 1
Char. 203: 1 → 2
Char. 204: 0 → 1
Char. 220: 0 → 1

***Colobomycter pholeter*:**
Char. 21: 0 → 1
Char. 25: 1 → 0
Char. 84: 1 → 0
Char. 154: 1 → 0
Char. 167: 0 → 1
Char. 267: 0 → 1

***Delorhynchus cifelli*:**
Char. 18: 0 → 1
Char. 21: 0 → 1
Char. 24: 0 → 1
Char. 26: 0 → 1
Char. 28: 0 → 1
Char. 29: 0 → 1
Char. 33: 01 → 2
Char. 39: 0 → 1
Char. 52: 0 → 1
Char. 57: 0 → 1
Char. 100: 0 → 1
Char. 116: 1 → 0
Char. 117: 0 → 1
Char. 119: 1 → 0
Char. 131: 0 → 1
Char. 147: 0 → 1
Char. 156: 1 → 0
Char. 167: 0 → 1
Char. 189: 0 → 1
Char. 191: 0 → 1
Char. 202: 1 → 0
Char. 204: 0 → 2
Char. 267: 0 → 1

**Diadectomorpha:**
Char. 0: 0 → 1
Char. 64: 0 → 1
Char. 70: 0 → 1
Char. 122: 0 → 1
Char. 123: 1 → 0
Char. 146: 1 → 0
Char. 275: 1 → 0
Char. 278: 3 → 0

***Emeroleter levis*:**
Char. 0: 0 → 2
Char. 51: 1 → 0

**Eosauropterygia:**
Char. 159: 1 → 0
Char. 166: 1 → 0
Char. 174: 0 → 1
Char. 194: 0 → 2
Char. 272: 1 → 0

***Eudibamus cursoris*:**
Char. 154: 1 → 2
Char. 163: 1 → 0

***Feeserpeton oklahomensis*:**
Char. 51: 0 → 1
Char. 70: 0 → 1
Char. 157: 0 → 1
Char. 158: 0 → 1

***Hovasaurus boulei*:**
Char. 41: 0 → 1
Char. 43: 0 → 1
Char. 50: 1 → 0
Char. 55: 1 → 0
Char. 59: 0 → 1
Char. 67: 0 → 1
Char. 77: 0 → 2
Char. 78: 1 → 0
Char. 79: 0 → 1
Char. 89: 0 → 1
Char. 93: 1 → 0
Char. 113: 0 → 1
Char. 127: 0 → 1
Char. 135: 0 → 1
Char. 136: 0 → 1
Char. 138: 0 → 1
Char. 146: 1 → 0
Char. 187: 0 → 1
Char. 188: 0 → 1
Char. 191: 0 → 1
Char. 193: 0 → 1
Char. 196: 0 → 1
Char. 201: 1 → 0
Char. 204: 0 → 2
Char. 206: 0 → 2
Char. 215: 0 → 1
Char. 220: 0 → 1
Char. 224: 0 → 1
Char. 265: 0 → 1
Char. 275: 1 → 0

**Kuehneosauridae:**
Char. 7: 0 → 1
Char. 24: 0 → 1
Char. 26: 0 → 1
Char. 27: 0 → 1
Char. 34: 0 → 1
Char. 36: 0 → 1
Char. 44: 1 → 0
Char. 79: 0 → 2
Char. 82: 0 → 1
Char. 98: 1 → 0
Char. 108: 1 → 0
Char. 113: 0 → 1
Char. 128: 0 → 1
Char. 148: 1 → 0
Char. 159: 1 → 0
Char. 181: 0 → 1
Char. 185: 0 → 1
Char. 206: 0 → 2
Char. 245: 0 → 1
Char. 272: 1 → 2
Char. 278: 0 → 3

***Lanthanosuchus watsoni*:**
Char. 25: 1 → 0
Char. 51: 0 → 1
Char. 76: 0 → 1
Char. 86: 0 → 1
Char. 98: 1 → 0
Char. 119: 1 → 0
Char. 138: 0 → 1
Char. 144: 1 → 0
Char. 154: 1 → 2

***Macroleter poezicus*:**
Char. 0: 0 → 1
Char. 52: 0 → 1
Char. 66: 1 → 2
Char. 84: 1 → 0
Char. 87: 0 → 1
Char. 140: 0 → 1
Char. 146: 1 → 0
Char. 169: 1 → 0
Char. 235: 0 → 1

***Mesosaurus* spp.:**
Char. 0: 0 → 1
Char. 2: 0 → 1
Char. 5: 1 → 0
Char. 6: 0 → 1
Char. 8: 0 → 1
Char. 9: 0 → 1
Char. 13: 0 → 1
Char. 19: 0 → 1
Char. 20: 1 → 0
Char. 23: 0 → 1
Char. 26: 0 → 1
Char. 38: 1 → 0
Char. 41: 0 → 1
Char. 50: 1 → 0
Char. 67: 0 → 1
Char. 76: 0 → 1
Char. 83: 0 → 1
Char. 84: 1 → 0
Char. 85: 1 → 0
Char. 94: 0 → 1
Char. 107: 0 → 1
Char. 109: 0 → 1
Char. 115: 0 → 1
Char. 146: 1 → 0
Char. 148: 0 → 1
Char. 149: 1 → 0
Char. 159: 1 → 0
Char. 163: 1 → 0
Char. 164: 0 → 1
Char. 166: 1 → 0
Char. 167: 0 → 1
Char. 176: 0 → 1
Char. 183: 1 → 0
Char. 184: 0 → 1
Char. 192: 1 → 0
Char. 199: 0 → 1
Char. 204: 0 → 2
Char. 206: 0 → 1
Char. 217: 0 → 1
Char. 219: 0 → 1
Char. 220: 0 → 1
Char. 231: 0 → 1
Char. 234: 1 → 0
Char. 240: 1 → 0
Char. 260: 2 → 0
Char. 272: 2 → 0
Char. 278: 3 → 0

***Microleter mckinzieorum*:**
Char. 0: 0 → 1
Char. 18: 0 → 1
Char. 20: 1 → 0
Char. 24: 0 → 1
Char. 25: 1 → 0
Char. 29: 0 → 1
Char. 36: 0 → 1
Char. 39: 0 → 1
Char. 51: 0 → 2
Char. 56: 0 → 1
Char. 70: 0 → 1
Char. 76: 0 → 1
Char. 79: 0 → 1
Char. 83: 0 → 1
Char. 94: 0 → 1
Char. 106: 0 → 1
Char. 110: 0 → 1
Char. 111: 1 → 0
Char. 132: 1 → 0
Char. 166: 1 → 0
Char. 276: 0 → 1
Char. 278: 3 → 1

**Millerettidae:**
Char. 5: 1 → 0
Char. 20: 1 → 0
Char. 24: 0 → 1
Char. 25: 1 → 0
Char. 29: 0 → 1
Char. 44: 1 → 0
Char. 56: 0 → 1
Char. 66: 0 → 2
Char. 78: 1 → 0
Char. 80: 1 → 0
Char. 84: 1 → 2
Char. 88: 1 → 0
Char. 96: 1 → 0
Char. 111: 1 → 0
Char. 117: 0 → 1
Char. 120: 1 → 0
Char. 121: 0 → 1
Char. 124: 0 → 1
Char. 127: 0 → 1
Char. 135: 0 → 1
Char. 145: 0 → 1
Char. 159: 1 → 0
Char. 163: 1 → 0
Char. 166: 1 → 0
Char. 180: 1 → 0
Char. 192: 1 → 0
Char. 211: 0 → 1
Char. 230: 0 → 1
Char. 248: 0 → 1
Char. 252: 0 → 1

***Nycteroleter ineptus*:**
Char. 278: 0 → 3

***Nyctiphruretus acudens*:**
Char. 0: 0 → 1
Char. 21: 0 → 1
Char. 33: 1 → 2
Char. 41: 0 → 1
Char. 66: 1 → 2
Char. 81: 1 → 0
Char. 83: 0 → 1
Char. 84: 1 → 2
Char. 94: 0 → 1
Char. 166: 1 → 0
Char. 167: 0 → 1
Char. 224: 0 → 1
Char. 266: 0 → 1
Char. 272: 2 → 1
Char. 276: 0 → 1

***Orovenator mayorum*:**
Char. 0: 0 → 1
Char. 8: 0 → 1
Char. 24: 0 → 1
Char. 25: 1 → 0
Char. 36: 0 → 1
Char. 62: 0 → 1
Char. 72: 1 → 2
Char. 89: 0 → 1
Char. 136: 0 → 1
Char. 141: 0 → 1
Char. 159: 1 → 0
Char. 160: 0 → 1
Char. 165: 0 → 1
Char. 278: 3 → 1

***Owenetta* spp.:**
Char. 169: 1 → 0

***Paleothyris acadiana*:**
Char. 38: 1 → 0
Char. 50: 1 → 0
Char. 66: 1 → 2
Char. 102: 0 → 1
Char. 146: 1 → 0
Char. 239: 0 → 1

***Placodus* spp.:**
Char. 0: 1 → 2
Char. 9: 0 → 1
Char. 12: 0 → 1
Char. 13: 0 → 1
Char. 19: 0 → 1
Char. 26: 0 → 1
Char. 31: 0 → 1
Char. 44: 1 → 0
Char. 46: 1 → 0
Char. 57: 0 → 1
Char. 78: 1 → 0
Char. 93: 1 → 0
Char. 102: 1 → 2
Char. 109: 1 → 0
Char. 140: 1 → 0
Char. 155: 0 → 1
Char. 163: 1 → 0
Char. 164: 0 → 1

***Procolophon* spp.:**
Char. 41: 0 → 1
Char. 69: 0 → 1
Char. 79: 0 → 1
Char. 83: 0 → 1
Char. 88: 0 → 1
Char. 117: 1 → 0
Char. 149: 1 → 0
Char. 204: 0 → 1
Char. 237: 0 → 1
Char. 238: 0 → 1
Char. 272: 2 → 1
Char. 278: 3 → 0

***Prolacerta broomi*:**
Char. 58: 1 → 0
Char. 66: 1 → 0
Char. 67: 1 → 0
Char. 80: 1 → 0
Char. 139: 1 → 0
Char. 192: 1 → 0
Char. 203: 1 → 2
Char. 206: 0 → 12

***Rhipaeosaurus* spp.:**
Char. 172: 0 → 1
Char. 277: 0 → 1

**Rhynchocephalia:**
Char. 0: 1 → 2
Char. 24: 0 → 1
Char. 77: 0 → 1
Char. 94: 1 → 0
Char. 117: 12 → 0
Char. 139: 1 → 0
Char. 167: 1 → 0
Char. 205: 1 → 0

**Rhynchosauria:**
Char. 0: 1 → 0
Char. 7: 0 → 1
Char. 9: 0 → 1
Char. 26: 0 → 1
Char. 44: 1 → 0
Char. 68: 0 → 1
Char. 99: 1 → 0
Char. 150: 1 → 0
Char. 160: 0 → 1
Char. 161: 0 → 1
Char. 171: 0 → 2
Char. 182: 1 → 0
Char. 223: 0 → 1
Char. 224: 0 → 1
Char. 241: 0 → 1

***Scutosaurus* spp.:**
Char. 175: 0 → 1
Char. 218: 0 → 1
Char. 243: 0 → 2
Char. 244: 0 → 1
Char. 251: 0 → 1

***Sinosaurosphargis yunguiensis*:**
Char. 8: 0 → 1
Char. 30: 0 → 1

Char. 53: 0 → 1
Char. 82: 0 → 1
Char. 89: 1 → 0
Char. 127: 1 → 0
Char. 150: 1 → 0
Char. 154: 0 → 2
Char. 167: 1 → 0
Char. 255: 0 → 1

**Squamata:**
Char. 26: 0 → 1
Char. 45: 0 → 1
Char. 79: 0 → 2
Char. 80: 1 → 0
Char. 82: 0 → 1
Char. 92: 1 → 0
Char. 109: 1 → 0
Char. 160: 0 → 1
Char. 245: 0 → 1

***Trilophosaurus buettneri*:**
Char. 5: 1 → 0
Char. 11: 0 → 1
Char. 55: 1 → 0
Char. 93: 1 → 0
Char. 104: 0 → 1
Char. 113: 0 → 1
Char. 122: 0 → 1
Char. 144: 1 → 0
Char. 154: 0 → 1
Char. 157: 0 → 1
Char. 159: 1 → 0
Char. 177: 0 → 12
Char. 194: 0 → 1
Char. 203: 1 → 2
Char. 207: 1 → 0
Char. 208: 1 → 0
Char. 272: 1 → 0

***Youngina capensis*:**
Char. 0: 0 → 1
Char. 5: 1 → 0
Char. 21: 0 → 1
Char. 27: 0 → 1
Char. 38: 1 → 0
Char. 40: 2 → 0
Char. 43: 0 → 1
Char. 44: 1 → 0
Char. 50: 1 → 0
Char. 56: 0 → 1
Char. 59: 0 → 1
Char. 60: 0 → 1
Char. 62: 0 → 1
Char. 67: 0 → 1
Char. 72: 1 → 2
Char. 75: 0 → 1
Char. 84: 1 → 0
Char. 89: 0 → 1
Char. 109: 0 → 1
Char. 127: 0 → 1
Char. 129: 0 → 1
Char. 134: 0 → 1
Char. 135: 0 → 1
Char. 141: 0 → 1
Char. 154: 1 → 0
Char. 163: 1 → 0
Char. 170: 0 → 1
Char. 179: 0 → 1
Char. 187: 0 → 1
Char. 191: 0 → 1
Char. 193: 0 → 1
Char. 196: 0 → 1
Char. 201: 1 → 0
Char. 211: 0 → 1
Char. 214: 0 → 1
Char. 215: 0 → 1
Char. 219: 0 → 1
Char. 224: 0 → 1
Char. 231: 0 → 1
Char. 239: 0 → 1
Char. 265: 0 → 1
Char. 267: 0 → 1
Char. 275: 1 → 0
Char. 278: 3 → 0

**Node 50:**
Char. 46: 1 → 0
Char. 88: 1 → 0
Char. 93: 1 → 0
Char. 176: 0 → 1
Char. 195: 0 → 2
Char. 198: 0 → 1
Char. 246: 1 → 2
Char. 259: 0 → 1
Char. 270: 0 → 1

**Node 51:**
Char. 65: 0 → 1
Char. 72: 1 → 2
Char. 131: 0 → 1
Char. 184: 0 → 1
Char. 205: 0 → 1
Char. 210: 0 → 1
Char. 241: 0 → 1
Char. 246: 0 → 1
Char. 254: 0 → 1
Char. 255: 0 → 1
Char. 256: 0 → 1
Char. 267: 0 → 1
Char. 268: 0 → 1
Char. 269: 0 → 1
Char. 278: 3 → 0

**Node 52:**
Char. 15: 0 → 1
Char. 21: 0 → 1
Char. 25: 1 → 0
Char. 33: 01 → 2
Char. 44: 1 → 0
Char. 64: 0 → 1
Char. 73: 0 → 1
Char. 84: 1 → 2
Char. 94: 0 → 1
Char. 127: 0 → 1
Char. 130: 0 → 1
Char. 134: 0 → 2
Char. 135: 0 → 1
Char. 136: 0 → 1
Char. 141: 0 → 1
Char. 145: 0 → 1
Char. 147: 0 → 1
Char. 148: 0 → 1
Char. 151: 0 → 1
Char. 152: 0 → 1
Char. 155: 0 → 1
Char. 158: 0 → 1
Char. 161: 0 → 1
Char. 174: 0 → 1
Char. 181: 0 → 1
Char. 188: 0 → 1
Char. 196: 0 → 1
Char. 201: 1 → 0
Char. 203: 1 → 2
Char. 204: 0 → 2
Char. 217: 0 → 1
Char. 220: 0 → 1
Char. 231: 0 → 1
Char. 247: 0 → 1
Char. 251: 0 → 1
Char. 252: 0 → 1

**Node 53:**
Char. 5: 0 → 1
Char. 20: 0 → 1
Char. 40: 0 → 2
Char. 57: 1 → 0
Char. 74: 0 → 1
Char. 80: 0 → 1
Char. 84: 0 → 1
Char. 88: 0 → 1
Char. 116: 0 → 1
Char. 119: 0 → 1
Char. 132: 0 → 1
Char. 149: 0 → 1
Char. 156: 0 → 1
Char. 163: 0 → 1
Char. 166: 0 → 1
Char. 169: 0 → 1
Char. 180: 0 → 1
Char. 192: 0 → 1
Char. 201: 0 → 1
Char. 202: 0 → 1
Char. 203: 0 → 1
Char. 209: 0 → 1
Char. 221: 0 → 1
Char. 234: 0 → 1
Char. 235: 0 → 1

**Node 54:**
Char. 72: 0 → 1
Char. 79: 1 → 0
Char. 81: 0 → 1
Char. 93: 0 → 1
Char. 97: 0 → 1
Char. 104: 1 → 0
Char. 144: 0 → 1
Char. 173: 0 → 1
Char. 183: 0 → 1

**Node 56:**
Char. 29: 0 → 1
Char. 95: 0 → 1
Char. 110: 0 → 1
Char. 111: 1 → 0
Char. 113: 0 → 1
Char. 114: 0 → 1
Char. 131: 0 → 1
Char. 137: 0 → 1
Char. 140: 0 → 2
Char. 147: 0 → 1

**Node 57:**
Char. 19: 0 → 1
Char. 92: 1 → 0

**Node 58:**
Char. 4: 0 → 1
Char. 15: 0 → 1
Char. 29: 0 → 1
Char. 213: 0 → 1
Char. 226: 0 → 2
Char. 228: 0 → 1

**Node 59:**
Char. 58: 0 → 1
Char. 61: 0 → 1
Char. 66: 0 → 1
Char. 69: 0 → 1
Char. 127: 0 → 1
Char. 141: 0 → 1
Char. 150: 0 → 1
Char. 167: 0 → 1
Char. 205: 0 → 1
Char. 208: 0 → 1
Char. 230: 0 → 1
Char. 239: 0 → 1

**Node 60:**
Char. 0: 0 → 1
Char. 23: 0 → 1
Char. 50: 1 → 0
Char. 59: 0 → 1
Char. 60: 0 → 1
Char. 62: 0 → 1
Char. 67: 0 → 1
Char. 70: 0 → 1
Char. 72: 1 → 2
Char. 73: 0 → 1
Char. 89: 0 → 1
Char. 94: 0 → 1
Char. 109: 0 → 1
Char. 117: 0 → 12
Char. 126: 0 → 1
Char. 129: 0 → 1
Char. 131: 0 → 1
Char. 148: 0 → 1
Char. 154: 1 → 0
Char. 179: 0 → 1
Char. 182: 0 → 1
Char. 188: 0 → 1
Char. 190: 0 → 1
Char. 191: 0 → 1
Char. 196: 0 → 1
Char. 214: 0 → 1
Char. 219: 0 → 1
Char. 265: 0 → 1
Char. 267: 0 → 1
Char. 272: 2 → 1
Char. 278: 3 → 0

**Node 61:**
Char. 73: 0 → 1
Char. 131: 1 → 0
Char. 205: 0 → 1
Char. 239: 0 → 1
Char. 276: 0 → 1

**Node 62:**
Char. 18: 0 → 1
Char. 37: 0 → 1
Char. 107: 0 → 1
Char. 118: 1 → 0
Char. 125: 1 → 0
Char. 150: 0 → 1

**Node 63:**
Char. 23: 0 → 1
Char. 71: 1 → 0
Char. 102: 0 → 1
Char. 103: 0 → 1
Char. 106: 0 → 1
Char. 167: 0 → 1
Char. 214: 0 → 1
Char. 216: 1 → 0
Char. 235: 0 → 2
Char. 241: 0 → 1

**Node 64:**
Char. 138: 0 → 1
Char. 141: 0 → 1

**Node 65:**
Char. 29: 0 → 1
Char. 38: 1 → 2
Char. 39: 0 → 1
Char. 44: 1 → 0
Char. 49: 0 → 1
Char. 51: 0 → 1
Char. 57: 0 → 1
Char. 66: 0 → 1
Char. 70: 0 → 1
Char. 76: 0 → 1
Char. 80: 1 → 0
Char. 88: 1 → 0
Char. 95: 0 → 1
Char. 111: 1 → 0
Char. 117: 0 → 1
Char. 118: 0 → 1
Char. 120: 1 → 0
Char. 125: 0 → 1
Char. 131: 0 → 1
Char. 137: 0 → 1
Char. 147: 0 → 1
Char. 148: 0 → 1
Char. 158: 0 → 1

Char. 183: 1 → 2
Char. 194: 0 → 1
Char. 201: 1 → 0
Char. 211: 0 → 1
Char. 234: 1 → 0
Char. 235: 1 → 0
Char. 240: 1 → 0
Char. 252: 0 → 1

**Node 66:**
Char. 87: 0 → 1

**Node 67:**
Char. 25: 1 → 0
Char. 76: 1 → 0

**Node 68:**
Char. 79: 0 → 1
Char. 93: 1 → 0
Char. 133: 0 → 1

**Node 69:**
Char. 147: 1 → 2
Char. 154: 1 → 0
Char. 239: 0 → 1
Char. 278: 3 → 0

**Node 70:**
Char. 20: 1 → 0
Char. 38: 1 → 2
Char. 39: 0 → 1
Char. 50: 1 → 0
Char. 57: 0 → 1
Char. 58: 0 → 1
Char. 59: 0 → 1
Char. 60: 0 → 1
Char. 72: 1 → 2
Char. 83: 0 → 1
Char. 85: 1 → 0
Char. 88: 1 → 0
Char. 95: 0 → 1
Char. 104: 0 → 1
Char. 105: 0 → 1
Char. 106: 0 → 2
Char. 107: 0 → 1
Char. 109: 0 → 1
Char. 110: 0 → 1
Char. 146: 1 → 0
Char. 148: 0 → 1
Char. 155: 0 → 2
Char. 183: 1 → 2

**Node 71:**
Char. 0: 0 → 2
Char. 33: 1 → 0
Char. 38: 2 → 1
Char. 39: 1 → 0
Char. 42: 0 → 1
Char. 43: 0 → 1
Char. 46: 1 → 0
Char. 49: 1 → 0
Char. 52: 0 → 1
Char. 83: 0 → 2
Char. 84: 1 → 0
Char. 87: 0 → 1
Char. 93: 1 → 0
Char. 161: 0 → 1
Char. 163: 1 → 0
Char. 172: 0 → 2
Char. 174: 0 → 1
Char. 188: 0 → 1
Char. 189: 0 → 1
Char. 195: 0 → 1
Char. 204: 0 → 2
Char. 212: 0 → 1
Char. 221: 1 → 0
Char. 236: 0 → 1
Char. 238: 0 → 2
Char. 242: 0 → 1
Char. 245: 0 → 1
Char. 274: 0 → 1
Char. 275: 1 → 0

**Node 72:**
Char. 5: 1 → 0
Char. 20: 1 → 0
Char. 44: 1 → 0
Char. 59: 0 → 1
Char. 60: 0 → 1
Char. 66: 0 → 1
Char. 67: 0 → 1
Char. 72: 1 → 2
Char. 78: 1 → 0
Char. 84: 1 → 0
Char. 88: 1 → 0
Char. 93: 1 → 0
Char. 123: 1 → 0
Char. 129: 0 → 1
Char. 154: 1 → 0
Char. 159: 1 → 0
Char. 163: 1 → 0
Char. 169: 1 → 0
Char. 170: 0 → 1
Char. 192: 1 → 0
Char. 197: 0 → 1
Char. 202: 1 → 0
Char. 207: 1 → 0
Char. 209: 1 → 0
Char. 221: 1 → 0
Char. 234: 1 → 0

**Node 73:**
Char. 29: 0 → 1
Char. 47: 0 → 1
Char. 57: 0 → 1
Char. 83: 0 → 1
Char. 85: 1 → 0
Char. 107: 0 → 1
Char. 110: 0 → 1
Char. 111: 1 → 0
Char. 169: 1 → 0
Char. 170: 0 → 1

**Node 74:**
Char. 2: 0 → 1
Char. 6: 0 → 1
Char. 98: 1 → 0
Char. 101: 0 → 1
Char. 113: 0 → 1
Char. 181: 0 → 1
Char. 186: 0 → 1
Char. 198: 0 → 1
Char. 206: 0 → 2
Char. 220: 0 → 1
Char. 239: 1 → 0
Char. 240: 1 → 0

**Node 75:**
Char. 21: 0 → 1
Char. 33: 1 → 2
Char. 68: 0 → 1
Char. 102: 0 → 1
Char. 103: 0 → 1
Char. 223: 0 → 1
Char. 229: 0 → 1
Char. 235: 1 → 2

**Node 76:**
Char. 17: 0 → 1
Char. 42: 0 → 1
Char. 251: 0 → 1
Char. 264: 0 → 1

**Node 77:**
Char. 41: 0 → 1
Char. 61: 1 → 2
Char. 112: 1 → 0
Char. 128: 0 → 1
Char. 138: 0 → 1
Char. 146: 1 → 0
Char. 155: 0 → 1
Char. 182: 1 → 0
Char. 192: 1 → 2
Char. 224: 0 → 1
Char. 226: 0 → 1
Char. 227: 0 → 1
Char. 233: 0 → 1

**Node 78:**
Char. 61: 1 → 3
Char. 90: 0 → 1
Char. 91: 0 → 1
Char. 109: 1 → 0
Char. 155: 0 → 1
Char. 209: 1 → 0

**ANALYSIS 72**
**(ALL TAXA, IMPLIED WEIGHTING, K = 2.75, NO CHARACTER 253)**

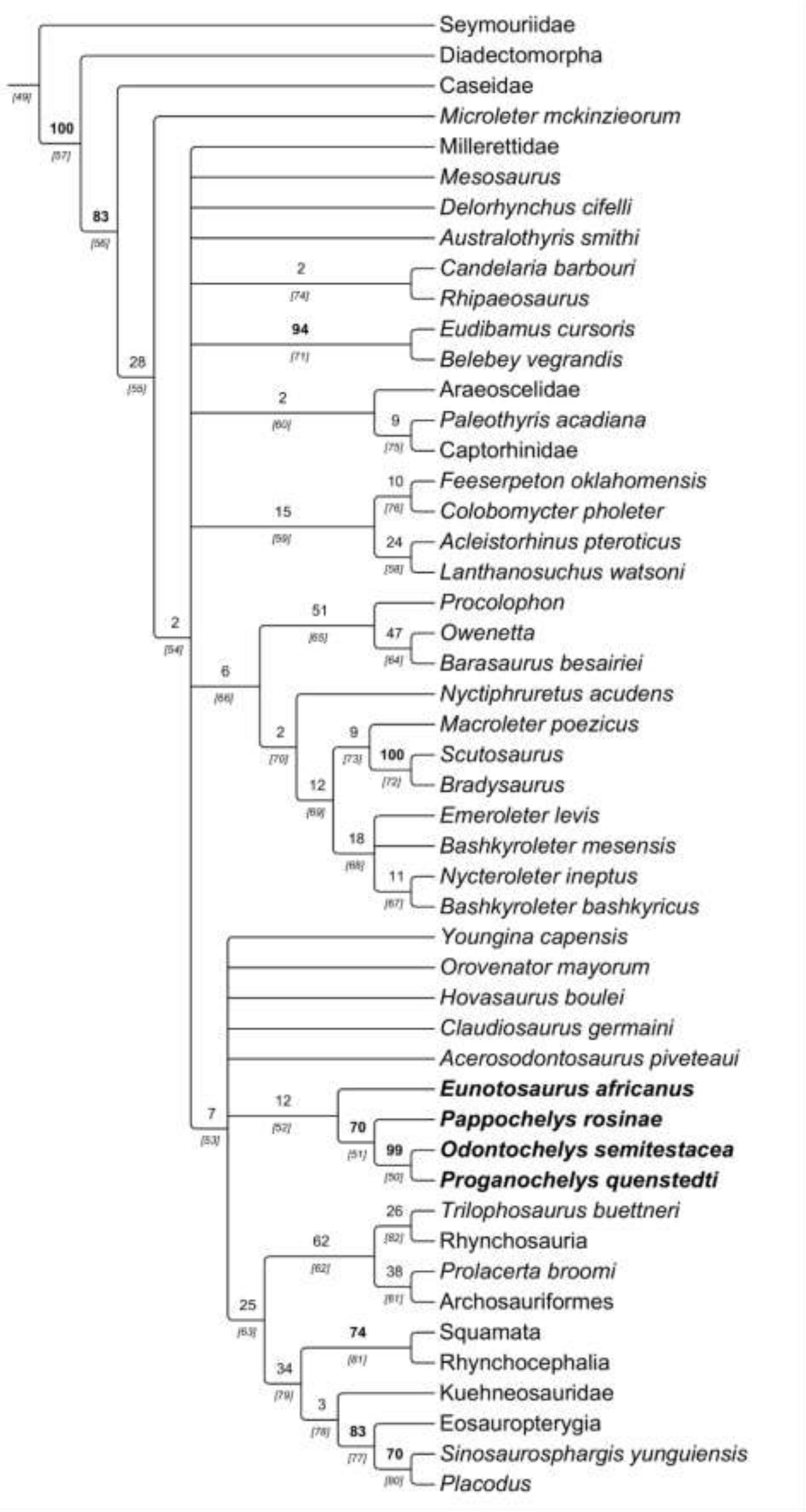

***Proganochelys quenstedti*:**
Char. 8: 0 → 1
Char. 11: 0 → 1
Char. 106: 0 → 1
Char. 108: 1 → 0
Char. 128: 0 → 1
Char. 175: 0 → 1
Char. 202: 1 → 0
Char. 207: 1 → 0
Char. 209: 1 → 0
Char. 244: 0 → 1
Char. 248: 0 → 1
Char. 250: 0 → 1
Char. 252: 1 → 0
Char. 262: 0 → 1

***Pappochelys rosinae*:**
Char. 0: 2 → 0
Char. 1: 0 → 1
Char. 5: 1 → 0
Char. 12: 0 → 1
Char. 41: 0 → 1
Char. 49: 0 → 1
Char. 75: 0 → 1
Char. 169: 1 → 0
Char. 260: 2 → 0

***Eunotosaurus africanus*:**
Char. 19: 0 → 1
Char. 59: 1 → 0
Char. 60: 1 → 0
Char. 72: 2 → 1
Char. 76: 0 → 1
Char. 97: 1 → 0
Char. 103: 0 → 1
Char. 153: 1 → 0
Char. 191: 1 → 0
Char. 192: 1 → 0
Char. 202: 1 → 0
Char. 211: 0 → 1
Char. 222: 1 → 0
Char. 237: 1 → 0
Char. 248: 0 → 1
Char. 249: 0 → 2
Char. 250: 0 → 1
Char. 263: 0 → 1
Char. 273: 0 → 1
Char. 274: 0 → 1
Char. 275: 0 → 1
Char. 276: 0 → 1
Char. 277: 0 → 1
Char. 278: 0 → 3

***Acerosodontosaurus piveteaui*:**
Char. 78: 1 → 0
Char. 81: 1 → 0
Char. 127: 1 → 0
Char. 128: 0 → 1
Char. 155: 0 → 1
Char. 206: 0 → 2
Char. 208: 0 → 1
Char. 278: 0 → 1

***Acleistorhinus pteroticus:***
Char. 21: 0 → 1
Char. 146: 1 → 0

**Araeoscelidae:**
Char. 0: 0 → 1
Char. 28: 0 → 1
Char. 43: 0 → 1
Char. 89: 0 → 1
Char. 106: 0 → 1
Char. 120: 0 → 1
Char. 193: 0 → 1
Char. 222: 0 → 1
Char. 224: 0 → 1
Char. 234: 0 → 1
Char. 266: 0 → 1
Char. 278: 3 → 1

**Archosauriformes:**
Char. 32: 0 → 1
Char. 94: 1 → 0
Char. 112: 1 → 0
Char. 152: 0 → 1
Char. 154: 01 → 2
Char. 166: 1 → 0
Char. 171: 0 → 1
Char. 185: 0 → 1
Char. 201: 0 → 1
Char. 204: 0 → 1
Char. 218: 0 → 3
Char. 224: 0 → 1
Char. 242: 0 → 1

***Australothyris smithi*:**
Char. 24: 0 → 1
Char. 34: 0 → 1
Char. 55: 1 → 0
Char. 71: 1 → 0
Char. 79: 0 → 1
Char. 85: 1 → 0
Char. 98: 1 → 0
Char. 100: 0 → 1
Char. 103: 0 → 1
Char. 110: 0 → 1
Char. 123: 1 → 0
Char. 129: 0 → 1
Char. 131: 0 → 1
Char. 132: 1 → 0
Char. 144: 1 → 0
Char. 147: 0 → 1
Char. 149: 1 → 0
Char. 150: 0 → 1
Char. 159: 1 → 0
Char. 192: 1 → 0

***Barasaurus besairiei*:**
Char. 33: 1 → 0

***Bashkyroleter bashkyricus*:**
Char. 275: 1 → 0

***Bashkyroleter mesensis*:**
Char. 12: 0 → 1
Char. 66: 1 → 0
Char. 169: 1 → 0

***Belebey vegrandis*:**
Char. 154: 1 → 0

***Bradysaurus* spp.:**
Char. 19: 0 → 1
Char. 25: 1 → 0
Char. 73: 0 → 1
Char. 79: 0 → 1
Char. 135: 1 → 0
Char. 249: 0 → 1

***Candelaria barbouri*:**
Char. 89: 0 → 1
Char. 159: 1 → 0

**Captorhinidae:**
Char. 3: 0 → 1
Char. 25: 1 → 0
Char. 26: 0 → 1
Char. 73: 0 → 1
Char. 75: 0 → 1
Char. 108: 1 → 0
Char. 180: 1 → 0
Char. 183: 1 → 0
Char. 201: 1 → 0
Char. 203: 1 → 2
Char. 216: 1 → 0

**Caseidae:**
Char. 38: 1 → 0
Char. 46: 1 → 0
Char. 170: 0 → 1
Char. 194: 0 → 1
Char. 273: 0 → 1
Char. 274: 0 → 1
Char. 278: 3 → 2

***Claudiosaurus germaini*:**
Char. 24: 0 → 1
Char. 27: 0 → 1
Char. 34: 0 → 1
Char. 36: 0 → 1
Char. 56: 0 → 1
Char. 64: 0 → 1
Char. 70: 0 → 1
Char. 84: 1 → 0
Char. 105: 0 → 1
Char. 106: 0 → 1
Char. 117: 01 → 2
Char. 126: 0 → 1
Char. 127: 1 → 0
Char. 130: 0 → 1
Char. 131: 0 → 1
Char. 141: 1 → 0
Char. 144: 1 → 0
Char. 166: 1 → 0
Char. 182: 0 → 1
Char. 187: 1 → 0
Char. 190: 0 → 1
Char. 199: 0 → 1
Char. 201: 0 → 1
Char. 203: 1 → 2
Char. 204: 02 → 1
Char. 222: 1 → 0
Char. 234: 1 → 0
Char. 272: 2 → 1

***Colobomycter pholeter*:**
Char. 21: 0 → 1
Char. 25: 1 → 0
Char. 84: 1 → 0
Char. 154: 1 → 0
Char. 167: 0 → 1
Char. 267: 0 → 1

***Delorhynchus cifelli*:**
Char. 18: 0 → 1
Char. 21: 0 → 1
Char. 23: 1 → 0
Char. 24: 0 → 1
Char. 26: 0 → 1
Char. 28: 0 → 1
Char. 33: 1 → 2
Char. 39: 0 → 1
Char. 52: 0 → 1
Char. 100: 0 → 1
Char. 111: 0 → 1
Char. 116: 1 → 0
Char. 119: 1 → 0
Char. 131: 0 → 1
Char. 147: 0 → 1
Char. 156: 1 → 0
Char. 167: 0 → 1
Char. 189: 0 → 1
Char. 191: 0 → 1
Char. 267: 0 → 1

**Diadectomorpha:**
Char. 0: 0 → 1
Char. 64: 0 → 1
Char. 70: 0 → 1
Char. 122: 0 → 1
Char. 123: 1 → 0
Char. 146: 1 → 0
Char. 275: 1 → 0
Char. 278: 3 → 0

***Emeroleter levis*:**
Char. 51: 1 → 0

**Eosauropterygia:**
Char. 166: 1 → 0
Char. 174: 0 → 1
Char. 178: 0 → 1
Char. 194: 0 → 2
Char. 272: 1 → 0

***Eudibamus cursoris*:**
Char. 154: 1 → 2

***Feeserpeton oklahomensis*:**
Char. 51: 0 → 1
Char. 157: 0 → 1
Char. 158: 0 → 1

***Hovasaurus boulei*:**
Char. 41: 0 → 1
Char. 55: 1 → 0
Char. 60: 1 → 0
Char. 72: 2 → 1
Char. 77: 0 → 2
Char. 78: 1 → 0
Char. 79: 0 → 1
Char. 93: 1 → 0
Char. 113: 0 → 1
Char. 138: 0 → 1
Char. 141: 1 → 0
Char. 146: 1 → 0
Char. 206: 0 → 2
Char. 215: 0 → 1
Char. 219: 1 → 0
Char. 224: 0 → 1
Char. 278: 0 → 3

**Kuehneosauridae:**
Char. 7: 0 → 1
Char. 24: 0 → 1
Char. 27: 0 → 1
Char. 43: 1 → 0
Char. 79: 0 → 2
Char. 107: 1 → 0
Char. 140: 1 → 0
Char. 147: 1 → 0
Char. 148: 1 → 0
Char. 245: 0 → 1
Char. 278: 0 → 3

***Lanthanosuchus watsoni*:**
Char. 25: 1 → 0
Char. 51: 0 → 1
Char. 86: 0 → 1
Char. 98: 1 → 0
Char. 138: 0 → 1
Char. 144: 1 → 0
Char. 154: 1 → 2

***Macroleter poezicus*:**
Char. 9: 0 → 1
Char. 26: 0 → 1
Char. 66: 1 → 2
Char. 126: 0 → 1
Char. 134: 0 → 2
Char. 140: 0 → 1
Char. 146: 1 → 0
Char. 169: 1 → 0

***Mesosaurus* spp.:**
Char. 0: 0 → 1
Char. 2: 0 → 1
Char. 6: 0 → 1
Char. 8: 0 → 1
Char. 9: 0 → 1
Char. 13: 0 → 1
Char. 19: 0 → 1
Char. 26: 0 → 1

Char. 29: 1 → 0
Char. 38: 1 → 0
Char. 41: 0 → 1
Char. 67: 0 → 1
Char. 84: 1 → 0
Char. 85: 1 → 0
Char. 94: 0 → 1
Char. 107: 0 → 1
Char. 109: 0 → 1
Char. 111: 0 → 1
Char. 115: 0 → 1
Char. 146: 1 → 0
Char. 149: 1 → 0
Char. 159: 1 → 0
Char. 164: 0 → 1
Char. 166: 1 → 0
Char. 167: 0 → 1
Char. 176: 0 → 1
Char. 183: 1 → 0
Char. 184: 0 → 1
Char. 192: 1 → 0
Char. 199: 0 → 1
Char. 202: 0 → 1
Char. 206: 0 → 1
Char. 209: 0 → 1
Char. 217: 0 → 1
Char. 219: 0 → 1
Char. 220: 0 → 1
Char. 231: 0 → 1
Char. 260: 2 → 0
Char. 272: 2 → 0
Char. 278: 3 → 0

***Microleter mckinzieorum*:**
Char. 0: 0 → 1
Char. 18: 0 → 1
Char. 39: 0 → 1
Char. 70: 0 → 1
Char. 94: 0 → 1
Char. 106: 0 → 1
Char. 110: 0 → 1
Char. 278: 3 → 1

**Millerettidae:**
Char. 23: 1 → 0
Char. 24: 0 → 1
Char. 25: 1 → 0
Char. 33: 1 → 0
Char. 44: 1 → 0
Char. 56: 0 → 1
Char. 66: 0 → 2
Char. 78: 1 → 0
Char. 80: 1 → 0
Char. 84: 1 → 2
Char. 88: 1 → 0
Char. 96: 1 → 0
Char. 121: 0 → 1
Char. 124: 0 → 1
Char. 127: 0 → 1
Char. 135: 0 → 1
Char. 145: 0 → 1
Char. 159: 1 → 0
Char. 166: 1 → 0
Char. 180: 1 → 0
Char. 192: 1 → 0
Char. 202: 0 → 1
Char. 211: 0 → 1
Char. 230: 0 → 1
Char. 234: 0 → 1
Char. 248: 0 → 1
Char. 252: 0 → 1

***Nycteroleter ineptus*:**
Char. 66: 1 → 0
Char. 278: 0 → 3

***Nyctiphruretus acudens*:**
Char. 21: 0 → 1
Char. 41: 0 → 1
Char. 66: 1 → 2
Char. 81: 1 → 0
Char. 84: 1 → 2
Char. 94: 0 → 1
Char. 166: 1 → 0
Char. 224: 0 → 1
Char. 266: 0 → 1
Char. 272: 2 → 1
Char. 276: 0 → 1

***Orovenator mayorum*:**
Char. 8: 0 → 1
Char. 23: 1 → 0
Char. 24: 0 → 1
Char. 33: 1 → 0
Char. 36: 0 → 1
Char. 50: 0 → 1
Char. 92: 1 → 0
Char. 94: 1 → 0
Char. 135: 1 → 0
Char. 159: 1 → 0
Char. 160: 0 → 1
Char. 165: 0 → 1
Char. 278: 0 → 1

***Owenetta* spp.:**
Char. 142: 1 → 0
Char. 169: 1 → 0

***Paleothyris acadiana*:**
Char. 23: 1 → 0
Char. 66: 1 → 2
Char. 102: 0 → 1
Char. 146: 1 → 0

***Placodus* spp.:**
Char. 0: 1 → 2
Char. 9: 0 → 1
Char. 12: 0 → 1
Char. 13: 0 → 1
Char. 19: 0 → 1
Char. 31: 0 → 1
Char. 46: 1 → 0
Char. 57: 0 → 1
Char. 78: 1 → 0
Char. 93: 1 → 0
Char. 102: 1 → 2
Char. 109: 1 → 0
Char. 140: 1 → 0
Char. 155: 0 → 1
Char. 163: 1 → 0
Char. 164: 0 → 1

***Procolophon* spp.:**
Char. 41: 0 → 1
Char. 69: 0 → 1
Char. 79: 0 → 1
Char. 86: 0 → 1
Char. 101: 0 → 1
Char. 141: 0 → 1
Char. 149: 1 → 0
Char. 203: 1 → 2
Char. 215: 0 → 1
Char. 230: 0 → 1
Char. 235: 1 → 2
Char. 237: 0 → 1
Char. 238: 0 → 1
Char. 272: 2 → 1
Char. 278: 3 → 0

***Prolacerta broomi*:**
Char. 58: 1 → 0
Char. 66: 1 → 0
Char. 67: 1 → 0
Char. 80: 1 → 0
Char. 139: 1 → 0
Char. 147: 1 → 0
Char. 192: 1 → 0
Char. 203: 1 → 2
Char. 206: 0 → 12

***Rhipaeosaurus* spp.:**
Char. 172: 0 → 1

**Rhynchocephalia:**
Char. 0: 1 → 2
Char. 23: 1 → 0
Char. 24: 0 → 1
Char. 77: 0 → 1
Char. 88: 1 → 0
Char. 94: 1 → 0
Char. 139: 1 → 0
Char. 167: 1 → 0
Char. 205: 1 → 0

**Rhynchosauria:**
Char. 0: 1 → 0
Char. 7: 0 → 1
Char. 9: 0 → 1
Char. 26: 0 → 1
Char. 44: 1 → 0
Char. 68: 0 → 1
Char. 99: 1 → 0
Char. 150: 1 → 0
Char. 160: 0 → 1
Char. 161: 0 → 1
Char. 171: 0 → 2
Char. 223: 0 → 1
Char. 224: 0 → 1
Char. 241: 0 → 1

***Scutosaurus* spp.:**
Char. 175: 0 → 1
Char. 190: 0 → 1
Char. 218: 0 → 1
Char. 243: 0 → 2
Char. 244: 0 → 1
Char. 251: 0 → 1

***Sinosaurosphargis yunguiensis*:**
Char. 8: 0 → 1
Char. 30: 0 → 1
Char. 53: 0 → 1
Char. 89: 1 → 0
Char. 127: 1 → 0
Char. 150: 1 → 0
Char. 154: 0 → 2
Char. 167: 1 → 0
Char. 255: 0 → 1

**Squamata:**
Char. 45: 0 → 1
Char. 79: 0 → 2
Char. 80: 1 → 0
Char. 92: 1 → 0
Char. 109: 1 → 0
Char. 160: 0 → 1
Char. 200: 0 → 1
Char. 245: 0 → 1

***Trilophosaurus buettneri*:**
Char. 5: 1 → 0
Char. 11: 0 → 1
Char. 55: 1 → 0
Char. 93: 1 → 0
Char. 104: 0 → 1
Char. 106: 0 → 1
Char. 113: 0 → 1
Char. 122: 0 → 1
Char. 136: 1 → 0
Char. 144: 1 → 0
Char. 157: 0 → 1
Char. 159: 1 → 0
Char. 177: 0 → 12
Char. 194: 0 → 1
Char. 203: 1 → 2
Char. 207: 1 → 0
Char. 208: 1 → 0
Char. 272: 1 → 0

***Youngina capensis*:**
Char. 5: 1 → 0
Char. 23: 1 → 0
Char. 25: 0 → 1
Char. 27: 0 → 1
Char. 38: 1 → 0
Char. 44: 1 → 0
Char. 56: 0 → 1
Char. 75: 0 → 1
Char. 84: 1 → 0
Char. 92: 1 → 0
Char. 94: 1 → 0
Char. 134: 0 → 1
Char. 163: 1 → 0
Char. 170: 0 → 1
Char. 211: 0 → 1
Char. 215: 0 → 1
Char. 224: 0 → 1
Char. 239: 0 → 1

**Node 50:**
Char. 46: 1 → 0
Char. 88: 1 → 0
Char. 89: 1 → 0
Char. 93: 1 → 0
Char. 176: 0 → 1
Char. 195: 0 → 2
Char. 198: 0 → 1
Char. 246: 1 → 2
Char. 259: 0 → 1
Char. 265: 1 → 0
Char. 270: 0 → 1

**Node 51:**
Char. 65: 0 → 1
Char. 131: 0 → 1
Char. 184: 0 → 1
Char. 205: 0 → 1
Char. 210: 0 → 1
Char. 219: 1 → 0
Char. 241: 0 → 1
Char. 246: 0 → 1
Char. 254: 0 → 1
Char. 255: 0 → 1
Char. 256: 0 → 1
Char. 268: 0 → 1
Char. 269: 0 → 1

**Node 52:**
Char. 0: 1 → 2
Char. 15: 0 → 1
Char. 33: 1 → 2
Char. 44: 1 → 0
Char. 62: 1 → 0
Char. 64: 0 → 1
Char. 84: 1 → 2
Char. 130: 0 → 1
Char. 134: 0 → 2
Char. 147: 0 → 1
Char. 152: 0 → 1
Char. 155: 0 → 1
Char. 158: 0 → 1
Char. 161: 0 → 1
Char. 174: 0 → 1
Char. 181: 0 → 1
Char. 203: 1 → 2
Char. 247: 0 → 1
Char. 251: 0 → 1
Char. 252: 0 → 1

**Node 53:**
Char. 0: 0 → 1
Char. 25: 1 → 0
Char. 29: 1 → 0
Char. 40: 2 → 0
Char. 59: 0 → 1

Char. 60: 0 → 1
Char. 62: 0 → 1
Char. 67: 0 → 1
Char. 72: 1 → 2
Char. 89: 0 → 1
Char. 92: 0 → 1
Char. 94: 0 → 1
Char. 109: 0 → 1
Char. 111: 0 → 1
Char. 120: 0 → 1
Char. 127: 0 → 1
Char. 129: 0 → 1
Char. 135: 0 → 1
Char. 136: 0 → 1
Char. 139: 0 → 1
Char. 141: 0 → 1
Char. 179: 0 → 1
Char. 187: 0 → 1
Char. 188: 0 → 1
Char. 191: 0 → 1
Char. 193: 0 → 1
Char. 196: 0 → 1
Char. 201: 1 → 0
Char. 202: 0 → 1
Char. 209: 0 → 1
Char. 219: 0 → 1
Char. 222: 0 → 1
Char. 234: 0 → 1
Char. 237: 0 → 1
Char. 265: 0 → 1
Char. 266: 0 → 1
Char. 275: 1 → 0
Char. 278: 3 → 0

**Node 54:**
Char. 23: 0 → 1
Char. 33: 0 → 1
Char. 132: 0 → 1
Char. 166: 0 → 1
Char. 276: 1 → 0

**Node 55:**
Char. 29: 0 → 1
Char. 84: 0 → 1
Char. 88: 0 → 1
Char. 169: 0 → 1

**Node 56:**
Char. 72: 0 → 1
Char. 81: 0 → 1
Char. 93: 0 → 1
Char. 97: 0 → 1
Char. 104: 1 → 0
Char. 144: 0 → 1
Char. 173: 0 → 1
Char. 183: 0 → 1

**Node 58:**
Char. 95: 0 → 1
Char. 113: 0 → 1
Char. 114: 0 → 1

**Node 59:**
Char. 23: 1 → 0
Char. 47: 0 → 1
Char. 79: 0 → 1
Char. 110: 0 → 1
Char. 131: 0 → 1
Char. 137: 0 → 1
Char. 140: 0 → 2
Char. 147: 0 → 1

**Node 60:**
Char. 29: 1 → 0
Char. 33: 1 → 0
Char. 59: 0 → 1
Char. 60: 0 → 1
Char. 67: 0 → 1
Char. 72: 1 → 2
Char. 84: 1 → 0
Char. 111: 0 → 1
Char. 116: 1 → 0
Char. 154: 1 → 0
Char. 159: 1 → 0
Char. 166: 1 → 0
Char. 169: 1 → 0
Char. 170: 0 → 1
Char. 197: 0 → 1
Char. 221: 1 → 0

**Node 61:**
Char. 19: 0 → 1
Char. 92: 1 → 0

**Node 62:**
Char. 4: 0 → 1
Char. 15: 0 → 1
Char. 29: 0 → 1
Char. 213: 0 → 1
Char. 226: 0 → 2
Char. 228: 0 → 1
Char. 275: 0 → 1

**Node 63:**
Char. 58: 0 → 1
Char. 61: 0 → 1
Char. 66: 0 → 1
Char. 69: 0 → 1
Char. 107: 0 → 1
Char. 126: 0 → 1
Char. 131: 0 → 1
Char. 134: 0 → 1
Char. 140: 0 → 1
Char. 147: 0 → 1
Char. 150: 0 → 1
Char. 167: 0 → 1
Char. 176: 0 → 1
Char. 190: 0 → 1
Char. 205: 0 → 1
Char. 208: 0 → 1
Char. 230: 0 → 1
Char. 239: 0 → 1
Char. 260: 2 → 1

**Node 64:**
Char. 73: 0 → 1
Char. 205: 0 → 1
Char. 273: 0 → 1
Char. 276: 0 → 1

**Node 65:**
Char. 18: 0 → 1
Char. 25: 1 → 0
Char. 37: 0 → 1
Char. 103: 0 → 1
Char. 106: 0 → 1
Char. 107: 0 → 1
Char. 110: 0 → 1
Char. 126: 0 → 1
Char. 150: 0 → 1

**Node 66:**
Char. 38: 1 → 2
Char. 39: 0 → 1
Char. 44: 1 → 0
Char. 49: 0 → 1
Char. 66: 0 → 1
Char. 80: 1 → 0
Char. 95: 0 → 1
Char. 135: 0 → 1
Char. 137: 0 → 1
Char. 147: 0 → 1
Char. 157: 0 → 1
Char. 158: 0 → 1
Char. 183: 1 → 2
Char. 186: 0 → 1
Char. 187: 0 → 1
Char. 194: 0 → 1
Char. 197: 0 → 1
Char. 201: 1 → 0
Char. 211: 0 → 1
Char. 252: 0 → 1

**Node 67:**
Char. 87: 0 → 1
Char. 110: 0 → 1

**Node 68:**
Char. 79: 0 → 1
Char. 133: 0 → 1

**Node 69:**
Char. 100: 0 → 1
Char. 113: 0 → 1

**Node 70:**
Char. 23: 1 → 0
Char. 70: 0 → 1
Char. 118: 0 → 1
Char. 125: 0 → 1

**Node 71:**
Char. 38: 1 → 2
Char. 39: 0 → 1
Char. 58: 0 → 1
Char. 59: 0 → 1
Char. 60: 0 → 1
Char. 72: 1 → 2
Char. 85: 1 → 0
Char. 88: 1 → 0
Char. 95: 0 → 1
Char. 104: 0 → 1
Char. 105: 0 → 1
Char. 106: 0 → 2
Char. 107: 0 → 1
Char. 109: 0 → 1
Char. 110: 0 → 1
Char. 146: 1 → 0
Char. 155: 0 → 2
Char. 183: 1 → 2

**Node 72:**
Char. 23: 0 → 1
Char. 33: 1 → 0
Char. 38: 2 → 1
Char. 39: 1 → 0
Char. 42: 0 → 1
Char. 43: 0 → 1
Char. 46: 1 → 0
Char. 49: 1 → 0
Char. 71: 1 → 0
Char. 83: 0 → 2
Char. 101: 0 → 1
Char. 103: 0 → 1
Char. 106: 0 → 1
Char. 161: 0 → 1
Char. 163: 1 → 0
Char. 172: 0 → 2
Char. 174: 0 → 1
Char. 188: 0 → 1
Char. 203: 1 → 2
Char. 212: 0 → 1
Char. 216: 1 → 0
Char. 221: 1 → 0
Char. 236: 0 → 1
Char. 238: 0 → 2
Char. 241: 0 → 1
Char. 242: 0 → 1
Char. 273: 0 → 1
Char. 274: 0 → 1
Char. 275: 1 → 0

**Node 73:**
Char. 52: 0 → 1
Char. 75: 0 → 1
Char. 84: 1 → 0
Char. 87: 0 → 1
Char. 110: 0 → 1
Char. 121: 0 → 1
Char. 180: 1 → 0
Char. 230: 0 → 1

**Node 74:**
Char. 277: 0 → 1

**Node 75:**
Char. 44: 1 → 0
Char. 66: 0 → 1
Char. 78: 1 → 0
Char. 88: 1 → 0
Char. 93: 1 → 0
Char. 123: 1 → 0
Char. 129: 0 → 1
Char. 192: 1 → 0

**Node 76:**
Char. 33: 1 → 0
Char. 85: 1 → 0
Char. 107: 0 → 1

**Node 77:**
Char. 2: 0 → 1
Char. 6: 0 → 1
Char. 186: 0 → 1
Char. 198: 0 → 1
Char. 200: 0 → 1
Char. 220: 0 → 1
Char. 239: 1 → 0

**Node 78:**
Char. 98: 1 → 0
Char. 113: 0 → 1
Char. 181: 0 → 1
Char. 206: 0 → 2

**Node 79:**
Char. 35: 0 → 1
Char. 102: 0 → 1
Char. 103: 0 → 1
Char. 184: 0 → 1
Char. 201: 0 → 1
Char. 210: 0 → 1
Char. 223: 0 → 1
Char. 229: 0 → 1

**Node 80:**
Char. 17: 0 → 1
Char. 42: 0 → 1
Char. 251: 0 → 1
Char. 264: 0 → 1

**Node 81:**
Char. 41: 0 → 1
Char. 61: 1 → 2
Char. 112: 1 → 0
Char. 138: 0 → 1
Char. 146: 1 → 0
Char. 155: 0 → 1
Char. 192: 1 → 2
Char. 224: 0 → 1
Char. 226: 0 → 1
Char. 227: 0 → 1
Char. 233: 0 → 1
Char. 267: 01 → 2

**Node 82:**
Char. 61: 1 → 3
Char. 90: 0 → 1
Char. 91: 0 → 1
Char. 109: 1 → 0
Char. 155: 0 → 1
Char. 209: 1 → 0

**ANALYSIS 73**
**(ALL TAXA, NO CHARACTERS 247 AND 253)**

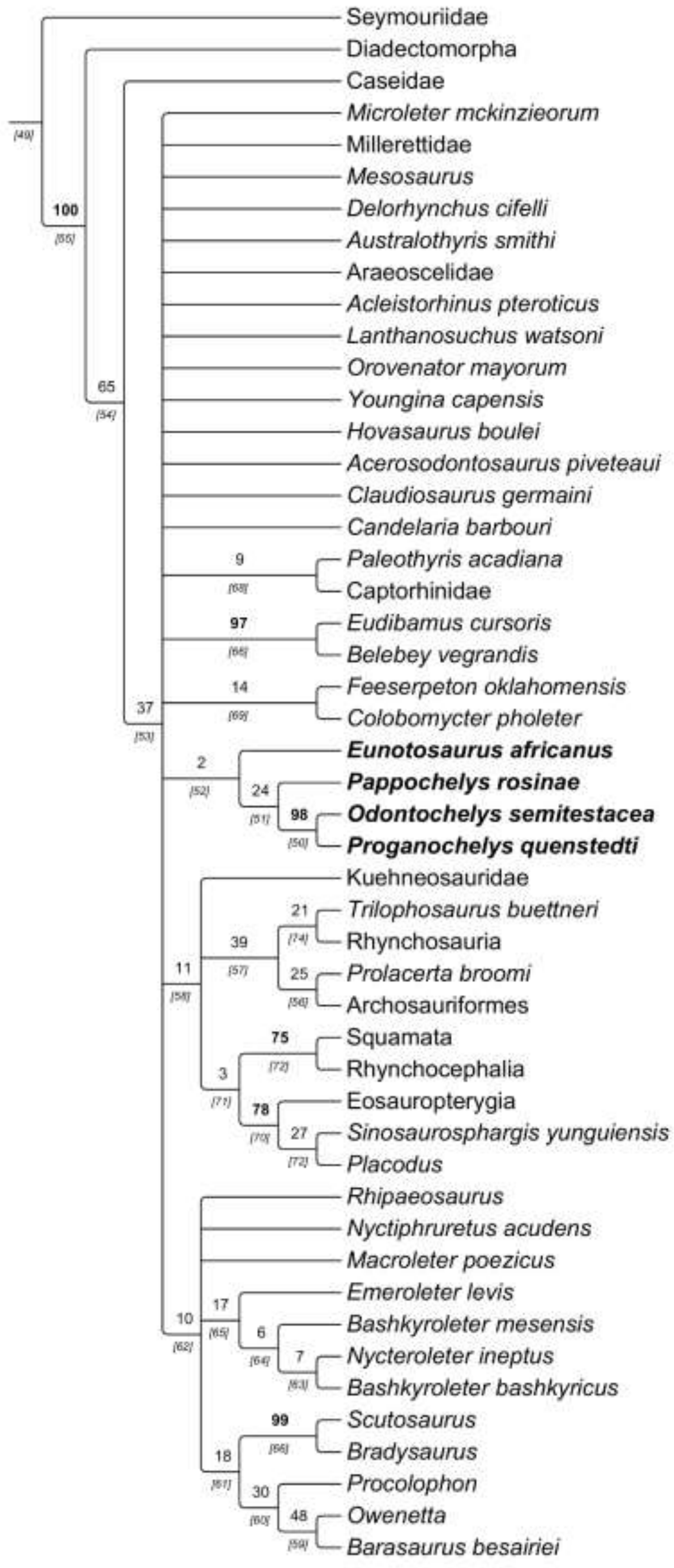

***Proganochelys quenstedti*:**
Char. 8: 0 → 1
Char. 11: 0 → 1
Char. 106: 0 → 1
Char. 108: 1 → 0
Char. 109: 0 → 1
Char. 128: 0 → 1
Char. 175: 0 → 1
Char. 202: 1 → 0
Char. 207: 1 → 0
Char. 209: 1 → 0
Char. 244: 0 → 1
Char. 248: 0 → 1
Char. 250: 0 → 1
Char. 252: 1 → 0
Char. 262: 0 → 1

***Pappochelys rosinae*:**
Char. 1: 0 → 1
Char. 5: 1 → 0
Char. 12: 0 → 1
Char. 23: 0 → 1
Char. 41: 0 → 1
Char. 49: 0 → 1
Char. 50: 1 → 0
Char. 75: 0 → 1
Char. 129: 0 → 1
Char. 169: 1 → 0
Char. 260: 2 → 0
Char. 265: 0 → 1

***Odontochelys semitestacea*:**
Char. 43: 0 → 1

***Eunotosaurus africanus*:**
Char. 19: 0 → 1
Char. 43: 0 → 1
Char. 76: 0 → 1
Char. 97: 1 → 0
Char. 103: 0 → 1
Char. 153: 1 → 0
Char. 192: 1 → 0
Char. 202: 1 → 0
Char. 211: 0 → 1
Char. 219: 0 → 1
Char. 237: 1 → 0
Char. 248: 0 → 1
Char. 249: 0 → 2
Char. 250: 0 → 1
Char. 263: 0 → 1
Char. 273: 0 → 1
Char. 274: 0 → 1
Char. 276: 0 → 1
Char. 277: 0 → 1

***Acerosodontosaurus piveteaui*:**
Char. 23: 0 → 1
Char. 50: 1 → 0
Char. 59: 0 → 1
Char. 78: 1 → 0
Char. 81: 1 → 0
Char. 89: 0 → 1
Char. 94: 0 → 1
Char. 128: 0 → 1
Char. 129: 0 → 1
Char. 155: 0 → 1
Char. 206: 0 → 2
Char. 208: 0 → 1
Char. 265: 0 → 1
Char. 266: 0 → 1
Char. 278: 3 → 1

***Acleistorhinus pteroticus:***
Char. 21: 0 → 1
Char. 29: 0 → 1
Char. 30: 0 → 1
Char. 47: 0 → 1
Char. 55: 1 → 0
Char. 56: 0 → 1
Char. 57: 0 → 1
Char. 64: 0 → 1
Char. 70: 0 → 1
Char. 79: 0 → 1
Char. 95: 0 → 1
Char. 110: 0 → 1
Char. 111: 1 → 0
Char. 112: 1 → 0
Char. 113: 0 → 1
Char. 114: 0 → 1
Char. 120: 1 → 0
Char. 131: 0 → 1
Char. 137: 0 → 1
Char. 140: 0 → 2
Char. 146: 1 → 0
Char. 147: 0 → 1
Char. 169: 1 → 0
Char. 170: 0 → 1
Char. 278: 3 → 1

**Araeoscelidae:**
Char. 5: 1 → 0
Char. 20: 1 → 0
Char. 23: 0 → 1
Char. 27: 0 → 1
Char. 28: 0 → 1
Char. 33: 1 → 0
Char. 38: 1 → 0
Char. 40: 2 → 0
Char. 43: 0 → 1
Char. 50: 1 → 0
Char. 59: 0 → 1
Char. 60: 0 → 1
Char. 72: 1 → 2
Char. 84: 1 → 0
Char. 89: 0 → 1
Char. 92: 1 → 0
Char. 106: 0 → 1
Char. 116: 1 → 0
Char. 117: 0 → 2
Char. 154: 1 → 0
Char. 159: 1 → 0
Char. 166: 1 → 0
Char. 169: 1 → 0
Char. 170: 0 → 1
Char. 193: 0 → 1
Char. 197: 0 → 1
Char. 202: 1 → 0
Char. 207: 1 → 0
Char. 209: 1 → 0
Char. 221: 1 → 0
Char. 224: 0 → 1
Char. 239: 0 → 1
Char. 266: 0 → 1
Char. 278: 3 → 1

**Archosauriformes:**
Char. 32: 0 → 1
Char. 94: 1 → 0
Char. 112: 1 → 0
Char. 152: 0 → 1
Char. 154: 0 → 2
Char. 166: 1 → 0
Char. 171: 0 → 1
Char. 185: 0 → 1
Char. 204: 0 → 1
Char. 218: 0 → 3
Char. 224: 0 → 1
Char. 242: 0 → 1

***Australothyris smithi*:**
Char. 23: 0 → 1
Char. 24: 0 → 1
Char. 29: 0 → 1
Char. 34: 0 → 1
Char. 55: 1 → 0
Char. 71: 1 → 0
Char. 79: 0 → 1
Char. 83: 0 → 1
Char. 85: 1 → 0
Char. 98: 1 → 0
Char. 100: 0 → 1
Char. 103: 0 → 1
Char. 110: 0 → 1
Char. 111: 1 → 0
Char. 123: 1 → 0
Char. 129: 0 → 1
Char. 131: 0 → 1
Char. 132: 1 → 0
Char. 144: 1 → 0
Char. 147: 0 → 1
Char. 149: 1 → 0
Char. 150: 0 → 1
Char. 159: 1 → 0
Char. 163: 1 → 0
Char. 192: 1 → 0

***Barasaurus besairiei*:**
Char. 33: 1 → 0
Char. 75: 1 → 0
Char. 216: 0 → 1

***Bashkyroleter bashkyricus*:**
Char. 275: 1 → 0

***Bashkyroleter mesensis*:**
Char. 169: 1 → 0

***Belebey vegrandis*:**
Char. 154: 1 → 0

***Bradysaurus* spp.:**
Char. 19: 0 → 1
Char. 73: 0 → 1
Char. 79: 0 → 1
Char. 135: 1 → 0
Char. 249: 0 → 1

***Candelaria barbouri*:**
Char. 1: 0 → 1
Char. 5: 1 → 0
Char. 8: 0 → 1
Char. 15: 0 → 1
Char. 23: 0 → 1
Char. 25: 1 → 0
Char. 33: 1 → 2
Char. 48: 1 → 0
Char. 49: 0 → 1
Char. 50: 1 → 0
Char. 55: 1 → 0
Char. 76: 0 → 1
Char. 79: 0 → 2
Char. 83: 0 → 1
Char. 88: 1 → 0
Char. 89: 0 → 1
Char. 92: 1 → 0
Char. 94: 0 → 1
Char. 95: 0 → 1
Char. 126: 0 → 1
Char. 127: 0 → 1
Char. 132: 1 → 0
Char. 154: 1 → 2
Char. 159: 1 → 0
Char. 169: 1 → 0
Char. 276: 0 → 1
Char. 277: 0 → 1

**Captorhinidae:**
Char. 3: 0 → 1
Char. 23: 0 → 1
Char. 25: 1 → 0
Char. 26: 0 → 1
Char. 73: 0 → 1
Char. 75: 0 → 1
Char. 83: 0 → 1
Char. 108: 1 → 0
Char. 180: 1 → 0
Char. 183: 1 → 0
Char. 201: 1 → 0
Char. 203: 1 → 2
Char. 216: 1 → 0
Char. 237: 1 → 0
Char. 240: 1 → 0

**Caseidae:**
Char. 24: 0 → 1
Char. 25: 1 → 0
Char. 36: 0 → 1
Char. 38: 1 → 0
Char. 46: 1 → 0
Char. 50: 1 → 0
Char. 56: 0 → 1
Char. 170: 0 → 1
Char. 194: 0 → 1
Char. 273: 0 → 1
Char. 274: 0 → 1
Char. 278: 3 → 2

***Claudiosaurus germaini*:**
Char. 23: 0 → 1
Char. 24: 0 → 1
Char. 27: 0 → 1
Char. 34: 0 → 1
Char. 36: 0 → 1
Char. 50: 1 → 0
Char. 56: 0 → 1
Char. 59: 0 → 1
Char. 60: 0 → 1
Char. 62: 0 → 1
Char. 64: 0 → 1
Char. 70: 0 → 1
Char. 72: 1 → 2
Char. 73: 0 → 1
Char. 84: 1 → 0
Char. 89: 0 → 1
Char. 94: 0 → 1
Char. 105: 0 → 1
Char. 106: 0 → 1
Char. 109: 0 → 1
Char. 117: 0 → 2
Char. 126: 0 → 1
Char. 129: 0 → 1
Char. 130: 0 → 1
Char. 131: 0 → 1
Char. 144: 1 → 0
Char. 148: 0 → 1
Char. 154: 1 → 0
Char. 166: 1 → 0
Char. 182: 0 → 1
Char. 188: 0 → 1
Char. 190: 0 → 1
Char. 191: 0 → 1
Char. 199: 0 → 1
Char. 203: 1 → 2
Char. 204: 0 → 1
Char. 214: 0 → 1
Char. 219: 0 → 1
Char. 220: 0 → 1
Char. 234: 1 → 0
Char. 265: 0 → 1
Char. 267: 0 → 1
Char. 272: 2 → 1
Char. 275: 1 → 0
Char. 278: 3 → 0

***Colobomycter pholeter*:**
Char. 21: 0 → 1
Char. 25: 1 → 0
Char. 84: 1 → 0
Char. 154: 1 → 0
Char. 167: 0 → 1
Char. 267: 0 → 1

***Delorhynchus cifelli*:**
Char. 18: 0 → 1
Char. 21: 0 → 1
Char. 24: 0 → 1
Char. 26: 0 → 1
Char. 28: 0 → 1
Char. 29: 0 → 1
Char. 33: 1 → 2
Char. 39: 0 → 1
Char. 52: 0 → 1
Char. 57: 0 → 1
Char. 100: 0 → 1
Char. 116: 1 → 0
Char. 117: 0 → 1
Char. 119: 1 → 0
Char. 131: 0 → 1
Char. 147: 0 → 1
Char. 156: 1 → 0
Char. 167: 0 → 1
Char. 189: 0 → 1
Char. 191: 0 → 1
Char. 202: 1 → 0
Char. 204: 0 → 2
Char. 267: 0 → 1

**Diadectomorpha:**
Char. 64: 0 → 1
Char. 70: 0 → 1
Char. 122: 0 → 1
Char. 123: 1 → 0
Char. 146: 1 → 0
Char. 275: 1 → 0
Char. 278: 3 → 0

***Emeroleter levis*:**
Char. 51: 1 → 0

**Eosauropterygia:**
Char. 159: 1 → 0
Char. 166: 1 → 0
Char. 174: 0 → 1
Char. 194: 0 → 2
Char. 272: 1 → 0

***Eudibamus cursoris*:**
Char. 154: 1 → 2
Char. 163: 1 → 0

***Feeserpeton oklahomensis*:**
Char. 51: 0 → 1
Char. 70: 0 → 1
Char. 157: 0 → 1
Char. 158: 0 → 1

***Hovasaurus boulei*:**
Char. 41: 0 → 1
Char. 43: 0 → 1
Char. 50: 1 → 0
Char. 55: 1 → 0
Char. 59: 0 → 1
Char. 77: 0 → 2
Char. 78: 1 → 0
Char. 79: 0 → 1
Char. 89: 0 → 1
Char. 93: 1 → 0
Char. 113: 0 → 1
Char. 127: 0 → 1
Char. 135: 0 → 1
Char. 136: 0 → 1
Char. 138: 0 → 1
Char. 146: 1 → 0
Char. 187: 0 → 1
Char. 188: 0 → 1
Char. 191: 0 → 1
Char. 193: 0 → 1
Char. 201: 1 → 0
Char. 204: 0 → 2
Char. 206: 0 → 2
Char. 215: 0 → 1
Char. 220: 0 → 1
Char. 224: 0 → 1
Char. 265: 0 → 1
Char. 275: 1 → 0

**Kuehneosauridae:**
Char. 7: 0 → 1
Char. 24: 0 → 1
Char. 26: 0 → 1
Char. 27: 0 → 1
Char. 34: 0 → 1
Char. 36: 0 → 1
Char. 44: 1 → 0
Char. 56: 0 → 1
Char. 79: 0 → 2
Char. 82: 0 → 1
Char. 98: 1 → 0
Char. 108: 1 → 0
Char. 113: 0 → 1
Char. 128: 0 → 1
Char. 159: 1 → 0
Char. 181: 0 → 1
Char. 185: 0 → 1
Char. 206: 0 → 2
Char. 245: 0 → 1

***Lanthanosuchus watsoni*:**
Char. 25: 1 → 0
Char. 29: 0 → 1
Char. 51: 0 → 1
Char. 76: 0 → 1
Char. 86: 0 → 1
Char. 95: 0 → 1
Char. 98: 1 → 0
Char. 110: 0 → 1
Char. 111: 1 → 0
Char. 113: 0 → 1
Char. 114: 0 → 1
Char. 119: 1 → 0
Char. 131: 0 → 1
Char. 137: 0 → 1
Char. 138: 0 → 1
Char. 140: 0 → 2
Char. 144: 1 → 0
Char. 147: 0 → 1
Char. 154: 1 → 2

***Macroleter poezicus*:**
Char. 9: 0 → 1
Char. 26: 0 → 1
Char. 52: 0 → 1
Char. 66: 1 → 2
Char. 75: 0 → 1
Char. 84: 1 → 0
Char. 87: 0 → 1
Char. 110: 0 → 1
Char. 121: 0 → 1
Char. 126: 0 → 1
Char. 134: 0 → 2
Char. 140: 0 → 1
Char. 143: 0 → 1
Char. 146: 1 → 0
Char. 147: 1 → 2
Char. 154: 1 → 0
Char. 169: 1 → 0
Char. 235: 0 → 1
Char. 278: 3 → 0

***Mesosaurus* spp.:**
Char. 2: 0 → 1
Char. 5: 1 → 0
Char. 6: 0 → 1
Char. 8: 0 → 1
Char. 9: 0 → 1
Char. 13: 0 → 1
Char. 19: 0 → 1
Char. 20: 1 → 0
Char. 23: 0 → 1
Char. 26: 0 → 1
Char. 38: 1 → 0
Char. 41: 0 → 1
Char. 50: 1 → 0
Char. 76: 0 → 1
Char. 83: 0 → 1
Char. 84: 1 → 0
Char. 85: 1 → 0
Char. 94: 0 → 1
Char. 107: 0 → 1
Char. 109: 0 → 1
Char. 112: 1 → 0
Char. 115: 0 → 1
Char. 146: 1 → 0
Char. 148: 0 → 1
Char. 149: 1 → 0
Char. 159: 1 → 0
Char. 163: 1 → 0
Char. 164: 0 → 1
Char. 166: 1 → 0
Char. 167: 0 → 1
Char. 176: 0 → 1
Char. 183: 1 → 0
Char. 184: 0 → 1
Char. 192: 1 → 0
Char. 199: 0 → 1
Char. 204: 0 → 2
Char. 206: 0 → 1
Char. 217: 0 → 1
Char. 219: 0 → 1
Char. 220: 0 → 1
Char. 231: 0 → 1
Char. 234: 1 → 0
Char. 237: 1 → 0
Char. 240: 1 → 0
Char. 260: 2 → 0
Char. 272: 2 → 0
Char. 278: 3 → 0

***Microleter mckinzieorum*:**
Char. 18: 0 → 1
Char. 20: 1 → 0
Char. 24: 0 → 1
Char. 25: 1 → 0
Char. 29: 0 → 1
Char. 33: 1 → 0
Char. 36: 0 → 1
Char. 39: 0 → 1
Char. 48: 1 → 0
Char. 51: 0 → 2
Char. 56: 0 → 1
Char. 70: 0 → 1
Char. 76: 0 → 1
Char. 79: 0 → 1
Char. 83: 0 → 1
Char. 94: 0 → 1
Char. 106: 0 → 1
Char. 110: 0 → 1
Char. 111: 1 → 0
Char. 112: 1 → 0
Char. 132: 1 → 0
Char. 166: 1 → 0
Char. 276: 0 → 1
Char. 278: 3 → 1

**Millerettidae:**
Char. 5: 1 → 0
Char. 20: 1 → 0
Char. 24: 0 → 1
Char. 25: 1 → 0
Char. 29: 0 → 1
Char. 33: 1 → 0
Char. 44: 1 → 0
Char. 48: 1 → 0
Char. 56: 0 → 1
Char. 66: 0 → 2
Char. 78: 1 → 0
Char. 80: 1 → 0
Char. 84: 1 → 2
Char. 88: 1 → 0
Char. 96: 1 → 0
Char. 111: 1 → 0
Char. 112: 1 → 0
Char. 117: 0 → 1
Char. 120: 1 → 0
Char. 121: 0 → 1
Char. 124: 0 → 1
Char. 127: 0 → 1
Char. 135: 0 → 1
Char. 145: 0 → 1
Char. 159: 1 → 0
Char. 163: 1 → 0
Char. 166: 1 → 0
Char. 180: 1 → 0
Char. 192: 1 → 0
Char. 211: 0 → 1
Char. 230: 0 → 1
Char. 248: 0 → 1
Char. 252: 0 → 1

***Nycteroleter ineptus*:**
Char. 278: 0 → 3

***Nyctiphruretus acudens*:**
Char. 21: 0 → 1
Char. 33: 1 → 2
Char. 41: 0 → 1
Char. 48: 1 → 0
Char. 66: 1 → 2
Char. 81: 1 → 0
Char. 83: 0 → 1
Char. 84: 1 → 2
Char. 85: 1 → 0
Char. 94: 0 → 1
Char. 166: 1 → 0
Char. 167: 0 → 1
Char. 207: 1 → 0
Char. 215: 1 → 0
Char. 224: 0 → 1
Char. 226: 1 → 0
Char. 266: 0 → 1
Char. 272: 2 → 1
Char. 276: 0 → 1

***Orovenator mayorum*:**
Char. 8: 0 → 1
Char. 24: 0 → 1
Char. 25: 1 → 0
Char. 33: 1 → 0
Char. 36: 0 → 1
Char. 48: 1 → 0
Char. 62: 0 → 1
Char. 72: 1 → 2
Char. 89: 0 → 1
Char. 92: 1 → 0
Char. 136: 0 → 1
Char. 141: 0 → 1
Char. 159: 1 → 0
Char. 160: 0 → 1
Char. 165: 0 → 1
Char. 278: 3 → 1

***Owenetta* spp.:**
Char. 169: 1 → 0

***Paleothyris acadiana*:**
Char. 38: 1 → 0
Char. 50: 1 → 0
Char. 66: 1 → 2
Char. 102: 0 → 1
Char. 146: 1 → 0
Char. 239: 0 → 1

***Placodus* spp.:**
Char. 0: 1 → 2
Char. 9: 0 → 1
Char. 12: 0 → 1
Char. 13: 0 → 1

Char. 19: 0 → 1
Char. 26: 0 → 1
Char. 31: 0 → 1
Char. 44: 1 → 0
Char. 46: 1 → 0
Char. 57: 0 → 1
Char. 78: 1 → 0
Char. 93: 1 → 0
Char. 102: 1 → 2
Char. 140: 1 → 0
Char. 155: 0 → 1
Char. 163: 1 → 0
Char. 164: 0 → 1

***Procolophon* spp.:**
Char. 41: 0 → 1
Char. 69: 0 → 1
Char. 79: 0 → 1
Char. 83: 0 → 1
Char. 88: 0 → 1
Char. 117: 1 → 0
Char. 149: 1 → 0
Char. 204: 0 → 1
Char. 237: 0 → 1
Char. 238: 0 → 1
Char. 272: 2 → 1
Char. 278: 3 → 0

***Prolacerta broomi*:**
Char. 58: 1 → 0
Char. 66: 1 → 0
Char. 67: 1 → 0
Char. 80: 1 → 0
Char. 139: 1 → 0
Char. 192: 1 → 0
Char. 203: 1 → 2
Char. 206: 0 → 12

***Rhipaeosaurus* spp.:**
Char. 172: 0 → 1
Char. 186: 1 → 0
Char. 207: 1 → 0
Char. 277: 0 → 1

**Rhynchocephalia:**
Char. 0: 1 → 2
Char. 24: 0 → 1
Char. 77: 0 → 1
Char. 94: 1 → 0
Char. 139: 1 → 0
Char. 167: 1 → 0
Char. 205: 1 → 0

**Rhynchosauria:**
Char. 0: 1 → 0
Char. 7: 0 → 1
Char. 9: 0 → 1
Char. 26: 0 → 1
Char. 44: 1 → 0
Char. 68: 0 → 1
Char. 99: 1 → 0
Char. 150: 1 → 0
Char. 160: 0 → 1
Char. 161: 0 → 1
Char. 171: 0 → 2
Char. 223: 0 → 1
Char. 224: 0 → 1
Char. 241: 0 → 1

***Scutosaurus* spp.:**
Char. 175: 0 → 1
Char. 218: 0 → 1
Char. 243: 0 → 2
Char. 244: 0 → 1
Char. 251: 0 → 1

***Sinosaurosphargis yunguiensis*:**
Char. 8: 0 → 1
Char. 30: 0 → 1
Char. 53: 0 → 1
Char. 82: 0 → 1
Char. 89: 1 → 0
Char. 127: 1 → 0
Char. 150: 1 → 0
Char. 154: 0 → 2
Char. 167: 1 → 0
Char. 255: 0 → 1

**Squamata:**
Char. 26: 0 → 1
Char. 45: 0 → 1
Char. 79: 0 → 2
Char. 80: 1 → 0
Char. 82: 0 → 1
Char. 92: 1 → 0
Char. 160: 0 → 1
Char. 245: 0 → 1

***Trilophosaurus buettneri*:**
Char. 5: 1 → 0
Char. 11: 0 → 1
Char. 55: 1 → 0
Char. 93: 1 → 0
Char. 104: 0 → 1
Char. 106: 0 → 1
Char. 113: 0 → 1
Char. 122: 0 → 1
Char. 144: 1 → 0
Char. 154: 0 → 1
Char. 157: 0 → 1
Char. 159: 1 → 0
Char. 177: 0 → 12
Char. 194: 0 → 1
Char. 203: 1 → 2
Char. 207: 1 → 0
Char. 208: 1 → 0
Char. 272: 1 → 0

***Youngina capensis*:**
Char. 5: 1 → 0
Char. 21: 0 → 1
Char. 27: 0 → 1
Char. 38: 1 → 0
Char. 40: 2 → 0
Char. 43: 0 → 1
Char. 44: 1 → 0
Char. 50: 1 → 0
Char. 56: 0 → 1
Char. 59: 0 → 1
Char. 60: 0 → 1
Char. 62: 0 → 1
Char. 72: 1 → 2
Char. 75: 0 → 1
Char. 84: 1 → 0
Char. 89: 0 → 1
Char. 92: 1 → 0
Char. 109: 0 → 1
Char. 127: 0 → 1
Char. 129: 0 → 1
Char. 134: 0 → 1
Char. 135: 0 → 1
Char. 141: 0 → 1
Char. 154: 1 → 0
Char. 163: 1 → 0
Char. 170: 0 → 1
Char. 187: 0 → 1
Char. 191: 0 → 1
Char. 193: 0 → 1
Char. 201: 1 → 0
Char. 211: 0 → 1
Char. 214: 0 → 1
Char. 215: 0 → 1
Char. 219: 0 → 1
Char. 224: 0 → 1
Char. 231: 0 → 1
Char. 239: 0 → 1
Char. 265: 0 → 1
Char. 267: 0 → 1
Char. 275: 1 → 0
Char. 278: 3 → 0

**Node 50:**
Char. 46: 1 → 0
Char. 88: 1 → 0
Char. 93: 1 → 0
Char. 176: 0 → 1
Char. 195: 0 → 2
Char. 198: 0 → 1
Char. 246: 1 → 2
Char. 259: 0 → 1
Char. 270: 0 → 1

**Node 51:**
Char. 65: 0 → 1
Char. 72: 1 → 2
Char. 131: 0 → 1
Char. 184: 0 → 1
Char. 205: 0 → 1
Char. 210: 0 → 1
Char. 241: 0 → 1
Char. 246: 0 → 1
Char. 254: 0 → 1
Char. 255: 0 → 1
Char. 256: 0 → 1
Char. 267: 0 → 1
Char. 268: 0 → 1
Char. 269: 0 → 1
Char. 278: 3 → 0

**Node 52:**
Char. 15: 0 → 1
Char. 21: 0 → 1
Char. 25: 1 → 0
Char. 33: 1 → 2
Char. 44: 1 → 0
Char. 64: 0 → 1
Char. 73: 0 → 1
Char. 84: 1 → 2
Char. 94: 0 → 1
Char. 127: 0 → 1
Char. 130: 0 → 1
Char. 134: 0 → 2
Char. 135: 0 → 1
Char. 136: 0 → 1
Char. 141: 0 → 1
Char. 145: 0 → 1
Char. 147: 0 → 1
Char. 148: 0 → 1
Char. 151: 0 → 1
Char. 152: 0 → 1
Char. 155: 0 → 1
Char. 158: 0 → 1
Char. 161: 0 → 1
Char. 174: 0 → 1
Char. 181: 0 → 1
Char. 188: 0 → 1
Char. 201: 1 → 0
Char. 203: 1 → 2
Char. 204: 0 → 2
Char. 217: 0 → 1
Char. 220: 0 → 1
Char. 231: 0 → 1
Char. 251: 0 → 1
Char. 252: 0 → 1

**Node 53:**
Char. 5: 0 → 1
Char. 20: 0 → 1
Char. 33: 0 → 1
Char. 40: 0 → 2
Char. 48: 0 → 1
Char. 57: 1 → 0
Char. 74: 0 → 1
Char. 80: 0 → 1
Char. 84: 0 → 1
Char. 88: 0 → 1
Char. 112: 0 → 1
Char. 116: 0 → 1
Char. 119: 0 → 1
Char. 132: 0 → 1
Char. 149: 0 → 1
Char. 156: 0 → 1
Char. 163: 0 → 1
Char. 166: 0 → 1
Char. 169: 0 → 1
Char. 180: 0 → 1
Char. 192: 0 → 1
Char. 201: 0 → 1
Char. 202: 0 → 1
Char. 203: 0 → 1
Char. 209: 0 → 1
Char. 221: 0 → 1
Char. 234: 0 → 1
Char. 235: 0 → 1
Char. 237: 0 → 1

**Node 54:**
Char. 72: 0 → 1
Char. 79: 1 → 0
Char. 81: 0 → 1
Char. 93: 0 → 1
Char. 97: 0 → 1
Char. 104: 1 → 0
Char. 144: 0 → 1
Char. 173: 0 → 1
Char. 183: 0 → 1

**Node 56:**
Char. 19: 0 → 1
Char. 92: 1 → 0

**Node 57:**
Char. 4: 0 → 1
Char. 15: 0 → 1
Char. 29: 0 → 1
Char. 48: 1 → 0
Char. 213: 0 → 1
Char. 226: 0 → 2
Char. 228: 0 → 1

**Node 58:**
Char. 25: 1 → 0
Char. 50: 1 → 0
Char. 58: 0 → 1
Char. 59: 0 → 1
Char. 60: 0 → 1
Char. 61: 0 → 1
Char. 62: 0 → 1
Char. 66: 0 → 1
Char. 69: 0 → 1
Char. 72: 1 → 2
Char. 73: 0 → 1
Char. 89: 0 → 1
Char. 94: 0 → 1
Char. 126: 0 → 1
Char. 127: 0 → 1
Char. 129: 0 → 1
Char. 131: 0 → 1
Char. 134: 0 → 1
Char. 135: 0 → 1
Char. 141: 0 → 1
Char. 145: 0 → 1
Char. 150: 0 → 1
Char. 151: 0 → 1
Char. 154: 1 → 0
Char. 167: 0 → 1
Char. 176: 0 → 1
Char. 188: 0 → 1
Char. 190: 0 → 1
Char. 191: 0 → 1
Char. 205: 0 → 1
Char. 208: 0 → 1
Char. 214: 0 → 1
Char. 219: 0 → 1
Char. 230: 0 → 1

Char. 239: 0 → 1
Char. 260: 2 → 1
Char. 265: 0 → 1

**Node 59:**
Char. 73: 0 → 1
Char. 131: 1 → 0
Char. 205: 0 → 1
Char. 276: 0 → 1

**Node 60:**
Char. 18: 0 → 1
Char. 37: 0 → 1
Char. 107: 0 → 1
Char. 118: 1 → 0
Char. 125: 1 → 0
Char. 126: 0 → 1
Char. 150: 0 → 1

**Node 61:**
Char. 23: 0 → 1
Char. 71: 1 → 0
Char. 75: 0 → 1
Char. 85: 1 → 0
Char. 102: 0 → 1
Char. 103: 0 → 1
Char. 106: 0 → 1
Char. 110: 0 → 1
Char. 155: 0 → 1
Char. 167: 0 → 1
Char. 214: 0 → 1
Char. 216: 1 → 0
Char. 235: 0 → 2
Char. 241: 0 → 1

**Node 62:**
Char. 29: 0 → 1
Char. 38: 1 → 2
Char. 39: 0 → 1
Char. 44: 1 → 0
Char. 49: 0 → 1
Char. 51: 0 → 1
Char. 57: 0 → 1
Char. 66: 0 → 1
Char. 70: 0 → 1
Char. 76: 0 → 1
Char. 80: 1 → 0
Char. 88: 1 → 0
Char. 95: 0 → 1
Char. 111: 1 → 0
Char. 117: 0 → 1
Char. 118: 0 → 1
Char. 120: 1 → 0
Char. 125: 0 → 1
Char. 131: 0 → 1
Char. 135: 0 → 1
Char. 137: 0 → 1
Char. 147: 0 → 1
Char. 148: 0 → 1
Char. 157: 0 → 1
Char. 158: 0 → 1
Char. 183: 1 → 2
Char. 186: 0 → 1
Char. 194: 0 → 1
Char. 197: 0 → 1
Char. 201: 1 → 0
Char. 209: 1 → 0
Char. 211: 0 → 1
Char. 215: 0 → 1
Char. 226: 0 → 1
Char. 234: 1 → 0
Char. 235: 1 → 0
Char. 237: 1 → 0
Char. 240: 1 → 0
Char. 252: 0 → 1

**Node 63:**
Char. 87: 0 → 1
Char. 110: 0 → 1

**Node 64:**
Char. 25: 1 → 0
Char. 76: 1 → 0

**Node 65:**
Char. 79: 0 → 1
Char. 86: 0 → 1
Char. 93: 1 → 0
Char. 133: 0 → 1
Char. 147: 1 → 2
Char. 154: 1 → 0
Char. 278: 3 → 0

**Node 66:**
Char. 20: 1 → 0
Char. 38: 1 → 2
Char. 39: 0 → 1
Char. 50: 1 → 0
Char. 57: 0 → 1
Char. 58: 0 → 1
Char. 59: 0 → 1
Char. 60: 0 → 1
Char. 72: 1 → 2
Char. 83: 0 → 1
Char. 85: 1 → 0
Char. 88: 1 → 0
Char. 95: 0 → 1
Char. 104: 0 → 1
Char. 105: 0 → 1
Char. 106: 0 → 2
Char. 107: 0 → 1
Char. 109: 0 → 1
Char. 110: 0 → 1
Char. 146: 1 → 0
Char. 148: 0 → 1
Char. 155: 0 → 2
Char. 183: 1 → 2

**Node 67:**
Char. 33: 1 → 0
Char. 38: 2 → 1
Char. 39: 1 → 0
Char. 42: 0 → 1
Char. 43: 0 → 1
Char. 46: 1 → 0
Char. 48: 1 → 0
Char. 49: 1 → 0
Char. 52: 0 → 1
Char. 83: 0 → 2
Char. 84: 1 → 0
Char. 87: 0 → 1
Char. 93: 1 → 0
Char. 143: 0 → 1
Char. 161: 0 → 1
Char. 163: 1 → 0
Char. 172: 0 → 2
Char. 174: 0 → 1
Char. 188: 0 → 1
Char. 189: 0 → 1
Char. 195: 0 → 1
Char. 204: 0 → 2
Char. 212: 0 → 1
Char. 221: 1 → 0
Char. 236: 0 → 1
Char. 238: 0 → 2
Char. 242: 0 → 1
Char. 245: 0 → 1
Char. 274: 0 → 1
Char. 275: 1 → 0

**Node 68:**
Char. 5: 1 → 0
Char. 20: 1 → 0
Char. 33: 1 → 0
Char. 44: 1 → 0
Char. 48: 1 → 0
Char. 59: 0 → 1
Char. 60: 0 → 1
Char. 66: 0 → 1
Char. 72: 1 → 2
Char. 78: 1 → 0
Char. 84: 1 → 0
Char. 88: 1 → 0
Char. 93: 1 → 0
Char. 112: 1 → 0
Char. 123: 1 → 0
Char. 129: 0 → 1
Char. 154: 1 → 0
Char. 159: 1 → 0
Char. 163: 1 → 0
Char. 169: 1 → 0
Char. 170: 0 → 1
Char. 192: 1 → 0
Char. 197: 0 → 1
Char. 202: 1 → 0
Char. 207: 1 → 0
Char. 209: 1 → 0
Char. 221: 1 → 0
Char. 234: 1 → 0

**Node 69:**
Char. 29: 0 → 1
Char. 33: 1 → 0
Char. 47: 0 → 1
Char. 57: 0 → 1
Char. 83: 0 → 1
Char. 85: 1 → 0
Char. 107: 0 → 1
Char. 110: 0 → 1
Char. 111: 1 → 0
Char. 169: 1 → 0
Char. 170: 0 → 1

**Node 70:**
Char. 2: 0 → 1
Char. 6: 0 → 1
Char. 98: 1 → 0
Char. 101: 0 → 1
Char. 113: 0 → 1
Char. 181: 0 → 1
Char. 186: 0 → 1
Char. 198: 0 → 1
Char. 206: 0 → 2
Char. 220: 0 → 1
Char. 234: 1 → 0
Char. 239: 1 → 0
Char. 240: 1 → 0

**Node 71:**
Char. 21: 0 → 1
Char. 33: 1 → 2
Char. 68: 0 → 1
Char. 102: 0 → 1
Char. 103: 0 → 1
Char. 223: 0 → 1
Char. 229: 0 → 1
Char. 235: 1 → 2

**Node 72:**
Char. 17: 0 → 1
Char. 42: 0 → 1
Char. 251: 0 → 1
Char. 264: 0 → 1

**Node 73:**
Char. 41: 0 → 1
Char. 48: 1 → 0
Char. 61: 1 → 2
Char. 112: 1 → 0
Char. 128: 0 → 1
Char. 138: 0 → 1
Char. 146: 1 → 0
Char. 155: 0 → 1
Char. 192: 1 → 2
Char. 224: 0 → 1
Char. 226: 0 → 1
Char. 227: 0 → 1
Char. 233: 0 → 1

**Node 74:**
Char. 61: 1 → 3
Char. 90: 0 → 1
Char. 91: 0 → 1
Char. 155: 0 → 1
Char. 209: 1 → 0

**ANALYSIS 74**

**(ALL TAXA, IMPLIED WEIGHTING, K = 2.75, NO CHARACTERS 247 AND 253)**

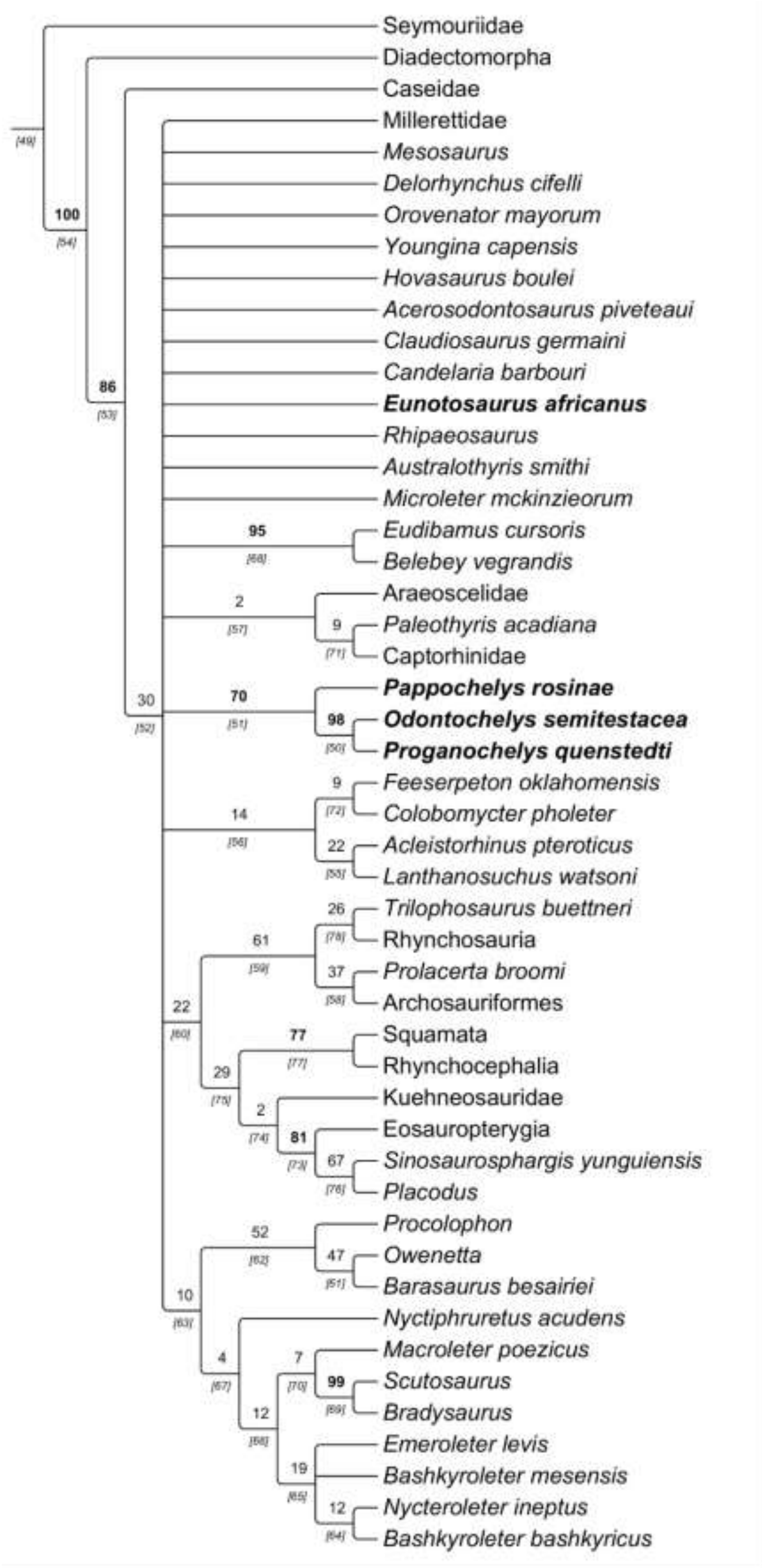

***Proganochelys quenstedti*:**
Char. 8: 0 → 1
Char. 11: 0 → 1
Char. 106: 0 → 1
Char. 108: 1 → 0
Char. 109: 0 → 1
Char. 128: 0 → 1
Char. 175: 0 → 1
Char. 202: 1 → 0
Char. 207: 1 → 0
Char. 209: 1 → 0
Char. 244: 0 → 1
Char. 248: 0 → 1
Char. 250: 0 → 1
Char. 262: 0 → 1

***Pappochelys rosinae*:**
Char. 1: 0 → 1
Char. 5: 1 → 0
Char. 12: 0 → 1
Char. 41: 0 → 1
Char. 48: 0 → 1
Char. 49: 0 → 1
Char. 55: 1 → 0
Char. 75: 0 → 1
Char. 89: 0 → 1
Char. 129: 0 → 1
Char. 169: 1 → 0
Char. 260: 2 → 0
Char. 265: 0 → 1

***Odontochelys semitestacea*:**
Char. 43: 0 → 1
Char. 112: 1 → 0
Char. 124: 0 → 1

***Eunotosaurus africanus*:**
Char. 0: 01 → 2
Char. 15: 0 → 1
Char. 19: 0 → 1
Char. 21: 0 → 1
Char. 24: 0 → 1
Char. 33: 1 → 2
Char. 40: 2 → 0
Char. 42: 0 → 1
Char. 43: 0 → 1
Char. 44: 1 → 0
Char. 50: 0 → 1
Char. 55: 1 → 0
Char. 57: 0 → 1
Char. 64: 0 → 1
Char. 73: 0 → 1
Char. 76: 0 → 1
Char. 81: 1 → 0
Char. 84: 1 → 2
Char. 89: 0 → 1
Char. 94: 0 → 1
Char. 97: 1 → 0
Char. 102: 0 → 1
Char. 103: 0 → 1
Char. 112: 1 → 0
Char. 124: 0 → 1
Char. 127: 0 → 1
Char. 130: 0 → 1
Char. 134: 0 → 2
Char. 136: 0 → 1
Char. 141: 0 → 1
Char. 145: 0 → 1
Char. 147: 0 → 1
Char. 151: 0 → 1
Char. 152: 0 → 1
Char. 153: 1 → 0
Char. 155: 0 → 1
Char. 158: 0 → 1
Char. 161: 0 → 1
Char. 174: 0 → 1
Char. 181: 0 → 1
Char. 188: 0 → 1
Char. 192: 1 → 0
Char. 194: 0 → 1
Char. 202: 1 → 0
Char. 203: 1 → 2
Char. 211: 0 → 1
Char. 217: 0 → 1
Char. 219: 0 → 1
Char. 231: 0 → 1
Char. 240: 1 → 0
Char. 248: 0 → 1
Char. 249: 0 → 2
Char. 250: 0 → 1
Char. 251: 0 → 1
Char. 252: 0 → 1
Char. 263: 0 → 1
Char. 273: 0 → 1
Char. 274: 0 → 1
Char. 276: 0 → 1
Char. 277: 0 → 1

***Acerosodontosaurus piveteaui*:**
Char. 59: 0 → 1
Char. 78: 1 → 0
Char. 81: 1 → 0
Char. 89: 0 → 1
Char. 94: 0 → 1
Char. 128: 0 → 1
Char. 129: 0 → 1
Char. 155: 0 → 1
Char. 204: 2 → 0
Char. 206: 0 → 2
Char. 208: 0 → 1
Char. 265: 0 → 1
Char. 266: 0 → 1
Char. 278: 3 → 1

***Acleistorhinus pteroticus:***
Char. 21: 0 → 1
Char. 25: 0 → 1
Char. 146: 1 → 0

**Araeoscelidae:**
Char. 28: 0 → 1
Char. 43: 0 → 1
Char. 48: 0 → 1
Char. 89: 0 → 1
Char. 106: 0 → 1
Char. 193: 0 → 1
Char. 222: 0 → 1
Char. 224: 0 → 1
Char. 266: 0 → 1
Char. 278: 3 → 1

**Archosauriformes:**
Char. 32: 0 → 1
Char. 94: 1 → 0
Char. 112: 1 → 0
Char. 152: 0 → 1
Char. 154: 01 → 2
Char. 166: 1 → 0
Char. 171: 0 → 1
Char. 185: 0 → 1
Char. 201: 0 → 1
Char. 204: 0 → 1
Char. 218: 0 → 3
Char. 224: 0 → 1
Char. 242: 0 → 1

***Australothyris smithi*:**
Char. 24: 0 → 1
Char. 25: 0 → 1
Char. 29: 0 → 1
Char. 34: 0 → 1
Char. 50: 0 → 1
Char. 55: 1 → 0
Char. 71: 1 → 0
Char. 79: 0 → 1
Char. 83: 0 → 1
Char. 85: 1 → 0
Char. 98: 1 → 0
Char. 100: 0 → 1
Char. 103: 0 → 1
Char. 110: 0 → 1
Char. 111: 1 → 0
Char. 123: 1 → 0
Char. 129: 0 → 1
Char. 131: 0 → 1
Char. 132: 1 → 0
Char. 144: 1 → 0
Char. 147: 0 → 1
Char. 148: 1 → 0
Char. 149: 1 → 0
Char. 150: 0 → 1
Char. 159: 1 → 0
Char. 163: 1 → 0
Char. 192: 1 → 0

***Barasaurus besairiei*:**
Char. 33: 1 → 0
Char. 50: 0 → 1

***Bashkyroleter bashkyricus*:**
Char. 275: 1 → 0

***Bashkyroleter mesensis*:**
Char. 12: 0 → 1
Char. 66: 1 → 0
Char. 169: 1 → 0

***Belebey vegrandis*:**
Char. 154: 1 → 0

***Bradysaurus* spp.:**
Char. 19: 0 → 1
Char. 73: 0 → 1
Char. 79: 0 → 1
Char. 135: 1 → 0
Char. 249: 0 → 1

***Candelaria barbouri*:**
Char. 1: 0 → 1
Char. 5: 1 → 0
Char. 8: 0 → 1
Char. 15: 0 → 1
Char. 33: 1 → 2
Char. 49: 0 → 1
Char. 55: 1 → 0
Char. 76: 0 → 1
Char. 79: 0 → 2
Char. 83: 0 → 1
Char. 88: 1 → 0
Char. 89: 0 → 1
Char. 92: 1 → 0
Char. 94: 0 → 1
Char. 95: 0 → 1
Char. 126: 0 → 1
Char. 127: 0 → 1
Char. 132: 1 → 0
Char. 154: 1 → 2
Char. 159: 1 → 0
Char. 169: 1 → 0
Char. 276: 0 → 1
Char. 277: 0 → 1

**Captorhinidae:**
Char. 3: 0 → 1
Char. 26: 0 → 1
Char. 50: 0 → 1
Char. 73: 0 → 1
Char. 75: 0 → 1
Char. 83: 0 → 1
Char. 108: 1 → 0
Char. 180: 1 → 0
Char. 183: 1 → 0
Char. 203: 1 → 2
Char. 216: 1 → 0
Char. 240: 1 → 0

**Caseidae:**
Char. 24: 0 → 1
Char. 36: 0 → 1
Char. 38: 1 → 0
Char. 46: 1 → 0
Char. 56: 0 → 1
Char. 170: 0 → 1
Char. 194: 0 → 1
Char. 273: 0 → 1
Char. 274: 0 → 1
Char. 278: 3 → 2

***Claudiosaurus germaini*:**
Char. 24: 0 → 1
Char. 27: 0 → 1
Char. 34: 0 → 1
Char. 36: 0 → 1
Char. 48: 0 → 1
Char. 56: 0 → 1
Char. 59: 0 → 1
Char. 60: 0 → 1
Char. 62: 0 → 1
Char. 64: 0 → 1
Char. 70: 0 → 1
Char. 72: 1 → 2
Char. 73: 0 → 1
Char. 84: 1 → 0
Char. 89: 0 → 1
Char. 94: 0 → 1
Char. 105: 0 → 1
Char. 106: 0 → 1
Char. 109: 0 → 1
Char. 117: 01 → 2
Char. 126: 0 → 1
Char. 129: 0 → 1
Char. 130: 0 → 1
Char. 131: 0 → 1
Char. 144: 1 → 0
Char. 154: 1 → 0
Char. 166: 1 → 0
Char. 182: 0 → 1
Char. 188: 0 → 1
Char. 190: 0 → 1
Char. 199: 0 → 1
Char. 201: 0 → 1
Char. 203: 1 → 2
Char. 204: 2 → 1
Char. 214: 0 → 1
Char. 219: 0 → 1
Char. 234: 1 → 0
Char. 265: 0 → 1
Char. 267: 0 → 1
Char. 272: 2 → 1
Char. 275: 1 → 0
Char. 278: 3 → 0

***Colobomycter pholeter*:**
Char. 21: 0 → 1
Char. 84: 1 → 0
Char. 154: 1 → 0
Char. 167: 0 → 1
Char. 267: 0 → 1

***Delorhynchus cifelli*:**
Char. 18: 0 → 1
Char. 21: 0 → 1
Char. 24: 0 → 1
Char. 25: 0 → 1
Char. 26: 0 → 1
Char. 28: 0 → 1
Char. 29: 0 → 1
Char. 33: 1 → 2
Char. 39: 0 → 1
Char. 48: 0 → 1
Char. 50: 0 → 1
Char. 52: 0 → 1
Char. 57: 0 → 1

Char. 100: 0 → 1
Char. 116: 1 → 0
Char. 119: 1 → 0
Char. 131: 0 → 1
Char. 147: 0 → 1
Char. 148: 1 → 0
Char. 156: 1 → 0
Char. 167: 0 → 1
Char. 189: 0 → 1
Char. 196: 1 → 0
Char. 201: 0 → 1
Char. 202: 1 → 0
Char. 267: 0 → 1

**Diadectomorpha:**
Char. 64: 0 → 1
Char. 70: 0 → 1
Char. 122: 0 → 1
Char. 123: 1 → 0
Char. 146: 1 → 0
Char. 275: 1 → 0
Char. 278: 3 → 0

***Emeroleter levis*:**
Char. 51: 1 → 0

**Eosauropterygia:**
Char. 166: 1 → 0
Char. 174: 0 → 1
Char. 178: 0 → 1
Char. 194: 0 → 2
Char. 272: 1 → 0

***Eudibamus cursoris*:**
Char. 154: 1 → 2
Char. 163: 1 → 0

***Feeserpeton oklahomensis*:**
Char. 25: 0 → 1
Char. 51: 0 → 1
Char. 157: 0 → 1
Char. 158: 0 → 1

***Hovasaurus boulei*:**
Char. 41: 0 → 1
Char. 43: 0 → 1
Char. 48: 0 → 1
Char. 55: 1 → 0
Char. 59: 0 → 1
Char. 77: 0 → 2
Char. 78: 1 → 0
Char. 79: 0 → 1
Char. 89: 0 → 1
Char. 93: 1 → 0
Char. 113: 0 → 1
Char. 127: 0 → 1
Char. 136: 0 → 1
Char. 138: 0 → 1
Char. 146: 1 → 0
Char. 148: 1 → 0
Char. 188: 0 → 1
Char. 193: 0 → 1
Char. 206: 0 → 2

Char. 215: 0 → 1
Char. 222: 0 → 1
Char. 224: 0 → 1
Char. 265: 0 → 1
Char. 275: 1 → 0

**Kuehneosauridae:**
Char. 7: 0 → 1
Char. 24: 0 → 1
Char. 27: 0 → 1
Char. 43: 1 → 0
Char. 79: 0 → 2
Char. 107: 1 → 0
Char. 140: 1 → 0
Char. 147: 1 → 0
Char. 148: 1 → 0
Char. 245: 0 → 1
Char. 278: 0 → 3

***Lanthanosuchus watsoni*:**
Char. 51: 0 → 1
Char. 76: 0 → 1
Char. 86: 0 → 1
Char. 98: 1 → 0
Char. 138: 0 → 1
Char. 144: 1 → 0
Char. 154: 1 → 2

***Macroleter poezicus*:**
Char. 9: 0 → 1
Char. 26: 0 → 1
Char. 66: 1 → 2
Char. 126: 0 → 1
Char. 134: 0 → 2
Char. 140: 0 → 1
Char. 146: 1 → 0
Char. 169: 1 → 0
Char. 204: 2 → 0

***Mesosaurus* spp.:**
Char. 2: 0 → 1
Char. 5: 1 → 0
Char. 6: 0 → 1
Char. 8: 0 → 1
Char. 9: 0 → 1
Char. 13: 0 → 1
Char. 19: 0 → 1
Char. 20: 1 → 0
Char. 25: 0 → 1
Char. 26: 0 → 1
Char. 38: 1 → 0
Char. 41: 0 → 1
Char. 48: 0 → 1
Char. 76: 0 → 1
Char. 83: 0 → 1
Char. 84: 1 → 0
Char. 85: 1 → 0
Char. 94: 0 → 1
Char. 107: 0 → 1
Char. 109: 0 → 1
Char. 112: 1 → 0
Char. 115: 0 → 1
Char. 146: 1 → 0

Char. 149: 1 → 0
Char. 159: 1 → 0
Char. 163: 1 → 0
Char. 164: 0 → 1
Char. 166: 1 → 0
Char. 167: 0 → 1
Char. 176: 0 → 1
Char. 183: 1 → 0
Char. 184: 0 → 1
Char. 192: 1 → 0
Char. 196: 1 → 0
Char. 199: 0 → 1
Char. 201: 0 → 1
Char. 206: 0 → 1
Char. 217: 0 → 1
Char. 219: 0 → 1
Char. 231: 0 → 1
Char. 234: 1 → 0
Char. 240: 1 → 0
Char. 260: 2 → 0
Char. 272: 2 → 0
Char. 278: 3 → 0

***Microleter mckinzieorum*:**
Char. 18: 0 → 1
Char. 20: 1 → 0
Char. 24: 0 → 1
Char. 29: 0 → 1
Char. 33: 1 → 0
Char. 36: 0 → 1
Char. 39: 0 → 1
Char. 50: 0 → 1
Char. 51: 0 → 2
Char. 56: 0 → 1
Char. 70: 0 → 1
Char. 76: 0 → 1
Char. 79: 0 → 1
Char. 83: 0 → 1
Char. 94: 0 → 1
Char. 106: 0 → 1
Char. 110: 0 → 1
Char. 111: 1 → 0
Char. 112: 1 → 0
Char. 132: 1 → 0
Char. 166: 1 → 0
Char. 276: 0 → 1
Char. 278: 3 → 1

**Millerettidae:**
Char. 5: 1 → 0
Char. 20: 1 → 0
Char. 24: 0 → 1
Char. 29: 0 → 1
Char. 33: 1 → 0
Char. 44: 1 → 0
Char. 50: 0 → 1
Char. 56: 0 → 1
Char. 66: 0 → 2
Char. 78: 1 → 0
Char. 80: 1 → 0
Char. 84: 1 → 2
Char. 88: 1 → 0
Char. 96: 1 → 0

Char. 111: 1 → 0
Char. 112: 1 → 0
Char. 120: 1 → 0
Char. 121: 0 → 1
Char. 124: 0 → 1
Char. 127: 0 → 1
Char. 145: 0 → 1
Char. 148: 1 → 0
Char. 159: 1 → 0
Char. 163: 1 → 0
Char. 166: 1 → 0
Char. 180: 1 → 0
Char. 192: 1 → 0
Char. 201: 0 → 1
Char. 211: 0 → 1
Char. 230: 0 → 1
Char. 248: 0 → 1
Char. 252: 0 → 1

***Nycteroleter ineptus*:**
Char. 66: 1 → 0
Char. 278: 0 → 3

***Nyctiphruretus acudens*:**
Char. 21: 0 → 1
Char. 41: 0 → 1
Char. 66: 1 → 2
Char. 81: 1 → 0
Char. 83: 0 → 1
Char. 84: 1 → 2
Char. 94: 0 → 1
Char. 166: 1 → 0
Char. 207: 1 → 0
Char. 224: 0 → 1
Char. 266: 0 → 1
Char. 272: 2 → 1
Char. 276: 0 → 1

***Orovenator mayorum*:**
Char. 8: 0 → 1
Char. 24: 0 → 1
Char. 33: 1 → 0
Char. 36: 0 → 1
Char. 50: 0 → 1
Char. 62: 0 → 1
Char. 72: 1 → 2
Char. 89: 0 → 1
Char. 92: 1 → 0
Char. 136: 0 → 1
Char. 141: 0 → 1
Char. 148: 1 → 0
Char. 159: 1 → 0
Char. 160: 0 → 1
Char. 165: 0 → 1
Char. 278: 3 → 1

***Owenetta* spp.:**
Char. 142: 1 → 0
Char. 169: 1 → 0

***Paleothyris acadiana*:**
Char. 66: 1 → 2

Char. 102: 0 → 1
Char. 146: 1 → 0

***Placodus* spp.:**
Char. 0: 1 → 2
Char. 9: 0 → 1
Char. 12: 0 → 1
Char. 13: 0 → 1
Char. 19: 0 → 1
Char. 31: 0 → 1
Char. 46: 1 → 0
Char. 57: 0 → 1
Char. 78: 1 → 0
Char. 93: 1 → 0
Char. 102: 1 → 2
Char. 140: 1 → 0
Char. 155: 0 → 1
Char. 163: 1 → 0
Char. 164: 0 → 1

***Procolophon* spp.:**
Char. 41: 0 → 1
Char. 69: 0 → 1
Char. 79: 0 → 1
Char. 83: 0 → 1
Char. 86: 0 → 1
Char. 101: 0 → 1
Char. 141: 0 → 1
Char. 149: 1 → 0
Char. 203: 1 → 2
Char. 215: 0 → 1
Char. 230: 0 → 1
Char. 235: 1 → 2
Char. 238: 0 → 1
Char. 272: 2 → 1
Char. 278: 3 → 0

***Prolacerta broomi*:**
Char. 58: 1 → 0
Char. 66: 1 → 0
Char. 67: 1 → 0
Char. 80: 1 → 0
Char. 139: 1 → 0
Char. 147: 1 → 0
Char. 192: 1 → 0
Char. 203: 1 → 2
Char. 206: 0 → 12

***Rhipaeosaurus* spp.:**
Char. 120: 1 → 0
Char. 172: 0 → 1
Char. 183: 1 → 2
Char. 194: 0 → 1
Char. 207: 1 → 0
Char. 211: 0 → 1
Char. 215: 0 → 1
Char. 226: 0 → 1
Char. 234: 1 → 0
Char. 235: 1 → 0
Char. 240: 1 → 0
Char. 252: 0 → 1
Char. 277: 0 → 1

**Rhynchocephalia:**
Char. 0: 1 → 2
Char. 24: 0 → 1
Char. 77: 0 → 1
Char. 88: 1 → 0
Char. 94: 1 → 0
Char. 139: 1 → 0
Char. 167: 1 → 0
Char. 205: 1 → 0

**Rhynchosauria:**
Char. 0: 1 → 0
Char. 7: 0 → 1
Char. 9: 0 → 1
Char. 26: 0 → 1
Char. 44: 1 → 0
Char. 68: 0 → 1
Char. 99: 1 → 0
Char. 150: 1 → 0
Char. 160: 0 → 1
Char. 161: 0 → 1
Char. 171: 0 → 2
Char. 223: 0 → 1
Char. 224: 0 → 1
Char. 241: 0 → 1

***Scutosaurus* spp.:**
Char. 175: 0 → 1
Char. 190: 0 → 1
Char. 218: 0 → 1
Char. 243: 0 → 2
Char. 244: 0 → 1
Char. 251: 0 → 1

***Sinosaurosphargis yunguiensis*:**
Char. 8: 0 → 1
Char. 30: 0 → 1
Char. 53: 0 → 1
Char. 89: 1 → 0
Char. 127: 1 → 0
Char. 150: 1 → 0
Char. 154: 0 → 2
Char. 167: 1 → 0
Char. 255: 0 → 1

**Squamata:**
Char. 45: 0 → 1
Char. 79: 0 → 2
Char. 80: 1 → 0
Char. 92: 1 → 0
Char. 160: 0 → 1
Char. 200: 0 → 1
Char. 245: 0 → 1

***Trilophosaurus buettneri*:**
Char. 5: 1 → 0
Char. 11: 0 → 1
Char. 55: 1 → 0
Char. 93: 1 → 0
Char. 104: 0 → 1
Char. 106: 0 → 1
Char. 113: 0 → 1
Char. 122: 0 → 1
Char. 144: 1 → 0
Char. 157: 0 → 1
Char. 159: 1 → 0
Char. 177: 0 → 12
Char. 194: 0 → 1
Char. 203: 1 → 2
Char. 207: 1 → 0
Char. 208: 1 → 0
Char. 272: 1 → 0

***Youngina capensis*:**
Char. 5: 1 → 0
Char. 21: 0 → 1
Char. 25: 0 → 1
Char. 27: 0 → 1
Char. 38: 1 → 0
Char. 40: 2 → 0
Char. 43: 0 → 1
Char. 44: 1 → 0
Char. 48: 0 → 1
Char. 56: 0 → 1
Char. 59: 0 → 1
Char. 60: 0 → 1
Char. 62: 0 → 1
Char. 72: 1 → 2
Char. 75: 0 → 1
Char. 84: 1 → 0
Char. 89: 0 → 1
Char. 92: 1 → 0
Char. 109: 0 → 1
Char. 127: 0 → 1
Char. 129: 0 → 1
Char. 134: 0 → 1
Char. 141: 0 → 1
Char. 148: 1 → 0
Char. 154: 1 → 0
Char. 163: 1 → 0
Char. 170: 0 → 1
Char. 193: 0 → 1
Char. 211: 0 → 1
Char. 214: 0 → 1
Char. 215: 0 → 1
Char. 219: 0 → 1
Char. 222: 0 → 1
Char. 224: 0 → 1
Char. 231: 0 → 1
Char. 239: 0 → 1
Char. 265: 0 → 1
Char. 267: 0 → 1
Char. 275: 1 → 0
Char. 278: 3 → 0

**Node 50:**
Char. 46: 1 → 0
Char. 50: 0 → 1
Char. 88: 1 → 0
Char. 93: 1 → 0
Char. 176: 0 → 1
Char. 195: 0 → 2
Char. 198: 0 → 1
Char. 240: 1 → 0
Char. 246: 1 → 2
Char. 259: 0 → 1
Char. 270: 0 → 1

**Node 51:**
Char. 44: 1 → 0
Char. 65: 0 → 1
Char. 72: 1 → 2
Char. 73: 0 → 1
Char. 78: 1 → 0
Char. 131: 0 → 1
Char. 184: 0 → 1
Char. 194: 0 → 2
Char. 205: 0 → 1
Char. 210: 0 → 1
Char. 217: 0 → 1
Char. 222: 0 → 1
Char. 241: 0 → 1
Char. 246: 0 → 1
Char. 254: 0 → 1
Char. 255: 0 → 1
Char. 256: 0 → 1
Char. 266: 0 → 1
Char. 267: 0 → 1
Char. 268: 0 → 1
Char. 269: 0 → 1
Char. 278: 3 → 0

**Node 52:**
Char. 5: 0 → 1
Char. 20: 0 → 1
Char. 33: 0 → 1
Char. 40: 0 → 2
Char. 57: 1 → 0
Char. 74: 0 → 1
Char. 80: 0 → 1
Char. 84: 0 → 1
Char. 88: 0 → 1
Char. 112: 0 → 1
Char. 116: 0 → 1
Char. 119: 0 → 1
Char. 132: 0 → 1
Char. 149: 0 → 1
Char. 156: 0 → 1
Char. 163: 0 → 1
Char. 166: 0 → 1
Char. 169: 0 → 1
Char. 179: 0 → 1
Char. 180: 0 → 1
Char. 192: 0 → 1
Char. 196: 0 → 1
Char. 202: 0 → 1
Char. 203: 0 → 1
Char. 204: 0 → 2
Char. 209: 0 → 1
Char. 221: 0 → 1
Char. 234: 0 → 1
Char. 235: 0 → 1

**Node 53:**
Char. 25: 1 → 0
Char. 72: 0 → 1
Char. 79: 1 → 0
Char. 81: 0 → 1
Char. 93: 0 → 1
Char. 97: 0 → 1
Char. 104: 1 → 0
Char. 144: 0 → 1
Char. 173: 0 → 1
Char. 183: 0 → 1

**Node 55:**
Char. 95: 0 → 1
Char. 113: 0 → 1
Char. 114: 0 → 1

**Node 56:**
Char. 29: 0 → 1
Char. 47: 0 → 1
Char. 48: 0 → 1
Char. 50: 0 → 1
Char. 57: 0 → 1
Char. 79: 0 → 1
Char. 110: 0 → 1
Char. 111: 1 → 0
Char. 112: 1 → 0
Char. 131: 0 → 1
Char. 137: 0 → 1
Char. 140: 0 → 2
Char. 147: 0 → 1
Char. 148: 1 → 0

**Node 57:**
Char. 5: 1 → 0
Char. 20: 1 → 0
Char. 33: 1 → 0
Char. 59: 0 → 1
Char. 60: 0 → 1
Char. 72: 1 → 2
Char. 84: 1 → 0
Char. 116: 1 → 0
Char. 154: 1 → 0
Char. 159: 1 → 0
Char. 166: 1 → 0
Char. 169: 1 → 0
Char. 170: 0 → 1
Char. 179: 1 → 0
Char. 196: 1 → 0
Char. 197: 0 → 1
Char. 202: 1 → 0
Char. 204: 2 → 0
Char. 207: 1 → 0
Char. 209: 1 → 0
Char. 221: 1 → 0

**Node 58:**
Char. 19: 0 → 1
Char. 92: 1 → 0

**Node 59:**
Char. 4: 0 → 1
Char. 15: 0 → 1
Char. 29: 0 → 1
Char. 213: 0 → 1
Char. 226: 0 → 2
Char. 228: 0 → 1

**Node 60:**
Char. 43: 0 → 1
Char. 58: 0 → 1
Char. 59: 0 → 1
Char. 60: 0 → 1
Char. 61: 0 → 1
Char. 62: 0 → 1
Char. 66: 0 → 1
Char. 69: 0 → 1
Char. 72: 1 → 2
Char. 73: 0 → 1
Char. 89: 0 → 1
Char. 94: 0 → 1
Char. 107: 0 → 1
Char. 126: 0 → 1
Char. 127: 0 → 1
Char. 129: 0 → 1
Char. 131: 0 → 1
Char. 134: 0 → 1
Char. 140: 0 → 1
Char. 141: 0 → 1
Char. 145: 0 → 1
Char. 147: 0 → 1
Char. 150: 0 → 1
Char. 151: 0 → 1
Char. 167: 0 → 1
Char. 176: 0 → 1
Char. 188: 0 → 1
Char. 190: 0 → 1
Char. 205: 0 → 1
Char. 208: 0 → 1
Char. 214: 0 → 1
Char. 219: 0 → 1
Char. 230: 0 → 1
Char. 239: 0 → 1
Char. 260: 2 → 1
Char. 265: 0 → 1
Char. 278: 3 → 0

**Node 61:**
Char. 73: 0 → 1
Char. 205: 0 → 1
Char. 273: 0 → 1
Char. 276: 0 → 1

**Node 62:**
Char. 18: 0 → 1
Char. 37: 0 → 1
Char. 103: 0 → 1
Char. 106: 0 → 1
Char. 107: 0 → 1
Char. 110: 0 → 1
Char. 126: 0 → 1
Char. 150: 0 → 1

**Node 63:**
Char. 29: 0 → 1
Char. 38: 1 → 2
Char. 39: 0 → 1
Char. 44: 1 → 0
Char. 49: 0 → 1
Char. 57: 0 → 1
Char. 66: 0 → 1

Char. 76: 0 → 1
Char. 80: 1 → 0
Char. 95: 0 → 1
Char. 111: 1 → 0
Char. 120: 1 → 0
Char. 137: 0 → 1
Char. 147: 0 → 1
Char. 157: 0 → 1
Char. 158: 0 → 1
Char. 179: 1 → 0
Char. 183: 1 → 2
Char. 186: 0 → 1
Char. 194: 0 → 1
Char. 197: 0 → 1
Char. 209: 1 → 0
Char. 211: 0 → 1
Char. 234: 1 → 0
Char. 240: 1 → 0
Char. 252: 0 → 1

**Node 64:**
Char. 87: 0 → 1
Char. 110: 0 → 1

**Node 65:**
Char. 79: 0 → 1
Char. 133: 0 → 1

**Node 66:**
Char. 100: 0 → 1
Char. 113: 0 → 1

**Node 67:**
Char. 50: 0 → 1
Char. 70: 0 → 1
Char. 118: 0 → 1
Char. 125: 0 → 1

**Node 68:**
Char. 20: 1 → 0
Char. 38: 1 → 2
Char. 39: 0 → 1
Char. 57: 0 → 1
Char. 58: 0 → 1
Char. 59: 0 → 1
Char. 60: 0 → 1
Char. 72: 1 → 2
Char. 83: 0 → 1
Char. 85: 1 → 0
Char. 88: 1 → 0
Char. 95: 0 → 1
Char. 104: 0 → 1
Char. 105: 0 → 1
Char. 106: 0 → 2
Char. 107: 0 → 1
Char. 109: 0 → 1
Char. 110: 0 → 1
Char. 146: 1 → 0
Char. 155: 0 → 2
Char. 183: 1 → 2

**Node 69:**
Char. 23: 0 → 1
Char. 33: 1 → 0
Char. 38: 2 → 1
Char. 39: 1 → 0
Char. 42: 0 → 1
Char. 43: 0 → 1
Char. 46: 1 → 0
Char. 49: 1 → 0
Char. 71: 1 → 0
Char. 83: 0 → 2
Char. 101: 0 → 1
Char. 103: 0 → 1
Char. 106: 0 → 1
Char. 161: 0 → 1
Char. 163: 1 → 0
Char. 172: 0 → 2
Char. 174: 0 → 1
Char. 188: 0 → 1
Char. 203: 1 → 2
Char. 212: 0 → 1
Char. 216: 1 → 0
Char. 221: 1 → 0
Char. 236: 0 → 1
Char. 238: 0 → 2
Char. 241: 0 → 1
Char. 242: 0 → 1
Char. 273: 0 → 1
Char. 274: 0 → 1
Char. 275: 1 → 0

**Node 70:**
Char. 52: 0 → 1
Char. 75: 0 → 1
Char. 84: 1 → 0
Char. 87: 0 → 1
Char. 110: 0 → 1
Char. 121: 0 → 1
Char. 180: 1 → 0
Char. 230: 0 → 1

**Node 71:**
Char. 44: 1 → 0
Char. 66: 0 → 1
Char. 78: 1 → 0
Char. 88: 1 → 0
Char. 93: 1 → 0
Char. 112: 1 → 0
Char. 123: 1 → 0
Char. 129: 0 → 1
Char. 163: 1 → 0
Char. 192: 1 → 0
Char. 234: 1 → 0

**Node 72:**
Char. 33: 1 → 0
Char. 83: 0 → 1
Char. 85: 1 → 0
Char. 107: 0 → 1

**Node 73:**
Char. 2: 0 → 1
Char. 6: 0 → 1
Char. 186: 0 → 1
Char. 198: 0 → 1
Char. 200: 0 → 1
Char. 220: 0 → 1
Char. 239: 1 → 0

**Node 74:**
Char. 48: 0 → 1
Char. 98: 1 → 0
Char. 113: 0 → 1
Char. 181: 0 → 1
Char. 206: 0 → 2

**Node 75:**
Char. 35: 0 → 1
Char. 102: 0 → 1
Char. 103: 0 → 1
Char. 184: 0 → 1
Char. 201: 0 → 1
Char. 210: 0 → 1
Char. 223: 0 → 1
Char. 229: 0 → 1

**Node 76:**
Char. 17: 0 → 1
Char. 42: 0 → 1
Char. 251: 0 → 1
Char. 264: 0 → 1

**Node 77:**
Char. 41: 0 → 1
Char. 61: 1 → 2
Char. 112: 1 → 0
Char. 138: 0 → 1
Char. 146: 1 → 0
Char. 155: 0 → 1
Char. 192: 1 → 2
Char. 224: 0 → 1
Char. 226: 0 → 1
Char. 227: 0 → 1
Char. 233: 0 → 1
Char. 267: 01 → 2

**Node 78:**
Char. 61: 1 → 3
Char. 90: 0 → 1
Char. 91: 0 → 1
Char. 155: 0 → 1
Char. 209: 1 → 0

**Analysis 75**
**(all taxa, no characters 247, 251, and 253)**

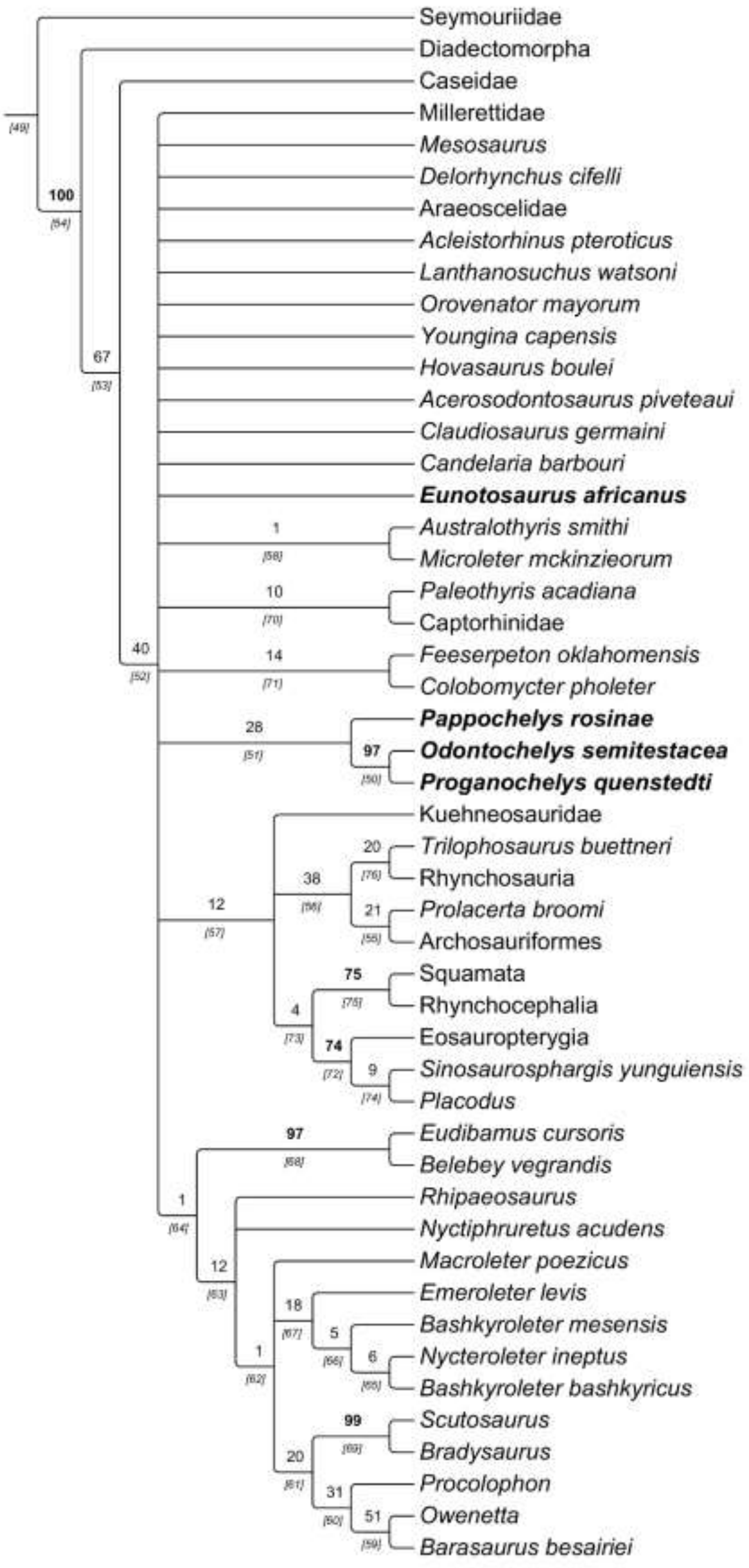

***Proganochelys quenstedti*:**
Char. 8: 0 → 1
Char. 11: 0 → 1
Char. 106: 0 → 1
Char. 108: 1 → 0
Char. 109: 0 → 1
Char. 128: 0 → 1
Char. 175: 0 → 1
Char. 202: 1 → 0
Char. 207: 1 → 0
Char. 209: 1 → 0
Char. 244: 0 → 1
Char. 248: 0 → 1
Char. 250: 0 → 1
Char. 262: 0 → 1

***Pappochelys rosinae*:**
Char. 1: 0 → 1
Char. 5: 1 → 0
Char. 12: 0 → 1
Char. 23: 0 → 1
Char. 41: 0 → 1
Char. 49: 0 → 1
Char. 55: 1 → 0
Char. 67: 1 → 0
Char. 75: 0 → 1
Char. 89: 0 → 1
Char. 129: 0 → 1
Char. 169: 1 → 0
Char. 260: 2 → 0
Char. 265: 0 → 1

***Odontochelys semitestacea*:**
Char. 43: 0 → 1
Char. 124: 0 → 1

***Eunotosaurus africanus*:**
Char. 0: 0 → 2
Char. 15: 0 → 1
Char. 19: 0 → 1
Char. 21: 0 → 1
Char. 24: 0 → 1
Char. 33: 1 → 2
Char. 40: 2 → 0
Char. 42: 0 → 1
Char. 43: 0 → 1
Char. 44: 1 → 0
Char. 55: 1 → 0
Char. 57: 0 → 1
Char. 64: 0 → 1
Char. 67: 1 → 0
Char. 73: 0 → 1
Char. 76: 0 → 1
Char. 81: 1 → 0
Char. 84: 1 → 2
Char. 89: 0 → 1
Char. 94: 0 → 1
Char. 97: 1 → 0
Char. 102: 0 → 1
Char. 103: 0 → 1
Char. 124: 0 → 1
Char. 127: 0 → 1
Char. 130: 0 → 1
Char. 134: 0 → 2
Char. 135: 0 → 1
Char. 141: 0 → 1
Char. 145: 0 → 1
Char. 147: 0 → 1
Char. 148: 0 → 1
Char. 151: 0 → 1
Char. 152: 0 → 1
Char. 153: 1 → 0
Char. 155: 0 → 1
Char. 158: 0 → 1
Char. 161: 0 → 1
Char. 174: 0 → 1
Char. 181: 0 → 1
Char. 188: 0 → 1
Char. 192: 1 → 0
Char. 194: 0 → 1
Char. 201: 1 → 0
Char. 202: 1 → 0
Char. 203: 1 → 2
Char. 211: 0 → 1
Char. 217: 0 → 1
Char. 219: 0 → 1
Char. 220: 0 → 1
Char. 231: 0 → 1
Char. 237: 1 → 0
Char. 240: 1 → 0
Char. 248: 0 → 1
Char. 249: 0 → 2
Char. 250: 0 → 1
Char. 252: 0 → 1
Char. 263: 0 → 1
Char. 273: 0 → 1
Char. 274: 0 → 1
Char. 276: 0 → 1
Char. 277: 0 → 1

***Acerosodontosaurus piveteaui*:**
Char. 23: 0 → 1
Char. 59: 0 → 1
Char. 78: 1 → 0
Char. 81: 1 → 0
Char. 89: 0 → 1
Char. 94: 0 → 1
Char. 128: 0 → 1
Char. 129: 0 → 1
Char. 155: 0 → 1
Char. 206: 0 → 2
Char. 208: 0 → 1
Char. 265: 0 → 1
Char. 266: 0 → 1
Char. 278: 3 → 1

***Acleistorhinus pteroticus:***
Char. 21: 0 → 1
Char. 25: 0 → 1
Char. 29: 0 → 1
Char. 30: 0 → 1
Char. 47: 0 → 1
Char. 55: 1 → 0
Char. 56: 0 → 1
Char. 57: 0 → 1
Char. 64: 0 → 1
Char. 67: 1 → 0
Char. 70: 0 → 1
Char. 79: 0 → 1
Char. 95: 0 → 1
Char. 110: 0 → 1
Char. 111: 1 → 0
Char. 113: 0 → 1
Char. 114: 0 → 1
Char. 120: 1 → 0
Char. 131: 0 → 1
Char. 137: 0 → 1
Char. 140: 0 → 2
Char. 146: 1 → 0
Char. 147: 0 → 1
Char. 169: 1 → 0
Char. 170: 0 → 1
Char. 278: 3 → 1

**Araeoscelidae:**
Char. 0: 0 → 1
Char. 5: 1 → 0
Char. 20: 1 → 0
Char. 23: 0 → 1
Char. 25: 0 → 1
Char. 27: 0 → 1
Char. 28: 0 → 1
Char. 33: 1 → 0
Char. 38: 1 → 0
Char. 40: 2 → 0
Char. 43: 0 → 1
Char. 59: 0 → 1
Char. 60: 0 → 1
Char. 72: 1 → 2
Char. 84: 1 → 0
Char. 89: 0 → 1
Char. 92: 1 → 0
Char. 106: 0 → 1
Char. 116: 1 → 0
Char. 117: 0 → 2
Char. 154: 1 → 0
Char. 159: 1 → 0
Char. 166: 1 → 0
Char. 169: 1 → 0
Char. 170: 0 → 1
Char. 193: 0 → 1
Char. 196: 1 → 0
Char. 197: 0 → 1
Char. 202: 1 → 0
Char. 207: 1 → 0
Char. 209: 1 → 0
Char. 221: 1 → 0
Char. 224: 0 → 1
Char. 239: 0 → 1
Char. 266: 0 → 1
Char. 278: 3 → 1

**Archosauriformes:**
Char. 32: 0 → 1
Char. 94: 1 → 0
Char. 112: 1 → 0
Char. 152: 0 → 1
Char. 154: 0 → 2
Char. 166: 1 → 0
Char. 171: 0 → 1
Char. 185: 0 → 1
Char. 204: 0 → 1
Char. 218: 0 → 3
Char. 224: 0 → 1
Char. 242: 0 → 1

***Australothyris smithi*:**
Char. 23: 0 → 1
Char. 25: 0 → 1
Char. 34: 0 → 1
Char. 55: 1 → 0
Char. 71: 1 → 0
Char. 85: 1 → 0
Char. 129: 0 → 1
Char. 131: 0 → 1
Char. 159: 1 → 0

***Barasaurus besairiei*:**
Char. 33: 1 → 0
Char. 75: 1 → 0
Char. 216: 0 → 1

***Bashkyroleter bashkyricus*:**
Char. 275: 1 → 0

***Bashkyroleter mesensis*:**
Char. 169: 1 → 0

***Belebey vegrandis*:**
Char. 154: 1 → 0

***Bradysaurus* spp.:**
Char. 19: 0 → 1
Char. 73: 0 → 1
Char. 79: 0 → 1
Char. 135: 1 → 0
Char. 249: 0 → 1

***Candelaria barbouri*:**
Char. 1: 0 → 1
Char. 5: 1 → 0
Char. 8: 0 → 1
Char. 15: 0 → 1
Char. 23: 0 → 1
Char. 33: 1 → 2
Char. 49: 0 → 1
Char. 55: 1 → 0
Char. 76: 0 → 1
Char. 79: 0 → 2
Char. 83: 0 → 1
Char. 88: 1 → 0
Char. 89: 0 → 1
Char. 92: 1 → 0
Char. 94: 0 → 1
Char. 95: 0 → 1
Char. 126: 0 → 1
Char. 127: 0 → 1
Char. 132: 1 → 0
Char. 154: 1 → 2
Char. 159: 1 → 0
Char. 169: 1 → 0
Char. 276: 0 → 1
Char. 277: 0 → 1

**Captorhinidae:**
Char. 3: 0 → 1
Char. 23: 0 → 1
Char. 26: 0 → 1
Char. 73: 0 → 1
Char. 75: 0 → 1
Char. 83: 0 → 1
Char. 108: 1 → 0
Char. 180: 1 → 0
Char. 183: 1 → 0
Char. 201: 1 → 0
Char. 203: 1 → 2
Char. 216: 1 → 0
Char. 237: 1 → 0
Char. 240: 1 → 0

**Caseidae:**
Char. 24: 0 → 1
Char. 36: 0 → 1
Char. 38: 1 → 0
Char. 46: 1 → 0
Char. 56: 0 → 1
Char. 170: 0 → 1
Char. 194: 0 → 1
Char. 273: 0 → 1
Char. 274: 0 → 1
Char. 278: 3 → 2

***Claudiosaurus germaini*:**
Char. 0: 0 → 1
Char. 23: 0 → 1
Char. 24: 0 → 1
Char. 27: 0 → 1
Char. 34: 0 → 1
Char. 36: 0 → 1
Char. 56: 0 → 1
Char. 59: 0 → 1
Char. 60: 0 → 1
Char. 62: 0 → 1
Char. 64: 0 → 1
Char. 70: 0 → 1
Char. 72: 1 → 2
Char. 73: 0 → 1
Char. 84: 1 → 0
Char. 89: 0 → 1
Char. 94: 0 → 1
Char. 105: 0 → 1
Char. 106: 0 → 1
Char. 109: 0 → 1
Char. 117: 0 → 2
Char. 126: 0 → 1
Char. 129: 0 → 1
Char. 130: 0 → 1
Char. 131: 0 → 1
Char. 144: 1 → 0
Char. 148: 0 → 1
Char. 154: 1 → 0
Char. 166: 1 → 0
Char. 182: 0 → 1

Char. 188: 0 → 1
Char. 190: 0 → 1
Char. 191: 0 → 1
Char. 199: 0 → 1
Char. 203: 1 → 2
Char. 204: 02 → 1
Char. 214: 0 → 1
Char. 219: 0 → 1
Char. 220: 0 → 1
Char. 234: 1 → 0
Char. 265: 0 → 1
Char. 267: 0 → 1
Char. 272: 2 → 1
Char. 275: 1 → 0
Char. 278: 3 → 0

***Colobomycter pholeter*:**
Char. 21: 0 → 1
Char. 84: 1 → 0
Char. 154: 1 → 0
Char. 167: 0 → 1
Char. 267: 0 → 1

***Delorhynchus cifelli*:**
Char. 18: 0 → 1
Char. 21: 0 → 1
Char. 24: 0 → 1
Char. 25: 0 → 1
Char. 26: 0 → 1
Char. 28: 0 → 1
Char. 29: 0 → 1
Char. 33: 1 → 2
Char. 39: 0 → 1
Char. 52: 0 → 1
Char. 57: 0 → 1
Char. 67: 1 → 0
Char. 100: 0 → 1
Char. 116: 1 → 0
Char. 117: 0 → 1
Char. 119: 1 → 0
Char. 131: 0 → 1
Char. 147: 0 → 1
Char. 156: 1 → 0
Char. 167: 0 → 1
Char. 189: 0 → 1
Char. 191: 0 → 1
Char. 196: 1 → 0
Char. 202: 1 → 0
Char. 267: 0 → 1

**Diadectomorpha:**
Char. 0: 0 → 1
Char. 64: 0 → 1
Char. 70: 0 → 1
Char. 122: 0 → 1
Char. 123: 1 → 0
Char. 146: 1 → 0
Char. 275: 1 → 0
Char. 278: 3 → 0

***Emeroleter levis*:**
Char. 51: 1 → 0

**Eosauropterygia:**
Char. 159: 1 → 0
Char. 166: 1 → 0
Char. 174: 0 → 1
Char. 194: 0 → 2
Char. 272: 1 → 0

***Eudibamus cursoris*:**
Char. 154: 1 → 2
Char. 163: 1 → 0

***Feeserpeton oklahomensis*:**
Char. 25: 0 → 1
Char. 51: 0 → 1
Char. 70: 0 → 1
Char. 157: 0 → 1
Char. 158: 0 → 1

***Hovasaurus boulei*:**
Char. 41: 0 → 1
Char. 43: 0 → 1
Char. 55: 1 → 0
Char. 59: 0 → 1
Char. 77: 0 → 2
Char. 78: 1 → 0
Char. 79: 0 → 1
Char. 89: 0 → 1
Char. 93: 1 → 0
Char. 113: 0 → 1
Char. 127: 0 → 1
Char. 135: 0 → 1
Char. 138: 0 → 1
Char. 146: 1 → 0
Char. 187: 0 → 1
Char. 188: 0 → 1
Char. 191: 0 → 1
Char. 193: 0 → 1
Char. 201: 1 → 0
Char. 206: 0 → 2
Char. 215: 0 → 1
Char. 220: 0 → 1
Char. 224: 0 → 1
Char. 265: 0 → 1
Char. 275: 1 → 0

**Kuehneosauridae:**
Char. 7: 0 → 1
Char. 24: 0 → 1
Char. 26: 0 → 1
Char. 27: 0 → 1
Char. 34: 0 → 1
Char. 36: 0 → 1
Char. 44: 1 → 0
Char. 56: 0 → 1
Char. 79: 0 → 2
Char. 82: 0 → 1
Char. 98: 1 → 0
Char. 108: 1 → 0
Char. 113: 0 → 1
Char. 128: 0 → 1
Char. 159: 1 → 0
Char. 181: 0 → 1
Char. 185: 0 → 1
Char. 206: 0 → 2
Char. 245: 0 → 1

***Lanthanosuchus watsoni*:**
Char. 29: 0 → 1
Char. 51: 0 → 1
Char. 76: 0 → 1
Char. 86: 0 → 1
Char. 95: 0 → 1
Char. 98: 1 → 0
Char. 110: 0 → 1
Char. 111: 1 → 0
Char. 113: 0 → 1
Char. 114: 0 → 1
Char. 119: 1 → 0
Char. 131: 0 → 1
Char. 137: 0 → 1
Char. 138: 0 → 1
Char. 140: 0 → 2
Char. 144: 1 → 0
Char. 147: 0 → 1
Char. 154: 1 → 2

***Macroleter poezicus*:**
Char. 9: 0 → 1
Char. 26: 0 → 1
Char. 52: 0 → 1
Char. 84: 1 → 0
Char. 87: 0 → 1
Char. 134: 0 → 2
Char. 140: 0 → 1
Char. 146: 1 → 0
Char. 169: 1 → 0
Char. 235: 0 → 1

***Mesosaurus* spp.:**
Char. 0: 0 → 1
Char. 2: 0 → 1
Char. 5: 1 → 0
Char. 6: 0 → 1
Char. 8: 0 → 1
Char. 9: 0 → 1
Char. 13: 0 → 1
Char. 19: 0 → 1
Char. 20: 1 → 0
Char. 23: 0 → 1
Char. 25: 0 → 1
Char. 26: 0 → 1
Char. 38: 1 → 0
Char. 41: 0 → 1
Char. 76: 0 → 1
Char. 83: 0 → 1
Char. 84: 1 → 0
Char. 85: 1 → 0
Char. 94: 0 → 1
Char. 107: 0 → 1
Char. 109: 0 → 1
Char. 115: 0 → 1
Char. 146: 1 → 0
Char. 148: 0 → 1
Char. 149: 1 → 0
Char. 159: 1 → 0
Char. 163: 1 → 0
Char. 164: 0 → 1
Char. 166: 1 → 0
Char. 167: 0 → 1
Char. 176: 0 → 1
Char. 183: 1 → 0
Char. 184: 0 → 1
Char. 192: 1 → 0
Char. 196: 1 → 0
Char. 199: 0 → 1
Char. 206: 0 → 1
Char. 217: 0 → 1
Char. 219: 0 → 1
Char. 220: 0 → 1
Char. 231: 0 → 1
Char. 234: 1 → 0
Char. 237: 1 → 0
Char. 240: 1 → 0
Char. 260: 2 → 0
Char. 272: 2 → 0
Char. 278: 3 → 0

***Microleter mckinzieorum*:**
Char. 0: 0 → 1
Char. 36: 0 → 1
Char. 39: 0 → 1
Char. 51: 0 → 2
Char. 56: 0 → 1
Char. 70: 0 → 1
Char. 94: 0 → 1
Char. 106: 0 → 1
Char. 166: 1 → 0
Char. 276: 0 → 1

**Millerettidae:**
Char. 5: 1 → 0
Char. 20: 1 → 0
Char. 24: 0 → 1
Char. 29: 0 → 1
Char. 33: 1 → 0
Char. 44: 1 → 0
Char. 56: 0 → 1
Char. 66: 0 → 2
Char. 67: 1 → 0
Char. 78: 1 → 0
Char. 80: 1 → 0
Char. 84: 1 → 2
Char. 88: 1 → 0
Char. 96: 1 → 0
Char. 111: 1 → 0
Char. 117: 0 → 1
Char. 120: 1 → 0
Char. 121: 0 → 1
Char. 124: 0 → 1
Char. 127: 0 → 1
Char. 135: 0 → 1
Char. 145: 0 → 1
Char. 159: 1 → 0
Char. 163: 1 → 0
Char. 166: 1 → 0
Char. 180: 1 → 0
Char. 192: 1 → 0
Char. 211: 0 → 1
Char. 230: 0 → 1
Char. 248: 0 → 1
Char. 252: 0 → 1

***Nycteroleter ineptus*:**
Char. 278: 0 → 3

***Nyctiphruretus acudens*:**
Char. 21: 0 → 1
Char. 33: 1 → 2
Char. 41: 0 → 1
Char. 81: 1 → 0
Char. 84: 1 → 2
Char. 94: 0 → 1
Char. 166: 1 → 0
Char. 167: 0 → 1
Char. 215: 1 → 0
Char. 224: 0 → 1
Char. 266: 0 → 1
Char. 272: 2 → 1
Char. 276: 0 → 1

***Orovenator mayorum*:**
Char. 0: 0 → 1
Char. 8: 0 → 1
Char. 24: 0 → 1
Char. 33: 1 → 0
Char. 36: 0 → 1
Char. 62: 0 → 1
Char. 72: 1 → 2
Char. 89: 0 → 1
Char. 92: 1 → 0
Char. 141: 0 → 1
Char. 159: 1 → 0
Char. 160: 0 → 1
Char. 165: 0 → 1
Char. 278: 3 → 1

***Owenetta* spp.:**
Char. 169: 1 → 0

***Paleothyris acadiana*:**
Char. 25: 0 → 1
Char. 38: 1 → 0
Char. 66: 1 → 2
Char. 102: 0 → 1
Char. 146: 1 → 0
Char. 239: 0 → 1

***Placodus* spp.:**
Char. 0: 1 → 2
Char. 9: 0 → 1
Char. 12: 0 → 1
Char. 13: 0 → 1
Char. 19: 0 → 1
Char. 26: 0 → 1
Char. 31: 0 → 1
Char. 44: 1 → 0
Char. 46: 1 → 0
Char. 57: 0 → 1
Char. 78: 1 → 0
Char. 93: 1 → 0
Char. 102: 1 → 2
Char. 140: 1 → 0

Char. 155: 0 → 1
Char. 163: 1 → 0
Char. 164: 0 → 1

***Procolophon* spp.:**
Char. 41: 0 → 1
Char. 69: 0 → 1
Char. 79: 0 → 1
Char. 83: 0 → 1
Char. 88: 0 → 1
Char. 117: 1 → 0
Char. 149: 1 → 0
Char. 237: 0 → 1
Char. 238: 0 → 1
Char. 272: 2 → 1

***Prolacerta broomi*:**
Char. 58: 1 → 0
Char. 66: 1 → 0
Char. 67: 1 → 0
Char. 80: 1 → 0
Char. 139: 1 → 0
Char. 192: 1 → 0
Char. 203: 1 → 2
Char. 206: 0 → 12

***Rhipaeosaurus* spp.:**
Char. 172: 0 → 1
Char. 277: 0 → 1

**Rhynchocephalia:**
Char. 0: 1 → 2
Char. 24: 0 → 1
Char. 77: 0 → 1
Char. 94: 1 → 0
Char. 139: 1 → 0
Char. 167: 1 → 0
Char. 205: 1 → 0

**Rhynchosauria:**
Char. 0: 1 → 0
Char. 7: 0 → 1
Char. 9: 0 → 1
Char. 26: 0 → 1
Char. 44: 1 → 0
Char. 68: 0 → 1
Char. 99: 1 → 0
Char. 150: 1 → 0
Char. 160: 0 → 1
Char. 161: 0 → 1
Char. 171: 0 → 2
Char. 223: 0 → 1
Char. 224: 0 → 1
Char. 241: 0 → 1

***Scutosaurus* spp.:**
Char. 175: 0 → 1
Char. 218: 0 → 1
Char. 243: 0 → 2
Char. 244: 0 → 1

***Sinosaurosphargis yunguiensis*:**
Char. 8: 0 → 1
Char. 30: 0 → 1
Char. 53: 0 → 1
Char. 82: 0 → 1
Char. 89: 1 → 0
Char. 127: 1 → 0
Char. 150: 1 → 0
Char. 154: 0 → 2
Char. 167: 1 → 0
Char. 255: 0 → 1

**Squamata:**
Char. 26: 0 → 1
Char. 45: 0 → 1
Char. 79: 0 → 2
Char. 80: 1 → 0
Char. 82: 0 → 1
Char. 92: 1 → 0
Char. 160: 0 → 1
Char. 245: 0 → 1

***Trilophosaurus buettneri*:**
Char. 5: 1 → 0
Char. 11: 0 → 1
Char. 55: 1 → 0
Char. 93: 1 → 0
Char. 104: 0 → 1
Char. 106: 0 → 1
Char. 113: 0 → 1
Char. 122: 0 → 1
Char. 144: 1 → 0
Char. 154: 0 → 1
Char. 157: 0 → 1
Char. 159: 1 → 0
Char. 177: 0 → 12
Char. 194: 0 → 1
Char. 203: 1 → 2
Char. 207: 1 → 0
Char. 208: 1 → 0
Char. 272: 1 → 0

***Youngina capensis*:**
Char. 0: 0 → 1
Char. 5: 1 → 0
Char. 21: 0 → 1
Char. 25: 0 → 1
Char. 27: 0 → 1
Char. 38: 1 → 0
Char. 40: 2 → 0
Char. 43: 0 → 1
Char. 44: 1 → 0
Char. 56: 0 → 1
Char. 59: 0 → 1
Char. 60: 0 → 1
Char. 62: 0 → 1
Char. 72: 1 → 2
Char. 75: 0 → 1
Char. 84: 1 → 0
Char. 89: 0 → 1
Char. 92: 1 → 0
Char. 109: 0 → 1
Char. 127: 0 → 1
Char. 129: 0 → 1
Char. 134: 0 → 1
Char. 135: 0 → 1
Char. 141: 0 → 1
Char. 154: 1 → 0
Char. 163: 1 → 0
Char. 170: 0 → 1
Char. 187: 0 → 1
Char. 191: 0 → 1
Char. 193: 0 → 1
Char. 201: 1 → 0
Char. 211: 0 → 1
Char. 214: 0 → 1
Char. 215: 0 → 1
Char. 219: 0 → 1
Char. 224: 0 → 1
Char. 231: 0 → 1
Char. 239: 0 → 1
Char. 265: 0 → 1
Char. 267: 0 → 1
Char. 275: 1 → 0
Char. 278: 3 → 0

**Node 50:**
Char. 0: 0 → 2
Char. 46: 1 → 0
Char. 88: 1 → 0
Char. 93: 1 → 0
Char. 176: 0 → 1
Char. 195: 0 → 2
Char. 198: 0 → 1
Char. 240: 1 → 0
Char. 246: 1 → 2
Char. 259: 0 → 1
Char. 270: 0 → 1

**Node 51:**
Char. 44: 1 → 0
Char. 65: 0 → 1
Char. 72: 1 → 2
Char. 73: 0 → 1
Char. 78: 1 → 0
Char. 131: 0 → 1
Char. 184: 0 → 1
Char. 187: 0 → 1
Char. 194: 0 → 2
Char. 201: 1 → 0
Char. 205: 0 → 1
Char. 210: 0 → 1
Char. 217: 0 → 1
Char. 220: 0 → 1
Char. 241: 0 → 1
Char. 246: 0 → 1
Char. 254: 0 → 1
Char. 255: 0 → 1
Char. 256: 0 → 1
Char. 266: 0 → 1
Char. 267: 0 → 1
Char. 268: 0 → 1
Char. 269: 0 → 1
Char. 278: 3 → 0

**Node 52:**
Char. 5: 0 → 1
Char. 20: 0 → 1
Char. 33: 0 → 1
Char. 40: 0 → 2
Char. 57: 1 → 0
Char. 67: 0 → 1
Char. 74: 0 → 1
Char. 80: 0 → 1
Char. 84: 0 → 1
Char. 88: 0 → 1
Char. 116: 0 → 1
Char. 119: 0 → 1
Char. 132: 0 → 1
Char. 149: 0 → 1
Char. 156: 0 → 1
Char. 163: 0 → 1
Char. 166: 0 → 1
Char. 169: 0 → 1
Char. 180: 0 → 1
Char. 192: 0 → 1
Char. 196: 0 → 1
Char. 201: 0 → 1
Char. 202: 0 → 1
Char. 203: 0 → 1
Char. 209: 0 → 1
Char. 221: 0 → 1
Char. 234: 0 → 1
Char. 235: 0 → 1
Char. 237: 0 → 1

**Node 53:**
Char. 25: 1 → 0
Char. 72: 0 → 1
Char. 79: 1 → 0
Char. 81: 0 → 1
Char. 93: 0 → 1
Char. 97: 0 → 1
Char. 104: 1 → 0
Char. 144: 0 → 1
Char. 173: 0 → 1
Char. 183: 0 → 1

**Node 55:**
Char. 19: 0 → 1
Char. 92: 1 → 0

**Node 56:**
Char. 4: 0 → 1
Char. 15: 0 → 1
Char. 29: 0 → 1
Char. 213: 0 → 1
Char. 226: 0 → 2
Char. 228: 0 → 1

**Node 57:**
Char. 0: 0 → 1
Char. 58: 0 → 1
Char. 59: 0 → 1
Char. 60: 0 → 1
Char. 61: 0 → 1
Char. 62: 0 → 1
Char. 66: 0 → 1
Char. 69: 0 → 1
Char. 72: 1 → 2
Char. 73: 0 → 1
Char. 89: 0 → 1
Char. 94: 0 → 1
Char. 126: 0 → 1
Char. 127: 0 → 1
Char. 129: 0 → 1
Char. 131: 0 → 1
Char. 134: 0 → 1
Char. 135: 0 → 1
Char. 141: 0 → 1
Char. 145: 0 → 1
Char. 150: 0 → 1
Char. 151: 0 → 1
Char. 154: 1 → 0
Char. 167: 0 → 1
Char. 176: 0 → 1
Char. 188: 0 → 1
Char. 190: 0 → 1
Char. 191: 0 → 1
Char. 205: 0 → 1
Char. 208: 0 → 1
Char. 214: 0 → 1
Char. 219: 0 → 1
Char. 230: 0 → 1
Char. 239: 0 → 1
Char. 260: 2 → 1
Char. 265: 0 → 1

**Node 58:**
Char. 24: 0 → 1
Char. 29: 0 → 1
Char. 67: 1 → 0
Char. 79: 0 → 1
Char. 83: 0 → 1
Char. 110: 0 → 1
Char. 111: 1 → 0
Char. 132: 1 → 0

**Node 59:**
Char. 73: 0 → 1
Char. 131: 1 → 0
Char. 205: 0 → 1
Char. 276: 0 → 1

**Node 60:**
Char. 18: 0 → 1
Char. 37: 0 → 1
Char. 100: 1 → 0
Char. 107: 0 → 1
Char. 113: 1 → 0
Char. 118: 1 → 0
Char. 125: 1 → 0
Char. 150: 0 → 1

**Node 61:**
Char. 23: 0 → 1
Char. 71: 1 → 0
Char. 102: 0 → 1
Char. 103: 0 → 1
Char. 106: 0 → 1
Char. 167: 0 → 1
Char. 214: 0 → 1
Char. 216: 1 → 0

Char. 235: 0 → 2
Char. 241: 0 → 1

**Node 62:**
Char. 113: 0 → 1
Char. 132: 1 → 0
Char. 138: 0 → 1
Char. 141: 0 → 1

**Node 63:**
Char. 44: 1 → 0
Char. 67: 1 → 0
Char. 70: 0 → 1
Char. 80: 1 → 0
Char. 117: 0 → 1
Char. 118: 0 → 1
Char. 125: 0 → 1
Char. 131: 0 → 1
Char. 147: 0 → 1
Char. 194: 0 → 1
Char. 201: 1 → 0
Char. 211: 0 → 1
Char. 234: 1 → 0
Char. 240: 1 → 0

**Node 64:**
Char. 25: 0 → 1
Char. 29: 0 → 1
Char. 38: 1 → 2
Char. 39: 0 → 1
Char. 49: 0 → 1
Char. 57: 0 → 1
Char. 66: 0 → 12
Char. 76: 0 → 1
Char. 88: 1 → 0
Char. 95: 0 → 1
Char. 111: 1 → 0
Char. 148: 0 → 1
Char. 158: 0 → 1

Char. 183: 1 → 2
Char. 215: 0 → 1
Char. 237: 1 → 0
Char. 239: 0 → 1

**Node 65:**
Char. 87: 0 → 1

**Node 66:**
Char. 25: 1 → 0
Char. 76: 1 → 0

**Node 67:**
Char. 79: 0 → 1
Char. 93: 1 → 0
Char. 133: 0 → 1

**Node 68:**
Char. 20: 1 → 0
Char. 58: 0 → 1
Char. 59: 0 → 1
Char. 60: 0 → 1
Char. 72: 1 → 2
Char. 104: 0 → 1
Char. 105: 0 → 1
Char. 106: 0 → 2
Char. 107: 0 → 1
Char. 109: 0 → 1
Char. 110: 0 → 1
Char. 146: 1 → 0
Char. 155: 0 → 2

**Node 69:**
Char. 33: 1 → 0
Char. 38: 2 → 1
Char. 39: 1 → 0
Char. 42: 0 → 1
Char. 43: 0 → 1
Char. 46: 1 → 0

Char. 48: 1 → 0
Char. 49: 1 → 0
Char. 52: 0 → 1
Char. 83: 0 → 2
Char. 84: 1 → 0
Char. 87: 0 → 1
Char. 93: 1 → 0
Char. 132: 0 → 1
Char. 161: 0 → 1
Char. 163: 1 → 0
Char. 172: 0 → 2
Char. 174: 0 → 1
Char. 188: 0 → 1
Char. 189: 0 → 1
Char. 195: 0 → 1
Char. 212: 0 → 1
Char. 221: 1 → 0
Char. 236: 0 → 1
Char. 238: 0 → 2
Char. 242: 0 → 1
Char. 274: 0 → 1
Char. 275: 1 → 0

**Node 70:**
Char. 5: 1 → 0
Char. 20: 1 → 0
Char. 33: 1 → 0
Char. 44: 1 → 0
Char. 59: 0 → 1
Char. 60: 0 → 1
Char. 66: 0 → 1
Char. 72: 1 → 2
Char. 78: 1 → 0
Char. 84: 1 → 0
Char. 88: 1 → 0
Char. 93: 1 → 0
Char. 123: 1 → 0
Char. 129: 0 → 1
Char. 154: 1 → 0
Char. 159: 1 → 0

Char. 163: 1 → 0
Char. 169: 1 → 0
Char. 170: 0 → 1
Char. 192: 1 → 0
Char. 196: 1 → 0
Char. 197: 0 → 1
Char. 202: 1 → 0
Char. 207: 1 → 0
Char. 209: 1 → 0
Char. 221: 1 → 0
Char. 234: 1 → 0

**Node 71:**
Char. 29: 0 → 1
Char. 33: 1 → 0
Char. 47: 0 → 1
Char. 57: 0 → 1
Char. 83: 0 → 1
Char. 85: 1 → 0
Char. 107: 0 → 1
Char. 110: 0 → 1
Char. 111: 1 → 0
Char. 169: 1 → 0
Char. 170: 0 → 1

**Node 72:**
Char. 2: 0 → 1
Char. 6: 0 → 1
Char. 98: 1 → 0
Char. 101: 0 → 1
Char. 113: 0 → 1
Char. 181: 0 → 1
Char. 186: 0 → 1
Char. 198: 0 → 1
Char. 206: 0 → 2
Char. 220: 0 → 1
Char. 234: 1 → 0
Char. 239: 1 → 0
Char. 240: 1 → 0

**Node 73:**
Char. 21: 0 → 1
Char. 33: 1 → 2
Char. 68: 0 → 1
Char. 102: 0 → 1
Char. 103: 0 → 1
Char. 223: 0 → 1
Char. 229: 0 → 1
Char. 235: 1 → 2

**Node 74:**
Char. 17: 0 → 1
Char. 42: 0 → 1
Char. 264: 0 → 1

**Node 75:**
Char. 41: 0 → 1
Char. 61: 1 → 2
Char. 112: 1 → 0
Char. 128: 0 → 1
Char. 138: 0 → 1
Char. 146: 1 → 0
Char. 155: 0 → 1
Char. 192: 1 → 2
Char. 224: 0 → 1
Char. 226: 0 → 1
Char. 227: 0 → 1
Char. 233: 0 → 1

**Node 76:**
Char. 61: 1 → 3
Char. 90: 0 → 1
Char. 91: 0 → 1
Char. 155: 0 → 1
Char. 209: 1 → 0

## ANALYSIS 76
## (ALL TAXA, IMPLIED WEIGHTING, K = 2.75, NO CHARACTERS 247, 251, AND 253)

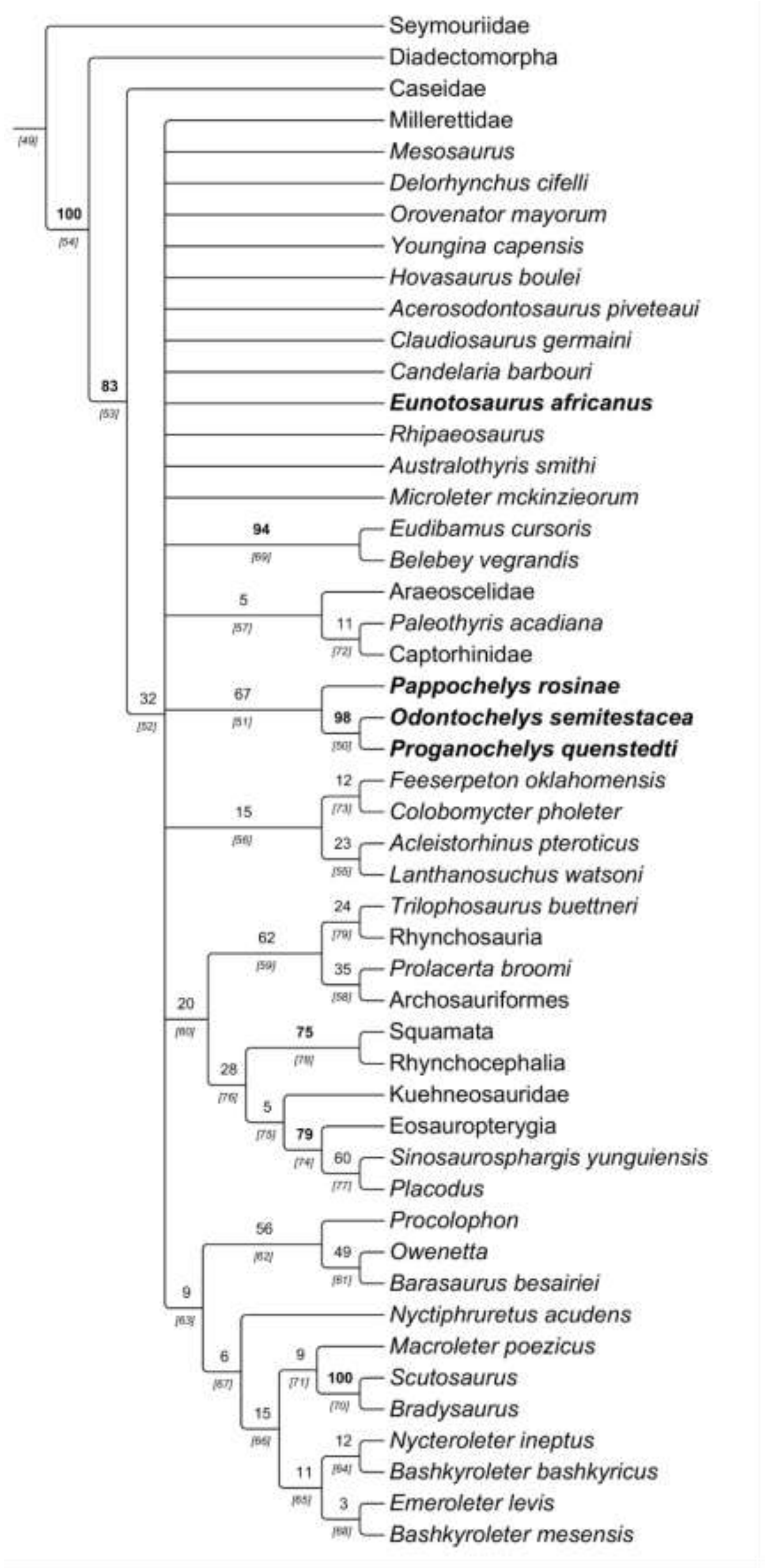

***Proganochelys quenstedti*:**
Char. 8: 0 → 1
Char. 11: 0 → 1
Char. 106: 0 → 1
Char. 108: 1 → 0
Char. 109: 0 → 1
Char. 128: 0 → 1
Char. 175: 0 → 1
Char. 202: 1 → 0
Char. 207: 1 → 0
Char. 209: 1 → 0
Char. 244: 0 → 1
Char. 248: 0 → 1
Char. 250: 0 → 1
Char. 262: 0 → 1

***Pappochelys rosinae*:**
Char. 1: 0 → 1
Char. 5: 1 → 0
Char. 12: 0 → 1
Char. 41: 0 → 1
Char. 48: 0 → 1
Char. 49: 0 → 1
Char. 55: 1 → 0
Char. 75: 0 → 1
Char. 89: 0 → 1
Char. 129: 0 → 1
Char. 169: 1 → 0
Char. 260: 2 → 0
Char. 265: 0 → 1

***Odontochelys semitestacea*:**
Char. 43: 0 → 1
Char. 112: 1 → 0
Char. 124: 0 → 1

***Eunotosaurus africanus*:**
Char. 0: 01 → 2
Char. 15: 0 → 1
Char. 19: 0 → 1
Char. 21: 0 → 1
Char. 24: 0 → 1
Char. 33: 1 → 2
Char. 40: 2 → 0
Char. 42: 0 → 1
Char. 43: 0 → 1
Char. 44: 1 → 0
Char. 50: 0 → 1
Char. 55: 1 → 0
Char. 57: 0 → 1
Char. 64: 0 → 1
Char. 73: 0 → 1
Char. 76: 0 → 1
Char. 81: 1 → 0
Char. 84: 1 → 2
Char. 89: 0 → 1
Char. 94: 0 → 1
Char. 97: 1 → 0
Char. 102: 0 → 1
Char. 103: 0 → 1
Char. 112: 1 → 0
Char. 124: 0 → 1
Char. 127: 0 → 1
Char. 130: 0 → 1
Char. 134: 0 → 2
Char. 136: 0 → 1
Char. 141: 0 → 1
Char. 145: 0 → 1
Char. 147: 0 → 1
Char. 151: 0 → 1
Char. 152: 0 → 1
Char. 153: 1 → 0
Char. 155: 0 → 1
Char. 158: 0 → 1
Char. 161: 0 → 1
Char. 174: 0 → 1
Char. 181: 0 → 1
Char. 188: 0 → 1
Char. 192: 1 → 0
Char. 194: 0 → 1
Char. 202: 1 → 0
Char. 203: 1 → 2
Char. 211: 0 → 1
Char. 217: 0 → 1
Char. 219: 0 → 1
Char. 231: 0 → 1
Char. 240: 1 → 0
Char. 248: 0 → 1
Char. 249: 0 → 2
Char. 250: 0 → 1
Char. 252: 0 → 1
Char. 263: 0 → 1
Char. 273: 0 → 1
Char. 274: 0 → 1
Char. 276: 0 → 1
Char. 277: 0 → 1

***Acerosodontosaurus piveteaui*:**
Char. 59: 0 → 1
Char. 78: 1 → 0
Char. 81: 1 → 0
Char. 89: 0 → 1
Char. 94: 0 → 1
Char. 128: 0 → 1
Char. 129: 0 → 1
Char. 155: 0 → 1
Char. 204: 2 → 0
Char. 206: 0 → 2
Char. 208: 0 → 1
Char. 265: 0 → 1
Char. 266: 0 → 1
Char. 278: 3 → 1

***Acleistorhinus pteroticus*:**
Char. 21: 0 → 1
Char. 25: 0 → 1
Char. 146: 1 → 0

**Araeoscelidae:**
Char. 28: 0 → 1
Char. 43: 0 → 1
Char. 48: 0 → 1
Char. 89: 0 → 1
Char. 106: 0 → 1
Char. 193: 0 → 1
Char. 222: 0 → 1
Char. 224: 0 → 1
Char. 266: 0 → 1
Char. 278: 3 → 1

**Archosauriformes:**
Char. 32: 0 → 1
Char. 94: 1 → 0
Char. 112: 1 → 0
Char. 152: 0 → 1
Char. 154: 01 → 2
Char. 166: 1 → 0
Char. 171: 0 → 1
Char. 185: 0 → 1
Char. 201: 0 → 1
Char. 204: 0 → 1
Char. 218: 0 → 3
Char. 224: 0 → 1
Char. 242: 0 → 1

***Australothyris smithi*:**
Char. 24: 0 → 1
Char. 25: 0 → 1
Char. 29: 0 → 1
Char. 34: 0 → 1
Char. 50: 0 → 1
Char. 55: 1 → 0
Char. 71: 1 → 0
Char. 79: 0 → 1
Char. 83: 0 → 1
Char. 85: 1 → 0
Char. 98: 1 → 0
Char. 100: 0 → 1
Char. 103: 0 → 1
Char. 110: 0 → 1
Char. 111: 1 → 0
Char. 123: 1 → 0
Char. 129: 0 → 1
Char. 131: 0 → 1
Char. 132: 1 → 0
Char. 144: 1 → 0
Char. 147: 0 → 1
Char. 148: 1 → 0
Char. 149: 1 → 0
Char. 150: 0 → 1
Char. 159: 1 → 0
Char. 163: 1 → 0
Char. 192: 1 → 0

***Barasaurus besairiei*:**
Char. 33: 1 → 0
Char. 50: 0 → 1

***Bashkyroleter bashkyricus*:**
Char. 275: 1 → 0

***Bashkyroleter mesensis*:**
Char. 12: 0 → 1
Char. 66: 1 → 0
Char. 169: 1 → 0

***Belebey vegrandis*:**
Char. 154: 1 → 0

***Bradysaurus* spp.:**
Char. 19: 0 → 1
Char. 73: 0 → 1
Char. 79: 0 → 1
Char. 135: 1 → 0
Char. 249: 0 → 1

***Candelaria barbouri*:**
Char. 1: 0 → 1
Char. 5: 1 → 0
Char. 8: 0 → 1
Char. 15: 0 → 1
Char. 33: 1 → 2
Char. 49: 0 → 1
Char. 55: 1 → 0
Char. 76: 0 → 1
Char. 79: 0 → 2
Char. 83: 0 → 1
Char. 88: 1 → 0
Char. 89: 0 → 1
Char. 92: 1 → 0
Char. 94: 0 → 1
Char. 95: 0 → 1
Char. 126: 0 → 1
Char. 127: 0 → 1
Char. 132: 1 → 0
Char. 154: 1 → 2
Char. 159: 1 → 0
Char. 169: 1 → 0
Char. 276: 0 → 1
Char. 277: 0 → 1

**Captorhinidae:**
Char. 3: 0 → 1
Char. 26: 0 → 1
Char. 50: 0 → 1
Char. 73: 0 → 1
Char. 75: 0 → 1
Char. 83: 0 → 1
Char. 108: 1 → 0
Char. 180: 1 → 0
Char. 183: 1 → 0
Char. 203: 1 → 2
Char. 216: 1 → 0
Char. 240: 1 → 0

**Caseidae:**
Char. 24: 0 → 1
Char. 36: 0 → 1
Char. 38: 1 → 0
Char. 46: 1 → 0
Char. 56: 0 → 1
Char. 170: 0 → 1
Char. 194: 0 → 1
Char. 273: 0 → 1
Char. 274: 0 → 1
Char. 278: 3 → 2

***Claudiosaurus germaini*:**
Char. 24: 0 → 1
Char. 27: 0 → 1
Char. 34: 0 → 1
Char. 36: 0 → 1
Char. 48: 0 → 1
Char. 56: 0 → 1
Char. 59: 0 → 1
Char. 60: 0 → 1
Char. 62: 0 → 1
Char. 64: 0 → 1
Char. 70: 0 → 1
Char. 72: 1 → 2
Char. 73: 0 → 1
Char. 84: 1 → 0
Char. 89: 0 → 1
Char. 94: 0 → 1
Char. 105: 0 → 1
Char. 106: 0 → 1
Char. 109: 0 → 1
Char. 117: 01 → 2
Char. 126: 0 → 1
Char. 129: 0 → 1
Char. 130: 0 → 1
Char. 131: 0 → 1
Char. 144: 1 → 0
Char. 154: 1 → 0
Char. 166: 1 → 0
Char. 182: 0 → 1
Char. 188: 0 → 1
Char. 190: 0 → 1
Char. 199: 0 → 1
Char. 201: 0 → 1
Char. 203: 1 → 2
Char. 204: 2 → 1
Char. 214: 0 → 1
Char. 219: 0 → 1
Char. 234: 1 → 0
Char. 265: 0 → 1
Char. 267: 0 → 1
Char. 272: 2 → 1
Char. 275: 1 → 0
Char. 278: 3 → 0

***Colobomycter pholeter*:**
Char. 21: 0 → 1
Char. 84: 1 → 0
Char. 154: 1 → 0
Char. 167: 0 → 1
Char. 267: 0 → 1

***Delorhynchus cifelli*:**
Char. 18: 0 → 1
Char. 21: 0 → 1
Char. 24: 0 → 1
Char. 25: 0 → 1
Char. 26: 0 → 1
Char. 28: 0 → 1
Char. 29: 0 → 1
Char. 33: 1 → 2
Char. 39: 0 → 1
Char. 48: 0 → 1
Char. 50: 0 → 1
Char. 52: 0 → 1
Char. 57: 0 → 1

Char. 100: 0 → 1
Char. 116: 1 → 0
Char. 119: 1 → 0
Char. 131: 0 → 1
Char. 147: 0 → 1
Char. 148: 1 → 0
Char. 156: 1 → 0
Char. 167: 0 → 1
Char. 189: 0 → 1
Char. 196: 1 → 0
Char. 201: 0 → 1
Char. 202: 1 → 0
Char. 267: 0 → 1

**Diadectomorpha:**
Char. 64: 0 → 1
Char. 70: 0 → 1
Char. 122: 0 → 1
Char. 123: 1 → 0
Char. 146: 1 → 0
Char. 275: 1 → 0
Char. 278: 3 → 0

***Emeroleter levis*:**
Char. 51: 1 → 0

**Eosauropterygia:**
Char. 166: 1 → 0
Char. 174: 0 → 1
Char. 178: 0 → 1
Char. 194: 0 → 2
Char. 272: 1 → 0

***Eudibamus cursoris*:**
Char. 154: 1 → 2
Char. 163: 1 → 0

***Feeserpeton oklahomensis*:**
Char. 25: 0 → 1
Char. 51: 0 → 1
Char. 157: 0 → 1
Char. 158: 0 → 1

***Hovasaurus boulei*:**
Char. 41: 0 → 1
Char. 43: 0 → 1
Char. 48: 0 → 1
Char. 55: 1 → 0
Char. 59: 0 → 1
Char. 77: 0 → 2
Char. 78: 1 → 0
Char. 79: 0 → 1
Char. 89: 0 → 1
Char. 93: 1 → 0
Char. 113: 0 → 1
Char. 127: 0 → 1
Char. 136: 0 → 1
Char. 138: 0 → 1
Char. 146: 1 → 0
Char. 148: 1 → 0
Char. 188: 0 → 1
Char. 193: 0 → 1
Char. 206: 0 → 2

Char. 215: 0 → 1
Char. 222: 0 → 1
Char. 224: 0 → 1
Char. 265: 0 → 1
Char. 275: 1 → 0

**Kuehneosauridae:**
Char. 7: 0 → 1
Char. 24: 0 → 1
Char. 27: 0 → 1
Char. 43: 1 → 0
Char. 79: 0 → 2
Char. 107: 1 → 0
Char. 140: 1 → 0
Char. 147: 1 → 0
Char. 148: 1 → 0
Char. 245: 0 → 1
Char. 278: 0 → 3

***Lanthanosuchus watsoni*:**
Char. 51: 0 → 1
Char. 76: 0 → 1
Char. 86: 0 → 1
Char. 98: 1 → 0
Char. 138: 0 → 1
Char. 144: 1 → 0
Char. 154: 1 → 2

***Macroleter poezicus*:**
Char. 9: 0 → 1
Char. 26: 0 → 1
Char. 66: 1 → 2
Char. 126: 0 → 1
Char. 134: 0 → 2
Char. 140: 0 → 1
Char. 146: 1 → 0
Char. 169: 1 → 0
Char. 204: 2 → 0

***Mesosaurus* spp.:**
Char. 2: 0 → 1
Char. 5: 1 → 0
Char. 6: 0 → 1
Char. 8: 0 → 1
Char. 9: 0 → 1
Char. 13: 0 → 1
Char. 19: 0 → 1
Char. 20: 1 → 0
Char. 25: 0 → 1
Char. 26: 0 → 1
Char. 38: 1 → 0
Char. 41: 0 → 1
Char. 48: 0 → 1
Char. 76: 0 → 1
Char. 83: 0 → 1
Char. 84: 1 → 0
Char. 85: 1 → 0
Char. 94: 0 → 1
Char. 107: 0 → 1
Char. 109: 0 → 1
Char. 112: 1 → 0
Char. 115: 0 → 1
Char. 146: 1 → 0

Char. 149: 1 → 0
Char. 159: 1 → 0
Char. 163: 1 → 0
Char. 164: 0 → 1
Char. 166: 1 → 0
Char. 167: 0 → 1
Char. 176: 0 → 1
Char. 183: 1 → 0
Char. 184: 0 → 1
Char. 192: 1 → 0
Char. 196: 1 → 0
Char. 199: 0 → 1
Char. 201: 0 → 1
Char. 206: 0 → 1
Char. 217: 0 → 1
Char. 219: 0 → 1
Char. 231: 0 → 1
Char. 234: 1 → 0
Char. 240: 1 → 0
Char. 260: 2 → 0
Char. 272: 2 → 0
Char. 278: 3 → 0

***Microleter mckinzieorum*:**
Char. 18: 0 → 1
Char. 20: 1 → 0
Char. 24: 0 → 1
Char. 29: 0 → 1
Char. 33: 1 → 0
Char. 36: 0 → 1
Char. 39: 0 → 1
Char. 50: 0 → 1
Char. 51: 0 → 2
Char. 56: 0 → 1
Char. 70: 0 → 1
Char. 76: 0 → 1
Char. 79: 0 → 1
Char. 83: 0 → 1
Char. 94: 0 → 1
Char. 106: 0 → 1
Char. 110: 0 → 1
Char. 111: 1 → 0
Char. 112: 1 → 0
Char. 132: 1 → 0
Char. 166: 1 → 0
Char. 276: 0 → 1
Char. 278: 3 → 1

**Millerettidae:**
Char. 5: 1 → 0
Char. 20: 1 → 0
Char. 24: 0 → 1
Char. 29: 0 → 1
Char. 33: 1 → 0
Char. 44: 1 → 0
Char. 50: 0 → 1
Char. 56: 0 → 1
Char. 66: 0 → 2
Char. 78: 1 → 0
Char. 80: 1 → 0
Char. 84: 1 → 2
Char. 88: 1 → 0
Char. 96: 1 → 0

Char. 111: 1 → 0
Char. 112: 1 → 0
Char. 120: 1 → 0
Char. 121: 0 → 1
Char. 124: 0 → 1
Char. 127: 0 → 1
Char. 145: 0 → 1
Char. 148: 1 → 0
Char. 159: 1 → 0
Char. 163: 1 → 0
Char. 166: 1 → 0
Char. 180: 1 → 0
Char. 192: 1 → 0
Char. 201: 0 → 1
Char. 211: 0 → 1
Char. 230: 0 → 1
Char. 248: 0 → 1
Char. 252: 0 → 1

***Nycteroleter ineptus*:**
Char. 66: 1 → 0

***Nyctiphruretus acudens*:**
Char. 21: 0 → 1
Char. 41: 0 → 1
Char. 66: 1 → 2
Char. 81: 1 → 0
Char. 83: 0 → 1
Char. 84: 1 → 2
Char. 94: 0 → 1
Char. 166: 1 → 0
Char. 207: 1 → 0
Char. 224: 0 → 1
Char. 266: 0 → 1
Char. 272: 2 → 1
Char. 276: 0 → 1

***Orovenator mayorum*:**
Char. 8: 0 → 1
Char. 24: 0 → 1
Char. 33: 1 → 0
Char. 36: 0 → 1
Char. 50: 0 → 1
Char. 62: 0 → 1
Char. 72: 1 → 2
Char. 89: 0 → 1
Char. 92: 1 → 0
Char. 136: 0 → 1
Char. 141: 0 → 1
Char. 148: 1 → 0
Char. 159: 1 → 0
Char. 160: 0 → 1
Char. 165: 0 → 1
Char. 278: 3 → 1

***Owenetta* spp.:**
Char. 142: 1 → 0
Char. 169: 1 → 0

***Paleothyris acadiana*:**
Char. 66: 1 → 2
Char. 102: 0 → 1
Char. 146: 1 → 0

***Placodus* spp.:**
Char. 0: 1 → 2
Char. 9: 0 → 1
Char. 12: 0 → 1
Char. 13: 0 → 1
Char. 19: 0 → 1
Char. 31: 0 → 1
Char. 46: 1 → 0
Char. 57: 0 → 1
Char. 78: 1 → 0
Char. 93: 1 → 0
Char. 102: 1 → 2
Char. 140: 1 → 0
Char. 155: 0 → 1
Char. 163: 1 → 0
Char. 164: 0 → 1

***Procolophon* spp.:**
Char. 41: 0 → 1
Char. 69: 0 → 1
Char. 79: 0 → 1
Char. 83: 0 → 1
Char. 86: 0 → 1
Char. 101: 0 → 1
Char. 141: 0 → 1
Char. 149: 1 → 0
Char. 203: 1 → 2
Char. 215: 0 → 1
Char. 230: 0 → 1
Char. 235: 1 → 2
Char. 238: 0 → 1
Char. 272: 2 → 1
Char. 278: 3 → 0

***Prolacerta broomi*:**
Char. 58: 1 → 0
Char. 66: 1 → 0
Char. 67: 1 → 0
Char. 80: 1 → 0
Char. 139: 1 → 0
Char. 147: 1 → 0
Char. 192: 1 → 0
Char. 203: 1 → 2
Char. 206: 0 → 12

***Rhipaeosaurus* spp.:**
Char. 120: 1 → 0
Char. 172: 0 → 1
Char. 183: 1 → 2
Char. 194: 0 → 1
Char. 207: 1 → 0
Char. 211: 0 → 1
Char. 215: 0 → 1
Char. 226: 0 → 1
Char. 234: 1 → 0
Char. 235: 1 → 0
Char. 240: 1 → 0
Char. 252: 0 → 1
Char. 277: 0 → 1

**Rhynchocephalia:**
Char. 0: 1 → 2
Char. 24: 0 → 1

Char. 77: 0 → 1
Char. 88: 1 → 0
Char. 94: 1 → 0
Char. 139: 1 → 0
Char. 167: 1 → 0
Char. 205: 1 → 0

**Rhynchosauria:**
Char. 0: 1 → 0
Char. 7: 0 → 1
Char. 9: 0 → 1
Char. 26: 0 → 1
Char. 44: 1 → 0
Char. 68: 0 → 1
Char. 99: 1 → 0
Char. 150: 1 → 0
Char. 160: 0 → 1
Char. 161: 0 → 1
Char. 171: 0 → 2
Char. 223: 0 → 1
Char. 224: 0 → 1
Char. 241: 0 → 1

***Scutosaurus* spp.:**
Char. 175: 0 → 1
Char. 190: 0 → 1
Char. 218: 0 → 1
Char. 243: 0 → 2
Char. 244: 0 → 1

***Sinosaurosphargis yunguiensis*:**
Char. 8: 0 → 1
Char. 30: 0 → 1
Char. 53: 0 → 1
Char. 89: 1 → 0
Char. 127: 1 → 0
Char. 150: 1 → 0
Char. 154: 0 → 2
Char. 167: 1 → 0
Char. 255: 0 → 1

**Squamata:**
Char. 45: 0 → 1
Char. 79: 0 → 2
Char. 80: 1 → 0
Char. 92: 1 → 0
Char. 160: 0 → 1
Char. 200: 0 → 1
Char. 245: 0 → 1

***Trilophosaurus buettneri*:**
Char. 5: 1 → 0
Char. 11: 0 → 1
Char. 55: 1 → 0
Char. 93: 1 → 0
Char. 104: 0 → 1
Char. 106: 0 → 1
Char. 113: 0 → 1
Char. 122: 0 → 1
Char. 144: 1 → 0
Char. 157: 0 → 1
Char. 159: 1 → 0
Char. 177: 0 → 12
Char. 194: 0 → 1
Char. 203: 1 → 2
Char. 207: 1 → 0
Char. 208: 1 → 0
Char. 272: 1 → 0

***Youngina capensis*:**
Char. 5: 1 → 0
Char. 21: 0 → 1
Char. 25: 0 → 1
Char. 27: 0 → 1
Char. 38: 1 → 0
Char. 40: 2 → 0
Char. 43: 0 → 1
Char. 44: 1 → 0
Char. 48: 0 → 1
Char. 56: 0 → 1
Char. 59: 0 → 1
Char. 60: 0 → 1
Char. 62: 0 → 1
Char. 72: 1 → 2
Char. 75: 0 → 1
Char. 84: 1 → 0
Char. 89: 0 → 1
Char. 92: 1 → 0
Char. 109: 0 → 1
Char. 127: 0 → 1
Char. 129: 0 → 1
Char. 134: 0 → 1
Char. 141: 0 → 1
Char. 148: 1 → 0
Char. 154: 1 → 0
Char. 163: 1 → 0
Char. 170: 0 → 1
Char. 193: 0 → 1
Char. 211: 0 → 1
Char. 214: 0 → 1
Char. 215: 0 → 1
Char. 219: 0 → 1
Char. 222: 0 → 1
Char. 224: 0 → 1
Char. 231: 0 → 1
Char. 239: 0 → 1
Char. 265: 0 → 1
Char. 267: 0 → 1
Char. 275: 1 → 0
Char. 278: 3 → 0

**Node 50:**
Char. 46: 1 → 0
Char. 50: 0 → 1
Char. 88: 1 → 0
Char. 93: 1 → 0
Char. 176: 0 → 1
Char. 195: 0 → 2
Char. 198: 0 → 1
Char. 240: 1 → 0
Char. 246: 1 → 2
Char. 259: 0 → 1
Char. 270: 0 → 1

**Node 51:**
Char. 44: 1 → 0
Char. 65: 0 → 1
Char. 72: 1 → 2
Char. 73: 0 → 1
Char. 78: 1 → 0
Char. 131: 0 → 1
Char. 184: 0 → 1
Char. 194: 0 → 2
Char. 205: 0 → 1
Char. 210: 0 → 1
Char. 217: 0 → 1
Char. 222: 0 → 1
Char. 241: 0 → 1
Char. 246: 0 → 1
Char. 254: 0 → 1
Char. 255: 0 → 1
Char. 256: 0 → 1
Char. 266: 0 → 1
Char. 267: 0 → 1
Char. 268: 0 → 1
Char. 269: 0 → 1
Char. 278: 3 → 0

**Node 52:**
Char. 5: 0 → 1
Char. 20: 0 → 1
Char. 33: 0 → 1
Char. 40: 0 → 2
Char. 57: 1 → 0
Char. 74: 0 → 1
Char. 80: 0 → 1
Char. 84: 0 → 1
Char. 88: 0 → 1
Char. 112: 0 → 1
Char. 116: 0 → 1
Char. 119: 0 → 1
Char. 132: 0 → 1
Char. 149: 0 → 1
Char. 156: 0 → 1
Char. 163: 0 → 1
Char. 166: 0 → 1
Char. 169: 0 → 1
Char. 179: 0 → 1
Char. 180: 0 → 1
Char. 192: 0 → 1
Char. 196: 0 → 1
Char. 202: 0 → 1
Char. 203: 0 → 1
Char. 204: 0 → 2
Char. 209: 0 → 1
Char. 221: 0 → 1
Char. 234: 0 → 1
Char. 235: 0 → 1

**Node 53:**
Char. 25: 1 → 0
Char. 72: 0 → 1
Char. 79: 1 → 0
Char. 81: 0 → 1
Char. 93: 0 → 1
Char. 97: 0 → 1
Char. 104: 1 → 0
Char. 144: 0 → 1
Char. 173: 0 → 1
Char. 183: 0 → 1

**Node 55:**
Char. 95: 0 → 1
Char. 113: 0 → 1
Char. 114: 0 → 1

**Node 56:**
Char. 29: 0 → 1
Char. 47: 0 → 1
Char. 48: 0 → 1
Char. 50: 0 → 1
Char. 57: 0 → 1
Char. 79: 0 → 1
Char. 110: 0 → 1
Char. 111: 1 → 0
Char. 112: 1 → 0
Char. 131: 0 → 1
Char. 137: 0 → 1
Char. 140: 0 → 2
Char. 147: 0 → 1
Char. 148: 1 → 0

**Node 57:**
Char. 5: 1 → 0
Char. 20: 1 → 0
Char. 33: 1 → 0
Char. 59: 0 → 1
Char. 60: 0 → 1
Char. 72: 1 → 2
Char. 84: 1 → 0
Char. 116: 1 → 0
Char. 154: 1 → 0
Char. 159: 1 → 0
Char. 166: 1 → 0
Char. 169: 1 → 0
Char. 170: 0 → 1
Char. 179: 1 → 0
Char. 196: 1 → 0
Char. 197: 0 → 1
Char. 202: 1 → 0
Char. 204: 2 → 0
Char. 207: 1 → 0
Char. 209: 1 → 0
Char. 221: 1 → 0

**Node 58:**
Char. 19: 0 → 1
Char. 92: 1 → 0

**Node 59:**
Char. 4: 0 → 1
Char. 15: 0 → 1
Char. 29: 0 → 1
Char. 213: 0 → 1
Char. 226: 0 → 2
Char. 228: 0 → 1

**Node 60:**
Char. 43: 0 → 1
Char. 58: 0 → 1
Char. 59: 0 → 1
Char. 60: 0 → 1
Char. 61: 0 → 1
Char. 62: 0 → 1
Char. 66: 0 → 1
Char. 69: 0 → 1
Char. 72: 1 → 2
Char. 73: 0 → 1
Char. 89: 0 → 1
Char. 94: 0 → 1
Char. 107: 0 → 1
Char. 126: 0 → 1
Char. 127: 0 → 1
Char. 129: 0 → 1
Char. 131: 0 → 1
Char. 134: 0 → 1
Char. 140: 0 → 1
Char. 141: 0 → 1
Char. 145: 0 → 1
Char. 147: 0 → 1
Char. 150: 0 → 1
Char. 151: 0 → 1
Char. 167: 0 → 1
Char. 176: 0 → 1
Char. 188: 0 → 1
Char. 190: 0 → 1
Char. 205: 0 → 1
Char. 208: 0 → 1
Char. 214: 0 → 1
Char. 219: 0 → 1
Char. 230: 0 → 1
Char. 239: 0 → 1
Char. 260: 2 → 1
Char. 265: 0 → 1
Char. 278: 3 → 0

**Node 61:**
Char. 73: 0 → 1
Char. 205: 0 → 1
Char. 273: 0 → 1
Char. 276: 0 → 1

**Node 62:**
Char. 18: 0 → 1
Char. 37: 0 → 1
Char. 103: 0 → 1
Char. 106: 0 → 1
Char. 107: 0 → 1
Char. 126: 0 → 1
Char. 150: 0 → 1

**Node 63:**
Char. 29: 0 → 1
Char. 38: 1 → 2
Char. 39: 0 → 1
Char. 44: 1 → 0
Char. 49: 0 → 1
Char. 57: 0 → 1
Char. 66: 0 → 1
Char. 76: 0 → 1
Char. 80: 1 → 0
Char. 95: 0 → 1
Char. 111: 1 → 0

Char. 120: 1 → 0
Char. 137: 0 → 1
Char. 147: 0 → 1
Char. 157: 0 → 1
Char. 158: 0 → 1
Char. 179: 1 → 0
Char. 183: 1 → 2
Char. 186: 0 → 1
Char. 194: 0 → 1
Char. 197: 0 → 1
Char. 209: 1 → 0
Char. 211: 0 → 1
Char. 234: 1 → 0
Char. 240: 1 → 0
Char. 252: 0 → 1

**Node 65:**
Char. 133: 0 → 1

**Node 66:**
Char. 100: 0 → 1
Char. 113: 0 → 1

**Node 67:**
Char. 50: 0 → 1
Char. 70: 0 → 1
Char. 118: 0 → 1
Char. 125: 0 → 1

**Node 69:**
Char. 20: 1 → 0
Char. 38: 1 → 2
Char. 39: 0 → 1
Char. 57: 0 → 1
Char. 58: 0 → 1
Char. 59: 0 → 1
Char. 60: 0 → 1
Char. 72: 1 → 2
Char. 83: 0 → 1
Char. 85: 1 → 0
Char. 88: 1 → 0
Char. 95: 0 → 1
Char. 104: 0 → 1
Char. 105: 0 → 1
Char. 106: 0 → 2
Char. 107: 0 → 1
Char. 109: 0 → 1
Char. 110: 0 → 1
Char. 146: 1 → 0
Char. 155: 0 → 2
Char. 183: 1 → 2

**Node 70:**
Char. 23: 0 → 1
Char. 33: 1 → 0
Char. 38: 2 → 1
Char. 39: 1 → 0
Char. 42: 0 → 1
Char. 43: 0 → 1
Char. 46: 1 → 0
Char. 49: 1 → 0
Char. 71: 1 → 0
Char. 83: 0 → 2
Char. 101: 0 → 1
Char. 103: 0 → 1
Char. 106: 0 → 1
Char. 161: 0 → 1
Char. 163: 1 → 0
Char. 172: 0 → 2
Char. 174: 0 → 1
Char. 188: 0 → 1
Char. 203: 1 → 2
Char. 212: 0 → 1
Char. 216: 1 → 0
Char. 221: 1 → 0
Char. 236: 0 → 1
Char. 238: 0 → 2
Char. 241: 0 → 1
Char. 242: 0 → 1
Char. 273: 0 → 1
Char. 274: 0 → 1
Char. 275: 1 → 0

**Node 71:**
Char. 52: 0 → 1
Char. 75: 0 → 1
Char. 84: 1 → 0
Char. 121: 0 → 1
Char. 180: 1 → 0
Char. 230: 0 → 1

**Node 72:**
Char. 44: 1 → 0
Char. 66: 0 → 1
Char. 78: 1 → 0
Char. 88: 1 → 0
Char. 93: 1 → 0
Char. 112: 1 → 0
Char. 123: 1 → 0
Char. 129: 0 → 1
Char. 163: 1 → 0
Char. 192: 1 → 0
Char. 234: 1 → 0

**Node 73:**
Char. 33: 1 → 0
Char. 83: 0 → 1
Char. 85: 1 → 0
Char. 107: 0 → 1

**Node 74:**
Char. 2: 0 → 1
Char. 6: 0 → 1
Char. 186: 0 → 1
Char. 198: 0 → 1
Char. 200: 0 → 1
Char. 220: 0 → 1
Char. 239: 1 → 0

**Node 75:**
Char. 48: 0 → 1
Char. 98: 1 → 0
Char. 113: 0 → 1
Char. 181: 0 → 1
Char. 206: 0 → 2

**Node 76:**
Char. 35: 0 → 1
Char. 102: 0 → 1
Char. 103: 0 → 1
Char. 184: 0 → 1
Char. 201: 0 → 1
Char. 210: 0 → 1
Char. 223: 0 → 1
Char. 229: 0 → 1

**Node 77:**
Char. 17: 0 → 1
Char. 42: 0 → 1
Char. 264: 0 → 1

**Node 78:**
Char. 41: 0 → 1
Char. 61: 1 → 2
Char. 112: 1 → 0
Char. 138: 0 → 1
Char. 146: 1 → 0
Char. 155: 0 → 1
Char. 192: 1 → 2
Char. 224: 0 → 1
Char. 226: 0 → 1
Char. 227: 0 → 1
Char. 233: 0 → 1
Char. 267: 01 → 2

**Node 79:**
Char. 61: 1 → 3
Char. 90: 0 → 1
Char. 91: 0 → 1
Char. 155: 0 → 1
Char. 209: 1 → 0

**ANALYSIS 77**
**(ALL TAXA, NO CHARACTERS 174, 247, 251, AND 253)**

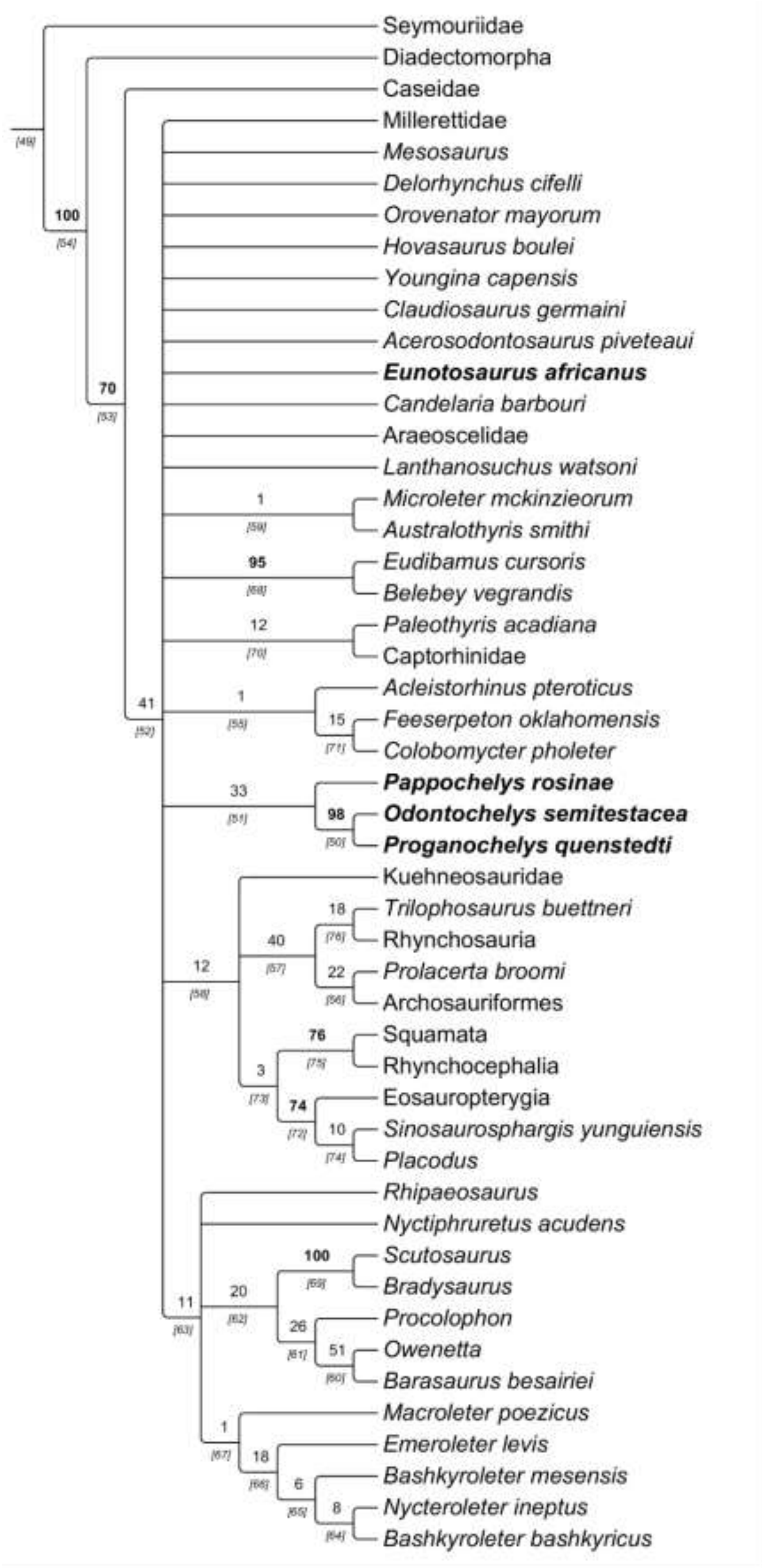

***Proganochelys quenstedti*:**
Char. 8: 0 → 1
Char. 11: 0 → 1
Char. 106: 0 → 1
Char. 108: 1 → 0
Char. 109: 0 → 1
Char. 128: 0 → 1
Char. 175: 0 → 1
Char. 202: 1 → 0
Char. 207: 1 → 0
Char. 209: 1 → 0
Char. 244: 0 → 1
Char. 248: 0 → 1
Char. 250: 0 → 1
Char. 262: 0 → 1

***Pappochelys rosinae*:**
Char. 1: 0 → 1
Char. 5: 1 → 0
Char. 12: 0 → 1
Char. 23: 0 → 1
Char. 41: 0 → 1
Char. 48: 0 → 1
Char. 49: 0 → 1
Char. 55: 1 → 0
Char. 67: 1 → 0
Char. 75: 0 → 1
Char. 89: 0 → 1
Char. 129: 0 → 1
Char. 169: 1 → 0
Char. 260: 2 → 0
Char. 265: 0 → 1

***Odontochelys semitestacea*:**
Char. 43: 0 → 1
Char. 112: 1 → 0
Char. 124: 0 → 1

***Eunotosaurus africanus*:**
Char. 0: 01 → 2
Char. 15: 0 → 1
Char. 19: 0 → 1
Char. 21: 0 → 1
Char. 24: 0 → 1
Char. 33: 1 → 2
Char. 40: 2 → 0
Char. 42: 0 → 1
Char. 43: 0 → 1
Char. 44: 1 → 0
Char. 50: 0 → 1
Char. 55: 1 → 0
Char. 57: 0 → 1
Char. 64: 0 → 1
Char. 67: 1 → 0
Char. 73: 0 → 1
Char. 76: 0 → 1
Char. 81: 1 → 0
Char. 84: 1 → 2
Char. 89: 0 → 1
Char. 94: 0 → 1
Char. 97: 1 → 0
Char. 102: 0 → 1
Char. 103: 0 → 1
Char. 112: 1 → 0
Char. 124: 0 → 1
Char. 127: 0 → 1
Char. 130: 0 → 1
Char. 134: 0 → 2
Char. 135: 0 → 1
Char. 136: 0 → 1
Char. 141: 0 → 1
Char. 145: 0 → 1
Char. 147: 0 → 1
Char. 148: 0 → 1
Char. 151: 0 → 1
Char. 152: 0 → 1
Char. 153: 1 → 0
Char. 155: 0 → 1
Char. 158: 0 → 1
Char. 161: 0 → 1
Char. 181: 0 → 1
Char. 188: 0 → 1
Char. 192: 1 → 0
Char. 194: 0 → 1
Char. 201: 1 → 0
Char. 202: 1 → 0
Char. 203: 1 → 2
Char. 211: 0 → 1
Char. 217: 0 → 1
Char. 219: 0 → 1
Char. 220: 0 → 1
Char. 231: 0 → 1
Char. 237: 1 → 0
Char. 240: 1 → 0
Char. 248: 0 → 1
Char. 249: 0 → 2
Char. 250: 0 → 1
Char. 252: 0 → 1
Char. 263: 0 → 1
Char. 273: 0 → 1
Char. 274: 0 → 1
Char. 276: 0 → 1
Char. 277: 0 → 1

***Acerosodontosaurus piveteaui*:**
Char. 23: 0 → 1
Char. 59: 0 → 1
Char. 78: 1 → 0
Char. 81: 1 → 0
Char. 89: 0 → 1
Char. 94: 0 → 1
Char. 128: 0 → 1
Char. 129: 0 → 1
Char. 155: 0 → 1
Char. 206: 0 → 2
Char. 208: 0 → 1
Char. 265: 0 → 1
Char. 266: 0 → 1
Char. 278: 3 → 1

***Acleistorhinus pteroticus:***
Char. 30: 0 → 1
Char. 55: 1 → 0
Char. 56: 0 → 1
Char. 64: 0 → 1
Char. 95: 0 → 1
Char. 113: 0 → 1
Char. 114: 0 → 1
Char. 146: 1 → 0
Char. 278: 3 → 1

**Araeoscelidae:**
Char. 5: 1 → 0
Char. 20: 1 → 0
Char. 23: 0 → 1
Char. 27: 0 → 1
Char. 28: 0 → 1
Char. 33: 1 → 0
Char. 38: 1 → 0
Char. 40: 2 → 0
Char. 43: 0 → 1
Char. 48: 0 → 1
Char. 59: 0 → 1
Char. 60: 0 → 1
Char. 72: 1 → 2
Char. 84: 1 → 0
Char. 89: 0 → 1
Char. 92: 1 → 0
Char. 106: 0 → 1
Char. 116: 1 → 0
Char. 117: 01 → 2
Char. 154: 1 → 0
Char. 159: 1 → 0
Char. 166: 1 → 0
Char. 169: 1 → 0
Char. 170: 0 → 1
Char. 193: 0 → 1
Char. 196: 1 → 0
Char. 197: 0 → 1
Char. 202: 1 → 0
Char. 207: 1 → 0
Char. 209: 1 → 0
Char. 221: 1 → 0
Char. 224: 0 → 1
Char. 239: 0 → 1
Char. 266: 0 → 1
Char. 278: 3 → 1

**Archosauriformes:**
Char. 32: 0 → 1
Char. 94: 1 → 0
Char. 112: 1 → 0
Char. 152: 0 → 1
Char. 154: 0 → 2
Char. 166: 1 → 0
Char. 171: 0 → 1
Char. 185: 0 → 1
Char. 204: 0 → 1
Char. 218: 0 → 3
Char. 224: 0 → 1
Char. 242: 0 → 1

***Australothyris smithi*:**
Char. 23: 0 → 1
Char. 34: 0 → 1
Char. 55: 1 → 0
Char. 71: 1 → 0
Char. 85: 1 → 0
Char. 129: 0 → 1
Char. 131: 0 → 1
Char. 159: 1 → 0

***Barasaurus besairiei*:**
Char. 33: 1 → 0
Char. 75: 1 → 0
Char. 216: 0 → 1

***Bashkyroleter bashkyricus*:**
Char. 275: 1 → 0

***Bashkyroleter mesensis*:**
Char. 169: 1 → 0

***Belebey vegrandis*:**
Char. 154: 1 → 0

***Bradysaurus* spp.:**
Char. 19: 0 → 1
Char. 73: 0 → 1
Char. 79: 0 → 1
Char. 135: 1 → 0
Char. 249: 0 → 1

***Candelaria barbouri*:**
Char. 1: 0 → 1
Char. 5: 1 → 0
Char. 8: 0 → 1
Char. 15: 0 → 1
Char. 23: 0 → 1
Char. 33: 1 → 2
Char. 49: 0 → 1
Char. 55: 1 → 0
Char. 76: 0 → 1
Char. 79: 0 → 2
Char. 83: 0 → 1
Char. 88: 1 → 0
Char. 89: 0 → 1
Char. 92: 1 → 0
Char. 94: 0 → 1
Char. 95: 0 → 1
Char. 126: 0 → 1
Char. 127: 0 → 1
Char. 132: 1 → 0
Char. 154: 1 → 2
Char. 159: 1 → 0
Char. 169: 1 → 0
Char. 276: 0 → 1
Char. 277: 0 → 1

**Captorhinidae:**
Char. 3: 0 → 1
Char. 23: 0 → 1
Char. 26: 0 → 1
Char. 50: 0 → 1
Char. 73: 0 → 1
Char. 75: 0 → 1
Char. 83: 0 → 1
Char. 108: 1 → 0
Char. 180: 1 → 0
Char. 183: 1 → 0
Char. 201: 1 → 0
Char. 203: 1 → 2
Char. 216: 1 → 0
Char. 237: 1 → 0
Char. 240: 1 → 0

**Caseidae:**
Char. 24: 0 → 1
Char. 36: 0 → 1
Char. 38: 1 → 0
Char. 46: 1 → 0
Char. 56: 0 → 1
Char. 170: 0 → 1
Char. 194: 0 → 1
Char. 273: 0 → 1
Char. 274: 0 → 1
Char. 278: 3 → 2

***Claudiosaurus germaini*:**
Char. 23: 0 → 1
Char. 24: 0 → 1
Char. 27: 0 → 1
Char. 34: 0 → 1
Char. 36: 0 → 1
Char. 48: 0 → 1
Char. 56: 0 → 1
Char. 59: 0 → 1
Char. 60: 0 → 1
Char. 62: 0 → 1
Char. 64: 0 → 1
Char. 70: 0 → 1
Char. 72: 1 → 2
Char. 73: 0 → 1
Char. 84: 1 → 0
Char. 89: 0 → 1
Char. 94: 0 → 1
Char. 105: 0 → 1
Char. 106: 0 → 1
Char. 109: 0 → 1
Char. 117: 01 → 2
Char. 126: 0 → 1
Char. 129: 0 → 1
Char. 130: 0 → 1
Char. 131: 0 → 1
Char. 144: 1 → 0
Char. 148: 0 → 1
Char. 154: 1 → 0
Char. 166: 1 → 0
Char. 182: 0 → 1
Char. 188: 0 → 1
Char. 190: 0 → 1
Char. 191: 0 → 1
Char. 199: 0 → 1
Char. 203: 1 → 2
Char. 204: 02 → 1
Char. 214: 0 → 1
Char. 219: 0 → 1
Char. 220: 0 → 1
Char. 234: 1 → 0
Char. 265: 0 → 1
Char. 267: 0 → 1
Char. 272: 2 → 1

Char. 275: 1 → 0
Char. 278: 3 → 0

***Colobomycter pholeter*:**
Char. 84: 1 → 0
Char. 154: 1 → 0
Char. 167: 0 → 1
Char. 267: 0 → 1

***Delorhynchus cifelli*:**
Char. 18: 0 → 1
Char. 21: 0 → 1
Char. 24: 0 → 1
Char. 26: 0 → 1
Char. 28: 0 → 1
Char. 29: 0 → 1
Char. 33: 1 → 2
Char. 39: 0 → 1
Char. 48: 0 → 1
Char. 50: 0 → 1
Char. 52: 0 → 1
Char. 57: 0 → 1
Char. 67: 1 → 0
Char. 100: 0 → 1
Char. 116: 1 → 0
Char. 119: 1 → 0
Char. 131: 0 → 1
Char. 147: 0 → 1
Char. 156: 1 → 0
Char. 167: 0 → 1
Char. 189: 0 → 1
Char. 191: 0 → 1
Char. 196: 1 → 0
Char. 202: 1 → 0
Char. 267: 0 → 1

**Diadectomorpha:**
Char. 64: 0 → 1
Char. 70: 0 → 1
Char. 122: 0 → 1
Char. 123: 1 → 0
Char. 146: 1 → 0
Char. 275: 1 → 0
Char. 278: 3 → 0

***Emeroleter levis*:**
Char. 51: 1 → 0

**Eosauropterygia:**
Char. 159: 1 → 0
Char. 166: 1 → 0
Char. 194: 0 → 2
Char. 272: 1 → 0

***Eudibamus cursoris*:**
Char. 154: 1 → 2
Char. 163: 1 → 0

***Feeserpeton oklahomensis*:**
Char. 51: 0 → 1
Char. 157: 0 → 1
Char. 158: 0 → 1

***Hovasaurus boulei*:**
Char. 41: 0 → 1
Char. 43: 0 → 1
Char. 48: 0 → 1
Char. 55: 1 → 0
Char. 59: 0 → 1
Char. 77: 0 → 2
Char. 78: 1 → 0
Char. 79: 0 → 1
Char. 89: 0 → 1
Char. 93: 1 → 0
Char. 113: 0 → 1
Char. 127: 0 → 1
Char. 135: 0 → 1
Char. 136: 0 → 1
Char. 138: 0 → 1
Char. 146: 1 → 0
Char. 187: 0 → 1
Char. 188: 0 → 1
Char. 191: 0 → 1
Char. 193: 0 → 1
Char. 201: 1 → 0
Char. 206: 0 → 2
Char. 215: 0 → 1
Char. 220: 0 → 1
Char. 224: 0 → 1
Char. 265: 0 → 1
Char. 275: 1 → 0

**Kuehneosauridae:**
Char. 7: 0 → 1
Char. 24: 0 → 1
Char. 26: 0 → 1
Char. 27: 0 → 1
Char. 34: 0 → 1
Char. 36: 0 → 1
Char. 44: 1 → 0
Char. 48: 0 → 1
Char. 56: 0 → 1
Char. 79: 0 → 2
Char. 82: 0 → 1
Char. 98: 1 → 0
Char. 108: 1 → 0
Char. 113: 0 → 1
Char. 128: 0 → 1
Char. 159: 1 → 0
Char. 181: 0 → 1
Char. 185: 0 → 1
Char. 206: 0 → 2
Char. 245: 0 → 1

***Lanthanosuchus watsoni*:**
Char. 29: 0 → 1
Char. 50: 0 → 1
Char. 51: 0 → 1
Char. 76: 0 → 1
Char. 86: 0 → 1
Char. 95: 0 → 1
Char. 98: 1 → 0
Char. 110: 0 → 1
Char. 111: 1 → 0
Char. 113: 0 → 1
Char. 114: 0 → 1
Char. 119: 1 → 0
Char. 131: 0 → 1
Char. 137: 0 → 1
Char. 138: 0 → 1
Char. 140: 0 → 2
Char. 144: 1 → 0
Char. 147: 0 → 1
Char. 154: 1 → 2

***Macroleter poezicus*:**
Char. 52: 0 → 1
Char. 66: 1 → 2
Char. 75: 0 → 1
Char. 84: 1 → 0
Char. 87: 0 → 1
Char. 110: 0 → 1
Char. 121: 0 → 1
Char. 140: 0 → 1
Char. 146: 1 → 0
Char. 169: 1 → 0
Char. 235: 0 → 1

***Mesosaurus* spp.:**
Char. 2: 0 → 1
Char. 5: 1 → 0
Char. 6: 0 → 1
Char. 8: 0 → 1
Char. 9: 0 → 1
Char. 13: 0 → 1
Char. 19: 0 → 1
Char. 20: 1 → 0
Char. 23: 0 → 1
Char. 26: 0 → 1
Char. 38: 1 → 0
Char. 41: 0 → 1
Char. 48: 0 → 1
Char. 76: 0 → 1
Char. 83: 0 → 1
Char. 84: 1 → 0
Char. 85: 1 → 0
Char. 94: 0 → 1
Char. 107: 0 → 1
Char. 109: 0 → 1
Char. 112: 1 → 0
Char. 115: 0 → 1
Char. 146: 1 → 0
Char. 148: 0 → 1
Char. 149: 1 → 0
Char. 159: 1 → 0
Char. 163: 1 → 0
Char. 164: 0 → 1
Char. 166: 1 → 0
Char. 167: 0 → 1
Char. 176: 0 → 1
Char. 183: 1 → 0
Char. 184: 0 → 1
Char. 192: 1 → 0
Char. 196: 1 → 0
Char. 199: 0 → 1
Char. 206: 0 → 1
Char. 217: 0 → 1
Char. 219: 0 → 1
Char. 220: 0 → 1
Char. 231: 0 → 1
Char. 234: 1 → 0
Char. 237: 1 → 0
Char. 240: 1 → 0
Char. 260: 2 → 0
Char. 272: 2 → 0
Char. 278: 3 → 0

***Microleter mckinzieorum*:**
Char. 36: 0 → 1
Char. 39: 0 → 1
Char. 51: 0 → 2
Char. 56: 0 → 1
Char. 70: 0 → 1
Char. 94: 0 → 1
Char. 106: 0 → 1
Char. 112: 1 → 0
Char. 166: 1 → 0
Char. 276: 0 → 1

**Millerettidae:**
Char. 5: 1 → 0
Char. 20: 1 → 0
Char. 24: 0 → 1
Char. 29: 0 → 1
Char. 33: 1 → 0
Char. 44: 1 → 0
Char. 50: 0 → 1
Char. 56: 0 → 1
Char. 66: 0 → 2
Char. 67: 1 → 0
Char. 78: 1 → 0
Char. 80: 1 → 0
Char. 84: 1 → 2
Char. 88: 1 → 0
Char. 96: 1 → 0
Char. 111: 1 → 0
Char. 112: 1 → 0
Char. 120: 1 → 0
Char. 121: 0 → 1
Char. 124: 0 → 1
Char. 127: 0 → 1
Char. 135: 0 → 1
Char. 145: 0 → 1
Char. 159: 1 → 0
Char. 163: 1 → 0
Char. 166: 1 → 0
Char. 180: 1 → 0
Char. 192: 1 → 0
Char. 211: 0 → 1
Char. 230: 0 → 1
Char. 248: 0 → 1
Char. 252: 0 → 1

***Nycteroleter ineptus*:**
Char. 278: 0 → 3

***Nyctiphruretus acudens*:**
Char. 21: 0 → 1
Char. 33: 1 → 2
Char. 41: 0 → 1
Char. 66: 1 → 2
Char. 81: 1 → 0
Char. 83: 0 → 1
Char. 84: 1 → 2
Char. 94: 0 → 1
Char. 166: 1 → 0
Char. 167: 0 → 1
Char. 207: 1 → 0
Char. 215: 1 → 0
Char. 224: 0 → 1
Char. 226: 1 → 0
Char. 266: 0 → 1
Char. 272: 2 → 1
Char. 276: 0 → 1

***Orovenator mayorum*:**
Char. 8: 0 → 1
Char. 24: 0 → 1
Char. 33: 1 → 0
Char. 36: 0 → 1
Char. 50: 0 → 1
Char. 62: 0 → 1
Char. 72: 1 → 2
Char. 89: 0 → 1
Char. 92: 1 → 0
Char. 136: 0 → 1
Char. 141: 0 → 1
Char. 159: 1 → 0
Char. 160: 0 → 1
Char. 165: 0 → 1
Char. 278: 3 → 1

***Owenetta* spp.:**
Char. 169: 1 → 0

***Paleothyris acadiana*:**
Char. 38: 1 → 0
Char. 66: 1 → 2
Char. 102: 0 → 1
Char. 146: 1 → 0
Char. 239: 0 → 1

***Placodus* spp.:**
Char. 0: 1 → 2
Char. 9: 0 → 1
Char. 12: 0 → 1
Char. 13: 0 → 1
Char. 19: 0 → 1
Char. 26: 0 → 1
Char. 31: 0 → 1
Char. 44: 1 → 0
Char. 46: 1 → 0
Char. 57: 0 → 1
Char. 78: 1 → 0
Char. 93: 1 → 0
Char. 102: 1 → 2
Char. 140: 1 → 0
Char. 155: 0 → 1
Char. 163: 1 → 0
Char. 164: 0 → 1

***Procolophon* spp.:**
Char. 41: 0 → 1
Char. 69: 0 → 1
Char. 79: 0 → 1

Char. 83: 0 → 1
Char. 88: 0 → 1
Char. 117: 1 → 0
Char. 149: 1 → 0
Char. 237: 0 → 1
Char. 238: 0 → 1
Char. 272: 2 → 1
Char. 278: 3 → 0

***Prolacerta broomi*:**
Char. 58: 1 → 0
Char. 66: 1 → 0
Char. 67: 1 → 0
Char. 80: 1 → 0
Char. 139: 1 → 0
Char. 192: 1 → 0
Char. 203: 1 → 2
Char. 206: 0 → 12

***Rhipaeosaurus* spp.:**
Char. 172: 0 → 1
Char. 186: 1 → 0
Char. 207: 1 → 0
Char. 277: 0 → 1

**Rhynchocephalia:**
Char. 0: 1 → 2
Char. 24: 0 → 1
Char. 77: 0 → 1
Char. 94: 1 → 0
Char. 139: 1 → 0
Char. 167: 1 → 0
Char. 205: 1 → 0

**Rhynchosauria:**
Char. 0: 1 → 0
Char. 7: 0 → 1
Char. 9: 0 → 1
Char. 26: 0 → 1
Char. 44: 1 → 0
Char. 68: 0 → 1
Char. 99: 1 → 0
Char. 150: 1 → 0
Char. 160: 0 → 1
Char. 161: 0 → 1
Char. 171: 0 → 2
Char. 223: 0 → 1
Char. 224: 0 → 1
Char. 241: 0 → 1

***Scutosaurus* spp.:**
Char. 175: 0 → 1
Char. 218: 0 → 1
Char. 243: 0 → 2
Char. 244: 0 → 1

***Sinosaurosphargis yunguiensis*:**
Char. 8: 0 → 1
Char. 30: 0 → 1
Char. 53: 0 → 1
Char. 82: 0 → 1
Char. 89: 1 → 0
Char. 127: 1 → 0
Char. 150: 1 → 0
Char. 154: 0 → 2
Char. 167: 1 → 0
Char. 255: 0 → 1

**Squamata:**
Char. 26: 0 → 1
Char. 45: 0 → 1
Char. 79: 0 → 2
Char. 80: 1 → 0
Char. 82: 0 → 1
Char. 92: 1 → 0
Char. 160: 0 → 1
Char. 245: 0 → 1

***Trilophosaurus buettneri*:**
Char. 5: 1 → 0
Char. 11: 0 → 1
Char. 55: 1 → 0
Char. 93: 1 → 0
Char. 104: 0 → 1
Char. 106: 0 → 1
Char. 113: 0 → 1
Char. 122: 0 → 1
Char. 144: 1 → 0
Char. 154: 0 → 1
Char. 157: 0 → 1
Char. 159: 1 → 0
Char. 177: 0 → 12
Char. 194: 0 → 1
Char. 203: 1 → 2
Char. 207: 1 → 0
Char. 208: 1 → 0
Char. 272: 1 → 0

***Youngina capensis*:**
Char. 5: 1 → 0
Char. 21: 0 → 1
Char. 27: 0 → 1
Char. 38: 1 → 0
Char. 40: 2 → 0
Char. 43: 0 → 1
Char. 44: 1 → 0
Char. 48: 0 → 1
Char. 56: 0 → 1
Char. 59: 0 → 1
Char. 60: 0 → 1
Char. 62: 0 → 1
Char. 72: 1 → 2
Char. 75: 0 → 1
Char. 84: 1 → 0
Char. 89: 0 → 1
Char. 92: 1 → 0
Char. 109: 0 → 1
Char. 127: 0 → 1
Char. 129: 0 → 1
Char. 134: 0 → 1
Char. 135: 0 → 1
Char. 141: 0 → 1
Char. 154: 1 → 0
Char. 163: 1 → 0
Char. 170: 0 → 1
Char. 187: 0 → 1
Char. 191: 0 → 1
Char. 193: 0 → 1
Char. 201: 1 → 0
Char. 211: 0 → 1
Char. 214: 0 → 1
Char. 215: 0 → 1
Char. 219: 0 → 1
Char. 224: 0 → 1
Char. 231: 0 → 1
Char. 239: 0 → 1
Char. 265: 0 → 1
Char. 267: 0 → 1
Char. 275: 1 → 0
Char. 278: 3 → 0

**Node 50:**
Char. 46: 1 → 0
Char. 50: 0 → 1
Char. 88: 1 → 0
Char. 93: 1 → 0
Char. 176: 0 → 1
Char. 195: 0 → 2
Char. 198: 0 → 1
Char. 240: 1 → 0
Char. 246: 1 → 2
Char. 259: 0 → 1
Char. 270: 0 → 1

**Node 51:**
Char. 44: 1 → 0
Char. 65: 0 → 1
Char. 72: 1 → 2
Char. 73: 0 → 1
Char. 78: 1 → 0
Char. 131: 0 → 1
Char. 184: 0 → 1
Char. 187: 0 → 1
Char. 194: 0 → 2
Char. 201: 1 → 0
Char. 205: 0 → 1
Char. 210: 0 → 1
Char. 217: 0 → 1
Char. 220: 0 → 1
Char. 241: 0 → 1
Char. 246: 0 → 1
Char. 254: 0 → 1
Char. 255: 0 → 1
Char. 256: 0 → 1
Char. 266: 0 → 1
Char. 267: 0 → 1
Char. 268: 0 → 1
Char. 269: 0 → 1
Char. 278: 3 → 0

**Node 52:**
Char. 5: 0 → 1
Char. 20: 0 → 1
Char. 33: 0 → 1
Char. 40: 0 → 2
Char. 57: 1 → 0
Char. 67: 0 → 1
Char. 74: 0 → 1
Char. 80: 0 → 1
Char. 84: 0 → 1
Char. 88: 0 → 1
Char. 112: 0 → 1
Char. 116: 0 → 1
Char. 119: 0 → 1
Char. 132: 0 → 1
Char. 149: 0 → 1
Char. 156: 0 → 1
Char. 163: 0 → 1
Char. 166: 0 → 1
Char. 169: 0 → 1
Char. 180: 0 → 1
Char. 192: 0 → 1
Char. 196: 0 → 1
Char. 201: 0 → 1
Char. 202: 0 → 1
Char. 203: 0 → 1
Char. 209: 0 → 1
Char. 221: 0 → 1
Char. 234: 0 → 1
Char. 235: 0 → 1
Char. 237: 0 → 1

**Node 53:**
Char. 72: 0 → 1
Char. 79: 1 → 0
Char. 81: 0 → 1
Char. 93: 0 → 1
Char. 97: 0 → 1
Char. 104: 1 → 0
Char. 144: 0 → 1
Char. 173: 0 → 1
Char. 183: 0 → 1

**Node 55:**
Char. 29: 0 → 1
Char. 47: 0 → 1
Char. 48: 0 → 1
Char. 50: 0 → 1
Char. 57: 0 → 1
Char. 67: 1 → 0
Char. 79: 0 → 1
Char. 110: 0 → 1
Char. 111: 1 → 0
Char. 112: 1 → 0
Char. 131: 0 → 1
Char. 137: 0 → 1
Char. 140: 0 → 2
Char. 147: 0 → 1
Char. 169: 1 → 0
Char. 170: 0 → 1

**Node 56:**
Char. 19: 0 → 1
Char. 92: 1 → 0

**Node 57:**
Char. 4: 0 → 1
Char. 15: 0 → 1
Char. 29: 0 → 1
Char. 213: 0 → 1
Char. 226: 0 → 2
Char. 228: 0 → 1

**Node 58:**
Char. 58: 0 → 1
Char. 59: 0 → 1
Char. 60: 0 → 1
Char. 61: 0 → 1
Char. 62: 0 → 1
Char. 66: 0 → 1
Char. 69: 0 → 1
Char. 72: 1 → 2
Char. 73: 0 → 1
Char. 89: 0 → 1
Char. 94: 0 → 1
Char. 126: 0 → 1
Char. 127: 0 → 1
Char. 129: 0 → 1
Char. 131: 0 → 1
Char. 134: 0 → 1
Char. 135: 0 → 1
Char. 141: 0 → 1
Char. 145: 0 → 1
Char. 150: 0 → 1
Char. 151: 0 → 1
Char. 154: 1 → 0
Char. 167: 0 → 1
Char. 176: 0 → 1
Char. 188: 0 → 1
Char. 190: 0 → 1
Char. 191: 0 → 1
Char. 205: 0 → 1
Char. 208: 0 → 1
Char. 214: 0 → 1
Char. 219: 0 → 1
Char. 230: 0 → 1
Char. 239: 0 → 1
Char. 260: 2 → 1
Char. 265: 0 → 1

**Node 59:**
Char. 24: 0 → 1
Char. 29: 0 → 1
Char. 50: 0 → 1
Char. 67: 1 → 0
Char. 79: 0 → 1
Char. 83: 0 → 1
Char. 110: 0 → 1
Char. 111: 1 → 0
Char. 132: 1 → 0

**Node 60:**
Char. 73: 0 → 1
Char. 131: 1 → 0
Char. 205: 0 → 1
Char. 239: 0 → 1
Char. 276: 0 → 1

**Node 61:**
Char. 18: 0 → 1
Char. 37: 0 → 1
Char. 48: 0 → 1
Char. 107: 0 → 1

Char. 118: 1 → 0
Char. 125: 1 → 0
Char. 126: 0 → 1
Char. 132: 1 → 0
Char. 150: 0 → 1

**Node 62:**
Char. 23: 0 → 1
Char. 71: 1 → 0
Char. 75: 0 → 1
Char. 102: 0 → 1
Char. 103: 0 → 1
Char. 106: 0 → 1
Char. 110: 0 → 1
Char. 155: 0 → 1
Char. 167: 0 → 1
Char. 214: 0 → 1
Char. 216: 1 → 0
Char. 235: 0 → 2
Char. 241: 0 → 1

**Node 63:**
Char. 29: 0 → 1
Char. 38: 1 → 2
Char. 39: 0 → 1
Char. 44: 1 → 0
Char. 49: 0 → 1
Char. 50: 0 → 1
Char. 51: 0 → 1
Char. 57: 0 → 1
Char. 66: 0 → 1
Char. 67: 1 → 0
Char. 70: 0 → 1
Char. 76: 0 → 1
Char. 80: 1 → 0
Char. 88: 1 → 0
Char. 95: 0 → 1
Char. 111: 1 → 0
Char. 118: 0 → 1
Char. 120: 1 → 0
Char. 125: 0 → 1
Char. 131: 0 → 1
Char. 135: 0 → 1
Char. 137: 0 → 1
Char. 147: 0 → 1
Char. 148: 0 → 1
Char. 157: 0 → 1
Char. 158: 0 → 1
Char. 183: 1 → 2
Char. 186: 0 → 1
Char. 194: 0 → 1
Char. 197: 0 → 1
Char. 201: 1 → 0
Char. 209: 1 → 0
Char. 211: 0 → 1
Char. 215: 0 → 1
Char. 226: 0 → 1
Char. 234: 1 → 0
Char. 235: 1 → 0
Char. 237: 1 → 0
Char. 240: 1 → 0
Char. 252: 0 → 1

**Node 64:**
Char. 87: 0 → 1
Char. 110: 0 → 1

**Node 65:**
Char. 76: 1 → 0

**Node 66:**
Char. 79: 0 → 1
Char. 86: 0 → 1
Char. 93: 1 → 0
Char. 133: 0 → 1

**Node 67:**
Char. 48: 0 → 1
Char. 100: 0 → 1
Char. 113: 0 → 1
Char. 132: 1 → 0
Char. 147: 1 → 2
Char. 154: 1 → 0
Char. 239: 0 → 1
Char. 278: 3 → 0

**Node 68:**
Char. 20: 1 → 0
Char. 38: 1 → 2
Char. 39: 0 → 1
Char. 57: 0 → 1
Char. 58: 0 → 1
Char. 59: 0 → 1
Char. 60: 0 → 1
Char. 72: 1 → 2
Char. 83: 0 → 1
Char. 85: 1 → 0
Char. 88: 1 → 0
Char. 95: 0 → 1
Char. 104: 0 → 1
Char. 105: 0 → 1
Char. 106: 0 → 2
Char. 107: 0 → 1
Char. 109: 0 → 1
Char. 110: 0 → 1
Char. 146: 1 → 0
Char. 148: 0 → 1
Char. 155: 0 → 2
Char. 183: 1 → 2

**Node 69:**
Char. 33: 1 → 0
Char. 38: 2 → 1
Char. 39: 1 → 0
Char. 42: 0 → 1
Char. 43: 0 → 1
Char. 46: 1 → 0
Char. 49: 1 → 0
Char. 52: 0 → 1
Char. 83: 0 → 2
Char. 84: 1 → 0
Char. 87: 0 → 1
Char. 93: 1 → 0
Char. 100: 0 → 1
Char. 113: 0 → 1
Char. 143: 0 → 1
Char. 161: 0 → 1
Char. 163: 1 → 0
Char. 172: 0 → 2
Char. 188: 0 → 1
Char. 189: 0 → 1
Char. 195: 0 → 1
Char. 212: 0 → 1
Char. 221: 1 → 0
Char. 236: 0 → 1
Char. 238: 0 → 2
Char. 242: 0 → 1
Char. 245: 0 → 1
Char. 274: 0 → 1
Char. 275: 1 → 0

**Node 70:**
Char. 5: 1 → 0
Char. 20: 1 → 0
Char. 33: 1 → 0
Char. 44: 1 → 0
Char. 59: 0 → 1
Char. 60: 0 → 1
Char. 66: 0 → 1
Char. 72: 1 → 2
Char. 78: 1 → 0
Char. 84: 1 → 0
Char. 88: 1 → 0
Char. 93: 1 → 0
Char. 112: 1 → 0
Char. 123: 1 → 0
Char. 129: 0 → 1
Char. 154: 1 → 0
Char. 159: 1 → 0
Char. 163: 1 → 0
Char. 169: 1 → 0
Char. 170: 0 → 1
Char. 192: 1 → 0
Char. 196: 1 → 0
Char. 197: 0 → 1
Char. 202: 1 → 0
Char. 207: 1 → 0
Char. 209: 1 → 0
Char. 221: 1 → 0
Char. 234: 1 → 0

**Node 71:**
Char. 33: 1 → 0
Char. 83: 0 → 1
Char. 85: 1 → 0
Char. 107: 0 → 1

**Node 72:**
Char. 2: 0 → 1
Char. 6: 0 → 1
Char. 98: 1 → 0
Char. 101: 0 → 1
Char. 113: 0 → 1
Char. 181: 0 → 1
Char. 186: 0 → 1
Char. 198: 0 → 1
Char. 206: 0 → 2
Char. 220: 0 → 1
Char. 234: 1 → 0
Char. 239: 1 → 0
Char. 240: 1 → 0

**Node 73:**
Char. 21: 0 → 1
Char. 33: 1 → 2
Char. 68: 0 → 1
Char. 102: 0 → 1
Char. 103: 0 → 1
Char. 223: 0 → 1
Char. 229: 0 → 1
Char. 235: 1 → 2

**Node 74:**
Char. 17: 0 → 1
Char. 42: 0 → 1
Char. 264: 0 → 1

**Node 75:**
Char. 41: 0 → 1
Char. 61: 1 → 2
Char. 112: 1 → 0
Char. 128: 0 → 1
Char. 138: 0 → 1
Char. 146: 1 → 0
Char. 155: 0 → 1
Char. 192: 1 → 2
Char. 224: 0 → 1
Char. 226: 0 → 1
Char. 227: 0 → 1
Char. 233: 0 → 1

**Node 76:**
Char. 61: 1 → 3
Char. 90: 0 → 1
Char. 91: 0 → 1
Char. 155: 0 → 1
Char. 209: 1 → 0

**ANALYSIS 78**

**(ALL TAXA, IMPLIED WEIGHTING, K = 2.75, NO CHARACTERS 174, 247, 251, AND 253)**

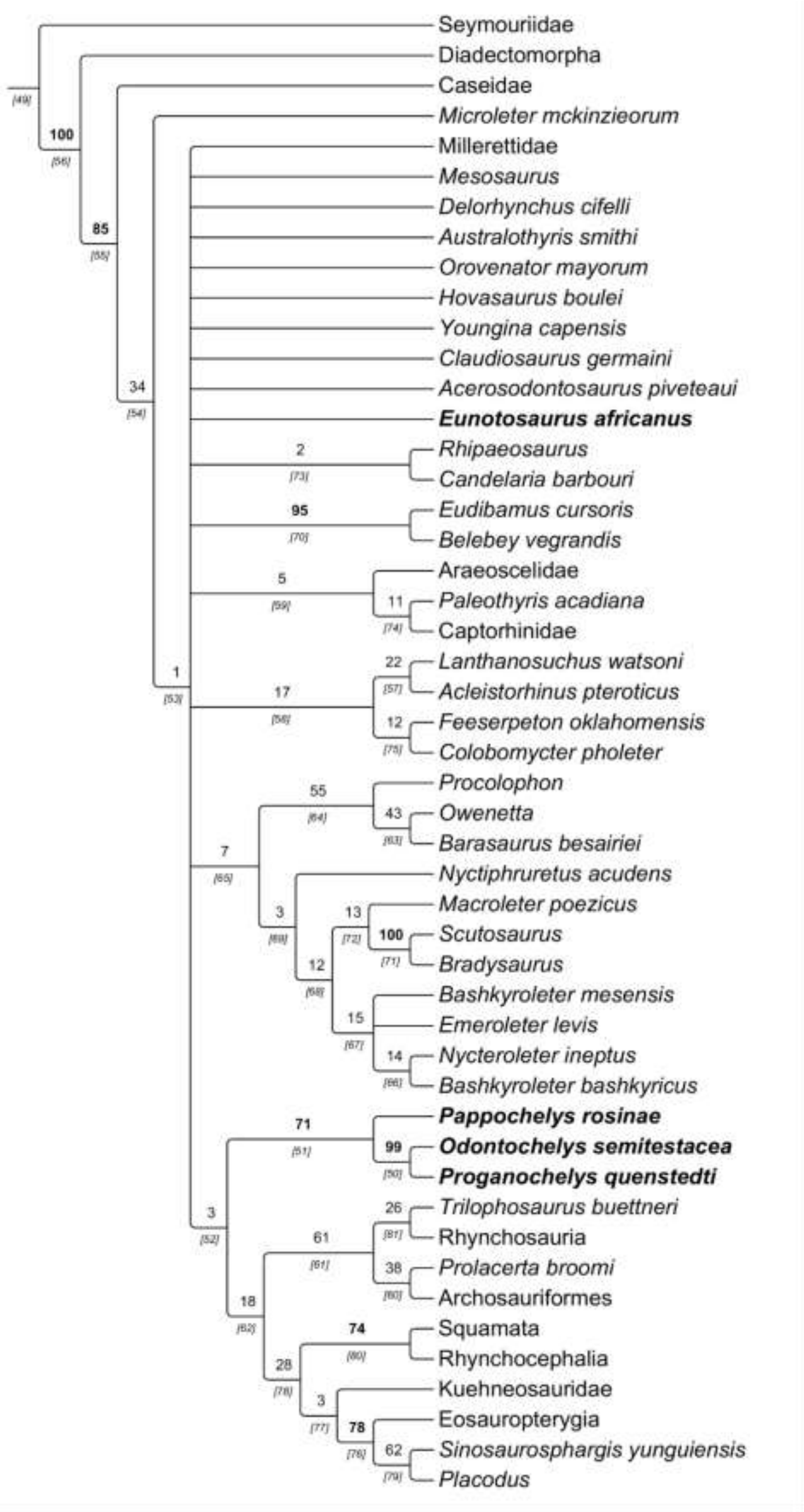

***Proganochelys quenstedti*:**
Char. 8: 0 → 1
Char. 11: 0 → 1
Char. 106: 0 → 1
Char. 108: 1 → 0
Char. 128: 0 → 1
Char. 175: 0 → 1
Char. 202: 1 → 0
Char. 207: 1 → 0
Char. 209: 1 → 0
Char. 244: 0 → 1
Char. 248: 0 → 1
Char. 250: 0 → 1
Char. 262: 0 → 1

***Pappochelys rosinae*:**
Char. 1: 0 → 1
Char. 5: 1 → 0
Char. 12: 0 → 1
Char. 41: 0 → 1
Char. 49: 0 → 1
Char. 55: 1 → 0
Char. 67: 1 → 0
Char. 169: 1 → 0
Char. 260: 2 → 0

***Odontochelys semitestacea*:**
Char. 43: 0 → 1
Char. 112: 1 → 0
Char. 124: 0 → 1
Char. 126: 1 → 0

***Eunotosaurus africanus*:**
Char. 0: 01 → 2
Char. 15: 0 → 1
Char. 19: 0 → 1
Char. 21: 0 → 1
Char. 23: 1 → 0
Char. 24: 0 → 1
Char. 33: 1 → 2
Char. 40: 2 → 0
Char. 42: 0 → 1
Char. 43: 0 → 1
Char. 44: 1 → 0
Char. 50: 0 → 1
Char. 55: 1 → 0
Char. 57: 0 → 1
Char. 64: 0 → 1
Char. 67: 1 → 0
Char. 73: 0 → 1
Char. 76: 0 → 1
Char. 81: 1 → 0
Char. 84: 1 → 2
Char. 89: 0 → 1
Char. 94: 0 → 1
Char. 97: 1 → 0
Char. 102: 0 → 1
Char. 103: 0 → 1
Char. 112: 1 → 0
Char. 124: 0 → 1
Char. 127: 0 → 1
Char. 130: 0 → 1
Char. 134: 0 → 2
Char. 135: 0 → 1
Char. 136: 0 → 1
Char. 141: 0 → 1
Char. 145: 0 → 1
Char. 147: 0 → 1
Char. 151: 0 → 1
Char. 152: 0 → 1
Char. 155: 0 → 1
Char. 158: 0 → 1
Char. 161: 0 → 1
Char. 181: 0 → 1
Char. 188: 0 → 1
Char. 192: 1 → 0
Char. 194: 0 → 1
Char. 202: 1 → 0
Char. 203: 1 → 2
Char. 211: 0 → 1
Char. 217: 0 → 1
Char. 219: 0 → 1
Char. 231: 0 → 1
Char. 240: 1 → 0
Char. 248: 0 → 1
Char. 249: 0 → 2
Char. 250: 0 → 1
Char. 252: 0 → 1
Char. 263: 0 → 1
Char. 273: 0 → 1
Char. 274: 0 → 1
Char. 276: 0 → 1
Char. 277: 0 → 1

***Acerosodontosaurus piveteaui*:**
Char. 59: 0 → 1
Char. 78: 1 → 0
Char. 81: 1 → 0
Char. 89: 0 → 1
Char. 94: 0 → 1
Char. 128: 0 → 1
Char. 129: 0 → 1
Char. 155: 0 → 1
Char. 204: 2 → 0
Char. 206: 0 → 2
Char. 208: 0 → 1
Char. 265: 0 → 1
Char. 266: 0 → 1
Char. 278: 3 → 1

***Acleistorhinus pteroticus*:**
Char. 21: 0 → 1
Char. 146: 1 → 0

**Araeoscelidae:**
Char. 28: 0 → 1
Char. 43: 0 → 1
Char. 89: 0 → 1
Char. 106: 0 → 1
Char. 193: 0 → 1
Char. 222: 0 → 1
Char. 224: 0 → 1
Char. 266: 0 → 1
Char. 278: 3 → 1

**Archosauriformes:**
Char. 32: 0 → 1
Char. 94: 1 → 0
Char. 112: 1 → 0
Char. 152: 0 → 1
Char. 154: 01 → 2
Char. 166: 1 → 0
Char. 171: 0 → 1
Char. 185: 0 → 1
Char. 201: 0 → 1
Char. 204: 0 → 1
Char. 218: 0 → 3
Char. 242: 0 → 1

***Australothyris smithi*:**
Char. 24: 0 → 1
Char. 29: 0 → 1
Char. 34: 0 → 1
Char. 50: 0 → 1
Char. 55: 1 → 0
Char. 67: 1 → 0
Char. 71: 1 → 0
Char. 79: 0 → 1
Char. 83: 0 → 1
Char. 85: 1 → 0
Char. 98: 1 → 0
Char. 100: 0 → 1
Char. 103: 0 → 1
Char. 110: 0 → 1
Char. 111: 1 → 0
Char. 123: 1 → 0
Char. 129: 0 → 1
Char. 131: 0 → 1
Char. 132: 1 → 0
Char. 144: 1 → 0
Char. 147: 0 → 1
Char. 149: 1 → 0
Char. 150: 0 → 1
Char. 159: 1 → 0
Char. 163: 1 → 0
Char. 192: 1 → 0

***Barasaurus besairiei*:**
Char. 33: 1 → 0
Char. 50: 0 → 1

***Bashkyroleter bashkyricus*:**
Char. 275: 1 → 0

***Bashkyroleter mesensis*:**
Char. 12: 0 → 1
Char. 66: 1 → 0
Char. 169: 1 → 0

***Belebey vegrandis*:**
Char. 154: 1 → 0

***Bradysaurus* spp.:**
Char. 19: 0 → 1
Char. 73: 0 → 1
Char. 79: 0 → 1
Char. 135: 1 → 0
Char. 249: 0 → 1

***Candelaria barbouri*:**
Char. 89: 0 → 1
Char. 159: 1 → 0

**Captorhinidae:**
Char. 3: 0 → 1
Char. 26: 0 → 1
Char. 50: 0 → 1
Char. 73: 0 → 1
Char. 75: 0 → 1
Char. 83: 0 → 1
Char. 108: 1 → 0
Char. 180: 1 → 0
Char. 183: 1 → 0
Char. 203: 1 → 2
Char. 216: 1 → 0
Char. 240: 1 → 0

**Caseidae:**
Char. 38: 1 → 0
Char. 46: 1 → 0
Char. 170: 0 → 1
Char. 194: 0 → 1
Char. 273: 0 → 1
Char. 274: 0 → 1
Char. 278: 3 → 2

***Claudiosaurus germaini*:**
Char. 24: 0 → 1
Char. 27: 0 → 1
Char. 34: 0 → 1
Char. 36: 0 → 1
Char. 56: 0 → 1
Char. 59: 0 → 1
Char. 60: 0 → 1
Char. 62: 0 → 1
Char. 64: 0 → 1
Char. 70: 0 → 1
Char. 72: 1 → 2
Char. 73: 0 → 1
Char. 84: 1 → 0
Char. 89: 0 → 1
Char. 94: 0 → 1
Char. 105: 0 → 1
Char. 106: 0 → 1
Char. 109: 0 → 1
Char. 117: 01 → 2
Char. 126: 0 → 1
Char. 129: 0 → 1
Char. 130: 0 → 1
Char. 131: 0 → 1
Char. 144: 1 → 0
Char. 154: 1 → 0
Char. 166: 1 → 0
Char. 182: 0 → 1
Char. 188: 0 → 1
Char. 190: 0 → 1
Char. 191: 0 → 1
Char. 199: 0 → 1
Char. 201: 0 → 1
Char. 203: 1 → 2
Char. 204: 2 → 1
Char. 214: 0 → 1
Char. 219: 0 → 1
Char. 234: 1 → 0
Char. 237: 0 → 1
Char. 265: 0 → 1
Char. 267: 0 → 1
Char. 272: 2 → 1
Char. 275: 1 → 0
Char. 278: 3 → 0

***Colobomycter pholeter*:**
Char. 21: 0 → 1
Char. 84: 1 → 0
Char. 154: 1 → 0
Char. 167: 0 → 1
Char. 267: 0 → 1

***Delorhynchus cifelli*:**
Char. 18: 0 → 1
Char. 21: 0 → 1
Char. 23: 1 → 0
Char. 24: 0 → 1
Char. 26: 0 → 1
Char. 28: 0 → 1
Char. 29: 0 → 1
Char. 33: 1 → 2
Char. 39: 0 → 1
Char. 50: 0 → 1
Char. 52: 0 → 1
Char. 57: 0 → 1
Char. 67: 1 → 0
Char. 100: 0 → 1
Char. 116: 1 → 0
Char. 119: 1 → 0
Char. 131: 0 → 1
Char. 147: 0 → 1
Char. 156: 1 → 0
Char. 167: 0 → 1
Char. 189: 0 → 1
Char. 191: 0 → 1
Char. 196: 1 → 0
Char. 201: 0 → 1
Char. 202: 1 → 0
Char. 267: 0 → 1

**Diadectomorpha:**
Char. 64: 0 → 1
Char. 70: 0 → 1
Char. 122: 0 → 1
Char. 123: 1 → 0
Char. 146: 1 → 0
Char. 275: 1 → 0
Char. 278: 3 → 0

***Emeroleter levis*:**
Char. 51: 1 → 0

**Eosauropterygia:**
Char. 166: 1 → 0
Char. 178: 0 → 1
Char. 194: 0 → 2
Char. 272: 1 → 0

***Eudibamus cursoris*:**
Char. 154: 1 → 2
Char. 163: 1 → 0

***Feeserpeton oklahomensis*:**
Char. 51: 0 → 1
Char. 157: 0 → 1
Char. 158: 0 → 1

***Hovasaurus boulei*:**
Char. 41: 0 → 1
Char. 43: 0 → 1
Char. 55: 1 → 0
Char. 59: 0 → 1
Char. 77: 0 → 2
Char. 78: 1 → 0
Char. 79: 0 → 1
Char. 89: 0 → 1
Char. 93: 1 → 0
Char. 113: 0 → 1
Char. 127: 0 → 1
Char. 135: 0 → 1
Char. 136: 0 → 1
Char. 138: 0 → 1
Char. 146: 1 → 0
Char. 187: 0 → 1
Char. 188: 0 → 1
Char. 191: 0 → 1
Char. 193: 0 → 1
Char. 206: 0 → 2
Char. 215: 0 → 1
Char. 222: 0 → 1
Char. 224: 0 → 1
Char. 237: 0 → 1
Char. 265: 0 → 1
Char. 275: 1 → 0

**Kuehneosauridae:**
Char. 7: 0 → 1
Char. 21: 1 → 0
Char. 24: 0 → 1
Char. 27: 0 → 1
Char. 43: 1 → 0
Char. 79: 0 → 2
Char. 107: 1 → 0
Char. 140: 1 → 0
Char. 147: 1 → 0
Char. 148: 1 → 0
Char. 245: 0 → 1
Char. 272: 1 → 2
Char. 278: 0 → 3

***Lanthanosuchus watsoni*:**
Char. 51: 0 → 1
Char. 76: 0 → 1
Char. 86: 0 → 1
Char. 98: 1 → 0
Char. 138: 0 → 1
Char. 144: 1 → 0
Char. 154: 1 → 2

***Macroleter poezicus*:**
Char. 9: 0 → 1
Char. 26: 0 → 1
Char. 66: 1 → 2
Char. 126: 0 → 1
Char. 134: 0 → 2
Char. 140: 0 → 1
Char. 146: 1 → 0
Char. 169: 1 → 0
Char. 204: 2 → 0

***Mesosaurus* spp.:**
Char. 2: 0 → 1
Char. 5: 1 → 0
Char. 6: 0 → 1
Char. 8: 0 → 1
Char. 9: 0 → 1
Char. 13: 0 → 1
Char. 19: 0 → 1
Char. 20: 1 → 0
Char. 26: 0 → 1
Char. 38: 1 → 0
Char. 41: 0 → 1
Char. 76: 0 → 1
Char. 83: 0 → 1
Char. 84: 1 → 0
Char. 85: 1 → 0
Char. 94: 0 → 1
Char. 107: 0 → 1
Char. 109: 0 → 1
Char. 112: 1 → 0
Char. 115: 0 → 1
Char. 146: 1 → 0
Char. 149: 1 → 0
Char. 159: 1 → 0
Char. 163: 1 → 0
Char. 164: 0 → 1
Char. 166: 1 → 0
Char. 167: 0 → 1
Char. 176: 0 → 1
Char. 183: 1 → 0
Char. 184: 0 → 1
Char. 192: 1 → 0
Char. 196: 1 → 0
Char. 199: 0 → 1
Char. 201: 0 → 1
Char. 206: 0 → 1
Char. 217: 0 → 1
Char. 219: 0 → 1
Char. 231: 0 → 1
Char. 234: 1 → 0
Char. 240: 1 → 0
Char. 260: 2 → 0
Char. 272: 2 → 0
Char. 278: 3 → 0

***Microleter mckinzieorum*:**
Char. 18: 0 → 1
Char. 29: 0 → 1
Char. 39: 0 → 1
Char. 70: 0 → 1
Char. 76: 0 → 1
Char. 83: 0 → 1
Char. 94: 0 → 1
Char. 106: 0 → 1
Char. 110: 0 → 1
Char. 278: 3 → 1

**Millerettidae:**
Char. 5: 1 → 0
Char. 20: 1 → 0
Char. 23: 1 → 0
Char. 24: 0 → 1
Char. 29: 0 → 1
Char. 33: 1 → 0
Char. 44: 1 → 0
Char. 50: 0 → 1
Char. 56: 0 → 1
Char. 66: 0 → 2
Char. 67: 1 → 0
Char. 78: 1 → 0
Char. 80: 1 → 0
Char. 84: 1 → 2
Char. 88: 1 → 0
Char. 96: 1 → 0
Char. 111: 1 → 0
Char. 112: 1 → 0
Char. 120: 1 → 0
Char. 121: 0 → 1
Char. 124: 0 → 1
Char. 127: 0 → 1
Char. 135: 0 → 1
Char. 145: 0 → 1
Char. 159: 1 → 0
Char. 163: 1 → 0
Char. 166: 1 → 0
Char. 180: 1 → 0
Char. 192: 1 → 0
Char. 201: 0 → 1
Char. 211: 0 → 1
Char. 230: 0 → 1
Char. 248: 0 → 1
Char. 252: 0 → 1

***Nycteroleter ineptus*:**
Char. 66: 1 → 0
Char. 278: 0 → 3

***Nyctiphruretus acudens*:**
Char. 21: 0 → 1
Char. 41: 0 → 1
Char. 66: 1 → 2
Char. 81: 1 → 0
Char. 83: 0 → 1
Char. 84: 1 → 2
Char. 94: 0 → 1
Char. 166: 1 → 0
Char. 207: 1 → 0
Char. 224: 0 → 1
Char. 266: 0 → 1
Char. 272: 2 → 1
Char. 276: 0 → 1

***Orovenator mayorum*:**
Char. 8: 0 → 1
Char. 23: 1 → 0
Char. 24: 0 → 1
Char. 33: 1 → 0
Char. 36: 0 → 1
Char. 50: 0 → 1
Char. 62: 0 → 1
Char. 72: 1 → 2
Char. 89: 0 → 1
Char. 92: 1 → 0
Char. 136: 0 → 1
Char. 141: 0 → 1
Char. 159: 1 → 0
Char. 160: 0 → 1
Char. 165: 0 → 1
Char. 278: 3 → 1

***Owenetta* spp.:**
Char. 142: 1 → 0
Char. 169: 1 → 0

***Paleothyris acadiana*:**
Char. 23: 1 → 0
Char. 66: 1 → 2
Char. 102: 0 → 1
Char. 146: 1 → 0

***Placodus* spp.:**
Char. 0: 1 → 2
Char. 9: 0 → 1
Char. 12: 0 → 1
Char. 13: 0 → 1
Char. 19: 0 → 1
Char. 31: 0 → 1
Char. 46: 1 → 0
Char. 57: 0 → 1
Char. 78: 1 → 0
Char. 93: 1 → 0
Char. 102: 1 → 2
Char. 140: 1 → 0
Char. 155: 0 → 1
Char. 163: 1 → 0
Char. 164: 0 → 1

***Procolophon* spp.:**
Char. 41: 0 → 1
Char. 69: 0 → 1
Char. 79: 0 → 1
Char. 83: 0 → 1
Char. 86: 0 → 1
Char. 101: 0 → 1
Char. 141: 0 → 1
Char. 149: 1 → 0
Char. 203: 1 → 2
Char. 215: 0 → 1
Char. 230: 0 → 1
Char. 235: 1 → 2
Char. 237: 0 → 1
Char. 238: 0 → 1
Char. 272: 2 → 1
Char. 278: 3 → 0

***Prolacerta broomi*:**
Char. 58: 1 → 0
Char. 66: 1 → 0
Char. 67: 1 → 0
Char. 80: 1 → 0
Char. 139: 1 → 0
Char. 147: 1 → 0
Char. 192: 1 → 0
Char. 203: 1 → 2
Char. 206: 0 → 12

***Rhipaeosaurus* spp.:**
Char. 172: 0 → 1

**Rhynchocephalia:**
Char. 0: 1 → 2
Char. 23: 1 → 0
Char. 24: 0 → 1
Char. 77: 0 → 1
Char. 88: 1 → 0
Char. 94: 1 → 0
Char. 139: 1 → 0
Char. 167: 1 → 0
Char. 205: 1 → 0

**Rhynchosauria:**
Char. 0: 1 → 0
Char. 7: 0 → 1
Char. 9: 0 → 1
Char. 63: 1 → 0
Char. 68: 0 → 1
Char. 99: 1 → 0
Char. 150: 1 → 0
Char. 160: 0 → 1
Char. 161: 0 → 1
Char. 171: 0 → 2
Char. 241: 0 → 1

***Scutosaurus* spp.:**
Char. 175: 0 → 1
Char. 190: 0 → 1
Char. 218: 0 → 1
Char. 243: 0 → 2
Char. 244: 0 → 1

***Sinosaurosphargis yunguiensis*:**
Char. 8: 0 → 1
Char. 30: 0 → 1
Char. 53: 0 → 1
Char. 89: 1 → 0
Char. 127: 1 → 0
Char. 150: 1 → 0
Char. 154: 0 → 2
Char. 167: 1 → 0
Char. 255: 0 → 1

**Squamata:**
Char. 45: 0 → 1
Char. 79: 0 → 2
Char. 80: 1 → 0
Char. 92: 1 → 0
Char. 160: 0 → 1
Char. 200: 0 → 1
Char. 245: 0 → 1

***Trilophosaurus buettneri*:**
Char. 5: 1 → 0

Char. 11: 0 → 1
Char. 55: 1 → 0
Char. 93: 1 → 0
Char. 104: 0 → 1
Char. 106: 0 → 1
Char. 113: 0 → 1
Char. 122: 0 → 1
Char. 136: 1 → 0
Char. 144: 1 → 0
Char. 157: 0 → 1
Char. 159: 1 → 0
Char. 177: 0 → 12
Char. 194: 0 → 1
Char. 203: 1 → 2
Char. 207: 1 → 0
Char. 208: 1 → 0
Char. 272: 1 → 0

***Youngina capensis*:**
Char. 5: 1 → 0
Char. 21: 0 → 1
Char. 23: 1 → 0
Char. 27: 0 → 1
Char. 38: 1 → 0
Char. 40: 2 → 0
Char. 43: 0 → 1
Char. 44: 1 → 0
Char. 56: 0 → 1
Char. 59: 0 → 1
Char. 60: 0 → 1
Char. 62: 0 → 1
Char. 72: 1 → 2
Char. 75: 0 → 1
Char. 84: 1 → 0
Char. 89: 0 → 1
Char. 92: 1 → 0
Char. 109: 0 → 1
Char. 127: 0 → 1
Char. 129: 0 → 1
Char. 134: 0 → 1
Char. 135: 0 → 1
Char. 139: 0 → 1
Char. 141: 0 → 1
Char. 154: 1 → 0
Char. 163: 1 → 0
Char. 170: 0 → 1
Char. 187: 0 → 1
Char. 191: 0 → 1
Char. 193: 0 → 1
Char. 211: 0 → 1
Char. 214: 0 → 1
Char. 215: 0 → 1
Char. 219: 0 → 1
Char. 222: 0 → 1
Char. 224: 0 → 1
Char. 231: 0 → 1
Char. 237: 0 → 1
Char. 239: 0 → 1
Char. 265: 0 → 1
Char. 267: 0 → 1
Char. 275: 1 → 0
Char. 278: 3 → 0

**Node 50:**
Char. 46: 1 → 0
Char. 50: 0 → 1
Char. 88: 1 → 0
Char. 93: 1 → 0
Char. 195: 0 → 2
Char. 198: 0 → 1
Char. 240: 1 → 0
Char. 246: 1 → 2
Char. 259: 0 → 1
Char. 270: 0 → 1

**Node 51:**
Char. 78: 1 → 0
Char. 194: 0 → 2
Char. 241: 0 → 1
Char. 246: 0 → 1
Char. 254: 0 → 1
Char. 255: 0 → 1
Char. 256: 0 → 1
Char. 268: 0 → 1
Char. 269: 0 → 1

**Node 52:**
Char. 21: 0 → 1
Char. 59: 0 → 1
Char. 60: 0 → 1
Char. 63: 0 → 1
Char. 69: 0 → 1
Char. 72: 1 → 2
Char. 73: 0 → 1
Char. 94: 0 → 1
Char. 126: 0 → 1
Char. 127: 0 → 1
Char. 131: 0 → 1
Char. 135: 0 → 1
Char. 136: 0 → 1
Char. 139: 0 → 1
Char. 141: 0 → 1
Char. 145: 0 → 1
Char. 147: 0 → 1
Char. 151: 0 → 1
Char. 187: 0 → 1
Char. 188: 0 → 1
Char. 191: 0 → 1
Char. 205: 0 → 1
Char. 208: 0 → 1
Char. 217: 0 → 1
Char. 222: 0 → 1
Char. 230: 0 → 1
Char. 231: 0 → 1
Char. 232: 0 → 1
Char. 237: 0 → 1
Char. 266: 0 → 1
Char. 267: 0 → 1
Char. 272: 2 → 1
Char. 278: 3 → 0

**Node 53:**
Char. 20: 0 → 1
Char. 23: 0 → 1
Char. 33: 0 → 1
Char. 67: 0 → 1
Char. 111: 0 → 1
Char. 112: 0 → 1
Char. 132: 0 → 1
Char. 166: 0 → 1
Char. 276: 1 → 0

**Node 54:**
Char. 57: 1 → 0
Char. 84: 0 → 1
Char. 88: 0 → 1
Char. 169: 0 → 1

**Node 55:**
Char. 72: 0 → 1
Char. 81: 0 → 1
Char. 93: 0 → 1
Char. 97: 0 → 1
Char. 104: 1 → 0
Char. 144: 0 → 1
Char. 173: 0 → 1
Char. 183: 0 → 1

**Node 57:**
Char. 95: 0 → 1
Char. 113: 0 → 1
Char. 114: 0 → 1

**Node 58:**
Char. 23: 1 → 0
Char. 29: 0 → 1
Char. 47: 0 → 1
Char. 50: 0 → 1
Char. 57: 0 → 1
Char. 67: 1 → 0
Char. 79: 0 → 1
Char. 110: 0 → 1
Char. 111: 1 → 0
Char. 112: 1 → 0
Char. 131: 0 → 1
Char. 137: 0 → 1
Char. 140: 0 → 2
Char. 147: 0 → 1

**Node 59:**
Char. 5: 1 → 0
Char. 20: 1 → 0
Char. 33: 1 → 0
Char. 59: 0 → 1
Char. 60: 0 → 1
Char. 72: 1 → 2
Char. 84: 1 → 0
Char. 116: 1 → 0
Char. 154: 1 → 0
Char. 159: 1 → 0
Char. 166: 1 → 0
Char. 169: 1 → 0
Char. 170: 0 → 1
Char. 196: 1 → 0
Char. 197: 0 → 1
Char. 202: 1 → 0
Char. 204: 2 → 0
Char. 207: 1 → 0
Char. 209: 1 → 0
Char. 221: 1 → 0

**Node 60:**
Char. 19: 0 → 1
Char. 21: 1 → 0
Char. 92: 1 → 0

**Node 61:**
Char. 4: 0 → 1
Char. 29: 0 → 1
Char. 213: 0 → 1
Char. 226: 01 → 2
Char. 228: 0 → 1

**Node 62:**
Char. 43: 0 → 1
Char. 58: 0 → 1
Char. 61: 0 → 1
Char. 62: 0 → 1
Char. 107: 0 → 1
Char. 140: 0 → 1
Char. 150: 0 → 1
Char. 167: 0 → 1
Char. 190: 0 → 1
Char. 214: 0 → 1
Char. 219: 0 → 1
Char. 239: 0 → 1
Char. 260: 2 → 1

**Node 63:**
Char. 73: 0 → 1
Char. 205: 0 → 1
Char. 273: 0 → 1
Char. 276: 0 → 1

**Node 64:**
Char. 18: 0 → 1
Char. 37: 0 → 1
Char. 103: 0 → 1
Char. 106: 0 → 1
Char. 107: 0 → 1
Char. 110: 0 → 1
Char. 126: 0 → 1
Char. 150: 0 → 1

**Node 65:**
Char. 29: 0 → 1
Char. 38: 1 → 2
Char. 39: 0 → 1
Char. 44: 1 → 0
Char. 49: 0 → 1
Char. 57: 0 → 1
Char. 66: 0 → 1
Char. 67: 1 → 0
Char. 76: 0 → 1
Char. 80: 1 → 0
Char. 95: 0 → 1
Char. 111: 1 → 0
Char. 120: 1 → 0
Char. 135: 0 → 1
Char. 137: 0 → 1
Char. 147: 0 → 1
Char. 157: 0 → 1
Char. 158: 0 → 1
Char. 183: 1 → 2
Char. 186: 0 → 1
Char. 187: 0 → 1
Char. 194: 0 → 1
Char. 197: 0 → 1
Char. 209: 1 → 0
Char. 211: 0 → 1
Char. 234: 1 → 0
Char. 240: 1 → 0
Char. 252: 0 → 1

**Node 66:**
Char. 87: 0 → 1
Char. 110: 0 → 1

**Node 67:**
Char. 79: 0 → 1
Char. 133: 0 → 1

**Node 68:**
Char. 100: 0 → 1
Char. 113: 0 → 1

**Node 69:**
Char. 23: 1 → 0
Char. 50: 0 → 1
Char. 70: 0 → 1
Char. 118: 0 → 1
Char. 125: 0 → 1

**Node 70:**
Char. 20: 1 → 0
Char. 38: 1 → 2
Char. 39: 0 → 1
Char. 57: 0 → 1
Char. 58: 0 → 1
Char. 59: 0 → 1
Char. 60: 0 → 1
Char. 72: 1 → 2
Char. 83: 0 → 1
Char. 85: 1 → 0
Char. 88: 1 → 0
Char. 95: 0 → 1
Char. 104: 0 → 1
Char. 105: 0 → 1
Char. 106: 0 → 2
Char. 107: 0 → 1
Char. 109: 0 → 1
Char. 110: 0 → 1
Char. 146: 1 → 0
Char. 155: 0 → 2
Char. 183: 1 → 2

**Node 71:**
Char. 23: 0 → 1
Char. 33: 1 → 0
Char. 38: 2 → 1
Char. 39: 1 → 0
Char. 42: 0 → 1
Char. 43: 0 → 1
Char. 46: 1 → 0

Char. 49: 1 → 0
Char. 71: 1 → 0
Char. 83: 0 → 2
Char. 101: 0 → 1
Char. 103: 0 → 1
Char. 106: 0 → 1
Char. 161: 0 → 1
Char. 163: 1 → 0
Char. 172: 0 → 2
Char. 188: 0 → 1
Char. 203: 1 → 2
Char. 212: 0 → 1
Char. 216: 1 → 0
Char. 221: 1 → 0
Char. 236: 0 → 1
Char. 238: 0 → 2
Char. 241: 0 → 1
Char. 242: 0 → 1
Char. 273: 0 → 1
Char. 274: 0 → 1
Char. 275: 1 → 0

**Node 72:**
Char. 52: 0 → 1
Char. 75: 0 → 1
Char. 84: 1 → 0
Char. 87: 0 → 1
Char. 110: 0 → 1
Char. 121: 0 → 1
Char. 180: 1 → 0
Char. 230: 0 → 1

**Node 73:**
Char. 277: 0 → 1

**Node 74:**
Char. 44: 1 → 0
Char. 66: 0 → 1
Char. 78: 1 → 0
Char. 88: 1 → 0
Char. 93: 1 → 0
Char. 112: 1 → 0
Char. 123: 1 → 0
Char. 129: 0 → 1
Char. 163: 1 → 0
Char. 192: 1 → 0
Char. 234: 1 → 0

**Node 75:**
Char. 33: 1 → 0
Char. 83: 0 → 1
Char. 85: 1 → 0
Char. 107: 0 → 1

**Node 76:**
Char. 2: 0 → 1
Char. 6: 0 → 1
Char. 186: 0 → 1
Char. 198: 0 → 1
Char. 200: 0 → 1
Char. 220: 0 → 1
Char. 239: 1 → 0
Char. 267: 1 → 0

**Node 77:**
Char. 98: 1 → 0
Char. 113: 0 → 1
Char. 181: 0 → 1
Char. 206: 0 → 2

**Node 78:**
Char. 35: 0 → 1
Char. 102: 0 → 1
Char. 103: 0 → 1
Char. 201: 0 → 1
Char. 229: 0 → 1

**Node 79:**
Char. 17: 0 → 1
Char. 42: 0 → 1
Char. 264: 0 → 1

**Node 80:**
Char. 41: 0 → 1
Char. 61: 1 → 2
Char. 112: 1 → 0
Char. 138: 0 → 1
Char. 146: 1 → 0
Char. 192: 1 → 2
Char. 227: 0 → 1
Char. 233: 0 → 1
Char. 267: 1 → 2

**Node 81:**
Char. 61: 1 → 3
Char. 90: 0 → 1
Char. 91: 0 → 1
Char. 209: 1 → 0

**ANALYSIS 79**
**(ALL TAXA, NO CHARACTERS 174, 204, 247, 251, AND 253)**

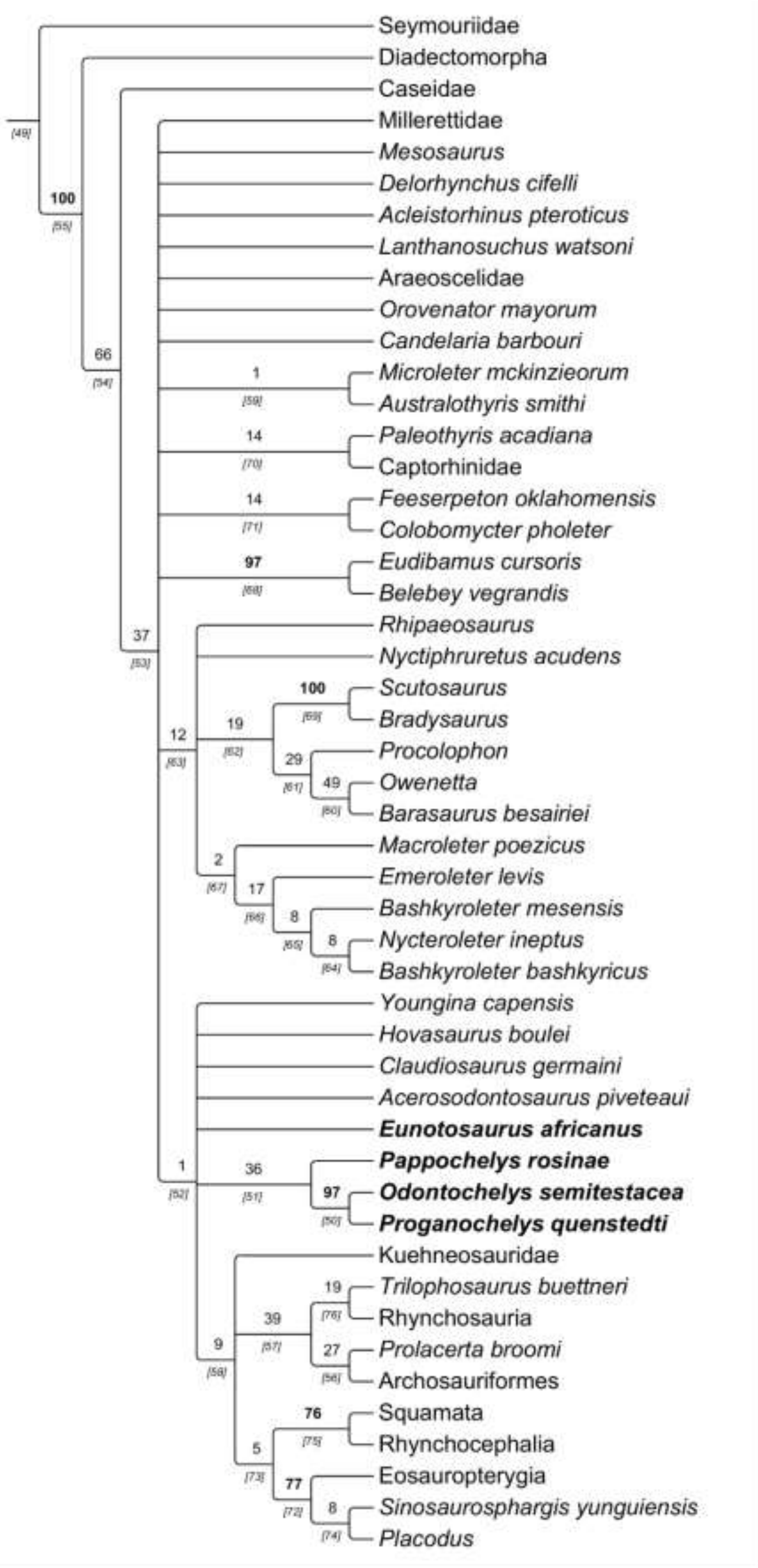

***Proganochelys quenstedti*:**
Char. 8: 0 → 1
Char. 11: 0 → 1
Char. 106: 0 → 1
Char. 108: 1 → 0
Char. 128: 0 → 1
Char. 175: 0 → 1
Char. 202: 1 → 0
Char. 207: 1 → 0
Char. 209: 1 → 0
Char. 244: 0 → 1
Char. 248: 0 → 1
Char. 250: 0 → 1
Char. 262: 0 → 1

***Pappochelys rosinae*:**
Char. 1: 0 → 1
Char. 5: 1 → 0
Char. 12: 0 → 1
Char. 41: 0 → 1
Char. 49: 0 → 1
Char. 55: 1 → 0
Char. 67: 1 → 0
Char. 75: 0 → 1
Char. 169: 1 → 0
Char. 260: 2 → 0

***Odontochelys semitestacea*:**
Char. 109: 1 → 0
Char. 112: 1 → 0
Char. 124: 0 → 1

***Eunotosaurus africanus*:**
Char. 0: 1 → 2
Char. 19: 0 → 1
Char. 24: 0 → 1
Char. 33: 1 → 2
Char. 42: 0 → 1
Char. 48: 1 → 0
Char. 50: 0 → 1
Char. 55: 1 → 0
Char. 57: 0 → 1
Char. 59: 1 → 0
Char. 60: 1 → 0
Char. 67: 1 → 0
Char. 72: 2 → 1
Char. 76: 0 → 1
Char. 81: 1 → 0
Char. 84: 1 → 2
Char. 97: 1 → 0
Char. 102: 0 → 1
Char. 103: 0 → 1
Char. 109: 1 → 0
Char. 112: 1 → 0
Char. 124: 0 → 1
Char. 129: 1 → 0
Char. 130: 0 → 1
Char. 147: 0 → 1
Char. 152: 0 → 1
Char. 153: 1 → 0
Char. 155: 0 → 1
Char. 158: 0 → 1
Char. 161: 0 → 1
Char. 181: 0 → 1
Char. 191: 1 → 0
Char. 192: 1 → 0
Char. 194: 0 → 1
Char. 202: 1 → 0
Char. 211: 0 → 1
Char. 222: 1 → 0
Char. 237: 1 → 0
Char. 240: 1 → 0
Char. 248: 0 → 1
Char. 249: 0 → 2
Char. 250: 0 → 1
Char. 252: 0 → 1
Char. 263: 0 → 1
Char. 267: 1 → 0
Char. 273: 0 → 1
Char. 274: 0 → 1
Char. 275: 0 → 1
Char. 276: 0 → 1
Char. 277: 0 → 1
Char. 278: 0 → 3

***Acerosodontosaurus piveteaui*:**
Char. 78: 1 → 0
Char. 81: 1 → 0
Char. 127: 1 → 0
Char. 128: 0 → 1
Char. 155: 0 → 1
Char. 208: 0 → 1
Char. 267: 1 → 0
Char. 278: 0 → 1

***Acleistorhinus pteroticus:***
Char. 21: 0 → 1
Char. 30: 0 → 1
Char. 33: 0 → 1
Char. 47: 0 → 1
Char. 48: 0 → 1
Char. 55: 1 → 0
Char. 56: 0 → 1
Char. 64: 0 → 1
Char. 70: 0 → 1
Char. 79: 0 → 1
Char. 95: 0 → 1
Char. 110: 0 → 1
Char. 113: 0 → 1
Char. 114: 0 → 1
Char. 131: 0 → 1
Char. 137: 0 → 1
Char. 140: 0 → 2
Char. 146: 1 → 0
Char. 147: 0 → 1
Char. 159: 0 → 1
Char. 169: 1 → 0
Char. 170: 0 → 1
Char. 278: 3 → 1

**Araeoscelidae:**
Char. 0: 0 → 1
Char. 5: 1 → 0
Char. 23: 0 → 1
Char. 27: 0 → 1
Char. 28: 0 → 1
Char. 29: 1 → 0
Char. 38: 1 → 0
Char. 40: 2 → 0
Char. 43: 0 → 1
Char. 48: 0 → 1
Char. 50: 1 → 0
Char. 57: 1 → 0
Char. 59: 0 → 1
Char. 60: 0 → 1
Char. 67: 0 → 1
Char. 72: 1 → 2
Char. 84: 1 → 0
Char. 89: 0 → 1
Char. 106: 0 → 1
Char. 111: 0 → 1
Char. 112: 0 → 1
Char. 116: 1 → 0
Char. 117: 0 → 2
Char. 120: 0 → 1
Char. 154: 1 → 0
Char. 166: 1 → 0
Char. 169: 1 → 0
Char. 170: 0 → 1
Char. 193: 0 → 1
Char. 197: 0 → 1
Char. 222: 0 → 1
Char. 224: 0 → 1
Char. 237: 0 → 1
Char. 239: 0 → 1
Char. 266: 0 → 1
Char. 278: 3 → 1

**Archosauriformes:**
Char. 32: 0 → 1
Char. 94: 1 → 0
Char. 112: 1 → 0
Char. 152: 0 → 1
Char. 154: 0 → 2
Char. 166: 1 → 0
Char. 171: 0 → 1
Char. 185: 0 → 1
Char. 218: 0 → 3
Char. 224: 0 → 1
Char. 242: 0 → 1

***Australothyris smithi*:**
Char. 23: 0 → 1
Char. 34: 0 → 1
Char. 55: 1 → 0
Char. 71: 1 → 0
Char. 85: 1 → 0
Char. 112: 0 → 1
Char. 129: 0 → 1
Char. 131: 0 → 1

***Barasaurus besairiei*:**
Char. 33: 1 → 0
Char. 75: 1 → 0
Char. 216: 0 → 1

***Bashkyroleter bashkyricus*:**
Char. 275: 1 → 0

***Bashkyroleter mesensis*:**
Char. 169: 1 → 0

***Belebey vegrandis*:**
Char. 154: 1 → 0

***Bradysaurus* spp.:**
Char. 19: 0 → 1
Char. 73: 0 → 1
Char. 79: 0 → 1
Char. 135: 1 → 0
Char. 249: 0 → 1

***Candelaria barbouri*:**
Char. 1: 0 → 1
Char. 5: 1 → 0
Char. 8: 0 → 1
Char. 15: 0 → 1
Char. 23: 0 → 1
Char. 25: 1 → 0
Char. 29: 1 → 0
Char. 33: 0 → 2
Char. 49: 0 → 1
Char. 50: 1 → 0
Char. 55: 1 → 0
Char. 57: 1 → 0
Char. 67: 0 → 1
Char. 76: 0 → 1
Char. 79: 0 → 2
Char. 83: 0 → 1
Char. 89: 0 → 1
Char. 94: 0 → 1
Char. 95: 0 → 1
Char. 126: 0 → 1
Char. 127: 0 → 1
Char. 132: 1 → 0
Char. 154: 1 → 2
Char. 169: 1 → 0
Char. 276: 0 → 1
Char. 277: 0 → 1

**Captorhinidae:**
Char. 3: 0 → 1
Char. 23: 0 → 1
Char. 25: 1 → 0
Char. 26: 0 → 1
Char. 73: 0 → 1
Char. 75: 0 → 1
Char. 83: 0 → 1
Char. 108: 1 → 0
Char. 180: 1 → 0
Char. 183: 1 → 0
Char. 201: 1 → 0
Char. 203: 1 → 2
Char. 216: 1 → 0
Char. 240: 1 → 0

**Caseidae:**
Char. 24: 0 → 1
Char. 25: 1 → 0
Char. 36: 0 → 1
Char. 38: 1 → 0
Char. 46: 1 → 0
Char. 50: 1 → 0
Char. 56: 0 → 1
Char. 170: 0 → 1
Char. 194: 0 → 1
Char. 273: 0 → 1
Char. 274: 0 → 1
Char. 278: 3 → 2

***Claudiosaurus germaini*:**
Char. 21: 1 → 0
Char. 24: 0 → 1
Char. 27: 0 → 1
Char. 34: 0 → 1
Char. 36: 0 → 1
Char. 43: 1 → 0
Char. 56: 0 → 1
Char. 70: 0 → 1
Char. 84: 1 → 0
Char. 105: 0 → 1
Char. 106: 0 → 1
Char. 117: 0 → 2
Char. 126: 0 → 1
Char. 127: 1 → 0
Char. 130: 0 → 1
Char. 131: 0 → 1
Char. 141: 1 → 0
Char. 144: 1 → 0
Char. 154: 1 → 0
Char. 166: 1 → 0
Char. 182: 0 → 1
Char. 187: 1 → 0
Char. 190: 0 → 1
Char. 199: 0 → 1
Char. 201: 0 → 1
Char. 214: 0 → 1
Char. 222: 1 → 0
Char. 231: 1 → 0
Char. 234: 1 → 0
Char. 272: 2 → 1

***Colobomycter pholeter*:**
Char. 21: 0 → 1
Char. 25: 1 → 0
Char. 84: 1 → 0
Char. 154: 1 → 0
Char. 167: 0 → 1
Char. 267: 0 → 1

***Delorhynchus cifelli*:**
Char. 18: 0 → 1
Char. 21: 0 → 1
Char. 24: 0 → 1
Char. 26: 0 → 1
Char. 28: 0 → 1
Char. 33: 0 → 2
Char. 39: 0 → 1
Char. 48: 0 → 1
Char. 52: 0 → 1
Char. 100: 0 → 1
Char. 111: 0 → 1

Char. 116: 1 → 0
Char. 117: 0 → 1
Char. 119: 1 → 0
Char. 131: 0 → 1
Char. 147: 0 → 1
Char. 156: 1 → 0
Char. 159: 0 → 1
Char. 167: 0 → 1
Char. 189: 0 → 1
Char. 191: 0 → 1
Char. 267: 0 → 1

**Diadectomorpha:**
Char. 0: 0 → 1
Char. 64: 0 → 1
Char. 70: 0 → 1
Char. 122: 0 → 1
Char. 123: 1 → 0
Char. 146: 1 → 0
Char. 275: 1 → 0
Char. 278: 3 → 0

***Emeroleter levis*:**
Char. 0: 0 → 2
Char. 51: 1 → 0

**Eosauropterygia:**
Char. 159: 1 → 0
Char. 166: 1 → 0
Char. 194: 0 → 2
Char. 272: 1 → 0

***Eudibamus cursoris*:**
Char. 154: 1 → 2
Char. 163: 1 → 0

***Feeserpeton oklahomensis*:**
Char. 51: 0 → 1
Char. 70: 0 → 1
Char. 157: 0 → 1
Char. 158: 0 → 1

***Hovasaurus boulei*:**
Char. 41: 0 → 1
Char. 44: 0 → 1
Char. 55: 1 → 0
Char. 60: 1 → 0
Char. 72: 2 → 1
Char. 73: 1 → 0
Char. 77: 0 → 2
Char. 78: 1 → 0
Char. 79: 0 → 1
Char. 93: 1 → 0
Char. 113: 0 → 1
Char. 138: 0 → 1
Char. 141: 1 → 0
Char. 146: 1 → 0
Char. 148: 1 → 0
Char. 215: 0 → 1
Char. 217: 1 → 0
Char. 219: 1 → 0
Char. 224: 0 → 1

Char. 231: 1 → 0
Char. 278: 0 → 3

**Kuehneosauridae:**
Char. 7: 0 → 1
Char. 21: 1 → 0
Char. 24: 0 → 1
Char. 26: 0 → 1
Char. 27: 0 → 1
Char. 34: 0 → 1
Char. 36: 0 → 1
Char. 43: 1 → 0
Char. 56: 0 → 1
Char. 79: 0 → 2
Char. 82: 0 → 1
Char. 98: 1 → 0
Char. 108: 1 → 0
Char. 113: 0 → 1
Char. 128: 0 → 1
Char. 148: 1 → 0
Char. 159: 1 → 0
Char. 181: 0 → 1
Char. 185: 0 → 1
Char. 245: 0 → 1
Char. 278: 0 → 3

***Lanthanosuchus watsoni*:**
Char. 25: 1 → 0
Char. 51: 0 → 1
Char. 76: 0 → 1
Char. 86: 0 → 1
Char. 95: 0 → 1
Char. 98: 1 → 0
Char. 110: 0 → 1
Char. 113: 0 → 1
Char. 114: 0 → 1
Char. 119: 1 → 0
Char. 131: 0 → 1
Char. 137: 0 → 1
Char. 138: 0 → 1
Char. 140: 0 → 2
Char. 144: 1 → 0
Char. 147: 0 → 1
Char. 154: 1 → 2

***Macroleter poezicus*:**
Char. 0: 0 → 1
Char. 52: 0 → 1
Char. 66: 1 → 2
Char. 75: 0 → 1
Char. 84: 1 → 0
Char. 87: 0 → 1
Char. 110: 0 → 1
Char. 121: 0 → 1
Char. 140: 0 → 1
Char. 146: 1 → 0
Char. 169: 1 → 0
Char. 235: 0 → 1

***Mesosaurus* spp.:**
Char. 0: 0 → 1
Char. 2: 0 → 1
Char. 5: 1 → 0
Char. 6: 0 → 1
Char. 8: 0 → 1
Char. 9: 0 → 1
Char. 13: 0 → 1
Char. 19: 0 → 1
Char. 23: 0 → 1
Char. 26: 0 → 1
Char. 29: 1 → 0
Char. 33: 0 → 1
Char. 38: 1 → 0
Char. 41: 0 → 1
Char. 48: 0 → 1
Char. 50: 1 → 0
Char. 67: 0 → 1
Char. 76: 0 → 1
Char. 83: 0 → 1
Char. 84: 1 → 0
Char. 85: 1 → 0
Char. 94: 0 → 1
Char. 107: 0 → 1
Char. 109: 0 → 1
Char. 111: 0 → 1
Char. 115: 0 → 1
Char. 146: 1 → 0
Char. 148: 0 → 1
Char. 149: 1 → 0
Char. 163: 1 → 0
Char. 164: 0 → 1
Char. 166: 1 → 0
Char. 167: 0 → 1
Char. 176: 0 → 1
Char. 183: 1 → 0
Char. 184: 0 → 1
Char. 192: 1 → 0
Char. 199: 0 → 1
Char. 202: 0 → 1
Char. 206: 0 → 1
Char. 207: 0 → 1
Char. 209: 0 → 1
Char. 217: 0 → 1
Char. 219: 0 → 1
Char. 220: 0 → 1
Char. 231: 0 → 1
Char. 240: 1 → 0
Char. 260: 2 → 0
Char. 272: 2 → 0
Char. 278: 3 → 0

***Microleter mckinzieorum*:**
Char. 0: 0 → 1
Char. 25: 1 → 0
Char. 36: 0 → 1
Char. 39: 0 → 1
Char. 51: 0 → 2
Char. 56: 0 → 1
Char. 70: 0 → 1
Char. 94: 0 → 1
Char. 106: 0 → 1
Char. 159: 0 → 1
Char. 166: 1 → 0
Char. 276: 0 → 1

**Millerettidae:**
Char. 5: 1 → 0
Char. 24: 0 → 1
Char. 25: 1 → 0
Char. 44: 1 → 0
Char. 56: 0 → 1
Char. 57: 1 → 0
Char. 66: 0 → 2
Char. 78: 1 → 0
Char. 80: 1 → 0
Char. 84: 1 → 2
Char. 96: 1 → 0
Char. 117: 0 → 1
Char. 121: 0 → 1
Char. 124: 0 → 1
Char. 127: 0 → 1
Char. 135: 0 → 1
Char. 145: 0 → 1
Char. 163: 1 → 0
Char. 166: 1 → 0
Char. 180: 1 → 0
Char. 192: 1 → 0
Char. 202: 0 → 1
Char. 211: 0 → 1
Char. 230: 0 → 1
Char. 248: 0 → 1
Char. 252: 0 → 1

***Nycteroleter ineptus*:**
Char. 278: 0 → 3

***Nyctiphruretus acudens*:**
Char. 0: 0 → 1
Char. 21: 0 → 1
Char. 33: 1 → 2
Char. 41: 0 → 1
Char. 66: 1 → 2
Char. 81: 1 → 0
Char. 83: 0 → 1
Char. 84: 1 → 2
Char. 94: 0 → 1
Char. 166: 1 → 0
Char. 167: 0 → 1
Char. 215: 1 → 0
Char. 224: 0 → 1
Char. 226: 1 → 0
Char. 266: 0 → 1
Char. 272: 2 → 1
Char. 276: 0 → 1

***Orovenator mayorum*:**
Char. 0: 0 → 1
Char. 8: 0 → 1
Char. 24: 0 → 1
Char. 25: 1 → 0
Char. 29: 1 → 0
Char. 36: 0 → 1
Char. 62: 0 → 1
Char. 72: 1 → 2
Char. 89: 0 → 1
Char. 111: 0 → 1
Char. 112: 0 → 1
Char. 120: 0 → 1

Char. 136: 0 → 1
Char. 141: 0 → 1
Char. 160: 0 → 1
Char. 165: 0 → 1
Char. 278: 3 → 1

***Owenetta* spp.:**
Char. 169: 1 → 0

***Paleothyris acadiana*:**
Char. 38: 1 → 0
Char. 50: 1 → 0
Char. 66: 1 → 2
Char. 102: 0 → 1
Char. 146: 1 → 0
Char. 237: 0 → 1
Char. 239: 0 → 1

***Placodus* spp.:**
Char. 0: 1 → 2
Char. 9: 0 → 1
Char. 12: 0 → 1
Char. 13: 0 → 1
Char. 19: 0 → 1
Char. 26: 0 → 1
Char. 31: 0 → 1
Char. 46: 1 → 0
Char. 57: 0 → 1
Char. 78: 1 → 0
Char. 93: 1 → 0
Char. 102: 1 → 2
Char. 109: 1 → 0
Char. 140: 1 → 0
Char. 155: 0 → 1
Char. 163: 1 → 0
Char. 164: 0 → 1

***Procolophon* spp.:**
Char. 41: 0 → 1
Char. 69: 0 → 1
Char. 79: 0 → 1
Char. 83: 0 → 1
Char. 88: 0 → 1
Char. 117: 1 → 0
Char. 149: 1 → 0
Char. 237: 0 → 1
Char. 238: 0 → 1
Char. 272: 2 → 1
Char. 278: 3 → 0

***Prolacerta broomi*:**
Char. 58: 1 → 0
Char. 66: 1 → 0
Char. 67: 1 → 0
Char. 80: 1 → 0
Char. 139: 1 → 0
Char. 192: 1 → 0
Char. 203: 1 → 2

***Rhipaeosaurus* spp.:**
Char. 172: 0 → 1
Char. 186: 1 → 0
Char. 277: 0 → 1

**Rhynchocephalia:**
Char. 0: 1 → 2
Char. 24: 0 → 1
Char. 77: 0 → 1
Char. 94: 1 → 0
Char. 139: 1 → 0
Char. 167: 1 → 0
Char. 205: 1 → 0

**Rhynchosauria:**
Char. 0: 1 → 0
Char. 7: 0 → 1
Char. 9: 0 → 1
Char. 26: 0 → 1
Char. 68: 0 → 1
Char. 99: 1 → 0
Char. 150: 1 → 0
Char. 160: 0 → 1
Char. 161: 0 → 1
Char. 171: 0 → 2
Char. 223: 0 → 1
Char. 224: 0 → 1
Char. 241: 0 → 1

***Scutosaurus* spp.:**
Char. 175: 0 → 1
Char. 218: 0 → 1
Char. 243: 0 → 2
Char. 244: 0 → 1

***Sinosaurosphargis yunguiensis*:**
Char. 8: 0 → 1
Char. 30: 0 → 1
Char. 53: 0 → 1
Char. 82: 0 → 1
Char. 89: 1 → 0
Char. 127: 1 → 0
Char. 150: 1 → 0
Char. 154: 0 → 2
Char. 167: 1 → 0
Char. 255: 0 → 1

**Squamata:**
Char. 26: 0 → 1
Char. 45: 0 → 1
Char. 79: 0 → 2
Char. 80: 1 → 0
Char. 82: 0 → 1
Char. 92: 1 → 0
Char. 109: 1 → 0
Char. 160: 0 → 1
Char. 245: 0 → 1

***Trilophosaurus buettneri*:**
Char. 5: 1 → 0
Char. 11: 0 → 1
Char. 44: 0 → 1
Char. 55: 1 → 0
Char. 93: 1 → 0
Char. 104: 0 → 1
Char. 106: 0 → 1
Char. 113: 0 → 1
Char. 122: 0 → 1
Char. 136: 1 → 0
Char. 144: 1 → 0
Char. 154: 0 → 1
Char. 157: 0 → 1
Char. 159: 1 → 0
Char. 177: 0 → 12
Char. 194: 0 → 1
Char. 203: 1 → 2
Char. 207: 1 → 0
Char. 208: 1 → 0
Char. 272: 1 → 0

***Youngina capensis*:**
Char. 5: 1 → 0
Char. 25: 0 → 1
Char. 27: 0 → 1
Char. 38: 1 → 0
Char. 56: 0 → 1
Char. 73: 1 → 0
Char. 75: 0 → 1
Char. 84: 1 → 0
Char. 92: 1 → 0
Char. 94: 1 → 0
Char. 145: 1 → 0
Char. 148: 1 → 0
Char. 151: 1 → 0
Char. 154: 1 → 0
Char. 163: 1 → 0
Char. 170: 0 → 1
Char. 211: 0 → 1
Char. 214: 0 → 1
Char. 215: 0 → 1
Char. 220: 1 → 0
Char. 224: 0 → 1
Char. 239: 0 → 1

**Node 50:**
Char. 46: 1 → 0
Char. 50: 0 → 1
Char. 88: 1 → 0
Char. 89: 1 → 0
Char. 93: 1 → 0
Char. 176: 0 → 1
Char. 195: 0 → 2
Char. 198: 0 → 1
Char. 240: 1 → 0
Char. 246: 1 → 2
Char. 259: 0 → 1
Char. 265: 1 → 0
Char. 270: 0 → 1

**Node 51:**
Char. 65: 0 → 1
Char. 78: 1 → 0
Char. 131: 0 → 1
Char. 184: 0 → 1
Char. 193: 1 → 0
Char. 194: 0 → 2
Char. 205: 0 → 1
Char. 210: 0 → 1
Char. 219: 1 → 0
Char. 241: 0 → 1
Char. 246: 0 → 1
Char. 254: 0 → 1
Char. 255: 0 → 1
Char. 256: 0 → 1
Char. 268: 0 → 1
Char. 269: 0 → 1

**Node 52:**
Char. 0: 0 → 1
Char. 21: 0 → 1
Char. 25: 1 → 0
Char. 29: 1 → 0
Char. 33: 0 → 1
Char. 40: 2 → 0
Char. 43: 0 → 1
Char. 44: 1 → 0
Char. 48: 0 → 1
Char. 50: 1 → 0
Char. 57: 1 → 0
Char. 59: 0 → 1
Char. 60: 0 → 1
Char. 67: 0 → 1
Char. 72: 1 → 2
Char. 73: 0 → 1
Char. 89: 0 → 1
Char. 92: 0 → 1
Char. 94: 0 → 1
Char. 109: 0 → 1
Char. 111: 0 → 1
Char. 112: 0 → 1
Char. 120: 0 → 1
Char. 127: 0 → 1
Char. 129: 0 → 1
Char. 135: 0 → 1
Char. 136: 0 → 1
Char. 139: 0 → 1
Char. 141: 0 → 1
Char. 145: 0 → 1
Char. 148: 0 → 1
Char. 151: 0 → 1
Char. 159: 0 → 1
Char. 179: 0 → 1
Char. 187: 0 → 1
Char. 188: 0 → 1
Char. 191: 0 → 1
Char. 193: 0 → 1
Char. 196: 0 → 1
Char. 201: 1 → 0
Char. 202: 0 → 1
Char. 207: 0 → 1
Char. 209: 0 → 1
Char. 217: 0 → 1
Char. 219: 0 → 1
Char. 220: 0 → 1
Char. 222: 0 → 1
Char. 231: 0 → 1
Char. 237: 0 → 1
Char. 265: 0 → 1
Char. 266: 0 → 1
Char. 267: 0 → 1
Char. 275: 1 → 0
Char. 278: 3 → 0

**Node 53:**
Char. 5: 0 → 1
Char. 29: 0 → 1
Char. 40: 0 → 2
Char. 74: 0 → 1
Char. 80: 0 → 1
Char. 84: 0 → 1
Char. 116: 0 → 1
Char. 119: 0 → 1
Char. 132: 0 → 1
Char. 149: 0 → 1
Char. 156: 0 → 1
Char. 163: 0 → 1
Char. 166: 0 → 1
Char. 169: 0 → 1
Char. 180: 0 → 1
Char. 192: 0 → 1
Char. 201: 0 → 1
Char. 203: 0 → 1
Char. 235: 0 → 1

**Node 54:**
Char. 72: 0 → 1
Char. 79: 1 → 0
Char. 81: 0 → 1
Char. 93: 0 → 1
Char. 97: 0 → 1
Char. 104: 1 → 0
Char. 144: 0 → 1
Char. 173: 0 → 1
Char. 183: 0 → 1

**Node 56:**
Char. 19: 0 → 1
Char. 21: 1 → 0
Char. 92: 1 → 0

**Node 57:**
Char. 4: 0 → 1
Char. 29: 0 → 1
Char. 48: 1 → 0
Char. 213: 0 → 1
Char. 226: 0 → 2
Char. 228: 0 → 1
Char. 275: 0 → 1

**Node 58:**
Char. 58: 0 → 1
Char. 61: 0 → 1
Char. 66: 0 → 1
Char. 69: 0 → 1
Char. 126: 0 → 1
Char. 131: 0 → 1
Char. 150: 0 → 1
Char. 154: 1 → 0
Char. 167: 0 → 1
Char. 176: 0 → 1
Char. 190: 0 → 1
Char. 205: 0 → 1
Char. 208: 0 → 1
Char. 214: 0 → 1
Char. 220: 1 → 0
Char. 230: 0 → 1
Char. 239: 0 → 1
Char. 260: 2 → 1

**Node 59:**
Char. 24: 0 → 1
Char. 57: 1 → 0
Char. 79: 0 → 1
Char. 83: 0 → 1
Char. 110: 0 → 1
Char. 132: 1 → 0

**Node 60:**
Char. 73: 0 → 1
Char. 131: 1 → 0
Char. 205: 0 → 1
Char. 239: 0 → 1
Char. 276: 0 → 1

**Node 61:**
Char. 18: 0 → 1
Char. 37: 0 → 1
Char. 48: 0 → 1
Char. 107: 0 → 1
Char. 118: 1 → 0
Char. 125: 1 → 0
Char. 126: 0 → 1
Char. 132: 1 → 0
Char. 150: 0 → 1

**Node 62:**
Char. 23: 0 → 1
Char. 71: 1 → 0
Char. 75: 0 → 1
Char. 102: 0 → 1
Char. 103: 0 → 1
Char. 106: 0 → 1
Char. 110: 0 → 1
Char. 155: 0 → 1
Char. 167: 0 → 1
Char. 207: 0 → 1
Char. 214: 0 → 1
Char. 216: 1 → 0
Char. 235: 0 → 2
Char. 241: 0 → 1

**Node 63:**
Char. 33: 0 → 1
Char. 38: 1 → 2
Char. 39: 0 → 1
Char. 44: 1 → 0
Char. 49: 0 → 1
Char. 51: 0 → 1
Char. 66: 0 → 1
Char. 70: 0 → 1
Char. 76: 0 → 1
Char. 80: 1 → 0
Char. 95: 0 → 1
Char. 112: 0 → 1
Char. 117: 0 → 1
Char. 118: 0 → 1
Char. 125: 0 → 1
Char. 131: 0 → 1
Char. 135: 0 → 1

Char. 137: 0 → 1
Char. 147: 0 → 1
Char. 148: 0 → 1
Char. 157: 0 → 1
Char. 158: 0 → 1
Char. 159: 0 → 1
Char. 183: 1 → 2
Char. 186: 0 → 1
Char. 194: 0 → 1
Char. 197: 0 → 1
Char. 201: 1 → 0
Char. 211: 0 → 1
Char. 215: 0 → 1
Char. 226: 0 → 1
Char. 235: 1 → 0
Char. 240: 1 → 0
Char. 252: 0 → 1

**Node 64:**
Char. 87: 0 → 1
Char. 110: 0 → 1

**Node 65:**
Char. 25: 1 → 0
Char. 76: 1 → 0

**Node 66:**
Char. 79: 0 → 1
Char. 86: 0 → 1
Char. 93: 1 → 0
Char. 133: 0 → 1

**Node 67:**
Char. 48: 0 → 1
Char. 100: 0 → 1
Char. 113: 0 → 1
Char. 132: 1 → 0
Char. 147: 1 → 2
Char. 154: 1 → 0
Char. 207: 0 → 1
Char. 239: 0 → 1
Char. 278: 3 → 0

**Node 68:**
Char. 38: 1 → 2
Char. 39: 0 → 1
Char. 50: 1 → 0
Char. 58: 0 → 1
Char. 59: 0 → 1
Char. 60: 0 → 1
Char. 72: 1 → 2
Char. 83: 0 → 1
Char. 85: 1 → 0
Char. 95: 0 → 1
Char. 104: 0 → 1
Char. 105: 0 → 1
Char. 106: 0 → 2
Char. 107: 0 → 1
Char. 109: 0 → 1
Char. 110: 0 → 1
Char. 146: 1 → 0
Char. 148: 0 → 1
Char. 155: 0 → 2
Char. 183: 1 → 2

**Node 69:**
Char. 0: 0 → 2
Char. 33: 1 → 0
Char. 38: 2 → 1
Char. 39: 1 → 0
Char. 42: 0 → 1
Char. 43: 0 → 1
Char. 46: 1 → 0
Char. 49: 1 → 0
Char. 52: 0 → 1
Char. 83: 0 → 2
Char. 84: 1 → 0
Char. 87: 0 → 1
Char. 93: 1 → 0
Char. 100: 0 → 1
Char. 113: 0 → 1
Char. 143: 0 → 1
Char. 161: 0 → 1
Char. 163: 1 → 0
Char. 172: 0 → 2
Char. 188: 0 → 1
Char. 189: 0 → 1
Char. 195: 0 → 1
Char. 212: 0 → 1
Char. 236: 0 → 1
Char. 238: 0 → 2
Char. 242: 0 → 1
Char. 245: 0 → 1
Char. 274: 0 → 1
Char. 275: 1 → 0

**Node 70:**
Char. 5: 1 → 0
Char. 29: 1 → 0
Char. 44: 1 → 0
Char. 59: 0 → 1
Char. 60: 0 → 1
Char. 66: 0 → 1
Char. 67: 0 → 1
Char. 72: 1 → 2
Char. 78: 1 → 0
Char. 84: 1 → 0
Char. 93: 1 → 0
Char. 111: 0 → 1
Char. 123: 1 → 0
Char. 129: 0 → 1
Char. 154: 1 → 0
Char. 163: 1 → 0
Char. 169: 1 → 0
Char. 170: 0 → 1
Char. 192: 1 → 0
Char. 197: 0 → 1

**Node 71:**
Char. 47: 0 → 1
Char. 83: 0 → 1
Char. 85: 1 → 0
Char. 107: 0 → 1
Char. 110: 0 → 1
Char. 169: 1 → 0
Char. 170: 0 → 1

**Node 72:**
Char. 2: 0 → 1
Char. 6: 0 → 1
Char. 98: 1 → 0
Char. 101: 0 → 1
Char. 113: 0 → 1
Char. 181: 0 → 1
Char. 186: 0 → 1
Char. 193: 1 → 0
Char. 198: 0 → 1
Char. 220: 0 → 1
Char. 231: 1 → 0
Char. 234: 1 → 0
Char. 239: 1 → 0
Char. 240: 1 → 0
Char. 266: 1 → 0

**Node 73:**
Char. 33: 1 → 2
Char. 68: 0 → 1
Char. 102: 0 → 1
Char. 103: 0 → 1
Char. 223: 0 → 1
Char. 229: 0 → 1
Char. 235: 1 → 2

**Node 74:**
Char. 17: 0 → 1
Char. 42: 0 → 1
Char. 264: 0 → 1

**Node 75:**
Char. 41: 0 → 1
Char. 48: 1 → 0
Char. 61: 1 → 2
Char. 112: 1 → 0
Char. 128: 0 → 1
Char. 138: 0 → 1
Char. 146: 1 → 0
Char. 155: 0 → 1
Char. 192: 1 → 2
Char. 224: 0 → 1
Char. 226: 0 → 1
Char. 227: 0 → 1
Char. 233: 0 → 1

**Node 76:**
Char. 61: 1 → 3
Char. 90: 0 → 1
Char. 91: 0 → 1
Char. 109: 1 → 0
Char. 155: 0 → 1
Char. 209: 1 → 0

**ANALYSIS 80**
**(ALL TAXA, IMPLIED WEIGHTING, K = 2.75, NO CHARACTERS 174, 204, 247, 251, AND 253)**

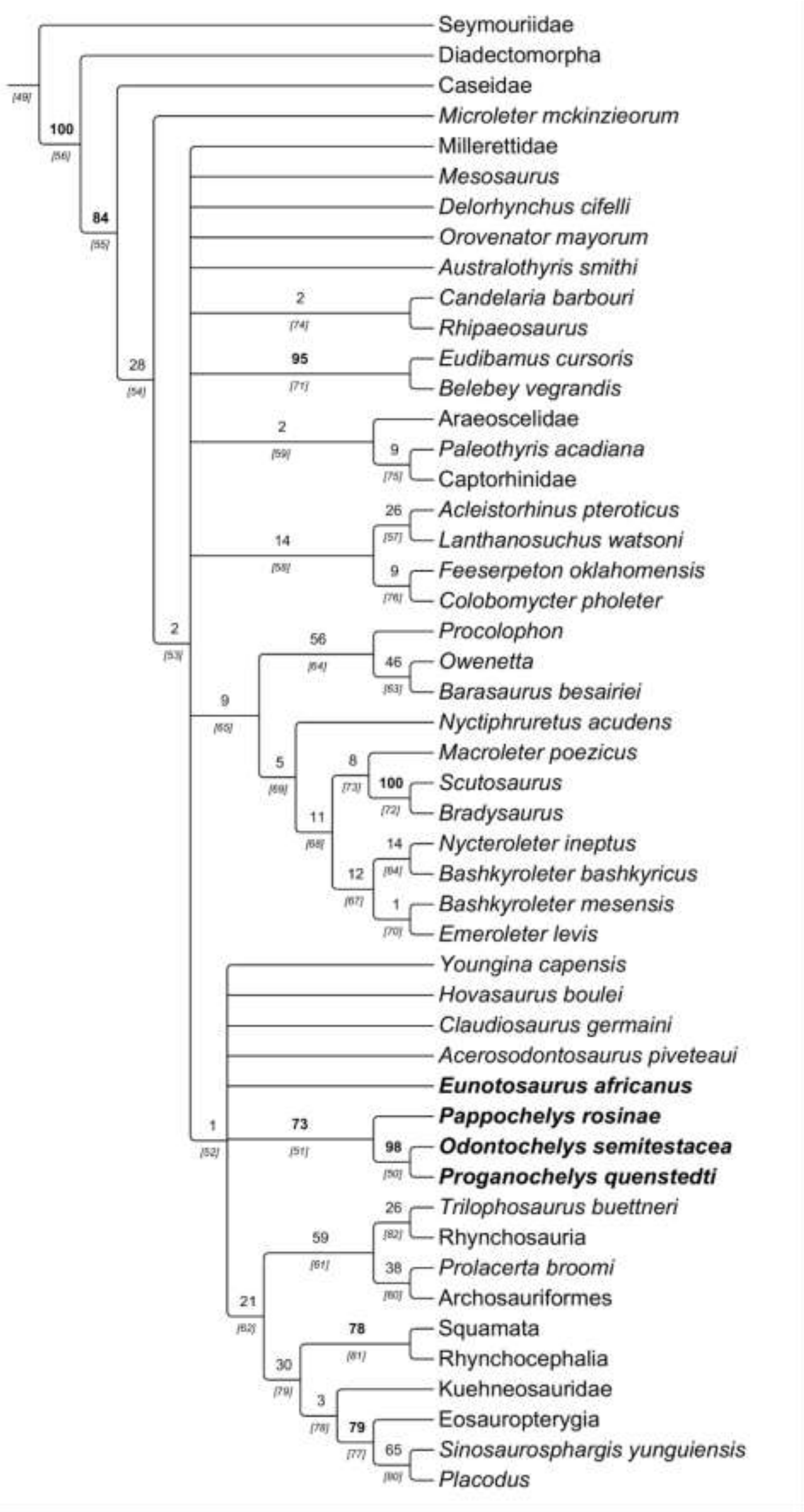

***Proganochelys quenstedti*:**
Char. 8: 0 → 1
Char. 11: 0 → 1
Char. 106: 0 → 1
Char. 108: 1 → 0
Char. 128: 0 → 1
Char. 175: 0 → 1
Char. 202: 1 → 0
Char. 207: 1 → 0
Char. 209: 1 → 0
Char. 244: 0 → 1
Char. 248: 0 → 1
Char. 250: 0 → 1
Char. 262: 0 → 1

***Pappochelys rosinae*:**
Char. 1: 0 → 1
Char. 5: 1 → 0
Char. 12: 0 → 1
Char. 41: 0 → 1
Char. 49: 0 → 1
Char. 55: 1 → 0
Char. 67: 1 → 0
Char. 75: 0 → 1
Char. 169: 1 → 0
Char. 260: 2 → 0

***Odontochelys semitestacea*:**
Char. 109: 1 → 0
Char. 112: 1 → 0
Char. 124: 0 → 1

***Eunotosaurus africanus*:**
Char. 0: 1 → 2
Char. 15: 0 → 1
Char. 19: 0 → 1
Char. 23: 1 → 0
Char. 24: 0 → 1
Char. 33: 1 → 2
Char. 42: 0 → 1
Char. 50: 0 → 1
Char. 55: 1 → 0
Char. 57: 0 → 1
Char. 59: 1 → 0
Char. 60: 1 → 0
Char. 62: 1 → 0
Char. 67: 1 → 0
Char. 72: 2 → 1
Char. 76: 0 → 1
Char. 81: 1 → 0
Char. 84: 1 → 2
Char. 97: 1 → 0
Char. 102: 0 → 1
Char. 103: 0 → 1
Char. 109: 1 → 0
Char. 112: 1 → 0
Char. 124: 0 → 1
Char. 129: 1 → 0
Char. 130: 0 → 1
Char. 134: 0 → 2
Char. 147: 0 → 1
Char. 152: 0 → 1
Char. 153: 1 → 0
Char. 155: 0 → 1
Char. 158: 0 → 1
Char. 161: 0 → 1
Char. 181: 0 → 1
Char. 191: 1 → 0
Char. 192: 1 → 0
Char. 194: 0 → 1
Char. 202: 1 → 0
Char. 211: 0 → 1
Char. 222: 1 → 0
Char. 237: 1 → 0
Char. 240: 1 → 0
Char. 248: 0 → 1
Char. 249: 0 → 2
Char. 250: 0 → 1
Char. 252: 0 → 1
Char. 263: 0 → 1
Char. 273: 0 → 1
Char. 274: 0 → 1
Char. 275: 0 → 1
Char. 276: 0 → 1
Char. 277: 0 → 1
Char. 278: 0 → 3

***Acerosodontosaurus piveteaui*:**
Char. 78: 1 → 0
Char. 81: 1 → 0
Char. 127: 1 → 0
Char. 128: 0 → 1
Char. 155: 0 → 1
Char. 206: 0 → 2
Char. 208: 0 → 1
Char. 278: 0 → 1

***Acleistorhinus pteroticus:***
Char. 21: 0 → 1
Char. 146: 1 → 0

**Araeoscelidae:**
Char. 0: 0 → 1
Char. 28: 0 → 1
Char. 43: 0 → 1
Char. 89: 0 → 1
Char. 106: 0 → 1
Char. 120: 0 → 1
Char. 193: 0 → 1
Char. 222: 0 → 1
Char. 224: 0 → 1
Char. 234: 0 → 1
Char. 266: 0 → 1
Char. 278: 3 → 1

**Archosauriformes:**
Char. 32: 0 → 1
Char. 94: 1 → 0
Char. 112: 1 → 0
Char. 152: 0 → 1
Char. 154: 01 → 2
Char. 166: 1 → 0
Char. 171: 0 → 1
Char. 185: 0 → 1
Char. 201: 0 → 1
Char. 218: 0 → 3
Char. 224: 0 → 1
Char. 242: 0 → 1

***Australothyris smithi*:**
Char. 24: 0 → 1
Char. 34: 0 → 1
Char. 55: 1 → 0
Char. 71: 1 → 0
Char. 79: 0 → 1
Char. 85: 1 → 0
Char. 98: 1 → 0
Char. 100: 0 → 1
Char. 103: 0 → 1
Char. 110: 0 → 1
Char. 123: 1 → 0
Char. 129: 0 → 1
Char. 131: 0 → 1
Char. 132: 1 → 0
Char. 144: 1 → 0
Char. 147: 0 → 1
Char. 148: 1 → 0
Char. 149: 1 → 0
Char. 150: 0 → 1
Char. 159: 1 → 0
Char. 192: 1 → 0

***Barasaurus besairiei*:**
Char. 33: 1 → 0

***Bashkyroleter bashkyricus*:**
Char. 275: 1 → 0

***Bashkyroleter mesensis*:**
Char. 12: 0 → 1
Char. 66: 1 → 0
Char. 169: 1 → 0

***Belebey vegrandis*:**
Char. 154: 1 → 0

***Bradysaurus* spp.:**
Char. 19: 0 → 1
Char. 25: 1 → 0
Char. 73: 0 → 1
Char. 79: 0 → 1
Char. 135: 1 → 0
Char. 249: 0 → 1

***Candelaria barbouri*:**
Char. 89: 0 → 1
Char. 159: 1 → 0

**Captorhinidae:**
Char. 3: 0 → 1
Char. 25: 1 → 0
Char. 26: 0 → 1
Char. 73: 0 → 1
Char. 75: 0 → 1
Char. 108: 1 → 0
Char. 180: 1 → 0
Char. 183: 1 → 0
Char. 201: 1 → 0
Char. 203: 1 → 2
Char. 216: 1 → 0

**Caseidae:**
Char. 38: 1 → 0
Char. 46: 1 → 0
Char. 170: 0 → 1
Char. 194: 0 → 1
Char. 273: 0 → 1
Char. 274: 0 → 1
Char. 278: 3 → 2

***Claudiosaurus germaini*:**
Char. 21: 1 → 0
Char. 24: 0 → 1
Char. 27: 0 → 1
Char. 34: 0 → 1
Char. 36: 0 → 1
Char. 56: 0 → 1
Char. 70: 0 → 1
Char. 84: 1 → 0
Char. 105: 0 → 1
Char. 106: 0 → 1
Char. 117: 01 → 2
Char. 126: 0 → 1
Char. 127: 1 → 0
Char. 130: 0 → 1
Char. 131: 0 → 1
Char. 141: 1 → 0
Char. 144: 1 → 0
Char. 154: 1 → 0
Char. 166: 1 → 0
Char. 182: 0 → 1
Char. 187: 1 → 0
Char. 190: 0 → 1
Char. 199: 0 → 1
Char. 201: 0 → 1
Char. 214: 0 → 1
Char. 222: 1 → 0
Char. 231: 1 → 0
Char. 234: 1 → 0
Char. 272: 2 → 1

***Colobomycter pholeter*:**
Char. 21: 0 → 1
Char. 25: 1 → 0
Char. 84: 1 → 0
Char. 154: 1 → 0
Char. 167: 0 → 1
Char. 267: 0 → 1

***Delorhynchus cifelli*:**
Char. 18: 0 → 1
Char. 21: 0 → 1
Char. 23: 1 → 0
Char. 24: 0 → 1
Char. 26: 0 → 1
Char. 28: 0 → 1
Char. 33: 1 → 2
Char. 39: 0 → 1
Char. 52: 0 → 1
Char. 100: 0 → 1
Char. 111: 0 → 1
Char. 116: 1 → 0
Char. 119: 1 → 0
Char. 131: 0 → 1
Char. 147: 0 → 1
Char. 148: 1 → 0
Char. 156: 1 → 0
Char. 167: 0 → 1
Char. 189: 0 → 1
Char. 191: 0 → 1
Char. 267: 0 → 1

**Diadectomorpha:**
Char. 0: 0 → 1
Char. 64: 0 → 1
Char. 70: 0 → 1
Char. 122: 0 → 1
Char. 123: 1 → 0
Char. 146: 1 → 0
Char. 275: 1 → 0
Char. 278: 3 → 0

***Emeroleter levis*:**
Char. 51: 1 → 0

**Eosauropterygia:**
Char. 166: 1 → 0
Char. 178: 0 → 1
Char. 194: 0 → 2
Char. 272: 1 → 0

***Eudibamus cursoris*:**
Char. 154: 1 → 2

***Feeserpeton oklahomensis*:**
Char. 51: 0 → 1
Char. 157: 0 → 1
Char. 158: 0 → 1

***Hovasaurus boulei*:**
Char. 41: 0 → 1
Char. 55: 1 → 0
Char. 60: 1 → 0
Char. 72: 2 → 1
Char. 73: 1 → 0
Char. 77: 0 → 2
Char. 78: 1 → 0
Char. 79: 0 → 1
Char. 93: 1 → 0
Char. 113: 0 → 1
Char. 138: 0 → 1
Char. 141: 1 → 0
Char. 146: 1 → 0
Char. 148: 1 → 0
Char. 206: 0 → 2
Char. 215: 0 → 1
Char. 217: 1 → 0
Char. 219: 1 → 0
Char. 224: 0 → 1
Char. 231: 1 → 0
Char. 278: 0 → 3

**Kuehneosauridae:**
Char. 7: 0 → 1
Char. 21: 1 → 0
Char. 24: 0 → 1
Char. 27: 0 → 1
Char. 43: 1 → 0
Char. 79: 0 → 2
Char. 107: 1 → 0
Char. 140: 1 → 0
Char. 147: 1 → 0
Char. 148: 1 → 0
Char. 245: 0 → 1
Char. 278: 0 → 3

***Lanthanosuchus watsoni*:**
Char. 25: 1 → 0
Char. 51: 0 → 1
Char. 86: 0 → 1
Char. 98: 1 → 0
Char. 138: 0 → 1
Char. 144: 1 → 0
Char. 154: 1 → 2

***Macroleter poezicus*:**
Char. 9: 0 → 1
Char. 26: 0 → 1
Char. 66: 1 → 2
Char. 126: 0 → 1
Char. 134: 0 → 2
Char. 140: 0 → 1
Char. 146: 1 → 0
Char. 169: 1 → 0

***Mesosaurus* spp.:**
Char. 0: 0 → 1
Char. 2: 0 → 1
Char. 6: 0 → 1
Char. 8: 0 → 1
Char. 9: 0 → 1
Char. 13: 0 → 1
Char. 19: 0 → 1
Char. 26: 0 → 1
Char. 29: 1 → 0
Char. 38: 1 → 0
Char. 41: 0 → 1
Char. 67: 0 → 1
Char. 84: 1 → 0
Char. 85: 1 → 0
Char. 94: 0 → 1
Char. 107: 0 → 1
Char. 109: 0 → 1
Char. 111: 0 → 1
Char. 115: 0 → 1
Char. 146: 1 → 0
Char. 149: 1 → 0
Char. 159: 1 → 0
Char. 164: 0 → 1
Char. 166: 1 → 0
Char. 167: 0 → 1
Char. 176: 0 → 1
Char. 183: 1 → 0
Char. 184: 0 → 1
Char. 192: 1 → 0
Char. 199: 0 → 1
Char. 202: 0 → 1
Char. 206: 0 → 1
Char. 209: 0 → 1
Char. 217: 0 → 1
Char. 219: 0 → 1
Char. 220: 0 → 1
Char. 231: 0 → 1
Char. 260: 2 → 0
Char. 272: 2 → 0
Char. 278: 3 → 0

***Microleter mckinzieorum*:**
Char. 0: 0 → 1
Char. 18: 0 → 1
Char. 39: 0 → 1
Char. 70: 0 → 1
Char. 94: 0 → 1
Char. 106: 0 → 1
Char. 110: 0 → 1
Char. 278: 3 → 1

**Millerettidae:**
Char. 23: 1 → 0
Char. 24: 0 → 1
Char. 25: 1 → 0
Char. 33: 1 → 0
Char. 44: 1 → 0
Char. 56: 0 → 1
Char. 66: 0 → 2
Char. 78: 1 → 0
Char. 80: 1 → 0
Char. 84: 1 → 2
Char. 88: 1 → 0
Char. 96: 1 → 0
Char. 121: 0 → 1
Char. 124: 0 → 1
Char. 127: 0 → 1
Char. 135: 0 → 1
Char. 145: 0 → 1
Char. 148: 1 → 0
Char. 159: 1 → 0
Char. 166: 1 → 0
Char. 180: 1 → 0
Char. 192: 1 → 0
Char. 202: 0 → 1
Char. 211: 0 → 1
Char. 230: 0 → 1
Char. 234: 0 → 1
Char. 248: 0 → 1
Char. 252: 0 → 1

***Nycteroleter ineptus*:**
Char. 66: 1 → 0

***Nyctiphruretus acudens*:**
Char. 21: 0 → 1
Char. 41: 0 → 1
Char. 66: 1 → 2
Char. 81: 1 → 0
Char. 84: 1 → 2
Char. 94: 0 → 1
Char. 166: 1 → 0
Char. 224: 0 → 1
Char. 266: 0 → 1
Char. 272: 2 → 1
Char. 276: 0 → 1

***Orovenator mayorum*:**
Char. 8: 0 → 1
Char. 21: 1 → 0
Char. 23: 1 → 0
Char. 24: 0 → 1
Char. 33: 1 → 0
Char. 36: 0 → 1
Char. 50: 0 → 1
Char. 92: 1 → 0
Char. 94: 1 → 0
Char. 135: 1 → 0
Char. 148: 1 → 0
Char. 159: 1 → 0
Char. 160: 0 → 1
Char. 165: 0 → 1
Char. 278: 0 → 1

***Owenetta* spp.:**
Char. 142: 1 → 0
Char. 169: 1 → 0

***Paleothyris acadiana*:**
Char. 23: 1 → 0
Char. 66: 1 → 2
Char. 102: 0 → 1
Char. 146: 1 → 0

***Placodus* spp.:**
Char. 0: 1 → 2
Char. 9: 0 → 1
Char. 12: 0 → 1
Char. 13: 0 → 1
Char. 19: 0 → 1
Char. 31: 0 → 1
Char. 46: 1 → 0
Char. 57: 0 → 1
Char. 78: 1 → 0
Char. 93: 1 → 0
Char. 102: 1 → 2
Char. 109: 1 → 0
Char. 140: 1 → 0
Char. 155: 0 → 1
Char. 163: 1 → 0
Char. 164: 0 → 1

***Procolophon* spp.:**
Char. 41: 0 → 1
Char. 69: 0 → 1
Char. 79: 0 → 1
Char. 86: 0 → 1
Char. 101: 0 → 1
Char. 141: 0 → 1
Char. 149: 1 → 0
Char. 203: 1 → 2
Char. 215: 0 → 1
Char. 230: 0 → 1
Char. 235: 1 → 2
Char. 237: 0 → 1
Char. 238: 0 → 1
Char. 272: 2 → 1
Char. 278: 3 → 0

***Prolacerta broomi*:**
Char. 58: 1 → 0
Char. 66: 1 → 0
Char. 67: 1 → 0
Char. 80: 1 → 0
Char. 139: 1 → 0
Char. 147: 1 → 0
Char. 192: 1 → 0
Char. 206: 0 → 12

***Rhipaeosaurus* spp.:**
Char. 172: 0 → 1

**Rhynchocephalia:**
Char. 0: 1 → 2
Char. 23: 1 → 0
Char. 24: 0 → 1
Char. 77: 0 → 1
Char. 88: 1 → 0
Char. 94: 1 → 0
Char. 139: 1 → 0
Char. 167: 1 → 0
Char. 205: 1 → 0

**Rhynchosauria:**
Char. 0: 1 → 0
Char. 7: 0 → 1
Char. 9: 0 → 1
Char. 26: 0 → 1
Char. 68: 0 → 1
Char. 99: 1 → 0
Char. 150: 1 → 0
Char. 160: 0 → 1
Char. 161: 0 → 1
Char. 171: 0 → 2
Char. 223: 0 → 1
Char. 224: 0 → 1
Char. 241: 0 → 1

***Scutosaurus* spp.:**
Char. 175: 0 → 1
Char. 190: 0 → 1
Char. 218: 0 → 1
Char. 243: 0 → 2
Char. 244: 0 → 1

***Sinosaurosphargis yunguiensis*:**
Char. 8: 0 → 1
Char. 30: 0 → 1
Char. 53: 0 → 1
Char. 89: 1 → 0
Char. 127: 1 → 0
Char. 150: 1 → 0
Char. 154: 0 → 2
Char. 167: 1 → 0
Char. 255: 0 → 1

**Squamata:**
Char. 45: 0 → 1
Char. 79: 0 → 2
Char. 80: 1 → 0
Char. 92: 1 → 0
Char. 109: 1 → 0
Char. 160: 0 → 1
Char. 200: 0 → 1
Char. 245: 0 → 1

***Trilophosaurus buettneri*:**
Char. 5: 1 → 0
Char. 11: 0 → 1
Char. 55: 1 → 0
Char. 93: 1 → 0
Char. 104: 0 → 1
Char. 106: 0 → 1
Char. 113: 0 → 1
Char. 122: 0 → 1
Char. 136: 1 → 0
Char. 144: 1 → 0
Char. 157: 0 → 1
Char. 159: 1 → 0
Char. 177: 0 → 12
Char. 194: 0 → 1
Char. 207: 1 → 0
Char. 208: 1 → 0
Char. 272: 1 → 0

***Youngina capensis*:**
Char. 5: 1 → 0
Char. 23: 1 → 0
Char. 25: 0 → 1
Char. 27: 0 → 1
Char. 38: 1 → 0
Char. 56: 0 → 1
Char. 73: 1 → 0
Char. 75: 0 → 1
Char. 84: 1 → 0
Char. 92: 1 → 0
Char. 94: 1 → 0
Char. 134: 0 → 1
Char. 145: 1 → 0
Char. 148: 1 → 0
Char. 151: 1 → 0
Char. 154: 1 → 0
Char. 163: 1 → 0
Char. 170: 0 → 1
Char. 211: 0 → 1
Char. 214: 0 → 1
Char. 215: 0 → 1
Char. 220: 1 → 0
Char. 224: 0 → 1
Char. 239: 0 → 1

**Node 50:**
Char. 46: 1 → 0
Char. 50: 0 → 1
Char. 88: 1 → 0
Char. 89: 1 → 0
Char. 93: 1 → 0
Char. 176: 0 → 1
Char. 195: 0 → 2

Char. 198: 0 → 1
Char. 240: 1 → 0
Char. 246: 1 → 2
Char. 259: 0 → 1
Char. 265: 1 → 0
Char. 270: 0 → 1

**Node 51:**
Char. 62: 1 → 0
Char. 65: 0 → 1
Char. 78: 1 → 0
Char. 131: 0 → 1
Char. 184: 0 → 1
Char. 193: 1 → 0
Char. 194: 0 → 2
Char. 205: 0 → 1
Char. 210: 0 → 1
Char. 219: 1 → 0
Char. 241: 0 → 1
Char. 246: 0 → 1
Char. 254: 0 → 1
Char. 255: 0 → 1
Char. 256: 0 → 1
Char. 268: 0 → 1
Char. 269: 0 → 1

**Node 52:**
Char. 0: 0 → 1
Char. 21: 0 → 1
Char. 25: 1 → 0
Char. 29: 1 → 0
Char. 40: 2 → 0
Char. 59: 0 → 1
Char. 60: 0 → 1
Char. 62: 0 → 1
Char. 67: 0 → 1
Char. 72: 1 → 2
Char. 73: 0 → 1
Char. 89: 0 → 1
Char. 92: 0 → 1
Char. 94: 0 → 1
Char. 109: 0 → 1
Char. 111: 0 → 1
Char. 120: 0 → 1
Char. 127: 0 → 1
Char. 129: 0 → 1
Char. 135: 0 → 1
Char. 136: 0 → 1
Char. 139: 0 → 1
Char. 141: 0 → 1
Char. 145: 0 → 1
Char. 151: 0 → 1
Char. 179: 0 → 1
Char. 187: 0 → 1
Char. 188: 0 → 1
Char. 191: 0 → 1
Char. 193: 0 → 1
Char. 196: 0 → 1
Char. 201: 1 → 0
Char. 202: 0 → 1
Char. 209: 0 → 1
Char. 217: 0 → 1
Char. 219: 0 → 1
Char. 220: 0 → 1
Char. 222: 0 → 1
Char. 231: 0 → 1
Char. 234: 0 → 1
Char. 237: 0 → 1
Char. 265: 0 → 1
Char. 266: 0 → 1
Char. 275: 1 → 0
Char. 278: 3 → 0

**Node 53:**
Char. 23: 0 → 1
Char. 33: 0 → 1
Char. 132: 0 → 1
Char. 166: 0 → 1
Char. 276: 1 → 0

**Node 54:**
Char. 29: 0 → 1
Char. 84: 0 → 1
Char. 88: 0 → 1
Char. 169: 0 → 1

**Node 55:**
Char. 72: 0 → 1
Char. 81: 0 → 1
Char. 93: 0 → 1
Char. 97: 0 → 1
Char. 104: 1 → 0
Char. 144: 0 → 1
Char. 173: 0 → 1
Char. 183: 0 → 1

**Node 57:**
Char. 95: 0 → 1
Char. 113: 0 → 1
Char. 114: 0 → 1

**Node 58:**
Char. 23: 1 → 0
Char. 47: 0 → 1
Char. 79: 0 → 1
Char. 110: 0 → 1
Char. 131: 0 → 1
Char. 137: 0 → 1
Char. 140: 0 → 2
Char. 147: 0 → 1
Char. 148: 1 → 0

**Node 59:**
Char. 29: 1 → 0
Char. 33: 1 → 0
Char. 59: 0 → 1
Char. 60: 0 → 1
Char. 67: 0 → 1
Char. 72: 1 → 2
Char. 84: 1 → 0
Char. 111: 0 → 1
Char. 116: 1 → 0
Char. 154: 1 → 0
Char. 159: 1 → 0
Char. 166: 1 → 0
Char. 169: 1 → 0
Char. 170: 0 → 1
Char. 197: 0 → 1
Char. 221: 1 → 0

**Node 60:**
Char. 19: 0 → 1
Char. 21: 1 → 0
Char. 92: 1 → 0

**Node 61:**
Char. 4: 0 → 1
Char. 15: 0 → 1
Char. 29: 0 → 1
Char. 213: 0 → 1
Char. 226: 0 → 2
Char. 228: 0 → 1
Char. 275: 0 → 1

**Node 62:**
Char. 58: 0 → 1
Char. 61: 0 → 1
Char. 66: 0 → 1
Char. 69: 0 → 1
Char. 107: 0 → 1
Char. 126: 0 → 1
Char. 131: 0 → 1
Char. 134: 0 → 1
Char. 140: 0 → 1
Char. 147: 0 → 1
Char. 150: 0 → 1
Char. 167: 0 → 1
Char. 176: 0 → 1
Char. 190: 0 → 1
Char. 205: 0 → 1
Char. 208: 0 → 1
Char. 214: 0 → 1
Char. 220: 1 → 0
Char. 230: 0 → 1
Char. 239: 0 → 1
Char. 260: 2 → 1

**Node 63:**
Char. 73: 0 → 1
Char. 205: 0 → 1
Char. 273: 0 → 1
Char. 276: 0 → 1

**Node 64:**
Char. 18: 0 → 1
Char. 25: 1 → 0
Char. 37: 0 → 1
Char. 103: 0 → 1
Char. 106: 0 → 1
Char. 107: 0 → 1
Char. 126: 0 → 1
Char. 150: 0 → 1

**Node 65:**
Char. 38: 1 → 2
Char. 39: 0 → 1
Char. 44: 1 → 0
Char. 49: 0 → 1
Char. 66: 0 → 1
Char. 80: 1 → 0
Char. 95: 0 → 1
Char. 135: 0 → 1
Char. 137: 0 → 1
Char. 147: 0 → 1
Char. 157: 0 → 1
Char. 158: 0 → 1
Char. 183: 1 → 2
Char. 186: 0 → 1
Char. 187: 0 → 1
Char. 194: 0 → 1
Char. 197: 0 → 1
Char. 201: 1 → 0
Char. 211: 0 → 1
Char. 252: 0 → 1

**Node 67:**
Char. 133: 0 → 1

**Node 68:**
Char. 100: 0 → 1
Char. 113: 0 → 1

**Node 69:**
Char. 23: 1 → 0
Char. 70: 0 → 1
Char. 118: 0 → 1
Char. 125: 0 → 1

**Node 71:**
Char. 38: 1 → 2
Char. 39: 0 → 1
Char. 58: 0 → 1
Char. 59: 0 → 1
Char. 60: 0 → 1
Char. 72: 1 → 2
Char. 85: 1 → 0
Char. 88: 1 → 0
Char. 95: 0 → 1
Char. 104: 0 → 1
Char. 105: 0 → 1
Char. 106: 0 → 2
Char. 107: 0 → 1
Char. 109: 0 → 1
Char. 110: 0 → 1
Char. 146: 1 → 0
Char. 155: 0 → 2
Char. 183: 1 → 2

**Node 72:**
Char. 23: 0 → 1
Char. 33: 1 → 0
Char. 38: 2 → 1
Char. 39: 1 → 0
Char. 42: 0 → 1
Char. 43: 0 → 1
Char. 46: 1 → 0
Char. 49: 1 → 0
Char. 71: 1 → 0
Char. 83: 0 → 2
Char. 101: 0 → 1
Char. 103: 0 → 1
Char. 106: 0 → 1
Char. 161: 0 → 1
Char. 163: 1 → 0
Char. 172: 0 → 2
Char. 188: 0 → 1
Char. 203: 1 → 2
Char. 212: 0 → 1
Char. 216: 1 → 0
Char. 221: 1 → 0
Char. 236: 0 → 1
Char. 238: 0 → 2
Char. 241: 0 → 1
Char. 242: 0 → 1
Char. 273: 0 → 1
Char. 274: 0 → 1
Char. 275: 1 → 0

**Node 73:**
Char. 52: 0 → 1
Char. 75: 0 → 1
Char. 84: 1 → 0
Char. 121: 0 → 1
Char. 180: 1 → 0
Char. 230: 0 → 1

**Node 74:**
Char. 277: 0 → 1

**Node 75:**
Char. 44: 1 → 0
Char. 66: 0 → 1
Char. 78: 1 → 0
Char. 88: 1 → 0
Char. 93: 1 → 0
Char. 123: 1 → 0
Char. 129: 0 → 1
Char. 192: 1 → 0

**Node 76:**
Char. 33: 1 → 0
Char. 85: 1 → 0
Char. 107: 0 → 1

**Node 77:**
Char. 2: 0 → 1
Char. 6: 0 → 1
Char. 186: 0 → 1
Char. 198: 0 → 1
Char. 200: 0 → 1
Char. 220: 0 → 1
Char. 239: 1 → 0

**Node 78:**
Char. 98: 1 → 0
Char. 113: 0 → 1
Char. 181: 0 → 1
Char. 206: 0 → 2

**Node 79:**
Char. 35: 0 → 1
Char. 102: 0 → 1
Char. 103: 0 → 1
Char. 184: 0 → 1
Char. 201: 0 → 1

Char. 210: 0 → 1
Char. 223: 0 → 1
Char. 229: 0 → 1

**Node 80:**
Char. 17: 0 → 1
Char. 42: 0 → 1
Char. 264: 0 → 1

**Node 81:**
Char. 41: 0 → 1
Char. 61: 1 → 2
Char. 112: 1 → 0
Char. 138: 0 → 1
Char. 146: 1 → 0
Char. 155: 0 → 1
Char. 192: 1 → 2
Char. 224: 0 → 1
Char. 226: 0 → 1
Char. 227: 0 → 1
Char. 233: 0 → 1
Char. 267: 01 → 2

**Node 82:**
Char. 61: 1 → 3
Char. 90: 0 → 1
Char. 91: 0 → 1
Char. 109: 1 → 0
Char. 155: 0 → 1
Char. 209: 1 → 0

**ANALYSIS 81**
**(NO *CANDELARIA BARBOURI*, NO CHARACTER 253)**

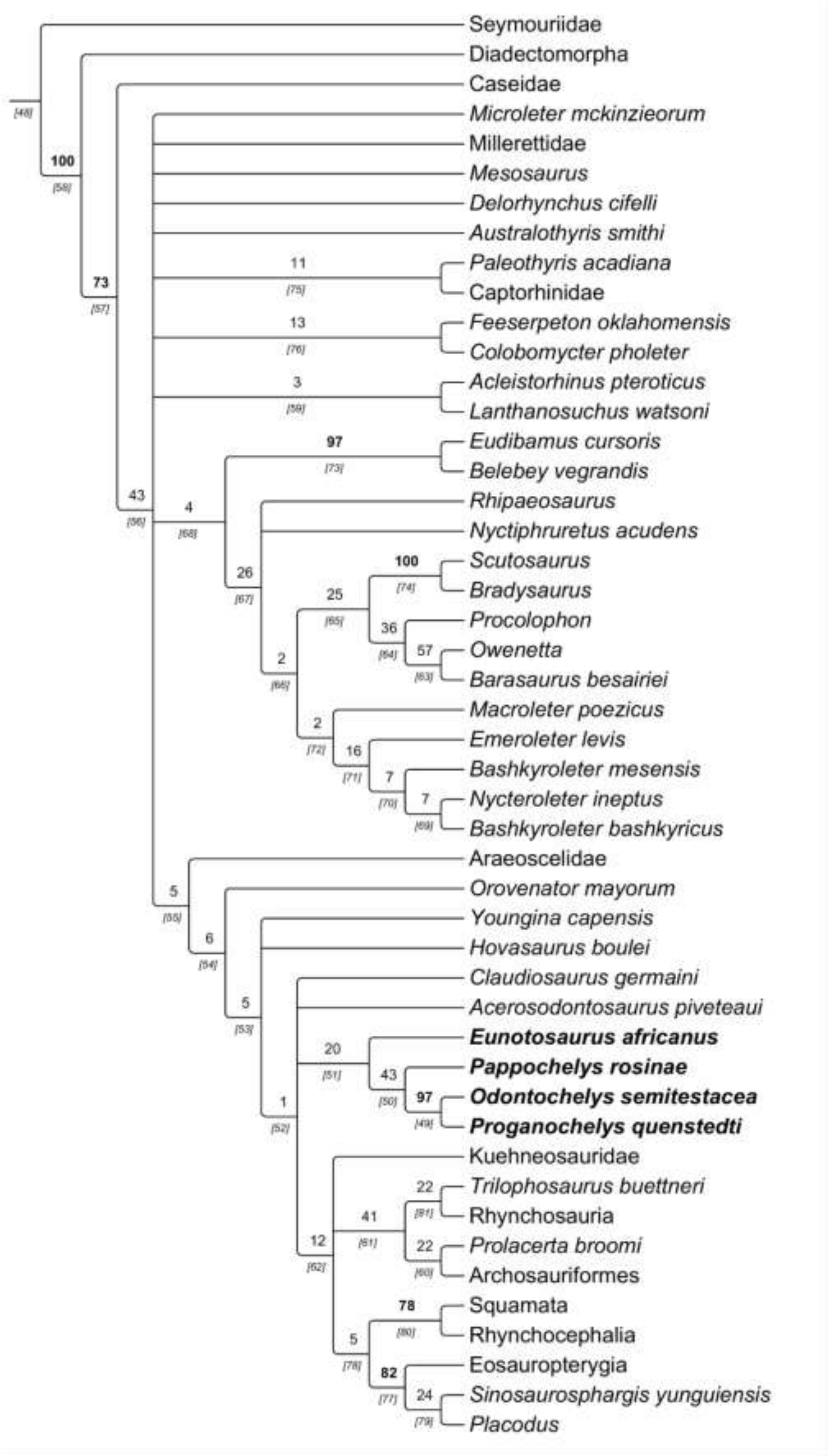

***Proganochelys quenstedti*:**
Char. 8: 0 → 1
Char. 11: 0 → 1
Char. 106: 0 → 1
Char. 108: 1 → 0
Char. 128: 0 → 1
Char. 175: 0 → 1
Char. 202: 1 → 0
Char. 207: 1 → 0
Char. 209: 1 → 0
Char. 244: 0 → 1
Char. 248: 0 → 1
Char. 250: 0 → 1
Char. 252: 1 → 0
Char. 262: 0 → 1

***Pappochelys rosinae*:**
Char. 0: 2 → 0
Char. 1: 0 → 1
Char. 5: 1 → 0
Char. 12: 0 → 1
Char. 41: 0 → 1
Char. 49: 0 → 1
Char. 75: 0 → 1
Char. 81: 0 → 1
Char. 169: 1 → 0
Char. 260: 2 → 0

***Eunotosaurus africanus*:**
Char. 19: 0 → 1
Char. 59: 1 → 0
Char. 60: 1 → 0
Char. 72: 2 → 1
Char. 76: 0 → 1
Char. 97: 1 → 0
Char. 103: 0 → 1
Char. 131: 1 → 0
Char. 153: 1 → 0
Char. 191: 1 → 0
Char. 192: 1 → 0
Char. 202: 1 → 0
Char. 211: 0 → 1
Char. 222: 1 → 0
Char. 237: 1 → 0
Char. 248: 0 → 1
Char. 249: 0 → 2
Char. 250: 0 → 1
Char. 263: 0 → 1
Char. 267: 1 → 0
Char. 272: 1 → 2
Char. 273: 0 → 1
Char. 274: 0 → 1
Char. 275: 0 → 1
Char. 276: 0 → 1
Char. 277: 0 → 1
Char. 278: 0 → 3

***Acerosodontosaurus piveteaui*:**
Char. 78: 1 → 0
Char. 127: 1 → 0
Char. 128: 0 → 1
Char. 155: 0 → 1
Char. 267: 1 → 0
Char. 278: 0 → 1

***Acleistorhinus pteroticus:***
Char. 21: 0 → 1
Char. 146: 1 → 0
Char. 169: 1 → 0
Char. 170: 0 → 1

**Araeoscelidae:**
Char. 23: 0 → 1
Char. 28: 0 → 1
Char. 106: 0 → 1
Char. 116: 1 → 0
Char. 117: 01 → 2
Char. 169: 1 → 0
Char. 170: 0 → 1
Char. 197: 0 → 1
Char. 239: 0 → 1

**Archosauriformes:**
Char. 32: 0 → 1
Char. 94: 1 → 0
Char. 112: 1 → 0
Char. 152: 0 → 1
Char. 154: 0 → 2
Char. 166: 1 → 0
Char. 171: 0 → 1
Char. 185: 0 → 1
Char. 204: 0 → 1
Char. 218: 0 → 3
Char. 224: 0 → 1
Char. 242: 0 → 1

***Australothyris smithi*:**
Char. 23: 0 → 1
Char. 24: 0 → 1
Char. 34: 0 → 1
Char. 55: 1 → 0
Char. 57: 1 → 0
Char. 71: 1 → 0
Char. 79: 0 → 1
Char. 85: 1 → 0
Char. 98: 1 → 0
Char. 100: 0 → 1
Char. 103: 0 → 1
Char. 110: 0 → 1
Char. 112: 0 → 1
Char. 123: 1 → 0
Char. 129: 0 → 1
Char. 131: 0 → 1
Char. 132: 1 → 0
Char. 144: 1 → 0
Char. 147: 0 → 1
Char. 149: 1 → 0
Char. 150: 0 → 1

***Barasaurus besairiei*:**
Char. 33: 1 → 0
Char. 75: 1 → 0
Char. 216: 0 → 1

***Bashkyroleter bashkyricus*:**
Char. 275: 1 → 0

***Bashkyroleter mesensis*:**
Char. 169: 1 → 0

***Belebey vegrandis*:**
Char. 154: 1 → 0

***Bradysaurus* spp.:**
Char. 19: 0 → 1
Char. 73: 0 → 1
Char. 79: 0 → 1
Char. 135: 1 → 0
Char. 249: 0 → 1

**Captorhinidae:**
Char. 3: 0 → 1
Char. 23: 0 → 1
Char. 25: 1 → 0
Char. 26: 0 → 1
Char. 73: 0 → 1
Char. 75: 0 → 1
Char. 108: 1 → 0
Char. 183: 1 → 0
Char. 201: 1 → 0
Char. 203: 1 → 2
Char. 216: 1 → 0

**Caseidae:**
Char. 24: 0 → 1
Char. 25: 1 → 0
Char. 36: 0 → 1
Char. 38: 1 → 0
Char. 46: 1 → 0
Char. 50: 1 → 0
Char. 56: 0 → 1
Char. 170: 0 → 1
Char. 194: 0 → 1
Char. 273: 0 → 1
Char. 274: 0 → 1
Char. 278: 3 → 2

***Claudiosaurus germaini*:**
Char. 24: 0 → 1
Char. 34: 0 → 1
Char. 36: 0 → 1
Char. 43: 1 → 0
Char. 56: 0 → 1
Char. 81: 0 → 1
Char. 105: 0 → 1
Char. 106: 0 → 1
Char. 117: 01 → 2
Char. 127: 1 → 0
Char. 141: 1 → 0
Char. 144: 1 → 0
Char. 166: 1 → 0
Char. 187: 1 → 0
Char. 190: 0 → 1
Char. 199: 0 → 1
Char. 204: 0 → 1
Char. 208: 1 → 0
Char. 222: 1 → 0
Char. 234: 1 → 0

***Colobomycter pholeter*:**
Char. 21: 0 → 1
Char. 25: 1 → 0
Char. 84: 1 → 0
Char. 154: 1 → 0
Char. 167: 0 → 1
Char. 267: 0 → 1

***Delorhynchus cifelli*:**
Char. 18: 0 → 1
Char. 20: 0 → 1
Char. 21: 0 → 1
Char. 24: 0 → 1
Char. 26: 0 → 1
Char. 28: 0 → 1
Char. 33: 0 → 2
Char. 39: 0 → 1
Char. 52: 0 → 1
Char. 100: 0 → 1
Char. 111: 0 → 1
Char. 116: 1 → 0
Char. 117: 0 → 1
Char. 131: 0 → 1
Char. 147: 0 → 1
Char. 156: 1 → 0
Char. 159: 0 → 1
Char. 167: 0 → 1
Char. 180: 0 → 1
Char. 189: 0 → 1
Char. 191: 0 → 1
Char. 192: 0 → 1
Char. 204: 0 → 2
Char. 267: 0 → 1

**Diadectomorpha:**
Char. 0: 0 → 1
Char. 64: 0 → 1
Char. 70: 0 → 1
Char. 122: 0 → 1
Char. 123: 1 → 0
Char. 146: 1 → 0
Char. 275: 1 → 0
Char. 278: 3 → 0

***Emeroleter levis*:**
Char. 51: 1 → 0

**Eosauropterygia:**
Char. 159: 1 → 0
Char. 166: 1 → 0
Char. 174: 0 → 1
Char. 194: 0 → 2
Char. 272: 1 → 0

***Eudibamus cursoris*:**
Char. 154: 1 → 2

***Feeserpeton oklahomensis*:**
Char. 51: 0 → 1
Char. 70: 0 → 1
Char. 157: 0 → 1
Char. 158: 0 → 1

***Hovasaurus boulei*:**
Char. 41: 0 → 1
Char. 55: 1 → 0
Char. 60: 1 → 0
Char. 72: 2 → 1
Char. 77: 0 → 2
Char. 78: 1 → 0
Char. 79: 0 → 1
Char. 93: 1 → 0
Char. 113: 0 → 1
Char. 138: 0 → 1
Char. 141: 1 → 0
Char. 146: 1 → 0
Char. 154: 0 → 1
Char. 204: 0 → 2
Char. 278: 0 → 3

**Kuehneosauridae:**
Char. 7: 0 → 1
Char. 24: 0 → 1
Char. 26: 0 → 1
Char. 34: 0 → 1
Char. 36: 0 → 1
Char. 43: 1 → 0
Char. 56: 0 → 1
Char. 79: 0 → 2
Char. 82: 0 → 1
Char. 98: 1 → 0
Char. 108: 1 → 0
Char. 113: 0 → 1
Char. 128: 0 → 1
Char. 148: 1 → 0
Char. 159: 1 → 0
Char. 181: 0 → 1
Char. 185: 0 → 1
Char. 245: 0 → 1
Char. 272: 1 → 2
Char. 278: 0 → 3

***Lanthanosuchus watsoni*:**
Char. 25: 1 → 0
Char. 51: 0 → 1
Char. 76: 0 → 1
Char. 86: 0 → 1
Char. 98: 1 → 0
Char. 138: 0 → 1
Char. 144: 1 → 0
Char. 154: 1 → 2

***Macroleter poezicus*:**
Char. 52: 0 → 1
Char. 84: 1 → 0
Char. 87: 0 → 1
Char. 140: 0 → 1
Char. 146: 1 → 0
Char. 169: 1 → 0
Char. 235: 0 → 1

***Mesosaurus* spp.:**
Char. 0: 0 → 1

Char. 2: 0 → 1
Char. 6: 0 → 1
Char. 8: 0 → 1
Char. 9: 0 → 1
Char. 13: 0 → 1
Char. 19: 0 → 1
Char. 23: 0 → 1
Char. 26: 0 → 1
Char. 29: 1 → 0
Char. 33: 0 → 1
Char. 38: 1 → 0
Char. 41: 0 → 1
Char. 50: 1 → 0
Char. 67: 0 → 1
Char. 76: 0 → 1
Char. 84: 1 → 0
Char. 85: 1 → 0
Char. 94: 0 → 1
Char. 107: 0 → 1
Char. 109: 0 → 1
Char. 111: 0 → 1
Char. 115: 0 → 1
Char. 146: 1 → 0
Char. 148: 0 → 1
Char. 149: 1 → 0
Char. 164: 0 → 1
Char. 167: 0 → 1
Char. 176: 0 → 1
Char. 183: 1 → 0
Char. 184: 0 → 1
Char. 199: 0 → 1
Char. 202: 0 → 1
Char. 204: 0 → 2
Char. 206: 0 → 1
Char. 207: 0 → 1
Char. 209: 0 → 1
Char. 217: 0 → 1
Char. 219: 0 → 1
Char. 220: 0 → 1
Char. 231: 0 → 1
Char. 260: 2 → 0
Char. 272: 2 → 0
Char. 278: 3 → 0

***Microleter mckinzieorum*:**
Char. 0: 0 → 1
Char. 18: 0 → 1
Char. 24: 0 → 1
Char. 25: 1 → 0
Char. 36: 0 → 1
Char. 39: 0 → 1
Char. 51: 0 → 2
Char. 56: 0 → 1
Char. 57: 1 → 0
Char. 70: 0 → 1
Char. 76: 0 → 1
Char. 79: 0 → 1
Char. 94: 0 → 1
Char. 106: 0 → 1
Char. 110: 0 → 1
Char. 132: 1 → 0
Char. 159: 0 → 1

Char. 276: 0 → 1
Char. 278: 3 → 1

**Millerettidae:**
Char. 24: 0 → 1
Char. 25: 1 → 0
Char. 44: 1 → 0
Char. 56: 0 → 1
Char. 57: 1 → 0
Char. 66: 0 → 2
Char. 78: 1 → 0
Char. 80: 1 → 0
Char. 84: 1 → 2
Char. 88: 1 → 0
Char. 96: 1 → 0
Char. 117: 0 → 1
Char. 124: 0 → 1
Char. 127: 0 → 1
Char. 135: 0 → 1
Char. 145: 0 → 1
Char. 202: 0 → 1
Char. 211: 0 → 1
Char. 230: 0 → 1
Char. 234: 0 → 1
Char. 248: 0 → 1
Char. 252: 0 → 1

***Nycteroleter ineptus*:**
Char. 278: 0 → 3

***Nyctiphruretus acudens*:**
Char. 21: 0 → 1
Char. 33: 1 → 2
Char. 41: 0 → 1
Char. 81: 1 → 0
Char. 84: 1 → 2
Char. 94: 0 → 1
Char. 167: 0 → 1
Char. 215: 1 → 0
Char. 224: 0 → 1
Char. 266: 0 → 1
Char. 272: 2 → 1
Char. 276: 0 → 1

***Orovenator mayorum*:**
Char. 8: 0 → 1
Char. 24: 0 → 1
Char. 36: 0 → 1
Char. 160: 0 → 1
Char. 165: 0 → 1

***Owenetta* spp.:**
Char. 169: 1 → 0

***Paleothyris acadiana*:**
Char. 38: 1 → 0
Char. 50: 1 → 0
Char. 66: 1 → 2
Char. 102: 0 → 1
Char. 146: 1 → 0
Char. 180: 0 → 1
Char. 237: 0 → 1
Char. 239: 0 → 1

***Placodus* spp.:**
Char. 0: 1 → 2
Char. 9: 0 → 1
Char. 12: 0 → 1
Char. 13: 0 → 1
Char. 19: 0 → 1
Char. 26: 0 → 1
Char. 31: 0 → 1
Char. 46: 1 → 0
Char. 57: 0 → 1
Char. 78: 1 → 0
Char. 93: 1 → 0
Char. 102: 1 → 2
Char. 109: 1 → 0
Char. 140: 1 → 0
Char. 155: 0 → 1
Char. 163: 1 → 0
Char. 164: 0 → 1

***Procolophon* spp.:**
Char. 41: 0 → 1
Char. 69: 0 → 1
Char. 79: 0 → 1
Char. 88: 0 → 1
Char. 117: 1 → 0
Char. 149: 1 → 0
Char. 180: 0 → 1
Char. 204: 0 → 1
Char. 237: 0 → 1
Char. 238: 0 → 1
Char. 272: 2 → 1
Char. 278: 3 → 0

***Prolacerta broomi*:**
Char. 58: 1 → 0
Char. 66: 1 → 0
Char. 67: 1 → 0
Char. 80: 1 → 0
Char. 139: 1 → 0
Char. 192: 1 → 0
Char. 203: 1 → 2

***Rhipaeosaurus* spp.:**
Char. 172: 0 → 1
Char. 277: 0 → 1

**Rhynchocephalia:**
Char. 0: 1 → 2
Char. 24: 0 → 1
Char. 77: 0 → 1
Char. 94: 1 → 0
Char. 139: 1 → 0
Char. 167: 1 → 0
Char. 205: 1 → 0

**Rhynchosauria:**
Char. 0: 1 → 0
Char. 7: 0 → 1
Char. 9: 0 → 1
Char. 26: 0 → 1
Char. 68: 0 → 1
Char. 99: 1 → 0
Char. 150: 1 → 0

Char. 160: 0 → 1
Char. 161: 0 → 1
Char. 171: 0 → 2
Char. 223: 0 → 1
Char. 224: 0 → 1
Char. 241: 0 → 1

***Scutosaurus* spp.:**
Char. 175: 0 → 1
Char. 218: 0 → 1
Char. 243: 0 → 2
Char. 244: 0 → 1
Char. 251: 0 → 1

***Sinosaurosphargis yunguiensis*:**
Char. 8: 0 → 1
Char. 30: 0 → 1
Char. 53: 0 → 1
Char. 82: 0 → 1
Char. 89: 1 → 0
Char. 127: 1 → 0
Char. 150: 1 → 0
Char. 154: 0 → 2
Char. 167: 1 → 0
Char. 255: 0 → 1

**Squamata:**
Char. 26: 0 → 1
Char. 45: 0 → 1
Char. 79: 0 → 2
Char. 80: 1 → 0
Char. 82: 0 → 1
Char. 92: 1 → 0
Char. 109: 1 → 0
Char. 160: 0 → 1
Char. 245: 0 → 1

***Trilophosaurus buettneri*:**
Char. 5: 1 → 0
Char. 11: 0 → 1
Char. 55: 1 → 0
Char. 93: 1 → 0
Char. 104: 0 → 1
Char. 106: 0 → 1
Char. 113: 0 → 1
Char. 122: 0 → 1
Char. 136: 1 → 0
Char. 144: 1 → 0
Char. 154: 0 → 1
Char. 157: 0 → 1
Char. 159: 1 → 0
Char. 177: 0 → 12
Char. 194: 0 → 1
Char. 203: 1 → 2
Char. 207: 1 → 0
Char. 208: 1 → 0
Char. 272: 1 → 0

***Youngina capensis*:**
Char. 56: 0 → 1
Char. 75: 0 → 1
Char. 170: 0 → 1

Char. 211: 0 → 1
Char. 239: 0 → 1

**Node 49:**
Char. 46: 1 → 0
Char. 88: 1 → 0
Char. 89: 1 → 0
Char. 93: 1 → 0
Char. 176: 0 → 1
Char. 195: 0 → 2
Char. 198: 0 → 1
Char. 246: 1 → 2
Char. 259: 0 → 1
Char. 265: 1 → 0
Char. 270: 0 → 1

**Node 50:**
Char. 65: 0 → 1
Char. 184: 0 → 1
Char. 205: 0 → 1
Char. 210: 0 → 1
Char. 219: 1 → 0
Char. 241: 0 → 1
Char. 246: 0 → 1
Char. 254: 0 → 1
Char. 255: 0 → 1
Char. 256: 0 → 1
Char. 268: 0 → 1
Char. 269: 0 → 1

**Node 51:**
Char. 0: 1 → 2
Char. 33: 1 → 2
Char. 62: 1 → 0
Char. 84: 01 → 2
Char. 152: 0 → 1
Char. 154: 0 → 1
Char. 155: 0 → 1
Char. 158: 0 → 1
Char. 161: 0 → 1
Char. 174: 0 → 1
Char. 181: 0 → 1
Char. 204: 0 → 2
Char. 247: 0 → 1
Char. 251: 0 → 1
Char. 252: 0 → 1

**Node 52:**
Char. 23: 0 → 1
Char. 73: 0 → 1
Char. 81: 1 → 0
Char. 94: 0 → 1
Char. 131: 0 → 1
Char. 145: 0 → 1
Char. 148: 0 → 1
Char. 151: 0 → 1
Char. 208: 0 → 1
Char. 217: 0 → 1
Char. 224: 1 → 0
Char. 272: 2 → 1

**Node 53:**
Char. 33: 0 → 1

Char. 135: 0 → 1
Char. 159: 0 → 1
Char. 267: 0 → 1
Char. 278: 1 → 0

**Node 54:**
Char. 20: 0 → 1
Char. 62: 0 → 1
Char. 141: 0 → 1

**Node 55:**
Char. 0: 0 → 1
Char. 29: 1 → 0
Char. 40: 2 → 0
Char. 57: 1 → 0
Char. 59: 0 → 1
Char. 60: 0 → 1
Char. 67: 0 → 1
Char. 72: 1 → 2
Char. 89: 0 → 1
Char. 111: 0 → 1
Char. 112: 0 → 1
Char. 120: 0 → 1
Char. 154: 1 → 0
Char. 180: 0 → 1
Char. 192: 0 → 1
Char. 193: 0 → 1
Char. 222: 0 → 1
Char. 224: 0 → 1
Char. 234: 0 → 1
Char. 237: 0 → 1
Char. 266: 0 → 1
Char. 278: 3 → 1

**Node 56:**
Char. 29: 0 → 1
Char. 40: 0 → 2
Char. 74: 0 → 1
Char. 80: 0 → 1
Char. 84: 0 → 1
Char. 88: 0 → 1
Char. 116: 0 → 1
Char. 132: 0 → 1
Char. 149: 0 → 1
Char. 156: 0 → 1
Char. 169: 0 → 1
Char. 201: 0 → 1
Char. 203: 0 → 1
Char. 235: 0 → 1

**Node 57:**
Char. 72: 0 → 1
Char. 79: 1 → 0
Char. 81: 0 → 1
Char. 93: 0 → 1
Char. 97: 0 → 1
Char. 104: 1 → 0
Char. 144: 0 → 1
Char. 173: 0 → 1
Char. 183: 0 → 1

**Node 59:**
Char. 95: 0 → 1
Char. 110: 0 → 1
Char. 113: 0 → 1
Char. 114: 0 → 1
Char. 131: 0 → 1
Char. 137: 0 → 1
Char. 140: 0 → 2
Char. 147: 0 → 1

**Node 60:**
Char. 19: 0 → 1
Char. 92: 1 → 0

**Node 61:**
Char. 4: 0 → 1
Char. 29: 0 → 1
Char. 48: 1 → 0
Char. 213: 0 → 1
Char. 226: 0 → 2
Char. 228: 0 → 1
Char. 275: 0 → 1

**Node 62:**
Char. 58: 0 → 1
Char. 61: 0 → 1
Char. 66: 0 → 1
Char. 69: 0 → 1
Char. 150: 0 → 1
Char. 167: 0 → 1
Char. 176: 0 → 1
Char. 190: 0 → 1
Char. 205: 0 → 1
Char. 230: 0 → 1
Char. 239: 0 → 1
Char. 260: 2 → 1

**Node 63:**
Char. 73: 0 → 1
Char. 131: 1 → 0
Char. 205: 0 → 1
Char. 276: 0 → 1

**Node 64:**
Char. 18: 0 → 1
Char. 37: 0 → 1
Char. 107: 0 → 1
Char. 118: 1 → 0
Char. 125: 1 → 0
Char. 150: 0 → 1

**Node 65:**
Char. 23: 0 → 1
Char. 71: 1 → 0
Char. 102: 0 → 1
Char. 103: 0 → 1
Char. 106: 0 → 1
Char. 167: 0 → 1
Char. 214: 0 → 1
Char. 216: 1 → 0
Char. 235: 0 → 2
Char. 241: 0 → 1

**Node 66:**
Char. 138: 0 → 1
Char. 141: 0 → 1
Char. 207: 0 → 1

**Node 67:**
Char. 20: 0 → 1
Char. 33: 0 → 1
Char. 44: 1 → 0
Char. 70: 0 → 1
Char. 80: 1 → 0
Char. 117: 0 → 1
Char. 118: 0 → 1
Char. 125: 0 → 1
Char. 131: 0 → 1
Char. 147: 0 → 1
Char. 194: 0 → 1
Char. 201: 1 → 0
Char. 211: 0 → 1

**Node 68:**
Char. 38: 1 → 2
Char. 39: 0 → 1
Char. 49: 0 → 1
Char. 66: 0 → 12
Char. 76: 0 → 1
Char. 85: 1 → 0
Char. 88: 1 → 0
Char. 95: 0 → 1
Char. 112: 0 → 1
Char. 148: 0 → 1
Char. 158: 0 → 1
Char. 159: 0 → 1
Char. 183: 1 → 2
Char. 192: 0 → 1
Char. 215: 0 → 1

**Node 69:**
Char. 87: 0 → 1

**Node 70:**
Char. 25: 1 → 0
Char. 76: 1 → 0

**Node 71:**
Char. 79: 0 → 1
Char. 93: 1 → 0
Char. 133: 0 → 1

**Node 72:**
Char. 85: 0 → 1
Char. 147: 1 → 2
Char. 154: 1 → 0
Char. 278: 3 → 0

**Node 73:**
Char. 50: 1 → 0
Char. 58: 0 → 1
Char. 59: 0 → 1
Char. 60: 0 → 1
Char. 72: 1 → 2
Char. 104: 0 → 1

Char. 105: 0 → 1
Char. 106: 0 → 2
Char. 107: 0 → 1
Char. 109: 0 → 1
Char. 110: 0 → 1
Char. 146: 1 → 0
Char. 155: 0 → 2

**Node 74:**
Char. 33: 1 → 0
Char. 38: 2 → 1
Char. 39: 1 → 0
Char. 42: 0 → 1
Char. 43: 0 → 1
Char. 46: 1 → 0
Char. 49: 1 → 0
Char. 52: 0 → 1
Char. 83: 01 → 2
Char. 84: 1 → 0
Char. 87: 0 → 1
Char. 93: 1 → 0
Char. 161: 0 → 1
Char. 163: 1 → 0
Char. 172: 0 → 2
Char. 174: 0 → 1
Char. 188: 0 → 1
Char. 189: 0 → 1
Char. 195: 0 → 1
Char. 204: 0 → 2
Char. 212: 0 → 1
Char. 236: 0 → 1
Char. 238: 0 → 2
Char. 242: 0 → 1
Char. 274: 0 → 1
Char. 275: 1 → 0

**Node 75:**
Char. 29: 1 → 0
Char. 44: 1 → 0
Char. 59: 0 → 1
Char. 60: 0 → 1
Char. 66: 0 → 1
Char. 67: 0 → 1
Char. 72: 1 → 2
Char. 78: 1 → 0
Char. 84: 1 → 0
Char. 88: 1 → 0
Char. 93: 1 → 0
Char. 111: 0 → 1
Char. 123: 1 → 0
Char. 129: 0 → 1
Char. 154: 1 → 0
Char. 169: 1 → 0
Char. 170: 0 → 1
Char. 197: 0 → 1

**Node 76:**
Char. 20: 0 → 1
Char. 47: 0 → 1
Char. 85: 1 → 0
Char. 107: 0 → 1
Char. 110: 0 → 1

Char. 169: 1 → 0
Char. 170: 0 → 1

**Node 77:**
Char. 2: 0 → 1
Char. 6: 0 → 1
Char. 98: 1 → 0
Char. 101: 0 → 1
Char. 113: 0 → 1
Char. 181: 0 → 1
Char. 186: 0 → 1
Char. 193: 1 → 0
Char. 198: 0 → 1
Char. 220: 0 → 1
Char. 234: 1 → 0
Char. 239: 1 → 0
Char. 240: 1 → 0
Char. 266: 1 → 0

**Node 78:**
Char. 33: 1 → 2
Char. 68: 0 → 1
Char. 102: 0 → 1
Char. 103: 0 → 1
Char. 223: 0 → 1
Char. 229: 0 → 1
Char. 235: 1 → 2

**Node 79:**
Char. 17: 0 → 1
Char. 42: 0 → 1
Char. 251: 0 → 1
Char. 264: 0 → 1

**Node 80:**
Char. 41: 0 → 1
Char. 48: 1 → 0
Char. 61: 1 → 2
Char. 112: 1 → 0
Char. 128: 0 → 1
Char. 138: 0 → 1
Char. 146: 1 → 0
Char. 155: 0 → 1
Char. 192: 1 → 2
Char. 224: 0 → 1
Char. 226: 0 → 1
Char. 227: 0 → 1
Char. 233: 0 → 1

**Node 81:**
Char. 61: 1 → 3
Char. 90: 0 → 1
Char. 91: 0 → 1
Char. 109: 1 → 0
Char. 155: 0 → 1
Char. 209: 1 → 0

**ANALYSIS 82**
**(NO *CANDELARIA BARBOURI*, IMPLIED WEIGHTING, K = 2.75, NO CHARACTER 253)**

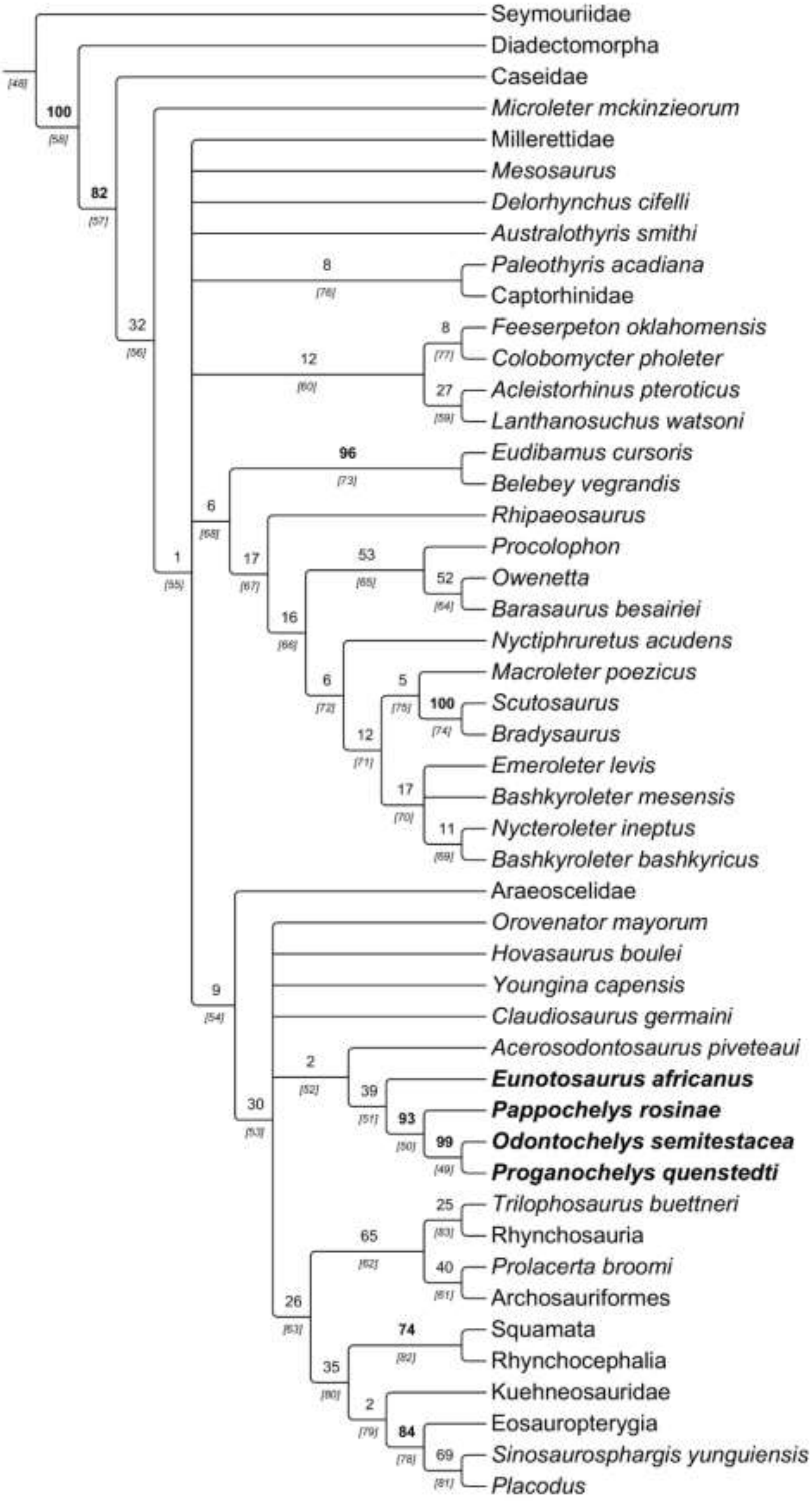

***Proganochelys quenstedti*:**
Char. 8: 0 → 1
Char. 11: 0 → 1
Char. 106: 0 → 1
Char. 108: 1 → 0
Char. 128: 0 → 1
Char. 175: 0 → 1
Char. 202: 1 → 0
Char. 207: 1 → 0
Char. 209: 1 → 0
Char. 244: 0 → 1
Char. 248: 0 → 1
Char. 250: 0 → 1
Char. 252: 1 → 0
Char. 262: 0 → 1

***Pappochelys rosinae*:**
Char. 0: 2 → 0
Char. 1: 0 → 1
Char. 5: 1 → 0
Char. 12: 0 → 1
Char. 41: 0 → 1
Char. 49: 0 → 1
Char. 75: 0 → 1
Char. 81: 0 → 1
Char. 169: 1 → 0
Char. 260: 2 → 0

***Eunotosaurus africanus*:**
Char. 19: 0 → 1
Char. 59: 1 → 0
Char. 60: 1 → 0
Char. 72: 2 → 1
Char. 76: 0 → 1
Char. 97: 1 → 0
Char. 103: 0 → 1
Char. 131: 1 → 0
Char. 153: 1 → 0
Char. 191: 1 → 0
Char. 192: 1 → 0
Char. 202: 1 → 0
Char. 211: 0 → 1
Char. 222: 1 → 0
Char. 237: 1 → 0
Char. 248: 0 → 1
Char. 249: 0 → 2
Char. 250: 0 → 1
Char. 263: 0 → 1
Char. 267: 1 → 0
Char. 272: 1 → 2
Char. 273: 0 → 1
Char. 274: 0 → 1
Char. 275: 0 → 1
Char. 276: 0 → 1
Char. 277: 0 → 1
Char. 278: 0 → 3

***Acerosodontosaurus piveteaui*:**
Char. 78: 1 → 0
Char. 127: 1 → 0
Char. 128: 0 → 1
Char. 206: 0 → 2
Char. 267: 1 → 0
Char. 278: 0 → 1

***Acleistorhinus pteroticus:***
Char. 21: 0 → 1
Char. 146: 1 → 0

**Araeoscelidae:**
Char. 23: 0 → 1
Char. 28: 0 → 1
Char. 106: 0 → 1
Char. 116: 1 → 0
Char. 117: 01 → 2
Char. 169: 1 → 0
Char. 170: 0 → 1
Char. 197: 0 → 1
Char. 239: 0 → 1

**Archosauriformes:**
Char. 32: 0 → 1
Char. 94: 1 → 0
Char. 112: 1 → 0
Char. 152: 0 → 1
Char. 154: 0 → 2
Char. 166: 1 → 0
Char. 171: 0 → 1
Char. 185: 0 → 1
Char. 201: 0 → 1
Char. 204: 0 → 1
Char. 218: 0 → 3
Char. 224: 0 → 1
Char. 242: 0 → 1

***Australothyris smithi*:**
Char. 23: 0 → 1
Char. 24: 0 → 1
Char. 34: 0 → 1
Char. 55: 1 → 0
Char. 57: 1 → 0
Char. 71: 1 → 0
Char. 79: 0 → 1
Char. 85: 1 → 0
Char. 98: 1 → 0
Char. 100: 0 → 1
Char. 103: 0 → 1
Char. 110: 0 → 1
Char. 112: 0 → 1
Char. 123: 1 → 0
Char. 129: 0 → 1
Char. 131: 0 → 1
Char. 132: 1 → 0
Char. 144: 1 → 0
Char. 147: 0 → 1
Char. 149: 1 → 0
Char. 150: 0 → 1

***Barasaurus besairiei*:**
Char. 33: 1 → 0

***Bashkyroleter bashkyricus*:**
Char. 275: 1 → 0

***Bashkyroleter mesensis*:**
Char. 12: 0 → 1
Char. 66: 1 → 0
Char. 169: 1 → 0

***Belebey vegrandis*:**
Char. 154: 1 → 0

***Bradysaurus* spp.:**
Char. 19: 0 → 1
Char. 25: 1 → 0
Char. 73: 0 → 1
Char. 79: 0 → 1
Char. 135: 1 → 0
Char. 249: 0 → 1

**Captorhinidae:**
Char. 3: 0 → 1
Char. 23: 0 → 1
Char. 25: 1 → 0
Char. 26: 0 → 1
Char. 73: 0 → 1
Char. 75: 0 → 1
Char. 108: 1 → 0
Char. 183: 1 → 0
Char. 201: 1 → 0
Char. 203: 1 → 2
Char. 216: 1 → 0

**Caseidae:**
Char. 38: 1 → 0
Char. 46: 1 → 0
Char. 50: 1 → 0
Char. 170: 0 → 1
Char. 194: 0 → 1
Char. 273: 0 → 1
Char. 274: 0 → 1
Char. 278: 3 → 2

***Claudiosaurus germaini*:**
Char. 21: 1 → 0
Char. 24: 0 → 1
Char. 34: 0 → 1
Char. 36: 0 → 1
Char. 43: 1 → 0
Char. 56: 0 → 1
Char. 81: 0 → 1
Char. 105: 0 → 1
Char. 106: 0 → 1
Char. 117: 01 → 2
Char. 127: 1 → 0
Char. 141: 1 → 0
Char. 144: 1 → 0
Char. 166: 1 → 0
Char. 187: 1 → 0
Char. 190: 0 → 1
Char. 199: 0 → 1
Char. 201: 0 → 1
Char. 204: 0 → 1
Char. 208: 1 → 0
Char. 222: 1 → 0
Char. 234: 1 → 0

***Colobomycter pholeter*:**
Char. 21: 0 → 1
Char. 25: 1 → 0
Char. 84: 1 → 0
Char. 154: 1 → 0
Char. 167: 0 → 1
Char. 267: 0 → 1

***Delorhynchus cifelli*:**
Char. 5: 0 → 1
Char. 18: 0 → 1
Char. 20: 0 → 1
Char. 21: 0 → 1
Char. 24: 0 → 1
Char. 26: 0 → 1
Char. 28: 0 → 1
Char. 33: 0 → 2
Char. 39: 0 → 1
Char. 52: 0 → 1
Char. 100: 0 → 1
Char. 111: 0 → 1
Char. 116: 1 → 0
Char. 131: 0 → 1
Char. 147: 0 → 1
Char. 156: 1 → 0
Char. 159: 0 → 1
Char. 163: 0 → 1
Char. 167: 0 → 1
Char. 189: 0 → 1
Char. 191: 0 → 1
Char. 192: 0 → 1
Char. 204: 0 → 2
Char. 267: 0 → 1

**Diadectomorpha:**
Char. 0: 0 → 1
Char. 64: 0 → 1
Char. 70: 0 → 1
Char. 122: 0 → 1
Char. 123: 1 → 0
Char. 146: 1 → 0
Char. 275: 1 → 0
Char. 278: 3 → 0

***Emeroleter levis*:**
Char. 51: 1 → 0

**Eosauropterygia:**
Char. 166: 1 → 0
Char. 174: 0 → 1
Char. 178: 0 → 1
Char. 194: 0 → 2
Char. 272: 1 → 0

***Eudibamus cursoris*:**
Char. 154: 1 → 2

***Feeserpeton oklahomensis*:**
Char. 51: 0 → 1
Char. 157: 0 → 1
Char. 158: 0 → 1

***Hovasaurus boulei*:**
Char. 41: 0 → 1
Char. 55: 1 → 0
Char. 60: 1 → 0
Char. 72: 2 → 1
Char. 77: 0 → 2
Char. 78: 1 → 0
Char. 79: 0 → 1
Char. 93: 1 → 0
Char. 113: 0 → 1
Char. 138: 0 → 1
Char. 141: 1 → 0
Char. 146: 1 → 0
Char. 154: 0 → 1
Char. 204: 0 → 2
Char. 206: 0 → 2
Char. 278: 0 → 3

**Kuehneosauridae:**
Char. 7: 0 → 1
Char. 21: 1 → 0
Char. 24: 0 → 1
Char. 27: 0 → 1
Char. 43: 1 → 0
Char. 79: 0 → 2
Char. 107: 1 → 0
Char. 140: 1 → 0
Char. 147: 1 → 0
Char. 148: 1 → 0
Char. 245: 0 → 1
Char. 272: 1 → 2
Char. 278: 0 → 3

***Lanthanosuchus watsoni*:**
Char. 25: 1 → 0
Char. 51: 0 → 1
Char. 76: 0 → 1
Char. 86: 0 → 1
Char. 98: 1 → 0
Char. 138: 0 → 1
Char. 144: 1 → 0
Char. 154: 1 → 2

***Macroleter poezicus*:**
Char. 9: 0 → 1
Char. 26: 0 → 1
Char. 66: 1 → 2
Char. 126: 0 → 1
Char. 134: 0 → 2
Char. 140: 0 → 1
Char. 146: 1 → 0
Char. 169: 1 → 0

***Mesosaurus* spp.:**
Char. 0: 0 → 1
Char. 2: 0 → 1
Char. 6: 0 → 1
Char. 8: 0 → 1
Char. 9: 0 → 1
Char. 13: 0 → 1
Char. 19: 0 → 1
Char. 23: 0 → 1
Char. 26: 0 → 1

Char. 29: 1 → 0
Char. 33: 0 → 1
Char. 38: 1 → 0
Char. 41: 0 → 1
Char. 50: 1 → 0
Char. 67: 0 → 1
Char. 76: 0 → 1
Char. 84: 1 → 0
Char. 85: 1 → 0
Char. 94: 0 → 1
Char. 107: 0 → 1
Char. 109: 0 → 1
Char. 111: 0 → 1
Char. 115: 0 → 1
Char. 146: 1 → 0
Char. 148: 0 → 1
Char. 149: 1 → 0
Char. 164: 0 → 1
Char. 167: 0 → 1
Char. 176: 0 → 1
Char. 183: 1 → 0
Char. 184: 0 → 1
Char. 199: 0 → 1
Char. 202: 0 → 1
Char. 204: 0 → 2
Char. 206: 0 → 1
Char. 207: 0 → 1
Char. 209: 0 → 1
Char. 217: 0 → 1
Char. 219: 0 → 1
Char. 220: 0 → 1
Char. 231: 0 → 1
Char. 260: 2 → 0
Char. 272: 2 → 0
Char. 278: 3 → 0

***Microleter mckinzieorum*:**
Char. 0: 0 → 1
Char. 18: 0 → 1
Char. 39: 0 → 1
Char. 57: 1 → 0
Char. 70: 0 → 1
Char. 76: 0 → 1
Char. 94: 0 → 1
Char. 106: 0 → 1
Char. 110: 0 → 1
Char. 159: 0 → 1
Char. 278: 3 → 1

**Millerettidae:**
Char. 24: 0 → 1
Char. 25: 1 → 0
Char. 44: 1 → 0
Char. 48: 1 → 0
Char. 56: 0 → 1
Char. 57: 1 → 0
Char. 66: 0 → 2
Char. 78: 1 → 0
Char. 80: 1 → 0
Char. 84: 1 → 2
Char. 88: 1 → 0
Char. 96: 1 → 0
Char. 121: 0 → 1
Char. 124: 0 → 1
Char. 127: 0 → 1
Char. 135: 0 → 1
Char. 145: 0 → 1
Char. 202: 0 → 1
Char. 211: 0 → 1
Char. 230: 0 → 1
Char. 234: 0 → 1
Char. 248: 0 → 1
Char. 252: 0 → 1

***Nycteroleter ineptus*:**
Char. 66: 1 → 0
Char. 278: 0 → 3

***Nyctiphruretus acudens*:**
Char. 21: 0 → 1
Char. 41: 0 → 1
Char. 48: 1 → 0
Char. 66: 1 → 2
Char. 81: 1 → 0
Char. 84: 1 → 2
Char. 94: 0 → 1
Char. 166: 1 → 0
Char. 215: 1 → 0
Char. 224: 0 → 1
Char. 226: 1 → 0
Char. 266: 0 → 1
Char. 272: 2 → 1
Char. 276: 0 → 1

***Orovenator mayorum*:**
Char. 8: 0 → 1
Char. 24: 0 → 1
Char. 36: 0 → 1
Char. 48: 1 → 0
Char. 160: 0 → 1
Char. 165: 0 → 1

***Owenetta* spp.:**
Char. 142: 1 → 0
Char. 169: 1 → 0

***Paleothyris acadiana*:**
Char. 38: 1 → 0
Char. 50: 1 → 0
Char. 66: 1 → 2
Char. 102: 0 → 1
Char. 146: 1 → 0
Char. 237: 0 → 1
Char. 239: 0 → 1

***Placodus* spp.:**
Char. 0: 1 → 2
Char. 9: 0 → 1
Char. 12: 0 → 1
Char. 13: 0 → 1
Char. 19: 0 → 1
Char. 31: 0 → 1
Char. 46: 1 → 0
Char. 57: 0 → 1
Char. 78: 1 → 0
Char. 93: 1 → 0
Char. 102: 1 → 2
Char. 109: 1 → 0
Char. 140: 1 → 0
Char. 155: 0 → 1
Char. 163: 1 → 0
Char. 164: 0 → 1

***Procolophon* spp.:**
Char. 41: 0 → 1
Char. 69: 0 → 1
Char. 79: 0 → 1
Char. 86: 0 → 1
Char. 88: 0 → 1
Char. 101: 0 → 1
Char. 141: 0 → 1
Char. 149: 1 → 0
Char. 203: 1 → 2
Char. 204: 0 → 1
Char. 230: 0 → 1
Char. 237: 0 → 1
Char. 238: 0 → 1
Char. 239: 1 → 0
Char. 272: 2 → 1
Char. 278: 3 → 0

***Prolacerta broomi*:**
Char. 58: 1 → 0
Char. 66: 1 → 0
Char. 67: 1 → 0
Char. 80: 1 → 0
Char. 139: 1 → 0
Char. 147: 1 → 0
Char. 192: 1 → 0
Char. 206: 0 → 12

***Rhipaeosaurus* spp.:**
Char. 172: 0 → 1
Char. 277: 0 → 1

**Rhynchocephalia:**
Char. 0: 1 → 2
Char. 23: 1 → 0
Char. 24: 0 → 1
Char. 77: 0 → 1
Char. 81: 0 → 1
Char. 88: 1 → 0
Char. 94: 1 → 0
Char. 139: 1 → 0
Char. 167: 1 → 0
Char. 205: 1 → 0

**Rhynchosauria:**
Char. 0: 1 → 0
Char. 7: 0 → 1
Char. 9: 0 → 1
Char. 26: 0 → 1
Char. 68: 0 → 1
Char. 99: 1 → 0
Char. 150: 1 → 0
Char. 160: 0 → 1
Char. 161: 0 → 1
Char. 171: 0 → 2
Char. 223: 0 → 1
Char. 224: 0 → 1
Char. 241: 0 → 1

***Scutosaurus* spp.:**
Char. 175: 0 → 1
Char. 190: 0 → 1
Char. 218: 0 → 1
Char. 243: 0 → 2
Char. 244: 0 → 1
Char. 251: 0 → 1

***Sinosaurosphargis yunguiensis*:**
Char. 8: 0 → 1
Char. 30: 0 → 1
Char. 53: 0 → 1
Char. 89: 1 → 0
Char. 127: 1 → 0
Char. 150: 1 → 0
Char. 154: 0 → 2
Char. 167: 1 → 0
Char. 255: 0 → 1

**Squamata:**
Char. 45: 0 → 1
Char. 79: 0 → 2
Char. 80: 1 → 0
Char. 92: 1 → 0
Char. 109: 1 → 0
Char. 160: 0 → 1
Char. 200: 0 → 1
Char. 245: 0 → 1

***Trilophosaurus buettneri*:**
Char. 5: 1 → 0
Char. 11: 0 → 1
Char. 55: 1 → 0
Char. 93: 1 → 0
Char. 104: 0 → 1
Char. 106: 0 → 1
Char. 113: 0 → 1
Char. 122: 0 → 1
Char. 136: 1 → 0
Char. 144: 1 → 0
Char. 154: 0 → 1
Char. 157: 0 → 1
Char. 159: 1 → 0
Char. 177: 0 → 12
Char. 194: 0 → 1
Char. 207: 1 → 0
Char. 208: 1 → 0
Char. 272: 1 → 0

***Youngina capensis*:**
Char. 56: 0 → 1
Char. 75: 0 → 1
Char. 170: 0 → 1
Char. 211: 0 → 1
Char. 239: 0 → 1

**Node 49:**
Char. 46: 1 → 0
Char. 88: 1 → 0
Char. 89: 1 → 0
Char. 93: 1 → 0
Char. 176: 0 → 1
Char. 195: 0 → 2
Char. 198: 0 → 1
Char. 246: 1 → 2
Char. 259: 0 → 1
Char. 265: 1 → 0
Char. 270: 0 → 1

**Node 50:**
Char. 65: 0 → 1
Char. 184: 0 → 1
Char. 205: 0 → 1
Char. 210: 0 → 1
Char. 219: 1 → 0
Char. 241: 0 → 1
Char. 246: 0 → 1
Char. 254: 0 → 1
Char. 255: 0 → 1
Char. 256: 0 → 1
Char. 268: 0 → 1
Char. 269: 0 → 1

**Node 51:**
Char. 0: 1 → 2
Char. 33: 1 → 2
Char. 62: 1 → 0
Char. 84: 01 → 2
Char. 152: 0 → 1
Char. 154: 0 → 1
Char. 158: 0 → 1
Char. 161: 0 → 1
Char. 174: 0 → 1
Char. 181: 0 → 1
Char. 204: 0 → 2
Char. 251: 0 → 1
Char. 252: 0 → 1

**Node 52:**
Char. 23: 0 → 1
Char. 73: 0 → 1
Char. 81: 1 → 0
Char. 94: 0 → 1
Char. 131: 0 → 1
Char. 145: 0 → 1
Char. 148: 0 → 1
Char. 151: 0 → 1
Char. 208: 0 → 1
Char. 217: 0 → 1
Char. 224: 1 → 0
Char. 272: 2 → 1

**Node 53:**
Char. 21: 0 → 1
Char. 33: 0 → 1
Char. 135: 0 → 1
Char. 159: 0 → 1
Char. 267: 0 → 1
Char. 278: 1 → 0

**Node 54:**
Char. 20: 0 → 1

Char. 62: 0 → 1
Char. 141: 0 → 1

**Node 55:**
Char. 0: 0 → 1
Char. 29: 1 → 0
Char. 40: 2 → 0
Char. 57: 1 → 0
Char. 59: 0 → 1
Char. 60: 0 → 1
Char. 67: 0 → 1
Char. 72: 1 → 2
Char. 89: 0 → 1
Char. 111: 0 → 1
Char. 112: 0 → 1
Char. 120: 0 → 1
Char. 154: 1 → 0
Char. 192: 0 → 1
Char. 193: 0 → 1
Char. 222: 0 → 1
Char. 224: 0 → 1
Char. 234: 0 → 1
Char. 237: 0 → 1
Char. 266: 0 → 1
Char. 278: 3 → 1

**Node 56:**
Char. 48: 0 → 1
Char. 132: 0 → 1
Char. 276: 1 → 0

**Node 57:**
Char. 29: 0 → 1
Char. 84: 0 → 1
Char. 88: 0 → 1
Char. 169: 0 → 1

**Node 58:**
Char. 72: 0 → 1
Char. 81: 0 → 1
Char. 93: 0 → 1
Char. 97: 0 → 1
Char. 104: 1 → 0
Char. 144: 0 → 1
Char. 173: 0 → 1
Char. 183: 0 → 1

**Node 60:**
Char. 95: 0 → 1
Char. 113: 0 → 1
Char. 114: 0 → 1

**Node 61:**
Char. 5: 0 → 1
Char. 20: 0 → 1
Char. 47: 0 → 1
Char. 79: 0 → 1
Char. 110: 0 → 1
Char. 131: 0 → 1
Char. 137: 0 → 1
Char. 140: 0 → 2
Char. 147: 0 → 1
Char. 159: 0 → 1
Char. 163: 0 → 1

**Node 62:**
Char. 19: 0 → 1
Char. 21: 1 → 0
Char. 92: 1 → 0

**Node 63:**
Char. 4: 0 → 1
Char. 29: 0 → 1
Char. 213: 0 → 1
Char. 226: 0 → 2
Char. 228: 0 → 1
Char. 275: 0 → 1

**Node 64:**
Char. 58: 0 → 1
Char. 61: 0 → 1
Char. 66: 0 → 1
Char. 69: 0 → 1
Char. 107: 0 → 1
Char. 140: 0 → 1
Char. 150: 0 → 1
Char. 167: 0 → 1
Char. 176: 0 → 1
Char. 190: 0 → 1
Char. 205: 0 → 1
Char. 230: 0 → 1
Char. 239: 0 → 1
Char. 260: 2 → 1

**Node 65:**
Char. 73: 0 → 1
Char. 205: 0 → 1
Char. 273: 0 → 1
Char. 276: 0 → 1

**Node 66:**
Char. 18: 0 → 1
Char. 25: 1 → 0
Char. 37: 0 → 1
Char. 106: 0 → 1
Char. 107: 0 → 1
Char. 110: 0 → 1
Char. 126: 0 → 1
Char. 150: 0 → 1

**Node 67:**
Char. 186: 0 → 1

**Node 68:**
Char. 194: 0 → 1
Char. 201: 1 → 0
Char. 211: 0 → 1
Char. 226: 0 → 1

**Node 69:**
Char. 5: 0 → 1
Char. 38: 1 → 2
Char. 39: 0 → 1
Char. 49: 0 → 1
Char. 66: 0 → 1
Char. 76: 0 → 1
Char. 85: 1 → 0
Char. 88: 1 → 0
Char. 95: 0 → 1
Char. 112: 0 → 1
Char. 148: 0 → 1
Char. 158: 0 → 1
Char. 159: 0 → 1
Char. 183: 1 → 2
Char. 192: 0 → 1
Char. 215: 0 → 1
Char. 239: 0 → 1

**Node 70:**
Char. 87: 0 → 1
Char. 110: 0 → 1

**Node 71:**
Char. 79: 0 → 1
Char. 133: 0 → 1

**Node 72:**
Char. 100: 0 → 1
Char. 113: 0 → 1

**Node 73:**
Char. 70: 0 → 1
Char. 118: 0 → 1
Char. 125: 0 → 1

**Node 74:**
Char. 58: 0 → 1
Char. 59: 0 → 1
Char. 60: 0 → 1
Char. 72: 1 → 2
Char. 104: 0 → 1
Char. 105: 0 → 1
Char. 106: 0 → 2
Char. 107: 0 → 1
Char. 109: 0 → 1
Char. 110: 0 → 1
Char. 146: 1 → 0

**Node 75:**
Char. 23: 0 → 1
Char. 33: 1 → 0
Char. 38: 2 → 1
Char. 39: 1 → 0
Char. 42: 0 → 1
Char. 43: 0 → 1
Char. 46: 1 → 0
Char. 48: 1 → 0
Char. 49: 1 → 0
Char. 71: 1 → 0
Char. 83: 0 → 2
Char. 101: 0 → 1
Char. 103: 0 → 1
Char. 106: 0 → 1
Char. 161: 0 → 1
Char. 163: 1 → 0
Char. 172: 0 → 2
Char. 174: 0 → 1
Char. 188: 0 → 1
Char. 203: 1 → 2
Char. 212: 0 → 1
Char. 216: 1 → 0
Char. 236: 0 → 1
Char. 238: 0 → 2
Char. 239: 1 → 0
Char. 241: 0 → 1
Char. 242: 0 → 1
Char. 273: 0 → 1
Char. 274: 0 → 1
Char. 275: 1 → 0

**Node 76:**
Char. 52: 0 → 1
Char. 75: 0 → 1
Char. 84: 1 → 0
Char. 87: 0 → 1
Char. 110: 0 → 1
Char. 121: 0 → 1
Char. 230: 0 → 1

**Node 77:**
Char. 29: 1 → 0
Char. 44: 1 → 0
Char. 48: 1 → 0
Char. 59: 0 → 1
Char. 60: 0 → 1
Char. 66: 0 → 1
Char. 67: 0 → 1
Char. 72: 1 → 2
Char. 78: 1 → 0
Char. 84: 1 → 0
Char. 88: 1 → 0
Char. 93: 1 → 0
Char. 111: 0 → 1
Char. 123: 1 → 0
Char. 129: 0 → 1
Char. 154: 1 → 0
Char. 169: 1 → 0
Char. 170: 0 → 1
Char. 197: 0 → 1

**Node 78:**
Char. 85: 1 → 0
Char. 107: 0 → 1

**Node 79:**
Char. 2: 0 → 1
Char. 6: 0 → 1
Char. 81: 0 → 1
Char. 186: 0 → 1
Char. 198: 0 → 1
Char. 200: 0 → 1
Char. 220: 0 → 1
Char. 239: 1 → 0
Char. 267: 1 → 0

**Node 80:**
Char. 98: 1 → 0
Char. 113: 0 → 1
Char. 181: 0 → 1
Char. 206: 0 → 2

**Node 81:**
Char. 35: 0 → 1
Char. 102: 0 → 1
Char. 103: 0 → 1
Char. 184: 0 → 1
Char. 201: 0 → 1
Char. 210: 0 → 1
Char. 223: 0 → 1
Char. 229: 0 → 1

**Node 82:**
Char. 17: 0 → 1
Char. 42: 0 → 1
Char. 251: 0 → 1
Char. 264: 0 → 1

**Node 83:**
Char. 41: 0 → 1
Char. 61: 1 → 2
Char. 112: 1 → 0
Char. 138: 0 → 1
Char. 146: 1 → 0
Char. 192: 1 → 2
Char. 224: 0 → 1
Char. 226: 0 → 1
Char. 227: 0 → 1
Char. 233: 0 → 1
Char. 267: 1 → 2

**Node 84:**
Char. 61: 1 → 3
Char. 90: 0 → 1
Char. 91: 0 → 1
Char. 109: 1 → 0
Char. 209: 1 → 0

**ANALYSIS 83**
**(NO *CANDELARIA BARBOURI*, NO CHARACTERS 247 AND 253)**

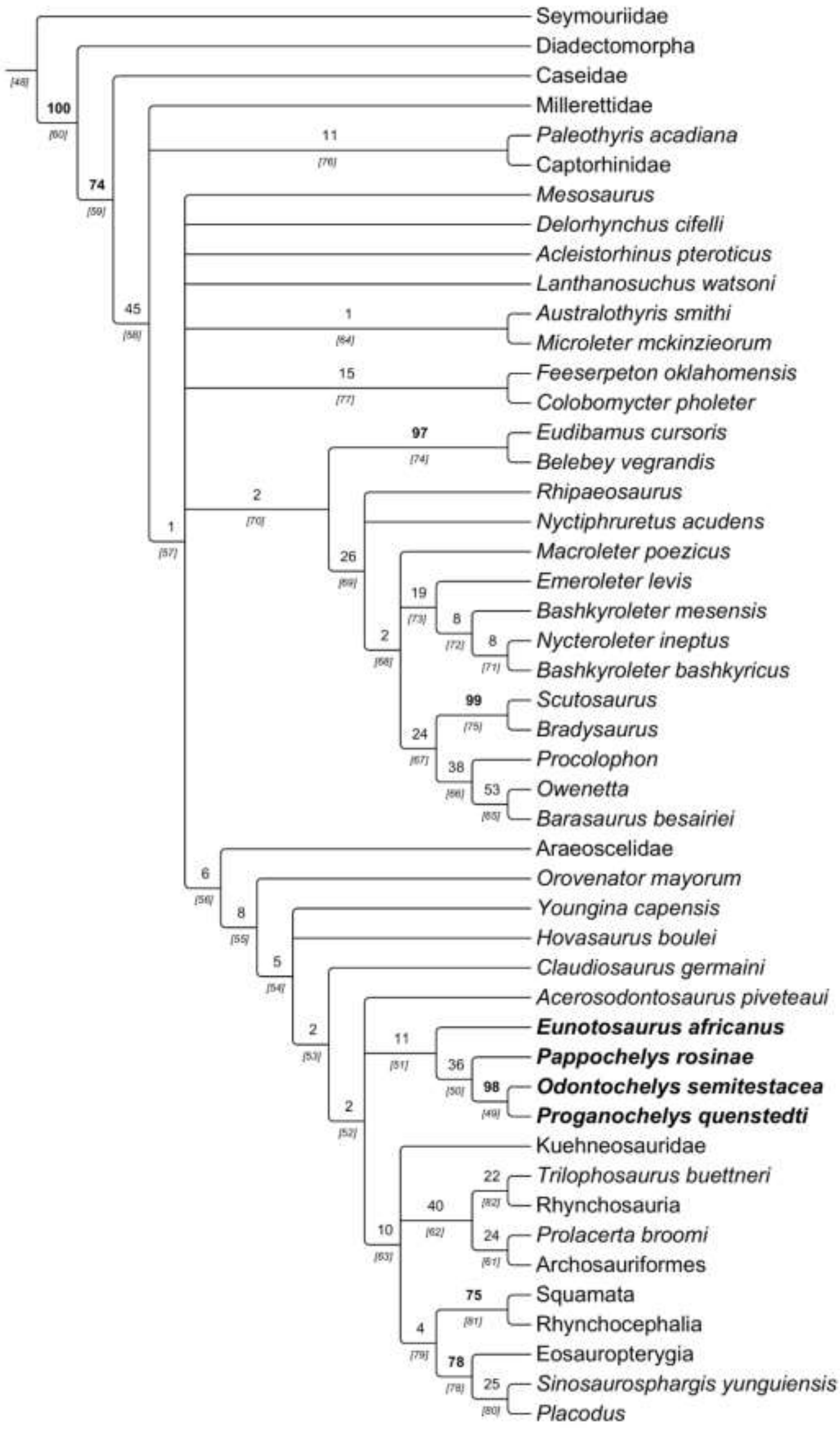

***Proganochelys quenstedti*:**
Char. 8: 0 → 1
Char. 11: 0 → 1
Char. 106: 0 → 1
Char. 108: 1 → 0
Char. 128: 0 → 1
Char. 175: 0 → 1
Char. 202: 1 → 0
Char. 206: 2 → 0
Char. 207: 1 → 0
Char. 209: 1 → 0
Char. 244: 0 → 1
Char. 248: 0 → 1
Char. 250: 0 → 1
Char. 252: 1 → 0
Char. 262: 0 → 1

***Pappochelys rosinae*:**
Char. 0: 2 → 0
Char. 1: 0 → 1
Char. 5: 1 → 0
Char. 12: 0 → 1
Char. 41: 0 → 1
Char. 49: 0 → 1
Char. 75: 0 → 1
Char. 81: 0 → 1
Char. 169: 1 → 0
Char. 260: 2 → 0

***Eunotosaurus africanus*:**
Char. 19: 0 → 1
Char. 59: 1 → 0
Char. 60: 1 → 0
Char. 72: 2 → 1
Char. 76: 0 → 1
Char. 97: 1 → 0
Char. 103: 0 → 1
Char. 131: 1 → 0
Char. 153: 1 → 0
Char. 191: 1 → 0
Char. 192: 1 → 0
Char. 202: 1 → 0
Char. 211: 0 → 1
Char. 222: 1 → 0
Char. 237: 1 → 0
Char. 248: 0 → 1
Char. 249: 0 → 2
Char. 250: 0 → 1
Char. 263: 0 → 1
Char. 267: 1 → 0
Char. 272: 1 → 2
Char. 273: 0 → 1
Char. 274: 0 → 1
Char. 275: 0 → 1
Char. 276: 0 → 1
Char. 277: 0 → 1
Char. 278: 0 → 3

***Acerosodontosaurus piveteaui*:**
Char. 127: 1 → 0
Char. 128: 0 → 1
Char. 267: 1 → 0
Char. 278: 0 → 1

***Acleistorhinus pteroticus*:**
Char. 21: 0 → 1
Char. 30: 0 → 1
Char. 33: 0 → 1
Char. 47: 0 → 1
Char. 55: 1 → 0
Char. 56: 0 → 1
Char. 64: 0 → 1
Char. 70: 0 → 1
Char. 79: 0 → 1
Char. 95: 0 → 1
Char. 113: 0 → 1
Char. 114: 0 → 1
Char. 140: 0 → 2
Char. 146: 1 → 0
Char. 169: 1 → 0
Char. 170: 0 → 1

**Araeoscelidae:**
Char. 5: 1 → 0
Char. 23: 0 → 1
Char. 28: 0 → 1
Char. 106: 0 → 1
Char. 116: 1 → 0
Char. 117: 01 → 2
Char. 169: 1 → 0
Char. 170: 0 → 1
Char. 197: 0 → 1
Char. 221: 1 → 0
Char. 239: 0 → 1

**Archosauriformes:**
Char. 32: 0 → 1
Char. 94: 1 → 0
Char. 112: 1 → 0
Char. 152: 0 → 1
Char. 154: 0 → 2
Char. 166: 1 → 0
Char. 171: 0 → 1
Char. 185: 0 → 1
Char. 204: 0 → 1
Char. 218: 0 → 3
Char. 224: 0 → 1
Char. 242: 0 → 1

***Australothyris smithi*:**
Char. 23: 0 → 1
Char. 34: 0 → 1
Char. 55: 1 → 0
Char. 71: 1 → 0
Char. 85: 1 → 0
Char. 112: 0 → 1
Char. 129: 0 → 1
Char. 159: 1 → 0

***Barasaurus besairiei*:**
Char. 33: 1 → 0
Char. 75: 1 → 0
Char. 216: 0 → 1

***Bashkyroleter bashkyricus*:**
Char. 275: 1 → 0

***Bashkyroleter mesensis*:**
Char. 169: 1 → 0

***Belebey vegrandis*:**
Char. 154: 1 → 0

***Bradysaurus* spp.:**
Char. 19: 0 → 1
Char. 73: 0 → 1
Char. 79: 0 → 1
Char. 135: 1 → 0
Char. 249: 0 → 1

**Captorhinidae:**
Char. 3: 0 → 1
Char. 23: 0 → 1
Char. 26: 0 → 1
Char. 73: 0 → 1
Char. 75: 0 → 1
Char. 83: 0 → 1
Char. 108: 1 → 0
Char. 183: 1 → 0
Char. 201: 1 → 0
Char. 203: 1 → 2
Char. 216: 1 → 0

**Caseidae:**
Char. 24: 0 → 1
Char. 36: 0 → 1
Char. 38: 1 → 0
Char. 46: 1 → 0
Char. 50: 1 → 0
Char. 56: 0 → 1
Char. 170: 0 → 1
Char. 194: 0 → 1
Char. 273: 0 → 1
Char. 274: 0 → 1
Char. 278: 3 → 2

***Claudiosaurus germaini*:**
Char. 24: 0 → 1
Char. 34: 0 → 1
Char. 36: 0 → 1
Char. 43: 1 → 0
Char. 56: 0 → 1
Char. 105: 0 → 1
Char. 106: 0 → 1
Char. 117: 01 → 2
Char. 127: 1 → 0
Char. 144: 1 → 0
Char. 187: 1 → 0
Char. 190: 0 → 1
Char. 199: 0 → 1
Char. 222: 1 → 0
Char. 234: 1 → 0

***Colobomycter pholeter*:**
Char. 21: 0 → 1
Char. 25: 1 → 0
Char. 84: 1 → 0
Char. 154: 1 → 0
Char. 167: 0 → 1
Char. 267: 0 → 1

***Delorhynchus cifelli*:**
Char. 18: 0 → 1
Char. 21: 0 → 1
Char. 24: 0 → 1
Char. 26: 0 → 1
Char. 28: 0 → 1
Char. 33: 0 → 2
Char. 39: 0 → 1
Char. 52: 0 → 1
Char. 100: 0 → 1
Char. 111: 0 → 1
Char. 116: 1 → 0
Char. 117: 0 → 1
Char. 156: 1 → 0
Char. 167: 0 → 1
Char. 189: 0 → 1
Char. 191: 0 → 1
Char. 267: 0 → 1

**Diadectomorpha:**
Char. 0: 0 → 1
Char. 44: 0 → 1
Char. 64: 0 → 1
Char. 70: 0 → 1
Char. 122: 0 → 1
Char. 123: 1 → 0
Char. 146: 1 → 0
Char. 275: 1 → 0
Char. 278: 3 → 0

***Emeroleter levis*:**
Char. 51: 1 → 0

**Eosauropterygia:**
Char. 159: 1 → 0
Char. 166: 1 → 0
Char. 174: 0 → 1
Char. 194: 0 → 2
Char. 272: 1 → 0

***Eudibamus cursoris*:**
Char. 154: 1 → 2
Char. 163: 1 → 0

***Feeserpeton oklahomensis*:**
Char. 51: 0 → 1
Char. 70: 0 → 1
Char. 157: 0 → 1
Char. 158: 0 → 1

***Hovasaurus boulei*:**
Char. 41: 0 → 1
Char. 55: 1 → 0
Char. 60: 1 → 0
Char. 72: 2 → 1
Char. 77: 0 → 2
Char. 78: 1 → 0
Char. 79: 0 → 1
Char. 93: 1 → 0
Char. 113: 0 → 1
Char. 138: 0 → 1
Char. 146: 1 → 0
Char. 154: 0 → 1
Char. 206: 0 → 2
Char. 278: 0 → 3

**Kuehneosauridae:**
Char. 7: 0 → 1
Char. 24: 0 → 1
Char. 26: 0 → 1
Char. 27: 0 → 1
Char. 34: 0 → 1
Char. 36: 0 → 1
Char. 43: 1 → 0
Char. 56: 0 → 1
Char. 79: 0 → 2
Char. 82: 0 → 1
Char. 98: 1 → 0
Char. 108: 1 → 0
Char. 113: 0 → 1
Char. 128: 0 → 1
Char. 147: 1 → 0
Char. 148: 1 → 0
Char. 159: 1 → 0
Char. 185: 0 → 1
Char. 245: 0 → 1
Char. 272: 1 → 2
Char. 278: 0 → 3

***Lanthanosuchus watsoni*:**
Char. 25: 1 → 0
Char. 51: 0 → 1
Char. 86: 0 → 1
Char. 95: 0 → 1
Char. 98: 1 → 0
Char. 113: 0 → 1
Char. 114: 0 → 1
Char. 138: 0 → 1
Char. 140: 0 → 2
Char. 144: 1 → 0
Char. 154: 1 → 2

***Macroleter poezicus*:**
Char. 9: 0 → 1
Char. 26: 0 → 1
Char. 52: 0 → 1
Char. 84: 1 → 0
Char. 87: 0 → 1
Char. 134: 0 → 2
Char. 140: 0 → 1
Char. 146: 1 → 0
Char. 169: 1 → 0
Char. 235: 0 → 1

***Mesosaurus* spp.:**
Char. 0: 0 → 1
Char. 2: 0 → 1
Char. 5: 1 → 0
Char. 6: 0 → 1
Char. 8: 0 → 1
Char. 9: 0 → 1
Char. 13: 0 → 1

Char. 19: 0 → 1
Char. 23: 0 → 1
Char. 26: 0 → 1
Char. 29: 1 → 0
Char. 33: 0 → 1
Char. 38: 1 → 0
Char. 41: 0 → 1
Char. 50: 1 → 0
Char. 67: 0 → 1
Char. 83: 0 → 1
Char. 84: 1 → 0
Char. 85: 1 → 0
Char. 94: 0 → 1
Char. 107: 0 → 1
Char. 109: 0 → 1
Char. 111: 0 → 1
Char. 115: 0 → 1
Char. 131: 1 → 0
Char. 146: 1 → 0
Char. 147: 1 → 0
Char. 148: 0 → 1
Char. 149: 1 → 0
Char. 159: 1 → 0
Char. 163: 1 → 0
Char. 164: 0 → 1
Char. 166: 1 → 0
Char. 167: 0 → 1
Char. 176: 0 → 1
Char. 183: 1 → 0
Char. 184: 0 → 1
Char. 192: 1 → 0
Char. 199: 0 → 1
Char. 202: 0 → 1
Char. 206: 0 → 1
Char. 207: 0 → 1
Char. 209: 0 → 1
Char. 217: 0 → 1
Char. 219: 0 → 1
Char. 220: 0 → 1
Char. 231: 0 → 1
Char. 260: 2 → 0
Char. 272: 2 → 0
Char. 278: 13 → 0

***Microleter mckinzieorum*:**
Char. 0: 0 → 1
Char. 25: 1 → 0
Char. 36: 0 → 1
Char. 39: 0 → 1
Char. 51: 0 → 2
Char. 56: 0 → 1
Char. 70: 0 → 1
Char. 94: 0 → 1
Char. 106: 0 → 1
Char. 131: 1 → 0
Char. 166: 1 → 0
Char. 276: 0 → 1

**Millerettidae:**
Char. 24: 0 → 1
Char. 56: 0 → 1
Char. 57: 1 → 0
Char. 66: 01 → 2
Char. 84: 01 → 2
Char. 117: 0 → 1
Char. 124: 0 → 1
Char. 127: 0 → 1
Char. 135: 0 → 1
Char. 145: 0 → 1
Char. 202: 0 → 1
Char. 211: 0 → 1
Char. 230: 0 → 1
Char. 234: 0 → 1
Char. 248: 0 → 1
Char. 252: 0 → 1

***Nycteroleter ineptus*:**
Char. 278: 0 → 3

***Nyctiphruretus acudens*:**
Char. 21: 0 → 1
Char. 33: 1 → 2
Char. 41: 0 → 1
Char. 81: 1 → 0
Char. 84: 1 → 2
Char. 94: 0 → 1
Char. 166: 1 → 0
Char. 167: 0 → 1
Char. 215: 1 → 0
Char. 224: 0 → 1
Char. 266: 0 → 1
Char. 272: 2 → 1
Char. 276: 0 → 1

***Orovenator mayorum*:**
Char. 8: 0 → 1
Char. 24: 0 → 1
Char. 36: 0 → 1
Char. 48: 1 → 0
Char. 160: 0 → 1
Char. 165: 0 → 1

***Owenetta* spp.:**
Char. 169: 1 → 0

***Paleothyris acadiana*:**
Char. 38: 1 → 0
Char. 50: 1 → 0
Char. 66: 1 → 2
Char. 102: 0 → 1
Char. 146: 1 → 0
Char. 180: 0 → 1
Char. 237: 0 → 1
Char. 239: 0 → 1

***Placodus* spp.:**
Char. 0: 1 → 2
Char. 9: 0 → 1
Char. 12: 0 → 1
Char. 13: 0 → 1
Char. 19: 0 → 1
Char. 26: 0 → 1
Char. 31: 0 → 1
Char. 46: 1 → 0
Char. 57: 0 → 1
Char. 78: 1 → 0
Char. 93: 1 → 0
Char. 102: 1 → 2
Char. 109: 1 → 0
Char. 140: 1 → 0
Char. 163: 1 → 0
Char. 164: 0 → 1

***Procolophon* spp.:**
Char. 41: 0 → 1
Char. 69: 0 → 1
Char. 79: 0 → 1
Char. 83: 0 → 1
Char. 88: 0 → 1
Char. 117: 1 → 0
Char. 149: 1 → 0
Char. 237: 0 → 1
Char. 238: 0 → 1
Char. 272: 2 → 1

***Prolacerta broomi*:**
Char. 58: 1 → 0
Char. 66: 1 → 0
Char. 67: 1 → 0
Char. 80: 1 → 0
Char. 139: 1 → 0
Char. 147: 1 → 0
Char. 192: 1 → 0
Char. 203: 1 → 2

***Rhipaeosaurus* spp.:**
Char. 172: 0 → 1
Char. 277: 0 → 1

**Rhynchocephalia:**
Char. 0: 1 → 2
Char. 24: 0 → 1
Char. 77: 0 → 1
Char. 94: 1 → 0
Char. 139: 1 → 0
Char. 167: 1 → 0
Char. 205: 1 → 0

**Rhynchosauria:**
Char. 0: 1 → 0
Char. 7: 0 → 1
Char. 9: 0 → 1
Char. 26: 0 → 1
Char. 68: 0 → 1
Char. 99: 1 → 0
Char. 150: 1 → 0
Char. 160: 0 → 1
Char. 161: 0 → 1
Char. 171: 0 → 2
Char. 223: 0 → 1
Char. 224: 0 → 1
Char. 241: 0 → 1

***Scutosaurus* spp.:**
Char. 175: 0 → 1
Char. 218: 0 → 1
Char. 243: 0 → 2
Char. 244: 0 → 1
Char. 251: 0 → 1

***Sinosaurosphargis yunguiensis*:**
Char. 8: 0 → 1
Char. 30: 0 → 1
Char. 53: 0 → 1
Char. 82: 0 → 1
Char. 89: 1 → 0
Char. 127: 1 → 0
Char. 150: 1 → 0
Char. 154: 0 → 2
Char. 167: 1 → 0
Char. 255: 0 → 1

**Squamata:**
Char. 26: 0 → 1
Char. 45: 0 → 1
Char. 79: 0 → 2
Char. 80: 1 → 0
Char. 82: 0 → 1
Char. 92: 1 → 0
Char. 109: 1 → 0
Char. 160: 0 → 1
Char. 245: 0 → 1

***Trilophosaurus buettneri*:**
Char. 5: 1 → 0
Char. 11: 0 → 1
Char. 55: 1 → 0
Char. 93: 1 → 0
Char. 104: 0 → 1
Char. 106: 0 → 1
Char. 113: 0 → 1
Char. 122: 0 → 1
Char. 136: 1 → 0
Char. 144: 1 → 0
Char. 154: 0 → 1
Char. 157: 0 → 1
Char. 159: 1 → 0
Char. 177: 0 → 12
Char. 194: 0 → 1
Char. 203: 1 → 2
Char. 207: 1 → 0
Char. 208: 1 → 0
Char. 272: 1 → 0

***Youngina capensis*:**
Char. 5: 1 → 0
Char. 56: 0 → 1
Char. 75: 0 → 1
Char. 163: 1 → 0
Char. 170: 0 → 1
Char. 211: 0 → 1
Char. 231: 0 → 1
Char. 239: 0 → 1

**Node 49:**
Char. 46: 1 → 0
Char. 88: 1 → 0
Char. 89: 1 → 0
Char. 93: 1 → 0
Char. 176: 0 → 1
Char. 195: 0 → 2
Char. 198: 0 → 1
Char. 246: 1 → 2
Char. 259: 0 → 1
Char. 265: 1 → 0
Char. 270: 0 → 1

**Node 50:**
Char. 65: 0 → 1
Char. 184: 0 → 1
Char. 205: 0 → 1
Char. 219: 1 → 0
Char. 241: 0 → 1
Char. 246: 0 → 1
Char. 254: 0 → 1
Char. 255: 0 → 1
Char. 256: 0 → 1
Char. 268: 0 → 1
Char. 269: 0 → 1

**Node 51:**
Char. 0: 1 → 2
Char. 33: 1 → 2
Char. 62: 1 → 0
Char. 84: 1 → 2
Char. 152: 0 → 1
Char. 154: 0 → 1
Char. 161: 0 → 1
Char. 174: 0 → 1
Char. 251: 0 → 1
Char. 252: 0 → 1

**Node 52:**
Char. 27: 1 → 0
Char. 81: 1 → 0
Char. 84: 0 → 1
Char. 147: 0 → 1
Char. 206: 0 → 2
Char. 208: 0 → 1
Char. 231: 0 → 1

**Node 53:**
Char. 23: 0 → 1
Char. 73: 0 → 1
Char. 94: 0 → 1
Char. 131: 0 → 1
Char. 148: 0 → 1
Char. 224: 1 → 0
Char. 272: 2 → 1

**Node 54:**
Char. 33: 0 → 1
Char. 135: 0 → 1
Char. 267: 0 → 1
Char. 278: 1 → 0

**Node 55:**
Char. 62: 0 → 1

**Node 56:**
Char. 0: 0 → 1
Char. 27: 0 → 1
Char. 29: 1 → 0
Char. 40: 2 → 0
Char. 57: 1 → 0

Char. 59: 0 → 1
Char. 60: 0 → 1
Char. 67: 0 → 1
Char. 72: 1 → 2
Char. 84: 1 → 0
Char. 89: 0 → 1
Char. 111: 0 → 1
Char. 112: 0 → 1
Char. 120: 0 → 1
Char. 131: 1 → 0
Char. 147: 1 → 0
Char. 154: 1 → 0
Char. 193: 0 → 1
Char. 224: 0 → 1
Char. 234: 0 → 1
Char. 237: 0 → 1
Char. 266: 0 → 1

**Node 57:**
Char. 5: 0 → 1
Char. 44: 0 → 1
Char. 48: 0 → 1
Char. 88: 0 → 1
Char. 96: 0 → 1
Char. 121: 1 → 0
Char. 131: 0 → 1
Char. 147: 0 → 1
Char. 159: 0 → 1
Char. 163: 0 → 1
Char. 166: 0 → 1
Char. 169: 0 → 1
Char. 192: 0 → 1
Char. 221: 0 → 1

**Node 58:**
Char. 40: 0 → 2
Char. 74: 0 → 1
Char. 132: 0 → 1
Char. 149: 0 → 1
Char. 156: 0 → 1
Char. 201: 0 → 1
Char. 203: 0 → 1
Char. 235: 0 → 1

**Node 59:**
Char. 72: 0 → 1
Char. 79: 1 → 0
Char. 81: 0 → 1
Char. 97: 0 → 1
Char. 104: 1 → 0
Char. 144: 0 → 1
Char. 173: 0 → 1
Char. 183: 0 → 1

**Node 61:**
Char. 19: 0 → 1
Char. 92: 1 → 0

**Node 62:**
Char. 4: 0 → 1
Char. 29: 0 → 1
Char. 213: 0 → 1
Char. 226: 0 → 2
Char. 228: 0 → 1
Char. 275: 0 → 1

**Node 63:**
Char. 58: 0 → 1
Char. 61: 0 → 1
Char. 66: 0 → 1
Char. 150: 0 → 1
Char. 167: 0 → 1
Char. 176: 0 → 1
Char. 190: 0 → 1
Char. 205: 0 → 1
Char. 239: 0 → 1
Char. 260: 2 → 1

**Node 64:**
Char. 24: 0 → 1
Char. 57: 1 → 0
Char. 79: 0 → 1
Char. 83: 0 → 1
Char. 132: 1 → 0

**Node 65:**
Char. 73: 0 → 1
Char. 131: 1 → 0
Char. 205: 0 → 1
Char. 276: 0 → 1

**Node 66:**
Char. 18: 0 → 1
Char. 37: 0 → 1
Char. 100: 1 → 0
Char. 107: 0 → 1
Char. 113: 1 → 0
Char. 118: 1 → 0
Char. 125: 1 → 0
Char. 150: 0 → 1

**Node 67:**
Char. 23: 0 → 1
Char. 71: 1 → 0
Char. 102: 0 → 1
Char. 103: 0 → 1
Char. 106: 0 → 1
Char. 167: 0 → 1
Char. 214: 0 → 1
Char. 216: 1 → 0
Char. 235: 0 → 2
Char. 241: 0 → 1

**Node 68:**
Char. 113: 0 → 1
Char. 132: 1 → 0
Char. 138: 0 → 1
Char. 141: 0 → 1
Char. 207: 0 → 1

**Node 69:**
Char. 33: 0 → 1
Char. 44: 1 → 0
Char. 70: 0 → 1
Char. 80: 1 → 0
Char. 117: 0 → 1
Char. 118: 0 → 1
Char. 125: 0 → 1
Char. 194: 0 → 1
Char. 201: 1 → 0
Char. 211: 0 → 1

**Node 70:**
Char. 38: 1 → 2
Char. 39: 0 → 1
Char. 49: 0 → 1
Char. 66: 0 → 12
Char. 88: 1 → 0
Char. 95: 0 → 1
Char. 112: 0 → 1
Char. 148: 0 → 1
Char. 158: 0 → 1
Char. 183: 1 → 2
Char. 215: 0 → 1
Char. 239: 0 → 1

**Node 71:**
Char. 87: 0 → 1

**Node 72:**
Char. 25: 1 → 0
Char. 76: 1 → 0

**Node 73:**
Char. 79: 0 → 1
Char. 93: 1 → 0
Char. 133: 0 → 1

**Node 74:**
Char. 50: 1 → 0
Char. 58: 0 → 1
Char. 59: 0 → 1
Char. 60: 0 → 1
Char. 72: 1 → 2
Char. 104: 0 → 1
Char. 105: 0 → 1
Char. 106: 0 → 2
Char. 107: 0 → 1
Char. 109: 0 → 1
Char. 146: 1 → 0
Char. 155: 0 → 2

**Node 75:**
Char. 33: 1 → 0
Char. 38: 2 → 1
Char. 39: 1 → 0
Char. 42: 0 → 1
Char. 43: 0 → 1
Char. 46: 1 → 0
Char. 48: 1 → 0
Char. 49: 1 → 0
Char. 52: 0 → 1
Char. 83: 0 → 2
Char. 84: 1 → 0
Char. 87: 0 → 1
Char. 93: 1 → 0
Char. 132: 0 → 1
Char. 161: 0 → 1
Char. 163: 1 → 0
Char. 172: 0 → 2
Char. 174: 0 → 1
Char. 188: 0 → 1
Char. 189: 0 → 1
Char. 195: 0 → 1
Char. 212: 0 → 1
Char. 221: 1 → 0
Char. 236: 0 → 1
Char. 238: 0 → 2
Char. 242: 0 → 1
Char. 274: 0 → 1
Char. 275: 1 → 0

**Node 76:**
Char. 59: 0 → 1
Char. 60: 0 → 1
Char. 67: 0 → 1
Char. 72: 1 → 2
Char. 111: 0 → 1
Char. 123: 1 → 0
Char. 129: 0 → 1
Char. 170: 0 → 1
Char. 197: 0 → 1

**Node 77:**
Char. 47: 0 → 1
Char. 83: 0 → 1
Char. 85: 1 → 0
Char. 107: 0 → 1
Char. 169: 1 → 0
Char. 170: 0 → 1

**Node 78:**
Char. 2: 0 → 1
Char. 6: 0 → 1
Char. 98: 1 → 0
Char. 101: 0 → 1
Char. 113: 0 → 1
Char. 186: 0 → 1
Char. 193: 1 → 0
Char. 198: 0 → 1
Char. 220: 0 → 1
Char. 231: 1 → 0
Char. 234: 1 → 0
Char. 239: 1 → 0
Char. 240: 1 → 0
Char. 266: 1 → 0

**Node 79:**
Char. 33: 1 → 2
Char. 68: 0 → 1
Char. 103: 0 → 1
Char. 223: 0 → 1
Char. 229: 0 → 1
Char. 235: 1 → 2

**Node 80:**
Char. 17: 0 → 1
Char. 42: 0 → 1
Char. 251: 0 → 1
Char. 264: 0 → 1

**Node 81:**
Char. 41: 0 → 1
Char. 61: 1 → 2
Char. 112: 1 → 0
Char. 128: 0 → 1
Char. 138: 0 → 1
Char. 146: 1 → 0
Char. 192: 1 → 2
Char. 206: 2 → 0
Char. 224: 0 → 1
Char. 226: 0 → 1
Char. 227: 0 → 1
Char. 233: 0 → 1

**Node 82:**
Char. 61: 1 → 3
Char. 90: 0 → 1
Char. 91: 0 → 1
Char. 109: 1 → 0
Char. 209: 1 → 0

**ANALYSIS 84**
**(NO *CANDELARIA BARBOURI*, IMPLIED WEIGHTING, K = 2.75, NO CHARACTERS 247 AND 253)**

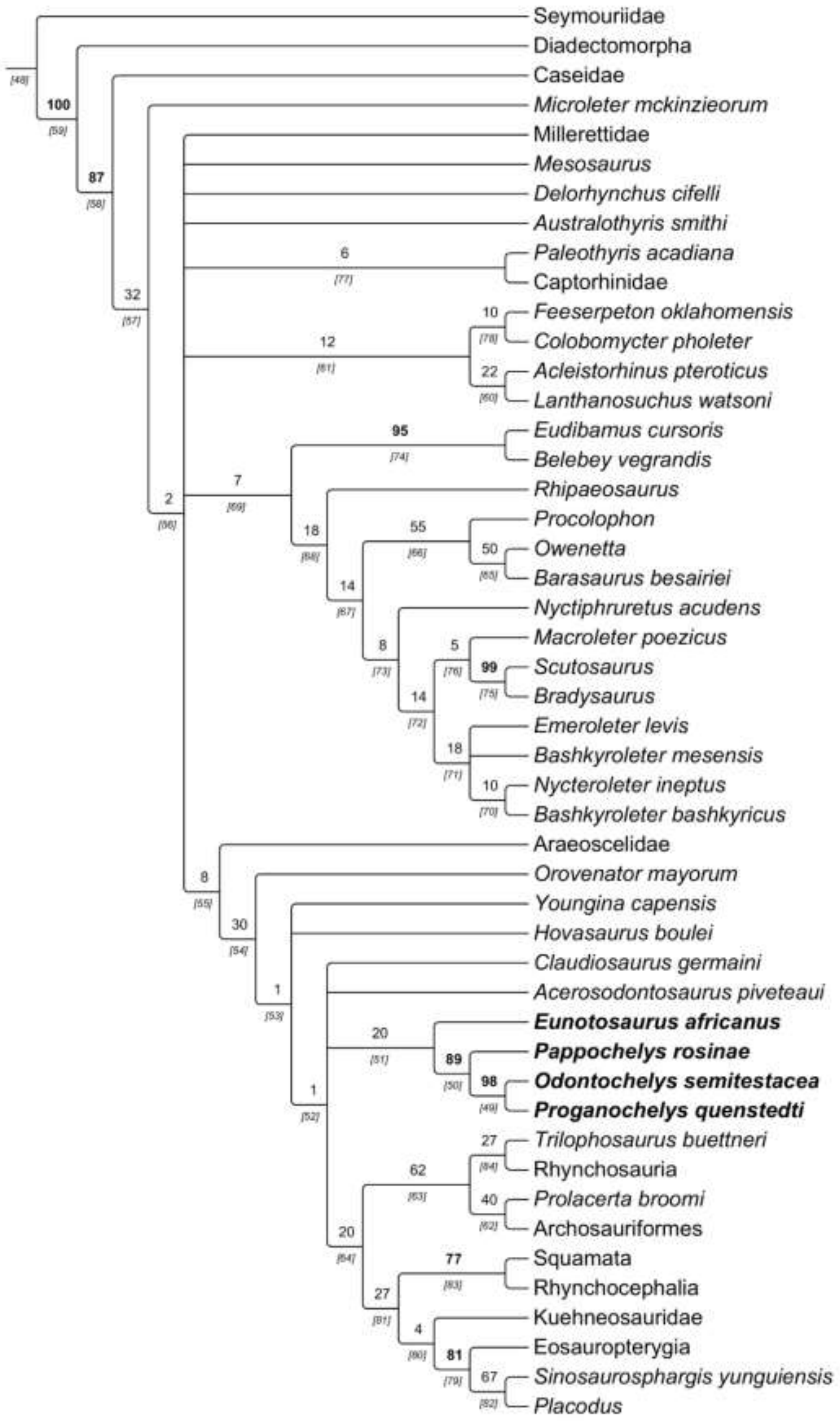

***Proganochelys quenstedti*:**
Char. 8: 0 → 1
Char. 11: 0 → 1
Char. 106: 0 → 1
Char. 108: 1 → 0
Char. 128: 0 → 1
Char. 175: 0 → 1
Char. 202: 1 → 0
Char. 207: 1 → 0
Char. 209: 1 → 0
Char. 244: 0 → 1
Char. 248: 0 → 1
Char. 250: 0 → 1
Char. 252: 1 → 0
Char. 262: 0 → 1

***Pappochelys rosinae*:**
Char. 0: 2 → 0
Char. 1: 0 → 1
Char. 5: 1 → 0
Char. 12: 0 → 1
Char. 41: 0 → 1
Char. 49: 0 → 1
Char. 75: 0 → 1
Char. 81: 0 → 1
Char. 169: 1 → 0
Char. 260: 2 → 0

***Eunotosaurus africanus*:**
Char. 19: 0 → 1
Char. 59: 1 → 0
Char. 60: 1 → 0
Char. 72: 2 → 1
Char. 76: 0 → 1
Char. 97: 1 → 0
Char. 103: 0 → 1
Char. 131: 1 → 0
Char. 153: 1 → 0
Char. 191: 1 → 0
Char. 192: 1 → 0
Char. 202: 1 → 0
Char. 211: 0 → 1
Char. 222: 1 → 0
Char. 237: 1 → 0
Char. 248: 0 → 1
Char. 249: 0 → 2
Char. 250: 0 → 1
Char. 263: 0 → 1
Char. 267: 1 → 0
Char. 272: 1 → 2
Char. 273: 0 → 1
Char. 274: 0 → 1
Char. 275: 0 → 1
Char. 276: 0 → 1
Char. 277: 0 → 1
Char. 278: 0 → 3

***Acerosodontosaurus piveteaui*:**
Char. 78: 1 → 0
Char. 127: 1 → 0
Char. 128: 0 → 1
Char. 206: 0 → 2
Char. 267: 1 → 0
Char. 278: 0 → 1

***Acleistorhinus pteroticus*:**
Char. 21: 0 → 1
Char. 146: 1 → 0

**Araeoscelidae:**
Char. 23: 0 → 1
Char. 28: 0 → 1
Char. 106: 0 → 1
Char. 116: 1 → 0
Char. 117: 01 → 2
Char. 169: 1 → 0
Char. 170: 0 → 1
Char. 197: 0 → 1
Char. 239: 0 → 1

**Archosauriformes:**
Char. 32: 0 → 1
Char. 94: 1 → 0
Char. 112: 1 → 0
Char. 152: 0 → 1
Char. 154: 0 → 2
Char. 166: 1 → 0
Char. 171: 0 → 1
Char. 185: 0 → 1
Char. 201: 0 → 1
Char. 204: 0 → 1
Char. 218: 0 → 3
Char. 224: 0 → 1
Char. 242: 0 → 1

***Australothyris smithi*:**
Char. 23: 0 → 1
Char. 24: 0 → 1
Char. 34: 0 → 1
Char. 55: 1 → 0
Char. 57: 1 → 0
Char. 71: 1 → 0
Char. 79: 0 → 1
Char. 85: 1 → 0
Char. 98: 1 → 0
Char. 100: 0 → 1
Char. 103: 0 → 1
Char. 110: 0 → 1
Char. 112: 0 → 1
Char. 123: 1 → 0
Char. 129: 0 → 1
Char. 131: 0 → 1
Char. 132: 1 → 0
Char. 144: 1 → 0
Char. 147: 0 → 1
Char. 149: 1 → 0
Char. 150: 0 → 1

***Barasaurus besairiei*:**
Char. 33: 1 → 0

***Bashkyroleter bashkyricus*:**
Char. 275: 1 → 0

***Bashkyroleter mesensis*:**
Char. 12: 0 → 1
Char. 66: 1 → 0
Char. 169: 1 → 0

***Belebey vegrandis*:**
Char. 154: 1 → 0

***Bradysaurus* spp.:**
Char. 19: 0 → 1
Char. 25: 1 → 0
Char. 73: 0 → 1
Char. 79: 0 → 1
Char. 135: 1 → 0
Char. 249: 0 → 1

**Captorhinidae:**
Char. 3: 0 → 1
Char. 23: 0 → 1
Char. 25: 1 → 0
Char. 26: 0 → 1
Char. 73: 0 → 1
Char. 75: 0 → 1
Char. 108: 1 → 0
Char. 183: 1 → 0
Char. 201: 1 → 0
Char. 203: 1 → 2
Char. 216: 1 → 0

**Caseidae:**
Char. 38: 1 → 0
Char. 46: 1 → 0
Char. 50: 1 → 0
Char. 170: 0 → 1
Char. 194: 0 → 1
Char. 273: 0 → 1
Char. 274: 0 → 1
Char. 278: 3 → 2

***Claudiosaurus germaini*:**
Char. 21: 1 → 0
Char. 24: 0 → 1
Char. 34: 0 → 1
Char. 36: 0 → 1
Char. 43: 1 → 0
Char. 56: 0 → 1
Char. 81: 0 → 1
Char. 105: 0 → 1
Char. 106: 0 → 1
Char. 117: 01 → 2
Char. 127: 1 → 0
Char. 141: 1 → 0
Char. 144: 1 → 0
Char. 166: 1 → 0
Char. 187: 1 → 0
Char. 190: 0 → 1
Char. 199: 0 → 1
Char. 201: 0 → 1
Char. 204: 0 → 1
Char. 208: 1 → 0
Char. 222: 1 → 0
Char. 234: 1 → 0

***Colobomycter pholeter*:**
Char. 21: 0 → 1
Char. 25: 1 → 0
Char. 84: 1 → 0
Char. 154: 1 → 0
Char. 167: 0 → 1
Char. 267: 0 → 1

***Delorhynchus cifelli*:**
Char. 5: 0 → 1
Char. 18: 0 → 1
Char. 20: 0 → 1
Char. 21: 0 → 1
Char. 24: 0 → 1
Char. 26: 0 → 1
Char. 28: 0 → 1
Char. 33: 0 → 2
Char. 39: 0 → 1
Char. 52: 0 → 1
Char. 100: 0 → 1
Char. 111: 0 → 1
Char. 116: 1 → 0
Char. 131: 0 → 1
Char. 147: 0 → 1
Char. 156: 1 → 0
Char. 159: 0 → 1
Char. 163: 0 → 1
Char. 167: 0 → 1
Char. 189: 0 → 1
Char. 191: 0 → 1
Char. 192: 0 → 1
Char. 204: 0 → 2
Char. 267: 0 → 1

**Diadectomorpha:**
Char. 0: 0 → 1
Char. 64: 0 → 1
Char. 70: 0 → 1
Char. 122: 0 → 1
Char. 123: 1 → 0
Char. 146: 1 → 0
Char. 275: 1 → 0
Char. 278: 3 → 0

***Emeroleter levis*:**
Char. 51: 1 → 0

**Eosauropterygia:**
Char. 166: 1 → 0
Char. 174: 0 → 1
Char. 178: 0 → 1
Char. 194: 0 → 2
Char. 272: 1 → 0

***Eudibamus cursoris*:**
Char. 154: 1 → 2

***Feeserpeton oklahomensis*:**
Char. 51: 0 → 1
Char. 157: 0 → 1
Char. 158: 0 → 1

***Hovasaurus boulei*:**
Char. 41: 0 → 1
Char. 55: 1 → 0
Char. 60: 1 → 0
Char. 72: 2 → 1
Char. 77: 0 → 2
Char. 78: 1 → 0
Char. 79: 0 → 1
Char. 93: 1 → 0
Char. 113: 0 → 1
Char. 138: 0 → 1
Char. 141: 1 → 0
Char. 146: 1 → 0
Char. 154: 0 → 1
Char. 204: 0 → 2
Char. 206: 0 → 2
Char. 278: 0 → 3

**Kuehneosauridae:**
Char. 7: 0 → 1
Char. 21: 1 → 0
Char. 24: 0 → 1
Char. 27: 0 → 1
Char. 43: 1 → 0
Char. 79: 0 → 2
Char. 107: 1 → 0
Char. 140: 1 → 0
Char. 147: 1 → 0
Char. 148: 1 → 0
Char. 245: 0 → 1
Char. 272: 1 → 2
Char. 278: 0 → 3

***Lanthanosuchus watsoni*:**
Char. 25: 1 → 0
Char. 51: 0 → 1
Char. 76: 0 → 1
Char. 86: 0 → 1
Char. 98: 1 → 0
Char. 138: 0 → 1
Char. 144: 1 → 0
Char. 154: 1 → 2

***Macroleter poezicus*:**
Char. 9: 0 → 1
Char. 26: 0 → 1
Char. 66: 1 → 2
Char. 126: 0 → 1
Char. 134: 0 → 2
Char. 140: 0 → 1
Char. 146: 1 → 0
Char. 169: 1 → 0

***Mesosaurus* spp.:**
Char. 0: 0 → 1
Char. 2: 0 → 1
Char. 6: 0 → 1
Char. 8: 0 → 1
Char. 9: 0 → 1
Char. 13: 0 → 1
Char. 19: 0 → 1
Char. 23: 0 → 1
Char. 26: 0 → 1

Char. 29: 1 → 0
Char. 33: 0 → 1
Char. 38: 1 → 0
Char. 41: 0 → 1
Char. 50: 1 → 0
Char. 67: 0 → 1
Char. 76: 0 → 1
Char. 84: 1 → 0
Char. 85: 1 → 0
Char. 94: 0 → 1
Char. 107: 0 → 1
Char. 109: 0 → 1
Char. 111: 0 → 1
Char. 115: 0 → 1
Char. 146: 1 → 0
Char. 148: 0 → 1
Char. 149: 1 → 0
Char. 164: 0 → 1
Char. 167: 0 → 1
Char. 176: 0 → 1
Char. 183: 1 → 0
Char. 184: 0 → 1
Char. 199: 0 → 1
Char. 202: 0 → 1
Char. 204: 0 → 2
Char. 206: 0 → 1
Char. 207: 0 → 1
Char. 209: 0 → 1
Char. 217: 0 → 1
Char. 219: 0 → 1
Char. 220: 0 → 1
Char. 231: 0 → 1
Char. 260: 2 → 0
Char. 272: 2 → 0
Char. 278: 3 → 0

***Microleter mckinzieorum*:**
Char. 0: 0 → 1
Char. 18: 0 → 1
Char. 39: 0 → 1
Char. 57: 1 → 0
Char. 70: 0 → 1
Char. 76: 0 → 1
Char. 94: 0 → 1
Char. 106: 0 → 1
Char. 110: 0 → 1
Char. 159: 0 → 1
Char. 278: 3 → 1

**Millerettidae:**
Char. 24: 0 → 1
Char. 25: 1 → 0
Char. 44: 1 → 0
Char. 48: 1 → 0
Char. 56: 0 → 1
Char. 57: 1 → 0
Char. 66: 0 → 2
Char. 78: 1 → 0
Char. 80: 1 → 0
Char. 84: 1 → 2
Char. 88: 1 → 0
Char. 96: 1 → 0
Char. 121: 0 → 1
Char. 124: 0 → 1
Char. 127: 0 → 1
Char. 135: 0 → 1
Char. 145: 0 → 1
Char. 202: 0 → 1
Char. 211: 0 → 1
Char. 230: 0 → 1
Char. 234: 0 → 1
Char. 248: 0 → 1
Char. 252: 0 → 1

***Nycteroleter ineptus*:**
Char. 66: 1 → 0
Char. 278: 0 → 3

***Nyctiphruretus acudens*:**
Char. 21: 0 → 1
Char. 41: 0 → 1
Char. 48: 1 → 0
Char. 66: 1 → 2
Char. 81: 1 → 0
Char. 84: 1 → 2
Char. 94: 0 → 1
Char. 166: 1 → 0
Char. 215: 1 → 0
Char. 224: 0 → 1
Char. 226: 1 → 0
Char. 266: 0 → 1
Char. 272: 2 → 1
Char. 276: 0 → 1

***Orovenator mayorum*:**
Char. 8: 0 → 1
Char. 24: 0 → 1
Char. 36: 0 → 1
Char. 48: 1 → 0
Char. 160: 0 → 1
Char. 165: 0 → 1

***Owenetta* spp.:**
Char. 142: 1 → 0
Char. 169: 1 → 0

***Paleothyris acadiana*:**
Char. 38: 1 → 0
Char. 50: 1 → 0
Char. 66: 1 → 2
Char. 102: 0 → 1
Char. 146: 1 → 0
Char. 237: 0 → 1
Char. 239: 0 → 1

***Placodus* spp.:**
Char. 0: 1 → 2
Char. 9: 0 → 1
Char. 12: 0 → 1
Char. 13: 0 → 1
Char. 19: 0 → 1
Char. 31: 0 → 1
Char. 46: 1 → 0
Char. 57: 0 → 1
Char. 78: 1 → 0
Char. 93: 1 → 0
Char. 102: 1 → 2
Char. 109: 1 → 0
Char. 140: 1 → 0
Char. 155: 0 → 1
Char. 163: 1 → 0
Char. 164: 0 → 1

***Procolophon* spp.:**
Char. 41: 0 → 1
Char. 69: 0 → 1
Char. 79: 0 → 1
Char. 86: 0 → 1
Char. 88: 0 → 1
Char. 101: 0 → 1
Char. 141: 0 → 1
Char. 149: 1 → 0
Char. 203: 1 → 2
Char. 204: 0 → 1
Char. 230: 0 → 1
Char. 237: 0 → 1
Char. 238: 0 → 1
Char. 239: 1 → 0
Char. 272: 2 → 1
Char. 278: 3 → 0

***Prolacerta broomi*:**
Char. 58: 1 → 0
Char. 66: 1 → 0
Char. 67: 1 → 0
Char. 80: 1 → 0
Char. 139: 1 → 0
Char. 147: 1 → 0
Char. 192: 1 → 0
Char. 206: 0 → 12

***Rhipaeosaurus* spp.:**
Char. 172: 0 → 1
Char. 277: 0 → 1

**Rhynchocephalia:**
Char. 0: 1 → 2
Char. 23: 1 → 0
Char. 24: 0 → 1
Char. 77: 0 → 1
Char. 81: 0 → 1
Char. 88: 1 → 0
Char. 94: 1 → 0
Char. 139: 1 → 0
Char. 167: 1 → 0
Char. 205: 1 → 0

**Rhynchosauria:**
Char. 0: 1 → 0
Char. 7: 0 → 1
Char. 9: 0 → 1
Char. 26: 0 → 1
Char. 68: 0 → 1
Char. 99: 1 → 0
Char. 150: 1 → 0
Char. 160: 0 → 1
Char. 161: 0 → 1
Char. 171: 0 → 2
Char. 223: 0 → 1
Char. 224: 0 → 1
Char. 241: 0 → 1

***Scutosaurus* spp.:**
Char. 175: 0 → 1
Char. 190: 0 → 1
Char. 218: 0 → 1
Char. 243: 0 → 2
Char. 244: 0 → 1
Char. 251: 0 → 1

***Sinosaurosphargis yunguiensis*:**
Char. 8: 0 → 1
Char. 30: 0 → 1
Char. 53: 0 → 1
Char. 89: 1 → 0
Char. 127: 1 → 0
Char. 150: 1 → 0
Char. 154: 0 → 2
Char. 167: 1 → 0
Char. 255: 0 → 1

**Squamata:**
Char. 45: 0 → 1
Char. 79: 0 → 2
Char. 80: 1 → 0
Char. 92: 1 → 0
Char. 109: 1 → 0
Char. 160: 0 → 1
Char. 200: 0 → 1
Char. 245: 0 → 1

***Trilophosaurus buettneri*:**
Char. 5: 1 → 0
Char. 11: 0 → 1
Char. 55: 1 → 0
Char. 93: 1 → 0
Char. 104: 0 → 1
Char. 106: 0 → 1
Char. 113: 0 → 1
Char. 122: 0 → 1
Char. 136: 1 → 0
Char. 144: 1 → 0
Char. 154: 0 → 1
Char. 157: 0 → 1
Char. 159: 1 → 0
Char. 177: 0 → 12
Char. 194: 0 → 1
Char. 207: 1 → 0
Char. 208: 1 → 0
Char. 272: 1 → 0

***Youngina capensis*:**
Char. 56: 0 → 1
Char. 75: 0 → 1
Char. 170: 0 → 1
Char. 211: 0 → 1
Char. 239: 0 → 1

**Node 49:**
Char. 46: 1 → 0
Char. 88: 1 → 0
Char. 89: 1 → 0
Char. 93: 1 → 0
Char. 176: 0 → 1
Char. 195: 0 → 2
Char. 198: 0 → 1
Char. 246: 1 → 2
Char. 259: 0 → 1
Char. 265: 1 → 0
Char. 270: 0 → 1

**Node 50:**
Char. 65: 0 → 1
Char. 184: 0 → 1
Char. 205: 0 → 1
Char. 210: 0 → 1
Char. 219: 1 → 0
Char. 241: 0 → 1
Char. 246: 0 → 1
Char. 254: 0 → 1
Char. 255: 0 → 1
Char. 256: 0 → 1
Char. 268: 0 → 1
Char. 269: 0 → 1

**Node 51:**
Char. 0: 1 → 2
Char. 33: 1 → 2
Char. 62: 1 → 0
Char. 84: 01 → 2
Char. 152: 0 → 1
Char. 154: 0 → 1
Char. 158: 0 → 1
Char. 161: 0 → 1
Char. 174: 0 → 1
Char. 181: 0 → 1
Char. 204: 0 → 2
Char. 251: 0 → 1
Char. 252: 0 → 1

**Node 52:**
Char. 23: 0 → 1
Char. 73: 0 → 1
Char. 81: 1 → 0
Char. 94: 0 → 1
Char. 131: 0 → 1
Char. 145: 0 → 1
Char. 148: 0 → 1
Char. 151: 0 → 1
Char. 208: 0 → 1
Char. 217: 0 → 1
Char. 224: 1 → 0
Char. 272: 2 → 1

**Node 53:**
Char. 21: 0 → 1
Char. 33: 0 → 1
Char. 135: 0 → 1
Char. 159: 0 → 1
Char. 267: 0 → 1
Char. 278: 1 → 0

**Node 54:**
Char. 20: 0 → 1

Char. 62: 0 → 1
Char. 141: 0 → 1

**Node 55:**
Char. 0: 0 → 1
Char. 29: 1 → 0
Char. 40: 2 → 0
Char. 57: 1 → 0
Char. 59: 0 → 1
Char. 60: 0 → 1
Char. 67: 0 → 1
Char. 72: 1 → 2
Char. 89: 0 → 1
Char. 111: 0 → 1
Char. 112: 0 → 1
Char. 120: 0 → 1
Char. 154: 1 → 0
Char. 192: 0 → 1
Char. 193: 0 → 1
Char. 222: 0 → 1
Char. 224: 0 → 1
Char. 234: 0 → 1
Char. 237: 0 → 1
Char. 266: 0 → 1
Char. 278: 3 → 1

**Node 56:**
Char. 48: 0 → 1
Char. 132: 0 → 1
Char. 276: 1 → 0

**Node 57:**
Char. 29: 0 → 1
Char. 84: 0 → 1
Char. 88: 0 → 1
Char. 169: 0 → 1

**Node 58:**
Char. 72: 0 → 1
Char. 81: 0 → 1
Char. 93: 0 → 1
Char. 97: 0 → 1
Char. 104: 1 → 0
Char. 144: 0 → 1
Char. 173: 0 → 1
Char. 183: 0 → 1

**Node 60:**
Char. 95: 0 → 1
Char. 113: 0 → 1
Char. 114: 0 → 1

**Node 61:**
Char. 5: 0 → 1
Char. 20: 0 → 1
Char. 47: 0 → 1
Char. 79: 0 → 1
Char. 110: 0 → 1
Char. 131: 0 → 1
Char. 137: 0 → 1
Char. 140: 0 → 2
Char. 147: 0 → 1
Char. 159: 0 → 1
Char. 163: 0 → 1

**Node 62:**
Char. 19: 0 → 1
Char. 21: 1 → 0
Char. 92: 1 → 0

**Node 63:**
Char. 4: 0 → 1
Char. 29: 0 → 1
Char. 213: 0 → 1
Char. 226: 0 → 2
Char. 228: 0 → 1
Char. 275: 0 → 1

**Node 64:**
Char. 58: 0 → 1
Char. 61: 0 → 1
Char. 66: 0 → 1
Char. 69: 0 → 1
Char. 107: 0 → 1
Char. 140: 0 → 1
Char. 150: 0 → 1
Char. 167: 0 → 1
Char. 176: 0 → 1
Char. 190: 0 → 1
Char. 205: 0 → 1
Char. 230: 0 → 1
Char. 239: 0 → 1
Char. 260: 2 → 1

**Node 65:**
Char. 73: 0 → 1
Char. 205: 0 → 1
Char. 273: 0 → 1
Char. 276: 0 → 1

**Node 66:**
Char. 18: 0 → 1
Char. 25: 1 → 0
Char. 37: 0 → 1
Char. 106: 0 → 1
Char. 107: 0 → 1
Char. 110: 0 → 1
Char. 126: 0 → 1
Char. 150: 0 → 1

**Node 67:**
Char. 186: 0 → 1

**Node 68:**
Char. 194: 0 → 1
Char. 201: 1 → 0
Char. 211: 0 → 1
Char. 226: 0 → 1

**Node 69:**
Char. 5: 0 → 1
Char. 38: 1 → 2
Char. 39: 0 → 1
Char. 49: 0 → 1
Char. 66: 0 → 1
Char. 76: 0 → 1
Char. 85: 1 → 0
Char. 88: 1 → 0
Char. 95: 0 → 1
Char. 112: 0 → 1
Char. 148: 0 → 1
Char. 158: 0 → 1
Char. 159: 0 → 1
Char. 183: 1 → 2
Char. 192: 0 → 1
Char. 215: 0 → 1
Char. 239: 0 → 1

**Node 70:**
Char. 87: 0 → 1
Char. 110: 0 → 1

**Node 71:**
Char. 79: 0 → 1
Char. 133: 0 → 1

**Node 72:**
Char. 100: 0 → 1
Char. 113: 0 → 1

**Node 73:**
Char. 70: 0 → 1
Char. 118: 0 → 1
Char. 125: 0 → 1

**Node 74:**
Char. 58: 0 → 1
Char. 59: 0 → 1
Char. 60: 0 → 1
Char. 72: 1 → 2
Char. 104: 0 → 1
Char. 105: 0 → 1
Char. 106: 0 → 2
Char. 107: 0 → 1
Char. 109: 0 → 1
Char. 110: 0 → 1
Char. 146: 1 → 0

**Node 75:**
Char. 23: 0 → 1
Char. 33: 1 → 0
Char. 38: 2 → 1
Char. 39: 1 → 0
Char. 42: 0 → 1
Char. 43: 0 → 1
Char. 46: 1 → 0
Char. 48: 1 → 0
Char. 49: 1 → 0
Char. 71: 1 → 0
Char. 83: 0 → 2
Char. 101: 0 → 1
Char. 103: 0 → 1
Char. 106: 0 → 1
Char. 161: 0 → 1
Char. 163: 1 → 0
Char. 172: 0 → 2
Char. 174: 0 → 1
Char. 188: 0 → 1
Char. 203: 1 → 2
Char. 212: 0 → 1
Char. 216: 1 → 0
Char. 236: 0 → 1
Char. 238: 0 → 2
Char. 239: 1 → 0
Char. 241: 0 → 1
Char. 242: 0 → 1
Char. 273: 0 → 1
Char. 274: 0 → 1
Char. 275: 1 → 0

**Node 76:**
Char. 52: 0 → 1
Char. 75: 0 → 1
Char. 84: 1 → 0
Char. 87: 0 → 1
Char. 110: 0 → 1
Char. 121: 0 → 1
Char. 230: 0 → 1

**Node 77:**
Char. 29: 1 → 0
Char. 44: 1 → 0
Char. 48: 1 → 0
Char. 59: 0 → 1
Char. 60: 0 → 1
Char. 66: 0 → 1
Char. 67: 0 → 1
Char. 72: 1 → 2
Char. 78: 1 → 0
Char. 84: 1 → 0
Char. 88: 1 → 0
Char. 93: 1 → 0
Char. 111: 0 → 1
Char. 123: 1 → 0
Char. 129: 0 → 1
Char. 154: 1 → 0
Char. 169: 1 → 0
Char. 170: 0 → 1
Char. 197: 0 → 1

**Node 78:**
Char. 85: 1 → 0
Char. 107: 0 → 1

**Node 79:**
Char. 2: 0 → 1
Char. 6: 0 → 1
Char. 81: 0 → 1
Char. 186: 0 → 1
Char. 198: 0 → 1
Char. 200: 0 → 1
Char. 220: 0 → 1
Char. 239: 1 → 0
Char. 267: 1 → 0

**Node 80:**
Char. 98: 1 → 0
Char. 113: 0 → 1
Char. 181: 0 → 1
Char. 206: 0 → 2

**Node 81:**
Char. 35: 0 → 1
Char. 102: 0 → 1
Char. 103: 0 → 1
Char. 184: 0 → 1
Char. 201: 0 → 1
Char. 210: 0 → 1
Char. 223: 0 → 1
Char. 229: 0 → 1

**Node 82:**
Char. 17: 0 → 1
Char. 42: 0 → 1
Char. 251: 0 → 1
Char. 264: 0 → 1

**Node 83:**
Char. 41: 0 → 1
Char. 61: 1 → 2
Char. 112: 1 → 0
Char. 138: 0 → 1
Char. 146: 1 → 0
Char. 192: 1 → 2
Char. 224: 0 → 1
Char. 226: 0 → 1
Char. 227: 0 → 1
Char. 233: 0 → 1
Char. 267: 1 → 2

**Node 84:**
Char. 61: 1 → 3
Char. 90: 0 → 1
Char. 91: 0 → 1
Char. 109: 1 → 0
Char. 209: 1 → 0

**ANALYSIS 85**
**(NO *CANDELARIA BARBOURI*, NO CHARACTERS 247, 251, AND 253)**

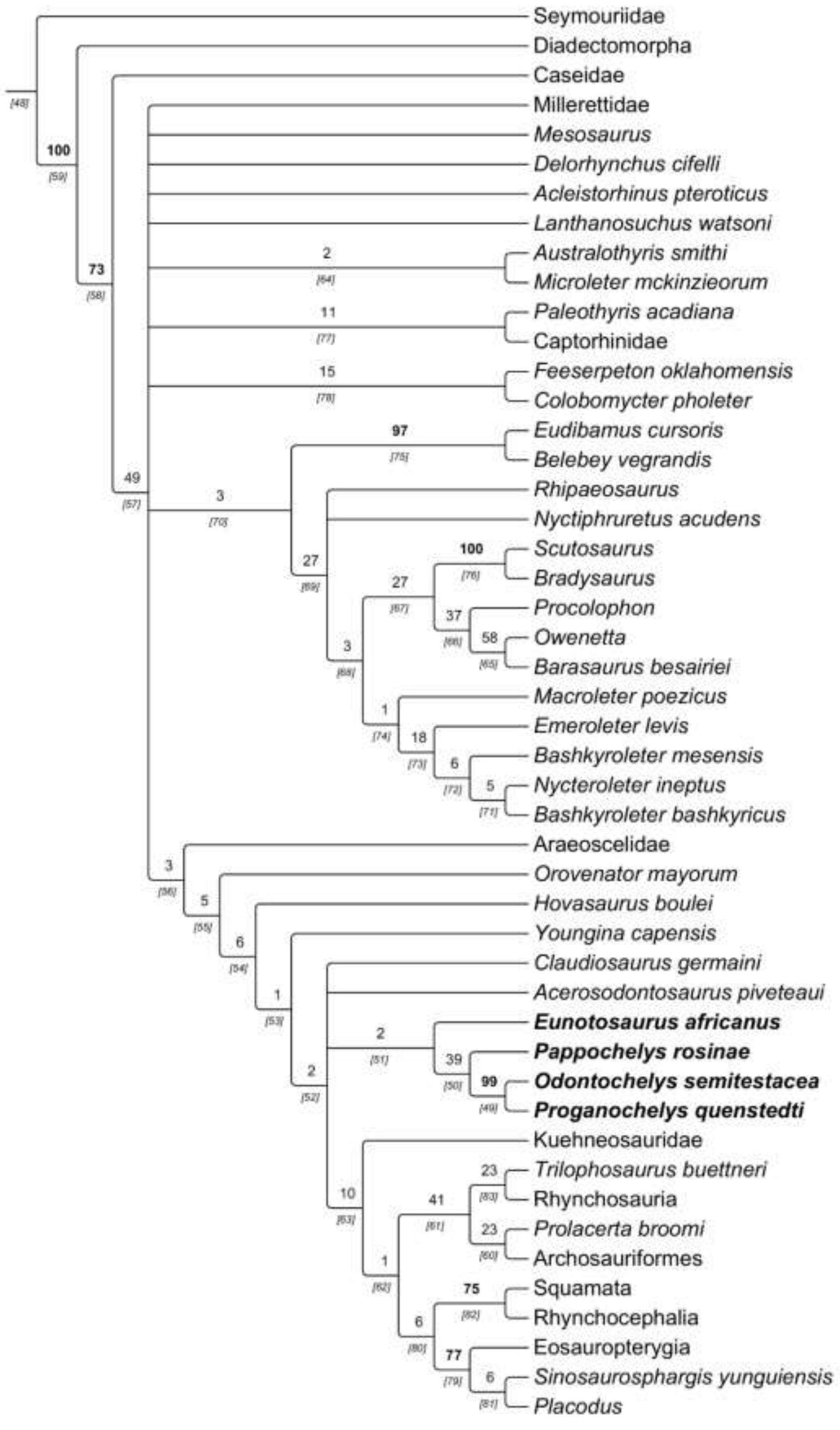

***Proganochelys quenstedti*:**
Char. 8: 0 → 1
Char. 11: 0 → 1
Char. 106: 0 → 1
Char. 108: 1 → 0
Char. 128: 0 → 1
Char. 175: 0 → 1
Char. 202: 1 → 0
Char. 207: 1 → 0
Char. 209: 1 → 0
Char. 244: 0 → 1
Char. 248: 0 → 1
Char. 250: 0 → 1
Char. 252: 1 → 0
Char. 262: 0 → 1

***Pappochelys rosinae*:**
Char. 0: 2 → 0
Char. 1: 0 → 1
Char. 5: 1 → 0
Char. 12: 0 → 1
Char. 41: 0 → 1
Char. 49: 0 → 1
Char. 75: 0 → 1
Char. 81: 0 → 1
Char. 169: 1 → 0
Char. 260: 2 → 0

***Eunotosaurus africanus*:**
Char. 19: 0 → 1
Char. 59: 1 → 0
Char. 60: 1 → 0
Char. 72: 2 → 1
Char. 76: 0 → 1
Char. 97: 1 → 0
Char. 103: 0 → 1
Char. 131: 1 → 0
Char. 153: 1 → 0
Char. 191: 1 → 0
Char. 192: 1 → 0
Char. 202: 1 → 0
Char. 211: 0 → 1
Char. 222: 1 → 0
Char. 237: 1 → 0
Char. 248: 0 → 1
Char. 249: 0 → 2
Char. 250: 0 → 1
Char. 263: 0 → 1
Char. 267: 1 → 0
Char. 273: 0 → 1
Char. 274: 0 → 1
Char. 275: 0 → 1
Char. 276: 0 → 1
Char. 277: 0 → 1
Char. 278: 0 → 3

***Acerosodontosaurus piveteaui*:**
Char. 78: 1 → 0
Char. 127: 1 → 0
Char. 128: 0 → 1
Char. 155: 0 → 1
Char. 267: 1 → 0
Char. 278: 0 → 1

***Acleistorhinus pteroticus:***
Char. 20: 0 → 1
Char. 21: 0 → 1
Char. 30: 0 → 1
Char. 33: 0 → 1
Char. 47: 0 → 1
Char. 55: 1 → 0
Char. 56: 0 → 1
Char. 64: 0 → 1
Char. 70: 0 → 1
Char. 79: 0 → 1
Char. 95: 0 → 1
Char. 110: 0 → 1
Char. 113: 0 → 1
Char. 114: 0 → 1
Char. 131: 0 → 1
Char. 137: 0 → 1
Char. 140: 0 → 2
Char. 146: 1 → 0
Char. 159: 0 → 1
Char. 169: 1 → 0
Char. 170: 0 → 1
Char. 278: 3 → 1

**Araeoscelidae:**
Char. 5: 1 → 0
Char. 23: 0 → 1
Char. 28: 0 → 1
Char. 106: 0 → 1
Char. 116: 1 → 0
Char. 117: 01 → 2
Char. 169: 1 → 0
Char. 170: 0 → 1
Char. 197: 0 → 1
Char. 239: 0 → 1

**Archosauriformes:**
Char. 32: 0 → 1
Char. 94: 1 → 0
Char. 112: 1 → 0
Char. 152: 0 → 1
Char. 154: 0 → 2
Char. 166: 1 → 0
Char. 171: 0 → 1
Char. 185: 0 → 1
Char. 204: 0 → 1
Char. 218: 0 → 3
Char. 224: 0 → 1
Char. 242: 0 → 1

***Australothyris smithi*:**
Char. 23: 0 → 1
Char. 34: 0 → 1
Char. 55: 1 → 0
Char. 71: 1 → 0
Char. 85: 1 → 0
Char. 112: 0 → 1
Char. 129: 0 → 1
Char. 131: 0 → 1

***Barasaurus besairiei*:**
Char. 33: 1 → 0
Char. 75: 1 → 0
Char. 216: 0 → 1

***Bashkyroleter bashkyricus*:**
Char. 275: 1 → 0

***Bashkyroleter mesensis*:**
Char. 169: 1 → 0

***Belebey vegrandis*:**
Char. 154: 1 → 0

***Bradysaurus* spp.:**
Char. 19: 0 → 1
Char. 73: 0 → 1
Char. 79: 0 → 1
Char. 135: 1 → 0
Char. 249: 0 → 1

**Captorhinidae:**
Char. 3: 0 → 1
Char. 23: 0 → 1
Char. 25: 1 → 0
Char. 26: 0 → 1
Char. 73: 0 → 1
Char. 75: 0 → 1
Char. 83: 0 → 1
Char. 108: 1 → 0
Char. 183: 1 → 0
Char. 201: 1 → 0
Char. 203: 1 → 2
Char. 216: 1 → 0

**Caseidae:**
Char. 24: 0 → 1
Char. 25: 1 → 0
Char. 36: 0 → 1
Char. 38: 1 → 0
Char. 46: 1 → 0
Char. 50: 1 → 0
Char. 56: 0 → 1
Char. 170: 0 → 1
Char. 194: 0 → 1
Char. 273: 0 → 1
Char. 274: 0 → 1
Char. 278: 3 → 2

***Claudiosaurus germaini*:**
Char. 34: 0 → 1
Char. 36: 0 → 1
Char. 81: 0 → 1
Char. 105: 0 → 1
Char. 106: 0 → 1
Char. 117: 01 → 2
Char. 127: 1 → 0
Char. 141: 1 → 0
Char. 144: 1 → 0
Char. 166: 1 → 0
Char. 187: 1 → 0
Char. 190: 0 → 1
Char. 199: 0 → 1
Char. 204: 0 → 1
Char. 208: 1 → 0
Char. 222: 1 → 0
Char. 231: 1 → 0
Char. 234: 1 → 0

***Colobomycter pholeter*:**
Char. 21: 0 → 1
Char. 25: 1 → 0
Char. 84: 1 → 0
Char. 154: 1 → 0
Char. 167: 0 → 1
Char. 267: 0 → 1

***Delorhynchus cifelli*:**
Char. 18: 0 → 1
Char. 20: 0 → 1
Char. 21: 0 → 1
Char. 24: 0 → 1
Char. 26: 0 → 1
Char. 28: 0 → 1
Char. 33: 0 → 2
Char. 39: 0 → 1
Char. 52: 0 → 1
Char. 100: 0 → 1
Char. 111: 0 → 1
Char. 116: 1 → 0
Char. 117: 0 → 1
Char. 131: 0 → 1
Char. 156: 1 → 0
Char. 159: 0 → 1
Char. 167: 0 → 1
Char. 180: 0 → 1
Char. 189: 0 → 1
Char. 191: 0 → 1
Char. 192: 0 → 1
Char. 204: 0 → 2
Char. 267: 0 → 1

**Diadectomorpha:**
Char. 0: 0 → 1
Char. 64: 0 → 1
Char. 70: 0 → 1
Char. 122: 0 → 1
Char. 123: 1 → 0
Char. 146: 1 → 0
Char. 275: 1 → 0
Char. 278: 3 → 0

***Emeroleter levis*:**
Char. 51: 1 → 0

**Eosauropterygia:**
Char. 159: 1 → 0
Char. 166: 1 → 0
Char. 174: 0 → 1
Char. 194: 0 → 2
Char. 272: 1 → 0

***Eudibamus cursoris*:**
Char. 154: 1 → 2

***Feeserpeton oklahomensis*:**
Char. 51: 0 → 1
Char. 70: 0 → 1
Char. 157: 0 → 1
Char. 158: 0 → 1

***Hovasaurus boulei*:**
Char. 41: 0 → 1
Char. 55: 1 → 0
Char. 72: 2 → 1
Char. 77: 0 → 2
Char. 78: 1 → 0
Char. 79: 0 → 1
Char. 93: 1 → 0
Char. 113: 0 → 1
Char. 138: 0 → 1
Char. 146: 1 → 0
Char. 204: 0 → 2

**Kuehneosauridae:**
Char. 7: 0 → 1
Char. 26: 0 → 1
Char. 34: 0 → 1
Char. 36: 0 → 1
Char. 79: 0 → 2
Char. 82: 0 → 1
Char. 98: 1 → 0
Char. 108: 1 → 0
Char. 113: 0 → 1
Char. 128: 0 → 1
Char. 148: 1 → 0
Char. 159: 1 → 0
Char. 181: 0 → 1
Char. 185: 0 → 1
Char. 245: 0 → 1
Char. 278: 0 → 3

***Lanthanosuchus watsoni*:**
Char. 25: 1 → 0
Char. 51: 0 → 1
Char. 76: 0 → 1
Char. 86: 0 → 1
Char. 95: 0 → 1
Char. 98: 1 → 0
Char. 110: 0 → 1
Char. 113: 0 → 1
Char. 114: 0 → 1
Char. 131: 0 → 1
Char. 137: 0 → 1
Char. 138: 0 → 1
Char. 140: 0 → 2
Char. 144: 1 → 0
Char. 154: 1 → 2
Char. 192: 0 → 1

***Macroleter poezicus*:**
Char. 52: 0 → 1
Char. 84: 1 → 0
Char. 87: 0 → 1
Char. 140: 0 → 1
Char. 146: 1 → 0
Char. 169: 1 → 0
Char. 235: 0 → 1

***Mesosaurus* spp.:**
Char. 0: 0 → 1
Char. 2: 0 → 1
Char. 5: 1 → 0
Char. 6: 0 → 1
Char. 8: 0 → 1
Char. 9: 0 → 1
Char. 13: 0 → 1
Char. 19: 0 → 1
Char. 23: 0 → 1
Char. 26: 0 → 1
Char. 29: 1 → 0
Char. 33: 0 → 1
Char. 38: 1 → 0
Char. 41: 0 → 1
Char. 50: 1 → 0
Char. 67: 0 → 1
Char. 76: 0 → 1
Char. 83: 0 → 1
Char. 84: 1 → 0
Char. 85: 1 → 0
Char. 94: 0 → 1
Char. 107: 0 → 1
Char. 109: 0 → 1
Char. 111: 0 → 1
Char. 115: 0 → 1
Char. 146: 1 → 0
Char. 148: 0 → 1
Char. 149: 1 → 0
Char. 164: 0 → 1
Char. 167: 0 → 1
Char. 176: 0 → 1
Char. 183: 1 → 0
Char. 184: 0 → 1
Char. 199: 0 → 1
Char. 202: 0 → 1
Char. 204: 0 → 2
Char. 206: 0 → 1
Char. 207: 0 → 1
Char. 209: 0 → 1
Char. 217: 0 → 1
Char. 219: 0 → 1
Char. 220: 0 → 1
Char. 231: 0 → 1
Char. 260: 2 → 0
Char. 272: 2 → 0
Char. 278: 3 → 0

***Microleter mckinzieorum*:**
Char. 0: 0 → 1
Char. 25: 1 → 0
Char. 36: 0 → 1
Char. 39: 0 → 1
Char. 51: 0 → 2
Char. 56: 0 → 1
Char. 70: 0 → 1
Char. 94: 0 → 1
Char. 106: 0 → 1
Char. 159: 0 → 1
Char. 276: 0 → 1

**Millerettidae:**
Char. 5: 1 → 0
Char. 24: 0 → 1
Char. 25: 1 → 0
Char. 44: 1 → 0
Char. 56: 0 → 1
Char. 57: 1 → 0
Char. 66: 0 → 2
Char. 78: 1 → 0
Char. 80: 1 → 0
Char. 84: 1 → 2
Char. 88: 1 → 0
Char. 96: 1 → 0
Char. 117: 0 → 1
Char. 124: 0 → 1
Char. 127: 0 → 1
Char. 135: 0 → 1
Char. 145: 0 → 1
Char. 202: 0 → 1
Char. 211: 0 → 1
Char. 230: 0 → 1
Char. 234: 0 → 1
Char. 248: 0 → 1
Char. 252: 0 → 1

***Nycteroleter ineptus*:**
Char. 278: 0 → 3

***Nyctiphruretus acudens*:**
Char. 21: 0 → 1
Char. 33: 1 → 2
Char. 41: 0 → 1
Char. 81: 1 → 0
Char. 84: 1 → 2
Char. 94: 0 → 1
Char. 167: 0 → 1
Char. 215: 1 → 0
Char. 224: 0 → 1
Char. 266: 0 → 1
Char. 272: 2 → 1
Char. 276: 0 → 1

***Orovenator mayorum*:**
Char. 8: 0 → 1
Char. 36: 0 → 1
Char. 160: 0 → 1
Char. 165: 0 → 1

***Owenetta* spp.:**
Char. 169: 1 → 0

***Paleothyris acadiana*:**
Char. 38: 1 → 0
Char. 50: 1 → 0
Char. 66: 1 → 2
Char. 102: 0 → 1
Char. 146: 1 → 0
Char. 180: 0 → 1
Char. 237: 0 → 1
Char. 239: 0 → 1

***Placodus* spp.:**
Char. 0: 1 → 2
Char. 9: 0 → 1
Char. 12: 0 → 1
Char. 13: 0 → 1
Char. 19: 0 → 1
Char. 26: 0 → 1
Char. 31: 0 → 1
Char. 46: 1 → 0
Char. 57: 0 → 1
Char. 78: 1 → 0
Char. 93: 1 → 0
Char. 102: 1 → 2
Char. 109: 1 → 0
Char. 140: 1 → 0
Char. 155: 0 → 1
Char. 163: 1 → 0
Char. 164: 0 → 1

***Procolophon* spp.:**
Char. 41: 0 → 1
Char. 69: 0 → 1
Char. 79: 0 → 1
Char. 88: 0 → 1
Char. 117: 1 → 0
Char. 149: 1 → 0
Char. 180: 0 → 1
Char. 204: 0 → 1
Char. 237: 0 → 1
Char. 238: 0 → 1
Char. 272: 2 → 1
Char. 278: 3 → 0

***Prolacerta broomi*:**
Char. 58: 1 → 0
Char. 66: 1 → 0
Char. 67: 1 → 0
Char. 80: 1 → 0
Char. 139: 1 → 0
Char. 147: 1 → 0
Char. 192: 1 → 0
Char. 203: 1 → 2

***Rhipaeosaurus* spp.:**
Char. 172: 0 → 1
Char. 277: 0 → 1

**Rhynchocephalia:**
Char. 0: 1 → 2
Char. 24: 0 → 1
Char. 75: 1 → 0
Char. 77: 0 → 1
Char. 94: 1 → 0
Char. 139: 1 → 0
Char. 167: 1 → 0
Char. 205: 1 → 0

**Rhynchosauria:**
Char. 0: 1 → 0
Char. 7: 0 → 1
Char. 9: 0 → 1
Char. 26: 0 → 1
Char. 68: 0 → 1
Char. 99: 1 → 0
Char. 150: 1 → 0
Char. 160: 0 → 1
Char. 161: 0 → 1
Char. 171: 0 → 2
Char. 223: 0 → 1
Char. 224: 0 → 1
Char. 241: 0 → 1

***Scutosaurus* spp.:**
Char. 175: 0 → 1
Char. 218: 0 → 1
Char. 243: 0 → 2
Char. 244: 0 → 1

***Sinosaurosphargis yunguiensis*:**
Char. 8: 0 → 1
Char. 30: 0 → 1
Char. 53: 0 → 1
Char. 82: 0 → 1
Char. 89: 1 → 0
Char. 127: 1 → 0
Char. 150: 1 → 0
Char. 154: 0 → 2
Char. 167: 1 → 0
Char. 255: 0 → 1

**Squamata:**
Char. 26: 0 → 1
Char. 45: 0 → 1
Char. 79: 0 → 2
Char. 80: 1 → 0
Char. 82: 0 → 1
Char. 92: 1 → 0
Char. 109: 1 → 0
Char. 160: 0 → 1
Char. 245: 0 → 1

***Trilophosaurus buettneri*:**
Char. 5: 1 → 0
Char. 11: 0 → 1
Char. 55: 1 → 0
Char. 93: 1 → 0
Char. 104: 0 → 1
Char. 106: 0 → 1
Char. 113: 0 → 1
Char. 122: 0 → 1
Char. 136: 1 → 0
Char. 144: 1 → 0
Char. 154: 0 → 1
Char. 157: 0 → 1
Char. 159: 1 → 0
Char. 177: 0 → 12
Char. 194: 0 → 1
Char. 203: 1 → 2
Char. 207: 1 → 0
Char. 208: 1 → 0
Char. 272: 1 → 0

***Youngina capensis*:**
Char. 5: 1 → 0
Char. 75: 0 → 1
Char. 170: 0 → 1
Char. 211: 0 → 1
Char. 239: 0 → 1

**Node 49:**
Char. 46: 1 → 0
Char. 88: 1 → 0
Char. 89: 1 → 0
Char. 93: 1 → 0
Char. 176: 0 → 1
Char. 195: 0 → 2
Char. 198: 0 → 1
Char. 246: 1 → 2
Char. 259: 0 → 1
Char. 265: 1 → 0
Char. 270: 0 → 1

**Node 50:**
Char. 65: 0 → 1
Char. 184: 0 → 1
Char. 205: 0 → 1
Char. 210: 0 → 1
Char. 219: 1 → 0
Char. 241: 0 → 1
Char. 246: 0 → 1
Char. 254: 0 → 1
Char. 255: 0 → 1
Char. 256: 0 → 1
Char. 268: 0 → 1
Char. 269: 0 → 1

**Node 51:**
Char. 0: 1 → 2
Char. 33: 1 → 2
Char. 62: 1 → 0
Char. 84: 01 → 2
Char. 134: 1 → 2
Char. 147: 0 → 1
Char. 152: 0 → 1
Char. 154: 0 → 1
Char. 155: 0 → 1
Char. 158: 0 → 1
Char. 161: 0 → 1
Char. 174: 0 → 1
Char. 181: 0 → 1
Char. 204: 0 → 2
Char. 214: 1 → 0
Char. 252: 0 → 1

**Node 52:**
Char. 23: 0 → 1
Char. 73: 0 → 1
Char. 81: 1 → 0
Char. 94: 0 → 1
Char. 131: 0 → 1
Char. 145: 0 → 1
Char. 148: 0 → 1
Char. 151: 0 → 1
Char. 208: 0 → 1
Char. 224: 1 → 0

**Node 53:**
Char. 44: 1 → 0
Char. 134: 0 → 1
Char. 214: 0 → 1
Char. 219: 0 → 1
Char. 231: 0 → 1

**Node 54:**
Char. 135: 0 → 1

**Node 55:**
Char. 20: 0 → 1
Char. 62: 0 → 1

**Node 56:**
Char. 0: 0 → 1
Char. 27: 0 → 1
Char. 29: 1 → 0
Char. 40: 2 → 0
Char. 57: 1 → 0
Char. 59: 0 → 1
Char. 67: 0 → 1
Char. 72: 1 → 2
Char. 89: 0 → 1
Char. 111: 0 → 1
Char. 112: 0 → 1
Char. 120: 0 → 1
Char. 180: 0 → 1
Char. 192: 0 → 1
Char. 193: 0 → 1
Char. 222: 0 → 1
Char. 224: 0 → 1
Char. 234: 0 → 1
Char. 237: 0 → 1
Char. 266: 0 → 1

**Node 57:**
Char. 5: 0 → 1
Char. 29: 0 → 1
Char. 40: 0 → 2
Char. 74: 0 → 1
Char. 80: 0 → 1
Char. 84: 0 → 1
Char. 88: 0 → 1
Char. 116: 0 → 1
Char. 132: 0 → 1
Char. 149: 0 → 1
Char. 156: 0 → 1
Char. 169: 0 → 1
Char. 201: 0 → 1
Char. 203: 0 → 1
Char. 235: 0 → 1

**Node 58:**
Char. 72: 0 → 1
Char. 79: 1 → 0
Char. 81: 0 → 1
Char. 93: 0 → 1
Char. 97: 0 → 1
Char. 104: 1 → 0
Char. 144: 0 → 1
Char. 173: 0 → 1
Char. 183: 0 → 1

**Node 60:**
Char. 19: 0 → 1
Char. 92: 1 → 0

**Node 61:**
Char. 4: 0 → 1
Char. 29: 0 → 1
Char. 213: 0 → 1
Char. 226: 0 → 2
Char. 228: 0 → 1
Char. 275: 0 → 1

**Node 62:**
Char. 27: 1 → 0
Char. 75: 0 → 1
Char. 107: 0 → 1
Char. 140: 0 → 1
Char. 147: 0 → 1

**Node 63:**
Char. 58: 0 → 1
Char. 61: 0 → 1
Char. 66: 0 → 1
Char. 69: 0 → 1
Char. 150: 0 → 1
Char. 167: 0 → 1
Char. 176: 0 → 1
Char. 205: 0 → 1
Char. 239: 0 → 1

**Node 64:**
Char. 24: 0 → 1
Char. 57: 1 → 0
Char. 79: 0 → 1
Char. 83: 0 → 1
Char. 110: 0 → 1
Char. 132: 1 → 0

**Node 65:**
Char. 73: 0 → 1
Char. 131: 1 → 0
Char. 205: 0 → 1
Char. 276: 0 → 1

**Node 66:**
Char. 18: 0 → 1
Char. 37: 0 → 1
Char. 107: 0 → 1
Char. 118: 1 → 0
Char. 125: 1 → 0
Char. 150: 0 → 1

**Node 67:**
Char. 23: 0 → 1
Char. 71: 1 → 0
Char. 102: 0 → 1
Char. 103: 0 → 1
Char. 106: 0 → 1
Char. 167: 0 → 1
Char. 214: 0 → 1
Char. 216: 1 → 0
Char. 235: 0 → 2
Char. 241: 0 → 1

**Node 68:**
Char. 138: 0 → 1
Char. 141: 0 → 1
Char. 207: 0 → 1

**Node 69:**
Char. 20: 0 → 1
Char. 33: 0 → 1
Char. 44: 1 → 0
Char. 70: 0 → 1
Char. 80: 1 → 0
Char. 117: 0 → 1
Char. 118: 0 → 1
Char. 125: 0 → 1
Char. 131: 0 → 1
Char. 194: 0 → 1
Char. 201: 1 → 0
Char. 211: 0 → 1

**Node 70:**
Char. 38: 1 → 2
Char. 39: 0 → 1
Char. 49: 0 → 1
Char. 66: 0 → 12
Char. 76: 0 → 1
Char. 85: 1 → 0
Char. 88: 1 → 0
Char. 95: 0 → 1
Char. 112: 0 → 1
Char. 148: 0 → 1
Char. 158: 0 → 1
Char. 159: 0 → 1
Char. 183: 1 → 2
Char. 192: 0 → 1
Char. 215: 0 → 1

**Node 71:**
Char. 87: 0 → 1

**Node 72:**
Char. 25: 1 → 0
Char. 76: 1 → 0

**Node 73:**
Char. 79: 0 → 1
Char. 93: 1 → 0
Char. 133: 0 → 1

**Node 74:**
Char. 85: 0 → 1
Char. 147: 1 → 2
Char. 154: 1 → 0
Char. 278: 3 → 0

**Node 75:**
Char. 50: 1 → 0
Char. 58: 0 → 1
Char. 59: 0 → 1
Char. 60: 0 → 1
Char. 72: 1 → 2
Char. 104: 0 → 1
Char. 105: 0 → 1
Char. 106: 0 → 2
Char. 107: 0 → 1
Char. 109: 0 → 1
Char. 110: 0 → 1
Char. 146: 1 → 0
Char. 155: 0 → 2

**Node 76:**
Char. 33: 1 → 0
Char. 38: 2 → 1
Char. 39: 1 → 0
Char. 42: 0 → 1
Char. 43: 0 → 1
Char. 46: 1 → 0
Char. 49: 1 → 0
Char. 52: 0 → 1
Char. 83: 01 → 2
Char. 84: 1 → 0
Char. 87: 0 → 1
Char. 93: 1 → 0
Char. 161: 0 → 1
Char. 163: 1 → 0
Char. 172: 0 → 2
Char. 174: 0 → 1
Char. 188: 0 → 1
Char. 189: 0 → 1
Char. 195: 0 → 1
Char. 204: 0 → 2
Char. 212: 0 → 1
Char. 236: 0 → 1
Char. 238: 0 → 2
Char. 242: 0 → 1
Char. 274: 0 → 1
Char. 275: 1 → 0

**Node 77:**
Char. 5: 1 → 0
Char. 29: 1 → 0
Char. 44: 1 → 0
Char. 59: 0 → 1
Char. 60: 0 → 1
Char. 66: 0 → 1
Char. 67: 0 → 1
Char. 72: 1 → 2
Char. 78: 1 → 0
Char. 84: 1 → 0
Char. 88: 1 → 0
Char. 93: 1 → 0
Char. 111: 0 → 1
Char. 123: 1 → 0
Char. 129: 0 → 1
Char. 154: 1 → 0
Char. 169: 1 → 0
Char. 170: 0 → 1
Char. 197: 0 → 1

**Node 78:**
Char. 20: 0 → 1
Char. 47: 0 → 1
Char. 83: 0 → 1
Char. 85: 1 → 0
Char. 107: 0 → 1
Char. 110: 0 → 1
Char. 169: 1 → 0
Char. 170: 0 → 1

**Node 79:**
Char. 2: 0 → 1
Char. 6: 0 → 1
Char. 98: 1 → 0
Char. 101: 0 → 1
Char. 113: 0 → 1
Char. 181: 0 → 1
Char. 186: 0 → 1
Char. 193: 1 → 0
Char. 198: 0 → 1
Char. 220: 0 → 1
Char. 231: 1 → 0
Char. 234: 1 → 0
Char. 239: 1 → 0
Char. 240: 1 → 0
Char. 266: 1 → 0

**Node 80:**
Char. 33: 1 → 2
Char. 68: 0 → 1
Char. 102: 0 → 1
Char. 103: 0 → 1
Char. 223: 0 → 1
Char. 229: 0 → 1
Char. 235: 1 → 2

**Node 81:**
Char. 17: 0 → 1
Char. 42: 0 → 1
Char. 264: 0 → 1

**Node 82:**
Char. 41: 0 → 1
Char. 61: 1 → 2
Char. 112: 1 → 0
Char. 128: 0 → 1
Char. 138: 0 → 1
Char. 146: 1 → 0
Char. 155: 0 → 1
Char. 192: 1 → 2
Char. 224: 0 → 1
Char. 226: 0 → 1
Char. 227: 0 → 1
Char. 233: 0 → 1

**Node 83:**
Char. 61: 1 → 3
Char. 90: 0 → 1
Char. 91: 0 → 1
Char. 109: 1 → 0
Char. 155: 0 → 1
Char. 209: 1 → 0

**ANALYSIS 86**
**(NO *CANDELARIA BARBOURI*, IMPLIED WEIGHTING, K = 2.75, NO CHARACTERS 247, 251, AND 253)**

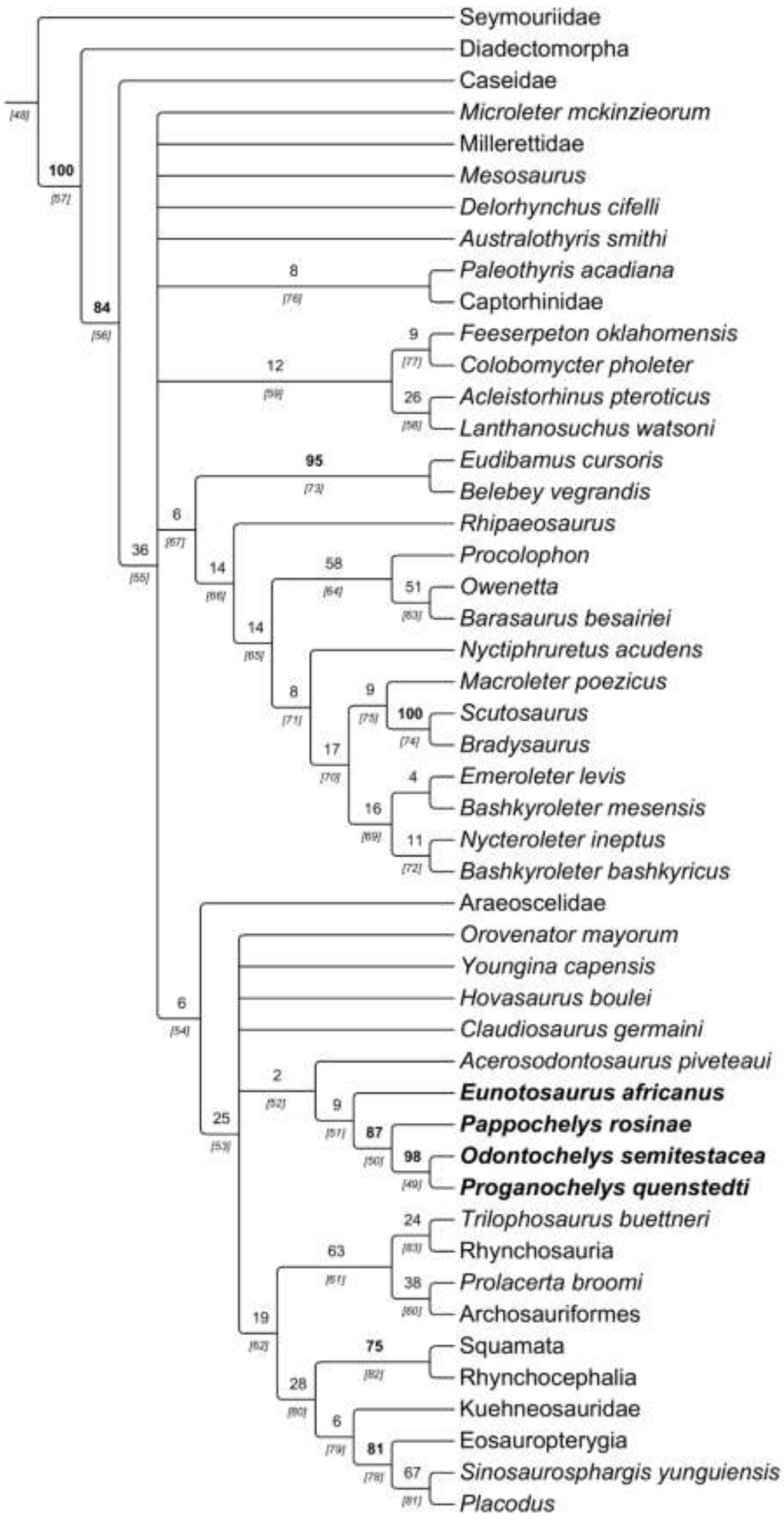

***Proganochelys quenstedti*:**
Char. 8: 0 → 1
Char. 11: 0 → 1
Char. 106: 0 → 1
Char. 108: 1 → 0
Char. 128: 0 → 1
Char. 175: 0 → 1
Char. 202: 1 → 0
Char. 206: 2 → 0
Char. 207: 1 → 0
Char. 209: 1 → 0
Char. 244: 0 → 1
Char. 248: 0 → 1
Char. 250: 0 → 1
Char. 252: 1 → 0
Char. 262: 0 → 1

***Pappochelys rosinae*:**
Char. 0: 2 → 0
Char. 1: 0 → 1
Char. 5: 1 → 0
Char. 12: 0 → 1
Char. 41: 0 → 1
Char. 49: 0 → 1
Char. 75: 0 → 1
Char. 81: 0 → 1
Char. 169: 1 → 0
Char. 260: 2 → 0

***Eunotosaurus africanus*:**
Char. 19: 0 → 1
Char. 59: 1 → 0
Char. 60: 1 → 0
Char. 72: 2 → 1
Char. 76: 0 → 1
Char. 97: 1 → 0
Char. 103: 0 → 1
Char. 153: 1 → 0
Char. 191: 1 → 0
Char. 192: 1 → 0
Char. 202: 1 → 0
Char. 211: 0 → 1
Char. 222: 1 → 0
Char. 237: 1 → 0
Char. 248: 0 → 1
Char. 249: 0 → 2
Char. 250: 0 → 1
Char. 263: 0 → 1
Char. 273: 0 → 1
Char. 274: 0 → 1
Char. 275: 0 → 1
Char. 276: 0 → 1
Char. 277: 0 → 1
Char. 278: 0 → 3

***Acerosodontosaurus piveteaui*:**
Char. 127: 1 → 0
Char. 128: 0 → 1
Char. 278: 0 → 1

***Acleistorhinus pteroticus*:**
Char. 21: 0 → 1
Char. 146: 1 → 0

**Araeoscelidae:**
Char. 28: 0 → 1
Char. 106: 0 → 1
Char. 116: 1 → 0
Char. 117: 01 → 2
Char. 169: 1 → 0
Char. 170: 0 → 1
Char. 197: 0 → 1
Char. 239: 0 → 1

**Archosauriformes:**
Char. 32: 0 → 1
Char. 94: 1 → 0
Char. 112: 1 → 0
Char. 152: 0 → 1
Char. 154: 0 → 2
Char. 166: 1 → 0
Char. 171: 0 → 1
Char. 185: 0 → 1
Char. 201: 0 → 1
Char. 204: 0 → 1
Char. 218: 0 → 3
Char. 242: 0 → 1

***Australothyris smithi*:**
Char. 23: 0 → 1
Char. 24: 0 → 1
Char. 34: 0 → 1
Char. 55: 1 → 0
Char. 57: 1 → 0
Char. 71: 1 → 0
Char. 79: 0 → 1
Char. 85: 1 → 0
Char. 98: 1 → 0
Char. 100: 0 → 1
Char. 103: 0 → 1
Char. 110: 0 → 1
Char. 112: 0 → 1
Char. 123: 1 → 0
Char. 129: 0 → 1
Char. 131: 0 → 1
Char. 132: 1 → 0
Char. 144: 1 → 0
Char. 147: 0 → 1
Char. 149: 1 → 0
Char. 150: 0 → 1
Char. 166: 0 → 1

***Barasaurus besairiei*:**
Char. 33: 1 → 0

***Bashkyroleter bashkyricus*:**
Char. 275: 1 → 0

***Bashkyroleter mesensis*:**
Char. 12: 0 → 1
Char. 66: 1 → 0
Char. 169: 1 → 0

***Belebey vegrandis*:**
Char. 154: 1 → 0

***Bradysaurus* spp.:**
Char. 19: 0 → 1
Char. 25: 1 → 0
Char. 73: 0 → 1
Char. 79: 0 → 1
Char. 135: 1 → 0
Char. 249: 0 → 1

**Captorhinidae:**
Char. 3: 0 → 1
Char. 23: 0 → 1
Char. 25: 1 → 0
Char. 26: 0 → 1
Char. 73: 0 → 1
Char. 75: 0 → 1
Char. 108: 1 → 0
Char. 183: 1 → 0
Char. 201: 1 → 0
Char. 203: 1 → 2
Char. 216: 1 → 0

**Caseidae:**
Char. 24: 0 → 1
Char. 25: 1 → 0
Char. 36: 0 → 1
Char. 38: 1 → 0
Char. 46: 1 → 0
Char. 50: 1 → 0
Char. 56: 0 → 1
Char. 170: 0 → 1
Char. 194: 0 → 1
Char. 273: 0 → 1
Char. 274: 0 → 1
Char. 278: 3 → 2

***Claudiosaurus germaini*:**
Char. 24: 0 → 1
Char. 34: 0 → 1
Char. 36: 0 → 1
Char. 43: 1 → 0
Char. 56: 0 → 1
Char. 64: 0 → 1
Char. 70: 0 → 1
Char. 105: 0 → 1
Char. 106: 0 → 1
Char. 117: 01 → 2
Char. 126: 0 → 1
Char. 127: 1 → 0
Char. 130: 0 → 1
Char. 131: 0 → 1
Char. 141: 1 → 0
Char. 144: 1 → 0
Char. 148: 0 → 1
Char. 182: 0 → 1
Char. 187: 1 → 0
Char. 190: 0 → 1
Char. 199: 0 → 1
Char. 201: 0 → 1
Char. 203: 1 → 2
Char. 204: 0 → 1
Char. 222: 1 → 0
Char. 234: 1 → 0
Char. 272: 2 → 1

***Colobomycter pholeter*:**
Char. 21: 0 → 1
Char. 25: 1 → 0
Char. 84: 1 → 0
Char. 154: 1 → 0
Char. 167: 0 → 1
Char. 267: 0 → 1

***Delorhynchus cifelli*:**
Char. 5: 0 → 1
Char. 18: 0 → 1
Char. 20: 0 → 1
Char. 21: 0 → 1
Char. 24: 0 → 1
Char. 26: 0 → 1
Char. 28: 0 → 1
Char. 33: 0 → 2
Char. 39: 0 → 1
Char. 52: 0 → 1
Char. 100: 0 → 1
Char. 111: 0 → 1
Char. 116: 1 → 0
Char. 131: 0 → 1
Char. 147: 0 → 1
Char. 156: 1 → 0
Char. 159: 0 → 1
Char. 163: 0 → 1
Char. 166: 0 → 1
Char. 167: 0 → 1
Char. 189: 0 → 1
Char. 191: 0 → 1
Char. 192: 0 → 1
Char. 204: 0 → 2
Char. 267: 0 → 1

**Diadectomorpha:**
Char. 0: 0 → 1
Char. 64: 0 → 1
Char. 70: 0 → 1
Char. 122: 0 → 1
Char. 123: 1 → 0
Char. 146: 1 → 0
Char. 275: 1 → 0
Char. 278: 3 → 0

***Emeroleter levis*:**
Char. 51: 1 → 0

**Eosauropterygia:**
Char. 166: 1 → 0
Char. 174: 0 → 1
Char. 178: 0 → 1
Char. 194: 0 → 2
Char. 272: 1 → 0

***Eudibamus cursoris*:**
Char. 154: 1 → 2

***Feeserpeton oklahomensis*:**
Char. 51: 0 → 1
Char. 157: 0 → 1
Char. 158: 0 → 1

***Hovasaurus boulei*:**
Char. 41: 0 → 1
Char. 55: 1 → 0
Char. 60: 1 → 0
Char. 72: 2 → 1
Char. 77: 0 → 2
Char. 78: 1 → 0
Char. 79: 0 → 1
Char. 93: 1 → 0
Char. 113: 0 → 1
Char. 138: 0 → 1
Char. 141: 1 → 0
Char. 146: 1 → 0
Char. 154: 0 → 1
Char. 204: 0 → 2
Char. 206: 0 → 2
Char. 215: 0 → 1
Char. 219: 1 → 0
Char. 278: 0 → 3

**Kuehneosauridae:**
Char. 7: 0 → 1
Char. 24: 0 → 1
Char. 27: 0 → 1
Char. 43: 1 → 0
Char. 79: 0 → 2
Char. 107: 1 → 0
Char. 140: 1 → 0
Char. 147: 1 → 0
Char. 148: 1 → 0
Char. 245: 0 → 1
Char. 278: 0 → 3

***Lanthanosuchus watsoni*:**
Char. 25: 1 → 0
Char. 51: 0 → 1
Char. 76: 0 → 1
Char. 86: 0 → 1
Char. 98: 1 → 0
Char. 138: 0 → 1
Char. 144: 1 → 0
Char. 154: 1 → 2

***Macroleter poezicus*:**
Char. 9: 0 → 1
Char. 26: 0 → 1
Char. 66: 1 → 2
Char. 126: 0 → 1
Char. 134: 0 → 2
Char. 140: 0 → 1
Char. 146: 1 → 0
Char. 169: 1 → 0

***Mesosaurus* spp.:**
Char. 0: 0 → 1
Char. 2: 0 → 1
Char. 6: 0 → 1
Char. 8: 0 → 1

Char. 9: 0 → 1
Char. 13: 0 → 1
Char. 19: 0 → 1
Char. 23: 0 → 1
Char. 26: 0 → 1
Char. 29: 1 → 0
Char. 33: 0 → 1
Char. 38: 1 → 0
Char. 41: 0 → 1
Char. 50: 1 → 0
Char. 67: 0 → 1
Char. 76: 0 → 1
Char. 84: 1 → 0
Char. 85: 1 → 0
Char. 94: 0 → 1
Char. 107: 0 → 1
Char. 109: 0 → 1
Char. 111: 0 → 1
Char. 115: 0 → 1
Char. 146: 1 → 0
Char. 148: 0 → 1
Char. 149: 1 → 0
Char. 164: 0 → 1
Char. 167: 0 → 1
Char. 176: 0 → 1
Char. 183: 1 → 0
Char. 184: 0 → 1
Char. 199: 0 → 1
Char. 202: 0 → 1
Char. 204: 0 → 2
Char. 206: 0 → 1
Char. 207: 0 → 1
Char. 209: 0 → 1
Char. 217: 0 → 1
Char. 219: 0 → 1
Char. 220: 0 → 1
Char. 231: 0 → 1
Char. 260: 2 → 0
Char. 272: 2 → 0
Char. 278: 3 → 0

***Microleter mckinzieorum*:**
Char. 0: 0 → 1
Char. 18: 0 → 1
Char. 24: 0 → 1
Char. 25: 1 → 0
Char. 36: 0 → 1
Char. 39: 0 → 1
Char. 51: 0 → 2
Char. 56: 0 → 1
Char. 57: 1 → 0
Char. 70: 0 → 1
Char. 76: 0 → 1
Char. 79: 0 → 1
Char. 94: 0 → 1
Char. 106: 0 → 1
Char. 110: 0 → 1
Char. 132: 1 → 0
Char. 159: 0 → 1
Char. 276: 0 → 1
Char. 278: 3 → 1

**Millerettidae:**
Char. 24: 0 → 1
Char. 25: 1 → 0
Char. 44: 1 → 0
Char. 56: 0 → 1
Char. 57: 1 → 0
Char. 66: 0 → 2
Char. 78: 1 → 0
Char. 80: 1 → 0
Char. 84: 1 → 2
Char. 88: 1 → 0
Char. 96: 1 → 0
Char. 121: 0 → 1
Char. 124: 0 → 1
Char. 127: 0 → 1
Char. 135: 0 → 1
Char. 145: 0 → 1
Char. 202: 0 → 1
Char. 211: 0 → 1
Char. 230: 0 → 1
Char. 234: 0 → 1
Char. 248: 0 → 1
Char. 252: 0 → 1

***Nycteroleter ineptus*:**
Char. 66: 1 → 0

***Nyctiphruretus acudens*:**
Char. 21: 0 → 1
Char. 41: 0 → 1
Char. 66: 1 → 2
Char. 81: 1 → 0
Char. 84: 1 → 2
Char. 94: 0 → 1
Char. 166: 1 → 0
Char. 215: 1 → 0
Char. 224: 0 → 1
Char. 226: 1 → 0
Char. 266: 0 → 1
Char. 272: 2 → 1
Char. 276: 0 → 1

***Orovenator mayorum*:**
Char. 8: 0 → 1
Char. 24: 0 → 1
Char. 33: 1 → 0
Char. 36: 0 → 1
Char. 43: 1 → 0
Char. 50: 0 → 1
Char. 92: 1 → 0
Char. 135: 1 → 0
Char. 159: 1 → 0
Char. 160: 0 → 1
Char. 165: 0 → 1
Char. 278: 0 → 1

***Owenetta* spp.:**
Char. 142: 1 → 0
Char. 169: 1 → 0

***Paleothyris acadiana*:**
Char. 38: 1 → 0
Char. 50: 1 → 0
Char. 66: 1 → 2
Char. 102: 0 → 1
Char. 146: 1 → 0
Char. 237: 0 → 1
Char. 239: 0 → 1

***Placodus* spp.:**
Char. 0: 1 → 2
Char. 9: 0 → 1
Char. 12: 0 → 1
Char. 13: 0 → 1
Char. 19: 0 → 1
Char. 31: 0 → 1
Char. 46: 1 → 0
Char. 57: 0 → 1
Char. 78: 1 → 0
Char. 93: 1 → 0
Char. 102: 1 → 2
Char. 109: 1 → 0
Char. 140: 1 → 0
Char. 155: 0 → 1
Char. 163: 1 → 0
Char. 164: 0 → 1

***Procolophon* spp.:**
Char. 41: 0 → 1
Char. 69: 0 → 1
Char. 79: 0 → 1
Char. 86: 0 → 1
Char. 88: 0 → 1
Char. 101: 0 → 1
Char. 141: 0 → 1
Char. 149: 1 → 0
Char. 203: 1 → 2
Char. 204: 0 → 1
Char. 230: 0 → 1
Char. 237: 0 → 1
Char. 238: 0 → 1
Char. 239: 1 → 0
Char. 272: 2 → 1
Char. 278: 3 → 0

***Prolacerta broomi*:**
Char. 58: 1 → 0
Char. 66: 1 → 0
Char. 67: 1 → 0
Char. 80: 1 → 0
Char. 139: 1 → 0
Char. 147: 1 → 0
Char. 192: 1 → 0
Char. 203: 1 → 2
Char. 206: 0 → 12

***Rhipaeosaurus* spp.:**
Char. 172: 0 → 1
Char. 277: 0 → 1

**Rhynchocephalia:**
Char. 0: 1 → 2
Char. 24: 0 → 1
Char. 77: 0 → 1
Char. 88: 1 → 0
Char. 94: 1 → 0
Char. 139: 1 → 0
Char. 167: 1 → 0
Char. 205: 1 → 0

**Rhynchosauria:**
Char. 0: 1 → 0
Char. 7: 0 → 1
Char. 9: 0 → 1
Char. 26: 0 → 1
Char. 44: 1 → 0
Char. 68: 0 → 1
Char. 99: 1 → 0
Char. 150: 1 → 0
Char. 160: 0 → 1
Char. 161: 0 → 1
Char. 171: 0 → 2
Char. 223: 0 → 1
Char. 241: 0 → 1

***Scutosaurus* spp.:**
Char. 175: 0 → 1
Char. 190: 0 → 1
Char. 218: 0 → 1
Char. 243: 0 → 2
Char. 244: 0 → 1

***Sinosaurosphargis yunguiensis*:**
Char. 8: 0 → 1
Char. 30: 0 → 1
Char. 53: 0 → 1
Char. 89: 1 → 0
Char. 127: 1 → 0
Char. 150: 1 → 0
Char. 154: 0 → 2
Char. 167: 1 → 0
Char. 255: 0 → 1

**Squamata:**
Char. 45: 0 → 1
Char. 79: 0 → 2
Char. 80: 1 → 0
Char. 92: 1 → 0
Char. 109: 1 → 0
Char. 160: 0 → 1
Char. 200: 0 → 1
Char. 245: 0 → 1

***Trilophosaurus buettneri*:**
Char. 5: 1 → 0
Char. 11: 0 → 1
Char. 55: 1 → 0
Char. 93: 1 → 0
Char. 104: 0 → 1
Char. 106: 0 → 1
Char. 113: 0 → 1
Char. 122: 0 → 1
Char. 136: 1 → 0
Char. 144: 1 → 0
Char. 154: 0 → 1
Char. 157: 0 → 1
Char. 159: 1 → 0
Char. 177: 0 → 12
Char. 194: 0 → 1
Char. 203: 1 → 2
Char. 207: 1 → 0
Char. 208: 1 → 0
Char. 272: 1 → 0

***Youngina capensis*:**
Char. 5: 1 → 0
Char. 25: 0 → 1
Char. 44: 1 → 0
Char. 56: 0 → 1
Char. 75: 0 → 1
Char. 92: 1 → 0
Char. 134: 0 → 1
Char. 163: 1 → 0
Char. 170: 0 → 1
Char. 211: 0 → 1
Char. 215: 0 → 1
Char. 239: 0 → 1

**Node 49:**
Char. 46: 1 → 0
Char. 88: 1 → 0
Char. 89: 1 → 0
Char. 93: 1 → 0
Char. 176: 0 → 1
Char. 195: 0 → 2
Char. 198: 0 → 1
Char. 246: 1 → 2
Char. 259: 0 → 1
Char. 265: 1 → 0
Char. 270: 0 → 1

**Node 50:**
Char. 65: 0 → 1
Char. 131: 0 → 1
Char. 184: 0 → 1
Char. 205: 0 → 1
Char. 210: 0 → 1
Char. 219: 1 → 0
Char. 241: 0 → 1
Char. 246: 0 → 1
Char. 254: 0 → 1
Char. 255: 0 → 1
Char. 256: 0 → 1
Char. 268: 0 → 1
Char. 269: 0 → 1

**Node 51:**
Char. 174: 0 → 1
Char. 204: 0 → 2

**Node 52:**
Char. 78: 1 → 0
Char. 81: 1 → 0
Char. 155: 0 → 1
Char. 206: 0 → 2
Char. 208: 0 → 1

**Node 53:**
Char. 5: 0 → 1
Char. 20: 0 → 1
Char. 25: 1 → 0

Char. 33: 0 → 1
Char. 62: 0 → 1
Char. 109: 0 → 1
Char. 127: 0 → 1
Char. 129: 0 → 1
Char. 135: 0 → 1
Char. 141: 0 → 1
Char. 159: 0 → 1
Char. 179: 0 → 1
Char. 187: 0 → 1
Char. 188: 0 → 1
Char. 191: 0 → 1
Char. 196: 0 → 1
Char. 201: 1 → 0
Char. 202: 0 → 1
Char. 207: 0 → 1
Char. 209: 0 → 1
Char. 219: 0 → 1
Char. 265: 0 → 1
Char. 275: 1 → 0

**Node 54:**
Char. 0: 0 → 1
Char. 29: 1 → 0
Char. 40: 2 → 0
Char. 43: 0 → 1
Char. 50: 1 → 0
Char. 57: 1 → 0
Char. 59: 0 → 1
Char. 60: 0 → 1
Char. 67: 0 → 1
Char. 72: 1 → 2
Char. 89: 0 → 1
Char. 111: 0 → 1
Char. 112: 0 → 1
Char. 120: 0 → 1
Char. 154: 1 → 0
Char. 163: 0 → 1
Char. 192: 0 → 1
Char. 193: 0 → 1
Char. 222: 0 → 1
Char. 234: 0 → 1
Char. 237: 0 → 1
Char. 266: 0 → 1

**Node 55:**
Char. 29: 0 → 1
Char. 40: 0 → 2
Char. 74: 0 → 1
Char. 80: 0 → 1
Char. 84: 0 → 1
Char. 88: 0 → 1
Char. 116: 0 → 1
Char. 132: 0 → 1
Char. 149: 0 → 1
Char. 156: 0 → 1
Char. 169: 0 → 1
Char. 201: 0 → 1
Char. 203: 0 → 1
Char. 235: 0 → 1

**Node 56:**
Char. 72: 0 → 1
Char. 79: 1 → 0
Char. 81: 0 → 1
Char. 93: 0 → 1
Char. 97: 0 → 1
Char. 104: 1 → 0
Char. 144: 0 → 1
Char. 173: 0 → 1
Char. 183: 0 → 1

**Node 58:**
Char. 95: 0 → 1
Char. 113: 0 → 1
Char. 114: 0 → 1

**Node 59:**
Char. 5: 0 → 1
Char. 20: 0 → 1
Char. 47: 0 → 1
Char. 79: 0 → 1
Char. 110: 0 → 1
Char. 131: 0 → 1
Char. 137: 0 → 1
Char. 140: 0 → 2
Char. 147: 0 → 1
Char. 159: 0 → 1
Char. 163: 0 → 1
Char. 166: 0 → 1

**Node 60:**
Char. 19: 0 → 1
Char. 92: 1 → 0

**Node 61:**
Char. 4: 0 → 1
Char. 15: 0 → 1
Char. 29: 0 → 1
Char. 213: 0 → 1
Char. 226: 0 → 2
Char. 228: 0 → 1
Char. 275: 0 → 1

**Node 62:**
Char. 58: 0 → 1
Char. 61: 0 → 1
Char. 66: 0 → 1
Char. 69: 0 → 1
Char. 107: 0 → 1
Char. 126: 0 → 1
Char. 131: 0 → 1
Char. 134: 0 → 1
Char. 140: 0 → 1
Char. 147: 0 → 1
Char. 148: 0 → 1
Char. 150: 0 → 1
Char. 167: 0 → 1
Char. 176: 0 → 1
Char. 190: 0 → 1
Char. 205: 0 → 1
Char. 208: 0 → 1
Char. 230: 0 → 1

Char. 239: 0 → 1
Char. 260: 2 → 1

**Node 63:**
Char. 73: 0 → 1
Char. 205: 0 → 1
Char. 273: 0 → 1
Char. 276: 0 → 1

**Node 64:**
Char. 18: 0 → 1
Char. 25: 1 → 0
Char. 37: 0 → 1
Char. 106: 0 → 1
Char. 107: 0 → 1
Char. 126: 0 → 1
Char. 150: 0 → 1

**Node 65:**
Char. 186: 0 → 1

**Node 66:**
Char. 194: 0 → 1
Char. 201: 1 → 0
Char. 211: 0 → 1
Char. 226: 0 → 1

**Node 67:**
Char. 5: 0 → 1
Char. 38: 1 → 2
Char. 39: 0 → 1
Char. 49: 0 → 1
Char. 66: 0 → 1
Char. 76: 0 → 1
Char. 85: 1 → 0
Char. 88: 1 → 0
Char. 95: 0 → 1
Char. 112: 0 → 1
Char. 148: 0 → 1
Char. 158: 0 → 1
Char. 159: 0 → 1
Char. 166: 0 → 1
Char. 183: 1 → 2
Char. 192: 0 → 1
Char. 215: 0 → 1
Char. 239: 0 → 1

**Node 69:**
Char. 133: 0 → 1

**Node 70:**
Char. 100: 0 → 1
Char. 113: 0 → 1

**Node 71:**
Char. 70: 0 → 1
Char. 118: 0 → 1
Char. 125: 0 → 1

**Node 73:**
Char. 58: 0 → 1
Char. 59: 0 → 1

Char. 60: 0 → 1
Char. 72: 1 → 2
Char. 104: 0 → 1
Char. 105: 0 → 1
Char. 106: 0 → 2
Char. 107: 0 → 1
Char. 109: 0 → 1
Char. 146: 1 → 0

**Node 74:**
Char. 23: 0 → 1
Char. 33: 1 → 0
Char. 38: 2 → 1
Char. 39: 1 → 0
Char. 42: 0 → 1
Char. 43: 0 → 1
Char. 46: 1 → 0
Char. 49: 1 → 0
Char. 71: 1 → 0
Char. 83: 0 → 2
Char. 101: 0 → 1
Char. 103: 0 → 1
Char. 106: 0 → 1
Char. 161: 0 → 1
Char. 163: 1 → 0
Char. 172: 0 → 2
Char. 174: 0 → 1
Char. 188: 0 → 1
Char. 203: 1 → 2
Char. 212: 0 → 1
Char. 216: 1 → 0
Char. 236: 0 → 1
Char. 238: 0 → 2
Char. 239: 1 → 0
Char. 241: 0 → 1
Char. 242: 0 → 1
Char. 273: 0 → 1
Char. 274: 0 → 1
Char. 275: 1 → 0

**Node 75:**
Char. 52: 0 → 1
Char. 75: 0 → 1
Char. 84: 1 → 0
Char. 121: 0 → 1
Char. 230: 0 → 1

**Node 76:**
Char. 29: 1 → 0
Char. 44: 1 → 0
Char. 59: 0 → 1
Char. 60: 0 → 1
Char. 66: 0 → 1
Char. 67: 0 → 1
Char. 72: 1 → 2
Char. 78: 1 → 0
Char. 84: 1 → 0
Char. 88: 1 → 0
Char. 93: 1 → 0
Char. 111: 0 → 1
Char. 123: 1 → 0
Char. 129: 0 → 1

Char. 154: 1 → 0
Char. 169: 1 → 0
Char. 170: 0 → 1
Char. 197: 0 → 1

**Node 77:**
Char. 85: 1 → 0
Char. 107: 0 → 1

**Node 78:**
Char. 2: 0 → 1
Char. 6: 0 → 1
Char. 186: 0 → 1
Char. 198: 0 → 1
Char. 200: 0 → 1
Char. 220: 0 → 1
Char. 239: 1 → 0

**Node 79:**
Char. 98: 1 → 0
Char. 113: 0 → 1
Char. 181: 0 → 1
Char. 206: 0 → 2

**Node 80:**
Char. 35: 0 → 1
Char. 102: 0 → 1
Char. 103: 0 → 1
Char. 184: 0 → 1
Char. 201: 0 → 1
Char. 210: 0 → 1
Char. 223: 0 → 1
Char. 229: 0 → 1

**Node 81:**
Char. 17: 0 → 1
Char. 42: 0 → 1
Char. 264: 0 → 1

**Node 82:**
Char. 41: 0 → 1
Char. 61: 1 → 2
Char. 112: 1 → 0
Char. 138: 0 → 1
Char. 146: 1 → 0
Char. 155: 0 → 1
Char. 192: 1 → 2
Char. 226: 0 → 1
Char. 227: 0 → 1
Char. 233: 0 → 1
Char. 267: 01 → 2

**Node 83:**
Char. 61: 1 → 3
Char. 90: 0 → 1
Char. 91: 0 → 1
Char. 109: 1 → 0
Char. 155: 0 → 1
Char. 209: 1 → 0

## Analysis 87
## (no *Candelaria barbouri*, no characters 174, 247, 251, and 253)

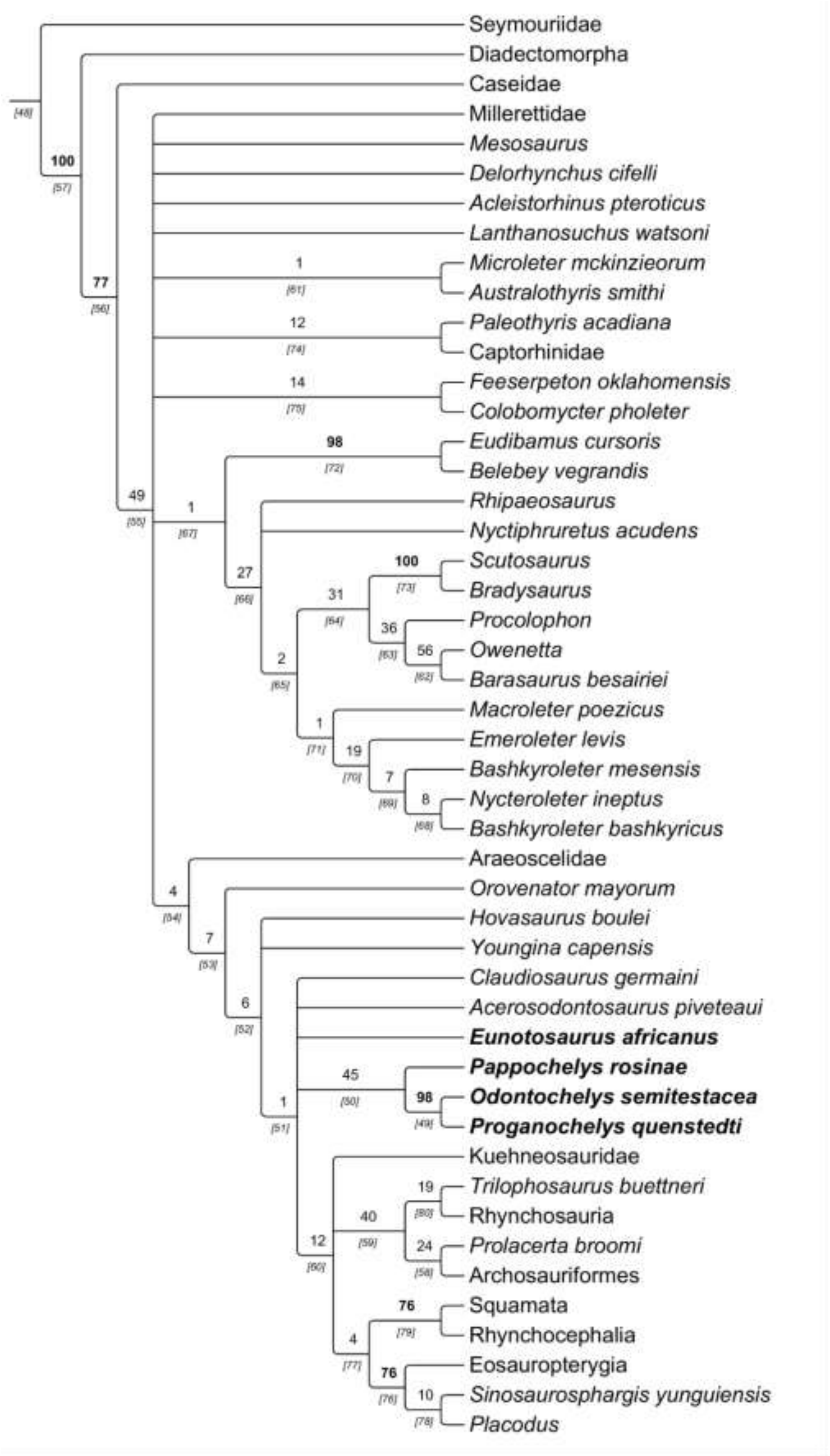

***Proganochelys quenstedti*:**
Char. 8: 0 → 1
Char. 11: 0 → 1
Char. 106: 0 → 1
Char. 108: 1 → 0
Char. 128: 0 → 1
Char. 175: 0 → 1
Char. 202: 1 → 0
Char. 207: 1 → 0
Char. 209: 1 → 0
Char. 244: 0 → 1
Char. 248: 0 → 1
Char. 250: 0 → 1
Char. 262: 0 → 1
Char. 192: 1 → 0
Char. 194: 0 → 1
Char. 202: 1 → 0
Char. 211: 0 → 1
Char. 222: 1 → 0
Char. 237: 1 → 0
Char. 240: 1 → 0
Char. 248: 0 → 1
Char. 249: 0 → 2
Char. 250: 0 → 1
Char. 252: 0 → 1
Char. 263: 0 → 1
Char. 267: 1 → 0
Char. 272: 1 → 2
Char. 273: 0 → 1
Char. 274: 0 → 1
Char. 275: 0 → 1
Char. 276: 0 → 1
Char. 277: 0 → 1
Char. 278: 0 → 3

***Pappochelys rosinae*:**
Char. 1: 0 → 1
Char. 5: 1 → 0
Char. 12: 0 → 1
Char. 41: 0 → 1
Char. 49: 0 → 1
Char. 55: 1 → 0
Char. 67: 1 → 0
Char. 75: 0 → 1
Char. 81: 0 → 1
Char. 169: 1 → 0
Char. 260: 2 → 0

***Odontochelys semitestacea*:**
Char. 109: 1 → 0
Char. 112: 1 → 0
Char. 124: 0 → 1

***Eunotosaurus africanus*:**
Char. 0: 1 → 2
Char. 19: 0 → 1
Char. 23: 1 → 0
Char. 24: 0 → 1
Char. 33: 1 → 2
Char. 42: 0 → 1
Char. 48: 1 → 0
Char. 50: 0 → 1
Char. 55: 1 → 0
Char. 57: 0 → 1
Char. 59: 1 → 0
Char. 60: 1 → 0
Char. 62: 1 → 0
Char. 67: 1 → 0
Char. 72: 2 → 1
Char. 76: 0 → 1
Char. 84: 1 → 2
Char. 97: 1 → 0
Char. 102: 0 → 1
Char. 103: 0 → 1
Char. 109: 1 → 0
Char. 112: 1 → 0
Char. 124: 0 → 1
Char. 129: 1 → 0
Char. 131: 1 → 0
Char. 153: 1 → 0
Char. 161: 0 → 1
Char. 191: 1 → 0

***Acerosodontosaurus piveteaui*:**
Char. 78: 1 → 0
Char. 127: 1 → 0
Char. 128: 0 → 1
Char. 267: 1 → 0
Char. 278: 0 → 1

***Acleistorhinus pteroticus:***
Char. 20: 0 → 1
Char. 21: 0 → 1
Char. 30: 0 → 1
Char. 33: 0 → 1
Char. 47: 0 → 1
Char. 55: 1 → 0
Char. 56: 0 → 1
Char. 64: 0 → 1
Char. 70: 0 → 1
Char. 79: 0 → 1
Char. 95: 0 → 1
Char. 110: 0 → 1
Char. 113: 0 → 1
Char. 114: 0 → 1
Char. 131: 0 → 1
Char. 137: 0 → 1
Char. 140: 0 → 2
Char. 146: 1 → 0
Char. 159: 0 → 1
Char. 169: 1 → 0
Char. 170: 0 → 1
Char. 278: 3 → 1

**Araeoscelidae:**
Char. 5: 1 → 0
Char. 23: 0 → 1
Char. 28: 0 → 1
Char. 106: 0 → 1
Char. 116: 1 → 0
Char. 117: 01 → 2
Char. 169: 1 → 0
Char. 170: 0 → 1
Char. 197: 0 → 1
Char. 239: 0 → 1

**Archosauriformes:**
Char. 32: 0 → 1
Char. 94: 1 → 0
Char. 112: 1 → 0
Char. 152: 0 → 1
Char. 154: 0 → 2
Char. 166: 1 → 0
Char. 171: 0 → 1
Char. 185: 0 → 1
Char. 204: 0 → 1
Char. 218: 0 → 3
Char. 224: 0 → 1
Char. 242: 0 → 1

***Australothyris smithi*:**
Char. 23: 0 → 1
Char. 34: 0 → 1
Char. 55: 1 → 0
Char. 71: 1 → 0
Char. 85: 1 → 0
Char. 112: 0 → 1
Char. 129: 0 → 1
Char. 131: 0 → 1

***Barasaurus besairiei*:**
Char. 33: 1 → 0
Char. 75: 1 → 0
Char. 216: 0 → 1

***Bashkyroleter bashkyricus*:**
Char. 275: 1 → 0

***Bashkyroleter mesensis*:**
Char. 169: 1 → 0

***Belebey vegrandis*:**
Char. 154: 1 → 0

***Bradysaurus* spp.:**
Char. 19: 0 → 1
Char. 73: 0 → 1
Char. 79: 0 → 1
Char. 135: 1 → 0
Char. 249: 0 → 1

**Captorhinidae:**
Char. 3: 0 → 1
Char. 23: 0 → 1
Char. 25: 1 → 0
Char. 26: 0 → 1
Char. 73: 0 → 1
Char. 75: 0 → 1
Char. 83: 0 → 1
Char. 108: 1 → 0
Char. 183: 1 → 0
Char. 201: 1 → 0
Char. 203: 1 → 2
Char. 216: 1 → 0

**Caseidae:**
Char. 24: 0 → 1
Char. 25: 1 → 0
Char. 36: 0 → 1
Char. 38: 1 → 0
Char. 46: 1 → 0
Char. 50: 1 → 0
Char. 56: 0 → 1
Char. 170: 0 → 1
Char. 194: 0 → 1
Char. 273: 0 → 1
Char. 274: 0 → 1
Char. 278: 3 → 2

***Claudiosaurus germaini*:**
Char. 21: 1 → 0
Char. 24: 0 → 1
Char. 34: 0 → 1
Char. 36: 0 → 1
Char. 43: 1 → 0
Char. 56: 0 → 1
Char. 81: 0 → 1
Char. 84: 1 → 0
Char. 105: 0 → 1
Char. 106: 0 → 1
Char. 117: 01 → 2
Char. 127: 1 → 0
Char. 141: 1 → 0
Char. 144: 1 → 0
Char. 147: 1 → 0
Char. 166: 1 → 0
Char. 182: 0 → 1
Char. 187: 1 → 0
Char. 190: 0 → 1
Char. 199: 0 → 1
Char. 201: 0 → 1
Char. 204: 02 → 1
Char. 208: 1 → 0
Char. 214: 0 → 1
Char. 222: 1 → 0
Char. 231: 1 → 0
Char. 234: 1 → 0

***Colobomycter pholeter*:**
Char. 21: 0 → 1
Char. 25: 1 → 0
Char. 84: 1 → 0
Char. 154: 1 → 0
Char. 167: 0 → 1
Char. 267: 0 → 1

***Delorhynchus cifelli*:**
Char. 18: 0 → 1
Char. 20: 0 → 1
Char. 21: 0 → 1
Char. 24: 0 → 1
Char. 26: 0 → 1
Char. 28: 0 → 1
Char. 33: 0 → 2
Char. 39: 0 → 1
Char. 52: 0 → 1
Char. 100: 0 → 1
Char. 111: 0 → 1
Char. 116: 1 → 0
Char. 117: 0 → 1
Char. 131: 0 → 1
Char. 156: 1 → 0
Char. 159: 0 → 1
Char. 167: 0 → 1
Char. 180: 0 → 1
Char. 189: 0 → 1
Char. 191: 0 → 1
Char. 192: 0 → 1
Char. 267: 0 → 1

**Diadectomorpha:**
Char. 0: 0 → 1
Char. 64: 0 → 1
Char. 70: 0 → 1
Char. 122: 0 → 1
Char. 123: 1 → 0
Char. 146: 1 → 0
Char. 275: 1 → 0
Char. 278: 3 → 0

***Emeroleter levis*:**
Char. 51: 1 → 0

**Eosauropterygia:**
Char. 159: 1 → 0
Char. 166: 1 → 0
Char. 194: 0 → 2
Char. 272: 1 → 0

***Eudibamus cursoris*:**
Char. 154: 1 → 2

***Feeserpeton oklahomensis*:**
Char. 51: 0 → 1
Char. 70: 0 → 1
Char. 157: 0 → 1
Char. 158: 0 → 1

***Hovasaurus boulei*:**
Char. 41: 0 → 1
Char. 55: 1 → 0
Char. 60: 1 → 0
Char. 72: 2 → 1
Char. 77: 0 → 2
Char. 78: 1 → 0
Char. 79: 0 → 1
Char. 93: 1 → 0
Char. 113: 0 → 1
Char. 138: 0 → 1
Char. 141: 1 → 0
Char. 146: 1 → 0
Char. 278: 0 → 3

**Kuehneosauridae:**
Char. 7: 0 → 1
Char. 21: 1 → 0
Char. 24: 0 → 1
Char. 26: 0 → 1
Char. 34: 0 → 1
Char. 36: 0 → 1
Char. 43: 1 → 0

Char. 56: 0 → 1
Char. 79: 0 → 2
Char. 82: 0 → 1
Char. 98: 1 → 0
Char. 108: 1 → 0
Char. 113: 0 → 1
Char. 128: 0 → 1
Char. 147: 1 → 0
Char. 148: 1 → 0
Char. 159: 1 → 0
Char. 185: 0 → 1
Char. 245: 0 → 1
Char. 272: 1 → 2
Char. 278: 0 → 3

***Lanthanosuchus watsoni*:**
Char. 25: 1 → 0
Char. 51: 0 → 1
Char. 76: 0 → 1
Char. 86: 0 → 1
Char. 95: 0 → 1
Char. 98: 1 → 0
Char. 110: 0 → 1
Char. 113: 0 → 1
Char. 114: 0 → 1
Char. 131: 0 → 1
Char. 137: 0 → 1
Char. 138: 0 → 1
Char. 140: 0 → 2
Char. 144: 1 → 0
Char. 154: 1 → 2
Char. 192: 0 → 1

***Macroleter poezicus*:**
Char. 52: 0 → 1
Char. 84: 1 → 0
Char. 87: 0 → 1
Char. 140: 0 → 1
Char. 146: 1 → 0
Char. 169: 1 → 0
Char. 235: 0 → 1

***Mesosaurus* spp.:**
Char. 0: 0 → 1
Char. 2: 0 → 1
Char. 5: 1 → 0
Char. 6: 0 → 1
Char. 8: 0 → 1
Char. 9: 0 → 1
Char. 13: 0 → 1
Char. 19: 0 → 1
Char. 23: 0 → 1
Char. 26: 0 → 1
Char. 29: 1 → 0
Char. 33: 0 → 1
Char. 38: 1 → 0
Char. 41: 0 → 1
Char. 50: 1 → 0
Char. 67: 0 → 1
Char. 76: 0 → 1
Char. 83: 0 → 1
Char. 84: 1 → 0
Char. 85: 1 → 0
Char. 94: 0 → 1
Char. 107: 0 → 1
Char. 109: 0 → 1
Char. 111: 0 → 1
Char. 115: 0 → 1
Char. 146: 1 → 0
Char. 148: 0 → 1
Char. 149: 1 → 0
Char. 164: 0 → 1
Char. 167: 0 → 1
Char. 176: 0 → 1
Char. 183: 1 → 0
Char. 184: 0 → 1
Char. 199: 0 → 1
Char. 202: 0 → 1
Char. 206: 0 → 1
Char. 207: 0 → 1
Char. 209: 0 → 1
Char. 217: 0 → 1
Char. 219: 0 → 1
Char. 220: 0 → 1
Char. 231: 0 → 1
Char. 260: 2 → 0
Char. 272: 2 → 0
Char. 278: 3 → 0

***Microleter mckinzieorum*:**
Char. 0: 0 → 1
Char. 25: 1 → 0
Char. 36: 0 → 1
Char. 39: 0 → 1
Char. 51: 0 → 2
Char. 56: 0 → 1
Char. 70: 0 → 1
Char. 94: 0 → 1
Char. 106: 0 → 1
Char. 159: 0 → 1
Char. 276: 0 → 1

**Millerettidae:**
Char. 5: 1 → 0
Char. 24: 0 → 1
Char. 25: 1 → 0
Char. 44: 1 → 0
Char. 56: 0 → 1
Char. 57: 1 → 0
Char. 66: 0 → 2
Char. 78: 1 → 0
Char. 80: 1 → 0
Char. 84: 1 → 2
Char. 88: 1 → 0
Char. 96: 1 → 0
Char. 117: 0 → 1
Char. 124: 0 → 1
Char. 127: 0 → 1
Char. 135: 0 → 1
Char. 145: 0 → 1
Char. 202: 0 → 1
Char. 211: 0 → 1
Char. 230: 0 → 1
Char. 234: 0 → 1
Char. 248: 0 → 1
Char. 252: 0 → 1

***Nycteroleter ineptus*:**
Char. 278: 0 → 3

***Nyctiphruretus acudens*:**
Char. 21: 0 → 1
Char. 33: 1 → 2
Char. 41: 0 → 1
Char. 81: 1 → 0
Char. 84: 1 → 2
Char. 94: 0 → 1
Char. 167: 0 → 1
Char. 215: 1 → 0
Char. 224: 0 → 1
Char. 266: 0 → 1
Char. 272: 2 → 1
Char. 276: 0 → 1

***Orovenator mayorum*:**
Char. 8: 0 → 1
Char. 24: 0 → 1
Char. 36: 0 → 1
Char. 160: 0 → 1
Char. 165: 0 → 1

***Owenetta* spp.:**
Char. 169: 1 → 0

***Paleothyris acadiana*:**
Char. 38: 1 → 0
Char. 50: 1 → 0
Char. 66: 1 → 2
Char. 102: 0 → 1
Char. 146: 1 → 0
Char. 180: 0 → 1
Char. 237: 0 → 1
Char. 239: 0 → 1

***Placodus* spp.:**
Char. 0: 1 → 2
Char. 9: 0 → 1
Char. 12: 0 → 1
Char. 13: 0 → 1
Char. 19: 0 → 1
Char. 26: 0 → 1
Char. 31: 0 → 1
Char. 46: 1 → 0
Char. 57: 0 → 1
Char. 78: 1 → 0
Char. 93: 1 → 0
Char. 102: 1 → 2
Char. 109: 1 → 0
Char. 140: 1 → 0
Char. 163: 1 → 0
Char. 164: 0 → 1

***Procolophon* spp.:**
Char. 41: 0 → 1
Char. 69: 0 → 1
Char. 79: 0 → 1
Char. 88: 0 → 1
Char. 117: 1 → 0
Char. 149: 1 → 0
Char. 180: 0 → 1
Char. 237: 0 → 1
Char. 238: 0 → 1
Char. 272: 2 → 1
Char. 278: 3 → 0

***Prolacerta broomi*:**
Char. 58: 1 → 0
Char. 66: 1 → 0
Char. 67: 1 → 0
Char. 80: 1 → 0
Char. 139: 1 → 0
Char. 147: 1 → 0
Char. 192: 1 → 0
Char. 203: 1 → 2

***Rhipaeosaurus* spp.:**
Char. 172: 0 → 1
Char. 277: 0 → 1

**Rhynchocephalia:**
Char. 0: 1 → 2
Char. 24: 0 → 1
Char. 77: 0 → 1
Char. 94: 1 → 0
Char. 139: 1 → 0
Char. 167: 1 → 0
Char. 205: 1 → 0

**Rhynchosauria:**
Char. 0: 1 → 0
Char. 7: 0 → 1
Char. 9: 0 → 1
Char. 26: 0 → 1
Char. 68: 0 → 1
Char. 99: 1 → 0
Char. 150: 1 → 0
Char. 160: 0 → 1
Char. 161: 0 → 1
Char. 171: 0 → 2
Char. 223: 0 → 1
Char. 224: 0 → 1
Char. 241: 0 → 1

***Scutosaurus* spp.:**
Char. 175: 0 → 1
Char. 218: 0 → 1
Char. 243: 0 → 2
Char. 244: 0 → 1

***Sinosaurosphargis yunguiensis*:**
Char. 8: 0 → 1
Char. 30: 0 → 1
Char. 53: 0 → 1
Char. 82: 0 → 1
Char. 89: 1 → 0
Char. 127: 1 → 0
Char. 150: 1 → 0
Char. 154: 0 → 2
Char. 167: 1 → 0
Char. 255: 0 → 1

**Squamata:**
Char. 26: 0 → 1
Char. 45: 0 → 1
Char. 79: 0 → 2
Char. 80: 1 → 0
Char. 82: 0 → 1
Char. 92: 1 → 0
Char. 109: 1 → 0
Char. 160: 0 → 1
Char. 245: 0 → 1

***Trilophosaurus buettneri*:**
Char. 5: 1 → 0
Char. 11: 0 → 1
Char. 44: 0 → 1
Char. 55: 1 → 0
Char. 93: 1 → 0
Char. 104: 0 → 1
Char. 106: 0 → 1
Char. 113: 0 → 1
Char. 122: 0 → 1
Char. 136: 1 → 0
Char. 144: 1 → 0
Char. 154: 0 → 1
Char. 157: 0 → 1
Char. 159: 1 → 0
Char. 177: 0 → 12
Char. 194: 0 → 1
Char. 203: 1 → 2
Char. 207: 1 → 0
Char. 208: 1 → 0
Char. 272: 1 → 0

***Youngina capensis*:**
Char. 5: 1 → 0
Char. 56: 0 → 1
Char. 75: 0 → 1
Char. 134: 0 → 1
Char. 170: 0 → 1
Char. 211: 0 → 1
Char. 214: 0 → 1
Char. 239: 0 → 1

**Node 49:**
Char. 46: 1 → 0
Char. 50: 0 → 1
Char. 88: 1 → 0
Char. 89: 1 → 0
Char. 93: 1 → 0
Char. 176: 0 → 1
Char. 195: 0 → 2
Char. 198: 0 → 1
Char. 240: 1 → 0
Char. 246: 1 → 2
Char. 259: 0 → 1
Char. 265: 1 → 0
Char. 270: 0 → 1

**Node 50:**
Char. 62: 1 → 0
Char. 65: 0 → 1
Char. 78: 1 → 0
Char. 184: 0 → 1

Char. 193: 1 → 0
Char. 194: 0 → 2
Char. 205: 0 → 1
Char. 210: 0 → 1
Char. 219: 1 → 0
Char. 241: 0 → 1
Char. 246: 0 → 1
Char. 254: 0 → 1
Char. 255: 0 → 1
Char. 256: 0 → 1
Char. 268: 0 → 1
Char. 269: 0 → 1

**Node 51:**
Char. 15: 0 → 1
Char. 23: 0 → 1
Char. 64: 0 → 1
Char. 73: 0 → 1
Char. 81: 1 → 0
Char. 94: 0 → 1
Char. 130: 0 → 1
Char. 131: 0 → 1
Char. 134: 0 → 2
Char. 145: 0 → 1
Char. 147: 0 → 1
Char. 148: 0 → 1
Char. 151: 0 → 1
Char. 203: 1 → 2
Char. 208: 0 → 1
Char. 217: 0 → 1
Char. 224: 1 → 0
Char. 272: 2 → 1

**Node 52:**
Char. 21: 0 → 1
Char. 33: 0 → 1
Char. 135: 0 → 1
Char. 159: 0 → 1
Char. 267: 0 → 1
Char. 278: 1 → 0

**Node 53:**
Char. 20: 0 → 1
Char. 62: 0 → 1
Char. 141: 0 → 1

**Node 54:**
Char. 0: 0 → 1
Char. 29: 1 → 0
Char. 40: 2 → 0
Char. 57: 1 → 0
Char. 59: 0 → 1
Char. 60: 0 → 1
Char. 67: 0 → 1
Char. 72: 1 → 2
Char. 89: 0 → 1
Char. 111: 0 → 1
Char. 112: 0 → 1
Char. 120: 0 → 1
Char. 180: 0 → 1
Char. 192: 0 → 1
Char. 193: 0 → 1
Char. 222: 0 → 1

Char. 224: 0 → 1
Char. 234: 0 → 1
Char. 237: 0 → 1
Char. 266: 0 → 1
Char. 278: 3 → 1

**Node 55:**
Char. 5: 0 → 1
Char. 29: 0 → 1
Char. 40: 0 → 2
Char. 74: 0 → 1
Char. 80: 0 → 1
Char. 84: 0 → 1
Char. 88: 0 → 1
Char. 116: 0 → 1
Char. 132: 0 → 1
Char. 149: 0 → 1
Char. 156: 0 → 1
Char. 169: 0 → 1
Char. 201: 0 → 1
Char. 203: 0 → 1
Char. 235: 0 → 1

**Node 56:**
Char. 72: 0 → 1
Char. 79: 1 → 0
Char. 81: 0 → 1
Char. 93: 0 → 1
Char. 97: 0 → 1
Char. 104: 1 → 0
Char. 144: 0 → 1
Char. 173: 0 → 1
Char. 183: 0 → 1

**Node 58:**
Char. 19: 0 → 1
Char. 21: 1 → 0
Char. 92: 1 → 0

**Node 59:**
Char. 4: 0 → 1
Char. 29: 0 → 1
Char. 48: 1 → 0
Char. 213: 0 → 1
Char. 226: 0 → 2
Char. 228: 0 → 1
Char. 275: 0 → 1

**Node 60:**
Char. 58: 0 → 1
Char. 61: 0 → 1
Char. 64: 1 → 0
Char. 66: 0 → 1
Char. 69: 0 → 1
Char. 130: 1 → 0
Char. 134: 2 → 1
Char. 150: 0 → 1
Char. 167: 0 → 1
Char. 176: 0 → 1
Char. 190: 0 → 1
Char. 203: 2 → 1
Char. 205: 0 → 1
Char. 214: 0 → 1

Char. 220: 1 → 0
Char. 230: 0 → 1
Char. 239: 0 → 1
Char. 260: 2 → 1

**Node 61:**
Char. 24: 0 → 1
Char. 57: 1 → 0
Char. 79: 0 → 1
Char. 83: 0 → 1
Char. 110: 0 → 1
Char. 132: 1 → 0

**Node 62:**
Char. 73: 0 → 1
Char. 131: 1 → 0
Char. 205: 0 → 1
Char. 276: 0 → 1

**Node 63:**
Char. 18: 0 → 1
Char. 37: 0 → 1
Char. 107: 0 → 1
Char. 118: 1 → 0
Char. 125: 1 → 0
Char. 150: 0 → 1

**Node 64:**
Char. 23: 0 → 1
Char. 71: 1 → 0
Char. 102: 0 → 1
Char. 103: 0 → 1
Char. 106: 0 → 1
Char. 167: 0 → 1
Char. 214: 0 → 1
Char. 216: 1 → 0
Char. 235: 0 → 2
Char. 241: 0 → 1

**Node 65:**
Char. 138: 0 → 1
Char. 141: 0 → 1
Char. 207: 0 → 1

**Node 66:**
Char. 20: 0 → 1
Char. 33: 0 → 1
Char. 44: 1 → 0
Char. 70: 0 → 1
Char. 80: 1 → 0
Char. 117: 0 → 1
Char. 118: 0 → 1
Char. 125: 0 → 1
Char. 131: 0 → 1
Char. 194: 0 → 1
Char. 201: 1 → 0
Char. 211: 0 → 1

**Node 67:**
Char. 38: 1 → 2
Char. 39: 0 → 1
Char. 49: 0 → 1
Char. 66: 0 → 12

Char. 76: 0 → 1
Char. 85: 1 → 0
Char. 88: 1 → 0
Char. 95: 0 → 1
Char. 112: 0 → 1
Char. 148: 0 → 1
Char. 158: 0 → 1
Char. 159: 0 → 1
Char. 183: 1 → 2
Char. 192: 0 → 1
Char. 215: 0 → 1

**Node 68:**
Char. 87: 0 → 1

**Node 69:**
Char. 25: 1 → 0
Char. 76: 1 → 0

**Node 70:**
Char. 79: 0 → 1
Char. 93: 1 → 0
Char. 133: 0 → 1

**Node 71:**
Char. 85: 0 → 1
Char. 147: 1 → 2
Char. 154: 1 → 0
Char. 278: 3 → 0

**Node 72:**
Char. 50: 1 → 0
Char. 58: 0 → 1
Char. 59: 0 → 1
Char. 60: 0 → 1
Char. 72: 1 → 2
Char. 104: 0 → 1
Char. 105: 0 → 1
Char. 106: 0 → 2
Char. 107: 0 → 1
Char. 109: 0 → 1
Char. 110: 0 → 1
Char. 146: 1 → 0
Char. 155: 0 → 2

**Node 73:**
Char. 33: 1 → 0
Char. 38: 2 → 1
Char. 39: 1 → 0
Char. 42: 0 → 1
Char. 43: 0 → 1
Char. 46: 1 → 0
Char. 49: 1 → 0
Char. 52: 0 → 1
Char. 83: 01 → 2
Char. 84: 1 → 0
Char. 87: 0 → 1
Char. 93: 1 → 0
Char. 161: 0 → 1
Char. 163: 1 → 0
Char. 172: 0 → 2
Char. 188: 0 → 1
Char. 189: 0 → 1

Char. 195: 0 → 1
Char. 212: 0 → 1
Char. 236: 0 → 1
Char. 238: 0 → 2
Char. 242: 0 → 1
Char. 274: 0 → 1
Char. 275: 1 → 0

**Node 74:**
Char. 5: 1 → 0
Char. 29: 1 → 0
Char. 44: 1 → 0
Char. 59: 0 → 1
Char. 60: 0 → 1
Char. 66: 0 → 1
Char. 67: 0 → 1
Char. 72: 1 → 2
Char. 78: 1 → 0
Char. 84: 1 → 0
Char. 88: 1 → 0
Char. 93: 1 → 0
Char. 111: 0 → 1
Char. 123: 1 → 0
Char. 129: 0 → 1
Char. 154: 1 → 0
Char. 169: 1 → 0
Char. 170: 0 → 1
Char. 197: 0 → 1

**Node 75:**
Char. 20: 0 → 1
Char. 47: 0 → 1
Char. 83: 0 → 1
Char. 85: 1 → 0
Char. 107: 0 → 1
Char. 110: 0 → 1
Char. 169: 1 → 0
Char. 170: 0 → 1

**Node 76:**
Char. 2: 0 → 1
Char. 6: 0 → 1
Char. 98: 1 → 0
Char. 101: 0 → 1
Char. 113: 0 → 1
Char. 186: 0 → 1
Char. 193: 1 → 0
Char. 198: 0 → 1
Char. 220: 0 → 1
Char. 231: 1 → 0
Char. 234: 1 → 0
Char. 239: 1 → 0
Char. 240: 1 → 0
Char. 266: 1 → 0

**Node 77:**
Char. 33: 1 → 2
Char. 68: 0 → 1
Char. 102: 0 → 1
Char. 103: 0 → 1
Char. 223: 0 → 1
Char. 229: 0 → 1
Char. 235: 1 → 2

**Node 78:**
Char. 17: 0 → 1
Char. 42: 0 → 1
Char. 264: 0 → 1

**Node 79:**
Char. 41: 0 → 1
Char. 48: 1 → 0
Char. 61: 1 → 2
Char. 112: 1 → 0
Char. 128: 0 → 1
Char. 138: 0 → 1
Char. 146: 1 → 0
Char. 192: 1 → 2
Char. 224: 0 → 1
Char. 226: 0 → 1
Char. 227: 0 → 1
Char. 233: 0 → 1

**Node 80:**
Char. 61: 1 → 3
Char. 90: 0 → 1
Char. 91: 0 → 1
Char. 109: 1 → 0
Char. 209: 1 → 0

**ANALYSIS 88**
**(NO *CANDELARIA BARBOURI*, IMPLIED WEIGHTING, K = 2.75, NO CHARACTERS 174, 247, 251, AND 253)**

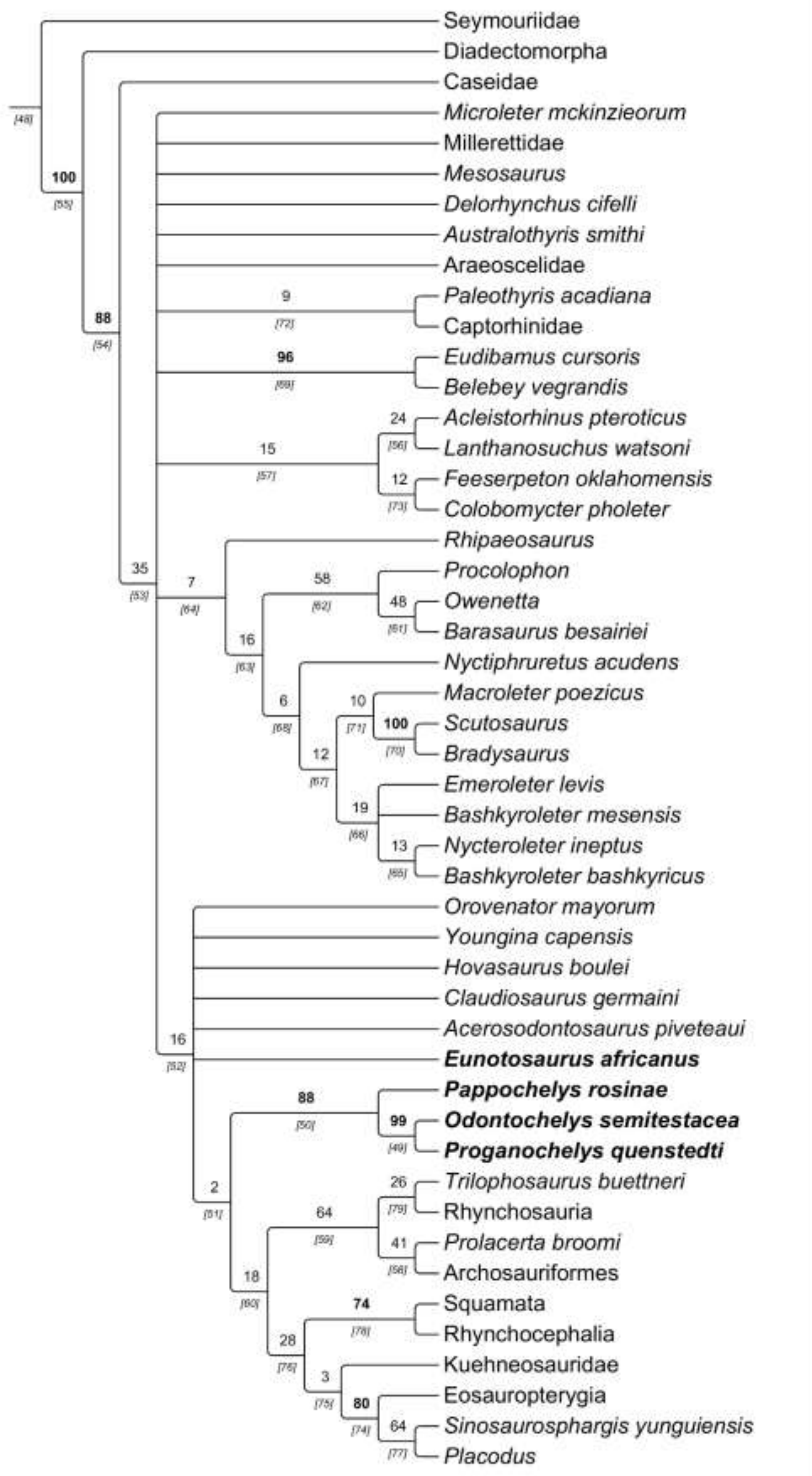

***Proganochelys quenstedti*:**
Char. 8: 0 → 1
Char. 11: 0 → 1
Char. 106: 0 → 1
Char. 108: 1 → 0
Char. 128: 0 → 1
Char. 175: 0 → 1
Char. 202: 1 → 0
Char. 207: 1 → 0
Char. 209: 1 → 0
Char. 244: 0 → 1
Char. 248: 0 → 1
Char. 250: 0 → 1
Char. 262: 0 → 1

***Pappochelys rosinae*:**
Char. 1: 0 → 1
Char. 5: 1 → 0
Char. 12: 0 → 1
Char. 41: 0 → 1
Char. 49: 0 → 1
Char. 55: 1 → 0
Char. 67: 1 → 0
Char. 169: 1 → 0
Char. 260: 2 → 0

***Odontochelys semitestacea*:**
Char. 109: 1 → 0
Char. 112: 1 → 0
Char. 124: 0 → 1
Char. 126: 1 → 0

***Eunotosaurus africanus*:**
Char. 0: 1 → 2
Char. 15: 0 → 1
Char. 19: 0 → 1
Char. 24: 0 → 1
Char. 33: 1 → 2
Char. 42: 0 → 1
Char. 50: 0 → 1
Char. 55: 1 → 0
Char. 57: 0 → 1
Char. 59: 1 → 0
Char. 62: 1 → 0
Char. 67: 1 → 0
Char. 72: 2 → 1
Char. 76: 0 → 1
Char. 81: 1 → 0
Char. 84: 1 → 2
Char. 97: 1 → 0
Char. 102: 0 → 1
Char. 103: 0 → 1
Char. 109: 1 → 0
Char. 112: 1 → 0
Char. 124: 0 → 1
Char. 129: 1 → 0
Char. 130: 0 → 1
Char. 134: 0 → 2
Char. 147: 0 → 1
Char. 148: 0 → 1
Char. 152: 0 → 1
Char. 155: 0 → 1
Char. 158: 0 → 1
Char. 161: 0 → 1
Char. 181: 0 → 1
Char. 191: 1 → 0
Char. 192: 1 → 0
Char. 194: 0 → 1
Char. 202: 1 → 0
Char. 211: 0 → 1
Char. 237: 1 → 0
Char. 240: 1 → 0
Char. 248: 0 → 1
Char. 249: 0 → 2
Char. 250: 0 → 1
Char. 252: 0 → 1
Char. 263: 0 → 1
Char. 273: 0 → 1
Char. 274: 0 → 1
Char. 275: 0 → 1
Char. 276: 0 → 1
Char. 277: 0 → 1

***Acerosodontosaurus piveteaui*:**
Char. 23: 0 → 1
Char. 78: 1 → 0
Char. 81: 1 → 0
Char. 127: 1 → 0
Char. 128: 0 → 1
Char. 155: 0 → 1
Char. 206: 0 → 2
Char. 208: 0 → 1
Char. 278: 03 → 1

***Acleistorhinus pteroticus:***
Char. 21: 0 → 1
Char. 146: 1 → 0

**Araeoscelidae:**
Char. 0: 0 → 1
Char. 23: 0 → 1
Char. 27: 0 → 1
Char. 28: 0 → 1
Char. 29: 1 → 0
Char. 38: 1 → 0
Char. 40: 2 → 0
Char. 43: 0 → 1
Char. 50: 1 → 0
Char. 57: 1 → 0
Char. 59: 0 → 1
Char. 60: 0 → 1
Char. 67: 0 → 1
Char. 72: 1 → 2
Char. 84: 1 → 0
Char. 89: 0 → 1
Char. 106: 0 → 1
Char. 111: 0 → 1
Char. 112: 0 → 1
Char. 116: 1 → 0
Char. 117: 01 → 2
Char. 120: 0 → 1
Char. 154: 1 → 0
Char. 169: 1 → 0
Char. 170: 0 → 1
Char. 193: 0 → 1
Char. 197: 0 → 1
Char. 222: 0 → 1
Char. 224: 0 → 1
Char. 237: 0 → 1
Char. 239: 0 → 1
Char. 266: 0 → 1
Char. 278: 3 → 1

**Archosauriformes:**
Char. 32: 0 → 1
Char. 94: 1 → 0
Char. 112: 1 → 0
Char. 152: 0 → 1
Char. 154: 01 → 2
Char. 166: 1 → 0
Char. 171: 0 → 1
Char. 185: 0 → 1
Char. 201: 0 → 1
Char. 204: 0 → 1
Char. 218: 0 → 3
Char. 242: 0 → 1

***Australothyris smithi*:**
Char. 23: 0 → 1
Char. 24: 0 → 1
Char. 34: 0 → 1
Char. 55: 1 → 0
Char. 57: 1 → 0
Char. 71: 1 → 0
Char. 79: 0 → 1
Char. 83: 0 → 1
Char. 85: 1 → 0
Char. 98: 1 → 0
Char. 100: 0 → 1
Char. 103: 0 → 1
Char. 110: 0 → 1
Char. 112: 0 → 1
Char. 123: 1 → 0
Char. 129: 0 → 1
Char. 131: 0 → 1
Char. 132: 1 → 0
Char. 144: 1 → 0
Char. 147: 0 → 1
Char. 149: 1 → 0
Char. 150: 0 → 1
Char. 192: 1 → 0

***Barasaurus besairiei*:**
Char. 33: 1 → 0

***Bashkyroleter bashkyricus*:**
Char. 275: 1 → 0

***Bashkyroleter mesensis*:**
Char. 12: 0 → 1
Char. 66: 1 → 0
Char. 169: 1 → 0

***Belebey vegrandis*:**
Char. 154: 1 → 0

***Bradysaurus* spp.:**
Char. 19: 0 → 1
Char. 25: 1 → 0
Char. 73: 0 → 1
Char. 79: 0 → 1
Char. 135: 1 → 0
Char. 249: 0 → 1

**Captorhinidae:**
Char. 3: 0 → 1
Char. 23: 0 → 1
Char. 25: 1 → 0
Char. 26: 0 → 1
Char. 73: 0 → 1
Char. 75: 0 → 1
Char. 83: 0 → 1
Char. 108: 1 → 0
Char. 180: 1 → 0
Char. 183: 1 → 0
Char. 201: 1 → 0
Char. 203: 1 → 2
Char. 216: 1 → 0
Char. 240: 1 → 0

**Caseidae:**
Char. 24: 0 → 1
Char. 25: 1 → 0
Char. 36: 0 → 1
Char. 38: 1 → 0
Char. 46: 1 → 0
Char. 50: 1 → 0
Char. 56: 0 → 1
Char. 170: 0 → 1
Char. 194: 0 → 1
Char. 273: 0 → 1
Char. 274: 0 → 1
Char. 278: 3 → 2

***Claudiosaurus germaini*:**
Char. 23: 0 → 1
Char. 24: 0 → 1
Char. 27: 0 → 1
Char. 34: 0 → 1
Char. 36: 0 → 1
Char. 56: 0 → 1
Char. 70: 0 → 1
Char. 84: 1 → 0
Char. 105: 0 → 1
Char. 106: 0 → 1
Char. 117: 01 → 2
Char. 126: 0 → 1
Char. 127: 1 → 0
Char. 130: 0 → 1
Char. 131: 0 → 1
Char. 141: 1 → 0
Char. 144: 1 → 0
Char. 148: 0 → 1
Char. 154: 1 → 0
Char. 166: 1 → 0
Char. 182: 0 → 1
Char. 187: 1 → 0
Char. 190: 0 → 1
Char. 199: 0 → 1
Char. 201: 0 → 1
Char. 204: 02 → 1
Char. 214: 0 → 1
Char. 234: 1 → 0
Char. 267: 0 → 1
Char. 272: 2 → 1

***Colobomycter pholeter*:**
Char. 21: 0 → 1
Char. 25: 1 → 0
Char. 84: 1 → 0
Char. 154: 1 → 0
Char. 167: 0 → 1
Char. 267: 0 → 1

***Delorhynchus cifelli*:**
Char. 18: 0 → 1
Char. 20: 0 → 1
Char. 21: 0 → 1
Char. 24: 0 → 1
Char. 26: 0 → 1
Char. 28: 0 → 1
Char. 33: 0 → 2
Char. 39: 0 → 1
Char. 52: 0 → 1
Char. 100: 0 → 1
Char. 111: 0 → 1
Char. 116: 1 → 0
Char. 119: 1 → 0
Char. 131: 0 → 1
Char. 147: 0 → 1
Char. 156: 1 → 0
Char. 167: 0 → 1
Char. 189: 0 → 1
Char. 191: 0 → 1
Char. 267: 0 → 1

**Diadectomorpha:**
Char. 0: 0 → 1
Char. 64: 0 → 1
Char. 70: 0 → 1
Char. 122: 0 → 1
Char. 123: 1 → 0
Char. 146: 1 → 0
Char. 275: 1 → 0
Char. 278: 3 → 0

***Emeroleter levis*:**
Char. 51: 1 → 0

**Eosauropterygia:**
Char. 166: 1 → 0
Char. 178: 0 → 1
Char. 194: 0 → 2
Char. 272: 1 → 0

***Eudibamus cursoris*:**
Char. 154: 1 → 2

***Feeserpeton oklahomensis*:**
Char. 51: 0 → 1
Char. 157: 0 → 1
Char. 158: 0 → 1

***Hovasaurus boulei*:**
Char. 41: 0 → 1
Char. 55: 1 → 0
Char. 72: 2 → 1
Char. 77: 0 → 2
Char. 78: 1 → 0
Char. 79: 0 → 1
Char. 93: 1 → 0
Char. 113: 0 → 1
Char. 138: 0 → 1
Char. 141: 1 → 0
Char. 146: 1 → 0
Char. 206: 0 → 2
Char. 215: 0 → 1
Char. 219: 1 → 0
Char. 224: 0 → 1

**Kuehneosauridae:**
Char. 7: 0 → 1
Char. 21: 1 → 0
Char. 24: 0 → 1
Char. 27: 0 → 1
Char. 43: 1 → 0
Char. 79: 0 → 2
Char. 107: 1 → 0
Char. 140: 1 → 0
Char. 147: 1 → 0
Char. 148: 1 → 0
Char. 245: 0 → 1
Char. 272: 1 → 2
Char. 278: 0 → 3

***Lanthanosuchus watsoni*:**
Char. 25: 1 → 0
Char. 51: 0 → 1
Char. 76: 0 → 1
Char. 86: 0 → 1
Char. 98: 1 → 0
Char. 138: 0 → 1
Char. 144: 1 → 0
Char. 154: 1 → 2

***Macroleter poezicus*:**
Char. 9: 0 → 1
Char. 26: 0 → 1
Char. 66: 1 → 2
Char. 126: 0 → 1
Char. 134: 0 → 2
Char. 140: 0 → 1
Char. 146: 1 → 0
Char. 169: 1 → 0

***Mesosaurus* spp.:**
Char. 0: 0 → 1
Char. 2: 0 → 1
Char. 6: 0 → 1
Char. 8: 0 → 1
Char. 9: 0 → 1
Char. 13: 0 → 1
Char. 19: 0 → 1
Char. 23: 0 → 1
Char. 26: 0 → 1
Char. 29: 1 → 0
Char. 33: 0 → 1
Char. 38: 1 → 0
Char. 41: 0 → 1
Char. 50: 1 → 0
Char. 67: 0 → 1
Char. 76: 0 → 1
Char. 83: 0 → 1
Char. 84: 1 → 0
Char. 85: 1 → 0
Char. 94: 0 → 1
Char. 107: 0 → 1
Char. 109: 0 → 1
Char. 111: 0 → 1
Char. 115: 0 → 1
Char. 146: 1 → 0
Char. 148: 0 → 1
Char. 149: 1 → 0
Char. 164: 0 → 1
Char. 167: 0 → 1
Char. 176: 0 → 1
Char. 183: 1 → 0
Char. 184: 0 → 1
Char. 192: 1 → 0
Char. 199: 0 → 1
Char. 202: 0 → 1
Char. 206: 0 → 1
Char. 207: 0 → 1
Char. 209: 0 → 1
Char. 217: 0 → 1
Char. 219: 0 → 1
Char. 220: 0 → 1
Char. 231: 0 → 1
Char. 240: 1 → 0
Char. 260: 2 → 0
Char. 272: 2 → 0
Char. 278: 3 → 0

***Microleter mckinzieorum*:**
Char. 0: 0 → 1
Char. 18: 0 → 1
Char. 24: 0 → 1
Char. 25: 1 → 0
Char. 36: 0 → 1
Char. 39: 0 → 1
Char. 51: 0 → 2
Char. 56: 0 → 1
Char. 57: 1 → 0
Char. 70: 0 → 1
Char. 76: 0 → 1
Char. 79: 0 → 1
Char. 83: 0 → 1
Char. 94: 0 → 1
Char. 106: 0 → 1
Char. 110: 0 → 1
Char. 132: 1 → 0
Char. 276: 0 → 1
Char. 278: 3 → 1

**Millerettidae:**
Char. 24: 0 → 1
Char. 25: 1 → 0
Char. 44: 1 → 0
Char. 56: 0 → 1
Char. 57: 1 → 0
Char. 66: 0 → 2
Char. 78: 1 → 0
Char. 80: 1 → 0
Char. 84: 1 → 2
Char. 88: 1 → 0
Char. 96: 1 → 0
Char. 121: 0 → 1
Char. 124: 0 → 1
Char. 127: 0 → 1
Char. 135: 0 → 1
Char. 145: 0 → 1
Char. 180: 1 → 0
Char. 192: 1 → 0
Char. 202: 0 → 1
Char. 211: 0 → 1
Char. 230: 0 → 1
Char. 248: 0 → 1
Char. 252: 0 → 1

***Nycteroleter ineptus*:**
Char. 66: 1 → 0
Char. 278: 0 → 3

***Nyctiphruretus acudens*:**
Char. 21: 0 → 1
Char. 41: 0 → 1
Char. 66: 1 → 2
Char. 81: 1 → 0
Char. 83: 0 → 1
Char. 84: 1 → 2
Char. 94: 0 → 1
Char. 224: 0 → 1
Char. 226: 1 → 0
Char. 266: 0 → 1
Char. 272: 2 → 1
Char. 276: 0 → 1

***Orovenator mayorum*:**
Char. 8: 0 → 1
Char. 24: 0 → 1
Char. 33: 1 → 0
Char. 36: 0 → 1
Char. 50: 0 → 1
Char. 92: 1 → 0
Char. 94: 1 → 0
Char. 135: 1 → 0
Char. 159: 1 → 0
Char. 160: 0 → 1
Char. 165: 0 → 1
Char. 278: 03 → 1

***Owenetta* spp.:**
Char. 142: 1 → 0
Char. 169: 1 → 0

***Paleothyris acadiana*:**
Char. 38: 1 → 0
Char. 50: 1 → 0
Char. 66: 1 → 2
Char. 102: 0 → 1
Char. 146: 1 → 0
Char. 237: 0 → 1
Char. 239: 0 → 1

***Placodus* spp.:**
Char. 0: 1 → 2
Char. 9: 0 → 1
Char. 12: 0 → 1
Char. 13: 0 → 1
Char. 19: 0 → 1
Char. 31: 0 → 1
Char. 46: 1 → 0
Char. 57: 0 → 1
Char. 78: 1 → 0
Char. 93: 1 → 0
Char. 102: 1 → 2
Char. 109: 1 → 0
Char. 140: 1 → 0
Char. 155: 0 → 1
Char. 163: 1 → 0
Char. 164: 0 → 1

***Procolophon* spp.:**
Char. 41: 0 → 1
Char. 69: 0 → 1
Char. 79: 0 → 1
Char. 83: 0 → 1
Char. 86: 0 → 1
Char. 101: 0 → 1
Char. 141: 0 → 1
Char. 149: 1 → 0
Char. 203: 1 → 2
Char. 230: 0 → 1
Char. 237: 0 → 1
Char. 238: 0 → 1
Char. 272: 2 → 1
Char. 278: 3 → 0

***Prolacerta broomi*:**
Char. 58: 1 → 0
Char. 66: 1 → 0
Char. 67: 1 → 0
Char. 80: 1 → 0
Char. 139: 1 → 0
Char. 147: 1 → 0
Char. 192: 1 → 0
Char. 206: 0 → 12

***Rhipaeosaurus* spp.:**
Char. 172: 0 → 1
Char. 277: 0 → 1

**Rhynchocephalia:**
Char. 0: 1 → 2
Char. 24: 0 → 1
Char. 77: 0 → 1
Char. 88: 1 → 0
Char. 94: 1 → 0
Char. 139: 1 → 0
Char. 167: 1 → 0
Char. 205: 1 → 0

**Rhynchosauria:**
Char. 0: 1 → 0
Char. 7: 0 → 1
Char. 9: 0 → 1
Char. 63: 1 → 0
Char. 68: 0 → 1
Char. 99: 1 → 0
Char. 150: 1 → 0
Char. 160: 0 → 1
Char. 161: 0 → 1
Char. 171: 0 → 2
Char. 241: 0 → 1

***Scutosaurus* spp.:**
Char. 175: 0 → 1
Char. 190: 0 → 1
Char. 218: 0 → 1
Char. 243: 0 → 2
Char. 244: 0 → 1

***Sinosaurosphargis yunguiensis*:**
Char. 8: 0 → 1
Char. 30: 0 → 1
Char. 53: 0 → 1
Char. 89: 1 → 0
Char. 127: 1 → 0
Char. 150: 1 → 0
Char. 154: 0 → 2
Char. 167: 1 → 0
Char. 255: 0 → 1

**Squamata:**
Char. 45: 0 → 1
Char. 79: 0 → 2
Char. 80: 1 → 0
Char. 92: 1 → 0
Char. 109: 1 → 0
Char. 160: 0 → 1
Char. 200: 0 → 1
Char. 245: 0 → 1

***Trilophosaurus buettneri*:**
Char. 5: 1 → 0
Char. 11: 0 → 1
Char. 55: 1 → 0
Char. 93: 1 → 0
Char. 104: 0 → 1
Char. 106: 0 → 1
Char. 113: 0 → 1
Char. 122: 0 → 1
Char. 136: 1 → 0
Char. 144: 1 → 0
Char. 157: 0 → 1
Char. 159: 1 → 0
Char. 177: 0 → 12
Char. 194: 0 → 1
Char. 207: 1 → 0
Char. 208: 1 → 0
Char. 272: 1 → 0

***Youngina capensis*:**
Char. 5: 1 → 0
Char. 25: 0 → 1
Char. 27: 0 → 1

Char. 38: 1 → 0
Char. 56: 0 → 1
Char. 75: 0 → 1
Char. 84: 1 → 0
Char. 92: 1 → 0
Char. 94: 1 → 0
Char. 134: 0 → 1
Char. 154: 1 → 0
Char. 163: 1 → 0
Char. 170: 0 → 1
Char. 211: 0 → 1
Char. 214: 0 → 1
Char. 215: 0 → 1
Char. 220: 1 → 0
Char. 224: 0 → 1
Char. 239: 0 → 1
Char. 267: 0 → 1

**Node 49:**
Char. 46: 1 → 0
Char. 50: 0 → 1
Char. 88: 1 → 0
Char. 89: 1 → 0
Char. 93: 1 → 0
Char. 195: 0 → 2
Char. 198: 0 → 1
Char. 240: 1 → 0
Char. 246: 1 → 2
Char. 259: 0 → 1
Char. 265: 1 → 0
Char. 270: 0 → 1

**Node 50:**
Char. 62: 1 → 0
Char. 78: 1 → 0
Char. 193: 1 → 0
Char. 194: 0 → 2
Char. 219: 1 → 0
Char. 241: 0 → 1
Char. 246: 0 → 1
Char. 254: 0 → 1
Char. 255: 0 → 1
Char. 256: 0 → 1
Char. 268: 0 → 1
Char. 269: 0 → 1

**Node 51:**
Char. 63: 0 → 1
Char. 69: 0 → 1
Char. 126: 0 → 1
Char. 131: 0 → 1
Char. 147: 0 → 1
Char. 148: 0 → 1
Char. 205: 0 → 1
Char. 208: 0 → 1
Char. 230: 0 → 1
Char. 232: 0 → 1
Char. 267: 0 → 1
Char. 272: 2 → 1

**Node 52:**
Char. 0: 0 → 1
Char. 20: 0 → 1

Char. 25: 1 → 0
Char. 29: 1 → 0
Char. 33: 0 → 1
Char. 40: 2 → 0
Char. 50: 1 → 0
Char. 57: 1 → 0
Char. 59: 0 → 1
Char. 62: 0 → 1
Char. 67: 0 → 1
Char. 72: 1 → 2
Char. 89: 0 → 1
Char. 94: 0 → 1
Char. 109: 0 → 1
Char. 111: 0 → 1
Char. 112: 0 → 1
Char. 120: 0 → 1
Char. 127: 0 → 1
Char. 129: 0 → 1
Char. 135: 0 → 1
Char. 136: 0 → 1
Char. 139: 0 → 1
Char. 141: 0 → 1
Char. 179: 0 → 1
Char. 187: 0 → 1
Char. 188: 0 → 1
Char. 191: 0 → 1
Char. 193: 0 → 1
Char. 196: 0 → 1
Char. 201: 1 → 0
Char. 202: 0 → 1
Char. 207: 0 → 1
Char. 209: 0 → 1
Char. 219: 0 → 1
Char. 220: 0 → 1
Char. 237: 0 → 1
Char. 265: 0 → 1
Char. 266: 0 → 1
Char. 275: 1 → 0

**Node 53:**
Char. 29: 0 → 1
Char. 40: 0 → 2
Char. 74: 0 → 1
Char. 80: 0 → 1
Char. 84: 0 → 1
Char. 88: 0 → 1
Char. 116: 0 → 1
Char. 119: 0 → 1
Char. 132: 0 → 1
Char. 149: 0 → 1
Char. 156: 0 → 1
Char. 169: 0 → 1
Char. 180: 0 → 1
Char. 192: 0 → 1
Char. 201: 0 → 1
Char. 203: 0 → 1
Char. 235: 0 → 1

**Node 54:**
Char. 72: 0 → 1
Char. 79: 1 → 0
Char. 81: 0 → 1
Char. 93: 0 → 1

Char. 97: 0 → 1
Char. 104: 1 → 0
Char. 144: 0 → 1
Char. 173: 0 → 1
Char. 183: 0 → 1

**Node 56:**
Char. 95: 0 → 1
Char. 113: 0 → 1
Char. 114: 0 → 1

**Node 57:**
Char. 20: 0 → 1
Char. 47: 0 → 1
Char. 79: 0 → 1
Char. 110: 0 → 1
Char. 131: 0 → 1
Char. 137: 0 → 1
Char. 140: 0 → 2
Char. 147: 0 → 1

**Node 58:**
Char. 19: 0 → 1
Char. 21: 1 → 0
Char. 92: 1 → 0

**Node 59:**
Char. 4: 0 → 1
Char. 29: 0 → 1
Char. 213: 0 → 1
Char. 226: 01 → 2
Char. 228: 0 → 1
Char. 275: 0 → 1

**Node 60:**
Char. 58: 0 → 1
Char. 61: 0 → 1
Char. 107: 0 → 1
Char. 140: 0 → 1
Char. 150: 0 → 1
Char. 167: 0 → 1
Char. 190: 0 → 1
Char. 214: 0 → 1
Char. 220: 1 → 0
Char. 239: 0 → 1
Char. 260: 2 → 1

**Node 61:**
Char. 73: 0 → 1
Char. 205: 0 → 1
Char. 273: 0 → 1
Char. 276: 0 → 1

**Node 62:**
Char. 18: 0 → 1
Char. 23: 0 → 1
Char. 25: 1 → 0
Char. 37: 0 → 1
Char. 103: 0 → 1
Char. 106: 0 → 1
Char. 107: 0 → 1
Char. 110: 0 → 1

Char. 126: 0 → 1
Char. 150: 0 → 1

**Node 63:**
Char. 186: 0 → 1

**Node 64:**
Char. 148: 0 → 1
Char. 183: 1 → 2
Char. 194: 0 → 1
Char. 201: 1 → 0
Char. 211: 0 → 1
Char. 226: 0 → 1
Char. 240: 1 → 0
Char. 252: 0 → 1

**Node 65:**
Char. 87: 0 → 1
Char. 110: 0 → 1

**Node 66:**
Char. 79: 0 → 1
Char. 133: 0 → 1

**Node 67:**
Char. 100: 0 → 1
Char. 113: 0 → 1

**Node 68:**
Char. 70: 0 → 1
Char. 118: 0 → 1
Char. 125: 0 → 1

**Node 69:**
Char. 38: 1 → 2
Char. 39: 0 → 1
Char. 50: 1 → 0
Char. 58: 0 → 1
Char. 59: 0 → 1
Char. 60: 0 → 1
Char. 72: 1 → 2
Char. 83: 0 → 1
Char. 85: 1 → 0
Char. 88: 1 → 0
Char. 95: 0 → 1
Char. 104: 0 → 1
Char. 105: 0 → 1
Char. 106: 0 → 2
Char. 107: 0 → 1
Char. 109: 0 → 1
Char. 110: 0 → 1
Char. 146: 1 → 0
Char. 148: 0 → 1
Char. 155: 0 → 2
Char. 183: 1 → 2

**Node 70:**
Char. 23: 0 → 1
Char. 33: 1 → 0
Char. 38: 2 → 1
Char. 39: 1 → 0
Char. 42: 0 → 1

Char. 43: 0 → 1
Char. 46: 1 → 0
Char. 49: 1 → 0
Char. 71: 1 → 0
Char. 83: 0 → 2
Char. 101: 0 → 1
Char. 103: 0 → 1
Char. 106: 0 → 1
Char. 161: 0 → 1
Char. 163: 1 → 0
Char. 172: 0 → 2
Char. 188: 0 → 1
Char. 203: 1 → 2
Char. 212: 0 → 1
Char. 216: 1 → 0
Char. 236: 0 → 1
Char. 238: 0 → 2
Char. 241: 0 → 1
Char. 242: 0 → 1
Char. 273: 0 → 1
Char. 274: 0 → 1
Char. 275: 1 → 0

**Node 71:**
Char. 52: 0 → 1
Char. 75: 0 → 1
Char. 84: 1 → 0
Char. 87: 0 → 1
Char. 110: 0 → 1
Char. 121: 0 → 1
Char. 180: 1 → 0
Char. 230: 0 → 1

**Node 72:**
Char. 29: 1 → 0
Char. 44: 1 → 0
Char. 59: 0 → 1
Char. 60: 0 → 1
Char. 66: 0 → 1
Char. 67: 0 → 1
Char. 72: 1 → 2
Char. 78: 1 → 0
Char. 84: 1 → 0
Char. 88: 1 → 0
Char. 93: 1 → 0
Char. 111: 0 → 1
Char. 123: 1 → 0
Char. 129: 0 → 1
Char. 154: 1 → 0
Char. 169: 1 → 0
Char. 170: 0 → 1
Char. 192: 1 → 0
Char. 197: 0 → 1

**Node 73:**
Char. 83: 0 → 1
Char. 85: 1 → 0
Char. 107: 0 → 1

**Node 74:**
Char. 2: 0 → 1
Char. 6: 0 → 1
Char. 186: 0 → 1

Char. 198: 0 → 1
Char. 200: 0 → 1
Char. 220: 0 → 1
Char. 239: 1 → 0
Char. 267: 1 → 0

**Node 75:**
Char. 98: 1 → 0
Char. 113: 0 → 1
Char. 181: 0 → 1
Char. 206: 0 → 2

**Node 76:**
Char. 35: 0 → 1
Char. 102: 0 → 1
Char. 103: 0 → 1
Char. 201: 0 → 1
Char. 229: 0 → 1

**Node 77:**
Char. 17: 0 → 1
Char. 42: 0 → 1
Char. 264: 0 → 1

**Node 78:**
Char. 41: 0 → 1
Char. 61: 1 → 2
Char. 112: 1 → 0
Char. 138: 0 → 1
Char. 146: 1 → 0
Char. 192: 1 → 2
Char. 227: 0 → 1
Char. 233: 0 → 1
Char. 267: 1 → 2

**Node 79:**
Char. 61: 1 → 3
Char. 90: 0 → 1
Char. 91: 0 → 1
Char. 109: 1 → 0
Char. 209: 1 → 0

**ANALYSIS 89**
**(NO *CANDELARIA BARBOURI*, NO CHARACTERS 174, 204, 247, 251, AND 253)**

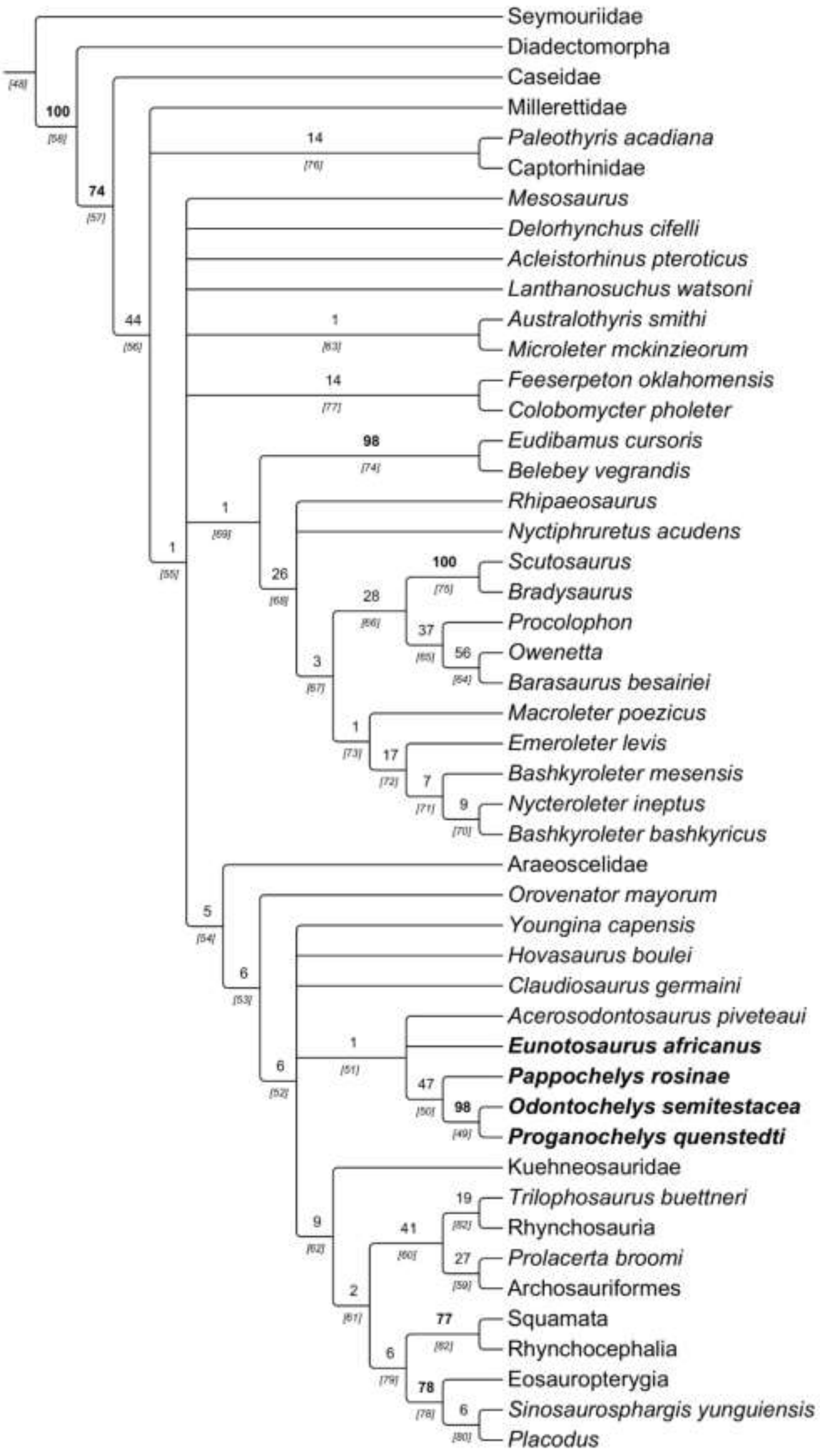

***Proganochelys quenstedti*:**
Char. 8: 0 → 1
Char. 11: 0 → 1
Char. 106: 0 → 1
Char. 108: 1 → 0
Char. 128: 0 → 1
Char. 175: 0 → 1
Char. 202: 1 → 0
Char. 206: 2 → 0
Char. 207: 1 → 0
Char. 209: 1 → 0
Char. 244: 0 → 1
Char. 248: 0 → 1
Char. 250: 0 → 1
Char. 252: 1 → 0
Char. 262: 0 → 1

***Pappochelys rosinae*:**
Char. 0: 2 → 0
Char. 1: 0 → 1
Char. 5: 1 → 0
Char. 12: 0 → 1
Char. 41: 0 → 1
Char. 49: 0 → 1
Char. 75: 0 → 1
Char. 81: 0 → 1
Char. 169: 1 → 0
Char. 260: 2 → 0

***Eunotosaurus africanus*:**
Char. 19: 0 → 1
Char. 42: 0 → 1
Char. 50: 0 → 1
Char. 57: 0 → 1
Char. 59: 1 → 0
Char. 60: 1 → 0
Char. 72: 2 → 1
Char. 76: 0 → 1
Char. 97: 1 → 0
Char. 103: 0 → 1
Char. 129: 1 → 0
Char. 153: 1 → 0
Char. 191: 1 → 0
Char. 192: 1 → 0
Char. 202: 1 → 0
Char. 211: 0 → 1
Char. 222: 1 → 0
Char. 237: 1 → 0
Char. 240: 1 → 0
Char. 248: 0 → 1
Char. 249: 0 → 2
Char. 250: 0 → 1
Char. 263: 0 → 1
Char. 273: 0 → 1
Char. 274: 0 → 1
Char. 275: 0 → 1
Char. 276: 0 → 1
Char. 277: 0 → 1
Char. 278: 0 → 3

***Acerosodontosaurus piveteaui*:**
Char. 127: 1 → 0
Char. 128: 0 → 1
Char. 278: 0 → 1

***Acleistorhinus pteroticus*:**
Char. 21: 0 → 1
Char. 30: 0 → 1
Char. 33: 0 → 1
Char. 47: 0 → 1
Char. 55: 1 → 0
Char. 56: 0 → 1
Char. 64: 0 → 1
Char. 70: 0 → 1
Char. 79: 0 → 1
Char. 95: 0 → 1
Char. 113: 0 → 1
Char. 114: 0 → 1
Char. 140: 0 → 2
Char. 146: 1 → 0
Char. 169: 1 → 0
Char. 170: 0 → 1

**Araeoscelidae:**
Char. 5: 1 → 0
Char. 28: 0 → 1
Char. 106: 0 → 1
Char. 116: 1 → 0
Char. 117: 01 → 2
Char. 166: 1 → 0
Char. 169: 1 → 0
Char. 170: 0 → 1
Char. 197: 0 → 1
Char. 221: 1 → 0
Char. 239: 0 → 1

**Archosauriformes:**
Char. 32: 0 → 1
Char. 94: 1 → 0
Char. 112: 1 → 0
Char. 152: 0 → 1
Char. 154: 0 → 2
Char. 166: 1 → 0
Char. 171: 0 → 1
Char. 185: 0 → 1
Char. 218: 0 → 3
Char. 242: 0 → 1

***Australothyris smithi*:**
Char. 23: 0 → 1
Char. 34: 0 → 1
Char. 55: 1 → 0
Char. 71: 1 → 0
Char. 85: 1 → 0
Char. 112: 0 → 1
Char. 129: 0 → 1
Char. 159: 1 → 0

***Barasaurus besairiei*:**
Char. 33: 1 → 0
Char. 75: 1 → 0
Char. 216: 0 → 1

***Bashkyroleter bashkyricus*:**
Char. 275: 1 → 0

***Bashkyroleter mesensis*:**
Char. 169: 1 → 0

***Belebey vegrandis*:**
Char. 154: 1 → 0

***Bradysaurus* spp.:**
Char. 19: 0 → 1
Char. 73: 0 → 1
Char. 79: 0 → 1
Char. 135: 1 → 0
Char. 249: 0 → 1

**Captorhinidae:**
Char. 3: 0 → 1
Char. 23: 0 → 1
Char. 26: 0 → 1
Char. 73: 0 → 1
Char. 75: 0 → 1
Char. 83: 0 → 1
Char. 108: 1 → 0
Char. 183: 1 → 0
Char. 201: 1 → 0
Char. 203: 1 → 2
Char. 216: 1 → 0

**Caseidae:**
Char. 24: 0 → 1
Char. 36: 0 → 1
Char. 38: 1 → 0
Char. 46: 1 → 0
Char. 50: 1 → 0
Char. 56: 0 → 1
Char. 170: 0 → 1
Char. 194: 0 → 1
Char. 273: 0 → 1
Char. 274: 0 → 1
Char. 278: 3 → 2

***Claudiosaurus germaini*:**
Char. 34: 0 → 1
Char. 36: 0 → 1
Char. 56: 0 → 1
Char. 64: 0 → 1
Char. 70: 0 → 1
Char. 105: 0 → 1
Char. 106: 0 → 1
Char. 117: 01 → 2
Char. 126: 0 → 1
Char. 127: 1 → 0
Char. 130: 0 → 1
Char. 141: 1 → 0
Char. 144: 1 → 0
Char. 148: 0 → 1
Char. 166: 1 → 0
Char. 182: 0 → 1
Char. 187: 1 → 0
Char. 190: 0 → 1
Char. 199: 0 → 1
Char. 203: 1 → 2
Char. 222: 1 → 0
Char. 234: 1 → 0
Char. 272: 2 → 1

***Colobomycter pholeter*:**
Char. 21: 0 → 1
Char. 25: 1 → 0
Char. 84: 1 → 0
Char. 154: 1 → 0
Char. 167: 0 → 1
Char. 267: 0 → 1

***Delorhynchus cifelli*:**
Char. 18: 0 → 1
Char. 21: 0 → 1
Char. 24: 0 → 1
Char. 26: 0 → 1
Char. 28: 0 → 1
Char. 33: 0 → 2
Char. 39: 0 → 1
Char. 52: 0 → 1
Char. 100: 0 → 1
Char. 111: 0 → 1
Char. 116: 1 → 0
Char. 117: 0 → 1
Char. 156: 1 → 0
Char. 167: 0 → 1
Char. 189: 0 → 1
Char. 191: 0 → 1
Char. 267: 0 → 1

**Diadectomorpha:**
Char. 0: 0 → 1
Char. 44: 0 → 1
Char. 64: 0 → 1
Char. 70: 0 → 1
Char. 122: 0 → 1
Char. 123: 1 → 0
Char. 146: 1 → 0
Char. 275: 1 → 0
Char. 278: 3 → 0

***Emeroleter levis*:**
Char. 51: 1 → 0

**Eosauropterygia:**
Char. 159: 1 → 0
Char. 166: 1 → 0
Char. 194: 0 → 2
Char. 272: 1 → 0

***Eudibamus cursoris*:**
Char. 154: 1 → 2
Char. 163: 1 → 0

***Feeserpeton oklahomensis*:**
Char. 51: 0 → 1
Char. 70: 0 → 1
Char. 157: 0 → 1
Char. 158: 0 → 1

***Hovasaurus boulei*:**
Char. 41: 0 → 1
Char. 55: 1 → 0
Char. 60: 1 → 0
Char. 72: 2 → 1
Char. 77: 0 → 2
Char. 78: 1 → 0
Char. 79: 0 → 1
Char. 93: 1 → 0
Char. 113: 0 → 1
Char. 138: 0 → 1
Char. 141: 1 → 0
Char. 146: 1 → 0
Char. 154: 0 → 1
Char. 206: 0 → 2
Char. 215: 0 → 1
Char. 219: 1 → 0
Char. 278: 0 → 3

**Kuehneosauridae:**
Char. 7: 0 → 1
Char. 26: 0 → 1
Char. 34: 0 → 1
Char. 36: 0 → 1
Char. 56: 0 → 1
Char. 79: 0 → 2
Char. 82: 0 → 1
Char. 98: 1 → 0
Char. 108: 1 → 0
Char. 113: 0 → 1
Char. 128: 0 → 1
Char. 159: 1 → 0
Char. 181: 0 → 1
Char. 185: 0 → 1
Char. 206: 0 → 2
Char. 245: 0 → 1
Char. 278: 0 → 3

***Lanthanosuchus watsoni*:**
Char. 25: 1 → 0
Char. 51: 0 → 1
Char. 86: 0 → 1
Char. 95: 0 → 1
Char. 98: 1 → 0
Char. 113: 0 → 1
Char. 114: 0 → 1
Char. 138: 0 → 1
Char. 140: 0 → 2
Char. 144: 1 → 0
Char. 154: 1 → 2

***Macroleter poezicus*:**
Char. 52: 0 → 1
Char. 84: 1 → 0
Char. 87: 0 → 1
Char. 140: 0 → 1
Char. 146: 1 → 0
Char. 169: 1 → 0
Char. 235: 0 → 1

***Mesosaurus* spp.:**
Char. 0: 0 → 1
Char. 2: 0 → 1
Char. 5: 1 → 0
Char. 6: 0 → 1
Char. 8: 0 → 1
Char. 9: 0 → 1
Char. 13: 0 → 1
Char. 19: 0 → 1

Char. 23: 0 → 1
Char. 26: 0 → 1
Char. 29: 1 → 0
Char. 33: 0 → 1
Char. 38: 1 → 0
Char. 41: 0 → 1
Char. 50: 1 → 0
Char. 67: 0 → 1
Char. 83: 0 → 1
Char. 84: 1 → 0
Char. 85: 1 → 0
Char. 94: 0 → 1
Char. 107: 0 → 1
Char. 109: 0 → 1
Char. 111: 0 → 1
Char. 115: 0 → 1
Char. 131: 1 → 0
Char. 146: 1 → 0
Char. 147: 1 → 0
Char. 148: 0 → 1
Char. 149: 1 → 0
Char. 159: 1 → 0
Char. 163: 1 → 0
Char. 164: 0 → 1
Char. 166: 1 → 0
Char. 167: 0 → 1
Char. 176: 0 → 1
Char. 183: 1 → 0
Char. 184: 0 → 1
Char. 192: 1 → 0
Char. 199: 0 → 1
Char. 202: 0 → 1
Char. 206: 0 → 1
Char. 207: 0 → 1
Char. 209: 0 → 1
Char. 217: 0 → 1
Char. 219: 0 → 1
Char. 220: 0 → 1
Char. 231: 0 → 1
Char. 260: 2 → 0
Char. 272: 2 → 0
Char. 278: 13 → 0

***Microleter mckinzieorum*:**
Char. 0: 0 → 1
Char. 25: 1 → 0
Char. 36: 0 → 1
Char. 39: 0 → 1
Char. 51: 0 → 2
Char. 56: 0 → 1
Char. 70: 0 → 1
Char. 94: 0 → 1
Char. 106: 0 → 1
Char. 131: 1 → 0
Char. 166: 1 → 0
Char. 276: 0 → 1

**Millerettidae:**
Char. 24: 0 → 1
Char. 56: 0 → 1
Char. 57: 1 → 0
Char. 66: 01 → 2
Char. 84: 01 → 2
Char. 117: 0 → 1
Char. 124: 0 → 1
Char. 127: 0 → 1
Char. 135: 0 → 1
Char. 145: 0 → 1
Char. 202: 0 → 1
Char. 211: 0 → 1
Char. 230: 0 → 1
Char. 234: 0 → 1
Char. 248: 0 → 1
Char. 252: 0 → 1

***Nycteroleter ineptus*:**
Char. 278: 0 → 3

***Nyctiphruretus acudens*:**
Char. 21: 0 → 1
Char. 33: 1 → 2
Char. 41: 0 → 1
Char. 81: 1 → 0
Char. 84: 1 → 2
Char. 94: 0 → 1
Char. 166: 1 → 0
Char. 167: 0 → 1
Char. 215: 1 → 0
Char. 224: 0 → 1
Char. 266: 0 → 1
Char. 272: 2 → 1
Char. 276: 0 → 1

***Orovenator mayorum*:**
Char. 8: 0 → 1
Char. 36: 0 → 1
Char. 48: 1 → 0
Char. 160: 0 → 1
Char. 165: 0 → 1

***Owenetta* spp.:**
Char. 169: 1 → 0

***Paleothyris acadiana*:**
Char. 38: 1 → 0
Char. 50: 1 → 0
Char. 66: 1 → 2
Char. 102: 0 → 1
Char. 146: 1 → 0
Char. 180: 0 → 1
Char. 237: 0 → 1
Char. 239: 0 → 1

***Placodus* spp.:**
Char. 0: 1 → 2
Char. 9: 0 → 1
Char. 12: 0 → 1
Char. 13: 0 → 1
Char. 19: 0 → 1
Char. 26: 0 → 1
Char. 31: 0 → 1
Char. 46: 1 → 0
Char. 57: 0 → 1
Char. 78: 1 → 0
Char. 93: 1 → 0
Char. 102: 1 → 2
Char. 109: 1 → 0
Char. 140: 1 → 0
Char. 155: 0 → 1
Char. 163: 1 → 0
Char. 164: 0 → 1

***Procolophon* spp.:**
Char. 41: 0 → 1
Char. 69: 0 → 1
Char. 79: 0 → 1
Char. 88: 0 → 1
Char. 117: 1 → 0
Char. 149: 1 → 0
Char. 237: 0 → 1
Char. 238: 0 → 1
Char. 272: 2 → 1
Char. 278: 3 → 0

***Prolacerta broomi*:**
Char. 58: 1 → 0
Char. 66: 1 → 0
Char. 67: 1 → 0
Char. 80: 1 → 0
Char. 139: 1 → 0
Char. 147: 1 → 0
Char. 192: 1 → 0
Char. 203: 1 → 2
Char. 206: 0 → 12

***Rhipaeosaurus* spp.:**
Char. 172: 0 → 1
Char. 277: 0 → 1

**Rhynchocephalia:**
Char. 0: 1 → 2
Char. 24: 0 → 1
Char. 75: 1 → 0
Char. 77: 0 → 1
Char. 94: 1 → 0
Char. 139: 1 → 0
Char. 167: 1 → 0
Char. 205: 1 → 0

**Rhynchosauria:**
Char. 0: 1 → 0
Char. 7: 0 → 1
Char. 9: 0 → 1
Char. 26: 0 → 1
Char. 68: 0 → 1
Char. 99: 1 → 0
Char. 150: 1 → 0
Char. 160: 0 → 1
Char. 161: 0 → 1
Char. 171: 0 → 2
Char. 223: 0 → 1
Char. 241: 0 → 1

***Scutosaurus* spp.:**
Char. 175: 0 → 1
Char. 218: 0 → 1
Char. 243: 0 → 2
Char. 244: 0 → 1

***Sinosaurosphargis yunguiensis*:**
Char. 8: 0 → 1
Char. 30: 0 → 1
Char. 53: 0 → 1
Char. 82: 0 → 1
Char. 89: 1 → 0
Char. 127: 1 → 0
Char. 150: 1 → 0
Char. 154: 0 → 2
Char. 167: 1 → 0
Char. 255: 0 → 1

**Squamata:**
Char. 26: 0 → 1
Char. 45: 0 → 1
Char. 79: 0 → 2
Char. 80: 1 → 0
Char. 82: 0 → 1
Char. 92: 1 → 0
Char. 109: 1 → 0
Char. 160: 0 → 1
Char. 245: 0 → 1

***Trilophosaurus buettneri*:**
Char. 5: 1 → 0
Char. 11: 0 → 1
Char. 55: 1 → 0
Char. 93: 1 → 0
Char. 104: 0 → 1
Char. 106: 0 → 1
Char. 113: 0 → 1
Char. 122: 0 → 1
Char. 136: 1 → 0
Char. 144: 1 → 0
Char. 154: 0 → 1
Char. 157: 0 → 1
Char. 159: 1 → 0
Char. 177: 0 → 12
Char. 194: 0 → 1
Char. 203: 1 → 2
Char. 207: 1 → 0
Char. 208: 1 → 0
Char. 272: 1 → 0

***Youngina capensis*:**
Char. 5: 1 → 0
Char. 25: 0 → 1
Char. 56: 0 → 1
Char. 75: 0 → 1
Char. 92: 1 → 0
Char. 94: 1 → 0
Char. 163: 1 → 0
Char. 170: 0 → 1
Char. 211: 0 → 1
Char. 215: 0 → 1
Char. 239: 0 → 1

**Node 49:**
Char. 46: 1 → 0
Char. 50: 0 → 1
Char. 88: 1 → 0
Char. 89: 1 → 0
Char. 93: 1 → 0
Char. 176: 0 → 1
Char. 195: 0 → 2
Char. 198: 0 → 1
Char. 240: 1 → 0
Char. 246: 1 → 2
Char. 259: 0 → 1
Char. 265: 1 → 0
Char. 270: 0 → 1

**Node 50:**
Char. 65: 0 → 1
Char. 184: 0 → 1
Char. 205: 0 → 1
Char. 210: 0 → 1
Char. 219: 1 → 0
Char. 241: 0 → 1
Char. 246: 0 → 1
Char. 254: 0 → 1
Char. 255: 0 → 1
Char. 256: 0 → 1
Char. 268: 0 → 1
Char. 269: 0 → 1

**Node 51:**
Char. 0: 1 → 2
Char. 15: 0 → 1
Char. 33: 1 → 2
Char. 62: 1 → 0
Char. 64: 0 → 1
Char. 78: 1 → 0
Char. 81: 1 → 0
Char. 84: 01 → 2
Char. 130: 0 → 1
Char. 134: 01 → 2
Char. 147: 0 → 1
Char. 148: 0 → 1
Char. 152: 0 → 1
Char. 154: 0 → 1
Char. 155: 0 → 1
Char. 158: 0 → 1
Char. 161: 0 → 1
Char. 181: 0 → 1
Char. 203: 1 → 2
Char. 206: 0 → 2
Char. 208: 0 → 1
Char. 252: 0 → 1

**Node 52:**
Char. 33: 0 → 1
Char. 92: 0 → 1
Char. 94: 0 → 1
Char. 135: 0 → 1
Char. 267: 0 → 1
Char. 278: 1 → 0

**Node 53:**
Char. 25: 1 → 0
Char. 62: 0 → 1
Char. 141: 0 → 1

**Node 54:**
Char. 0: 0 → 1

Char. 27: 0 → 1
Char. 29: 1 → 0
Char. 40: 2 → 0
Char. 57: 1 → 0
Char. 59: 0 → 1
Char. 60: 0 → 1
Char. 67: 0 → 1
Char. 72: 1 → 2
Char. 89: 0 → 1
Char. 111: 0 → 1
Char. 112: 0 → 1
Char. 120: 0 → 1
Char. 147: 1 → 0
Char. 154: 1 → 0
Char. 193: 0 → 1
Char. 234: 0 → 1
Char. 237: 0 → 1
Char. 266: 0 → 1

**Node 55:**
Char. 5: 0 → 1
Char. 44: 0 → 1
Char. 48: 0 → 1
Char. 88: 0 → 1
Char. 96: 0 → 1
Char. 121: 1 → 0
Char. 131: 0 → 1
Char. 147: 0 → 1
Char. 159: 0 → 1
Char. 163: 0 → 1
Char. 166: 0 → 1
Char. 169: 0 → 1
Char. 192: 0 → 1
Char. 221: 0 → 1

**Node 56:**
Char. 40: 0 → 2
Char. 74: 0 → 1
Char. 132: 0 → 1
Char. 149: 0 → 1
Char. 156: 0 → 1
Char. 201: 0 → 1
Char. 203: 0 → 1
Char. 235: 0 → 1

**Node 57:**
Char. 72: 0 → 1
Char. 79: 1 → 0
Char. 81: 0 → 1
Char. 97: 0 → 1
Char. 104: 1 → 0
Char. 144: 0 → 1
Char. 173: 0 → 1
Char. 183: 0 → 1

**Node 59:**
Char. 19: 0 → 1
Char. 92: 1 → 0

**Node 60:**
Char. 4: 0 → 1
Char. 15: 0 → 1
Char. 29: 0 → 1
Char. 213: 0 → 1
Char. 226: 0 → 2
Char. 228: 0 → 1
Char. 275: 0 → 1

**Node 61:**
Char. 27: 1 → 0
Char. 75: 0 → 1
Char. 107: 0 → 1
Char. 140: 0 → 1
Char. 147: 0 → 1
Char. 148: 0 → 1
Char. 272: 2 → 1

**Node 62:**
Char. 58: 0 → 1
Char. 61: 0 → 1
Char. 66: 0 → 1
Char. 69: 0 → 1
Char. 126: 0 → 1
Char. 150: 0 → 1
Char. 167: 0 → 1
Char. 176: 0 → 1
Char. 205: 0 → 1
Char. 208: 0 → 1
Char. 239: 0 → 1

**Node 63:**
Char. 24: 0 → 1
Char. 57: 1 → 0
Char. 79: 0 → 1
Char. 83: 0 → 1
Char. 132: 1 → 0

**Node 64:**
Char. 73: 0 → 1
Char. 131: 1 → 0
Char. 205: 0 → 1
Char. 276: 0 → 1

**Node 65:**
Char. 18: 0 → 1
Char. 37: 0 → 1
Char. 107: 0 → 1
Char. 118: 1 → 0
Char. 125: 1 → 0
Char. 150: 0 → 1

**Node 66:**
Char. 23: 0 → 1
Char. 71: 1 → 0
Char. 102: 0 → 1
Char. 103: 0 → 1
Char. 106: 0 → 1

Char. 167: 0 → 1
Char. 214: 0 → 1
Char. 216: 1 → 0
Char. 235: 0 → 2
Char. 241: 0 → 1

**Node 67:**
Char. 138: 0 → 1
Char. 141: 0 → 1
Char. 207: 0 → 1

**Node 68:**
Char. 33: 0 → 1
Char. 44: 1 → 0
Char. 70: 0 → 1
Char. 80: 1 → 0
Char. 117: 0 → 1
Char. 118: 0 → 1
Char. 125: 0 → 1
Char. 194: 0 → 1
Char. 201: 1 → 0
Char. 211: 0 → 1

**Node 69:**
Char. 38: 1 → 2
Char. 39: 0 → 1
Char. 49: 0 → 1
Char. 66: 0 → 12
Char. 85: 1 → 0
Char. 88: 1 → 0
Char. 95: 0 → 1
Char. 112: 0 → 1
Char. 148: 0 → 1
Char. 158: 0 → 1
Char. 183: 1 → 2
Char. 215: 0 → 1

**Node 70:**
Char. 87: 0 → 1

**Node 71:**
Char. 25: 1 → 0
Char. 76: 1 → 0

**Node 72:**
Char. 79: 0 → 1
Char. 93: 1 → 0
Char. 133: 0 → 1

**Node 73:**
Char. 85: 0 → 1
Char. 147: 1 → 2
Char. 154: 1 → 0
Char. 278: 3 → 0

**Node 74:**
Char. 50: 1 → 0
Char. 58: 0 → 1

Char. 59: 0 → 1
Char. 60: 0 → 1
Char. 72: 1 → 2
Char. 104: 0 → 1
Char. 105: 0 → 1
Char. 106: 0 → 2
Char. 107: 0 → 1
Char. 109: 0 → 1
Char. 146: 1 → 0
Char. 155: 0 → 2

**Node 75:**
Char. 33: 1 → 0
Char. 38: 2 → 1
Char. 39: 1 → 0
Char. 42: 0 → 1
Char. 43: 0 → 1
Char. 46: 1 → 0
Char. 49: 1 → 0
Char. 52: 0 → 1
Char. 83: 01 → 2
Char. 84: 1 → 0
Char. 87: 0 → 1
Char. 93: 1 → 0
Char. 161: 0 → 1
Char. 163: 1 → 0
Char. 172: 0 → 2
Char. 188: 0 → 1
Char. 189: 0 → 1
Char. 195: 0 → 1
Char. 212: 0 → 1
Char. 221: 1 → 0
Char. 236: 0 → 1
Char. 238: 0 → 2
Char. 242: 0 → 1
Char. 274: 0 → 1
Char. 275: 1 → 0

**Node 76:**
Char. 59: 0 → 1
Char. 60: 0 → 1
Char. 67: 0 → 1
Char. 72: 1 → 2
Char. 111: 0 → 1
Char. 123: 1 → 0
Char. 129: 0 → 1
Char. 170: 0 → 1
Char. 197: 0 → 1

**Node 77:**
Char. 47: 0 → 1
Char. 83: 0 → 1
Char. 85: 1 → 0
Char. 107: 0 → 1
Char. 169: 1 → 0
Char. 170: 0 → 1

**Node 78:**
Char. 2: 0 → 1
Char. 6: 0 → 1
Char. 98: 1 → 0
Char. 101: 0 → 1
Char. 113: 0 → 1
Char. 181: 0 → 1
Char. 186: 0 → 1
Char. 193: 1 → 0
Char. 198: 0 → 1
Char. 206: 0 → 2
Char. 220: 0 → 1
Char. 234: 1 → 0
Char. 239: 1 → 0
Char. 240: 1 → 0
Char. 266: 1 → 0

**Node 79:**
Char. 33: 1 → 2
Char. 68: 0 → 1
Char. 102: 0 → 1
Char. 103: 0 → 1
Char. 223: 0 → 1
Char. 229: 0 → 1
Char. 235: 1 → 2

**Node 80:**
Char. 17: 0 → 1
Char. 42: 0 → 1
Char. 264: 0 → 1

**Node 81:**
Char. 41: 0 → 1
Char. 61: 1 → 2
Char. 112: 1 → 0
Char. 128: 0 → 1
Char. 138: 0 → 1
Char. 146: 1 → 0
Char. 155: 0 → 1
Char. 192: 1 → 2
Char. 226: 0 → 1
Char. 227: 0 → 1
Char. 233: 0 → 1

**Node 82:**
Char. 61: 1 → 3
Char. 90: 0 → 1
Char. 91: 0 → 1
Char. 109: 1 → 0
Char. 155: 0 → 1
Char. 209: 1 → 0

**ANALYSIS 90**

**(NO *CANDELARIA BARBOURI*, IMPLIED WEIGHTING, K = 2.75, NO CHARACTERS 174, 204, 247, 251, AND 253)**

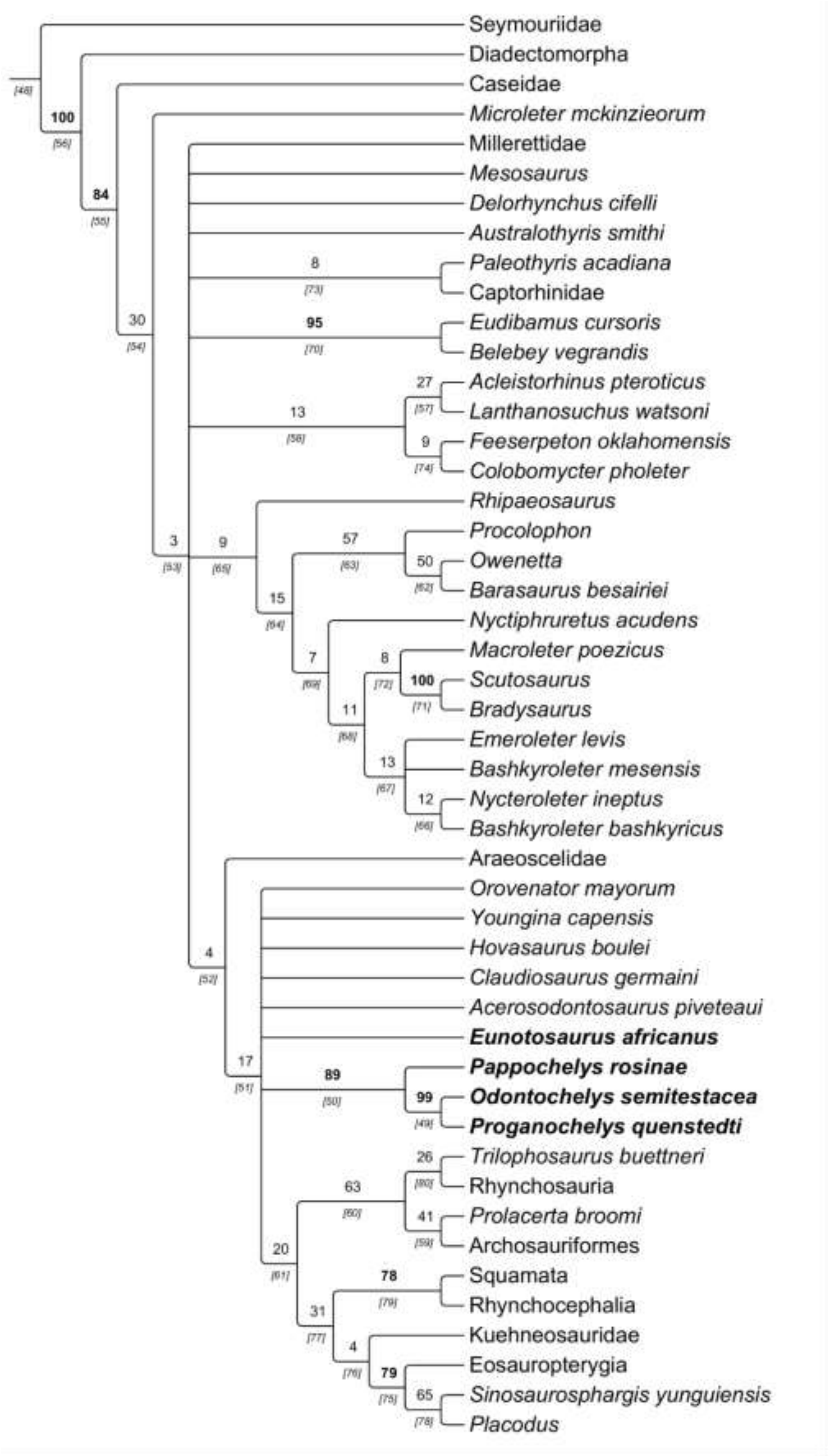

***Proganochelys quenstedti*:**
Char. 8: 0 → 1
Char. 11: 0 → 1
Char. 106: 0 → 1
Char. 108: 1 → 0
Char. 128: 0 → 1
Char. 175: 0 → 1
Char. 202: 1 → 0
Char. 207: 1 → 0
Char. 209: 1 → 0
Char. 244: 0 → 1
Char. 248: 0 → 1
Char. 250: 0 → 1
Char. 262: 0 → 1

***Pappochelys rosinae*:**
Char. 1: 0 → 1
Char. 5: 1 → 0
Char. 12: 0 → 1
Char. 41: 0 → 1
Char. 49: 0 → 1
Char. 55: 1 → 0
Char. 67: 1 → 0
Char. 75: 0 → 1
Char. 169: 1 → 0
Char. 260: 2 → 0

***Odontochelys semitestacea*:**
Char. 109: 1 → 0
Char. 112: 1 → 0
Char. 124: 0 → 1

***Eunotosaurus africanus*:**
Char. 0: 1 → 2
Char. 15: 0 → 1
Char. 19: 0 → 1
Char. 24: 0 → 1
Char. 33: 1 → 2
Char. 42: 0 → 1
Char. 48: 1 → 0
Char. 50: 0 → 1
Char. 55: 1 → 0
Char. 57: 0 → 1
Char. 59: 1 → 0
Char. 60: 1 → 0
Char. 62: 1 → 0
Char. 67: 1 → 0
Char. 72: 2 → 1
Char. 76: 0 → 1
Char. 81: 1 → 0
Char. 84: 1 → 2
Char. 97: 1 → 0
Char. 102: 0 → 1
Char. 103: 0 → 1
Char. 109: 1 → 0
Char. 112: 1 → 0
Char. 124: 0 → 1
Char. 129: 1 → 0
Char. 130: 0 → 1
Char. 134: 0 → 2
Char. 147: 0 → 1
Char. 152: 0 → 1
Char. 153: 1 → 0
Char. 155: 0 → 1
Char. 158: 0 → 1
Char. 161: 0 → 1
Char. 181: 0 → 1
Char. 191: 1 → 0
Char. 192: 1 → 0
Char. 194: 0 → 1
Char. 202: 1 → 0
Char. 211: 0 → 1
Char. 222: 1 → 0
Char. 237: 1 → 0
Char. 240: 1 → 0
Char. 248: 0 → 1
Char. 249: 0 → 2
Char. 250: 0 → 1
Char. 252: 0 → 1
Char. 263: 0 → 1
Char. 273: 0 → 1
Char. 274: 0 → 1
Char. 275: 0 → 1
Char. 276: 0 → 1
Char. 277: 0 → 1
Char. 278: 0 → 3

***Acerosodontosaurus piveteaui*:**
Char. 78: 1 → 0
Char. 81: 1 → 0
Char. 127: 1 → 0
Char. 128: 0 → 1
Char. 155: 0 → 1
Char. 206: 0 → 2
Char. 208: 0 → 1
Char. 278: 0 → 1

***Acleistorhinus pteroticus:***
Char. 21: 0 → 1
Char. 146: 1 → 0

**Araeoscelidae:**
Char. 28: 0 → 1
Char. 84: 1 → 0
Char. 106: 0 → 1
Char. 116: 1 → 0
Char. 117: 01 → 2
Char. 166: 1 → 0
Char. 169: 1 → 0
Char. 170: 0 → 1
Char. 197: 0 → 1
Char. 239: 0 → 1

**Archosauriformes:**
Char. 32: 0 → 1
Char. 94: 1 → 0
Char. 112: 1 → 0
Char. 152: 0 → 1
Char. 154: 01 → 2
Char. 166: 1 → 0
Char. 171: 0 → 1
Char. 185: 0 → 1
Char. 201: 0 → 1
Char. 218: 0 → 3
Char. 242: 0 → 1

***Australothyris smithi*:**
Char. 24: 0 → 1
Char. 34: 0 → 1
Char. 55: 1 → 0
Char. 57: 1 → 0
Char. 71: 1 → 0
Char. 79: 0 → 1
Char. 98: 1 → 0
Char. 100: 0 → 1
Char. 103: 0 → 1
Char. 110: 0 → 1
Char. 112: 0 → 1
Char. 123: 1 → 0
Char. 129: 0 → 1
Char. 131: 0 → 1
Char. 132: 1 → 0
Char. 144: 1 → 0
Char. 147: 0 → 1
Char. 149: 1 → 0
Char. 150: 0 → 1

***Barasaurus besairiei*:**
Char. 33: 1 → 0

***Bashkyroleter bashkyricus*:**
Char. 275: 1 → 0

***Bashkyroleter mesensis*:**
Char. 12: 0 → 1
Char. 66: 1 → 0
Char. 169: 1 → 0

***Belebey vegrandis*:**
Char. 154: 1 → 0
Char. 163: 0 → 1

***Bradysaurus* spp.:**
Char. 19: 0 → 1
Char. 25: 1 → 0
Char. 73: 0 → 1
Char. 79: 0 → 1
Char. 135: 1 → 0
Char. 249: 0 → 1

**Captorhinidae:**
Char. 3: 0 → 1
Char. 25: 1 → 0
Char. 26: 0 → 1
Char. 73: 0 → 1
Char. 75: 0 → 1
Char. 108: 1 → 0
Char. 183: 1 → 0
Char. 201: 1 → 0
Char. 203: 1 → 2
Char. 216: 1 → 0

**Caseidae:**
Char. 38: 1 → 0
Char. 46: 1 → 0
Char. 50: 1 → 0
Char. 170: 0 → 1
Char. 194: 0 → 1
Char. 273: 0 → 1
Char. 274: 0 → 1
Char. 278: 3 → 2

***Claudiosaurus germaini*:**
Char. 21: 1 → 0
Char. 24: 0 → 1
Char. 34: 0 → 1
Char. 36: 0 → 1
Char. 43: 1 → 0
Char. 56: 0 → 1
Char. 70: 0 → 1
Char. 84: 1 → 0
Char. 105: 0 → 1
Char. 106: 0 → 1
Char. 117: 01 → 2
Char. 126: 0 → 1
Char. 127: 1 → 0
Char. 130: 0 → 1
Char. 131: 0 → 1
Char. 141: 1 → 0
Char. 144: 1 → 0
Char. 166: 1 → 0
Char. 182: 0 → 1
Char. 187: 1 → 0
Char. 190: 0 → 1
Char. 199: 0 → 1
Char. 201: 0 → 1
Char. 214: 0 → 1
Char. 222: 1 → 0
Char. 231: 1 → 0
Char. 234: 1 → 0
Char. 272: 2 → 1

***Colobomycter pholeter*:**
Char. 21: 0 → 1
Char. 25: 1 → 0
Char. 84: 1 → 0
Char. 154: 1 → 0
Char. 167: 0 → 1
Char. 267: 0 → 1

***Delorhynchus cifelli*:**
Char. 18: 0 → 1
Char. 20: 0 → 1
Char. 21: 0 → 1
Char. 24: 0 → 1
Char. 26: 0 → 1
Char. 28: 0 → 1
Char. 33: 0 → 2
Char. 39: 0 → 1
Char. 52: 0 → 1
Char. 100: 0 → 1
Char. 111: 0 → 1
Char. 116: 1 → 0
Char. 131: 0 → 1
Char. 147: 0 → 1
Char. 156: 1 → 0
Char. 163: 0 → 1
Char. 167: 0 → 1
Char. 189: 0 → 1
Char. 191: 0 → 1
Char. 267: 0 → 1

**Diadectomorpha:**
Char. 0: 0 → 1
Char. 64: 0 → 1
Char. 70: 0 → 1
Char. 122: 0 → 1
Char. 123: 1 → 0
Char. 146: 1 → 0
Char. 275: 1 → 0
Char. 278: 3 → 0

***Emeroleter levis*:**
Char. 51: 1 → 0

**Eosauropterygia:**
Char. 166: 1 → 0
Char. 178: 0 → 1
Char. 194: 0 → 2
Char. 272: 1 → 0

***Eudibamus cursoris*:**
Char. 154: 1 → 2

***Feeserpeton oklahomensis*:**
Char. 51: 0 → 1
Char. 157: 0 → 1
Char. 158: 0 → 1

***Hovasaurus boulei*:**
Char. 41: 0 → 1
Char. 55: 1 → 0
Char. 60: 1 → 0
Char. 72: 2 → 1
Char. 73: 1 → 0
Char. 77: 0 → 2
Char. 78: 1 → 0
Char. 79: 0 → 1
Char. 93: 1 → 0
Char. 113: 0 → 1
Char. 138: 0 → 1
Char. 141: 1 → 0
Char. 146: 1 → 0
Char. 206: 0 → 2
Char. 215: 0 → 1
Char. 217: 1 → 0
Char. 219: 1 → 0
Char. 231: 1 → 0
Char. 278: 0 → 3

**Kuehneosauridae:**
Char. 7: 0 → 1
Char. 21: 1 → 0
Char. 24: 0 → 1
Char. 27: 0 → 1
Char. 43: 1 → 0
Char. 79: 0 → 2
Char. 107: 1 → 0
Char. 140: 1 → 0
Char. 147: 1 → 0
Char. 148: 1 → 0

Char. 245: 0 → 1
Char. 278: 0 → 3

***Lanthanosuchus watsoni*:**
Char. 25: 1 → 0
Char. 51: 0 → 1
Char. 76: 0 → 1
Char. 86: 0 → 1
Char. 98: 1 → 0
Char. 138: 0 → 1
Char. 144: 1 → 0
Char. 154: 1 → 2

***Macroleter poezicus*:**
Char. 9: 0 → 1
Char. 26: 0 → 1
Char. 66: 1 → 2
Char. 126: 0 → 1
Char. 134: 0 → 2
Char. 140: 0 → 1
Char. 146: 1 → 0
Char. 169: 1 → 0

***Mesosaurus* spp.:**
Char. 0: 0 → 1
Char. 2: 0 → 1
Char. 6: 0 → 1
Char. 8: 0 → 1
Char. 9: 0 → 1
Char. 13: 0 → 1
Char. 19: 0 → 1
Char. 26: 0 → 1
Char. 29: 1 → 0
Char. 33: 0 → 1
Char. 38: 1 → 0
Char. 41: 0 → 1
Char. 50: 1 → 0
Char. 67: 0 → 1
Char. 76: 0 → 1
Char. 84: 1 → 0
Char. 94: 0 → 1
Char. 107: 0 → 1
Char. 109: 0 → 1
Char. 111: 0 → 1
Char. 115: 0 → 1
Char. 146: 1 → 0
Char. 149: 1 → 0
Char. 164: 0 → 1
Char. 166: 1 → 0
Char. 167: 0 → 1
Char. 176: 0 → 1
Char. 183: 1 → 0
Char. 184: 0 → 1
Char. 199: 0 → 1
Char. 202: 0 → 1
Char. 206: 0 → 1
Char. 207: 0 → 1
Char. 209: 0 → 1
Char. 217: 0 → 1
Char. 219: 0 → 1
Char. 220: 0 → 1
Char. 231: 0 → 1
Char. 260: 2 → 0

Char. 272: 2 → 0
Char. 278: 3 → 0

***Microleter mckinzieorum*:**
Char. 0: 0 → 1
Char. 18: 0 → 1
Char. 39: 0 → 1
Char. 57: 1 → 0
Char. 70: 0 → 1
Char. 76: 0 → 1
Char. 94: 0 → 1
Char. 106: 0 → 1
Char. 110: 0 → 1
Char. 278: 3 → 1

**Millerettidae:**
Char. 24: 0 → 1
Char. 25: 1 → 0
Char. 44: 1 → 0
Char. 48: 1 → 0
Char. 56: 0 → 1
Char. 57: 1 → 0
Char. 66: 0 → 2
Char. 78: 1 → 0
Char. 80: 1 → 0
Char. 84: 1 → 2
Char. 88: 1 → 0
Char. 96: 1 → 0
Char. 121: 0 → 1
Char. 124: 0 → 1
Char. 127: 0 → 1
Char. 135: 0 → 1
Char. 145: 0 → 1
Char. 166: 1 → 0
Char. 202: 0 → 1
Char. 211: 0 → 1
Char. 230: 0 → 1
Char. 234: 0 → 1
Char. 248: 0 → 1
Char. 252: 0 → 1

***Nycteroleter ineptus*:**
Char. 66: 1 → 0
Char. 278: 0 → 3

***Nyctiphruretus acudens*:**
Char. 21: 0 → 1
Char. 41: 0 → 1
Char. 48: 1 → 0
Char. 66: 1 → 2
Char. 81: 1 → 0
Char. 84: 1 → 2
Char. 94: 0 → 1
Char. 166: 1 → 0
Char. 224: 0 → 1
Char. 226: 1 → 0
Char. 266: 0 → 1
Char. 272: 2 → 1
Char. 276: 0 → 1

***Orovenator mayorum*:**
Char. 8: 0 → 1
Char. 21: 1 → 0

Char. 24: 0 → 1
Char. 33: 1 → 0
Char. 36: 0 → 1
Char. 43: 1 → 0
Char. 48: 1 → 0
Char. 50: 0 → 1
Char. 92: 1 → 0
Char. 94: 1 → 0
Char. 135: 1 → 0
Char. 159: 1 → 0
Char. 160: 0 → 1
Char. 165: 0 → 1
Char. 278: 0 → 1

***Owenetta* spp.:**
Char. 142: 1 → 0
Char. 169: 1 → 0

***Paleothyris acadiana*:**
Char. 38: 1 → 0
Char. 50: 1 → 0
Char. 66: 1 → 2
Char. 102: 0 → 1
Char. 146: 1 → 0
Char. 237: 0 → 1
Char. 239: 0 → 1

***Placodus* spp.:**
Char. 0: 1 → 2
Char. 9: 0 → 1
Char. 12: 0 → 1
Char. 13: 0 → 1
Char. 19: 0 → 1
Char. 31: 0 → 1
Char. 46: 1 → 0
Char. 57: 0 → 1
Char. 78: 1 → 0
Char. 93: 1 → 0
Char. 102: 1 → 2
Char. 109: 1 → 0
Char. 140: 1 → 0
Char. 155: 0 → 1
Char. 163: 1 → 0
Char. 164: 0 → 1

***Procolophon* spp.:**
Char. 41: 0 → 1
Char. 69: 0 → 1
Char. 79: 0 → 1
Char. 86: 0 → 1
Char. 101: 0 → 1
Char. 141: 0 → 1
Char. 149: 1 → 0
Char. 203: 1 → 2
Char. 230: 0 → 1
Char. 237: 0 → 1
Char. 238: 0 → 1
Char. 272: 2 → 1
Char. 278: 3 → 0

***Prolacerta broomi*:**
Char. 58: 1 → 0
Char. 66: 1 → 0

Char. 67: 1 → 0
Char. 80: 1 → 0
Char. 139: 1 → 0
Char. 147: 1 → 0
Char. 192: 1 → 0
Char. 206: 0 → 12

***Rhipaeosaurus* spp.:**
Char. 172: 0 → 1
Char. 277: 0 → 1

**Rhynchocephalia:**
Char. 0: 1 → 2
Char. 24: 0 → 1
Char. 77: 0 → 1
Char. 88: 1 → 0
Char. 94: 1 → 0
Char. 139: 1 → 0
Char. 167: 1 → 0
Char. 205: 1 → 0

**Rhynchosauria:**
Char. 0: 1 → 0
Char. 7: 0 → 1
Char. 9: 0 → 1
Char. 26: 0 → 1
Char. 68: 0 → 1
Char. 99: 1 → 0
Char. 150: 1 → 0
Char. 160: 0 → 1
Char. 161: 0 → 1
Char. 171: 0 → 2
Char. 223: 0 → 1
Char. 241: 0 → 1

***Scutosaurus* spp.:**
Char. 175: 0 → 1
Char. 190: 0 → 1
Char. 218: 0 → 1
Char. 243: 0 → 2
Char. 244: 0 → 1

***Sinosaurosphargis yunguiensis*:**
Char. 8: 0 → 1
Char. 30: 0 → 1
Char. 53: 0 → 1
Char. 89: 1 → 0
Char. 127: 1 → 0
Char. 150: 1 → 0
Char. 154: 0 → 2
Char. 167: 1 → 0
Char. 255: 0 → 1

**Squamata:**
Char. 45: 0 → 1
Char. 79: 0 → 2
Char. 80: 1 → 0
Char. 92: 1 → 0
Char. 109: 1 → 0
Char. 160: 0 → 1
Char. 200: 0 → 1
Char. 245: 0 → 1

***Trilophosaurus buettneri*:**
Char. 5: 1 → 0
Char. 11: 0 → 1
Char. 55: 1 → 0
Char. 93: 1 → 0
Char. 104: 0 → 1
Char. 106: 0 → 1
Char. 113: 0 → 1
Char. 122: 0 → 1
Char. 136: 1 → 0
Char. 144: 1 → 0
Char. 157: 0 → 1
Char. 159: 1 → 0
Char. 177: 0 → 12
Char. 194: 0 → 1
Char. 207: 1 → 0
Char. 208: 1 → 0
Char. 272: 1 → 0

***Youngina capensis*:**
Char. 5: 1 → 0
Char. 25: 0 → 1
Char. 56: 0 → 1
Char. 73: 1 → 0
Char. 75: 0 → 1
Char. 84: 1 → 0
Char. 92: 1 → 0
Char. 94: 1 → 0
Char. 134: 0 → 1
Char. 145: 1 → 0
Char. 151: 1 → 0
Char. 163: 1 → 0
Char. 170: 0 → 1
Char. 211: 0 → 1
Char. 214: 0 → 1
Char. 215: 0 → 1
Char. 220: 1 → 0
Char. 239: 0 → 1

**Node 49:**
Char. 46: 1 → 0
Char. 50: 0 → 1
Char. 88: 1 → 0
Char. 89: 1 → 0
Char. 93: 1 → 0
Char. 176: 0 → 1
Char. 195: 0 → 2
Char. 198: 0 → 1
Char. 240: 1 → 0
Char. 246: 1 → 2
Char. 259: 0 → 1
Char. 265: 1 → 0
Char. 270: 0 → 1

**Node 50:**
Char. 62: 1 → 0
Char. 65: 0 → 1
Char. 78: 1 → 0
Char. 131: 0 → 1
Char. 184: 0 → 1
Char. 193: 1 → 0
Char. 194: 0 → 2
Char. 205: 0 → 1

Char. 210: 0 → 1
Char. 219: 1 → 0
Char. 241: 0 → 1
Char. 246: 0 → 1
Char. 254: 0 → 1
Char. 255: 0 → 1
Char. 256: 0 → 1
Char. 268: 0 → 1
Char. 269: 0 → 1

**Node 51:**
Char. 20: 0 → 1
Char. 21: 0 → 1
Char. 25: 1 → 0
Char. 33: 0 → 1
Char. 62: 0 → 1
Char. 73: 0 → 1
Char. 94: 0 → 1
Char. 109: 0 → 1
Char. 127: 0 → 1
Char. 129: 0 → 1
Char. 135: 0 → 1
Char. 141: 0 → 1
Char. 151: 0 → 1
Char. 179: 0 → 1
Char. 187: 0 → 1
Char. 188: 0 → 1
Char. 191: 0 → 1
Char. 196: 0 → 1
Char. 201: 1 → 0
Char. 202: 0 → 1
Char. 207: 0 → 1
Char. 209: 0 → 1
Char. 217: 0 → 1
Char. 219: 0 → 1
Char. 220: 0 → 1
Char. 231: 0 → 1
Char. 265: 0 → 1
Char. 275: 1 → 0

**Node 52:**
Char. 0: 0 → 1
Char. 29: 1 → 0
Char. 40: 2 → 0
Char. 43: 0 → 1
Char. 50: 1 → 0
Char. 57: 1 → 0
Char. 59: 0 → 1
Char. 60: 0 → 1
Char. 67: 0 → 1
Char. 72: 1 → 2
Char. 89: 0 → 1
Char. 111: 0 → 1
Char. 112: 0 → 1
Char. 120: 0 → 1
Char. 163: 0 → 1
Char. 193: 0 → 1
Char. 222: 0 → 1
Char. 234: 0 → 1
Char. 237: 0 → 1
Char. 266: 0 → 1

**Node 53:**
Char. 48: 0 → 1
Char. 132: 0 → 1
Char. 166: 0 → 1
Char. 276: 1 → 0

**Node 54:**
Char. 29: 0 → 1
Char. 84: 0 → 1
Char. 88: 0 → 1
Char. 169: 0 → 1

**Node 55:**
Char. 72: 0 → 1
Char. 81: 0 → 1
Char. 93: 0 → 1
Char. 97: 0 → 1
Char. 104: 1 → 0
Char. 144: 0 → 1
Char. 173: 0 → 1
Char. 183: 0 → 1

**Node 57:**
Char. 95: 0 → 1
Char. 113: 0 → 1
Char. 114: 0 → 1

**Node 58:**
Char. 20: 0 → 1
Char. 47: 0 → 1
Char. 79: 0 → 1
Char. 110: 0 → 1
Char. 131: 0 → 1
Char. 137: 0 → 1
Char. 140: 0 → 2
Char. 147: 0 → 1
Char. 163: 0 → 1

**Node 59:**
Char. 19: 0 → 1
Char. 21: 1 → 0
Char. 92: 1 → 0

**Node 60:**
Char. 4: 0 → 1
Char. 15: 0 → 1
Char. 29: 0 → 1
Char. 213: 0 → 1
Char. 226: 0 → 2
Char. 228: 0 → 1
Char. 275: 0 → 1

**Node 61:**
Char. 58: 0 → 1
Char. 61: 0 → 1
Char. 66: 0 → 1
Char. 69: 0 → 1
Char. 107: 0 → 1
Char. 126: 0 → 1
Char. 131: 0 → 1
Char. 134: 0 → 1
Char. 140: 0 → 1
Char. 147: 0 → 1
Char. 150: 0 → 1
Char. 167: 0 → 1
Char. 176: 0 → 1
Char. 190: 0 → 1
Char. 205: 0 → 1
Char. 208: 0 → 1
Char. 214: 0 → 1
Char. 220: 1 → 0
Char. 230: 0 → 1
Char. 239: 0 → 1
Char. 260: 2 → 1

**Node 62:**
Char. 73: 0 → 1
Char. 205: 0 → 1
Char. 273: 0 → 1
Char. 276: 0 → 1

**Node 63:**
Char. 18: 0 → 1
Char. 25: 1 → 0
Char. 37: 0 → 1
Char. 103: 0 → 1
Char. 106: 0 → 1
Char. 107: 0 → 1
Char. 110: 0 → 1
Char. 126: 0 → 1
Char. 150: 0 → 1

**Node 64:**
Char. 186: 0 → 1

**Node 65:**
Char. 183: 1 → 2
Char. 194: 0 → 1
Char. 201: 1 → 0
Char. 211: 0 → 1
Char. 226: 0 → 1
Char. 252: 0 → 1

**Node 66:**
Char. 87: 0 → 1
Char. 110: 0 → 1

**Node 67:**
Char. 79: 0 → 1
Char. 133: 0 → 1

**Node 68:**
Char. 100: 0 → 1
Char. 113: 0 → 1

**Node 69:**
Char. 70: 0 → 1
Char. 118: 0 → 1
Char. 125: 0 → 1

**Node 70:**
Char. 38: 1 → 2
Char. 39: 0 → 1
Char. 50: 1 → 0
Char. 58: 0 → 1
Char. 59: 0 → 1
Char. 60: 0 → 1
Char. 72: 1 → 2
Char. 88: 1 → 0
Char. 95: 0 → 1
Char. 104: 0 → 1
Char. 105: 0 → 1
Char. 106: 0 → 2
Char. 107: 0 → 1
Char. 109: 0 → 1
Char. 110: 0 → 1
Char. 146: 1 → 0
Char. 155: 0 → 2
Char. 183: 1 → 2

**Node 71:**
Char. 23: 0 → 1
Char. 33: 1 → 0
Char. 38: 2 → 1
Char. 39: 1 → 0
Char. 42: 0 → 1
Char. 43: 0 → 1
Char. 46: 1 → 0
Char. 48: 1 → 0
Char. 49: 1 → 0
Char. 71: 1 → 0
Char. 83: 0 → 2
Char. 101: 0 → 1
Char. 103: 0 → 1
Char. 106: 0 → 1
Char. 161: 0 → 1
Char. 163: 1 → 0
Char. 172: 0 → 2
Char. 188: 0 → 1
Char. 203: 1 → 2
Char. 212: 0 → 1
Char. 216: 1 → 0
Char. 236: 0 → 1
Char. 238: 0 → 2
Char. 241: 0 → 1
Char. 242: 0 → 1
Char. 273: 0 → 1
Char. 274: 0 → 1
Char. 275: 1 → 0

**Node 72:**
Char. 52: 0 → 1
Char. 75: 0 → 1
Char. 84: 1 → 0
Char. 87: 0 → 1
Char. 110: 0 → 1
Char. 121: 0 → 1
Char. 230: 0 → 1

**Node 73:**
Char. 29: 1 → 0
Char. 44: 1 → 0
Char. 48: 1 → 0
Char. 59: 0 → 1
Char. 60: 0 → 1
Char. 66: 0 → 1
Char. 67: 0 → 1
Char. 72: 1 → 2
Char. 78: 1 → 0
Char. 84: 1 → 0
Char. 88: 1 → 0
Char. 93: 1 → 0
Char. 111: 0 → 1
Char. 123: 1 → 0
Char. 129: 0 → 1
Char. 154: 1 → 0
Char. 169: 1 → 0
Char. 170: 0 → 1
Char. 197: 0 → 1

**Node 74:**
Char. 107: 0 → 1

**Node 75:**
Char. 2: 0 → 1
Char. 6: 0 → 1
Char. 186: 0 → 1
Char. 198: 0 → 1
Char. 200: 0 → 1
Char. 220: 0 → 1
Char. 239: 1 → 0

**Node 76:**
Char. 98: 1 → 0
Char. 113: 0 → 1
Char. 181: 0 → 1
Char. 206: 0 → 2

**Node 77:**
Char. 35: 0 → 1
Char. 102: 0 → 1
Char. 103: 0 → 1
Char. 184: 0 → 1
Char. 201: 0 → 1
Char. 210: 0 → 1
Char. 223: 0 → 1
Char. 229: 0 → 1

**Node 78:**
Char. 17: 0 → 1
Char. 42: 0 → 1
Char. 264: 0 → 1

**Node 79:**
Char. 41: 0 → 1
Char. 61: 1 → 2
Char. 112: 1 → 0
Char. 138: 0 → 1
Char. 146: 1 → 0
Char. 155: 0 → 1
Char. 192: 1 → 2
Char. 226: 0 → 1
Char. 227: 0 → 1
Char. 233: 0 → 1
Char. 267: 01 → 2

**Node 80:**
Char. 61: 1 → 3
Char. 90: 0 → 1
Char. 91: 0 → 1

Char. 109: 1 → 0
Char. 155: 0 → 1
Char. 209: 1 → 0

**ANALYSIS 91**
**(NO *CANDELARIA BARBOURI* AND *EUNOTOSAURUS AFRICANUS*, NO CHARACTERS 174, 247, 251, AND 253)**

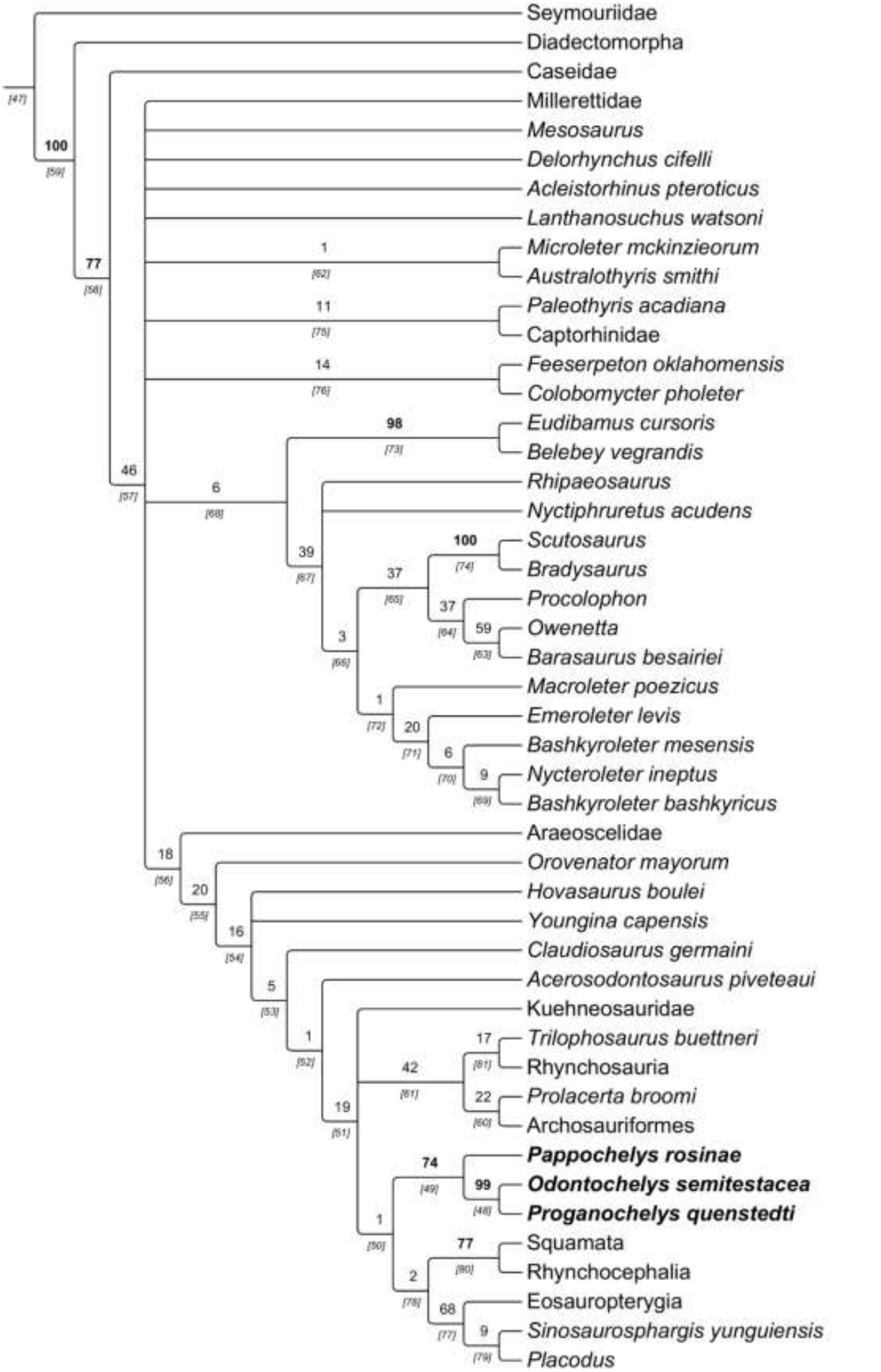

***Proganochelys quenstedti*:**
Char. 8: 0 → 1
Char. 11: 0 → 1
Char. 108: 1 → 0
Char. 128: 0 → 1
Char. 175: 0 → 1
Char. 202: 1 → 0
Char. 206: 2 → 0
Char. 207: 1 → 0
Char. 209: 1 → 0
Char. 235: 2 → 0
Char. 244: 0 → 1
Char. 248: 0 → 1
Char. 250: 0 → 1
Char. 262: 0 → 1

***Pappochelys rosinae*:**
Char. 1: 0 → 1
Char. 5: 1 → 0
Char. 12: 0 → 1
Char. 41: 0 → 1
Char. 49: 0 → 1
Char. 55: 1 → 0
Char. 67: 1 → 0
Char. 169: 1 → 0
Char. 176: 1 → 0

***Odontochelys semitestacea*:**
Char. 109: 1 → 0
Char. 112: 1 → 0
Char. 124: 0 → 1
Char. 126: 1 → 0

***Acerosodontosaurus piveteaui*:**
Char. 78: 1 → 0
Char. 128: 0 → 1
Char. 267: 1 → 0
Char. 278: 0 → 1

***Acleistorhinus pteroticus*:**
Char. 20: 0 → 1
Char. 21: 0 → 1
Char. 30: 0 → 1
Char. 33: 0 → 1
Char. 47: 0 → 1
Char. 55: 1 → 0
Char. 56: 0 → 1
Char. 64: 0 → 1
Char. 70: 0 → 1
Char. 79: 0 → 1
Char. 95: 0 → 1
Char. 110: 0 → 1
Char. 113: 0 → 1
Char. 114: 0 → 1
Char. 131: 0 → 1
Char. 137: 0 → 1
Char. 140: 0 → 2
Char. 146: 1 → 0
Char. 159: 0 → 1
Char. 166: 0 → 1
Char. 169: 1 → 0
Char. 170: 0 → 1
Char. 278: 3 → 1

**Araeoscelidae:**
Char. 5: 1 → 0
Char. 23: 0 → 1
Char. 28: 0 → 1
Char. 106: 0 → 1
Char. 116: 1 → 0
Char. 169: 1 → 0
Char. 170: 0 → 1
Char. 197: 0 → 1
Char. 239: 0 → 1

**Archosauriformes:**
Char. 32: 0 → 1
Char. 94: 1 → 0
Char. 112: 1 → 0
Char. 123: 1 → 0
Char. 152: 0 → 1
Char. 154: 0 → 2
Char. 166: 1 → 0
Char. 171: 0 → 1
Char. 185: 0 → 1
Char. 204: 0 → 1
Char. 218: 0 → 3
Char. 242: 0 → 1

***Australothyris smithi*:**
Char. 23: 0 → 1
Char. 34: 0 → 1
Char. 55: 1 → 0
Char. 71: 1 → 0
Char. 85: 1 → 0
Char. 112: 0 → 1
Char. 129: 0 → 1
Char. 131: 0 → 1
Char. 166: 0 → 1

***Barasaurus besairiei:***
Char. 33: 1 → 0
Char. 75: 1 → 0
Char. 216: 0 → 1

***Bashkyroleter bashkyricus:***
Char. 275: 1 → 0

***Bashkyroleter mesensis*:**
Char. 169: 1 → 0

***Belebey vegrandis:***
Char. 154: 1 → 0

***Bradysaurus* spp.:**
Char. 19: 0 → 1
Char. 73: 0 → 1
Char. 79: 0 → 1
Char. 135: 1 → 0
Char. 249: 0 → 1

**Captorhinidae:**
Char. 3: 0 → 1
Char. 23: 0 → 1
Char. 25: 1 → 0
Char. 26: 0 → 1
Char. 73: 0 → 1
Char. 75: 0 → 1
Char. 83: 0 → 1
Char. 108: 1 → 0
Char. 183: 1 → 0
Char. 201: 1 → 0
Char. 203: 1 → 2
Char. 216: 1 → 0

**Caseidae:**
Char. 24: 0 → 1
Char. 25: 1 → 0
Char. 36: 0 → 1
Char. 38: 1 → 0
Char. 46: 1 → 0
Char. 50: 1 → 0
Char. 56: 0 → 1
Char. 170: 0 → 1
Char. 194: 0 → 1
Char. 273: 0 → 1
Char. 274: 0 → 1
Char. 278: 3 → 2

***Claudiosaurus germaini:***
Char. 24: 0 → 1
Char. 34: 0 → 1
Char. 36: 0 → 1
Char. 56: 0 → 1
Char. 64: 0 → 1
Char. 105: 0 → 1
Char. 130: 0 → 1
Char. 144: 1 → 0
Char. 187: 1 → 0
Char. 199: 0 → 1
Char. 203: 1 → 2
Char. 204: 0 → 1
Char. 220: 0 → 1
Char. 222: 1 → 0
Char. 234: 1 → 0

***Colobomycter pholeter:***
Char. 21: 0 → 1
Char. 25: 1 → 0
Char. 84: 1 → 0
Char. 154: 1 → 0
Char. 167: 0 → 1
Char. 267: 0 → 1

***Delorhynchus cifelli:***
Char. 18: 0 → 1
Char. 20: 0 → 1
Char. 21: 0 → 1
Char. 24: 0 → 1
Char. 26: 0 → 1
Char. 28: 0 → 1
Char. 33: 0 → 2
Char. 39: 0 → 1
Char. 52: 0 → 1
Char. 100: 0 → 1
Char. 111: 0 → 1
Char. 116: 1 → 0
Char. 117: 0 → 1
Char. 131: 0 → 1
Char. 156: 1 → 0
Char. 159: 0 → 1
Char. 166: 0 → 1
Char. 167: 0 → 1
Char. 180: 0 → 1
Char. 189: 0 → 1
Char. 191: 0 → 1
Char. 192: 0 → 1
Char. 204: 0 → 2
Char. 267: 0 → 1

**Diadectomorpha:**
Char. 0: 0 → 1
Char. 64: 0 → 1
Char. 70: 0 → 1
Char. 122: 0 → 1
Char. 123: 1 → 0
Char. 146: 1 → 0
Char. 275: 1 → 0
Char. 278: 3 → 0

***Emeroleter levis:***
Char. 51: 1 → 0

**Eosauropterygia:**
Char. 158: 1 → 0
Char. 159: 1 → 0
Char. 166: 1 → 0
Char. 168: 0 → 1
Char. 194: 0 → 2
Char. 272: 1 → 0

***Eudibamus cursoris:***
Char. 154: 1 → 2

***Feeserpeton oklahomensis:***
Char. 51: 0 → 1
Char. 70: 0 → 1
Char. 157: 0 → 1
Char. 158: 0 → 1

***Hovasaurus boulei:***
Char. 41: 0 → 1
Char. 55: 1 → 0
Char. 60: 1 → 0
Char. 72: 2 → 1
Char. 77: 0 → 2
Char. 78: 1 → 0
Char. 79: 0 → 1
Char. 93: 1 → 0
Char. 113: 0 → 1
Char. 138: 0 → 1
Char. 146: 1 → 0
Char. 154: 0 → 1
Char. 204: 0 → 2
Char. 206: 0 → 2
Char. 220: 0 → 1
Char. 278: 0 → 3

**Kuehneosauridae:**
Char. 7: 0 → 1
Char. 24: 0 → 1
Char. 34: 0 → 1
Char. 35: 0 → 1
Char. 36: 0 → 1
Char. 56: 0 → 1
Char. 79: 0 → 2
Char. 82: 0 → 1
Char. 98: 1 → 0
Char. 108: 1 → 0
Char. 113: 0 → 1
Char. 128: 0 → 1
Char. 148: 1 → 0
Char. 159: 1 → 0
Char. 165: 0 → 1
Char. 185: 0 → 1
Char. 245: 0 → 1
Char. 272: 1 → 2
Char. 278: 0 → 3

***Lanthanosuchus watsoni:***
Char. 25: 1 → 0
Char. 51: 0 → 1
Char. 76: 0 → 1
Char. 86: 0 → 1
Char. 95: 0 → 1
Char. 98: 1 → 0
Char. 110: 0 → 1
Char. 113: 0 → 1
Char. 114: 0 → 1
Char. 131: 0 → 1
Char. 137: 0 → 1
Char. 138: 0 → 1
Char. 140: 0 → 2
Char. 144: 1 → 0
Char. 154: 1 → 2
Char. 192: 0 → 1

***Macroleter poezicus:***
Char. 52: 0 → 1
Char. 84: 1 → 0
Char. 87: 0 → 1
Char. 140: 0 → 1
Char. 146: 1 → 0
Char. 169: 1 → 0
Char. 235: 0 → 1

***Mesosaurus* spp.:**
Char. 0: 0 → 1
Char. 2: 0 → 1
Char. 5: 1 → 0
Char. 6: 0 → 1
Char. 8: 0 → 1
Char. 9: 0 → 1
Char. 13: 0 → 1
Char. 19: 0 → 1
Char. 23: 0 → 1
Char. 26: 0 → 1
Char. 29: 1 → 0
Char. 33: 0 → 1
Char. 38: 1 → 0
Char. 41: 0 → 1

Char. 50: 1 → 0
Char. 67: 0 → 1
Char. 76: 0 → 1
Char. 83: 0 → 1
Char. 84: 1 → 0
Char. 85: 1 → 0
Char. 94: 0 → 1
Char. 107: 0 → 1
Char. 109: 0 → 1
Char. 111: 0 → 1
Char. 115: 0 → 1
Char. 146: 1 → 0
Char. 148: 0 → 1
Char. 149: 1 → 0
Char. 164: 0 → 1
Char. 167: 0 → 1
Char. 176: 0 → 1
Char. 183: 1 → 0
Char. 184: 0 → 1
Char. 199: 0 → 1
Char. 202: 0 → 1
Char. 204: 0 → 2
Char. 206: 0 → 1
Char. 207: 0 → 1
Char. 209: 0 → 1
Char. 217: 0 → 1
Char. 219: 0 → 1
Char. 220: 0 → 1
Char. 231: 0 → 1
Char. 272: 2 → 0
Char. 278: 3 → 0

***Microleter mckinzieorum:***
Char. 0: 0 → 1
Char. 25: 1 → 0
Char. 36: 0 → 1
Char. 39: 0 → 1
Char. 51: 0 → 2
Char. 56: 0 → 1
Char. 70: 0 → 1
Char. 94: 0 → 1
Char. 106: 0 → 1
Char. 159: 0 → 1
Char. 276: 0 → 1

**Millerettidae:**
Char. 5: 1 → 0
Char. 24: 0 → 1
Char. 25: 1 → 0
Char. 44: 1 → 0
Char. 56: 0 → 1
Char. 57: 1 → 0
Char. 66: 0 → 2
Char. 78: 1 → 0
Char. 80: 1 → 0
Char. 84: 1 → 2
Char. 88: 1 → 0
Char. 96: 1 → 0
Char. 117: 0 → 1
Char. 124: 0 → 1
Char. 127: 0 → 1
Char. 135: 0 → 1
Char. 145: 0 → 1
Char. 202: 0 → 1
Char. 211: 0 → 1
Char. 230: 0 → 1
Char. 234: 0 → 1
Char. 248: 0 → 1
Char. 252: 0 → 1

***Nycteroleter ineptus:***
Char. 278: 0 → 3

***Nyctiphruretus acudens:***
Char. 21: 0 → 1
Char. 33: 1 → 2
Char. 41: 0 → 1
Char. 81: 1 → 0
Char. 84: 1 → 2
Char. 94: 0 → 1
Char. 167: 0 → 1
Char. 215: 1 → 0
Char. 224: 0 → 1
Char. 266: 0 → 1
Char. 272: 2 → 1
Char. 276: 0 → 1

***Orovenator mayorum:***
Char. 8: 0 → 1
Char. 24: 0 → 1
Char. 36: 0 → 1
Char. 160: 0 → 1
Char. 165: 0 → 1

***Owenetta* spp.:**
Char. 169: 1 → 0

***Paleothyris acadiana:***
Char. 38: 1 → 0
Char. 50: 1 → 0
Char. 66: 1 → 2
Char. 102: 0 → 1
Char. 146: 1 → 0
Char. 180: 0 → 1
Char. 237: 0 → 1
Char. 239: 0 → 1

***Placodus* spp.:**
Char. 9: 0 → 1
Char. 12: 0 → 1
Char. 13: 0 → 1
Char. 19: 0 → 1
Char. 31: 0 → 1
Char. 46: 1 → 0
Char. 57: 0 → 1
Char. 78: 1 → 0
Char. 93: 1 → 0
Char. 102: 1 → 2
Char. 109: 1 → 0
Char. 140: 1 → 0
Char. 163: 1 → 0
Char. 164: 0 → 1

***Procolophon* spp.:**
Char. 41: 0 → 1
Char. 69: 0 → 1
Char. 79: 0 → 1
Char. 88: 0 → 1
Char. 117: 1 → 0
Char. 149: 1 → 0
Char. 180: 0 → 1
Char. 204: 0 → 1
Char. 237: 0 → 1
Char. 238: 0 → 1
Char. 272: 2 → 1
Char. 278: 3 → 0

***Prolacerta broomi:***
Char. 58: 1 → 0
Char. 66: 1 → 0
Char. 67: 1 → 0
Char. 80: 1 → 0
Char. 139: 1 → 0
Char. 192: 1 → 0
Char. 203: 1 → 2

***Rhipaeosaurus* spp.:**
Char. 172: 0 → 1
Char. 277: 0 → 1

**Rhynchocephalia:**
Char. 24: 0 → 1
Char. 77: 0 → 1
Char. 94: 1 → 0
Char. 117: 12 → 0
Char. 139: 1 → 0
Char. 167: 1 → 0
Char. 200: 1 → 0
Char. 205: 1 → 0

**Rhynchosauria:**
Char. 0: 1 → 0
Char. 7: 0 → 1
Char. 9: 0 → 1
Char. 68: 0 → 1
Char. 99: 1 → 0
Char. 150: 1 → 0
Char. 160: 0 → 1
Char. 161: 0 → 1
Char. 171: 0 → 2
Char. 223: 0 → 1
Char. 241: 0 → 1

***Scutosaurus* spp.:**
Char. 175: 0 → 1
Char. 218: 0 → 1
Char. 243: 0 → 2
Char. 244: 0 → 1

***Sinosaurosphargis yunguiensis*:**
Char. 8: 0 → 1
Char. 30: 0 → 1
Char. 53: 0 → 1
Char. 82: 0 → 1
Char. 89: 1 → 0
Char. 127: 1 → 0
Char. 150: 1 → 0
Char. 154: 0 → 2
Char. 167: 1 → 0
Char. 255: 0 → 1

**Squamata:**
Char. 45: 0 → 1
Char. 79: 0 → 2
Char. 80: 1 → 0
Char. 82: 0 → 1
Char. 92: 1 → 0
Char. 109: 1 → 0
Char. 160: 0 → 1
Char. 245: 0 → 1

***Trilophosaurus buettneri*:**
Char. 5: 1 → 0
Char. 11: 0 → 1
Char. 55: 1 → 0
Char. 93: 1 → 0
Char. 104: 0 → 1
Char. 113: 0 → 1
Char. 122: 0 → 1
Char. 123: 1 → 0
Char. 136: 1 → 0
Char. 144: 1 → 0
Char. 154: 0 → 1
Char. 157: 0 → 1
Char. 159: 1 → 0
Char. 177: 0 → 12
Char. 194: 0 → 1
Char. 203: 1 → 2
Char. 207: 1 → 0
Char. 208: 1 → 0
Char. 272: 1 → 0

***Youngina capensis:***
Char. 5: 1 → 0
Char. 21: 0 → 1
Char. 56: 0 → 1
Char. 75: 0 → 1
Char. 170: 0 → 1
Char. 211: 0 → 1
Char. 231: 0 → 1
Char. 239: 0 → 1

**Node 48:**
Char. 46: 1 → 0
Char. 50: 0 → 1
Char. 89: 1 → 0
Char. 93: 1 → 0
Char. 195: 0 → 2
Char. 198: 0 → 1
Char. 240: 1 → 0
Char. 246: 1 → 2
Char. 259: 0 → 1
Char. 265: 1 → 0
Char. 270: 0 → 1

**Node 49:**
Char. 61: 1 → 0
Char. 62: 1 → 0
Char. 65: 0 → 1
Char. 78: 1 → 0
Char. 194: 0 → 2
Char. 214: 1 → 0
Char. 219: 1 → 0
Char. 241: 0 → 1
Char. 246: 0 → 1
Char. 254: 0 → 1
Char. 255: 0 → 1
Char. 256: 0 → 1
Char. 268: 0 → 1
Char. 269: 0 → 1

**Node 50:**
Char. 21: 0 → 1
Char. 33: 1 → 2
Char. 158: 0 → 1
Char. 200: 0 → 1
Char. 223: 0 → 1
Char. 235: 1 → 2

**Node 51:**
Char. 176: 0 → 1
Char. 205: 0 → 1

**Node 52:**
Char. 81: 1 → 0
Char. 166: 0 → 1
Char. 206: 0 → 2
Char. 208: 0 → 1

**Node 53:**
Char. 23: 0 → 1
Char. 70: 0 → 1
Char. 73: 0 → 1
Char. 94: 0 → 1
Char. 131: 0 → 1
Char. 148: 0 → 1
Char. 272: 2 → 1

**Node 54:**
Char. 33: 0 → 1
Char. 135: 0 → 1
Char. 159: 0 → 1
Char. 267: 0 → 1
Char. 278: 1 → 0

**Node 55:**
Char. 20: 0 → 1
Char. 62: 0 → 1

**Node 56:**
Char. 0: 0 → 1
Char. 27: 0 → 1
Char. 29: 1 → 0
Char. 38: 1 → 0
Char. 40: 2 → 0
Char. 57: 1 → 0
Char. 59: 0 → 1
Char. 60: 0 → 1
Char. 67: 0 → 1
Char. 72: 1 → 2
Char. 84: 1 → 0
Char. 89: 0 → 1
Char. 111: 0 → 1
Char. 112: 0 → 1

Char. 120: 0 → 1
Char. 154: 1 → 0
Char. 180: 0 → 1
Char. 192: 0 → 1
Char. 193: 0 → 1
Char. 222: 0 → 1
Char. 224: 0 → 1
Char. 234: 0 → 1
Char. 237: 0 → 1
Char. 266: 0 → 1
Char. 278: 3 → 1

**Node 57:**
Char. 5: 0 → 1
Char. 29: 0 → 1
Char. 40: 0 → 2
Char. 74: 0 → 1
Char. 80: 0 → 1
Char. 84: 0 → 1
Char. 88: 0 → 1
Char. 116: 0 → 1
Char. 132: 0 → 1
Char. 149: 0 → 1
Char. 156: 0 → 1
Char. 169: 0 → 1
Char. 201: 0 → 1
Char. 203: 0 → 1
Char. 235: 0 → 1

**Node 58:**
Char. 72: 0 → 1
Char. 79: 1 → 0
Char. 81: 0 → 1
Char. 93: 0 → 1
Char. 97: 0 → 1
Char. 104: 1 → 0
Char. 144: 0 → 1
Char. 173: 0 → 1
Char. 183: 0 → 1

**Node 60:**
Char. 19: 0 → 1
Char. 92: 1 → 0

**Node 61:**
Char. 4: 0 → 1
Char. 29: 0 → 1
Char. 48: 1 → 0
Char. 107: 0 → 1
Char. 140: 0 → 1
Char. 213: 0 → 1
Char. 226: 0 → 2

Char. 228: 0 → 1
Char. 275: 0 → 1

**Node 62:**
Char. 24: 0 → 1
Char. 57: 1 → 0
Char. 79: 0 → 1
Char. 83: 0 → 1
Char. 110: 0 → 1
Char. 132: 1 → 0

**Node 63:**
Char. 73: 0 → 1
Char. 131: 1 → 0
Char. 205: 0 → 1
Char. 276: 0 → 1

**Node 64:**
Char. 18: 0 → 1
Char. 37: 0 → 1
Char. 107: 0 → 1
Char. 118: 1 → 0
Char. 125: 1 → 0
Char. 150: 0 → 1

**Node 65:**
Char. 23: 0 → 1
Char. 71: 1 → 0
Char. 102: 0 → 1
Char. 103: 0 → 1
Char. 106: 0 → 1
Char. 167: 0 → 1
Char. 214: 0 → 1
Char. 216: 1 → 0
Char. 235: 0 → 2
Char. 241: 0 → 1

**Node 66:**
Char. 138: 0 → 1
Char. 141: 0 → 1
Char. 207: 0 → 1

**Node 67:**
Char. 20: 0 → 1
Char. 33: 0 → 1
Char. 44: 1 → 0
Char. 70: 0 → 1
Char. 80: 1 → 0
Char. 117: 0 → 1
Char. 118: 0 → 1
Char. 125: 0 → 1
Char. 131: 0 → 1
Char. 194: 0 → 1

Char. 201: 1 → 0
Char. 211: 0 → 1

**Node 68:**
Char. 38: 1 → 2
Char. 39: 0 → 1
Char. 49: 0 → 1
Char. 66: 0 → 12
Char. 76: 0 → 1
Char. 85: 1 → 0
Char. 88: 1 → 0
Char. 95: 0 → 1
Char. 112: 0 → 1
Char. 148: 0 → 1
Char. 158: 0 → 1
Char. 159: 0 → 1
Char. 183: 1 → 2
Char. 192: 0 → 1
Char. 215: 0 → 1

**Node 69:**
Char. 87: 0 → 1

**Node 70:**
Char. 25: 1 → 0
Char. 76: 1 → 0

**Node 71:**
Char. 79: 0 → 1
Char. 93: 1 → 0
Char. 133: 0 → 1

**Node 72:**
Char. 85: 0 → 1
Char. 147: 1 → 2
Char. 154: 1 → 0
Char. 278: 3 → 0

**Node 73:**
Char. 50: 1 → 0
Char. 58: 0 → 1
Char. 59: 0 → 1
Char. 60: 0 → 1
Char. 72: 1 → 2
Char. 104: 0 → 1
Char. 105: 0 → 1
Char. 106: 0 → 2
Char. 107: 0 → 1
Char. 109: 0 → 1
Char. 110: 0 → 1
Char. 146: 1 → 0
Char. 155: 0 → 2

**Node 74:**
Char. 33: 1 → 0
Char. 38: 2 → 1
Char. 39: 1 → 0
Char. 42: 0 → 1
Char. 43: 0 → 1
Char. 46: 1 → 0
Char. 49: 1 → 0
Char. 52: 0 → 1
Char. 83: 01 → 2
Char. 84: 1 → 0
Char. 87: 0 → 1
Char. 93: 1 → 0
Char. 161: 0 → 1
Char. 163: 1 → 0
Char. 172: 0 → 2
Char. 188: 0 → 1
Char. 189: 0 → 1
Char. 195: 0 → 1
Char. 204: 0 → 2
Char. 212: 0 → 1
Char. 236: 0 → 1
Char. 238: 0 → 2
Char. 242: 0 → 1
Char. 274: 0 → 1
Char. 275: 1 → 0

**Node 75:**
Char. 5: 1 → 0
Char. 29: 1 → 0
Char. 44: 1 → 0
Char. 59: 0 → 1
Char. 60: 0 → 1
Char. 66: 0 → 1
Char. 67: 0 → 1
Char. 72: 1 → 2
Char. 78: 1 → 0
Char. 84: 1 → 0
Char. 88: 1 → 0
Char. 93: 1 → 0
Char. 111: 0 → 1
Char. 123: 1 → 0
Char. 129: 0 → 1
Char. 154: 1 → 0
Char. 169: 1 → 0
Char. 170: 0 → 1
Char. 197: 0 → 1

**Node 76:**
Char. 20: 0 → 1
Char. 47: 0 → 1
Char. 83: 0 → 1
Char. 85: 1 → 0

Char. 107: 0 → 1
Char. 110: 0 → 1
Char. 169: 1 → 0
Char. 170: 0 → 1

**Node 77:**
Char. 2: 0 → 1
Char. 6: 0 → 1
Char. 98: 1 → 0
Char. 101: 0 → 1
Char. 113: 0 → 1
Char. 186: 0 → 1
Char. 198: 0 → 1
Char. 231: 1 → 0
Char. 232: 1 → 0
Char. 234: 1 → 0
Char. 240: 1 → 0
Char. 266: 1 → 0

**Node 78:**
Char. 35: 0 → 1
Char. 68: 0 → 1
Char. 103: 0 → 1
Char. 107: 0 → 1
Char. 123: 1 → 0
Char. 140: 0 → 1
Char. 229: 0 → 1

**Node 79:**
Char. 17: 0 → 1
Char. 42: 0 → 1
Char. 264: 0 → 1

**Node 80:**
Char. 41: 0 → 1
Char. 48: 1 → 0
Char. 61: 1 → 2
Char. 112: 1 → 0
Char. 128: 0 → 1
Char. 146: 1 → 0
Char. 165: 0 → 1
Char. 192: 1 → 2
Char. 206: 2 → 0
Char. 233: 0 → 1

**Node 81:**
Char. 61: 1 → 3
Char. 90: 0 → 1
Char. 91: 0 → 1
Char. 109: 1 → 0
Char. 209: 1 → 0

## ANALYSIS 92
### (NO *CANDELARIA BARBOURI* AND *EUNOTOSAURUS AFRICANUS*, NO CHARACTERS 174, 247, 251, AND 253)

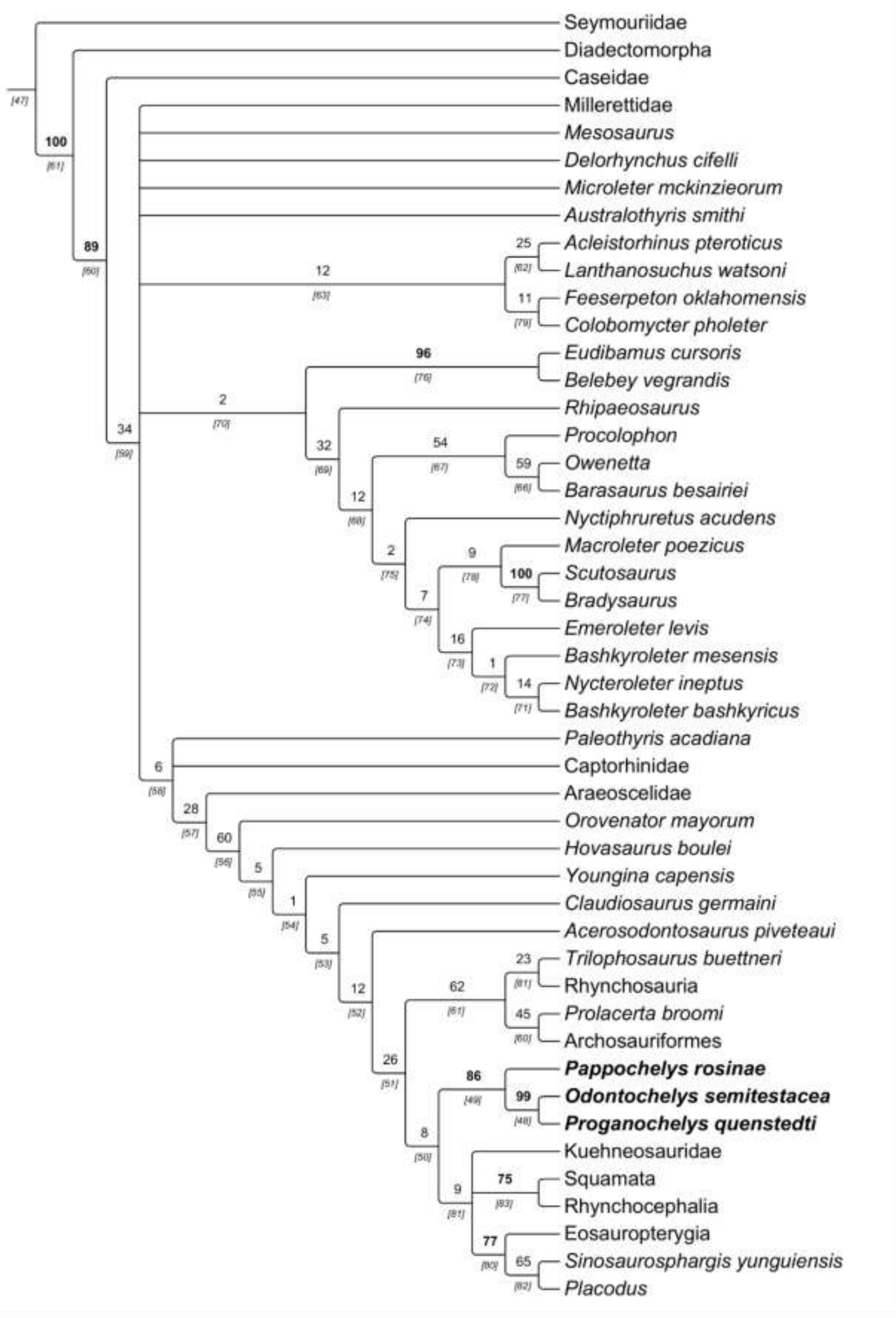

***Proganochelys quenstedti*:**
Char. 8: 0 → 1
Char. 11: 0 → 1
Char. 128: 0 → 1
Char. 175: 0 → 1
Char. 202: 1 → 0
Char. 206: 2 → 0
Char. 207: 1 → 0
Char. 209: 1 → 0
Char. 235: 2 → 0
Char. 244: 0 → 1
Char. 248: 0 → 1
Char. 250: 0 → 1
Char. 262: 0 → 1

***Pappochelys rosinae*:**
Char. 1: 0 → 1
Char. 5: 1 → 0
Char. 12: 0 → 1
Char. 41: 0 → 1
Char. 49: 0 → 1
Char. 55: 1 → 0
Char. 67: 1 → 0
Char. 81: 0 → 1
Char. 169: 1 → 0
Char. 176: 1 → 0

***Odontochelys semitestacea*:**
Char. 109: 1 → 0
Char. 112: 1 → 0
Char. 124: 0 → 1
Char. 126: 1 → 0

***Acerosodontosaurus piveteaui*:**
Char. 78: 1 → 0
Char. 128: 0 → 1
Char. 267: 1 → 0
Char. 278: 0 → 1

***Acleistorhinus pteroticus*:**
Char. 21: 0 → 1
Char. 119: 0 → 1
Char. 146: 1 → 0

**Araeoscelidae:**
Char. 23: 0 → 1
Char. 28: 0 → 1
Char. 106: 0 → 1
Char. 129: 1 → 0
Char. 239: 0 → 1

**Archosauriformes:**
Char. 32: 0 → 1
Char. 94: 1 → 0
Char. 112: 1 → 0
Char. 123: 1 → 0
Char. 152: 0 → 1
Char. 154: 0 → 2
Char. 166: 1 → 0
Char. 171: 0 → 1
Char. 185: 0 → 1
Char. 201: 0 → 1
Char. 204: 0 → 1
Char. 218: 0 → 3
Char. 242: 0 → 1

***Australothyris smithi*:**
Char. 23: 0 → 1
Char. 34: 0 → 1
Char. 55: 1 → 0
Char. 57: 1 → 0
Char. 71: 1 → 0
Char. 79: 0 → 1
Char. 98: 1 → 0
Char. 100: 0 → 1
Char. 103: 0 → 1
Char. 110: 0 → 1
Char. 112: 0 → 1
Char. 123: 1 → 0
Char. 129: 0 → 1
Char. 131: 0 → 1
Char. 132: 1 → 0
Char. 144: 1 → 0
Char. 147: 0 → 1
Char. 149: 1 → 0
Char. 150: 0 → 1
Char. 166: 0 → 1

***Barasaurus besairiei*:**
Char. 33: 1 → 0

***Bashkyroleter bashkyricus*:**
Char. 275: 1 → 0

***Bashkyroleter mesensis*:**
Char. 169: 1 → 0

***Belebey vegrandis*:**
Char. 154: 1 → 0

***Bradysaurus* spp.:**
Char. 19: 0 → 1
Char. 25: 1 → 0
Char. 73: 0 → 1
Char. 79: 0 → 1
Char. 135: 1 → 0
Char. 249: 0 → 1

**Captorhinidae:**
Char. 3: 0 → 1
Char. 23: 0 → 1
Char. 25: 1 → 0
Char. 26: 0 → 1
Char. 51: 0 → 2
Char. 73: 0 → 1
Char. 75: 0 → 1
Char. 108: 1 → 0
Char. 115: 0 → 1
Char. 121: 0 → 1
Char. 183: 1 → 0
Char. 201: 1 → 0
Char. 203: 1 → 2
Char. 216: 1 → 0

**Caseidae:**
Char. 25: 1 → 0
Char. 36: 0 → 1
Char. 38: 1 → 0
Char. 46: 1 → 0
Char. 50: 1 → 0
Char. 56: 0 → 1
Char. 170: 0 → 1
Char. 194: 0 → 1
Char. 273: 0 → 1
Char. 274: 0 → 1
Char. 278: 3 → 2

***Claudiosaurus germaini*:**
Char. 24: 0 → 1
Char. 34: 0 → 1
Char. 36: 0 → 1
Char. 43: 1 → 0
Char. 64: 0 → 1
Char. 105: 0 → 1
Char. 130: 0 → 1
Char. 144: 1 → 0
Char. 187: 1 → 0
Char. 199: 0 → 1
Char. 201: 0 → 1
Char. 204: 0 → 1
Char. 220: 0 → 1
Char. 222: 1 → 0
Char. 234: 1 → 0

***Colobomycter pholeter*:**
Char. 21: 0 → 1
Char. 25: 1 → 0
Char. 84: 1 → 0
Char. 154: 1 → 0
Char. 167: 0 → 1
Char. 267: 0 → 1

***Delorhynchus cifelli*:**
Char. 5: 0 → 1
Char. 18: 0 → 1
Char. 20: 0 → 1
Char. 21: 0 → 1
Char. 26: 0 → 1
Char. 28: 0 → 1
Char. 33: 0 → 2
Char. 39: 0 → 1
Char. 48: 0 → 1
Char. 52: 0 → 1
Char. 100: 0 → 1
Char. 111: 0 → 1
Char. 116: 1 → 0
Char. 131: 0 → 1
Char. 147: 0 → 1
Char. 156: 1 → 0
Char. 159: 0 → 1
Char. 163: 0 → 1
Char. 166: 0 → 1
Char. 167: 0 → 1
Char. 189: 0 → 1
Char. 191: 0 → 1
Char. 192: 0 → 1
Char. 267: 0 → 1

**Diadectomorpha:**
Char. 0: 0 → 1
Char. 64: 0 → 1
Char. 70: 0 → 1
Char. 122: 0 → 1
Char. 123: 1 → 0
Char. 146: 1 → 0
Char. 275: 1 → 0
Char. 278: 3 → 0

***Emeroleter levis*:**
Char. 51: 1 → 0

**Eosauropterygia:**
Char. 158: 1 → 0
Char. 159: 1 → 0
Char. 166: 1 → 0
Char. 168: 0 → 1
Char. 178: 0 → 1
Char. 194: 0 → 2
Char. 272: 1 → 0

***Eudibamus cursoris*:**
Char. 154: 1 → 2

***Feeserpeton oklahomensis*:**
Char. 51: 0 → 1
Char. 157: 0 → 1
Char. 158: 0 → 1

***Hovasaurus boulei*:**
Char. 41: 0 → 1
Char. 55: 1 → 0
Char. 60: 1 → 0
Char. 72: 2 → 1
Char. 77: 0 → 2
Char. 78: 1 → 0
Char. 79: 0 → 1
Char. 93: 1 → 0
Char. 113: 0 → 1
Char. 138: 0 → 1
Char. 146: 1 → 0
Char. 154: 0 → 1
Char. 204: 0 → 2
Char. 206: 0 → 2
Char. 220: 0 → 1

**Kuehneosauridae:**
Char. 7: 0 → 1
Char. 21: 1 → 0
Char. 24: 0 → 1
Char. 27: 0 → 1
Char. 33: 2 → 1
Char. 34: 0 → 1
Char. 36: 0 → 1
Char. 43: 1 → 0
Char. 79: 0 → 2
Char. 147: 1 → 0
Char. 148: 1 → 0
Char. 159: 1 → 0
Char. 168: 0 → 1
Char. 185: 0 → 1
Char. 235: 2 → 1
Char. 245: 0 → 1
Char. 272: 1 → 2
Char. 278: 0 → 3

***Lanthanosuchus watsoni*:**
Char. 25: 1 → 0
Char. 51: 0 → 1
Char. 76: 0 → 1
Char. 86: 0 → 1
Char. 98: 1 → 0
Char. 138: 0 → 1
Char. 144: 1 → 0
Char. 154: 1 → 2

***Macroleter poezicus*:**
Char. 9: 0 → 1
Char. 26: 0 → 1
Char. 66: 1 → 2
Char. 126: 0 → 1
Char. 134: 0 → 2
Char. 140: 0 → 1
Char. 146: 1 → 0
Char. 169: 1 → 0

***Mesosaurus* spp.:**
Char. 0: 0 → 1
Char. 2: 0 → 1
Char. 6: 0 → 1
Char. 8: 0 → 1
Char. 9: 0 → 1
Char. 13: 0 → 1
Char. 19: 0 → 1
Char. 23: 0 → 1
Char. 26: 0 → 1
Char. 29: 1 → 0
Char. 33: 0 → 1
Char. 38: 1 → 0
Char. 41: 0 → 1
Char. 48: 0 → 1
Char. 50: 1 → 0
Char. 67: 0 → 1
Char. 76: 0 → 1
Char. 84: 1 → 0
Char. 94: 0 → 1
Char. 107: 0 → 1
Char. 109: 0 → 1
Char. 111: 0 → 1
Char. 115: 0 → 1
Char. 146: 1 → 0
Char. 148: 0 → 1
Char. 149: 1 → 0
Char. 164: 0 → 1
Char. 167: 0 → 1
Char. 176: 0 → 1
Char. 183: 1 → 0
Char. 184: 0 → 1
Char. 199: 0 → 1
Char. 202: 0 → 1
Char. 206: 0 → 1
Char. 207: 0 → 1
Char. 209: 0 → 1
Char. 217: 0 → 1
Char. 219: 0 → 1

Char. 220: 0 → 1
Char. 231: 0 → 1
Char. 272: 2 → 0
Char. 278: 3 → 0

***Microleter mckinzieorum:***
Char. 0: 0 → 1
Char. 18: 0 → 1
Char. 25: 1 → 0
Char. 36: 0 → 1
Char. 39: 0 → 1
Char. 51: 0 → 2
Char. 56: 0 → 1
Char. 57: 1 → 0
Char. 70: 0 → 1
Char. 76: 0 → 1
Char. 79: 0 → 1
Char. 94: 0 → 1
Char. 106: 0 → 1
Char. 110: 0 → 1
Char. 132: 1 → 0
Char. 159: 0 → 1
Char. 276: 0 → 1
Char. 278: 3 → 1

**Millerettidae:**
Char. 25: 1 → 0
Char. 44: 1 → 0
Char. 56: 0 → 1
Char. 57: 1 → 0
Char. 66: 0 → 2
Char. 80: 1 → 0
Char. 84: 1 → 2
Char. 88: 1 → 0
Char. 96: 1 → 0
Char. 119: 0 → 1
Char. 121: 0 → 1
Char. 124: 0 → 1
Char. 127: 0 → 1
Char. 135: 0 → 1
Char. 145: 0 → 1
Char. 202: 0 → 1
Char. 211: 0 → 1
Char. 230: 0 → 1
Char. 234: 0 → 1
Char. 248: 0 → 1
Char. 252: 0 → 1

***Nycteroleter ineptus:***
Char. 278: 0 → 3

***Nyctiphruretus acudens:***
Char. 21: 0 → 1
Char. 41: 0 → 1
Char. 66: 1 → 2
Char. 81: 1 → 0
Char. 84: 1 → 2
Char. 94: 0 → 1
Char. 166: 1 → 0
Char. 215: 1 → 0
Char. 224: 0 → 1
Char. 226: 1 → 0
Char. 266: 0 → 1

Char. 272: 2 → 1
Char. 276: 0 → 1

***Orovenator mayorum:***
Char. 8: 0 → 1
Char. 24: 0 → 1
Char. 36: 0 → 1
Char. 160: 0 → 1
Char. 165: 0 → 1

***Owenetta* spp.:**
Char. 142: 1 → 0
Char. 169: 1 → 0

***Paleothyris acadiana:***
Char. 66: 01 → 2
Char. 102: 0 → 1
Char. 146: 1 → 0
Char. 239: 0 → 1

***Placodus* spp.:**
Char. 0: 1 → 2
Char. 9: 0 → 1
Char. 12: 0 → 1
Char. 13: 0 → 1
Char. 19: 0 → 1
Char. 31: 0 → 1
Char. 46: 1 → 0
Char. 57: 0 → 1
Char. 78: 1 → 0
Char. 93: 1 → 0
Char. 102: 1 → 2
Char. 109: 1 → 0
Char. 163: 1 → 0
Char. 164: 0 → 1

***Procolophon* spp.:**
Char. 41: 0 → 1
Char. 69: 0 → 1
Char. 79: 0 → 1
Char. 86: 0 → 1
Char. 88: 0 → 1
Char. 101: 0 → 1
Char. 141: 0 → 1
Char. 149: 1 → 0
Char. 203: 1 → 2
Char. 230: 0 → 1
Char. 237: 0 → 1
Char. 238: 0 → 1
Char. 239: 1 → 0
Char. 272: 2 → 1
Char. 278: 3 → 0

***Prolacerta broomi:***
Char. 66: 1 → 0
Char. 67: 1 → 0
Char. 80: 1 → 0
Char. 139: 1 → 0
Char. 147: 1 → 0
Char. 192: 1 → 0

***Rhipaeosaurus* spp.:**
Char. 172: 0 → 1
Char. 277: 0 → 1

**Rhynchocephalia:**
Char. 0: 1 → 2
Char. 23: 1 → 0
Char. 24: 0 → 1
Char. 26: 1 → 0
Char. 77: 0 → 1
Char. 81: 0 → 1
Char. 88: 1 → 0
Char. 94: 1 → 0
Char. 117: 12 → 0
Char. 139: 1 → 0
Char. 167: 1 → 0
Char. 205: 1 → 0

**Rhynchosauria:**
Char. 0: 1 → 0
Char. 7: 0 → 1
Char. 9: 0 → 1
Char. 26: 0 → 1
Char. 68: 0 → 1
Char. 99: 1 → 0
Char. 160: 0 → 1
Char. 161: 0 → 1
Char. 171: 0 → 2
Char. 223: 0 → 1
Char. 241: 0 → 1

***Scutosaurus* spp.:**
Char. 175: 0 → 1
Char. 190: 0 → 1
Char. 218: 0 → 1
Char. 243: 0 → 2
Char. 244: 0 → 1

***Sinosaurosphargis yunguiensis*:**
Char. 8: 0 → 1
Char. 26: 1 → 0
Char. 30: 0 → 1
Char. 44: 0 → 1
Char. 53: 0 → 1
Char. 89: 1 → 0
Char. 127: 1 → 0
Char. 150: 1 → 0
Char. 154: 0 → 2
Char. 167: 1 → 0
Char. 255: 0 → 1

**Squamata:**
Char. 45: 0 → 1
Char. 79: 0 → 2
Char. 80: 1 → 0
Char. 92: 1 → 0
Char. 109: 1 → 0
Char. 160: 0 → 1
Char. 245: 0 → 1

***Trilophosaurus buettneri*:**
Char. 5: 1 → 0
Char. 11: 0 → 1
Char. 44: 0 → 1
Char. 55: 1 → 0
Char. 93: 1 → 0
Char. 104: 0 → 1
Char. 113: 0 → 1
Char. 122: 0 → 1
Char. 123: 1 → 0
Char. 136: 1 → 0
Char. 144: 1 → 0
Char. 154: 0 → 1
Char. 157: 0 → 1
Char. 159: 1 → 0
Char. 177: 0 → 12
Char. 194: 0 → 1
Char. 207: 1 → 0
Char. 208: 1 → 0
Char. 272: 1 → 0

***Youngina capensis:***
Char. 75: 0 → 1
Char. 211: 0 → 1
Char. 239: 0 → 1

**Node 48:**
Char. 46: 1 → 0
Char. 50: 0 → 1
Char. 88: 1 → 0
Char. 89: 1 → 0
Char. 93: 1 → 0
Char. 195: 0 → 2
Char. 198: 0 → 1
Char. 240: 1 → 0
Char. 246: 1 → 2
Char. 259: 0 → 1
Char. 265: 1 → 0
Char. 270: 0 → 1

**Node 49:**
Char. 62: 1 → 0
Char. 78: 1 → 0
Char. 194: 0 → 2
Char. 214: 1 → 0
Char. 219: 1 → 0
Char. 220: 0 → 1
Char. 241: 0 → 1
Char. 246: 0 → 1
Char. 254: 0 → 1
Char. 255: 0 → 1
Char. 256: 0 → 1
Char. 268: 0 → 1
Char. 269: 0 → 1

**Node 50:**
Char. 26: 0 → 1
Char. 33: 1 → 2
Char. 158: 0 → 1
Char. 181: 0 → 1
Char. 184: 0 → 1
Char. 210: 0 → 1
Char. 223: 0 → 1
Char. 235: 1 → 2

**Node 51:**
Char. 176: 0 → 1
Char. 205: 0 → 1

**Node 52:**
Char. 81: 1 → 0
Char. 166: 0 → 1
Char. 208: 0 → 1

**Node 53:**
Char. 23: 0 → 1
Char. 70: 0 → 1
Char. 73: 0 → 1
Char. 94: 0 → 1
Char. 131: 0 → 1
Char. 148: 0 → 1
Char. 272: 2 → 1

**Node 54:**
Char. 44: 1 → 0
Char. 134: 0 → 1
Char. 214: 0 → 1
Char. 219: 0 → 1

**Node 55:**
Char. 135: 0 → 1

**Node 56:**
Char. 20: 0 → 1
Char. 62: 0 → 1
Char. 169: 0 → 1

**Node 57:**
Char. 0: 0 → 1
Char. 57: 1 → 0
Char. 89: 0 → 1
Char. 112: 0 → 1
Char. 120: 0 → 1
Char. 192: 0 → 1
Char. 193: 0 → 1
Char. 222: 0 → 1
Char. 224: 0 → 1
Char. 234: 0 → 1
Char. 266: 0 → 1

**Node 58:**
Char. 29: 1 → 0
Char. 59: 0 → 1
Char. 60: 0 → 1
Char. 67: 0 → 1
Char. 72: 1 → 2
Char. 84: 1 → 0
Char. 111: 0 → 1
Char. 119: 0 → 1
Char. 129: 0 → 1
Char. 154: 1 → 0
Char. 169: 1 → 0
Char. 170: 0 → 1
Char. 197: 0 → 1

**Node 59:**
Char. 29: 0 → 1

Char. 40: 0 → 2
Char. 74: 0 → 1
Char. 80: 0 → 1
Char. 84: 0 → 1
Char. 88: 0 → 1
Char. 116: 0 → 1
Char. 132: 0 → 1
Char. 149: 0 → 1
Char. 156: 0 → 1
Char. 169: 0 → 1
Char. 201: 0 → 1
Char. 203: 0 → 1
Char. 235: 0 → 1

**Node 60:**
Char. 72: 0 → 1
Char. 79: 1 → 0
Char. 81: 0 → 1
Char. 93: 0 → 1
Char. 97: 0 → 1
Char. 104: 1 → 0
Char. 144: 0 → 1
Char. 173: 0 → 1
Char. 183: 0 → 1

**Node 62:**
Char. 95: 0 → 1
Char. 113: 0 → 1
Char. 114: 0 → 1

**Node 63:**
Char. 5: 0 → 1
Char. 20: 0 → 1
Char. 47: 0 → 1
Char. 48: 0 → 1
Char. 79: 0 → 1
Char. 110: 0 → 1
Char. 131: 0 → 1
Char. 137: 0 → 1
Char. 140: 0 → 2
Char. 147: 0 → 1
Char. 159: 0 → 1
Char. 163: 0 → 1
Char. 166: 0 → 1

**Node 64:**
Char. 19: 0 → 1
Char. 92: 1 → 0

**Node 65:**
Char. 4: 0 → 1
Char. 29: 0 → 1
Char. 48: 1 → 0
Char. 213: 0 → 1
Char. 226: 0 → 2
Char. 228: 0 → 1
Char. 275: 0 → 1

**Node 66:**
Char. 73: 0 → 1
Char. 205: 0 → 1
Char. 273: 0 → 1
Char. 276: 0 → 1

**Node 67:**
Char. 18: 0 → 1
Char. 25: 1 → 0
Char. 37: 0 → 1
Char. 106: 0 → 1
Char. 107: 0 → 1
Char. 110: 0 → 1
Char. 126: 0 → 1
Char. 150: 0 → 1

**Node 68:**
Char. 186: 0 → 1

**Node 69:**
Char. 119: 0 → 1
Char. 194: 0 → 1
Char. 201: 1 → 0
Char. 211: 0 → 1
Char. 226: 0 → 1

**Node 70:**
Char. 5: 0 → 1
Char. 38: 1 → 2
Char. 39: 0 → 1
Char. 49: 0 → 1
Char. 66: 0 → 1
Char. 76: 0 → 1
Char. 88: 1 → 0
Char. 95: 0 → 1
Char. 112: 0 → 1
Char. 148: 0 → 1
Char. 158: 0 → 1
Char. 159: 0 → 1
Char. 166: 0 → 1
Char. 183: 1 → 2
Char. 192: 0 → 1
Char. 215: 0 → 1
Char. 239: 0 → 1

**Node 71:**
Char. 87: 0 → 1
Char. 110: 0 → 1

**Node 72:**
Char. 25: 1 → 0
Char. 76: 1 → 0

**Node 73:**
Char. 79: 0 → 1
Char. 133: 0 → 1

**Node 74:**
Char. 100: 0 → 1
Char. 113: 0 → 1

**Node 75:**
Char. 70: 0 → 1
Char. 118: 0 → 1
Char. 125: 0 → 1

**Node 76:**
Char. 58: 0 → 1
Char. 59: 0 → 1
Char. 60: 0 → 1
Char. 72: 1 → 2
Char. 104: 0 → 1
Char. 105: 0 → 1
Char. 106: 0 → 2
Char. 107: 0 → 1
Char. 109: 0 → 1
Char. 110: 0 → 1
Char. 146: 1 → 0

**Node 77:**
Char. 23: 0 → 1
Char. 33: 1 → 0
Char. 38: 2 → 1
Char. 39: 1 → 0
Char. 42: 0 → 1
Char. 43: 0 → 1
Char. 46: 1 → 0
Char. 49: 1 → 0
Char. 71: 1 → 0
Char. 83: 0 → 2
Char. 101: 0 → 1
Char. 103: 0 → 1
Char. 106: 0 → 1
Char. 161: 0 → 1
Char. 163: 1 → 0
Char. 172: 0 → 2
Char. 188: 0 → 1
Char. 203: 1 → 2
Char. 212: 0 → 1
Char. 216: 1 → 0
Char. 236: 0 → 1
Char. 238: 0 → 2
Char. 239: 1 → 0
Char. 241: 0 → 1
Char. 242: 0 → 1
Char. 273: 0 → 1
Char. 274: 0 → 1
Char. 275: 1 → 0

**Node 78:**
Char. 52: 0 → 1
Char. 75: 0 → 1
Char. 84: 1 → 0
Char. 87: 0 → 1
Char. 110: 0 → 1
Char. 121: 0 → 1
Char. 230: 0 → 1

**Node 79:**
Char. 107: 0 → 1

**Node 80:**
Char. 2: 0 → 1
Char. 6: 0 → 1
Char. 81: 0 → 1
Char. 101: 0 → 1
Char. 186: 0 → 1
Char. 198: 0 → 1
Char. 220: 0 → 1
Char. 231: 1 → 0
Char. 232: 1 → 0
Char. 234: 1 → 0
Char. 240: 1 → 0
Char. 266: 1 → 0
Char. 267: 1 → 0

**Node 81:**
Char. 35: 0 → 1
Char. 103: 0 → 1
Char. 123: 1 → 0
Char. 201: 0 → 1
Char. 229: 0 → 1

**Node 82:**
Char. 17: 0 → 1
Char. 42: 0 → 1
Char. 264: 0 → 1

**Node 83:**
Char. 41: 0 → 1
Char. 44: 0 → 1
Char. 48: 1 → 0
Char. 61: 1 → 2
Char. 112: 1 → 0
Char. 138: 0 → 1
Char. 146: 1 → 0
Char. 181: 1 → 0
Char. 192: 1 → 2
Char. 206: 2 → 0
Char. 226: 0 → 1
Char. 233: 0 → 1
Char. 267: 1 → 2

**Node 84:**
Char. 61: 1 → 3
Char. 90: 0 → 1
Char. 91: 0 → 1
Char. 109: 1 → 0
Char. 209: 1 → 0

# Tables

**Supplementary Table 2.** Number of obtained trees, best score (Tree Bisection-Reconnection), Consistency Index, Retention Index and bootstrap values (BS, 1000 replicates) for clades consisting of *Eunotosaurus africanus* and more derived pan-testudinates (ET), *Pappochelys rosinae* and more derived pan-testudinates (PT), and *Odontochelys semitestacea* and *Proganochelys quenstedti* (OT) from analyses 1-32 (1000 replicates, different combinations of taxa, no implied weighting). The most optimal values and position within Diapsida are indicated in green, the less optimal and position outside of Diapsida in red.

| # | *Proganochelys* | *Candelaria* | *Pappochelys* | *Odontochelys* | *Eunotosaurus* | Trees | Best score | Consistency Index | Retention Index | BS | | | Position of Pan-Testudinata |
|---|---|---|---|---|---|---|---|---|---|---|---|---|---|
| | | | | | | | | | | ET | PT | OT | |
| 1 | + | + | + | + | + | 47 | 1198 | 0.266 | 0.574 | 9 | 29 | **97** | In large polytomy |
| 2 | - | + | + | + | + | 2 | 1143 | 0.279 | 0.601 | 7 | 21 | - | (*Candelaria* (*Odontochelys* + *Pappochelys*)) with BS 5, in clade with *Eunotosaurus* in large polytomy |
| 3 | + | - | + | + | + | 2 | 1179 | 0.271 | 0.583 | 29 | 42 | **97** | In clade with *Acerosodontosaurus* with BS 1, in polytomy with *Claudiosaurus* and Sauria with BS 2 |
| 4 | + | + | - | + | + | 30 | 1175 | 0.271 | 0.585 | 62 | - | **98** | In polytomy with *Hovasaurus*, *Youngina*, *Acerosodontosaurus*, and (*Claudiosaurus* + Sauria) with BS 2 |
| 5 | + | + | + | - | + | 30 | 1191 | 0.268 | 0.578 | 1 | 11 | - | (*Candelaria* (*Eunotosaurus* (*Pappochelys* + *Proganochelys*))) with BS 1 in large polytomy |
| 6 | + | + | + | + | - | 17 | 1146 | 0.278 | 0.599 | - | 50 | **98** | In polytomy with Kuehneosauridae, Archosauromoprha, and (Lepidosauria + Sauropterygia) with BS 10 |
| 7 | - | - | + | + | + | 2 | 1127 | 0.283 | 0.609 | 15 | 62 | - | In polytomy with *Hovasaurus*, *Youngina*, *Acerosodontosaurus*, and (*Claudiosaurus* + Sauria) with BS 3 |
| 8 | - | + | - | + | + | 21 | 1125 | 0.284 | 0.609 | 42 | - | - | (*Candelaria* (*Eunotosaurus* + *Odontochelys*)) with BS 2 in large polytomy |
| 9 | - | + | + | - | + | 2 | 1114 | 0.286 | 0.615 | - | - | - | (*Eunotosaurus* + *Candelaria*) with BS 6 in large polytomy *Pappochelys* in polytomy with Kuehneosauridae, Archosauromorpha, Lepidosauria, and Sauropterygia with BS 11 |
| 10 | - | + | + | + | - | 154 | 1089 | 0.293 | 0.627 | - | 55 | - | In polytomy with Kuehneosauridae, Archosauromorpha, Lepidosauria and Sauropterygia with BS 12 |
| 11 | + | - | - | + | + | 6 | 1158 | 0.275 | 0.594 | **74** | - | **99** | In polytomy with *Acerosodontosaurus* and (*Clauriosaurus* + Sauria) with BS 1 |
| 12 | + | - | + | - | + | 2 | 1173 | 0.272 | 0.586 | 16 | 18 | - | In polytomy with *Acerosodontosaurus*, *Claudiosaurus*, and Sauria with BS 1 |
| 13 | + | - | + | + | - | 2 | 1127 | 0.283 | 0.609 | - | **76** | **98** | In polytomy with Lepidosauria and Sauropterygia with BS 1 |
| 14 | + | + | - | - | + | 30 | 1165 | 0.274 | 0.59 | 57 | - | - | In large polytomy |
| 15 | + | + | - | + | - | 299 | 1126 | 0.283 | 0.609 | - | - | **97** | In clade with Sauropterygia with BS 13 |
| 16 | + | + | + | - | - | 171 | 1137 | 0.281 | 0.604 | - | 47 | - | In polytomy with Kuehneosauridae, Archosauromoprha, and (Lepidosauria + Sauropterygia) with BS 8 |
| 17 | - | - | - | + | + | 28 | 1107 | 0.288 | 0.618 | 58 | - | - | In large polytomy |
| 18 | - | + | - | - | + | 3 | 1085 | 0.294 | 0.629 | - | - | - | (*Candelaria* + *Eunotosaurus*) with BS 9 in large polytomy |
| 19 | - | + | + | - | - | 139 | 1053 | 0.303 | 0.644 | - | - | - | In clade with Sauropterygia with BS 1 |
| 20 | - | - | + | - | + | 11 | 1097 | 0.291 | 0.623 | - | - | - | *Eunotosaurus* in large polytomy *Pappochelys* in clade with Sauropterygia with BS 6 |
| 21 | - | - | + | + | - | 4 | 1070 | 0.298 | 0.636 | - | **83** | - | In clade with Sauropterygia with BS 3 |
| 22 | - | + | - | + | - | 111 | 1070 | 0.298 | 0.636 | - | - | - | In clade with Sauropterygia with BS 1 |
| 23 | - | - | - | - | + | 10 | 1068 | 0.299 | 0.637 | - | - | - | *Eunotosaurus* in large polytomy |
| 24 | - | - | - | + | - | 6 | 1051 | 0.304 | 0.645 | - | - | - | In clade with Sauropterygia with BS 1 |
| 25 | - | - | + | - | - | 4 | 1034 | 0.309 | 0.654 | - | - | - | In clade with Sauropterygia with BS 8 |
| 26 | - | + | - | - | - | 186 | 1023 | 0.312 | 0.659 | - | - | - | |
| 27 | + | - | - | - | - | 8 | 1097 | 0.291 | 0.623 | - | - | - | In clade with Sauropterygia with BS 7 |
| 28 | + | + | - | - | - | 158 | 1116 | 0.286 | 0.614 | - | - | - | In clade with Sauropterygia with BS 6 |
| 29 | + | - | + | - | - | 6 | 1118 | 0.285 | 0.613 | - | **79** | - | In polytomy with Archosauromorpha and (Lepidosauria + Sauropterygia) with BS 2 |
| 30 | + | - | - | + | - | 14 | 1107 | 0.288 | 0.618 | - | - | **98** | In clade with Sauropterygia with BS 15 |
| 31 | + | - | - | - | + | 6 | 1148 | 0.278 | 0.598 | **72** | - | - | In polytomy with *Youngina*, (*Acerosodontosaurus* + *Hovasaurus*), and (*Claudiosaurus* + Sauria) with BS 3 |
| 32 | - | - | - | - | - | 6 | 1004 | 0.318 | 0.668 | - | - | - | |

**Supplementary Table 3.** K-values, number of obtained trees, best score (Tree Bisection-Reconnection), Consistency Index, Retention Index and bootstrap values (BS, 1000 replicates) for clades consisting of *Eunotosaurus africanus* and more derived pan-testudinates (ET), *Pappochelys rosinae* and more derived pan-testudinates (PT), and *Odontochelys semitestacea* and *Proganochelys quenstedti* (OT) from analyses 33-64 (1000 replicates, all taxa, implied weighting). The most optimal values and position within Diapsida are indicated in green, the less optimal and position outside of Diapsida in red.

| # | *Proganochelys* | *Candelaria* | *Pappochelys* | *Odontochelys* | *Eunotosaurus* | K | Trees | Best score | Consistency Index | Retention Index | BS | | | Position of Pan-Testudinata |
|---|---|---|---|---|---|---|---|---|---|---|---|---|---|---|
| | | | | | | | | | | | ET | PT | OT | |
| **33** | + | + | + | + | + | 1 | 1 | 172.44187 | 0.259 | 0.557 | 10 | 63 | **99** | In clade with *Candelaria* with BS 2, in large polytomy |
| **34** | + | + | + | + | + | 2 | 1 | 140.06369 | 0.262 | 0.564 | 15 | 67 | **99** | In polytomy with *Candelaria*, *Youngina*, *Orovenator*, *Hovasaurus*, *Claudiosaurus*, *Acerosodontosaurus*, and Sauria with BS 1 |
| **35** | + | + | + | + | + | 2.375 | 1 | 131.19086 | 0.262 | 0.565 | 16 | 69 | **99** | In large polytomy |
| **36** | + | + | + | + | + | 2.5 | 1 | 128.50642 | 0.262 | 0.565 | 17 | **70** | **99** | In polytomy with *Youngina*, *Orovenator*, *Hovasaurus*, *Claudiosaurus*, *Acerosodontosaurus*, and Sauria with BS 2 |
| **37** | + | + | + | + | + | 2.625 | 1 | 125.94249 | 0.262 | 0.565 | 15 | **70** | **99** | In polytomy with *Youngina*, *Orovenator*, *Hovasaurus*, *Claudiosaurus*, *Acerosodontosaurus*, and Sauria with BS 1 |
| **38** | + | + | + | + | + | 2.75 | 1 | 123.49036 | 0.262 | 0.565 | 15 | **71** | **99** | In polytomy with *Youngina*, *Orovenator*, *Hovasaurus*, *Claudiosaurus*, *Acerosodontosaurus*, and Sauria with BS 1 |
| **39** | + | + | + | + | + | 2.875 | 1 | 121.14222 | 0.262 | 0.565 | 16 | **70** | **99** | In polytomy with *Youngina*, *Orovenator*, *Hovasaurus*, *Claudiosaurus*, *Acerosodontosaurus*, and Sauria with BS 1 |
| **40** | + | + | + | + | + | 3 | 1 | 118.89103 | 0.262 | 0.565 | 13 | **70** | **99** | In polytomy with *Youngina*, *Orovenator*, *Hovasaurus*, *Claudiosaurus*, *Acerosodontosaurus*, and Sauria with BS 1 |
| **41** | + | + | + | + | + | 3.125 | 1 | 116.72797 | 0.263 | 0.566 | 13 | **71** | **99** | In polytomy with *Youngina*, *Orovenator*, *Hovasaurus*, *Claudiosaurus*, *Acerosodontosaurus*, and Sauria with BS 1 |
| **42** | + | + | + | + | + | 3.25 | 1 | 114.64962 | 0.263 | 0.566 | 14 | **71** | **99** | In polytomy with *Youngina*, *Orovenator*, *Hovasaurus*, *Claudiosaurus*, *Acerosodontosaurus*, and Sauria with BS 1 |
| **43** | + | + | + | + | + | 3.375 | 1 | 112.65085 | 0.263 | 0.566 | 14 | **71** | **99** | In polytomy with *Youngina*, *Orovenator*, *Hovasaurus*, *Claudiosaurus*, *Acerosodontosaurus*, and Sauria with BS 1 |
| **44** | + | + | + | + | + | 3.5 | 1 | 110.72684 | 0.263 | 0.566 | 14 | 69 | **99** | In polytomy with *Candelaria*, *Youngina*, *Orovenator*, *Hovasaurus*, *Acerosodontosaurus*, and (*Claudiosaurus* + Sauria) with BS 1 |
| **45** | + | + | + | + | + | 3.625 | 1 | 108.87316 | 0.263 | 0.566 | 14 | **70** | **99** | In large polytomy |
| **46** | + | + | + | + | + | 3.75 | 1 | 107.08576 | 0.263 | 0.566 | 14 | 69 | **99** | In polytomy with *Candelaria*, *Youngina*, *Orovenator*, *Hovasaurus*, *Claudiosaurus*, *Acerosodontosaurus*, and Sauria with BS 2 |
| **47** | + | + | + | + | + | 3.875 | 1 | 105.36088 | 0.263 | 0.566 | 15 | 69 | **99** | In polytomy with *Candelaria*, *Youngina*, *Orovenator*, *Hovasaurus*, *Claudiosaurus*, *Acerosodontosaurus*, and Sauria with BS 1 |
| **48** | + | + | + | + | + | 4 | 1 | 103.69509 | 0.263 | 0.566 | 16 | **70** | **99** | In polytomy with *Youngina*, *Orovenator*, *Hovasaurus*, *Claudiosaurus*, *Acerosodontosaurus*, and Sauria with BS 1 |
| **49** | + | + | + | + | + | 4.125 | 1 | 102.0852 | 0.263 | 0.566 | 14 | **71** | **99** | In polytomy with *Candelaria*, *Youngina*, *Orovenator*, *Hovasaurus*, *Claudiosaurus*, *Acerosodontosaurus*, and Sauria with BS 2 |
| **50** | + | + | + | + | + | 4.25 | 1 | 100.52826 | 0.263 | 0.566 | 13 | **71** | **99** | In polytomy with *Candelaria*, *Youngina*, *Orovenator*, *Hovasaurus*, *Claudiosaurus*, *Acerosodontosaurus*, and Sauria with BS 2 |
| **51** | + | + | + | + | + | 4.375 | 1 | 99.02155 | 0.263 | 0.566 | 14 | **71** | **99** | In polytomy with *Youngina*, *Orovenator*, *Hovasaurus*, *Claudiosaurus*, *Acerosodontosaurus*, and Sauria with BS 1 |
| **52** | + | + | + | + | + | 4.5 | 1 | 97.56252 | 0.263 | 0.566 | 15 | **70** | **99** | In polytomy with *Youngina*, *Orovenator*, *Hovasaurus*, *Claudiosaurus*, *Acerosodontosaurus*, and Sauria with BS 2 |
| **53** | + | + | + | + | + | 4.625 | 1 | 96.14842 | 0.264 | 0.568 | 15 | **70** | **99** | In polytomy with *Candelaria*, *Youngina*, *Orovenator*, *Hovasaurus*, *Claudiosaurus*, *Acerosodontosaurus*, and Sauria with BS 2 |
| **54** | + | + | + | + | + | 4.75 | 1 | 94.77363 | 0.264 | 0.568 | 14 | **70** | **99** | In polytomy with *Candelaria*, *Youngina*, *Orovenator*, *Hovasaurus*, *Claudiosaurus*, *Acerosodontosaurus*, and Sauria with BS 3 |
| **55** | + | + | + | + | + | 4.875 | 1 | 93.44011 | 0.264 | 0.568 | 15 | **70** | **99** | In polytomy with *Candelaria*, *Youngina*, *Orovenator*, *Hovasaurus*, *Claudiosaurus*, *Acerosodontosaurus*, and Sauria with BS 2 |
| **56** | + | + | + | + | + | 5 | 1 | 92.14593 | 0.264 | 0.568 | 15 | **71** | **99** | In polytomy with *Candelaria*, *Youngina*, *Orovenator*, *Hovasaurus*, *Claudiosaurus*, *Acerosodontosaurus*, and Sauria with BS 3 |
| **57** | + | + | + | + | + | 5.125 | 1 | 90.88929 | 0.264 | 0.568 | 15 | **71** | **99** | In polytomy with *Candelaria*, *Youngina*, *Orovenator*, *Hovasaurus*, *Claudiosaurus*, *Acerosodontosaurus*, and Sauria with BS 1 |
| **58** | + | + | + | + | + | 5.25 | 1 | 89.66848 | 0.264 | 0.568 | 17 | **70** | **99** | In polytomy with *Candelaria*, *Youngina*, *Orovenator*, *Hovasaurus*, *Claudiosaurus*, *Acerosodontosaurus*, and Sauria with BS 1 |
| **59** | + | + | + | + | + | 5.375 | 1 | 88.48192 | 0.264 | 0.568 | 16 | 69 | **99** | In polytomy with *Youngina*, *Orovenator*, *Hovasaurus*, *Claudiosaurus*, *Acerosodontosaurus*, and Sauria with BS 1 |
| **60** | + | + | + | + | + | 5.5 | 1 | 87.32812 | 0.264 | 0.568 | 15 | 69 | **98** | In polytomy with *Candelaria*, *Youngina*, *Orovenator*, *Hovasaurus*, *Claudiosaurus*, *Acerosodontosaurus*, and Sauria with BS 2 |
| **61** | + | + | + | + | + | 6 | 1 | 83.00872 | 0.266 | 0.573 | 15 | 67 | **98** | In large polytomy |
| **62** | + | + | + | + | + | 10 | 1 | 59.61361 | 0.266 | 0.574 | 15 | 65 | **98** | In large polytomy |
| **63** | + | + | + | + | + | 100 | 1 | 8.36121 | 0.266 | 0.574 | 11 | 38 | **97** | In clade with *Candelaria* with BS 1, in large polytomy |
| **64** | + | + | + | + | + | 1000 | 1 | 0.87446 | 0.266 | 0.574 | 9 | 35 | **97** | In large polytomy |

**Supplementary Table 4.** K-values, number of obtained trees, best score (Tree Bisection-Reconnection), Consistency Index, Retention Index and bootstrap values (BS, 1000 replicates) for clades consisting of *Eunotosaurus africanus* and more derived pan-testudinates (ET), *Pappochelys rosinae* and more derived pan-testudinates (PT), and *Odontochelys semitestacea* and *Proganochelys quenstedti* (OT) from analyses 65-70 (1000 replicates, different combinations of taxa, implied weighting). The most optimal values and position within Diapsida are indicated in green, the less optimal and position outside of Diapsida in red.

| # | *Proganochelys* | *Candelaria* | *Pappochelys* | *Odontochelys* | *Eunotosaurus* | K | Trees | Best score | Consistency Index | Retention Index | BS | | | Position of Pan-Testudinata |
|---|---|---|---|---|---|---|---|---|---|---|---|---|---|---|
| | | | | | | | | | | | ET | PT | OT | |
| **65** | + | - | + | + | - | 2,75 | 1 | 118,12216 | 0,276 | 0,578 | - | 90 | **99** | In clade with (Lepidosauria + Kuehneosauridae + Sauropterygia) with BS 6 |
| **66** | - | - | + | + | - | 2.75 | 1 | 111.97834 | 0.288 | 0.583 | - | **93** | - | In clade with (Lepidosauria (Kuehneosauridae + Sauropterygia)) with BS 8 |
| **67** | + | - | - | + | + | 2.5 | 2 | 125.62109 | 0.272 | 0.571 | 58 | - | **99** | In clade with (*Orovernator* + *Hovasaurus* + *Youngina* + *Acerosodontosaurus* + (*Claudiosaurus* + Sauria)) with BS 26 |
| **68** | + | - | - | - | + | 2.5 | 2 | 124.59944 | 0.274 | 0.564 | 64 | - | - | In clade with (*Orovernator* + *Youngina* (*Hovasaurus* + *Acerosodontosaurus*) + (*Claudiosaurus* + Sauria)) with BS 28 |
| **69** | - | - | + | - | + | 2.75 | 2 | 115.26717 | 0.282 | 0.576 | - | - | - | *Eunotosaurus* in large polytomy *Pappochelys* in polytomy with Kuehneosauridae, Lepidosauria, Archosauromorpha and Sauropterygia with BS 5 |
| **70** | + | - | + | + | + | 2.75 | 2 | 122.40146 | 0.267 | 0.570 | 53 | **92** | **99** | In clade with *Acerosodontosaurus* with BS 9, sister clade to Sauria with BS 3 |

**Supplementary Table 5.** K-values, number of obtained trees, best score (Tree Bisection-Reconnection), Consistency Index, Retention Index and bootstrap values (BS, 1000 replicates) for clades consisting of *Eunotosaurus africanus* and more derived pan-testudinates (ET), *Pappochelys rosinae* and more derived pan-testudinates (PT), and *Odontochelys semitestacea* and *Proganochelys quenstedti* (OT) from analyses 71-90 (1000 replicates, different combinations of characters and taxa, with or without implied weighting [IW]). The most optimal values and position within Diapsida are indicated in green, the less optimal and position outside of Diapsida in red.

| # | *Candelaria* | *Eunotosaurus* | Characters | | | | | IW | K | Trees | Best score | Consistency Index | Retention Index | BS | | | Position of Pan-Testudinata |
|---|---|---|---|---|---|---|---|---|---|---|---|---|---|---|---|---|---|
| | | | 174 | 204 | 247 | 251 | 253 | | | | | | | ET | PT | OT | |
| 71 | + | + | + | + | + | + | - | - | - | 47 | 1194 | 0.265 | 0.574 | 6 | 32 | 96 | In large polytomy |
| 72 | + | + | + | + | + | + | - | + | 2.75 | 1 | 123.06930 | 0.262 | 0.565 | 12 | 70 | 99 | In polytomy with *Youngina*, *Orovenator*, *Hovasaurus*, *Claudiosaurus*, *Acerosodontosaurus*, and Sauria with BS 7 |
| 73 | + | + | + | + | - | + | - | - | - | 47 | 1193 | 0.265 | 0.573 | 2 | 24 | 98 | In large polytomy |
| 74 | + | + | + | + | - | + | - | + | 2.75 | 1 | 123.06930 | 0.261 | 0.564 | - | 70 | 98 | *Eunotosaurus* in large polytomy<br>Pan-Testudinata in large polytomy |
| 75 | + | + | + | + | - | - | - | - | - | 122 | 1190 | 0.265 | 0.573 | - | 28 | 97 | *Eunotosaurus* in large polytomy<br>Pan-Testudinata in large polytomy |
| 76 | + | + | + | + | - | - | - | + | 2.75 | 1 | 122.64825 | 0.261 | 0.564 | - | 67 | 98 | *Eunotosaurus* in large polytomy<br>Pan-Testudinata in large polytomy |
| 77 | + | + | - | + | - | - | - | - | - | 75 | 1186 | 0.265 | 0.574 | - | 33 | 98 | *Eunotosaurus* in large polytomy<br>Pan-Testudinata in large polytomy |
| 78 | + | + | - | + | - | - | - | + | 2.75 | 1 | 122.16863 | 0.261 | 0.565 | - | 71 | 99 | *Eunotosaurus* in large polytomy<br>Pan-Testudinata in clade with Sauria with BS 3 |
| 79 | + | + | - | - | - | - | - | - | - | 30 | 1175 | 0.266 | 0.576 | - | 36 | 97 | Polytomy *Youngina*, *Hovasaurus*, *Claudiosaurus*, *Acerosodontosaurus*, *Eunotosaurus*, Pan-Testudinata, and Sauria with BS 1 |
| 80 | + | + | - | - | - | - | - | + | 2.75 | 1 | 121.42445 | 0.261 | 0.567 | - | 73 | 98 | Polytomy *Youngina*, *Hovasaurus*, *Claudiosaurus*, *Acerosodontosaurus*, *Eunotosaurus*, Pan-Testudinata, and Sauria with BS 1 |
| 81 | - | + | + | + | + | + | - | - | - | 2 | 1175 | 0.270 | 0.577 | 20 | 43 | 97 | In polytomy with *Claudiosaurus*, *Acerosodontosaurus* and Sauria with BS 1 |
| 82 | - | + | + | + | + | + | - | + | 2.75 | 2 | 121.98041 | 0.266 | 0.569 | 39 | 93 | 99 | In clade with *Acerosodontosaurus* with BS 2, in polytomy with *Orovenator*, *Youngina*, *Hovasaurus*, *Claudiosaurus*, and Sauria with BS 30 |
| 83 | - | + | + | + | - | + | - | - | - | 2 | 1174 | 0.269 | 0.577 | 11 | 36 | 98 | In polytomy with *Acerosodontosaurus* and Sauria with BS 2 |
| 84 | - | + | + | + | - | + | - | + | 2.75 | 2 | 121.98041 | 0.266 | 0.569 | 20 | 89 | 98 | In polytomy with *Claudiosaurus*, *Acerosodontosaurus*, and Sauria with BS 1 |
| 85 | - | + | + | + | - | - | - | - | - | 2 | 1171 | 0.269 | 0.577 | 2 | 39 | 99 | In polytomy with *Claudiosaurus*, *Acerosodontosaurus*, and Sauria with BS 2 |
| 86 | - | + | + | + | - | - | - | + | 2.75 | 2 | 121.55936 | 0.265 | 0.569 | 9 | 87 | 98 | In clade with *Acerosodontosaurus* with BS 2, in polytomy with *Orovenator*, *Youngina*, *Hovasaurus*, *Claudiosaurus*, and Sauria with BS 30 |
| 87 | - | + | - | + | - | - | - | - | - | 6 | 1168 | 0.269 | 0.577 | - | 45 | 98 | Polytomy *Claudiosaurus*, *Acerosodontosaurus*, *Eunotosaurus*, Pan-Testudinata, and Sauria with BS 1 |
| 88 | - | + | - | + | - | - | - | + | 2.75 | 2 | 121.07142 | 0.265 | 0.569 | - | 88 | 99 | Polytomy *Eunotosaurus*, *Orovenator*, *Youngina*, *Hovasaurus*, *Claudiosaurus*, *Acerosodontosaurus*, and (Pan-Testudinata + Sauria (BS 2)) with BS 16 |
| 89 | - | + | - | - | - | - | - | - | - | 4 | 1158 | 0.269 | 0.578 | - | 47 | 98 | Polytomy *Eunotosaurus*, *Acerosodontosaurus*, and Pan-Testudinata (BS 1) in polytomy with Sauria, *Youngina*, *Hovasaurus*, and *Claudiosaurus* (BS 6) |
| 90 | - | + | - | - | - | - | - | + | 2.75 | 2 | 120.31953 | 0.266 | 0.571 | - | 89 | 99 | Polytomy *Eunotosaurus*, *Orovenator*, *Youngina*, *Hovasaurus*, *Claudiosaurus*, *Acerosodontosaurus*, Pan-Testudinata, and Sauria with BS 17 |
| 91 | - | - | - | + | - | - | - | - | - | 4 | 1116 | 0.279 | 0.588 | - | 74 | 99 | In clade with (Lepidosauria + Sauropterygia) with BS 1, in polytomy with Archosauromorpha and Kuehneosauridae with BS 19 |
| 92 | - | - | - | + | - | - | - | + | 2.75 | 1 | 116.85901 | 0.274 | 0.578 | - | 86 | 99 | In clade with (Kuehneosauridae, Lepidosauria, Sauropterygia) with BS 8, in clade with Archosauromorpha with BS 26 |

**Supplementary Table 6.** The synapomorphies supporting the exclusive turtle-*Eunotosaurus* clade in analyses 1-32. Given are the number of analyses in which the character state occurs as a synapomorphy of the exclusive turtle-*Eunotosaurus* clade, character number, name, and state, number of other occurrences of that state in the matrix (excluding *E. africanus*, *Pappochelys rosinae*, *Odontochelys semitestacea* and *Proganochelys quenstedti*), and number of times the character appears in the matrix scored as unknown (?; P – *Pa. rosinae*, O – *O. semitestacea*, Pr – *Prog. quenstedti*). Column - P indicates whether the given synapomorphy is present only in analyses without *Pa. rosinae*, column PT if it only appears in analyses including both *Pa. rosinae* and more derived pan-testudinates (*O. semitestacea* and/or *P. quenstedti*) and column Polytomy the synapomorphies exclusive to analyses in which the turtle-*Eunotosaurus* clade is located in a large polytomy. The widespread character codings (appearing in at least 1/3 of all the taxa) are indicated in red. Character 0 (light gray) is scored as 0 for *Pa. rosinae*, and in all the analyses including this taxon the algorithm treats this as a reversion. Characters in dark grey have different states for *Pa. rosinae* than for *O. semitestacea*, *Prog. quenstedti* and *E. africanus* and thus only appear as synapomorphies in analyses excluding the former taxon. Characters 202, 248, and 250 (yellow) are scored respectively 1, 0, and 0 for *Pa. rosinae* and *O. semitestacea*, which is also treated as reversions in these taxa. Character 277 state is unknown for *Pa. rosinae* and *O. semitestacea*, and for *Prog. quenstedti* its state is 1, and still the algorithm treats it as synapomorphy in analysis 2.

| Occurrences | Character | | | - P | PT | Polytomy | Other occurrences in matrix | ? |
|---|---|---|---|---|---|---|---|---|
| | # | Name | State | | | | | |
| 10 | 174 | Presacral vertebral count | 1 | | | | 5 | 14 + P |
| 10 | 247 | T-shaped ribs | 1 | | | | 0 | 10 |
| 10 | 251 | Trunk length | 1 | | | | 3 | 13 + P |
| 10 | 253 | Distinctly broadened ribs | 2 | | | | 0 | 11 + P |
| 9 | 33 | Lacrimal morphology | 2 | | | | 11 | 7 + P |
| 8 | 0 | Skull proportions | 2 | | | | 6 | 7 |
| 7 | 84 | Quadratojugal, vertical process | 2 | | | | 2 | 12 + P + O |
| 7 | 147 | Parasphenoid, cultriform process | 1 | | | | 19 | 7 + P |
| 7 | 148 | Parasphenoid, teeth | 1 | | | | 30 | 3 + P |
| 6 | 73 | Tabular | 1 | | | | 16 | 8 + O |
| 6 | 152 | "Laterosphenoid" | 1 | | | | 1 | 20 + P + O |
| 6 | 158 | Meckelian fossa, length | 1 | | | | 14 | 14 + P + O |
| 6 | 161 | Surangular, contribution to articular facet | 1 | | | | 4 | 12 + P + O |
| 6 | 181 | Cervical intercentra | 1 | | | | 3 | 18 + P + O |
| 6 | 204 | Ectepicondylar groove | 2 | | | | 9 | 18 + P + O |
| 5 | 15 | Septomaxilla | 1 | | | | 5 | 20 + P + O |
| 5 | 62 | Parietal, shelf for adductor musculature | 0 | | | | 24 | 8 |
| 5 | 155 | Coronoid process | 1 | | | | 10 | 14 + P + O |
| 4 | 42 | Frontal proportions | 1 | + | | | 4 | 8 + O |
| 4 | 64 | Parietal-opisthotic contact | 1 | | | | 3 | 22 + P + O |
| 4 | 127 | Quadrate, lateral exposure | 1 | | | | 13 | 2 + P |
| 4 | 130 | Quadrate-prootic contact | 1 | | | | 1 | 23 + P + O |
| 4 | 134 | Stapes, shape | 2 | | | | 1 | 24 + P + O |
| 4 | 145 | Basioccipital/basisphenoid relationship | 1 | | | | 12 | 22 + P |
| 4 | 188 | Caudal ribs (transverse processes) | 1 | | | | 13 | 20 + P |
| 4 | 203 | Supinator process | 2 | | | | 7 | 15 + P + O |
| 4 | 220 | Distal femoral condyles | 1 | | | | 6 | 22 |
| 4 | 231 | Metatarsal V | 1 | | | | 9 | 19 + P |
| 4 | 240 | Manus and pes | 0 | + | | | 15 | 15 |
| 3 | 21 | Maxilla-prefrontal contact | 1 | | | + | 11 | 7 + P + O |
| 3 | 25 | Maxilla-quadratojugal contact | 0 | | | + | 20 | 10 + O |
| 3 | 44 | Frontal-nasal suture | 0 | | | + | 19 | 11 + O |
| 3 | 48 | Frontal, posterolateral processes | 0 | + | | | 20 | 10 + O |
| 3 | 50 | Dermal sculpturing | 1 | + | | | 21 | 2 |
| 3 | 57 | Postorbital, shape of posterior process | 1 | + | | | 22 | 6 + O |
| 3 | 81 | Squamosal, occipital flange | 0 | + | | | 9 | 13 + O |
| 3 | 124 | Vomer, distribution of teeth | 1 | | | | 2 | 24 + P |
| 3 | 129 | Quadrate, anterior process | 0 | + | | | 22 | 7 + O |
| 3 | 217 | Fourth trochanter of femur | 1 | | | + | 7 | 23 + O |
| 3 | 252 | Sacral rib distal overlap | 1 | | + | | 9 | 17 |
| 2 | 24 | Maxilla, orbital exposure | 1 | + | | | 11 | 9 + O |
| 2 | 43 | Frontal-parietal length ratio | 1 | + | | | 13 | 8 |
| 2 | 55 | Postorbital-parietal contact | 0 | | + | | 9 | 11 + O |
| 2 | 94 | Lower temporal fenestra, ventral margin | 1 | | + | + | 13 | 13 + O + Pr |
| 2 | 112 | Basipterygoid processes, orientation | 0 | | + | | 13 | 12 + P |
| 2 | 135 | Stapes, dorsal process | 1 | | | + | 13 | 20 + P + O |
| 2 | 136 | Epipterygoid, dorsal wing | 1 | | | + | 7 | 28 + P + O |
| 2 | 141 | Paroccipital process, distal end | 1 | | | + | 15 | 15 + P + O |
| 2 | 151 | Sphenethmoid | 1 | | | + | 8 | 25 + P + O |
| 2 | 196 | Supraglenoid buttress | 1 | | | + | 12 | 25 + P + O |
| 2 | 201 | Humeral shaft-distal end ratio | 0 | | | + | 17 | 14 |
| 2 | 248 | Ribs | 1 | + | | | 1 | 15 |
| 2 | 250 | Length of dorsal vertebrae | 1 | + | | | 0 | 15 |
| 1 | 29 | Maxilla, anterolateral foramen | 0 | | + | + | 15 | 6 + O |
| 1 | 67 | Parietal-squamosal contact | 0 | | + | | 21 | 7 + O |
| 1 | 89 | Upper temporal fenestra | 1 | | + | + | 16 | 0 |
| 1 | 111 | Ptergyoid, aruate flange | 1 | + | | | 18 | 5 + P |
| 1 | 154 | Mandibular joint, position relative to occipital condyle | 1 | | + | | 15 | 4 + P |
| 1 | 202 | Humeral distal articulation | 0 | + | | | 13 | 18 |
| 1 | 277 | Quatratojugal exposition in lateral view | 1 | | + | + | 2 | 6 + P + O |

**Supplementary Table 7.** The synapomorphies supporting the exclusive turtle-*Eunotosaurus* clade in analyses 33-70. Given are the number of analyses in which the character state occurs as a synapomorphy of the exclusive turtle-*Eunotosaurus* clade, character number, name, and state, number of other occurrences of that state in the matrix (excluding *E. africanus*, *Pappochelys rosinae*, *Odontochelys semitestacea* and *Proganochelys quenstedti*) and number of times the character appears in the matrix scored as unknown (?; P – *Pa. rosinae*, O – *O. semitestacea*, Pr – *Prog. quenstedti*). Column - P indicates whether the given synapomorphy is present only in analyses without *Pa. rosinae*, column PT if it only appears in analyses including both *Pa. rosinae* and more derived pan-testudinates (*O. semitestacea* and/or *P. quenstedti*) and column Polytomy the synapomorphies exclusive to analyses in which the turtle-*Eunotosaurus* clade is located in a large polytomy. The widespread character codings (appearing in at least 1/3 of all the taxa) are indicated in red. Character 0 (light gray) is scored as 0 for *Pa. rosinae*, and in all the analyses including this taxon the algorithm treats this as a reversion. Characters in dark grey have different states for *Pa. rosinae* than for *O. semitestacea*, *Prog. quenstedti* and *E. africanus* and thus only appear as synapomorphies in analyses excluding the former taxon. Characters 202, 248, and 250 (yellow) are scored respectively 1, 0, and 0 for *Pa. rosinae* and *O. semitestacea*, which is also treated as reversions in these taxa.

| Occurrences | Character | | | - P | PT | Polytomy | Other occurrences in matrix | ? |
|---|---|---|---|---|---|---|---|---|
| | # | Name | State | | | | | |
| 33 | 84 | Quadratojugal, vertical process | 2 | | | | 2 | 12 + P + O |
| 32 | 15 | Septomaxilla | 1 | | | | 5 | 20 + P + O |
| 32 | 33 | Lacrimal morphology | 2 | | | | 11 | 7 + P |
| 32 | 147 | Parasphenoid, cultriform process | 1 | | | | 19 | 7 + P |
| 32 | 247 | T-shaped ribs | 1 | | | | 0 | 10 |
| 32 | 251 | Trunk length | 1 | | | | 3 | 13 + P |
| 32 | 253 | Distinctly broadened ribs | 2 | | | | 0 | 11 + P |
| 31 | 44 | Frontal-nasal suture | 0 | | | | 19 | 11 + O |
| 31 | 64 | Parietal-opisthotic contact | 1 | | | | 3 | 22 + P + O |
| 31 | 130 | Quadrate-prootic contact | 1 | | | | 1 | 23 + P + O |
| 31 | 134 | Stapes, shape | 2 | | | | 1 | 24 + P + O |
| 31 | 152 | "Laterosphenoid" | 1 | | | | 1 | 20 + P + O |
| 31 | 155 | Coronoid process | 1 | | | | 10 | 14 + P + O |
| 31 | 158 | Meckelian fossa, length | 1 | | | | 14 | 14 + P + O |
| 31 | 161 | Surangular, contribution to articular facet | 1 | | | | 4 | 12 + P + O |
| 31 | 174 | Presacral vertebral count | 1 | | | | 5 | 14 + P |
| 31 | 181 | Cervical intercentra | 1 | | | | 3 | 18 + P + O |
| 29 | 0 | Skull proportions | 2 | | | | 6 | 7 |
| 29 | 252 | Sacral rib distal overlap | 1 | | + | | 9 | 17 |
| 28 | 203 | Supinator process | 2 | | | | 7 | 15 + P + O |
| 26 | 204 | Ectepicondylar groove | 2 | | | | 9 | 18 + P + O |
| 24 | 62 | Parietal, shelf for adductor musculature | 0 | | + | | 24 | 8 |
| 19 | 21 | Maxilla-prefrontal contact | 1 | | + | | 11 | 7 + P + O |
| 16 | 148 | Parasphenoid, teeth | 1 | | | | 30 | 3 + P |
| 7 | 73 | Tabular | 1 | | | | 16 | 8 + O |
| 5 | 94 | Lower temporal fenestra, ventral margin | 1 | | + | + | 13 | 13 + O + Pr |
| 5 | 127 | Quadrate, lateral exposure | 1 | | + | + | 13 | 2 + P |
| 4 | 25 | Maxilla-quadratojugal contact | 0 | | + | + | 20 | 10 + O |
| 4 | 135 | Stapes, dorsal process | 1 | | + | + | 13 | 20 + P + O |
| 4 | 136 | Epipterygoid, dorsal wing | 1 | | + | + | 7 | 28 + P + O |
| 4 | 141 | Paroccipital process, distal end | 1 | | + | + | 15 | 15 + P + O |
| 4 | 145 | Basioccipital/basisphenoid relationship | 1 | | + | + | 12 | 22 + P |
| 4 | 151 | Sphenethmoid | 1 | | + | + | 8 | 25 + P + O |
| 4 | 188 | Caudal ribs (transverse processes) | 1 | | + | + | 13 | 20 + P |
| 4 | 217 | Fourth trochanter of femur | 1 | | + | + | 7 | 23 + O |
| 4 | 220 | Distal femoral condyles | 1 | | + | + | 6 | 22 |
| 4 | 231 | Metatarsal V | 1 | | + | + | 9 | 19 + P |
| 3 | 201 | Humeral shaft-distal end ratio | 0 | | + | + | 17 | 14 |
| 3 | 202 | Humeral distal articulation | 2 | | + | + | 13 | 18 |
| 2 | 42 | Frontal proportions | 1 | + | | | 4 | 8 + O |
| 2 | 81 | Squamosal, occipital flange | 0 | + | | | 9 | 13 + O |
| 2 | 196 | Supraglenoid buttress | 1 | | + | + | 12 | 25 + P + O |
| 1 | 48 | Frontal, posterolateral processes | 0 | + | | | 20 | 10 + O |
| 1 | 55 | Postorbital-parietal contact | 0 | | + | + | 9 | 11 + O |
| 1 | 89 | Upper temporal fenestra | 1 | | + | + | 16 | 0 |
| 1 | 248 | Ribs | 1 | + | | | 1 | 15 |
| 1 | 250 | Length of dorsal vertebrae | 1 | + | | | 0 | 15 |
